# Deepening of In Silico Evaluation of SARS-CoV-2 Detection RT-qPCR Assays in the Context of New Variants

**DOI:** 10.3390/genes12040565

**Published:** 2021-04-13

**Authors:** Mathieu Gand, Kevin Vanneste, Isabelle Thomas, Steven Van Gucht, Arnaud Capron, Philippe Herman, Nancy H. C. Roosens, Sigrid C. J. De Keersmaecker

**Affiliations:** 1 Transversal Activities in Applied Genomics, Sciensano, J. Wytsmanstraat 14, B-1050 Brussels, Belgium; mathieu.gand@sciensano.be (M.G.); kevin.vanneste@sciensano.be (K.V.); Nancy.Roosens@sciensano.be (N.H.C.R.); 2 Viral Diseases, Sciensano, J. Wytsmanstraat 14, B-1050 Brussels, Belgium; Isabelle.Thomas@sciensano.be (I.T.); Steven.VanGucht@sciensano.be (S.V.G.); 3 Quality of Laboratories, Sciensano, J. Wytsmanstraat 14, B-1050 Brussels, Belgium; Arnaud.Capron@sciensano.be; 4 Expertise and Service Provision, Sciensano, J. Wytsmanstraat 14, B-1050 Brussels, Belgium; Philippe.Herman@sciensano.be

**Keywords:** SARS-CoV-2, COVID-19, variants, detection, RT-qPCR, in silico specificity evaluation, bioinformatics tool, WGS data, mismatches, primers and probes

## Abstract

For 1 year now, the world is undergoing a coronavirus disease-2019 (COVID-19) pandemic due to the severe acute respiratory syndrome coronavirus 2 (SARS-CoV-2). The most widely used method for COVID-19 diagnosis is the detection of viral RNA by RT-qPCR with a specific set of primers and probe. It is important to frequently evaluate the performance of these tests and this can be done first by an in silico approach. Previously, we reported some mismatches between the oligonucleotides of publicly available RT-qPCR assays and SARS-CoV-2 genomes collected from GISAID and NCBI, potentially impacting proper detection of the virus. In the present study, 11 primers and probe sets investigated during the first study were evaluated again with 84,305 new SARS-CoV-2 unique genomes collected between June 2020 and January 2021. The lower inclusivity of the China CDC assay targeting the gene N has continued to decrease with new mismatches detected, whereas the other evaluated assays kept their inclusivity above 99%. Additionally, some mutations specific to new SARS-CoV-2 variants of concern were found to be located in oligonucleotide annealing sites. This might impact the strategy to be considered for future SARS-CoV-2 testing. Given the potential threat of the new variants, it is crucial to assess if they can still be correctly targeted by the primers and probes of the RT-qPCR assays. Our study highlights that considering the evolution of the virus and the emergence of new variants, an in silico (re-)evaluation should be performed on a regular basis. Ideally, this should be done for all the RT-qPCR assays employed for SARS-CoV-2 detection, including also commercial tests, although the primer and probe sequences used in these kits are rarely disclosed, which impedes independent performance evaluation.

## 1. Introduction

The severe acute respiratory syndrome coronavirus 2 (SARS-CoV-2) is responsible for the coronavirus disease (COVID-19). It emerged at the end of 2019 in Wuhan (China) and spread globally in 2020, leading to a massive pandemic still ongoing. This potentially life-threatening new coronavirus was estimated to be responsible for already 2,728,732 deaths in 192 countries and 123,968,726 confirmed COVID-19 cases (COVID-19 dashboard accessed 23 March 2021 [1]), putting health care systems under severe pressure [2,3,4,5]. Regarding the burden of SARS-CoV-2, many countries have set in place control measures to mitigate its spread, such as travel restrictions, social distancing, curfews, home-working policies, and lockdowns. Although these measures have proven to be effective to reduce new cases, they have dramatic consequences on the economy and a negative psycho-social impact on the population [6,7,8]. During the first COVID-19 wave, some countries in Asia, e.g., Hong-Kong, Singapore, South-Korea, and Taiwan, succeeded to limit the number of COVID-19 cases and had a relatively small case-facility rate, in comparison with European countries and the United States, by also implementing massive testing and tracing campaigns. This allowed the fast identification of infected individuals, including those with silent symptoms who can unconsciously infect other people [9,10,11,12]. Thanks to this intensive testing strategy, COVID-19 diagnosed people were rapidly, for the severe cases, taken care of by the medical system, or for the patients developing mild to moderate symptoms, isolated to mitigate the spread of the virus in the community. The way these countries managed the pandemic showcased how early diagnosis of SARS-CoV-2-positive individuals is a key strategy to control the dissemination of the virus.

During the past year, several technologies, such as Nucleic Acid Amplification Test (NAAT) and immunological assays (antibodies/antigens detection), have been employed to develop COVID-19 diagnostic assays. The “gold-standard” widely used method is a NAAT: the Reverse Transcription real-time Polymerase Chain Reaction (RT-qPCR) [9,13]. Based on specific hybridization between oligonucleotides, i.e., primers and probe, and their respective target sequences in the viral genome, followed by replication and production of a fluorescence signal at each amplification step, RT-qPCR is able to detect the presence of SARS-CoV-2 RNA in human samples such as nasopharyngeal swabs. The RNA sequence targeted by the primers and probe can be located in genes coding for structural proteins such as Envelope (E), Spike (S), and Nucleocapsid (N) or in Open-Reading Frames (ORFs) such as ORF1ab that contains for instance the gene coding for the RNA-dependent RNA polymerase (RdRp). Early in the pandemic, i.e., 24 January 2020, the World Health Organization (WHO) recommended publicly available RT-qPCR tests for SARS-CoV-2 detection in their technical guidance [14]. Later during the year, in parallel with the publication of other RT-qPCR methods in the scientific literature, a race between detection kit suppliers began to put commercial RT-qPCR assays on the market. An unusual false negative rate was first reported when using RT-qPCR methods for COVID-19 diagnosis, but it was later shown that these incorrect results were more often due to improper sample collection or collection time, i.e., too soon before symptom onset or too late after symptom release, rather than to a lack of specificity [9,15,16,17]. However, as 2–3 mismatches between oligonucleotides and their complementary sequences, or a single mismatch located in the 3’ end of primers, can result in decreased NAAT performance or even test failure [18,19,20,21], it is important to monitor the SARS-CoV-2 mutations that can have an impact on RT-qPCR outcome.

The evolutionary rate of SARS-CoV-2 is estimated to be low, i.e., 1–2 substitutions per genome per month [22], in comparison to other RNA viruses, probably thanks to a proof-reading system included within the virus polymerase [23,24]. Nevertheless, the emergence and spread of new SARS-CoV-2 mutants is expected to increase with the use of vaccines and therapeutics, introducing selection pressure [25,26], as already observed for influenza [27]. Additionally, it was demonstrated that long-term infections and infections in immunocompromised patients can also promote the acquisition of new mutations [28,29]. Several SARS-CoV-2 new variants carrying an unusual number of mutations have already emerged. Some of them were for instance detected in the United-Kingdom (UK), South-Africa (SA), and Brazil. The UK variant, called Variant of Concern (VOC) 202012/01, belongs to Global Initiative on Sharing Avian Influenza Data (GISAID) clade GR, Pangolin lineage B.1.1.7, and Nextstrain clade 20I/501Y.V1 and is defined by 23 mutations: 13 non-synonymous mutations, 4 deletions, and 6 synonymous mutations [30,31,32]. The SA variant belongs to GISAID clade GH, Pangolin lineage B.1.351, and Nextstrain clade 20H/501Y.V2 and contains up to 22 non-synonymous mutations [33]. Finally, the Brazil variant belongs to GISAID clade GR, Pangolin lineage P.1, and Nextstrain clade 20J/501Y.V3 and carries 20 non-synonymous and 6 synonymous mutations [34,35]. These variants are considered "of concern" by the scientific community and public health authorities because they are linked to multiple amino-acid changes in the S protein, with some of them (K417N, K417T, E484K, and N501Y) located in the Receptor Binding Domain (RBD), the main functional motif interacting with the human Angiotensin-Converting Enzyme 2 (ACE2) receptor for cell entry [36,37]. These changes are thought to improve the interaction of SARS-CoV-2 with human cells, in line with the epidemiological data showing a sudden rise of COVID-19 cases in UK and SA, associated with the prevalence of their respective new variants [30,31,32,33,38]. In addition to this enhanced transmissibility, concerns exist regarding the immunological response and vaccine efficiency, as the S protein is the primary target of neutralizing antibodies and the currently distributed vaccines [38,39,40,41]. 

Given the potential threat of the new variants 20I/501Y.V1, 20H/501Y.V2, and 20J/501Y.V3, it is crucial to assess if these can be correctly detected by the RT-qPCR assays currently used for COVID-19 diagnosis. As these 3 variants carry several mutations, if some of them are located in oligonucleotide annealing sites, the resulting mismatches can lead to test failure or loss of sensitivity [18,19,20,21]. However, this kind of specificity (inclusivity) evaluation can be difficult to perform in the wet-lab as SARS-CoV-2 is a new virus and no laboratory has a complete representative collection of circulating strains, including all new emerging variants. To overcome this limitation, bioinformatics tools were previously used to perform an in silico specificity evaluation, as a first step of a full evaluation process. This was done by taking benefit of the huge sequencing efforts, making large amounts of Whole Genome Sequencing (WGS) data available in public databases such as NCBI and GISAID [42,43,44]. Ideally, this kind of in silico evaluation has to be performed on a frequent basis, considering that new genomes are continuously uploaded into the databases and especially when new SARS-CoV-2 VOC are identified. In addition, not only the effects of the mutations defining some specific VOC need to be investigated but the impact of all the mutations present in the variant genomes should to be taken into account, including nucleotide changes that were slowly acquired through time or emerged independently and are not representative of the variants population.

We previously used a BLAST-based user-friendly open-access bioinformatics tool named “SCREENED” (polymeraSe Chain Reaction Evaluation through largE-scale miNing of gEnomic Data) [45] to investigate mismatches between 30 primers and probe sets and large amounts of WGS data downloaded from the databases cited above [46]. For each oligonucleotide set and analyzed genomes, SCREENED generates mismatch scores and estimates the production of a positive or negative theoretical RT-qPCR signal according to the total number of mutations present in the annealing sites as well as their positions. In the present study, a selection of these primers and probe sets were evaluated again for their inclusivity using SCREENED, with 84,305 new SARS-CoV-2 unique genomes collected between June 2020 and January 2021. Additionally, a specific focus was put on the new variants 20I/501Y.V1, 20H/501Y.V2 and 20J/501Y.V3 by investigating the effect of their mutations on the evaluated assays.

## 2. Materials and Methods

### 2.1. SARS-CoV-2 WGS Dataset

On 7 January 2021, 31,244 and 199,600 SARS-CoV-2 genomes, coming from samples collected since 7 June 2020, were, respectively, downloaded from the NCBI Virus (https://www.ncbi.nlm.nih.gov/labs/virus/, accessed on 7 January 2021); Supplementary Materials File S1) and GISAID EpiCoV (https://www.epicov.org, accessed on 7 January 2021; Supplementary Materials File S2) databases. When downloaded from GISAID, only the complete genomes coming from human samples were selected, and the low coverage genomes were excluded. NCBI genomes were complete sequences with “SARS-CoV-2” as the species (taxid: 2697049) and *Homo sapiens* as the “host” (taxid: 9606).

### 2.2. SARS-CoV-2 Lineage Assignment

For the GISAID genomes, the lineage assignment was extracted from the associated metadata in the database (Supplementary Materials File S2). For the NCBI genomes, lineage assignment was performed using the tool Pangolin (version 2.1.10; https://github.com/cov-lineages/pangolin, accessed on 7 January 2021; [47]) with pangoLEARN 02-01-2021 and default parameters (Supplementary Materials File S1). From the total number of downloaded genomes, 8860 belonged to lineage B.1.1.7, i.e., 20I/501Y.V1, and 366 to lineage B.1.351, i.e., 20H/501Y.V2. None of the downloaded genomes were determined to belong to lineage P.1, i.e., 20J/501Y.V3.

### 2.3. Selection of High-Quality Representative SARS-CoV-2 Genomes 

From the downloaded dataset, genomes showing more than one undetermined nucleotide "N" in their sequences were discarded, to retain only high-quality genomes (154,602). Finally, to avoid redundancies in the dataset, all the identical genomes were clustered together using CD-HIT-EST v4.6.8 (https://github.com/weizhongli/cdhit, accessed on 7 January 2021; [48,49]) with sequence identity cut-off equal to 1.0 (other parameters were left at default settings). Only the representative genomes (84,305; Supplementary Materials File S3) of each cluster were used for further analyses.

### 2.4. SCREENED Settings

To determine the theoretical production of RT-qPCR signals, SCREENED v1.0 [45] was used as described in our previous study, with identical settings. Briefly, SCREENED performs a two-step BLAST approach to first fish out in each genome the complete amplicon sequence targeted by the evaluated primers and probe sets, and secondly to produce mismatch statistics from the hybridization between these oligonucleotides and their corresponding annealing sites in the amplicon. For more details, we refer to [45,46]. In the present study, if no mismatch was detected in the first 5 nucleotides of primers‘ 3’ end, if the total number of mismatches did not exceed 10% of oligonucleotides length, and if at least 90% of the oligonucleotides sequence aligned correctly with their targets, SCREENED considered that a positive RT-qPCR signal was produced. These criteria were selected according to what is generally described in the scientific literature for mismatches potentially affecting the performance of PCR-like methods [18,19,20,21]. Considering the primers and probe sets investigated in this study, none exceeding a length of 30 nucleotides except for the forward primer of Assay 8 S (Table 1), this meant that no more than 1–2 mismatches were tolerated. For the Assay 8 S forward primer with a length of 30 nucleotides, no more than 3 mismatches were tolerated. Finally, greedy clustering of the amplicon was enabled as an option in SCREENED.

As input, SCREENED used a FASTA file containing the 84,305 representative SARS-CoV-2 sequences (Supplementary Materials File S3) and a tab-delimited text file containing the sequences of the primers and probes to be evaluated and their corresponding amplicon sequence to be mined in the genomes (Supplementary Materials File S4).

### 2.5. In Silico Analytical Specificity Evaluation

As only SARS-CoV-2 genomes were used in our study for the evaluation of COVID-19 diagnostic RT-qPCR assays, every negative signal reported by SCREENED was considered as a theoretical False Negative (FN) result and used for in silico inclusivity evaluation as follows (1):

(1) $$Inclusivity\;\left(\%\right)=\left(1-\;\frac{Number\;of\;FN}{Total\;number\;of\;representative\;SARS-CoV-2\;genomes}\right)\times 100$$

Only inclusivity was assessed here as exclusivity was already verified during our previous evaluation with genomes belonging to other coronaviruses and common respiratory viruses, as well as the human reference genome, and would not change for the same RT-qPCR assays evaluated [46].

## 3. Results

### 3.1. SCREENED In Silico Specificity Re-Evaluation of RT-qPCR Assays Used for SARS-CoV-2 Detection

In May 2020, the in silico specificity of 30 primers and probe sets was investigated with SCREENED [46]. In the current study, 11 of these sets, listed in Table 1, were evaluated again for their inclusivity with a new dataset of 84,305 representative SARS-CoV-2 genomes (obtained from 230,844 SARS-CoV-2 sequences; see Materials and Methods section) coming from samples collected between 7 June 2020 and 7 January 2021. These 11 oligonucleotides sets were selected because they belong to RT-qPCR tests commonly used as reference methods for comparison studies (Assay 2 RdRp-P2, Assay 2 E, Assay 2 N, Assay 3 RdRp_IP2, Assay 3 RdRp_IP4, Assay 4 N-1, Assay 4 N-2, and Assay 4-N3) [52,53,54,55,56,57,58,59], or because they showed the best (Assay 8 S and Assay 9 ORF1a) and worst (Assay 1 N) specificity results during our first evaluation in May 2020 [46].

From the 84,305 representative SARS-CoV-2 genomes analyzed, 30,445 gave a negative theoretical RT-qPCR signal when evaluating the primers and probe set of Assay 1 N, resulting in an inclusivity of 63.89%. This low inclusivity was mostly due to a 3-nucleotides substitution (GGG to AAC) in the 5’ end of the forward primer, as reported previously [46], sometimes in combination with other nucleotide changes. In total, 64 different combinations of substitutions and deletions in the forward primer sequence were reported by SCREENED as potentially leading to Assay 1 N failure (Supplementary Material File S5). Furthermore, the AAC substitution was found in all the analyzed 20I/501Y.V1 genomes representing 13% of the FN results obtained with Assay 1 N. In contrast, all other assays showed inclusivity results above 99%, as previously obtained [46], with only between 8 (Assay 2 E) and 833 (Assay 4 N-2) negative theoretical RT-qPCR signals. The 3 best inclusivity results were obtained for Assay 2 E (99.99%), Assay 8 S (99.97%), and Assay 3 RdRp_IP4 (99.95%) (Table 2). Nevertheless, amongst these 3 best assays, only Assay 2 E and Assay 3 RdRp_IP4 were estimated to correctly detect all the investigated variant genomes included in the analysis.

Except for the Assay 8 S forward primer, all the primers and probes evaluated here had a length below 30 nucleotides (Table 1), which means that no more than 1–2 mismatches could be tolerated for these oligonucleotides according to the applied SCREENED criteria (see Section 2.4). The forward primer of Assay 8 S is composed of 30 nucleotides and 3 mismatches would still result in a positive RT-qPCR theoretical signal with SCREENED. As it was already demonstrated that more than 2 mismatches can potentially impact the performance of PCR-based methods [19,21], the number of mismatches for the Assay 8 S forward primer was investigated in the detailed output data produced by SCREENED (data not shown). The detailed SCREENED results showed that no more than one mismatch was reported for this oligonucleotide sequence over all analyzed genomes, thus confirming the excellent inclusivity of this assay.

In addition to the production of mismatch statistics, SCREENED allowed clustering of the sequences amplified by the evaluated primers sets from all analyzed genomes. Table 3 shows the total number of clusters obtained per set and the repartition of the genomes in the 3 first clusters, ordered from largest to smallest. With these data, the level of conservation of the amplicons targeted by the different assays can be assessed. The highest number of generated amplicon clusters (496) was observed for Assay 1 N, with a repartition of the analyzed genomes in 3 main clusters containing each between 26.9% and 30.3% of all the WGS data, thus illustrating the high level of diversity in this region of the SARS-CoV-2 genome. In contrast, for the other investigated assays, the majority of the analyzed genomes were clustered together in one main cluster, i.e., the first cluster, with a repartition ranging from 98.6% (Assay 8 S) to 91.1% (Assay 9 ORF1a) of the genomes in this cluster. It can be noticed that the second cluster of Assay 9 ORF1a contained 4.8% of analyzed genomes, whereas no more than 1.8% is included in the second cluster of Assays 2 to 8, all targets considered. This Assay 9 ORF1a second cluster contained almost solely genomes sequenced in England and Wales during the end of 2020 and belonging to Pangolin lineage B1.1.7, the specific lineage of SARS-CoV-2 variant 20I/501Y.V1 (Supplementary Materials File S6).

### 3.2. Impact of Emerging SARS-CoV-2 VOC on RT-qPCR Assays

As 20I/501Y.V1, 20H/501Y.V2, and 20J/501Y.V3 were recently identified as SARS-CoV-2 VOC, at the time of analysis, they were not well represented in the dataset downloaded from GISAID and NCBI, which was used for the specificity evaluation performed in this study using SCREENED (Section 3.1). Genomes belonging to Pangolin lineage B.1.1.7 and B.1.351, i.e., 20I/501Y.V1 and 20H/501Y.V2, constituted only 3.8% (8860/230,844) and 0.2% (366/230,844) of the downloaded dataset, and no genomes belonging to the Pangolin lineage P.1, i.e., 20J/501Y.V3, were available at that time (Supplementary Materials Files S1 and S2). Therefore, to have a better view on the specific impact of the mutations defining the new variants on the 11 evaluated assays, it was first verified if their position in the SARS-CoV-2 genomes (variants’ mutations and their positions are listed in Supplementary Materials File S7) was located between the starting and ending position of the corresponding 11 amplicon sequences (Table 1). When this was the case, their presence in the primers’ and probes’ annealing sites, in addition to their potential impact on the RT-qPCR outcome according to the criteria used for the SCREENED analysis (see Section 2.4), was investigated.

Six nucleotide changes, C3267T and C28977T from variant 20I/501Y.V1, G22813T, and C28887T from variant 20H/501Y.V2, and C12778T and A22812C from variant 20J/501Y.V3, were found in sequences amplified by oligonucleotide sets evaluated in this study (Table 4). The 2 mutations C3267T and C12778T were found in the amplicon of Assay 9 ORF1a and Assay 3 RdRp_IP2 but with no impact on the tests’ outcome because they were located in neither the primers’ nor probe’s annealing sites. Interestingly, the substitution C3267T of variant 20I/501Y.V1 is the nucleotide change defining the amplicon representative sequence of the second cluster of Assay 9 ORF1a (Table 3), what is in line with the content of this cluster made up mostly of genomes sequenced in UK and belonging to lineage B.1.1.7 (Supplementary Materials File S6). The mutations A22812C and G22813T, responsible for 2 amino acid changes, i.e., K417T and K417N, located in the RBD of the S protein, were found in the probe annealing site of Assay 8 S. Finally, the nucleotide substitutions C28887T and C28977T were reported to be, respectively, located in the forward and reverse primer sequences of Assay 1 N. Surprisingly, another study [42] evaluating the impact of variants’ mutations on publicly available assays (including Assay 1 N) with BLAST and 20I/501Y.V1 genome EPI_ISL_744131, did not report the mismatch in the Assay 1 N forward primer due to the C28977T mutation. To better understand the reason of these inconsistent results, the same BLAST analysis was reproduced. This indicated an issue with the manual interpretation of the BLAST output in the other study [42], as in the reproduction of their analysis, we could not find a perfect match between the complete forward primer sequence of Assay 1 N and EPI_ISL_744131 (Supplementary Materials File S8).

Considered alone, the mutations defining the new variants were, based on the SCREENED criteria, not estimated to impact the outcome of the evaluated RT-qPCR assays (Table 4). Nevertheless, it can be noticed that the 20I/501Y.V1 mutation C28977T, present in reverse primer of Assay 1 N, was found to be combined with another non-variant specific mutation present in four B.1.1.7 genomes (Supplementary Materials File S5), resulting in FN results when evaluating Assay 1 N with SCREENED (Section 3.1). Moreover, during the SCREENED analysis (Section 3.1), some variant genomes led to FN results (Table 2) due to other mutations than those defining these new variants (Supplementary Materials File S7). For instance, the 3-nucleotides substitution (GGG to AAC) observed in the Assay 1 N forward sequence was found in all the 20I/501Y.V1 genomes but is not specific to this variant (Supplementary Materials Files S5 and S7). This demonstrates that despite the fact that variant defining mutations were not estimated to impact RT-qPCR outcome on their own, the variants can carry additional mutations, all together potentially impacting the test’s performance.

## 4. Discussion

As some RT-qPCR tests for SARS-CoV-2 detection were developed early in the pandemic based on WGS data available at that time, there is a need to periodically evaluate whether these assays are still performant to detect the virus that has evolved since its first occurrence 1 year ago. As this kind of specificity evaluation would not be feasible in the wet-lab due to the lack of a representative strains collection, in the present study, this was performed in silico for 11 primers and probe sets using the bioinformatics tool SCREENED and 84,305 representative SARS-CoV-2 genomes obtained from GISAID and NCBI. The WGS data used in this study were obtained from samples collected between 7 June 2020 and 7 January 2021. Therefore, this allowed comparison with our previous study that evaluated the same 11 primers and probe sets between April and May 2020 with WGS data available at that time and determined Assay 1 N and Assay 8 S as the least and most specific assays, respectively [46]. 

The Assay 1 from China CDC targeting the gene N was once again the one showing the lowest inclusivity (63.89%), which continued to decrease since April (86.03%) and May (74.54%) 2020. This low score is again mostly due to a substitution of 3 nucleotides (GGG to AAC) in the 5’ end of the forward primer. Nevertheless, although in our first study only 4 different combinations of nucleotide substitutions were reported in the forward primer of Assay 1 N, 64 combinations of substitutions and even deletions were identified in the same sequence with the new dataset until January 2021. This, in combination with the diversity observed in the amplicon sequences of this assay, clearly demonstrates how the accumulation of mutations in some parts of the SARS-CoV-2 genome can dramatically affect the specificity of an RT-qPCR test. In comparison, the 10 other evaluated primers and probe sets, including those from the widely used tests developed by the Charité Hospital, Institut Pasteur Paris and US CDC, retained their high inclusivity above 99%. The Assay 8 S, determined as the best assay during our first evaluation, showed the second highest inclusivity result (99.97%) after Assay 2 E (99.99%), with a high level of amplicon conservation, illustrated by one major cluster containing 98.6% of the amplified sequences.

The data generated with SCREENED, for the 11 primers and probe sets evaluated in this study, showed how the virus evolution can potentially impact the performance of RT-qPCR assays used for SARS-CoV-2 detection. Recently, this virus evolution took a new turn with the emergence of new SARS-CoV-2 variants. At the time we started this analysis, three main new variants, i.e., 20I/501Y.V1, 20H/501Y.V2, and 20J/501Y.V3, were reported to carry an abnormal number of mutations, with some resulting in an estimated enhanced transmissibility and concerns about the effects on the immunological response and vaccine efficiency [32,33,34,38,40]. Four of these mutations, among which two are of concern because they effectuate amino acid changes (K417T and K417N) in the RBD of the S protein, were found to be located in oligonucleotide sequences of the China CDC assay targeting gene N (Assay 1 N) and the Chan et al. assay targeting gene S (Assay 8 S) evaluated in this study. Although these mutations were not estimated to cause a total test failure, they might affect the sensitivity of the RT-qPCR. Furthermore, it cannot be excluded that the variants will continue to evolve and acquire additional nucleotide changes, which can impact the test’s performance when combined to their lineage-specific mutations. This was already shown in the present study for four 20I/501Y.V1 genomes in which the C28977T variant mutation was combined with other nucleotide changes, leading to FN results. Additionally, some nucleotide changes previously acquired by the variant lineages (such as the GGG to AAC change in the Assay 1 N forward sequence), and not specific to those variants, can also lead to FN results. Therefore, when evaluating if primers and probe sets can still cover variant detection, it is also important to consider all the nucleotide changes that can be present in the variant population, as done in the present study. As soon as more variant genomes, especially belonging to 20H/501Y.V2 and 20J/501Y.V3, will be available in the database, the current analytical procedure should be reproduced, preferably with dedicated datasets per variants and in a more dynamic set-up (e.g., dataset per variant, per month). Furthermore, with the efforts made to improve the global surveillance of circulating SARS-CoV-2 strains, an increasing number of VOCs will be identified and genomes belonging to these should be included in future in silico specificity evaluation study as well.

The most accurate RT-qPCR test is required for proper detection of SARS-CoV-2, including its variants of concern for COVID-19 diagnosis and SARS-CoV-2 surveillance. Concerning the assays for which a low inclusivity was obtained (Assay 1 N) or for which mutations belonging to variants was identified in the sequence of their oligonucleotides (Assay 1 N and Assay 8 S), it could be considered to correct these to improve their specificity for SARS-CoV-2 detection. However, this would require modifying the concerned primers and probes with degenerated nucleotides (taking into account all the possibilities; Supplementary Material File S5), with the aim to be specific to both SARS-CoV-2 variants and historical strains and to validate these new assays experimentally. To avoid this extra work, and also to avoid to target regions affected by mutations, we would rather recommend to use Assay 2 E and Assay 3 RdRp_IP4, determined as the best ones based on the data obtained in this study. These 2 assays showed an excellent inclusivity, a high level of conservation in their amplicon sequence, and no variant mutations in the annealing site of their primers and probes. Assay 3 RdRp_IP4 was initially designed to be strictly specific to SARS-CoV-2, whereas Assay 2 E has a broader intended specificity to *Sarbecovirus* and is usually used for results’ confirmation [14,46]. These 2 assays could be included in a dual-target RT-qPCR test for reliable detection of SARS-CoV-2, also considering that the use of 2 molecular markers in a RT-qPCR test is usually recommended to lower the probability of incorrect results in case of mutational drift in one of the targets. Moreover, RT-qPCR detection is also a good approach for SARS-CoV-2 variants surveillance and a good alternative to WGS that is more expensive and time consuming. This surveillance is definitely needed to monitor the spread of variants having mutations potentially impacting vaccine efficiency, which might increase once the vaccination reaches full speed. However, it would be more meaningful to develop RT-qPCR assays targeting some key mutations, e.g., E484K and N501Y, that have been already identified as being of concern based on epidemiological and experimental data [38,40], rather than identifying the variants themselves according to their lineage or region of emergence. This would also be more efficient as more variant lineages, and other VOC, are expected to emerge in the future [60]. To develop such tests, SCREENED can be employed to produce evidence-based data from thousands of SARS-CoV-2 genomes available in NCBI and GISAID, to evaluate the in silico specificity of the designed primers and probes targeting mutations of concern (or significant mutations).

In the present study, potential FN RT-qPCR results were predicted by SCREENED, based on mismatch scores, for oligonucleotide sets coming from publicly available assays, which we previously evaluated in the first phase of the pandemic. We re-evaluated some of these assays, as a proof-of-concept, to assess the impact of the evolution of the pandemic, and hence the evolution of the virus, on the inclusivity of those RT-qPCR assays. This was possible as for these RT-qPCRs, their full sequences have been described. However, COVID-19 diagnosis at large scale is usually done in many laboratories with commercial diagnostic kits. Considering the results obtained in our study for some publicly available assays, it would not be surprising that SARS-CoV-2 mutations can also impact the performance of commercial assays, as suggested by some data in the scientific literature. For instance, the presence of the SARS-CoV-2 mutations S:Δ69–70 deletion (specific to the 20I/501Y.V1 variant) and E:C26340T was demonstrated to be strongly associated with detection failure of the TaqPath™ COVID-19 kit (Thermo Fisher) [30] and Cobas SARS-CoV-2 assay (Roche) [61] for one of their corresponding targets (S and E), respectively. Therefore, it is highly suspected that these mutations are responsible for mismatches between primers and probes of these kits, resulting in FN results. Unfortunately, these kinds of assumptions are difficult to demonstrate. Indeed, unlike the publicly available methods that are well described in the scientific literature, commercial kits are usually black boxes with only the targeted SARS-CoV-2 genes known, and neither the corresponding sequences of the oligonucleotides nor the exact location of their annealing sites in the viral genome are specified, even when asked to the kits’ manufacturers [61]. Information on which and how many genomes were used to verify the specificity of the primers and probes during the validation process is also often very limited. Consequently, when there is a suspicion of FN results obtained with a commercial kit because of mutations in the SARS-CoV-2 genome, this cannot be completely verified, even though this could be meaningful to avoid further inaccurate diagnoses. Additionally, the lack of communication on the primers and probe sequences included in the kits makes the specificity assessment, such as performed with SCREENED in the present study, of these commercial methods by external and independent laboratories nearly impossible. It can also not be properly assessed whether a failure in the test might be the result of a modified inclusivity of those primers and probe sets because of the evolving virus or because of possible unwanted mismatches in the primers and probes introduced during the synthesis process. Although the latter is less likely, given the quality control systems in place at the commercial vendors, the inclusion of a positive control for each RT-qPCR assay will be required to elucidate this. Next to this, standardized and reference materials are not always available for the kit’s manufacturers to properly evaluate the specificity of their products in the wet-lab. Fortunately, the S-drop out of the TaqPath ™ COVID-19 kit could be used as a proxy for the 20I/501Y.V1 variant detection, as still two other SARS-CoV-2 specific genes were detected by the kit. Nevertheless, considering the situation highlighted above, it would be recommended that commercial companies, if not publicly disclosing their primer and probe sequences, (continue to) collaborate more intensively with public health organizations (such as the WHO) to regularly evaluate if their assays are impacted by SARS-CoV-2 mutations. A central repository, containing the sequences of all the commercial and publicly available primers and probe sets used for SARS-CoV-2 detection, could be created and made available to a central RT-qPCR evaluation team composed of a panel of scientific experts with testing expertise worldwide. This team would be in charge of the regular evaluation of the sets, including the monitoring of mutations of concern in the oligonucleotide sequences. It could then be communicated to the clinical laboratories and public health community if some kits were or should be adapted to take the virus evolution into account for accurate detection.

In conclusion, the data presented in this study show the importance of regularly assessing the impact of SARS-CoV-2 evolution on the performance of RT-qPCR assays widely used as the gold-standard method for COVID-19 diagnosis, especially in the context of new emerging variants accumulating high numbers of mutations. This can easily be done in a first step by identifying potentially impacting mismatches using bioinformatics tools, such as SCREENED or others, and WGS data being deposited on a daily basis in publicly available repositories. Of course, this in silico approach does not take into account all the other in vitro parameters that can affect PCR-like reactions. However, this preliminary in silico analysis is valuable to know specifically what should be tested in the laboratory, in a second step, to confirm experimentally the effect of these mismatches on RT-qPCR performance. Nevertheless, only the RT-qPCR assays fully described in the scientific literature can be evaluated in this manner and not the commercial kits commonly used for COVID-19 testing at large scale because these remain black boxes. This situation is unfortunate, now more than ever, as so-called “third waves” are threatening several countries in Europe [62]. This makes the correct detection of all circulating SARS-CoV-2 strains, including their emerging variants with eventually more dedicated VOC detection assays, crucial to limit their spread in the population.

## Figures and Tables

**Table 1:** List of investigated primers and probe sets.

Assay *	Target	Oligonucleotide Sequence (5’–3’)	Length (bp)	Amplicon Location **	Source
1	N	Fw GGGGAACTTCTCCTGCTAGAAT Rv CAGACATTTTGCTCTCAAGCTG P TTGCTGCTGCTTGACAGATT	22 22 20	28880–28979	China CDC, China [14]
2	RdRp-P2	Fw GTGARATGGTCATGTGTGGCGG Rv CARATGTTAAASACACTATTAGCATA P CAGGTGGAACCTCATCAGGAGATGC	22 26 25	15430–15530	Charité Hospital, Germany [14]
2 ***	E ***	Fw ACAGGTACGTTAATAGTTAATAGCGT Rv ATATTGCAGCAGTACGCACACA P ACACTAGCCATCCTTACTGCGCTTCG	26 22 26	26268–26381	
2	N	Fw CACATTGGCACCCGCAATC Rv GAGGAACGAGAAGAGGCTTG P ACTTCCTCAAGGAACAACATTGCCA	19 20 25	28705–28833	
3	RdRp_IP2	Fw ATGAGCTTAGTCCTGTTG Rv CTCCCTTTGTTGTGTTGT P AGATGTCTTGTGCTGCCGGTA	18 18 21	12689–12797	Institut Pasteur Paris, France [14]
3	RdRp_IP4	Fw GGTAACTGGTATGATTTCG Rv CTGGTCAAGGTTAATATAGG P TCATACAAACCACGCCAGG	19 20 19	14079–14186	
4	N-1	Fw GACCCCAAAATCAGCGAAAT Rv TCTGGTTACTGCCAGTTGAATCTG P ACCCCGCATTACGTTTGGTGGACC	20 24 24	28286–28358	US CDC, USA [14]
4	N-2	Fw TTACAAACATTGGCCGCAAA Rv GCGCGACATTCCGAAGAA P ACAATTTGCCCCCAGCGCTTCAG	20 18 23	29163–29230	
4	N-3	Fw GGGAGCCTTGAATACACCAAAA Rv ACAATTTGCCCCCAGCGCTTCAG P AYCACATTGGCACCCGCAATCCTG	22 23 24	28680–28752	
8	S	Fw CCTACTAAATTAAATGATCTCTGCTTTACT Rv CAAGCTATAACGCAGCCTGTA P CGCTCCAGGGCAAACTGGAAAG	30 21 22	22711–22869	Chan et al. [50]
9	ORF1a	Fw AGAAGATTGGTTAGATGATGATAGT Rv TTCCATCTCTAATTGAGGTTGAACC P TCCTCACTGCCGTCTTGTTGACCA	25 25 24	3192–3310	Lu et al. [51]

*: to allow comparisons with the study of Gand et al., 2020 [46], the assay numbering as used in this previous study was conserved. **: starting and ending position of the sequence amplified by the corresponding forward and reverse primers, in the NCBI SARS-CoV-2 reference sequence NC_045512. ***: this primers and probe set is also used in the RT-qPCR test from Institut Pasteur Paris (France) [14]. Fw: forward primer; Rv: reverse primer; P: probe.

**Table 2:** Inclusivity evaluation of primers and probe sets using polymeraSe Chain Reaction Evaluation through largE-scale miNing of gEnomic Data (SCREENED).

Assay	Target	Negative RT-qPCR Result ^†,^*	Inclusivity (Present Study *)	Inclusivity (Previous Study **)
1	N	30,445 (13%)	63.89%	86.03%
2	RdRp-P2	96 (9.4%)	99.89%	100%
2	E	8 (0%)	99.99%	100%
2	N	316 (2.2%)	99.63%	99.81%
3	RdRp_IP2	169 (6.5%)	99.80%	99.88%
3	RdRp_IP4	44 (0%)	99.95%	100%
4	N-1	181 (2.2%)	99.79%	99.73%
4	N-2	833 (0%)	99.01%	99.96%
4	N-3	100 (1%)	99.88%	100%
8	S	28 (3.6%)	99.97%	100%
9	ORF1a	95 (0%)	99.89%	100%

^†^: number of representative genomes that produced a theoretical negative RT-qPCR signal according to the SCREENED settings (detailed in Section 2.4) and, consequently, considered as false negative (FN). The percentage of the genomes resulting in negative results and belonging to one of the new variants is indicated between brackets. All turned out to belong to the B.1.1.7 lineage (20I/501Y.V1). * results obtained in the present study with 84,305 representative SARS-CoV-2 genomes collected between 7 June 2020 and 7 January 2021. ** results obtained in the previous study with 2569 representative SARS-CoV-2 genomes collected up to 7 April 2020 [46].

**Table 3:** Diversity among the sequences amplified by the evaluated primers and probe sets.

Assay	1	2	2	2	3	3	4	4	4	8	9
Target	N	RdRp-P2	E	N	RdRp _IP2	RdRp _IP4	N-1	N-2	N-3	S	ORF1a
Clusters *	496	89	116	291	157	124	199	123	152	160	231
First cluster **	30.3%	97.5%	98.2%	94.7%	98.4%	98.4%	96.1%	95.7%	97.1%	98.6%	91.1%
Second cluster **	28.8%	1.3%	0.5%	0.7%	0.2%	0.3%	0.5%	1.8%	0.7%	0.2%	4.8%
Third cluster **	26.9%	0.4%	0.4%	0.6%	0.1%	0.1%	0.4%	0.9%	0.2%	0.1%	1.7%

*: number of amplicon clusters produced by SCREENED for each evaluated primers and probe set. **: Repartition of the number of amplicons among the clusters for the 3 largest clusters for each evaluated primers and probe set.

**Table 4:** Presence of severe acute respiratory syndrome coronavirus 2 (SARS-CoV-2) variants’ mutations in the sequence amplified by the evaluated primers and probe sets.

Assay	Target	Amplicon Start Pos. *	Amplicon End Pos. *	Nucleotide Change*	Amino Acid Change	Impact on Primer or Probe Sequences (5’–3’) **	FN Results ***
9	ORF1a	3192	3310	C3267T ^v1^	T1001I	None	No
1	N	28880	28979	C28977T ^v1^	S235F	Rv CA**A**ACATTTTGCTCTCAAGCTG	Depending on other mutations
8	S	22711	22869	G22813T ^v2^	K417N	P CGCTCCAGGGCAAACTGGAAA**T**	No
1	N	28880	28979	C28887T ^v2^	T205I	Fw GGGGAA**T**TTCTCCTGCTAGAAT	No
3	RdRp_IP2	12689	12797	C12778T ^v3^	Synonymous	None	No
8	S	22711	22869	A22812C ^v3^	K417T	P CGCTCCAGGGCAAACTGGAA**C**G	No

*: positions were determined according to SARS-CoV-2 reference sequence NC_045512.2. **: The nucleotide change is indicated in the primer or probe sequence. If “none” is indicated, the nucleotide change is located in the amplicon sequence but outside primers and probe annealing sites. ***: a “No” is indicated if no mutation was detected in the annealing site or if a mutation in the annealing site would not lead to a FN result according to the criteria applied in SCREENED (see Section 2.4). A “Depending on other mutations” is indicated if a mutation in the annealing site would result in a FN result when combined with another mismatch identified with SCREENED (Supplementary Material File S1). ^v1^: Mutation in 20I/501Y.V1 variant [30,32]; ^v2^: mutation in 20H/501Y.V2 variant [33]; ^v3^: mutation in variant 20J/501Y.V3 [34]; Fw: forward primer; Rv: reverse primer; P: probe.

## Data Availability

Publicly available datasets were analyzed in this study. This data can be found here: https://www.ncbi.nlm.nih.gov/labs/virus/, (accessed on 7 January 2021), (accession numbers available in Supplementary Materials File S1) and https://www.epicov.org, (accessed on 7 January 2021), (accession numbers available in Supplementary Materials File S2). The data presented in this study are available within the text and in Supplementary Materials. The detailed SCREENED output data are available upon request.

## Supplementary Materials

The following are available online at https://www.mdpi.com/article/10.3390/genes12040565/s1, Supplementary Materials File S1: NCBI genomes and their Pangolin lineage; Supplementary Materials File S2: Global Initiative on Sharing Avian Influenza Data (GISAID) genomes and their metadata; Supplementary Materials File S3: Representative SARS-CoV-2 genomes used in polymeraSe Chain Reaction Evaluation through largE-scale miNing of gEnomic Data (SCREENED); Supplementary Materials File S4: Primer, probe, and reference amplicon sequences for evaluation with SCREENED; Supplementary Materials File S5: Nucleotide modifications in the evaluated primer and probe sequences resulting in a negative theoretical RT-qPCR signal with SCREENED; Supplementary Materials File S6: List of genomes belonging to the second SCREENED cluster of Assay 9 ORF1a; Supplementary Materials File S7: List of mutations defining the SARS-CoV-2 variants 20I/501Y.V1, 20H/501Y.V2, and 20J/501Y.V3; Supplementary Materials File S8: Evaluation of Assay 1 N reverse primer with EPI_ISL_744131 using BLAST; Supplementary Materials File S9: GISAID acknowledgement table.
