# Supplementary material for: Deepening of In Silico Evaluation of SARS-CoV-2 Detection RT-qPCR Assays in the Context of New Variants
Source: Genes (Basel). 2021 Apr 13;12(4):565. doi: 10.3390/genes12040565 (PMC8069896; doi:10.3390/genes12040565)
Supplement: Supplementary file 1 [file genes-12-00565-s001.zip › Supplementary file S8.docx]

**Supplementary file S6**

In the present study, the impact of variants' mutations on publicly available RT-qPCR assays for SARS-CoV-2 detection was evaluated. Amongst other identified mismatches, the C28977T mutation specific to 20I/501Y.V1 was found to be located in the annealing site of the reverse primer of Assay 1 (China CDC) N (section 3.2), as reported by SCREENED (Supplementary file S5). In a similar previously reported study, the Basic Local Alignment Search Tool (BLAST; <https://blast.ncbi.nlm.nih.gov/Blast.cgi>) was used to evaluate the presence of mismatches between the same primer from the China CDC assay and 20I/501Y.V1 genome EPI_ISL_744131 (GISAID) [39]. Surprisingly, the authors of this study reported a perfect match between the reverse primer of the China CDC assay targeting the gene N and EPI_ISL_744131, which contradict our results identifying C28977T to be present in the annealing site of this oligonucleotide. Therefore, to better understand the reason of these inconsistent results, the same BLAST analysis [39] was performed with the identical genome and settings. The output result can be found below:

Query: Assay 1 N (China CDC) Reverse sequence

Query ID: lcl|Query_33383 Length: 22

>hCoV-19/Wales/PHWC-4A3742/2020|EPI_ISL_744131|2020-12-15

Sequence ID: Query_33385 Length: 29881

Range 1: 28936 to 28954

Score:38.2 bits(19), Expect:1e-06,

Identities:19/19(100%), Gaps:0/19(0%), Strand: Plus/Minus

Query 4 ACATTTTGCTCTCAAGCTG 22

|||||||||||||||||||

Sbjct 28954 ACATTTTGCTCTCAAGCTG 28936

The BLAST output shows the alignment results for the last 19 nucleotides of the Assay 1 N reverse primer, with a perfect match. Nevertheless, the total length of Assay 1 N reverse primer is 22 nucleotides and the perfect match obtained with BLAST concerned only the nucleotide from position 4 to 22. The C28977T mismatch was not displayed in the BLAST output results because located in the 3 first nucleotides of the primer's 5' end, at the third position (cfr. query starting at position 4 instead of 1). However, this mismatch was correctly identified in our study using SCREENED.
