# Supplementary material for: Deepening of In Silico Evaluation of SARS-CoV-2 Detection RT-qPCR Assays in the Context of New Variants
Source: Genes (Basel). 2021 Apr 13;12(4):565. doi: 10.3390/genes12040565 (PMC8069896; doi:10.3390/genes12040565)
Supplement: Supplementary file 1 [file genes-12-00565-s001.zip › Supplementary file S9.zip!/Acknowledgement tables/gisaid_hcov-19_acknowledgement_table_10.pdf]

We gratefully acknowledge the following Authors from the Originating laboratories responsible for obtaining the specimens, as well as the Submitting laboratories where the genome data were generated and shared via GISAID, on which this research is based.

All Submitters of data may be contacted directly via www.gisaid.org

Authors are sorted alphabetically.

Accession ID	Originating Laboratory	Submitting Laboratory	Authors
EPI_ISL_629221, EPI_ISL_629222, EPI_ISL_629223, EPI_ISL_629224, EPI_ISL_629225, EPI_ISL_629226, EPI_ISL_629227, EPI_ISL_629228, EPI_ISL_629229, EPI_ISL_629230, EPI_ISL_629231, EPI_ISL_629232, EPI_ISL_629233, EPI_ISL_629234, EPI_ISL_629235, EPI_ISL_629236, EPI_ISL_629237, EPI_ISL_629238, EPI_ISL_629239, EPI_ISL_629240, EPI_ISL_629241, EPI_ISL_629242, EPI_ISL_629243, EPI_ISL_629244			
see above	Lighthouse Lab in Milton Keynes	Wellcome Sanger Institute for the COVID-19 Genomics UK (COG-UK) consortium	The Lighthouse Lab in Milton Keynes and Alex Alderton, Roberto Amato, Sonia Goncalves, Ewan Harrison, David K. Jackson, Ian Johnston, Dominic Kwiatkowski, Cordelia Langford, John Sillitoe on behalf of the Wellcome Sanger Institute COVID-19 Surveillance Team
EPI_ISL_629245	Lighthouse Lab in Milton Keynes	Wellcome Sanger Institute for the COVID-19 Genomics UK (COG-UK) Consortium	The Lighthouse Lab in Milton Keynes and Alex Alderton, Roberto Amato, Sonia Goncalves, Ewan Harrison, David K. Jackson, Ian Johnston, Dominic Kwiatkowski, Cordelia Langford, John Sillitoe on behalf of the Wellcome Sanger Institute COVID-19 Surveillance Team
EPI_ISL_629246, EPI_ISL_629247, EPI_ISL_629248, EPI_ISL_629249, EPI_ISL_629250, EPI_ISL_629251, EPI_ISL_629252, EPI_ISL_629253, EPI_ISL_629254, EPI_ISL_629255, EPI_ISL_629256, EPI_ISL_629257, EPI_ISL_629258, EPI_ISL_629259, EPI_ISL_629260, EPI_ISL_629261, EPI_ISL_629262, EPI_ISL_629263, EPI_ISL_629264, EPI_ISL_629265, EPI_ISL_629266, EPI_ISL_629267, EPI_ISL_629268, EPI_ISL_629269, EPI_ISL_629270, EPI_ISL_629271, EPI_ISL_629272, EPI_ISL_629273, EPI_ISL_629274, EPI_ISL_629275, EPI_ISL_629276, EPI_ISL_629277, EPI_ISL_629278, EPI_ISL_629279, EPI_ISL_629280, EPI_ISL_629281, EPI_ISL_629282, EPI_ISL_629283, EPI_ISL_629284, EPI_ISL_629285, EPI_ISL_629286, EPI_ISL_629287, EPI_ISL_629288, EPI_ISL_629289, EPI_ISL_629290, EPI_ISL_629291, EPI_ISL_629292, EPI_ISL_629293, EPI_ISL_629294, EPI_ISL_629295, EPI_ISL_629296, EPI_ISL_629297, EPI_ISL_629298, EPI_ISL_629299, EPI_ISL_629300, EPI_ISL_629301, EPI_ISL_629302, EPI_ISL_629303, EPI_ISL_629304, EPI_ISL_629305, EPI_ISL_629306, EPI_ISL_629307, EPI_ISL_629308, EPI_ISL_629309, EPI_ISL_629310, EPI_ISL_629311, EPI_ISL_629312, EPI_ISL_629313, EPI_ISL_629314, EPI_ISL_629315, EPI_ISL_629316, EPI_ISL_629317, EPI_ISL_629318, EPI_ISL_629319, EPI_ISL_629320, EPI_ISL_629321, EPI_ISL_629322, EPI_ISL_629323, EPI_ISL_629324, EPI_ISL_629325, EPI_ISL_629326, EPI_ISL_629327, EPI_ISL_629328, EPI_ISL_629329, EPI_ISL_629330, EPI_ISL_629331, EPI_ISL_629332, EPI_ISL_629333, EPI_ISL_629334, EPI_ISL_629335, EPI_ISL_629336, EPI_ISL_629337, EPI_ISL_629338, EPI_ISL_629339, EPI_ISL_629340, EPI_ISL_629341, EPI_ISL_629342, EPI_ISL_629343, EPI_ISL_629344, EPI_ISL_629345, EPI_ISL_629346, EPI_ISL_629347, EPI_ISL_629348, EPI_ISL_629349, EPI_ISL_629350, EPI_ISL_629351, EPI_ISL_629352, EPI_ISL_629353, EPI_ISL_629354, EPI_ISL_629355, EPI_ISL_629356, EPI_ISL_629357, EPI_ISL_629358, EPI_ISL_629359, EPI_ISL_629360, EPI_ISL_629361, EPI_ISL_629362, EPI_ISL_629363, EPI_ISL_629364, EPI_ISL_629365, EPI_ISL_629366, EPI_ISL_629367, EPI_ISL_629368, EPI_ISL_629369, EPI_ISL_629370, EPI_ISL_629371, EPI_ISL_629372, EPI_ISL_629373, EPI_ISL_629374, EPI_ISL_629375, EPI_ISL_629376, EPI_ISL_629377, EPI_ISL_629378, EPI_ISL_629379, EPI_ISL_629380, EPI_ISL_629381, EPI_ISL_629382, EPI_ISL_629383, EPI_ISL_629384, EPI_ISL_629385, EPI_ISL_629386, EPI_ISL_629387, EPI_ISL_629388, EPI_ISL_629389, EPI_ISL_629390, EPI_ISL_629391, EPI_ISL_629392, EPI_ISL_629393, EPI_ISL_629394, EPI_ISL_629395, EPI_ISL_629396, EPI_ISL_629397, EPI_ISL_629398, EPI_ISL_629399, EPI_ISL_629400, EPI_ISL_629401, EPI_ISL_629402, EPI_ISL_629403, EPI_ISL_629404, EPI_ISL_629405, EPI_ISL_629406, EPI_ISL_629407, EPI_ISL_629408			
see above	Lighthouse Lab in Milton Keynes	Wellcome Sanger Institute for the COVID-19 Genomics UK (COG-UK) consortium	The Lighthouse Lab in Milton Keynes and Alex Alderton, Roberto Amato, Sonia Goncalves, Ewan Harrison, David K. Jackson, Ian Johnston, Dominic Kwiatkowski, Cordelia Langford, John Sillitoe on behalf of the Wellcome Sanger Institute COVID-19 Surveillance Team
EPI_ISL_629409	Lighthouse Lab in Milton Keynes	Wellcome Sanger Institute for the COVID-19 Genomics UK (COG-UK) Consortium	The Lighthouse Lab in Milton Keynes and Alex Alderton, Roberto Amato, Sonia Goncalves, Ewan Harrison, David K. Jackson, Ian Johnston, Dominic Kwiatkowski, Cordelia Langford, John Sillitoe on behalf of the Wellcome Sanger Institute COVID-19 Surveillance Team
EPI_ISL_629410, EPI_ISL_629411, EPI_ISL_629412, EPI_ISL_629413, EPI_ISL_629414, EPI_ISL_629415, EPI_ISL_629416, EPI_ISL_629417, EPI_ISL_629418, EPI_ISL_629419, EPI_ISL_629420, EPI_ISL_629421, EPI_ISL_629422, EPI_ISL_629423, EPI_ISL_629424, EPI_ISL_629425, EPI_ISL_629426, EPI_ISL_629427, EPI_ISL_629428, EPI_ISL_629429, EPI_ISL_629430, EPI_ISL_629431, EPI_ISL_629432, EPI_ISL_629433, EPI_ISL_629434, EPI_ISL_629435, EPI_ISL_629436, EPI_ISL_629437, EPI_ISL_629438, EPI_ISL_629439, EPI_ISL_629440, EPI_ISL_629441, EPI_ISL_629442, EPI_ISL_629443, EPI_ISL_629444, EPI_ISL_629445, EPI_ISL_629446, EPI_ISL_629447, EPI_ISL_629448, EPI_ISL_629449, EPI_ISL_629451, EPI_ISL_629452, EPI_ISL_629453, EPI_ISL_629455, EPI_ISL_629456, EPI_ISL_629457, EPI_ISL_629458, EPI_ISL_629459, EPI_ISL_629460, EPI_ISL_629461, EPI_ISL_629462, EPI_ISL_629463, EPI_ISL_629464, EPI_ISL_629465, EPI_ISL_629466, EPI_ISL_629467, EPI_ISL_629468, EPI_ISL_629469, EPI_ISL_629470, EPI_ISL_629471, EPI_ISL_629472, EPI_ISL_629473, EPI_ISL_629474, EPI_ISL_629475, EPI_ISL_629476, EPI_ISL_629477, EPI_ISL_629478, EPI_ISL_629479, EPI_ISL_629480, EPI_ISL_629481, EPI_ISL_629482, EPI_ISL_629483, EPI_ISL_629485, EPI_ISL_629486, EPI_ISL_629487, EPI_ISL_629488, EPI_ISL_629489			
see above	Lighthouse Lab in Milton Keynes	Wellcome Sanger Institute for the COVID-19 Genomics UK (COG-UK) consortium	The Lighthouse Lab in Milton Keynes and Alex Alderton, Roberto Amato, Sonia Goncalves, Ewan Harrison, David K. Jackson, Ian Johnston, Dominic Kwiatkowski, Cordelia Langford, John Sillitoe on behalf of the Wellcome Sanger Institute COVID-19 Surveillance Team
EPI_ISL_629490	Lighthouse Lab in Milton Keynes	Wellcome Sanger Institute for the COVID-19 Genomics UK (COG-UK) Consortium	The Lighthouse Lab in Milton Keynes and Alex Alderton, Roberto Amato, Sonia Goncalves, Ewan Harrison, David K. Jackson, Ian Johnston, Dominic Kwiatkowski, Cordelia Langford, John Sillitoe on behalf of the Wellcome Sanger Institute COVID-19 Surveillance Team
EPI_ISL_629491, EPI_ISL_629492, EPI_ISL_629493, EPI_ISL_629494, EPI_ISL_629495, EPI_ISL_629496, EPI_ISL_629497, EPI_ISL_629498, EPI_ISL_629499, EPI_ISL_629500, EPI_ISL_629501, EPI_ISL_629502, EPI_ISL_629503, EPI_ISL_629504, EPI_ISL_629505, EPI_ISL_629506, EPI_ISL_629507, EPI_ISL_629508, EPI_ISL_629509, EPI_ISL_629510, EPI_ISL_629511			
see above	Lighthouse Lab in Milton Keynes	Wellcome Sanger Institute for the COVID-19 Genomics UK (COG-UK) consortium	The Lighthouse Lab in Milton Keynes and Alex Alderton, Roberto Amato, Sonia Goncalves, Ewan Harrison, David K. Jackson, Ian Johnston, Dominic Kwiatkowski, Cordelia Langford, John Sillitoe on behalf of the Wellcome Sanger Institute COVID-19 Surveillance Team
EPI_ISL_629512	Lighthouse Lab in Milton Keynes	Wellcome Sanger Institute for the COVID-19 Genomics UK (COG-UK) Consortium	The Lighthouse Lab in Milton Keynes and Alex Alderton, Roberto Amato, Sonia Goncalves, Ewan Harrison, David K. Jackson, Ian Johnston, Dominic Kwiatkowski, Cordelia Langford, John Sillitoe on behalf of the Wellcome Sanger Institute COVID-19 Surveillance Team
EPI_ISL_629513, EPI_ISL_629514, EPI_ISL_629515, EPI_ISL_629516, EPI_ISL_629517, EPI_ISL_629518, EPI_ISL_629519, EPI_ISL_629521, EPI_ISL_629522, EPI_ISL_629523, EPI_ISL_629524, EPI_ISL_629525			
see above	Lighthouse Lab in Milton Keynes	Wellcome Sanger Institute for the COVID-19 Genomics UK (COG-UK) consortium	The Lighthouse Lab in Milton Keynes and Alex Alderton, Roberto Amato, Sonia Goncalves, Ewan Harrison, David K. Jackson, Ian Johnston, Dominic Kwiatkowski, Cordelia Langford, John Sillitoe on behalf of the Wellcome Sanger Institute COVID-19 Surveillance Team
EPI_ISL_629526	Lighthouse Lab in Milton Keynes	Wellcome Sanger Institute for the COVID-19 Genomics UK (COG-UK) Consortium	The Lighthouse Lab in Milton Keynes and Alex Alderton, Roberto Amato, Sonia Goncalves, Ewan Harrison, David K. Jackson, Ian Johnston, Dominic Kwiatkowski, Cordelia Langford, John Sillitoe on behalf of the Wellcome Sanger Institute COVID-19 Surveillance Team
EPI_ISL_629527, EPI_ISL_629528, EPI_ISL_629529, EPI_ISL_629530, EPI_ISL_629531, EPI_ISL_629532, EPI_ISL_629533, EPI_ISL_629534, EPI_ISL_629535, EPI_ISL_629536, EPI_ISL_629537, EPI_ISL_629538, EPI_ISL_629539, EPI_ISL_629540, EPI_ISL_629541, EPI_ISL_629542, EPI_ISL_629543, EPI_ISL_629544, EPI_ISL_629545, EPI_ISL_629546, EPI_ISL_629547, EPI_ISL_629548, EPI_ISL_629549, EPI_ISL_629550, EPI_ISL_629551, EPI_ISL_629552, EPI_ISL_629554, EPI_ISL_629555, EPI_ISL_629556, EPI_ISL_629557, EPI_ISL_629558, EPI_ISL_629559, EPI_ISL_629560, EPI_ISL_629561, EPI_ISL_629562, EPI_ISL_629563, EPI_ISL_629564, EPI_ISL_629565, EPI_ISL_629566, EPI_ISL_629567, EPI_ISL_629568, EPI_ISL_629569, EPI_ISL_629570, EPI_ISL_629571, EPI_ISL_629572, EPI_ISL_629573, EPI_ISL_629574, EPI_ISL_629575, EPI_ISL_629576, EPI_ISL_629577, EPI_ISL_629578, EPI_ISL_629579			
see above	Lighthouse Lab in Milton Keynes	Wellcome Sanger Institute for the COVID-19 Genomics UK (COG-UK) consortium	The Lighthouse Lab in Milton Keynes and Alex Alderton, Roberto Amato, Sonia Goncalves, Ewan Harrison, David K. Jackson, Ian Johnston, Dominic Kwiatkowski, Cordelia Langford, John Sillitoe on behalf of the Wellcome Sanger Institute COVID-19 Surveillance Team
EPI_ISL_629580	Lighthouse Lab in Milton Keynes	Wellcome Sanger Institute for the COVID-19 Genomics UK (COG-UK) Consortium	The Lighthouse Lab in Milton Keynes and Alex Alderton, Roberto Amato, Sonia Goncalves, Ewan Harrison, David K. Jackson, Ian Johnston, Dominic Kwiatkowski, Cordelia Langford, John Sillitoe on behalf of the Wellcome Sanger Institute COVID-19 Surveillance Team
EPI_ISL_629581, EPI_ISL_629582, EPI_ISL_629584, EPI_ISL_629585, EPI_ISL_629586, EPI_ISL_629587, EPI_ISL_629588, EPI_ISL_629589	Lighthouse Lab in Milton Keynes	Wellcome Sanger Institute for the COVID-19 Genomics UK (COG-UK) consortium	The Lighthouse Lab in Milton Keynes and Alex Alderton, Roberto Amato, Sonia Goncalves, Ewan Harrison, David K. Jackson, Ian Johnston, Dominic Kwiatkowski, Cordelia Langford, John Sillitoe on behalf of the Wellcome Sanger Institute COVID-19 Surveillance Team
EPI_ISL_629590	Lighthouse Lab in Milton Keynes	Wellcome Sanger Institute for the COVID-19 Genomics UK (COG-UK) Consortium	The Lighthouse Lab in Milton Keynes and Alex Alderton, Roberto Amato, Sonia Goncalves, Ewan Harrison, David K. Jackson, Ian Johnston, Dominic Kwiatkowski, Cordelia Langford, John Sillitoe on behalf of the Wellcome Sanger Institute COVID-19 Surveillance Team
EPI_ISL_629591	Lighthouse Lab in Milton Keynes	Wellcome Sanger Institute for the COVID-19 Genomics UK (COG-UK) consortium	The Lighthouse Lab in Milton Keynes and Alex Alderton, Roberto Amato, Sonia Goncalves, Ewan Harrison, David K. Jackson, Ian Johnston, Dominic Kwiatkowski, Cordelia Langford, John Sillitoe on behalf of the Wellcome Sanger Institute COVID-19 Surveillance Team
EPI_ISL_629592	Lighthouse Lab in Milton Keynes	Wellcome Sanger Institute for the COVID-19 Genomics UK (COG-UK) Consortium	The Lighthouse Lab in Milton Keynes and Alex Alderton, Roberto Amato, Sonia Goncalves, Ewan Harrison, David K. Jackson, Ian Johnston, Dominic Kwiatkowski, Cordelia Langford, John Sillitoe on behalf of the Wellcome Sanger Institute COVID-19 Surveillance Team
EPI_ISL_629593, EPI_ISL_629594, EPI_ISL_629595, EPI_ISL_629596, EPI_ISL_629597, EPI_ISL_629598, EPI_ISL_629599, EPI_ISL_629600, EPI_ISL_629601, EPI_ISL_629602, EPI_ISL_629603, EPI_ISL_629604, EPI_ISL_629605, EPI_ISL_629606, EPI_ISL_629608, EPI_ISL_629609, EPI_ISL_629610, EPI_ISL_629611, EPI_ISL_629612, EPI_ISL_629613, EPI_ISL_629614, EPI_ISL_629615, EPI_ISL_629616, EPI_ISL_629617, EPI_ISL_629618, EPI_ISL_629619, EPI_ISL_629620, EPI_ISL_629621, EPI_ISL_629622, EPI_ISL_629623, EPI_ISL_629624, EPI_ISL_629625, EPI_ISL_629626, EPI_ISL_629627, EPI_ISL_629628, EPI_ISL_629629, EPI_ISL_629630, EPI_ISL_629631, EPI_ISL_629633, EPI_ISL_629634, EPI_ISL_629635, EPI_ISL_629636, EPI_ISL_629637, EPI_ISL_629638, EPI_ISL_629639, EPI_ISL_629640, EPI_ISL_629641, EPI_ISL_629642, EPI_ISL_629643, EPI_ISL_629644, EPI_ISL_629645, EPI_ISL_629646, EPI_ISL_629647, EPI_ISL_629648, EPI_ISL_629649, EPI_ISL_629650, EPI_ISL_629651, EPI_ISL_629652, EPI_ISL_629653, EPI_ISL_629654, EPI_ISL_629655, EPI_ISL_629656, EPI_ISL_629657, EPI_ISL_629658, EPI_ISL_629659, EPI_ISL_629660, EPI_ISL_629661, EPI_ISL_629662, EPI_ISL_629663, EPI_ISL_629664, EPI_ISL_629665, EPI_ISL_629666, EPI_ISL_629667, EPI_ISL_629669, EPI_ISL_629670, EPI_ISL_629671, EPI_ISL_629673, EPI_ISL_629674, EPI_ISL_629675, EPI_ISL_629676, EPI_ISL_629677, EPI_ISL_629678, EPI_ISL_629679, EPI_ISL_629680, EPI_ISL_629681, EPI_ISL_629682, EPI_ISL_629683, EPI_ISL_629684, EPI_ISL_629685, EPI_ISL_629686,			

EPI_ISL_629687, EPI_ISL_629688, EPI_ISL_629689, EPI_ISL_629690, EPI_ISL_629691, EPI_ISL_629692, EPI_ISL_629693, EPI_ISL_629694, EPI_ISL_629695, EPI_ISL_629696, EPI_ISL_629697, EPI_ISL_629698, EPI_ISL_629699, EPI_ISL_629700, EPI_ISL_629701, EPI_ISL_629702, EPI_ISL_629703, EPI_ISL_629704, EPI_ISL_629705, EPI_ISL_629706, EPI_ISL_629707, EPI_ISL_629708, EPI_ISL_629709, EPI_ISL_629711, EPI_ISL_629712, EPI_ISL_629713, EPI_ISL_629714, EPI_ISL_629715, EPI_ISL_629716, EPI_ISL_629717, EPI_ISL_629718, EPI_ISL_629719, EPI_ISL_629720, EPI_ISL_629721, EPI_ISL_629722, EPI_ISL_629723, EPI_ISL_629724, EPI_ISL_629725, EPI_ISL_629726			
see above	Lighthouse Lab in Milton Keynes	Wellcome Sanger Institute for the COVID-19 Genomics UK (COG-UK) consortium	The Lighthouse Lab in Milton Keynes and Alex Alderton, Roberto Amato, Sonia Goncalves, Ewan Harrison, David K. Jackson, Ian Johnston, Dominic Kwiatkowski, Cordelia Langford, John Sillitoe on behalf of the Wellcome Sanger Institute COVID-19 Surveillance Team
EPI_ISL_629727	Lighthouse Lab in Milton Keynes	Wellcome Sanger Institute for the COVID-19 Genomics UK (COG-UK) Consortium	The Lighthouse Lab in Milton Keynes and Alex Alderton, Roberto Amato, Sonia Goncalves, Ewan Harrison, David K. Jackson, Ian Johnston, Dominic Kwiatkowski, Cordelia Langford, John Sillitoe on behalf of the Wellcome Sanger Institute COVID-19 Surveillance Team
EPI_ISL_629728, EPI_ISL_629729, EPI_ISL_629730, EPI_ISL_629731, EPI_ISL_629732, EPI_ISL_629733, EPI_ISL_629734, EPI_ISL_629735, EPI_ISL_629737, EPI_ISL_629738, EPI_ISL_629739, EPI_ISL_629740, EPI_ISL_629741, EPI_ISL_629742, EPI_ISL_629743, EPI_ISL_629744, EPI_ISL_629745, EPI_ISL_629746, EPI_ISL_629747, EPI_ISL_629748, EPI_ISL_629749, EPI_ISL_629750, EPI_ISL_629751, EPI_ISL_629752, EPI_ISL_629753			
see above	Lighthouse Lab in Milton Keynes	Wellcome Sanger Institute for the COVID-19 Genomics UK (COG-UK) consortium	The Lighthouse Lab in Milton Keynes and Alex Alderton, Roberto Amato, Sonia Goncalves, Ewan Harrison, David K. Jackson, Ian Johnston, Dominic Kwiatkowski, Cordelia Langford, John Sillitoe on behalf of the Wellcome Sanger Institute COVID-19 Surveillance Team
EPI_ISL_629754, EPI_ISL_629755, EPI_ISL_629756, EPI_ISL_629757, EPI_ISL_629758, EPI_ISL_629759, EPI_ISL_629760, EPI_ISL_629761, EPI_ISL_629762, EPI_ISL_629763, EPI_ISL_629764, EPI_ISL_629765, EPI_ISL_629766, EPI_ISL_629767, EPI_ISL_629768, EPI_ISL_629769, EPI_ISL_629770, EPI_ISL_629771, EPI_ISL_629772, EPI_ISL_629773, EPI_ISL_629774, EPI_ISL_629775, EPI_ISL_629776, EPI_ISL_629777, EPI_ISL_629778, EPI_ISL_629779, EPI_ISL_629780, EPI_ISL_629781, EPI_ISL_629782, EPI_ISL_629784, EPI_ISL_629785, EPI_ISL_629786, EPI_ISL_629787, EPI_ISL_629788, EPI_ISL_629789, EPI_ISL_629790, EPI_ISL_629791, EPI_ISL_629792, EPI_ISL_629793, EPI_ISL_629794, EPI_ISL_629795, EPI_ISL_629796, EPI_ISL_629797, EPI_ISL_629798, EPI_ISL_629799, EPI_ISL_629800, EPI_ISL_629801, EPI_ISL_629802, EPI_ISL_629803, EPI_ISL_629804, EPI_ISL_629805, EPI_ISL_629806, EPI_ISL_629807, EPI_ISL_629808, EPI_ISL_629809, EPI_ISL_629810, EPI_ISL_629811, EPI_ISL_629812, EPI_ISL_629813, EPI_ISL_629814, EPI_ISL_629815, EPI_ISL_629816, EPI_ISL_629817, EPI_ISL_629818, EPI_ISL_629819, EPI_ISL_629820, EPI_ISL_629821, EPI_ISL_629822, EPI_ISL_629823, EPI_ISL_629824, EPI_ISL_629825, EPI_ISL_629826, EPI_ISL_629827, EPI_ISL_629828, EPI_ISL_629829, EPI_ISL_629830, EPI_ISL_629831, EPI_ISL_629832, EPI_ISL_629833, EPI_ISL_629834, EPI_ISL_629835, EPI_ISL_629836, EPI_ISL_629837, EPI_ISL_629838, EPI_ISL_629839, EPI_ISL_629840, EPI_ISL_629841, EPI_ISL_629842, EPI_ISL_629843, EPI_ISL_629844, EPI_ISL_629845, EPI_ISL_629846, EPI_ISL_629847, EPI_ISL_629848, EPI_ISL_629849, EPI_ISL_629850, EPI_ISL_629851, EPI_ISL_629852, EPI_ISL_629853, EPI_ISL_629854, EPI_ISL_629855, EPI_ISL_629856, EPI_ISL_629857, EPI_ISL_629858, EPI_ISL_629859, EPI_ISL_629860, EPI_ISL_629861, EPI_ISL_629862, EPI_ISL_629863, EPI_ISL_629864, EPI_ISL_629865, EPI_ISL_629866, EPI_ISL_629867, EPI_ISL_629868, EPI_ISL_629869, EPI_ISL_629870, EPI_ISL_629871, EPI_ISL_629872, EPI_ISL_629873, EPI_ISL_629874, EPI_ISL_629875, EPI_ISL_629876, EPI_ISL_629878, EPI_ISL_629879, EPI_ISL_629880, EPI_ISL_629881, EPI_ISL_629882, EPI_ISL_629883, EPI_ISL_629884, EPI_ISL_629885, EPI_ISL_629886, EPI_ISL_629887, EPI_ISL_629888, EPI_ISL_629889, EPI_ISL_629890, EPI_ISL_629891, EPI_ISL_629892, EPI_ISL_629893, EPI_ISL_629894, EPI_ISL_629895, EPI_ISL_629896, EPI_ISL_629897, EPI_ISL_629898, EPI_ISL_629899, EPI_ISL_629900, EPI_ISL_629901, EPI_ISL_629902, EPI_ISL_629903, EPI_ISL_629904, EPI_ISL_629905, EPI_ISL_629906, EPI_ISL_629907, EPI_ISL_629908, EPI_ISL_629909, EPI_ISL_629910, EPI_ISL_629911, EPI_ISL_629912, EPI_ISL_629913, EPI_ISL_629915, EPI_ISL_629916, EPI_ISL_629917, EPI_ISL_629918, EPI_ISL_629919, EPI_ISL_629920, EPI_ISL_629921, EPI_ISL_629922, EPI_ISL_629923, EPI_ISL_629924, EPI_ISL_629925, EPI_ISL_629926, EPI_ISL_629927, EPI_ISL_629928, EPI_ISL_629929, EPI_ISL_629930, EPI_ISL_629931, EPI_ISL_629932, EPI_ISL_629933, EPI_ISL_629934, EPI_ISL_629935, EPI_ISL_629936, EPI_ISL_629937, EPI_ISL_629938, EPI_ISL_629939			
see above	Lighthouse Lab in Glasgow	Wellcome Sanger Institute for the COVID-19 Genomics UK (COG-UK) consortium	Harper VanSteenhouse, Yumi Kasai, David Gray, Carol Clugston, Anna Dominiczak and Alex Alderton, Roberto Amato, Sonia Goncalves, Ewan Harrison, David K. Jackson, Ian Johnston, Dominic Kwiatkowski, Cordelia Langford, John Sillitoe on behalf of the Wellcome Sanger Institute COVID-19 Surveillance Team
EPI_ISL_629940	Lighthouse Lab in Glasgow	Wellcome Sanger Institute for the COVID-19 Genomics UK (COG-UK) Consortium	Harper VanSteenhouse, Yumi Kasai, David Gray, Carol Clugston, Anna Dominiczak and Alex Alderton, Roberto Amato, Sonia Goncalves, Ewan Harrison, David K. Jackson, Ian Johnston, Dominic Kwiatkowski, Cordelia Langford, John Sillitoe on behalf of the Wellcome Sanger Institute COVID-19 Surveillance Team
EPI_ISL_629941, EPI_ISL_629942, EPI_ISL_629943, EPI_ISL_629944, EPI_ISL_629945, EPI_ISL_629946, EPI_ISL_629947, EPI_ISL_629948, EPI_ISL_629949, EPI_ISL_629950, EPI_ISL_629951, EPI_ISL_629952, EPI_ISL_629953, EPI_ISL_629954, EPI_ISL_629955, EPI_ISL_629956, EPI_ISL_629957, EPI_ISL_629958, EPI_ISL_629959, EPI_ISL_629960, EPI_ISL_629961, EPI_ISL_629962, EPI_ISL_629963, EPI_ISL_629964, EPI_ISL_629965, EPI_ISL_629966, EPI_ISL_629967, EPI_ISL_629968, EPI_ISL_629969, EPI_ISL_629971, EPI_ISL_629972, EPI_ISL_629973, EPI_ISL_629974, EPI_ISL_629975, EPI_ISL_629976, EPI_ISL_629977, EPI_ISL_629979, EPI_ISL_629980, EPI_ISL_629981, EPI_ISL_629982, EPI_ISL_629983, EPI_ISL_629984, EPI_ISL_629985, EPI_ISL_629986, EPI_ISL_629987, EPI_ISL_629988, EPI_ISL_629989, EPI_ISL_629990, EPI_ISL_629991, EPI_ISL_629992, EPI_ISL_629993, EPI_ISL_629995, EPI_ISL_629996, EPI_ISL_629997, EPI_ISL_629998, EPI_ISL_629999, EPI_ISL_630001, EPI_ISL_630002, EPI_ISL_630003, EPI_ISL_630004, EPI_ISL_630005, EPI_ISL_630006			
see above	Lighthouse Lab in Glasgow	Wellcome Sanger Institute for the COVID-19 Genomics UK (COG-UK) consortium	Harper VanSteenhouse, Yumi Kasai, David Gray, Carol Clugston, Anna Dominiczak and Alex Alderton, Roberto Amato, Sonia Goncalves, Ewan Harrison, David K. Jackson, Ian Johnston, Dominic Kwiatkowski, Cordelia Langford, John Sillitoe on behalf of the Wellcome Sanger Institute COVID-19 Surveillance Team
EPI_ISL_630007, EPI_ISL_630008, EPI_ISL_630009, EPI_ISL_630010, EPI_ISL_630011, EPI_ISL_630012, EPI_ISL_630013, EPI_ISL_630014, EPI_ISL_630015, EPI_ISL_630016, EPI_ISL_630017, EPI_ISL_630018, EPI_ISL_630019, EPI_ISL_630020, EPI_ISL_630021, EPI_ISL_630022, EPI_ISL_630023, EPI_ISL_630024, EPI_ISL_630025, EPI_ISL_630027, EPI_ISL_630028, EPI_ISL_630029, EPI_ISL_630030, EPI_ISL_630031, EPI_ISL_630032, EPI_ISL_630033, EPI_ISL_630034, EPI_ISL_630035, EPI_ISL_630036, EPI_ISL_630037, EPI_ISL_630038, EPI_ISL_630039, EPI_ISL_630040, EPI_ISL_630041, EPI_ISL_630042, EPI_ISL_630043, EPI_ISL_630044, EPI_ISL_630045, EPI_ISL_630046, EPI_ISL_630047, EPI_ISL_630048, EPI_ISL_630049, EPI_ISL_630050, EPI_ISL_630052, EPI_ISL_630053, EPI_ISL_630054, EPI_ISL_630055, EPI_ISL_630056, EPI_ISL_630057, EPI_ISL_630058, EPI_ISL_630059, EPI_ISL_630060, EPI_ISL_630061, EPI_ISL_630062, EPI_ISL_630063, EPI_ISL_630064, EPI_ISL_630065, EPI_ISL_630066, EPI_ISL_630067, EPI_ISL_630068, EPI_ISL_630069, EPI_ISL_630070, EPI_ISL_630071, EPI_ISL_630072, EPI_ISL_630073, EPI_ISL_630074, EPI_ISL_630075, EPI_ISL_630076, EPI_ISL_630077, EPI_ISL_630078, EPI_ISL_630079, EPI_ISL_630080, EPI_ISL_630081, EPI_ISL_630082, EPI_ISL_630083, EPI_ISL_630084, EPI_ISL_630085, EPI_ISL_630086, EPI_ISL_630087, EPI_ISL_630088, EPI_ISL_630089, EPI_ISL_630090, EPI_ISL_630091, EPI_ISL_630092, EPI_ISL_630093, EPI_ISL_630094, EPI_ISL_630095, EPI_ISL_630096, EPI_ISL_630097, EPI_ISL_630098, EPI_ISL_630099, EPI_ISL_630100, EPI_ISL_630101, EPI_ISL_630102, EPI_ISL_630103, EPI_ISL_630104, EPI_ISL_630105, EPI_ISL_630106, EPI_ISL_630107, EPI_ISL_630108, EPI_ISL_630109, EPI_ISL_630110, EPI_ISL_630111, EPI_ISL_630112, EPI_ISL_630113, EPI_ISL_630114, EPI_ISL_630115, EPI_ISL_630116, EPI_ISL_630117, EPI_ISL_630118, EPI_ISL_630119, EPI_ISL_630120, EPI_ISL_630121, EPI_ISL_630122, EPI_ISL_630123, EPI_ISL_630124, EPI_ISL_630125, EPI_ISL_630126, EPI_ISL_630127, EPI_ISL_630128, EPI_ISL_630129, EPI_ISL_630130, EPI_ISL_630131, EPI_ISL_630132, EPI_ISL_630133, EPI_ISL_630134, EPI_ISL_630135, EPI_ISL_630136, EPI_ISL_630137, EPI_ISL_630138, EPI_ISL_630139, EPI_ISL_630140, EPI_ISL_630141, EPI_ISL_630142, EPI_ISL_630143, EPI_ISL_630144, EPI_ISL_630145, EPI_ISL_630146, EPI_ISL_630147, EPI_ISL_630148, EPI_ISL_630149, EPI_ISL_630150, EPI_ISL_630151, EPI_ISL_630152, EPI_ISL_630153, EPI_ISL_630154, EPI_ISL_630155, EPI_ISL_630156, EPI_ISL_630157, EPI_ISL_630158, EPI_ISL_630159, EPI_ISL_630160, EPI_ISL_630161, EPI_ISL_630162, EPI_ISL_630163, EPI_ISL_630164, EPI_ISL_630165, EPI_ISL_630166, EPI_ISL_630167, EPI_ISL_630168, EPI_ISL_630169, EPI_ISL_630170, EPI_ISL_630171, EPI_ISL_630172, EPI_ISL_630173, EPI_ISL_630174, EPI_ISL_630175, EPI_ISL_630176, EPI_ISL_630177, EPI_ISL_630178, EPI_ISL_630180, EPI_ISL_630181, EPI_ISL_630182, EPI_ISL_630183, EPI_ISL_630184, EPI_ISL_630185, EPI_ISL_630186, EPI_ISL_630187, EPI_ISL_630188, EPI_ISL_630189, EPI_ISL_630190			
see above	Lighthouse Lab in Milton Keynes	Wellcome Sanger Institute for the COVID-19 Genomics UK (COG-UK) consortium	The Lighthouse Lab in Milton Keynes and Alex Alderton, Roberto Amato, Sonia Goncalves, Ewan Harrison, David K. Jackson, Ian Johnston, Dominic Kwiatkowski, Cordelia Langford, John Sillitoe on behalf of the Wellcome Sanger Institute COVID-19 Surveillance Team
EPI_ISL_630191	Lighthouse Lab in Milton Keynes	Wellcome Sanger Institute for the COVID-19 Genomics UK (COG-UK) Consortium	The Lighthouse Lab in Milton Keynes and Alex Alderton, Roberto Amato, Sonia Goncalves, Ewan Harrison, David K. Jackson, Ian Johnston, Dominic Kwiatkowski, Cordelia Langford, John Sillitoe on behalf of the Wellcome Sanger Institute COVID-19 Surveillance Team
EPI_ISL_630192, EPI_ISL_630193, EPI_ISL_630194, EPI_ISL_630195, EPI_ISL_630196, EPI_ISL_630197, EPI_ISL_630198, EPI_ISL_630199, EPI_ISL_630200, EPI_ISL_630201, EPI_ISL_630202, EPI_ISL_630203, EPI_ISL_630204, EPI_ISL_630206, EPI_ISL_630207, EPI_ISL_630208, EPI_ISL_630209, EPI_ISL_630210, EPI_ISL_630211, EPI_ISL_630212, EPI_ISL_630213, EPI_ISL_630214, EPI_ISL_630215, EPI_ISL_630216, EPI_ISL_630217, EPI_ISL_630218			
see above	Lighthouse Lab in Milton Keynes	Wellcome Sanger Institute for the COVID-19 Genomics UK (COG-UK) consortium	The Lighthouse Lab in Milton Keynes and Alex Alderton, Roberto Amato, Sonia Goncalves, Ewan Harrison, David K. Jackson, Ian Johnston, Dominic Kwiatkowski, Cordelia Langford, John Sillitoe on behalf of the Wellcome Sanger Institute COVID-19 Surveillance Team
EPI_ISL_630219	Lighthouse Lab in Milton Keynes	Wellcome Sanger Institute for the COVID-19 Genomics UK (COG-UK) Consortium	The Lighthouse Lab in Milton Keynes and Alex Alderton, Roberto Amato, Sonia Goncalves, Ewan Harrison, David K. Jackson, Ian Johnston, Dominic Kwiatkowski, Cordelia Langford, John Sillitoe on behalf of the Wellcome Sanger Institute COVID-19 Surveillance Team
EPI_ISL_630220, EPI_ISL_630221, EPI_ISL_630222, EPI_ISL_630223, EPI_ISL_630225, EPI_ISL_630226, EPI_ISL_630227, EPI_ISL_630228, EPI_ISL_630229, EPI_ISL_630230, EPI_ISL_630231, EPI_ISL_630232, EPI_ISL_630233, EPI_ISL_630234, EPI_ISL_630235, EPI_ISL_630236, EPI_ISL_630237, EPI_ISL_630238, EPI_ISL_630239, EPI_ISL_630240, EPI_ISL_630241, EPI_ISL_630242, EPI_ISL_630243, EPI_ISL_630244, EPI_ISL_630245, EPI_ISL_630246, EPI_ISL_630247, EPI_ISL_630248			
see above	Lighthouse Lab in Milton Keynes	Wellcome Sanger Institute for the COVID-19 Genomics UK (COG-UK) consortium	The Lighthouse Lab in Milton Keynes and Alex Alderton, Roberto Amato, Sonia Goncalves, Ewan Harrison, David K. Jackson, Ian Johnston, Dominic Kwiatkowski, Cordelia Langford, John Sillitoe on behalf of the Wellcome Sanger Institute COVID-19 Surveillance Team
EPI_ISL_630249	Lighthouse Lab in Milton Keynes	Wellcome Sanger Institute for the COVID-19 Genomics UK (COG-UK) Consortium	The Lighthouse Lab in Milton Keynes and Alex Alderton, Roberto Amato, Sonia Goncalves, Ewan Harrison, David K. Jackson, Ian Johnston, Dominic Kwiatkowski, Cordelia Langford, John Sillitoe on behalf of the Wellcome Sanger Institute COVID-19 Surveillance Team
EPI_ISL_630250, EPI_ISL_630251, EPI_ISL_630252, EPI_ISL_630253, EPI_ISL_630254, EPI_ISL_630255, EPI_ISL_630256, EPI_ISL_630257, EPI_ISL_630258, EPI_ISL_630259, EPI_ISL_630260, EPI_ISL_630261, EPI_ISL_630262, EPI_ISL_630263, EPI_ISL_630264, EPI_ISL_630265, EPI_ISL_630266, EPI_ISL_630267, EPI_ISL_630268, EPI_ISL_630269, EPI_ISL_630270, EPI_ISL_630271, EPI_ISL_630272, EPI_ISL_630273, EPI_ISL_630274, EPI_ISL_630275, EPI_ISL_630276, EPI_ISL_630277, EPI_ISL_630278, EPI_ISL_630279, EPI_ISL_630280, EPI_ISL_630281, EPI_ISL_630282, EPI_ISL_630283, EPI_ISL_630284, EPI_ISL_630285, EPI_ISL_630286, EPI_ISL_630287, EPI_ISL_630288, EPI_ISL_630289, EPI_ISL_630290, EPI_ISL_630291, EPI_ISL_630292, EPI_ISL_630293, EPI_ISL_630294, EPI_ISL_630295, EPI_ISL_630296, EPI_ISL_630297, EPI_ISL_630298, EPI_ISL_630299, EPI_ISL_630300, EPI_ISL_630301, EPI_ISL_630302, EPI_ISL_630303, EPI_ISL_630304, EPI_ISL_630305, EPI_ISL_630306, EPI_ISL_630307, EPI_ISL_630308, EPI_ISL_630309, EPI_ISL_630310, EPI_ISL_630311, EPI_ISL_630312, EPI_ISL_630313, EPI_ISL_630314, EPI_ISL_630315, EPI_ISL_630316, EPI_ISL_630317, EPI_ISL_630318, EPI_ISL_630319, EPI_ISL_630320, EPI_ISL_630321, EPI_ISL_630322, EPI_ISL_630323, EPI_ISL_630324, EPI_ISL_630325, EPI_ISL_630326, EPI_ISL_630327, EPI_ISL_630328, EPI_ISL_630329, EPI_ISL_630330, EPI_ISL_630331, EPI_ISL_630332, EPI_ISL_630333, EPI_ISL_630334, EPI_ISL_630335, EPI_ISL_630336, EPI_ISL_630337, EPI_ISL_630338, EPI_ISL_630339, EPI_ISL_630340, EPI_ISL_630341, EPI_ISL_630342, EPI_ISL_630343, EPI_ISL_630344, EPI_ISL_630345, EPI_ISL_630346, EPI_ISL_630347			
see above	Lighthouse Lab in Milton Keynes	Wellcome Sanger Institute for the COVID-19 Genomics UK (COG-UK) consortium	The Lighthouse Lab in Milton Keynes and Alex Alderton, Roberto Amato, Sonia Goncalves, Ewan Harrison, David K. Jackson, Ian Johnston, Dominic Kwiatkowski, Cordelia Langford, John Sillitoe on behalf of the Wellcome Sanger Institute COVID-19 Surveillance Team
EPI_ISL_630348, EPI_ISL_630349, EPI_ISL_630350, EPI_ISL_630351, EPI_ISL_630352, EPI_ISL_630353, EPI_ISL_630354, EPI_ISL_630355, EPI_ISL_630356, EPI_ISL_630357, EPI_ISL_630358, EPI_ISL_630359, EPI_ISL_630360, EPI_ISL_630361, EPI_ISL_630362, EPI_ISL_630363, EPI_ISL_630364, EPI_ISL_630365, EPI_ISL_630366, EPI_ISL_630367, EPI_ISL_630368, EPI_ISL_630369, EPI_ISL_630370, EPI_ISL_630371, EPI_ISL_630372, EPI_ISL_630373, EPI_ISL_630374, EPI_ISL_630375, EPI_ISL_630376, EPI_ISL_630377, EPI_ISL_630378, EPI_ISL_630379, EPI_ISL_630380, EPI_ISL_630381, EPI_ISL_630382, EPI_ISL_630383, EPI_ISL_630384, EPI_ISL_630385, EPI_ISL_630386, EPI_ISL_630387, EPI_ISL_630388, EPI_ISL_630389, EPI_ISL_630390, EPI_ISL_630391, EPI_ISL_630392, EPI_ISL_630393, EPI_ISL_630394, EPI_ISL_630395, EPI_ISL_630396, EPI_ISL_630397, EPI_ISL_630398, EPI_ISL_630399, EPI_ISL_630400, EPI_ISL_630401, EPI_ISL_630402, EPI_ISL_630403, EPI_ISL_630404, EPI_ISL_630405, EPI_ISL_630406, EPI_ISL_630407, EPI_ISL_630408, EPI_ISL_630409, EPI_ISL_630410, EPI_ISL_630411, EPI_ISL_630412, EPI_ISL_630413, EPI_ISL_630414, EPI_ISL_630415, EPI_ISL_630416, EPI_ISL_630417, EPI_ISL_630418, EPI_ISL_630419, EPI_ISL_630420, EPI_ISL_630421, EPI_ISL_630422, EPI_ISL_630423, EPI_ISL_630424, EPI_ISL_630425, EPI_ISL_630426, EPI_ISL_630427, EPI_ISL_630428, EPI_ISL_630429, EPI_ISL_630430, EPI_ISL_630431, EPI_ISL_630432, EPI_ISL_630433, EPI_ISL_630434, EPI_ISL_630435, EPI_ISL_630436, EPI_ISL_630437, EPI_ISL_630438, EPI_ISL_630439, EPI_ISL_630440, EPI_ISL_630441, EPI_ISL_630442, EPI_ISL_630443, EPI_ISL_630444, EPI_ISL_630445, EPI_ISL_630446, EPI_ISL_630447, EPI_ISL_630448, EPI_ISL_630449, EPI_ISL_630450, EPI_ISL_630451, EPI_ISL_630452, EPI_ISL_630453, EPI_ISL_630454, EPI_ISL_630455, EPI_ISL_630456, EPI_ISL_630457, EPI_ISL_630458, EPI_ISL_630459, EPI_ISL_630460, EPI_ISL_630461, EPI_ISL_630462, EPI_ISL_630463, EPI_ISL_630464, EPI_ISL_630465, EPI_ISL_630466, EPI_ISL_630467, EPI_ISL_630468, EPI_ISL_630469, EPI_ISL_630470, EPI_ISL_630471, EPI_ISL_630472, EPI_ISL_630473			

see above	Lighthouse Lab in Alderley Park	Wellcome Sanger Institute for the COVID-19 Genomics UK (COG-UK) consortium	Jacquelyn Wynn, Mairead Hyland, The Lighthouse Lab in Alderley Park and Alex Alderton, Roberto Amato, Sonia Goncalves, Ewan Harrison, David K. Jackson, Ian Johnston, Dominic Kwiatkowski, Cordelia Langford, John Sillitoe on behalf of the Wellcome Sanger Institute COVID-19 Surveillance Team
EPI_ISL_630474	Lighthouse Lab in Alderley Park	Wellcome Sanger Institute for the COVID-19 Genomics UK (COG-UK) Consortium	Jacquelyn Wynn, Mairead Hyland, The Lighthouse Lab in Alderley Park and Alex Alderton, Roberto Amato, Sonia Goncalves, Ewan Harrison, David K. Jackson, Ian Johnston, Dominic Kwiatkowski, Cordelia Langford, John Sillitoe on behalf of the Wellcome Sanger Institute COVID-19 Surveillance Team
EPI_ISL_630475, EPI_ISL_630476, EPI_ISL_630477, EPI_ISL_630478, EPI_ISL_630479, EPI_ISL_630480, EPI_ISL_630481, EPI_ISL_630482, EPI_ISL_630483, EPI_ISL_630484, EPI_ISL_630485, EPI_ISL_630486, EPI_ISL_630487, EPI_ISL_630488, EPI_ISL_630489, EPI_ISL_630490, EPI_ISL_630491, EPI_ISL_630492, EPI_ISL_630493, EPI_ISL_630494, EPI_ISL_630495, EPI_ISL_630496, EPI_ISL_630497, EPI_ISL_630498, EPI_ISL_630499, EPI_ISL_630500, EPI_ISL_630501, EPI_ISL_630502, EPI_ISL_630503, EPI_ISL_630504, EPI_ISL_630505, EPI_ISL_630506, EPI_ISL_630507, EPI_ISL_630508, EPI_ISL_630509, EPI_ISL_630510, EPI_ISL_630511, EPI_ISL_630512, EPI_ISL_630513, EPI_ISL_630514, EPI_ISL_630515, EPI_ISL_630516, EPI_ISL_630517, EPI_ISL_630518, EPI_ISL_630519, EPI_ISL_630520, EPI_ISL_630521, EPI_ISL_630522, EPI_ISL_630523, EPI_ISL_630524, EPI_ISL_630525, EPI_ISL_630526, EPI_ISL_630528, EPI_ISL_630529, EPI_ISL_630530, EPI_ISL_630531, EPI_ISL_630532			
see above	Lighthouse Lab in Alderley Park	Wellcome Sanger Institute for the COVID-19 Genomics UK (COG-UK) consortium	Jacquelyn Wynn, Mairead Hyland, The Lighthouse Lab in Alderley Park and Alex Alderton, Roberto Amato, Sonia Goncalves, Ewan Harrison, David K. Jackson, Ian Johnston, Dominic Kwiatkowski, Cordelia Langford, John Sillitoe on behalf of the Wellcome Sanger Institute COVID-19 Surveillance Team
EPI_ISL_630533	Lighthouse Lab in Alderley Park	Wellcome Sanger Institute for the COVID-19 Genomics UK (COG-UK) Consortium	Jacquelyn Wynn, Mairead Hyland, The Lighthouse Lab in Alderley Park and Alex Alderton, Roberto Amato, Sonia Goncalves, Ewan Harrison, David K. Jackson, Ian Johnston, Dominic Kwiatkowski, Cordelia Langford, John Sillitoe on behalf of the Wellcome Sanger Institute COVID-19 Surveillance Team
EPI_ISL_630534, EPI_ISL_630535, EPI_ISL_630536, EPI_ISL_630537, EPI_ISL_630538, EPI_ISL_630539, EPI_ISL_630540, EPI_ISL_630541, EPI_ISL_630542, EPI_ISL_630543, EPI_ISL_630544, EPI_ISL_630545, EPI_ISL_630546			
see above	Lighthouse Lab in Alderley Park	Wellcome Sanger Institute for the COVID-19 Genomics UK (COG-UK) consortium	Jacquelyn Wynn, Mairead Hyland, The Lighthouse Lab in Alderley Park and Alex Alderton, Roberto Amato, Sonia Goncalves, Ewan Harrison, David K. Jackson, Ian Johnston, Dominic Kwiatkowski, Cordelia Langford, John Sillitoe on behalf of the Wellcome Sanger Institute COVID-19 Surveillance Team
EPI_ISL_630547	Lighthouse Lab in Alderley Park	Wellcome Sanger Institute for the COVID-19 Genomics UK (COG-UK) Consortium	Jacquelyn Wynn, Mairead Hyland, The Lighthouse Lab in Alderley Park and Alex Alderton, Roberto Amato, Sonia Goncalves, Ewan Harrison, David K. Jackson, Ian Johnston, Dominic Kwiatkowski, Cordelia Langford, John Sillitoe on behalf of the Wellcome Sanger Institute COVID-19 Surveillance Team
EPI_ISL_630548, EPI_ISL_630549, EPI_ISL_630550, EPI_ISL_630551, EPI_ISL_630552, EPI_ISL_630553, EPI_ISL_630554, EPI_ISL_630555, EPI_ISL_630556, EPI_ISL_630557, EPI_ISL_630558, EPI_ISL_630559, EPI_ISL_630560, EPI_ISL_630561, EPI_ISL_630562, EPI_ISL_630563, EPI_ISL_630564, EPI_ISL_630565, EPI_ISL_630566, EPI_ISL_630567, EPI_ISL_630568, EPI_ISL_630569, EPI_ISL_630570, EPI_ISL_630571, EPI_ISL_630572, EPI_ISL_630573, EPI_ISL_630574, EPI_ISL_630575, EPI_ISL_630577, EPI_ISL_630578, EPI_ISL_630579, EPI_ISL_630581, EPI_ISL_630582, EPI_ISL_630583, EPI_ISL_630584, EPI_ISL_630585, EPI_ISL_630586, EPI_ISL_630587, EPI_ISL_630588, EPI_ISL_630589, EPI_ISL_630590, EPI_ISL_630591, EPI_ISL_630592, EPI_ISL_630593, EPI_ISL_630594, EPI_ISL_630595, EPI_ISL_630596, EPI_ISL_630597, EPI_ISL_630598, EPI_ISL_630599, EPI_ISL_630600, EPI_ISL_630601, EPI_ISL_630602, EPI_ISL_630603, EPI_ISL_630604, EPI_ISL_630605, EPI_ISL_630606, EPI_ISL_630607, EPI_ISL_630608, EPI_ISL_630609, EPI_ISL_630610, EPI_ISL_630611, EPI_ISL_630612, EPI_ISL_630613, EPI_ISL_630614, EPI_ISL_630615, EPI_ISL_630616, EPI_ISL_630617, EPI_ISL_630618, EPI_ISL_630619, EPI_ISL_630620, EPI_ISL_630622, EPI_ISL_630623, EPI_ISL_630624, EPI_ISL_630625, EPI_ISL_630626, EPI_ISL_630627, EPI_ISL_630628, EPI_ISL_630629, EPI_ISL_630630, EPI_ISL_630631, EPI_ISL_630632, EPI_ISL_630633, EPI_ISL_630634, EPI_ISL_630635, EPI_ISL_630636, EPI_ISL_630637, EPI_ISL_630638, EPI_ISL_630639, EPI_ISL_630640, EPI_ISL_630641, EPI_ISL_630642, EPI_ISL_630643, EPI_ISL_630644, EPI_ISL_630645, EPI_ISL_630646, EPI_ISL_630647, EPI_ISL_630648, EPI_ISL_630649, EPI_ISL_630650, EPI_ISL_630651, EPI_ISL_630652, EPI_ISL_630653, EPI_ISL_630654, EPI_ISL_630655, EPI_ISL_630656, EPI_ISL_630657, EPI_ISL_630658, EPI_ISL_630659, EPI_ISL_630660, EPI_ISL_630661, EPI_ISL_630662, EPI_ISL_630663, EPI_ISL_630664, EPI_ISL_630665, EPI_ISL_630666, EPI_ISL_630667, EPI_ISL_630668, EPI_ISL_630669, EPI_ISL_630670, EPI_ISL_630671, EPI_ISL_630672, EPI_ISL_630673, EPI_ISL_630674, EPI_ISL_630675, EPI_ISL_630676, EPI_ISL_630677, EPI_ISL_630678, EPI_ISL_630679, EPI_ISL_630680, EPI_ISL_630681, EPI_ISL_630682, EPI_ISL_630683, EPI_ISL_630684, EPI_ISL_630685, EPI_ISL_630686, EPI_ISL_630687, EPI_ISL_630688, EPI_ISL_630689, EPI_ISL_630690, EPI_ISL_630691, EPI_ISL_630692, EPI_ISL_630693, EPI_ISL_630694, EPI_ISL_630695, EPI_ISL_630696, EPI_ISL_630697, EPI_ISL_630698, EPI_ISL_630699, EPI_ISL_630700, EPI_ISL_630701			
see above	Lighthouse Lab in Alderley Park	Wellcome Sanger Institute for the COVID-19 Genomics UK (COG-UK) consortium	Jacquelyn Wynn, Mairead Hyland, The Lighthouse Lab in Alderley Park and Alex Alderton, Roberto Amato, Sonia Goncalves, Ewan Harrison, David K. Jackson, Ian Johnston, Dominic Kwiatkowski, Cordelia Langford, John Sillitoe on behalf of the Wellcome Sanger Institute COVID-19 Surveillance Team
EPI_ISL_630702, EPI_ISL_630703, EPI_ISL_630704, EPI_ISL_630705	Lighthouse Lab in Glasgow	Wellcome Sanger Institute for the COVID-19 Genomics UK (COG-UK) consortium	Harper VanSteenhouse, Yumi Kasai, David Gray, Carol Clugston, Anna Dominiczak and Alex Alderton, Roberto Amato, Sonia Goncalves, Ewan Harrison, David K. Jackson, Ian Johnston, Dominic Kwiatkowski, Cordelia Langford, John Sillitoe on behalf of the Wellcome Sanger Institute COVID-19 Surveillance Team
EPI_ISL_630707, EPI_ISL_630708, EPI_ISL_630709, EPI_ISL_630711, EPI_ISL_630713, EPI_ISL_630714, EPI_ISL_630715, EPI_ISL_630716, EPI_ISL_630717	Lighthouse Lab in Alderley Park	Wellcome Sanger Institute for the COVID-19 Genomics UK (COG-UK) consortium	Jacquelyn Wynn, Mairead Hyland, The Lighthouse Lab in Alderley Park and Alex Alderton, Roberto Amato, Sonia Goncalves, Ewan Harrison, David K. Jackson, Ian Johnston, Dominic Kwiatkowski, Cordelia Langford, John Sillitoe on behalf of the Wellcome Sanger Institute COVID-19 Surveillance Team
EPI_ISL_630718, EPI_ISL_630719	Lighthouse Lab in Glasgow	Wellcome Sanger Institute for the COVID-19 Genomics UK (COG-UK) consortium	Harper VanSteenhouse, Yumi Kasai, David Gray, Carol Clugston, Anna Dominiczak and Alex Alderton, Roberto Amato, Sonia Goncalves, Ewan Harrison, David K. Jackson, Ian Johnston, Dominic Kwiatkowski, Cordelia Langford, John Sillitoe on behalf of the Wellcome Sanger Institute COVID-19 Surveillance Team
EPI_ISL_630720, EPI_ISL_630721, EPI_ISL_630722, EPI_ISL_630723, EPI_ISL_630724	Lighthouse Lab in Alderley Park	Wellcome Sanger Institute for the COVID-19 Genomics UK (COG-UK) consortium	Jacquelyn Wynn, Mairead Hyland, The Lighthouse Lab in Alderley Park and Alex Alderton, Roberto Amato, Sonia Goncalves, Ewan Harrison, David K. Jackson, Ian Johnston, Dominic Kwiatkowski, Cordelia Langford, John Sillitoe on behalf of the Wellcome Sanger Institute COVID-19 Surveillance Team
EPI_ISL_630725	Lighthouse Lab in Glasgow	Wellcome Sanger Institute for the COVID-19 Genomics UK (COG-UK) consortium	Harper VanSteenhouse, Yumi Kasai, David Gray, Carol Clugston, Anna Dominiczak and Alex Alderton, Roberto Amato, Sonia Goncalves, Ewan Harrison, David K. Jackson, Ian Johnston, Dominic Kwiatkowski, Cordelia Langford, John Sillitoe on behalf of the Wellcome Sanger Institute COVID-19 Surveillance Team
EPI_ISL_630726, EPI_ISL_630727, EPI_ISL_630728, EPI_ISL_630729, EPI_ISL_630730, EPI_ISL_630732	Lighthouse Lab in Alderley Park	Wellcome Sanger Institute for the COVID-19 Genomics UK (COG-UK) consortium	Jacquelyn Wynn, Mairead Hyland, The Lighthouse Lab in Alderley Park and Alex Alderton, Roberto Amato, Sonia Goncalves, Ewan Harrison, David K. Jackson, Ian Johnston, Dominic Kwiatkowski, Cordelia Langford, John Sillitoe on behalf of the Wellcome Sanger Institute COVID-19 Surveillance Team
EPI_ISL_630733	Lighthouse Lab in Glasgow	Wellcome Sanger Institute for the COVID-19 Genomics UK (COG-UK) consortium	Harper VanSteenhouse, Yumi Kasai, David Gray, Carol Clugston, Anna Dominiczak and Alex Alderton, Roberto Amato, Sonia Goncalves, Ewan Harrison, David K. Jackson, Ian Johnston, Dominic Kwiatkowski, Cordelia Langford, John Sillitoe on behalf of the Wellcome Sanger Institute COVID-19 Surveillance Team
EPI_ISL_630734, EPI_ISL_630735, EPI_ISL_630736, EPI_ISL_630737, EPI_ISL_630738, EPI_ISL_630739, EPI_ISL_630740	Lighthouse Lab in Alderley Park	Wellcome Sanger Institute for the COVID-19 Genomics UK (COG-UK) consortium	Jacquelyn Wynn, Mairead Hyland, The Lighthouse Lab in Alderley Park and Alex Alderton, Roberto Amato, Sonia Goncalves, Ewan Harrison, David K. Jackson, Ian Johnston, Dominic Kwiatkowski, Cordelia Langford, John Sillitoe on behalf of the Wellcome Sanger Institute COVID-19 Surveillance Team
EPI_ISL_630741	Lighthouse Lab in Glasgow	Wellcome Sanger Institute for the COVID-19 Genomics UK (COG-UK) consortium	Harper VanSteenhouse, Yumi Kasai, David Gray, Carol Clugston, Anna Dominiczak and Alex Alderton, Roberto Amato, Sonia Goncalves, Ewan Harrison, David K. Jackson, Ian Johnston, Dominic Kwiatkowski, Cordelia Langford, John Sillitoe on behalf of the Wellcome Sanger Institute COVID-19 Surveillance Team
EPI_ISL_630742, EPI_ISL_630743, EPI_ISL_630744	Lighthouse Lab in Alderley Park	Wellcome Sanger Institute for the COVID-19 Genomics UK (COG-UK) consortium	Jacquelyn Wynn, Mairead Hyland, The Lighthouse Lab in Alderley Park and Alex Alderton, Roberto Amato, Sonia Goncalves, Ewan Harrison, David K. Jackson, Ian Johnston, Dominic Kwiatkowski, Cordelia Langford, John Sillitoe on behalf of the Wellcome Sanger Institute COVID-19 Surveillance Team
EPI_ISL_630745	Lighthouse Lab in Glasgow	Wellcome Sanger Institute for the COVID-19 Genomics UK (COG-UK) consortium	Harper VanSteenhouse, Yumi Kasai, David Gray, Carol Clugston, Anna Dominiczak and Alex Alderton, Roberto Amato, Sonia Goncalves, Ewan Harrison, David K. Jackson, Ian Johnston, Dominic Kwiatkowski, Cordelia Langford, John Sillitoe on behalf of the Wellcome Sanger Institute COVID-19 Surveillance Team
EPI_ISL_630746, EPI_ISL_630747, EPI_ISL_630748, EPI_ISL_630750, EPI_ISL_630751, EPI_ISL_630752, EPI_ISL_630753, EPI_ISL_630754, EPI_ISL_630755, EPI_ISL_630756, EPI_ISL_630757, EPI_ISL_630758, EPI_ISL_630759, EPI_ISL_630760			
see above	Lighthouse Lab in Alderley Park	Wellcome Sanger Institute for the COVID-19 Genomics UK (COG-UK) consortium	Jacquelyn Wynn, Mairead Hyland, The Lighthouse Lab in Alderley Park and Alex Alderton, Roberto Amato, Sonia Goncalves, Ewan Harrison, David K. Jackson, Ian Johnston, Dominic Kwiatkowski, Cordelia Langford, John Sillitoe on behalf of the Wellcome Sanger Institute COVID-19 Surveillance Team
EPI_ISL_630761	Lighthouse Lab in Glasgow	Wellcome Sanger Institute for the COVID-19 Genomics UK (COG-UK) consortium	Harper VanSteenhouse, Yumi Kasai, David Gray, Carol Clugston, Anna Dominiczak and Alex Alderton, Roberto Amato, Sonia Goncalves, Ewan Harrison, David K. Jackson, Ian Johnston, Dominic Kwiatkowski, Cordelia Langford, John Sillitoe on behalf of the Wellcome Sanger Institute COVID-19 Surveillance Team
EPI_ISL_630762, EPI_ISL_630763	Lighthouse Lab in Alderley Park	Wellcome Sanger Institute for the COVID-19 Genomics UK (COG-UK) consortium	Jacquelyn Wynn, Mairead Hyland, The Lighthouse Lab in Alderley Park and Alex Alderton, Roberto Amato, Sonia Goncalves, Ewan Harrison, David K. Jackson, Ian Johnston, Dominic Kwiatkowski, Cordelia Langford, John Sillitoe on behalf of the Wellcome Sanger Institute COVID-19 Surveillance Team
EPI_ISL_630764	Lighthouse Lab in Glasgow	Wellcome Sanger Institute for the COVID-19 Genomics UK (COG-UK) consortium	Harper VanSteenhouse, Yumi Kasai, David Gray, Carol Clugston, Anna Dominiczak and Alex Alderton, Roberto Amato, Sonia Goncalves, Ewan Harrison, David K. Jackson, Ian Johnston, Dominic Kwiatkowski, Cordelia Langford, John Sillitoe on behalf of the Wellcome Sanger Institute COVID-19 Surveillance Team

EPI_ISL_630765, EPI_ISL_630766, EPI_ISL_630767, EPI_ISL_630768, EPI_ISL_630769, EPI_ISL_630771, EPI_ISL_630772, EPI_ISL_630773, EPI_ISL_630774	Lighthouse Lab in Alderley Park	Wellcome Sanger Institute for the COVID-19 Genomics UK (COG-UK) consortium	Jacquelyn Wynn, Mairead Hyland, The Lighthouse Lab in Alderley Park and Alex Alderton, Roberto Amato, Sonia Goncalves, Ewan Harrison, David K. Jackson, Ian Johnston, Dominic Kwiatkowski, Cordelia Langford, John Sillitoe on behalf of the Wellcome Sanger Institute COVID-19 Surveillance Team
EPI_ISL_630775	Lighthouse Lab in Glasgow	Wellcome Sanger Institute for the COVID-19 Genomics UK (COG-UK) consortium	Harper VanSteenhouse, Yumi Kasai, David Gray, Carol Clugston, Anna Dominiczak and Alex Alderton, Roberto Amato, Sonia Goncalves, Ewan Harrison, David K. Jackson, Ian Johnston, Dominic Kwiatkowski, Cordelia Langford, John Sillitoe on behalf of the Wellcome Sanger Institute COVID-19 Surveillance Team
EPI_ISL_630776, EPI_ISL_630777	Lighthouse Lab in Alderley Park	Wellcome Sanger Institute for the COVID-19 Genomics UK (COG-UK) consortium	Jacquelyn Wynn, Mairead Hyland, The Lighthouse Lab in Alderley Park and Alex Alderton, Roberto Amato, Sonia Goncalves, Ewan Harrison, David K. Jackson, Ian Johnston, Dominic Kwiatkowski, Cordelia Langford, John Sillitoe on behalf of the Wellcome Sanger Institute COVID-19 Surveillance Team
EPI_ISL_630778	Lighthouse Lab in Glasgow	Wellcome Sanger Institute for the COVID-19 Genomics UK (COG-UK) consortium	Harper VanSteenhouse, Yumi Kasai, David Gray, Carol Clugston, Anna Dominiczak and Alex Alderton, Roberto Amato, Sonia Goncalves, Ewan Harrison, David K. Jackson, Ian Johnston, Dominic Kwiatkowski, Cordelia Langford, John Sillitoe on behalf of the Wellcome Sanger Institute COVID-19 Surveillance Team
EPI_ISL_630779	Lighthouse Lab in Alderley Park	Wellcome Sanger Institute for the COVID-19 Genomics UK (COG-UK) consortium	Jacquelyn Wynn, Mairead Hyland, The Lighthouse Lab in Alderley Park and Alex Alderton, Roberto Amato, Sonia Goncalves, Ewan Harrison, David K. Jackson, Ian Johnston, Dominic Kwiatkowski, Cordelia Langford, John Sillitoe on behalf of the Wellcome Sanger Institute COVID-19 Surveillance Team
EPI_ISL_630780	Lighthouse Lab in Alderley Park	Wellcome Sanger Institute for the COVID-19 Genomics UK (COG-UK) Consortium	Jacquelyn Wynn, Mairead Hyland, The Lighthouse Lab in Alderley Park and Alex Alderton, Roberto Amato, Sonia Goncalves, Ewan Harrison, David K. Jackson, Ian Johnston, Dominic Kwiatkowski, Cordelia Langford, John Sillitoe on behalf of the Wellcome Sanger Institute COVID-19 Surveillance Team
EPI_ISL_630781	Lighthouse Lab in Alderley Park	Wellcome Sanger Institute for the COVID-19 Genomics UK (COG-UK) consortium	Jacquelyn Wynn, Mairead Hyland, The Lighthouse Lab in Alderley Park and Alex Alderton, Roberto Amato, Sonia Goncalves, Ewan Harrison, David K. Jackson, Ian Johnston, Dominic Kwiatkowski, Cordelia Langford, John Sillitoe on behalf of the Wellcome Sanger Institute COVID-19 Surveillance Team
EPI_ISL_630782	Lighthouse Lab in Glasgow	Wellcome Sanger Institute for the COVID-19 Genomics UK (COG-UK) consortium	Harper VanSteenhouse, Yumi Kasai, David Gray, Carol Clugston, Anna Dominiczak and Alex Alderton, Roberto Amato, Sonia Goncalves, Ewan Harrison, David K. Jackson, Ian Johnston, Dominic Kwiatkowski, Cordelia Langford, John Sillitoe on behalf of the Wellcome Sanger Institute COVID-19 Surveillance Team
EPI_ISL_630783, EPI_ISL_630784, EPI_ISL_630785	Lighthouse Lab in Alderley Park	Wellcome Sanger Institute for the COVID-19 Genomics UK (COG-UK) consortium	Jacquelyn Wynn, Mairead Hyland, The Lighthouse Lab in Alderley Park and Alex Alderton, Roberto Amato, Sonia Goncalves, Ewan Harrison, David K. Jackson, Ian Johnston, Dominic Kwiatkowski, Cordelia Langford, John Sillitoe on behalf of the Wellcome Sanger Institute COVID-19 Surveillance Team
EPI_ISL_630787	Lighthouse Lab in Glasgow	Wellcome Sanger Institute for the COVID-19 Genomics UK (COG-UK) consortium	Harper VanSteenhouse, Yumi Kasai, David Gray, Carol Clugston, Anna Dominiczak and Alex Alderton, Roberto Amato, Sonia Goncalves, Ewan Harrison, David K. Jackson, Ian Johnston, Dominic Kwiatkowski, Cordelia Langford, John Sillitoe on behalf of the Wellcome Sanger Institute COVID-19 Surveillance Team
EPI_ISL_630788, EPI_ISL_630789, EPI_ISL_630790, EPI_ISL_630791, EPI_ISL_630792, EPI_ISL_630793, EPI_ISL_630795, EPI_ISL_630796, EPI_ISL_630797, EPI_ISL_630798, EPI_ISL_630799, EPI_ISL_630800, EPI_ISL_630801, EPI_ISL_630802, EPI_ISL_630803			
see above	Lighthouse Lab in Alderley Park	Wellcome Sanger Institute for the COVID-19 Genomics UK (COG-UK) consortium	Jacquelyn Wynn, Mairead Hyland, The Lighthouse Lab in Alderley Park and Alex Alderton, Roberto Amato, Sonia Goncalves, Ewan Harrison, David K. Jackson, Ian Johnston, Dominic Kwiatkowski, Cordelia Langford, John Sillitoe on behalf of the Wellcome Sanger Institute COVID-19 Surveillance Team
EPI_ISL_630804	Lighthouse Lab in Glasgow	Wellcome Sanger Institute for the COVID-19 Genomics UK (COG-UK) consortium	Harper VanSteenhouse, Yumi Kasai, David Gray, Carol Clugston, Anna Dominiczak and Alex Alderton, Roberto Amato, Sonia Goncalves, Ewan Harrison, David K. Jackson, Ian Johnston, Dominic Kwiatkowski, Cordelia Langford, John Sillitoe on behalf of the Wellcome Sanger Institute COVID-19 Surveillance Team
EPI_ISL_630805, EPI_ISL_630806, EPI_ISL_630807, EPI_ISL_630808, EPI_ISL_630809, EPI_ISL_630810, EPI_ISL_630811, EPI_ISL_630812	Lighthouse Lab in Alderley Park	Wellcome Sanger Institute for the COVID-19 Genomics UK (COG-UK) consortium	Jacquelyn Wynn, Mairead Hyland, The Lighthouse Lab in Alderley Park and Alex Alderton, Roberto Amato, Sonia Goncalves, Ewan Harrison, David K. Jackson, Ian Johnston, Dominic Kwiatkowski, Cordelia Langford, John Sillitoe on behalf of the Wellcome Sanger Institute COVID-19 Surveillance Team
EPI_ISL_630813	Lighthouse Lab in Glasgow	Wellcome Sanger Institute for the COVID-19 Genomics UK (COG-UK) consortium	Harper VanSteenhouse, Yumi Kasai, David Gray, Carol Clugston, Anna Dominiczak and Alex Alderton, Roberto Amato, Sonia Goncalves, Ewan Harrison, David K. Jackson, Ian Johnston, Dominic Kwiatkowski, Cordelia Langford, John Sillitoe on behalf of the Wellcome Sanger Institute COVID-19 Surveillance Team
EPI_ISL_630814	Lighthouse Lab in Alderley Park	Wellcome Sanger Institute for the COVID-19 Genomics UK (COG-UK) consortium	Jacquelyn Wynn, Mairead Hyland, The Lighthouse Lab in Alderley Park and Alex Alderton, Roberto Amato, Sonia Goncalves, Ewan Harrison, David K. Jackson, Ian Johnston, Dominic Kwiatkowski, Cordelia Langford, John Sillitoe on behalf of the Wellcome Sanger Institute COVID-19 Surveillance Team
EPI_ISL_630815	Lighthouse Lab in Glasgow	Wellcome Sanger Institute for the COVID-19 Genomics UK (COG-UK) consortium	Harper VanSteenhouse, Yumi Kasai, David Gray, Carol Clugston, Anna Dominiczak and Alex Alderton, Roberto Amato, Sonia Goncalves, Ewan Harrison, David K. Jackson, Ian Johnston, Dominic Kwiatkowski, Cordelia Langford, John Sillitoe on behalf of the Wellcome Sanger Institute COVID-19 Surveillance Team
EPI_ISL_630816, EPI_ISL_630817, EPI_ISL_630818, EPI_ISL_630819, EPI_ISL_630820, EPI_ISL_630821, EPI_ISL_630822, EPI_ISL_630823	Lighthouse Lab in Alderley Park	Wellcome Sanger Institute for the COVID-19 Genomics UK (COG-UK) consortium	Jacquelyn Wynn, Mairead Hyland, The Lighthouse Lab in Alderley Park and Alex Alderton, Roberto Amato, Sonia Goncalves, Ewan Harrison, David K. Jackson, Ian Johnston, Dominic Kwiatkowski, Cordelia Langford, John Sillitoe on behalf of the Wellcome Sanger Institute COVID-19 Surveillance Team
EPI_ISL_630824	Lighthouse Lab in Glasgow	Wellcome Sanger Institute for the COVID-19 Genomics UK (COG-UK) consortium	Harper VanSteenhouse, Yumi Kasai, David Gray, Carol Clugston, Anna Dominiczak and Alex Alderton, Roberto Amato, Sonia Goncalves, Ewan Harrison, David K. Jackson, Ian Johnston, Dominic Kwiatkowski, Cordelia Langford, John Sillitoe on behalf of the Wellcome Sanger Institute COVID-19 Surveillance Team
EPI_ISL_630825, EPI_ISL_630826	Lighthouse Lab in Alderley Park	Wellcome Sanger Institute for the COVID-19 Genomics UK (COG-UK) consortium	Jacquelyn Wynn, Mairead Hyland, The Lighthouse Lab in Alderley Park and Alex Alderton, Roberto Amato, Sonia Goncalves, Ewan Harrison, David K. Jackson, Ian Johnston, Dominic Kwiatkowski, Cordelia Langford, John Sillitoe on behalf of the Wellcome Sanger Institute COVID-19 Surveillance Team
EPI_ISL_630827	Lighthouse Lab in Glasgow	Wellcome Sanger Institute for the COVID-19 Genomics UK (COG-UK) consortium	Harper VanSteenhouse, Yumi Kasai, David Gray, Carol Clugston, Anna Dominiczak and Alex Alderton, Roberto Amato, Sonia Goncalves, Ewan Harrison, David K. Jackson, Ian Johnston, Dominic Kwiatkowski, Cordelia Langford, John Sillitoe on behalf of the Wellcome Sanger Institute COVID-19 Surveillance Team
EPI_ISL_630828, EPI_ISL_630829, EPI_ISL_630830, EPI_ISL_630831, EPI_ISL_630832	Lighthouse Lab in Alderley Park	Wellcome Sanger Institute for the COVID-19 Genomics UK (COG-UK) consortium	Jacquelyn Wynn, Mairead Hyland, The Lighthouse Lab in Alderley Park and Alex Alderton, Roberto Amato, Sonia Goncalves, Ewan Harrison, David K. Jackson, Ian Johnston, Dominic Kwiatkowski, Cordelia Langford, John Sillitoe on behalf of the Wellcome Sanger Institute COVID-19 Surveillance Team
EPI_ISL_630833	Lighthouse Lab in Glasgow	Wellcome Sanger Institute for the COVID-19 Genomics UK (COG-UK) consortium	Harper VanSteenhouse, Yumi Kasai, David Gray, Carol Clugston, Anna Dominiczak and Alex Alderton, Roberto Amato, Sonia Goncalves, Ewan Harrison, David K. Jackson, Ian Johnston, Dominic Kwiatkowski, Cordelia Langford, John Sillitoe on behalf of the Wellcome Sanger Institute COVID-19 Surveillance Team
EPI_ISL_630835, EPI_ISL_630836, EPI_ISL_630837	Lighthouse Lab in Alderley Park	Wellcome Sanger Institute for the COVID-19 Genomics UK (COG-UK) consortium	Jacquelyn Wynn, Mairead Hyland, The Lighthouse Lab in Alderley Park and Alex Alderton, Roberto Amato, Sonia Goncalves, Ewan Harrison, David K. Jackson, Ian Johnston, Dominic Kwiatkowski, Cordelia Langford, John Sillitoe on behalf of the Wellcome Sanger Institute COVID-19 Surveillance Team
EPI_ISL_630838	Lighthouse Lab in Glasgow	Wellcome Sanger Institute for the COVID-19 Genomics UK (COG-UK) consortium	Harper VanSteenhouse, Yumi Kasai, David Gray, Carol Clugston, Anna Dominiczak and Alex Alderton, Roberto Amato, Sonia Goncalves, Ewan Harrison, David K. Jackson, Ian Johnston, Dominic Kwiatkowski, Cordelia Langford, John Sillitoe on behalf of the Wellcome Sanger Institute COVID-19 Surveillance Team
EPI_ISL_630839, EPI_ISL_630840, EPI_ISL_630841, EPI_ISL_630842, EPI_ISL_630843, EPI_ISL_630844, EPI_ISL_630845, EPI_ISL_630846, EPI_ISL_630847, EPI_ISL_630848, EPI_ISL_630849			
see above	Lighthouse Lab in Alderley Park	Wellcome Sanger Institute for the COVID-19 Genomics UK (COG-UK) consortium	Jacquelyn Wynn, Mairead Hyland, The Lighthouse Lab in Alderley Park and Alex Alderton, Roberto Amato, Sonia Goncalves, Ewan Harrison, David K. Jackson, Ian Johnston, Dominic Kwiatkowski, Cordelia Langford, John Sillitoe on behalf of the Wellcome Sanger Institute COVID-19 Surveillance Team
EPI_ISL_630850, EPI_ISL_630851	Lighthouse Lab in Glasgow	Wellcome Sanger Institute for the COVID-19 Genomics UK (COG-UK) consortium	Harper VanSteenhouse, Yumi Kasai, David Gray, Carol Clugston, Anna Dominiczak and Alex Alderton, Roberto Amato, Sonia Goncalves, Ewan Harrison, David K. Jackson, Ian Johnston, Dominic Kwiatkowski, Cordelia Langford, John Sillitoe on behalf of the Wellcome Sanger Institute COVID-19 Surveillance Team

EPI_ISL_630852	Lighthouse Lab in Alderley Park	Wellcome Sanger Institute for the COVID-19 Genomics UK (COG-UK) consortium	Jacquelyn Wynn, Mairead Hyland, The Lighthouse Lab in Alderley Park and Alex Alderton, Roberto Amato, Sonia Goncalves, Ewan Harrison, David K. Jackson, Ian Johnston, Dominic Kwiatkowski, Cordelia Langford, John Sillitoe on behalf of the Wellcome Sanger Institute COVID-19 Surveillance Team
EPI_ISL_630853	Lighthouse Lab in Glasgow	Wellcome Sanger Institute for the COVID-19 Genomics UK (COG-UK) consortium	Harper VanSteenhouse, Yumi Kasai, David Gray, Carol Clugston, Anna Dominiczak and Alex Alderton, Roberto Amato, Sonia Goncalves, Ewan Harrison, David K. Jackson, Ian Johnston, Dominic Kwiatkowski, Cordelia Langford, John Sillitoe on behalf of the Wellcome Sanger Institute COVID-19 Surveillance Team
EPI_ISL_630854, EPI_ISL_630855, EPI_ISL_630856, EPI_ISL_630857, EPI_ISL_630858	Lighthouse Lab in Alderley Park	Wellcome Sanger Institute for the COVID-19 Genomics UK (COG-UK) consortium	Jacquelyn Wynn, Mairead Hyland, The Lighthouse Lab in Alderley Park and Alex Alderton, Roberto Amato, Sonia Goncalves, Ewan Harrison, David K. Jackson, Ian Johnston, Dominic Kwiatkowski, Cordelia Langford, John Sillitoe on behalf of the Wellcome Sanger Institute COVID-19 Surveillance Team
EPI_ISL_630859	Lighthouse Lab in Glasgow	Wellcome Sanger Institute for the COVID-19 Genomics UK (COG-UK) consortium	Harper VanSteenhouse, Yumi Kasai, David Gray, Carol Clugston, Anna Dominiczak and Alex Alderton, Roberto Amato, Sonia Goncalves, Ewan Harrison, David K. Jackson, Ian Johnston, Dominic Kwiatkowski, Cordelia Langford, John Sillitoe on behalf of the Wellcome Sanger Institute COVID-19 Surveillance Team
EPI_ISL_630860, EPI_ISL_630861	Lighthouse Lab in Alderley Park	Wellcome Sanger Institute for the COVID-19 Genomics UK (COG-UK) consortium	Jacquelyn Wynn, Mairead Hyland, The Lighthouse Lab in Alderley Park and Alex Alderton, Roberto Amato, Sonia Goncalves, Ewan Harrison, David K. Jackson, Ian Johnston, Dominic Kwiatkowski, Cordelia Langford, John Sillitoe on behalf of the Wellcome Sanger Institute COVID-19 Surveillance Team
EPI_ISL_630862	Lighthouse Lab in Glasgow	Wellcome Sanger Institute for the COVID-19 Genomics UK (COG-UK) consortium	Harper VanSteenhouse, Yumi Kasai, David Gray, Carol Clugston, Anna Dominiczak and Alex Alderton, Roberto Amato, Sonia Goncalves, Ewan Harrison, David K. Jackson, Ian Johnston, Dominic Kwiatkowski, Cordelia Langford, John Sillitoe on behalf of the Wellcome Sanger Institute COVID-19 Surveillance Team
EPI_ISL_630863	Lighthouse Lab in Alderley Park	Wellcome Sanger Institute for the COVID-19 Genomics UK (COG-UK) consortium	Jacquelyn Wynn, Mairead Hyland, The Lighthouse Lab in Alderley Park and Alex Alderton, Roberto Amato, Sonia Goncalves, Ewan Harrison, David K. Jackson, Ian Johnston, Dominic Kwiatkowski, Cordelia Langford, John Sillitoe on behalf of the Wellcome Sanger Institute COVID-19 Surveillance Team
EPI_ISL_630864	Lighthouse Lab in Glasgow	Wellcome Sanger Institute for the COVID-19 Genomics UK (COG-UK) consortium	Harper VanSteenhouse, Yumi Kasai, David Gray, Carol Clugston, Anna Dominiczak and Alex Alderton, Roberto Amato, Sonia Goncalves, Ewan Harrison, David K. Jackson, Ian Johnston, Dominic Kwiatkowski, Cordelia Langford, John Sillitoe on behalf of the Wellcome Sanger Institute COVID-19 Surveillance Team
EPI_ISL_630865, EPI_ISL_630866, EPI_ISL_630867, EPI_ISL_630868, EPI_ISL_630869	Lighthouse Lab in Alderley Park	Wellcome Sanger Institute for the COVID-19 Genomics UK (COG-UK) consortium	Jacquelyn Wynn, Mairead Hyland, The Lighthouse Lab in Alderley Park and Alex Alderton, Roberto Amato, Sonia Goncalves, Ewan Harrison, David K. Jackson, Ian Johnston, Dominic Kwiatkowski, Cordelia Langford, John Sillitoe on behalf of the Wellcome Sanger Institute COVID-19 Surveillance Team
EPI_ISL_630870, EPI_ISL_630871, EPI_ISL_630872, EPI_ISL_630873	Lighthouse Lab in Glasgow	Wellcome Sanger Institute for the COVID-19 Genomics UK (COG-UK) consortium	Harper VanSteenhouse, Yumi Kasai, David Gray, Carol Clugston, Anna Dominiczak and Alex Alderton, Roberto Amato, Sonia Goncalves, Ewan Harrison, David K. Jackson, Ian Johnston, Dominic Kwiatkowski, Cordelia Langford, John Sillitoe on behalf of the Wellcome Sanger Institute COVID-19 Surveillance Team
EPI_ISL_630874	Lighthouse Lab in Alderley Park	Wellcome Sanger Institute for the COVID-19 Genomics UK (COG-UK) consortium	Jacquelyn Wynn, Mairead Hyland, The Lighthouse Lab in Alderley Park and Alex Alderton, Roberto Amato, Sonia Goncalves, Ewan Harrison, David K. Jackson, Ian Johnston, Dominic Kwiatkowski, Cordelia Langford, John Sillitoe on behalf of the Wellcome Sanger Institute COVID-19 Surveillance Team
EPI_ISL_630875, EPI_ISL_630876	Lighthouse Lab in Glasgow	Wellcome Sanger Institute for the COVID-19 Genomics UK (COG-UK) consortium	Harper VanSteenhouse, Yumi Kasai, David Gray, Carol Clugston, Anna Dominiczak and Alex Alderton, Roberto Amato, Sonia Goncalves, Ewan Harrison, David K. Jackson, Ian Johnston, Dominic Kwiatkowski, Cordelia Langford, John Sillitoe on behalf of the Wellcome Sanger Institute COVID-19 Surveillance Team
EPI_ISL_630877, EPI_ISL_630878, EPI_ISL_630879, EPI_ISL_630880, EPI_ISL_630881, EPI_ISL_630882	Lighthouse Lab in Alderley Park	Wellcome Sanger Institute for the COVID-19 Genomics UK (COG-UK) consortium	Jacquelyn Wynn, Mairead Hyland, The Lighthouse Lab in Alderley Park and Alex Alderton, Roberto Amato, Sonia Goncalves, Ewan Harrison, David K. Jackson, Ian Johnston, Dominic Kwiatkowski, Cordelia Langford, John Sillitoe on behalf of the Wellcome Sanger Institute COVID-19 Surveillance Team
EPI_ISL_630883	Lighthouse Lab in Glasgow	Wellcome Sanger Institute for the COVID-19 Genomics UK (COG-UK) consortium	Harper VanSteenhouse, Yumi Kasai, David Gray, Carol Clugston, Anna Dominiczak and Alex Alderton, Roberto Amato, Sonia Goncalves, Ewan Harrison, David K. Jackson, Ian Johnston, Dominic Kwiatkowski, Cordelia Langford, John Sillitoe on behalf of the Wellcome Sanger Institute COVID-19 Surveillance Team
EPI_ISL_630884, EPI_ISL_630885, EPI_ISL_630886, EPI_ISL_630887	Lighthouse Lab in Alderley Park	Wellcome Sanger Institute for the COVID-19 Genomics UK (COG-UK) consortium	Jacquelyn Wynn, Mairead Hyland, The Lighthouse Lab in Alderley Park and Alex Alderton, Roberto Amato, Sonia Goncalves, Ewan Harrison, David K. Jackson, Ian Johnston, Dominic Kwiatkowski, Cordelia Langford, John Sillitoe on behalf of the Wellcome Sanger Institute COVID-19 Surveillance Team
EPI_ISL_630888	Lighthouse Lab in Glasgow	Wellcome Sanger Institute for the COVID-19 Genomics UK (COG-UK) consortium	Harper VanSteenhouse, Yumi Kasai, David Gray, Carol Clugston, Anna Dominiczak and Alex Alderton, Roberto Amato, Sonia Goncalves, Ewan Harrison, David K. Jackson, Ian Johnston, Dominic Kwiatkowski, Cordelia Langford, John Sillitoe on behalf of the Wellcome Sanger Institute COVID-19 Surveillance Team
EPI_ISL_630889, EPI_ISL_630890	Lighthouse Lab in Alderley Park	Wellcome Sanger Institute for the COVID-19 Genomics UK (COG-UK) consortium	Jacquelyn Wynn, Mairead Hyland, The Lighthouse Lab in Alderley Park and Alex Alderton, Roberto Amato, Sonia Goncalves, Ewan Harrison, David K. Jackson, Ian Johnston, Dominic Kwiatkowski, Cordelia Langford, John Sillitoe on behalf of the Wellcome Sanger Institute COVID-19 Surveillance Team
EPI_ISL_630891	Lighthouse Lab in Alderley Park	Wellcome Sanger Institute for the COVID-19 Genomics UK (COG-UK) Consortium	Jacquelyn Wynn, Mairead Hyland, The Lighthouse Lab in Alderley Park and Alex Alderton, Roberto Amato, Sonia Goncalves, Ewan Harrison, David K. Jackson, Ian Johnston, Dominic Kwiatkowski, Cordelia Langford, John Sillitoe on behalf of the Wellcome Sanger Institute COVID-19 Surveillance Team
EPI_ISL_630892, EPI_ISL_630893, EPI_ISL_630894, EPI_ISL_630895, EPI_ISL_630896, EPI_ISL_630897, EPI_ISL_630898, EPI_ISL_630899	Lighthouse Lab in Alderley Park	Wellcome Sanger Institute for the COVID-19 Genomics UK (COG-UK) consortium	Jacquelyn Wynn, Mairead Hyland, The Lighthouse Lab in Alderley Park and Alex Alderton, Roberto Amato, Sonia Goncalves, Ewan Harrison, David K. Jackson, Ian Johnston, Dominic Kwiatkowski, Cordelia Langford, John Sillitoe on behalf of the Wellcome Sanger Institute COVID-19 Surveillance Team
EPI_ISL_630900	Lighthouse Lab in Glasgow	Wellcome Sanger Institute for the COVID-19 Genomics UK (COG-UK) consortium	Harper VanSteenhouse, Yumi Kasai, David Gray, Carol Clugston, Anna Dominiczak and Alex Alderton, Roberto Amato, Sonia Goncalves, Ewan Harrison, David K. Jackson, Ian Johnston, Dominic Kwiatkowski, Cordelia Langford, John Sillitoe on behalf of the Wellcome Sanger Institute COVID-19 Surveillance Team
EPI_ISL_630901, EPI_ISL_630902, EPI_ISL_630903, EPI_ISL_630904, EPI_ISL_630905, EPI_ISL_630906, EPI_ISL_630907, EPI_ISL_630908, EPI_ISL_630909, EPI_ISL_630910, EPI_ISL_630911, EPI_ISL_630912, EPI_ISL_630913, EPI_ISL_630914			
see above	Lighthouse Lab in Alderley Park	Wellcome Sanger Institute for the COVID-19 Genomics UK (COG-UK) consortium	Jacquelyn Wynn, Mairead Hyland, The Lighthouse Lab in Alderley Park and Alex Alderton, Roberto Amato, Sonia Goncalves, Ewan Harrison, David K. Jackson, Ian Johnston, Dominic Kwiatkowski, Cordelia Langford, John Sillitoe on behalf of the Wellcome Sanger Institute COVID-19 Surveillance Team
EPI_ISL_630915	Lighthouse Lab in Glasgow	Wellcome Sanger Institute for the COVID-19 Genomics UK (COG-UK) consortium	Harper VanSteenhouse, Yumi Kasai, David Gray, Carol Clugston, Anna Dominiczak and Alex Alderton, Roberto Amato, Sonia Goncalves, Ewan Harrison, David K. Jackson, Ian Johnston, Dominic Kwiatkowski, Cordelia Langford, John Sillitoe on behalf of the Wellcome Sanger Institute COVID-19 Surveillance Team
EPI_ISL_630916, EPI_ISL_630917, EPI_ISL_630918, EPI_ISL_630920, EPI_ISL_630921, EPI_ISL_630922	Lighthouse Lab in Alderley Park	Wellcome Sanger Institute for the COVID-19 Genomics UK (COG-UK) consortium	Jacquelyn Wynn, Mairead Hyland, The Lighthouse Lab in Alderley Park and Alex Alderton, Roberto Amato, Sonia Goncalves, Ewan Harrison, David K. Jackson, Ian Johnston, Dominic Kwiatkowski, Cordelia Langford, John Sillitoe on behalf of the Wellcome Sanger Institute COVID-19 Surveillance Team
EPI_ISL_630923	Lighthouse Lab in Glasgow	Wellcome Sanger Institute for the COVID-19 Genomics UK (COG-UK) consortium	Harper VanSteenhouse, Yumi Kasai, David Gray, Carol Clugston, Anna Dominiczak and Alex Alderton, Roberto Amato, Sonia Goncalves, Ewan Harrison, David K. Jackson, Ian Johnston, Dominic Kwiatkowski, Cordelia Langford, John Sillitoe on behalf of the Wellcome Sanger Institute COVID-19 Surveillance Team
EPI_ISL_630924, EPI_ISL_630925	Lighthouse Lab in Alderley Park	Wellcome Sanger Institute for the COVID-19 Genomics UK (COG-UK) consortium	Jacquelyn Wynn, Mairead Hyland, The Lighthouse Lab in Alderley Park and Alex Alderton, Roberto Amato, Sonia Goncalves, Ewan Harrison, David K. Jackson, Ian Johnston, Dominic Kwiatkowski, Cordelia Langford, John Sillitoe on behalf of the Wellcome Sanger Institute COVID-19 Surveillance Team
EPI_ISL_630926	Lighthouse Lab in Glasgow	Wellcome Sanger Institute for the COVID-19 Genomics UK (COG-UK) consortium	Harper VanSteenhouse, Yumi Kasai, David Gray, Carol Clugston, Anna Dominiczak and Alex Alderton, Roberto Amato, Sonia Goncalves, Ewan Harrison, David K. Jackson, Ian Johnston, Dominic Kwiatkowski, Cordelia Langford, John Sillitoe on behalf of the Wellcome Sanger Institute COVID-19 Surveillance Team
EPI_ISL_630927, EPI_ISL_630928, EPI_ISL_630929, EPI_ISL_630930	Lighthouse Lab in Alderley Park	Wellcome Sanger Institute for the COVID-19 Genomics UK (COG-UK) consortium	Jacquelyn Wynn, Mairead Hyland, The Lighthouse Lab in Alderley Park and Alex Alderton, Roberto Amato, Sonia Goncalves, Ewan Harrison, David K. Jackson, Ian Johnston, Dominic Kwiatkowski, Cordelia Langford, John Sillitoe on behalf of the Wellcome Sanger Institute COVID-19 Surveillance Team

EPI_ISL_630931	Lighthouse Lab in Glasgow	Wellcome Sanger Institute for the COVID-19 Genomics UK (COG-UK) consortium	Harper VanSteenhouse, Yumi Kasai, David Gray, Carol Clugston, Anna Dominiczak and Alex Alderton, Roberto Amato, Sonia Goncalves, Ewan Harrison, David K. Jackson, Ian Johnston, Dominic Kwiatkowski, Cordelia Langford, John Sillitoe on behalf of the Wellcome Sanger Institute COVID-19 Surveillance Team
EPI_ISL_630932	Lighthouse Lab in Alderley Park	Wellcome Sanger Institute for the COVID-19 Genomics UK (COG-UK) consortium	Jacquelyn Wynn, Mairead Hyland, The Lighthouse Lab in Alderley Park and Alex Alderton, Roberto Amato, Sonia Goncalves, Ewan Harrison, David K. Jackson, Ian Johnston, Dominic Kwiatkowski, Cordelia Langford, John Sillitoe on behalf of the Wellcome Sanger Institute COVID-19 Surveillance Team
EPI_ISL_630933	Lighthouse Lab in Glasgow	Wellcome Sanger Institute for the COVID-19 Genomics UK (COG-UK) consortium	Harper VanSteenhouse, Yumi Kasai, David Gray, Carol Clugston, Anna Dominiczak and Alex Alderton, Roberto Amato, Sonia Goncalves, Ewan Harrison, David K. Jackson, Ian Johnston, Dominic Kwiatkowski, Cordelia Langford, John Sillitoe on behalf of the Wellcome Sanger Institute COVID-19 Surveillance Team
EPI_ISL_630934, EPI_ISL_630935, EPI_ISL_630937, EPI_ISL_630938, EPI_ISL_630939, EPI_ISL_630940	Lighthouse Lab in Alderley Park	Wellcome Sanger Institute for the COVID-19 Genomics UK (COG-UK) consortium	Jacquelyn Wynn, Mairead Hyland, The Lighthouse Lab in Alderley Park and Alex Alderton, Roberto Amato, Sonia Goncalves, Ewan Harrison, David K. Jackson, Ian Johnston, Dominic Kwiatkowski, Cordelia Langford, John Sillitoe on behalf of the Wellcome Sanger Institute COVID-19 Surveillance Team
EPI_ISL_630941	Lighthouse Lab in Glasgow	Wellcome Sanger Institute for the COVID-19 Genomics UK (COG-UK) consortium	Harper VanSteenhouse, Yumi Kasai, David Gray, Carol Clugston, Anna Dominiczak and Alex Alderton, Roberto Amato, Sonia Goncalves, Ewan Harrison, David K. Jackson, Ian Johnston, Dominic Kwiatkowski, Cordelia Langford, John Sillitoe on behalf of the Wellcome Sanger Institute COVID-19 Surveillance Team
EPI_ISL_630942, EPI_ISL_630943, EPI_ISL_630944, EPI_ISL_630945, EPI_ISL_630946, EPI_ISL_630947	Lighthouse Lab in Alderley Park	Wellcome Sanger Institute for the COVID-19 Genomics UK (COG-UK) consortium	Jacquelyn Wynn, Mairead Hyland, The Lighthouse Lab in Alderley Park and Alex Alderton, Roberto Amato, Sonia Goncalves, Ewan Harrison, David K. Jackson, Ian Johnston, Dominic Kwiatkowski, Cordelia Langford, John Sillitoe on behalf of the Wellcome Sanger Institute COVID-19 Surveillance Team
EPI_ISL_630948, EPI_ISL_630949	Lighthouse Lab in Glasgow	Wellcome Sanger Institute for the COVID-19 Genomics UK (COG-UK) consortium	Harper VanSteenhouse, Yumi Kasai, David Gray, Carol Clugston, Anna Dominiczak and Alex Alderton, Roberto Amato, Sonia Goncalves, Ewan Harrison, David K. Jackson, Ian Johnston, Dominic Kwiatkowski, Cordelia Langford, John Sillitoe on behalf of the Wellcome Sanger Institute COVID-19 Surveillance Team
EPI_ISL_630950	Lighthouse Lab in Alderley Park	Wellcome Sanger Institute for the COVID-19 Genomics UK (COG-UK) consortium	Jacquelyn Wynn, Mairead Hyland, The Lighthouse Lab in Alderley Park and Alex Alderton, Roberto Amato, Sonia Goncalves, Ewan Harrison, David K. Jackson, Ian Johnston, Dominic Kwiatkowski, Cordelia Langford, John Sillitoe on behalf of the Wellcome Sanger Institute COVID-19 Surveillance Team
EPI_ISL_630951	Lighthouse Lab in Glasgow	Wellcome Sanger Institute for the COVID-19 Genomics UK (COG-UK) consortium	Harper VanSteenhouse, Yumi Kasai, David Gray, Carol Clugston, Anna Dominiczak and Alex Alderton, Roberto Amato, Sonia Goncalves, Ewan Harrison, David K. Jackson, Ian Johnston, Dominic Kwiatkowski, Cordelia Langford, John Sillitoe on behalf of the Wellcome Sanger Institute COVID-19 Surveillance Team
EPI_ISL_630952, EPI_ISL_630953, EPI_ISL_630954	Lighthouse Lab in Alderley Park	Wellcome Sanger Institute for the COVID-19 Genomics UK (COG-UK) consortium	Jacquelyn Wynn, Mairead Hyland, The Lighthouse Lab in Alderley Park and Alex Alderton, Roberto Amato, Sonia Goncalves, Ewan Harrison, David K. Jackson, Ian Johnston, Dominic Kwiatkowski, Cordelia Langford, John Sillitoe on behalf of the Wellcome Sanger Institute COVID-19 Surveillance Team
EPI_ISL_630955	Lighthouse Lab in Glasgow	Wellcome Sanger Institute for the COVID-19 Genomics UK (COG-UK) consortium	Harper VanSteenhouse, Yumi Kasai, David Gray, Carol Clugston, Anna Dominiczak and Alex Alderton, Roberto Amato, Sonia Goncalves, Ewan Harrison, David K. Jackson, Ian Johnston, Dominic Kwiatkowski, Cordelia Langford, John Sillitoe on behalf of the Wellcome Sanger Institute COVID-19 Surveillance Team
EPI_ISL_630956	Lighthouse Lab in Alderley Park	Wellcome Sanger Institute for the COVID-19 Genomics UK (COG-UK) consortium	Jacquelyn Wynn, Mairead Hyland, The Lighthouse Lab in Alderley Park and Alex Alderton, Roberto Amato, Sonia Goncalves, Ewan Harrison, David K. Jackson, Ian Johnston, Dominic Kwiatkowski, Cordelia Langford, John Sillitoe on behalf of the Wellcome Sanger Institute COVID-19 Surveillance Team
EPI_ISL_630957	Lighthouse Lab in Glasgow	Wellcome Sanger Institute for the COVID-19 Genomics UK (COG-UK) consortium	Harper VanSteenhouse, Yumi Kasai, David Gray, Carol Clugston, Anna Dominiczak and Alex Alderton, Roberto Amato, Sonia Goncalves, Ewan Harrison, David K. Jackson, Ian Johnston, Dominic Kwiatkowski, Cordelia Langford, John Sillitoe on behalf of the Wellcome Sanger Institute COVID-19 Surveillance Team
EPI_ISL_630958	Lighthouse Lab in Alderley Park	Wellcome Sanger Institute for the COVID-19 Genomics UK (COG-UK) consortium	Jacquelyn Wynn, Mairead Hyland, The Lighthouse Lab in Alderley Park and Alex Alderton, Roberto Amato, Sonia Goncalves, Ewan Harrison, David K. Jackson, Ian Johnston, Dominic Kwiatkowski, Cordelia Langford, John Sillitoe on behalf of the Wellcome Sanger Institute COVID-19 Surveillance Team
EPI_ISL_630959	Lighthouse Lab in Glasgow	Wellcome Sanger Institute for the COVID-19 Genomics UK (COG-UK) consortium	Harper VanSteenhouse, Yumi Kasai, David Gray, Carol Clugston, Anna Dominiczak and Alex Alderton, Roberto Amato, Sonia Goncalves, Ewan Harrison, David K. Jackson, Ian Johnston, Dominic Kwiatkowski, Cordelia Langford, John Sillitoe on behalf of the Wellcome Sanger Institute COVID-19 Surveillance Team
EPI_ISL_630960, EPI_ISL_630961, EPI_ISL_630962, EPI_ISL_630963, EPI_ISL_630964	Lighthouse Lab in Alderley Park	Wellcome Sanger Institute for the COVID-19 Genomics UK (COG-UK) consortium	Jacquelyn Wynn, Mairead Hyland, The Lighthouse Lab in Alderley Park and Alex Alderton, Roberto Amato, Sonia Goncalves, Ewan Harrison, David K. Jackson, Ian Johnston, Dominic Kwiatkowski, Cordelia Langford, John Sillitoe on behalf of the Wellcome Sanger Institute COVID-19 Surveillance Team
EPI_ISL_630965	Lighthouse Lab in Glasgow	Wellcome Sanger Institute for the COVID-19 Genomics UK (COG-UK) consortium	Harper VanSteenhouse, Yumi Kasai, David Gray, Carol Clugston, Anna Dominiczak and Alex Alderton, Roberto Amato, Sonia Goncalves, Ewan Harrison, David K. Jackson, Ian Johnston, Dominic Kwiatkowski, Cordelia Langford, John Sillitoe on behalf of the Wellcome Sanger Institute COVID-19 Surveillance Team
EPI_ISL_630966, EPI_ISL_630967, EPI_ISL_630968, EPI_ISL_630969, EPI_ISL_630970, EPI_ISL_630971	Lighthouse Lab in Alderley Park	Wellcome Sanger Institute for the COVID-19 Genomics UK (COG-UK) consortium	Jacquelyn Wynn, Mairead Hyland, The Lighthouse Lab in Alderley Park and Alex Alderton, Roberto Amato, Sonia Goncalves, Ewan Harrison, David K. Jackson, Ian Johnston, Dominic Kwiatkowski, Cordelia Langford, John Sillitoe on behalf of the Wellcome Sanger Institute COVID-19 Surveillance Team
EPI_ISL_630972	Lighthouse Lab in Cambridge	Wellcome Sanger Institute for the COVID-19 Genomics UK (COG-UK) consortium	Rob Howes, The Lighthouse Lab in Cambridge and Alex Alderton, Roberto Amato, Sonia Goncalves, Ewan Harrison, David K. Jackson, Ian Johnston, Dominic Kwiatkowski, Cordelia Langford, John Sillitoe on behalf of the Wellcome Sanger Institute COVID-19 Surveillance Team
EPI_ISL_630973, EPI_ISL_630974	Lighthouse Lab in Milton Keynes	Wellcome Sanger Institute for the COVID-19 Genomics UK (COG-UK) consortium	The Lighthouse Lab in Milton Keynes and Alex Alderton, Roberto Amato, Sonia Goncalves, Ewan Harrison, David K. Jackson, Ian Johnston, Dominic Kwiatkowski, Cordelia Langford, John Sillitoe on behalf of the Wellcome Sanger Institute COVID-19 Surveillance Team
EPI_ISL_630975, EPI_ISL_630976	Lighthouse Lab in Alderley Park	Wellcome Sanger Institute for the COVID-19 Genomics UK (COG-UK) consortium	Jacquelyn Wynn, Mairead Hyland, The Lighthouse Lab in Alderley Park and Alex Alderton, Roberto Amato, Sonia Goncalves, Ewan Harrison, David K. Jackson, Ian Johnston, Dominic Kwiatkowski, Cordelia Langford, John Sillitoe on behalf of the Wellcome Sanger Institute COVID-19 Surveillance Team
EPI_ISL_630977	Lighthouse Lab in Cambridge	Wellcome Sanger Institute for the COVID-19 Genomics UK (COG-UK) consortium	Rob Howes, The Lighthouse Lab in Cambridge and Alex Alderton, Roberto Amato, Sonia Goncalves, Ewan Harrison, David K. Jackson, Ian Johnston, Dominic Kwiatkowski, Cordelia Langford, John Sillitoe on behalf of the Wellcome Sanger Institute COVID-19 Surveillance Team
EPI_ISL_630978	Lighthouse Lab in Milton Keynes	Wellcome Sanger Institute for the COVID-19 Genomics UK (COG-UK) consortium	The Lighthouse Lab in Milton Keynes and Alex Alderton, Roberto Amato, Sonia Goncalves, Ewan Harrison, David K. Jackson, Ian Johnston, Dominic Kwiatkowski, Cordelia Langford, John Sillitoe on behalf of the Wellcome Sanger Institute COVID-19 Surveillance Team
EPI_ISL_630979	Lighthouse Lab in Cambridge	Wellcome Sanger Institute for the COVID-19 Genomics UK (COG-UK) consortium	Rob Howes, The Lighthouse Lab in Cambridge and Alex Alderton, Roberto Amato, Sonia Goncalves, Ewan Harrison, David K. Jackson, Ian Johnston, Dominic Kwiatkowski, Cordelia Langford, John Sillitoe on behalf of the Wellcome Sanger Institute COVID-19 Surveillance Team
EPI_ISL_630981	Lighthouse Lab in Milton Keynes	Wellcome Sanger Institute for the COVID-19 Genomics UK (COG-UK) consortium	The Lighthouse Lab in Milton Keynes and Alex Alderton, Roberto Amato, Sonia Goncalves, Ewan Harrison, David K. Jackson, Ian Johnston, Dominic Kwiatkowski, Cordelia Langford, John Sillitoe on behalf of the Wellcome Sanger Institute COVID-19 Surveillance Team
EPI_ISL_630982, EPI_ISL_630983	Lighthouse Lab in Alderley Park	Wellcome Sanger Institute for the COVID-19 Genomics UK (COG-UK) consortium	Jacquelyn Wynn, Mairead Hyland, The Lighthouse Lab in Alderley Park and Alex Alderton, Roberto Amato, Sonia Goncalves, Ewan Harrison, David K. Jackson, Ian Johnston, Dominic Kwiatkowski, Cordelia Langford, John Sillitoe on behalf of the Wellcome Sanger Institute COVID-19 Surveillance Team
EPI_ISL_630984	Lighthouse Lab in Cambridge	Wellcome Sanger Institute for the COVID-19 Genomics UK (COG-UK) consortium	Rob Howes, The Lighthouse Lab in Cambridge and Alex Alderton, Roberto Amato, Sonia Goncalves, Ewan Harrison, David K. Jackson, Ian Johnston, Dominic Kwiatkowski, Cordelia Langford, John Sillitoe on behalf of the Wellcome Sanger Institute COVID-19 Surveillance Team
EPI_ISL_630985	Lighthouse Lab in Alderley Park	Wellcome Sanger Institute for the COVID-19 Genomics UK (COG-UK) consortium	Jacquelyn Wynn, Mairead Hyland, The Lighthouse Lab in Alderley Park and Alex Alderton, Roberto Amato, Sonia Goncalves, Ewan Harrison, David K. Jackson, Ian Johnston, Dominic Kwiatkowski, Cordelia Langford, John Sillitoe on behalf of the Wellcome Sanger Institute COVID-19 Surveillance Team
EPI_ISL_630986, EPI_ISL_630987	Lighthouse Lab in Milton Keynes	Wellcome Sanger Institute for the COVID-19 Genomics UK (COG-UK) consortium	The Lighthouse Lab in Milton Keynes and Alex Alderton, Roberto Amato, Sonia Goncalves, Ewan Harrison, David K. Jackson, Ian Johnston, Dominic Kwiatkowski, Cordelia Langford, John Sillitoe on behalf of the Wellcome Sanger Institute COVID-19 Surveillance Team
EPI_ISL_630988	Lighthouse Lab in Cambridge	Wellcome Sanger Institute for the COVID-19 Genomics UK (COG-UK) consortium	Rob Howes, The Lighthouse Lab in Cambridge and Alex Alderton, Roberto Amato, Sonia Goncalves, Ewan Harrison, David K. Jackson, Ian Johnston, Dominic Kwiatkowski, Cordelia Langford, John Sillitoe on behalf of the Wellcome Sanger Institute COVID-19 Surveillance Team

EPI_ISL_630990, EPI_ISL_630991	Lighthouse Lab in Milton Keynes	Wellcome Sanger Institute for the COVID-19 Genomics UK (COG-UK) consortium	The Lighthouse Lab in Milton Keynes and Alex Alderton, Roberto Amato, Sonia Goncalves, Ewan Harrison, David K. Jackson, Ian Johnston, Dominic Kwiatkowski, Cordelia Langford, John Sillitoe on behalf of the Wellcome Sanger Institute COVID-19 Surveillance Team
EPI_ISL_630992	Lighthouse Lab in Alderley Park	Wellcome Sanger Institute for the COVID-19 Genomics UK (COG-UK) consortium	Jacquelyn Wynn, Mairead Hyland, The Lighthouse Lab in Alderley Park and Alex Alderton, Roberto Amato, Sonia Goncalves, Ewan Harrison, David K. Jackson, Ian Johnston, Dominic Kwiatkowski, Cordelia Langford, John Sillitoe on behalf of the Wellcome Sanger Institute COVID-19 Surveillance Team
EPI_ISL_630993	Lighthouse Lab in Milton Keynes	Wellcome Sanger Institute for the COVID-19 Genomics UK (COG-UK) consortium	The Lighthouse Lab in Milton Keynes and Alex Alderton, Roberto Amato, Sonia Goncalves, Ewan Harrison, David K. Jackson, Ian Johnston, Dominic Kwiatkowski, Cordelia Langford, John Sillitoe on behalf of the Wellcome Sanger Institute COVID-19 Surveillance Team
EPI_ISL_630994	Lighthouse Lab in Alderley Park	Wellcome Sanger Institute for the COVID-19 Genomics UK (COG-UK) consortium	Jacquelyn Wynn, Mairead Hyland, The Lighthouse Lab in Alderley Park and Alex Alderton, Roberto Amato, Sonia Goncalves, Ewan Harrison, David K. Jackson, Ian Johnston, Dominic Kwiatkowski, Cordelia Langford, John Sillitoe on behalf of the Wellcome Sanger Institute COVID-19 Surveillance Team
EPI_ISL_630995	Lighthouse Lab in Milton Keynes	Wellcome Sanger Institute for the COVID-19 Genomics UK (COG-UK) consortium	The Lighthouse Lab in Milton Keynes and Alex Alderton, Roberto Amato, Sonia Goncalves, Ewan Harrison, David K. Jackson, Ian Johnston, Dominic Kwiatkowski, Cordelia Langford, John Sillitoe on behalf of the Wellcome Sanger Institute COVID-19 Surveillance Team
EPI_ISL_630996, EPI_ISL_630997	Lighthouse Lab in Cambridge	Wellcome Sanger Institute for the COVID-19 Genomics UK (COG-UK) consortium	Rob Howes, The Lighthouse Lab in Cambridge and Alex Alderton, Roberto Amato, Sonia Goncalves, Ewan Harrison, David K. Jackson, Ian Johnston, Dominic Kwiatkowski, Cordelia Langford, John Sillitoe on behalf of the Wellcome Sanger Institute COVID-19 Surveillance Team
EPI_ISL_630998	Lighthouse Lab in Milton Keynes	Wellcome Sanger Institute for the COVID-19 Genomics UK (COG-UK) consortium	The Lighthouse Lab in Milton Keynes and Alex Alderton, Roberto Amato, Sonia Goncalves, Ewan Harrison, David K. Jackson, Ian Johnston, Dominic Kwiatkowski, Cordelia Langford, John Sillitoe on behalf of the Wellcome Sanger Institute COVID-19 Surveillance Team
EPI_ISL_630999	Lighthouse Lab in Alderley Park	Wellcome Sanger Institute for the COVID-19 Genomics UK (COG-UK) consortium	Jacquelyn Wynn, Mairead Hyland, The Lighthouse Lab in Alderley Park and Alex Alderton, Roberto Amato, Sonia Goncalves, Ewan Harrison, David K. Jackson, Ian Johnston, Dominic Kwiatkowski, Cordelia Langford, John Sillitoe on behalf of the Wellcome Sanger Institute COVID-19 Surveillance Team
EPI_ISL_631000	Lighthouse Lab in Milton Keynes	Wellcome Sanger Institute for the COVID-19 Genomics UK (COG-UK) consortium	The Lighthouse Lab in Milton Keynes and Alex Alderton, Roberto Amato, Sonia Goncalves, Ewan Harrison, David K. Jackson, Ian Johnston, Dominic Kwiatkowski, Cordelia Langford, John Sillitoe on behalf of the Wellcome Sanger Institute COVID-19 Surveillance Team
EPI_ISL_631001	Lighthouse Lab in Cambridge	Wellcome Sanger Institute for the COVID-19 Genomics UK (COG-UK) consortium	Rob Howes, The Lighthouse Lab in Cambridge and Alex Alderton, Roberto Amato, Sonia Goncalves, Ewan Harrison, David K. Jackson, Ian Johnston, Dominic Kwiatkowski, Cordelia Langford, John Sillitoe on behalf of the Wellcome Sanger Institute COVID-19 Surveillance Team
EPI_ISL_631002	Lighthouse Lab in Milton Keynes	Wellcome Sanger Institute for the COVID-19 Genomics UK (COG-UK) consortium	The Lighthouse Lab in Milton Keynes and Alex Alderton, Roberto Amato, Sonia Goncalves, Ewan Harrison, David K. Jackson, Ian Johnston, Dominic Kwiatkowski, Cordelia Langford, John Sillitoe on behalf of the Wellcome Sanger Institute COVID-19 Surveillance Team
EPI_ISL_631003	Lighthouse Lab in Cambridge	Wellcome Sanger Institute for the COVID-19 Genomics UK (COG-UK) consortium	Rob Howes, The Lighthouse Lab in Cambridge and Alex Alderton, Roberto Amato, Sonia Goncalves, Ewan Harrison, David K. Jackson, Ian Johnston, Dominic Kwiatkowski, Cordelia Langford, John Sillitoe on behalf of the Wellcome Sanger Institute COVID-19 Surveillance Team
EPI_ISL_631004	Lighthouse Lab in Milton Keynes	Wellcome Sanger Institute for the COVID-19 Genomics UK (COG-UK) consortium	The Lighthouse Lab in Milton Keynes and Alex Alderton, Roberto Amato, Sonia Goncalves, Ewan Harrison, David K. Jackson, Ian Johnston, Dominic Kwiatkowski, Cordelia Langford, John Sillitoe on behalf of the Wellcome Sanger Institute COVID-19 Surveillance Team
EPI_ISL_631005	Lighthouse Lab in Cambridge	Wellcome Sanger Institute for the COVID-19 Genomics UK (COG-UK) consortium	Rob Howes, The Lighthouse Lab in Cambridge and Alex Alderton, Roberto Amato, Sonia Goncalves, Ewan Harrison, David K. Jackson, Ian Johnston, Dominic Kwiatkowski, Cordelia Langford, John Sillitoe on behalf of the Wellcome Sanger Institute COVID-19 Surveillance Team
EPI_ISL_631006, EPI_ISL_631007	Lighthouse Lab in Milton Keynes	Wellcome Sanger Institute for the COVID-19 Genomics UK (COG-UK) consortium	The Lighthouse Lab in Milton Keynes and Alex Alderton, Roberto Amato, Sonia Goncalves, Ewan Harrison, David K. Jackson, Ian Johnston, Dominic Kwiatkowski, Cordelia Langford, John Sillitoe on behalf of the Wellcome Sanger Institute COVID-19 Surveillance Team
EPI_ISL_631008	Lighthouse Lab in Cambridge	Wellcome Sanger Institute for the COVID-19 Genomics UK (COG-UK) consortium	Rob Howes, The Lighthouse Lab in Cambridge and Alex Alderton, Roberto Amato, Sonia Goncalves, Ewan Harrison, David K. Jackson, Ian Johnston, Dominic Kwiatkowski, Cordelia Langford, John Sillitoe on behalf of the Wellcome Sanger Institute COVID-19 Surveillance Team
EPI_ISL_631009, EPI_ISL_631010	Lighthouse Lab in Milton Keynes	Wellcome Sanger Institute for the COVID-19 Genomics UK (COG-UK) consortium	The Lighthouse Lab in Milton Keynes and Alex Alderton, Roberto Amato, Sonia Goncalves, Ewan Harrison, David K. Jackson, Ian Johnston, Dominic Kwiatkowski, Cordelia Langford, John Sillitoe on behalf of the Wellcome Sanger Institute COVID-19 Surveillance Team
EPI_ISL_631011, EPI_ISL_631012	Lighthouse Lab in Alderley Park	Wellcome Sanger Institute for the COVID-19 Genomics UK (COG-UK) consortium	Jacquelyn Wynn, Mairead Hyland, The Lighthouse Lab in Alderley Park and Alex Alderton, Roberto Amato, Sonia Goncalves, Ewan Harrison, David K. Jackson, Ian Johnston, Dominic Kwiatkowski, Cordelia Langford, John Sillitoe on behalf of the Wellcome Sanger Institute COVID-19 Surveillance Team
EPI_ISL_631013	Lighthouse Lab in Milton Keynes	Wellcome Sanger Institute for the COVID-19 Genomics UK (COG-UK) consortium	The Lighthouse Lab in Milton Keynes and Alex Alderton, Roberto Amato, Sonia Goncalves, Ewan Harrison, David K. Jackson, Ian Johnston, Dominic Kwiatkowski, Cordelia Langford, John Sillitoe on behalf of the Wellcome Sanger Institute COVID-19 Surveillance Team
EPI_ISL_631014	Lighthouse Lab in Alderley Park	Wellcome Sanger Institute for the COVID-19 Genomics UK (COG-UK) consortium	Jacquelyn Wynn, Mairead Hyland, The Lighthouse Lab in Alderley Park and Alex Alderton, Roberto Amato, Sonia Goncalves, Ewan Harrison, David K. Jackson, Ian Johnston, Dominic Kwiatkowski, Cordelia Langford, John Sillitoe on behalf of the Wellcome Sanger Institute COVID-19 Surveillance Team
EPI_ISL_631015	Lighthouse Lab in Milton Keynes	Wellcome Sanger Institute for the COVID-19 Genomics UK (COG-UK) consortium	The Lighthouse Lab in Milton Keynes and Alex Alderton, Roberto Amato, Sonia Goncalves, Ewan Harrison, David K. Jackson, Ian Johnston, Dominic Kwiatkowski, Cordelia Langford, John Sillitoe on behalf of the Wellcome Sanger Institute COVID-19 Surveillance Team
EPI_ISL_631016, EPI_ISL_631017	Lighthouse Lab in Cambridge	Wellcome Sanger Institute for the COVID-19 Genomics UK (COG-UK) consortium	Rob Howes, The Lighthouse Lab in Cambridge and Alex Alderton, Roberto Amato, Sonia Goncalves, Ewan Harrison, David K. Jackson, Ian Johnston, Dominic Kwiatkowski, Cordelia Langford, John Sillitoe on behalf of the Wellcome Sanger Institute COVID-19 Surveillance Team
EPI_ISL_631018	Lighthouse Lab in Alderley Park	Wellcome Sanger Institute for the COVID-19 Genomics UK (COG-UK) consortium	Jacquelyn Wynn, Mairead Hyland, The Lighthouse Lab in Alderley Park and Alex Alderton, Roberto Amato, Sonia Goncalves, Ewan Harrison, David K. Jackson, Ian Johnston, Dominic Kwiatkowski, Cordelia Langford, John Sillitoe on behalf of the Wellcome Sanger Institute COVID-19 Surveillance Team
EPI_ISL_631019, EPI_ISL_631020, EPI_ISL_631021, EPI_ISL_631022	Lighthouse Lab in Milton Keynes	Wellcome Sanger Institute for the COVID-19 Genomics UK (COG-UK) consortium	The Lighthouse Lab in Milton Keynes and Alex Alderton, Roberto Amato, Sonia Goncalves, Ewan Harrison, David K. Jackson, Ian Johnston, Dominic Kwiatkowski, Cordelia Langford, John Sillitoe on behalf of the Wellcome Sanger Institute COVID-19 Surveillance Team
EPI_ISL_631023	Lighthouse Lab in Alderley Park	Wellcome Sanger Institute for the COVID-19 Genomics UK (COG-UK) consortium	Jacquelyn Wynn, Mairead Hyland, The Lighthouse Lab in Alderley Park and Alex Alderton, Roberto Amato, Sonia Goncalves, Ewan Harrison, David K. Jackson, Ian Johnston, Dominic Kwiatkowski, Cordelia Langford, John Sillitoe on behalf of the Wellcome Sanger Institute COVID-19 Surveillance Team
EPI_ISL_631024	Lighthouse Lab in Milton Keynes	Wellcome Sanger Institute for the COVID-19 Genomics UK (COG-UK) consortium	The Lighthouse Lab in Milton Keynes and Alex Alderton, Roberto Amato, Sonia Goncalves, Ewan Harrison, David K. Jackson, Ian Johnston, Dominic Kwiatkowski, Cordelia Langford, John Sillitoe on behalf of the Wellcome Sanger Institute COVID-19 Surveillance Team
EPI_ISL_631025	Lighthouse Lab in Cambridge	Wellcome Sanger Institute for the COVID-19 Genomics UK (COG-UK) consortium	Rob Howes, The Lighthouse Lab in Cambridge and Alex Alderton, Roberto Amato, Sonia Goncalves, Ewan Harrison, David K. Jackson, Ian Johnston, Dominic Kwiatkowski, Cordelia Langford, John Sillitoe on behalf of the Wellcome Sanger Institute COVID-19 Surveillance Team
EPI_ISL_631026	Lighthouse Lab in Milton Keynes	Wellcome Sanger Institute for the COVID-19 Genomics UK (COG-UK) consortium	The Lighthouse Lab in Milton Keynes and Alex Alderton, Roberto Amato, Sonia Goncalves, Ewan Harrison, David K. Jackson, Ian Johnston, Dominic Kwiatkowski, Cordelia Langford, John Sillitoe on behalf of the Wellcome Sanger Institute COVID-19 Surveillance Team
EPI_ISL_631027	Lighthouse Lab in Alderley Park	Wellcome Sanger Institute for the COVID-19 Genomics UK (COG-UK) consortium	Jacquelyn Wynn, Mairead Hyland, The Lighthouse Lab in Alderley Park and Alex Alderton, Roberto Amato, Sonia Goncalves, Ewan Harrison, David K. Jackson, Ian Johnston, Dominic Kwiatkowski, Cordelia Langford, John Sillitoe on behalf of the Wellcome Sanger Institute COVID-19 Surveillance Team
EPI_ISL_631028	Lighthouse Lab in Cambridge	Wellcome Sanger Institute for the COVID-19 Genomics UK (COG-UK) consortium	Rob Howes, The Lighthouse Lab in Cambridge and Alex Alderton, Roberto Amato, Sonia Goncalves, Ewan Harrison, David K. Jackson, Ian Johnston, Dominic Kwiatkowski, Cordelia Langford, John Sillitoe on behalf of the Wellcome Sanger Institute COVID-19 Surveillance Team
EPI_ISL_631029, EPI_ISL_631030	Lighthouse Lab in Milton Keynes	Wellcome Sanger Institute for the COVID-19 Genomics UK (COG-UK) consortium	The Lighthouse Lab in Milton Keynes and Alex Alderton, Roberto Amato, Sonia Goncalves, Ewan Harrison, David K. Jackson, Ian Johnston, Dominic Kwiatkowski, Cordelia Langford, John Sillitoe on behalf of the Wellcome Sanger Institute COVID-19 Surveillance Team
EPI_ISL_631031	Lighthouse Lab in Alderley Park	Wellcome Sanger Institute for the COVID-19 Genomics UK (COG-UK) consortium	Jacquelyn Wynn, Mairead Hyland, The Lighthouse Lab in Alderley Park and Alex Alderton, Roberto Amato, Sonia Goncalves, Ewan Harrison, David K. Jackson, Ian Johnston, Dominic Kwiatkowski, Cordelia Langford, John Sillitoe on behalf of the Wellcome Sanger Institute COVID-19 Surveillance Team
EPI_ISL_631032	Lighthouse Lab in Milton Keynes	Wellcome Sanger Institute for the COVID-19 Genomics UK (COG-UK) consortium	The Lighthouse Lab in Milton Keynes and Alex Alderton, Roberto Amato, Sonia Goncalves, Ewan Harrison, David K. Jackson, Ian Johnston, Dominic Kwiatkowski, Cordelia Langford, John Sillitoe on behalf of the Wellcome Sanger Institute COVID-19 Surveillance Team
EPI_ISL_631033	Lighthouse Lab in Alderley Park	Wellcome Sanger Institute for the COVID-19 Genomics UK (COG-UK) consortium	Jacquelyn Wynn, Mairead Hyland, The Lighthouse Lab in Alderley Park and Alex Alderton, Roberto Amato, Sonia Goncalves, Ewan Harrison, David K. Jackson, Ian Johnston, Dominic Kwiatkowski, Cordelia Langford, John Sillitoe on behalf of the Wellcome Sanger Institute COVID-19 Surveillance Team
EPI_ISL_631034	Lighthouse Lab in Milton Keynes	Wellcome Sanger Institute for the COVID-19 Genomics UK (COG-UK) consortium	The Lighthouse Lab in Milton Keynes and Alex Alderton, Roberto Amato, Sonia Goncalves, Ewan Harrison, David K. Jackson, Ian Johnston, Dominic Kwiatkowski, Cordelia Langford, John Sillitoe on behalf of the Wellcome Sanger Institute COVID-19 Surveillance Team

EPI_ISL_631035	Lighthouse Lab in Alderley Park	Wellcome Sanger Institute for the COVID-19 Genomics UK (COG-UK) consortium	Jacquelyn Wynn, Mairead Hyland, The Lighthouse Lab in Alderley Park and Alex Alderton, Roberto Amato, Sonia Goncalves, Ewan Harrison, David K. Jackson, Ian Johnston, Dominic Kwiatkowski, Cordelia Langford, John Sillitoe on behalf of the Wellcome Sanger Institute COVID-19 Surveillance Team
EPI_ISL_631036, EPI_ISL_631037	Lighthouse Lab in Milton Keynes	Wellcome Sanger Institute for the COVID-19 Genomics UK (COG-UK) consortium	The Lighthouse Lab in Milton Keynes and Alex Alderton, Roberto Amato, Sonia Goncalves, Ewan Harrison, David K. Jackson, Ian Johnston, Dominic Kwiatkowski, Cordelia Langford, John Sillitoe on behalf of the Wellcome Sanger Institute COVID-19 Surveillance Team
EPI_ISL_631038, EPI_ISL_631039	Lighthouse Lab in Cambridge	Wellcome Sanger Institute for the COVID-19 Genomics UK (COG-UK) consortium	Rob Howes, The Lighthouse Lab in Cambridge and Alex Alderton, Roberto Amato, Sonia Goncalves, Ewan Harrison, David K. Jackson, Ian Johnston, Dominic Kwiatkowski, Cordelia Langford, John Sillitoe on behalf of the Wellcome Sanger Institute COVID-19 Surveillance Team
EPI_ISL_631040	Lighthouse Lab in Milton Keynes	Wellcome Sanger Institute for the COVID-19 Genomics UK (COG-UK) consortium	The Lighthouse Lab in Milton Keynes and Alex Alderton, Roberto Amato, Sonia Goncalves, Ewan Harrison, David K. Jackson, Ian Johnston, Dominic Kwiatkowski, Cordelia Langford, John Sillitoe on behalf of the Wellcome Sanger Institute COVID-19 Surveillance Team
EPI_ISL_631041	Lighthouse Lab in Alderley Park	Wellcome Sanger Institute for the COVID-19 Genomics UK (COG-UK) consortium	Jacquelyn Wynn, Mairead Hyland, The Lighthouse Lab in Alderley Park and Alex Alderton, Roberto Amato, Sonia Goncalves, Ewan Harrison, David K. Jackson, Ian Johnston, Dominic Kwiatkowski, Cordelia Langford, John Sillitoe on behalf of the Wellcome Sanger Institute COVID-19 Surveillance Team
EPI_ISL_631042, EPI_ISL_631043	Lighthouse Lab in Milton Keynes	Wellcome Sanger Institute for the COVID-19 Genomics UK (COG-UK) consortium	The Lighthouse Lab in Milton Keynes and Alex Alderton, Roberto Amato, Sonia Goncalves, Ewan Harrison, David K. Jackson, Ian Johnston, Dominic Kwiatkowski, Cordelia Langford, John Sillitoe on behalf of the Wellcome Sanger Institute COVID-19 Surveillance Team
EPI_ISL_631044	Lighthouse Lab in Alderley Park	Wellcome Sanger Institute for the COVID-19 Genomics UK (COG-UK) consortium	Jacquelyn Wynn, Mairead Hyland, The Lighthouse Lab in Alderley Park and Alex Alderton, Roberto Amato, Sonia Goncalves, Ewan Harrison, David K. Jackson, Ian Johnston, Dominic Kwiatkowski, Cordelia Langford, John Sillitoe on behalf of the Wellcome Sanger Institute COVID-19 Surveillance Team
EPI_ISL_631045	Lighthouse Lab in Cambridge	Wellcome Sanger Institute for the COVID-19 Genomics UK (COG-UK) consortium	Rob Howes, The Lighthouse Lab in Cambridge and Alex Alderton, Roberto Amato, Sonia Goncalves, Ewan Harrison, David K. Jackson, Ian Johnston, Dominic Kwiatkowski, Cordelia Langford, John Sillitoe on behalf of the Wellcome Sanger Institute COVID-19 Surveillance Team
EPI_ISL_631046	Lighthouse Lab in Alderley Park	Wellcome Sanger Institute for the COVID-19 Genomics UK (COG-UK) consortium	Jacquelyn Wynn, Mairead Hyland, The Lighthouse Lab in Alderley Park and Alex Alderton, Roberto Amato, Sonia Goncalves, Ewan Harrison, David K. Jackson, Ian Johnston, Dominic Kwiatkowski, Cordelia Langford, John Sillitoe on behalf of the Wellcome Sanger Institute COVID-19 Surveillance Team
EPI_ISL_631047, EPI_ISL_631048, EPI_ISL_631049	Lighthouse Lab in Milton Keynes	Wellcome Sanger Institute for the COVID-19 Genomics UK (COG-UK) consortium	The Lighthouse Lab in Milton Keynes and Alex Alderton, Roberto Amato, Sonia Goncalves, Ewan Harrison, David K. Jackson, Ian Johnston, Dominic Kwiatkowski, Cordelia Langford, John Sillitoe on behalf of the Wellcome Sanger Institute COVID-19 Surveillance Team
EPI_ISL_631050	Lighthouse Lab in Alderley Park	Wellcome Sanger Institute for the COVID-19 Genomics UK (COG-UK) consortium	Jacquelyn Wynn, Mairead Hyland, The Lighthouse Lab in Alderley Park and Alex Alderton, Roberto Amato, Sonia Goncalves, Ewan Harrison, David K. Jackson, Ian Johnston, Dominic Kwiatkowski, Cordelia Langford, John Sillitoe on behalf of the Wellcome Sanger Institute COVID-19 Surveillance Team
EPI_ISL_631051, EPI_ISL_631052	Lighthouse Lab in Milton Keynes	Wellcome Sanger Institute for the COVID-19 Genomics UK (COG-UK) consortium	The Lighthouse Lab in Milton Keynes and Alex Alderton, Roberto Amato, Sonia Goncalves, Ewan Harrison, David K. Jackson, Ian Johnston, Dominic Kwiatkowski, Cordelia Langford, John Sillitoe on behalf of the Wellcome Sanger Institute COVID-19 Surveillance Team
EPI_ISL_631053	Lighthouse Lab in Cambridge	Wellcome Sanger Institute for the COVID-19 Genomics UK (COG-UK) consortium	Rob Howes, The Lighthouse Lab in Cambridge and Alex Alderton, Roberto Amato, Sonia Goncalves, Ewan Harrison, David K. Jackson, Ian Johnston, Dominic Kwiatkowski, Cordelia Langford, John Sillitoe on behalf of the Wellcome Sanger Institute COVID-19 Surveillance Team
EPI_ISL_631054	Lighthouse Lab in Milton Keynes	Wellcome Sanger Institute for the COVID-19 Genomics UK (COG-UK) consortium	The Lighthouse Lab in Milton Keynes and Alex Alderton, Roberto Amato, Sonia Goncalves, Ewan Harrison, David K. Jackson, Ian Johnston, Dominic Kwiatkowski, Cordelia Langford, John Sillitoe on behalf of the Wellcome Sanger Institute COVID-19 Surveillance Team
EPI_ISL_631055	Lighthouse Lab in Cambridge	Wellcome Sanger Institute for the COVID-19 Genomics UK (COG-UK) consortium	Rob Howes, The Lighthouse Lab in Cambridge and Alex Alderton, Roberto Amato, Sonia Goncalves, Ewan Harrison, David K. Jackson, Ian Johnston, Dominic Kwiatkowski, Cordelia Langford, John Sillitoe on behalf of the Wellcome Sanger Institute COVID-19 Surveillance Team
EPI_ISL_631056, EPI_ISL_631057	Lighthouse Lab in Alderley Park	Wellcome Sanger Institute for the COVID-19 Genomics UK (COG-UK) consortium	Jacquelyn Wynn, Mairead Hyland, The Lighthouse Lab in Alderley Park and Alex Alderton, Roberto Amato, Sonia Goncalves, Ewan Harrison, David K. Jackson, Ian Johnston, Dominic Kwiatkowski, Cordelia Langford, John Sillitoe on behalf of the Wellcome Sanger Institute COVID-19 Surveillance Team
EPI_ISL_631058	Lighthouse Lab in Milton Keynes	Wellcome Sanger Institute for the COVID-19 Genomics UK (COG-UK) consortium	The Lighthouse Lab in Milton Keynes and Alex Alderton, Roberto Amato, Sonia Goncalves, Ewan Harrison, David K. Jackson, Ian Johnston, Dominic Kwiatkowski, Cordelia Langford, John Sillitoe on behalf of the Wellcome Sanger Institute COVID-19 Surveillance Team
EPI_ISL_631059, EPI_ISL_631060	Lighthouse Lab in Alderley Park	Wellcome Sanger Institute for the COVID-19 Genomics UK (COG-UK) consortium	Jacquelyn Wynn, Mairead Hyland, The Lighthouse Lab in Alderley Park and Alex Alderton, Roberto Amato, Sonia Goncalves, Ewan Harrison, David K. Jackson, Ian Johnston, Dominic Kwiatkowski, Cordelia Langford, John Sillitoe on behalf of the Wellcome Sanger Institute COVID-19 Surveillance Team
EPI_ISL_631061	Lighthouse Lab in Milton Keynes	Wellcome Sanger Institute for the COVID-19 Genomics UK (COG-UK) consortium	The Lighthouse Lab in Milton Keynes and Alex Alderton, Roberto Amato, Sonia Goncalves, Ewan Harrison, David K. Jackson, Ian Johnston, Dominic Kwiatkowski, Cordelia Langford, John Sillitoe on behalf of the Wellcome Sanger Institute COVID-19 Surveillance Team
EPI_ISL_631062, EPI_ISL_631063	Lighthouse Lab in Cambridge	Wellcome Sanger Institute for the COVID-19 Genomics UK (COG-UK) consortium	Rob Howes, The Lighthouse Lab in Cambridge and Alex Alderton, Roberto Amato, Sonia Goncalves, Ewan Harrison, David K. Jackson, Ian Johnston, Dominic Kwiatkowski, Cordelia Langford, John Sillitoe on behalf of the Wellcome Sanger Institute COVID-19 Surveillance Team
EPI_ISL_631064, EPI_ISL_631065, EPI_ISL_631066, EPI_ISL_631067, EPI_ISL_631068	Lighthouse Lab in Milton Keynes	Wellcome Sanger Institute for the COVID-19 Genomics UK (COG-UK) consortium	The Lighthouse Lab in Milton Keynes and Alex Alderton, Roberto Amato, Sonia Goncalves, Ewan Harrison, David K. Jackson, Ian Johnston, Dominic Kwiatkowski, Cordelia Langford, John Sillitoe on behalf of the Wellcome Sanger Institute COVID-19 Surveillance Team
EPI_ISL_631069	Lighthouse Lab in Cambridge	Wellcome Sanger Institute for the COVID-19 Genomics UK (COG-UK) consortium	Rob Howes, The Lighthouse Lab in Cambridge and Alex Alderton, Roberto Amato, Sonia Goncalves, Ewan Harrison, David K. Jackson, Ian Johnston, Dominic Kwiatkowski, Cordelia Langford, John Sillitoe on behalf of the Wellcome Sanger Institute COVID-19 Surveillance Team
EPI_ISL_631070, EPI_ISL_631071, EPI_ISL_631072, EPI_ISL_631073	Lighthouse Lab in Milton Keynes	Wellcome Sanger Institute for the COVID-19 Genomics UK (COG-UK) consortium	The Lighthouse Lab in Milton Keynes and Alex Alderton, Roberto Amato, Sonia Goncalves, Ewan Harrison, David K. Jackson, Ian Johnston, Dominic Kwiatkowski, Cordelia Langford, John Sillitoe on behalf of the Wellcome Sanger Institute COVID-19 Surveillance Team
EPI_ISL_631074	Lighthouse Lab in Alderley Park	Wellcome Sanger Institute for the COVID-19 Genomics UK (COG-UK) consortium	Jacquelyn Wynn, Mairead Hyland, The Lighthouse Lab in Alderley Park and Alex Alderton, Roberto Amato, Sonia Goncalves, Ewan Harrison, David K. Jackson, Ian Johnston, Dominic Kwiatkowski, Cordelia Langford, John Sillitoe on behalf of the Wellcome Sanger Institute COVID-19 Surveillance Team
EPI_ISL_631075, EPI_ISL_631076, EPI_ISL_631077	Lighthouse Lab in Milton Keynes	Wellcome Sanger Institute for the COVID-19 Genomics UK (COG-UK) consortium	The Lighthouse Lab in Milton Keynes and Alex Alderton, Roberto Amato, Sonia Goncalves, Ewan Harrison, David K. Jackson, Ian Johnston, Dominic Kwiatkowski, Cordelia Langford, John Sillitoe on behalf of the Wellcome Sanger Institute COVID-19 Surveillance Team
EPI_ISL_631078	Lighthouse Lab in Cambridge	Wellcome Sanger Institute for the COVID-19 Genomics UK (COG-UK) consortium	Rob Howes, The Lighthouse Lab in Cambridge and Alex Alderton, Roberto Amato, Sonia Goncalves, Ewan Harrison, David K. Jackson, Ian Johnston, Dominic Kwiatkowski, Cordelia Langford, John Sillitoe on behalf of the Wellcome Sanger Institute COVID-19 Surveillance Team
EPI_ISL_631079	Lighthouse Lab in Milton Keynes	Wellcome Sanger Institute for the COVID-19 Genomics UK (COG-UK) consortium	The Lighthouse Lab in Milton Keynes and Alex Alderton, Roberto Amato, Sonia Goncalves, Ewan Harrison, David K. Jackson, Ian Johnston, Dominic Kwiatkowski, Cordelia Langford, John Sillitoe on behalf of the Wellcome Sanger Institute COVID-19 Surveillance Team
EPI_ISL_631080	Lighthouse Lab in Cambridge	Wellcome Sanger Institute for the COVID-19 Genomics UK (COG-UK) consortium	Rob Howes, The Lighthouse Lab in Cambridge and Alex Alderton, Roberto Amato, Sonia Goncalves, Ewan Harrison, David K. Jackson, Ian Johnston, Dominic Kwiatkowski, Cordelia Langford, John Sillitoe on behalf of the Wellcome Sanger Institute COVID-19 Surveillance Team
EPI_ISL_631081	Lighthouse Lab in Alderley Park	Wellcome Sanger Institute for the COVID-19 Genomics UK (COG-UK) consortium	Jacquelyn Wynn, Mairead Hyland, The Lighthouse Lab in Alderley Park and Alex Alderton, Roberto Amato, Sonia Goncalves, Ewan Harrison, David K. Jackson, Ian Johnston, Dominic Kwiatkowski, Cordelia Langford, John Sillitoe on behalf of the Wellcome Sanger Institute COVID-19 Surveillance Team
EPI_ISL_631082, EPI_ISL_631083	Lighthouse Lab in Milton Keynes	Wellcome Sanger Institute for the COVID-19 Genomics UK (COG-UK) consortium	The Lighthouse Lab in Milton Keynes and Alex Alderton, Roberto Amato, Sonia Goncalves, Ewan Harrison, David K. Jackson, Ian Johnston, Dominic Kwiatkowski, Cordelia Langford, John Sillitoe on behalf of the Wellcome Sanger Institute COVID-19 Surveillance Team
EPI_ISL_631084	Lighthouse Lab in Alderley Park	Wellcome Sanger Institute for the COVID-19 Genomics UK (COG-UK) consortium	Jacquelyn Wynn, Mairead Hyland, The Lighthouse Lab in Alderley Park and Alex Alderton, Roberto Amato, Sonia Goncalves, Ewan Harrison, David K. Jackson, Ian Johnston, Dominic Kwiatkowski, Cordelia Langford, John Sillitoe on behalf of the Wellcome Sanger Institute COVID-19 Surveillance Team
EPI_ISL_631085, EPI_ISL_631086, EPI_ISL_631087, EPI_ISL_631088	Lighthouse Lab in Milton Keynes	Wellcome Sanger Institute for the COVID-19 Genomics UK (COG-UK) consortium	The Lighthouse Lab in Milton Keynes and Alex Alderton, Roberto Amato, Sonia Goncalves, Ewan Harrison, David K. Jackson, Ian Johnston, Dominic Kwiatkowski, Cordelia Langford, John Sillitoe on behalf of the Wellcome Sanger Institute COVID-19 Surveillance Team
EPI_ISL_631089	Lighthouse Lab in Alderley Park	Wellcome Sanger Institute for the COVID-19 Genomics UK (COG-UK) consortium	Jacquelyn Wynn, Mairead Hyland, The Lighthouse Lab in Alderley Park and Alex Alderton, Roberto Amato, Sonia Goncalves, Ewan Harrison, David K. Jackson, Ian Johnston, Dominic Kwiatkowski, Cordelia Langford, John Sillitoe on behalf of the Wellcome Sanger Institute COVID-19 Surveillance Team
EPI_ISL_631090	Lighthouse Lab in Milton Keynes	Wellcome Sanger Institute for the COVID-19 Genomics UK (COG-UK) Consortium	The Lighthouse Lab in Milton Keynes and Alex Alderton, Roberto Amato, Sonia Goncalves, Ewan Harrison, David K. Jackson, Ian Johnston, Dominic Kwiatkowski, Cordelia Langford, John Sillitoe on behalf of the Wellcome Sanger Institute COVID-19 Surveillance Team
EPI_ISL_631091, EPI_ISL_631092	Lighthouse Lab in Milton Keynes	Wellcome Sanger Institute for the COVID-19 Genomics UK (COG-UK) consortium	The Lighthouse Lab in Milton Keynes and Alex Alderton, Roberto Amato, Sonia Goncalves, Ewan Harrison, David K. Jackson, Ian Johnston, Dominic Kwiatkowski, Cordelia Langford, John Sillitoe on behalf of the Wellcome Sanger Institute COVID-19 Surveillance Team

EPI_ISL_631093	Lighthouse Lab in Alderley Park	Wellcome Sanger Institute for the COVID-19 Genomics UK (COG-UK) consortium	Jacquelyn Wynn, Mairead Hyland, The Lighthouse Lab in Alderley Park and Alex Alderton, Roberto Amato, Sonia Goncalves, Ewan Harrison, David K. Jackson, Ian Johnston, Dominic Kwiatkowski, Cordelia Langford, John Sillitoe on behalf of the Wellcome Sanger Institute COVID-19 Surveillance Team
EPI_ISL_631094	Lighthouse Lab in Cambridge	Wellcome Sanger Institute for the COVID-19 Genomics UK (COG-UK) consortium	Rob Howes, The Lighthouse Lab in Cambridge and Alex Alderton, Roberto Amato, Sonia Goncalves, Ewan Harrison, David K. Jackson, Ian Johnston, Dominic Kwiatkowski, Cordelia Langford, John Sillitoe on behalf of the Wellcome Sanger Institute COVID-19 Surveillance Team
EPI_ISL_631095	Lighthouse Lab in Milton Keynes	Wellcome Sanger Institute for the COVID-19 Genomics UK (COG-UK) consortium	The Lighthouse Lab in Milton Keynes and Alex Alderton, Roberto Amato, Sonia Goncalves, Ewan Harrison, David K. Jackson, Ian Johnston, Dominic Kwiatkowski, Cordelia Langford, John Sillitoe on behalf of the Wellcome Sanger Institute COVID-19 Surveillance Team
EPI_ISL_631096	Lighthouse Lab in Alderley Park	Wellcome Sanger Institute for the COVID-19 Genomics UK (COG-UK) consortium	Jacquelyn Wynn, Mairead Hyland, The Lighthouse Lab in Alderley Park and Alex Alderton, Roberto Amato, Sonia Goncalves, Ewan Harrison, David K. Jackson, Ian Johnston, Dominic Kwiatkowski, Cordelia Langford, John Sillitoe on behalf of the Wellcome Sanger Institute COVID-19 Surveillance Team
EPI_ISL_631097, EPI_ISL_631098	Lighthouse Lab in Milton Keynes	Wellcome Sanger Institute for the COVID-19 Genomics UK (COG-UK) consortium	The Lighthouse Lab in Milton Keynes and Alex Alderton, Roberto Amato, Sonia Goncalves, Ewan Harrison, David K. Jackson, Ian Johnston, Dominic Kwiatkowski, Cordelia Langford, John Sillitoe on behalf of the Wellcome Sanger Institute COVID-19 Surveillance Team
EPI_ISL_631099	Lighthouse Lab in Alderley Park	Wellcome Sanger Institute for the COVID-19 Genomics UK (COG-UK) consortium	Jacquelyn Wynn, Mairead Hyland, The Lighthouse Lab in Alderley Park and Alex Alderton, Roberto Amato, Sonia Goncalves, Ewan Harrison, David K. Jackson, Ian Johnston, Dominic Kwiatkowski, Cordelia Langford, John Sillitoe on behalf of the Wellcome Sanger Institute COVID-19 Surveillance Team
EPI_ISL_631100	Lighthouse Lab in Milton Keynes	Wellcome Sanger Institute for the COVID-19 Genomics UK (COG-UK) consortium	The Lighthouse Lab in Milton Keynes and Alex Alderton, Roberto Amato, Sonia Goncalves, Ewan Harrison, David K. Jackson, Ian Johnston, Dominic Kwiatkowski, Cordelia Langford, John Sillitoe on behalf of the Wellcome Sanger Institute COVID-19 Surveillance Team
EPI_ISL_631101	Lighthouse Lab in Cambridge	Wellcome Sanger Institute for the COVID-19 Genomics UK (COG-UK) consortium	Rob Howes, The Lighthouse Lab in Cambridge and Alex Alderton, Roberto Amato, Sonia Goncalves, Ewan Harrison, David K. Jackson, Ian Johnston, Dominic Kwiatkowski, Cordelia Langford, John Sillitoe on behalf of the Wellcome Sanger Institute COVID-19 Surveillance Team
EPI_ISL_631103, EPI_ISL_631104	Lighthouse Lab in Milton Keynes	Wellcome Sanger Institute for the COVID-19 Genomics UK (COG-UK) consortium	The Lighthouse Lab in Milton Keynes and Alex Alderton, Roberto Amato, Sonia Goncalves, Ewan Harrison, David K. Jackson, Ian Johnston, Dominic Kwiatkowski, Cordelia Langford, John Sillitoe on behalf of the Wellcome Sanger Institute COVID-19 Surveillance Team
EPI_ISL_631105	Lighthouse Lab in Cambridge	Wellcome Sanger Institute for the COVID-19 Genomics UK (COG-UK) consortium	Rob Howes, The Lighthouse Lab in Cambridge and Alex Alderton, Roberto Amato, Sonia Goncalves, Ewan Harrison, David K. Jackson, Ian Johnston, Dominic Kwiatkowski, Cordelia Langford, John Sillitoe on behalf of the Wellcome Sanger Institute COVID-19 Surveillance Team
EPI_ISL_631106	Lighthouse Lab in Milton Keynes	Wellcome Sanger Institute for the COVID-19 Genomics UK (COG-UK) consortium	The Lighthouse Lab in Milton Keynes and Alex Alderton, Roberto Amato, Sonia Goncalves, Ewan Harrison, David K. Jackson, Ian Johnston, Dominic Kwiatkowski, Cordelia Langford, John Sillitoe on behalf of the Wellcome Sanger Institute COVID-19 Surveillance Team
EPI_ISL_631107	Lighthouse Lab in Alderley Park	Wellcome Sanger Institute for the COVID-19 Genomics UK (COG-UK) consortium	Jacquelyn Wynn, Mairead Hyland, The Lighthouse Lab in Alderley Park and Alex Alderton, Roberto Amato, Sonia Goncalves, Ewan Harrison, David K. Jackson, Ian Johnston, Dominic Kwiatkowski, Cordelia Langford, John Sillitoe on behalf of the Wellcome Sanger Institute COVID-19 Surveillance Team
EPI_ISL_631108	Lighthouse Lab in Milton Keynes	Wellcome Sanger Institute for the COVID-19 Genomics UK (COG-UK) consortium	The Lighthouse Lab in Milton Keynes and Alex Alderton, Roberto Amato, Sonia Goncalves, Ewan Harrison, David K. Jackson, Ian Johnston, Dominic Kwiatkowski, Cordelia Langford, John Sillitoe on behalf of the Wellcome Sanger Institute COVID-19 Surveillance Team
EPI_ISL_631109	Lighthouse Lab in Cambridge	Wellcome Sanger Institute for the COVID-19 Genomics UK (COG-UK) consortium	Rob Howes, The Lighthouse Lab in Cambridge and Alex Alderton, Roberto Amato, Sonia Goncalves, Ewan Harrison, David K. Jackson, Ian Johnston, Dominic Kwiatkowski, Cordelia Langford, John Sillitoe on behalf of the Wellcome Sanger Institute COVID-19 Surveillance Team
EPI_ISL_631110	Lighthouse Lab in Milton Keynes	Wellcome Sanger Institute for the COVID-19 Genomics UK (COG-UK) consortium	The Lighthouse Lab in Milton Keynes and Alex Alderton, Roberto Amato, Sonia Goncalves, Ewan Harrison, David K. Jackson, Ian Johnston, Dominic Kwiatkowski, Cordelia Langford, John Sillitoe on behalf of the Wellcome Sanger Institute COVID-19 Surveillance Team
EPI_ISL_631111	Lighthouse Lab in Alderley Park	Wellcome Sanger Institute for the COVID-19 Genomics UK (COG-UK) consortium	Jacquelyn Wynn, Mairead Hyland, The Lighthouse Lab in Alderley Park and Alex Alderton, Roberto Amato, Sonia Goncalves, Ewan Harrison, David K. Jackson, Ian Johnston, Dominic Kwiatkowski, Cordelia Langford, John Sillitoe on behalf of the Wellcome Sanger Institute COVID-19 Surveillance Team
EPI_ISL_631112	Lighthouse Lab in Milton Keynes	Wellcome Sanger Institute for the COVID-19 Genomics UK (COG-UK) consortium	The Lighthouse Lab in Milton Keynes and Alex Alderton, Roberto Amato, Sonia Goncalves, Ewan Harrison, David K. Jackson, Ian Johnston, Dominic Kwiatkowski, Cordelia Langford, John Sillitoe on behalf of the Wellcome Sanger Institute COVID-19 Surveillance Team
EPI_ISL_631113	Lighthouse Lab in Alderley Park	Wellcome Sanger Institute for the COVID-19 Genomics UK (COG-UK) consortium	Jacquelyn Wynn, Mairead Hyland, The Lighthouse Lab in Alderley Park and Alex Alderton, Roberto Amato, Sonia Goncalves, Ewan Harrison, David K. Jackson, Ian Johnston, Dominic Kwiatkowski, Cordelia Langford, John Sillitoe on behalf of the Wellcome Sanger Institute COVID-19 Surveillance Team
EPI_ISL_631114, EPI_ISL_631115	Lighthouse Lab in Milton Keynes	Wellcome Sanger Institute for the COVID-19 Genomics UK (COG-UK) consortium	The Lighthouse Lab in Milton Keynes and Alex Alderton, Roberto Amato, Sonia Goncalves, Ewan Harrison, David K. Jackson, Ian Johnston, Dominic Kwiatkowski, Cordelia Langford, John Sillitoe on behalf of the Wellcome Sanger Institute COVID-19 Surveillance Team
EPI_ISL_631116	Lighthouse Lab in Alderley Park	Wellcome Sanger Institute for the COVID-19 Genomics UK (COG-UK) consortium	Jacquelyn Wynn, Mairead Hyland, The Lighthouse Lab in Alderley Park and Alex Alderton, Roberto Amato, Sonia Goncalves, Ewan Harrison, David K. Jackson, Ian Johnston, Dominic Kwiatkowski, Cordelia Langford, John Sillitoe on behalf of the Wellcome Sanger Institute COVID-19 Surveillance Team
EPI_ISL_631117, EPI_ISL_631118	Lighthouse Lab in Cambridge	Wellcome Sanger Institute for the COVID-19 Genomics UK (COG-UK) consortium	Rob Howes, The Lighthouse Lab in Cambridge and Alex Alderton, Roberto Amato, Sonia Goncalves, Ewan Harrison, David K. Jackson, Ian Johnston, Dominic Kwiatkowski, Cordelia Langford, John Sillitoe on behalf of the Wellcome Sanger Institute COVID-19 Surveillance Team
EPI_ISL_631119, EPI_ISL_631120, EPI_ISL_631121, EPI_ISL_631122	Lighthouse Lab in Milton Keynes	Wellcome Sanger Institute for the COVID-19 Genomics UK (COG-UK) consortium	The Lighthouse Lab in Milton Keynes and Alex Alderton, Roberto Amato, Sonia Goncalves, Ewan Harrison, David K. Jackson, Ian Johnston, Dominic Kwiatkowski, Cordelia Langford, John Sillitoe on behalf of the Wellcome Sanger Institute COVID-19 Surveillance Team
EPI_ISL_631123	Lighthouse Lab in Cambridge	Wellcome Sanger Institute for the COVID-19 Genomics UK (COG-UK) consortium	Rob Howes, The Lighthouse Lab in Cambridge and Alex Alderton, Roberto Amato, Sonia Goncalves, Ewan Harrison, David K. Jackson, Ian Johnston, Dominic Kwiatkowski, Cordelia Langford, John Sillitoe on behalf of the Wellcome Sanger Institute COVID-19 Surveillance Team
EPI_ISL_631124, EPI_ISL_631125, EPI_ISL_631126	Lighthouse Lab in Milton Keynes	Wellcome Sanger Institute for the COVID-19 Genomics UK (COG-UK) consortium	The Lighthouse Lab in Milton Keynes and Alex Alderton, Roberto Amato, Sonia Goncalves, Ewan Harrison, David K. Jackson, Ian Johnston, Dominic Kwiatkowski, Cordelia Langford, John Sillitoe on behalf of the Wellcome Sanger Institute COVID-19 Surveillance Team
EPI_ISL_631127, EPI_ISL_631128	Lighthouse Lab in Alderley Park	Wellcome Sanger Institute for the COVID-19 Genomics UK (COG-UK) consortium	Jacquelyn Wynn, Mairead Hyland, The Lighthouse Lab in Alderley Park and Alex Alderton, Roberto Amato, Sonia Goncalves, Ewan Harrison, David K. Jackson, Ian Johnston, Dominic Kwiatkowski, Cordelia Langford, John Sillitoe on behalf of the Wellcome Sanger Institute COVID-19 Surveillance Team
EPI_ISL_631129	Lighthouse Lab in Cambridge	Wellcome Sanger Institute for the COVID-19 Genomics UK (COG-UK) consortium	Rob Howes, The Lighthouse Lab in Cambridge and Alex Alderton, Roberto Amato, Sonia Goncalves, Ewan Harrison, David K. Jackson, Ian Johnston, Dominic Kwiatkowski, Cordelia Langford, John Sillitoe on behalf of the Wellcome Sanger Institute COVID-19 Surveillance Team
EPI_ISL_631130	Lighthouse Lab in Milton Keynes	Wellcome Sanger Institute for the COVID-19 Genomics UK (COG-UK) consortium	The Lighthouse Lab in Milton Keynes and Alex Alderton, Roberto Amato, Sonia Goncalves, Ewan Harrison, David K. Jackson, Ian Johnston, Dominic Kwiatkowski, Cordelia Langford, John Sillitoe on behalf of the Wellcome Sanger Institute COVID-19 Surveillance Team
EPI_ISL_631131	Lighthouse Lab in Alderley Park	Wellcome Sanger Institute for the COVID-19 Genomics UK (COG-UK) consortium	Jacquelyn Wynn, Mairead Hyland, The Lighthouse Lab in Alderley Park and Alex Alderton, Roberto Amato, Sonia Goncalves, Ewan Harrison, David K. Jackson, Ian Johnston, Dominic Kwiatkowski, Cordelia Langford, John Sillitoe on behalf of the Wellcome Sanger Institute COVID-19 Surveillance Team
EPI_ISL_631132	Lighthouse Lab in Cambridge	Wellcome Sanger Institute for the COVID-19 Genomics UK (COG-UK) consortium	Rob Howes, The Lighthouse Lab in Cambridge and Alex Alderton, Roberto Amato, Sonia Goncalves, Ewan Harrison, David K. Jackson, Ian Johnston, Dominic Kwiatkowski, Cordelia Langford, John Sillitoe on behalf of the Wellcome Sanger Institute COVID-19 Surveillance Team
EPI_ISL_631133	Lighthouse Lab in Alderley Park	Wellcome Sanger Institute for the COVID-19 Genomics UK (COG-UK) consortium	Jacquelyn Wynn, Mairead Hyland, The Lighthouse Lab in Alderley Park and Alex Alderton, Roberto Amato, Sonia Goncalves, Ewan Harrison, David K. Jackson, Ian Johnston, Dominic Kwiatkowski, Cordelia Langford, John Sillitoe on behalf of the Wellcome Sanger Institute COVID-19 Surveillance Team
EPI_ISL_631134	Lighthouse Lab in Milton Keynes	Wellcome Sanger Institute for the COVID-19 Genomics UK (COG-UK) consortium	The Lighthouse Lab in Milton Keynes and Alex Alderton, Roberto Amato, Sonia Goncalves, Ewan Harrison, David K. Jackson, Ian Johnston, Dominic Kwiatkowski, Cordelia Langford, John Sillitoe on behalf of the Wellcome Sanger Institute COVID-19 Surveillance Team
EPI_ISL_631135	Lighthouse Lab in Cambridge	Wellcome Sanger Institute for the COVID-19 Genomics UK (COG-UK) consortium	Rob Howes, The Lighthouse Lab in Cambridge and Alex Alderton, Roberto Amato, Sonia Goncalves, Ewan Harrison, David K. Jackson, Ian Johnston, Dominic Kwiatkowski, Cordelia Langford, John Sillitoe on behalf of the Wellcome Sanger Institute COVID-19 Surveillance Team
EPI_ISL_631136	Lighthouse Lab in Milton Keynes	Wellcome Sanger Institute for the COVID-19 Genomics UK (COG-UK) consortium	The Lighthouse Lab in Milton Keynes and Alex Alderton, Roberto Amato, Sonia Goncalves, Ewan Harrison, David K. Jackson, Ian Johnston, Dominic Kwiatkowski, Cordelia Langford, John Sillitoe on behalf of the Wellcome Sanger Institute COVID-19 Surveillance Team
EPI_ISL_631137	Lighthouse Lab in Cambridge	Wellcome Sanger Institute for the COVID-19 Genomics UK (COG-UK) consortium	Rob Howes, The Lighthouse Lab in Cambridge and Alex Alderton, Roberto Amato, Sonia Goncalves, Ewan Harrison, David K. Jackson, Ian Johnston, Dominic Kwiatkowski, Cordelia Langford, John Sillitoe on behalf of the Wellcome Sanger Institute COVID-19 Surveillance Team
EPI_ISL_631138, EPI_ISL_631139, EPI_ISL_631140, EPI_ISL_631141	Lighthouse Lab in Milton Keynes	Wellcome Sanger Institute for the COVID-19 Genomics UK (COG-UK) consortium	The Lighthouse Lab in Milton Keynes and Alex Alderton, Roberto Amato, Sonia Goncalves, Ewan Harrison, David K. Jackson, Ian Johnston, Dominic Kwiatkowski, Cordelia Langford, John Sillitoe on behalf of the Wellcome Sanger Institute COVID-19 Surveillance Team

EPI_ISL_631142	Lighthouse Lab in Cambridge	Wellcome Sanger Institute for the COVID-19 Genomics UK (COG-UK) consortium	Rob Howes, The Lighthouse Lab in Cambridge and Alex Alderton, Roberto Amato, Sonia Goncalves, Ewan Harrison, David K. Jackson, Ian Johnston, Dominic Kwiatkowski, Cordelia Langford, John Sillitoe on behalf of the Wellcome Sanger Institute COVID-19 Surveillance Team
EPI_ISL_631143	Lighthouse Lab in Milton Keynes	Wellcome Sanger Institute for the COVID-19 Genomics UK (COG-UK) consortium	The Lighthouse Lab in Milton Keynes and Alex Alderton, Roberto Amato, Sonia Goncalves, Ewan Harrison, David K. Jackson, Ian Johnston, Dominic Kwiatkowski, Cordelia Langford, John Sillitoe on behalf of the Wellcome Sanger Institute COVID-19 Surveillance Team
EPI_ISL_631144	Lighthouse Lab in Cambridge	Wellcome Sanger Institute for the COVID-19 Genomics UK (COG-UK) consortium	Rob Howes, The Lighthouse Lab in Cambridge and Alex Alderton, Roberto Amato, Sonia Goncalves, Ewan Harrison, David K. Jackson, Ian Johnston, Dominic Kwiatkowski, Cordelia Langford, John Sillitoe on behalf of the Wellcome Sanger Institute COVID-19 Surveillance Team
EPI_ISL_631145, EPI_ISL_631146, EPI_ISL_631147	Lighthouse Lab in Milton Keynes	Wellcome Sanger Institute for the COVID-19 Genomics UK (COG-UK) consortium	The Lighthouse Lab in Milton Keynes and Alex Alderton, Roberto Amato, Sonia Goncalves, Ewan Harrison, David K. Jackson, Ian Johnston, Dominic Kwiatkowski, Cordelia Langford, John Sillitoe on behalf of the Wellcome Sanger Institute COVID-19 Surveillance Team
EPI_ISL_631148, EPI_ISL_631149	Lighthouse Lab in Cambridge	Wellcome Sanger Institute for the COVID-19 Genomics UK (COG-UK) consortium	Rob Howes, The Lighthouse Lab in Cambridge and Alex Alderton, Roberto Amato, Sonia Goncalves, Ewan Harrison, David K. Jackson, Ian Johnston, Dominic Kwiatkowski, Cordelia Langford, John Sillitoe on behalf of the Wellcome Sanger Institute COVID-19 Surveillance Team
EPI_ISL_631150	Lighthouse Lab in Alderley Park	Wellcome Sanger Institute for the COVID-19 Genomics UK (COG-UK) consortium	Jacquelyn Wynn, Mairead Hyland, The Lighthouse Lab in Alderley Park and Alex Alderton, Roberto Amato, Sonia Goncalves, Ewan Harrison, David K. Jackson, Ian Johnston, Dominic Kwiatkowski, Cordelia Langford, John Sillitoe on behalf of the Wellcome Sanger Institute COVID-19 Surveillance Team
EPI_ISL_631151, EPI_ISL_631152, EPI_ISL_631153	Lighthouse Lab in Milton Keynes	Wellcome Sanger Institute for the COVID-19 Genomics UK (COG-UK) consortium	The Lighthouse Lab in Milton Keynes and Alex Alderton, Roberto Amato, Sonia Goncalves, Ewan Harrison, David K. Jackson, Ian Johnston, Dominic Kwiatkowski, Cordelia Langford, John Sillitoe on behalf of the Wellcome Sanger Institute COVID-19 Surveillance Team
EPI_ISL_631154, EPI_ISL_631155	Lighthouse Lab in Cambridge	Wellcome Sanger Institute for the COVID-19 Genomics UK (COG-UK) consortium	Rob Howes, The Lighthouse Lab in Cambridge and Alex Alderton, Roberto Amato, Sonia Goncalves, Ewan Harrison, David K. Jackson, Ian Johnston, Dominic Kwiatkowski, Cordelia Langford, John Sillitoe on behalf of the Wellcome Sanger Institute COVID-19 Surveillance Team
EPI_ISL_631156, EPI_ISL_631157, EPI_ISL_631158	Lighthouse Lab in Milton Keynes	Wellcome Sanger Institute for the COVID-19 Genomics UK (COG-UK) consortium	The Lighthouse Lab in Milton Keynes and Alex Alderton, Roberto Amato, Sonia Goncalves, Ewan Harrison, David K. Jackson, Ian Johnston, Dominic Kwiatkowski, Cordelia Langford, John Sillitoe on behalf of the Wellcome Sanger Institute COVID-19 Surveillance Team
EPI_ISL_631160	Lighthouse Lab in Cambridge	Wellcome Sanger Institute for the COVID-19 Genomics UK (COG-UK) consortium	Rob Howes, The Lighthouse Lab in Cambridge and Alex Alderton, Roberto Amato, Sonia Goncalves, Ewan Harrison, David K. Jackson, Ian Johnston, Dominic Kwiatkowski, Cordelia Langford, John Sillitoe on behalf of the Wellcome Sanger Institute COVID-19 Surveillance Team
EPI_ISL_631161, EPI_ISL_631162	Lighthouse Lab in Milton Keynes	Wellcome Sanger Institute for the COVID-19 Genomics UK (COG-UK) consortium	The Lighthouse Lab in Milton Keynes and Alex Alderton, Roberto Amato, Sonia Goncalves, Ewan Harrison, David K. Jackson, Ian Johnston, Dominic Kwiatkowski, Cordelia Langford, John Sillitoe on behalf of the Wellcome Sanger Institute COVID-19 Surveillance Team
EPI_ISL_631163	Lighthouse Lab in Alderley Park	Wellcome Sanger Institute for the COVID-19 Genomics UK (COG-UK) consortium	Jacquelyn Wynn, Mairead Hyland, The Lighthouse Lab in Alderley Park and Alex Alderton, Roberto Amato, Sonia Goncalves, Ewan Harrison, David K. Jackson, Ian Johnston, Dominic Kwiatkowski, Cordelia Langford, John Sillitoe on behalf of the Wellcome Sanger Institute COVID-19 Surveillance Team
EPI_ISL_631164	Lighthouse Lab in Cambridge	Wellcome Sanger Institute for the COVID-19 Genomics UK (COG-UK) consortium	Rob Howes, The Lighthouse Lab in Cambridge and Alex Alderton, Roberto Amato, Sonia Goncalves, Ewan Harrison, David K. Jackson, Ian Johnston, Dominic Kwiatkowski, Cordelia Langford, John Sillitoe on behalf of the Wellcome Sanger Institute COVID-19 Surveillance Team
EPI_ISL_631165	Lighthouse Lab in Alderley Park	Wellcome Sanger Institute for the COVID-19 Genomics UK (COG-UK) consortium	Jacquelyn Wynn, Mairead Hyland, The Lighthouse Lab in Alderley Park and Alex Alderton, Roberto Amato, Sonia Goncalves, Ewan Harrison, David K. Jackson, Ian Johnston, Dominic Kwiatkowski, Cordelia Langford, John Sillitoe on behalf of the Wellcome Sanger Institute COVID-19 Surveillance Team
EPI_ISL_631166, EPI_ISL_631167, EPI_ISL_631168, EPI_ISL_631169, EPI_ISL_631170	Lighthouse Lab in Milton Keynes	Wellcome Sanger Institute for the COVID-19 Genomics UK (COG-UK) consortium	The Lighthouse Lab in Milton Keynes and Alex Alderton, Roberto Amato, Sonia Goncalves, Ewan Harrison, David K. Jackson, Ian Johnston, Dominic Kwiatkowski, Cordelia Langford, John Sillitoe on behalf of the Wellcome Sanger Institute COVID-19 Surveillance Team
EPI_ISL_631171	Lighthouse Lab in Cambridge	Wellcome Sanger Institute for the COVID-19 Genomics UK (COG-UK) consortium	Rob Howes, The Lighthouse Lab in Cambridge and Alex Alderton, Roberto Amato, Sonia Goncalves, Ewan Harrison, David K. Jackson, Ian Johnston, Dominic Kwiatkowski, Cordelia Langford, John Sillitoe on behalf of the Wellcome Sanger Institute COVID-19 Surveillance Team
EPI_ISL_631172	Lighthouse Lab in Milton Keynes	Wellcome Sanger Institute for the COVID-19 Genomics UK (COG-UK) consortium	The Lighthouse Lab in Milton Keynes and Alex Alderton, Roberto Amato, Sonia Goncalves, Ewan Harrison, David K. Jackson, Ian Johnston, Dominic Kwiatkowski, Cordelia Langford, John Sillitoe on behalf of the Wellcome Sanger Institute COVID-19 Surveillance Team
EPI_ISL_631173	Lighthouse Lab in Alderley Park	Wellcome Sanger Institute for the COVID-19 Genomics UK (COG-UK) consortium	Jacquelyn Wynn, Mairead Hyland, The Lighthouse Lab in Alderley Park and Alex Alderton, Roberto Amato, Sonia Goncalves, Ewan Harrison, David K. Jackson, Ian Johnston, Dominic Kwiatkowski, Cordelia Langford, John Sillitoe on behalf of the Wellcome Sanger Institute COVID-19 Surveillance Team
EPI_ISL_631174	Lighthouse Lab in Cambridge	Wellcome Sanger Institute for the COVID-19 Genomics UK (COG-UK) consortium	Rob Howes, The Lighthouse Lab in Cambridge and Alex Alderton, Roberto Amato, Sonia Goncalves, Ewan Harrison, David K. Jackson, Ian Johnston, Dominic Kwiatkowski, Cordelia Langford, John Sillitoe on behalf of the Wellcome Sanger Institute COVID-19 Surveillance Team
EPI_ISL_631175	Lighthouse Lab in Alderley Park	Wellcome Sanger Institute for the COVID-19 Genomics UK (COG-UK) consortium	Jacquelyn Wynn, Mairead Hyland, The Lighthouse Lab in Alderley Park and Alex Alderton, Roberto Amato, Sonia Goncalves, Ewan Harrison, David K. Jackson, Ian Johnston, Dominic Kwiatkowski, Cordelia Langford, John Sillitoe on behalf of the Wellcome Sanger Institute COVID-19 Surveillance Team
EPI_ISL_631176	Lighthouse Lab in Milton Keynes	Wellcome Sanger Institute for the COVID-19 Genomics UK (COG-UK) consortium	The Lighthouse Lab in Milton Keynes and Alex Alderton, Roberto Amato, Sonia Goncalves, Ewan Harrison, David K. Jackson, Ian Johnston, Dominic Kwiatkowski, Cordelia Langford, John Sillitoe on behalf of the Wellcome Sanger Institute COVID-19 Surveillance Team
EPI_ISL_631177	Lighthouse Lab in Cambridge	Wellcome Sanger Institute for the COVID-19 Genomics UK (COG-UK) consortium	Rob Howes, The Lighthouse Lab in Cambridge and Alex Alderton, Roberto Amato, Sonia Goncalves, Ewan Harrison, David K. Jackson, Ian Johnston, Dominic Kwiatkowski, Cordelia Langford, John Sillitoe on behalf of the Wellcome Sanger Institute COVID-19 Surveillance Team
EPI_ISL_631178, EPI_ISL_631179	Lighthouse Lab in Milton Keynes	Wellcome Sanger Institute for the COVID-19 Genomics UK (COG-UK) consortium	The Lighthouse Lab in Milton Keynes and Alex Alderton, Roberto Amato, Sonia Goncalves, Ewan Harrison, David K. Jackson, Ian Johnston, Dominic Kwiatkowski, Cordelia Langford, John Sillitoe on behalf of the Wellcome Sanger Institute COVID-19 Surveillance Team
EPI_ISL_631180, EPI_ISL_631181	Lighthouse Lab in Cambridge	Wellcome Sanger Institute for the COVID-19 Genomics UK (COG-UK) consortium	Rob Howes, The Lighthouse Lab in Cambridge and Alex Alderton, Roberto Amato, Sonia Goncalves, Ewan Harrison, David K. Jackson, Ian Johnston, Dominic Kwiatkowski, Cordelia Langford, John Sillitoe on behalf of the Wellcome Sanger Institute COVID-19 Surveillance Team
EPI_ISL_631182, EPI_ISL_631183	Lighthouse Lab in Milton Keynes	Wellcome Sanger Institute for the COVID-19 Genomics UK (COG-UK) consortium	The Lighthouse Lab in Milton Keynes and Alex Alderton, Roberto Amato, Sonia Goncalves, Ewan Harrison, David K. Jackson, Ian Johnston, Dominic Kwiatkowski, Cordelia Langford, John Sillitoe on behalf of the Wellcome Sanger Institute COVID-19 Surveillance Team
EPI_ISL_631184	Lighthouse Lab in Alderley Park	Wellcome Sanger Institute for the COVID-19 Genomics UK (COG-UK) consortium	Jacquelyn Wynn, Mairead Hyland, The Lighthouse Lab in Alderley Park and Alex Alderton, Roberto Amato, Sonia Goncalves, Ewan Harrison, David K. Jackson, Ian Johnston, Dominic Kwiatkowski, Cordelia Langford, John Sillitoe on behalf of the Wellcome Sanger Institute COVID-19 Surveillance Team
EPI_ISL_631185	Lighthouse Lab in Cambridge	Wellcome Sanger Institute for the COVID-19 Genomics UK (COG-UK) consortium	Rob Howes, The Lighthouse Lab in Cambridge and Alex Alderton, Roberto Amato, Sonia Goncalves, Ewan Harrison, David K. Jackson, Ian Johnston, Dominic Kwiatkowski, Cordelia Langford, John Sillitoe on behalf of the Wellcome Sanger Institute COVID-19 Surveillance Team
EPI_ISL_631186, EPI_ISL_631187, EPI_ISL_631188	Lighthouse Lab in Milton Keynes	Wellcome Sanger Institute for the COVID-19 Genomics UK (COG-UK) consortium	The Lighthouse Lab in Milton Keynes and Alex Alderton, Roberto Amato, Sonia Goncalves, Ewan Harrison, David K. Jackson, Ian Johnston, Dominic Kwiatkowski, Cordelia Langford, John Sillitoe on behalf of the Wellcome Sanger Institute COVID-19 Surveillance Team
EPI_ISL_631189, EPI_ISL_631190	Lighthouse Lab in Alderley Park	Wellcome Sanger Institute for the COVID-19 Genomics UK (COG-UK) consortium	Jacquelyn Wynn, Mairead Hyland, The Lighthouse Lab in Alderley Park and Alex Alderton, Roberto Amato, Sonia Goncalves, Ewan Harrison, David K. Jackson, Ian Johnston, Dominic Kwiatkowski, Cordelia Langford, John Sillitoe on behalf of the Wellcome Sanger Institute COVID-19 Surveillance Team
EPI_ISL_631191, EPI_ISL_631192	Lighthouse Lab in Cambridge	Wellcome Sanger Institute for the COVID-19 Genomics UK (COG-UK) consortium	Rob Howes, The Lighthouse Lab in Cambridge and Alex Alderton, Roberto Amato, Sonia Goncalves, Ewan Harrison, David K. Jackson, Ian Johnston, Dominic Kwiatkowski, Cordelia Langford, John Sillitoe on behalf of the Wellcome Sanger Institute COVID-19 Surveillance Team
EPI_ISL_631193	Lighthouse Lab in Milton Keynes	Wellcome Sanger Institute for the COVID-19 Genomics UK (COG-UK) consortium	The Lighthouse Lab in Milton Keynes and Alex Alderton, Roberto Amato, Sonia Goncalves, Ewan Harrison, David K. Jackson, Ian Johnston, Dominic Kwiatkowski, Cordelia Langford, John Sillitoe on behalf of the Wellcome Sanger Institute COVID-19 Surveillance Team
EPI_ISL_631194	Lighthouse Lab in Cambridge	Wellcome Sanger Institute for the COVID-19 Genomics UK (COG-UK) consortium	Rob Howes, The Lighthouse Lab in Cambridge and Alex Alderton, Roberto Amato, Sonia Goncalves, Ewan Harrison, David K. Jackson, Ian Johnston, Dominic Kwiatkowski, Cordelia Langford, John Sillitoe on behalf of the Wellcome Sanger Institute COVID-19 Surveillance Team
EPI_ISL_631195, EPI_ISL_631196, EPI_ISL_631197, EPI_ISL_631198	Lighthouse Lab in Milton Keynes	Wellcome Sanger Institute for the COVID-19 Genomics UK (COG-UK) consortium	The Lighthouse Lab in Milton Keynes and Alex Alderton, Roberto Amato, Sonia Goncalves, Ewan Harrison, David K. Jackson, Ian Johnston, Dominic Kwiatkowski, Cordelia Langford, John Sillitoe on behalf of the Wellcome Sanger Institute COVID-19 Surveillance Team
EPI_ISL_631199	Lighthouse Lab in Cambridge	Wellcome Sanger Institute for the COVID-19 Genomics UK (COG-UK) consortium	Rob Howes, The Lighthouse Lab in Cambridge and Alex Alderton, Roberto Amato, Sonia Goncalves, Ewan Harrison, David K. Jackson, Ian Johnston, Dominic Kwiatkowski, Cordelia Langford, John Sillitoe on behalf of the Wellcome Sanger Institute COVID-19 Surveillance Team
EPI_ISL_631200, EPI_ISL_631201	Lighthouse Lab in Milton Keynes	Wellcome Sanger Institute for the COVID-19 Genomics UK (COG-UK) consortium	The Lighthouse Lab in Milton Keynes and Alex Alderton, Roberto Amato, Sonia Goncalves, Ewan Harrison, David K. Jackson, Ian Johnston, Dominic Kwiatkowski, Cordelia Langford, John Sillitoe on behalf of the Wellcome Sanger Institute COVID-19 Surveillance Team

EPI_ISL_631202	Lighthouse Lab in Alderley Park	Wellcome Sanger Institute for the COVID-19 Genomics UK (COG-UK) consortium	Jacquelyn Wynn, Mairead Hyland, The Lighthouse Lab in Alderley Park and Alex Alderton, Roberto Amato, Sonia Goncalves, Ewan Harrison, David K. Jackson, Ian Johnston, Dominic Kwiatkowski, Cordelia Langford, John Sillitoe on behalf of the Wellcome Sanger Institute COVID-19 Surveillance Team
EPI_ISL_631203	Lighthouse Lab in Milton Keynes	Wellcome Sanger Institute for the COVID-19 Genomics UK (COG-UK) consortium	The Lighthouse Lab in Milton Keynes and Alex Alderton, Roberto Amato, Sonia Goncalves, Ewan Harrison, David K. Jackson, Ian Johnston, Dominic Kwiatkowski, Cordelia Langford, John Sillitoe on behalf of the Wellcome Sanger Institute COVID-19 Surveillance Team
EPI_ISL_631204	Lighthouse Lab in Cambridge	Wellcome Sanger Institute for the COVID-19 Genomics UK (COG-UK) consortium	Rob Howes, The Lighthouse Lab in Cambridge and Alex Alderton, Roberto Amato, Sonia Goncalves, Ewan Harrison, David K. Jackson, Ian Johnston, Dominic Kwiatkowski, Cordelia Langford, John Sillitoe on behalf of the Wellcome Sanger Institute COVID-19 Surveillance Team
EPI_ISL_631205, EPI_ISL_631206, EPI_ISL_631207	Lighthouse Lab in Milton Keynes	Wellcome Sanger Institute for the COVID-19 Genomics UK (COG-UK) consortium	The Lighthouse Lab in Milton Keynes and Alex Alderton, Roberto Amato, Sonia Goncalves, Ewan Harrison, David K. Jackson, Ian Johnston, Dominic Kwiatkowski, Cordelia Langford, John Sillitoe on behalf of the Wellcome Sanger Institute COVID-19 Surveillance Team
EPI_ISL_631208	Lighthouse Lab in Cambridge	Wellcome Sanger Institute for the COVID-19 Genomics UK (COG-UK) consortium	Rob Howes, The Lighthouse Lab in Cambridge and Alex Alderton, Roberto Amato, Sonia Goncalves, Ewan Harrison, David K. Jackson, Ian Johnston, Dominic Kwiatkowski, Cordelia Langford, John Sillitoe on behalf of the Wellcome Sanger Institute COVID-19 Surveillance Team
EPI_ISL_631209, EPI_ISL_631210, EPI_ISL_631211	Lighthouse Lab in Milton Keynes	Wellcome Sanger Institute for the COVID-19 Genomics UK (COG-UK) consortium	The Lighthouse Lab in Milton Keynes and Alex Alderton, Roberto Amato, Sonia Goncalves, Ewan Harrison, David K. Jackson, Ian Johnston, Dominic Kwiatkowski, Cordelia Langford, John Sillitoe on behalf of the Wellcome Sanger Institute COVID-19 Surveillance Team
EPI_ISL_631212	Lighthouse Lab in Alderley Park	Wellcome Sanger Institute for the COVID-19 Genomics UK (COG-UK) consortium	Jacquelyn Wynn, Mairead Hyland, The Lighthouse Lab in Alderley Park and Alex Alderton, Roberto Amato, Sonia Goncalves, Ewan Harrison, David K. Jackson, Ian Johnston, Dominic Kwiatkowski, Cordelia Langford, John Sillitoe on behalf of the Wellcome Sanger Institute COVID-19 Surveillance Team
EPI_ISL_631213, EPI_ISL_631214	Lighthouse Lab in Milton Keynes	Wellcome Sanger Institute for the COVID-19 Genomics UK (COG-UK) consortium	The Lighthouse Lab in Milton Keynes and Alex Alderton, Roberto Amato, Sonia Goncalves, Ewan Harrison, David K. Jackson, Ian Johnston, Dominic Kwiatkowski, Cordelia Langford, John Sillitoe on behalf of the Wellcome Sanger Institute COVID-19 Surveillance Team
EPI_ISL_631215	Lighthouse Lab in Alderley Park	Wellcome Sanger Institute for the COVID-19 Genomics UK (COG-UK) consortium	Jacquelyn Wynn, Mairead Hyland, The Lighthouse Lab in Alderley Park and Alex Alderton, Roberto Amato, Sonia Goncalves, Ewan Harrison, David K. Jackson, Ian Johnston, Dominic Kwiatkowski, Cordelia Langford, John Sillitoe on behalf of the Wellcome Sanger Institute COVID-19 Surveillance Team
EPI_ISL_631216	Lighthouse Lab in Milton Keynes	Wellcome Sanger Institute for the COVID-19 Genomics UK (COG-UK) consortium	The Lighthouse Lab in Milton Keynes and Alex Alderton, Roberto Amato, Sonia Goncalves, Ewan Harrison, David K. Jackson, Ian Johnston, Dominic Kwiatkowski, Cordelia Langford, John Sillitoe on behalf of the Wellcome Sanger Institute COVID-19 Surveillance Team
EPI_ISL_631217	Lighthouse Lab in Alderley Park	Wellcome Sanger Institute for the COVID-19 Genomics UK (COG-UK) consortium	Jacquelyn Wynn, Mairead Hyland, The Lighthouse Lab in Alderley Park and Alex Alderton, Roberto Amato, Sonia Goncalves, Ewan Harrison, David K. Jackson, Ian Johnston, Dominic Kwiatkowski, Cordelia Langford, John Sillitoe on behalf of the Wellcome Sanger Institute COVID-19 Surveillance Team
EPI_ISL_631218	Lighthouse Lab in Milton Keynes	Wellcome Sanger Institute for the COVID-19 Genomics UK (COG-UK) consortium	The Lighthouse Lab in Milton Keynes and Alex Alderton, Roberto Amato, Sonia Goncalves, Ewan Harrison, David K. Jackson, Ian Johnston, Dominic Kwiatkowski, Cordelia Langford, John Sillitoe on behalf of the Wellcome Sanger Institute COVID-19 Surveillance Team
EPI_ISL_631219, EPI_ISL_631220, EPI_ISL_631221	Lighthouse Lab in Cambridge	Wellcome Sanger Institute for the COVID-19 Genomics UK (COG-UK) consortium	Rob Howes, The Lighthouse Lab in Cambridge and Alex Alderton, Roberto Amato, Sonia Goncalves, Ewan Harrison, David K. Jackson, Ian Johnston, Dominic Kwiatkowski, Cordelia Langford, John Sillitoe on behalf of the Wellcome Sanger Institute COVID-19 Surveillance Team
EPI_ISL_631222	Lighthouse Lab in Alderley Park	Wellcome Sanger Institute for the COVID-19 Genomics UK (COG-UK) consortium	Jacquelyn Wynn, Mairead Hyland, The Lighthouse Lab in Alderley Park and Alex Alderton, Roberto Amato, Sonia Goncalves, Ewan Harrison, David K. Jackson, Ian Johnston, Dominic Kwiatkowski, Cordelia Langford, John Sillitoe on behalf of the Wellcome Sanger Institute COVID-19 Surveillance Team
EPI_ISL_631223	Lighthouse Lab in Milton Keynes	Wellcome Sanger Institute for the COVID-19 Genomics UK (COG-UK) consortium	The Lighthouse Lab in Milton Keynes and Alex Alderton, Roberto Amato, Sonia Goncalves, Ewan Harrison, David K. Jackson, Ian Johnston, Dominic Kwiatkowski, Cordelia Langford, John Sillitoe on behalf of the Wellcome Sanger Institute COVID-19 Surveillance Team
EPI_ISL_631224, EPI_ISL_631225	Lighthouse Lab in Cambridge	Wellcome Sanger Institute for the COVID-19 Genomics UK (COG-UK) consortium	Rob Howes, The Lighthouse Lab in Cambridge and Alex Alderton, Roberto Amato, Sonia Goncalves, Ewan Harrison, David K. Jackson, Ian Johnston, Dominic Kwiatkowski, Cordelia Langford, John Sillitoe on behalf of the Wellcome Sanger Institute COVID-19 Surveillance Team
EPI_ISL_631226, EPI_ISL_631227	Lighthouse Lab in Milton Keynes	Wellcome Sanger Institute for the COVID-19 Genomics UK (COG-UK) consortium	The Lighthouse Lab in Milton Keynes and Alex Alderton, Roberto Amato, Sonia Goncalves, Ewan Harrison, David K. Jackson, Ian Johnston, Dominic Kwiatkowski, Cordelia Langford, John Sillitoe on behalf of the Wellcome Sanger Institute COVID-19 Surveillance Team
EPI_ISL_631228	Lighthouse Lab in Alderley Park	Wellcome Sanger Institute for the COVID-19 Genomics UK (COG-UK) consortium	Jacquelyn Wynn, Mairead Hyland, The Lighthouse Lab in Alderley Park and Alex Alderton, Roberto Amato, Sonia Goncalves, Ewan Harrison, David K. Jackson, Ian Johnston, Dominic Kwiatkowski, Cordelia Langford, John Sillitoe on behalf of the Wellcome Sanger Institute COVID-19 Surveillance Team
EPI_ISL_631229, EPI_ISL_631230	Lighthouse Lab in Milton Keynes	Wellcome Sanger Institute for the COVID-19 Genomics UK (COG-UK) consortium	The Lighthouse Lab in Milton Keynes and Alex Alderton, Roberto Amato, Sonia Goncalves, Ewan Harrison, David K. Jackson, Ian Johnston, Dominic Kwiatkowski, Cordelia Langford, John Sillitoe on behalf of the Wellcome Sanger Institute COVID-19 Surveillance Team
EPI_ISL_631231	Lighthouse Lab in Alderley Park	Wellcome Sanger Institute for the COVID-19 Genomics UK (COG-UK) consortium	Jacquelyn Wynn, Mairead Hyland, The Lighthouse Lab in Alderley Park and Alex Alderton, Roberto Amato, Sonia Goncalves, Ewan Harrison, David K. Jackson, Ian Johnston, Dominic Kwiatkowski, Cordelia Langford, John Sillitoe on behalf of the Wellcome Sanger Institute COVID-19 Surveillance Team
EPI_ISL_631232, EPI_ISL_631233, EPI_ISL_631234	Lighthouse Lab in Milton Keynes	Wellcome Sanger Institute for the COVID-19 Genomics UK (COG-UK) consortium	The Lighthouse Lab in Milton Keynes and Alex Alderton, Roberto Amato, Sonia Goncalves, Ewan Harrison, David K. Jackson, Ian Johnston, Dominic Kwiatkowski, Cordelia Langford, John Sillitoe on behalf of the Wellcome Sanger Institute COVID-19 Surveillance Team
EPI_ISL_631235	Lighthouse Lab in Cambridge	Wellcome Sanger Institute for the COVID-19 Genomics UK (COG-UK) consortium	Rob Howes, The Lighthouse Lab in Cambridge and Alex Alderton, Roberto Amato, Sonia Goncalves, Ewan Harrison, David K. Jackson, Ian Johnston, Dominic Kwiatkowski, Cordelia Langford, John Sillitoe on behalf of the Wellcome Sanger Institute COVID-19 Surveillance Team
EPI_ISL_631236	Lighthouse Lab in Milton Keynes	Wellcome Sanger Institute for the COVID-19 Genomics UK (COG-UK) consortium	The Lighthouse Lab in Milton Keynes and Alex Alderton, Roberto Amato, Sonia Goncalves, Ewan Harrison, David K. Jackson, Ian Johnston, Dominic Kwiatkowski, Cordelia Langford, John Sillitoe on behalf of the Wellcome Sanger Institute COVID-19 Surveillance Team
EPI_ISL_631237	Lighthouse Lab in Cambridge	Wellcome Sanger Institute for the COVID-19 Genomics UK (COG-UK) consortium	Rob Howes, The Lighthouse Lab in Cambridge and Alex Alderton, Roberto Amato, Sonia Goncalves, Ewan Harrison, David K. Jackson, Ian Johnston, Dominic Kwiatkowski, Cordelia Langford, John Sillitoe on behalf of the Wellcome Sanger Institute COVID-19 Surveillance Team
EPI_ISL_631238, EPI_ISL_631239, EPI_ISL_631240	Lighthouse Lab in Milton Keynes	Wellcome Sanger Institute for the COVID-19 Genomics UK (COG-UK) consortium	The Lighthouse Lab in Milton Keynes and Alex Alderton, Roberto Amato, Sonia Goncalves, Ewan Harrison, David K. Jackson, Ian Johnston, Dominic Kwiatkowski, Cordelia Langford, John Sillitoe on behalf of the Wellcome Sanger Institute COVID-19 Surveillance Team
EPI_ISL_631241	Lighthouse Lab in Alderley Park	Wellcome Sanger Institute for the COVID-19 Genomics UK (COG-UK) consortium	Jacquelyn Wynn, Mairead Hyland, The Lighthouse Lab in Alderley Park and Alex Alderton, Roberto Amato, Sonia Goncalves, Ewan Harrison, David K. Jackson, Ian Johnston, Dominic Kwiatkowski, Cordelia Langford, John Sillitoe on behalf of the Wellcome Sanger Institute COVID-19 Surveillance Team
EPI_ISL_631242, EPI_ISL_631243, EPI_ISL_631244	Lighthouse Lab in Milton Keynes	Wellcome Sanger Institute for the COVID-19 Genomics UK (COG-UK) consortium	The Lighthouse Lab in Milton Keynes and Alex Alderton, Roberto Amato, Sonia Goncalves, Ewan Harrison, David K. Jackson, Ian Johnston, Dominic Kwiatkowski, Cordelia Langford, John Sillitoe on behalf of the Wellcome Sanger Institute COVID-19 Surveillance Team
EPI_ISL_631245	Lighthouse Lab in Cambridge	Wellcome Sanger Institute for the COVID-19 Genomics UK (COG-UK) consortium	Rob Howes, The Lighthouse Lab in Cambridge and Alex Alderton, Roberto Amato, Sonia Goncalves, Ewan Harrison, David K. Jackson, Ian Johnston, Dominic Kwiatkowski, Cordelia Langford, John Sillitoe on behalf of the Wellcome Sanger Institute COVID-19 Surveillance Team
EPI_ISL_631246, EPI_ISL_631247, EPI_ISL_631248, EPI_ISL_631249	Lighthouse Lab in Milton Keynes	Wellcome Sanger Institute for the COVID-19 Genomics UK (COG-UK) consortium	The Lighthouse Lab in Milton Keynes and Alex Alderton, Roberto Amato, Sonia Goncalves, Ewan Harrison, David K. Jackson, Ian Johnston, Dominic Kwiatkowski, Cordelia Langford, John Sillitoe on behalf of the Wellcome Sanger Institute COVID-19 Surveillance Team
EPI_ISL_631250	Lighthouse Lab in Alderley Park	Wellcome Sanger Institute for the COVID-19 Genomics UK (COG-UK) consortium	Jacquelyn Wynn, Mairead Hyland, The Lighthouse Lab in Alderley Park and Alex Alderton, Roberto Amato, Sonia Goncalves, Ewan Harrison, David K. Jackson, Ian Johnston, Dominic Kwiatkowski, Cordelia Langford, John Sillitoe on behalf of the Wellcome Sanger Institute COVID-19 Surveillance Team
EPI_ISL_631251, EPI_ISL_631252, EPI_ISL_631253	Lighthouse Lab in Milton Keynes	Wellcome Sanger Institute for the COVID-19 Genomics UK (COG-UK) consortium	The Lighthouse Lab in Milton Keynes and Alex Alderton, Roberto Amato, Sonia Goncalves, Ewan Harrison, David K. Jackson, Ian Johnston, Dominic Kwiatkowski, Cordelia Langford, John Sillitoe on behalf of the Wellcome Sanger Institute COVID-19 Surveillance Team
EPI_ISL_631254	Lighthouse Lab in Cambridge	Wellcome Sanger Institute for the COVID-19 Genomics UK (COG-UK) consortium	Rob Howes, The Lighthouse Lab in Cambridge and Alex Alderton, Roberto Amato, Sonia Goncalves, Ewan Harrison, David K. Jackson, Ian Johnston, Dominic Kwiatkowski, Cordelia Langford, John Sillitoe on behalf of the Wellcome Sanger Institute COVID-19 Surveillance Team
EPI_ISL_631255, EPI_ISL_631256	Lighthouse Lab in Milton Keynes	Wellcome Sanger Institute for the COVID-19 Genomics UK (COG-UK) consortium	The Lighthouse Lab in Milton Keynes and Alex Alderton, Roberto Amato, Sonia Goncalves, Ewan Harrison, David K. Jackson, Ian Johnston, Dominic Kwiatkowski, Cordelia Langford, John Sillitoe on behalf of the Wellcome Sanger Institute COVID-19 Surveillance Team
EPI_ISL_631257	Lighthouse Lab in Cambridge	Wellcome Sanger Institute for the COVID-19 Genomics UK (COG-UK) consortium	Rob Howes, The Lighthouse Lab in Cambridge and Alex Alderton, Roberto Amato, Sonia Goncalves, Ewan Harrison, David K. Jackson, Ian Johnston, Dominic Kwiatkowski, Cordelia Langford, John Sillitoe on behalf of the Wellcome Sanger Institute COVID-19 Surveillance Team
EPI_ISL_631258, EPI_ISL_631259, EPI_ISL_631260, EPI_ISL_631261	Lighthouse Lab in Milton Keynes	Wellcome Sanger Institute for the COVID-19 Genomics UK (COG-UK) consortium	The Lighthouse Lab in Milton Keynes and Alex Alderton, Roberto Amato, Sonia Goncalves, Ewan Harrison, David K. Jackson, Ian Johnston, Dominic Kwiatkowski, Cordelia Langford, John Sillitoe on behalf of the Wellcome Sanger Institute COVID-19 Surveillance Team

EPI_ISL_631262	Lighthouse Lab in Cambridge	Wellcome Sanger Institute for the COVID-19 Genomics UK (COG-UK) consortium	Rob Howes, The Lighthouse Lab in Cambridge and Alex Alderton, Roberto Amato, Sonia Goncalves, Ewan Harrison, David K. Jackson, Ian Johnston, Dominic Kwiatkowski, Cordelia Langford, John Sillitoe on behalf of the Wellcome Sanger Institute COVID-19 Surveillance Team
EPI_ISL_631263, EPI_ISL_631264	Lighthouse Lab in Milton Keynes	Wellcome Sanger Institute for the COVID-19 Genomics UK (COG-UK) consortium	The Lighthouse Lab in Milton Keynes and Alex Alderton, Roberto Amato, Sonia Goncalves, Ewan Harrison, David K. Jackson, Ian Johnston, Dominic Kwiatkowski, Cordelia Langford, John Sillitoe on behalf of the Wellcome Sanger Institute COVID-19 Surveillance Team
EPI_ISL_631265	Lighthouse Lab in Alderley Park	Wellcome Sanger Institute for the COVID-19 Genomics UK (COG-UK) consortium	Jacquelyn Wynn, Mairead Hyland, The Lighthouse Lab in Alderley Park and Alex Alderton, Roberto Amato, Sonia Goncalves, Ewan Harrison, David K. Jackson, Ian Johnston, Dominic Kwiatkowski, Cordelia Langford, John Sillitoe on behalf of the Wellcome Sanger Institute COVID-19 Surveillance Team
EPI_ISL_631266	Lighthouse Lab in Cambridge	Wellcome Sanger Institute for the COVID-19 Genomics UK (COG-UK) consortium	Rob Howes, The Lighthouse Lab in Cambridge and Alex Alderton, Roberto Amato, Sonia Goncalves, Ewan Harrison, David K. Jackson, Ian Johnston, Dominic Kwiatkowski, Cordelia Langford, John Sillitoe on behalf of the Wellcome Sanger Institute COVID-19 Surveillance Team
EPI_ISL_631267	Lighthouse Lab in Milton Keynes	Wellcome Sanger Institute for the COVID-19 Genomics UK (COG-UK) consortium	The Lighthouse Lab in Milton Keynes and Alex Alderton, Roberto Amato, Sonia Goncalves, Ewan Harrison, David K. Jackson, Ian Johnston, Dominic Kwiatkowski, Cordelia Langford, John Sillitoe on behalf of the Wellcome Sanger Institute COVID-19 Surveillance Team
EPI_ISL_631268	Lighthouse Lab in Cambridge	Wellcome Sanger Institute for the COVID-19 Genomics UK (COG-UK) consortium	Rob Howes, The Lighthouse Lab in Cambridge and Alex Alderton, Roberto Amato, Sonia Goncalves, Ewan Harrison, David K. Jackson, Ian Johnston, Dominic Kwiatkowski, Cordelia Langford, John Sillitoe on behalf of the Wellcome Sanger Institute COVID-19 Surveillance Team
EPI_ISL_631269	Lighthouse Lab in Alderley Park	Wellcome Sanger Institute for the COVID-19 Genomics UK (COG-UK) consortium	Jacquelyn Wynn, Mairead Hyland, The Lighthouse Lab in Alderley Park and Alex Alderton, Roberto Amato, Sonia Goncalves, Ewan Harrison, David K. Jackson, Ian Johnston, Dominic Kwiatkowski, Cordelia Langford, John Sillitoe on behalf of the Wellcome Sanger Institute COVID-19 Surveillance Team
EPI_ISL_631270, EPI_ISL_631271, EPI_ISL_631272, EPI_ISL_631273, EPI_ISL_631274	Lighthouse Lab in Cambridge	Wellcome Sanger Institute for the COVID-19 Genomics UK (COG-UK) consortium	Rob Howes, The Lighthouse Lab in Cambridge and Alex Alderton, Roberto Amato, Sonia Goncalves, Ewan Harrison, David K. Jackson, Ian Johnston, Dominic Kwiatkowski, Cordelia Langford, John Sillitoe on behalf of the Wellcome Sanger Institute COVID-19 Surveillance Team
EPI_ISL_631275	Lighthouse Lab in Milton Keynes	Wellcome Sanger Institute for the COVID-19 Genomics UK (COG-UK) consortium	The Lighthouse Lab in Milton Keynes and Alex Alderton, Roberto Amato, Sonia Goncalves, Ewan Harrison, David K. Jackson, Ian Johnston, Dominic Kwiatkowski, Cordelia Langford, John Sillitoe on behalf of the Wellcome Sanger Institute COVID-19 Surveillance Team
EPI_ISL_631277, EPI_ISL_631278, EPI_ISL_631279, EPI_ISL_631280, EPI_ISL_631281, EPI_ISL_631282, EPI_ISL_631283, EPI_ISL_631284, EPI_ISL_631285, EPI_ISL_631286, EPI_ISL_631287, EPI_ISL_631288, EPI_ISL_631289, EPI_ISL_631290, EPI_ISL_631291, EPI_ISL_631292, EPI_ISL_631293, EPI_ISL_631294, EPI_ISL_631295, EPI_ISL_631296, EPI_ISL_631297, EPI_ISL_631298, EPI_ISL_631299, EPI_ISL_631303			
see above	MVZ DIAMEDIS Diagnostische Medizin Sennestadt GmbH	Bielefeld University	David Brandt, Tobias Busche, Markus Haak, Jörn Kalinowski, Levin-Joe Klages, Christiane Scherer, Alexander Sczyrba, Marina Simunovic, Svenja Vinke
EPI_ISL_631304, EPI_ISL_631305, EPI_ISL_631306, EPI_ISL_631307, EPI_ISL_631308, EPI_ISL_631309, EPI_ISL_631310, EPI_ISL_631311, EPI_ISL_631312, EPI_ISL_631313, EPI_ISL_631314, EPI_ISL_631315, EPI_ISL_631316, EPI_ISL_631317, EPI_ISL_631318, EPI_ISL_631319, EPI_ISL_631320, EPI_ISL_631344, EPI_ISL_631345, EPI_ISL_631346, EPI_ISL_631347, EPI_ISL_631348, EPI_ISL_631349, EPI_ISL_631350, EPI_ISL_631351, EPI_ISL_631352, EPI_ISL_631353, EPI_ISL_631354, EPI_ISL_631355, EPI_ISL_631356, EPI_ISL_631357, EPI_ISL_631358, EPI_ISL_631359, EPI_ISL_631360, EPI_ISL_631361, EPI_ISL_631362, EPI_ISL_631363, EPI_ISL_631364, EPI_ISL_631365, EPI_ISL_631366, EPI_ISL_631367, EPI_ISL_631368, EPI_ISL_631369, EPI_ISL_631370, EPI_ISL_631371, EPI_ISL_631372, EPI_ISL_631373, EPI_ISL_631374, EPI_ISL_631375, EPI_ISL_631376			
see above	ZOTZ KLIMAS MVZ Düsseldorf-Centrum GbR ÜBAG für Labormedizin, Genetik, Zytologie, Pathologie	Center of Medical Microbiology, Virology, and Hospital Hygiene, University of Duesseldorf	Maximilian Damagnez, Alexander Dilthey, Ashley-Jane Duplessis, Patrick Finzer, Katrin Hoffmann, Torsten Houwaart, Lisanna Hülse, Malte Kohns Vasconcelos, Marek Korencak, Nadine Lübke, Jessica Nicolai, Klaus Pfeffer, Daniel Strelow, Jörg Timm, Andreas Walker, Tobias Wienemann, Rainer Zotz
EPI_ISL_631377, EPI_ISL_631378, EPI_ISL_631379, EPI_ISL_631380, EPI_ISL_631381, EPI_ISL_631382, EPI_ISL_631383, EPI_ISL_631384, EPI_ISL_631385, EPI_ISL_631386	University Hospital Cologne	Center of Medical Microbiology, Virology, and Hospital Hygiene, University of Duesseldorf	Maximilian Damagnez, Alexander Dilthey, Ashley-Jane Duplessis, Eva Heger, Torsten Houwaart, Rolf Kaiser, Florian Klein, Elena Knops, Malte Kohns Vasconcelos, Jessica Nicolai, Klaus Pfeffer, Gibran Rubio Quintanares, Saleta Sierra-Aragón, Daniel Strelow, Jörg Timm, Andreas Walker, Tobias Wienemann
EPI_ISL_631387	ZOTZ KLIMAS MVZ Düsseldorf-Centrum GbR ÜBAG für Labormedizin, Genetik, Zytologie, Pathologie	Center of Medical Microbiology, Virology, and Hospital Hygiene, University of Duesseldorf	Maximilian Damagnez, Alexander Dilthey, Ashley-Jane Duplessis, Patrick Finzer, Katrin Hoffmann, Torsten Houwaart, Lisanna Hülse, Malte Kohns Vasconcelos, Marek Korencak, Nadine Lübke, Jessica Nicolai, Klaus Pfeffer, Daniel Strelow, Jörg Timm, Andreas Walker, Tobias Wienemann, Rainer Zotz
EPI_ISL_631388	University Hospital Cologne	Center of Medical Microbiology, Virology, and Hospital Hygiene, University of Duesseldorf	Maximilian Damagnez, Alexander Dilthey, Ashley-Jane Duplessis, Eva Heger, Torsten Houwaart, Rolf Kaiser, Florian Klein, Elena Knops, Malte Kohns Vasconcelos, Jessica Nicolai, Klaus Pfeffer, Gibran Rubio Quintanares, Saleta Sierra-Aragón, Daniel Strelow, Jörg Timm, Andreas Walker, Tobias Wienemann
EPI_ISL_631389, EPI_ISL_631397, EPI_ISL_631398, EPI_ISL_631399, EPI_ISL_631400, EPI_ISL_631401, EPI_ISL_631402, EPI_ISL_631403, EPI_ISL_631404, EPI_ISL_631405, EPI_ISL_631406, EPI_ISL_631407, EPI_ISL_631408, EPI_ISL_631409, EPI_ISL_631410, EPI_ISL_631411, EPI_ISL_631412, EPI_ISL_631413, EPI_ISL_631414, EPI_ISL_631415, EPI_ISL_631416, EPI_ISL_631417, EPI_ISL_631418, EPI_ISL_631419, EPI_ISL_631420, EPI_ISL_631421, EPI_ISL_631422, EPI_ISL_631423, EPI_ISL_631424, EPI_ISL_631425, EPI_ISL_631426, EPI_ISL_631427, EPI_ISL_631428, EPI_ISL_631429, EPI_ISL_631430, EPI_ISL_631431, EPI_ISL_631432, EPI_ISL_631433, EPI_ISL_631434, EPI_ISL_631435, EPI_ISL_631436, EPI_ISL_631437, EPI_ISL_631438, EPI_ISL_631439, EPI_ISL_631440, EPI_ISL_631441, EPI_ISL_631442, EPI_ISL_631443, EPI_ISL_631444, EPI_ISL_631445, EPI_ISL_631446, EPI_ISL_631447, EPI_ISL_631448, EPI_ISL_631449, EPI_ISL_631450, EPI_ISL_631451, EPI_ISL_631452, EPI_ISL_631453, EPI_ISL_631454, EPI_ISL_631455, EPI_ISL_631456, EPI_ISL_631457, EPI_ISL_631458, EPI_ISL_631459, EPI_ISL_631460, EPI_ISL_631461, EPI_ISL_631462, EPI_ISL_631463, EPI_ISL_631464, EPI_ISL_631465, EPI_ISL_631466, EPI_ISL_631467, EPI_ISL_631468, EPI_ISL_631469, EPI_ISL_631470, EPI_ISL_631471, EPI_ISL_631472, EPI_ISL_631473, EPI_ISL_631474, EPI_ISL_631475, EPI_ISL_631476, EPI_ISL_631477, EPI_ISL_631478, EPI_ISL_631479, EPI_ISL_631480, EPI_ISL_631481, EPI_ISL_631482, EPI_ISL_631483, EPI_ISL_631484, EPI_ISL_631485, EPI_ISL_631486, EPI_ISL_631487, EPI_ISL_631488, EPI_ISL_631489, EPI_ISL_631490, EPI_ISL_631491, EPI_ISL_631492, EPI_ISL_631493, EPI_ISL_631494, EPI_ISL_631495, EPI_ISL_631496, EPI_ISL_631497, EPI_ISL_631498, EPI_ISL_631499, EPI_ISL_631500, EPI_ISL_631501			
see above	Wisconsin State Laboratory of Hygiene Communicable Disease Division	Wisconsin State Laboratory of Hygiene Communicable Disease Division	Kelsey R. Florek, Abigail C. Shockey
EPI_ISL_631640, EPI_ISL_631641, EPI_ISL_631642, EPI_ISL_631645, EPI_ISL_631646, EPI_ISL_631647, EPI_ISL_631648, EPI_ISL_631649	Texas Department of State Health Services	Texas Department of State Health Services	Rashmi Tuladhar, Bonnie Oh, Jenny Zhang, Maliha Rahman, Anita Pokharel, Myong Koag, Chung Wang, Rachel Lee, Grace Kubin, Mayela Pedrueza
EPI_ISL_632250, EPI_ISL_632251, EPI_ISL_632252, EPI_ISL_632253, EPI_ISL_632254, EPI_ISL_632255	Communicable Disease Laboratory, Public Health Directorate	Communicable Disease Laboratory, Public Health Directorate	AlWasti,H., Altaif,Z., AlHujairi,Z., AlAbbas,Z.
EPI_ISL_632256, EPI_ISL_632257, EPI_ISL_632258	Communicable Disease Laboratory, Public Health Directorate	Communicable Disease Laboratory, Public Health Directorate	AlHujairi,Z., Altaif,Z., AlWasti,H., AlAbbas,Z.
EPI_ISL_632259, EPI_ISL_632260	Communicable Disease Laboratory, Public Health Directorate	Communicable Disease Laboratory, Public Health Directorate	AlTaif,Z., AlWasti,H., AlHujairi,Z., AlAbbas,Z.
EPI_ISL_632261, EPI_ISL_632262, EPI_ISL_632263, EPI_ISL_632264, EPI_ISL_632265, EPI_ISL_632266, EPI_ISL_632267, EPI_ISL_632268, EPI_ISL_632269, EPI_ISL_632270, EPI_ISL_632271, EPI_ISL_632272, EPI_ISL_632273, EPI_ISL_632274, EPI_ISL_632275, EPI_ISL_632276, EPI_ISL_632277, EPI_ISL_632278, EPI_ISL_632279, EPI_ISL_632280, EPI_ISL_632281, EPI_ISL_632282, EPI_ISL_632284			
see above	Communicable Disease Laboratory, Public Health Directorate	Communicable Disease Laboratory, Public Health Directorate	AlWasti,H., AlTaif,Z., AlHujairi,Z., AlAbbas,Z.
EPI_ISL_632286	Laboratory Services Section, Texas Department of State Health Services	Laboratory Services Section, Texas Department of State Health Services	Tuladhar,R., Oh,B., Zhang,J., Rahman,M., Pokharel,A., Pedrueza,M.,Bonser,J., Koag,M., Wang,C., Lee,R. and Kubin,G.
EPI_ISL_632287	Environmental and Global Health, University of Florida	Environmental and Global Health, University of Florida	Alam,M.M., Lauzardo,M., Elbadry,M.A., Stephenson,C.J., Gibson,J.C., Morris,J.G. and Lednicky,J.A.
EPI_ISL_632310	1-Laboratory of Microbiology, National Reference Lab, Charles Nicolle Hospital; 2-University of Tunis ElManar, Faculty of Medicine of Tunis, LR99ES09, Tunis, Tunisia	1-Clinical and Experimental Pharmacology Lab, LR16SP02, National Center of Pharmacovigilance, University of Tunis El Manar, Tunis, Tunisia. 2-Neurodegenerative diseases and psychiatric troubles, LR18SP03, Razi Hospital, University of Tunis El Manar, Tunis, Tunisia. 3- Ministry of Health, National Observatory of New and Emerging Diseases, 1006, Tunis, Tunisia	Ilhem Boutiba-Ben Boubaker, Sameh Trabelsi, Nissaf Ben Alaya, Maher Kharrat, Alia Ben Kahla, Jalila Ben Khelil, Salma Abid, Sana Ferjani, Mouna Ben Sassi, Mouna Safer, Imen Mkada, Imen Kacem, Gaies Emna, Soumaya Rammeh, Riadh Daghfous, Riadh Gouider.
EPI_ISL_632312	NU-sjukvården	Clinical microbiology, Sahlgrenska University Hospital	Johan Ringlander, Josefin Olausson, Hedvig Engström Jakobsson, Magnus Lindh
EPI_ISL_632321, EPI_ISL_632322, EPI_ISL_632323, EPI_ISL_632325, EPI_ISL_632326, EPI_ISL_632327, EPI_ISL_632328, EPI_ISL_632329, EPI_ISL_632330, EPI_ISL_632331, EPI_ISL_632332, EPI_ISL_632333, EPI_ISL_632335, EPI_ISL_632336, EPI_ISL_632337, EPI_ISL_632338, EPI_ISL_632339, EPI_ISL_632340, EPI_ISL_632341, EPI_ISL_632342, EPI_ISL_632343, EPI_ISL_632344, EPI_ISL_632345, EPI_ISL_632346, EPI_ISL_632347, EPI_ISL_632349, EPI_ISL_632350, EPI_ISL_632352, EPI_ISL_632354, EPI_ISL_632355, EPI_ISL_632356, EPI_ISL_632359, EPI_ISL_632360, EPI_ISL_632361, EPI_ISL_632362, EPI_ISL_632363, EPI_ISL_632364, EPI_ISL_632366, EPI_ISL_632367, EPI_ISL_632368, EPI_ISL_632369, EPI_ISL_632370, EPI_ISL_632371, EPI_ISL_632372, EPI_ISL_632373, EPI_ISL_632374, EPI_ISL_632375, EPI_ISL_632376, EPI_ISL_632377, EPI_ISL_632378, EPI_ISL_632379, EPI_ISL_632381, EPI_ISL_632383, EPI_ISL_632386, EPI_ISL_632387, EPI_ISL_632388, EPI_ISL_632389, EPI_ISL_632390, EPI_ISL_632391, EPI_ISL_632440, EPI_ISL_632441, EPI_ISL_632442, EPI_ISL_632443, EPI_ISL_632444, EPI_ISL_632445, EPI_ISL_632446, EPI_ISL_632447, EPI_ISL_632448, EPI_ISL_632449, EPI_ISL_632460, EPI_ISL_632461, EPI_ISL_632480,			

EPI_ISL_632481, EPI_ISL_632482, EPI_ISL_632493, EPI_ISL_632494, EPI_ISL_632495, EPI_ISL_632496, EPI_ISL_632497, EPI_ISL_632498, EPI_ISL_632499, EPI_ISL_632500, EPI_ISL_632501, EPI_ISL_632502, EPI_ISL_632503, EPI_ISL_632504, EPI_ISL_632505, EPI_ISL_632506, EPI_ISL_632507, EPI_ISL_632508, EPI_ISL_632509, EPI_ISL_632510, EPI_ISL_632511, EPI_ISL_632512, EPI_ISL_632513, EPI_ISL_632514, EPI_ISL_632515, EPI_ISL_632516, EPI_ISL_632517, EPI_ISL_632518, EPI_ISL_632519, EPI_ISL_632520, EPI_ISL_632521, EPI_ISL_632522, EPI_ISL_632523, EPI_ISL_632524, EPI_ISL_632525, EPI_ISL_632526, EPI_ISL_632527, EPI_ISL_632528, EPI_ISL_632529, EPI_ISL_632530, EPI_ISL_632531, EPI_ISL_632532, EPI_ISL_632533, EPI_ISL_632534, EPI_ISL_632535, EPI_ISL_632536, EPI_ISL_632537, EPI_ISL_632538, EPI_ISL_632539, EPI_ISL_632540, EPI_ISL_632541, EPI_ISL_632542, EPI_ISL_632543, EPI_ISL_632544, EPI_ISL_632545, EPI_ISL_632546, EPI_ISL_632547, EPI_ISL_632548, EPI_ISL_632549, EPI_ISL_632550, EPI_ISL_632551, EPI_ISL_632552, EPI_ISL_632553, EPI_ISL_632554, EPI_ISL_632555, EPI_ISL_632556, EPI_ISL_632557, EPI_ISL_632558, EPI_ISL_632559, EPI_ISL_632560, EPI_ISL_632561, EPI_ISL_632562, EPI_ISL_632563, EPI_ISL_632564, EPI_ISL_632565, EPI_ISL_632566, EPI_ISL_632567, EPI_ISL_632568, EPI_ISL_632569, EPI_ISL_632570, EPI_ISL_632571, EPI_ISL_632572, EPI_ISL_632573, EPI_ISL_632574, EPI_ISL_632575, EPI_ISL_632576, EPI_ISL_632577, EPI_ISL_632578, EPI_ISL_632579, EPI_ISL_632580, EPI_ISL_632581, EPI_ISL_632582, EPI_ISL_632583, EPI_ISL_632584, EPI_ISL_632585, EPI_ISL_632586, EPI_ISL_632587, EPI_ISL_632588, EPI_ISL_632589, EPI_ISL_632590, EPI_ISL_632591, EPI_ISL_632592, EPI_ISL_632593, EPI_ISL_632594, EPI_ISL_632595, EPI_ISL_632596, EPI_ISL_632597, EPI_ISL_632598, EPI_ISL_632599, EPI_ISL_632600, EPI_ISL_632601, EPI_ISL_632602, EPI_ISL_632603, EPI_ISL_632604, EPI_ISL_632605, EPI_ISL_632606, EPI_ISL_632607, EPI_ISL_632608, EPI_ISL_632609, EPI_ISL_632610, EPI_ISL_632611, EPI_ISL_632612, EPI_ISL_632613, EPI_ISL_632614, EPI_ISL_632615, EPI_ISL_632616, EPI_ISL_632617, EPI_ISL_632618, EPI_ISL_632619, EPI_ISL_632620, EPI_ISL_632621, EPI_ISL_632622, EPI_ISL_632623, EPI_ISL_632624, EPI_ISL_632625, EPI_ISL_632626, EPI_ISL_632627, EPI_ISL_632628, EPI_ISL_632629, EPI_ISL_632630, EPI_ISL_632631, EPI_ISL_632632, EPI_ISL_632633, EPI_ISL_632634, EPI_ISL_632635, EPI_ISL_632636, EPI_ISL_632637, EPI_ISL_632638, EPI_ISL_632639, EPI_ISL_632640, EPI_ISL_632641, EPI_ISL_632642, EPI_ISL_632643, EPI_ISL_632644, EPI_ISL_632645, EPI_ISL_632646, EPI_ISL_632647, EPI_ISL_632648, EPI_ISL_632649, EPI_ISL_632650, EPI_ISL_632651, EPI_ISL_632652, EPI_ISL_632653, EPI_ISL_632654, EPI_ISL_632655, EPI_ISL_632656, EPI_ISL_632657, EPI_ISL_632658, EPI_ISL_632659, EPI_ISL_632660, EPI_ISL_632661, EPI_ISL_632662, EPI_ISL_632663, EPI_ISL_632664, EPI_ISL_632665, EPI_ISL_632666, EPI_ISL_632667, EPI_ISL_632668, EPI_ISL_632669, EPI_ISL_632670, EPI_ISL_632671, EPI_ISL_632672, EPI_ISL_632673, EPI_ISL_632674, EPI_ISL_632675, EPI_ISL_632676, EPI_ISL_632677, EPI_ISL_632678, EPI_ISL_632679, EPI_ISL_632680, EPI_ISL_632681, EPI_ISL_632682, EPI_ISL_632683, EPI_ISL_632684, EPI_ISL_632685, EPI_ISL_632686, EPI_ISL_632687, EPI_ISL_632688, EPI_ISL_632689, EPI_ISL_632690, EPI_ISL_632691, EPI_ISL_632692, EPI_ISL_632693, EPI_ISL_632694, EPI_ISL_632695, EPI_ISL_632696, EPI_ISL_632697, EPI_ISL_632698, EPI_ISL_632699, EPI_ISL_632700, EPI_ISL_632701, EPI_ISL_632702, EPI_ISL_632703, EPI_ISL_632704, EPI_ISL_632705, EPI_ISL_632706, EPI_ISL_632707, EPI_ISL_632708, EPI_ISL_632709, EPI_ISL_632710, EPI_ISL_632711, EPI_ISL_632712, EPI_ISL_632713, EPI_ISL_632714, EPI_ISL_632715, EPI_ISL_632716, EPI_ISL_632717, EPI_ISL_632718, EPI_ISL_632719, EPI_ISL_632720, EPI_ISL_632721, EPI_ISL_632722, EPI_ISL_632723, EPI_ISL_632724, EPI_ISL_632725, EPI_ISL_632726, EPI_ISL_632727, EPI_ISL_632728, EPI_ISL_632729, EPI_ISL_632730, EPI_ISL_632731, EPI_ISL_632732, EPI_ISL_632733, EPI_ISL_632734, EPI_ISL_632735, EPI_ISL_632736, EPI_ISL_632737, EPI_ISL_632738, EPI_ISL_632739, EPI_ISL_632740, EPI_ISL_632741, EPI_ISL_632742, EPI_ISL_632743, EPI_ISL_632744, EPI_ISL_632745, EPI_ISL_632746, EPI_ISL_632747, EPI_ISL_632748, EPI_ISL_632749, EPI_ISL_632750, EPI_ISL_632751, EPI_ISL_632752, EPI_ISL_632753, EPI_ISL_632754, EPI_ISL_632755, EPI_ISL_632756, EPI_ISL_632757, EPI_ISL_632758, EPI_ISL_632759, EPI_ISL_632760, EPI_ISL_632761, EPI_ISL_632762, EPI_ISL_632763, EPI_ISL_632764, EPI_ISL_632765, EPI_ISL_632766, EPI_ISL_632767, EPI_ISL_632768, EPI_ISL_632769, EPI_ISL_632770, EPI_ISL_632771, EPI_ISL_632772, EPI_ISL_632773, EPI_ISL_632774, EPI_ISL_632775, EPI_ISL_632776, EPI_ISL_632777, EPI_ISL_632778, EPI_ISL_632779, EPI_ISL_632780, EPI_ISL_632781, EPI_ISL_632782, EPI_ISL_632783, EPI_ISL_632784, EPI_ISL_632785, EPI_ISL_632786, EPI_ISL_632787, EPI_ISL_632788, EPI_ISL_632789, EPI_ISL_632790, EPI_ISL_632791, EPI_ISL_632792, EPI_ISL_632793, EPI_ISL_632794, EPI_ISL_632795			
see above	Dutch COVID-19 response team	Erasmus Medical Center	Bas Oude Munnink, David Nieuwenhuijse, Reina Sikkema, Claudia Schapendonk, Irina Chestakova, Anne van der Linden, Theo Bestebroer, Stefan van Nieuwkoop, Mark Pronk, Pascal Lexmond, Corien Swaan, Manon Haverkate, Madelief Mollers, Mart Stein, Sandra Kengne Kamga Mobou, Jeroen van Kampen, Jolanda Voermans, Aura Timen, Corine GeurtsvanKessel, Annemiek van der Eijk, Richard Molenkamp, Marion Koopmans, on behalf of the Dutch national COVID-19 response team.
EPI_ISL_632796	Respiratory Virus Unit, Microbiology Services Colindale, Public Health England	Respiratory Virus Unit, Microbiology Services Colindale, Public Health England	PHE Covid Sequencing Team
EPI_ISL_632797, EPI_ISL_632798, EPI_ISL_632799, EPI_ISL_632800, EPI_ISL_632801, EPI_ISL_632802, EPI_ISL_632803, EPI_ISL_632804, EPI_ISL_632805, EPI_ISL_632806, EPI_ISL_632807, EPI_ISL_632808, EPI_ISL_632809, EPI_ISL_632810, EPI_ISL_632811, EPI_ISL_632812, EPI_ISL_632813, EPI_ISL_632815, EPI_ISL_632816, EPI_ISL_632817, EPI_ISL_632818, EPI_ISL_632819, EPI_ISL_632820, EPI_ISL_632821, EPI_ISL_632822			
see above	Microbiology, Department of Pathology, St. Bernard's Hospital, Gibraltar Health Authority	Respiratory Virus Unit, Microbiology Services Colindale, Public Health England	PHE Covid Sequencing Team, Dr Nicholas Cortes (Gibraltar), Charlotte Gillborn-Jones (Gibraltar)
EPI_ISL_632823, EPI_ISL_632824, EPI_ISL_632825, EPI_ISL_632826, EPI_ISL_632827, EPI_ISL_632828, EPI_ISL_632829, EPI_ISL_632830, EPI_ISL_632831, EPI_ISL_632832, EPI_ISL_632833, EPI_ISL_632834, EPI_ISL_632835, EPI_ISL_632836, EPI_ISL_632837, EPI_ISL_632838, EPI_ISL_632839, EPI_ISL_632840, EPI_ISL_632841, EPI_ISL_632842, EPI_ISL_632843, EPI_ISL_632844, EPI_ISL_632845			
see above	New Mexico Department of Health Scientific Laboratory	New Mexico Department of Health Scientific Laboratory	Ellie Johnson, Anastacia Griego-Fisher, D'Eldra Malone
EPI_ISL_632890, EPI_ISL_632891, EPI_ISL_632892, EPI_ISL_632893, EPI_ISL_632894, EPI_ISL_632895, EPI_ISL_632896, EPI_ISL_632897, EPI_ISL_632898	University of Wisconsin-Madison AIDS Vaccine Research Laboratories	University of Wisconsin-Madison AIDS Vaccine Research Laboratories	Gage Moreno, Katarina Braun, et al. AIDS Vaccine Research Laboratories
EPI_ISL_632899, EPI_ISL_632900, EPI_ISL_632901, EPI_ISL_632902, EPI_ISL_632903	Communicable Disease Laboratory, Public Health Directorate	Communicable Disease Laboratory, Public Health Directorate	AlAbbas,Z., Altaif,Z., AlWasti,H., Alhujairi,Z.
EPI_ISL_632904	Communicable Disease Laboratory, Public Health Directorate	Communicable Disease Laboratory, Public Health Directorate	AlTaif,Z., AlHujairi,Z., AlWasti,H., AlAbbas,Z.
EPI_ISL_632905	Communicable Disease Laboratory, Public Health Directorate	Communicable Disease Laboratory, Public Health Directorate	AlHujairi,Z., Altaif,Z., AlWasti,H., AlAbbas,Z.
EPI_ISL_632906, EPI_ISL_632907	Communicable Disease Laboratory, Public Health Directorate	Communicable Disease Laboratory, Public Health Directorate	AlAbbas,Z., Altaif,Z., AlWasti,H., Alhujairi,Z.
EPI_ISL_632934	Department of Acute Infectious Diseases Control and Prevention,Yunnan Provincial Center for Disease Control and Prevention	Department of Acute Infectious Diseases Control and Prevention,Yunnan Provincial Center for Disease Control and Prevention	Meiling Zhang,Jienan Zhou,Senquan Jia,Xiaonan Zhao,Xiaoqing Fu
EPI_ISL_632935	Department of Acute Infectious Diseases Control and Prevention, Yunnan Provincial Center for Disease Control and Prevention	Department of Acute Infectious Diseases Control and Prevention, Yunnan Provincial Center for Disease Control and Prevention	Meiling Zhang, Jienan Zhou, Senquan Jia, Xiaonan Zhao, Xiaoqing Fu
EPI_ISL_632936	Puskesmas Mlati 1 Sleman	Genetics Working Group (Pokja Genetik) Faculty of Medicine, Public Health and Nursing Universitas Gadjah Mada (FK-KMK UGM); Disease Investigation Center Wates Ministry of Agriculture Indonesia; Department of Microbiology FK-KMK UGM; Laboratorium Diagnostik Yayasan Tahija World Mosquito Program (WMP) Yogyakarta Center for Tropical Medicine FK-KMK UGM; Integrated Research Center FK-KMK UGM; Department of Computer Science and Electronics FMIPA UGM; RSUP Dr. Sardjito	Gunadi, Hendra Wibawa, Marcellus, Mohamad S. Hakim, Edwin W. Daniwijaya, Ludhang P. Rizki, Endah Supriyati, Eggi Arguni, Titik Nuryastuti, Tri Wibawa, Dwi AA Nugrahaningsih, Afiahayati, Siswanto, Kristy Iskandar, Nungki Anggorowati, Ika Trisnawati, Sumardi, Eko Budiono, Bambang Sigit Riyanto, Heni Retnowulan, Munawar Gani, Satria Maulana, Nur Rahmi Ananda, Riat El Khair, Yunika Puspadewi, Osman Sianipar, Umi Solekhah Intansari, Elizabeth Henny Herningtiyas, Ira Puspitawati, Nur Imma Fatimah Harahap, Ernawati, Untung Wirawan , William Widitjiarso
EPI_ISL_632937	RSUD Saptosari Gunung Kidul	Genetics Working Group (Pokja Genetik) Faculty of Medicine, Public Health and Nursing Universitas Gadjah Mada (FK-KMK UGM); Disease Investigation Center Wates Ministry of Agriculture Indonesia; Department of Microbiology FK-KMK UGM; Laboratorium Diagnostik Yayasan Tahija World Mosquito Program (WMP) Yogyakarta Center for Tropical Medicine FK-KMK UGM; Integrated Research Center FK-KMK UGM; Department of Computer Science and Electronics FMIPA UGM; Balai Besar Teknik Kesehatan Lingkungan dan Pengendalian Penyakit (BBTKLPP) Yogyakarta	Gunadi, Hendra Wibawa, Marcellus, Mohamad S. Hakim, Edwin W. Daniwijaya, Ludhang P. Rizki, Endah Supriyati, Eggi Arguni, Titik Nuryastuti, Tri Wibawa, Dwi AA Nugrahaningsih, Afiahayati, Siswanto, Kristy Iskandar, Nungki Anggorowati, Irene, Indaryati, Havid Setyawan, Eko Darmawan, Maria Patricia Inggriani, Audric Kenny Tedja
EPI_ISL_632963	NYC Department Of Health And Mental Hygiene	New York City Public Health Laboratory	Jade Wang, et al.
EPI_ISL_632964	Department of Homeless Services	New York City Public Health Laboratory	Jade Wang, et al.
EPI_ISL_632976, EPI_ISL_632977, EPI_ISL_632978, EPI_ISL_632979	Empire City Laboratories	New York City Public Health Laboratory	Jade Wang, et al.
EPI_ISL_632980, EPI_ISL_632981, EPI_ISL_632982, EPI_ISL_632983, EPI_ISL_632984, EPI_ISL_632985, EPI_ISL_632986, EPI_ISL_632987, EPI_ISL_632988, EPI_ISL_632989, EPI_ISL_632990, EPI_ISL_632991, EPI_ISL_632992, EPI_ISL_632993			
see above	DOHMH Corona	New York City Public Health Laboratory	Jade Wang, et al.
EPI_ISL_632994, EPI_ISL_632995, EPI_ISL_632996, EPI_ISL_632997, EPI_ISL_632998	DOHMH Crown Heights	New York City Public Health Laboratory	Jade Wang, et al.
EPI_ISL_632999, EPI_ISL_633000,	DOHMH Jamaica	New York City Public Health Laboratory	Jade Wang, et al.

EPI_ISL_633001, EPI_ISL_633002, EPI_ISL_633003, EPI_ISL_633004, EPI_ISL_633005, EPI_ISL_633006, EPI_ISL_633007, EPI_ISL_633008			
EPI_ISL_633009, EPI_ISL_633010	DOHMH Central Harlem	New York City Public Health Laboratory	Jade Wang, et al.
EPI_ISL_633011	DOHMH PHL	New York City Public Health Laboratory	Jade Wang, et al.
EPI_ISL_633012	DOHMH Riverside	New York City Public Health Laboratory	Jade Wang, et al.
EPI_ISL_633013	DOHMH PHL	New York City Public Health Laboratory	Jade Wang, et al.
EPI_ISL_633014	DOHMH Chelsea	New York City Public Health Laboratory	Jade Wang, et al.
EPI_ISL_633015	DOHMH Morrisania	New York City Public Health Laboratory	Jade Wang, et al.
EPI_ISL_633016, EPI_ISL_633017	DOHMH Chelsea	New York City Public Health Laboratory	Jade Wang, et al.
EPI_ISL_633018	DOHMH Central Harlem	New York City Public Health Laboratory	Jade Wang, et al.
EPI_ISL_633019, EPI_ISL_633020, EPI_ISL_633021, EPI_ISL_633022	DOHMH Morrisania	New York City Public Health Laboratory	Jade Wang, et al.
EPI_ISL_633023	DOHMH Chelsea	New York City Public Health Laboratory	Jade Wang, et al.
EPI_ISL_633024	DOHMH Morrisania	New York City Public Health Laboratory	Jade Wang, et al.
EPI_ISL_633025	DOHMH Chelsea	New York City Public Health Laboratory	Jade Wang, et al.
EPI_ISL_633026	DOHMH PHL	New York City Public Health Laboratory	Jade Wang, et al.
EPI_ISL_633027, EPI_ISL_633028, EPI_ISL_633029, EPI_ISL_633030	DOHMH Morrisania	New York City Public Health Laboratory	Jade Wang, et al.
EPI_ISL_633031	DOHMH Chelsea	New York City Public Health Laboratory	Jade Wang, et al.
EPI_ISL_633032	DOHMH Morrisania	New York City Public Health Laboratory	Jade Wang, et al.
EPI_ISL_633033	DOHMH Chelsea	New York City Public Health Laboratory	Jade Wang, et al.
EPI_ISL_633034	DOHMH Riverside	New York City Public Health Laboratory	Jade Wang, et al.
EPI_ISL_633035	DOHMH PHL	New York City Public Health Laboratory	Jade Wang, et al.
EPI_ISL_633036	DOHMH Morrisania	New York City Public Health Laboratory	Jade Wang, et al.
EPI_ISL_633037	DOHMH Central Harlem	New York City Public Health Laboratory	Jade Wang, et al.
EPI_ISL_633038	DOHMH PHL	New York City Public Health Laboratory	Jade Wang, et al.
EPI_ISL_633039, EPI_ISL_633040	DOHMH Chelsea	New York City Public Health Laboratory	Jade Wang, et al.
EPI_ISL_633041, EPI_ISL_633042, EPI_ISL_633043	DOHMH Riverside	New York City Public Health Laboratory	Jade Wang, et al.
EPI_ISL_633044	DOHMH Morrisania	New York City Public Health Laboratory	Jade Wang, et al.
EPI_ISL_633045	DOHMH PHL	New York City Public Health Laboratory	Jade Wang, et al.
EPI_ISL_633046, EPI_ISL_633047	DOHMH Chelsea	New York City Public Health Laboratory	Jade Wang, et al.
EPI_ISL_633048, EPI_ISL_633049	DOHMH Morrisania	New York City Public Health Laboratory	Jade Wang, et al.
EPI_ISL_633050, EPI_ISL_633051	DOHMH PHL	New York City Public Health Laboratory	Jade Wang, et al.
EPI_ISL_633052	DOHMH Chelsea	New York City Public Health Laboratory	Jade Wang, et al.
EPI_ISL_633053	DOHMH Morrisania	New York City Public Health Laboratory	Jade Wang, et al.
EPI_ISL_633054, EPI_ISL_633055	DOHMH PHL	New York City Public Health Laboratory	Jade Wang, et al.
EPI_ISL_633056	DOHMH Central Harlem	New York City Public Health Laboratory	Jade Wang, et al.
EPI_ISL_633057, EPI_ISL_633058	DOHMH Morrisania	New York City Public Health Laboratory	Jade Wang, et al.
EPI_ISL_633059	DOHMH Corona	New York City Public Health Laboratory	Jade Wang, et al.
EPI_ISL_633060, EPI_ISL_633061, EPI_ISL_633062	DOHMH Jamaica	New York City Public Health Laboratory	Jade Wang, et al.
EPI_ISL_633063	DOHMH Crown Heights	New York City Public Health Laboratory	Jade Wang, et al.
EPI_ISL_633064	DOHMH Jamaica	New York City Public Health Laboratory	Jade Wang, et al.
EPI_ISL_633065	DOHMH Morrisania	New York City Public Health Laboratory	Jade Wang, et al.
EPI_ISL_633066	DOHMH Chelsea	New York City Public Health Laboratory	Jade Wang, et al.
EPI_ISL_633067, EPI_ISL_633068	DOHMH Morrisania	New York City Public Health Laboratory	Jade Wang, et al.
EPI_ISL_633069	DOHMH Chelsea	New York City Public Health Laboratory	Jade Wang, et al.
EPI_ISL_633071	DOHMH PHL	New York City Public Health Laboratory	Jade Wang, et al.
EPI_ISL_633072	DOHMH Riverside	New York City Public Health Laboratory	Jade Wang, et al.
EPI_ISL_633073	DOHMH Fort Greene	New York City Public Health Laboratory	Jade Wang, et al.
EPI_ISL_633074	DOHMH Corona	New York City Public Health Laboratory	Jade Wang, et al.
EPI_ISL_633075	DOHMH Morrisania	New York City Public Health Laboratory	Jade Wang, et al.
EPI_ISL_633076, EPI_ISL_633077, EPI_ISL_633078	DOHMH Chelsea	New York City Public Health Laboratory	Jade Wang, et al.
EPI_ISL_633079	DOHMH PHL	New York City Public Health Laboratory	Jade Wang, et al.
EPI_ISL_633080, EPI_ISL_633081, EPI_ISL_633082	DOHMH Morrisania	New York City Public Health Laboratory	Jade Wang, et al.
EPI_ISL_633083, EPI_ISL_633084, EPI_ISL_633085, EPI_ISL_633086, EPI_ISL_633087, EPI_ISL_633088, EPI_ISL_633089, EPI_ISL_633090, EPI_ISL_633091, EPI_ISL_633092, EPI_ISL_633093, EPI_ISL_633094, EPI_ISL_633095, EPI_ISL_633096, EPI_ISL_633097, EPI_ISL_633098, EPI_ISL_633099			
see above	MD PHL	MD PHL	Maryland Department of Health Laboratories Administration

EPI_ISL_633100, EPI_ISL_633101, EPI_ISL_633102	Lighthouse Lab in Cambridge	Wellcome Sanger Institute for the COVID-19 Genomics UK (COG-UK) consortium	Rob Howes, The Lighthouse Lab in Cambridge and Alex Alderton, Roberto Amato, Sonia Goncalves, Ewan Harrison, David K. Jackson, Ian Johnston, Dominic Kwiatkowski, Cordelia Langford, John Sillitoe on behalf of the Wellcome Sanger Institute COVID-19 Surveillance Team
EPI_ISL_633103	Lighthouse Lab in Glasgow	Wellcome Sanger Institute for the COVID-19 Genomics UK (COG-UK) consortium	Harper VanSteenhouse, Yumi Kasai, David Gray, Carol Clugston, Anna Dominiczak and Alex Alderton, Roberto Amato, Sonia Goncalves, Ewan Harrison, David K. Jackson, Ian Johnston, Dominic Kwiatkowski, Cordelia Langford, John Sillitoe on behalf of the Wellcome Sanger Institute COVID-19 Surveillance Team
EPI_ISL_633104, EPI_ISL_633105, EPI_ISL_633106, EPI_ISL_633107, EPI_ISL_633108, EPI_ISL_633109, EPI_ISL_633110	Lighthouse Lab in Cambridge	Wellcome Sanger Institute for the COVID-19 Genomics UK (COG-UK) consortium	Rob Howes, The Lighthouse Lab in Cambridge and Alex Alderton, Roberto Amato, Sonia Goncalves, Ewan Harrison, David K. Jackson, Ian Johnston, Dominic Kwiatkowski, Cordelia Langford, John Sillitoe on behalf of the Wellcome Sanger Institute COVID-19 Surveillance Team
EPI_ISL_633111, EPI_ISL_633112, EPI_ISL_633113	Lighthouse Lab in Glasgow	Wellcome Sanger Institute for the COVID-19 Genomics UK (COG-UK) consortium	Harper VanSteenhouse, Yumi Kasai, David Gray, Carol Clugston, Anna Dominiczak and Alex Alderton, Roberto Amato, Sonia Goncalves, Ewan Harrison, David K. Jackson, Ian Johnston, Dominic Kwiatkowski, Cordelia Langford, John Sillitoe on behalf of the Wellcome Sanger Institute COVID-19 Surveillance Team
EPI_ISL_633114	Lighthouse Lab in Cambridge	Wellcome Sanger Institute for the COVID-19 Genomics UK (COG-UK) consortium	Rob Howes, The Lighthouse Lab in Cambridge and Alex Alderton, Roberto Amato, Sonia Goncalves, Ewan Harrison, David K. Jackson, Ian Johnston, Dominic Kwiatkowski, Cordelia Langford, John Sillitoe on behalf of the Wellcome Sanger Institute COVID-19 Surveillance Team
EPI_ISL_633115	Lighthouse Lab in Glasgow	Wellcome Sanger Institute for the COVID-19 Genomics UK (COG-UK) consortium	Harper VanSteenhouse, Yumi Kasai, David Gray, Carol Clugston, Anna Dominiczak and Alex Alderton, Roberto Amato, Sonia Goncalves, Ewan Harrison, David K. Jackson, Ian Johnston, Dominic Kwiatkowski, Cordelia Langford, John Sillitoe on behalf of the Wellcome Sanger Institute COVID-19 Surveillance Team
EPI_ISL_633116, EPI_ISL_633117	Lighthouse Lab in Cambridge	Wellcome Sanger Institute for the COVID-19 Genomics UK (COG-UK) consortium	Rob Howes, The Lighthouse Lab in Cambridge and Alex Alderton, Roberto Amato, Sonia Goncalves, Ewan Harrison, David K. Jackson, Ian Johnston, Dominic Kwiatkowski, Cordelia Langford, John Sillitoe on behalf of the Wellcome Sanger Institute COVID-19 Surveillance Team
EPI_ISL_633118	Lighthouse Lab in Glasgow	Wellcome Sanger Institute for the COVID-19 Genomics UK (COG-UK) Consortium	Harper VanSteenhouse, Yumi Kasai, David Gray, Carol Clugston, Anna Dominiczak and Alex Alderton, Roberto Amato, Sonia Goncalves, Ewan Harrison, David K. Jackson, Ian Johnston, Dominic Kwiatkowski, Cordelia Langford, John Sillitoe on behalf of the Wellcome Sanger Institute COVID-19 Surveillance Team
EPI_ISL_633119	Lighthouse Lab in Glasgow	Wellcome Sanger Institute for the COVID-19 Genomics UK (COG-UK) consortium	Harper VanSteenhouse, Yumi Kasai, David Gray, Carol Clugston, Anna Dominiczak and Alex Alderton, Roberto Amato, Sonia Goncalves, Ewan Harrison, David K. Jackson, Ian Johnston, Dominic Kwiatkowski, Cordelia Langford, John Sillitoe on behalf of the Wellcome Sanger Institute COVID-19 Surveillance Team
EPI_ISL_633120, EPI_ISL_633121, EPI_ISL_633122, EPI_ISL_633123, EPI_ISL_633124	Lighthouse Lab in Cambridge	Wellcome Sanger Institute for the COVID-19 Genomics UK (COG-UK) consortium	Rob Howes, The Lighthouse Lab in Cambridge and Alex Alderton, Roberto Amato, Sonia Goncalves, Ewan Harrison, David K. Jackson, Ian Johnston, Dominic Kwiatkowski, Cordelia Langford, John Sillitoe on behalf of the Wellcome Sanger Institute COVID-19 Surveillance Team
EPI_ISL_633125	Lighthouse Lab in Glasgow	Wellcome Sanger Institute for the COVID-19 Genomics UK (COG-UK) consortium	Harper VanSteenhouse, Yumi Kasai, David Gray, Carol Clugston, Anna Dominiczak and Alex Alderton, Roberto Amato, Sonia Goncalves, Ewan Harrison, David K. Jackson, Ian Johnston, Dominic Kwiatkowski, Cordelia Langford, John Sillitoe on behalf of the Wellcome Sanger Institute COVID-19 Surveillance Team
EPI_ISL_633126, EPI_ISL_633127, EPI_ISL_633128, EPI_ISL_633129, EPI_ISL_633130, EPI_ISL_633131	Lighthouse Lab in Cambridge	Wellcome Sanger Institute for the COVID-19 Genomics UK (COG-UK) consortium	Rob Howes, The Lighthouse Lab in Cambridge and Alex Alderton, Roberto Amato, Sonia Goncalves, Ewan Harrison, David K. Jackson, Ian Johnston, Dominic Kwiatkowski, Cordelia Langford, John Sillitoe on behalf of the Wellcome Sanger Institute COVID-19 Surveillance Team
EPI_ISL_633132, EPI_ISL_633133	Lighthouse Lab in Glasgow	Wellcome Sanger Institute for the COVID-19 Genomics UK (COG-UK) consortium	Harper VanSteenhouse, Yumi Kasai, David Gray, Carol Clugston, Anna Dominiczak and Alex Alderton, Roberto Amato, Sonia Goncalves, Ewan Harrison, David K. Jackson, Ian Johnston, Dominic Kwiatkowski, Cordelia Langford, John Sillitoe on behalf of the Wellcome Sanger Institute COVID-19 Surveillance Team
EPI_ISL_633135, EPI_ISL_633136	Lighthouse Lab in Cambridge	Wellcome Sanger Institute for the COVID-19 Genomics UK (COG-UK) consortium	Rob Howes, The Lighthouse Lab in Cambridge and Alex Alderton, Roberto Amato, Sonia Goncalves, Ewan Harrison, David K. Jackson, Ian Johnston, Dominic Kwiatkowski, Cordelia Langford, John Sillitoe on behalf of the Wellcome Sanger Institute COVID-19 Surveillance Team
EPI_ISL_633137, EPI_ISL_633138, EPI_ISL_633139	Lighthouse Lab in Glasgow	Wellcome Sanger Institute for the COVID-19 Genomics UK (COG-UK) consortium	Harper VanSteenhouse, Yumi Kasai, David Gray, Carol Clugston, Anna Dominiczak and Alex Alderton, Roberto Amato, Sonia Goncalves, Ewan Harrison, David K. Jackson, Ian Johnston, Dominic Kwiatkowski, Cordelia Langford, John Sillitoe on behalf of the Wellcome Sanger Institute COVID-19 Surveillance Team
EPI_ISL_633140, EPI_ISL_633141, EPI_ISL_633142	Lighthouse Lab in Cambridge	Wellcome Sanger Institute for the COVID-19 Genomics UK (COG-UK) consortium	Rob Howes, The Lighthouse Lab in Cambridge and Alex Alderton, Roberto Amato, Sonia Goncalves, Ewan Harrison, David K. Jackson, Ian Johnston, Dominic Kwiatkowski, Cordelia Langford, John Sillitoe on behalf of the Wellcome Sanger Institute COVID-19 Surveillance Team
EPI_ISL_633143, EPI_ISL_633144, EPI_ISL_633145, EPI_ISL_633146	Lighthouse Lab in Glasgow	Wellcome Sanger Institute for the COVID-19 Genomics UK (COG-UK) consortium	Harper VanSteenhouse, Yumi Kasai, David Gray, Carol Clugston, Anna Dominiczak and Alex Alderton, Roberto Amato, Sonia Goncalves, Ewan Harrison, David K. Jackson, Ian Johnston, Dominic Kwiatkowski, Cordelia Langford, John Sillitoe on behalf of the Wellcome Sanger Institute COVID-19 Surveillance Team
EPI_ISL_633147, EPI_ISL_633148, EPI_ISL_633149, EPI_ISL_633150	Lighthouse Lab in Cambridge	Wellcome Sanger Institute for the COVID-19 Genomics UK (COG-UK) consortium	Rob Howes, The Lighthouse Lab in Cambridge and Alex Alderton, Roberto Amato, Sonia Goncalves, Ewan Harrison, David K. Jackson, Ian Johnston, Dominic Kwiatkowski, Cordelia Langford, John Sillitoe on behalf of the Wellcome Sanger Institute COVID-19 Surveillance Team
EPI_ISL_633151, EPI_ISL_633152	Lighthouse Lab in Glasgow	Wellcome Sanger Institute for the COVID-19 Genomics UK (COG-UK) consortium	Harper VanSteenhouse, Yumi Kasai, David Gray, Carol Clugston, Anna Dominiczak and Alex Alderton, Roberto Amato, Sonia Goncalves, Ewan Harrison, David K. Jackson, Ian Johnston, Dominic Kwiatkowski, Cordelia Langford, John Sillitoe on behalf of the Wellcome Sanger Institute COVID-19 Surveillance Team
EPI_ISL_633153	Lighthouse Lab in Cambridge	Wellcome Sanger Institute for the COVID-19 Genomics UK (COG-UK) consortium	Rob Howes, The Lighthouse Lab in Cambridge and Alex Alderton, Roberto Amato, Sonia Goncalves, Ewan Harrison, David K. Jackson, Ian Johnston, Dominic Kwiatkowski, Cordelia Langford, John Sillitoe on behalf of the Wellcome Sanger Institute COVID-19 Surveillance Team
EPI_ISL_633154	Lighthouse Lab in Glasgow	Wellcome Sanger Institute for the COVID-19 Genomics UK (COG-UK) consortium	Harper VanSteenhouse, Yumi Kasai, David Gray, Carol Clugston, Anna Dominiczak and Alex Alderton, Roberto Amato, Sonia Goncalves, Ewan Harrison, David K. Jackson, Ian Johnston, Dominic Kwiatkowski, Cordelia Langford, John Sillitoe on behalf of the Wellcome Sanger Institute COVID-19 Surveillance Team
EPI_ISL_633155, EPI_ISL_633156, EPI_ISL_633157	Lighthouse Lab in Cambridge	Wellcome Sanger Institute for the COVID-19 Genomics UK (COG-UK) consortium	Rob Howes, The Lighthouse Lab in Cambridge and Alex Alderton, Roberto Amato, Sonia Goncalves, Ewan Harrison, David K. Jackson, Ian Johnston, Dominic Kwiatkowski, Cordelia Langford, John Sillitoe on behalf of the Wellcome Sanger Institute COVID-19 Surveillance Team
EPI_ISL_633158	Lighthouse Lab in Glasgow	Wellcome Sanger Institute for the COVID-19 Genomics UK (COG-UK) consortium	Harper VanSteenhouse, Yumi Kasai, David Gray, Carol Clugston, Anna Dominiczak and Alex Alderton, Roberto Amato, Sonia Goncalves, Ewan Harrison, David K. Jackson, Ian Johnston, Dominic Kwiatkowski, Cordelia Langford, John Sillitoe on behalf of the Wellcome Sanger Institute COVID-19 Surveillance Team
EPI_ISL_633159	Lighthouse Lab in Cambridge	Wellcome Sanger Institute for the COVID-19 Genomics UK (COG-UK) consortium	Rob Howes, The Lighthouse Lab in Cambridge and Alex Alderton, Roberto Amato, Sonia Goncalves, Ewan Harrison, David K. Jackson, Ian Johnston, Dominic Kwiatkowski, Cordelia Langford, John Sillitoe on behalf of the Wellcome Sanger Institute COVID-19 Surveillance Team
EPI_ISL_633160	Lighthouse Lab in Glasgow	Wellcome Sanger Institute for the COVID-19 Genomics UK (COG-UK) consortium	Harper VanSteenhouse, Yumi Kasai, David Gray, Carol Clugston, Anna Dominiczak and Alex Alderton, Roberto Amato, Sonia Goncalves, Ewan Harrison, David K. Jackson, Ian Johnston, Dominic Kwiatkowski, Cordelia Langford, John Sillitoe on behalf of the Wellcome Sanger Institute COVID-19 Surveillance Team
EPI_ISL_633161	Lighthouse Lab in Cambridge	Wellcome Sanger Institute for the COVID-19 Genomics UK (COG-UK) consortium	Rob Howes, The Lighthouse Lab in Cambridge and Alex Alderton, Roberto Amato, Sonia Goncalves, Ewan Harrison, David K. Jackson, Ian Johnston, Dominic Kwiatkowski, Cordelia Langford, John Sillitoe on behalf of the Wellcome Sanger Institute COVID-19 Surveillance Team
EPI_ISL_633162	Lighthouse Lab in Glasgow	Wellcome Sanger Institute for the COVID-19 Genomics UK (COG-UK) consortium	Harper VanSteenhouse, Yumi Kasai, David Gray, Carol Clugston, Anna Dominiczak and Alex Alderton, Roberto Amato, Sonia Goncalves, Ewan Harrison, David K. Jackson, Ian Johnston, Dominic Kwiatkowski, Cordelia Langford, John Sillitoe on behalf of the Wellcome Sanger Institute COVID-19 Surveillance Team
EPI_ISL_633163, EPI_ISL_633164	Lighthouse Lab in Cambridge	Wellcome Sanger Institute for the COVID-19 Genomics UK (COG-UK) consortium	Rob Howes, The Lighthouse Lab in Cambridge and Alex Alderton, Roberto Amato, Sonia Goncalves, Ewan Harrison, David K. Jackson, Ian Johnston, Dominic Kwiatkowski, Cordelia Langford, John Sillitoe on behalf of the Wellcome Sanger Institute COVID-19 Surveillance Team

EPI_ISL_633165	Lighthouse Lab in Cambridge	Wellcome Sanger Institute for the COVID-19 Genomics UK (COG-UK) Consortium	Rob Howes, The Lighthouse Lab in Cambridge and Alex Alderton, Roberto Amato, Sonia Goncalves, Ewan Harrison, David K. Jackson, Ian Johnston, Dominic Kwiatkowski, Cordelia Langford, John Sillitoe on behalf of the Wellcome Sanger Institute COVID-19 Surveillance Team
EPI_ISL_633166, EPI_ISL_633167, EPI_ISL_633168, EPI_ISL_633169	Lighthouse Lab in Glasgow	Wellcome Sanger Institute for the COVID-19 Genomics UK (COG-UK) consortium	Harper VanSteenhouse, Yumi Kasai, David Gray, Carol Clugston, Anna Dominiczak and Alex Alderton, Roberto Amato, Sonia Goncalves, Ewan Harrison, David K. Jackson, Ian Johnston, Dominic Kwiatkowski, Cordelia Langford, John Sillitoe on behalf of the Wellcome Sanger Institute COVID-19 Surveillance Team
EPI_ISL_633170	Lighthouse Lab in Cambridge	Wellcome Sanger Institute for the COVID-19 Genomics UK (COG-UK) consortium	Rob Howes, The Lighthouse Lab in Cambridge and Alex Alderton, Roberto Amato, Sonia Goncalves, Ewan Harrison, David K. Jackson, Ian Johnston, Dominic Kwiatkowski, Cordelia Langford, John Sillitoe on behalf of the Wellcome Sanger Institute COVID-19 Surveillance Team
EPI_ISL_633171	Lighthouse Lab in Glasgow	Wellcome Sanger Institute for the COVID-19 Genomics UK (COG-UK) consortium	Harper VanSteenhouse, Yumi Kasai, David Gray, Carol Clugston, Anna Dominiczak and Alex Alderton, Roberto Amato, Sonia Goncalves, Ewan Harrison, David K. Jackson, Ian Johnston, Dominic Kwiatkowski, Cordelia Langford, John Sillitoe on behalf of the Wellcome Sanger Institute COVID-19 Surveillance Team
EPI_ISL_633172, EPI_ISL_633173	Lighthouse Lab in Cambridge	Wellcome Sanger Institute for the COVID-19 Genomics UK (COG-UK) consortium	Rob Howes, The Lighthouse Lab in Cambridge and Alex Alderton, Roberto Amato, Sonia Goncalves, Ewan Harrison, David K. Jackson, Ian Johnston, Dominic Kwiatkowski, Cordelia Langford, John Sillitoe on behalf of the Wellcome Sanger Institute COVID-19 Surveillance Team
EPI_ISL_633174, EPI_ISL_633175	Lighthouse Lab in Glasgow	Wellcome Sanger Institute for the COVID-19 Genomics UK (COG-UK) consortium	Harper VanSteenhouse, Yumi Kasai, David Gray, Carol Clugston, Anna Dominiczak and Alex Alderton, Roberto Amato, Sonia Goncalves, Ewan Harrison, David K. Jackson, Ian Johnston, Dominic Kwiatkowski, Cordelia Langford, John Sillitoe on behalf of the Wellcome Sanger Institute COVID-19 Surveillance Team
EPI_ISL_633176, EPI_ISL_633177	Lighthouse Lab in Cambridge	Wellcome Sanger Institute for the COVID-19 Genomics UK (COG-UK) consortium	Rob Howes, The Lighthouse Lab in Cambridge and Alex Alderton, Roberto Amato, Sonia Goncalves, Ewan Harrison, David K. Jackson, Ian Johnston, Dominic Kwiatkowski, Cordelia Langford, John Sillitoe on behalf of the Wellcome Sanger Institute COVID-19 Surveillance Team
EPI_ISL_633178, EPI_ISL_633179, EPI_ISL_633180, EPI_ISL_633181, EPI_ISL_633182	Lighthouse Lab in Glasgow	Wellcome Sanger Institute for the COVID-19 Genomics UK (COG-UK) consortium	Harper VanSteenhouse, Yumi Kasai, David Gray, Carol Clugston, Anna Dominiczak and Alex Alderton, Roberto Amato, Sonia Goncalves, Ewan Harrison, David K. Jackson, Ian Johnston, Dominic Kwiatkowski, Cordelia Langford, John Sillitoe on behalf of the Wellcome Sanger Institute COVID-19 Surveillance Team
EPI_ISL_633183	Lighthouse Lab in Cambridge	Wellcome Sanger Institute for the COVID-19 Genomics UK (COG-UK) consortium	Rob Howes, The Lighthouse Lab in Cambridge and Alex Alderton, Roberto Amato, Sonia Goncalves, Ewan Harrison, David K. Jackson, Ian Johnston, Dominic Kwiatkowski, Cordelia Langford, John Sillitoe on behalf of the Wellcome Sanger Institute COVID-19 Surveillance Team
EPI_ISL_633184, EPI_ISL_633185	Lighthouse Lab in Glasgow	Wellcome Sanger Institute for the COVID-19 Genomics UK (COG-UK) consortium	Harper VanSteenhouse, Yumi Kasai, David Gray, Carol Clugston, Anna Dominiczak and Alex Alderton, Roberto Amato, Sonia Goncalves, Ewan Harrison, David K. Jackson, Ian Johnston, Dominic Kwiatkowski, Cordelia Langford, John Sillitoe on behalf of the Wellcome Sanger Institute COVID-19 Surveillance Team
EPI_ISL_633186, EPI_ISL_633187, EPI_ISL_633188, EPI_ISL_633189	Lighthouse Lab in Cambridge	Wellcome Sanger Institute for the COVID-19 Genomics UK (COG-UK) consortium	Rob Howes, The Lighthouse Lab in Cambridge and Alex Alderton, Roberto Amato, Sonia Goncalves, Ewan Harrison, David K. Jackson, Ian Johnston, Dominic Kwiatkowski, Cordelia Langford, John Sillitoe on behalf of the Wellcome Sanger Institute COVID-19 Surveillance Team
EPI_ISL_633190, EPI_ISL_633191	Lighthouse Lab in Glasgow	Wellcome Sanger Institute for the COVID-19 Genomics UK (COG-UK) consortium	Harper VanSteenhouse, Yumi Kasai, David Gray, Carol Clugston, Anna Dominiczak and Alex Alderton, Roberto Amato, Sonia Goncalves, Ewan Harrison, David K. Jackson, Ian Johnston, Dominic Kwiatkowski, Cordelia Langford, John Sillitoe on behalf of the Wellcome Sanger Institute COVID-19 Surveillance Team
EPI_ISL_633192	Lighthouse Lab in Cambridge	Wellcome Sanger Institute for the COVID-19 Genomics UK (COG-UK) consortium	Rob Howes, The Lighthouse Lab in Cambridge and Alex Alderton, Roberto Amato, Sonia Goncalves, Ewan Harrison, David K. Jackson, Ian Johnston, Dominic Kwiatkowski, Cordelia Langford, John Sillitoe on behalf of the Wellcome Sanger Institute COVID-19 Surveillance Team
EPI_ISL_633193	Lighthouse Lab in Glasgow	Wellcome Sanger Institute for the COVID-19 Genomics UK (COG-UK) consortium	Harper VanSteenhouse, Yumi Kasai, David Gray, Carol Clugston, Anna Dominiczak and Alex Alderton, Roberto Amato, Sonia Goncalves, Ewan Harrison, David K. Jackson, Ian Johnston, Dominic Kwiatkowski, Cordelia Langford, John Sillitoe on behalf of the Wellcome Sanger Institute COVID-19 Surveillance Team
EPI_ISL_633194	Lighthouse Lab in Cambridge	Wellcome Sanger Institute for the COVID-19 Genomics UK (COG-UK) consortium	Rob Howes, The Lighthouse Lab in Cambridge and Alex Alderton, Roberto Amato, Sonia Goncalves, Ewan Harrison, David K. Jackson, Ian Johnston, Dominic Kwiatkowski, Cordelia Langford, John Sillitoe on behalf of the Wellcome Sanger Institute COVID-19 Surveillance Team
EPI_ISL_633195, EPI_ISL_633196	Lighthouse Lab in Glasgow	Wellcome Sanger Institute for the COVID-19 Genomics UK (COG-UK) consortium	Harper VanSteenhouse, Yumi Kasai, David Gray, Carol Clugston, Anna Dominiczak and Alex Alderton, Roberto Amato, Sonia Goncalves, Ewan Harrison, David K. Jackson, Ian Johnston, Dominic Kwiatkowski, Cordelia Langford, John Sillitoe on behalf of the Wellcome Sanger Institute COVID-19 Surveillance Team
EPI_ISL_633197, EPI_ISL_633198, EPI_ISL_633199, EPI_ISL_633200	Lighthouse Lab in Cambridge	Wellcome Sanger Institute for the COVID-19 Genomics UK (COG-UK) consortium	Rob Howes, The Lighthouse Lab in Cambridge and Alex Alderton, Roberto Amato, Sonia Goncalves, Ewan Harrison, David K. Jackson, Ian Johnston, Dominic Kwiatkowski, Cordelia Langford, John Sillitoe on behalf of the Wellcome Sanger Institute COVID-19 Surveillance Team
EPI_ISL_633201, EPI_ISL_633202, EPI_ISL_633203, EPI_ISL_633204	Lighthouse Lab in Glasgow	Wellcome Sanger Institute for the COVID-19 Genomics UK (COG-UK) consortium	Harper VanSteenhouse, Yumi Kasai, David Gray, Carol Clugston, Anna Dominiczak and Alex Alderton, Roberto Amato, Sonia Goncalves, Ewan Harrison, David K. Jackson, Ian Johnston, Dominic Kwiatkowski, Cordelia Langford, John Sillitoe on behalf of the Wellcome Sanger Institute COVID-19 Surveillance Team
EPI_ISL_633205	Lighthouse Lab in Cambridge	Wellcome Sanger Institute for the COVID-19 Genomics UK (COG-UK) consortium	Rob Howes, The Lighthouse Lab in Cambridge and Alex Alderton, Roberto Amato, Sonia Goncalves, Ewan Harrison, David K. Jackson, Ian Johnston, Dominic Kwiatkowski, Cordelia Langford, John Sillitoe on behalf of the Wellcome Sanger Institute COVID-19 Surveillance Team
EPI_ISL_633206, EPI_ISL_633207	Lighthouse Lab in Glasgow	Wellcome Sanger Institute for the COVID-19 Genomics UK (COG-UK) consortium	Harper VanSteenhouse, Yumi Kasai, David Gray, Carol Clugston, Anna Dominiczak and Alex Alderton, Roberto Amato, Sonia Goncalves, Ewan Harrison, David K. Jackson, Ian Johnston, Dominic Kwiatkowski, Cordelia Langford, John Sillitoe on behalf of the Wellcome Sanger Institute COVID-19 Surveillance Team
EPI_ISL_633208	Lighthouse Lab in Cambridge	Wellcome Sanger Institute for the COVID-19 Genomics UK (COG-UK) consortium	Rob Howes, The Lighthouse Lab in Cambridge and Alex Alderton, Roberto Amato, Sonia Goncalves, Ewan Harrison, David K. Jackson, Ian Johnston, Dominic Kwiatkowski, Cordelia Langford, John Sillitoe on behalf of the Wellcome Sanger Institute COVID-19 Surveillance Team
EPI_ISL_633209, EPI_ISL_633210	Lighthouse Lab in Glasgow	Wellcome Sanger Institute for the COVID-19 Genomics UK (COG-UK) consortium	Harper VanSteenhouse, Yumi Kasai, David Gray, Carol Clugston, Anna Dominiczak and Alex Alderton, Roberto Amato, Sonia Goncalves, Ewan Harrison, David K. Jackson, Ian Johnston, Dominic Kwiatkowski, Cordelia Langford, John Sillitoe on behalf of the Wellcome Sanger Institute COVID-19 Surveillance Team
EPI_ISL_633211, EPI_ISL_633212	Lighthouse Lab in Cambridge	Wellcome Sanger Institute for the COVID-19 Genomics UK (COG-UK) consortium	Rob Howes, The Lighthouse Lab in Cambridge and Alex Alderton, Roberto Amato, Sonia Goncalves, Ewan Harrison, David K. Jackson, Ian Johnston, Dominic Kwiatkowski, Cordelia Langford, John Sillitoe on behalf of the Wellcome Sanger Institute COVID-19 Surveillance Team
EPI_ISL_633213	Lighthouse Lab in Glasgow	Wellcome Sanger Institute for the COVID-19 Genomics UK (COG-UK) consortium	Harper VanSteenhouse, Yumi Kasai, David Gray, Carol Clugston, Anna Dominiczak and Alex Alderton, Roberto Amato, Sonia Goncalves, Ewan Harrison, David K. Jackson, Ian Johnston, Dominic Kwiatkowski, Cordelia Langford, John Sillitoe on behalf of the Wellcome Sanger Institute COVID-19 Surveillance Team
EPI_ISL_633214, EPI_ISL_633215, EPI_ISL_633216, EPI_ISL_633217, EPI_ISL_633218, EPI_ISL_633219, EPI_ISL_633220	Lighthouse Lab in Cambridge	Wellcome Sanger Institute for the COVID-19 Genomics UK (COG-UK) consortium	Rob Howes, The Lighthouse Lab in Cambridge and Alex Alderton, Roberto Amato, Sonia Goncalves, Ewan Harrison, David K. Jackson, Ian Johnston, Dominic Kwiatkowski, Cordelia Langford, John Sillitoe on behalf of the Wellcome Sanger Institute COVID-19 Surveillance Team
EPI_ISL_633221	Lighthouse Lab in Cambridge	Wellcome Sanger Institute for the COVID-19 Genomics UK (COG-UK) Consortium	Rob Howes, The Lighthouse Lab in Cambridge and Alex Alderton, Roberto Amato, Sonia Goncalves, Ewan Harrison, David K. Jackson, Ian Johnston, Dominic Kwiatkowski, Cordelia Langford, John Sillitoe on behalf of the Wellcome Sanger Institute COVID-19 Surveillance Team
EPI_ISL_633222	Lighthouse Lab in Glasgow	Wellcome Sanger Institute for the COVID-19 Genomics UK (COG-UK) consortium	Harper VanSteenhouse, Yumi Kasai, David Gray, Carol Clugston, Anna Dominiczak and Alex Alderton, Roberto Amato, Sonia Goncalves, Ewan Harrison, David K. Jackson, Ian Johnston, Dominic Kwiatkowski, Cordelia Langford, John Sillitoe on behalf of the Wellcome Sanger Institute COVID-19 Surveillance Team
EPI_ISL_633223, EPI_ISL_633224, EPI_ISL_633225	Lighthouse Lab in Cambridge	Wellcome Sanger Institute for the COVID-19 Genomics UK (COG-UK) consortium	Rob Howes, The Lighthouse Lab in Cambridge and Alex Alderton, Roberto Amato, Sonia Goncalves, Ewan Harrison, David K. Jackson, Ian Johnston, Dominic Kwiatkowski, Cordelia Langford, John Sillitoe on behalf of the Wellcome Sanger Institute COVID-19 Surveillance Team
EPI_ISL_633226	Lighthouse Lab in Glasgow	Wellcome Sanger Institute for the COVID-19 Genomics UK (COG-UK) consortium	Harper VanSteenhouse, Yumi Kasai, David Gray, Carol Clugston, Anna Dominiczak and Alex Alderton, Roberto Amato, Sonia Goncalves, Ewan Harrison, David K. Jackson, Ian Johnston, Dominic Kwiatkowski, Cordelia Langford, John Sillitoe on behalf of the Wellcome Sanger Institute COVID-19 Surveillance

			Team
EPI_ISL_633227	Lighthouse Lab in Cambridge	Wellcome Sanger Institute for the COVID-19 Genomics UK (COG-UK) consortium	Rob Howes, The Lighthouse Lab in Cambridge and Alex Alderton, Roberto Amato, Sonia Goncalves, Ewan Harrison, David K. Jackson, Ian Johnston, Dominic Kwiatkowski, Cordelia Langford, John Sillitoe on behalf of the Wellcome Sanger Institute COVID-19 Surveillance Team
EPI_ISL_633228	Lighthouse Lab in Glasgow	Wellcome Sanger Institute for the COVID-19 Genomics UK (COG-UK) consortium	Harper VanSteenhouse, Yumi Kasai, David Gray, Carol Clugston, Anna Dominiczak and Alex Alderton, Roberto Amato, Sonia Goncalves, Ewan Harrison, David K. Jackson, Ian Johnston, Dominic Kwiatkowski, Cordelia Langford, John Sillitoe on behalf of the Wellcome Sanger Institute COVID-19 Surveillance Team
EPI_ISL_633229	Lighthouse Lab in Cambridge	Wellcome Sanger Institute for the COVID-19 Genomics UK (COG-UK) consortium	Rob Howes, The Lighthouse Lab in Cambridge and Alex Alderton, Roberto Amato, Sonia Goncalves, Ewan Harrison, David K. Jackson, Ian Johnston, Dominic Kwiatkowski, Cordelia Langford, John Sillitoe on behalf of the Wellcome Sanger Institute COVID-19 Surveillance Team
EPI_ISL_633230	Lighthouse Lab in Glasgow	Wellcome Sanger Institute for the COVID-19 Genomics UK (COG-UK) consortium	Harper VanSteenhouse, Yumi Kasai, David Gray, Carol Clugston, Anna Dominiczak and Alex Alderton, Roberto Amato, Sonia Goncalves, Ewan Harrison, David K. Jackson, Ian Johnston, Dominic Kwiatkowski, Cordelia Langford, John Sillitoe on behalf of the Wellcome Sanger Institute COVID-19 Surveillance Team
EPI_ISL_633231	Lighthouse Lab in Cambridge	Wellcome Sanger Institute for the COVID-19 Genomics UK (COG-UK) consortium	Rob Howes, The Lighthouse Lab in Cambridge and Alex Alderton, Roberto Amato, Sonia Goncalves, Ewan Harrison, David K. Jackson, Ian Johnston, Dominic Kwiatkowski, Cordelia Langford, John Sillitoe on behalf of the Wellcome Sanger Institute COVID-19 Surveillance Team
EPI_ISL_633232, EPI_ISL_633233	Lighthouse Lab in Glasgow	Wellcome Sanger Institute for the COVID-19 Genomics UK (COG-UK) consortium	Harper VanSteenhouse, Yumi Kasai, David Gray, Carol Clugston, Anna Dominiczak and Alex Alderton, Roberto Amato, Sonia Goncalves, Ewan Harrison, David K. Jackson, Ian Johnston, Dominic Kwiatkowski, Cordelia Langford, John Sillitoe on behalf of the Wellcome Sanger Institute COVID-19 Surveillance Team
EPI_ISL_633234, EPI_ISL_633235, EPI_ISL_633236	Lighthouse Lab in Cambridge	Wellcome Sanger Institute for the COVID-19 Genomics UK (COG-UK) consortium	Rob Howes, The Lighthouse Lab in Cambridge and Alex Alderton, Roberto Amato, Sonia Goncalves, Ewan Harrison, David K. Jackson, Ian Johnston, Dominic Kwiatkowski, Cordelia Langford, John Sillitoe on behalf of the Wellcome Sanger Institute COVID-19 Surveillance Team
EPI_ISL_633237	Lighthouse Lab in Glasgow	Wellcome Sanger Institute for the COVID-19 Genomics UK (COG-UK) consortium	Harper VanSteenhouse, Yumi Kasai, David Gray, Carol Clugston, Anna Dominiczak and Alex Alderton, Roberto Amato, Sonia Goncalves, Ewan Harrison, David K. Jackson, Ian Johnston, Dominic Kwiatkowski, Cordelia Langford, John Sillitoe on behalf of the Wellcome Sanger Institute COVID-19 Surveillance Team
EPI_ISL_633238, EPI_ISL_633239	Lighthouse Lab in Cambridge	Wellcome Sanger Institute for the COVID-19 Genomics UK (COG-UK) consortium	Rob Howes, The Lighthouse Lab in Cambridge and Alex Alderton, Roberto Amato, Sonia Goncalves, Ewan Harrison, David K. Jackson, Ian Johnston, Dominic Kwiatkowski, Cordelia Langford, John Sillitoe on behalf of the Wellcome Sanger Institute COVID-19 Surveillance Team
EPI_ISL_633240, EPI_ISL_633241	Lighthouse Lab in Glasgow	Wellcome Sanger Institute for the COVID-19 Genomics UK (COG-UK) consortium	Harper VanSteenhouse, Yumi Kasai, David Gray, Carol Clugston, Anna Dominiczak and Alex Alderton, Roberto Amato, Sonia Goncalves, Ewan Harrison, David K. Jackson, Ian Johnston, Dominic Kwiatkowski, Cordelia Langford, John Sillitoe on behalf of the Wellcome Sanger Institute COVID-19 Surveillance Team
EPI_ISL_633242	Lighthouse Lab in Cambridge	Wellcome Sanger Institute for the COVID-19 Genomics UK (COG-UK) consortium	Rob Howes, The Lighthouse Lab in Cambridge and Alex Alderton, Roberto Amato, Sonia Goncalves, Ewan Harrison, David K. Jackson, Ian Johnston, Dominic Kwiatkowski, Cordelia Langford, John Sillitoe on behalf of the Wellcome Sanger Institute COVID-19 Surveillance Team
EPI_ISL_633243, EPI_ISL_633244	Lighthouse Lab in Glasgow	Wellcome Sanger Institute for the COVID-19 Genomics UK (COG-UK) consortium	Harper VanSteenhouse, Yumi Kasai, David Gray, Carol Clugston, Anna Dominiczak and Alex Alderton, Roberto Amato, Sonia Goncalves, Ewan Harrison, David K. Jackson, Ian Johnston, Dominic Kwiatkowski, Cordelia Langford, John Sillitoe on behalf of the Wellcome Sanger Institute COVID-19 Surveillance Team
EPI_ISL_633245, EPI_ISL_633246, EPI_ISL_633247, EPI_ISL_633248, EPI_ISL_633249, EPI_ISL_633250	Lighthouse Lab in Cambridge	Wellcome Sanger Institute for the COVID-19 Genomics UK (COG-UK) consortium	Rob Howes, The Lighthouse Lab in Cambridge and Alex Alderton, Roberto Amato, Sonia Goncalves, Ewan Harrison, David K. Jackson, Ian Johnston, Dominic Kwiatkowski, Cordelia Langford, John Sillitoe on behalf of the Wellcome Sanger Institute COVID-19 Surveillance Team
EPI_ISL_633251	Lighthouse Lab in Glasgow	Wellcome Sanger Institute for the COVID-19 Genomics UK (COG-UK) consortium	Harper VanSteenhouse, Yumi Kasai, David Gray, Carol Clugston, Anna Dominiczak and Alex Alderton, Roberto Amato, Sonia Goncalves, Ewan Harrison, David K. Jackson, Ian Johnston, Dominic Kwiatkowski, Cordelia Langford, John Sillitoe on behalf of the Wellcome Sanger Institute COVID-19 Surveillance Team
EPI_ISL_633252, EPI_ISL_633253, EPI_ISL_633254, EPI_ISL_633255	Lighthouse Lab in Cambridge	Wellcome Sanger Institute for the COVID-19 Genomics UK (COG-UK) consortium	Rob Howes, The Lighthouse Lab in Cambridge and Alex Alderton, Roberto Amato, Sonia Goncalves, Ewan Harrison, David K. Jackson, Ian Johnston, Dominic Kwiatkowski, Cordelia Langford, John Sillitoe on behalf of the Wellcome Sanger Institute COVID-19 Surveillance Team
EPI_ISL_633256	Lighthouse Lab in Glasgow	Wellcome Sanger Institute for the COVID-19 Genomics UK (COG-UK) consortium	Harper VanSteenhouse, Yumi Kasai, David Gray, Carol Clugston, Anna Dominiczak and Alex Alderton, Roberto Amato, Sonia Goncalves, Ewan Harrison, David K. Jackson, Ian Johnston, Dominic Kwiatkowski, Cordelia Langford, John Sillitoe on behalf of the Wellcome Sanger Institute COVID-19 Surveillance Team
EPI_ISL_633257, EPI_ISL_633258	Lighthouse Lab in Cambridge	Wellcome Sanger Institute for the COVID-19 Genomics UK (COG-UK) consortium	Rob Howes, The Lighthouse Lab in Cambridge and Alex Alderton, Roberto Amato, Sonia Goncalves, Ewan Harrison, David K. Jackson, Ian Johnston, Dominic Kwiatkowski, Cordelia Langford, John Sillitoe on behalf of the Wellcome Sanger Institute COVID-19 Surveillance Team
EPI_ISL_633259	Lighthouse Lab in Glasgow	Wellcome Sanger Institute for the COVID-19 Genomics UK (COG-UK) consortium	Harper VanSteenhouse, Yumi Kasai, David Gray, Carol Clugston, Anna Dominiczak and Alex Alderton, Roberto Amato, Sonia Goncalves, Ewan Harrison, David K. Jackson, Ian Johnston, Dominic Kwiatkowski, Cordelia Langford, John Sillitoe on behalf of the Wellcome Sanger Institute COVID-19 Surveillance Team
EPI_ISL_633260	Lighthouse Lab in Cambridge	Wellcome Sanger Institute for the COVID-19 Genomics UK (COG-UK) consortium	Rob Howes, The Lighthouse Lab in Cambridge and Alex Alderton, Roberto Amato, Sonia Goncalves, Ewan Harrison, David K. Jackson, Ian Johnston, Dominic Kwiatkowski, Cordelia Langford, John Sillitoe on behalf of the Wellcome Sanger Institute COVID-19 Surveillance Team
EPI_ISL_633261, EPI_ISL_633262	Lighthouse Lab in Glasgow	Wellcome Sanger Institute for the COVID-19 Genomics UK (COG-UK) consortium	Harper VanSteenhouse, Yumi Kasai, David Gray, Carol Clugston, Anna Dominiczak and Alex Alderton, Roberto Amato, Sonia Goncalves, Ewan Harrison, David K. Jackson, Ian Johnston, Dominic Kwiatkowski, Cordelia Langford, John Sillitoe on behalf of the Wellcome Sanger Institute COVID-19 Surveillance Team
EPI_ISL_633263, EPI_ISL_633264, EPI_ISL_633265, EPI_ISL_633266, EPI_ISL_633267	Lighthouse Lab in Cambridge	Wellcome Sanger Institute for the COVID-19 Genomics UK (COG-UK) consortium	Rob Howes, The Lighthouse Lab in Cambridge and Alex Alderton, Roberto Amato, Sonia Goncalves, Ewan Harrison, David K. Jackson, Ian Johnston, Dominic Kwiatkowski, Cordelia Langford, John Sillitoe on behalf of the Wellcome Sanger Institute COVID-19 Surveillance Team
EPI_ISL_633268, EPI_ISL_633269	Lighthouse Lab in Glasgow	Wellcome Sanger Institute for the COVID-19 Genomics UK (COG-UK) consortium	Harper VanSteenhouse, Yumi Kasai, David Gray, Carol Clugston, Anna Dominiczak and Alex Alderton, Roberto Amato, Sonia Goncalves, Ewan Harrison, David K. Jackson, Ian Johnston, Dominic Kwiatkowski, Cordelia Langford, John Sillitoe on behalf of the Wellcome Sanger Institute COVID-19 Surveillance Team
EPI_ISL_633270	Lighthouse Lab in Cambridge	Wellcome Sanger Institute for the COVID-19 Genomics UK (COG-UK) consortium	Rob Howes, The Lighthouse Lab in Cambridge and Alex Alderton, Roberto Amato, Sonia Goncalves, Ewan Harrison, David K. Jackson, Ian Johnston, Dominic Kwiatkowski, Cordelia Langford, John Sillitoe on behalf of the Wellcome Sanger Institute COVID-19 Surveillance Team
EPI_ISL_633271, EPI_ISL_633272	Lighthouse Lab in Glasgow	Wellcome Sanger Institute for the COVID-19 Genomics UK (COG-UK) consortium	Harper VanSteenhouse, Yumi Kasai, David Gray, Carol Clugston, Anna Dominiczak and Alex Alderton, Roberto Amato, Sonia Goncalves, Ewan Harrison, David K. Jackson, Ian Johnston, Dominic Kwiatkowski, Cordelia Langford, John Sillitoe on behalf of the Wellcome Sanger Institute COVID-19 Surveillance Team
EPI_ISL_633273	Lighthouse Lab in Cambridge	Wellcome Sanger Institute for the COVID-19 Genomics UK (COG-UK) consortium	Rob Howes, The Lighthouse Lab in Cambridge and Alex Alderton, Roberto Amato, Sonia Goncalves, Ewan Harrison, David K. Jackson, Ian Johnston, Dominic Kwiatkowski, Cordelia Langford, John Sillitoe on behalf of the Wellcome Sanger Institute COVID-19 Surveillance Team
EPI_ISL_633274	Lighthouse Lab in Glasgow	Wellcome Sanger Institute for the COVID-19 Genomics UK (COG-UK) consortium	Harper VanSteenhouse, Yumi Kasai, David Gray, Carol Clugston, Anna Dominiczak and Alex Alderton, Roberto Amato, Sonia Goncalves, Ewan Harrison, David K. Jackson, Ian Johnston, Dominic Kwiatkowski, Cordelia Langford, John Sillitoe on behalf of the Wellcome Sanger Institute COVID-19 Surveillance Team
EPI_ISL_633275	Lighthouse Lab in Cambridge	Wellcome Sanger Institute for the COVID-19 Genomics UK (COG-UK) consortium	Rob Howes, The Lighthouse Lab in Cambridge and Alex Alderton, Roberto Amato, Sonia Goncalves, Ewan Harrison, David K. Jackson, Ian Johnston, Dominic Kwiatkowski, Cordelia Langford, John Sillitoe on behalf of the Wellcome Sanger Institute COVID-19 Surveillance Team
EPI_ISL_633276	Lighthouse Lab in Glasgow	Wellcome Sanger Institute for the COVID-19 Genomics UK (COG-UK) consortium	Harper VanSteenhouse, Yumi Kasai, David Gray, Carol Clugston, Anna Dominiczak and Alex Alderton, Roberto Amato, Sonia Goncalves, Ewan Harrison, David K. Jackson, Ian Johnston, Dominic Kwiatkowski, Cordelia Langford, John Sillitoe on behalf of the Wellcome Sanger Institute COVID-19 Surveillance Team

EPI_ISL_633277, EPI_ISL_633278	Lighthouse Lab in Cambridge	Wellcome Sanger Institute for the COVID-19 Genomics UK (COG-UK) consortium	Rob Howes, The Lighthouse Lab in Cambridge and Alex Alderton, Roberto Amato, Sonia Goncalves, Ewan Harrison, David K. Jackson, Ian Johnston, Dominic Kwiatkowski, Cordelia Langford, John Sillitoe on behalf of the Wellcome Sanger Institute COVID-19 Surveillance Team
EPI_ISL_633279, EPI_ISL_633280	Lighthouse Lab in Glasgow	Wellcome Sanger Institute for the COVID-19 Genomics UK (COG-UK) consortium	Harper VanSteenhouse, Yumi Kasai, David Gray, Carol Clugston, Anna Dominiczak and Alex Alderton, Roberto Amato, Sonia Goncalves, Ewan Harrison, David K. Jackson, Ian Johnston, Dominic Kwiatkowski, Cordelia Langford, John Sillitoe on behalf of the Wellcome Sanger Institute COVID-19 Surveillance Team
EPI_ISL_633281	Lighthouse Lab in Cambridge	Wellcome Sanger Institute for the COVID-19 Genomics UK (COG-UK) consortium	Rob Howes, The Lighthouse Lab in Cambridge and Alex Alderton, Roberto Amato, Sonia Goncalves, Ewan Harrison, David K. Jackson, Ian Johnston, Dominic Kwiatkowski, Cordelia Langford, John Sillitoe on behalf of the Wellcome Sanger Institute COVID-19 Surveillance Team
EPI_ISL_633282	Lighthouse Lab in Glasgow	Wellcome Sanger Institute for the COVID-19 Genomics UK (COG-UK) Consortium	Harper VanSteenhouse, Yumi Kasai, David Gray, Carol Clugston, Anna Dominiczak and Alex Alderton, Roberto Amato, Sonia Goncalves, Ewan Harrison, David K. Jackson, Ian Johnston, Dominic Kwiatkowski, Cordelia Langford, John Sillitoe on behalf of the Wellcome Sanger Institute COVID-19 Surveillance Team
EPI_ISL_633283	Lighthouse Lab in Glasgow	Wellcome Sanger Institute for the COVID-19 Genomics UK (COG-UK) consortium	Harper VanSteenhouse, Yumi Kasai, David Gray, Carol Clugston, Anna Dominiczak and Alex Alderton, Roberto Amato, Sonia Goncalves, Ewan Harrison, David K. Jackson, Ian Johnston, Dominic Kwiatkowski, Cordelia Langford, John Sillitoe on behalf of the Wellcome Sanger Institute COVID-19 Surveillance Team
EPI_ISL_633284	Lighthouse Lab in Cambridge	Wellcome Sanger Institute for the COVID-19 Genomics UK (COG-UK) consortium	Rob Howes, The Lighthouse Lab in Cambridge and Alex Alderton, Roberto Amato, Sonia Goncalves, Ewan Harrison, David K. Jackson, Ian Johnston, Dominic Kwiatkowski, Cordelia Langford, John Sillitoe on behalf of the Wellcome Sanger Institute COVID-19 Surveillance Team
EPI_ISL_633285	Lighthouse Lab in Glasgow	Wellcome Sanger Institute for the COVID-19 Genomics UK (COG-UK) consortium	Harper VanSteenhouse, Yumi Kasai, David Gray, Carol Clugston, Anna Dominiczak and Alex Alderton, Roberto Amato, Sonia Goncalves, Ewan Harrison, David K. Jackson, Ian Johnston, Dominic Kwiatkowski, Cordelia Langford, John Sillitoe on behalf of the Wellcome Sanger Institute COVID-19 Surveillance Team
EPI_ISL_633286	Lighthouse Lab in Cambridge	Wellcome Sanger Institute for the COVID-19 Genomics UK (COG-UK) consortium	Rob Howes, The Lighthouse Lab in Cambridge and Alex Alderton, Roberto Amato, Sonia Goncalves, Ewan Harrison, David K. Jackson, Ian Johnston, Dominic Kwiatkowski, Cordelia Langford, John Sillitoe on behalf of the Wellcome Sanger Institute COVID-19 Surveillance Team
EPI_ISL_633287	Lighthouse Lab in Glasgow	Wellcome Sanger Institute for the COVID-19 Genomics UK (COG-UK) consortium	Harper VanSteenhouse, Yumi Kasai, David Gray, Carol Clugston, Anna Dominiczak and Alex Alderton, Roberto Amato, Sonia Goncalves, Ewan Harrison, David K. Jackson, Ian Johnston, Dominic Kwiatkowski, Cordelia Langford, John Sillitoe on behalf of the Wellcome Sanger Institute COVID-19 Surveillance Team
EPI_ISL_633288	Lighthouse Lab in Cambridge	Wellcome Sanger Institute for the COVID-19 Genomics UK (COG-UK) consortium	Rob Howes, The Lighthouse Lab in Cambridge and Alex Alderton, Roberto Amato, Sonia Goncalves, Ewan Harrison, David K. Jackson, Ian Johnston, Dominic Kwiatkowski, Cordelia Langford, John Sillitoe on behalf of the Wellcome Sanger Institute COVID-19 Surveillance Team
EPI_ISL_633289, EPI_ISL_633290	Lighthouse Lab in Glasgow	Wellcome Sanger Institute for the COVID-19 Genomics UK (COG-UK) consortium	Harper VanSteenhouse, Yumi Kasai, David Gray, Carol Clugston, Anna Dominiczak and Alex Alderton, Roberto Amato, Sonia Goncalves, Ewan Harrison, David K. Jackson, Ian Johnston, Dominic Kwiatkowski, Cordelia Langford, John Sillitoe on behalf of the Wellcome Sanger Institute COVID-19 Surveillance Team
EPI_ISL_633291, EPI_ISL_633292	Lighthouse Lab in Cambridge	Wellcome Sanger Institute for the COVID-19 Genomics UK (COG-UK) consortium	Rob Howes, The Lighthouse Lab in Cambridge and Alex Alderton, Roberto Amato, Sonia Goncalves, Ewan Harrison, David K. Jackson, Ian Johnston, Dominic Kwiatkowski, Cordelia Langford, John Sillitoe on behalf of the Wellcome Sanger Institute COVID-19 Surveillance Team
EPI_ISL_633293	Lighthouse Lab in Glasgow	Wellcome Sanger Institute for the COVID-19 Genomics UK (COG-UK) consortium	Harper VanSteenhouse, Yumi Kasai, David Gray, Carol Clugston, Anna Dominiczak and Alex Alderton, Roberto Amato, Sonia Goncalves, Ewan Harrison, David K. Jackson, Ian Johnston, Dominic Kwiatkowski, Cordelia Langford, John Sillitoe on behalf of the Wellcome Sanger Institute COVID-19 Surveillance Team
EPI_ISL_633294, EPI_ISL_633295	Lighthouse Lab in Cambridge	Wellcome Sanger Institute for the COVID-19 Genomics UK (COG-UK) consortium	Rob Howes, The Lighthouse Lab in Cambridge and Alex Alderton, Roberto Amato, Sonia Goncalves, Ewan Harrison, David K. Jackson, Ian Johnston, Dominic Kwiatkowski, Cordelia Langford, John Sillitoe on behalf of the Wellcome Sanger Institute COVID-19 Surveillance Team
EPI_ISL_633296	Lighthouse Lab in Glasgow	Wellcome Sanger Institute for the COVID-19 Genomics UK (COG-UK) consortium	Harper VanSteenhouse, Yumi Kasai, David Gray, Carol Clugston, Anna Dominiczak and Alex Alderton, Roberto Amato, Sonia Goncalves, Ewan Harrison, David K. Jackson, Ian Johnston, Dominic Kwiatkowski, Cordelia Langford, John Sillitoe on behalf of the Wellcome Sanger Institute COVID-19 Surveillance Team
EPI_ISL_633298, EPI_ISL_633299, EPI_ISL_633300	Lighthouse Lab in Cambridge	Wellcome Sanger Institute for the COVID-19 Genomics UK (COG-UK) consortium	Rob Howes, The Lighthouse Lab in Cambridge and Alex Alderton, Roberto Amato, Sonia Goncalves, Ewan Harrison, David K. Jackson, Ian Johnston, Dominic Kwiatkowski, Cordelia Langford, John Sillitoe on behalf of the Wellcome Sanger Institute COVID-19 Surveillance Team
EPI_ISL_633301	Lighthouse Lab in Glasgow	Wellcome Sanger Institute for the COVID-19 Genomics UK (COG-UK) consortium	Harper VanSteenhouse, Yumi Kasai, David Gray, Carol Clugston, Anna Dominiczak and Alex Alderton, Roberto Amato, Sonia Goncalves, Ewan Harrison, David K. Jackson, Ian Johnston, Dominic Kwiatkowski, Cordelia Langford, John Sillitoe on behalf of the Wellcome Sanger Institute COVID-19 Surveillance Team
EPI_ISL_633302	Lighthouse Lab in Cambridge	Wellcome Sanger Institute for the COVID-19 Genomics UK (COG-UK) consortium	Rob Howes, The Lighthouse Lab in Cambridge and Alex Alderton, Roberto Amato, Sonia Goncalves, Ewan Harrison, David K. Jackson, Ian Johnston, Dominic Kwiatkowski, Cordelia Langford, John Sillitoe on behalf of the Wellcome Sanger Institute COVID-19 Surveillance Team
EPI_ISL_633303, EPI_ISL_633304, EPI_ISL_633305, EPI_ISL_633306, EPI_ISL_633307	Lighthouse Lab in Glasgow	Wellcome Sanger Institute for the COVID-19 Genomics UK (COG-UK) consortium	Harper VanSteenhouse, Yumi Kasai, David Gray, Carol Clugston, Anna Dominiczak and Alex Alderton, Roberto Amato, Sonia Goncalves, Ewan Harrison, David K. Jackson, Ian Johnston, Dominic Kwiatkowski, Cordelia Langford, John Sillitoe on behalf of the Wellcome Sanger Institute COVID-19 Surveillance Team
EPI_ISL_633308	Lighthouse Lab in Cambridge	Wellcome Sanger Institute for the COVID-19 Genomics UK (COG-UK) consortium	Rob Howes, The Lighthouse Lab in Cambridge and Alex Alderton, Roberto Amato, Sonia Goncalves, Ewan Harrison, David K. Jackson, Ian Johnston, Dominic Kwiatkowski, Cordelia Langford, John Sillitoe on behalf of the Wellcome Sanger Institute COVID-19 Surveillance Team
EPI_ISL_633309, EPI_ISL_633310	Lighthouse Lab in Glasgow	Wellcome Sanger Institute for the COVID-19 Genomics UK (COG-UK) consortium	Harper VanSteenhouse, Yumi Kasai, David Gray, Carol Clugston, Anna Dominiczak and Alex Alderton, Roberto Amato, Sonia Goncalves, Ewan Harrison, David K. Jackson, Ian Johnston, Dominic Kwiatkowski, Cordelia Langford, John Sillitoe on behalf of the Wellcome Sanger Institute COVID-19 Surveillance Team
EPI_ISL_633311	Lighthouse Lab in Cambridge	Wellcome Sanger Institute for the COVID-19 Genomics UK (COG-UK) consortium	Rob Howes, The Lighthouse Lab in Cambridge and Alex Alderton, Roberto Amato, Sonia Goncalves, Ewan Harrison, David K. Jackson, Ian Johnston, Dominic Kwiatkowski, Cordelia Langford, John Sillitoe on behalf of the Wellcome Sanger Institute COVID-19 Surveillance Team
EPI_ISL_633312	Lighthouse Lab in Glasgow	Wellcome Sanger Institute for the COVID-19 Genomics UK (COG-UK) consortium	Harper VanSteenhouse, Yumi Kasai, David Gray, Carol Clugston, Anna Dominiczak and Alex Alderton, Roberto Amato, Sonia Goncalves, Ewan Harrison, David K. Jackson, Ian Johnston, Dominic Kwiatkowski, Cordelia Langford, John Sillitoe on behalf of the Wellcome Sanger Institute COVID-19 Surveillance Team
EPI_ISL_633313, EPI_ISL_633314, EPI_ISL_633315	Lighthouse Lab in Cambridge	Wellcome Sanger Institute for the COVID-19 Genomics UK (COG-UK) consortium	Rob Howes, The Lighthouse Lab in Cambridge and Alex Alderton, Roberto Amato, Sonia Goncalves, Ewan Harrison, David K. Jackson, Ian Johnston, Dominic Kwiatkowski, Cordelia Langford, John Sillitoe on behalf of the Wellcome Sanger Institute COVID-19 Surveillance Team
EPI_ISL_633316, EPI_ISL_633317, EPI_ISL_633318	Lighthouse Lab in Glasgow	Wellcome Sanger Institute for the COVID-19 Genomics UK (COG-UK) consortium	Harper VanSteenhouse, Yumi Kasai, David Gray, Carol Clugston, Anna Dominiczak and Alex Alderton, Roberto Amato, Sonia Goncalves, Ewan Harrison, David K. Jackson, Ian Johnston, Dominic Kwiatkowski, Cordelia Langford, John Sillitoe on behalf of the Wellcome Sanger Institute COVID-19 Surveillance Team
EPI_ISL_633319	Lighthouse Lab in Cambridge	Wellcome Sanger Institute for the COVID-19 Genomics UK (COG-UK) consortium	Rob Howes, The Lighthouse Lab in Cambridge and Alex Alderton, Roberto Amato, Sonia Goncalves, Ewan Harrison, David K. Jackson, Ian Johnston, Dominic Kwiatkowski, Cordelia Langford, John Sillitoe on behalf of the Wellcome Sanger Institute COVID-19 Surveillance Team
EPI_ISL_633320	Lighthouse Lab in Glasgow	Wellcome Sanger Institute for the COVID-19 Genomics UK (COG-UK) consortium	Harper VanSteenhouse, Yumi Kasai, David Gray, Carol Clugston, Anna Dominiczak and Alex Alderton, Roberto Amato, Sonia Goncalves, Ewan Harrison, David K. Jackson, Ian Johnston, Dominic Kwiatkowski, Cordelia Langford, John Sillitoe on behalf of the Wellcome Sanger Institute COVID-19 Surveillance Team
EPI_ISL_633321, EPI_ISL_633322	Lighthouse Lab in Cambridge	Wellcome Sanger Institute for the COVID-19 Genomics UK (COG-UK) consortium	Rob Howes, The Lighthouse Lab in Cambridge and Alex Alderton, Roberto Amato, Sonia Goncalves, Ewan Harrison, David K. Jackson, Ian Johnston, Dominic Kwiatkowski, Cordelia Langford, John Sillitoe on behalf of the Wellcome Sanger Institute COVID-19 Surveillance Team
EPI_ISL_633323	Lighthouse Lab in Glasgow	Wellcome Sanger Institute for the COVID-19 Genomics UK (COG-UK) consortium	Harper VanSteenhouse, Yumi Kasai, David Gray, Carol Clugston, Anna Dominiczak and Alex Alderton, Roberto Amato, Sonia Goncalves, Ewan Harrison, David K. Jackson, Ian Johnston, Dominic Kwiatkowski, Cordelia Langford, John Sillitoe on behalf of the Wellcome Sanger Institute COVID-19 Surveillance Team

EPI_ISL_633324, EPI_ISL_633325	Lighthouse Lab in Cambridge	Wellcome Sanger Institute for the COVID-19 Genomics UK (COG-UK) consortium	Rob Howes, The Lighthouse Lab in Cambridge and Alex Alderton, Roberto Amato, Sonia Goncalves, Ewan Harrison, David K. Jackson, Ian Johnston, Dominic Kwiatkowski, Cordelia Langford, John Sillitoe on behalf of the Wellcome Sanger Institute COVID-19 Surveillance Team
EPI_ISL_633326, EPI_ISL_633327, EPI_ISL_633328	Lighthouse Lab in Glasgow	Wellcome Sanger Institute for the COVID-19 Genomics UK (COG-UK) consortium	Harper VanSteenhouse, Yumi Kasai, David Gray, Carol Clugston, Anna Dominiczak and Alex Alderton, Roberto Amato, Sonia Goncalves, Ewan Harrison, David K. Jackson, Ian Johnston, Dominic Kwiatkowski, Cordelia Langford, John Sillitoe on behalf of the Wellcome Sanger Institute COVID-19 Surveillance Team
EPI_ISL_633329, EPI_ISL_633330	Lighthouse Lab in Cambridge	Wellcome Sanger Institute for the COVID-19 Genomics UK (COG-UK) consortium	Rob Howes, The Lighthouse Lab in Cambridge and Alex Alderton, Roberto Amato, Sonia Goncalves, Ewan Harrison, David K. Jackson, Ian Johnston, Dominic Kwiatkowski, Cordelia Langford, John Sillitoe on behalf of the Wellcome Sanger Institute COVID-19 Surveillance Team
EPI_ISL_633331, EPI_ISL_633332	Lighthouse Lab in Glasgow	Wellcome Sanger Institute for the COVID-19 Genomics UK (COG-UK) consortium	Harper VanSteenhouse, Yumi Kasai, David Gray, Carol Clugston, Anna Dominiczak and Alex Alderton, Roberto Amato, Sonia Goncalves, Ewan Harrison, David K. Jackson, Ian Johnston, Dominic Kwiatkowski, Cordelia Langford, John Sillitoe on behalf of the Wellcome Sanger Institute COVID-19 Surveillance Team
EPI_ISL_633333, EPI_ISL_633334	Lighthouse Lab in Cambridge	Wellcome Sanger Institute for the COVID-19 Genomics UK (COG-UK) consortium	Rob Howes, The Lighthouse Lab in Cambridge and Alex Alderton, Roberto Amato, Sonia Goncalves, Ewan Harrison, David K. Jackson, Ian Johnston, Dominic Kwiatkowski, Cordelia Langford, John Sillitoe on behalf of the Wellcome Sanger Institute COVID-19 Surveillance Team
EPI_ISL_633335	Lighthouse Lab in Glasgow	Wellcome Sanger Institute for the COVID-19 Genomics UK (COG-UK) consortium	Harper VanSteenhouse, Yumi Kasai, David Gray, Carol Clugston, Anna Dominiczak and Alex Alderton, Roberto Amato, Sonia Goncalves, Ewan Harrison, David K. Jackson, Ian Johnston, Dominic Kwiatkowski, Cordelia Langford, John Sillitoe on behalf of the Wellcome Sanger Institute COVID-19 Surveillance Team
EPI_ISL_633336, EPI_ISL_633337	Lighthouse Lab in Cambridge	Wellcome Sanger Institute for the COVID-19 Genomics UK (COG-UK) consortium	Rob Howes, The Lighthouse Lab in Cambridge and Alex Alderton, Roberto Amato, Sonia Goncalves, Ewan Harrison, David K. Jackson, Ian Johnston, Dominic Kwiatkowski, Cordelia Langford, John Sillitoe on behalf of the Wellcome Sanger Institute COVID-19 Surveillance Team
EPI_ISL_633338, EPI_ISL_633339	Lighthouse Lab in Glasgow	Wellcome Sanger Institute for the COVID-19 Genomics UK (COG-UK) consortium	Harper VanSteenhouse, Yumi Kasai, David Gray, Carol Clugston, Anna Dominiczak and Alex Alderton, Roberto Amato, Sonia Goncalves, Ewan Harrison, David K. Jackson, Ian Johnston, Dominic Kwiatkowski, Cordelia Langford, John Sillitoe on behalf of the Wellcome Sanger Institute COVID-19 Surveillance Team
EPI_ISL_633340, EPI_ISL_633341	Lighthouse Lab in Cambridge	Wellcome Sanger Institute for the COVID-19 Genomics UK (COG-UK) consortium	Rob Howes, The Lighthouse Lab in Cambridge and Alex Alderton, Roberto Amato, Sonia Goncalves, Ewan Harrison, David K. Jackson, Ian Johnston, Dominic Kwiatkowski, Cordelia Langford, John Sillitoe on behalf of the Wellcome Sanger Institute COVID-19 Surveillance Team
EPI_ISL_633342, EPI_ISL_633343	Lighthouse Lab in Glasgow	Wellcome Sanger Institute for the COVID-19 Genomics UK (COG-UK) consortium	Harper VanSteenhouse, Yumi Kasai, David Gray, Carol Clugston, Anna Dominiczak and Alex Alderton, Roberto Amato, Sonia Goncalves, Ewan Harrison, David K. Jackson, Ian Johnston, Dominic Kwiatkowski, Cordelia Langford, John Sillitoe on behalf of the Wellcome Sanger Institute COVID-19 Surveillance Team
EPI_ISL_633344, EPI_ISL_633345, EPI_ISL_633346, EPI_ISL_633347	Lighthouse Lab in Cambridge	Wellcome Sanger Institute for the COVID-19 Genomics UK (COG-UK) consortium	Rob Howes, The Lighthouse Lab in Cambridge and Alex Alderton, Roberto Amato, Sonia Goncalves, Ewan Harrison, David K. Jackson, Ian Johnston, Dominic Kwiatkowski, Cordelia Langford, John Sillitoe on behalf of the Wellcome Sanger Institute COVID-19 Surveillance Team
EPI_ISL_633348	Lighthouse Lab in Glasgow	Wellcome Sanger Institute for the COVID-19 Genomics UK (COG-UK) consortium	Harper VanSteenhouse, Yumi Kasai, David Gray, Carol Clugston, Anna Dominiczak and Alex Alderton, Roberto Amato, Sonia Goncalves, Ewan Harrison, David K. Jackson, Ian Johnston, Dominic Kwiatkowski, Cordelia Langford, John Sillitoe on behalf of the Wellcome Sanger Institute COVID-19 Surveillance Team
EPI_ISL_633349, EPI_ISL_633350	Lighthouse Lab in Cambridge	Wellcome Sanger Institute for the COVID-19 Genomics UK (COG-UK) consortium	Rob Howes, The Lighthouse Lab in Cambridge and Alex Alderton, Roberto Amato, Sonia Goncalves, Ewan Harrison, David K. Jackson, Ian Johnston, Dominic Kwiatkowski, Cordelia Langford, John Sillitoe on behalf of the Wellcome Sanger Institute COVID-19 Surveillance Team
EPI_ISL_633351, EPI_ISL_633352, EPI_ISL_633353, EPI_ISL_633354, EPI_ISL_633355	Lighthouse Lab in Glasgow	Wellcome Sanger Institute for the COVID-19 Genomics UK (COG-UK) consortium	Harper VanSteenhouse, Yumi Kasai, David Gray, Carol Clugston, Anna Dominiczak and Alex Alderton, Roberto Amato, Sonia Goncalves, Ewan Harrison, David K. Jackson, Ian Johnston, Dominic Kwiatkowski, Cordelia Langford, John Sillitoe on behalf of the Wellcome Sanger Institute COVID-19 Surveillance Team
EPI_ISL_633356, EPI_ISL_633357	Lighthouse Lab in Cambridge	Wellcome Sanger Institute for the COVID-19 Genomics UK (COG-UK) consortium	Rob Howes, The Lighthouse Lab in Cambridge and Alex Alderton, Roberto Amato, Sonia Goncalves, Ewan Harrison, David K. Jackson, Ian Johnston, Dominic Kwiatkowski, Cordelia Langford, John Sillitoe on behalf of the Wellcome Sanger Institute COVID-19 Surveillance Team
EPI_ISL_633358	Lighthouse Lab in Glasgow	Wellcome Sanger Institute for the COVID-19 Genomics UK (COG-UK) consortium	Harper VanSteenhouse, Yumi Kasai, David Gray, Carol Clugston, Anna Dominiczak and Alex Alderton, Roberto Amato, Sonia Goncalves, Ewan Harrison, David K. Jackson, Ian Johnston, Dominic Kwiatkowski, Cordelia Langford, John Sillitoe on behalf of the Wellcome Sanger Institute COVID-19 Surveillance Team
EPI_ISL_633359, EPI_ISL_633360	Lighthouse Lab in Cambridge	Wellcome Sanger Institute for the COVID-19 Genomics UK (COG-UK) consortium	Rob Howes, The Lighthouse Lab in Cambridge and Alex Alderton, Roberto Amato, Sonia Goncalves, Ewan Harrison, David K. Jackson, Ian Johnston, Dominic Kwiatkowski, Cordelia Langford, John Sillitoe on behalf of the Wellcome Sanger Institute COVID-19 Surveillance Team
EPI_ISL_633362	Lighthouse Lab in Glasgow	Wellcome Sanger Institute for the COVID-19 Genomics UK (COG-UK) consortium	Harper VanSteenhouse, Yumi Kasai, David Gray, Carol Clugston, Anna Dominiczak and Alex Alderton, Roberto Amato, Sonia Goncalves, Ewan Harrison, David K. Jackson, Ian Johnston, Dominic Kwiatkowski, Cordelia Langford, John Sillitoe on behalf of the Wellcome Sanger Institute COVID-19 Surveillance Team
EPI_ISL_633363	Lighthouse Lab in Cambridge	Wellcome Sanger Institute for the COVID-19 Genomics UK (COG-UK) consortium	Rob Howes, The Lighthouse Lab in Cambridge and Alex Alderton, Roberto Amato, Sonia Goncalves, Ewan Harrison, David K. Jackson, Ian Johnston, Dominic Kwiatkowski, Cordelia Langford, John Sillitoe on behalf of the Wellcome Sanger Institute COVID-19 Surveillance Team
EPI_ISL_633364	Lighthouse Lab in Glasgow	Wellcome Sanger Institute for the COVID-19 Genomics UK (COG-UK) consortium	Harper VanSteenhouse, Yumi Kasai, David Gray, Carol Clugston, Anna Dominiczak and Alex Alderton, Roberto Amato, Sonia Goncalves, Ewan Harrison, David K. Jackson, Ian Johnston, Dominic Kwiatkowski, Cordelia Langford, John Sillitoe on behalf of the Wellcome Sanger Institute COVID-19 Surveillance Team
EPI_ISL_633365, EPI_ISL_633366	Lighthouse Lab in Cambridge	Wellcome Sanger Institute for the COVID-19 Genomics UK (COG-UK) consortium	Rob Howes, The Lighthouse Lab in Cambridge and Alex Alderton, Roberto Amato, Sonia Goncalves, Ewan Harrison, David K. Jackson, Ian Johnston, Dominic Kwiatkowski, Cordelia Langford, John Sillitoe on behalf of the Wellcome Sanger Institute COVID-19 Surveillance Team
EPI_ISL_633367, EPI_ISL_633368	Lighthouse Lab in Glasgow	Wellcome Sanger Institute for the COVID-19 Genomics UK (COG-UK) consortium	Harper VanSteenhouse, Yumi Kasai, David Gray, Carol Clugston, Anna Dominiczak and Alex Alderton, Roberto Amato, Sonia Goncalves, Ewan Harrison, David K. Jackson, Ian Johnston, Dominic Kwiatkowski, Cordelia Langford, John Sillitoe on behalf of the Wellcome Sanger Institute COVID-19 Surveillance Team
EPI_ISL_633369, EPI_ISL_633370	Lighthouse Lab in Cambridge	Wellcome Sanger Institute for the COVID-19 Genomics UK (COG-UK) consortium	Rob Howes, The Lighthouse Lab in Cambridge and Alex Alderton, Roberto Amato, Sonia Goncalves, Ewan Harrison, David K. Jackson, Ian Johnston, Dominic Kwiatkowski, Cordelia Langford, John Sillitoe on behalf of the Wellcome Sanger Institute COVID-19 Surveillance Team
EPI_ISL_633371	Lighthouse Lab in Glasgow	Wellcome Sanger Institute for the COVID-19 Genomics UK (COG-UK) consortium	Harper VanSteenhouse, Yumi Kasai, David Gray, Carol Clugston, Anna Dominiczak and Alex Alderton, Roberto Amato, Sonia Goncalves, Ewan Harrison, David K. Jackson, Ian Johnston, Dominic Kwiatkowski, Cordelia Langford, John Sillitoe on behalf of the Wellcome Sanger Institute COVID-19 Surveillance Team
EPI_ISL_633372, EPI_ISL_633373, EPI_ISL_633374, EPI_ISL_633375	Lighthouse Lab in Cambridge	Wellcome Sanger Institute for the COVID-19 Genomics UK (COG-UK) consortium	Rob Howes, The Lighthouse Lab in Cambridge and Alex Alderton, Roberto Amato, Sonia Goncalves, Ewan Harrison, David K. Jackson, Ian Johnston, Dominic Kwiatkowski, Cordelia Langford, John Sillitoe on behalf of the Wellcome Sanger Institute COVID-19 Surveillance Team
EPI_ISL_633376, EPI_ISL_633377	Lighthouse Lab in Glasgow	Wellcome Sanger Institute for the COVID-19 Genomics UK (COG-UK) consortium	Harper VanSteenhouse, Yumi Kasai, David Gray, Carol Clugston, Anna Dominiczak and Alex Alderton, Roberto Amato, Sonia Goncalves, Ewan Harrison, David K. Jackson, Ian Johnston, Dominic Kwiatkowski, Cordelia Langford, John Sillitoe on behalf of the Wellcome Sanger Institute COVID-19 Surveillance Team
EPI_ISL_633378	Lighthouse Lab in Cambridge	Wellcome Sanger Institute for the COVID-19 Genomics UK (COG-UK) consortium	Rob Howes, The Lighthouse Lab in Cambridge and Alex Alderton, Roberto Amato, Sonia Goncalves, Ewan Harrison, David K. Jackson, Ian Johnston, Dominic Kwiatkowski, Cordelia Langford, John Sillitoe on behalf of the Wellcome Sanger Institute COVID-19 Surveillance Team
EPI_ISL_633379	Lighthouse Lab in Glasgow	Wellcome Sanger Institute for the COVID-19 Genomics UK (COG-UK) consortium	Harper VanSteenhouse, Yumi Kasai, David Gray, Carol Clugston, Anna Dominiczak and Alex Alderton, Roberto Amato, Sonia Goncalves, Ewan Harrison, David K. Jackson, Ian Johnston, Dominic Kwiatkowski, Cordelia Langford, John Sillitoe on behalf of the Wellcome Sanger Institute COVID-19 Surveillance Team
EPI_ISL_633380	Lighthouse Lab in Cambridge	Wellcome Sanger Institute for the COVID-19 Genomics UK (COG-UK) consortium	Rob Howes, The Lighthouse Lab in Cambridge and Alex Alderton, Roberto Amato, Sonia Goncalves, Ewan Harrison, David K. Jackson, Ian Johnston, Dominic Kwiatkowski, Cordelia Langford, John Sillitoe on behalf of the Wellcome Sanger Institute COVID-19 Surveillance Team
EPI_ISL_633381, EPI_ISL_633382	Lighthouse Lab in Glasgow	Wellcome Sanger Institute for the COVID-19 Genomics UK	Harper VanSteenhouse, Yumi Kasai, David Gray, Carol Clugston, Anna Dominiczak and Alex Alderton, Roberto Amato, Sonia Goncalves, Ewan Harrison,

		(COG-UK) consortium	David K. Jackson, Ian Johnston, Dominic Kwiatkowski, Cordelia Langford, John Sillitoe on behalf of the Wellcome Sanger Institute COVID-19 Surveillance Team
EPI_ISL_633383	Lighthouse Lab in Cambridge	Wellcome Sanger Institute for the COVID-19 Genomics UK (COG-UK) consortium	Rob Howes, The Lighthouse Lab in Cambridge and Alex Alderton, Roberto Amato, Sonia Goncalves, Ewan Harrison, David K. Jackson, Ian Johnston, Dominic Kwiatkowski, Cordelia Langford, John Sillitoe on behalf of the Wellcome Sanger Institute COVID-19 Surveillance Team
EPI_ISL_633384	Lighthouse Lab in Glasgow	Wellcome Sanger Institute for the COVID-19 Genomics UK (COG-UK) consortium	Harper VanSteenhouse, Yumi Kasai, David Gray, Carol Clugston, Anna Dominiczak and Alex Alderton, Roberto Amato, Sonia Goncalves, Ewan Harrison, David K. Jackson, Ian Johnston, Dominic Kwiatkowski, Cordelia Langford, John Sillitoe on behalf of the Wellcome Sanger Institute COVID-19 Surveillance Team
EPI_ISL_633385	Lighthouse Lab in Cambridge	Wellcome Sanger Institute for the COVID-19 Genomics UK (COG-UK) consortium	Rob Howes, The Lighthouse Lab in Cambridge and Alex Alderton, Roberto Amato, Sonia Goncalves, Ewan Harrison, David K. Jackson, Ian Johnston, Dominic Kwiatkowski, Cordelia Langford, John Sillitoe on behalf of the Wellcome Sanger Institute COVID-19 Surveillance Team
EPI_ISL_633386, EPI_ISL_633387	Lighthouse Lab in Glasgow	Wellcome Sanger Institute for the COVID-19 Genomics UK (COG-UK) consortium	Harper VanSteenhouse, Yumi Kasai, David Gray, Carol Clugston, Anna Dominiczak and Alex Alderton, Roberto Amato, Sonia Goncalves, Ewan Harrison, David K. Jackson, Ian Johnston, Dominic Kwiatkowski, Cordelia Langford, John Sillitoe on behalf of the Wellcome Sanger Institute COVID-19 Surveillance Team
EPI_ISL_633388, EPI_ISL_633389, EPI_ISL_633390	Lighthouse Lab in Cambridge	Wellcome Sanger Institute for the COVID-19 Genomics UK (COG-UK) consortium	Rob Howes, The Lighthouse Lab in Cambridge and Alex Alderton, Roberto Amato, Sonia Goncalves, Ewan Harrison, David K. Jackson, Ian Johnston, Dominic Kwiatkowski, Cordelia Langford, John Sillitoe on behalf of the Wellcome Sanger Institute COVID-19 Surveillance Team
EPI_ISL_633391, EPI_ISL_633392	Lighthouse Lab in Glasgow	Wellcome Sanger Institute for the COVID-19 Genomics UK (COG-UK) consortium	Harper VanSteenhouse, Yumi Kasai, David Gray, Carol Clugston, Anna Dominiczak and Alex Alderton, Roberto Amato, Sonia Goncalves, Ewan Harrison, David K. Jackson, Ian Johnston, Dominic Kwiatkowski, Cordelia Langford, John Sillitoe on behalf of the Wellcome Sanger Institute COVID-19 Surveillance Team
EPI_ISL_633393	Lighthouse Lab in Cambridge	Wellcome Sanger Institute for the COVID-19 Genomics UK (COG-UK) consortium	Rob Howes, The Lighthouse Lab in Cambridge and Alex Alderton, Roberto Amato, Sonia Goncalves, Ewan Harrison, David K. Jackson, Ian Johnston, Dominic Kwiatkowski, Cordelia Langford, John Sillitoe on behalf of the Wellcome Sanger Institute COVID-19 Surveillance Team
EPI_ISL_633394	Lighthouse Lab in Glasgow	Wellcome Sanger Institute for the COVID-19 Genomics UK (COG-UK) consortium	Harper VanSteenhouse, Yumi Kasai, David Gray, Carol Clugston, Anna Dominiczak and Alex Alderton, Roberto Amato, Sonia Goncalves, Ewan Harrison, David K. Jackson, Ian Johnston, Dominic Kwiatkowski, Cordelia Langford, John Sillitoe on behalf of the Wellcome Sanger Institute COVID-19 Surveillance Team
EPI_ISL_633395	Lighthouse Lab in Cambridge	Wellcome Sanger Institute for the COVID-19 Genomics UK (COG-UK) consortium	Rob Howes, The Lighthouse Lab in Cambridge and Alex Alderton, Roberto Amato, Sonia Goncalves, Ewan Harrison, David K. Jackson, Ian Johnston, Dominic Kwiatkowski, Cordelia Langford, John Sillitoe on behalf of the Wellcome Sanger Institute COVID-19 Surveillance Team
EPI_ISL_633396	Lighthouse Lab in Glasgow	Wellcome Sanger Institute for the COVID-19 Genomics UK (COG-UK) consortium	Harper VanSteenhouse, Yumi Kasai, David Gray, Carol Clugston, Anna Dominiczak and Alex Alderton, Roberto Amato, Sonia Goncalves, Ewan Harrison, David K. Jackson, Ian Johnston, Dominic Kwiatkowski, Cordelia Langford, John Sillitoe on behalf of the Wellcome Sanger Institute COVID-19 Surveillance Team
EPI_ISL_633397, EPI_ISL_633398, EPI_ISL_633399	Lighthouse Lab in Cambridge	Wellcome Sanger Institute for the COVID-19 Genomics UK (COG-UK) consortium	Rob Howes, The Lighthouse Lab in Cambridge and Alex Alderton, Roberto Amato, Sonia Goncalves, Ewan Harrison, David K. Jackson, Ian Johnston, Dominic Kwiatkowski, Cordelia Langford, John Sillitoe on behalf of the Wellcome Sanger Institute COVID-19 Surveillance Team
EPI_ISL_633400, EPI_ISL_633401, EPI_ISL_633402	Lighthouse Lab in Glasgow	Wellcome Sanger Institute for the COVID-19 Genomics UK (COG-UK) consortium	Harper VanSteenhouse, Yumi Kasai, David Gray, Carol Clugston, Anna Dominiczak and Alex Alderton, Roberto Amato, Sonia Goncalves, Ewan Harrison, David K. Jackson, Ian Johnston, Dominic Kwiatkowski, Cordelia Langford, John Sillitoe on behalf of the Wellcome Sanger Institute COVID-19 Surveillance Team
EPI_ISL_633403	Lighthouse Lab in Cambridge	Wellcome Sanger Institute for the COVID-19 Genomics UK (COG-UK) consortium	Rob Howes, The Lighthouse Lab in Cambridge and Alex Alderton, Roberto Amato, Sonia Goncalves, Ewan Harrison, David K. Jackson, Ian Johnston, Dominic Kwiatkowski, Cordelia Langford, John Sillitoe on behalf of the Wellcome Sanger Institute COVID-19 Surveillance Team
EPI_ISL_633404	Lighthouse Lab in Glasgow	Wellcome Sanger Institute for the COVID-19 Genomics UK (COG-UK) consortium	Harper VanSteenhouse, Yumi Kasai, David Gray, Carol Clugston, Anna Dominiczak and Alex Alderton, Roberto Amato, Sonia Goncalves, Ewan Harrison, David K. Jackson, Ian Johnston, Dominic Kwiatkowski, Cordelia Langford, John Sillitoe on behalf of the Wellcome Sanger Institute COVID-19 Surveillance Team
EPI_ISL_633405	Lighthouse Lab in Cambridge	Wellcome Sanger Institute for the COVID-19 Genomics UK (COG-UK) consortium	Rob Howes, The Lighthouse Lab in Cambridge and Alex Alderton, Roberto Amato, Sonia Goncalves, Ewan Harrison, David K. Jackson, Ian Johnston, Dominic Kwiatkowski, Cordelia Langford, John Sillitoe on behalf of the Wellcome Sanger Institute COVID-19 Surveillance Team
EPI_ISL_633406	Lighthouse Lab in Glasgow	Wellcome Sanger Institute for the COVID-19 Genomics UK (COG-UK) consortium	Harper VanSteenhouse, Yumi Kasai, David Gray, Carol Clugston, Anna Dominiczak and Alex Alderton, Roberto Amato, Sonia Goncalves, Ewan Harrison, David K. Jackson, Ian Johnston, Dominic Kwiatkowski, Cordelia Langford, John Sillitoe on behalf of the Wellcome Sanger Institute COVID-19 Surveillance Team
EPI_ISL_633407, EPI_ISL_633408	Lighthouse Lab in Cambridge	Wellcome Sanger Institute for the COVID-19 Genomics UK (COG-UK) consortium	Rob Howes, The Lighthouse Lab in Cambridge and Alex Alderton, Roberto Amato, Sonia Goncalves, Ewan Harrison, David K. Jackson, Ian Johnston, Dominic Kwiatkowski, Cordelia Langford, John Sillitoe on behalf of the Wellcome Sanger Institute COVID-19 Surveillance Team
EPI_ISL_633409, EPI_ISL_633410, EPI_ISL_633411, EPI_ISL_633412, EPI_ISL_633413, EPI_ISL_633414, EPI_ISL_633415, EPI_ISL_633416, EPI_ISL_633417, EPI_ISL_633418, EPI_ISL_633419, EPI_ISL_633420, EPI_ISL_633421, EPI_ISL_633422, EPI_ISL_633423, EPI_ISL_633424, EPI_ISL_633425, EPI_ISL_633426, EPI_ISL_633427, EPI_ISL_633428, EPI_ISL_633429, EPI_ISL_633430, EPI_ISL_633431, EPI_ISL_633432, EPI_ISL_633433, EPI_ISL_633434, EPI_ISL_633435, EPI_ISL_633436, EPI_ISL_633437, EPI_ISL_633438, EPI_ISL_633439, EPI_ISL_633440, EPI_ISL_633441, EPI_ISL_633442, EPI_ISL_633443, EPI_ISL_633444, EPI_ISL_633445, EPI_ISL_633446, EPI_ISL_633447, EPI_ISL_633448, EPI_ISL_633449, EPI_ISL_633450, EPI_ISL_633451, EPI_ISL_633452, EPI_ISL_633453, EPI_ISL_633454, EPI_ISL_633455, EPI_ISL_633456, EPI_ISL_633457, EPI_ISL_633458, EPI_ISL_633459, EPI_ISL_633460, EPI_ISL_633461, EPI_ISL_633462, EPI_ISL_633463, EPI_ISL_633464, EPI_ISL_633465, EPI_ISL_633466, EPI_ISL_633467, EPI_ISL_633468, EPI_ISL_633469, EPI_ISL_633470, EPI_ISL_633471, EPI_ISL_633472, EPI_ISL_633473, EPI_ISL_633474, EPI_ISL_633475, EPI_ISL_633476, EPI_ISL_633477, EPI_ISL_633478, EPI_ISL_633479, EPI_ISL_633480, EPI_ISL_633481, EPI_ISL_633482, EPI_ISL_633483, EPI_ISL_633484, EPI_ISL_633485, EPI_ISL_633486, EPI_ISL_633487, EPI_ISL_633488, EPI_ISL_633489, EPI_ISL_633490, EPI_ISL_633491			
see above	Lighthouse Lab in Glasgow	Wellcome Sanger Institute for the COVID-19 Genomics UK (COG-UK) consortium	Harper VanSteenhouse, Yumi Kasai, David Gray, Carol Clugston, Anna Dominiczak and Alex Alderton, Roberto Amato, Sonia Goncalves, Ewan Harrison, David K. Jackson, Ian Johnston, Dominic Kwiatkowski, Cordelia Langford, John Sillitoe on behalf of the Wellcome Sanger Institute COVID-19 Surveillance Team
EPI_ISL_633492	Lighthouse Lab in Glasgow	Wellcome Sanger Institute for the COVID-19 Genomics UK (COG-UK) Consortium	Harper VanSteenhouse, Yumi Kasai, David Gray, Carol Clugston, Anna Dominiczak and Alex Alderton, Roberto Amato, Sonia Goncalves, Ewan Harrison, David K. Jackson, Ian Johnston, Dominic Kwiatkowski, Cordelia Langford, John Sillitoe on behalf of the Wellcome Sanger Institute COVID-19 Surveillance Team
EPI_ISL_633493, EPI_ISL_633494, EPI_ISL_633495, EPI_ISL_633496, EPI_ISL_633497, EPI_ISL_633498, EPI_ISL_633499, EPI_ISL_633500, EPI_ISL_633501, EPI_ISL_633502, EPI_ISL_633503, EPI_ISL_633504, EPI_ISL_633505, EPI_ISL_633506, EPI_ISL_633507, EPI_ISL_633508, EPI_ISL_633509, EPI_ISL_633510, EPI_ISL_633511, EPI_ISL_633512, EPI_ISL_633513, EPI_ISL_633514, EPI_ISL_633515, EPI_ISL_633516, EPI_ISL_633517, EPI_ISL_633518, EPI_ISL_633519, EPI_ISL_633520, EPI_ISL_633521, EPI_ISL_633522, EPI_ISL_633523, EPI_ISL_633524, EPI_ISL_633525, EPI_ISL_633526, EPI_ISL_633527, EPI_ISL_633528, EPI_ISL_633529, EPI_ISL_633530, EPI_ISL_633531, EPI_ISL_633532, EPI_ISL_633533, EPI_ISL_633534, EPI_ISL_633535, EPI_ISL_633536, EPI_ISL_633537, EPI_ISL_633538, EPI_ISL_633539, EPI_ISL_633540, EPI_ISL_633541, EPI_ISL_633542, EPI_ISL_633543, EPI_ISL_633544, EPI_ISL_633545, EPI_ISL_633546, EPI_ISL_633547, EPI_ISL_633548, EPI_ISL_633549, EPI_ISL_633550, EPI_ISL_633551, EPI_ISL_633552, EPI_ISL_633553, EPI_ISL_633554, EPI_ISL_633555, EPI_ISL_633556, EPI_ISL_633557, EPI_ISL_633558, EPI_ISL_633559, EPI_ISL_633560, EPI_ISL_633561, EPI_ISL_633562, EPI_ISL_633563, EPI_ISL_633564, EPI_ISL_633565, EPI_ISL_633566, EPI_ISL_633567, EPI_ISL_633568, EPI_ISL_633569, EPI_ISL_633570, EPI_ISL_633571, EPI_ISL_633572, EPI_ISL_633573, EPI_ISL_633574, EPI_ISL_633575, EPI_ISL_633576			
see above	Lighthouse Lab in Glasgow	Wellcome Sanger Institute for the COVID-19 Genomics UK (COG-UK) consortium	Harper VanSteenhouse, Yumi Kasai, David Gray, Carol Clugston, Anna Dominiczak and Alex Alderton, Roberto Amato, Sonia Goncalves, Ewan Harrison, David K. Jackson, Ian Johnston, Dominic Kwiatkowski, Cordelia Langford, John Sillitoe on behalf of the Wellcome Sanger Institute COVID-19 Surveillance Team
EPI_ISL_633577	Lighthouse Lab in Glasgow	Wellcome Sanger Institute for the COVID-19 Genomics UK (COG-UK) Consortium	Harper VanSteenhouse, Yumi Kasai, David Gray, Carol Clugston, Anna Dominiczak and Alex Alderton, Roberto Amato, Sonia Goncalves, Ewan Harrison, David K. Jackson, Ian Johnston, Dominic Kwiatkowski, Cordelia Langford, John Sillitoe on behalf of the Wellcome Sanger Institute COVID-19 Surveillance Team
EPI_ISL_633578, EPI_ISL_633579, EPI_ISL_633580, EPI_ISL_633581, EPI_ISL_633582, EPI_ISL_633583, EPI_ISL_633584, EPI_ISL_633585, EPI_ISL_633586, EPI_ISL_633587, EPI_ISL_633588, EPI_ISL_633589, EPI_ISL_633590, EPI_ISL_633591, EPI_ISL_633592, EPI_ISL_633593, EPI_ISL_633594, EPI_ISL_633595, EPI_ISL_633596, EPI_ISL_633597, EPI_ISL_633598, EPI_ISL_633599, EPI_ISL_633600, EPI_ISL_633601, EPI_ISL_633602, EPI_ISL_633603, EPI_ISL_633604, EPI_ISL_633605, EPI_ISL_633606, EPI_ISL_633607, EPI_ISL_633608, EPI_ISL_633609, EPI_ISL_633610, EPI_ISL_633611, EPI_ISL_633612, EPI_ISL_633613, EPI_ISL_633614, EPI_ISL_633616, EPI_ISL_633617, EPI_ISL_633618, EPI_ISL_633619, EPI_ISL_633620, EPI_ISL_633621, EPI_ISL_633622, EPI_ISL_633623, EPI_ISL_633624, EPI_ISL_633625, EPI_ISL_633626, EPI_ISL_633627, EPI_ISL_633628, EPI_ISL_633629, EPI_ISL_633630, EPI_ISL_633631, EPI_ISL_633632, EPI_ISL_633633, EPI_ISL_633634, EPI_ISL_633635, EPI_ISL_633636, EPI_ISL_633637, EPI_ISL_633638, EPI_ISL_633639, EPI_ISL_633640, EPI_ISL_633641, EPI_ISL_633642, EPI_ISL_633643, EPI_ISL_633644, EPI_ISL_633645, EPI_ISL_633646, EPI_ISL_633647, EPI_ISL_633648, EPI_ISL_633649, EPI_ISL_633650, EPI_ISL_633651, EPI_ISL_633652			
see above	Lighthouse Lab in Glasgow	Wellcome Sanger Institute for the COVID-19 Genomics UK (COG-UK) consortium	Harper VanSteenhouse, Yumi Kasai, David Gray, Carol Clugston, Anna Dominiczak and Alex Alderton, Roberto Amato, Sonia Goncalves, Ewan Harrison, David K. Jackson, Ian Johnston, Dominic Kwiatkowski, Cordelia Langford, John Sillitoe on behalf of the Wellcome Sanger Institute COVID-19 Surveillance Team

EPI_ISL_633653	Lighthouse Lab in Glasgow	Wellcome Sanger Institute for the COVID-19 Genomics UK (COG-UK) Consortium	Harper VanSteenhouse, Yumi Kasai, David Gray, Carol Clugston, Anna Dominiczak and Alex Alderton, Roberto Amato, Sonia Goncalves, Ewan Harrison, David K. Jackson, Ian Johnston, Dominic Kwiatkowski, Cordelia Langford, John Sillitoe on behalf of the Wellcome Sanger Institute COVID-19 Surveillance Team
EPI_ISL_633654, EPI_ISL_633655, EPI_ISL_633656, EPI_ISL_633657, EPI_ISL_633658, EPI_ISL_633659, EPI_ISL_633660, EPI_ISL_633661, EPI_ISL_633662, EPI_ISL_633663, EPI_ISL_633664, EPI_ISL_633665, EPI_ISL_633666, EPI_ISL_633667, EPI_ISL_633668, EPI_ISL_633669, EPI_ISL_633670, EPI_ISL_633671, EPI_ISL_633672, EPI_ISL_633673, EPI_ISL_633674, EPI_ISL_633675, EPI_ISL_633676, EPI_ISL_633677, EPI_ISL_633678, EPI_ISL_633679, EPI_ISL_633680, EPI_ISL_633681, EPI_ISL_633682, EPI_ISL_633683, EPI_ISL_633684, EPI_ISL_633685, EPI_ISL_633686, EPI_ISL_633687, EPI_ISL_633688, EPI_ISL_633689, EPI_ISL_633690, EPI_ISL_633691, EPI_ISL_633692, EPI_ISL_633693, EPI_ISL_633694, EPI_ISL_633695, EPI_ISL_633697, EPI_ISL_633698, EPI_ISL_633699			
see above	Lighthouse Lab in Glasgow	Wellcome Sanger Institute for the COVID-19 Genomics UK (COG-UK) consortium	Harper VanSteenhouse, Yumi Kasai, David Gray, Carol Clugston, Anna Dominiczak and Alex Alderton, Roberto Amato, Sonia Goncalves, Ewan Harrison, David K. Jackson, Ian Johnston, Dominic Kwiatkowski, Cordelia Langford, John Sillitoe on behalf of the Wellcome Sanger Institute COVID-19 Surveillance Team
EPI_ISL_633700	Lighthouse Lab in Glasgow	Wellcome Sanger Institute for the COVID-19 Genomics UK (COG-UK) Consortium	Harper VanSteenhouse, Yumi Kasai, David Gray, Carol Clugston, Anna Dominiczak and Alex Alderton, Roberto Amato, Sonia Goncalves, Ewan Harrison, David K. Jackson, Ian Johnston, Dominic Kwiatkowski, Cordelia Langford, John Sillitoe on behalf of the Wellcome Sanger Institute COVID-19 Surveillance Team
EPI_ISL_633701, EPI_ISL_633702, EPI_ISL_633703, EPI_ISL_633704, EPI_ISL_633705, EPI_ISL_633706, EPI_ISL_633707, EPI_ISL_633708, EPI_ISL_633709, EPI_ISL_633710, EPI_ISL_633711, EPI_ISL_633712, EPI_ISL_633713, EPI_ISL_633714, EPI_ISL_633715, EPI_ISL_633716, EPI_ISL_633717, EPI_ISL_633718, EPI_ISL_633719, EPI_ISL_633720, EPI_ISL_633721, EPI_ISL_633722, EPI_ISL_633723, EPI_ISL_633724, EPI_ISL_633725, EPI_ISL_633726, EPI_ISL_633727, EPI_ISL_633728, EPI_ISL_633729, EPI_ISL_633730, EPI_ISL_633731, EPI_ISL_633732, EPI_ISL_633733, EPI_ISL_633734, EPI_ISL_633735, EPI_ISL_633736, EPI_ISL_633737, EPI_ISL_633738, EPI_ISL_633739, EPI_ISL_633740, EPI_ISL_633741, EPI_ISL_633742, EPI_ISL_633743, EPI_ISL_633744, EPI_ISL_633745, EPI_ISL_633746, EPI_ISL_633747, EPI_ISL_633748, EPI_ISL_633749, EPI_ISL_633750, EPI_ISL_633751, EPI_ISL_633752, EPI_ISL_633753, EPI_ISL_633754, EPI_ISL_633755, EPI_ISL_633756, EPI_ISL_633757, EPI_ISL_633758, EPI_ISL_633759			
see above	Lighthouse Lab in Glasgow	Wellcome Sanger Institute for the COVID-19 Genomics UK (COG-UK) consortium	Harper VanSteenhouse, Yumi Kasai, David Gray, Carol Clugston, Anna Dominiczak and Alex Alderton, Roberto Amato, Sonia Goncalves, Ewan Harrison, David K. Jackson, Ian Johnston, Dominic Kwiatkowski, Cordelia Langford, John Sillitoe on behalf of the Wellcome Sanger Institute COVID-19 Surveillance Team
EPI_ISL_633760	Lighthouse Lab in Glasgow	Wellcome Sanger Institute for the COVID-19 Genomics UK (COG-UK) Consortium	Harper VanSteenhouse, Yumi Kasai, David Gray, Carol Clugston, Anna Dominiczak and Alex Alderton, Roberto Amato, Sonia Goncalves, Ewan Harrison, David K. Jackson, Ian Johnston, Dominic Kwiatkowski, Cordelia Langford, John Sillitoe on behalf of the Wellcome Sanger Institute COVID-19 Surveillance Team
EPI_ISL_633761, EPI_ISL_633762, EPI_ISL_633763, EPI_ISL_633764, EPI_ISL_633765, EPI_ISL_633766, EPI_ISL_633767, EPI_ISL_633768, EPI_ISL_633769, EPI_ISL_633770, EPI_ISL_633771, EPI_ISL_633772, EPI_ISL_633773, EPI_ISL_633774, EPI_ISL_633775, EPI_ISL_633776, EPI_ISL_633777, EPI_ISL_633778, EPI_ISL_633779, EPI_ISL_633780, EPI_ISL_633781			
see above	Lighthouse Lab in Glasgow	Wellcome Sanger Institute for the COVID-19 Genomics UK (COG-UK) consortium	Harper VanSteenhouse, Yumi Kasai, David Gray, Carol Clugston, Anna Dominiczak and Alex Alderton, Roberto Amato, Sonia Goncalves, Ewan Harrison, David K. Jackson, Ian Johnston, Dominic Kwiatkowski, Cordelia Langford, John Sillitoe on behalf of the Wellcome Sanger Institute COVID-19 Surveillance Team
EPI_ISL_633782	Lighthouse Lab in Glasgow	Wellcome Sanger Institute for the COVID-19 Genomics UK (COG-UK) Consortium	Harper VanSteenhouse, Yumi Kasai, David Gray, Carol Clugston, Anna Dominiczak and Alex Alderton, Roberto Amato, Sonia Goncalves, Ewan Harrison, David K. Jackson, Ian Johnston, Dominic Kwiatkowski, Cordelia Langford, John Sillitoe on behalf of the Wellcome Sanger Institute COVID-19 Surveillance Team
EPI_ISL_633783, EPI_ISL_633784, EPI_ISL_633785, EPI_ISL_633786, EPI_ISL_633787, EPI_ISL_633788, EPI_ISL_633789, EPI_ISL_633790, EPI_ISL_633791, EPI_ISL_633792, EPI_ISL_633793, EPI_ISL_633795, EPI_ISL_633796, EPI_ISL_633797, EPI_ISL_633798			
see above	Lighthouse Lab in Glasgow	Wellcome Sanger Institute for the COVID-19 Genomics UK (COG-UK) consortium	Harper VanSteenhouse, Yumi Kasai, David Gray, Carol Clugston, Anna Dominiczak and Alex Alderton, Roberto Amato, Sonia Goncalves, Ewan Harrison, David K. Jackson, Ian Johnston, Dominic Kwiatkowski, Cordelia Langford, John Sillitoe on behalf of the Wellcome Sanger Institute COVID-19 Surveillance Team
EPI_ISL_633799	Lighthouse Lab in Glasgow	Wellcome Sanger Institute for the COVID-19 Genomics UK (COG-UK) Consortium	Harper VanSteenhouse, Yumi Kasai, David Gray, Carol Clugston, Anna Dominiczak and Alex Alderton, Roberto Amato, Sonia Goncalves, Ewan Harrison, David K. Jackson, Ian Johnston, Dominic Kwiatkowski, Cordelia Langford, John Sillitoe on behalf of the Wellcome Sanger Institute COVID-19 Surveillance Team
EPI_ISL_633801, EPI_ISL_633802, EPI_ISL_633803, EPI_ISL_633804, EPI_ISL_633805, EPI_ISL_633806, EPI_ISL_633807, EPI_ISL_633808, EPI_ISL_633809, EPI_ISL_633810, EPI_ISL_633811, EPI_ISL_633812, EPI_ISL_633813, EPI_ISL_633814, EPI_ISL_633815, EPI_ISL_633816, EPI_ISL_633817, EPI_ISL_633818, EPI_ISL_633819, EPI_ISL_633820, EPI_ISL_633822, EPI_ISL_633823, EPI_ISL_633824, EPI_ISL_633826, EPI_ISL_633827, EPI_ISL_633828, EPI_ISL_633829, EPI_ISL_633830, EPI_ISL_633831, EPI_ISL_633832, EPI_ISL_633834, EPI_ISL_633835, EPI_ISL_633836, EPI_ISL_633837, EPI_ISL_633838, EPI_ISL_633839, EPI_ISL_633840, EPI_ISL_633841, EPI_ISL_633842, EPI_ISL_633843, EPI_ISL_633844, EPI_ISL_633845, EPI_ISL_633846, EPI_ISL_633847, EPI_ISL_633848, EPI_ISL_633849, EPI_ISL_633850, EPI_ISL_633852, EPI_ISL_633853, EPI_ISL_633854, EPI_ISL_633855, EPI_ISL_633856, EPI_ISL_633857, EPI_ISL_633858, EPI_ISL_633859, EPI_ISL_633860, EPI_ISL_633861, EPI_ISL_633862, EPI_ISL_633863, EPI_ISL_633864, EPI_ISL_633865, EPI_ISL_633866, EPI_ISL_633867, EPI_ISL_633868, EPI_ISL_633869, EPI_ISL_633870, EPI_ISL_633871, EPI_ISL_633872, EPI_ISL_633873, EPI_ISL_633874, EPI_ISL_633875, EPI_ISL_633876, EPI_ISL_633877, EPI_ISL_633878, EPI_ISL_633879, EPI_ISL_633880, EPI_ISL_633881, EPI_ISL_633882, EPI_ISL_633883, EPI_ISL_633884, EPI_ISL_633885, EPI_ISL_633886, EPI_ISL_633887, EPI_ISL_633888, EPI_ISL_633889, EPI_ISL_633890, EPI_ISL_633891, EPI_ISL_633892, EPI_ISL_633893, EPI_ISL_633894, EPI_ISL_633895, EPI_ISL_633896, EPI_ISL_633897, EPI_ISL_633898, EPI_ISL_633899, EPI_ISL_633900, EPI_ISL_633901, EPI_ISL_633902, EPI_ISL_633903, EPI_ISL_633904, EPI_ISL_633905, EPI_ISL_633906, EPI_ISL_633907, EPI_ISL_633908, EPI_ISL_633909, EPI_ISL_633910, EPI_ISL_633911, EPI_ISL_633912, EPI_ISL_633913, EPI_ISL_633914, EPI_ISL_633915, EPI_ISL_633916, EPI_ISL_633917, EPI_ISL_633918, EPI_ISL_633919, EPI_ISL_633920, EPI_ISL_633921, EPI_ISL_633922, EPI_ISL_633924, EPI_ISL_633925, EPI_ISL_633926, EPI_ISL_633927, EPI_ISL_633928, EPI_ISL_633929, EPI_ISL_633930, EPI_ISL_633931, EPI_ISL_633932, EPI_ISL_633933, EPI_ISL_633934, EPI_ISL_633935, EPI_ISL_633936, EPI_ISL_633937, EPI_ISL_633938, EPI_ISL_633939, EPI_ISL_633940, EPI_ISL_633941, EPI_ISL_633942, EPI_ISL_633943, EPI_ISL_633944, EPI_ISL_633945, EPI_ISL_633946, EPI_ISL_633947, EPI_ISL_633948, EPI_ISL_633949, EPI_ISL_633950, EPI_ISL_633951, EPI_ISL_633952, EPI_ISL_633953, EPI_ISL_633954, EPI_ISL_633955, EPI_ISL_633956, EPI_ISL_633957, EPI_ISL_633958, EPI_ISL_633959, EPI_ISL_633960, EPI_ISL_633961, EPI_ISL_633962, EPI_ISL_633963, EPI_ISL_633964, EPI_ISL_633965, EPI_ISL_633966, EPI_ISL_633967, EPI_ISL_633968, EPI_ISL_633969, EPI_ISL_633970, EPI_ISL_633971, EPI_ISL_633972, EPI_ISL_633973, EPI_ISL_633974, EPI_ISL_633975, EPI_ISL_633976, EPI_ISL_633977, EPI_ISL_633978, EPI_ISL_633979, EPI_ISL_633980, EPI_ISL_633981, EPI_ISL_633982, EPI_ISL_633983, EPI_ISL_633984, EPI_ISL_633985, EPI_ISL_633986, EPI_ISL_633987, EPI_ISL_633988, EPI_ISL_633989, EPI_ISL_633990, EPI_ISL_633991, EPI_ISL_633992, EPI_ISL_633993, EPI_ISL_633994, EPI_ISL_633995, EPI_ISL_633996, EPI_ISL_633997, EPI_ISL_633998, EPI_ISL_633999, EPI_ISL_634000, EPI_ISL_634001, EPI_ISL_634002, EPI_ISL_634003, EPI_ISL_634004, EPI_ISL_634005, EPI_ISL_634006, EPI_ISL_634007, EPI_ISL_634008, EPI_ISL_634009, EPI_ISL_634010, EPI_ISL_634011, EPI_ISL_634012, EPI_ISL_634013, EPI_ISL_634014, EPI_ISL_634015, EPI_ISL_634016, EPI_ISL_634017, EPI_ISL_634018, EPI_ISL_634019, EPI_ISL_634020, EPI_ISL_634021, EPI_ISL_634022, EPI_ISL_634023, EPI_ISL_634024, EPI_ISL_634025, EPI_ISL_634026, EPI_ISL_634027, EPI_ISL_634028, EPI_ISL_634029, EPI_ISL_634030, EPI_ISL_634031, EPI_ISL_634032, EPI_ISL_634033, EPI_ISL_634034, EPI_ISL_634035, EPI_ISL_634036, EPI_ISL_634037, EPI_ISL_634038, EPI_ISL_634039, EPI_ISL_634040, EPI_ISL_634041, EPI_ISL_634042, EPI_ISL_634043, EPI_ISL_634044, EPI_ISL_634045, EPI_ISL_634046, EPI_ISL_634047, EPI_ISL_634048, EPI_ISL_634049, EPI_ISL_634050, EPI_ISL_634051, EPI_ISL_634052, EPI_ISL_634053, EPI_ISL_634054, EPI_ISL_634055, EPI_ISL_634056, EPI_ISL_634057, EPI_ISL_634058, EPI_ISL_634059, EPI_ISL_634060, EPI_ISL_634061, EPI_ISL_634062, EPI_ISL_634063, EPI_ISL_634064, EPI_ISL_634065, EPI_ISL_634066, EPI_ISL_634067, EPI_ISL_634068, EPI_ISL_634069, EPI_ISL_634070, EPI_ISL_634071, EPI_ISL_634072, EPI_ISL_634073, EPI_ISL_634074, EPI_ISL_634075, EPI_ISL_634076, EPI_ISL_634077, EPI_ISL_634078, EPI_ISL_634079, EPI_ISL_634080, EPI_ISL_634081, EPI_ISL_634082, EPI_ISL_634083, EPI_ISL_634084, EPI_ISL_634085, EPI_ISL_634086, EPI_ISL_634087, EPI_ISL_634088, EPI_ISL_634089, EPI_ISL_634090, EPI_ISL_634091, EPI_ISL_634093, EPI_ISL_634094, EPI_ISL_634095, EPI_ISL_634096, EPI_ISL_634097, EPI_ISL_634098, EPI_ISL_634099, EPI_ISL_634100, EPI_ISL_634101, EPI_ISL_634102, EPI_ISL_634103, EPI_ISL_634104, EPI_ISL_634105, EPI_ISL_634106, EPI_ISL_634108, EPI_ISL_634109, EPI_ISL_634110, EPI_ISL_634111, EPI_ISL_634112, EPI_ISL_634114, EPI_ISL_634115, EPI_ISL_634116, EPI_ISL_634117, EPI_ISL_634118, EPI_ISL_634119, EPI_ISL_634120, EPI_ISL_634121, EPI_ISL_634122, EPI_ISL_634123, EPI_ISL_634124, EPI_ISL_634125, EPI_ISL_634126, EPI_ISL_634127, EPI_ISL_634128, EPI_ISL_634129, EPI_ISL_634130, EPI_ISL_634131, EPI_ISL_634132, EPI_ISL_634133, EPI_ISL_634134, EPI_ISL_634135, EPI_ISL_634136, EPI_ISL_634137, EPI_ISL_634138, EPI_ISL_634139, EPI_ISL_634140, EPI_ISL_634141, EPI_ISL_634142, EPI_ISL_634143, EPI_ISL_634144, EPI_ISL_634145, EPI_ISL_634146, EPI_ISL_634147, EPI_ISL_634148, EPI_ISL_634149, EPI_ISL_634150, EPI_ISL_634151, EPI_ISL_634152, EPI_ISL_634153, EPI_ISL_634154, EPI_ISL_634155, EPI_ISL_634157, EPI_ISL_634158, EPI_ISL_634159, EPI_ISL_634160, EPI_ISL_634161, EPI_ISL_634162, EPI_ISL_634163, EPI_ISL_634164, EPI_ISL_634165, EPI_ISL_634166, EPI_ISL_634167, EPI_ISL_634168, EPI_ISL_634169, EPI_ISL_634170, EPI_ISL_634171, EPI_ISL_634172, EPI_ISL_634173, EPI_ISL_634174, EPI_ISL_634175, EPI_ISL_634176, EPI_ISL_634177, EPI_ISL_634178, EPI_ISL_634179, EPI_ISL_634180, EPI_ISL_634181, EPI_ISL_634182, EPI_ISL_634183, EPI_ISL_634184, EPI_ISL_634185, EPI_ISL_634186, EPI_ISL_634187, EPI_ISL_634188, EPI_ISL_634189, EPI_ISL_634190, EPI_ISL_634191, EPI_ISL_634192, EPI_ISL_634193, EPI_ISL_634194, EPI_ISL_634195, EPI_ISL_634196, EPI_ISL_634197, EPI_ISL_634198, EPI_ISL_634199, EPI_ISL_634200, EPI_ISL_634201, EPI_ISL_634202, EPI_ISL_634203, EPI_ISL_634204, EPI_ISL_634205, EPI_ISL_634206, EPI_ISL_634207, EPI_ISL_634208, EPI_ISL_634209, EPI_ISL_634210, EPI_ISL_634211, EPI_ISL_634212, EPI_ISL_634213, EPI_ISL_634214, EPI_ISL_634215, EPI_ISL_634216, EPI_ISL_634217, EPI_ISL_634218, EPI_ISL_634219, EPI_ISL_634220, EPI_ISL_634221, EPI_ISL_634222, EPI_ISL_634223, EPI_ISL_634224, EPI_ISL_634225, EPI_ISL_634226, EPI_ISL_634227, EPI_ISL_634228, EPI_ISL_634229, EPI_ISL_634230, EPI_ISL_634231, EPI_ISL_634232, EPI_ISL_634233, EPI_ISL_634234, EPI_ISL_634235, EPI_ISL_634236, EPI_ISL_634237, EPI_ISL_634238, EPI_ISL_634239, EPI_ISL_634240, EPI_ISL_634241, EPI_ISL_634242, EPI_ISL_634243, EPI_ISL_634244, EPI_ISL_634245, EPI_ISL_634246, EPI_ISL_634247, EPI_ISL_634248, EPI_ISL_634249, EPI_ISL_634250, EPI_ISL_634251, EPI_ISL_634252, EPI_ISL_634253, EPI_ISL_634255			
see above	Lighthouse Lab in Glasgow	Wellcome Sanger Institute for the COVID-19 Genomics UK (COG-UK) consortium	Harper VanSteenhouse, Yumi Kasai, David Gray, Carol Clugston, Anna Dominiczak and Alex Alderton, Roberto Amato, Sonia Goncalves, Ewan Harrison, David K. Jackson, Ian Johnston, Dominic Kwiatkowski, Cordelia Langford, John Sillitoe on behalf of the Wellcome Sanger Institute COVID-19 Surveillance Team
EPI_ISL_634256, EPI_ISL_634258, EPI_ISL_634259, EPI_ISL_634260, EPI_ISL_634261, EPI_ISL_634262, EPI_ISL_634263, EPI_ISL_634264, EPI_ISL_634265	Lighthouse Lab in Milton Keynes	Wellcome Sanger Institute for the COVID-19 Genomics UK (COG-UK) consortium	The Lighthouse Lab in Milton Keynes and Alex Alderton, Roberto Amato, Sonia Goncalves, Ewan Harrison, David K. Jackson, Ian Johnston, Dominic Kwiatkowski, Cordelia Langford, John Sillitoe on behalf of the Wellcome Sanger Institute COVID-19 Surveillance Team
EPI_ISL_634266	Lighthouse Lab in Milton Keynes	Wellcome Sanger Institute for the COVID-19 Genomics UK (COG-UK) Consortium	The Lighthouse Lab in Milton Keynes and Alex Alderton, Roberto Amato, Sonia Goncalves, Ewan Harrison, David K. Jackson, Ian Johnston, Dominic Kwiatkowski, Cordelia Langford, John Sillitoe on behalf of the Wellcome Sanger Institute COVID-19 Surveillance Team
EPI_ISL_634267, EPI_ISL_634268, EPI_ISL_634269, EPI_ISL_634270, EPI_ISL_634271, EPI_ISL_634272, EPI_ISL_634273, EPI_ISL_634274, EPI_ISL_634275, EPI_ISL_634276, EPI_ISL_634277, EPI_ISL_634278, EPI_ISL_634279, EPI_ISL_634280, EPI_ISL_634281, EPI_ISL_634282, EPI_ISL_634283, EPI_ISL_634284, EPI_ISL_634285, EPI_ISL_634286, EPI_ISL_634287, EPI_ISL_634288, EPI_ISL_634289, EPI_ISL_634290, EPI_ISL_634291, EPI_ISL_634292, EPI_ISL_634293, EPI_ISL_634294, EPI_ISL_634295, EPI_ISL_634296, EPI_ISL_634297, EPI_ISL_634298, EPI_ISL_634299, EPI_ISL_634300, EPI_ISL_634301, EPI_ISL_634302, EPI_ISL_634303, EPI_ISL_634304, EPI_ISL_634305, EPI_ISL_634306, EPI_ISL_634307, EPI_ISL_634308, EPI_ISL_634309, EPI_ISL_634310, EPI_ISL_634311, EPI_ISL_634312, EPI_ISL_634313, EPI_ISL_634314, EPI_ISL_634315, EPI_ISL_634316, EPI_ISL_634317, EPI_ISL_634318, EPI_ISL_634319, EPI_ISL_634320, EPI_ISL_634321, EPI_ISL_634322, EPI_ISL_634323, EPI_ISL_634324, EPI_ISL_634325, EPI_ISL_634326, EPI_ISL_634327, EPI_ISL_634328, EPI_ISL_634329, EPI_ISL_634330, EPI_ISL_634331, EPI_ISL_634332, EPI_ISL_634333, EPI_ISL_634334, EPI_ISL_634335, EPI_ISL_634336, EPI_ISL_634337, EPI_ISL_634338, EPI_ISL_634339, EPI_ISL_634340			
see above	Lighthouse Lab in Milton Keynes	Wellcome Sanger Institute for the COVID-19 Genomics UK	The Lighthouse Lab in Milton Keynes and Alex Alderton, Roberto Amato, Sonia Goncalves, Ewan Harrison, David K. Jackson, Ian Johnston, Dominic

		(COG-UK) consortium	Kwiatkowski, Cordelia Langford, John Sillitoe on behalf of the Wellcome Sanger Institute COVID-19 Surveillance Team
EPI_ISL_634341	Lighthouse Lab in Milton Keynes	Wellcome Sanger Institute for the COVID-19 Genomics UK (COG-UK) Consortium	The Lighthouse Lab in Milton Keynes and Alex Alderton, Roberto Amato, Sonia Goncalves, Ewan Harrison, David K. Jackson, Ian Johnston, Dominic Kwiatkowski, Cordelia Langford, John Sillitoe on behalf of the Wellcome Sanger Institute COVID-19 Surveillance Team
EPI_ISL_634342, EPI_ISL_634343, EPI_ISL_634344, EPI_ISL_634345, EPI_ISL_634346, EPI_ISL_634347, EPI_ISL_634348, EPI_ISL_634349, EPI_ISL_634350, EPI_ISL_634351, EPI_ISL_634352, EPI_ISL_634353, EPI_ISL_634354, EPI_ISL_634355, EPI_ISL_634356, EPI_ISL_634357, EPI_ISL_634358, EPI_ISL_634359, EPI_ISL_634360, EPI_ISL_634361, EPI_ISL_634362, EPI_ISL_634364, EPI_ISL_634365, EPI_ISL_634366, EPI_ISL_634367, EPI_ISL_634368, EPI_ISL_634369, EPI_ISL_634370, EPI_ISL_634371, EPI_ISL_634372, EPI_ISL_634373, EPI_ISL_634374, EPI_ISL_634375			
see above	Lighthouse Lab in Milton Keynes	Wellcome Sanger Institute for the COVID-19 Genomics UK (COG-UK) consortium	The Lighthouse Lab in Milton Keynes and Alex Alderton, Roberto Amato, Sonia Goncalves, Ewan Harrison, David K. Jackson, Ian Johnston, Dominic Kwiatkowski, Cordelia Langford, John Sillitoe on behalf of the Wellcome Sanger Institute COVID-19 Surveillance Team
EPI_ISL_634376	Lighthouse Lab in Milton Keynes	Wellcome Sanger Institute for the COVID-19 Genomics UK (COG-UK) Consortium	The Lighthouse Lab in Milton Keynes and Alex Alderton, Roberto Amato, Sonia Goncalves, Ewan Harrison, David K. Jackson, Ian Johnston, Dominic Kwiatkowski, Cordelia Langford, John Sillitoe on behalf of the Wellcome Sanger Institute COVID-19 Surveillance Team
EPI_ISL_634377, EPI_ISL_634378, EPI_ISL_634379, EPI_ISL_634380, EPI_ISL_634381, EPI_ISL_634382, EPI_ISL_634383	Lighthouse Lab in Milton Keynes	Wellcome Sanger Institute for the COVID-19 Genomics UK (COG-UK) consortium	The Lighthouse Lab in Milton Keynes and Alex Alderton, Roberto Amato, Sonia Goncalves, Ewan Harrison, David K. Jackson, Ian Johnston, Dominic Kwiatkowski, Cordelia Langford, John Sillitoe on behalf of the Wellcome Sanger Institute COVID-19 Surveillance Team
EPI_ISL_634384, EPI_ISL_634385	Lighthouse Lab in Milton Keynes	Wellcome Sanger Institute for the COVID-19 Genomics UK (COG-UK) Consortium	The Lighthouse Lab in Milton Keynes and Alex Alderton, Roberto Amato, Sonia Goncalves, Ewan Harrison, David K. Jackson, Ian Johnston, Dominic Kwiatkowski, Cordelia Langford, John Sillitoe on behalf of the Wellcome Sanger Institute COVID-19 Surveillance Team
EPI_ISL_634386, EPI_ISL_634387, EPI_ISL_634388, EPI_ISL_634389, EPI_ISL_634390, EPI_ISL_634391, EPI_ISL_634392, EPI_ISL_634393, EPI_ISL_634394, EPI_ISL_634395, EPI_ISL_634396, EPI_ISL_634397, EPI_ISL_634398, EPI_ISL_634399, EPI_ISL_634400, EPI_ISL_634401, EPI_ISL_634402, EPI_ISL_634403, EPI_ISL_634404, EPI_ISL_634405			
see above	Lighthouse Lab in Milton Keynes	Wellcome Sanger Institute for the COVID-19 Genomics UK (COG-UK) consortium	The Lighthouse Lab in Milton Keynes and Alex Alderton, Roberto Amato, Sonia Goncalves, Ewan Harrison, David K. Jackson, Ian Johnston, Dominic Kwiatkowski, Cordelia Langford, John Sillitoe on behalf of the Wellcome Sanger Institute COVID-19 Surveillance Team
EPI_ISL_634406	Lighthouse Lab in Milton Keynes	Wellcome Sanger Institute for the COVID-19 Genomics UK (COG-UK) Consortium	The Lighthouse Lab in Milton Keynes and Alex Alderton, Roberto Amato, Sonia Goncalves, Ewan Harrison, David K. Jackson, Ian Johnston, Dominic Kwiatkowski, Cordelia Langford, John Sillitoe on behalf of the Wellcome Sanger Institute COVID-19 Surveillance Team
EPI_ISL_634407, EPI_ISL_634408, EPI_ISL_634409, EPI_ISL_634410, EPI_ISL_634411, EPI_ISL_634412, EPI_ISL_634413, EPI_ISL_634414, EPI_ISL_634415, EPI_ISL_634416, EPI_ISL_634417, EPI_ISL_634418, EPI_ISL_634419, EPI_ISL_634420, EPI_ISL_634421, EPI_ISL_634422, EPI_ISL_634423, EPI_ISL_634424, EPI_ISL_634425, EPI_ISL_634426, EPI_ISL_634427			
see above	Lighthouse Lab in Milton Keynes	Wellcome Sanger Institute for the COVID-19 Genomics UK (COG-UK) consortium	The Lighthouse Lab in Milton Keynes and Alex Alderton, Roberto Amato, Sonia Goncalves, Ewan Harrison, David K. Jackson, Ian Johnston, Dominic Kwiatkowski, Cordelia Langford, John Sillitoe on behalf of the Wellcome Sanger Institute COVID-19 Surveillance Team
EPI_ISL_634428	Lighthouse Lab in Milton Keynes	Wellcome Sanger Institute for the COVID-19 Genomics UK (COG-UK) Consortium	The Lighthouse Lab in Milton Keynes and Alex Alderton, Roberto Amato, Sonia Goncalves, Ewan Harrison, David K. Jackson, Ian Johnston, Dominic Kwiatkowski, Cordelia Langford, John Sillitoe on behalf of the Wellcome Sanger Institute COVID-19 Surveillance Team
EPI_ISL_634429, EPI_ISL_634430, EPI_ISL_634431, EPI_ISL_634432, EPI_ISL_634433, EPI_ISL_634434, EPI_ISL_634435, EPI_ISL_634436, EPI_ISL_634437, EPI_ISL_634438, EPI_ISL_634439, EPI_ISL_634440, EPI_ISL_634441, EPI_ISL_634442, EPI_ISL_634443, EPI_ISL_634444, EPI_ISL_634445, EPI_ISL_634446, EPI_ISL_634447, EPI_ISL_634448, EPI_ISL_634449, EPI_ISL_634450, EPI_ISL_634451, EPI_ISL_634452, EPI_ISL_634453, EPI_ISL_634454, EPI_ISL_634455, EPI_ISL_634456, EPI_ISL_634457, EPI_ISL_634458, EPI_ISL_634459, EPI_ISL_634460, EPI_ISL_634461, EPI_ISL_634462, EPI_ISL_634463, EPI_ISL_634464			
see above	Lighthouse Lab in Milton Keynes	Wellcome Sanger Institute for the COVID-19 Genomics UK (COG-UK) consortium	The Lighthouse Lab in Milton Keynes and Alex Alderton, Roberto Amato, Sonia Goncalves, Ewan Harrison, David K. Jackson, Ian Johnston, Dominic Kwiatkowski, Cordelia Langford, John Sillitoe on behalf of the Wellcome Sanger Institute COVID-19 Surveillance Team
EPI_ISL_634465	Lighthouse Lab in Milton Keynes	Wellcome Sanger Institute for the COVID-19 Genomics UK (COG-UK) Consortium	The Lighthouse Lab in Milton Keynes and Alex Alderton, Roberto Amato, Sonia Goncalves, Ewan Harrison, David K. Jackson, Ian Johnston, Dominic Kwiatkowski, Cordelia Langford, John Sillitoe on behalf of the Wellcome Sanger Institute COVID-19 Surveillance Team
EPI_ISL_634466, EPI_ISL_634467, EPI_ISL_634468, EPI_ISL_634469, EPI_ISL_634470, EPI_ISL_634471, EPI_ISL_634472, EPI_ISL_634473, EPI_ISL_634474, EPI_ISL_634475, EPI_ISL_634476, EPI_ISL_634477, EPI_ISL_634478, EPI_ISL_634479, EPI_ISL_634480, EPI_ISL_634481, EPI_ISL_634482			
see above	Lighthouse Lab in Milton Keynes	Wellcome Sanger Institute for the COVID-19 Genomics UK (COG-UK) consortium	The Lighthouse Lab in Milton Keynes and Alex Alderton, Roberto Amato, Sonia Goncalves, Ewan Harrison, David K. Jackson, Ian Johnston, Dominic Kwiatkowski, Cordelia Langford, John Sillitoe on behalf of the Wellcome Sanger Institute COVID-19 Surveillance Team
EPI_ISL_634483	Lighthouse Lab in Milton Keynes	Wellcome Sanger Institute for the COVID-19 Genomics UK (COG-UK) Consortium	The Lighthouse Lab in Milton Keynes and Alex Alderton, Roberto Amato, Sonia Goncalves, Ewan Harrison, David K. Jackson, Ian Johnston, Dominic Kwiatkowski, Cordelia Langford, John Sillitoe on behalf of the Wellcome Sanger Institute COVID-19 Surveillance Team
EPI_ISL_634484, EPI_ISL_634485, EPI_ISL_634486, EPI_ISL_634487, EPI_ISL_634488, EPI_ISL_634489, EPI_ISL_634490, EPI_ISL_634491, EPI_ISL_634492, EPI_ISL_634493, EPI_ISL_634494, EPI_ISL_634495, EPI_ISL_634496, EPI_ISL_634497, EPI_ISL_634498			
see above	Lighthouse Lab in Milton Keynes	Wellcome Sanger Institute for the COVID-19 Genomics UK (COG-UK) consortium	The Lighthouse Lab in Milton Keynes and Alex Alderton, Roberto Amato, Sonia Goncalves, Ewan Harrison, David K. Jackson, Ian Johnston, Dominic Kwiatkowski, Cordelia Langford, John Sillitoe on behalf of the Wellcome Sanger Institute COVID-19 Surveillance Team
EPI_ISL_634499	Lighthouse Lab in Milton Keynes	Wellcome Sanger Institute for the COVID-19 Genomics UK (COG-UK) Consortium	The Lighthouse Lab in Milton Keynes and Alex Alderton, Roberto Amato, Sonia Goncalves, Ewan Harrison, David K. Jackson, Ian Johnston, Dominic Kwiatkowski, Cordelia Langford, John Sillitoe on behalf of the Wellcome Sanger Institute COVID-19 Surveillance Team
EPI_ISL_634500, EPI_ISL_634501, EPI_ISL_634502, EPI_ISL_634503, EPI_ISL_634504, EPI_ISL_634505, EPI_ISL_634506, EPI_ISL_634507, EPI_ISL_634508, EPI_ISL_634509, EPI_ISL_634510, EPI_ISL_634511, EPI_ISL_634512, EPI_ISL_634513, EPI_ISL_634514, EPI_ISL_634515, EPI_ISL_634516, EPI_ISL_634517, EPI_ISL_634518, EPI_ISL_634519			
see above	Lighthouse Lab in Milton Keynes	Wellcome Sanger Institute for the COVID-19 Genomics UK (COG-UK) consortium	The Lighthouse Lab in Milton Keynes and Alex Alderton, Roberto Amato, Sonia Goncalves, Ewan Harrison, David K. Jackson, Ian Johnston, Dominic Kwiatkowski, Cordelia Langford, John Sillitoe on behalf of the Wellcome Sanger Institute COVID-19 Surveillance Team
EPI_ISL_634520, EPI_ISL_634521, EPI_ISL_634522, EPI_ISL_634523, EPI_ISL_634524, EPI_ISL_634525, EPI_ISL_634526, EPI_ISL_634527, EPI_ISL_634528, EPI_ISL_634529, EPI_ISL_634530, EPI_ISL_634531, EPI_ISL_634532, EPI_ISL_634533, EPI_ISL_634534, EPI_ISL_634535, EPI_ISL_634536, EPI_ISL_634537, EPI_ISL_634538, EPI_ISL_634539, EPI_ISL_634540, EPI_ISL_634541, EPI_ISL_634542, EPI_ISL_634543, EPI_ISL_634544, EPI_ISL_634545, EPI_ISL_634546, EPI_ISL_634547, EPI_ISL_634548, EPI_ISL_634549, EPI_ISL_634550, EPI_ISL_634551, EPI_ISL_634552, EPI_ISL_634553, EPI_ISL_634554, EPI_ISL_634555, EPI_ISL_634556, EPI_ISL_634557, EPI_ISL_634558, EPI_ISL_634559, EPI_ISL_634560, EPI_ISL_634561, EPI_ISL_634562, EPI_ISL_634563, EPI_ISL_634564, EPI_ISL_634565, EPI_ISL_634566, EPI_ISL_634567, EPI_ISL_634568, EPI_ISL_634569, EPI_ISL_634570, EPI_ISL_634571, EPI_ISL_634572, EPI_ISL_634573, EPI_ISL_634574, EPI_ISL_634575, EPI_ISL_634576, EPI_ISL_634577, EPI_ISL_634578, EPI_ISL_634579, EPI_ISL_634580, EPI_ISL_634581, EPI_ISL_634582, EPI_ISL_634583, EPI_ISL_634584, EPI_ISL_634585, EPI_ISL_634586, EPI_ISL_634587, EPI_ISL_634588, EPI_ISL_634589, EPI_ISL_634591, EPI_ISL_634592, EPI_ISL_634593, EPI_ISL_634594, EPI_ISL_634595, EPI_ISL_634596, EPI_ISL_634597, EPI_ISL_634598, EPI_ISL_634599			
see above	Lighthouse Lab in Glasgow	Wellcome Sanger Institute for the COVID-19 Genomics UK (COG-UK) consortium	Harper VanSteenhouse, Yumi Kasai, David Gray, Carol Clugston, Anna Dominiczak and Alex Alderton, Roberto Amato, Sonia Goncalves, Ewan Harrison, David K. Jackson, Ian Johnston, Dominic Kwiatkowski, Cordelia Langford, John Sillitoe on behalf of the Wellcome Sanger Institute COVID-19 Surveillance Team
EPI_ISL_634600, EPI_ISL_634601, EPI_ISL_634602, EPI_ISL_634603, EPI_ISL_634604, EPI_ISL_634605, EPI_ISL_634606, EPI_ISL_634607, EPI_ISL_634608, EPI_ISL_634609, EPI_ISL_634610, EPI_ISL_634611, EPI_ISL_634612, EPI_ISL_634614, EPI_ISL_634615, EPI_ISL_634616, EPI_ISL_634617, EPI_ISL_634618, EPI_ISL_634619, EPI_ISL_634620, EPI_ISL_634621, EPI_ISL_634622, EPI_ISL_634623, EPI_ISL_634624, EPI_ISL_634625, EPI_ISL_634626, EPI_ISL_634627, EPI_ISL_634628, EPI_ISL_634629, EPI_ISL_634630, EPI_ISL_634631, EPI_ISL_634632, EPI_ISL_634633, EPI_ISL_634634, EPI_ISL_634635, EPI_ISL_634636, EPI_ISL_634637, EPI_ISL_634638, EPI_ISL_634639, EPI_ISL_634640, EPI_ISL_634641, EPI_ISL_634642, EPI_ISL_634643, EPI_ISL_634644, EPI_ISL_634645, EPI_ISL_634646, EPI_ISL_634647, EPI_ISL_634648, EPI_ISL_634649, EPI_ISL_634650, EPI_ISL_634651, EPI_ISL_634652, EPI_ISL_634653, EPI_ISL_634654, EPI_ISL_634655, EPI_ISL_634656, EPI_ISL_634657, EPI_ISL_634658, EPI_ISL_634659, EPI_ISL_634660, EPI_ISL_634661, EPI_ISL_634662, EPI_ISL_634663, EPI_ISL_634664, EPI_ISL_634666, EPI_ISL_634667, EPI_ISL_634668, EPI_ISL_634669, EPI_ISL_634670, EPI_ISL_634671, EPI_ISL_634672, EPI_ISL_634673, EPI_ISL_634674, EPI_ISL_634675, EPI_ISL_634676, EPI_ISL_634677			
see above	Lighthouse Lab in Glasgow	Wellcome Sanger Institute for the COVID-19 Genomics UK (COG-UK) consortium	Harper VanSteenhouse, Yumi Kasai, David Gray, Carol Clugston, Anna Dominiczak and Alex Alderton, Roberto Amato, Sonia Goncalves, Ewan Harrison, David K. Jackson, Ian Johnston, Dominic Kwiatkowski, Cordelia Langford, John Sillitoe on behalf of the Wellcome Sanger Institute COVID-19 Surveillance Team (http://www.sanger.ac.uk/covid-team)
EPI_ISL_634678	Lighthouse Lab in Glasgow	Wellcome Sanger Institute for the COVID-19 Genomics UK (COG-UK) Consortium	Harper VanSteenhouse, Yumi Kasai, David Gray, Carol Clugston, Anna Dominiczak and Alex Alderton, Roberto Amato, Sonia Goncalves, Ewan Harrison, David K. Jackson, Ian Johnston, Dominic Kwiatkowski, Cordelia Langford, John Sillitoe on behalf of the Wellcome Sanger Institute COVID-19 Surveillance Team (http://www.sanger.ac.uk/covid-team)
EPI_ISL_634679, EPI_ISL_634680, EPI_ISL_634681, EPI_ISL_634682, EPI_ISL_634683, EPI_ISL_634684, EPI_ISL_634685, EPI_ISL_634686, EPI_ISL_634687, EPI_ISL_634688, EPI_ISL_634689, EPI_ISL_634690, EPI_ISL_634691, EPI_ISL_634692, EPI_ISL_634693, EPI_ISL_634694, EPI_ISL_634695, EPI_ISL_634696, EPI_ISL_634697, EPI_ISL_634698, EPI_ISL_634699, EPI_ISL_634700, EPI_ISL_634701, EPI_ISL_634702, EPI_ISL_634703, EPI_ISL_634704, EPI_ISL_634705, EPI_ISL_634706, EPI_ISL_634707, EPI_ISL_634708, EPI_ISL_634709, EPI_ISL_634710, EPI_ISL_634711, EPI_ISL_634712, EPI_ISL_634713, EPI_ISL_634714, EPI_ISL_634715, EPI_ISL_634716, EPI_ISL_634717, EPI_ISL_634718, EPI_ISL_634720, EPI_ISL_634721, EPI_ISL_634722, EPI_ISL_634723, EPI_ISL_634724, EPI_ISL_634725, EPI_ISL_634726, EPI_ISL_634727, EPI_ISL_634728, EPI_ISL_634729, EPI_ISL_634730, EPI_ISL_634731, EPI_ISL_634732, EPI_ISL_634733, EPI_ISL_634734, EPI_ISL_634735, EPI_ISL_634736, EPI_ISL_634737, EPI_ISL_634738, EPI_ISL_634739, EPI_ISL_634740, EPI_ISL_634741, EPI_ISL_634742, EPI_ISL_634743, EPI_ISL_634744, EPI_ISL_634745, EPI_ISL_634746, EPI_ISL_634747, EPI_ISL_634748, EPI_ISL_634749, EPI_ISL_634750, EPI_ISL_634751, EPI_ISL_634752, EPI_ISL_634753			
see above	Lighthouse Lab in Glasgow	Wellcome Sanger Institute for the COVID-19 Genomics UK (COG-UK) consortium	Harper VanSteenhouse, Yumi Kasai, David Gray, Carol Clugston, Anna Dominiczak and Alex Alderton, Roberto Amato, Sonia Goncalves, Ewan Harrison, David K. Jackson, Ian Johnston, Dominic Kwiatkowski, Cordelia Langford, John Sillitoe on behalf of the Wellcome Sanger Institute COVID-19 Surveillance

			Team (http://www.sanger.ac.uk/covid-team)
EPI_ISL_634754, EPI_ISL_634755, EPI_ISL_634756, EPI_ISL_634757, EPI_ISL_634758, EPI_ISL_634759, EPI_ISL_634760, EPI_ISL_634761, EPI_ISL_634762, EPI_ISL_634763, EPI_ISL_634764, EPI_ISL_634765, EPI_ISL_634766, EPI_ISL_634767, EPI_ISL_634768, EPI_ISL_634769, EPI_ISL_634770, EPI_ISL_634771, EPI_ISL_634772, EPI_ISL_634773, EPI_ISL_634774, EPI_ISL_634775, EPI_ISL_634776, EPI_ISL_634777, EPI_ISL_634778, EPI_ISL_634779, EPI_ISL_634780, EPI_ISL_634781, EPI_ISL_634782, EPI_ISL_634783, EPI_ISL_634784, EPI_ISL_634785, EPI_ISL_634786, EPI_ISL_634787, EPI_ISL_634788			
see above	Lighthouse Lab in Alderley Park	Wellcome Sanger Institute for the COVID-19 Genomics UK (COG-UK) consortium	Jacquelyn Wynn, Mairead Hyland, The Lighthouse Lab in Alderley Park and Alex Alderton, Roberto Amato, Sonia Goncalves, Ewan Harrison, David K. Jackson, Ian Johnston, Dominic Kwiatkowski, Cordelia Langford, John Sillitoe on behalf of the Wellcome Sanger Institute COVID-19 Surveillance Team (http://www.sanger.ac.uk/covid-team)
EPI_ISL_634790, EPI_ISL_634791, EPI_ISL_634792, EPI_ISL_634793, EPI_ISL_634794, EPI_ISL_634795, EPI_ISL_634796, EPI_ISL_634797, EPI_ISL_634798, EPI_ISL_634799, EPI_ISL_634800, EPI_ISL_634801, EPI_ISL_634802, EPI_ISL_634803, EPI_ISL_634804, EPI_ISL_634805, EPI_ISL_634806, EPI_ISL_634807, EPI_ISL_634808, EPI_ISL_634809, EPI_ISL_634810, EPI_ISL_634811, EPI_ISL_634812, EPI_ISL_634813, EPI_ISL_634814, EPI_ISL_634815, EPI_ISL_634816			
see above	Lighthouse Lab in Cambridge	Wellcome Sanger Institute for the COVID-19 Genomics UK (COG-UK) consortium	Rob Howes, The Lighthouse Lab in Cambridge and Alex Alderton, Roberto Amato, Sonia Goncalves, Ewan Harrison, David K. Jackson, Ian Johnston, Dominic Kwiatkowski, Cordelia Langford, John Sillitoe on behalf of the Wellcome Sanger Institute COVID-19 Surveillance Team (http://www.sanger.ac.uk/covid-team)
EPI_ISL_634838, EPI_ISL_634839, EPI_ISL_634840, EPI_ISL_634841, EPI_ISL_634842, EPI_ISL_634843, EPI_ISL_634844, EPI_ISL_634845, EPI_ISL_634846, EPI_ISL_634847, EPI_ISL_634848, EPI_ISL_634849, EPI_ISL_634850, EPI_ISL_634851, EPI_ISL_634852, EPI_ISL_634853, EPI_ISL_634854, EPI_ISL_634855, EPI_ISL_634856			
see above	Minnesota Department of Health, Public Health Laboratory	Minnesota Department of Health, Public Health Laboratory	Matt Plumb, Jacob Garfin, Alexandra Lorentz, and Xiong Wang
EPI_ISL_634857	Minneapolis VA Health Care System	Minnesota Department of Health, Public Health Laboratory	Matt Plumb, Jacob Garfin, Alexandra Lorentz, and Xiong Wang
EPI_ISL_634858, EPI_ISL_634859, EPI_ISL_634860, EPI_ISL_634861, EPI_ISL_634862, EPI_ISL_634863	Minnesota Department of Health, Public Health Laboratory	Minnesota Department of Health, Public Health Laboratory	Matt Plumb, Jacob Garfin, Alexandra Lorentz, and Xiong Wang
EPI_ISL_634864, EPI_ISL_634865	HealthPartners Central Lab	Minnesota Department of Health, Public Health Laboratory	Matt Plumb, Jacob Garfin, Alexandra Lorentz, and Xiong Wang
EPI_ISL_634866, EPI_ISL_634867, EPI_ISL_634868, EPI_ISL_634869, EPI_ISL_634870, EPI_ISL_634871, EPI_ISL_634872, EPI_ISL_634873, EPI_ISL_634874, EPI_ISL_634875	Minnesota Department of Health, Public Health Laboratory	Minnesota Department of Health, Public Health Laboratory	Matt Plumb, Jacob Garfin, Alexandra Lorentz, and Xiong Wang
EPI_ISL_634877, EPI_ISL_634878, EPI_ISL_634879, EPI_ISL_634880, EPI_ISL_634881, EPI_ISL_634882, EPI_ISL_634883, EPI_ISL_634884, EPI_ISL_634885	Lab voor klinische biologie	Onderzoeksgroep Virologie	Laurens Lambrechts, Nick Vereecke, Marthe Pauwels, Bruno Verhasselt, Linos Vandekerckhove, Hans Nauwynck, Sebastiaan Theuns
EPI_ISL_634886, EPI_ISL_634887, EPI_ISL_634888, EPI_ISL_634889, EPI_ISL_634890, EPI_ISL_634891, EPI_ISL_634892, EPI_ISL_634893, EPI_ISL_634894, EPI_ISL_634895	Lab voor klinische biologie	Onderzoeksgroep Virologie	Nick Vereecke, Laurens Lambrechts, Marthe Pauwels, Bruno Verhasselt, Linos Vandekerckhove, Hans Nauwynck, Sebastiaan Theuns
EPI_ISL_634923, EPI_ISL_634924, EPI_ISL_634925, EPI_ISL_634926, EPI_ISL_634927, EPI_ISL_634928, EPI_ISL_634929, EPI_ISL_634931, EPI_ISL_634932, EPI_ISL_634933	Utah Public Health Laboratory	Utah Public Health Laboratory	Erin L. Young, Kelly F. Oakeson
EPI_ISL_634934, EPI_ISL_634935, EPI_ISL_634937, EPI_ISL_634938, EPI_ISL_634941, EPI_ISL_634942, EPI_ISL_634943, EPI_ISL_634944, EPI_ISL_634946, EPI_ISL_634947, EPI_ISL_634948, EPI_ISL_634949, EPI_ISL_634950, EPI_ISL_634951, EPI_ISL_634952, EPI_ISL_634953, EPI_ISL_634954, EPI_ISL_634955, EPI_ISL_634956, EPI_ISL_634958, EPI_ISL_634959, EPI_ISL_634961, EPI_ISL_634962, EPI_ISL_634964, EPI_ISL_634966, EPI_ISL_634967, EPI_ISL_634968, EPI_ISL_634969, EPI_ISL_634970, EPI_ISL_634971, EPI_ISL_634972, EPI_ISL_634973, EPI_ISL_634975			
see above	Texas Department of State Health Services	Texas Department of State Health Services	Rashmi Tuladhar, Bonnie Oh, Jenny Zhang, Maliha Rahman, Anita Pokharel, Myong Koag, Chung Wang, Rachel Lee, Grace Kubin, Mayela Pedrueza
EPI_ISL_634977	1-Laboratory of Microbiology, National Reference Lab, Charles Nicolle Hospital; 2-University of Tunis ElManar, Faculty of Medicine of Tunis, LR99ES09, Tunis, Tunisia	1-Clinical and Experimental Pharmacology Lab, LR16SP02, National Center of Pharmacovigilance, University of Tunis El Manar, Tunis, Tunisia. 2-Neurodegenerative diseases and psychiatric troubles, LR18SP03, Razi Hospital, University of Tunis El Manar, Tunis, Tunisia. 3- Ministry of Health, National Observatory of New and Emerging Diseases, 1006, Tunis, Tunisia	Ilhem Boutiba-Ben Boubaker, Sameh Trabelsi, Nissaf Ben Alaya, Maher Kharrat, Alia Ben Kahla, Jalila Ben Khelil, Salma Abid, Sana Ferjani, Mouna Ben Sassi, Mouna Safer, Imen Mkada, Imen Kacem, Gaies Emna, Soumaya Rammeh, Riadh Daghfous, Riadh Gouider.
EPI_ISL_634978, EPI_ISL_634979, EPI_ISL_634981, EPI_ISL_634983, EPI_ISL_634984, EPI_ISL_634985, EPI_ISL_634986, EPI_ISL_634987, EPI_ISL_634988, EPI_ISL_634989, EPI_ISL_634990, EPI_ISL_634991, EPI_ISL_634993, EPI_ISL_634994, EPI_ISL_634995, EPI_ISL_634996, EPI_ISL_634997, EPI_ISL_634998, EPI_ISL_634999, EPI_ISL_635000, EPI_ISL_635001, EPI_ISL_635002, EPI_ISL_635003, EPI_ISL_635004, EPI_ISL_635005, EPI_ISL_635006, EPI_ISL_635007, EPI_ISL_635008, EPI_ISL_635009, EPI_ISL_635010, EPI_ISL_635011, EPI_ISL_635012, EPI_ISL_635013, EPI_ISL_635014, EPI_ISL_635015, EPI_ISL_635016, EPI_ISL_635017, EPI_ISL_635018, EPI_ISL_635019, EPI_ISL_635020, EPI_ISL_635021, EPI_ISL_635022, EPI_ISL_635024, EPI_ISL_635025, EPI_ISL_635026, EPI_ISL_635027, EPI_ISL_635028, EPI_ISL_635029, EPI_ISL_635030, EPI_ISL_635031, EPI_ISL_635032, EPI_ISL_635033, EPI_ISL_635034, EPI_ISL_635035, EPI_ISL_635036, EPI_ISL_635037, EPI_ISL_635038, EPI_ISL_635039, EPI_ISL_635040, EPI_ISL_635041, EPI_ISL_635042, EPI_ISL_635043, EPI_ISL_635044, EPI_ISL_635045, EPI_ISL_635046, EPI_ISL_635047, EPI_ISL_635048, EPI_ISL_635049, EPI_ISL_635050, EPI_ISL_635051, EPI_ISL_635052, EPI_ISL_635053, EPI_ISL_635054, EPI_ISL_635055, EPI_ISL_635056, EPI_ISL_635057, EPI_ISL_635058			
see above	National Health Laboratory Service - Inkosi Albert Luthuli Central Hospital (NHLS-IALCH)	KRISP, KZN Research Innovation and Sequencing Platform	Giandhari J, Pillay S, Lessells R, Mdlalose K, York D, Khan S, Tegally H, Wilkinson E, de Oliveira T
EPI_ISL_635059, EPI_ISL_635060, EPI_ISL_635061, EPI_ISL_635062	1-Laboratory of Microbiology, National Reference Lab, Charles Nicolle Hospital; 2-University of Tunis ElManar, Faculty of Medicine of Tunis, LR99ES09, Tunis, Tunisia	1-Clinical and Experimental Pharmacology Lab, LR16SP02, National Center of Pharmacovigilance, University of Tunis El Manar, Tunis, Tunisia. 2-Neurodegenerative diseases and psychiatric troubles, LR18SP03, Razi Hospital, University of Tunis El Manar, Tunis, Tunisia. 3- Ministry of Health, National Observatory of New and Emerging Diseases, 1006, Tunis, Tunisia	Ilhem Boutiba-Ben Boubaker, Sameh Trabelsi, Nissaf Ben Alaya, Maher Kharrat, Alia Ben Kahla, Jalila Ben Khelil, Salma Abid, Sana Ferjani, Mouna Ben Sassi, Mouna Safer, Imen Mkada, Imen Kacem, Gaies Emna, Soumaya Rammeh, Riadh Daghfous, Riadh Gouider.
EPI_ISL_635063, EPI_ISL_635064, EPI_ISL_635065	Foerde Hospital, Department of Microbiology	Norwegian Institute of Public Health, Department of Virology	Kathrine Stene-Johansen, Kamilla Heddeland Instefjord, Hilde Elshaug, Marie Paulsen Madsen, Rasmus Riis Kopperud, Hilde Vollan, Karoline Bragstad, Olav Hungnes
EPI_ISL_635066, EPI_ISL_635067	Norwegian Institute of Public Health, Department of Virology	Norwegian Institute of Public Health, Department of Virology	Kathrine Stene-Johansen, Kamilla Heddeland Instefjord, Hilde Elshaug, Marie Paulsen Madsen, Rasmus Riis Kopperud, Hilde Vollan, Karoline Bragstad, Olav Hungnes
EPI_ISL_635068, EPI_ISL_635069, EPI_ISL_635070, EPI_ISL_635071, EPI_ISL_635072, EPI_ISL_635073	University Hospital of Northern Norway, Department for Microbiology and Infectious Disease Control	Norwegian Institute of Public Health, Department of Virology	Kathrine Stene-Johansen, Kamilla Heddeland Instefjord, Hilde Elshaug, Marie Paulsen Madsen, Rasmus Riis Kopperud, Hilde Vollan, Karoline Bragstad, Olav Hungnes
EPI_ISL_635074, EPI_ISL_635075, EPI_ISL_635076, EPI_ISL_635077, EPI_ISL_635078, EPI_ISL_635079	Norwegian Institute of Public Health, Department of Virology	Norwegian Institute of Public Health, Department of Virology	Kathrine Stene-Johansen, Kamilla Heddeland Instefjord, Hilde Elshaug, Marie Paulsen Madsen, Rasmus Riis Kopperud, Hilde Vollan, Karoline Bragstad, Olav Hungnes
EPI_ISL_635080, EPI_ISL_635081, EPI_ISL_635082, EPI_ISL_635083,	Haukeland University Hospital, Department of Medical Microbiology	Norwegian Institute of Public Health, Department of Virology	Kathrine Stene-Johansen, Kamilla Heddeland Instefjord, Hilde Elshaug, Marie Paulsen Madsen, Rasmus Riis Kopperud, Hilde Vollan, Karoline Bragstad, Olav Hungnes

EPI_ISL_635084			
EPI_ISL_635085	Oslo University Hospital, Department of Medical Microbiology	Norwegian Institute of Public Health, Department of Virology	Kathrine Stene-Johansen, Kamilla Heddeland Instefjord, Hilde Elshaug, Marie Paulsen Madsen, Rasmus Riis Kopperud, Hilde Vollan, Karoline Bragstad, Olav Hungnes
EPI_ISL_635086, EPI_ISL_635087	Furst Medical Laboratory	Norwegian Institute of Public Health, Department of Virology	Kathrine Stene-Johansen, Kamilla Heddeland Instefjord, Hilde Elshaug, Marie Paulsen Madsen, Rasmus Riis Kopperud, Hilde Vollan, Karoline Bragstad, Olav Hungnes
EPI_ISL_635088	Medical Microbiology Unit, Department for Laboratory Medicine, Drammen Hospital, Vestre Viken Health Trust,	Norwegian Institute of Public Health, Department of Virology	Kathrine Stene-Johansen, Kamilla Heddeland Instefjord, Hilde Elshaug, Marie Paulsen Madsen, Rasmus Riis Kopperud, Hilde Vollan, Karoline Bragstad, Olav Hungnes
EPI_ISL_635089, EPI_ISL_635090, EPI_ISL_635091	Haukeland University Hospital, Department of Medical Microbiology	Norwegian Institute of Public Health, Department of Virology	Kathrine Stene-Johansen, Kamilla Heddeland Instefjord, Hilde Elshaug, Marie Paulsen Madsen, Rasmus Riis Kopperud, Hilde Vollan, Karoline Bragstad, Olav Hungnes
EPI_ISL_635092	Medical Microbiology Unit, Department for Laboratory Medicine, Drammen Hospital, Vestre Viken Health Trust,	Norwegian Institute of Public Health, Department of Virology	Kathrine Stene-Johansen, Kamilla Heddeland Instefjord, Hilde Elshaug, Marie Paulsen Madsen, Rasmus Riis Kopperud, Hilde Vollan, Karoline Bragstad, Olav Hungnes
EPI_ISL_635093, EPI_ISL_635094, EPI_ISL_635095	Oslo University Hospital, Department of Medical Microbiology	Norwegian Institute of Public Health, Department of Virology	Kathrine Stene-Johansen, Kamilla Heddeland Instefjord, Hilde Elshaug, Marie Paulsen Madsen, Rasmus Riis Kopperud, Hilde Vollan, Karoline Bragstad, Olav Hungnes
EPI_ISL_635096	Department of Medical Microbiology, St. Olavs hospital	Norwegian Institute of Public Health, Department of Virology	Kathrine Stene-Johansen, Kamilla Heddeland Instefjord, Hilde Elshaug, Marie Paulsen Madsen, Rasmus Riis Kopperud, Hilde Vollan, Karoline Bragstad, Olav Hungnes
EPI_ISL_635097	Hospital of Southern Norway - Kristiansand, Department of Medical Microbiology	Norwegian Institute of Public Health, Department of Virology	Kathrine Stene-Johansen, Kamilla Heddeland Instefjord, Hilde Elshaug, Marie Paulsen Madsen, Rasmus Riis Kopperud, Hilde Vollan, Karoline Bragstad, Olav Hungnes
EPI_ISL_635098	Foerde Hospital, Department of Microbiology	Norwegian Institute of Public Health, Department of Virology	Kathrine Stene-Johansen, Kamilla Heddeland Instefjord, Hilde Elshaug, Marie Paulsen Madsen, Rasmus Riis Kopperud, Hilde Vollan, Karoline Bragstad, Olav Hungnes
EPI_ISL_635099	Medical Microbiology Unit, Department for Laboratory Medicine, Drammen Hospital, Vestre Viken Health Trust,	Norwegian Institute of Public Health, Department of Virology	Kathrine Stene-Johansen, Kamilla Heddeland Instefjord, Hilde Elshaug, Marie Paulsen Madsen, Rasmus Riis Kopperud, Hilde Vollan, Karoline Bragstad, Olav Hungnes
EPI_ISL_635100	Department of Medical Microbiology, St. Olavs hospital	Norwegian Institute of Public Health, Department of Virology	Kathrine Stene-Johansen, Kamilla Heddeland Instefjord, Hilde Elshaug, Marie Paulsen Madsen, Rasmus Riis Kopperud, Hilde Vollan, Karoline Bragstad, Olav Hungnes
EPI_ISL_635101	Medical Microbiology Unit, Department for Laboratory Medicine, Drammen Hospital, Vestre Viken Health Trust,	Norwegian Institute of Public Health, Department of Virology	Kathrine Stene-Johansen, Kamilla Heddeland Instefjord, Hilde Elshaug, Marie Paulsen Madsen, Rasmus Riis Kopperud, Hilde Vollan, Karoline Bragstad, Olav Hungnes
EPI_ISL_635102	Akershus University Hospital, Department for Microbiology and Infectious Disease Control	Norwegian Institute of Public Health, Department of Virology	Kathrine Stene-Johansen, Kamilla Heddeland Instefjord, Hilde Elshaug, Marie Paulsen Madsen, Rasmus Riis Kopperud, Hilde Vollan, Karoline Bragstad, Olav Hungnes
EPI_ISL_635103	Vestfold Hospital, Toensberg Department of Microbiology	Norwegian Institute of Public Health, Department of Virology	Kathrine Stene-Johansen, Kamilla Heddeland Instefjord, Hilde Elshaug, Marie Paulsen Madsen, Rasmus Riis Kopperud, Hilde Vollan, Karoline Bragstad, Olav Hungnes
EPI_ISL_635104	Unilabs Laboratory Medicine	Norwegian Institute of Public Health, Department of Virology	Kathrine Stene-Johansen, Kamilla Heddeland Instefjord, Hilde Elshaug, Marie Paulsen Madsen, Rasmus Riis Kopperud, Hilde Vollan, Karoline Bragstad, Olav Hungnes
EPI_ISL_635105, EPI_ISL_635106	Vestfold Hospital, Toensberg Department of Microbiology	Norwegian Institute of Public Health, Department of Virology	Kathrine Stene-Johansen, Kamilla Heddeland Instefjord, Hilde Elshaug, Marie Paulsen Madsen, Rasmus Riis Kopperud, Hilde Vollan, Karoline Bragstad, Olav Hungnes
EPI_ISL_635107, EPI_ISL_635108, EPI_ISL_635109	Ostfold Hospital Trust - Kalnes, Centre for Laboratory Medicine, Section for gene technology and infection serology	Norwegian Institute of Public Health, Department of Virology	Kathrine Stene-Johansen, Kamilla Heddeland Instefjord, Hilde Elshaug, Marie Paulsen Madsen, Rasmus Riis Kopperud, Hilde Vollan, Karoline Bragstad, Olav Hungnes
EPI_ISL_635110, EPI_ISL_635111	Department of Medical Microbiology, St. Olavs hospital	Norwegian Institute of Public Health, Department of Virology	Kathrine Stene-Johansen, Kamilla Heddeland Instefjord, Hilde Elshaug, Marie Paulsen Madsen, Rasmus Riis Kopperud, Hilde Vollan, Karoline Bragstad, Olav Hungnes
EPI_ISL_635112	Vestfold Hospital, Toensberg Department of Microbiology	Norwegian Institute of Public Health, Department of Virology	Kathrine Stene-Johansen, Kamilla Heddeland Instefjord, Hilde Elshaug, Marie Paulsen Madsen, Rasmus Riis Kopperud, Hilde Vollan, Karoline Bragstad, Olav Hungnes
EPI_ISL_635113, EPI_ISL_635114, EPI_ISL_635115, EPI_ISL_635117, EPI_ISL_635118	University Hospital of Northern Norway, Department for Microbiology and Infectious Disease Control	Norwegian Institute of Public Health, Department of Virology	Kathrine Stene-Johansen, Kamilla Heddeland Instefjord, Hilde Elshaug, Marie Paulsen Madsen, Rasmus Riis Kopperud, Hilde Vollan, Karoline Bragstad, Olav Hungnes
EPI_ISL_635119	Oslo University Hospital, Department of Medical Microbiology	Norwegian Institute of Public Health, Department of Virology	Kathrine Stene-Johansen, Kamilla Heddeland Instefjord, Hilde Elshaug, Marie Paulsen Madsen, Rasmus Riis Kopperud, Hilde Vollan, Karoline Bragstad, Olav Hungnes
EPI_ISL_635120	Dept. of Medical Microbiology, Stavanger University Hospital, Helse Stavanger HF	Norwegian Institute of Public Health, Department of Virology	Kathrine Stene-Johansen, Kamilla Heddeland Instefjord, Hilde Elshaug, Marie Paulsen Madsen, Rasmus Riis Kopperud, Hilde Vollan, Karoline Bragstad, Olav Hungnes
EPI_ISL_635121, EPI_ISL_635122, EPI_ISL_635123	Hospital of Southern Norway - Kristiansand, Department of Medical Microbiology	Norwegian Institute of Public Health, Department of Virology	Kathrine Stene-Johansen, Kamilla Heddeland Instefjord, Hilde Elshaug, Marie Paulsen Madsen, Rasmus Riis Kopperud, Hilde Vollan, Karoline Bragstad, Olav Hungnes
EPI_ISL_635124, EPI_ISL_635125	Innlandet Hospital Trust, Division Lillehammer, Department for Medical Microbiology	Norwegian Institute of Public Health, Department of Virology	Kathrine Stene-Johansen, Kamilla Heddeland Instefjord, Hilde Elshaug, Marie Paulsen Madsen, Rasmus Riis Kopperud, Hilde Vollan, Karoline Bragstad, Olav Hungnes
EPI_ISL_635126	Department of Medical Microbiology, St. Olavs hospital	Norwegian Institute of Public Health, Department of Virology	Kathrine Stene-Johansen, Kamilla Heddeland Instefjord, Hilde Elshaug, Marie Paulsen Madsen, Rasmus Riis Kopperud, Hilde Vollan, Karoline Bragstad, Olav Hungnes
EPI_ISL_635127	Foerde Hospital, Department of Microbiology	Norwegian Institute of Public Health, Department of Virology	Kathrine Stene-Johansen, Kamilla Heddeland Instefjord, Hilde Elshaug, Marie Paulsen Madsen, Rasmus Riis Kopperud, Hilde Vollan, Karoline Bragstad, Olav Hungnes
EPI_ISL_635128	Haukeland University Hospital, Dept. of Microbiology	Norwegian Institute of Public Health, Department of Virology	Kathrine Stene-Johansen, Kamilla Heddeland Instefjord, Hilde Elshaug, Marie Paulsen Madsen, Rasmus Riis Kopperud, Hilde Vollan, Karoline Bragstad, Olav Hungnes
EPI_ISL_635129	Oslo University Hospital, Department of Medical Microbiology	Norwegian Institute of Public Health, Department of Virology	Kathrine Stene-Johansen, Kamilla Heddeland Instefjord, Hilde Elshaug, Marie Paulsen Madsen, Rasmus Riis Kopperud, Hilde Vollan, Karoline Bragstad, Olav Hungnes
EPI_ISL_635130, EPI_ISL_635131	Innlandet Hospital Trust, Division Lillehammer, Department for Medical Microbiology	Norwegian Institute of Public Health, Department of Virology	Kathrine Stene-Johansen, Kamilla Heddeland Instefjord, Hilde Elshaug, Marie Paulsen Madsen, Rasmus Riis Kopperud, Hilde Vollan, Karoline Bragstad, Olav Hungnes
EPI_ISL_635132	Unilabs Laboratory Medicine	Norwegian Institute of Public Health, Department of Virology	Kathrine Stene-Johansen, Kamilla Heddeland Instefjord, Hilde Elshaug, Marie Paulsen Madsen, Rasmus Riis Kopperud, Hilde Vollan, Karoline Bragstad, Olav Hungnes
EPI_ISL_635133	Haukeland University Hospital, Department of Medical Microbiology	Norwegian Institute of Public Health, Department of Virology	Kathrine Stene-Johansen, Kamilla Heddeland Instefjord, Hilde Elshaug, Marie Paulsen Madsen, Rasmus Riis Kopperud, Hilde Vollan, Karoline Bragstad, Olav Hungnes
EPI_ISL_635134	Medical Microbiology Unit, Department for Laboratory Medicine, Drammen Hospital, Vestre Viken Health Trust,	Norwegian Institute of Public Health, Department of Virology	Kathrine Stene-Johansen, Kamilla Heddeland Instefjord, Hilde Elshaug, Marie Paulsen Madsen, Rasmus Riis Kopperud, Hilde Vollan, Karoline Bragstad, Olav Hungnes
EPI_ISL_635135, EPI_ISL_635136	Department of Medical Microbiology, St. Olavs hospital	Norwegian Institute of Public Health, Department of Virology	Kathrine Stene-Johansen, Kamilla Heddeland Instefjord, Hilde Elshaug, Marie Paulsen Madsen, Rasmus Riis Kopperud, Hilde Vollan, Karoline Bragstad, Olav Hungnes
EPI_ISL_635137	University Hospital of Northern Norway, Department for Microbiology and Infectious Disease Control	Norwegian Institute of Public Health, Department of Virology	Kathrine Stene-Johansen, Kamilla Heddeland Instefjord, Hilde Elshaug, Marie Paulsen Madsen, Rasmus Riis Kopperud, Hilde Vollan, Karoline Bragstad, Olav Hungnes

EPI_ISL_635138	Hospital of Southern Norway - Kristiansand, Department of Medical Microbiology	Norwegian Institute of Public Health, Department of Virology	Kathrine Stene-Johansen, Kamilla Heddeland Instefjord, Hilde Elshaug, Marie Paulsen Madsen, Rasmus Riis Kopperud, Hilde Vollan, Karoline Bragstad, Olav Hungnes
EPI_ISL_635139, EPI_ISL_635140	Vestfold Hospital, Toensberg Department of Microbiology	Norwegian Institute of Public Health, Department of Virology	Kathrine Stene-Johansen, Kamilla Heddeland Instefjord, Hilde Elshaug, Marie Paulsen Madsen, Rasmus Riis Kopperud, Hilde Vollan, Karoline Bragstad, Olav Hungnes
EPI_ISL_635141	University Hospital of Northern Norway, Department for Microbiology and Infectious Disease Control	Norwegian Institute of Public Health, Department of Virology	Kathrine Stene-Johansen, Kamilla Heddeland Instefjord, Hilde Elshaug, Marie Paulsen Madsen, Rasmus Riis Kopperud, Hilde Vollan, Karoline Bragstad, Olav Hungnes
EPI_ISL_635142	Nordland Hospital - Bodo, Laboratory Department, Molecular Biology Unit	Norwegian Institute of Public Health, Department of Virology	Kathrine Stene-Johansen, Kamilla Heddeland Instefjord, Hilde Elshaug, Marie Paulsen Madsen, Rasmus Riis Kopperud, Hilde Vollan, Karoline Bragstad, Olav Hungnes
EPI_ISL_635143	Hospital of Southern Norway - Kristiansand, Department of Medical Microbiology	Norwegian Institute of Public Health, Department of Virology	Kathrine Stene-Johansen, Kamilla Heddeland Instefjord, Hilde Elshaug, Marie Paulsen Madsen, Rasmus Riis Kopperud, Hilde Vollan, Karoline Bragstad, Olav Hungnes
EPI_ISL_635144	Dept. of Medical Microbiology, Stavanger University Hospital, Helse Stavanger HF	Norwegian Institute of Public Health, Department of Virology	Kathrine Stene-Johansen, Kamilla Heddeland Instefjord, Hilde Elshaug, Marie Paulsen Madsen, Rasmus Riis Kopperud, Hilde Vollan, Karoline Bragstad, Olav Hungnes
EPI_ISL_635145	Nordland Hospital - Bodo, Laboratory Department, Molecular Biology Unit	Norwegian Institute of Public Health, Department of Virology	Kathrine Stene-Johansen, Kamilla Heddeland Instefjord, Hilde Elshaug, Marie Paulsen Madsen, Rasmus Riis Kopperud, Hilde Vollan, Karoline Bragstad, Olav Hungnes
EPI_ISL_635146	Dept. of Medical Microbiology, Stavanger University Hospital, Helse Stavanger HF	Norwegian Institute of Public Health, Department of Virology	Kathrine Stene-Johansen, Kamilla Heddeland Instefjord, Hilde Elshaug, Marie Paulsen Madsen, Rasmus Riis Kopperud, Hilde Vollan, Karoline Bragstad, Olav Hungnes
EPI_ISL_635147, EPI_ISL_635148	University Hospital of Northern Norway, Department for Microbiology and Infectious Disease Control	Norwegian Institute of Public Health, Department of Virology	Kathrine Stene-Johansen, Kamilla Heddeland Instefjord, Hilde Elshaug, Marie Paulsen Madsen, Rasmus Riis Kopperud, Hilde Vollan, Karoline Bragstad, Olav Hungnes
EPI_ISL_635149, EPI_ISL_635150, EPI_ISL_635151, EPI_ISL_635152	Ostfold Hospital Trust - Kalnes, Centre for Laboratory Medicine, Section for gene technology and infection serology	Norwegian Institute of Public Health, Department of Virology	Kathrine Stene-Johansen, Kamilla Heddeland Instefjord, Hilde Elshaug, Marie Paulsen Madsen, Rasmus Riis Kopperud, Hilde Vollan, Karoline Bragstad, Olav Hungnes
EPI_ISL_635153, EPI_ISL_635154	Unilabs Laboratory Medicine	Norwegian Institute of Public Health, Department of Virology	Kathrine Stene-Johansen, Kamilla Heddeland Instefjord, Hilde Elshaug, Marie Paulsen Madsen, Rasmus Riis Kopperud, Hilde Vollan, Karoline Bragstad, Olav Hungnes
EPI_ISL_635155	Oslo University Hospital, Department of Medical Microbiology	Norwegian Institute of Public Health, Department of Virology	Kathrine Stene-Johansen, Kamilla Heddeland Instefjord, Hilde Elshaug, Marie Paulsen Madsen, Rasmus Riis Kopperud, Hilde Vollan, Karoline Bragstad, Olav Hungnes
EPI_ISL_635156, EPI_ISL_635157	University Hospital of Northern Norway, Department for Microbiology and Infectious Disease Control	Norwegian Institute of Public Health, Department of Virology	Kathrine Stene-Johansen, Kamilla Heddeland Instefjord, Hilde Elshaug, Marie Paulsen Madsen, Rasmus Riis Kopperud, Hilde Vollan, Karoline Bragstad, Olav Hungnes
EPI_ISL_635158	Unilabs Laboratory Medicine	Norwegian Institute of Public Health, Department of Virology	Kathrine Stene-Johansen, Kamilla Heddeland Instefjord, Hilde Elshaug, Marie Paulsen Madsen, Rasmus Riis Kopperud, Hilde Vollan, Karoline Bragstad, Olav Hungnes
EPI_ISL_635159	Medical Microbiology Unit, Department for Laboratory Medicine, Drammen Hospital, Vestre Viken Health Trust,	Norwegian Institute of Public Health, Department of Virology	Kathrine Stene-Johansen, Kamilla Heddeland Instefjord, Hilde Elshaug, Marie Paulsen Madsen, Rasmus Riis Kopperud, Hilde Vollan, Karoline Bragstad, Olav Hungnes
EPI_ISL_635160	University Hospital of Northern Norway, Department for Microbiology and Infectious Disease Control	Norwegian Institute of Public Health, Department of Virology	Kathrine Stene-Johansen, Kamilla Heddeland Instefjord, Hilde Elshaug, Marie Paulsen Madsen, Rasmus Riis Kopperud, Hilde Vollan, Karoline Bragstad, Olav Hungnes
EPI_ISL_635161	Hospital of Southern Norway - Kristiansand, Department of Medical Microbiology	Norwegian Institute of Public Health, Department of Virology	Kathrine Stene-Johansen, Kamilla Heddeland Instefjord, Hilde Elshaug, Marie Paulsen Madsen, Rasmus Riis Kopperud, Hilde Vollan, Karoline Bragstad, Olav Hungnes
EPI_ISL_635162	Unilabs Laboratory Medicine	Norwegian Institute of Public Health, Department of Virology	Kathrine Stene-Johansen, Kamilla Heddeland Instefjord, Hilde Elshaug, Marie Paulsen Madsen, Rasmus Riis Kopperud, Hilde Vollan, Karoline Bragstad, Olav Hungnes
EPI_ISL_635163	University Hospital of Northern Norway, Department for Microbiology and Infectious Disease Control	Norwegian Institute of Public Health, Department of Virology	Kathrine Stene-Johansen, Kamilla Heddeland Instefjord, Hilde Elshaug, Marie Paulsen Madsen, Rasmus Riis Kopperud, Hilde Vollan, Karoline Bragstad, Olav Hungnes
EPI_ISL_635164	Ostfold Hospital Trust - Kalnes, Centre for Laboratory Medicine, Section for gene technology and infection serology	Norwegian Institute of Public Health, Department of Virology	Kathrine Stene-Johansen, Kamilla Heddeland Instefjord, Hilde Elshaug, Marie Paulsen Madsen, Rasmus Riis Kopperud, Hilde Vollan, Karoline Bragstad, Olav Hungnes
EPI_ISL_635165	Department of Medical Microbiology, St. Olavs hospital	Norwegian Institute of Public Health, Department of Virology	Kathrine Stene-Johansen, Kamilla Heddeland Instefjord, Hilde Elshaug, Marie Paulsen Madsen, Rasmus Riis Kopperud, Hilde Vollan, Karoline Bragstad, Olav Hungnes
EPI_ISL_635166	Dept. of Medical Microbiology, Stavanger University Hospital, Helse Stavanger HF	Norwegian Institute of Public Health, Department of Virology	Kathrine Stene-Johansen, Kamilla Heddeland Instefjord, Hilde Elshaug, Marie Paulsen Madsen, Rasmus Riis Kopperud, Hilde Vollan, Karoline Bragstad, Olav Hungnes
EPI_ISL_635167	Foerde Hospital, Department of Microbiology	Norwegian Institute of Public Health, Department of Virology	Kathrine Stene-Johansen, Kamilla Heddeland Instefjord, Hilde Elshaug, Marie Paulsen Madsen, Rasmus Riis Kopperud, Hilde Vollan, Karoline Bragstad, Olav Hungnes
EPI_ISL_635168	Vestfold Hospital, Toensberg Department of Microbiology	Norwegian Institute of Public Health, Department of Virology	Kathrine Stene-Johansen, Kamilla Heddeland Instefjord, Hilde Elshaug, Marie Paulsen Madsen, Rasmus Riis Kopperud, Hilde Vollan, Karoline Bragstad, Olav Hungnes
EPI_ISL_635169	Medical Microbiology Unit, Department for Laboratory Medicine, Drammen Hospital, Vestre Viken Health Trust,	Norwegian Institute of Public Health, Department of Virology	Kathrine Stene-Johansen, Kamilla Heddeland Instefjord, Hilde Elshaug, Marie Paulsen Madsen, Rasmus Riis Kopperud, Hilde Vollan, Karoline Bragstad, Olav Hungnes
EPI_ISL_635170	Foerde Hospital, Department of Microbiology	Norwegian Institute of Public Health, Department of Virology	Kathrine Stene-Johansen, Kamilla Heddeland Instefjord, Hilde Elshaug, Marie Paulsen Madsen, Rasmus Riis Kopperud, Hilde Vollan, Karoline Bragstad, Olav Hungnes
EPI_ISL_635171	Vestfold Hospital, Toensberg Department of Microbiology	Norwegian Institute of Public Health, Department of Virology	Kathrine Stene-Johansen, Kamilla Heddeland Instefjord, Hilde Elshaug, Marie Paulsen Madsen, Rasmus Riis Kopperud, Hilde Vollan, Karoline Bragstad, Olav Hungnes
EPI_ISL_635172	Oslo University Hospital, Department of Medical Microbiology	Norwegian Institute of Public Health, Department of Virology	Kathrine Stene-Johansen, Kamilla Heddeland Instefjord, Hilde Elshaug, Marie Paulsen Madsen, Rasmus Riis Kopperud, Hilde Vollan, Karoline Bragstad, Olav Hungnes
EPI_ISL_635173	Foerde Hospital, Department of Microbiology	Norwegian Institute of Public Health, Department of Virology	Kathrine Stene-Johansen, Kamilla Heddeland Instefjord, Hilde Elshaug, Marie Paulsen Madsen, Rasmus Riis Kopperud, Hilde Vollan, Karoline Bragstad, Olav Hungnes
EPI_ISL_635174, EPI_ISL_635175	Ostfold Hospital Trust - Kalnes, Centre for Laboratory Medicine, Section for gene technology and infection serology	Norwegian Institute of Public Health, Department of Virology	Kathrine Stene-Johansen, Kamilla Heddeland Instefjord, Hilde Elshaug, Marie Paulsen Madsen, Rasmus Riis Kopperud, Hilde Vollan, Karoline Bragstad, Olav Hungnes
EPI_ISL_635176, EPI_ISL_635177	Foerde Hospital, Department of Microbiology	Norwegian Institute of Public Health, Department of Virology	Kathrine Stene-Johansen, Kamilla Heddeland Instefjord, Hilde Elshaug, Marie Paulsen Madsen, Rasmus Riis Kopperud, Hilde Vollan, Karoline Bragstad, Olav Hungnes
EPI_ISL_635178	Norwegian Institute of Public Health, Department of Virology	Norwegian Institute of Public Health, Department of Virology	Kathrine Stene-Johansen, Kamilla Heddeland Instefjord, Hilde Elshaug, Marie Paulsen Madsen, Rasmus Riis Kopperud, Hilde Vollan, Karoline Bragstad, Olav Hungnes
EPI_ISL_635179, EPI_ISL_635180, EPI_ISL_635181	University Hospital of Northern Norway, Department for Microbiology and Infectious Disease Control	Norwegian Institute of Public Health, Department of Virology	Kathrine Stene-Johansen, Kamilla Heddeland Instefjord, Hilde Elshaug, Marie Paulsen Madsen, Rasmus Riis Kopperud, Hilde Vollan, Karoline Bragstad, Olav Hungnes
EPI_ISL_635182	Nordland Hospital - Bodo, Laboratory Department, Molecular Biology Unit	Norwegian Institute of Public Health, Department of Virology	Kathrine Stene-Johansen, Kamilla Heddeland Instefjord, Hilde Elshaug, Marie Paulsen Madsen, Rasmus Riis Kopperud, Hilde Vollan, Karoline Bragstad, Olav Hungnes
EPI_ISL_635183	Innlandet Hospital Trust, Division Lillehammer, Department for Medical Microbiology	Norwegian Institute of Public Health, Department of Virology	Kathrine Stene-Johansen, Kamilla Heddeland Instefjord, Hilde Elshaug, Marie Paulsen Madsen, Rasmus Riis Kopperud, Hilde Vollan, Karoline Bragstad, Olav Hungnes

EPI_ISL_635184	Haukeland University Hospital, Department of Medical Microbiology	Norwegian Institute of Public Health, Department of Virology	Kathrine Stene-Johansen, Kamilla Heddeland Instefjord, Hilde Elshaug, Marie Paulsen Madsen, Rasmus Riis Kopperud, Hilde Vollan, Karoline Bragstad, Olav Hungnes
EPI_ISL_635185	Ostfold Hospital Trust - Kalnes, Centre for Laboratory Medicine, Section for gene technology and infection serology	Norwegian Institute of Public Health, Department of Virology	Kathrine Stene-Johansen, Kamilla Heddeland Instefjord, Hilde Elshaug, Marie Paulsen Madsen, Rasmus Riis Kopperud, Hilde Vollan, Karoline Bragstad, Olav Hungnes
EPI_ISL_635186	University Hospital of Northern Norway, Department for Microbiology and Infectious Disease Control	Norwegian Institute of Public Health, Department of Virology	Kathrine Stene-Johansen, Kamilla Heddeland Instefjord, Hilde Elshaug, Marie Paulsen Madsen, Rasmus Riis Kopperud, Hilde Vollan, Karoline Bragstad, Olav Hungnes
EPI_ISL_635187, EPI_ISL_635188	Norwegian Institute of Public Health, Department of Virology	Norwegian Institute of Public Health, Department of Virology	Kathrine Stene-Johansen, Kamilla Heddeland Instefjord, Hilde Elshaug, Marie Paulsen Madsen, Rasmus Riis Kopperud, Hilde Vollan, Karoline Bragstad, Olav Hungnes
EPI_ISL_635189	Hospital of Southern Norway - Kristiansand, Department of Medical Microbiology	Norwegian Institute of Public Health, Department of Virology	Kathrine Stene-Johansen, Kamilla Heddeland Instefjord, Hilde Elshaug, Marie Paulsen Madsen, Rasmus Riis Kopperud, Hilde Vollan, Karoline Bragstad, Olav Hungnes
EPI_ISL_635190	University Hospital of Northern Norway, Department for Microbiology and Infectious Disease Control	Norwegian Institute of Public Health, Department of Virology	Kathrine Stene-Johansen, Kamilla Heddeland Instefjord, Hilde Elshaug, Marie Paulsen Madsen, Rasmus Riis Kopperud, Hilde Vollan, Karoline Bragstad, Olav Hungnes
EPI_ISL_635191	Unilabs Laboratory Medicine	Norwegian Institute of Public Health, Department of Virology	Kathrine Stene-Johansen, Kamilla Heddeland Instefjord, Hilde Elshaug, Marie Paulsen Madsen, Rasmus Riis Kopperud, Hilde Vollan, Karoline Bragstad, Olav Hungnes
EPI_ISL_635192, EPI_ISL_635193	Medical Microbiology Unit, Department for Laboratory Medicine, Drammen Hospital, Vestre Viken Health Trust,	Norwegian Institute of Public Health, Department of Virology	Kathrine Stene-Johansen, Kamilla Heddeland Instefjord, Hilde Elshaug, Marie Paulsen Madsen, Rasmus Riis Kopperud, Hilde Vollan, Karoline Bragstad, Olav Hungnes
EPI_ISL_635194	Akershus University Hospital, Department for Microbiology and Infectious Disease Control	Norwegian Institute of Public Health, Department of Virology	Kathrine Stene-Johansen, Kamilla Heddeland Instefjord, Hilde Elshaug, Marie Paulsen Madsen, Rasmus Riis Kopperud, Hilde Vollan, Karoline Bragstad, Olav Hungnes
EPI_ISL_635195	Vestfold Hospital, Toensberg Department of Microbiology	Norwegian Institute of Public Health, Department of Virology	Kathrine Stene-Johansen, Kamilla Heddeland Instefjord, Hilde Elshaug, Marie Paulsen Madsen, Rasmus Riis Kopperud, Hilde Vollan, Karoline Bragstad, Olav Hungnes
EPI_ISL_635196	Dept. of Medical Microbiology, Stavanger University Hospital, Helse Stavanger HF	Norwegian Institute of Public Health, Department of Virology	Kathrine Stene-Johansen, Kamilla Heddeland Instefjord, Hilde Elshaug, Marie Paulsen Madsen, Rasmus Riis Kopperud, Hilde Vollan, Karoline Bragstad, Olav Hungnes
EPI_ISL_635197	Oslo University Hospital, Department of Medical Microbiology	Norwegian Institute of Public Health, Department of Virology	Kathrine Stene-Johansen, Kamilla Heddeland Instefjord, Hilde Elshaug, Marie Paulsen Madsen, Rasmus Riis Kopperud, Hilde Vollan, Karoline Bragstad, Olav Hungnes
EPI_ISL_635198	University Hospital of Northern Norway, Department for Microbiology and Infectious Disease Control	Norwegian Institute of Public Health, Department of Virology	Kathrine Stene-Johansen, Kamilla Heddeland Instefjord, Hilde Elshaug, Marie Paulsen Madsen, Rasmus Riis Kopperud, Hilde Vollan, Karoline Bragstad, Olav Hungnes
EPI_ISL_635334, EPI_ISL_635343, EPI_ISL_635347, EPI_ISL_635354, EPI_ISL_635362, EPI_ISL_635363, EPI_ISL_635364, EPI_ISL_635365, EPI_ISL_635366, EPI_ISL_635367, EPI_ISL_635368, EPI_ISL_635369, EPI_ISL_635370, EPI_ISL_635371, EPI_ISL_635372, EPI_ISL_635373, EPI_ISL_635376, EPI_ISL_635377, EPI_ISL_635378, EPI_ISL_635379, EPI_ISL_635380, EPI_ISL_635381, EPI_ISL_635382, EPI_ISL_635383, EPI_ISL_635384, EPI_ISL_635385, EPI_ISL_635386, EPI_ISL_635387, EPI_ISL_635388, EPI_ISL_635389, EPI_ISL_635390, EPI_ISL_635391, EPI_ISL_635392, EPI_ISL_635393, EPI_ISL_635394, EPI_ISL_635395, EPI_ISL_635396, EPI_ISL_635397, EPI_ISL_635398, EPI_ISL_635399, EPI_ISL_635400, EPI_ISL_635401, EPI_ISL_635402, EPI_ISL_635403, EPI_ISL_635404, EPI_ISL_635405, EPI_ISL_635406, EPI_ISL_635407, EPI_ISL_635408, EPI_ISL_635409, EPI_ISL_635410, EPI_ISL_635411, EPI_ISL_635412, EPI_ISL_635413, EPI_ISL_635414, EPI_ISL_635415, EPI_ISL_635416, EPI_ISL_635417, EPI_ISL_635418, EPI_ISL_635419, EPI_ISL_635420, EPI_ISL_635421, EPI_ISL_635422, EPI_ISL_635423, EPI_ISL_635424, EPI_ISL_635425, EPI_ISL_635426, EPI_ISL_635427, EPI_ISL_635428, EPI_ISL_635429, EPI_ISL_635430, EPI_ISL_635431, EPI_ISL_635432, EPI_ISL_635433, EPI_ISL_635434, EPI_ISL_635435, EPI_ISL_635436, EPI_ISL_635437, EPI_ISL_635438, EPI_ISL_635439, EPI_ISL_635440, EPI_ISL_635441, EPI_ISL_635442, EPI_ISL_635443, EPI_ISL_635444, EPI_ISL_635445, EPI_ISL_635446, EPI_ISL_635447, EPI_ISL_635448, EPI_ISL_635449, EPI_ISL_635450, EPI_ISL_635451, EPI_ISL_635452, EPI_ISL_635453, EPI_ISL_635454, EPI_ISL_635455, EPI_ISL_635456, EPI_ISL_635457, EPI_ISL_635458, EPI_ISL_635459, EPI_ISL_635460, EPI_ISL_635461, EPI_ISL_635462, EPI_ISL_635463, EPI_ISL_635464, EPI_ISL_635465, EPI_ISL_635466, EPI_ISL_635467, EPI_ISL_635468, EPI_ISL_635470, EPI_ISL_635471, EPI_ISL_635472, EPI_ISL_635473, EPI_ISL_635474, EPI_ISL_635475, EPI_ISL_635476, EPI_ISL_635477, EPI_ISL_635478			
see above	San Diego County Public Health Laboratory	Andersen lab at Scripps Research	SEARCH Alliance San Diego with Tracy Basler, Jovan Shephard, Brett Austin
EPI_ISL_635506, EPI_ISL_635507, EPI_ISL_635508, EPI_ISL_635509, EPI_ISL_635510, EPI_ISL_635511, EPI_ISL_635512, EPI_ISL_635513, EPI_ISL_635514, EPI_ISL_635515, EPI_ISL_635516, EPI_ISL_635517, EPI_ISL_635518, EPI_ISL_635519, EPI_ISL_635520, EPI_ISL_635521, EPI_ISL_635522, EPI_ISL_635523, EPI_ISL_635524, EPI_ISL_635525, EPI_ISL_635526, EPI_ISL_635527, EPI_ISL_635528, EPI_ISL_635529, EPI_ISL_635530, EPI_ISL_635531, EPI_ISL_635532, EPI_ISL_635533, EPI_ISL_635534, EPI_ISL_635535, EPI_ISL_635536, EPI_ISL_635537, EPI_ISL_635538, EPI_ISL_635539, EPI_ISL_635540, EPI_ISL_635541, EPI_ISL_635542, EPI_ISL_635543, EPI_ISL_635544, EPI_ISL_635545, EPI_ISL_635546, EPI_ISL_635547, EPI_ISL_635548, EPI_ISL_635549, EPI_ISL_635550, EPI_ISL_635551, EPI_ISL_635552, EPI_ISL_635553, EPI_ISL_635554, EPI_ISL_635555, EPI_ISL_635556, EPI_ISL_635557, EPI_ISL_635558, EPI_ISL_635559, EPI_ISL_635560, EPI_ISL_635561, EPI_ISL_635562, EPI_ISL_635563, EPI_ISL_635564, EPI_ISL_635565, EPI_ISL_635566, EPI_ISL_635567, EPI_ISL_635568, EPI_ISL_635569, EPI_ISL_635570, EPI_ISL_635571, EPI_ISL_635572, EPI_ISL_635573, EPI_ISL_635574, EPI_ISL_635575, EPI_ISL_635576			
see above	Centro de Diagnstico COVID-19 UABC Tijuana	Andersen lab at Scripps Research	SEARCH Alliance San Diego with Idanya Rubí Serafín Higuera, Manuel Sánchez Alavez, Jorge Luis Jiménez Niebla, Germán Ibarra, Jonathan Vincent Baena, Oscar Efrén Zazueta Fierro
EPI_ISL_635577, EPI_ISL_635578, EPI_ISL_635579, EPI_ISL_635580, EPI_ISL_635581, EPI_ISL_635582, EPI_ISL_635583, EPI_ISL_635584, EPI_ISL_635585, EPI_ISL_635586, EPI_ISL_635587, EPI_ISL_635588, EPI_ISL_635589, EPI_ISL_635590, EPI_ISL_635591, EPI_ISL_635592, EPI_ISL_635593, EPI_ISL_635594, EPI_ISL_635595, EPI_ISL_635596, EPI_ISL_635597, EPI_ISL_635598, EPI_ISL_635599, EPI_ISL_635600, EPI_ISL_635601, EPI_ISL_635602, EPI_ISL_635603, EPI_ISL_635604, EPI_ISL_635605, EPI_ISL_635606, EPI_ISL_635607, EPI_ISL_635608, EPI_ISL_635609, EPI_ISL_635610, EPI_ISL_635611, EPI_ISL_635612, EPI_ISL_635613, EPI_ISL_635614, EPI_ISL_635615, EPI_ISL_635616, EPI_ISL_635617, EPI_ISL_635618, EPI_ISL_635619, EPI_ISL_635620, EPI_ISL_635621, EPI_ISL_635622, EPI_ISL_635623, EPI_ISL_635624, EPI_ISL_635625, EPI_ISL_635626, EPI_ISL_635627, EPI_ISL_635628, EPI_ISL_635629, EPI_ISL_635630, EPI_ISL_635631, EPI_ISL_635632, EPI_ISL_635633, EPI_ISL_635634, EPI_ISL_635635, EPI_ISL_635636, EPI_ISL_635637, EPI_ISL_635638, EPI_ISL_635639, EPI_ISL_635640, EPI_ISL_635641, EPI_ISL_635642, EPI_ISL_635643, EPI_ISL_635644, EPI_ISL_635645, EPI_ISL_635646, EPI_ISL_635647, EPI_ISL_635648, EPI_ISL_635649, EPI_ISL_635650, EPI_ISL_635651, EPI_ISL_635652, EPI_ISL_635653, EPI_ISL_635654, EPI_ISL_635655, EPI_ISL_635656, EPI_ISL_635657, EPI_ISL_635658, EPI_ISL_635659, EPI_ISL_635660, EPI_ISL_635661, EPI_ISL_635662, EPI_ISL_635663, EPI_ISL_635664, EPI_ISL_635665, EPI_ISL_635666, EPI_ISL_635667, EPI_ISL_635668, EPI_ISL_635669, EPI_ISL_635670, EPI_ISL_635671, EPI_ISL_635672, EPI_ISL_635673, EPI_ISL_635674, EPI_ISL_635675, EPI_ISL_635676, EPI_ISL_635677, EPI_ISL_635678, EPI_ISL_635679, EPI_ISL_635680, EPI_ISL_635681, EPI_ISL_635682, EPI_ISL_635683, EPI_ISL_635684, EPI_ISL_635685, EPI_ISL_635686, EPI_ISL_635687, EPI_ISL_635688, EPI_ISL_635689, EPI_ISL_635690, EPI_ISL_635691, EPI_ISL_635692, EPI_ISL_635693, EPI_ISL_635694, EPI_ISL_635695, EPI_ISL_635696, EPI_ISL_635697, EPI_ISL_635698, EPI_ISL_635699, EPI_ISL_635700, EPI_ISL_635701, EPI_ISL_635702, EPI_ISL_635703, EPI_ISL_635704, EPI_ISL_635705, EPI_ISL_635706, EPI_ISL_635707, EPI_ISL_635708, EPI_ISL_635709, EPI_ISL_635710, EPI_ISL_635711, EPI_ISL_635712, EPI_ISL_635713, EPI_ISL_635714, EPI_ISL_635715, EPI_ISL_635716, EPI_ISL_635717, EPI_ISL_635718, EPI_ISL_635719, EPI_ISL_635720, EPI_ISL_635721, EPI_ISL_635722, EPI_ISL_635723, EPI_ISL_635724, EPI_ISL_635725, EPI_ISL_635726, EPI_ISL_635727, EPI_ISL_635728, EPI_ISL_635729, EPI_ISL_635730, EPI_ISL_635731, EPI_ISL_635732, EPI_ISL_635733, EPI_ISL_635734, EPI_ISL_635735, EPI_ISL_635736, EPI_ISL_635737, EPI_ISL_635738, EPI_ISL_635739, EPI_ISL_635740, EPI_ISL_635741, EPI_ISL_635742, EPI_ISL_635743, EPI_ISL_635744, EPI_ISL_635745, EPI_ISL_635746, EPI_ISL_635747, EPI_ISL_635748, EPI_ISL_635749, EPI_ISL_635750, EPI_ISL_635751, EPI_ISL_635752, EPI_ISL_635753, EPI_ISL_635754, EPI_ISL_635755, EPI_ISL_635756, EPI_ISL_635757, EPI_ISL_635758, EPI_ISL_635759, EPI_ISL_635760, EPI_ISL_635761, EPI_ISL_635762, EPI_ISL_635763, EPI_ISL_635764, EPI_ISL_635765, EPI_ISL_635766, EPI_ISL_635767, EPI_ISL_635768, EPI_ISL_635769, EPI_ISL_635770, EPI_ISL_635771, EPI_ISL_635772, EPI_ISL_635773, EPI_ISL_635774, EPI_ISL_635775, EPI_ISL_635776			
see above	San Diego County Public Health Laboratory	Andersen lab at Scripps Research	SEARCH Alliance San Diego with Tracy Basler, Jovan Shephard, Brett Austin
EPI_ISL_635777, EPI_ISL_635778, EPI_ISL_635779, EPI_ISL_635780, EPI_ISL_635781, EPI_ISL_635782	Biolab Diagnostic Laboratories	Andersen lab at Scripps Research	Issa Abu-Dayyeh, Ahmad Tibi, Lama Hussein, Lina Mohammad, Zein Naber, Amid Abdelnour with SEARCH Alliance San Diego
EPI_ISL_635783, EPI_ISL_635784, EPI_ISL_635785, EPI_ISL_635786, EPI_ISL_635787, EPI_ISL_635788, EPI_ISL_635789, EPI_ISL_635790, EPI_ISL_635791, EPI_ISL_635792, EPI_ISL_635793, EPI_ISL_635794, EPI_ISL_635795, EPI_ISL_635796, EPI_ISL_635797, EPI_ISL_635798, EPI_ISL_635799, EPI_ISL_635800, EPI_ISL_635801, EPI_ISL_635802, EPI_ISL_635803, EPI_ISL_635804, EPI_ISL_635805, EPI_ISL_635806, EPI_ISL_635807, EPI_ISL_635808, EPI_ISL_635809, EPI_ISL_635810, EPI_ISL_635811, EPI_ISL_635812, EPI_ISL_635813, EPI_ISL_635814, EPI_ISL_635815, EPI_ISL_635816, EPI_ISL_635817, EPI_ISL_635818, EPI_ISL_635819, EPI_ISL_635820, EPI_ISL_635821, EPI_ISL_635822, EPI_ISL_635823, EPI_ISL_635824, EPI_ISL_635825, EPI_ISL_635826, EPI_ISL_635827, EPI_ISL_635828, EPI_ISL_635829, EPI_ISL_635830, EPI_ISL_635831, EPI_ISL_635832, EPI_ISL_635833, EPI_ISL_635834, EPI_ISL_635835, EPI_ISL_635836, EPI_ISL_635837, EPI_ISL_635838, EPI_ISL_635839, EPI_ISL_635840, EPI_ISL_635841, EPI_ISL_635842, EPI_ISL_635843, EPI_ISL_635844, EPI_ISL_635845, EPI_ISL_635846, EPI_ISL_635847, EPI_ISL_635848, EPI_ISL_635849, EPI_ISL_635850, EPI_ISL_635851, EPI_ISL_635852, EPI_ISL_635853, EPI_ISL_635854, EPI_ISL_635855, EPI_ISL_635856, EPI_ISL_635857, EPI_ISL_635858, EPI_ISL_635859, EPI_ISL_635860, EPI_ISL_635861, EPI_ISL_635862, EPI_ISL_635863, EPI_ISL_635864, EPI_ISL_635865, EPI_ISL_635866, EPI_ISL_635867, EPI_ISL_635868, EPI_ISL_635869, EPI_ISL_635870, EPI_ISL_635871, EPI_ISL_635872, EPI_ISL_635873, EPI_ISL_635874, EPI_ISL_635875, EPI_ISL_635876, EPI_ISL_635877, EPI_ISL_635878, EPI_ISL_635879, EPI_ISL_635880, EPI_ISL_635881, EPI_ISL_635882, EPI_ISL_635883, EPI_ISL_635884, EPI_ISL_635885, EPI_ISL_635886, EPI_ISL_635887, EPI_ISL_635888, EPI_ISL_635889, EPI_ISL_635890, EPI_ISL_635891, EPI_ISL_635892, EPI_ISL_635893, EPI_ISL_635894, EPI_ISL_635895, EPI_ISL_635896, EPI_ISL_635897, EPI_ISL_635898, EPI_ISL_635899, EPI_ISL_635900, EPI_ISL_635901, EPI_ISL_635902, EPI_ISL_635903, EPI_ISL_635904, EPI_ISL_635905, EPI_ISL_635906, EPI_ISL_635907, EPI_ISL_635908, EPI_ISL_635909, EPI_ISL_635910, EPI_ISL_635911, EPI_ISL_635912, EPI_ISL_635913, EPI_ISL_635914, EPI_ISL_635915, EPI_ISL_635916, EPI_ISL_635917, EPI_ISL_635918, EPI_ISL_635919, EPI_ISL_635920, EPI_ISL_635921, EPI_ISL_635922, EPI_ISL_635923, EPI_ISL_635924, EPI_ISL_635925, EPI_ISL_635926, EPI_ISL_635927, EPI_ISL_635928, EPI_ISL_635929, EPI_ISL_635930, EPI_ISL_635931, EPI_ISL_635932, EPI_ISL_635933, EPI_ISL_635934, EPI_ISL_635935, EPI_ISL_635936, EPI_ISL_635937, EPI_ISL_635938, EPI_ISL_635939, EPI_ISL_635940, EPI_ISL_635941, EPI_ISL_635942, EPI_ISL_635943, EPI_ISL_635944, EPI_ISL_635945, EPI_ISL_635946, EPI_ISL_635947, EPI_ISL_635948, EPI_ISL_635949, EPI_ISL_635950, EPI_ISL_635951, EPI_ISL_635952, EPI_ISL_635953, EPI_ISL_635954, EPI_ISL_635955, EPI_ISL_635956, EPI_ISL_635957, EPI_ISL_635958, EPI_ISL_635959, EPI_ISL_635960, EPI_ISL_635961, EPI_ISL_635962, EPI_ISL_635963, EPI_ISL_635964, EPI_ISL_635965, EPI_ISL_635966, EPI_ISL_635967, EPI_ISL_635968, EPI_ISL_635969, EPI_ISL_635970, EPI_ISL_635971, EPI_ISL_635972, EPI_ISL_635973, EPI_ISL_635974, EPI_ISL_635975, EPI_ISL_635976, EPI_ISL_635977, EPI_ISL_635978, EPI_ISL_635979, EPI_ISL_635980, EPI_ISL_635981, EPI_ISL_635982, EPI_ISL_635985, EPI_ISL_635986, EPI_ISL_635987, EPI_ISL_635988, EPI_ISL_635989, EPI_ISL_635994, EPI_ISL_635995, EPI_ISL_635996, EPI_ISL_635997, EPI_ISL_635998, EPI_ISL_635999, EPI_ISL_636000, EPI_ISL_636001, EPI_ISL_636002, EPI_ISL_636003, EPI_ISL_636004, EPI_ISL_636005, EPI_ISL_636006, EPI_ISL_636007, EPI_ISL_636008, EPI_ISL_636009, EPI_ISL_636010, EPI_ISL_636011, EPI_ISL_636012, EPI_ISL_636013, EPI_ISL_636014, EPI_ISL_636015, EPI_ISL_636016, EPI_ISL_636017, EPI_ISL_636018, EPI_ISL_636019, EPI_ISL_636020, EPI_ISL_636021, EPI_ISL_636023, EPI_ISL_636025, EPI_ISL_636030, EPI_ISL_636031, EPI_ISL_636032, EPI_ISL_636033, EPI_ISL_636034, EPI_ISL_636035, EPI_ISL_636036, EPI_ISL_636037, EPI_ISL_636038, EPI_ISL_636039, EPI_ISL_636040, EPI_ISL_636041, EPI_ISL_636042, EPI_ISL_636043, EPI_ISL_636044, EPI_ISL_636045, EPI_ISL_636046, EPI_ISL_636047, EPI_ISL_636048, EPI_ISL_636049, EPI_ISL_636050, EPI_ISL_636051, EPI_ISL_636052, EPI_ISL_636053, EPI_ISL_636054, EPI_ISL_636055, EPI_ISL_636056, EPI_ISL_636057, EPI_ISL_636058, EPI_ISL_636059, EPI_ISL_636060, EPI_ISL_636061, EPI_ISL_636062, EPI_ISL_636063, EPI_ISL_636064, EPI_ISL_636065, EPI_ISL_636066, EPI_ISL_636067, EPI_ISL_636068, EPI_ISL_636069, EPI_ISL_636070, EPI_ISL_636071, EPI_ISL_636072, EPI_ISL_636073, EPI_ISL_636074, EPI_ISL_636075, EPI_ISL_636076, EPI_ISL_636077, EPI_ISL_636078, EPI_ISL_636079, EPI_ISL_636080, EPI_ISL_636081, EPI_ISL_636082, EPI_ISL_636083, EPI_ISL_636084, EPI_ISL_636085, EPI_ISL_636086, EPI_ISL_636087, EPI_ISL_636088, EPI_ISL_636089, EPI_ISL_636090, EPI_ISL_636091, EPI_ISL_636092, EPI_ISL_636093, EPI_ISL_636094, EPI_ISL_636095, EPI_ISL_636096, EPI_ISL_636097, EPI_ISL_636098, EPI_ISL_636099, EPI_ISL_636100, EPI_ISL_636101, EPI_ISL_636102, EPI_ISL_636103, EPI_ISL_636104, EPI_ISL_636105, EPI_ISL_636106, EPI_ISL_636107, EPI_ISL_636108, EPI_ISL_636109, EPI_ISL_636110, EPI_ISL_636111, EPI_ISL_636112			
see above	San Diego County Public Health Laboratory	Andersen lab at Scripps Research	SEARCH Alliance San Diego with Tracy Basler, Jovan Shephard, Brett Austin
EPI_ISL_636113, EPI_ISL_636114,	Sharp HealthCare Laboratory	Andersen lab at Scripps Research	SEARCH Alliance San Diego with Aaron Harding, Jacquelyn Berumen, Cathy Woerle, Liam McGinnis

EPI_ISL_636116, EPI_ISL_636117, EPI_ISL_636120			
EPI_ISL_636121, EPI_ISL_636122, EPI_ISL_636123, EPI_ISL_636124, EPI_ISL_636125, EPI_ISL_636126, EPI_ISL_636127, EPI_ISL_636128, EPI_ISL_636129, EPI_ISL_636130, EPI_ISL_636131, EPI_ISL_636132, EPI_ISL_636134, EPI_ISL_636135, EPI_ISL_636136, EPI_ISL_636137, EPI_ISL_636138, EPI_ISL_636140, EPI_ISL_636141, EPI_ISL_636142, EPI_ISL_636144, EPI_ISL_636145, EPI_ISL_636146, EPI_ISL_636147, EPI_ISL_636148, EPI_ISL_636149, EPI_ISL_636150, EPI_ISL_636151, EPI_ISL_636152, EPI_ISL_636153, EPI_ISL_636154, EPI_ISL_636155, EPI_ISL_636156, EPI_ISL_636157, EPI_ISL_636158, EPI_ISL_636159, EPI_ISL_636160, EPI_ISL_636161, EPI_ISL_636162, EPI_ISL_636163, EPI_ISL_636164, EPI_ISL_636166, EPI_ISL_636167, EPI_ISL_636171, EPI_ISL_636172, EPI_ISL_636174, EPI_ISL_636175, EPI_ISL_636176, EPI_ISL_636177, EPI_ISL_636180, EPI_ISL_636181, EPI_ISL_636182, EPI_ISL_636183, EPI_ISL_636184, EPI_ISL_636187, EPI_ISL_636188, EPI_ISL_636190, EPI_ISL_636194, EPI_ISL_636195, EPI_ISL_636199, EPI_ISL_636200, EPI_ISL_636203, EPI_ISL_636204, EPI_ISL_636206, EPI_ISL_636207, EPI_ISL_636211, EPI_ISL_636212, EPI_ISL_636213, EPI_ISL_636216, EPI_ISL_636217, EPI_ISL_636218, EPI_ISL_636219, EPI_ISL_636220, EPI_ISL_636221, EPI_ISL_636222, EPI_ISL_636223, EPI_ISL_636224, EPI_ISL_636225, EPI_ISL_636226, EPI_ISL_636227, EPI_ISL_636228, EPI_ISL_636229, EPI_ISL_636230, EPI_ISL_636231, EPI_ISL_636232, EPI_ISL_636233, EPI_ISL_636234, EPI_ISL_636235, EPI_ISL_636236, EPI_ISL_636237, EPI_ISL_636238, EPI_ISL_636239, EPI_ISL_636240, EPI_ISL_636241, EPI_ISL_636242, EPI_ISL_636243, EPI_ISL_636244, EPI_ISL_636245, EPI_ISL_636246, EPI_ISL_636247, EPI_ISL_636248, EPI_ISL_636249, EPI_ISL_636250, EPI_ISL_636251, EPI_ISL_636252, EPI_ISL_636253, EPI_ISL_636254, EPI_ISL_636255, EPI_ISL_636256, EPI_ISL_636257, EPI_ISL_636258, EPI_ISL_636259, EPI_ISL_636260, EPI_ISL_636261, EPI_ISL_636262, EPI_ISL_636263, EPI_ISL_636264, EPI_ISL_636265, EPI_ISL_636266			
see above	San Diego County Public Health Laboratory	Andersen lab at Scripps Research	SEARCH Alliance San Diego with Tracy Basler, Jovan Shephard, Brett Austin
EPI_ISL_636267, EPI_ISL_636268, EPI_ISL_636269, EPI_ISL_636270, EPI_ISL_636271, EPI_ISL_636272, EPI_ISL_636273, EPI_ISL_636274, EPI_ISL_636275, EPI_ISL_636276, EPI_ISL_636277, EPI_ISL_636278, EPI_ISL_636279, EPI_ISL_636280, EPI_ISL_636281, EPI_ISL_636282, EPI_ISL_636283, EPI_ISL_636284, EPI_ISL_636285, EPI_ISL_636286, EPI_ISL_636287, EPI_ISL_636288, EPI_ISL_636289, EPI_ISL_636290, EPI_ISL_636291, EPI_ISL_636292, EPI_ISL_636293, EPI_ISL_636294, EPI_ISL_636295, EPI_ISL_636296, EPI_ISL_636297, EPI_ISL_636298, EPI_ISL_636299, EPI_ISL_636300, EPI_ISL_636301, EPI_ISL_636302, EPI_ISL_636303, EPI_ISL_636304, EPI_ISL_636305, EPI_ISL_636306, EPI_ISL_636307, EPI_ISL_636308, EPI_ISL_636309, EPI_ISL_636310, EPI_ISL_636311, EPI_ISL_636312, EPI_ISL_636313, EPI_ISL_636314, EPI_ISL_636315, EPI_ISL_636316, EPI_ISL_636317, EPI_ISL_636318, EPI_ISL_636319, EPI_ISL_636320, EPI_ISL_636321, EPI_ISL_636322, EPI_ISL_636323, EPI_ISL_636324, EPI_ISL_636325, EPI_ISL_636326, EPI_ISL_636327, EPI_ISL_636328, EPI_ISL_636329, EPI_ISL_636330, EPI_ISL_636331, EPI_ISL_636332, EPI_ISL_636333, EPI_ISL_636334, EPI_ISL_636335, EPI_ISL_636336, EPI_ISL_636337, EPI_ISL_636338, EPI_ISL_636339, EPI_ISL_636340, EPI_ISL_636341, EPI_ISL_636342, EPI_ISL_636343, EPI_ISL_636344, EPI_ISL_636345, EPI_ISL_636346, EPI_ISL_636347, EPI_ISL_636348, EPI_ISL_636349, EPI_ISL_636350, EPI_ISL_636351, EPI_ISL_636352, EPI_ISL_636353, EPI_ISL_636354, EPI_ISL_636355, EPI_ISL_636356, EPI_ISL_636357, EPI_ISL_636358, EPI_ISL_636359, EPI_ISL_636360, EPI_ISL_636361, EPI_ISL_636362, EPI_ISL_636363, EPI_ISL_636364, EPI_ISL_636365, EPI_ISL_636366, EPI_ISL_636367, EPI_ISL_636368, EPI_ISL_636369, EPI_ISL_636370, EPI_ISL_636371, EPI_ISL_636372, EPI_ISL_636373, EPI_ISL_636374, EPI_ISL_636375, EPI_ISL_636376, EPI_ISL_636377, EPI_ISL_636378, EPI_ISL_636379, EPI_ISL_636380, EPI_ISL_636381, EPI_ISL_636382, EPI_ISL_636383, EPI_ISL_636384, EPI_ISL_636385, EPI_ISL_636386, EPI_ISL_636387, EPI_ISL_636388, EPI_ISL_636389, EPI_ISL_636390, EPI_ISL_636391, EPI_ISL_636392, EPI_ISL_636393, EPI_ISL_636394, EPI_ISL_636395, EPI_ISL_636396, EPI_ISL_636397, EPI_ISL_636398, EPI_ISL_636399, EPI_ISL_636400, EPI_ISL_636401, EPI_ISL_636402, EPI_ISL_636403, EPI_ISL_636404, EPI_ISL_636405, EPI_ISL_636406, EPI_ISL_636407, EPI_ISL_636408, EPI_ISL_636409, EPI_ISL_636410, EPI_ISL_636411, EPI_ISL_636412, EPI_ISL_636413, EPI_ISL_636414, EPI_ISL_636415, EPI_ISL_636416, EPI_ISL_636417, EPI_ISL_636418, EPI_ISL_636419, EPI_ISL_636420, EPI_ISL_636421, EPI_ISL_636422, EPI_ISL_636423, EPI_ISL_636424, EPI_ISL_636425, EPI_ISL_636426, EPI_ISL_636427, EPI_ISL_636428, EPI_ISL_636429, EPI_ISL_636430, EPI_ISL_636431, EPI_ISL_636432, EPI_ISL_636433, EPI_ISL_636434, EPI_ISL_636435, EPI_ISL_636436, EPI_ISL_636437, EPI_ISL_636438, EPI_ISL_636439, EPI_ISL_636440, EPI_ISL_636441, EPI_ISL_636442, EPI_ISL_636443, EPI_ISL_636444, EPI_ISL_636445, EPI_ISL_636446, EPI_ISL_636447, EPI_ISL_636448, EPI_ISL_636449, EPI_ISL_636450, EPI_ISL_636451, EPI_ISL_636452, EPI_ISL_636453, EPI_ISL_636454, EPI_ISL_636455, EPI_ISL_636456, EPI_ISL_636457, EPI_ISL_636458			
see above	Biolab Diagnostic Laboratories	Andersen lab at Scripps Research	Issa Abu-Dayyeh, Ahmad Tibi, Lama Hussein, Lina Mohammad, Zein Naber, Amid Abdelnour with SEARCH Alliance San Diego
EPI_ISL_636465, EPI_ISL_636466	ULSS6 Euganea	Istituto Zooprofilattico Sperimentale delle Venezie	Adelaide Milani, Alessia Schivo, Annalisa Salviato, Erika Giorgia Quaranta, Ambra Pastori, Bianca Zecchin, Alice Fusaro, Isabella Monne, Calogero Terregino, Antonia Ricci
EPI_ISL_636491, EPI_ISL_636514, EPI_ISL_636515, EPI_ISL_636516, EPI_ISL_636517, EPI_ISL_636518, EPI_ISL_636519, EPI_ISL_636520, EPI_ISL_636521, EPI_ISL_636533, EPI_ISL_636534, EPI_ISL_636535, EPI_ISL_636536, EPI_ISL_636537, EPI_ISL_636538, EPI_ISL_636548, EPI_ISL_636549, EPI_ISL_636550, EPI_ISL_636551, EPI_ISL_636556, EPI_ISL_636557, EPI_ISL_636558, EPI_ISL_636559, EPI_ISL_636560, EPI_ISL_636566, EPI_ISL_636567, EPI_ISL_636568, EPI_ISL_636569, EPI_ISL_636570, EPI_ISL_636571, EPI_ISL_636576, EPI_ISL_636577, EPI_ISL_636578, EPI_ISL_636579, EPI_ISL_636580, EPI_ISL_636581, EPI_ISL_636582, EPI_ISL_636583, EPI_ISL_636584, EPI_ISL_636585, EPI_ISL_636587, EPI_ISL_636588, EPI_ISL_636589, EPI_ISL_636590, EPI_ISL_636591, EPI_ISL_636600, EPI_ISL_636601, EPI_ISL_636602, EPI_ISL_636603			
see above	Dutch COVID-19 response team	National Institute for Public Health and the Environment (RIVM)	Adam Meijer, Harry Vennema, Jeroen Cremer, Sharon van den Brink, Bas van der Veer, AnneMarie van den Brandt, Florian Zwagemaker, Dennis Schmitz, Chantal Reusken, on behalf of the national COVID-19 response team
EPI_ISL_636604	Lithuanian University of Health Sciences Hospital, Department of Laboratory Medicine	Lithuanian University of Health Sciences, Molecular cardiology lab.	Lukas Zemaitis, Ingrida Olendrait, Arnoldas Pautienius, Kamile Tamusauskaite, Dovydas Gecys, Laura Pareckaite, Vaiva Lesauskaite, Astra Vitkauskiene
EPI_ISL_636605, EPI_ISL_636606, EPI_ISL_636607, EPI_ISL_636608, EPI_ISL_636609, EPI_ISL_636610, EPI_ISL_636611, EPI_ISL_636612, EPI_ISL_636613, EPI_ISL_636614, EPI_ISL_636615, EPI_ISL_636616, EPI_ISL_636617, EPI_ISL_636618, EPI_ISL_636619, EPI_ISL_636620, EPI_ISL_636621, EPI_ISL_636622, EPI_ISL_636623, EPI_ISL_636624, EPI_ISL_636625, EPI_ISL_636626, EPI_ISL_636627, EPI_ISL_636628, EPI_ISL_636629, EPI_ISL_636630, EPI_ISL_636631, EPI_ISL_636632, EPI_ISL_636633, EPI_ISL_636634, EPI_ISL_636635, EPI_ISL_636636, EPI_ISL_636637, EPI_ISL_636638, EPI_ISL_636639, EPI_ISL_636640, EPI_ISL_636641, EPI_ISL_636642, EPI_ISL_636643, EPI_ISL_636644, EPI_ISL_636645, EPI_ISL_636646, EPI_ISL_636647, EPI_ISL_636648, EPI_ISL_636649, EPI_ISL_636650, EPI_ISL_636651, EPI_ISL_636652, EPI_ISL_636653, EPI_ISL_636654, EPI_ISL_636655, EPI_ISL_636656, EPI_ISL_636657, EPI_ISL_636658, EPI_ISL_636659, EPI_ISL_636660, EPI_ISL_636661, EPI_ISL_636662, EPI_ISL_636663, EPI_ISL_636664, EPI_ISL_636665, EPI_ISL_636666, EPI_ISL_636667, EPI_ISL_636668, EPI_ISL_636669, EPI_ISL_636670, EPI_ISL_636671, EPI_ISL_636672, EPI_ISL_636673, EPI_ISL_636674, EPI_ISL_636675, EPI_ISL_636676, EPI_ISL_636677, EPI_ISL_636678, EPI_ISL_636679, EPI_ISL_636680, EPI_ISL_636681, EPI_ISL_636682, EPI_ISL_636683, EPI_ISL_636684, EPI_ISL_636685			
see above	Department of Clinical Microbiology	GIGA Medical Genomics	Keith Durkin, Maria Artesi, Sébastien Bontems, Raphaël Boreux, Bouchra Boujemla, Cécile Meex, Pierrette Melin, Marie-Pierre Hayette, Vincent Bours
EPI_ISL_636688, EPI_ISL_636689, EPI_ISL_636690, EPI_ISL_636691, EPI_ISL_636692, EPI_ISL_636693, EPI_ISL_636694, EPI_ISL_636695, EPI_ISL_636696, EPI_ISL_636697, EPI_ISL_636698, EPI_ISL_636699, EPI_ISL_636700, EPI_ISL_636701, EPI_ISL_636702, EPI_ISL_636703, EPI_ISL_636705, EPI_ISL_636706, EPI_ISL_636707, EPI_ISL_636708, EPI_ISL_636709, EPI_ISL_636710, EPI_ISL_636711, EPI_ISL_636712, EPI_ISL_636713, EPI_ISL_636714, EPI_ISL_636715, EPI_ISL_636716, EPI_ISL_636719, EPI_ISL_636720, EPI_ISL_636721, EPI_ISL_636722, EPI_ISL_636723, EPI_ISL_636725, EPI_ISL_636726, EPI_ISL_636727, EPI_ISL_636729, EPI_ISL_636730, EPI_ISL_636732, EPI_ISL_636733, EPI_ISL_636734, EPI_ISL_636735			
see above	Respiratory Virus Unit, Microbiology Services Colindale, Public Health England	Respiratory Virus Unit, Microbiology Services Colindale, Public Health England	PHE Covid Sequencing Team
EPI_ISL_636739, EPI_ISL_636740, EPI_ISL_636741, EPI_ISL_636742, EPI_ISL_636743, EPI_ISL_636746, EPI_ISL_636747, EPI_ISL_636748, EPI_ISL_636749, EPI_ISL_636750, EPI_ISL_636752, EPI_ISL_636753, EPI_ISL_636754, EPI_ISL_636755, EPI_ISL_636756, EPI_ISL_636757, EPI_ISL_636758, EPI_ISL_636759, EPI_ISL_636760, EPI_ISL_636761, EPI_ISL_636762, EPI_ISL_636766, EPI_ISL_636768, EPI_ISL_636770, EPI_ISL_636774, EPI_ISL_636776, EPI_ISL_636777, EPI_ISL_636778, EPI_ISL_636779, EPI_ISL_636780, EPI_ISL_636781, EPI_ISL_636784, EPI_ISL_636785, EPI_ISL_636787, EPI_ISL_636788, EPI_ISL_636789, EPI_ISL_636790, EPI_ISL_636791, EPI_ISL_636792, EPI_ISL_636795, EPI_ISL_636797, EPI_ISL_636798, EPI_ISL_636801, EPI_ISL_636802, EPI_ISL_636804, EPI_ISL_636805, EPI_ISL_636806, EPI_ISL_636809, EPI_ISL_636810, EPI_ISL_636811, EPI_ISL_636812, EPI_ISL_636813, EPI_ISL_636815, EPI_ISL_636816, EPI_ISL_636817, EPI_ISL_636818, EPI_ISL_636819, EPI_ISL_636820, EPI_ISL_636821, EPI_ISL_636822, EPI_ISL_636823, EPI_ISL_636824, EPI_ISL_636825, EPI_ISL_636828, EPI_ISL_636829, EPI_ISL_636830, EPI_ISL_636831, EPI_ISL_636832			
see above	National Centre for Disease control (NCDC)	NCDC/CSIR-IGIB	Mahesh S. Dhar1*, Bharathram Uppili2*, Robin Marwal1*, Pooja Sharma2*, RadhaKrishnan VS, Vivekanand A, Nishu Tyagi, Shaista Khan, Simmi Tiwari, Manish Kumar, Ajit Shewale, Ishtaq Ahmed, Asangla Kamai, Aparna Swaminathan, Saruchi Wadhwa, Tushar Nale, Sandhya Kabra, Sujeet Singh, Mohammed Faruq#, Anurag Agrawal#, Partha Rakshit#
EPI_ISL_636839, EPI_ISL_636840, EPI_ISL_636841, EPI_ISL_636842, EPI_ISL_636843, EPI_ISL_636844, EPI_ISL_636845, EPI_ISL_636846, EPI_ISL_636847, EPI_ISL_636848, EPI_ISL_636849, EPI_ISL_636850, EPI_ISL_636851, EPI_ISL_636852, EPI_ISL_636853, EPI_ISL_636854, EPI_ISL_636855, EPI_ISL_636856, EPI_ISL_636857, EPI_ISL_636858, EPI_ISL_636859, EPI_ISL_636860, EPI_ISL_636861, EPI_ISL_636862, EPI_ISL_636863, EPI_ISL_636864, EPI_ISL_636865, EPI_ISL_636866, EPI_ISL_636867, EPI_ISL_636868, EPI_ISL_636869, EPI_ISL_636870, EPI_ISL_636871, EPI_ISL_636872, EPI_ISL_636873, EPI_ISL_636874, EPI_ISL_636875, EPI_ISL_636876, EPI_ISL_636877, EPI_ISL_636878, EPI_ISL_636879, EPI_ISL_636880, EPI_ISL_636881, EPI_ISL_636882, EPI_ISL_636883, EPI_ISL_636884, EPI_ISL_636885, EPI_ISL_636886, EPI_ISL_636887, EPI_ISL_636888, EPI_ISL_636889, EPI_ISL_636890, EPI_ISL_636891, EPI_ISL_636892, EPI_ISL_636893, EPI_ISL_636894, EPI_ISL_636895, EPI_ISL_636896, EPI_ISL_636897, EPI_ISL_636898, EPI_ISL_636899, EPI_ISL_636900, EPI_ISL_636901, EPI_ISL_636902, EPI_ISL_636903, EPI_ISL_636904, EPI_ISL_636905, EPI_ISL_636906			
see above	Lithuanian University of Health Sciences Hospital, Department of Laboratory Medicine	Lithuanian University of Health Sciences, Molecular cardiology lab.	Lukas Zemaitis, Ingrida Olendrait, Arnoldas Pautienius, Kamile Tamusauskaite, Dovydas Gecys, Laura Pareckaite, Vaiva Lesauskaite, Astra Vitkauskiene
EPI_ISL_636965	Pathogen Genomics Lab King Abdullah University of Science and Technology(KAUST)	Pathogen Genomics Lab King Abdullah University of Science and Technology(KAUST)	Raeece Naeem, Rahul P Salunke, Sharif Hala, Sara Mfarrej, Amit Kumar Subudhi, Fadwa Alofi, Fathia Ben Rached, Afrah Alsomali, Asim Khogeer, Ahmad Bakur Mahmoud, Anwar Hashem, Naif Almontashiri, Arnab Pain
EPI_ISL_636966, EPI_ISL_636967, EPI_ISL_636968	Pathogen Genomics Lab King Abdullah University of Science and Technology(KAUST)	Pathogen Genomics Lab King Abdullah University of Science and Technology(KAUST)	Fathia Ben Rached, Raeece Naeem, Sharif Hala, Fadwa Alofi, Rahul P Salunke, Sara Mfarrej, Amit Kumar Subudhi, Afrah Alsomali, Asim Khogeer, Ahmad Bakur Mahmoud, Anwar Hashem, Naif Almontashiri, Arnab Pain
EPI_ISL_636973	Public Health Lab	Public Health Lab	Alwasti, H
EPI_ISL_636975, EPI_ISL_636977	HP Pemba	KRISP, KZN Research Innovation and Sequencing Platform	Ismael N, Giandhari J, Pillay S, Tegally H, Wilkinson E, de Oliveira T, Nadia Sitoe, Paulo Arnaldo, Nedio Mabunda
EPI_ISL_636980	CS Xai Xai	KRISP, KZN Research Innovation and Sequencing Platform	Ismael N, Giandhari J, Pillay S, Tegally H, Wilkinson E, de Oliveira T, Nadia Sitoe, Paulo Arnaldo, Nedio Mabunda
EPI_ISL_636995, EPI_ISL_636996, EPI_ISL_636997, EPI_ISL_636998, EPI_ISL_636999, EPI_ISL_637000, EPI_ISL_637001, EPI_ISL_637002, EPI_ISL_637003, EPI_ISL_637004, EPI_ISL_637005, EPI_ISL_637006, EPI_ISL_637007, EPI_ISL_637008, EPI_ISL_637009, EPI_ISL_637010, EPI_ISL_637011, EPI_ISL_637012, EPI_ISL_637013, EPI_ISL_637014, EPI_ISL_637015, EPI_ISL_637016, EPI_ISL_637017, EPI_ISL_637018, EPI_ISL_637019, EPI_ISL_637020, EPI_ISL_637021			
see above	Department of Infectious Diseases and Immunology, National Hospital Organization Nagoya Medical Center	Clinical Research Center, National Hospital Organization Nagoya Medical Center	Yoshihiro Nakata, Hirotaka Ode, Mai Kubota, Masakazu Matsuda, Kazuhiro Matsuoka, Miho Nakasuji, Mikiko Mori, Mayumi Imahashi, Yoshiyuki Yokomaku, Yasumasa Iwatani
EPI_ISL_637084	LabTests	Institute of Environmental Science and Research (ESR)	Xiaoyun Ren, Matt Storey, Nikki Freed, Muhammad Faisal, Jing Wang, Hermes Perez, Anja Werno, Antje van der Linden, Arlo Upton, Chris Mansell, David Hammer, Dragana Drinkovic, Gary McAuliffe, Hana Sofia Andersson, James Ussher, Jill Sherwood, Josh Freeman, Julia Howard, Juliet Elvy, Mary DeAlmeida, Matt Blakiston, Matthew Rogers, Max Bloomfield, Michael Addidle, Michelle Balm, Sally Roberts, Sarah Jefferies, Sharmini Muttaiyah, Susan Morpeth, Susan Taylor, Timothy Blackmore, Vani Sathyendran, Veronica Playle, Virginia Hope, Erasmus Smit, Lauren Jelly, Olin Silander, Joep de Ligt
EPI_ISL_637085	Wellington SCL (WN)	Institute of Environmental Science and Research (ESR)	Xiaoyun Ren, Matt Storey, Nikki Freed, Muhammad Faisal, Jing Wang, Hermes Perez, Anja Werno, Antje van der Linden, Arlo Upton, Chris Mansell, David Hammer, Dragana Drinkovic, Gary McAuliffe, Hana Sofia Andersson, James Ussher, Jill Sherwood, Josh Freeman, Julia Howard, Juliet Elvy, Mary DeAlmeida, Matt Blakiston, Matthew Rogers, Max Bloomfield, Michael Addidle, Michelle Balm, Sally Roberts, Sarah Jefferies, Sharmini Muttaiyah, Susan Morpeth, Susan Taylor, Timothy Blackmore, Vani Sathyendran, Veronica Playle, Virginia Hope, Erasmus Smit, Lauren Jelly, Olin Silander, Joep de Ligt

EPI_ISL_637086, EPI_ISL_637087	Middlemore Hospital	Institute of Environmental Science and Research (ESR)	Xiaoyun Ren, Matt Storey, Nikki Freed, Muhammad Faisal, Jing Wang, Hermes Perez, Anja Werno, Antje van der Linden, Arlo Upton, Chris Mansell, David Hammer, Dragana Drinkovic, Gary McAuliffe, Hana Sofia Andersson, James Ussher, Jill Sherwood, Josh Freeman, Julia Howard, Juliet Elvy, Mary DeAlmeida, Matt Blakiston, Matthew Rogers, Max Bloomfield, Michael Addidle, Michelle Balm, Sally Roberts, Sarah Jefferies, Sharmini Muttaiyah, Susan Morpeth, Susan Taylor, Timothy Blackmore, Vani Sathyendran, Veronica Playle, Virginia Hope, Erasmus Smit, Lauren Jelly, Olin Silander, Joep de Ligt
EPI_ISL_637088	PathLab Bay of Plenty	Institute of Environmental Science and Research (ESR)	Xiaoyun Ren, Matt Storey, Nikki Freed, Muhammad Faisal, Jing Wang, Hermes Perez, Anja Werno, Antje van der Linden, Arlo Upton, Chris Mansell, David Hammer, Dragana Drinkovic, Gary McAuliffe, Hana Sofia Andersson, James Ussher, Jill Sherwood, Josh Freeman, Julia Howard, Juliet Elvy, Mary DeAlmeida, Matt Blakiston, Matthew Rogers, Max Bloomfield, Michael Addidle, Michelle Balm, Sally Roberts, Sarah Jefferies, Sharmini Muttaiyah, Susan Morpeth, Susan Taylor, Timothy Blackmore, Vani Sathyendran, Veronica Playle, Virginia Hope, Erasmus Smit, Lauren Jelly, Olin Silander, Joep de Ligt
EPI_ISL_637089, EPI_ISL_637091, EPI_ISL_637093, EPI_ISL_637094, EPI_ISL_637095	LabPLUS	Institute of Environmental Science and Research (ESR)	Xiaoyun Ren, Matt Storey, Nikki Freed, Muhammad Faisal, Jing Wang, Hermes Perez, Anja Werno, Antje van der Linden, Arlo Upton, Chris Mansell, David Hammer, Dragana Drinkovic, Gary McAuliffe, Hana Sofia Andersson, James Ussher, Jill Sherwood, Josh Freeman, Julia Howard, Juliet Elvy, Mary DeAlmeida, Matt Blakiston, Matthew Rogers, Max Bloomfield, Michael Addidle, Michelle Balm, Sally Roberts, Sarah Jefferies, Sharmini Muttaiyah, Susan Morpeth, Susan Taylor, Timothy Blackmore, Vani Sathyendran, Veronica Playle, Virginia Hope, Erasmus Smit, Lauren Jelly, Olin Silander, Joep de Ligt
EPI_ISL_637096	LabTests	Institute of Environmental Science and Research (ESR)	Xiaoyun Ren, Matt Storey, Nikki Freed, Muhammad Faisal, Jing Wang, Hermes Perez, Anja Werno, Antje van der Linden, Arlo Upton, Chris Mansell, David Hammer, Dragana Drinkovic, Gary McAuliffe, Hana Sofia Andersson, James Ussher, Jill Sherwood, Josh Freeman, Julia Howard, Juliet Elvy, Mary DeAlmeida, Matt Blakiston, Matthew Rogers, Max Bloomfield, Michael Addidle, Michelle Balm, Sally Roberts, Sarah Jefferies, Sharmini Muttaiyah, Susan Morpeth, Susan Taylor, Timothy Blackmore, Vani Sathyendran, Veronica Playle, Virginia Hope, Erasmus Smit, Lauren Jelly, Olin Silander, Joep de Ligt
EPI_ISL_637097	Respiratory Virus Unit, Microbiology Services Colindale, Public Health England	Respiratory Virus Unit, Microbiology Services Colindale, Public Health England	PHE Covid Sequencing Team
EPI_ISL_637110, EPI_ISL_637112, EPI_ISL_637113	Rafik Hariri University Hospital	Microbial Pathogenomics Lab	Georgi Merhi, Tamara Salloum, Rita Feghali, Sima Tokajian
EPI_ISL_637114, EPI_ISL_637115, EPI_ISL_637116, EPI_ISL_637117, EPI_ISL_637118, EPI_ISL_637119, EPI_ISL_637120, EPI_ISL_637121, EPI_ISL_637122, EPI_ISL_637123, EPI_ISL_637124, EPI_ISL_637125, EPI_ISL_637126, EPI_ISL_637127, EPI_ISL_637128, EPI_ISL_637129, EPI_ISL_637130, EPI_ISL_637131, EPI_ISL_637132, EPI_ISL_637133, EPI_ISL_637134, EPI_ISL_637135, EPI_ISL_637136, EPI_ISL_637137, EPI_ISL_637138, EPI_ISL_637139, EPI_ISL_637140, EPI_ISL_637141, EPI_ISL_637143, EPI_ISL_637144, EPI_ISL_637145, EPI_ISL_637146, EPI_ISL_637147, EPI_ISL_637148, EPI_ISL_637149, EPI_ISL_637150, EPI_ISL_637151, EPI_ISL_637152, EPI_ISL_637154, EPI_ISL_637155, EPI_ISL_637156, EPI_ISL_637157, EPI_ISL_637158, EPI_ISL_637159, EPI_ISL_637160, EPI_ISL_637161, EPI_ISL_637162, EPI_ISL_637163, EPI_ISL_637164, EPI_ISL_637165, EPI_ISL_637166, EPI_ISL_637167, EPI_ISL_637168, EPI_ISL_637169, EPI_ISL_637170, EPI_ISL_637171, EPI_ISL_637172, EPI_ISL_637173, EPI_ISL_637174, EPI_ISL_637175, EPI_ISL_637176, EPI_ISL_637177, EPI_ISL_637178, EPI_ISL_637179, EPI_ISL_637180, EPI_ISL_637181, EPI_ISL_637182, EPI_ISL_637183, EPI_ISL_637184			
see above	Respiratory Virus Unit, Microbiology Services Colindale, Public Health England	COVID-19 Genomics UK (COG-UK) Consortium	PHE Covid Sequencing Team
EPI_ISL_637256	Wales Specialist Virology Centre Sequencing lab: Pathogen Genomics Unit	COVID-19 Genomics UK (COG-UK) Consortium	Catherine Moore, Johnathan Evans, Laura Gifford, Malorie Perry, Simon Cottrell, Angela Marchbank, Alec Birchley, Alexander Adams, Amy Gaskin, Bree Gatica-Wilcox, Jason Coombes, Joel Southgate, Lauren Gilbert, Lee Graham, Nicole Pacchiarini, Sara Kumziene-Summerhayes, Sarah Taylor, Sophie Jones, Sara Rey, Matthew Bull, Joanne Watkins, Sally Corden, Tom Connor
EPI_ISL_637257	Liverpool Clinical Laboratories	COVID-19 Genomics UK (COG-UK) Consortium	Sam Haldenby, Anita Lucaci, Steve Paterson, Julian Hiscox, Alistair Darby, M Almsaud, A Alrezaihi, Muhannad Alruwaili, Stuart D Armstrong, Jones Benjamin, Eleanor G Bentley, Anu Chawla, Jordan J Clark, Angela Cowell, Richard Eccles, Isabel García-Dorival, Matthew Gemmell, Alessandro Gerada, PKF Gilmore, Richard Gregory, Ximeng Han, Catherine Hartley, Margaret Hughes, Miren Iturriza-Gomara, James Johnson, L Luu, Jenifer Manson, Charlotte Nelson, Elaine O'Toole, Cassie Olateju, Rebekah Penrice-Randal , Lucille Rainbow, N.P Randle, Trevor Ian Robinson, Parul Sharma, Ghada T Shawli, James P Stewart, Neil Swainston, Ecaterina Vamos, Joanne Watts, Mark Whitehead
EPI_ISL_637258	Wales Specialist Virology Centre Sequencing lab: Pathogen Genomics Unit	COVID-19 Genomics UK (COG-UK) Consortium	Catherine Moore, Johnathan Evans, Laura Gifford, Malorie Perry, Simon Cottrell, Angela Marchbank, Alec Birchley, Alexander Adams, Amy Gaskin, Bree Gatica-Wilcox, Jason Coombes, Joel Southgate, Lauren Gilbert, Lee Graham, Nicole Pacchiarini, Sara Kumziene-Summerhayes, Sarah Taylor, Sophie Jones, Sara Rey, Matthew Bull, Joanne Watkins, Sally Corden, Tom Connor
EPI_ISL_637259	Liverpool Clinical Laboratories	COVID-19 Genomics UK (COG-UK) Consortium	Sam Haldenby, Anita Lucaci, Steve Paterson, Julian Hiscox, Alistair Darby, M Almsaud, A Alrezaihi, Muhannad Alruwaili, Stuart D Armstrong, Jones Benjamin, Eleanor G Bentley, Anu Chawla, Jordan J Clark, Angela Cowell, Richard Eccles, Isabel García-Dorival, Matthew Gemmell, Alessandro Gerada, PKF Gilmore, Richard Gregory, Ximeng Han, Catherine Hartley, Margaret Hughes, Miren Iturriza-Gomara, James Johnson, L Luu, Jenifer Manson, Charlotte Nelson, Elaine O'Toole, Cassie Olateju, Rebekah Penrice-Randal , Lucille Rainbow, N.P Randle, Trevor Ian Robinson, Parul Sharma, Ghada T Shawli, James P Stewart, Neil Swainston, Ecaterina Vamos, Joanne Watts, Mark Whitehead
EPI_ISL_637260, EPI_ISL_637261	Department of Pathology, University of Cambridge	COVID-19 Genomics UK (COG-UK) Consortium	Aminu S. Jahun, Yasmin Chaudhry, Grant Hall, Iliana Georgana, Myra Hosmillo, Martin D. Curran, Malte Pinckert, Surendra Parmar, Ian Goodfellow
EPI_ISL_637262, EPI_ISL_637263	Liverpool Clinical Laboratories	COVID-19 Genomics UK (COG-UK) Consortium	Sam Haldenby, Anita Lucaci, Steve Paterson, Julian Hiscox, Alistair Darby, M Almsaud, A Alrezaihi, Muhannad Alruwaili, Stuart D Armstrong, Jones Benjamin, Eleanor G Bentley, Anu Chawla, Jordan J Clark, Angela Cowell, Richard Eccles, Isabel García-Dorival, Matthew Gemmell, Alessandro Gerada, PKF Gilmore, Richard Gregory, Ximeng Han, Catherine Hartley, Margaret Hughes, Miren Iturriza-Gomara, James Johnson, L Luu, Jenifer Manson, Charlotte Nelson, Elaine O'Toole, Cassie Olateju, Rebekah Penrice-Randal , Lucille Rainbow, N.P Randle, Trevor Ian Robinson, Parul Sharma, Ghada T Shawli, James P Stewart, Neil Swainston, Ecaterina Vamos, Joanne Watts, Mark Whitehead
EPI_ISL_637264	Department of Pathology, University of Cambridge	COVID-19 Genomics UK (COG-UK) Consortium	Aminu S. Jahun, Yasmin Chaudhry, Grant Hall, Iliana Georgana, Myra Hosmillo, Martin D. Curran, Malte Pinckert, Surendra Parmar, Ian Goodfellow
EPI_ISL_637265	Liverpool Clinical Laboratories	COVID-19 Genomics UK (COG-UK) Consortium	Sam Haldenby, Anita Lucaci, Steve Paterson, Julian Hiscox, Alistair Darby, M Almsaud, A Alrezaihi, Muhannad Alruwaili, Stuart D Armstrong, Jones Benjamin, Eleanor G Bentley, Anu Chawla, Jordan J Clark, Angela Cowell, Richard Eccles, Isabel García-Dorival, Matthew Gemmell, Alessandro Gerada, PKF Gilmore, Richard Gregory, Ximeng Han, Catherine Hartley, Margaret Hughes, Miren Iturriza-Gomara, James Johnson, L Luu, Jenifer Manson, Charlotte Nelson, Elaine O'Toole, Cassie Olateju, Rebekah Penrice-Randal , Lucille Rainbow, N.P Randle, Trevor Ian Robinson, Parul Sharma, Ghada T Shawli, James P Stewart, Neil Swainston, Ecaterina Vamos, Joanne Watts, Mark Whitehead
EPI_ISL_637266	Virology Department, Sheffield Teaching Hospitals NHS Foundation Trust/Department of Infection, Immunity and Cardiovascular Disease, The Medical School, University of Sheffield	COVID-19 Genomics UK (COG-UK) Consortium	Thushan de Silva, Matthew Parker, Nikki Smith, Adri Angyal, Rebecca Brown, Luke Green, Rachel Tucker, Paul Parsons, Danielle Groves, Katie Johnson, Laura Carrilero, Alex Keeley, Dave Partridge, Matthew Wyles, Benjamin Lindsey, Mehmet Yavuz, Mohammad Raza, Cariad Evans
EPI_ISL_637267, EPI_ISL_637268, EPI_ISL_637269, EPI_ISL_637270, EPI_ISL_637271, EPI_ISL_637272	Department of Pathology, University of Cambridge	COVID-19 Genomics UK (COG-UK) Consortium	Aminu S. Jahun, Yasmin Chaudhry, Grant Hall, Iliana Georgana, Myra Hosmillo, Martin D. Curran, Malte Pinckert, Surendra Parmar, Ian Goodfellow
EPI_ISL_637273	Virology Department, Sheffield Teaching Hospitals NHS Foundation Trust/Department of Infection, Immunity and Cardiovascular Disease, The Medical School, University of Sheffield	COVID-19 Genomics UK (COG-UK) Consortium	Thushan de Silva, Matthew Parker, Nikki Smith, Adri Angyal, Rebecca Brown, Luke Green, Rachel Tucker, Paul Parsons, Danielle Groves, Katie Johnson, Laura Carrilero, Alex Keeley, Dave Partridge, Matthew Wyles, Benjamin Lindsey, Mehmet Yavuz, Mohammad Raza, Cariad Evans
EPI_ISL_637274	Liverpool Clinical Laboratories	COVID-19 Genomics UK (COG-UK) Consortium	Sam Haldenby, Anita Lucaci, Steve Paterson, Julian Hiscox, Alistair Darby, M Almsaud, A Alrezaihi, Muhannad Alruwaili, Stuart D Armstrong, Jones Benjamin, Eleanor G Bentley, Anu Chawla, Jordan J Clark, Angela Cowell, Richard Eccles, Isabel García-Dorival, Matthew Gemmell, Alessandro Gerada, PKF Gilmore, Richard Gregory, Ximeng Han, Catherine Hartley, Margaret Hughes, Miren Iturriza-Gomara, James Johnson, L Luu, Jenifer Manson, Charlotte Nelson, Elaine O'Toole, Cassie Olateju, Rebekah Penrice-Randal , Lucille Rainbow, N.P Randle, Trevor Ian Robinson, Parul Sharma, Ghada T Shawli, James P Stewart, Neil Swainston, Ecaterina Vamos, Joanne Watts, Mark Whitehead
EPI_ISL_637275, EPI_ISL_637276, EPI_ISL_637277	Department of Pathology, University of Cambridge	COVID-19 Genomics UK (COG-UK) Consortium	Aminu S. Jahun, Yasmin Chaudhry, Grant Hall, Iliana Georgana, Myra Hosmillo, Martin D. Curran, Malte Pinckert, Surendra Parmar, Ian Goodfellow
EPI_ISL_637278, EPI_ISL_637279	Centre for Enzyme Innovation, University of Portsmouth / Translational Research Laboratory, Portsmouth Hospitals NHS Trust	COVID-19 Genomics UK (COG-UK) Consortium	Angela Beckett,Yann Bourgeois,Garry Scarlett,Sharon Glaysher,Scott Elliott,Kelly Bicknell,Robert Impey,Allyson Lloyd,Sarah Wyllie,Ethan Butcher,Anoop Chauhan,Samuel Robson
EPI_ISL_637280	Northumbria University / South Tees Hospitals NHS	COVID-19 Genomics UK (COG-UK) Consortium	Darren L Smith,Andrew Nelson,Matthew Bashton,Greg R Young,Joshua Loh,John Allan,Mohammad A Tariq,Giles S Holt,Gary Black,Wen C Yew,Lynn

	Foundation Trust / North Cumbria Integrated Care NHS Foundation Trust / North Tees and Hartlepool NHS Foundation Trust / Newcastle Hospitals NHS Foundation Trust		Dover,Paul Baker,Steve Liggett,Sarah Essex,Jane Greenaway,Debra Padgett,Clive Graham,Garren Scott,Edward Barton,Emma Swindells,Brendan Payne,Jennifer Collins,Yusri Taha,Gary Eltringham
EPI_ISL_637281, EPI_ISL_637282	Department of Pathology, University of Cambridge	COVID-19 Genomics UK (COG-UK) Consortium	Aminu S. Jahun, Yasmin Chaudhry, Grant Hall, Iliana Georgana, Myra Hosmillo, Martin D. Curran, Malte Pinckert, Surendra Parmar, Ian Goodfellow
EPI_ISL_637283	Northumbria University / South Tees Hospitals NHS Foundation Trust / North Cumbria Integrated Care NHS Foundation Trust / North Tees and Hartlepool NHS Foundation Trust / Newcastle Hospitals NHS Foundation Trust	COVID-19 Genomics UK (COG-UK) Consortium	Darren L Smith,Andrew Nelson,Matthew Bashton,Greg R Young,Joshua Loh,John Allan,Mohammad A Tariq,Giles S Holt,Gary Black,Wen C Yew,Lynn Dover,Paul Baker,Steve Liggett,Sarah Essex,Jane Greenaway,Debra Padgett,Clive Graham,Garren Scott,Edward Barton,Emma Swindells,Brendan Payne,Jennifer Collins,Yusri Taha,Gary Eltringham
EPI_ISL_637284	Quadram Institute Bioscience	COVID-19 Genomics UK (COG-UK) Consortium	Dave J. Baker, Gemma L. Kay, Alp Aydin, Thanh Le-Viet, Steven Rudder, Ana P. Tedim, Anastasia Kolyva, Maria Diaz, Leonardo de Oliveira Martins, Nabil-Fareed Alikhan, Lizzie Meadows, Rachael Stanley, Ngozi Elumogo, Muhammed Yasir, Nicholas M. Thomson, Alexander J Trotter, Rachel Gilroy, Samuel Bloomfield, Claire Stuart, Andrew Bell, Reenesh Prakash, Samir Dervisevic, Alison E. Mather, John Wain, Mark Webber, Andrew J. Page, Justin O'Grady
EPI_ISL_637285	Virology Department, Sheffield Teaching Hospitals NHS Foundation Trust/Department of Infection, Immunity and Cardiovascular Disease, The Medical School, University of Sheffield	COVID-19 Genomics UK (COG-UK) Consortium	Thushan de Silva, Matthew Parker, Nikki Smith, Adri Angyal, Rebecca Brown, Luke Green, Rachel Tucker, Paul Parsons, Danielle Groves, Katie Johnson, Laura Carrilero, Alex Keeley, Dave Partridge, Matthew Wyles, Benjamin Lindsey, Mehmet Yavuz, Mohammad Raza, Cariad Evans
EPI_ISL_637286	Quadram Institute Bioscience	COVID-19 Genomics UK (COG-UK) Consortium	Dave J. Baker, Gemma L. Kay, Alp Aydin, Thanh Le-Viet, Steven Rudder, Ana P. Tedim, Anastasia Kolyva, Maria Diaz, Leonardo de Oliveira Martins, Nabil-Fareed Alikhan, Lizzie Meadows, Rachael Stanley, Ngozi Elumogo, Muhammed Yasir, Nicholas M. Thomson, Alexander J Trotter, Rachel Gilroy, Samuel Bloomfield, Claire Stuart, Andrew Bell, Reenesh Prakash, Samir Dervisevic, Alison E. Mather, John Wain, Mark Webber, Andrew J. Page, Justin O'Grady
EPI_ISL_637287	Department of Pathology, University of Cambridge	COVID-19 Genomics UK (COG-UK) Consortium	Aminu S. Jahun, Yasmin Chaudhry, Grant Hall, Iliana Georgana, Myra Hosmillo, Martin D. Curran, Malte Pinckert, Surendra Parmar, Ian Goodfellow
EPI_ISL_637288, EPI_ISL_637289	Wales Specialist Virology Centre Sequencing lab: Pathogen Genomics Unit	COVID-19 Genomics UK (COG-UK) Consortium	Catherine Moore, Johnathan Evans, Laura Gifford, Malorie Perry, Simon Cottrell, Angela Marchbank, Alec Birchley, Alexander Adams, Amy Gaskin, Bree Gatica-Wilcox, Jason Coombes, Joel Southgate, Lauren Gilbert, Lee Graham, Nicole Pacchiarini, Sara Kumziene-Summerhayes, Sarah Taylor, Sophie Jones, Sara Rey, Matthew Bull, Joanne Watkins, Sally Corden, Tom Connor
EPI_ISL_637290, EPI_ISL_637291	Virology Department, Sheffield Teaching Hospitals NHS Foundation Trust/Department of Infection, Immunity and Cardiovascular Disease, The Medical School, University of Sheffield	COVID-19 Genomics UK (COG-UK) Consortium	Thushan de Silva, Matthew Parker, Nikki Smith, Adri Angyal, Rebecca Brown, Luke Green, Rachel Tucker, Paul Parsons, Danielle Groves, Katie Johnson, Laura Carrilero, Alex Keeley, Dave Partridge, Matthew Wyles, Benjamin Lindsey, Mehmet Yavuz, Mohammad Raza, Cariad Evans
EPI_ISL_637292	Department of Pathology, University of Cambridge	COVID-19 Genomics UK (COG-UK) Consortium	Aminu S. Jahun, Yasmin Chaudhry, Grant Hall, Iliana Georgana, Myra Hosmillo, Martin D. Curran, Malte Pinckert, Surendra Parmar, Ian Goodfellow
EPI_ISL_637293	Liverpool Clinical Laboratories	COVID-19 Genomics UK (COG-UK) Consortium	Sam Haldenby, Anita Lucaci, Steve Paterson, Julian Hiscox, Alistair Darby, M Almsaud, A Alrezaihi, Muhannad Alruwaili, Stuart D Armstrong, Jones Benjamin, Eleanor G Bentley, Anu Chawla, Jordan J Clark, Angela Cowell, Richard Eccles, Isabel García-Dorival, Matthew Gemmell, Alessandro Gerada, PKF Gilmore, Richard Gregory, Ximeng Han, Catherine Hartley, Margaret Hughes, Miren Iturriza-Gomara, James Johnson, L Luu, Jenifer Manson, Charlotte Nelson, Elaine O'Toole, Cassie Olateju, Rebekah Penrice-Randal , Lucille Rainbow, N.P Randle, Trevor Ian Robinson, Parul Sharma, Ghada T Shawli, James P Stewart, Neil Swainston, Ecaterina Vamos, Joanne Watts, Mark Whitehead
EPI_ISL_637294	Department of Pathology, University of Cambridge	COVID-19 Genomics UK (COG-UK) Consortium	Aminu S. Jahun, Yasmin Chaudhry, Grant Hall, Iliana Georgana, Myra Hosmillo, Martin D. Curran, Malte Pinckert, Surendra Parmar, Ian Goodfellow
EPI_ISL_637295, EPI_ISL_637296	Liverpool Clinical Laboratories	COVID-19 Genomics UK (COG-UK) Consortium	Sam Haldenby, Anita Lucaci, Steve Paterson, Julian Hiscox, Alistair Darby, M Almsaud, A Alrezaihi, Muhannad Alruwaili, Stuart D Armstrong, Jones Benjamin, Eleanor G Bentley, Anu Chawla, Jordan J Clark, Angela Cowell, Richard Eccles, Isabel García-Dorival, Matthew Gemmell, Alessandro Gerada, PKF Gilmore, Richard Gregory, Ximeng Han, Catherine Hartley, Margaret Hughes, Miren Iturriza-Gomara, James Johnson, L Luu, Jenifer Manson, Charlotte Nelson, Elaine O'Toole, Cassie Olateju, Rebekah Penrice-Randal , Lucille Rainbow, N.P Randle, Trevor Ian Robinson, Parul Sharma, Ghada T Shawli, James P Stewart, Neil Swainston, Ecaterina Vamos, Joanne Watts, Mark Whitehead
EPI_ISL_637297	Virology Department, Sheffield Teaching Hospitals NHS Foundation Trust/Department of Infection, Immunity and Cardiovascular Disease, The Medical School, University of Sheffield	COVID-19 Genomics UK (COG-UK) Consortium	Thushan de Silva, Matthew Parker, Nikki Smith, Adri Angyal, Rebecca Brown, Luke Green, Rachel Tucker, Paul Parsons, Danielle Groves, Katie Johnson, Laura Carrilero, Alex Keeley, Dave Partridge, Matthew Wyles, Benjamin Lindsey, Mehmet Yavuz, Mohammad Raza, Cariad Evans
EPI_ISL_637298	Queens Medical Centre, Clinical Microbiology Department / DeepSeq Nottingham	COVID-19 Genomics UK (COG-UK) Consortium	Gemma Clark, Wendy Smith, Manjinder Khakh, Vicki M Fleming, Michelle M Lister, Hannah Howson-Wells, Jonathan Ball, Patrick McClure, Joseph Chappell, Theocharis Tsoleridis, Nadine Holmes, Matthew Carlisle, Christopher Moore, Fei Sang, Johnny Debebe, Victoria Wright, Matthew Loose
EPI_ISL_637299	Department of Pathology, University of Cambridge	COVID-19 Genomics UK (COG-UK) Consortium	Aminu S. Jahun, Yasmin Chaudhry, Grant Hall, Iliana Georgana, Myra Hosmillo, Martin D. Curran, Malte Pinckert, Surendra Parmar, Ian Goodfellow
EPI_ISL_637300	Queens Medical Centre, Clinical Microbiology Department / DeepSeq Nottingham	COVID-19 Genomics UK (COG-UK) Consortium	Gemma Clark, Wendy Smith, Manjinder Khakh, Vicki M Fleming, Michelle M Lister, Hannah Howson-Wells, Jonathan Ball, Patrick McClure, Joseph Chappell, Theocharis Tsoleridis, Nadine Holmes, Matthew Carlisle, Christopher Moore, Fei Sang, Johnny Debebe, Victoria Wright, Matthew Loose
EPI_ISL_637301	Quadram Institute Bioscience	COVID-19 Genomics UK (COG-UK) Consortium	Dave J. Baker, Gemma L. Kay, Alp Aydin, Thanh Le-Viet, Steven Rudder, Ana P. Tedim, Anastasia Kolyva, Maria Diaz, Leonardo de Oliveira Martins, Nabil-Fareed Alikhan, Lizzie Meadows, Rachael Stanley, Ngozi Elumogo, Muhammed Yasir, Nicholas M. Thomson, Alexander J Trotter, Rachel Gilroy, Samuel Bloomfield, Claire Stuart, Andrew Bell, Reenesh Prakash, Samir Dervisevic, Alison E. Mather, John Wain, Mark Webber, Andrew J. Page, Justin O'Grady
EPI_ISL_637302	Wales Specialist Virology Centre Sequencing lab: Pathogen Genomics Unit	COVID-19 Genomics UK (COG-UK) Consortium	Catherine Moore, Johnathan Evans, Laura Gifford, Malorie Perry, Simon Cottrell, Angela Marchbank, Alec Birchley, Alexander Adams, Amy Gaskin, Bree Gatica-Wilcox, Jason Coombes, Joel Southgate, Lauren Gilbert, Lee Graham, Nicole Pacchiarini, Sara Kumziene-Summerhayes, Sarah Taylor, Sophie Jones, Sara Rey, Matthew Bull, Joanne Watkins, Sally Corden, Tom Connor
EPI_ISL_637303	Liverpool Clinical Laboratories	COVID-19 Genomics UK (COG-UK) Consortium	Sam Haldenby, Anita Lucaci, Steve Paterson, Julian Hiscox, Alistair Darby, M Almsaud, A Alrezaihi, Muhannad Alruwaili, Stuart D Armstrong, Jones Benjamin, Eleanor G Bentley, Anu Chawla, Jordan J Clark, Angela Cowell, Richard Eccles, Isabel García-Dorival, Matthew Gemmell, Alessandro Gerada, PKF Gilmore, Richard Gregory, Ximeng Han, Catherine Hartley, Margaret Hughes, Miren Iturriza-Gomara, James Johnson, L Luu, Jenifer Manson, Charlotte Nelson, Elaine O'Toole, Cassie Olateju, Rebekah Penrice-Randal , Lucille Rainbow, N.P Randle, Trevor Ian Robinson, Parul Sharma, Ghada T Shawli, James P Stewart, Neil Swainston, Ecaterina Vamos, Joanne Watts, Mark Whitehead
EPI_ISL_637304	Department of Pathology, University of Cambridge	COVID-19 Genomics UK (COG-UK) Consortium	Aminu S. Jahun, Yasmin Chaudhry, Grant Hall, Iliana Georgana, Myra Hosmillo, Martin D. Curran, Malte Pinckert, Surendra Parmar, Ian Goodfellow
EPI_ISL_637305	Centre for Enzyme Innovation, University of Portsmouth / Translational Research Laboratory, Portsmouth Hospitals NHS Trust	COVID-19 Genomics UK (COG-UK) Consortium	Angela Beckett,Yann Bourgeois,Garry Scarlett,Sharon Glaysher,Scott Elliott,Kelly Bicknell,Robert Impey,Allyson Lloyd,Sarah Wyllie,Ethan Butcher,Anoop Chauhan,Samuel Robson
EPI_ISL_637306	Liverpool Clinical Laboratories	COVID-19 Genomics UK (COG-UK) Consortium	Sam Haldenby, Anita Lucaci, Steve Paterson, Julian Hiscox, Alistair Darby, M Almsaud, A Alrezaihi, Muhannad Alruwaili, Stuart D Armstrong, Jones Benjamin, Eleanor G Bentley, Anu Chawla, Jordan J Clark, Angela Cowell, Richard Eccles, Isabel García-Dorival, Matthew Gemmell, Alessandro Gerada, PKF Gilmore, Richard Gregory, Ximeng Han, Catherine Hartley, Margaret Hughes, Miren Iturriza-Gomara, James Johnson, L Luu, Jenifer Manson, Charlotte Nelson, Elaine O'Toole, Cassie Olateju, Rebekah Penrice-Randal , Lucille Rainbow, N.P Randle, Trevor Ian Robinson, Parul Sharma, Ghada T Shawli, James P Stewart, Neil Swainston, Ecaterina Vamos, Joanne Watts, Mark Whitehead
EPI_ISL_637307, EPI_ISL_637308, EPI_ISL_637309, EPI_ISL_637310	Department of Pathology, University of Cambridge	COVID-19 Genomics UK (COG-UK) Consortium	Aminu S. Jahun, Yasmin Chaudhry, Grant Hall, Iliana Georgana, Myra Hosmillo, Martin D. Curran, Malte Pinckert, Surendra Parmar, Ian Goodfellow
EPI_ISL_637311, EPI_ISL_637312	Northumbria University / South Tees Hospitals NHS Foundation Trust / North Cumbria Integrated Care NHS Foundation Trust / North Tees and Hartlepool NHS	COVID-19 Genomics UK (COG-UK) Consortium	Darren L Smith,Andrew Nelson,Matthew Bashton,Greg R Young,Joshua Loh,John Allan,Mohammad A Tariq,Giles S Holt,Gary Black,Wen C Yew,Lynn Dover,Paul Baker,Steve Liggett,Sarah Essex,Jane Greenaway,Debra Padgett,Clive Graham,Garren Scott,Edward Barton,Emma Swindells,Brendan Payne,Jennifer Collins,Yusri Taha,Gary Eltringham

	Foundation Trust / Newcastle Hospitals NHS Foundation Trust		
EPI_ISL_637313	Liverpool Clinical Laboratories	COVID-19 Genomics UK (COG-UK) Consortium	Sam Haldenby, Anita Lucaci, Steve Paterson, Julian Hiscox, Alistair Darby, M Almsaud, A Alrezaihi, Muhannad Alruwaili, Stuart D Armstrong, Jones Benjamin, Eleanor G Bentley, Anu Chawla, Jordan J Clark, Angela Cowell, Richard Eccles, Isabel García-Dorival, Matthew Gemmell, Alessandro Gerada, PKF Gilmore, Richard Gregory, Ximeng Han, Catherine Hartley, Margaret Hughes, Miren Iturriza-Gomara, James Johnson, L Luu, Jenifer Manson, Charlotte Nelson, Elaine O'Toole, Cassie Olateju, Rebekah Penrice-Randal , Lucille Rainbow, N.P Randle, Trevor Ian Robinson, Parul Sharma, Ghada T Shawli, James P Stewart, Neil Swainston, Ecaterina Vamos, Joanne Watts, Mark Whitehead
EPI_ISL_637314, EPI_ISL_637315, EPI_ISL_637316, EPI_ISL_637317, EPI_ISL_637318	Department of Pathology, University of Cambridge	COVID-19 Genomics UK (COG-UK) Consortium	Aminu S. Jahun, Yasmin Chaudhry, Grant Hall, Iliana Georgana, Myra Hosmillo, Martin D. Curran, Malte Pinckert, Surendra Parmar, Ian Goodfellow
EPI_ISL_637319	Wales Specialist Virology Centre Sequencing lab: Pathogen Genomics Unit	COVID-19 Genomics UK (COG-UK) Consortium	Catherine Moore, Johnathan Evans, Laura Gifford, Malorie Perry, Simon Cottrell, Angela Marchbank, Alec Birchley, Alexander Adams, Amy Gaskin, Bree Gatica-Wilcox, Jason Coombes, Joel Southgate, Lauren Gilbert, Lee Graham, Nicole Pacchiarini, Sara Kumziene-Summerhayes, Sarah Taylor, Sophie Jones, Sara Rey, Matthew Bull, Joanne Watkins, Sally Corden, Tom Connor
EPI_ISL_637320	Virology Department, Sheffield Teaching Hospitals NHS Foundation Trust/Department of Infection, Immunity and Cardiovascular Disease, The Medical School, University of Sheffield	COVID-19 Genomics UK (COG-UK) Consortium	Thushan de Silva, Matthew Parker, Nikki Smith, Adri Angyal, Rebecca Brown, Luke Green, Rachel Tucker, Paul Parsons, Danielle Groves, Katie Johnson, Laura Carrilero, Alex Keeley, Dave Partridge, Matthew Wyles, Benjamin Lindsey, Mehmet Yavuz, Mohammad Raza, Cariad Evans
EPI_ISL_637321	Department of Pathology, University of Cambridge	COVID-19 Genomics UK (COG-UK) Consortium	Aminu S. Jahun, Yasmin Chaudhry, Grant Hall, Iliana Georgana, Myra Hosmillo, Martin D. Curran, Malte Pinckert, Surendra Parmar, Ian Goodfellow
EPI_ISL_637322	Northumbria University / South Tees Hospitals NHS Foundation Trust / North Cumbria Integrated Care NHS Foundation Trust / North Tees and Hartlepool NHS Foundation Trust / Newcastle Hospitals NHS Foundation Trust	COVID-19 Genomics UK (COG-UK) Consortium	Darren L Smith,Andrew Nelson,Matthew Bashton,Greg R Young,Joshua Loh,John Allan,Mohammad A Tariq,Giles S Holt,Gary Black,Wen C Yew,Lynn Dover,Paul Baker,Steve Liggett,Sarah Essex,Jane Greenaway,Debra Padgett,Clive Graham,Garren Scott,Edward Barton,Emma Swindells,Brendan Payne,Jennifer Collins,Yusri Taha,Gary Eltringham
EPI_ISL_637323	Liverpool Clinical Laboratories	COVID-19 Genomics UK (COG-UK) Consortium	Sam Haldenby, Anita Lucaci, Steve Paterson, Julian Hiscox, Alistair Darby, M Almsaud, A Alrezaihi, Muhannad Alruwaili, Stuart D Armstrong, Jones Benjamin, Eleanor G Bentley, Anu Chawla, Jordan J Clark, Angela Cowell, Richard Eccles, Isabel García-Dorival, Matthew Gemmell, Alessandro Gerada, PKF Gilmore, Richard Gregory, Ximeng Han, Catherine Hartley, Margaret Hughes, Miren Iturriza-Gomara, James Johnson, L Luu, Jenifer Manson, Charlotte Nelson, Elaine O'Toole, Cassie Olateju, Rebekah Penrice-Randal , Lucille Rainbow, N.P Randle, Trevor Ian Robinson, Parul Sharma, Ghada T Shawli, James P Stewart, Neil Swainston, Ecaterina Vamos, Joanne Watts, Mark Whitehead
EPI_ISL_637324	Department of Pathology, University of Cambridge	COVID-19 Genomics UK (COG-UK) Consortium	Aminu S. Jahun, Yasmin Chaudhry, Grant Hall, Iliana Georgana, Myra Hosmillo, Martin D. Curran, Malte Pinckert, Surendra Parmar, Ian Goodfellow
EPI_ISL_637325	Liverpool Clinical Laboratories	COVID-19 Genomics UK (COG-UK) Consortium	Sam Haldenby, Anita Lucaci, Steve Paterson, Julian Hiscox, Alistair Darby, M Almsaud, A Alrezaihi, Muhannad Alruwaili, Stuart D Armstrong, Jones Benjamin, Eleanor G Bentley, Anu Chawla, Jordan J Clark, Angela Cowell, Richard Eccles, Isabel García-Dorival, Matthew Gemmell, Alessandro Gerada, PKF Gilmore, Richard Gregory, Ximeng Han, Catherine Hartley, Margaret Hughes, Miren Iturriza-Gomara, James Johnson, L Luu, Jenifer Manson, Charlotte Nelson, Elaine O'Toole, Cassie Olateju, Rebekah Penrice-Randal , Lucille Rainbow, N.P Randle, Trevor Ian Robinson, Parul Sharma, Ghada T Shawli, James P Stewart, Neil Swainston, Ecaterina Vamos, Joanne Watts, Mark Whitehead
EPI_ISL_637326	Virology Department, Sheffield Teaching Hospitals NHS Foundation Trust/Department of Infection, Immunity and Cardiovascular Disease, The Medical School, University of Sheffield	COVID-19 Genomics UK (COG-UK) Consortium	Thushan de Silva, Matthew Parker, Nikki Smith, Adri Angyal, Rebecca Brown, Luke Green, Rachel Tucker, Paul Parsons, Danielle Groves, Katie Johnson, Laura Carrilero, Alex Keeley, Dave Partridge, Matthew Wyles, Benjamin Lindsey, Mehmet Yavuz, Mohammad Raza, Cariad Evans
EPI_ISL_637327	Wales Specialist Virology Centre Sequencing lab: Pathogen Genomics Unit	COVID-19 Genomics UK (COG-UK) Consortium	Catherine Moore, Johnathan Evans, Laura Gifford, Malorie Perry, Simon Cottrell, Angela Marchbank, Alec Birchley, Alexander Adams, Amy Gaskin, Bree Gatica-Wilcox, Jason Coombes, Joel Southgate, Lauren Gilbert, Lee Graham, Nicole Pacchiarini, Sara Kumziene-Summerhayes, Sarah Taylor, Sophie Jones, Sara Rey, Matthew Bull, Joanne Watkins, Sally Corden, Tom Connor
EPI_ISL_637328	Department of Pathology, University of Cambridge	COVID-19 Genomics UK (COG-UK) Consortium	Aminu S. Jahun, Yasmin Chaudhry, Grant Hall, Iliana Georgana, Myra Hosmillo, Martin D. Curran, Malte Pinckert, Surendra Parmar, Ian Goodfellow
EPI_ISL_637329	Liverpool Clinical Laboratories	COVID-19 Genomics UK (COG-UK) Consortium	Sam Haldenby, Anita Lucaci, Steve Paterson, Julian Hiscox, Alistair Darby, M Almsaud, A Alrezaihi, Muhannad Alruwaili, Stuart D Armstrong, Jones Benjamin, Eleanor G Bentley, Anu Chawla, Jordan J Clark, Angela Cowell, Richard Eccles, Isabel García-Dorival, Matthew Gemmell, Alessandro Gerada, PKF Gilmore, Richard Gregory, Ximeng Han, Catherine Hartley, Margaret Hughes, Miren Iturriza-Gomara, James Johnson, L Luu, Jenifer Manson, Charlotte Nelson, Elaine O'Toole, Cassie Olateju, Rebekah Penrice-Randal , Lucille Rainbow, N.P Randle, Trevor Ian Robinson, Parul Sharma, Ghada T Shawli, James P Stewart, Neil Swainston, Ecaterina Vamos, Joanne Watts, Mark Whitehead
EPI_ISL_637330	Wales Specialist Virology Centre Sequencing lab: Pathogen Genomics Unit	COVID-19 Genomics UK (COG-UK) Consortium	Catherine Moore, Johnathan Evans, Laura Gifford, Malorie Perry, Simon Cottrell, Angela Marchbank, Alec Birchley, Alexander Adams, Amy Gaskin, Bree Gatica-Wilcox, Jason Coombes, Joel Southgate, Lauren Gilbert, Lee Graham, Nicole Pacchiarini, Sara Kumziene-Summerhayes, Sarah Taylor, Sophie Jones, Sara Rey, Matthew Bull, Joanne Watkins, Sally Corden, Tom Connor
EPI_ISL_637331	Department of Pathology, University of Cambridge	COVID-19 Genomics UK (COG-UK) Consortium	Aminu S. Jahun, Yasmin Chaudhry, Grant Hall, Iliana Georgana, Myra Hosmillo, Martin D. Curran, Malte Pinckert, Surendra Parmar, Ian Goodfellow
EPI_ISL_637332	Wales Specialist Virology Centre Sequencing lab: Pathogen Genomics Unit	COVID-19 Genomics UK (COG-UK) Consortium	Catherine Moore, Johnathan Evans, Laura Gifford, Malorie Perry, Simon Cottrell, Angela Marchbank, Alec Birchley, Alexander Adams, Amy Gaskin, Bree Gatica-Wilcox, Jason Coombes, Joel Southgate, Lauren Gilbert, Lee Graham, Nicole Pacchiarini, Sara Kumziene-Summerhayes, Sarah Taylor, Sophie Jones, Sara Rey, Matthew Bull, Joanne Watkins, Sally Corden, Tom Connor
EPI_ISL_637334, EPI_ISL_637335	Department of Pathology, University of Cambridge	COVID-19 Genomics UK (COG-UK) Consortium	Aminu S. Jahun, Yasmin Chaudhry, Grant Hall, Iliana Georgana, Myra Hosmillo, Martin D. Curran, Malte Pinckert, Surendra Parmar, Ian Goodfellow
EPI_ISL_637336, EPI_ISL_637337, EPI_ISL_637338, EPI_ISL_637339	Liverpool Clinical Laboratories	COVID-19 Genomics UK (COG-UK) Consortium	Sam Haldenby, Anita Lucaci, Steve Paterson, Julian Hiscox, Alistair Darby, M Almsaud, A Alrezaihi, Muhannad Alruwaili, Stuart D Armstrong, Jones Benjamin, Eleanor G Bentley, Anu Chawla, Jordan J Clark, Angela Cowell, Richard Eccles, Isabel García-Dorival, Matthew Gemmell, Alessandro Gerada, PKF Gilmore, Richard Gregory, Ximeng Han, Catherine Hartley, Margaret Hughes, Miren Iturriza-Gomara, James Johnson, L Luu, Jenifer Manson, Charlotte Nelson, Elaine O'Toole, Cassie Olateju, Rebekah Penrice-Randal , Lucille Rainbow, N.P Randle, Trevor Ian Robinson, Parul Sharma, Ghada T Shawli, James P Stewart, Neil Swainston, Ecaterina Vamos, Joanne Watts, Mark Whitehead
EPI_ISL_637340, EPI_ISL_637341	Department of Pathology, University of Cambridge	COVID-19 Genomics UK (COG-UK) Consortium	Aminu S. Jahun, Yasmin Chaudhry, Grant Hall, Iliana Georgana, Myra Hosmillo, Martin D. Curran, Malte Pinckert, Surendra Parmar, Ian Goodfellow
EPI_ISL_637342, EPI_ISL_637343, EPI_ISL_637344	Queens Medical Centre, Clinical Microbiology Department / DeepSeq Nottingham	COVID-19 Genomics UK (COG-UK) Consortium	Gemma Clark, Wendy Smith, Manjinder Khakh, Vicki M Fleming, Michelle M Lister, Hannah Howson-Wells, Jonathan Ball, Patrick McClure, Joseph Chappell, Theocharis Tsoleridis, Nadine Holmes, Matthew Carlisle, Christopher Moore, Fei Sang, Johnny Debebe, Victoria Wright, Matthew Loose
EPI_ISL_637345	Department of Pathology, University of Cambridge	COVID-19 Genomics UK (COG-UK) Consortium	Aminu S. Jahun, Yasmin Chaudhry, Grant Hall, Iliana Georgana, Myra Hosmillo, Martin D. Curran, Malte Pinckert, Surendra Parmar, Ian Goodfellow
EPI_ISL_637346, EPI_ISL_637347	Wales Specialist Virology Centre Sequencing lab: Pathogen Genomics Unit	COVID-19 Genomics UK (COG-UK) Consortium	Catherine Moore, Johnathan Evans, Laura Gifford, Malorie Perry, Simon Cottrell, Angela Marchbank, Alec Birchley, Alexander Adams, Amy Gaskin, Bree Gatica-Wilcox, Jason Coombes, Joel Southgate, Lauren Gilbert, Lee Graham, Nicole Pacchiarini, Sara Kumziene-Summerhayes, Sarah Taylor, Sophie Jones, Sara Rey, Matthew Bull, Joanne Watkins, Sally Corden, Tom Connor
EPI_ISL_637348	Department of Pathology, University of Cambridge	COVID-19 Genomics UK (COG-UK) Consortium	Aminu S. Jahun, Yasmin Chaudhry, Grant Hall, Iliana Georgana, Myra Hosmillo, Martin D. Curran, Malte Pinckert, Surendra Parmar, Ian Goodfellow
EPI_ISL_637349	Wales Specialist Virology Centre Sequencing lab: Pathogen Genomics Unit	COVID-19 Genomics UK (COG-UK) Consortium	Catherine Moore, Johnathan Evans, Laura Gifford, Malorie Perry, Simon Cottrell, Angela Marchbank, Alec Birchley, Alexander Adams, Amy Gaskin, Bree Gatica-Wilcox, Jason Coombes, Joel Southgate, Lauren Gilbert, Lee Graham, Nicole Pacchiarini, Sara Kumziene-Summerhayes, Sarah Taylor, Sophie Jones, Sara Rey, Matthew Bull, Joanne Watkins, Sally Corden, Tom Connor
EPI_ISL_637350	Liverpool Clinical Laboratories	COVID-19 Genomics UK (COG-UK) Consortium	Sam Haldenby, Anita Lucaci, Steve Paterson, Julian Hiscox, Alistair Darby, M Almsaud, A Alrezaihi, Muhannad Alruwaili, Stuart D Armstrong, Jones Benjamin, Eleanor G Bentley, Anu Chawla, Jordan J Clark, Angela Cowell, Richard Eccles, Isabel García-Dorival, Matthew Gemmell, Alessandro Gerada, PKF Gilmore, Richard Gregory, Ximeng Han, Catherine Hartley, Margaret Hughes, Miren Iturriza-Gomara, James Johnson, L Luu, Jenifer Manson, Charlotte Nelson, Elaine O'Toole, Cassie Olateju, Rebekah Penrice-Randal , Lucille Rainbow, N.P Randle, Trevor Ian Robinson, Parul Sharma, Ghada T Shawli, James P Stewart, Neil Swainston, Ecaterina Vamos, Joanne Watts, Mark Whitehead

EPI_ISL_637351	Wales Specialist Virology Centre Sequencing lab: Pathogen Genomics Unit	COVID-19 Genomics UK (COG-UK) Consortium	Catherine Moore, Johnathan Evans, Laura Gifford, Malorie Perry, Simon Cottrell, Angela Marchbank, Alec Birchley, Alexander Adams, Amy Gaskin, Bree Gatica-Wilcox, Jason Coombes, Joel Southgate, Lauren Gilbert, Lee Graham, Nicole Pacchiarini, Sara Kumziene-Summerhayes, Sarah Taylor, Sophie Jones, Sara Rey, Matthew Bull, Joanne Watkins, Sally Corden, Tom Connor
EPI_ISL_637352	Northumbria University / South Tees Hospitals NHS Foundation Trust / North Cumbria Integrated Care NHS Foundation Trust / North Tees and Hartlepool NHS Foundation Trust / Newcastle Hospitals NHS Foundation Trust	COVID-19 Genomics UK (COG-UK) Consortium	Darren L Smith,Andrew Nelson,Matthew Bashton,Greg R Young,Joshua Loh,John Allan,Mohammad A Tariq,Giles S Holt,Gary Black,Wen C Yew,Lynn Dover,Paul Baker,Steve Liggett,Sarah Essex,Jane Greenaway,Debra Padgett,Clive Graham,Garren Scott,Edward Barton,Emma Swindells,Brendan Payne,Jennifer Collins,Yusri Taha,Gary Eltringham
EPI_ISL_637353, EPI_ISL_637354, EPI_ISL_637355, EPI_ISL_637356, EPI_ISL_637357	Department of Pathology, University of Cambridge	COVID-19 Genomics UK (COG-UK) Consortium	Aminu S. Jahun, Yasmin Chaudhry, Grant Hall, Iliana Georgana, Myra Hosmillo, Martin D. Curran, Malte Pinckert, Surendra Parmar, Ian Goodfellow
EPI_ISL_637358	Virology Department, Sheffield Teaching Hospitals NHS Foundation Trust/Department of Infection, Immunity and Cardiovascular Disease, The Medical School, University of Sheffield	COVID-19 Genomics UK (COG-UK) Consortium	Thushan de Silva, Matthew Parker, Nikki Smith, Adri Angyal, Rebecca Brown, Luke Green, Rachel Tucker, Paul Parsons, Danielle Groves, Katie Johnson, Laura Carrilero, Alex Keeley, Dave Partridge, Matthew Wyles, Benjamin Lindsey, Mehmet Yavuz, Mohammad Raza, Cariad Evans
EPI_ISL_637359	Quadram Institute Bioscience	COVID-19 Genomics UK (COG-UK) Consortium	Dave J. Baker, Gemma L. Kay, Alp Aydin, Thanh Le-Viet, Steven Rudder, Ana P. Tedim, Anastasia Kolyva, Maria Diaz, Leonardo de Oliveira Martins, Nabil-Fareed Alikhan, Lizzie Meadows, Rachael Stanley, Ngozi Elumogo, Muhammed Yasir, Nicholas M. Thomson, Alexander J Trotter, Rachel Gilroy, Samuel Bloomfield, Claire Stuart, Andrew Bell, Reenesh Prakash, Samir Dervisevic, Alison E. Mather, John Wain, Mark Webber, Andrew J. Page, Justin O'Grady
EPI_ISL_637360	Department of Pathology, University of Cambridge	COVID-19 Genomics UK (COG-UK) Consortium	Aminu S. Jahun, Yasmin Chaudhry, Grant Hall, Iliana Georgana, Myra Hosmillo, Martin D. Curran, Malte Pinckert, Surendra Parmar, Ian Goodfellow
EPI_ISL_637361	Virology Department, Sheffield Teaching Hospitals NHS Foundation Trust/Department of Infection, Immunity and Cardiovascular Disease, The Medical School, University of Sheffield	COVID-19 Genomics UK (COG-UK) Consortium	Thushan de Silva, Matthew Parker, Nikki Smith, Adri Angyal, Rebecca Brown, Luke Green, Rachel Tucker, Paul Parsons, Danielle Groves, Katie Johnson, Laura Carrilero, Alex Keeley, Dave Partridge, Matthew Wyles, Benjamin Lindsey, Mehmet Yavuz, Mohammad Raza, Cariad Evans
EPI_ISL_637362, EPI_ISL_637363, EPI_ISL_637364, EPI_ISL_637365, EPI_ISL_637366, EPI_ISL_637367, EPI_ISL_637368	Department of Pathology, University of Cambridge	COVID-19 Genomics UK (COG-UK) Consortium	Aminu S. Jahun, Yasmin Chaudhry, Grant Hall, Iliana Georgana, Myra Hosmillo, Martin D. Curran, Malte Pinckert, Surendra Parmar, Ian Goodfellow
EPI_ISL_637369, EPI_ISL_637370, EPI_ISL_637371, EPI_ISL_637372	Centre for Enzyme Innovation, University of Portsmouth / Translational Research Laboratory, Portsmouth Hospitals NHS Trust	COVID-19 Genomics UK (COG-UK) Consortium	Angela Beckett,Yann Bourgeois,Garry Scarlett,Sharon Glaysher,Scott Elliott,Kelly Bicknell,Robert Impey,Allyson Lloyd,Sarah Wyllie,Ethan Butcher,Anoop Chauhan,Samuel Robson
EPI_ISL_637373, EPI_ISL_637374, EPI_ISL_637375, EPI_ISL_637376, EPI_ISL_637377	Northumbria University / South Tees Hospitals NHS Foundation Trust / North Cumbria Integrated Care NHS Foundation Trust / North Tees and Hartlepool NHS Foundation Trust / Newcastle Hospitals NHS Foundation Trust	COVID-19 Genomics UK (COG-UK) Consortium	Darren L Smith,Andrew Nelson,Matthew Bashton,Greg R Young,Joshua Loh,John Allan,Mohammad A Tariq,Giles S Holt,Gary Black,Wen C Yew,Lynn Dover,Paul Baker,Steve Liggett,Sarah Essex,Jane Greenaway,Debra Padgett,Clive Graham,Garren Scott,Edward Barton,Emma Swindells,Brendan Payne,Jennifer Collins,Yusri Taha,Gary Eltringham
EPI_ISL_637378, EPI_ISL_637379, EPI_ISL_637380	Wales Specialist Virology Centre Sequencing lab: Pathogen Genomics Unit	COVID-19 Genomics UK (COG-UK) Consortium	Catherine Moore, Johnathan Evans, Laura Gifford, Malorie Perry, Simon Cottrell, Angela Marchbank, Alec Birchley, Alexander Adams, Amy Gaskin, Bree Gatica-Wilcox, Jason Coombes, Joel Southgate, Lauren Gilbert, Lee Graham, Nicole Pacchiarini, Sara Kumziene-Summerhayes, Sarah Taylor, Sophie Jones, Sara Rey, Matthew Bull, Joanne Watkins, Sally Corden, Tom Connor
EPI_ISL_637381	Virology Department, Sheffield Teaching Hospitals NHS Foundation Trust/Department of Infection, Immunity and Cardiovascular Disease, The Medical School, University of Sheffield	COVID-19 Genomics UK (COG-UK) Consortium	Thushan de Silva, Matthew Parker, Nikki Smith, Adri Angyal, Rebecca Brown, Luke Green, Rachel Tucker, Paul Parsons, Danielle Groves, Katie Johnson, Laura Carrilero, Alex Keeley, Dave Partridge, Matthew Wyles, Benjamin Lindsey, Mehmet Yavuz, Mohammad Raza, Cariad Evans
EPI_ISL_637382, EPI_ISL_637383	Northumbria University / South Tees Hospitals NHS Foundation Trust / North Cumbria Integrated Care NHS Foundation Trust / North Tees and Hartlepool NHS Foundation Trust / Newcastle Hospitals NHS Foundation Trust	COVID-19 Genomics UK (COG-UK) Consortium	Darren L Smith,Andrew Nelson,Matthew Bashton,Greg R Young,Joshua Loh,John Allan,Mohammad A Tariq,Giles S Holt,Gary Black,Wen C Yew,Lynn Dover,Paul Baker,Steve Liggett,Sarah Essex,Jane Greenaway,Debra Padgett,Clive Graham,Garren Scott,Edward Barton,Emma Swindells,Brendan Payne,Jennifer Collins,Yusri Taha,Gary Eltringham
EPI_ISL_637384	Department of Pathology, University of Cambridge	COVID-19 Genomics UK (COG-UK) Consortium	Aminu S. Jahun, Yasmin Chaudhry, Grant Hall, Iliana Georgana, Myra Hosmillo, Martin D. Curran, Malte Pinckert, Surendra Parmar, Ian Goodfellow
EPI_ISL_637385	Wales Specialist Virology Centre Sequencing lab: Pathogen Genomics Unit	COVID-19 Genomics UK (COG-UK) Consortium	Catherine Moore, Johnathan Evans, Laura Gifford, Malorie Perry, Simon Cottrell, Angela Marchbank, Alec Birchley, Alexander Adams, Amy Gaskin, Bree Gatica-Wilcox, Jason Coombes, Joel Southgate, Lauren Gilbert, Lee Graham, Nicole Pacchiarini, Sara Kumziene-Summerhayes, Sarah Taylor, Sophie Jones, Sara Rey, Matthew Bull, Joanne Watkins, Sally Corden, Tom Connor
EPI_ISL_637386	Department of Pathology, University of Cambridge	COVID-19 Genomics UK (COG-UK) Consortium	Aminu S. Jahun, Yasmin Chaudhry, Grant Hall, Iliana Georgana, Myra Hosmillo, Martin D. Curran, Malte Pinckert, Surendra Parmar, Ian Goodfellow
EPI_ISL_637387	Centre for Enzyme Innovation, University of Portsmouth / Translational Research Laboratory, Portsmouth Hospitals NHS Trust	COVID-19 Genomics UK (COG-UK) Consortium	Angela Beckett,Yann Bourgeois,Garry Scarlett,Sharon Glaysher,Scott Elliott,Kelly Bicknell,Robert Impey,Allyson Lloyd,Sarah Wyllie,Ethan Butcher,Anoop Chauhan,Samuel Robson
EPI_ISL_637388, EPI_ISL_637389	Department of Pathology, University of Cambridge	COVID-19 Genomics UK (COG-UK) Consortium	Aminu S. Jahun, Yasmin Chaudhry, Grant Hall, Iliana Georgana, Myra Hosmillo, Martin D. Curran, Malte Pinckert, Surendra Parmar, Ian Goodfellow
EPI_ISL_637390	Liverpool Clinical Laboratories	COVID-19 Genomics UK (COG-UK) Consortium	Sam Haldenby, Anita Lucaci, Steve Paterson, Julian Hiscox, Alistair Darby, M Almsaud, A Alrezaihi, Muhannad Alruwaili, Stuart D Armstrong, Jones Benjamin, Eleanor G Bentley, Anu Chawla, Jordan J Clark, Angela Cowell, Richard Eccles, Isabel García-Dorival, Matthew Gemmell, Alessandro Gerada, PKF Gilmore, Richard Gregory, Ximeng Han, Catherine Hartley, Margaret Hughes, Miren Iturriza-Gomara, James Johnson, L Luu, Jenifer Manson, Charlotte Nelson, Elaine O'Toole, Cassie Olateju, Rebekah Penrice-Randal , Lucille Rainbow, N.P Randle, Trevor Ian Robinson, Parul Sharma, Ghada T Shawli, James P Stewart, Neil Swainston, Ecaterina Vamos, Joanne Watts, Mark Whitehead
EPI_ISL_637391, EPI_ISL_637392, EPI_ISL_637393, EPI_ISL_637394	Department of Pathology, University of Cambridge	COVID-19 Genomics UK (COG-UK) Consortium	Aminu S. Jahun, Yasmin Chaudhry, Grant Hall, Iliana Georgana, Myra Hosmillo, Martin D. Curran, Malte Pinckert, Surendra Parmar, Ian Goodfellow
EPI_ISL_637395	Wales Specialist Virology Centre Sequencing lab: Pathogen Genomics Unit	COVID-19 Genomics UK (COG-UK) Consortium	Catherine Moore, Johnathan Evans, Laura Gifford, Malorie Perry, Simon Cottrell, Angela Marchbank, Alec Birchley, Alexander Adams, Amy Gaskin, Bree Gatica-Wilcox, Jason Coombes, Joel Southgate, Lauren Gilbert, Lee Graham, Nicole Pacchiarini, Sara Kumziene-Summerhayes, Sarah Taylor, Sophie Jones, Sara Rey, Matthew Bull, Joanne Watkins, Sally Corden, Tom Connor
EPI_ISL_637396, EPI_ISL_637397, EPI_ISL_637398, EPI_ISL_637399	Department of Pathology, University of Cambridge	COVID-19 Genomics UK (COG-UK) Consortium	Aminu S. Jahun, Yasmin Chaudhry, Grant Hall, Iliana Georgana, Myra Hosmillo, Martin D. Curran, Malte Pinckert, Surendra Parmar, Ian Goodfellow
EPI_ISL_637400	Wales Specialist Virology Centre Sequencing lab: Pathogen Genomics Unit	COVID-19 Genomics UK (COG-UK) Consortium	Catherine Moore, Johnathan Evans, Laura Gifford, Malorie Perry, Simon Cottrell, Angela Marchbank, Alec Birchley, Alexander Adams, Amy Gaskin, Bree Gatica-Wilcox, Jason Coombes, Joel Southgate, Lauren Gilbert, Lee Graham, Nicole Pacchiarini, Sara Kumziene-Summerhayes, Sarah Taylor, Sophie Jones, Sara Rey, Matthew Bull, Joanne Watkins, Sally Corden, Tom Connor
EPI_ISL_637401	Liverpool Clinical Laboratories	COVID-19 Genomics UK (COG-UK) Consortium	Sam Haldenby, Anita Lucaci, Steve Paterson, Julian Hiscox, Alistair Darby, M Almsaud, A Alrezaihi, Muhannad Alruwaili, Stuart D Armstrong, Jones Benjamin, Eleanor G Bentley, Anu Chawla, Jordan J Clark, Angela Cowell, Richard Eccles, Isabel García-Dorival, Matthew Gemmell, Alessandro Gerada, PKF Gilmore, Richard Gregory, Ximeng Han, Catherine Hartley, Margaret Hughes, Miren Iturriza-Gomara, James Johnson, L Luu, Jenifer Manson, Charlotte Nelson, Elaine O'Toole, Cassie Olateju, Rebekah Penrice-Randal , Lucille Rainbow, N.P Randle, Trevor Ian Robinson, Parul Sharma, Ghada T Shawli, James P Stewart, Neil Swainston, Ecaterina Vamos, Joanne Watts, Mark Whitehead
EPI_ISL_637402	Centre for Enzyme Innovation, University of Portsmouth /	COVID-19 Genomics UK (COG-UK) Consortium	Angela Beckett,Yann Bourgeois,Garry Scarlett,Sharon Glaysher,Scott Elliott,Kelly Bicknell,Robert Impey,Allyson Lloyd,Sarah Wyllie,Ethan Butcher,Anoop

	Translational Research Laboratory, Portsmouth Hospitals NHS Trust		Chauhan,Samuel Robson
EPI_ISL_637403, EPI_ISL_637404, EPI_ISL_637405, EPI_ISL_637406, EPI_ISL_637407, EPI_ISL_637408	Department of Pathology, University of Cambridge	COVID-19 Genomics UK (COG-UK) Consortium	Aminu S. Jahun, Yasmin Chaudhry, Grant Hall, Iliana Georgana, Myra Hosmillo, Martin D. Curran, Malte Pinckert, Surendra Parmar, Ian Goodfellow
EPI_ISL_637409	Virology Department, Sheffield Teaching Hospitals NHS Foundation Trust/Department of Infection, Immunity and Cardiovascular Disease, The Medical School, University of Sheffield	COVID-19 Genomics UK (COG-UK) Consortium	Thushan de Silva, Matthew Parker, Nikki Smith, Adri Angyal, Rebecca Brown, Luke Green, Rachel Tucker, Paul Parsons, Danielle Groves, Katie Johnson, Laura Carrilero, Alex Keeley, Dave Partridge, Matthew Wyles, Benjamin Lindsey, Mehmet Yavuz, Mohammad Raza, Cariad Evans
EPI_ISL_637410	Department of Pathology, University of Cambridge	COVID-19 Genomics UK (COG-UK) Consortium	Aminu S. Jahun, Yasmin Chaudhry, Grant Hall, Iliana Georgana, Myra Hosmillo, Martin D. Curran, Malte Pinckert, Surendra Parmar, Ian Goodfellow
EPI_ISL_637411	Centre for Enzyme Innovation, University of Portsmouth / Translational Research Laboratory, Portsmouth Hospitals NHS Trust	COVID-19 Genomics UK (COG-UK) Consortium	Angela Beckett,Yann Bourgeois,Garry Scarlett,Sharon Glaysher,Scott Elliott,Kelly Bicknell,Robert Impey,Allyson Lloyd,Sarah Wyllie,Ethan Butcher,Anoop Chauhan,Samuel Robson
EPI_ISL_637412	Wales Specialist Virology Centre Sequencing lab: Pathogen Genomics Unit	COVID-19 Genomics UK (COG-UK) Consortium	Catherine Moore, Johnathan Evans, Laura Gifford, Malorie Perry, Simon Cottrell, Angela Marchbank, Alec Birchley, Alexander Adams, Amy Gaskin, Bree Gatica-Wilcox, Jason Coombes, Joel Southgate, Lauren Gilbert, Lee Graham, Nicole Pacchiarini, Sara Kumziene-Summerhayes, Sarah Taylor, Sophie Jones, Sara Rey, Matthew Bull, Joanne Watkins, Sally Corden, Tom Connor
EPI_ISL_637413, EPI_ISL_637414, EPI_ISL_637415, EPI_ISL_637416, EPI_ISL_637417, EPI_ISL_637418	Department of Pathology, University of Cambridge	COVID-19 Genomics UK (COG-UK) Consortium	Aminu S. Jahun, Yasmin Chaudhry, Grant Hall, Iliana Georgana, Myra Hosmillo, Martin D. Curran, Malte Pinckert, Surendra Parmar, Ian Goodfellow
EPI_ISL_637419	Queens Medical Centre, Clinical Microbiology Department / DeepSeq Nottingham	COVID-19 Genomics UK (COG-UK) Consortium	Gemma Clark, Wendy Smith, Manjinder Khakh, Vicki M Fleming, Michelle M Lister, Hannah Howson-Wells, Jonathan Ball, Patrick McClure, Joseph Chappell, Theocharis Tsoleridis, Nadine Holmes, Matthew Carlisle, Christopher Moore, Fei Sang, Johnny Debebe, Victoria Wright, Matthew Loose
EPI_ISL_637420, EPI_ISL_637421	Department of Pathology, University of Cambridge	COVID-19 Genomics UK (COG-UK) Consortium	Aminu S. Jahun, Yasmin Chaudhry, Grant Hall, Iliana Georgana, Myra Hosmillo, Martin D. Curran, Malte Pinckert, Surendra Parmar, Ian Goodfellow
EPI_ISL_637422	Wales Specialist Virology Centre Sequencing lab: Pathogen Genomics Unit	COVID-19 Genomics UK (COG-UK) Consortium	Catherine Moore, Johnathan Evans, Laura Gifford, Malorie Perry, Simon Cottrell, Angela Marchbank, Alec Birchley, Alexander Adams, Amy Gaskin, Bree Gatica-Wilcox, Jason Coombes, Joel Southgate, Lauren Gilbert, Lee Graham, Nicole Pacchiarini, Sara Kumziene-Summerhayes, Sarah Taylor, Sophie Jones, Sara Rey, Matthew Bull, Joanne Watkins, Sally Corden, Tom Connor
EPI_ISL_637424, EPI_ISL_637425, EPI_ISL_637426	Department of Pathology, University of Cambridge	COVID-19 Genomics UK (COG-UK) Consortium	Aminu S. Jahun, Yasmin Chaudhry, Grant Hall, Iliana Georgana, Myra Hosmillo, Martin D. Curran, Malte Pinckert, Surendra Parmar, Ian Goodfellow
EPI_ISL_637427	Queens Medical Centre, Clinical Microbiology Department / DeepSeq Nottingham	COVID-19 Genomics UK (COG-UK) Consortium	Gemma Clark, Wendy Smith, Manjinder Khakh, Vicki M Fleming, Michelle M Lister, Hannah Howson-Wells, Jonathan Ball, Patrick McClure, Joseph Chappell, Theocharis Tsoleridis, Nadine Holmes, Matthew Carlisle, Christopher Moore, Fei Sang, Johnny Debebe, Victoria Wright, Matthew Loose
EPI_ISL_637428, EPI_ISL_637429, EPI_ISL_637430, EPI_ISL_637431	Department of Pathology, University of Cambridge	COVID-19 Genomics UK (COG-UK) Consortium	Aminu S. Jahun, Yasmin Chaudhry, Grant Hall, Iliana Georgana, Myra Hosmillo, Martin D. Curran, Malte Pinckert, Surendra Parmar, Ian Goodfellow
EPI_ISL_637432	Centre for Enzyme Innovation, University of Portsmouth / Translational Research Laboratory, Portsmouth Hospitals NHS Trust	COVID-19 Genomics UK (COG-UK) Consortium	Angela Beckett,Yann Bourgeois,Garry Scarlett,Sharon Glaysher,Scott Elliott,Kelly Bicknell,Robert Impey,Allyson Lloyd,Sarah Wyllie,Ethan Butcher,Anoop Chauhan,Samuel Robson
EPI_ISL_637433	Department of Pathology, University of Cambridge	COVID-19 Genomics UK (COG-UK) Consortium	Aminu S. Jahun, Yasmin Chaudhry, Grant Hall, Iliana Georgana, Myra Hosmillo, Martin D. Curran, Malte Pinckert, Surendra Parmar, Ian Goodfellow
EPI_ISL_637434	Liverpool Clinical Laboratories	COVID-19 Genomics UK (COG-UK) Consortium	Sam Haldenby, Anita Lucaci, Steve Paterson, Julian Hiscox, Alistair Darby, M Almsaud, A Alrezaihi, Muhannad Alruwaili, Stuart D Armstrong, Jones Benjamin, Eleanor G Bentley, Anu Chawla, Jordan J Clark, Angela Cowell, Richard Eccles, Isabel García-Dorival, Matthew Gemmell, Alessandro Gerada, PKF Gilmore, Richard Gregory, Ximeng Han, Catherine Hartley, Margaret Hughes, Miren Iturriza-Gomara, James Johnson, L Luu, Jenifer Manson, Charlotte Nelson, Elaine O'Toole, Cassie Olateju, Rebekah Penrice-Randal , Lucille Rainbow, N.P Randle, Trevor Ian Robinson, Parul Sharma, Ghada T Shawli, James P Stewart, Neil Swainston, Ecaterina Vamos, Joanne Watts, Mark Whitehead
EPI_ISL_637435	Department of Pathology, University of Cambridge	COVID-19 Genomics UK (COG-UK) Consortium	Aminu S. Jahun, Yasmin Chaudhry, Grant Hall, Iliana Georgana, Myra Hosmillo, Martin D. Curran, Malte Pinckert, Surendra Parmar, Ian Goodfellow
EPI_ISL_637436	Virology Department, Sheffield Teaching Hospitals NHS Foundation Trust/Department of Infection, Immunity and Cardiovascular Disease, The Medical School, University of Sheffield	COVID-19 Genomics UK (COG-UK) Consortium	Thushan de Silva, Matthew Parker, Nikki Smith, Adri Angyal, Rebecca Brown, Luke Green, Rachel Tucker, Paul Parsons, Danielle Groves, Katie Johnson, Laura Carrilero, Alex Keeley, Dave Partridge, Matthew Wyles, Benjamin Lindsey, Mehmet Yavuz, Mohammad Raza, Cariad Evans
EPI_ISL_637437	Wales Specialist Virology Centre Sequencing lab: Pathogen Genomics Unit	COVID-19 Genomics UK (COG-UK) Consortium	Catherine Moore, Johnathan Evans, Laura Gifford, Malorie Perry, Simon Cottrell, Angela Marchbank, Alec Birchley, Alexander Adams, Amy Gaskin, Bree Gatica-Wilcox, Jason Coombes, Joel Southgate, Lauren Gilbert, Lee Graham, Nicole Pacchiarini, Sara Kumziene-Summerhayes, Sarah Taylor, Sophie Jones, Sara Rey, Matthew Bull, Joanne Watkins, Sally Corden, Tom Connor
EPI_ISL_637438, EPI_ISL_637439	Liverpool Clinical Laboratories	COVID-19 Genomics UK (COG-UK) Consortium	Sam Haldenby, Anita Lucaci, Steve Paterson, Julian Hiscox, Alistair Darby, M Almsaud, A Alrezaihi, Muhannad Alruwaili, Stuart D Armstrong, Jones Benjamin, Eleanor G Bentley, Anu Chawla, Jordan J Clark, Angela Cowell, Richard Eccles, Isabel García-Dorival, Matthew Gemmell, Alessandro Gerada, PKF Gilmore, Richard Gregory, Ximeng Han, Catherine Hartley, Margaret Hughes, Miren Iturriza-Gomara, James Johnson, L Luu, Jenifer Manson, Charlotte Nelson, Elaine O'Toole, Cassie Olateju, Rebekah Penrice-Randal , Lucille Rainbow, N.P Randle, Trevor Ian Robinson, Parul Sharma, Ghada T Shawli, James P Stewart, Neil Swainston, Ecaterina Vamos, Joanne Watts, Mark Whitehead
EPI_ISL_637440	Department of Pathology, University of Cambridge	COVID-19 Genomics UK (COG-UK) Consortium	Aminu S. Jahun, Yasmin Chaudhry, Grant Hall, Iliana Georgana, Myra Hosmillo, Martin D. Curran, Malte Pinckert, Surendra Parmar, Ian Goodfellow
EPI_ISL_637442	Quadram Institute Bioscience	COVID-19 Genomics UK (COG-UK) Consortium	Dave J. Baker, Gemma L. Kay, Alp Aydin, Thanh Le-Viet, Steven Rudder, Ana P. Tedim, Anastasia Kolyva, Maria Diaz, Leonardo de Oliveira Martins, Nabil-Fareed Alikhan, Lizzie Meadows, Rachael Stanley, Ngozi Elumogo, Muhammed Yasir, Nicholas M. Thomson, Alexander J Trotter, Rachel Gilroy, Samuel Bloomfield, Claire Stuart, Andrew Bell, Reenesh Prakash, Samir Dervisevic, Alison E. Mather, John Wain, Mark Webber, Andrew J. Page, Justin O'Grady
EPI_ISL_637443, EPI_ISL_637444	Liverpool Clinical Laboratories	COVID-19 Genomics UK (COG-UK) Consortium	Sam Haldenby, Anita Lucaci, Steve Paterson, Julian Hiscox, Alistair Darby, M Almsaud, A Alrezaihi, Muhannad Alruwaili, Stuart D Armstrong, Jones Benjamin, Eleanor G Bentley, Anu Chawla, Jordan J Clark, Angela Cowell, Richard Eccles, Isabel García-Dorival, Matthew Gemmell, Alessandro Gerada, PKF Gilmore, Richard Gregory, Ximeng Han, Catherine Hartley, Margaret Hughes, Miren Iturriza-Gomara, James Johnson, L Luu, Jenifer Manson, Charlotte Nelson, Elaine O'Toole, Cassie Olateju, Rebekah Penrice-Randal , Lucille Rainbow, N.P Randle, Trevor Ian Robinson, Parul Sharma, Ghada T Shawli, James P Stewart, Neil Swainston, Ecaterina Vamos, Joanne Watts, Mark Whitehead
EPI_ISL_637445	Wales Specialist Virology Centre Sequencing lab: Pathogen Genomics Unit	COVID-19 Genomics UK (COG-UK) Consortium	Catherine Moore, Johnathan Evans, Laura Gifford, Malorie Perry, Simon Cottrell, Angela Marchbank, Alec Birchley, Alexander Adams, Amy Gaskin, Bree Gatica-Wilcox, Jason Coombes, Joel Southgate, Lauren Gilbert, Lee Graham, Nicole Pacchiarini, Sara Kumziene-Summerhayes, Sarah Taylor, Sophie Jones, Sara Rey, Matthew Bull, Joanne Watkins, Sally Corden, Tom Connor
EPI_ISL_637446	University College London, Great Ormond Street Hospital for Children NHS Foundation Trust, Imperial College Healthcare NHS Trust	COVID-19 Genomics UK (COG-UK) Consortium	Sergi Castellano, Rachel Williams, Mark Kristiansen, Paola Resende Silva, Sunando Roy, Tony Brooks, Helena Tutill, Paola Niola, Patricia Dyal, Charlotte Williams, Leysa Forrest, Yasmin Panchbhaya, Jacqueline Findlay, Samuel Weeks, Julianne Brown, Kathryn Harris, Paul Randell, James Price, Alison Holmes, Judith Breuer
EPI_ISL_637447, EPI_ISL_637448, EPI_ISL_637449, EPI_ISL_637450	Wales Specialist Virology Centre Sequencing lab: Pathogen Genomics Unit	COVID-19 Genomics UK (COG-UK) Consortium	Catherine Moore, Johnathan Evans, Laura Gifford, Malorie Perry, Simon Cottrell, Angela Marchbank, Alec Birchley, Alexander Adams, Amy Gaskin, Bree Gatica-Wilcox, Jason Coombes, Joel Southgate, Lauren Gilbert, Lee Graham, Nicole Pacchiarini, Sara Kumziene-Summerhayes, Sarah Taylor, Sophie Jones, Sara Rey, Matthew Bull, Joanne Watkins, Sally Corden, Tom Connor
EPI_ISL_637451	Department of Pathology, University of Cambridge	COVID-19 Genomics UK (COG-UK) Consortium	Aminu S. Jahun, Yasmin Chaudhry, Grant Hall, Iliana Georgana, Myra Hosmillo, Martin D. Curran, Malte Pinckert, Surendra Parmar, Ian Goodfellow
EPI_ISL_637452	Wales Specialist Virology Centre Sequencing lab: Pathogen	COVID-19 Genomics UK (COG-UK) Consortium	Catherine Moore, Johnathan Evans, Laura Gifford, Malorie Perry, Simon Cottrell, Angela Marchbank, Alec Birchley, Alexander Adams, Amy Gaskin, Bree

	Genomics Unit		Gatica-Wilcox, Jason Coombes, Joel Southgate, Lauren Gilbert, Lee Graham, Nicole Pacchiarini, Sara Kumziene-Summerhayes, Sarah Taylor, Sophie Jones, Sara Rey, Matthew Bull, Joanne Watkins, Sally Corden, Tom Connor
EPI_ISL_637453	Centre for Enzyme Innovation, University of Portsmouth / Translational Research Laboratory, Portsmouth Hospitals NHS Trust	COVID-19 Genomics UK (COG-UK) Consortium	Angela Beckett,Yann Bourgeois,Garry Scarlett,Sharon Glaysher,Scott Elliott,Kelly Bicknell,Robert Impey,Allyson Lloyd,Sarah Wyllie,Ethan Butcher,Anoop Chauhan,Samuel Robson
EPI_ISL_637454	Wales Specialist Virology Centre Sequencing lab: Pathogen Genomics Unit	COVID-19 Genomics UK (COG-UK) Consortium	Catherine Moore, Johnathan Evans, Laura Gifford, Malorie Perry, Simon Cottrell, Angela Marchbank, Alec Birchley, Alexander Adams, Amy Gaskin, Bree Gatica-Wilcox, Jason Coombes, Joel Southgate, Lauren Gilbert, Lee Graham, Nicole Pacchiarini, Sara Kumziene-Summerhayes, Sarah Taylor, Sophie Jones, Sara Rey, Matthew Bull, Joanne Watkins, Sally Corden, Tom Connor
EPI_ISL_637455	Northumbria University / South Tees Hospitals NHS Foundation Trust / North Cumbria Integrated Care NHS Foundation Trust / North Tees and Hartlepool NHS Foundation Trust / Newcastle Hospitals NHS Foundation Trust	COVID-19 Genomics UK (COG-UK) Consortium	Darren L Smith,Andrew Nelson,Matthew Bashton,Greg R Young,Joshua Loh,John Allan,Mohammad A Tariq,Giles S Holt,Gary Black,Wen C Yew,Lynn Dover,Paul Baker,Steve Liggett,Sarah Essex,Jane Greenaway,Debra Padgett,Clive Graham,Garren Scott,Edward Barton,Emma Swindells,Brendan Payne,Jennifer Collins,Yusri Taha,Gary Eltringham
EPI_ISL_637456, EPI_ISL_637457	Department of Pathology, University of Cambridge	COVID-19 Genomics UK (COG-UK) Consortium	Aminu S. Jahun, Yasmin Chaudhry, Grant Hall, Iliana Georgana, Myra Hosmillo, Martin D. Curran, Malte Pinckert, Surendra Parmar, Ian Goodfellow
EPI_ISL_637458	Wales Specialist Virology Centre Sequencing lab: Pathogen Genomics Unit	COVID-19 Genomics UK (COG-UK) Consortium	Catherine Moore, Johnathan Evans, Laura Gifford, Malorie Perry, Simon Cottrell, Angela Marchbank, Alec Birchley, Alexander Adams, Amy Gaskin, Bree Gatica-Wilcox, Jason Coombes, Joel Southgate, Lauren Gilbert, Lee Graham, Nicole Pacchiarini, Sara Kumziene-Summerhayes, Sarah Taylor, Sophie Jones, Sara Rey, Matthew Bull, Joanne Watkins, Sally Corden, Tom Connor
EPI_ISL_637459	Centre for Enzyme Innovation, University of Portsmouth / Translational Research Laboratory, Portsmouth Hospitals NHS Trust	COVID-19 Genomics UK (COG-UK) Consortium	Angela Beckett,Yann Bourgeois,Garry Scarlett,Sharon Glaysher,Scott Elliott,Kelly Bicknell,Robert Impey,Allyson Lloyd,Sarah Wyllie,Ethan Butcher,Anoop Chauhan,Samuel Robson
EPI_ISL_637460, EPI_ISL_637461, EPI_ISL_637462	Department of Pathology, University of Cambridge	COVID-19 Genomics UK (COG-UK) Consortium	Aminu S. Jahun, Yasmin Chaudhry, Grant Hall, Iliana Georgana, Myra Hosmillo, Martin D. Curran, Malte Pinckert, Surendra Parmar, Ian Goodfellow
EPI_ISL_637463	Northumbria University / South Tees Hospitals NHS Foundation Trust / North Cumbria Integrated Care NHS Foundation Trust / North Tees and Hartlepool NHS Foundation Trust / Newcastle Hospitals NHS Foundation Trust	COVID-19 Genomics UK (COG-UK) Consortium	Darren L Smith,Andrew Nelson,Matthew Bashton,Greg R Young,Joshua Loh,John Allan,Mohammad A Tariq,Giles S Holt,Gary Black,Wen C Yew,Lynn Dover,Paul Baker,Steve Liggett,Sarah Essex,Jane Greenaway,Debra Padgett,Clive Graham,Garren Scott,Edward Barton,Emma Swindells,Brendan Payne,Jennifer Collins,Yusri Taha,Gary Eltringham
EPI_ISL_637464, EPI_ISL_637465	Department of Pathology, University of Cambridge	COVID-19 Genomics UK (COG-UK) Consortium	Aminu S. Jahun, Yasmin Chaudhry, Grant Hall, Iliana Georgana, Myra Hosmillo, Martin D. Curran, Malte Pinckert, Surendra Parmar, Ian Goodfellow
EPI_ISL_637466, EPI_ISL_637467	Centre for Enzyme Innovation, University of Portsmouth / Translational Research Laboratory, Portsmouth Hospitals NHS Trust	COVID-19 Genomics UK (COG-UK) Consortium	Angela Beckett,Yann Bourgeois,Garry Scarlett,Sharon Glaysher,Scott Elliott,Kelly Bicknell,Robert Impey,Allyson Lloyd,Sarah Wyllie,Ethan Butcher,Anoop Chauhan,Samuel Robson
EPI_ISL_637468, EPI_ISL_637469, EPI_ISL_637470, EPI_ISL_637471, EPI_ISL_637472, EPI_ISL_637473, EPI_ISL_637474, EPI_ISL_637475, EPI_ISL_637476, EPI_ISL_637477, EPI_ISL_637478, EPI_ISL_637479, EPI_ISL_637480, EPI_ISL_637481, EPI_ISL_637482, EPI_ISL_637483, EPI_ISL_637484, EPI_ISL_637485, EPI_ISL_637486, EPI_ISL_637487, EPI_ISL_637488, EPI_ISL_637489, EPI_ISL_637490			
see above	Department of Pathology, University of Cambridge	COVID-19 Genomics UK (COG-UK) Consortium	Aminu S. Jahun, Yasmin Chaudhry, Grant Hall, Iliana Georgana, Myra Hosmillo, Martin D. Curran, Malte Pinckert, Surendra Parmar, Ian Goodfellow
EPI_ISL_637491, EPI_ISL_637492, EPI_ISL_637493, EPI_ISL_637494, EPI_ISL_637495, EPI_ISL_637496, EPI_ISL_637497, EPI_ISL_637498, EPI_ISL_637499	Centre for Enzyme Innovation, University of Portsmouth / Translational Research Laboratory, Portsmouth Hospitals NHS Trust	COVID-19 Genomics UK (COG-UK) Consortium	Angela Beckett,Yann Bourgeois,Garry Scarlett,Sharon Glaysher,Scott Elliott,Kelly Bicknell,Robert Impey,Allyson Lloyd,Sarah Wyllie,Ethan Butcher,Anoop Chauhan,Samuel Robson
EPI_ISL_637500, EPI_ISL_637501	Department of Pathology, University of Cambridge	COVID-19 Genomics UK (COG-UK) Consortium	Aminu S. Jahun, Yasmin Chaudhry, Grant Hall, Iliana Georgana, Myra Hosmillo, Martin D. Curran, Malte Pinckert, Surendra Parmar, Ian Goodfellow
EPI_ISL_637502	Centre for Enzyme Innovation, University of Portsmouth / Translational Research Laboratory, Portsmouth Hospitals NHS Trust	COVID-19 Genomics UK (COG-UK) Consortium	Angela Beckett,Yann Bourgeois,Garry Scarlett,Sharon Glaysher,Scott Elliott,Kelly Bicknell,Robert Impey,Allyson Lloyd,Sarah Wyllie,Ethan Butcher,Anoop Chauhan,Samuel Robson
EPI_ISL_637503, EPI_ISL_637504, EPI_ISL_637505, EPI_ISL_637506, EPI_ISL_637507, EPI_ISL_637508, EPI_ISL_637509, EPI_ISL_637510, EPI_ISL_637511, EPI_ISL_637512, EPI_ISL_637513, EPI_ISL_637514, EPI_ISL_637515, EPI_ISL_637516, EPI_ISL_637517, EPI_ISL_637518, EPI_ISL_637519			
see above	Department of Pathology, University of Cambridge	COVID-19 Genomics UK (COG-UK) Consortium	Aminu S. Jahun, Yasmin Chaudhry, Grant Hall, Iliana Georgana, Myra Hosmillo, Martin D. Curran, Malte Pinckert, Surendra Parmar, Ian Goodfellow
EPI_ISL_637520, EPI_ISL_637521, EPI_ISL_637522, EPI_ISL_637523	Northumbria University / South Tees Hospitals NHS Foundation Trust / North Cumbria Integrated Care NHS Foundation Trust / North Tees and Hartlepool NHS Foundation Trust / Newcastle Hospitals NHS Foundation Trust	COVID-19 Genomics UK (COG-UK) Consortium	Darren L Smith,Andrew Nelson,Matthew Bashton,Greg R Young,Joshua Loh,John Allan,Mohammad A Tariq,Giles S Holt,Gary Black,Wen C Yew,Lynn Dover,Paul Baker,Steve Liggett,Sarah Essex,Jane Greenaway,Debra Padgett,Clive Graham,Garren Scott,Edward Barton,Emma Swindells,Brendan Payne,Jennifer Collins,Yusri Taha,Gary Eltringham
EPI_ISL_637524, EPI_ISL_637525	Wales Specialist Virology Centre Sequencing lab: Pathogen Genomics Unit	COVID-19 Genomics UK (COG-UK) Consortium	Catherine Moore, Johnathan Evans, Laura Gifford, Malorie Perry, Simon Cottrell, Angela Marchbank, Alec Birchley, Alexander Adams, Amy Gaskin, Bree Gatica-Wilcox, Jason Coombes, Joel Southgate, Lauren Gilbert, Lee Graham, Nicole Pacchiarini, Sara Kumziene-Summerhayes, Sarah Taylor, Sophie Jones, Sara Rey, Matthew Bull, Joanne Watkins, Sally Corden, Tom Connor
EPI_ISL_637526, EPI_ISL_637527	Department of Pathology, University of Cambridge	COVID-19 Genomics UK (COG-UK) Consortium	Aminu S. Jahun, Yasmin Chaudhry, Grant Hall, Iliana Georgana, Myra Hosmillo, Martin D. Curran, Malte Pinckert, Surendra Parmar, Ian Goodfellow
EPI_ISL_637528	Centre for Enzyme Innovation, University of Portsmouth / Translational Research Laboratory, Portsmouth Hospitals NHS Trust	COVID-19 Genomics UK (COG-UK) Consortium	Angela Beckett,Yann Bourgeois,Garry Scarlett,Sharon Glaysher,Scott Elliott,Kelly Bicknell,Robert Impey,Allyson Lloyd,Sarah Wyllie,Ethan Butcher,Anoop Chauhan,Samuel Robson
EPI_ISL_637529, EPI_ISL_637530	Department of Pathology, University of Cambridge	COVID-19 Genomics UK (COG-UK) Consortium	Aminu S. Jahun, Yasmin Chaudhry, Grant Hall, Iliana Georgana, Myra Hosmillo, Martin D. Curran, Malte Pinckert, Surendra Parmar, Ian Goodfellow
EPI_ISL_637531	Virology Department, Sheffield Teaching Hospitals NHS Foundation Trust/Department of Infection, Immunity and Cardiovascular Disease, The Medical School, University of Sheffield	COVID-19 Genomics UK (COG-UK) Consortium	Thushan de Silva, Matthew Parker, Nikki Smith, Adri Angyal, Rebecca Brown, Luke Green, Rachel Tucker, Paul Parsons, Danielle Groves, Katie Johnson, Laura Carrilero, Alex Keeley, Dave Partridge, Matthew Wyles, Benjamin Lindsey, Mehmet Yavuz, Mohammad Raza, Cariad Evans
EPI_ISL_637532	Department of Pathology, University of Cambridge	COVID-19 Genomics UK (COG-UK) Consortium	Aminu S. Jahun, Yasmin Chaudhry, Grant Hall, Iliana Georgana, Myra Hosmillo, Martin D. Curran, Malte Pinckert, Surendra Parmar, Ian Goodfellow
EPI_ISL_637533	Virology Department, Sheffield Teaching Hospitals NHS Foundation Trust/Department of Infection, Immunity and Cardiovascular Disease, The Medical School, University of Sheffield	COVID-19 Genomics UK (COG-UK) Consortium	Thushan de Silva, Matthew Parker, Nikki Smith, Adri Angyal, Rebecca Brown, Luke Green, Rachel Tucker, Paul Parsons, Danielle Groves, Katie Johnson, Laura Carrilero, Alex Keeley, Dave Partridge, Matthew Wyles, Benjamin Lindsey, Mehmet Yavuz, Mohammad Raza, Cariad Evans
EPI_ISL_637534	Department of Pathology, University of Cambridge	COVID-19 Genomics UK (COG-UK) Consortium	Aminu S. Jahun, Yasmin Chaudhry, Grant Hall, Iliana Georgana, Myra Hosmillo, Martin D. Curran, Malte Pinckert, Surendra Parmar, Ian Goodfellow
EPI_ISL_637535	Virology Department, Sheffield Teaching Hospitals NHS Foundation Trust/Department of Infection, Immunity and Cardiovascular Disease, The Medical School, University of Sheffield	COVID-19 Genomics UK (COG-UK) Consortium	Thushan de Silva, Matthew Parker, Nikki Smith, Adri Angyal, Rebecca Brown, Luke Green, Rachel Tucker, Paul Parsons, Danielle Groves, Katie Johnson, Laura Carrilero, Alex Keeley, Dave Partridge, Matthew Wyles, Benjamin Lindsey, Mehmet Yavuz, Mohammad Raza, Cariad Evans
EPI_ISL_637536	Quadram Institute Bioscience	COVID-19 Genomics UK (COG-UK) Consortium	Dave J. Baker, Gemma L. Kay, Alp Aydin, Thanh Le-Viet, Steven Rudder, Ana P. Tedim, Anastasia Kolyva, Maria Diaz, Leonardo de Oliveira Martins, Nabil-Fareed Alikhan, Lizzie Meadows, Rachael Stanley, Ngozi Elumogo, Muhammed Yasir, Nicholas M. Thomson, Alexander J Trotter, Rachel Gilroy, Samuel Bloomfield, Claire Stuart, Andrew Bell, Reenesh Prakash, Samir Dervisevic, Alison E. Mather, John Wain, Mark Webber, Andrew J. Page, Justin O'Grady

EPI_ISL_637537	Wales Specialist Virology Centre Sequencing lab: Pathogen Genomics Unit	COVID-19 Genomics UK (COG-UK) Consortium	Catherine Moore, Johnathan Evans, Laura Gifford, Malorie Perry, Simon Cottrell, Angela Marchbank, Alec Birchley, Alexander Adams, Amy Gaskin, Bree Gatica-Wilcox, Jason Coombes, Joel Southgate, Lauren Gilbert, Lee Graham, Nicole Pacchiarini, Sara Kumziene-Summerhayes, Sarah Taylor, Sophie Jones, Sara Rey, Matthew Bull, Joanne Watkins, Sally Corden, Tom Connor
EPI_ISL_637538, EPI_ISL_637539, EPI_ISL_637540, EPI_ISL_637541	Virology Department, Sheffield Teaching Hospitals NHS Foundation Trust/Department of Infection, Immunity and Cardiovascular Disease, The Medical School, University of Sheffield	COVID-19 Genomics UK (COG-UK) Consortium	Thushan de Silva, Matthew Parker, Nikki Smith, Adri Angyal, Rebecca Brown, Luke Green, Rachel Tucker, Paul Parsons, Danielle Groves, Katie Johnson, Laura Carrilero, Alex Keeley, Dave Partridge, Matthew Wyles, Benjamin Lindsey, Mehmet Yavuz, Mohammad Raza, Cariad Evans
EPI_ISL_637542, EPI_ISL_637543, EPI_ISL_637544	Wales Specialist Virology Centre Sequencing lab: Pathogen Genomics Unit	COVID-19 Genomics UK (COG-UK) Consortium	Catherine Moore, Johnathan Evans, Laura Gifford, Malorie Perry, Simon Cottrell, Angela Marchbank, Alec Birchley, Alexander Adams, Amy Gaskin, Bree Gatica-Wilcox, Jason Coombes, Joel Southgate, Lauren Gilbert, Lee Graham, Nicole Pacchiarini, Sara Kumziene-Summerhayes, Sarah Taylor, Sophie Jones, Sara Rey, Matthew Bull, Joanne Watkins, Sally Corden, Tom Connor
EPI_ISL_637545	Department of Pathology, University of Cambridge	COVID-19 Genomics UK (COG-UK) Consortium	Aminu S. Jahun, Yasmin Chaudhry, Grant Hall, Iliana Georgana, Myra Hosmillo, Martin D. Curran, Malte Pinckert, Surendra Parmar, Ian Goodfellow
EPI_ISL_637546	Wales Specialist Virology Centre Sequencing lab: Pathogen Genomics Unit	COVID-19 Genomics UK (COG-UK) Consortium	Catherine Moore, Johnathan Evans, Laura Gifford, Malorie Perry, Simon Cottrell, Angela Marchbank, Alec Birchley, Alexander Adams, Amy Gaskin, Bree Gatica-Wilcox, Jason Coombes, Joel Southgate, Lauren Gilbert, Lee Graham, Nicole Pacchiarini, Sara Kumziene-Summerhayes, Sarah Taylor, Sophie Jones, Sara Rey, Matthew Bull, Joanne Watkins, Sally Corden, Tom Connor
EPI_ISL_637547, EPI_ISL_637548, EPI_ISL_637549	Department of Pathology, University of Cambridge	COVID-19 Genomics UK (COG-UK) Consortium	Aminu S. Jahun, Yasmin Chaudhry, Grant Hall, Iliana Georgana, Myra Hosmillo, Martin D. Curran, Malte Pinckert, Surendra Parmar, Ian Goodfellow
EPI_ISL_637550	Quadram Institute Bioscience	COVID-19 Genomics UK (COG-UK) Consortium	Dave J. Baker, Gemma L. Kay, Alp Aydin, Thanh Le-Viet, Steven Rudder, Ana P. Tedim, Anastasia Kolyva, Maria Diaz, Leonardo de Oliveira Martins, Nabil-Fareed Alikhan, Lizzie Meadows, Rachael Stanley, Ngozi Elumogo, Muhammed Yasir, Nicholas M. Thomson, Alexander J Trotter, Rachel Gilroy, Samuel Bloomfield, Claire Stuart, Andrew Bell, Reenesh Prakash, Samir Dervisevic, Alison E. Mather, John Wain, Mark Webber, Andrew J. Page, Justin O'Grady
EPI_ISL_637551	Wales Specialist Virology Centre Sequencing lab: Pathogen Genomics Unit	COVID-19 Genomics UK (COG-UK) Consortium	Catherine Moore, Johnathan Evans, Laura Gifford, Malorie Perry, Simon Cottrell, Angela Marchbank, Alec Birchley, Alexander Adams, Amy Gaskin, Bree Gatica-Wilcox, Jason Coombes, Joel Southgate, Lauren Gilbert, Lee Graham, Nicole Pacchiarini, Sara Kumziene-Summerhayes, Sarah Taylor, Sophie Jones, Sara Rey, Matthew Bull, Joanne Watkins, Sally Corden, Tom Connor
EPI_ISL_637552	Department of Pathology, University of Cambridge	COVID-19 Genomics UK (COG-UK) Consortium	Aminu S. Jahun, Yasmin Chaudhry, Grant Hall, Iliana Georgana, Myra Hosmillo, Martin D. Curran, Malte Pinckert, Surendra Parmar, Ian Goodfellow
EPI_ISL_637553	Virology Department, Sheffield Teaching Hospitals NHS Foundation Trust/Department of Infection, Immunity and Cardiovascular Disease, The Medical School, University of Sheffield	COVID-19 Genomics UK (COG-UK) Consortium	Thushan de Silva, Matthew Parker, Nikki Smith, Adri Angyal, Rebecca Brown, Luke Green, Rachel Tucker, Paul Parsons, Danielle Groves, Katie Johnson, Laura Carrilero, Alex Keeley, Dave Partridge, Matthew Wyles, Benjamin Lindsey, Mehmet Yavuz, Mohammad Raza, Cariad Evans
EPI_ISL_637554	Department of Pathology, University of Cambridge	COVID-19 Genomics UK (COG-UK) Consortium	Aminu S. Jahun, Yasmin Chaudhry, Grant Hall, Iliana Georgana, Myra Hosmillo, Martin D. Curran, Malte Pinckert, Surendra Parmar, Ian Goodfellow
EPI_ISL_637555	Queens Medical Centre, Clinical Microbiology Department / DeepSeq Nottingham	COVID-19 Genomics UK (COG-UK) Consortium	Gemma Clark, Wendy Smith, Manjinder Khakh, Vicki M Fleming, Michelle M Lister, Hannah Howson-Wells, Jonathan Ball, Patrick McClure, Joseph Chappell, Theocharis Tsoleridis, Nadine Holmes, Matthew Carlisle, Christopher Moore, Fei Sang, Johnny Debebe, Victoria Wright, Matthew Loose
EPI_ISL_637556	Wales Specialist Virology Centre Sequencing lab: Pathogen Genomics Unit	COVID-19 Genomics UK (COG-UK) Consortium	Catherine Moore, Johnathan Evans, Laura Gifford, Malorie Perry, Simon Cottrell, Angela Marchbank, Alec Birchley, Alexander Adams, Amy Gaskin, Bree Gatica-Wilcox, Jason Coombes, Joel Southgate, Lauren Gilbert, Lee Graham, Nicole Pacchiarini, Sara Kumziene-Summerhayes, Sarah Taylor, Sophie Jones, Sara Rey, Matthew Bull, Joanne Watkins, Sally Corden, Tom Connor
EPI_ISL_637557	Quadram Institute Bioscience	COVID-19 Genomics UK (COG-UK) Consortium	Dave J. Baker, Gemma L. Kay, Alp Aydin, Thanh Le-Viet, Steven Rudder, Ana P. Tedim, Anastasia Kolyva, Maria Diaz, Leonardo de Oliveira Martins, Nabil-Fareed Alikhan, Lizzie Meadows, Rachael Stanley, Ngozi Elumogo, Muhammed Yasir, Nicholas M. Thomson, Alexander J Trotter, Rachel Gilroy, Samuel Bloomfield, Claire Stuart, Andrew Bell, Reenesh Prakash, Samir Dervisevic, Alison E. Mather, John Wain, Mark Webber, Andrew J. Page, Justin O'Grady
EPI_ISL_637558	Queens Medical Centre, Clinical Microbiology Department / DeepSeq Nottingham	COVID-19 Genomics UK (COG-UK) Consortium	Gemma Clark, Wendy Smith, Manjinder Khakh, Vicki M Fleming, Michelle M Lister, Hannah Howson-Wells, Jonathan Ball, Patrick McClure, Joseph Chappell, Theocharis Tsoleridis, Nadine Holmes, Matthew Carlisle, Christopher Moore, Fei Sang, Johnny Debebe, Victoria Wright, Matthew Loose
EPI_ISL_637559	Virology Department, Sheffield Teaching Hospitals NHS Foundation Trust/Department of Infection, Immunity and Cardiovascular Disease, The Medical School, University of Sheffield	COVID-19 Genomics UK (COG-UK) Consortium	Thushan de Silva, Matthew Parker, Nikki Smith, Adri Angyal, Rebecca Brown, Luke Green, Rachel Tucker, Paul Parsons, Danielle Groves, Katie Johnson, Laura Carrilero, Alex Keeley, Dave Partridge, Matthew Wyles, Benjamin Lindsey, Mehmet Yavuz, Mohammad Raza, Cariad Evans
EPI_ISL_637560	Department of Pathology, University of Cambridge	COVID-19 Genomics UK (COG-UK) Consortium	Aminu S. Jahun, Yasmin Chaudhry, Grant Hall, Iliana Georgana, Myra Hosmillo, Martin D. Curran, Malte Pinckert, Surendra Parmar, Ian Goodfellow
EPI_ISL_637561	Virology Department, Sheffield Teaching Hospitals NHS Foundation Trust/Department of Infection, Immunity and Cardiovascular Disease, The Medical School, University of Sheffield	COVID-19 Genomics UK (COG-UK) Consortium	Thushan de Silva, Matthew Parker, Nikki Smith, Adri Angyal, Rebecca Brown, Luke Green, Rachel Tucker, Paul Parsons, Danielle Groves, Katie Johnson, Laura Carrilero, Alex Keeley, Dave Partridge, Matthew Wyles, Benjamin Lindsey, Mehmet Yavuz, Mohammad Raza, Cariad Evans
EPI_ISL_637562, EPI_ISL_637563, EPI_ISL_637564, EPI_ISL_637565, EPI_ISL_637566, EPI_ISL_637567, EPI_ISL_637568, EPI_ISL_637569, EPI_ISL_637570, EPI_ISL_637571, EPI_ISL_637572, EPI_ISL_637573, EPI_ISL_637574, EPI_ISL_637575, EPI_ISL_637576, EPI_ISL_637577, EPI_ISL_637578, EPI_ISL_637579			
see above	Department of Pathology, University of Cambridge	COVID-19 Genomics UK (COG-UK) Consortium	Aminu S. Jahun, Yasmin Chaudhry, Grant Hall, Iliana Georgana, Myra Hosmillo, Martin D. Curran, Malte Pinckert, Surendra Parmar, Ian Goodfellow
EPI_ISL_637580	Virology Department, Sheffield Teaching Hospitals NHS Foundation Trust/Department of Infection, Immunity and Cardiovascular Disease, The Medical School, University of Sheffield	COVID-19 Genomics UK (COG-UK) Consortium	Thushan de Silva, Matthew Parker, Nikki Smith, Adri Angyal, Rebecca Brown, Luke Green, Rachel Tucker, Paul Parsons, Danielle Groves, Katie Johnson, Laura Carrilero, Alex Keeley, Dave Partridge, Matthew Wyles, Benjamin Lindsey, Mehmet Yavuz, Mohammad Raza, Cariad Evans
EPI_ISL_637581	Northumbria University / South Tees Hospitals NHS Foundation Trust / North Cumbria Integrated Care NHS Foundation Trust / North Tees and Hartlepool NHS Foundation Trust / Newcastle Hospitals NHS Foundation Trust	COVID-19 Genomics UK (COG-UK) Consortium	Darren L Smith,Andrew Nelson,Matthew Bashton,Greg R Young,Joshua Loh,John Allan,Mohammad A Tariq,Giles S Holt,Gary Black,Wen C Yew,Lynn Dover,Paul Baker,Steve Liggett,Sarah Essex,Jane Greenaway,Debra Padgett,Clive Graham,Garren Scott,Edward Barton,Emma Swindells,Brendan Payne,Jennifer Collins,Yusri Taha,Gary Eltringham
EPI_ISL_637582	Queens Medical Centre, Clinical Microbiology Department / DeepSeq Nottingham	COVID-19 Genomics UK (COG-UK) Consortium	Gemma Clark, Wendy Smith, Manjinder Khakh, Vicki M Fleming, Michelle M Lister, Hannah Howson-Wells, Jonathan Ball, Patrick McClure, Joseph Chappell, Theocharis Tsoleridis, Nadine Holmes, Matthew Carlisle, Christopher Moore, Fei Sang, Johnny Debebe, Victoria Wright, Matthew Loose
EPI_ISL_637583	Department of Pathology, University of Cambridge	COVID-19 Genomics UK (COG-UK) Consortium	Aminu S. Jahun, Yasmin Chaudhry, Grant Hall, Iliana Georgana, Myra Hosmillo, Martin D. Curran, Malte Pinckert, Surendra Parmar, Ian Goodfellow
EPI_ISL_637584, EPI_ISL_637585	Virology Department, Sheffield Teaching Hospitals NHS Foundation Trust/Department of Infection, Immunity and Cardiovascular Disease, The Medical School, University of Sheffield	COVID-19 Genomics UK (COG-UK) Consortium	Thushan de Silva, Matthew Parker, Nikki Smith, Adri Angyal, Rebecca Brown, Luke Green, Rachel Tucker, Paul Parsons, Danielle Groves, Katie Johnson, Laura Carrilero, Alex Keeley, Dave Partridge, Matthew Wyles, Benjamin Lindsey, Mehmet Yavuz, Mohammad Raza, Cariad Evans
EPI_ISL_637586, EPI_ISL_637587	Wales Specialist Virology Centre Sequencing lab: Pathogen Genomics Unit	COVID-19 Genomics UK (COG-UK) Consortium	Catherine Moore, Johnathan Evans, Laura Gifford, Malorie Perry, Simon Cottrell, Angela Marchbank, Alec Birchley, Alexander Adams, Amy Gaskin, Bree Gatica-Wilcox, Jason Coombes, Joel Southgate, Lauren Gilbert, Lee Graham, Nicole Pacchiarini, Sara Kumziene-Summerhayes, Sarah Taylor, Sophie Jones, Sara Rey, Matthew Bull, Joanne Watkins, Sally Corden, Tom Connor
EPI_ISL_637588, EPI_ISL_637589	Quadram Institute Bioscience	COVID-19 Genomics UK (COG-UK) Consortium	Dave J. Baker, Gemma L. Kay, Alp Aydin, Thanh Le-Viet, Steven Rudder, Ana P. Tedim, Anastasia Kolyva, Maria Diaz, Leonardo de Oliveira Martins, Nabil-Fareed Alikhan, Lizzie Meadows, Rachael Stanley, Ngozi Elumogo, Muhammed Yasir, Nicholas M. Thomson, Alexander J Trotter, Rachel Gilroy, Samuel Bloomfield, Claire Stuart, Andrew Bell, Reenesh Prakash, Samir Dervisevic, Alison E. Mather, John Wain, Mark Webber, Andrew J. Page, Justin O'Grady
EPI_ISL_637590, EPI_ISL_637591,	Department of Pathology, University of Cambridge	COVID-19 Genomics UK (COG-UK) Consortium	Aminu S. Jahun, Yasmin Chaudhry, Grant Hall, Iliana Georgana, Myra Hosmillo, Martin D. Curran, Malte Pinckert, Surendra Parmar, Ian Goodfellow

EPI_ISL_637592			
EPI_ISL_637593	Wales Specialist Virology Centre Sequencing lab: Pathogen Genomics Unit	COVID-19 Genomics UK (COG-UK) Consortium	Catherine Moore, Johnathan Evans, Laura Gifford, Malorie Perry, Simon Cottrell, Angela Marchbank, Alec Birchley, Alexander Adams, Amy Gaskin, Bree Gatica-Wilcox, Jason Coombes, Joel Southgate, Lauren Gilbert, Lee Graham, Nicole Pacchiarini, Sara Kumziene-Summerhayes, Sarah Taylor, Sophie Jones, Sara Rey, Matthew Bull, Joanne Watkins, Sally Corden, Tom Connor
EPI_ISL_637594	Virology Department, Sheffield Teaching Hospitals NHS Foundation Trust/Department of Infection, Immunity and Cardiovascular Disease, The Medical School, University of Sheffield	COVID-19 Genomics UK (COG-UK) Consortium	Thushan de Silva, Matthew Parker, Nikki Smith, Adri Angyal, Rebecca Brown, Luke Green, Rachel Tucker, Paul Parsons, Danielle Groves, Katie Johnson, Laura Carrilero, Alex Keeley, Dave Partridge, Matthew Wyles, Benjamin Lindsey, Mehmet Yavuz, Mohammad Raza, Cariad Evans
EPI_ISL_637595	Department of Pathology, University of Cambridge	COVID-19 Genomics UK (COG-UK) Consortium	Aminu S. Jahun, Yasmin Chaudhry, Grant Hall, Iliana Georgana, Myra Hosmillo, Martin D. Curran, Malte Pinckert, Surendra Parmar, Ian Goodfellow
EPI_ISL_637596, EPI_ISL_637597	Wales Specialist Virology Centre Sequencing lab: Pathogen Genomics Unit	COVID-19 Genomics UK (COG-UK) Consortium	Catherine Moore, Johnathan Evans, Laura Gifford, Malorie Perry, Simon Cottrell, Angela Marchbank, Alec Birchley, Alexander Adams, Amy Gaskin, Bree Gatica-Wilcox, Jason Coombes, Joel Southgate, Lauren Gilbert, Lee Graham, Nicole Pacchiarini, Sara Kumziene-Summerhayes, Sarah Taylor, Sophie Jones, Sara Rey, Matthew Bull, Joanne Watkins, Sally Corden, Tom Connor
EPI_ISL_637598	Department of Pathology, University of Cambridge	COVID-19 Genomics UK (COG-UK) Consortium	Aminu S. Jahun, Yasmin Chaudhry, Grant Hall, Iliana Georgana, Myra Hosmillo, Martin D. Curran, Malte Pinckert, Surendra Parmar, Ian Goodfellow
EPI_ISL_637599, EPI_ISL_637600, EPI_ISL_637601	Wales Specialist Virology Centre Sequencing lab: Pathogen Genomics Unit	COVID-19 Genomics UK (COG-UK) Consortium	Catherine Moore, Johnathan Evans, Laura Gifford, Malorie Perry, Simon Cottrell, Angela Marchbank, Alec Birchley, Alexander Adams, Amy Gaskin, Bree Gatica-Wilcox, Jason Coombes, Joel Southgate, Lauren Gilbert, Lee Graham, Nicole Pacchiarini, Sara Kumziene-Summerhayes, Sarah Taylor, Sophie Jones, Sara Rey, Matthew Bull, Joanne Watkins, Sally Corden, Tom Connor
EPI_ISL_637602	Quadram Institute Bioscience	COVID-19 Genomics UK (COG-UK) Consortium	Dave J. Baker, Gemma L. Kay, Alp Aydin, Thanh Le-Viet, Steven Rudder, Ana P. Tedim, Anastasia Kolyva, Maria Diaz, Leonardo de Oliveira Martins, Nabil-Fareed Alikhan, Lizzie Meadows, Rachael Stanley, Ngozi Elumogo, Muhammed Yasir, Nicholas M. Thomson, Alexander J Trotter, Rachel Gilroy, Samuel Bloomfield, Claire Stuart, Andrew Bell, Reenesh Prakash, Samir Dervisevic, Alison E. Mather, John Wain, Mark Webber, Andrew J. Page, Justin O'Grady
EPI_ISL_637603, EPI_ISL_637604	Wales Specialist Virology Centre Sequencing lab: Pathogen Genomics Unit	COVID-19 Genomics UK (COG-UK) Consortium	Catherine Moore, Johnathan Evans, Laura Gifford, Malorie Perry, Simon Cottrell, Angela Marchbank, Alec Birchley, Alexander Adams, Amy Gaskin, Bree Gatica-Wilcox, Jason Coombes, Joel Southgate, Lauren Gilbert, Lee Graham, Nicole Pacchiarini, Sara Kumziene-Summerhayes, Sarah Taylor, Sophie Jones, Sara Rey, Matthew Bull, Joanne Watkins, Sally Corden, Tom Connor
EPI_ISL_637605	Northumbria University / South Tees Hospitals NHS Foundation Trust / North Cumbria Integrated Care NHS Foundation Trust / North Tees and Hartlepool NHS Foundation Trust / Newcastle Hospitals NHS Foundation Trust	COVID-19 Genomics UK (COG-UK) Consortium	Darren L Smith,Andrew Nelson,Matthew Bashton,Greg R Young,Joshua Loh,John Allan,Mohammad A Tariq,Giles S Holt,Gary Black,Wen C Yew,Lynn Dover,Paul Baker,Steve Liggett,Sarah Essex,Jane Greenaway,Debra Padgett,Clive Graham,Garren Scott,Edward Barton,Emma Swindells,Brendan Payne,Jennifer Collins,Yusri Taha,Gary Eltringham
EPI_ISL_637606, EPI_ISL_637607, EPI_ISL_637608, EPI_ISL_637609	Wales Specialist Virology Centre Sequencing lab: Pathogen Genomics Unit	COVID-19 Genomics UK (COG-UK) Consortium	Catherine Moore, Johnathan Evans, Laura Gifford, Malorie Perry, Simon Cottrell, Angela Marchbank, Alec Birchley, Alexander Adams, Amy Gaskin, Bree Gatica-Wilcox, Jason Coombes, Joel Southgate, Lauren Gilbert, Lee Graham, Nicole Pacchiarini, Sara Kumziene-Summerhayes, Sarah Taylor, Sophie Jones, Sara Rey, Matthew Bull, Joanne Watkins, Sally Corden, Tom Connor
EPI_ISL_637610	Centre for Enzyme Innovation, University of Portsmouth / Translational Research Laboratory, Portsmouth Hospitals NHS Trust	COVID-19 Genomics UK (COG-UK) Consortium	Angela Beckett,Yann Bourgeois,Garry Scarlett,Sharon Glaysher,Scott Elliott,Kelly Bicknell,Robert Impey,Allyson Lloyd,Sarah Wyllie,Ethan Butcher,Anoop Chauhan,Samuel Robson
EPI_ISL_637611, EPI_ISL_637612	Wales Specialist Virology Centre Sequencing lab: Pathogen Genomics Unit	COVID-19 Genomics UK (COG-UK) Consortium	Catherine Moore, Johnathan Evans, Laura Gifford, Malorie Perry, Simon Cottrell, Angela Marchbank, Alec Birchley, Alexander Adams, Amy Gaskin, Bree Gatica-Wilcox, Jason Coombes, Joel Southgate, Lauren Gilbert, Lee Graham, Nicole Pacchiarini, Sara Kumziene-Summerhayes, Sarah Taylor, Sophie Jones, Sara Rey, Matthew Bull, Joanne Watkins, Sally Corden, Tom Connor
EPI_ISL_637613	Department of Pathology, University of Cambridge	COVID-19 Genomics UK (COG-UK) Consortium	Aminu S. Jahun, Yasmin Chaudhry, Grant Hall, Iliana Georgana, Myra Hosmillo, Martin D. Curran, Malte Pinckert, Surendra Parmar, Ian Goodfellow
EPI_ISL_637614, EPI_ISL_637615, EPI_ISL_637616	Wales Specialist Virology Centre Sequencing lab: Pathogen Genomics Unit	COVID-19 Genomics UK (COG-UK) Consortium	Catherine Moore, Johnathan Evans, Laura Gifford, Malorie Perry, Simon Cottrell, Angela Marchbank, Alec Birchley, Alexander Adams, Amy Gaskin, Bree Gatica-Wilcox, Jason Coombes, Joel Southgate, Lauren Gilbert, Lee Graham, Nicole Pacchiarini, Sara Kumziene-Summerhayes, Sarah Taylor, Sophie Jones, Sara Rey, Matthew Bull, Joanne Watkins, Sally Corden, Tom Connor
EPI_ISL_637617	Liverpool Clinical Laboratories	COVID-19 Genomics UK (COG-UK) Consortium	Sam Haldenby, Anita Lucaci, Steve Paterson, Julian Hiscox, Alistair Darby, M Almsaud, A Alrezaihi, Muhannad Alruwaili, Stuart D Armstrong, Jones Benjamin, Eleanor G Bentley, Anu Chawla, Jordan J Clark, Angela Cowell, Richard Eccles, Isabel García-Dorival, Matthew Gemmell, Alessandro Gerada, PKF Gilmore, Richard Gregory, Ximeng Han, Catherine Hartley, Margaret Hughes, Miren Iturriza-Gomara, James Johnson, L Luu, Jenifer Manson, Charlotte Nelson, Elaine O'Toole, Cassie Olateju, Rebekah Penrice-Randal , Lucille Rainbow, N.P Randle, Trevor Ian Robinson, Parul Sharma, Ghada T Shawli, James P Stewart, Neil Swainston, Ecaterina Vamos, Joanne Watts, Mark Whitehead
EPI_ISL_637618, EPI_ISL_637619	Wales Specialist Virology Centre Sequencing lab: Pathogen Genomics Unit	COVID-19 Genomics UK (COG-UK) Consortium	Catherine Moore, Johnathan Evans, Laura Gifford, Malorie Perry, Simon Cottrell, Angela Marchbank, Alec Birchley, Alexander Adams, Amy Gaskin, Bree Gatica-Wilcox, Jason Coombes, Joel Southgate, Lauren Gilbert, Lee Graham, Nicole Pacchiarini, Sara Kumziene-Summerhayes, Sarah Taylor, Sophie Jones, Sara Rey, Matthew Bull, Joanne Watkins, Sally Corden, Tom Connor
EPI_ISL_637620	Department of Pathology, University of Cambridge	COVID-19 Genomics UK (COG-UK) Consortium	Aminu S. Jahun, Yasmin Chaudhry, Grant Hall, Iliana Georgana, Myra Hosmillo, Martin D. Curran, Malte Pinckert, Surendra Parmar, Ian Goodfellow
EPI_ISL_637621	Virology Department, Sheffield Teaching Hospitals NHS Foundation Trust/Department of Infection, Immunity and Cardiovascular Disease, The Medical School, University of Sheffield	COVID-19 Genomics UK (COG-UK) Consortium	Thushan de Silva, Matthew Parker, Nikki Smith, Adri Angyal, Rebecca Brown, Luke Green, Rachel Tucker, Paul Parsons, Danielle Groves, Katie Johnson, Laura Carrilero, Alex Keeley, Dave Partridge, Matthew Wyles, Benjamin Lindsey, Mehmet Yavuz, Mohammad Raza, Cariad Evans
EPI_ISL_637622	Wales Specialist Virology Centre Sequencing lab: Pathogen Genomics Unit	COVID-19 Genomics UK (COG-UK) Consortium	Catherine Moore, Johnathan Evans, Laura Gifford, Malorie Perry, Simon Cottrell, Angela Marchbank, Alec Birchley, Alexander Adams, Amy Gaskin, Bree Gatica-Wilcox, Jason Coombes, Joel Southgate, Lauren Gilbert, Lee Graham, Nicole Pacchiarini, Sara Kumziene-Summerhayes, Sarah Taylor, Sophie Jones, Sara Rey, Matthew Bull, Joanne Watkins, Sally Corden, Tom Connor
EPI_ISL_637623, EPI_ISL_637624	Department of Pathology, University of Cambridge	COVID-19 Genomics UK (COG-UK) Consortium	Aminu S. Jahun, Yasmin Chaudhry, Grant Hall, Iliana Georgana, Myra Hosmillo, Martin D. Curran, Malte Pinckert, Surendra Parmar, Ian Goodfellow
EPI_ISL_637625, EPI_ISL_637626, EPI_ISL_637627	Wales Specialist Virology Centre Sequencing lab: Pathogen Genomics Unit	COVID-19 Genomics UK (COG-UK) Consortium	Catherine Moore, Johnathan Evans, Laura Gifford, Malorie Perry, Simon Cottrell, Angela Marchbank, Alec Birchley, Alexander Adams, Amy Gaskin, Bree Gatica-Wilcox, Jason Coombes, Joel Southgate, Lauren Gilbert, Lee Graham, Nicole Pacchiarini, Sara Kumziene-Summerhayes, Sarah Taylor, Sophie Jones, Sara Rey, Matthew Bull, Joanne Watkins, Sally Corden, Tom Connor
EPI_ISL_637628, EPI_ISL_637629, EPI_ISL_637630, EPI_ISL_637631, EPI_ISL_637632, EPI_ISL_637633, EPI_ISL_637634, EPI_ISL_637635, EPI_ISL_637636, EPI_ISL_637637, EPI_ISL_637638, EPI_ISL_637640, EPI_ISL_637641, EPI_ISL_637642, EPI_ISL_637643, EPI_ISL_637644, EPI_ISL_637645, EPI_ISL_637646, EPI_ISL_637647, EPI_ISL_637648, EPI_ISL_637649, EPI_ISL_637650, EPI_ISL_637651, EPI_ISL_637652, EPI_ISL_637653, EPI_ISL_637654, EPI_ISL_637655, EPI_ISL_637656, EPI_ISL_637657, EPI_ISL_637658			
see above	Department of Pathology, University of Cambridge	COVID-19 Genomics UK (COG-UK) Consortium	Aminu S. Jahun, Yasmin Chaudhry, Grant Hall, Iliana Georgana, Myra Hosmillo, Martin D. Curran, Malte Pinckert, Surendra Parmar, Ian Goodfellow
EPI_ISL_637659	Centre for Enzyme Innovation, University of Portsmouth / Translational Research Laboratory, Portsmouth Hospitals NHS Trust	COVID-19 Genomics UK (COG-UK) Consortium	Angela Beckett,Yann Bourgeois,Garry Scarlett,Sharon Glaysher,Scott Elliott,Kelly Bicknell,Robert Impey,Allyson Lloyd,Sarah Wyllie,Ethan Butcher,Anoop Chauhan,Samuel Robson
EPI_ISL_637660, EPI_ISL_637661	Queens Medical Centre, Clinical Microbiology Department / DeepSeq Nottingham	COVID-19 Genomics UK (COG-UK) Consortium	Gemma Clark, Wendy Smith, Manjinder Khakh, Vicki M Fleming, Michelle M Lister, Hannah Howson-Wells, Jonathan Ball, Patrick McClure, Joseph Chappell, Theocharis Tsoleridis, Nadine Holmes, Matthew Carlisle, Christopher Moore, Fei Sang, Johnny Debebe, Victoria Wright, Matthew Loose
EPI_ISL_637662, EPI_ISL_637663, EPI_ISL_637664, EPI_ISL_637665, EPI_ISL_637666	Department of Pathology, University of Cambridge	COVID-19 Genomics UK (COG-UK) Consortium	Aminu S. Jahun, Yasmin Chaudhry, Grant Hall, Iliana Georgana, Myra Hosmillo, Martin D. Curran, Malte Pinckert, Surendra Parmar, Ian Goodfellow
EPI_ISL_637667	Wales Specialist Virology Centre Sequencing lab: Pathogen Genomics Unit	COVID-19 Genomics UK (COG-UK) Consortium	Catherine Moore, Johnathan Evans, Laura Gifford, Malorie Perry, Simon Cottrell, Angela Marchbank, Alec Birchley, Alexander Adams, Amy Gaskin, Bree Gatica-Wilcox, Jason Coombes, Joel Southgate, Lauren Gilbert, Lee Graham, Nicole Pacchiarini, Sara Kumziene-Summerhayes, Sarah Taylor, Sophie Jones, Sara Rey, Matthew Bull, Joanne Watkins, Sally Corden, Tom Connor

EPI_ISL_637668, EPI_ISL_637669, EPI_ISL_637670, EPI_ISL_637671, EPI_ISL_637672, EPI_ISL_637673	Department of Pathology, University of Cambridge	COVID-19 Genomics UK (COG-UK) Consortium	Aminu S. Jahun, Yasmin Chaudhry, Grant Hall, Iliana Georgana, Myra Hosmillo, Martin D. Curran, Malte Pinckert, Surendra Parmar, Ian Goodfellow
EPI_ISL_637674	Centre for Enzyme Innovation, University of Portsmouth / Translational Research Laboratory, Portsmouth Hospitals NHS Trust	COVID-19 Genomics UK (COG-UK) Consortium	Angela Beckett,Yann Bourgeois,Garry Scarlett,Sharon Glaysher,Scott Elliott,Kelly Bicknell,Robert Impey,Allyson Lloyd,Sarah Wyllie,Ethan Butcher,Anoop Chauhan,Samuel Robson
EPI_ISL_637675	Wales Specialist Virology Centre Sequencing lab: Pathogen Genomics Unit	COVID-19 Genomics UK (COG-UK) Consortium	Catherine Moore, Johnathan Evans, Laura Gifford, Malorie Perry, Simon Cottrell, Angela Marchbank, Alec Birchley, Alexander Adams, Amy Gaskin, Bree Gatica-Wilcox, Jason Coombes, Joel Southgate, Lauren Gilbert, Lee Graham, Nicole Pacchiarini, Sara Kumziene-Summerhayes, Sarah Taylor, Sophie Jones, Sara Rey, Matthew Bull, Joanne Watkins, Sally Corden, Tom Connor
EPI_ISL_637676, EPI_ISL_637677, EPI_ISL_637678, EPI_ISL_637680, EPI_ISL_637681, EPI_ISL_637682, EPI_ISL_637683, EPI_ISL_637684, EPI_ISL_637685, EPI_ISL_637686, EPI_ISL_637687, EPI_ISL_637688, EPI_ISL_637689, EPI_ISL_637690, EPI_ISL_637691, EPI_ISL_637692, EPI_ISL_637693, EPI_ISL_637694, EPI_ISL_637695, EPI_ISL_637696, EPI_ISL_637697, EPI_ISL_637698, EPI_ISL_637699, EPI_ISL_637700, EPI_ISL_637701, EPI_ISL_637702, EPI_ISL_637703, EPI_ISL_637704, EPI_ISL_637705, EPI_ISL_637706, EPI_ISL_637707, EPI_ISL_637708, EPI_ISL_637709, EPI_ISL_637710, EPI_ISL_637711, EPI_ISL_637712, EPI_ISL_637713			
see above	Department of Pathology, University of Cambridge	COVID-19 Genomics UK (COG-UK) Consortium	Aminu S. Jahun, Yasmin Chaudhry, Grant Hall, Iliana Georgana, Myra Hosmillo, Martin D. Curran, Malte Pinckert, Surendra Parmar, Ian Goodfellow
EPI_ISL_637714, EPI_ISL_637715	Centre for Enzyme Innovation, University of Portsmouth / Translational Research Laboratory, Portsmouth Hospitals NHS Trust	COVID-19 Genomics UK (COG-UK) Consortium	Angela Beckett,Yann Bourgeois,Garry Scarlett,Sharon Glaysher,Scott Elliott,Kelly Bicknell,Robert Impey,Allyson Lloyd,Sarah Wyllie,Ethan Butcher,Anoop Chauhan,Samuel Robson
EPI_ISL_637716	Department of Pathology, University of Cambridge	COVID-19 Genomics UK (COG-UK) Consortium	Aminu S. Jahun, Yasmin Chaudhry, Grant Hall, Iliana Georgana, Myra Hosmillo, Martin D. Curran, Malte Pinckert, Surendra Parmar, Ian Goodfellow
EPI_ISL_637717, EPI_ISL_637718, EPI_ISL_637719, EPI_ISL_637720	Centre for Enzyme Innovation, University of Portsmouth / Translational Research Laboratory, Portsmouth Hospitals NHS Trust	COVID-19 Genomics UK (COG-UK) Consortium	Angela Beckett,Yann Bourgeois,Garry Scarlett,Sharon Glaysher,Scott Elliott,Kelly Bicknell,Robert Impey,Allyson Lloyd,Sarah Wyllie,Ethan Butcher,Anoop Chauhan,Samuel Robson
EPI_ISL_637721, EPI_ISL_637722, EPI_ISL_637723, EPI_ISL_637724, EPI_ISL_637725, EPI_ISL_637727, EPI_ISL_637728, EPI_ISL_637729, EPI_ISL_637730, EPI_ISL_637731, EPI_ISL_637732, EPI_ISL_637733, EPI_ISL_637734, EPI_ISL_637735, EPI_ISL_637736, EPI_ISL_637737, EPI_ISL_637738, EPI_ISL_637739, EPI_ISL_637740, EPI_ISL_637741, EPI_ISL_637742, EPI_ISL_637743, EPI_ISL_637744, EPI_ISL_637745, EPI_ISL_637746, EPI_ISL_637747, EPI_ISL_637748, EPI_ISL_637749, EPI_ISL_637750, EPI_ISL_637751, EPI_ISL_637752, EPI_ISL_637753, EPI_ISL_637754, EPI_ISL_637755, EPI_ISL_637756, EPI_ISL_637757, EPI_ISL_637758, EPI_ISL_637759, EPI_ISL_637760, EPI_ISL_637761, EPI_ISL_637762, EPI_ISL_637763, EPI_ISL_637764, EPI_ISL_637765, EPI_ISL_637766, EPI_ISL_637767, EPI_ISL_637768, EPI_ISL_637769, EPI_ISL_637770, EPI_ISL_637771, EPI_ISL_637772, EPI_ISL_637773, EPI_ISL_637774, EPI_ISL_637775, EPI_ISL_637776, EPI_ISL_637777, EPI_ISL_637778, EPI_ISL_637779			
see above	Department of Pathology, University of Cambridge	COVID-19 Genomics UK (COG-UK) Consortium	Aminu S. Jahun, Yasmin Chaudhry, Grant Hall, Iliana Georgana, Myra Hosmillo, Martin D. Curran, Malte Pinckert, Surendra Parmar, Ian Goodfellow
EPI_ISL_637780, EPI_ISL_637781, EPI_ISL_637782, EPI_ISL_637783	Centre for Enzyme Innovation, University of Portsmouth / Translational Research Laboratory, Portsmouth Hospitals NHS Trust	COVID-19 Genomics UK (COG-UK) Consortium	Angela Beckett,Yann Bourgeois,Garry Scarlett,Sharon Glaysher,Scott Elliott,Kelly Bicknell,Robert Impey,Allyson Lloyd,Sarah Wyllie,Ethan Butcher,Anoop Chauhan,Samuel Robson
EPI_ISL_637784, EPI_ISL_637785, EPI_ISL_637786, EPI_ISL_637787, EPI_ISL_637788, EPI_ISL_637789, EPI_ISL_637790, EPI_ISL_637791, EPI_ISL_637792, EPI_ISL_637793, EPI_ISL_637794, EPI_ISL_637795, EPI_ISL_637796, EPI_ISL_637797			
see above	Northumbria University / South Tees Hospitals NHS Foundation Trust / North Cumbria Integrated Care NHS Foundation Trust / North Tees and Hartlepool NHS Foundation Trust / Newcastle Hospitals NHS Foundation Trust	COVID-19 Genomics UK (COG-UK) Consortium	Darren L Smith,Andrew Nelson,Matthew Bashton,Greg R Young,Joshua Loh,John Allan,Mohammad A Tariq,Giles S Holt,Gary Black,Wen C Yew,Lynn Dover,Paul Baker,Steve Liggett,Sarah Essex,Jane Greenaway,Debra Padgett,Clive Graham,Garren Scott,Edward Barton,Emma Swindells,Brendan Payne,Jennifer Collins,Yusri Taha,Gary Eltringham
EPI_ISL_637798, EPI_ISL_637799, EPI_ISL_637800	Queens Medical Centre, Clinical Microbiology Department / DeepSeq Nottingham	COVID-19 Genomics UK (COG-UK) Consortium	Gemma Clark, Wendy Smith, Manjinder Khakh, Vicki M Fleming, Michelle M Lister, Hannah Howson-Wells, Jonathan Ball, Patrick McClure, Joseph Chappell, Theocharis Tsoleridis, Nadine Holmes, Matthew Carlisle, Christopher Moore, Fei Sang, Johnny Debebe, Victoria Wright, Matthew Loose
EPI_ISL_637801, EPI_ISL_637802, EPI_ISL_637803, EPI_ISL_637804, EPI_ISL_637805, EPI_ISL_637806, EPI_ISL_637807, EPI_ISL_637808, EPI_ISL_637809, EPI_ISL_637810, EPI_ISL_637811, EPI_ISL_637812, EPI_ISL_637813, EPI_ISL_637814, EPI_ISL_637815, EPI_ISL_637816, EPI_ISL_637817, EPI_ISL_637818, EPI_ISL_637819, EPI_ISL_637820, EPI_ISL_637821, EPI_ISL_637822, EPI_ISL_637823, EPI_ISL_637824, EPI_ISL_637825, EPI_ISL_637826, EPI_ISL_637827, EPI_ISL_637828, EPI_ISL_637829, EPI_ISL_637830, EPI_ISL_637831, EPI_ISL_637832, EPI_ISL_637833, EPI_ISL_637834, EPI_ISL_637835, EPI_ISL_637836, EPI_ISL_637837, EPI_ISL_637838, EPI_ISL_637839, EPI_ISL_637840, EPI_ISL_637841, EPI_ISL_637842, EPI_ISL_637843, EPI_ISL_637844, EPI_ISL_637845, EPI_ISL_637846, EPI_ISL_637847, EPI_ISL_637848, EPI_ISL_637849, EPI_ISL_637850, EPI_ISL_637851, EPI_ISL_637852			
see above	Department of Pathology, University of Cambridge	COVID-19 Genomics UK (COG-UK) Consortium	Aminu S. Jahun, Yasmin Chaudhry, Grant Hall, Iliana Georgana, Myra Hosmillo, Martin D. Curran, Malte Pinckert, Surendra Parmar, Ian Goodfellow
EPI_ISL_637853	Centre for Enzyme Innovation, University of Portsmouth / Translational Research Laboratory, Portsmouth Hospitals NHS Trust	COVID-19 Genomics UK (COG-UK) Consortium	Angela Beckett,Yann Bourgeois,Garry Scarlett,Sharon Glaysher,Scott Elliott,Kelly Bicknell,Robert Impey,Allyson Lloyd,Sarah Wyllie,Ethan Butcher,Anoop Chauhan,Samuel Robson
EPI_ISL_637854	Wales Specialist Virology Centre Sequencing lab: Pathogen Genomics Unit	COVID-19 Genomics UK (COG-UK) Consortium	Catherine Moore, Johnathan Evans, Laura Gifford, Malorie Perry, Simon Cottrell, Angela Marchbank, Alec Birchley, Alexander Adams, Amy Gaskin, Bree Gatica-Wilcox, Jason Coombes, Joel Southgate, Lauren Gilbert, Lee Graham, Nicole Pacchiarini, Sara Kumziene-Summerhayes, Sarah Taylor, Sophie Jones, Sara Rey, Matthew Bull, Joanne Watkins, Sally Corden, Tom Connor
EPI_ISL_637855	Oxford Viromics, NDM, University of Oxford; Oxford University Hospitals; Basingstoke and North Hampshire Hospital	COVID-19 Genomics UK (COG-UK) Consortium	Tanya Golubchik, David Bonsall, George Macintyre, Amy Trebes, Mariateresa de Cesare, Catrin Moore, Alex Mobbs, Anita Justice, Robert Shaw, Monique Andersson, Timothy Peto, Emma Wise, Nathan Moore, Jessica Lynch, Nick Cortes, Matilde Mori, Stephen Kidd, David Buck, John Todd, Christophe Fraser
EPI_ISL_637856	Department of Pathology, University of Cambridge	COVID-19 Genomics UK (COG-UK) Consortium	Aminu S. Jahun, Yasmin Chaudhry, Grant Hall, Iliana Georgana, Myra Hosmillo, Martin D. Curran, Malte Pinckert, Surendra Parmar, Ian Goodfellow
EPI_ISL_637857	Quadram Institute Bioscience	COVID-19 Genomics UK (COG-UK) Consortium	Dave J. Baker, Gemma L. Kay, Alp Aydin, Thanh Le-Viet, Steven Rudder, Ana P. Tedim, Anastasia Kolyva, Maria Diaz, Leonardo de Oliveira Martins, Nabil-Fareed Alikhan, Lizzie Meadows, Rachael Stanley, Ngozi Elumogo, Muhammed Yasir, Nicholas M. Thomson, Alexander J Trotter, Rachel Gilroy, Samuel Bloomfield, Claire Stuart, Andrew Bell, Reenesh Prakash, Samir Dervisevic, Alison E. Mather, John Wain, Mark Webber, Andrew J. Page, Justin O'Grady
EPI_ISL_637858	Oxford Viromics, NDM, University of Oxford; Oxford University Hospitals; Basingstoke and North Hampshire Hospital	COVID-19 Genomics UK (COG-UK) Consortium	Tanya Golubchik, David Bonsall, George Macintyre, Amy Trebes, Mariateresa de Cesare, Catrin Moore, Alex Mobbs, Anita Justice, Robert Shaw, Monique Andersson, Timothy Peto, Emma Wise, Nathan Moore, Jessica Lynch, Nick Cortes, Matilde Mori, Stephen Kidd, David Buck, John Todd, Christophe Fraser
EPI_ISL_637859, EPI_ISL_637860	Department of Pathology, University of Cambridge	COVID-19 Genomics UK (COG-UK) Consortium	Aminu S. Jahun, Yasmin Chaudhry, Grant Hall, Iliana Georgana, Myra Hosmillo, Martin D. Curran, Malte Pinckert, Surendra Parmar, Ian Goodfellow
EPI_ISL_637861	Oxford Viromics, NDM, University of Oxford; Oxford University Hospitals; Basingstoke and North Hampshire Hospital	COVID-19 Genomics UK (COG-UK) Consortium	Tanya Golubchik, David Bonsall, George Macintyre, Amy Trebes, Mariateresa de Cesare, Catrin Moore, Alex Mobbs, Anita Justice, Robert Shaw, Monique Andersson, Timothy Peto, Emma Wise, Nathan Moore, Jessica Lynch, Nick Cortes, Matilde Mori, Stephen Kidd, David Buck, John Todd, Christophe Fraser
EPI_ISL_637862, EPI_ISL_637863	Virology Department, Sheffield Teaching Hospitals NHS Foundation Trust/Department of Infection, Immunity and Cardiovascular Disease, The Medical School, University of Sheffield	COVID-19 Genomics UK (COG-UK) Consortium	Thushan de Silva, Matthew Parker, Nikki Smith, Adri Angyal, Rebecca Brown, Luke Green, Rachel Tucker, Paul Parsons, Danielle Groves, Katie Johnson, Laura Carrilero, Alex Keeley, Dave Partridge, Matthew Wyles, Benjamin Lindsey, Mehmet Yavuz, Mohammad Raza, Cariad Evans
EPI_ISL_637864, EPI_ISL_637865, EPI_ISL_637866	Quadram Institute Bioscience	COVID-19 Genomics UK (COG-UK) Consortium	Dave J. Baker, Gemma L. Kay, Alp Aydin, Thanh Le-Viet, Steven Rudder, Ana P. Tedim, Anastasia Kolyva, Maria Diaz, Leonardo de Oliveira Martins, Nabil-Fareed Alikhan, Lizzie Meadows, Rachael Stanley, Ngozi Elumogo, Muhammed Yasir, Nicholas M. Thomson, Alexander J Trotter, Rachel Gilroy, Samuel Bloomfield, Claire Stuart, Andrew Bell, Reenesh Prakash, Samir Dervisevic, Alison E. Mather, John Wain, Mark Webber, Andrew J. Page, Justin O'Grady
EPI_ISL_637867	Oxford Viromics, NDM, University of Oxford; Oxford University Hospitals; Basingstoke and North Hampshire Hospital	COVID-19 Genomics UK (COG-UK) Consortium	Tanya Golubchik, David Bonsall, George Macintyre, Amy Trebes, Mariateresa de Cesare, Catrin Moore, Alex Mobbs, Anita Justice, Robert Shaw, Monique Andersson, Timothy Peto, Emma Wise, Nathan Moore, Jessica Lynch, Nick Cortes, Matilde Mori, Stephen Kidd, David Buck, John Todd, Christophe Fraser
EPI_ISL_637868, EPI_ISL_637869, EPI_ISL_637870	Wales Specialist Virology Centre Sequencing lab: Pathogen Genomics Unit	COVID-19 Genomics UK (COG-UK) Consortium	Catherine Moore, Johnathan Evans, Laura Gifford, Malorie Perry, Simon Cottrell, Angela Marchbank, Alec Birchley, Alexander Adams, Amy Gaskin, Bree Gatica-Wilcox, Jason Coombes, Joel Southgate, Lauren Gilbert, Lee Graham, Nicole Pacchiarini, Sara Kumziene-Summerhayes, Sarah Taylor, Sophie Jones, Sara Rey, Matthew Bull, Joanne Watkins, Sally Corden, Tom Connor
EPI_ISL_637871, EPI_ISL_638037	Oxford Viromics, NDM, University of Oxford; Oxford University Hospitals; Basingstoke and North Hampshire Hospital	COVID-19 Genomics UK (COG-UK) Consortium	Tanya Golubchik, David Bonsall, George Macintyre, Amy Trebes, Mariateresa de Cesare, Catrin Moore, Alex Mobbs, Anita Justice, Robert Shaw, Monique Andersson, Timothy Peto, Emma Wise, Nathan Moore, Jessica Lynch, Nick Cortes, Matilde Mori, Stephen Kidd, David Buck, John Todd, Christophe Fraser
EPI_ISL_638038, EPI_ISL_638039,	Department of Pathology, University of Cambridge	COVID-19 Genomics UK (COG-UK) Consortium	Aminu S. Jahun, Yasmin Chaudhry, Grant Hall, Iliana Georgana, Myra Hosmillo, Martin D. Curran, Malte Pinckert, Surendra Parmar, Ian Goodfellow

EPI_ISL_638040			
EPI_ISL_638041, EPI_ISL_638042, EPI_ISL_638043, EPI_ISL_638044, EPI_ISL_638045, EPI_ISL_638046, EPI_ISL_638047, EPI_ISL_638048, EPI_ISL_638049	Wales Specialist Virology Centre Sequencing lab: Pathogen Genomics Unit	COVID-19 Genomics UK (COG-UK) Consortium	Catherine Moore, Johnathan Evans, Laura Gifford, Malorie Perry, Simon Cottrell, Angela Marchbank, Alec Birchley, Alexander Adams, Amy Gaskin, Bree Gatica-Wilcox, Jason Coombes, Joel Southgate, Lauren Gilbert, Lee Graham, Nicole Pacchiarini, Sara Kumziene-Summerhayes, Sarah Taylor, Sophie Jones, Sara Rey, Matthew Bull, Joanne Watkins, Sally Corden, Tom Connor
EPI_ISL_638050	Centre for Enzyme Innovation, University of Portsmouth / Translational Research Laboratory, Portsmouth Hospitals NHS Trust	COVID-19 Genomics UK (COG-UK) Consortium	Angela Beckett,Yann Bourgeois,Garry Scarlett,Sharon Glaysher,Scott Elliott,Kelly Bicknell,Robert Impey,Allyson Lloyd,Sarah Wyllie,Ethan Butcher,Anoop Chauhan,Samuel Robson
EPI_ISL_638051, EPI_ISL_638052, EPI_ISL_638053, EPI_ISL_638054, EPI_ISL_638055, EPI_ISL_638056, EPI_ISL_638057, EPI_ISL_638058, EPI_ISL_638059, EPI_ISL_638060, EPI_ISL_638061, EPI_ISL_638062, EPI_ISL_638063, EPI_ISL_638064			
see above	Wales Specialist Virology Centre Sequencing lab: Pathogen Genomics Unit	COVID-19 Genomics UK (COG-UK) Consortium	Catherine Moore, Johnathan Evans, Laura Gifford, Malorie Perry, Simon Cottrell, Angela Marchbank, Alec Birchley, Alexander Adams, Amy Gaskin, Bree Gatica-Wilcox, Jason Coombes, Joel Southgate, Lauren Gilbert, Lee Graham, Nicole Pacchiarini, Sara Kumziene-Summerhayes, Sarah Taylor, Sophie Jones, Sara Rey, Matthew Bull, Joanne Watkins, Sally Corden, Tom Connor
EPI_ISL_638065	Centre for Enzyme Innovation, University of Portsmouth / Translational Research Laboratory, Portsmouth Hospitals NHS Trust	COVID-19 Genomics UK (COG-UK) Consortium	Angela Beckett,Yann Bourgeois,Garry Scarlett,Sharon Glaysher,Scott Elliott,Kelly Bicknell,Robert Impey,Allyson Lloyd,Sarah Wyllie,Ethan Butcher,Anoop Chauhan,Samuel Robson
EPI_ISL_638066, EPI_ISL_638067, EPI_ISL_638068, EPI_ISL_638069, EPI_ISL_638070, EPI_ISL_638071, EPI_ISL_638072	Department of Pathology, University of Cambridge	COVID-19 Genomics UK (COG-UK) Consortium	Aminu S. Jahun, Yasmin Chaudhry, Grant Hall, Iliana Georgana, Myra Hosmillo, Martin D. Curran, Malte Pinckert, Surendra Parmar, Ian Goodfellow
EPI_ISL_638073, EPI_ISL_638074, EPI_ISL_638075, EPI_ISL_638076, EPI_ISL_638077, EPI_ISL_638078	Oxford Viromics, NDM, University of Oxford; Oxford University Hospitals; Basingstoke and North Hampshire Hospital	COVID-19 Genomics UK (COG-UK) Consortium	Tanya Golubchik, David Bonsall, George Macintyre, Amy Trebes, Mariateresa de Cesare, Catrin Moore, Alex Mobbs, Anita Justice, Robert Shaw, Monique Andersson, Timothy Peto, Emma Wise, Nathan Moore, Jessica Lynch, Nick Cortes, Matilde Mori, Stephen Kidd, David Buck, John Todd, Christophe Fraser
EPI_ISL_638079, EPI_ISL_638080, EPI_ISL_638081, EPI_ISL_638082, EPI_ISL_638083	Department of Pathology, University of Cambridge	COVID-19 Genomics UK (COG-UK) Consortium	Aminu S. Jahun, Yasmin Chaudhry, Grant Hall, Iliana Georgana, Myra Hosmillo, Martin D. Curran, Malte Pinckert, Surendra Parmar, Ian Goodfellow
EPI_ISL_638084, EPI_ISL_638085	Wales Specialist Virology Centre Sequencing lab: Pathogen Genomics Unit	COVID-19 Genomics UK (COG-UK) Consortium	Catherine Moore, Johnathan Evans, Laura Gifford, Malorie Perry, Simon Cottrell, Angela Marchbank, Alec Birchley, Alexander Adams, Amy Gaskin, Bree Gatica-Wilcox, Jason Coombes, Joel Southgate, Lauren Gilbert, Lee Graham, Nicole Pacchiarini, Sara Kumziene-Summerhayes, Sarah Taylor, Sophie Jones, Sara Rey, Matthew Bull, Joanne Watkins, Sally Corden, Tom Connor
EPI_ISL_638086, EPI_ISL_638087, EPI_ISL_638088, EPI_ISL_638089	Oxford Viromics, NDM, University of Oxford; Oxford University Hospitals; Basingstoke and North Hampshire Hospital	COVID-19 Genomics UK (COG-UK) Consortium	Tanya Golubchik, David Bonsall, George Macintyre, Amy Trebes, Mariateresa de Cesare, Catrin Moore, Alex Mobbs, Anita Justice, Robert Shaw, Monique Andersson, Timothy Peto, Emma Wise, Nathan Moore, Jessica Lynch, Nick Cortes, Matilde Mori, Stephen Kidd, David Buck, John Todd, Christophe Fraser
EPI_ISL_638090	Virology Department, Sheffield Teaching Hospitals NHS Foundation Trust/Department of Infection, Immunity and Cardiovascular Disease, The Medical School, University of Sheffield	COVID-19 Genomics UK (COG-UK) Consortium	Thushan de Silva, Matthew Parker, Nikki Smith, Adri Angyal, Rebecca Brown, Luke Green, Rachel Tucker, Paul Parsons, Danielle Groves, Katie Johnson, Laura Carrilero, Alex Keeley, Dave Partridge, Matthew Wyles, Benjamin Lindsey, Mehmet Yavuz, Mohammad Raza, Cariad Evans
EPI_ISL_638091, EPI_ISL_638092, EPI_ISL_638093, EPI_ISL_638094, EPI_ISL_638095, EPI_ISL_638096, EPI_ISL_638097, EPI_ISL_638098, EPI_ISL_638099, EPI_ISL_638100, EPI_ISL_638101, EPI_ISL_638102, EPI_ISL_638103, EPI_ISL_638104, EPI_ISL_638105, EPI_ISL_638106, EPI_ISL_638107, EPI_ISL_638108, EPI_ISL_638109, EPI_ISL_638110, EPI_ISL_638111, EPI_ISL_638112, EPI_ISL_638113, EPI_ISL_638114, EPI_ISL_638115, EPI_ISL_638116, EPI_ISL_638117, EPI_ISL_638118, EPI_ISL_638119, EPI_ISL_638120, EPI_ISL_638121, EPI_ISL_638122, EPI_ISL_638123, EPI_ISL_638124, EPI_ISL_638125, EPI_ISL_638126, EPI_ISL_638127, EPI_ISL_638128, EPI_ISL_638129, EPI_ISL_638130			
see above	Wales Specialist Virology Centre Sequencing lab: Pathogen Genomics Unit	COVID-19 Genomics UK (COG-UK) Consortium	Catherine Moore, Johnathan Evans, Laura Gifford, Malorie Perry, Simon Cottrell, Angela Marchbank, Alec Birchley, Alexander Adams, Amy Gaskin, Bree Gatica-Wilcox, Jason Coombes, Joel Southgate, Lauren Gilbert, Lee Graham, Nicole Pacchiarini, Sara Kumziene-Summerhayes, Sarah Taylor, Sophie Jones, Sara Rey, Matthew Bull, Joanne Watkins, Sally Corden, Tom Connor
EPI_ISL_638131, EPI_ISL_638132, EPI_ISL_638133	Department of Pathology, University of Cambridge	COVID-19 Genomics UK (COG-UK) Consortium	Aminu S. Jahun, Yasmin Chaudhry, Grant Hall, Iliana Georgana, Myra Hosmillo, Martin D. Curran, Malte Pinckert, Surendra Parmar, Ian Goodfellow
EPI_ISL_638134, EPI_ISL_638135, EPI_ISL_638136, EPI_ISL_638137	Centre for Enzyme Innovation, University of Portsmouth / Translational Research Laboratory, Portsmouth Hospitals NHS Trust	COVID-19 Genomics UK (COG-UK) Consortium	Angela Beckett,Yann Bourgeois,Garry Scarlett,Sharon Glaysher,Scott Elliott,Kelly Bicknell,Robert Impey,Allyson Lloyd,Sarah Wyllie,Ethan Butcher,Anoop Chauhan,Samuel Robson
EPI_ISL_638138, EPI_ISL_638140, EPI_ISL_638141, EPI_ISL_638142, EPI_ISL_638143	Department of Pathology, University of Cambridge	COVID-19 Genomics UK (COG-UK) Consortium	Aminu S. Jahun, Yasmin Chaudhry, Grant Hall, Iliana Georgana, Myra Hosmillo, Martin D. Curran, Malte Pinckert, Surendra Parmar, Ian Goodfellow
EPI_ISL_638144, EPI_ISL_638145	Oxford Viromics, NDM, University of Oxford; Oxford University Hospitals; Basingstoke and North Hampshire Hospital	COVID-19 Genomics UK (COG-UK) Consortium	Tanya Golubchik, David Bonsall, George Macintyre, Amy Trebes, Mariateresa de Cesare, Catrin Moore, Alex Mobbs, Anita Justice, Robert Shaw, Monique Andersson, Timothy Peto, Emma Wise, Nathan Moore, Jessica Lynch, Nick Cortes, Matilde Mori, Stephen Kidd, David Buck, John Todd, Christophe Fraser
EPI_ISL_638146	Department of Pathology, University of Cambridge	COVID-19 Genomics UK (COG-UK) Consortium	Aminu S. Jahun, Yasmin Chaudhry, Grant Hall, Iliana Georgana, Myra Hosmillo, Martin D. Curran, Malte Pinckert, Surendra Parmar, Ian Goodfellow
EPI_ISL_638147, EPI_ISL_638148, EPI_ISL_638149, EPI_ISL_638150, EPI_ISL_638151, EPI_ISL_638152, EPI_ISL_638153, EPI_ISL_638154, EPI_ISL_638155, EPI_ISL_638156, EPI_ISL_638157, EPI_ISL_638158, EPI_ISL_638159, EPI_ISL_638160, EPI_ISL_638161, EPI_ISL_638162, EPI_ISL_638163			
see above	Wales Specialist Virology Centre Sequencing lab: Pathogen Genomics Unit	COVID-19 Genomics UK (COG-UK) Consortium	Catherine Moore, Johnathan Evans, Laura Gifford, Malorie Perry, Simon Cottrell, Angela Marchbank, Alec Birchley, Alexander Adams, Amy Gaskin, Bree Gatica-Wilcox, Jason Coombes, Joel Southgate, Lauren Gilbert, Lee Graham, Nicole Pacchiarini, Sara Kumziene-Summerhayes, Sarah Taylor, Sophie Jones, Sara Rey, Matthew Bull, Joanne Watkins, Sally Corden, Tom Connor
EPI_ISL_638164	Oxford Viromics, NDM, University of Oxford; Oxford University Hospitals; Basingstoke and North Hampshire Hospital	COVID-19 Genomics UK (COG-UK) Consortium	Tanya Golubchik, David Bonsall, George Macintyre, Amy Trebes, Mariateresa de Cesare, Catrin Moore, Alex Mobbs, Anita Justice, Robert Shaw, Monique Andersson, Timothy Peto, Emma Wise, Nathan Moore, Jessica Lynch, Nick Cortes, Matilde Mori, Stephen Kidd, David Buck, John Todd, Christophe Fraser
EPI_ISL_638165, EPI_ISL_638166, EPI_ISL_638167	Department of Pathology, University of Cambridge	COVID-19 Genomics UK (COG-UK) Consortium	Aminu S. Jahun, Yasmin Chaudhry, Grant Hall, Iliana Georgana, Myra Hosmillo, Martin D. Curran, Malte Pinckert, Surendra Parmar, Ian Goodfellow
EPI_ISL_638168	Virology Department, Sheffield Teaching Hospitals NHS Foundation Trust/Department of Infection, Immunity and Cardiovascular Disease, The Medical School, University of Sheffield	COVID-19 Genomics UK (COG-UK) Consortium	Thushan de Silva, Matthew Parker, Nikki Smith, Adri Angyal, Rebecca Brown, Luke Green, Rachel Tucker, Paul Parsons, Danielle Groves, Katie Johnson, Laura Carrilero, Alex Keeley, Dave Partridge, Matthew Wyles, Benjamin Lindsey, Mehmet Yavuz, Mohammad Raza, Cariad Evans
EPI_ISL_638169	Department of Pathology, University of Cambridge	COVID-19 Genomics UK (COG-UK) Consortium	Aminu S. Jahun, Yasmin Chaudhry, Grant Hall, Iliana Georgana, Myra Hosmillo, Martin D. Curran, Malte Pinckert, Surendra Parmar, Ian Goodfellow
EPI_ISL_638170, EPI_ISL_638171	Oxford Viromics, NDM, University of Oxford; Oxford University Hospitals; Basingstoke and North Hampshire Hospital	COVID-19 Genomics UK (COG-UK) Consortium	Tanya Golubchik, David Bonsall, George Macintyre, Amy Trebes, Mariateresa de Cesare, Catrin Moore, Alex Mobbs, Anita Justice, Robert Shaw, Monique Andersson, Timothy Peto, Emma Wise, Nathan Moore, Jessica Lynch, Nick Cortes, Matilde Mori, Stephen Kidd, David Buck, John Todd, Christophe Fraser
EPI_ISL_638172	Wales Specialist Virology Centre Sequencing lab: Pathogen Genomics Unit	COVID-19 Genomics UK (COG-UK) Consortium	Catherine Moore, Johnathan Evans, Laura Gifford, Malorie Perry, Simon Cottrell, Angela Marchbank, Alec Birchley, Alexander Adams, Amy Gaskin, Bree Gatica-Wilcox, Jason Coombes, Joel Southgate, Lauren Gilbert, Lee Graham, Nicole Pacchiarini, Sara Kumziene-Summerhayes, Sarah Taylor, Sophie Jones, Sara Rey, Matthew Bull, Joanne Watkins, Sally Corden, Tom Connor
EPI_ISL_638173	Oxford Viromics, NDM, University of Oxford; Oxford University Hospitals; Basingstoke and North Hampshire Hospital	COVID-19 Genomics UK (COG-UK) Consortium	Tanya Golubchik, David Bonsall, George Macintyre, Amy Trebes, Mariateresa de Cesare, Catrin Moore, Alex Mobbs, Anita Justice, Robert Shaw, Monique Andersson, Timothy Peto, Emma Wise, Nathan Moore, Jessica Lynch, Nick Cortes, Matilde Mori, Stephen Kidd, David Buck, John Todd, Christophe Fraser
EPI_ISL_638174	Department of Pathology, University of Cambridge	COVID-19 Genomics UK (COG-UK) Consortium	Aminu S. Jahun, Yasmin Chaudhry, Grant Hall, Iliana Georgana, Myra Hosmillo, Martin D. Curran, Malte Pinckert, Surendra Parmar, Ian Goodfellow

EPI_ISL_638175, EPI_ISL_638176	Oxford Viromics, NDM, University of Oxford; Oxford University Hospitals; Basingstoke and North Hampshire Hospital	COVID-19 Genomics UK (COG-UK) Consortium	Tanya Golubchik, David Bonsall, George Macintyre, Amy Trebes, Mariateresa de Cesare, Catrin Moore, Alex Mobbs, Anita Justice, Robert Shaw, Monique Andersson, Timothy Peto, Emma Wise, Nathan Moore, Jessica Lynch, Nick Cortes, Matilde Mori, Stephen Kidd, David Buck, John Todd, Christophe Fraser
EPI_ISL_638177, EPI_ISL_638178, EPI_ISL_638179, EPI_ISL_638180, EPI_ISL_638181, EPI_ISL_638182, EPI_ISL_638183, EPI_ISL_638184, EPI_ISL_638185, EPI_ISL_638186, EPI_ISL_638187, EPI_ISL_638188, EPI_ISL_638189, EPI_ISL_638190, EPI_ISL_638191, EPI_ISL_638192, EPI_ISL_638193, EPI_ISL_638194, EPI_ISL_638195, EPI_ISL_638196, EPI_ISL_638197, EPI_ISL_638198, EPI_ISL_638199, EPI_ISL_638200, EPI_ISL_638201, EPI_ISL_638202, EPI_ISL_638203, EPI_ISL_638204, EPI_ISL_638205			
see above	Department of Pathology, University of Cambridge	COVID-19 Genomics UK (COG-UK) Consortium	Aminu S. Jahun, Yasmin Chaudhry, Grant Hall, Iliana Georgana, Myra Hosmillo, Martin D. Curran, Malte Pinckert, Surendra Parmar, Ian Goodfellow
EPI_ISL_638206	Wales Specialist Virology Centre Sequencing lab: Pathogen Genomics Unit	COVID-19 Genomics UK (COG-UK) Consortium	Catherine Moore, Johnathan Evans, Laura Gifford, Malorie Perry, Simon Cottrell, Angela Marchbank, Alec Birchley, Alexander Adams, Amy Gaskin, Bree Gatica-Wilcox, Jason Coombes, Joel Southgate, Lauren Gilbert, Lee Graham, Nicole Pacchiarini, Sara Kumziene-Summerhayes, Sarah Taylor, Sophie Jones, Sara Rey, Matthew Bull, Joanne Watkins, Sally Corden, Tom Connor
EPI_ISL_638207	Oxford Viromics, NDM, University of Oxford; Oxford University Hospitals; Basingstoke and North Hampshire Hospital	COVID-19 Genomics UK (COG-UK) Consortium	Tanya Golubchik, David Bonsall, George Macintyre, Amy Trebes, Mariateresa de Cesare, Catrin Moore, Alex Mobbs, Anita Justice, Robert Shaw, Monique Andersson, Timothy Peto, Emma Wise, Nathan Moore, Jessica Lynch, Nick Cortes, Matilde Mori, Stephen Kidd, David Buck, John Todd, Christophe Fraser
EPI_ISL_638208, EPI_ISL_638209, EPI_ISL_638210, EPI_ISL_638211, EPI_ISL_638212, EPI_ISL_638213, EPI_ISL_638214, EPI_ISL_638215, EPI_ISL_638216, EPI_ISL_638217, EPI_ISL_638218, EPI_ISL_638219, EPI_ISL_638220, EPI_ISL_638221, EPI_ISL_638222, EPI_ISL_638223, EPI_ISL_638224, EPI_ISL_638225, EPI_ISL_638226, EPI_ISL_638227, EPI_ISL_638228, EPI_ISL_638229, EPI_ISL_638230, EPI_ISL_638231, EPI_ISL_638232, EPI_ISL_638233, EPI_ISL_638234, EPI_ISL_638235, EPI_ISL_638236, EPI_ISL_638237, EPI_ISL_638238, EPI_ISL_638239, EPI_ISL_638240, EPI_ISL_638241, EPI_ISL_638242, EPI_ISL_638243, EPI_ISL_638244, EPI_ISL_638245, EPI_ISL_638246, EPI_ISL_638247, EPI_ISL_638248			
see above	Department of Pathology, University of Cambridge	COVID-19 Genomics UK (COG-UK) Consortium	Aminu S. Jahun, Yasmin Chaudhry, Grant Hall, Iliana Georgana, Myra Hosmillo, Martin D. Curran, Malte Pinckert, Surendra Parmar, Ian Goodfellow
EPI_ISL_638249, EPI_ISL_638250, EPI_ISL_638251, EPI_ISL_638252, EPI_ISL_638253, EPI_ISL_638254, EPI_ISL_638255, EPI_ISL_638256, EPI_ISL_638257	Centre for Enzyme Innovation, University of Portsmouth / Translational Research Laboratory, Portsmouth Hospitals NHS Trust	COVID-19 Genomics UK (COG-UK) Consortium	Angela Beckett,Yann Bourgeois,Garry Scarlett,Sharon Glaysher,Scott Elliott,Kelly Bicknell,Robert Impey,Allyson Lloyd,Sarah Wyllie,Ethan Butcher,Anoop Chauhan,Samuel Robson
EPI_ISL_638258, EPI_ISL_638259, EPI_ISL_638260, EPI_ISL_638261, EPI_ISL_638262, EPI_ISL_638263, EPI_ISL_638264	Department of Pathology, University of Cambridge	COVID-19 Genomics UK (COG-UK) Consortium	Aminu S. Jahun, Yasmin Chaudhry, Grant Hall, Iliana Georgana, Myra Hosmillo, Martin D. Curran, Malte Pinckert, Surendra Parmar, Ian Goodfellow
EPI_ISL_638265, EPI_ISL_638266, EPI_ISL_638267, EPI_ISL_638268, EPI_ISL_638269, EPI_ISL_638270, EPI_ISL_638271, EPI_ISL_638272, EPI_ISL_638273, EPI_ISL_638274, EPI_ISL_638275, EPI_ISL_638276, EPI_ISL_638277, EPI_ISL_638278, EPI_ISL_638279, EPI_ISL_638280, EPI_ISL_638281, EPI_ISL_638282, EPI_ISL_638283, EPI_ISL_638284, EPI_ISL_638285, EPI_ISL_638286, EPI_ISL_638287, EPI_ISL_638288, EPI_ISL_638289, EPI_ISL_638290, EPI_ISL_638291, EPI_ISL_638292			
see above	Centre for Enzyme Innovation, University of Portsmouth / Translational Research Laboratory, Portsmouth Hospitals NHS Trust	COVID-19 Genomics UK (COG-UK) Consortium	Angela Beckett,Yann Bourgeois,Garry Scarlett,Sharon Glaysher,Scott Elliott,Kelly Bicknell,Robert Impey,Allyson Lloyd,Sarah Wyllie,Ethan Butcher,Anoop Chauhan,Samuel Robson
EPI_ISL_638293, EPI_ISL_638294, EPI_ISL_638295, EPI_ISL_638296, EPI_ISL_638297, EPI_ISL_638298, EPI_ISL_638299, EPI_ISL_638302, EPI_ISL_638303, EPI_ISL_638304, EPI_ISL_638305, EPI_ISL_638306, EPI_ISL_638307, EPI_ISL_638308, EPI_ISL_638309, EPI_ISL_638310, EPI_ISL_638311, EPI_ISL_638312, EPI_ISL_638313, EPI_ISL_638314, EPI_ISL_638316, EPI_ISL_638317, EPI_ISL_638318, EPI_ISL_638319, EPI_ISL_638320, EPI_ISL_638321, EPI_ISL_638322, EPI_ISL_638323, EPI_ISL_638324, EPI_ISL_638325, EPI_ISL_638327, EPI_ISL_638328, EPI_ISL_638329, EPI_ISL_638330, EPI_ISL_638332, EPI_ISL_638333, EPI_ISL_638334, EPI_ISL_638335, EPI_ISL_638337, EPI_ISL_638338, EPI_ISL_638339, EPI_ISL_638340, EPI_ISL_638341, EPI_ISL_638342, EPI_ISL_638343, EPI_ISL_638344, EPI_ISL_638345, EPI_ISL_638346, EPI_ISL_638347, EPI_ISL_638348, EPI_ISL_638349, EPI_ISL_638350, EPI_ISL_638351, EPI_ISL_638352, EPI_ISL_638353, EPI_ISL_638354, EPI_ISL_638355, EPI_ISL_638356, EPI_ISL_638357, EPI_ISL_638358, EPI_ISL_638359, EPI_ISL_638360, EPI_ISL_638361, EPI_ISL_638362, EPI_ISL_638363, EPI_ISL_638364, EPI_ISL_638365, EPI_ISL_638366, EPI_ISL_638367, EPI_ISL_638368, EPI_ISL_638369, EPI_ISL_638370, EPI_ISL_638371, EPI_ISL_638372, EPI_ISL_638373, EPI_ISL_638374, EPI_ISL_638375, EPI_ISL_638376, EPI_ISL_638377, EPI_ISL_638378, EPI_ISL_638379, EPI_ISL_638380, EPI_ISL_638381, EPI_ISL_638382, EPI_ISL_638383, EPI_ISL_638384, EPI_ISL_638385, EPI_ISL_638386, EPI_ISL_638387, EPI_ISL_638388, EPI_ISL_638389, EPI_ISL_638390, EPI_ISL_638391, EPI_ISL_638392, EPI_ISL_638393, EPI_ISL_638394, EPI_ISL_638395, EPI_ISL_638396, EPI_ISL_638397, EPI_ISL_638398, EPI_ISL_638399, EPI_ISL_638400, EPI_ISL_638401, EPI_ISL_638402, EPI_ISL_638403, EPI_ISL_638404, EPI_ISL_638405, EPI_ISL_638406, EPI_ISL_638407, EPI_ISL_638408, EPI_ISL_638409, EPI_ISL_638410, EPI_ISL_638411, EPI_ISL_638412, EPI_ISL_638413, EPI_ISL_638414, EPI_ISL_638415, EPI_ISL_638416, EPI_ISL_638417, EPI_ISL_638418, EPI_ISL_638419, EPI_ISL_638420, EPI_ISL_638421, EPI_ISL_638422, EPI_ISL_638423, EPI_ISL_638424, EPI_ISL_638425, EPI_ISL_638426, EPI_ISL_638427, EPI_ISL_638428, EPI_ISL_638429, EPI_ISL_638430, EPI_ISL_638431, EPI_ISL_638432, EPI_ISL_638433, EPI_ISL_638434, EPI_ISL_638435, EPI_ISL_638436, EPI_ISL_638437, EPI_ISL_638438, EPI_ISL_638439, EPI_ISL_638440, EPI_ISL_638441, EPI_ISL_638442, EPI_ISL_638443, EPI_ISL_638444, EPI_ISL_638445, EPI_ISL_638446, EPI_ISL_638447, EPI_ISL_638448			
see above	Department of Pathology, University of Cambridge	COVID-19 Genomics UK (COG-UK) Consortium	Aminu S. Jahun, Yasmin Chaudhry, Grant Hall, Iliana Georgana, Myra Hosmillo, Martin D. Curran, Malte Pinckert, Surendra Parmar, Ian Goodfellow
EPI_ISL_638449, EPI_ISL_638450, EPI_ISL_638451, EPI_ISL_638452, EPI_ISL_638453	Liverpool Clinical Laboratories	COVID-19 Genomics UK (COG-UK) Consortium	Sam Haldenby, Anita Lucaci, Steve Paterson, Julian Hiscox, Alistair Darby, M Almsaud, A Alrezaihi, Muhannad Alruwaili, Stuart D Armstrong, Jones Benjamin, Eleanor G Bentley, Anu Chawla, Jordan J Clark, Angela Cowell, Richard Eccles, Isabel García-Dorival, Matthew Gemmell, Alessandro Gerada, PKF Gilmore, Richard Gregory, Ximeng Han, Catherine Hartley, Margaret Hughes, Miren Iturriza-Gomara, James Johnson, L Luu, Jenifer Manson, Charlotte Nelson, Elaine O'Toole, Cassie Olateju, Rebekah Penrice-Randal , Lucille Rainbow, N.P Randle, Trevor Ian Robinson, Parul Sharma, Ghada T Shawli, James P Stewart, Neil Swainston, Ecaterina Vamos, Joanne Watts, Mark Whitehead
EPI_ISL_638454, EPI_ISL_638455, EPI_ISL_638456, EPI_ISL_638457, EPI_ISL_638458	Centre for Enzyme Innovation, University of Portsmouth / Translational Research Laboratory, Portsmouth Hospitals NHS Trust	COVID-19 Genomics UK (COG-UK) Consortium	Angela Beckett,Yann Bourgeois,Garry Scarlett,Sharon Glaysher,Scott Elliott,Kelly Bicknell,Robert Impey,Allyson Lloyd,Sarah Wyllie,Ethan Butcher,Anoop Chauhan,Samuel Robson
EPI_ISL_638459, EPI_ISL_638460, EPI_ISL_638461, EPI_ISL_638462, EPI_ISL_638463, EPI_ISL_638464, EPI_ISL_638465, EPI_ISL_638466, EPI_ISL_638467, EPI_ISL_638468, EPI_ISL_638469, EPI_ISL_638470, EPI_ISL_638471, EPI_ISL_638472, EPI_ISL_638473, EPI_ISL_638474, EPI_ISL_638475, EPI_ISL_638476, EPI_ISL_638477, EPI_ISL_638478, EPI_ISL_638479, EPI_ISL_638480, EPI_ISL_638481, EPI_ISL_638482, EPI_ISL_638483, EPI_ISL_638484, EPI_ISL_638485, EPI_ISL_638486, EPI_ISL_638487, EPI_ISL_638488, EPI_ISL_638489, EPI_ISL_638490, EPI_ISL_638491, EPI_ISL_638492, EPI_ISL_638493, EPI_ISL_638494, EPI_ISL_638495, EPI_ISL_638496, EPI_ISL_638497, EPI_ISL_638498, EPI_ISL_638499, EPI_ISL_638500, EPI_ISL_638501, EPI_ISL_638502, EPI_ISL_638503, EPI_ISL_638504, EPI_ISL_638505, EPI_ISL_638506, EPI_ISL_638507, EPI_ISL_638508, EPI_ISL_638509, EPI_ISL_638510, EPI_ISL_638511, EPI_ISL_638512, EPI_ISL_638513, EPI_ISL_638514, EPI_ISL_638515, EPI_ISL_638516, EPI_ISL_638517, EPI_ISL_638518, EPI_ISL_638519, EPI_ISL_638520, EPI_ISL_638521, EPI_ISL_638522, EPI_ISL_638523, EPI_ISL_638524, EPI_ISL_638525, EPI_ISL_638526, EPI_ISL_638527, EPI_ISL_638528, EPI_ISL_638529, EPI_ISL_638530, EPI_ISL_638531			
see above	Oxford Viromics, NDM, University of Oxford; Oxford University Hospitals; Basingstoke and North Hampshire Hospital	COVID-19 Genomics UK (COG-UK) Consortium	Tanya Golubchik, David Bonsall, George Macintyre, Amy Trebes, Mariateresa de Cesare, Catrin Moore, Alex Mobbs, Anita Justice, Robert Shaw, Monique Andersson, Timothy Peto, Emma Wise, Nathan Moore, Jessica Lynch, Nick Cortes, Matilde Mori, Stephen Kidd, David Buck, John Todd, Christophe Fraser
EPI_ISL_638532, EPI_ISL_638533, EPI_ISL_638534, EPI_ISL_638535, EPI_ISL_638536, EPI_ISL_638537, EPI_ISL_638538, EPI_ISL_638539, EPI_ISL_638540, EPI_ISL_638541, EPI_ISL_638542, EPI_ISL_638543, EPI_ISL_638544, EPI_ISL_638545, EPI_ISL_638546, EPI_ISL_638547, EPI_ISL_638548, EPI_ISL_638549, EPI_ISL_638550, EPI_ISL_638551, EPI_ISL_638552, EPI_ISL_638553, EPI_ISL_638554			
see above	Virology Department, Sheffield Teaching Hospitals NHS Foundation Trust/Department of Infection, Immunity and Cardiovascular Disease, The Medical School, University of Sheffield	COVID-19 Genomics UK (COG-UK) Consortium	Thushan de Silva, Matthew Parker, Nikki Smith, Adri Angyal, Rebecca Brown, Luke Green, Rachel Tucker, Paul Parsons, Danielle Groves, Katie Johnson, Laura Carrilero, Alex Keeley, Dave Partridge, Matthew Wyles, Benjamin Lindsey, Mehmet Yavuz, Mohammad Raza, Cariad Evans
EPI_ISL_638555, EPI_ISL_638556, EPI_ISL_638557, EPI_ISL_638558, EPI_ISL_638559, EPI_ISL_638560, EPI_ISL_638561, EPI_ISL_638562, EPI_ISL_638563, EPI_ISL_638564, EPI_ISL_638565, EPI_ISL_638566, EPI_ISL_638567, EPI_ISL_638568, EPI_ISL_638569, EPI_ISL_638570, EPI_ISL_638571, EPI_ISL_638572, EPI_ISL_638573, EPI_ISL_638574, EPI_ISL_638575, EPI_ISL_638576, EPI_ISL_638577, EPI_ISL_638578, EPI_ISL_638579, EPI_ISL_638580			
see above	Northumbria University / South Tees Hospitals NHS Foundation Trust / North Cumbria Integrated Care NHS Foundation Trust / North Tees and Hartlepool NHS Foundation Trust / Newcastle Hospitals NHS Foundation Trust	COVID-19 Genomics UK (COG-UK) Consortium	Darren L Smith,Andrew Nelson,Matthew Bashton,Greg R Young,Joshua Loh,John Allan,Mohammad A Tariq,Giles S Holt,Gary Black,Wen C Yew,Lynn Dover,Paul Baker,Steve Liggett,Sarah Essex,Jane Greenaway,Debra Padgett,Clive Graham,Garren Scott,Edward Barton,Emma Swindells,Brendan Payne,Jennifer Collins,Yusri Taha,Gary Eltringham
EPI_ISL_638581, EPI_ISL_638582, EPI_ISL_638583	Quadram Institute Bioscience	COVID-19 Genomics UK (COG-UK) Consortium	Dave J. Baker, Gemma L. Kay, Alp Aydin, Thanh Le-Viet, Steven Rudder, Ana P. Tedim, Anastasia Kolyva, Maria Diaz, Leonardo de Oliveira Martins, Nabil-Fareed Alikhan, Lizzie Meadows, Rachael Stanley, Ngozi Elumogo, Muhammed Yasir, Nicholas M. Thomson, Alexander J Trotter, Rachel Gilroy, Samuel Bloomfield, Claire Stuart, Andrew Bell, Reenesh Prakash, Samir Dervisevic, Alison E. Mather, John Wain, Mark Webber, Andrew J. Page, Justin O'Grady
EPI_ISL_638584, EPI_ISL_638585, EPI_ISL_638586, EPI_ISL_638587, EPI_ISL_638588, EPI_ISL_638589, EPI_ISL_638590, EPI_ISL_638591, EPI_ISL_638592, EPI_ISL_638593, EPI_ISL_638594, EPI_ISL_638595, EPI_ISL_638596, EPI_ISL_638597, EPI_ISL_638598, EPI_ISL_638599, EPI_ISL_638600, EPI_ISL_638601, EPI_ISL_638602, EPI_ISL_638603, EPI_ISL_638604, EPI_ISL_638605, EPI_ISL_638606, EPI_ISL_638607, EPI_ISL_638608, EPI_ISL_638609, EPI_ISL_638610, EPI_ISL_638611, EPI_ISL_638612, EPI_ISL_638613			
see above	Queens Medical Centre, Clinical Microbiology Department / DeepSeq Nottingham	COVID-19 Genomics UK (COG-UK) Consortium	Gemma Clark, Wendy Smith, Manjinder Khakh, Vicki M Fleming, Michelle M Lister, Hannah Howson-Wells, Jonathan Ball, Patrick McClure, Joseph Chappell, Theocharis Tsoleridis, Nadine Holmes, Matthew Carlisle, Christopher Moore, Fei Sang, Johnny Debebe, Victoria Wright, Matthew Loose
EPI_ISL_638614, EPI_ISL_638615, EPI_ISL_638616, EPI_ISL_638617, EPI_ISL_638618, EPI_ISL_638619, EPI_ISL_638620, EPI_ISL_638621, EPI_ISL_638622, EPI_ISL_638623, EPI_ISL_638624, EPI_ISL_638625, EPI_ISL_638626, EPI_ISL_638627, EPI_ISL_638628, EPI_ISL_638629, EPI_ISL_638630, EPI_ISL_638631, EPI_ISL_638632, EPI_ISL_638633, EPI_ISL_638634, EPI_ISL_638635, EPI_ISL_638636, EPI_ISL_638637, EPI_ISL_638638, EPI_ISL_638639, EPI_ISL_638640, EPI_ISL_638641, EPI_ISL_638642, EPI_ISL_638643, EPI_ISL_638644, EPI_ISL_638645, EPI_ISL_638646, EPI_ISL_638647, EPI_ISL_638648, EPI_ISL_638649,			

EPI_ISL_638650, EPI_ISL_638651, EPI_ISL_638652, EPI_ISL_638653, EPI_ISL_638654, EPI_ISL_638655, EPI_ISL_638656, EPI_ISL_638657, EPI_ISL_638658, EPI_ISL_638659, EPI_ISL_638660, EPI_ISL_638661, EPI_ISL_638662, EPI_ISL_638663, EPI_ISL_638664, EPI_ISL_638665, EPI_ISL_638666, EPI_ISL_638667, EPI_ISL_638668, EPI_ISL_638669, EPI_ISL_638670, EPI_ISL_638671, EPI_ISL_638672, EPI_ISL_638673, EPI_ISL_638674, EPI_ISL_638675, EPI_ISL_638676, EPI_ISL_638677, EPI_ISL_638678, EPI_ISL_638679, EPI_ISL_638680, EPI_ISL_638681			
see above	Department of Pathology, University of Cambridge	COVID-19 Genomics UK (COG-UK) Consortium	Aminu S. Jahun, Yasmin Chaudhry, Grant Hall, Iliana Georgana, Myra Hosmillo, Martin D. Curran, Malte Pinckert, Surendra Parmar, Ian Goodfellow
EPI_ISL_638682, EPI_ISL_638683, EPI_ISL_638684, EPI_ISL_638685, EPI_ISL_638686, EPI_ISL_638687, EPI_ISL_638688, EPI_ISL_638689, EPI_ISL_638690, EPI_ISL_638691, EPI_ISL_638692, EPI_ISL_638693, EPI_ISL_638694, EPI_ISL_638695, EPI_ISL_638696, EPI_ISL_638697, EPI_ISL_638698, EPI_ISL_638699, EPI_ISL_638700, EPI_ISL_638701, EPI_ISL_638702, EPI_ISL_638703, EPI_ISL_638704, EPI_ISL_638705, EPI_ISL_638706, EPI_ISL_638707, EPI_ISL_638708, EPI_ISL_638709, EPI_ISL_638710, EPI_ISL_638711, EPI_ISL_638712, EPI_ISL_638713, EPI_ISL_638714, EPI_ISL_638715, EPI_ISL_638716, EPI_ISL_638717, EPI_ISL_638718, EPI_ISL_638719, EPI_ISL_638720, EPI_ISL_638721, EPI_ISL_638722, EPI_ISL_638723, EPI_ISL_638724, EPI_ISL_638725, EPI_ISL_638726, EPI_ISL_638727, EPI_ISL_638728, EPI_ISL_638729, EPI_ISL_638730			
see above	Wales Specialist Virology Centre Sequencing lab: Pathogen Genomics Unit	COVID-19 Genomics UK (COG-UK) Consortium	Catherine Moore, Johnathan Evans, Laura Gifford, Malorie Perry, Simon Cottrell, Angela Marchbank, Alec Birchley, Alexander Adams, Amy Gaskin, Bree Gatica-Wilcox, Jason Coombes, Joel Southgate, Lauren Gilbert, Lee Graham, Nicole Pacchiarini, Sara Kumziene-Summerhayes, Sarah Taylor, Sophie Jones, Sara Rey, Matthew Bull, Joanne Watkins, Sally Corden, Tom Connor
EPI_ISL_638731, EPI_ISL_638732, EPI_ISL_638733, EPI_ISL_638734, EPI_ISL_638735	Department of Pathology, University of Cambridge	COVID-19 Genomics UK (COG-UK) Consortium	Aminu S. Jahun, Yasmin Chaudhry, Grant Hall, Iliana Georgana, Myra Hosmillo, Martin D. Curran, Malte Pinckert, Surendra Parmar, Ian Goodfellow
EPI_ISL_638736, EPI_ISL_638737, EPI_ISL_638738, EPI_ISL_638739, EPI_ISL_638740, EPI_ISL_638741, EPI_ISL_638742, EPI_ISL_638743, EPI_ISL_638744, EPI_ISL_638745, EPI_ISL_638746, EPI_ISL_638747, EPI_ISL_638748, EPI_ISL_638749, EPI_ISL_638750, EPI_ISL_638751, EPI_ISL_638752, EPI_ISL_638753, EPI_ISL_638754, EPI_ISL_638755, EPI_ISL_638756, EPI_ISL_638757, EPI_ISL_638758, EPI_ISL_638759, EPI_ISL_638760, EPI_ISL_638761, EPI_ISL_638762, EPI_ISL_638763, EPI_ISL_638764, EPI_ISL_638765, EPI_ISL_638766, EPI_ISL_638767, EPI_ISL_638768, EPI_ISL_638769, EPI_ISL_638770, EPI_ISL_638771, EPI_ISL_638772, EPI_ISL_638773, EPI_ISL_638774, EPI_ISL_638775, EPI_ISL_638776, EPI_ISL_638777, EPI_ISL_638778, EPI_ISL_638779, EPI_ISL_638780, EPI_ISL_638781, EPI_ISL_638782, EPI_ISL_638783, EPI_ISL_638784, EPI_ISL_638785, EPI_ISL_638786, EPI_ISL_638787, EPI_ISL_638788, EPI_ISL_638789, EPI_ISL_638790, EPI_ISL_638791, EPI_ISL_638792, EPI_ISL_638793, EPI_ISL_638794, EPI_ISL_638795, EPI_ISL_638796, EPI_ISL_638797, EPI_ISL_638798, EPI_ISL_638799, EPI_ISL_638800, EPI_ISL_638801, EPI_ISL_638802, EPI_ISL_638803, EPI_ISL_638804, EPI_ISL_638805, EPI_ISL_638806, EPI_ISL_638807, EPI_ISL_638808, EPI_ISL_638809, EPI_ISL_638810, EPI_ISL_638811, EPI_ISL_638812, EPI_ISL_638813, EPI_ISL_638814, EPI_ISL_638815, EPI_ISL_638816, EPI_ISL_638817, EPI_ISL_638818, EPI_ISL_638819, EPI_ISL_638820, EPI_ISL_638821, EPI_ISL_638822, EPI_ISL_638823, EPI_ISL_638824, EPI_ISL_638825, EPI_ISL_638826, EPI_ISL_638827, EPI_ISL_638828, EPI_ISL_638829, EPI_ISL_638830, EPI_ISL_638831, EPI_ISL_638832, EPI_ISL_638833, EPI_ISL_638834, EPI_ISL_638835, EPI_ISL_638836, EPI_ISL_638837, EPI_ISL_638838, EPI_ISL_638839, EPI_ISL_638840, EPI_ISL_638841, EPI_ISL_638842, EPI_ISL_638843, EPI_ISL_638844, EPI_ISL_638845, EPI_ISL_638846, EPI_ISL_638847, EPI_ISL_638848, EPI_ISL_638849, EPI_ISL_638850, EPI_ISL_638851, EPI_ISL_638852, EPI_ISL_638853, EPI_ISL_638854, EPI_ISL_638855, EPI_ISL_638856, EPI_ISL_638857, EPI_ISL_638858, EPI_ISL_638859, EPI_ISL_638860, EPI_ISL_638861, EPI_ISL_638862, EPI_ISL_638863, EPI_ISL_638864, EPI_ISL_638865, EPI_ISL_638866, EPI_ISL_638867, EPI_ISL_638868, EPI_ISL_638869, EPI_ISL_638870, EPI_ISL_638871, EPI_ISL_638872, EPI_ISL_638873, EPI_ISL_638874, EPI_ISL_638875, EPI_ISL_638876			
see above	Oxford Viromics, NDM, University of Oxford; Oxford University Hospitals; Basingstoke and North Hampshire Hospital	COVID-19 Genomics UK (COG-UK) Consortium	Tanya Golubchik, David Bonsall, George Macintyre, Amy Trebes, Mariateresa de Cesare, Catrin Moore, Alex Mobbs, Anita Justice, Robert Shaw, Monique Andersson, Timothy Peto, Emma Wise, Nathan Moore, Jessica Lynch, Nick Cortes, Matilde Mori, Stephen Kidd, David Buck, John Todd, Christophe Fraser
EPI_ISL_638877, EPI_ISL_638878, EPI_ISL_638879, EPI_ISL_638880, EPI_ISL_638881, EPI_ISL_638882, EPI_ISL_638883, EPI_ISL_638884, EPI_ISL_638885, EPI_ISL_638886, EPI_ISL_638887, EPI_ISL_638888, EPI_ISL_638889, EPI_ISL_638890, EPI_ISL_638891, EPI_ISL_638892, EPI_ISL_638893			
see above	Virology Department, Sheffield Teaching Hospitals NHS Foundation Trust/Department of Infection, Immunity and Cardiovascular Disease, The Medical School, University of Sheffield	COVID-19 Genomics UK (COG-UK) Consortium	Thushan de Silva, Matthew Parker, Nikki Smith, Adri Angyal, Rebecca Brown, Luke Green, Rachel Tucker, Paul Parsons, Danielle Groves, Katie Johnson, Laura Carrilero, Alex Keeley, Dave Partridge, Matthew Wyles, Benjamin Lindsey, Mehmet Yavuz, Mohammad Raza, Cariad Evans
EPI_ISL_638894	Oxford Viromics, NDM, University of Oxford; Oxford University Hospitals; Basingstoke and North Hampshire Hospital	COVID-19 Genomics UK (COG-UK) Consortium	Tanya Golubchik, David Bonsall, George Macintyre, Amy Trebes, Mariateresa de Cesare, Catrin Moore, Alex Mobbs, Anita Justice, Robert Shaw, Monique Andersson, Timothy Peto, Emma Wise, Nathan Moore, Jessica Lynch, Nick Cortes, Matilde Mori, Stephen Kidd, David Buck, John Todd, Christophe Fraser
EPI_ISL_638895, EPI_ISL_638896, EPI_ISL_638897, EPI_ISL_638898, EPI_ISL_638899, EPI_ISL_638900, EPI_ISL_638901, EPI_ISL_638902, EPI_ISL_638903, EPI_ISL_638904, EPI_ISL_638905, EPI_ISL_638906, EPI_ISL_638907, EPI_ISL_638908, EPI_ISL_638909, EPI_ISL_638910			
see above	Department of Pathology, University of Cambridge	COVID-19 Genomics UK (COG-UK) Consortium	Aminu S. Jahun, Yasmin Chaudhry, Grant Hall, Iliana Georgana, Myra Hosmillo, Martin D. Curran, Malte Pinckert, Surendra Parmar, Ian Goodfellow
EPI_ISL_638911, EPI_ISL_638912, EPI_ISL_638913	Centre for Enzyme Innovation, University of Portsmouth / Translational Research Laboratory, Portsmouth Hospitals NHS Trust	COVID-19 Genomics UK (COG-UK) Consortium	Angela Beckett,Yann Bourgeois,Garry Scarlett,Sharon Glaysher,Scott Elliott,Kelly Bicknell,Robert Impey,Allyson Lloyd,Sarah Wyllie,Ethan Butcher,Anoop Chauhan,Samuel Robson
EPI_ISL_638914, EPI_ISL_638915, EPI_ISL_638916, EPI_ISL_638917, EPI_ISL_638918, EPI_ISL_638919, EPI_ISL_638920, EPI_ISL_638921, EPI_ISL_638922, EPI_ISL_638923, EPI_ISL_638924, EPI_ISL_638925, EPI_ISL_638926, EPI_ISL_638927			
see above	Department of Pathology, University of Cambridge	COVID-19 Genomics UK (COG-UK) Consortium	Aminu S. Jahun, Yasmin Chaudhry, Grant Hall, Iliana Georgana, Myra Hosmillo, Martin D. Curran, Malte Pinckert, Surendra Parmar, Ian Goodfellow
EPI_ISL_638928, EPI_ISL_638929, EPI_ISL_638930, EPI_ISL_638931, EPI_ISL_638932, EPI_ISL_638933, EPI_ISL_638934, EPI_ISL_638935	Wales Specialist Virology Centre Sequencing lab: Pathogen Genomics Unit	COVID-19 Genomics UK (COG-UK) Consortium	Catherine Moore, Johnathan Evans, Laura Gifford, Malorie Perry, Simon Cottrell, Angela Marchbank, Alec Birchley, Alexander Adams, Amy Gaskin, Bree Gatica-Wilcox, Jason Coombes, Joel Southgate, Lauren Gilbert, Lee Graham, Nicole Pacchiarini, Sara Kumziene-Summerhayes, Sarah Taylor, Sophie Jones, Sara Rey, Matthew Bull, Joanne Watkins, Sally Corden, Tom Connor
EPI_ISL_638936, EPI_ISL_638937, EPI_ISL_638938, EPI_ISL_638939, EPI_ISL_638940, EPI_ISL_638941, EPI_ISL_638942, EPI_ISL_638943	Department of Pathology, University of Cambridge	COVID-19 Genomics UK (COG-UK) Consortium	Aminu S. Jahun, Yasmin Chaudhry, Grant Hall, Iliana Georgana, Myra Hosmillo, Martin D. Curran, Malte Pinckert, Surendra Parmar, Ian Goodfellow
EPI_ISL_638944, EPI_ISL_638945, EPI_ISL_638946, EPI_ISL_638947, EPI_ISL_638948, EPI_ISL_638949, EPI_ISL_638950, EPI_ISL_638951, EPI_ISL_638952, EPI_ISL_638953, EPI_ISL_638954, EPI_ISL_638955, EPI_ISL_638956, EPI_ISL_638957, EPI_ISL_638958, EPI_ISL_638959, EPI_ISL_638960, EPI_ISL_638961, EPI_ISL_638962, EPI_ISL_638963, EPI_ISL_638964, EPI_ISL_638965, EPI_ISL_638966, EPI_ISL_638967, EPI_ISL_638968, EPI_ISL_638969, EPI_ISL_638970, EPI_ISL_638971, EPI_ISL_638972, EPI_ISL_638973, EPI_ISL_638974, EPI_ISL_638975, EPI_ISL_638976, EPI_ISL_638977, EPI_ISL_638978, EPI_ISL_638979, EPI_ISL_638980, EPI_ISL_638981, EPI_ISL_638982, EPI_ISL_638983, EPI_ISL_638984, EPI_ISL_638985, EPI_ISL_638986, EPI_ISL_638987, EPI_ISL_638988, EPI_ISL_638989, EPI_ISL_638990, EPI_ISL_638991, EPI_ISL_638992, EPI_ISL_638993, EPI_ISL_638994, EPI_ISL_638995, EPI_ISL_638996, EPI_ISL_638997, EPI_ISL_638998, EPI_ISL_638999, EPI_ISL_639000, EPI_ISL_639001			
see above	Oxford Viromics, NDM, University of Oxford; Oxford University Hospitals; Basingstoke and North Hampshire Hospital	COVID-19 Genomics UK (COG-UK) Consortium	Tanya Golubchik, David Bonsall, George Macintyre, Amy Trebes, Mariateresa de Cesare, Catrin Moore, Alex Mobbs, Anita Justice, Robert Shaw, Monique Andersson, Timothy Peto, Emma Wise, Nathan Moore, Jessica Lynch, Nick Cortes, Matilde Mori, Stephen Kidd, David Buck, John Todd, Christophe Fraser
EPI_ISL_639002	Liverpool Clinical Laboratories	COVID-19 Genomics UK (COG-UK) Consortium	Sam Haldenby, Anita Lucaci, Steve Paterson, Julian Hiscox, Alistair Darby, M Almsaud, A Alrezaihi, Muhannad Alruwaili, Stuart D Armstrong, Jones Benjamin, Eleanor G Bentley, Anu Chawla, Jordan J Clark, Angela Cowell, Richard Eccles, Isabel García-Dorival, Matthew Gemmell, Alessandro Gerada, PKF Gilmore, Richard Gregory, Ximeng Han, Catherine Hartley, Margaret Hughes, Miren Iturriza-Gomara, James Johnson, L Luu, Jenifer Manson, Charlotte Nelson, Elaine O'Toole, Cassie Olateju, Rebekah Penrice-Randal , Lucille Rainbow, N.P Randle, Trevor Ian Robinson, Parul Sharma, Ghada T Shawli, James P Stewart, Neil Swainston, Ecaterina Vamos, Joanne Watts, Mark Whitehead
EPI_ISL_639003, EPI_ISL_639004, EPI_ISL_639005	Department of Pathology, University of Cambridge	COVID-19 Genomics UK (COG-UK) Consortium	Aminu S. Jahun, Yasmin Chaudhry, Grant Hall, Iliana Georgana, Myra Hosmillo, Martin D. Curran, Malte Pinckert, Surendra Parmar, Ian Goodfellow
EPI_ISL_639006, EPI_ISL_639007	Centre for Enzyme Innovation, University of Portsmouth / Translational Research Laboratory, Portsmouth Hospitals NHS Trust	COVID-19 Genomics UK (COG-UK) Consortium	Angela Beckett,Yann Bourgeois,Garry Scarlett,Sharon Glaysher,Scott Elliott,Kelly Bicknell,Robert Impey,Allyson Lloyd,Sarah Wyllie,Ethan Butcher,Anoop Chauhan,Samuel Robson
EPI_ISL_639008, EPI_ISL_639009, EPI_ISL_639010	Virology Department, Sheffield Teaching Hospitals NHS Foundation Trust/Department of Infection, Immunity and Cardiovascular Disease, The Medical School, University of Sheffield	COVID-19 Genomics UK (COG-UK) Consortium	Thushan de Silva, Matthew Parker, Nikki Smith, Adri Angyal, Rebecca Brown, Luke Green, Rachel Tucker, Paul Parsons, Danielle Groves, Katie Johnson, Laura Carrilero, Alex Keeley, Dave Partridge, Matthew Wyles, Benjamin Lindsey, Mehmet Yavuz, Mohammad Raza, Cariad Evans
EPI_ISL_639011, EPI_ISL_639012, EPI_ISL_639013, EPI_ISL_639014, EPI_ISL_639015, EPI_ISL_639016, EPI_ISL_639017, EPI_ISL_639018, EPI_ISL_639019, EPI_ISL_639020, EPI_ISL_639021, EPI_ISL_639022, EPI_ISL_639023, EPI_ISL_639024, EPI_ISL_639025, EPI_ISL_639026, EPI_ISL_639027, EPI_ISL_639028, EPI_ISL_639029, EPI_ISL_639030, EPI_ISL_639031, EPI_ISL_639032, EPI_ISL_639033, EPI_ISL_639034, EPI_ISL_639035, EPI_ISL_639036, EPI_ISL_639037, EPI_ISL_639038, EPI_ISL_639039, EPI_ISL_639040, EPI_ISL_639041, EPI_ISL_639042, EPI_ISL_639043, EPI_ISL_639044, EPI_ISL_639045, EPI_ISL_639046, EPI_ISL_639047, EPI_ISL_639048, EPI_ISL_639049, EPI_ISL_639050, EPI_ISL_639051, EPI_ISL_639052, EPI_ISL_639053, EPI_ISL_639054, EPI_ISL_639055, EPI_ISL_639056, EPI_ISL_639057, EPI_ISL_639058, EPI_ISL_639059, EPI_ISL_639060, EPI_ISL_639061, EPI_ISL_639062, EPI_ISL_639063, EPI_ISL_639064, EPI_ISL_639065, EPI_ISL_639066, EPI_ISL_639067, EPI_ISL_639068, EPI_ISL_639069, EPI_ISL_639070, EPI_ISL_639071, EPI_ISL_639072, EPI_ISL_639073, EPI_ISL_639074, EPI_ISL_639075, EPI_ISL_639076, EPI_ISL_639077, EPI_ISL_639078, EPI_ISL_639079, EPI_ISL_639080, EPI_ISL_639081, EPI_ISL_639082, EPI_ISL_639083, EPI_ISL_639084, EPI_ISL_639085, EPI_ISL_639086, EPI_ISL_639087, EPI_ISL_639088, EPI_ISL_639089, EPI_ISL_639090, EPI_ISL_639091, EPI_ISL_639092, EPI_ISL_639093, EPI_ISL_639094, EPI_ISL_639095, EPI_ISL_639096, EPI_ISL_639097, EPI_ISL_639098, EPI_ISL_639099, EPI_ISL_639100, EPI_ISL_639101, EPI_ISL_639102, EPI_ISL_639103, EPI_ISL_639104, EPI_ISL_639105, EPI_ISL_639106, EPI_ISL_639107, EPI_ISL_639108, EPI_ISL_639109, EPI_ISL_639110, EPI_ISL_639111, EPI_ISL_639112, EPI_ISL_639113, EPI_ISL_639114, EPI_ISL_639115, EPI_ISL_639116, EPI_ISL_639117, EPI_ISL_639118, EPI_ISL_639119, EPI_ISL_639120, EPI_ISL_639121, EPI_ISL_639122, EPI_ISL_639123, EPI_ISL_639124, EPI_ISL_639125, EPI_ISL_639126, EPI_ISL_639127, EPI_ISL_639128, EPI_ISL_639129, EPI_ISL_639130, EPI_ISL_639131, EPI_ISL_639132, EPI_ISL_639133, EPI_ISL_639134, EPI_ISL_639135, EPI_ISL_639136, EPI_ISL_639137, EPI_ISL_639138, EPI_ISL_639139, EPI_ISL_639140, EPI_ISL_639141, EPI_ISL_639142, EPI_ISL_639143, EPI_ISL_639144, EPI_ISL_639145, EPI_ISL_639146, EPI_ISL_639147, EPI_ISL_639148, EPI_ISL_639149, EPI_ISL_639150, EPI_ISL_639151, EPI_ISL_639152, EPI_ISL_639153, EPI_ISL_639154, EPI_ISL_639155, EPI_ISL_639156, EPI_ISL_639157, EPI_ISL_639158, EPI_ISL_639159, EPI_ISL_639160, EPI_ISL_639161, EPI_ISL_639162, EPI_ISL_639163, EPI_ISL_639164, EPI_ISL_639165, EPI_ISL_639166, EPI_ISL_639167, EPI_ISL_639168, EPI_ISL_639169, EPI_ISL_639170, EPI_ISL_639171, EPI_ISL_639172, EPI_ISL_639173, EPI_ISL_639174, EPI_ISL_639175, EPI_ISL_639176, EPI_ISL_639177, EPI_ISL_639178, EPI_ISL_639179, EPI_ISL_639180, EPI_ISL_639181, EPI_ISL_639182, EPI_ISL_639183, EPI_ISL_639184, EPI_ISL_639185, EPI_ISL_639186, EPI_ISL_639187, EPI_ISL_639188, EPI_ISL_639189, EPI_ISL_639190, EPI_ISL_639191, EPI_ISL_639192, EPI_ISL_639193, EPI_ISL_639194, EPI_ISL_639195, EPI_ISL_639196, EPI_ISL_639197, EPI_ISL_639198, EPI_ISL_639199, EPI_ISL_639200, EPI_ISL_639201, EPI_ISL_639202, EPI_ISL_639203, EPI_ISL_639204, EPI_ISL_639205, EPI_ISL_639206, EPI_ISL_639207, EPI_ISL_639208, EPI_ISL_639209, EPI_ISL_639210, EPI_ISL_639211, EPI_ISL_639212, EPI_ISL_639213, EPI_ISL_639214, EPI_ISL_639215, EPI_ISL_639216, EPI_ISL_639217, EPI_ISL_639218, EPI_ISL_639219, EPI_ISL_639220, EPI_ISL_639221, EPI_ISL_639222, EPI_ISL_639223, EPI_ISL_639224, EPI_ISL_639225, EPI_ISL_639226, EPI_ISL_639227,			

EPI_ISL_639228, EPI_ISL_639229, EPI_ISL_639230, EPI_ISL_639231, EPI_ISL_639232, EPI_ISL_639233, EPI_ISL_639234, EPI_ISL_639235, EPI_ISL_639236, EPI_ISL_639237, EPI_ISL_639238, EPI_ISL_639239, EPI_ISL_639240, EPI_ISL_639241, EPI_ISL_639242, EPI_ISL_639243, EPI_ISL_639244, EPI_ISL_639245, EPI_ISL_639246, EPI_ISL_639247, EPI_ISL_639248, EPI_ISL_639249, EPI_ISL_639250, EPI_ISL_639251, EPI_ISL_639252, EPI_ISL_639253, EPI_ISL_639254, EPI_ISL_639255, EPI_ISL_639256, EPI_ISL_639257, EPI_ISL_639258, EPI_ISL_639259, EPI_ISL_639260, EPI_ISL_639261, EPI_ISL_639262, EPI_ISL_639263, EPI_ISL_639264, EPI_ISL_639265, EPI_ISL_639266, EPI_ISL_639267, EPI_ISL_639268, EPI_ISL_639269, EPI_ISL_639270, EPI_ISL_639271, EPI_ISL_639272, EPI_ISL_639273, EPI_ISL_639274, EPI_ISL_639275, EPI_ISL_639276, EPI_ISL_639277, EPI_ISL_639278, EPI_ISL_639279, EPI_ISL_639280, EPI_ISL_639281, EPI_ISL_639282, EPI_ISL_639283, EPI_ISL_639284, EPI_ISL_639285, EPI_ISL_639286, EPI_ISL_639287, EPI_ISL_639288, EPI_ISL_639289, EPI_ISL_639290, EPI_ISL_639291, EPI_ISL_639292, EPI_ISL_639293, EPI_ISL_639294, EPI_ISL_639295, EPI_ISL_639296, EPI_ISL_639297, EPI_ISL_639298, EPI_ISL_639299, EPI_ISL_639300, EPI_ISL_639301, EPI_ISL_639302, EPI_ISL_639303, EPI_ISL_639304, EPI_ISL_639305, EPI_ISL_639306, EPI_ISL_639307, EPI_ISL_639308, EPI_ISL_639309, EPI_ISL_639310, EPI_ISL_639311, EPI_ISL_639312, EPI_ISL_639313, EPI_ISL_639314, EPI_ISL_639315, EPI_ISL_639316, EPI_ISL_639317, EPI_ISL_639318, EPI_ISL_639319, EPI_ISL_639320, EPI_ISL_639321, EPI_ISL_639322, EPI_ISL_639323, EPI_ISL_639324, EPI_ISL_639325, EPI_ISL_639326, EPI_ISL_639327, EPI_ISL_639328, EPI_ISL_639329, EPI_ISL_639330, EPI_ISL_639331, EPI_ISL_639332, EPI_ISL_639333, EPI_ISL_639334, EPI_ISL_639335, EPI_ISL_639336, EPI_ISL_639337, EPI_ISL_639338, EPI_ISL_639339, EPI_ISL_639340, EPI_ISL_639341, EPI_ISL_639342, EPI_ISL_639343, EPI_ISL_639344, EPI_ISL_639345, EPI_ISL_639346, EPI_ISL_639347, EPI_ISL_639348, EPI_ISL_639349, EPI_ISL_639350, EPI_ISL_639351, EPI_ISL_639352, EPI_ISL_639353, EPI_ISL_639354, EPI_ISL_639355, EPI_ISL_639356, EPI_ISL_639357, EPI_ISL_639358, EPI_ISL_639359, EPI_ISL_639360, EPI_ISL_639361, EPI_ISL_639362, EPI_ISL_639363, EPI_ISL_639364, EPI_ISL_639365, EPI_ISL_639366, EPI_ISL_639367, EPI_ISL_639368, EPI_ISL_639369, EPI_ISL_639370, EPI_ISL_639371, EPI_ISL_639372, EPI_ISL_639373, EPI_ISL_639374, EPI_ISL_639375, EPI_ISL_639376, EPI_ISL_639377, EPI_ISL_639378, EPI_ISL_639379, EPI_ISL_639380, EPI_ISL_639381, EPI_ISL_639382, EPI_ISL_639383, EPI_ISL_639384, EPI_ISL_639385, EPI_ISL_639386, EPI_ISL_639387, EPI_ISL_639388, EPI_ISL_639389, EPI_ISL_639390, EPI_ISL_639391, EPI_ISL_639392, EPI_ISL_639393, EPI_ISL_639394, EPI_ISL_639395, EPI_ISL_639396, EPI_ISL_639397, EPI_ISL_639398, EPI_ISL_639399, EPI_ISL_639400, EPI_ISL_639401, EPI_ISL_639402, EPI_ISL_639403, EPI_ISL_639404, EPI_ISL_639405, EPI_ISL_639406, EPI_ISL_639407, EPI_ISL_639408, EPI_ISL_639409, EPI_ISL_639410, EPI_ISL_639411, EPI_ISL_639412, EPI_ISL_639413, EPI_ISL_639414, EPI_ISL_639415, EPI_ISL_639416, EPI_ISL_639417, EPI_ISL_639418, EPI_ISL_639419, EPI_ISL_639420, EPI_ISL_639421, EPI_ISL_639422, EPI_ISL_639423, EPI_ISL_639424, EPI_ISL_639425, EPI_ISL_639426, EPI_ISL_639427, EPI_ISL_639428, EPI_ISL_639429, EPI_ISL_639430, EPI_ISL_639431, EPI_ISL_639432, EPI_ISL_639433, EPI_ISL_639434, EPI_ISL_639435, EPI_ISL_639436, EPI_ISL_639437, EPI_ISL_639438, EPI_ISL_639439, EPI_ISL_639440, EPI_ISL_639441, EPI_ISL_639442, EPI_ISL_639443, EPI_ISL_639444, EPI_ISL_639445, EPI_ISL_639446, EPI_ISL_639447, EPI_ISL_639448, EPI_ISL_639449, EPI_ISL_639450, EPI_ISL_639451, EPI_ISL_639452, EPI_ISL_639453, EPI_ISL_639454, EPI_ISL_639455, EPI_ISL_639456, EPI_ISL_639457, EPI_ISL_639458, EPI_ISL_639459, EPI_ISL_639460, EPI_ISL_639461, EPI_ISL_639462, EPI_ISL_639463, EPI_ISL_639464, EPI_ISL_639465, EPI_ISL_639466, EPI_ISL_639467, EPI_ISL_639468, EPI_ISL_639469, EPI_ISL_639470, EPI_ISL_639471, EPI_ISL_639472, EPI_ISL_639473, EPI_ISL_639474, EPI_ISL_639475, EPI_ISL_639476, EPI_ISL_639477, EPI_ISL_639478, EPI_ISL_639479, EPI_ISL_639480, EPI_ISL_639481, EPI_ISL_639482, EPI_ISL_639483, EPI_ISL_639484, EPI_ISL_639485, EPI_ISL_639486, EPI_ISL_639487, EPI_ISL_639488, EPI_ISL_639489, EPI_ISL_639490, EPI_ISL_639491, EPI_ISL_639492, EPI_ISL_639493, EPI_ISL_639494, EPI_ISL_639495, EPI_ISL_639496, EPI_ISL_639497, EPI_ISL_639498, EPI_ISL_639499, EPI_ISL_639500, EPI_ISL_639501, EPI_ISL_639502, EPI_ISL_639503, EPI_ISL_639504, EPI_ISL_639505, EPI_ISL_639506, EPI_ISL_639507, EPI_ISL_639508, EPI_ISL_639509, EPI_ISL_639510, EPI_ISL_639511, EPI_ISL_639512, EPI_ISL_639513, EPI_ISL_639514, EPI_ISL_639515, EPI_ISL_639516, EPI_ISL_639517, EPI_ISL_639518, EPI_ISL_639519, EPI_ISL_639520, EPI_ISL_639521, EPI_ISL_639522, EPI_ISL_639523, EPI_ISL_639524, EPI_ISL_639525, EPI_ISL_639526, EPI_ISL_639527, EPI_ISL_639528, EPI_ISL_639529, EPI_ISL_639530, EPI_ISL_639531, EPI_ISL_639532, EPI_ISL_639533, EPI_ISL_639534, EPI_ISL_639535, EPI_ISL_639536, EPI_ISL_639537, EPI_ISL_639538, EPI_ISL_639539, EPI_ISL_639540, EPI_ISL_639541, EPI_ISL_639542, EPI_ISL_639543, EPI_ISL_639544, EPI_ISL_639545, EPI_ISL_639546, EPI_ISL_639547, EPI_ISL_639548, EPI_ISL_639549, EPI_ISL_639550, EPI_ISL_639551, EPI_ISL_639552, EPI_ISL_639553, EPI_ISL_639554, EPI_ISL_639555, EPI_ISL_639556, EPI_ISL_639557, EPI_ISL_639558, EPI_ISL_639559, EPI_ISL_639560, EPI_ISL_639561, EPI_ISL_639562, EPI_ISL_639563, EPI_ISL_639564, EPI_ISL_639565, EPI_ISL_639566, EPI_ISL_639567, EPI_ISL_639568, EPI_ISL_639569, EPI_ISL_639570, EPI_ISL_639571, EPI_ISL_639572, EPI_ISL_639573, EPI_ISL_639574, EPI_ISL_639575, EPI_ISL_639576, EPI_ISL_639577, EPI_ISL_639578, EPI_ISL_639579, EPI_ISL_639580, EPI_ISL_639581, EPI_ISL_639582, EPI_ISL_639583, EPI_ISL_639584, EPI_ISL_639585, EPI_ISL_639586, EPI_ISL_639587, EPI_ISL_639588, EPI_ISL_639589, EPI_ISL_639590, EPI_ISL_639591, EPI_ISL_639592, EPI_ISL_639593, EPI_ISL_639594, EPI_ISL_639595, EPI_ISL_639596, EPI_ISL_639597, EPI_ISL_639598, EPI_ISL_639599, EPI_ISL_639600, EPI_ISL_639601, EPI_ISL_639602, EPI_ISL_639603, EPI_ISL_639604, EPI_ISL_639605, EPI_ISL_639606, EPI_ISL_639607, EPI_ISL_639608, EPI_ISL_639609, EPI_ISL_639610, EPI_ISL_639611, EPI_ISL_639612, EPI_ISL_639613, EPI_ISL_639614, EPI_ISL_639615, EPI_ISL_639616, EPI_ISL_639617, EPI_ISL_639618, EPI_ISL_639619, EPI_ISL_639620, EPI_ISL_639621, EPI_ISL_639622, EPI_ISL_639623			
see above	Wales Specialist Virology Centre Sequencing lab: Pathogen Genomics Unit	COVID-19 Genomics UK (COG-UK) Consortium	Catherine Moore, Johnathan Evans, Laura Gifford, Malorie Perry, Simon Cottrell, Angela Marchbank, Alec Birchley, Alexander Adams, Amy Gaskin, Bree Gatica-Wilcox, Jason Coombes, Joel Southgate, Lauren Gilbert, Lee Graham, Nicole Pacchiarini, Sara Kumziene-Summerhayes, Sarah Taylor, Sophie Jones, Sara Rey, Matthew Bull, Joanne Watkins, Sally Corden, Tom Connor
EPI_ISL_639624, EPI_ISL_639625	Department of Pathology, University of Cambridge	COVID-19 Genomics UK (COG-UK) Consortium	Aminu S. Jahun, Yasmin Chaudhry, Grant Hall, Iliana Georgana, Myra Hosmillo, Martin D. Curran, Malte Pinckert, Surendra Parmar, Ian Goodfellow
EPI_ISL_639626	Wales Specialist Virology Centre Sequencing lab: Pathogen Genomics Unit	COVID-19 Genomics UK (COG-UK) Consortium	Catherine Moore, Johnathan Evans, Laura Gifford, Malorie Perry, Simon Cottrell, Angela Marchbank, Alec Birchley, Alexander Adams, Amy Gaskin, Bree Gatica-Wilcox, Jason Coombes, Joel Southgate, Lauren Gilbert, Lee Graham, Nicole Pacchiarini, Sara Kumziene-Summerhayes, Sarah Taylor, Sophie Jones, Sara Rey, Matthew Bull, Joanne Watkins, Sally Corden, Tom Connor
EPI_ISL_639627, EPI_ISL_639628, EPI_ISL_639629, EPI_ISL_639630	Oxford Viromics, NDM, University of Oxford; Oxford University Hospitals; Basingstoke and North Hampshire Hospital	COVID-19 Genomics UK (COG-UK) Consortium	Tanya Golubchik, David Bonsall, George Macintyre, Amy Trebes, Mariateresa de Cesare, Catrin Moore, Alex Mobbs, Anita Justice, Robert Shaw, Monique Andersson, Timothy Peto, Emma Wise, Nathan Moore, Jessica Lynch, Nick Cortes, Matilde Mori, Stephen Kidd, David Buck, John Todd, Christophe Fraser
EPI_ISL_639631	E. Gulbja Laboratorija	Latvian Biomedical Research and Study Centre	Ivars Silamielis, Kaspars Megnis, Monta Ustinova, ikita Zrelovs, Vita Rovte, Mikus Gavars, Dmitrijs Perminovs, Uga Dumpis, Jnis Kloviš
EPI_ISL_639632, EPI_ISL_639633	Centrl Laboratorija	Latvian Biomedical Research and Study Centre	Ivars Silamielis, Kaspars Megnis, Monta Ustinova, ikita Zrelovs, Vita Rovte, Stella Lapia, Jana Oste, Marta Priedte, Uga Dumpis, Jnis Kloviš
EPI_ISL_639634	Latvijas Infektoloijas centrs	Latvian Biomedical Research and Study Centre	Ivars Silamielis, Kaspars Megnis, Monta Ustinova, ikita Zrelovs, Vita Rovte, Jeena Storoženko, Tatjana Kolupajeva, Oksana Savicka, Uga Dumpis, Jnis Kloviš
EPI_ISL_639635	E. Gulbja Laboratorija	Latvian Biomedical Research and Study Centre	Ivars Silamielis, Kaspars Megnis, Monta Ustinova, ikita Zrelovs, Vita Rovte, Mikus Gavars, Dmitrijs Perminovs, Uga Dumpis, Jnis Kloviš
EPI_ISL_639636	Centrl Laboratorija	Latvian Biomedical Research and Study Centre	Ivars Silamielis, Kaspars Megnis, Monta Ustinova, ikita Zrelovs, Vita Rovte, Stella Lapia, Jana Oste, Marta Priedte, Uga Dumpis, Jnis Kloviš
EPI_ISL_639637, EPI_ISL_639638	E. Gulbja Laboratorija	Latvian Biomedical Research and Study Centre	Ivars Silamielis, Kaspars Megnis, Monta Ustinova, ikita Zrelovs, Vita Rovte, Mikus Gavars, Dmitrijs Perminovs, Uga Dumpis, Jnis Kloviš
EPI_ISL_639639	Latvijas Infektoloijas centrs	Latvian Biomedical Research and Study Centre	Ivars Silamielis, Kaspars Megnis, Monta Ustinova, ikita Zrelovs, Vita Rovte, Jeena Storoženko, Tatjana Kolupajeva, Oksana Savicka, Uga Dumpis, Jnis Kloviš
EPI_ISL_639640	Centrl Laboratorija	Latvian Biomedical Research and Study Centre	Ivars Silamielis, Kaspars Megnis, Monta Ustinova, ikita Zrelovs, Vita Rovte, Stella Lapia, Jana Oste, Marta Priedte, Uga Dumpis, Jnis Kloviš
EPI_ISL_639641	E. Gulbja Laboratorija	Latvian Biomedical Research and Study Centre	Ivars Silamielis, Kaspars Megnis, Monta Ustinova, ikita Zrelovs, Vita Rovte, Mikus Gavars, Dmitrijs Perminovs, Uga Dumpis, Jnis Kloviš
EPI_ISL_639642	Centrl Laboratorija	Latvian Biomedical Research and Study Centre	Ivars Silamielis, Kaspars Megnis, Monta Ustinova, ikita Zrelovs, Vita Rovte, Stella Lapia, Jana Oste, Marta Priedte, Uga Dumpis, Jnis Kloviš
EPI_ISL_639643, EPI_ISL_639644, EPI_ISL_639645, EPI_ISL_639646, EPI_ISL_639647	E. Gulbja Laboratorija	Latvian Biomedical Research and Study Centre	Ivars Silamielis, Kaspars Megnis, Monta Ustinova, ikita Zrelovs, Vita Rovte, Mikus Gavars, Dmitrijs Perminovs, Uga Dumpis, Jnis Kloviš
EPI_ISL_639648	Centrl Laboratorija	Latvian Biomedical Research and Study Centre	Ivars Silamielis, Kaspars Megnis, Monta Ustinova, ikita Zrelovs, Vita Rovte, Stella Lapia, Jana Oste, Marta Priedte, Uga Dumpis, Jnis Kloviš
EPI_ISL_639649	E. Gulbja Laboratorija	Latvian Biomedical Research and Study Centre	Ivars Silamielis, Kaspars Megnis, Monta Ustinova, ikita Zrelovs, Vita Rovte, Mikus Gavars, Dmitrijs Perminovs, Uga Dumpis, Jnis Kloviš
EPI_ISL_639650, EPI_ISL_639651	Centrl Laboratorija	Latvian Biomedical Research and Study Centre	Ivars Silamielis, Kaspars Megnis, Monta Ustinova, ikita Zrelovs, Vita Rovte, Stella Lapia, Jana Oste, Marta Priedte, Uga Dumpis, Jnis Kloviš
EPI_ISL_639652, EPI_ISL_639653	E. Gulbja Laboratorija	Latvian Biomedical Research and Study Centre	Ivars Silamielis, Kaspars Megnis, Monta Ustinova, ikita Zrelovs, Vita Rovte, Mikus Gavars, Dmitrijs Perminovs, Uga Dumpis, Jnis Kloviš
EPI_ISL_639654, EPI_ISL_639655, EPI_ISL_639656, EPI_ISL_639657	Centrl Laboratorija	Latvian Biomedical Research and Study Centre	Ivars Silamielis, Kaspars Megnis, Monta Ustinova, ikita Zrelovs, Vita Rovte, Stella Lapia, Jana Oste, Marta Priedte, Uga Dumpis, Jnis Kloviš
EPI_ISL_639658, EPI_ISL_639659, EPI_ISL_639660, EPI_ISL_639661, EPI_ISL_639662	E. Gulbja Laboratorija	Latvian Biomedical Research and Study Centre	Ivars Silamielis, Kaspars Megnis, Monta Ustinova, ikita Zrelovs, Vita Rovte, Mikus Gavars, Dmitrijs Perminovs, Uga Dumpis, Jnis Kloviš
EPI_ISL_639663	Centrl Laboratorija	Latvian Biomedical Research and Study Centre	Ivars Silamielis, Kaspars Megnis, Monta Ustinova, ikita Zrelovs, Vita Rovte, Stella Lapia, Jana Oste, Marta Priedte, Uga Dumpis, Jnis Kloviš
EPI_ISL_639664, EPI_ISL_639665, EPI_ISL_639666, EPI_ISL_639667, EPI_ISL_639668, EPI_ISL_639669, EPI_ISL_639670, EPI_ISL_639671	E. Gulbja Laboratorija	Latvian Biomedical Research and Study Centre	Ivars Silamielis, Kaspars Megnis, Monta Ustinova, ikita Zrelovs, Vita Rovte, Mikus Gavars, Dmitrijs Perminovs, Uga Dumpis, Jnis Kloviš
EPI_ISL_639672	Centrl Laboratorija	Latvian Biomedical Research and Study Centre	Ivars Silamielis, Kaspars Megnis, Monta Ustinova, ikita Zrelovs, Vita Rovte, Stella Lapia, Jana Oste, Marta Priedte, Uga Dumpis, Jnis Kloviš
EPI_ISL_639673, EPI_ISL_639674, EPI_ISL_639675	E. Gulbja Laboratorija	Latvian Biomedical Research and Study Centre	Ivars Silamielis, Kaspars Megnis, Monta Ustinova, ikita Zrelovs, Vita Rovte, Mikus Gavars, Dmitrijs Perminovs, Uga Dumpis, Jnis Kloviš
EPI_ISL_639676, EPI_ISL_639677, EPI_ISL_639678, EPI_ISL_639679, EPI_ISL_639680	Centrl Laboratorija	Latvian Biomedical Research and Study Centre	Ivars Silamielis, Kaspars Megnis, Monta Ustinova, ikita Zrelovs, Vita Rovte, Stella Lapia, Jana Oste, Marta Priedte, Uga Dumpis, Jnis Kloviš
EPI_ISL_639681, EPI_ISL_639682	E. Gulbja Laboratorija	Latvian Biomedical Research and Study Centre	Ivars Silamielis, Kaspars Megnis, Monta Ustinova, ikita Zrelovs, Vita Rovte, Mikus Gavars, Dmitrijs Perminovs, Uga Dumpis, Jnis Kloviš
EPI_ISL_639683	Centrl Laboratorija	Latvian Biomedical Research and Study Centre	Ivars Silamielis, Kaspars Megnis, Monta Ustinova, ikita Zrelovs, Vita Rovte, Stella Lapia, Jana Oste, Marta Priedte, Uga Dumpis, Jnis Kloviš
EPI_ISL_639684, EPI_ISL_639685, EPI_ISL_639686, EPI_ISL_639687, EPI_ISL_639688, EPI_ISL_639689, EPI_ISL_639690, EPI_ISL_639691, EPI_ISL_639692, EPI_ISL_639693, EPI_ISL_639694			
see above	E. Gulbja Laboratorija	Latvian Biomedical Research and Study Centre	Ivars Silamielis, Kaspars Megnis, Monta Ustinova, ikita Zrelovs, Vita Rovte, Mikus Gavars, Dmitrijs Perminovs, Uga Dumpis, Jnis Kloviš
EPI_ISL_639729, EPI_ISL_639730	Sydney South West Pathology Service (SSWPS) - Liverpool Hospital - NSW Health Pathology	NSW Health Pathology - Institute of Clinical Pathology and Medical Research; Westmead Hospital; University of Sydney	CIDM-PH et al.

EPI_ISL_639731	Laverty Pathology	NSW Health Pathology - Institute of Clinical Pathology and Medical Research; Westmead Hospital; University of Sydney	CIDM-PH et al.
EPI_ISL_639732	Sydney South West Pathology Service (SSWPS) - Liverpool Hospital - NSW Health Pathology	NSW Health Pathology - Institute of Clinical Pathology and Medical Research; Westmead Hospital; University of Sydney	CIDM-PH et al.
EPI_ISL_639733	Laverty Pathology	NSW Health Pathology - Institute of Clinical Pathology and Medical Research; Westmead Hospital; University of Sydney	CIDM-PH et al.
EPI_ISL_639734, EPI_ISL_639735	Sydney South West Pathology Service (SSWPS) - Liverpool Hospital - NSW Health Pathology	NSW Health Pathology - Institute of Clinical Pathology and Medical Research; Westmead Hospital; University of Sydney	CIDM-PH et al.
EPI_ISL_639736, EPI_ISL_639737, EPI_ISL_639738	South Eastern Area Laboratory Services (SEALS)	NSW Health Pathology - Institute of Clinical Pathology and Medical Research; Westmead Hospital; University of Sydney	CIDM-PH et al.
EPI_ISL_639739	Centre of Nanotechnologies, INCD IMT-Bucuresti (National Institute for Research and Development in Microtechnologies - Bucharest)	Centre of Nanotechnologies, INCD IMT-Bucuresti (National Institute for Research and Development in Microtechnologies - Bucharest)	Salceanu,A., Gogianu,L. and Baisan,M.
EPI_ISL_639750, EPI_ISL_639818, EPI_ISL_639819	unknown	Public Health Virology Laboratory, Forensic and Scientific Services (PHV-FSS)	Son Nguyen et al.
EPI_ISL_639821, EPI_ISL_639822	Respiratory Virus Unit, Microbiology Services Colindale, Public Health England	COVID-19 Genomics UK (COG-UK) Consortium	PHE Covid Sequencing Team
EPI_ISL_639825, EPI_ISL_639826, EPI_ISL_639827, EPI_ISL_639828, EPI_ISL_639829, EPI_ISL_639830, EPI_ISL_639831, EPI_ISL_639833, EPI_ISL_639834, EPI_ISL_639835, EPI_ISL_639836, EPI_ISL_639837, EPI_ISL_639838, EPI_ISL_639839, EPI_ISL_639840, EPI_ISL_639841, EPI_ISL_639842, EPI_ISL_639843, EPI_ISL_639844, EPI_ISL_639845, EPI_ISL_639846, EPI_ISL_639847, EPI_ISL_639848, EPI_ISL_639849, EPI_ISL_639850, EPI_ISL_639851, EPI_ISL_639852, EPI_ISL_639854, EPI_ISL_639855, EPI_ISL_639856, EPI_ISL_639857, EPI_ISL_639858, EPI_ISL_639859, EPI_ISL_639860, EPI_ISL_639861, EPI_ISL_639862, EPI_ISL_639863, EPI_ISL_639864, EPI_ISL_639865, EPI_ISL_639866, EPI_ISL_639867, EPI_ISL_639868, EPI_ISL_639869, EPI_ISL_639870, EPI_ISL_639871, EPI_ISL_639872, EPI_ISL_639873, EPI_ISL_639874, EPI_ISL_639875, EPI_ISL_639876, EPI_ISL_639877, EPI_ISL_639878, EPI_ISL_639879, EPI_ISL_639880, EPI_ISL_639881, EPI_ISL_639882, EPI_ISL_639883, EPI_ISL_639884, EPI_ISL_639885, EPI_ISL_639886, EPI_ISL_639887, EPI_ISL_639888, EPI_ISL_639889, EPI_ISL_639890, EPI_ISL_639891, EPI_ISL_639892, EPI_ISL_639893, EPI_ISL_639894, EPI_ISL_639895, EPI_ISL_639896, EPI_ISL_639897, EPI_ISL_639898, EPI_ISL_639899, EPI_ISL_639900, EPI_ISL_639901, EPI_ISL_639902, EPI_ISL_639903, EPI_ISL_639904, EPI_ISL_639905, EPI_ISL_639906, EPI_ISL_639907, EPI_ISL_639908			
see above	National Virus Reference Laboratory	National Virus Reference Laboratory	Michael Carr, Gabriel Gonzalez, Jonathan Dean, Daniel Hare, Cillian F De Gascun
EPI_ISL_639912, EPI_ISL_639913, EPI_ISL_639914, EPI_ISL_639915, EPI_ISL_639916, EPI_ISL_639917, EPI_ISL_639918, EPI_ISL_639919, EPI_ISL_639920, EPI_ISL_639921, EPI_ISL_639922, EPI_ISL_639924, EPI_ISL_639925, EPI_ISL_639926, EPI_ISL_639930, EPI_ISL_639932, EPI_ISL_639934, EPI_ISL_639941, EPI_ISL_639942, EPI_ISL_639943, EPI_ISL_639944, EPI_ISL_639945, EPI_ISL_639947			
see above	Omsk Research Institute of Natural Focal Infections	WHO National Influenza Centre Russian Federation	Artem Fadeev, Ekaterina Gradoboeva, Ekaterina Savkina, Daria Nashatyreva, Elena Poleshchuk, Aleksei Vasilenko, Valery Yakimenko, Andrey Komissarov
EPI_ISL_639948, EPI_ISL_639949, EPI_ISL_639950, EPI_ISL_639951, EPI_ISL_639952, EPI_ISL_639953, EPI_ISL_639954, EPI_ISL_639955, EPI_ISL_639956, EPI_ISL_639957, EPI_ISL_639958, EPI_ISL_639959, EPI_ISL_639960, EPI_ISL_639961, EPI_ISL_639962, EPI_ISL_639963, EPI_ISL_639964, EPI_ISL_639965, EPI_ISL_639966, EPI_ISL_639967, EPI_ISL_639968, EPI_ISL_639969, EPI_ISL_639970, EPI_ISL_639971, EPI_ISL_639972, EPI_ISL_639973			
see above	HELIX LLC	WHO National Influenza Centre Russian Federation	Andrey Komissarov, Artem Fadeev, Kseniya Komissarova, Anna Ivanova, Dmitry Bazhenov, Daria Danilenko
EPI_ISL_639974, EPI_ISL_639975, EPI_ISL_639976, EPI_ISL_639977	CNR Virus des Infections Respiratoires - France SUD	CNR Virus des Infections Respiratoires - France SUD	Antonin Bal, Gregory Destras, Gwendolyne Burfin, Hadrien Règue, Alexandre Gaymard, Maude Bouscambert-Duchamp, Florence Morfin-Sherpa, Martine Valette, Bruno Lina, Laurence Josset
EPI_ISL_639978	Centre Hospitalier Saint Joseph Saint Luc	CNR Virus des Infections Respiratoires - France SUD	Antonin Bal, Gregory Destras, Gwendolyne Burfin, Hadrien Règue, Alexandre Gaymard, Maude Bouscambert-Duchamp, Florence Morfin-Sherpa, Martine Valette, Bruno Lina, Laurence Josset
EPI_ISL_639979, EPI_ISL_639980, EPI_ISL_639981, EPI_ISL_639982, EPI_ISL_639983	CNR Virus des Infections Respiratoires - France SUD	CNR Virus des Infections Respiratoires - France SUD	Antonin Bal, Gregory Destras, Gwendolyne Burfin, Hadrien Règue, Alexandre Gaymard, Maude Bouscambert-Duchamp, Florence Morfin-Sherpa, Martine Valette, Bruno Lina, Laurence Josset
EPI_ISL_639984	Centre Hospitalier de Bourg en Bresse	CNR Virus des Infections Respiratoires - France SUD	Antonin Bal, Gregory Destras, Gwendolyne Burfin, Hadrien Règue, Alexandre Gaymard, Maude Bouscambert-Duchamp, Florence Morfin-Sherpa, Martine Valette, Bruno Lina, Laurence Josset
EPI_ISL_639985, EPI_ISL_639986, EPI_ISL_639987, EPI_ISL_639988, EPI_ISL_639989, EPI_ISL_639990, EPI_ISL_639991, EPI_ISL_639992, EPI_ISL_639993, EPI_ISL_639994, EPI_ISL_639995, EPI_ISL_639996, EPI_ISL_639997, EPI_ISL_639998, EPI_ISL_639999, EPI_ISL_640000, EPI_ISL_640001, EPI_ISL_640002, EPI_ISL_640003, EPI_ISL_640004, EPI_ISL_640005, EPI_ISL_640006, EPI_ISL_640007, EPI_ISL_640008, EPI_ISL_640009, EPI_ISL_640010, EPI_ISL_640011, EPI_ISL_640012, EPI_ISL_640013, EPI_ISL_640014			
see above	CNR Virus des Infections Respiratoires - France SUD	CNR Virus des Infections Respiratoires - France SUD	Antonin Bal, Gregory Destras, Gwendolyne Burfin, Hadrien Règue, Alexandre Gaymard, Maude Bouscambert-Duchamp, Florence Morfin-Sherpa, Martine Valette, Bruno Lina, Laurence Josset
EPI_ISL_640015	Conville CDC wc CVC	NHLS/UCT	Arash Iranzadeh, Deelan Doolabh, Lynn Tyers, Bruna Galvao, Innocent Mudau, Marvin Hsiao, Kruger Marais, Diana Hardie, Stephen Korsman, Carolyn Williamson
EPI_ISL_640016	George Hospital wc GRH	NHLS/UCT	Arash Iranzadeh, Deelan Doolabh, Lynn Tyers, Bruna Galvao, Innocent Mudau, Marvin Hsiao, Kruger Marais, Diana Hardie, Stephen Korsman, Carolyn Williamson
EPI_ISL_640017	Oudtshoorn Hospital wc OUD	NHLS/UCT	Arash Iranzadeh, Deelan Doolabh, Lynn Tyers, Bruna Galvao, Innocent Mudau, Marvin Hsiao, Kruger Marais, Diana Hardie, Stephen Korsman, Carolyn Williamson
EPI_ISL_640018	Heidelberg Clinic wc HBC	NHLS/UCT	Arash Iranzadeh, Deelan Doolabh, Lynn Tyers, Bruna Galvao, Innocent Mudau, Marvin Hsiao, Kruger Marais, Diana Hardie, Stephen Korsman, Carolyn Williamson
EPI_ISL_640019	Thembalethu CDC wc THC	NHLS/UCT	Arash Iranzadeh, Deelan Doolabh, Lynn Tyers, Bruna Galvao, Innocent Mudau, Marvin Hsiao, Kruger Marais, Diana Hardie, Stephen Korsman, Carolyn Williamson
EPI_ISL_640020	Mitchells Plain Hospital wc MPH	NHLS/UCT	Arash Iranzadeh, Deelan Doolabh, Lynn Tyers, Bruna Galvao, Innocent Mudau, Marvin Hsiao, Kruger Marais, Diana Hardie, Stephen Korsman, Carolyn Williamson
EPI_ISL_640021	Victoria Hospital wc VHW	NHLS/UCT	Arash Iranzadeh, Deelan Doolabh, Lynn Tyers, Bruna Galvao, Innocent Mudau, Marvin Hsiao, Kruger Marais, Diana Hardie, Stephen Korsman, Carolyn Williamson
EPI_ISL_640022	Knysna Hospital wc KNY	NHLS/UCT	Arash Iranzadeh, Deelan Doolabh, Lynn Tyers, Bruna Galvao, Innocent Mudau, Marvin Hsiao, Kruger Marais, Diana Hardie, Stephen Korsman, Carolyn Williamson
EPI_ISL_640023	Oudtshoorn Hospital wc OUD	NHLS/UCT	Arash Iranzadeh, Deelan Doolabh, Lynn Tyers, Bruna Galvao, Innocent Mudau, Marvin Hsiao, Kruger Marais, Diana Hardie, Stephen Korsman, Carolyn Williamson
EPI_ISL_640024	Dysselsdorp Clinic wc DDC	NHLS/UCT	Arash Iranzadeh, Deelan Doolabh, Lynn Tyers, Bruna Galvao, Innocent Mudau, Marvin Hsiao, Kruger Marais, Diana Hardie, Stephen Korsman, Carolyn Williamson
EPI_ISL_640025	Mitchells Plain CHC wc MHC	NHLS/UCT	Arash Iranzadeh, Deelan Doolabh, Lynn Tyers, Bruna Galvao, Innocent Mudau, Marvin Hsiao, Kruger Marais, Diana Hardie, Stephen Korsman, Carolyn Williamson
EPI_ISL_640026	Thembalethu CDC wc THC	NHLS/UCT	Arash Iranzadeh, Deelan Doolabh, Lynn Tyers, Bruna Galvao, Innocent Mudau, Marvin Hsiao, Kruger Marais, Diana Hardie, Stephen Korsman, Carolyn Williamson
EPI_ISL_640027	George Hospital wc GRH	NHLS/UCT	Arash Iranzadeh, Deelan Doolabh, Lynn Tyers, Bruna Galvao, Innocent Mudau, Marvin Hsiao, Kruger Marais, Diana Hardie, Stephen Korsman, Carolyn Williamson
EPI_ISL_640028	Mitchells Plain Hospital wc MPH	NHLS/UCT	Arash Iranzadeh, Deelan Doolabh, Lynn Tyers, Bruna Galvao, Innocent Mudau, Marvin Hsiao, Kruger Marais, Diana Hardie, Stephen Korsman, Carolyn Williamson

EPI_ISL_640029	D'Almeida Clinic wc DAL	NHLS/UCT	Arash Iranzadeh, Deelan Doolabh, Lynn Tyers, Bruna Galvao, Innocent Mudau, Marvin Hsiao, Kruger Marais, Diana Hardie, Stephen Korsman, Carolyn Williamson
EPI_ISL_640030	Kwamandlenkosi Clinic wc KMN	NHLS/UCT	Arash Iranzadeh, Deelan Doolabh, Lynn Tyers, Bruna Galvao, Innocent Mudau, Marvin Hsiao, Kruger Marais, Diana Hardie, Stephen Korsman, Carolyn Williamson
EPI_ISL_640031	Beaufort West Hospital wc BWH	NHLS/UCT	Arash Iranzadeh, Deelan Doolabh, Lynn Tyers, Bruna Galvao, Innocent Mudau, Marvin Hsiao, Kruger Marais, Diana Hardie, Stephen Korsman, Carolyn Williamson
EPI_ISL_640032	Victoria Hospital wc VHW	NHLS/UCT	Arash Iranzadeh, Deelan Doolabh, Lynn Tyers, Bruna Galvao, Innocent Mudau, Marvin Hsiao, Kruger Marais, Diana Hardie, Stephen Korsman, Carolyn Williamson
EPI_ISL_640033	False Bay Hospital wc FBH	NHLS/UCT	Arash Iranzadeh, Deelan Doolabh, Lynn Tyers, Bruna Galvao, Innocent Mudau, Marvin Hsiao, Kruger Marais, Diana Hardie, Stephen Korsman, Carolyn Williamson
EPI_ISL_640034	Sedgefield Clinic wc SGE	NHLS/UCT	Arash Iranzadeh, Deelan Doolabh, Lynn Tyers, Bruna Galvao, Innocent Mudau, Marvin Hsiao, Kruger Marais, Diana Hardie, Stephen Korsman, Carolyn Williamson
EPI_ISL_640035	Riversdale Clinic wc RAV	NHLS/UCT	Arash Iranzadeh, Deelan Doolabh, Lynn Tyers, Bruna Galvao, Innocent Mudau, Marvin Hsiao, Kruger Marais, Diana Hardie, Stephen Korsman, Carolyn Williamson
EPI_ISL_640036	Bridgeton CDC wc BTC	NHLS/UCT	Arash Iranzadeh, Deelan Doolabh, Lynn Tyers, Bruna Galvao, Innocent Mudau, Marvin Hsiao, Kruger Marais, Diana Hardie, Stephen Korsman, Carolyn Williamson
EPI_ISL_640037, EPI_ISL_640046	Valkenberg Hospital wc VBH	NHLS/UCT	Arash Iranzadeh, Deelan Doolabh, Lynn Tyers, Bruna Galvao, Innocent Mudau, Marvin Hsiao, Kruger Marais, Diana Hardie, Stephen Korsman, Carolyn Williamson
EPI_ISL_640047	Beaufort West Hospital wc BWH	NHLS/UCT	Arash Iranzadeh, Deelan Doolabh, Lynn Tyers, Bruna Galvao, Innocent Mudau, Marvin Hsiao, Kruger Marais, Diana Hardie, Stephen Korsman, Carolyn Williamson
EPI_ISL_640048	D'Almeida Clinic wc DAL	NHLS/UCT	Arash Iranzadeh, Deelan Doolabh, Lynn Tyers, Bruna Galvao, Innocent Mudau, Marvin Hsiao, Kruger Marais, Diana Hardie, Stephen Korsman, Carolyn Williamson
EPI_ISL_640049	George Road Sat Clinic wc GWM	NHLS/UCT	Arash Iranzadeh, Deelan Doolabh, Lynn Tyers, Bruna Galvao, Innocent Mudau, Marvin Hsiao, Kruger Marais, Diana Hardie, Stephen Korsman, Carolyn Williamson
EPI_ISL_640064	Ethembeni Clinic [Prieska]	NHLS/UCT	Arash Iranzadeh, Deelan Doolabh, Lynn Tyers, Bruna Galvao, Innocent Mudau, Marvin Hsiao, Kruger Marais, Diana Hardie, Stephen Korsman, Carolyn Williamson
EPI_ISL_640065	Mitchells Plain Hospital wc MPH	NHLS/UCT	Arash Iranzadeh, Deelan Doolabh, Lynn Tyers, Bruna Galvao, Innocent Mudau, Marvin Hsiao, Kruger Marais, Diana Hardie, Stephen Korsman, Carolyn Williamson
EPI_ISL_640067	Groote Schuur Hospital wc GSH	NHLS/UCT	Arash Iranzadeh, Deelan Doolabh, Lynn Tyers, Bruna Galvao, Innocent Mudau, Marvin Hsiao, Kruger Marais, Diana Hardie, Stephen Korsman, Carolyn Williamson
EPI_ISL_640068	Clinic-in-Asla	NHLS/UCT	Arash Iranzadeh, Deelan Doolabh, Lynn Tyers, Bruna Galvao, Innocent Mudau, Marvin Hsiao, Kruger Marais, Diana Hardie, Stephen Korsman, Carolyn Williamson
EPI_ISL_640069, EPI_ISL_640070	Guguletu CHC wc GDH	NHLS/UCT	Arash Iranzadeh, Deelan Doolabh, Lynn Tyers, Bruna Galvao, Innocent Mudau, Marvin Hsiao, Kruger Marais, Diana Hardie, Stephen Korsman, Carolyn Williamson
EPI_ISL_640072	Vanguard CHC wc VGC	NHLS/UCT	Arash Iranzadeh, Deelan Doolabh, Lynn Tyers, Bruna Galvao, Innocent Mudau, Marvin Hsiao, Kruger Marais, Diana Hardie, Stephen Korsman, Carolyn Williamson
EPI_ISL_640074	Mamre CDC wc MRC	NHLS/UCT	Arash Iranzadeh, Deelan Doolabh, Lynn Tyers, Bruna Galvao, Innocent Mudau, Marvin Hsiao, Kruger Marais, Diana Hardie, Stephen Korsman, Carolyn Williamson
EPI_ISL_640075	Bothasig CDC wc BLD	NHLS/UCT	Arash Iranzadeh, Deelan Doolabh, Lynn Tyers, Bruna Galvao, Innocent Mudau, Marvin Hsiao, Kruger Marais, Diana Hardie, Stephen Korsman, Carolyn Williamson
EPI_ISL_640076	Knysna Hospital wc KNY	NHLS/UCT	Arash Iranzadeh, Deelan Doolabh, Lynn Tyers, Bruna Galvao, Innocent Mudau, Marvin Hsiao, Kruger Marais, Diana Hardie, Stephen Korsman, Carolyn Williamson
EPI_ISL_640077	Oudtshoorn Hospital wc OUD	NHLS/UCT	Arash Iranzadeh, Deelan Doolabh, Lynn Tyers, Bruna Galvao, Innocent Mudau, Marvin Hsiao, Kruger Marais, Diana Hardie, Stephen Korsman, Carolyn Williamson
EPI_ISL_640078	2 Military Hospital wc MAA	NHLS/UCT	Arash Iranzadeh, Deelan Doolabh, Lynn Tyers, Bruna Galvao, Innocent Mudau, Marvin Hsiao, Kruger Marais, Diana Hardie, Stephen Korsman, Carolyn Williamson
EPI_ISL_640079	Stellenbosch Hospital wc STB	NHLS/UCT	Arash Iranzadeh, Deelan Doolabh, Lynn Tyers, Bruna Galvao, Innocent Mudau, Marvin Hsiao, Kruger Marais, Diana Hardie, Stephen Korsman, Carolyn Williamson
EPI_ISL_640084	Groote Schuur Hospital wc GSH	NHLS/UCT	Arash Iranzadeh, Deelan Doolabh, Lynn Tyers, Bruna Galvao, Innocent Mudau, Marvin Hsiao, Kruger Marais, Diana Hardie, Stephen Korsman, Carolyn Williamson
EPI_ISL_640085	Victoria Hospital wc VHW	NHLS/UCT	Arash Iranzadeh, Deelan Doolabh, Lynn Tyers, Bruna Galvao, Innocent Mudau, Marvin Hsiao, Kruger Marais, Diana Hardie, Stephen Korsman, Carolyn Williamson
EPI_ISL_640086	Red Cross Children's Hospital wc RXH	NHLS/UCT	Arash Iranzadeh, Deelan Doolabh, Lynn Tyers, Bruna Galvao, Innocent Mudau, Marvin Hsiao, Kruger Marais, Diana Hardie, Stephen Korsman, Carolyn Williamson
EPI_ISL_640087	Victoria Hospital wc VHW	NHLS/UCT	Arash Iranzadeh, Deelan Doolabh, Lynn Tyers, Bruna Galvao, Innocent Mudau, Marvin Hsiao, Kruger Marais, Diana Hardie, Stephen Korsman, Carolyn Williamson
EPI_ISL_640088	False Bay Hospital wc FBH	NHLS/UCT	Arash Iranzadeh, Deelan Doolabh, Lynn Tyers, Bruna Galvao, Innocent Mudau, Marvin Hsiao, Kruger Marais, Diana Hardie, Stephen Korsman, Carolyn Williamson
EPI_ISL_640089	2 Military Hospital wc MAA	NHLS/UCT	Arash Iranzadeh, Deelan Doolabh, Lynn Tyers, Bruna Galvao, Innocent Mudau, Marvin Hsiao, Kruger Marais, Diana Hardie, Stephen Korsman, Carolyn Williamson
EPI_ISL_640090	False Bay Hospital wc FBH	NHLS/UCT	Arash Iranzadeh, Deelan Doolabh, Lynn Tyers, Bruna Galvao, Innocent Mudau, Marvin Hsiao, Kruger Marais, Diana Hardie, Stephen Korsman, Carolyn Williamson
EPI_ISL_640091	Du Noon CDC wc DNC	NHLS/UCT	Arash Iranzadeh, Deelan Doolabh, Lynn Tyers, Bruna Galvao, Innocent Mudau, Marvin Hsiao, Kruger Marais, Diana Hardie, Stephen Korsman, Carolyn Williamson
EPI_ISL_640092, EPI_ISL_640093	Kensington CDC wc KSC	NHLS/UCT	Arash Iranzadeh, Deelan Doolabh, Lynn Tyers, Bruna Galvao, Innocent Mudau, Marvin Hsiao, Kruger Marais, Diana Hardie, Stephen Korsman, Carolyn Williamson
EPI_ISL_640094, EPI_ISL_640095	Du Noon CDC wc DNC	NHLS/UCT	Arash Iranzadeh, Deelan Doolabh, Lynn Tyers, Bruna Galvao, Innocent Mudau, Marvin Hsiao, Kruger Marais, Diana Hardie, Stephen Korsman, Carolyn Williamson
EPI_ISL_640107	2 Military Hospital wc MAA	NHLS/UCT	Arash Iranzadeh, Deelan Doolabh, Lynn Tyers, Bruna Galvao, Innocent Mudau, Marvin Hsiao, Kruger Marais, Diana Hardie, Stephen Korsman, Carolyn Williamson

EPI_ISL_640108, EPI_ISL_640109, EPI_ISL_640110	Victoria Hospital wc VHW	NHLS/UCT	Arash Iranzadeh, Deelan Doolabh, Lynn Tyers, Bruna Galvao, Innocent Mudau, Marvin Hsiao, Kruger Marais, Diana Hardie, Stephen Korsman, Carolyn Williamson
EPI_ISL_640111, EPI_ISL_640112	Groote Schuur Hospital wc GSH	NHLS/UCT	Arash Iranzadeh, Deelan Doolabh, Lynn Tyers, Bruna Galvao, Innocent Mudau, Marvin Hsiao, Kruger Marais, Diana Hardie, Stephen Korsman, Carolyn Williamson
EPI_ISL_640113	Victoria Hospital wc VHW	NHLS/UCT	Arash Iranzadeh, Deelan Doolabh, Lynn Tyers, Bruna Galvao, Innocent Mudau, Marvin Hsiao, Kruger Marais, Diana Hardie, Stephen Korsman, Carolyn Williamson
EPI_ISL_640114	Valkenberg Hospital wc VBH	NHLS/UCT	Arash Iranzadeh, Deelan Doolabh, Lynn Tyers, Bruna Galvao, Innocent Mudau, Marvin Hsiao, Kruger Marais, Diana Hardie, Stephen Korsman, Carolyn Williamson
EPI_ISL_640115	2 Military Hospital wc MAA	NHLS/UCT	Arash Iranzadeh, Deelan Doolabh, Lynn Tyers, Bruna Galvao, Innocent Mudau, Marvin Hsiao, Kruger Marais, Diana Hardie, Stephen Korsman, Carolyn Williamson
EPI_ISL_640116	False Bay Hospital wc FBH	NHLS/UCT	Arash Iranzadeh, Deelan Doolabh, Lynn Tyers, Bruna Galvao, Innocent Mudau, Marvin Hsiao, Kruger Marais, Diana Hardie, Stephen Korsman, Carolyn Williamson
EPI_ISL_640117	2 Military Hospital wc MAA	NHLS/UCT	Arash Iranzadeh, Deelan Doolabh, Lynn Tyers, Bruna Galvao, Innocent Mudau, Marvin Hsiao, Kruger Marais, Diana Hardie, Stephen Korsman, Carolyn Williamson
EPI_ISL_640118	Conville CDC wc CVC	NHLS/UCT	Arash Iranzadeh, Deelan Doolabh, Lynn Tyers, Bruna Galvao, Innocent Mudau, Marvin Hsiao, Kruger Marais, Diana Hardie, Stephen Korsman, Carolyn Williamson
EPI_ISL_640119, EPI_ISL_640120	Groote Schuur Hospital wc GSH	NHLS/UCT	Arash Iranzadeh, Deelan Doolabh, Lynn Tyers, Bruna Galvao, Innocent Mudau, Marvin Hsiao, Kruger Marais, Diana Hardie, Stephen Korsman, Carolyn Williamson
EPI_ISL_640121	False Bay Hospital wc FBH	NHLS/UCT	Arash Iranzadeh, Deelan Doolabh, Lynn Tyers, Bruna Galvao, Innocent Mudau, Marvin Hsiao, Kruger Marais, Diana Hardie, Stephen Korsman, Carolyn Williamson
EPI_ISL_640122	Groote Schuur Hospital wc GSH	NHLS/UCT	Arash Iranzadeh, Deelan Doolabh, Lynn Tyers, Bruna Galvao, Innocent Mudau, Marvin Hsiao, Kruger Marais, Diana Hardie, Stephen Korsman, Carolyn Williamson
EPI_ISL_640123, EPI_ISL_640124, EPI_ISL_640125	False Bay Hospital wc FBH	NHLS/UCT	Arash Iranzadeh, Deelan Doolabh, Lynn Tyers, Bruna Galvao, Innocent Mudau, Marvin Hsiao, Kruger Marais, Diana Hardie, Stephen Korsman, Carolyn Williamson
EPI_ISL_640126	Victoria Hospital wc VHW	NHLS/UCT	Arash Iranzadeh, Deelan Doolabh, Lynn Tyers, Bruna Galvao, Innocent Mudau, Marvin Hsiao, Kruger Marais, Diana Hardie, Stephen Korsman, Carolyn Williamson
EPI_ISL_640127	2 Military Hospital wc MAA	NHLS/UCT	Arash Iranzadeh, Deelan Doolabh, Lynn Tyers, Bruna Galvao, Innocent Mudau, Marvin Hsiao, Kruger Marais, Diana Hardie, Stephen Korsman, Carolyn Williamson
EPI_ISL_640128, EPI_ISL_640129, EPI_ISL_640130, EPI_ISL_640131, EPI_ISL_640132, EPI_ISL_640133, EPI_ISL_640134, EPI_ISL_640135	Groote Schuur Hospital wc GSH	NHLS/UCT	Arash Iranzadeh, Deelan Doolabh, Lynn Tyers, Bruna Galvao, Innocent Mudau, Marvin Hsiao, Kruger Marais, Diana Hardie, Stephen Korsman, Carolyn Williamson
EPI_ISL_640136	Red Cross Children's Hospital wc RXH	NHLS/UCT	Arash Iranzadeh, Deelan Doolabh, Lynn Tyers, Bruna Galvao, Innocent Mudau, Marvin Hsiao, Kruger Marais, Diana Hardie, Stephen Korsman, Carolyn Williamson
EPI_ISL_640137, EPI_ISL_640138, EPI_ISL_640139, EPI_ISL_640140	Groote Schuur Hospital wc GSH	NHLS/UCT	Arash Iranzadeh, Deelan Doolabh, Lynn Tyers, Bruna Galvao, Innocent Mudau, Marvin Hsiao, Kruger Marais, Diana Hardie, Stephen Korsman, Carolyn Williamson
EPI_ISL_640141	Victoria Hospital wc VHW	NHLS/UCT	Arash Iranzadeh, Deelan Doolabh, Lynn Tyers, Bruna Galvao, Innocent Mudau, Marvin Hsiao, Kruger Marais, Diana Hardie, Stephen Korsman, Carolyn Williamson
EPI_ISL_640142, EPI_ISL_640143, EPI_ISL_640144, EPI_ISL_640145, EPI_ISL_640146, EPI_ISL_640147, EPI_ISL_640148, EPI_ISL_640149, EPI_ISL_640150, EPI_ISL_640151, EPI_ISL_640152, EPI_ISL_640153, EPI_ISL_640154, EPI_ISL_640155, EPI_ISL_640156, EPI_ISL_640157, EPI_ISL_640158, EPI_ISL_640159, EPI_ISL_640160, EPI_ISL_640161, EPI_ISL_640162, EPI_ISL_640163, EPI_ISL_640164, EPI_ISL_640165, EPI_ISL_640166, EPI_ISL_640167, EPI_ISL_640168, EPI_ISL_640169, EPI_ISL_640170, EPI_ISL_640171, EPI_ISL_640172, EPI_ISL_640173, EPI_ISL_640174, EPI_ISL_640175, EPI_ISL_640176, EPI_ISL_640177, EPI_ISL_640178, EPI_ISL_640179, EPI_ISL_640180, EPI_ISL_640181, EPI_ISL_640182, EPI_ISL_640183, EPI_ISL_640184, EPI_ISL_640185, EPI_ISL_640186, EPI_ISL_640187, EPI_ISL_640188, EPI_ISL_640189, EPI_ISL_640190, EPI_ISL_640191, EPI_ISL_640192, EPI_ISL_640193, EPI_ISL_640194, EPI_ISL_640195, EPI_ISL_640196, EPI_ISL_640197, EPI_ISL_640198, EPI_ISL_640199, EPI_ISL_640200, EPI_ISL_640201, EPI_ISL_640202, EPI_ISL_640203, EPI_ISL_640204, EPI_ISL_640205, EPI_ISL_640206, EPI_ISL_640207, EPI_ISL_640208, EPI_ISL_640209, EPI_ISL_640210, EPI_ISL_640211, EPI_ISL_640212, EPI_ISL_640213, EPI_ISL_640214, EPI_ISL_640215, EPI_ISL_640216, EPI_ISL_640217, EPI_ISL_640218, EPI_ISL_640273, EPI_ISL_640274, EPI_ISL_640275			
see above	University of Michigan Clinical Microbiology Laboratory	Lauring Lab, University of Michigan, Department of Microbiology and Immunology	Valesano
EPI_ISL_640279, EPI_ISL_640280, EPI_ISL_640281, EPI_ISL_640288, EPI_ISL_640289, EPI_ISL_640290, EPI_ISL_640298, EPI_ISL_640299, EPI_ISL_640300	Utah Public Health Laboratory	Utah Public Health Laboratory	Erin Young, Kelly Oakeson
EPI_ISL_640301, EPI_ISL_640303, EPI_ISL_640304, EPI_ISL_640305, EPI_ISL_640306, EPI_ISL_640307, EPI_ISL_640308, EPI_ISL_640309, EPI_ISL_640310, EPI_ISL_640312, EPI_ISL_640313, EPI_ISL_640314, EPI_ISL_640315, EPI_ISL_640316, EPI_ISL_640317, EPI_ISL_640318, EPI_ISL_640320, EPI_ISL_640321, EPI_ISL_640322, EPI_ISL_640323, EPI_ISL_640324, EPI_ISL_640325, EPI_ISL_640327, EPI_ISL_640328, EPI_ISL_640329, EPI_ISL_640331, EPI_ISL_640332, EPI_ISL_640333, EPI_ISL_640334, EPI_ISL_640335, EPI_ISL_640336, EPI_ISL_640338, EPI_ISL_640339, EPI_ISL_640340, EPI_ISL_640341, EPI_ISL_640343, EPI_ISL_640344, EPI_ISL_640345, EPI_ISL_640346, EPI_ISL_640347, EPI_ISL_640348, EPI_ISL_640351, EPI_ISL_640352, EPI_ISL_640353, EPI_ISL_640354, EPI_ISL_640355, EPI_ISL_640356, EPI_ISL_640357, EPI_ISL_640359, EPI_ISL_640360, EPI_ISL_640361, EPI_ISL_640362, EPI_ISL_640363, EPI_ISL_640364, EPI_ISL_640365, EPI_ISL_640367, EPI_ISL_640368, EPI_ISL_640369, EPI_ISL_640370, EPI_ISL_640371, EPI_ISL_640373, EPI_ISL_640374, EPI_ISL_640376, EPI_ISL_640377, EPI_ISL_640378, EPI_ISL_640379, EPI_ISL_640380, EPI_ISL_640381, EPI_ISL_640382, EPI_ISL_640383, EPI_ISL_640384, EPI_ISL_640385, EPI_ISL_640387, EPI_ISL_640388, EPI_ISL_640390			
see above	Microbiological Diagnostic Unit - Public Health Laboratory (MDU-PHL)	MDU-PHL	Seemann T., Schultz M.B., Sait, M.L., Sherry, N.L.
EPI_ISL_640391	Victorian Infectious Diseases Reference Laboratory (VIDRL)	VIDRL and MDU-PHL	Caly L., Seemann T., Sait, M.L., Schultz M.B., Druce J., Sherry, N.L.
EPI_ISL_640392, EPI_ISL_640394, EPI_ISL_640395, EPI_ISL_640396, EPI_ISL_640397, EPI_ISL_640398, EPI_ISL_640399, EPI_ISL_640400, EPI_ISL_640401, EPI_ISL_640402, EPI_ISL_640403, EPI_ISL_640404, EPI_ISL_640405, EPI_ISL_640406, EPI_ISL_640408, EPI_ISL_640409, EPI_ISL_640410, EPI_ISL_640411, EPI_ISL_640412, EPI_ISL_640413, EPI_ISL_640414, EPI_ISL_640415, EPI_ISL_640416, EPI_ISL_640417, EPI_ISL_640418, EPI_ISL_640420, EPI_ISL_640421, EPI_ISL_640422, EPI_ISL_640423, EPI_ISL_640424, EPI_ISL_640425, EPI_ISL_640426, EPI_ISL_640427, EPI_ISL_640428, EPI_ISL_640430, EPI_ISL_640432, EPI_ISL_640433, EPI_ISL_640434, EPI_ISL_640435, EPI_ISL_640436, EPI_ISL_640439, EPI_ISL_640441, EPI_ISL_640442, EPI_ISL_640443, EPI_ISL_640444, EPI_ISL_640445, EPI_ISL_640446, EPI_ISL_640448, EPI_ISL_640449, EPI_ISL_640450, EPI_ISL_640451, EPI_ISL_640452, EPI_ISL_640454, EPI_ISL_640455, EPI_ISL_640456, EPI_ISL_640457, EPI_ISL_640458, EPI_ISL_640459, EPI_ISL_640460, EPI_ISL_640461, EPI_ISL_640462, EPI_ISL_640464, EPI_ISL_640466, EPI_ISL_640467, EPI_ISL_640468, EPI_ISL_640469, EPI_ISL_640470, EPI_ISL_640471, EPI_ISL_640472, EPI_ISL_640473, EPI_ISL_640474, EPI_ISL_640475, EPI_ISL_640478, EPI_ISL_640479, EPI_ISL_640480, EPI_ISL_640481, EPI_ISL_640482, EPI_ISL_640484, EPI_ISL_640485, EPI_ISL_640486, EPI_ISL_640487, EPI_ISL_640490, EPI_ISL_640491, EPI_ISL_640492, EPI_ISL_640493, EPI_ISL_640494, EPI_ISL_640495, EPI_ISL_640496, EPI_ISL_640497, EPI_ISL_640498, EPI_ISL_640499, EPI_ISL_640500, EPI_ISL_640501, EPI_ISL_640502, EPI_ISL_640503, EPI_ISL_640504, EPI_ISL_640505, EPI_ISL_640506, EPI_ISL_640507, EPI_ISL_640508, EPI_ISL_640510, EPI_ISL_640511, EPI_ISL_640513, EPI_ISL_640514, EPI_ISL_640515, EPI_ISL_640516, EPI_ISL_640517, EPI_ISL_640518			
see above	Microbiological Diagnostic Unit - Public Health Laboratory (MDU-PHL)	MDU-PHL	Seemann T., Schultz M.B., Sait, M.L., Sherry, N.L.
EPI_ISL_640519	Victorian Infectious Diseases Reference Laboratory (VIDRL)	VIDRL and MDU-PHL	Caly L., Seemann T., Sait, M.L., Schultz M.B., Druce J., Sherry, N.L.
EPI_ISL_640520, EPI_ISL_640521, EPI_ISL_640522, EPI_ISL_640523, EPI_ISL_640524, EPI_ISL_640526, EPI_ISL_640527, EPI_ISL_640528, EPI_ISL_640529, EPI_ISL_640530, EPI_ISL_640531, EPI_ISL_640532, EPI_ISL_640533, EPI_ISL_640535, EPI_ISL_640536, EPI_ISL_640537, EPI_ISL_640538, EPI_ISL_640539, EPI_ISL_640540, EPI_ISL_640541, EPI_ISL_640542, EPI_ISL_640543, EPI_ISL_640544, EPI_ISL_640545, EPI_ISL_640546, EPI_ISL_640547, EPI_ISL_640549, EPI_ISL_640551, EPI_ISL_640552, EPI_ISL_640553, EPI_ISL_640554, EPI_ISL_640555, EPI_ISL_640556, EPI_ISL_640557, EPI_ISL_640558, EPI_ISL_640559, EPI_ISL_640560, EPI_ISL_640561, EPI_ISL_640564, EPI_ISL_640565, EPI_ISL_640566, EPI_ISL_640568, EPI_ISL_640569, EPI_ISL_640570, EPI_ISL_640571, EPI_ISL_640572, EPI_ISL_640573, EPI_ISL_640574, EPI_ISL_640575, EPI_ISL_640576, EPI_ISL_640577, EPI_ISL_640578, EPI_ISL_640579, EPI_ISL_640581, EPI_ISL_640582			
see above	Microbiological Diagnostic Unit - Public Health Laboratory (MDU-PHL)	MDU-PHL	Seemann T., Schultz M.B., Sait, M.L., Sherry, N.L.

EPI_ISL_640583	Victorian Infectious Diseases Reference Laboratory (VIDRL)	VIDRL and MDU-PHL	Caly L., Seemann T., Sait, M.L., Schultz M.B., Druce J., Sherry, N.L.
EPI_ISL_640584, EPI_ISL_640586, EPI_ISL_640587	Microbiological Diagnostic Unit - Public Health Laboratory (MDU-PHL)	MDU-PHL	Seemann T., Schultz M.B., Sait, M.L., Sherry, N.L.
EPI_ISL_640588	Victorian Infectious Diseases Reference Laboratory (VIDRL)	VIDRL and MDU-PHL	Caly L., Seemann T., Sait, M.L., Schultz M.B., Druce J., Sherry, N.L.
EPI_ISL_640589, EPI_ISL_640591, EPI_ISL_640592, EPI_ISL_640593, EPI_ISL_640594, EPI_ISL_640595, EPI_ISL_640597, EPI_ISL_640598, EPI_ISL_640599, EPI_ISL_640600, EPI_ISL_640601, EPI_ISL_640602, EPI_ISL_640603, EPI_ISL_640604, EPI_ISL_640606			
see above	Microbiological Diagnostic Unit - Public Health Laboratory (MDU-PHL)	MDU-PHL	Seemann T., Schultz M.B., Sait, M.L., Sherry, N.L.
EPI_ISL_640607	Victorian Infectious Diseases Reference Laboratory (VIDRL)	VIDRL and MDU-PHL	Caly L., Seemann T., Sait, M.L., Schultz M.B., Druce J., Sherry, N.L.
EPI_ISL_640609, EPI_ISL_640610, EPI_ISL_640611, EPI_ISL_640612, EPI_ISL_640613, EPI_ISL_640614, EPI_ISL_640615, EPI_ISL_640616, EPI_ISL_640617, EPI_ISL_640618, EPI_ISL_640619, EPI_ISL_640620, EPI_ISL_640621, EPI_ISL_640622, EPI_ISL_640623, EPI_ISL_640624, EPI_ISL_640625, EPI_ISL_640627, EPI_ISL_640628, EPI_ISL_640629, EPI_ISL_640631, EPI_ISL_640632, EPI_ISL_640633, EPI_ISL_640634, EPI_ISL_640635, EPI_ISL_640636, EPI_ISL_640637, EPI_ISL_640638, EPI_ISL_640639, EPI_ISL_640640, EPI_ISL_640641, EPI_ISL_640642, EPI_ISL_640644, EPI_ISL_640645, EPI_ISL_640647, EPI_ISL_640648, EPI_ISL_640649, EPI_ISL_640650, EPI_ISL_640651, EPI_ISL_640652, EPI_ISL_640653, EPI_ISL_640654, EPI_ISL_640655, EPI_ISL_640657, EPI_ISL_640658, EPI_ISL_640659, EPI_ISL_640661, EPI_ISL_640662, EPI_ISL_640663, EPI_ISL_640664, EPI_ISL_640665, EPI_ISL_640666, EPI_ISL_640667, EPI_ISL_640668, EPI_ISL_640669, EPI_ISL_640670, EPI_ISL_640671, EPI_ISL_640672, EPI_ISL_640673, EPI_ISL_640675, EPI_ISL_640676, EPI_ISL_640677, EPI_ISL_640678, EPI_ISL_640679, EPI_ISL_640680, EPI_ISL_640682, EPI_ISL_640683, EPI_ISL_640684, EPI_ISL_640685, EPI_ISL_640686, EPI_ISL_640687, EPI_ISL_640688, EPI_ISL_640689, EPI_ISL_640690, EPI_ISL_640691, EPI_ISL_640692, EPI_ISL_640693, EPI_ISL_640694, EPI_ISL_640695, EPI_ISL_640696, EPI_ISL_640697, EPI_ISL_640698, EPI_ISL_640699, EPI_ISL_640701, EPI_ISL_640702, EPI_ISL_640703, EPI_ISL_640704, EPI_ISL_640705, EPI_ISL_640706, EPI_ISL_640707, EPI_ISL_640708, EPI_ISL_640709, EPI_ISL_640710, EPI_ISL_640711, EPI_ISL_640713, EPI_ISL_640714, EPI_ISL_640715, EPI_ISL_640716, EPI_ISL_640717, EPI_ISL_640718, EPI_ISL_640719, EPI_ISL_640721, EPI_ISL_640723, EPI_ISL_640724, EPI_ISL_640725, EPI_ISL_640726, EPI_ISL_640727, EPI_ISL_640728, EPI_ISL_640729, EPI_ISL_640730, EPI_ISL_640731, EPI_ISL_640732, EPI_ISL_640733, EPI_ISL_640734, EPI_ISL_640735, EPI_ISL_640736, EPI_ISL_640737, EPI_ISL_640738, EPI_ISL_640739, EPI_ISL_640742, EPI_ISL_640743, EPI_ISL_640744, EPI_ISL_640745, EPI_ISL_640746, EPI_ISL_640747, EPI_ISL_640748, EPI_ISL_640749, EPI_ISL_640750, EPI_ISL_640752, EPI_ISL_640753, EPI_ISL_640754, EPI_ISL_640755, EPI_ISL_640756, EPI_ISL_640757, EPI_ISL_640758, EPI_ISL_640759, EPI_ISL_640761, EPI_ISL_640762			
see above	Microbiological Diagnostic Unit - Public Health Laboratory (MDU-PHL)	MDU-PHL	Seemann T., Schultz M.B., Sait, M.L., Sherry, N.L.
EPI_ISL_640763	Victorian Infectious Diseases Reference Laboratory (VIDRL)	VIDRL and MDU-PHL	Caly L., Seemann T., Sait, M.L., Schultz M.B., Druce J., Sherry, N.L.
EPI_ISL_640764, EPI_ISL_640765, EPI_ISL_640766, EPI_ISL_640767, EPI_ISL_640768, EPI_ISL_640770, EPI_ISL_640771, EPI_ISL_640772, EPI_ISL_640773, EPI_ISL_640774, EPI_ISL_640776, EPI_ISL_640777, EPI_ISL_640778, EPI_ISL_640779, EPI_ISL_640780, EPI_ISL_640781, EPI_ISL_640782, EPI_ISL_640783			
see above	Microbiological Diagnostic Unit - Public Health Laboratory (MDU-PHL)	MDU-PHL	Seemann T., Schultz M.B., Sait, M.L., Sherry, N.L.
EPI_ISL_640784	Victorian Infectious Diseases Reference Laboratory (VIDRL)	VIDRL and MDU-PHL	Caly L., Seemann T., Sait, M.L., Schultz M.B., Druce J., Sherry, N.L.
EPI_ISL_640785, EPI_ISL_640786, EPI_ISL_640787, EPI_ISL_640788, EPI_ISL_640789, EPI_ISL_640790, EPI_ISL_640791, EPI_ISL_640792, EPI_ISL_640793, EPI_ISL_640794, EPI_ISL_640796, EPI_ISL_640798, EPI_ISL_640799, EPI_ISL_640801, EPI_ISL_640802, EPI_ISL_640805, EPI_ISL_640806, EPI_ISL_640807, EPI_ISL_640808, EPI_ISL_640809, EPI_ISL_640810, EPI_ISL_640811, EPI_ISL_640812, EPI_ISL_640813, EPI_ISL_640816, EPI_ISL_640817, EPI_ISL_640818, EPI_ISL_640820, EPI_ISL_640821, EPI_ISL_640822, EPI_ISL_640823, EPI_ISL_640824, EPI_ISL_640825, EPI_ISL_640826, EPI_ISL_640828, EPI_ISL_640829, EPI_ISL_640830, EPI_ISL_640831, EPI_ISL_640832, EPI_ISL_640833, EPI_ISL_640834, EPI_ISL_640835, EPI_ISL_640837, EPI_ISL_640838, EPI_ISL_640839, EPI_ISL_640840, EPI_ISL_640841, EPI_ISL_640842, EPI_ISL_640843, EPI_ISL_640844, EPI_ISL_640846, EPI_ISL_640847, EPI_ISL_640849, EPI_ISL_640850, EPI_ISL_640851, EPI_ISL_640852, EPI_ISL_640853, EPI_ISL_640854, EPI_ISL_640855, EPI_ISL_640856, EPI_ISL_640857, EPI_ISL_640858, EPI_ISL_640859, EPI_ISL_640860, EPI_ISL_640862, EPI_ISL_640864, EPI_ISL_640865, EPI_ISL_640866, EPI_ISL_640867, EPI_ISL_640868, EPI_ISL_640869, EPI_ISL_640870, EPI_ISL_640871, EPI_ISL_640872, EPI_ISL_640873, EPI_ISL_640874, EPI_ISL_640875, EPI_ISL_640876, EPI_ISL_640877, EPI_ISL_640878, EPI_ISL_640879, EPI_ISL_640880, EPI_ISL_640881, EPI_ISL_640882, EPI_ISL_640883, EPI_ISL_640884, EPI_ISL_640885, EPI_ISL_640886, EPI_ISL_640887, EPI_ISL_640888, EPI_ISL_640889, EPI_ISL_640890, EPI_ISL_640891, EPI_ISL_640892, EPI_ISL_640893, EPI_ISL_640894, EPI_ISL_640895, EPI_ISL_640896, EPI_ISL_640897, EPI_ISL_640899, EPI_ISL_640900, EPI_ISL_640901, EPI_ISL_640902, EPI_ISL_640903, EPI_ISL_640904, EPI_ISL_640905, EPI_ISL_640906, EPI_ISL_640907, EPI_ISL_640908, EPI_ISL_640909, EPI_ISL_640910, EPI_ISL_640911, EPI_ISL_640913, EPI_ISL_640914, EPI_ISL_640915, EPI_ISL_640916, EPI_ISL_640917, EPI_ISL_640918, EPI_ISL_640919, EPI_ISL_640920, EPI_ISL_640922, EPI_ISL_640923, EPI_ISL_640924, EPI_ISL_640925, EPI_ISL_640927, EPI_ISL_640928, EPI_ISL_640929, EPI_ISL_640930, EPI_ISL_640931, EPI_ISL_640932, EPI_ISL_640933, EPI_ISL_640934, EPI_ISL_640935, EPI_ISL_640936, EPI_ISL_640937, EPI_ISL_640938			
see above	Microbiological Diagnostic Unit - Public Health Laboratory (MDU-PHL)	MDU-PHL	Seemann T., Schultz M.B., Sait, M.L., Sherry, N.L.
EPI_ISL_640939	Victorian Infectious Diseases Reference Laboratory (VIDRL)	VIDRL and MDU-PHL	Caly L., Seemann T., Sait, M.L., Schultz M.B., Druce J., Sherry, N.L.
EPI_ISL_640940, EPI_ISL_640941, EPI_ISL_640943, EPI_ISL_640944, EPI_ISL_640945, EPI_ISL_640946, EPI_ISL_640948, EPI_ISL_640949, EPI_ISL_640950, EPI_ISL_640951, EPI_ISL_640952, EPI_ISL_640953, EPI_ISL_640954, EPI_ISL_640955, EPI_ISL_640956, EPI_ISL_640957, EPI_ISL_640960, EPI_ISL_640961, EPI_ISL_640962, EPI_ISL_640963, EPI_ISL_640964, EPI_ISL_640966, EPI_ISL_640967, EPI_ISL_640968, EPI_ISL_640969, EPI_ISL_640970, EPI_ISL_640971, EPI_ISL_640972, EPI_ISL_640973, EPI_ISL_640974, EPI_ISL_640975, EPI_ISL_640976, EPI_ISL_640977, EPI_ISL_640978, EPI_ISL_640979, EPI_ISL_640980, EPI_ISL_640981, EPI_ISL_640983, EPI_ISL_640985, EPI_ISL_640987, EPI_ISL_640989, EPI_ISL_640990, EPI_ISL_640991, EPI_ISL_640993, EPI_ISL_640994, EPI_ISL_640995, EPI_ISL_640996, EPI_ISL_640997, EPI_ISL_640998, EPI_ISL_640999, EPI_ISL_641000, EPI_ISL_641001, EPI_ISL_641002, EPI_ISL_641003, EPI_ISL_641004, EPI_ISL_641005, EPI_ISL_641006, EPI_ISL_641008, EPI_ISL_641009, EPI_ISL_641010, EPI_ISL_641011			
see above	Microbiological Diagnostic Unit - Public Health Laboratory (MDU-PHL)	MDU-PHL	Seemann T., Schultz M.B., Sait, M.L., Sherry, N.L.
EPI_ISL_641012	Victorian Infectious Diseases Reference Laboratory (VIDRL)	VIDRL and MDU-PHL	Caly L., Seemann T., Sait, M.L., Schultz M.B., Druce J., Sherry, N.L.
EPI_ISL_641013, EPI_ISL_641014, EPI_ISL_641015, EPI_ISL_641016	Microbiological Diagnostic Unit - Public Health Laboratory (MDU-PHL)	MDU-PHL	Seemann T., Schultz M.B., Sait, M.L., Sherry, N.L.
EPI_ISL_641017	Victorian Infectious Diseases Reference Laboratory (VIDRL)	VIDRL and MDU-PHL	Caly L., Seemann T., Sait, M.L., Schultz M.B., Druce J., Sherry, N.L.
EPI_ISL_641018, EPI_ISL_641019, EPI_ISL_641020, EPI_ISL_641021, EPI_ISL_641022, EPI_ISL_641023, EPI_ISL_641024, EPI_ISL_641025, EPI_ISL_641026, EPI_ISL_641027, EPI_ISL_641028, EPI_ISL_641029, EPI_ISL_641030, EPI_ISL_641031, EPI_ISL_641032, EPI_ISL_641034, EPI_ISL_641036, EPI_ISL_641037, EPI_ISL_641038, EPI_ISL_641040, EPI_ISL_641041, EPI_ISL_641042, EPI_ISL_641043, EPI_ISL_641045, EPI_ISL_641046, EPI_ISL_641047, EPI_ISL_641048, EPI_ISL_641050, EPI_ISL_641051, EPI_ISL_641052, EPI_ISL_641053, EPI_ISL_641054, EPI_ISL_641055, EPI_ISL_641056, EPI_ISL_641057, EPI_ISL_641058, EPI_ISL_641060, EPI_ISL_641061, EPI_ISL_641062, EPI_ISL_641063, EPI_ISL_641064, EPI_ISL_641065, EPI_ISL_641066, EPI_ISL_641068, EPI_ISL_641070, EPI_ISL_641071, EPI_ISL_641072, EPI_ISL_641073, EPI_ISL_641074, EPI_ISL_641078, EPI_ISL_641079, EPI_ISL_641080, EPI_ISL_641081, EPI_ISL_641082, EPI_ISL_641083, EPI_ISL_641084, EPI_ISL_641085, EPI_ISL_641086, EPI_ISL_641087, EPI_ISL_641089, EPI_ISL_641090, EPI_ISL_641091, EPI_ISL_641092, EPI_ISL_641093, EPI_ISL_641094, EPI_ISL_641095, EPI_ISL_641096, EPI_ISL_641098, EPI_ISL_641099, EPI_ISL_641100, EPI_ISL_641101			
see above	Microbiological Diagnostic Unit - Public Health Laboratory (MDU-PHL)	MDU-PHL	Seemann T., Schultz M.B., Sait, M.L., Sherry, N.L.
EPI_ISL_641102	Victorian Infectious Diseases Reference Laboratory (VIDRL)	VIDRL and MDU-PHL	Caly L., Seemann T., Sait, M.L., Schultz M.B., Druce J., Sherry, N.L.
EPI_ISL_641103, EPI_ISL_641104, EPI_ISL_641105, EPI_ISL_641106, EPI_ISL_641107, EPI_ISL_641108, EPI_ISL_641109, EPI_ISL_641111, EPI_ISL_641112, EPI_ISL_641113, EPI_ISL_641114, EPI_ISL_641115, EPI_ISL_641117, EPI_ISL_641118, EPI_ISL_641120			
see above	Microbiological Diagnostic Unit - Public Health Laboratory (MDU-PHL)	MDU-PHL	Seemann T., Schultz M.B., Sait, M.L., Sherry, N.L.
EPI_ISL_641121	Victorian Infectious Diseases Reference Laboratory (VIDRL)	VIDRL and MDU-PHL	Caly L., Seemann T., Sait, M.L., Schultz M.B., Druce J., Sherry, N.L.
EPI_ISL_641122, EPI_ISL_641123, EPI_ISL_641124	Microbiological Diagnostic Unit - Public Health Laboratory (MDU-PHL)	MDU-PHL	Seemann T., Schultz M.B., Sait, M.L., Sherry, N.L.
EPI_ISL_641125	Victorian Infectious Diseases Reference Laboratory (VIDRL)	VIDRL and MDU-PHL	Caly L., Seemann T., Sait, M.L., Schultz M.B., Druce J., Sherry, N.L.
EPI_ISL_641126, EPI_ISL_641127, EPI_ISL_641128, EPI_ISL_641129, EPI_ISL_641130, EPI_ISL_641131, EPI_ISL_641132, EPI_ISL_641133, EPI_ISL_641134, EPI_ISL_641135, EPI_ISL_641136, EPI_ISL_641137, EPI_ISL_641138, EPI_ISL_641139, EPI_ISL_641141, EPI_ISL_641142, EPI_ISL_641145, EPI_ISL_641146, EPI_ISL_641147, EPI_ISL_641148, EPI_ISL_641149, EPI_ISL_641150, EPI_ISL_641151, EPI_ISL_641152, EPI_ISL_641153, EPI_ISL_641154, EPI_ISL_641155, EPI_ISL_641158, EPI_ISL_641159, EPI_ISL_641160, EPI_ISL_641161, EPI_ISL_641162, EPI_ISL_641163, EPI_ISL_641164, EPI_ISL_641165, EPI_ISL_641167, EPI_ISL_641168, EPI_ISL_641169, EPI_ISL_641170, EPI_ISL_641171, EPI_ISL_641172, EPI_ISL_641173, EPI_ISL_641175, EPI_ISL_641176, EPI_ISL_641177, EPI_ISL_641178, EPI_ISL_641179, EPI_ISL_641181, EPI_ISL_641183, EPI_ISL_641185, EPI_ISL_641186, EPI_ISL_641187, EPI_ISL_641189, EPI_ISL_641190, EPI_ISL_641192, EPI_ISL_641193, EPI_ISL_641194, EPI_ISL_641195, EPI_ISL_641196, EPI_ISL_641197, EPI_ISL_641198, EPI_ISL_641199, EPI_ISL_641200, EPI_ISL_641201, EPI_ISL_641203, EPI_ISL_641204, EPI_ISL_641205, EPI_ISL_641206, EPI_ISL_641208, EPI_ISL_641209, EPI_ISL_641211, EPI_ISL_641212, EPI_ISL_641213, EPI_ISL_641214, EPI_ISL_641216, EPI_ISL_641217, EPI_ISL_641219, EPI_ISL_641220, EPI_ISL_641221, EPI_ISL_641222, EPI_ISL_641223, EPI_ISL_641224, EPI_ISL_641225, EPI_ISL_641226, EPI_ISL_641227, EPI_ISL_641228, EPI_ISL_641229, EPI_ISL_641230, EPI_ISL_641233, EPI_ISL_641234, EPI_ISL_641235, EPI_ISL_641236, EPI_ISL_641238, EPI_ISL_641241, EPI_ISL_641242, EPI_ISL_641243, EPI_ISL_641244, EPI_ISL_641245, EPI_ISL_641246, EPI_ISL_641247, EPI_ISL_641248, EPI_ISL_641249, EPI_ISL_641250, EPI_ISL_641251, EPI_ISL_641252, EPI_ISL_641254, EPI_ISL_641255, EPI_ISL_641256, EPI_ISL_641257, EPI_ISL_641258, EPI_ISL_641259, EPI_ISL_641260, EPI_ISL_641261, EPI_ISL_641262, EPI_ISL_641263, EPI_ISL_641264, EPI_ISL_641266, EPI_ISL_641267, EPI_ISL_641268, EPI_ISL_641269, EPI_ISL_641270, EPI_ISL_641271, EPI_ISL_641272, EPI_ISL_641274, EPI_ISL_641275, EPI_ISL_641276, EPI_ISL_641278, EPI_ISL_641280, EPI_ISL_641281, EPI_ISL_641282, EPI_ISL_641283, EPI_ISL_641284, EPI_ISL_641285, EPI_ISL_641286, EPI_ISL_641288, EPI_ISL_641289, EPI_ISL_641290, EPI_ISL_641291, EPI_ISL_641293, EPI_ISL_641294, EPI_ISL_641295, EPI_ISL_641296, EPI_ISL_641297, EPI_ISL_641298, EPI_ISL_641299, EPI_ISL_641300, EPI_ISL_641302, EPI_ISL_641303, EPI_ISL_641304, EPI_ISL_641305, EPI_ISL_641306, EPI_ISL_641307, EPI_ISL_641308			
see above	Microbiological Diagnostic Unit - Public Health Laboratory (MDU-PHL)	MDU-PHL	Seemann T., Schultz M.B., Sait, M.L., Sherry, N.L.
EPI_ISL_641317	Rocky Mountain Laboratories, RTS Genomics Unit, National Institute of Allergy and Infectious Diseases, National Institutes of Health	Rocky Mountain Laboratories, RTS Genomics Unit, National Institute of Allergy and Infectious Diseases, National Institutes of Health	Avanzato,V.A., Matson,M.J., Seifert,S.N., Pryce,R., Williamson,B.N., Anzick,S.L., Barbian,K., Judson,S.D., Fischer,E.R., Martens,C., Bowden,T.A., de Wit,E., Riedo,F.X., Munster,V.J., Siefert,S.N., WIlliamson,B.N., Anzick,S., Martens,C.A.

EPI_ISL_641318	Environmental and Global Health, University of Florida	Environmental and Global Health, University of Florida	Nannu-Shankar,S., Witanachchi,C.T., Alam,M.M., Loeb,J.C., Stephenson,C.J., Fan,Z.H., Wu,C.-Y., Lednicky,J.A.
EPI_ISL_641557, EPI_ISL_641558, EPI_ISL_641559, EPI_ISL_641560, EPI_ISL_641561, EPI_ISL_641562, EPI_ISL_641563, EPI_ISL_641564, EPI_ISL_641565, EPI_ISL_641566, EPI_ISL_641567, EPI_ISL_641568, EPI_ISL_641569, EPI_ISL_641570, EPI_ISL_641571, EPI_ISL_641572, EPI_ISL_641573, EPI_ISL_641574, EPI_ISL_641575, EPI_ISL_641576, EPI_ISL_641577, EPI_ISL_641578, EPI_ISL_641579, EPI_ISL_641580, EPI_ISL_641581, EPI_ISL_641582, EPI_ISL_641583, EPI_ISL_641584, EPI_ISL_641585, EPI_ISL_641586, EPI_ISL_641587, EPI_ISL_641588, EPI_ISL_641589, EPI_ISL_641590, EPI_ISL_641591, EPI_ISL_641592, EPI_ISL_641593, EPI_ISL_641594, EPI_ISL_641595, EPI_ISL_641596, EPI_ISL_641597, EPI_ISL_641598, EPI_ISL_641599, EPI_ISL_641600, EPI_ISL_641601, EPI_ISL_641602, EPI_ISL_641603, EPI_ISL_641604, EPI_ISL_641605, EPI_ISL_641606			
see above	Department of Clinical Microbiology	GIGA Medical Genomics	Keith Durkin, Maria Artesi, Sébastien Bontems, Raphaël Boreux, Bouchra Boujemla, Cécile Meex, Pierrette Melin, Marie-Pierre Hayette, Vincent Bours
EPI_ISL_641607, EPI_ISL_641608, EPI_ISL_641609, EPI_ISL_641610, EPI_ISL_641611, EPI_ISL_641612, EPI_ISL_641613, EPI_ISL_641614, EPI_ISL_641615, EPI_ISL_641616, EPI_ISL_641617, EPI_ISL_641618, EPI_ISL_641619, EPI_ISL_641620, EPI_ISL_641621, EPI_ISL_641622, EPI_ISL_641623, EPI_ISL_641624, EPI_ISL_641625, EPI_ISL_641626, EPI_ISL_641627, EPI_ISL_641628, EPI_ISL_641629, EPI_ISL_641630, EPI_ISL_641631, EPI_ISL_641632, EPI_ISL_641633, EPI_ISL_641634, EPI_ISL_641635, EPI_ISL_641636, EPI_ISL_641637, EPI_ISL_641638, EPI_ISL_641639, EPI_ISL_641640, EPI_ISL_641641, EPI_ISL_641642, EPI_ISL_641643, EPI_ISL_641644, EPI_ISL_641645, EPI_ISL_641646, EPI_ISL_641647, EPI_ISL_641648, EPI_ISL_641649, EPI_ISL_641650, EPI_ISL_641651, EPI_ISL_641652, EPI_ISL_641653, EPI_ISL_641654, EPI_ISL_641655, EPI_ISL_641656, EPI_ISL_641657, EPI_ISL_641658, EPI_ISL_641659, EPI_ISL_641660, EPI_ISL_641661, EPI_ISL_641662, EPI_ISL_641663, EPI_ISL_641664, EPI_ISL_641665, EPI_ISL_641666, EPI_ISL_641667, EPI_ISL_641668, EPI_ISL_641669, EPI_ISL_641670, EPI_ISL_641671, EPI_ISL_641672, EPI_ISL_641673, EPI_ISL_641674, EPI_ISL_641675, EPI_ISL_641676, EPI_ISL_641677, EPI_ISL_641678, EPI_ISL_641679, EPI_ISL_641680, EPI_ISL_641681, EPI_ISL_641682, EPI_ISL_641683, EPI_ISL_641684, EPI_ISL_641685, EPI_ISL_641686, EPI_ISL_641687, EPI_ISL_641688, EPI_ISL_641689, EPI_ISL_641690, EPI_ISL_641691, EPI_ISL_641692, EPI_ISL_641693, EPI_ISL_641694, EPI_ISL_641695, EPI_ISL_641696, EPI_ISL_641697, EPI_ISL_641698, EPI_ISL_641699, EPI_ISL_641700, EPI_ISL_641701, EPI_ISL_641702, EPI_ISL_641703, EPI_ISL_641704, EPI_ISL_641705, EPI_ISL_641706, EPI_ISL_641707, EPI_ISL_641708, EPI_ISL_641709, EPI_ISL_641710, EPI_ISL_641711, EPI_ISL_641712, EPI_ISL_641713, EPI_ISL_641714, EPI_ISL_641715, EPI_ISL_641716, EPI_ISL_641717, EPI_ISL_641718, EPI_ISL_641719, EPI_ISL_641720, EPI_ISL_641721, EPI_ISL_641722, EPI_ISL_641723, EPI_ISL_641724, EPI_ISL_641725, EPI_ISL_641726, EPI_ISL_641727, EPI_ISL_641728, EPI_ISL_641729, EPI_ISL_641730, EPI_ISL_641731, EPI_ISL_641732, EPI_ISL_641733, EPI_ISL_641734, EPI_ISL_641735, EPI_ISL_641736, EPI_ISL_641737, EPI_ISL_641738, EPI_ISL_641739, EPI_ISL_641740, EPI_ISL_641741, EPI_ISL_641742, EPI_ISL_641743, EPI_ISL_641744, EPI_ISL_641745, EPI_ISL_641746, EPI_ISL_641747, EPI_ISL_641748			
see above	Lighthouse Lab in Glasgow	Wellcome Sanger Institute for the COVID-19 Genomics UK (COG-UK) Consortium	Harper VanSteenhouse, Yumi Kasai, David Gray, Carol Clugston, Anna Dominiczak and Alex Alderton, Roberto Amato, Sonia Goncalves, Ewan Harrison, David K. Jackson, Ian Johnston, Dominic Kwiatkowski, Cordelia Langford, John Sillitoe on behalf of the Wellcome Sanger Institute COVID-19 Surveillance Team
