# Supplementary material for: Deepening of In Silico Evaluation of SARS-CoV-2 Detection RT-qPCR Assays in the Context of New Variants
Source: Genes (Basel). 2021 Apr 13;12(4):565. doi: 10.3390/genes12040565 (PMC8069896; doi:10.3390/genes12040565)
Supplement: Supplementary file 1 [file genes-12-00565-s001.zip › Supplementary file S9.zip!/Acknowledgement tables/gisaid_hcov-19_acknowledgement_table_14.pdf]

We gratefully acknowledge the following Authors from the Originating laboratories responsible for obtaining the specimens, as well as the Submitting laboratories where the genome data were generated and shared via GISAID, on which this research is based.

All Submitters of data may be contacted directly via www.gisaid.org

Authors are sorted alphabetically.

Accession ID	Originating Laboratory	Submitting Laboratory	Authors
EPI_ISL_674912	Lighthouse Lab in Milton Keynes	Wellcome Sanger Institute for the COVID-19 Genomics UK (COG-UK) Consortium	The Lighthouse Lab in Milton Keynes and Alex Alderton, Roberto Amato, Sonia Goncalves, Ewan Harrison, David K. Jackson, Ian Johnston, Dominic Kwiatkowski, Cordelia Langford, John Sillitoe on behalf of the Wellcome Sanger Institute COVID-19 Surveillance Team
EPI_ISL_674913	Lighthouse Lab in Cambridge	Wellcome Sanger Institute for the COVID-19 Genomics UK (COG-UK) Consortium	Rob Howes, The Lighthouse Lab in Cambridge and Alex Alderton, Roberto Amato, Sonia Goncalves, Ewan Harrison, David K. Jackson, Ian Johnston, Dominic Kwiatkowski, Cordelia Langford, John Sillitoe on behalf of the Wellcome Sanger Institute COVID-19 Surveillance Team
EPI_ISL_674914, EPI_ISL_674915, EPI_ISL_674916	Lighthouse Lab in Milton Keynes	Wellcome Sanger Institute for the COVID-19 Genomics UK (COG-UK) Consortium	The Lighthouse Lab in Milton Keynes and Alex Alderton, Roberto Amato, Sonia Goncalves, Ewan Harrison, David K. Jackson, Ian Johnston, Dominic Kwiatkowski, Cordelia Langford, John Sillitoe on behalf of the Wellcome Sanger Institute COVID-19 Surveillance Team
EPI_ISL_674917	Lighthouse Lab in Cambridge	Wellcome Sanger Institute for the COVID-19 Genomics UK (COG-UK) Consortium	Rob Howes, The Lighthouse Lab in Cambridge and Alex Alderton, Roberto Amato, Sonia Goncalves, Ewan Harrison, David K. Jackson, Ian Johnston, Dominic Kwiatkowski, Cordelia Langford, John Sillitoe on behalf of the Wellcome Sanger Institute COVID-19 Surveillance Team
EPI_ISL_674918	Lighthouse Lab in Milton Keynes	Wellcome Sanger Institute for the COVID-19 Genomics UK (COG-UK) Consortium	The Lighthouse Lab in Milton Keynes and Alex Alderton, Roberto Amato, Sonia Goncalves, Ewan Harrison, David K. Jackson, Ian Johnston, Dominic Kwiatkowski, Cordelia Langford, John Sillitoe on behalf of the Wellcome Sanger Institute COVID-19 Surveillance Team
EPI_ISL_674919, EPI_ISL_674920	Lighthouse Lab in Cambridge	Wellcome Sanger Institute for the COVID-19 Genomics UK (COG-UK) Consortium	Rob Howes, The Lighthouse Lab in Cambridge and Alex Alderton, Roberto Amato, Sonia Goncalves, Ewan Harrison, David K. Jackson, Ian Johnston, Dominic Kwiatkowski, Cordelia Langford, John Sillitoe on behalf of the Wellcome Sanger Institute COVID-19 Surveillance Team
EPI_ISL_674921	Lighthouse Lab in Milton Keynes	Wellcome Sanger Institute for the COVID-19 Genomics UK (COG-UK) Consortium	The Lighthouse Lab in Milton Keynes and Alex Alderton, Roberto Amato, Sonia Goncalves, Ewan Harrison, David K. Jackson, Ian Johnston, Dominic Kwiatkowski, Cordelia Langford, John Sillitoe on behalf of the Wellcome Sanger Institute COVID-19 Surveillance Team
EPI_ISL_674922, EPI_ISL_674923, EPI_ISL_674924, EPI_ISL_674926	Lighthouse Lab in Cambridge	Wellcome Sanger Institute for the COVID-19 Genomics UK (COG-UK) Consortium	Rob Howes, The Lighthouse Lab in Cambridge and Alex Alderton, Roberto Amato, Sonia Goncalves, Ewan Harrison, David K. Jackson, Ian Johnston, Dominic Kwiatkowski, Cordelia Langford, John Sillitoe on behalf of the Wellcome Sanger Institute COVID-19 Surveillance Team
EPI_ISL_674927, EPI_ISL_674928, EPI_ISL_674929, EPI_ISL_674930, EPI_ISL_674931, EPI_ISL_674932	Lighthouse Lab in Milton Keynes	Wellcome Sanger Institute for the COVID-19 Genomics UK (COG-UK) Consortium	The Lighthouse Lab in Milton Keynes and Alex Alderton, Roberto Amato, Sonia Goncalves, Ewan Harrison, David K. Jackson, Ian Johnston, Dominic Kwiatkowski, Cordelia Langford, John Sillitoe on behalf of the Wellcome Sanger Institute COVID-19 Surveillance Team
EPI_ISL_674933	Lighthouse Lab in Cambridge	Wellcome Sanger Institute for the COVID-19 Genomics UK (COG-UK) Consortium	Rob Howes, The Lighthouse Lab in Cambridge and Alex Alderton, Roberto Amato, Sonia Goncalves, Ewan Harrison, David K. Jackson, Ian Johnston, Dominic Kwiatkowski, Cordelia Langford, John Sillitoe on behalf of the Wellcome Sanger Institute COVID-19 Surveillance Team
EPI_ISL_674934, EPI_ISL_674935	Lighthouse Lab in Milton Keynes	Wellcome Sanger Institute for the COVID-19 Genomics UK (COG-UK) Consortium	The Lighthouse Lab in Milton Keynes and Alex Alderton, Roberto Amato, Sonia Goncalves, Ewan Harrison, David K. Jackson, Ian Johnston, Dominic Kwiatkowski, Cordelia Langford, John Sillitoe on behalf of the Wellcome Sanger Institute COVID-19 Surveillance Team
EPI_ISL_674936	Lighthouse Lab in Cambridge	Wellcome Sanger Institute for the COVID-19 Genomics UK (COG-UK) Consortium	Rob Howes, The Lighthouse Lab in Cambridge and Alex Alderton, Roberto Amato, Sonia Goncalves, Ewan Harrison, David K. Jackson, Ian Johnston, Dominic Kwiatkowski, Cordelia Langford, John Sillitoe on behalf of the Wellcome Sanger Institute COVID-19 Surveillance Team
EPI_ISL_674937, EPI_ISL_674938	Lighthouse Lab in Milton Keynes	Wellcome Sanger Institute for the COVID-19 Genomics UK (COG-UK) Consortium	The Lighthouse Lab in Milton Keynes and Alex Alderton, Roberto Amato, Sonia Goncalves, Ewan Harrison, David K. Jackson, Ian Johnston, Dominic Kwiatkowski, Cordelia Langford, John Sillitoe on behalf of the Wellcome Sanger Institute COVID-19 Surveillance Team
EPI_ISL_674939, EPI_ISL_674940	Lighthouse Lab in Cambridge	Wellcome Sanger Institute for the COVID-19 Genomics UK (COG-UK) Consortium	Rob Howes, The Lighthouse Lab in Cambridge and Alex Alderton, Roberto Amato, Sonia Goncalves, Ewan Harrison, David K. Jackson, Ian Johnston, Dominic Kwiatkowski, Cordelia Langford, John Sillitoe on behalf of the Wellcome Sanger Institute COVID-19 Surveillance Team
EPI_ISL_674941, EPI_ISL_674942, EPI_ISL_674943	Lighthouse Lab in Milton Keynes	Wellcome Sanger Institute for the COVID-19 Genomics UK (COG-UK) Consortium	The Lighthouse Lab in Milton Keynes and Alex Alderton, Roberto Amato, Sonia Goncalves, Ewan Harrison, David K. Jackson, Ian Johnston, Dominic Kwiatkowski, Cordelia Langford, John Sillitoe on behalf of the Wellcome Sanger Institute COVID-19 Surveillance Team
EPI_ISL_674944	Lighthouse Lab in Cambridge	Wellcome Sanger Institute for the COVID-19 Genomics UK (COG-UK) Consortium	Rob Howes, The Lighthouse Lab in Cambridge and Alex Alderton, Roberto Amato, Sonia Goncalves, Ewan Harrison, David K. Jackson, Ian Johnston, Dominic Kwiatkowski, Cordelia Langford, John Sillitoe on behalf of the Wellcome Sanger Institute COVID-19 Surveillance Team
EPI_ISL_674945	Lighthouse Lab in Milton Keynes	Wellcome Sanger Institute for the COVID-19 Genomics UK (COG-UK) Consortium	The Lighthouse Lab in Milton Keynes and Alex Alderton, Roberto Amato, Sonia Goncalves, Ewan Harrison, David K. Jackson, Ian Johnston, Dominic Kwiatkowski, Cordelia Langford, John Sillitoe on behalf of the Wellcome Sanger Institute COVID-19 Surveillance Team
EPI_ISL_674946	Lighthouse Lab in Cambridge	Wellcome Sanger Institute for the COVID-19 Genomics UK (COG-UK) Consortium	Rob Howes, The Lighthouse Lab in Cambridge and Alex Alderton, Roberto Amato, Sonia Goncalves, Ewan Harrison, David K. Jackson, Ian Johnston, Dominic Kwiatkowski, Cordelia Langford, John Sillitoe on behalf of the Wellcome Sanger Institute COVID-19 Surveillance Team
EPI_ISL_674947, EPI_ISL_674948	Lighthouse Lab in Milton Keynes	Wellcome Sanger Institute for the COVID-19 Genomics UK (COG-UK) Consortium	The Lighthouse Lab in Milton Keynes and Alex Alderton, Roberto Amato, Sonia Goncalves, Ewan Harrison, David K. Jackson, Ian Johnston, Dominic Kwiatkowski, Cordelia Langford, John Sillitoe on behalf of the Wellcome Sanger Institute COVID-19 Surveillance Team
EPI_ISL_674949, EPI_ISL_674950, EPI_ISL_674951, EPI_ISL_674952, EPI_ISL_674953	Lighthouse Lab in Cambridge	Wellcome Sanger Institute for the COVID-19 Genomics UK (COG-UK) Consortium	Rob Howes, The Lighthouse Lab in Cambridge and Alex Alderton, Roberto Amato, Sonia Goncalves, Ewan Harrison, David K. Jackson, Ian Johnston, Dominic Kwiatkowski, Cordelia Langford, John Sillitoe on behalf of the Wellcome Sanger Institute COVID-19 Surveillance Team
EPI_ISL_674954, EPI_ISL_674955, EPI_ISL_674956	Lighthouse Lab in Milton Keynes	Wellcome Sanger Institute for the COVID-19 Genomics UK (COG-UK) Consortium	The Lighthouse Lab in Milton Keynes and Alex Alderton, Roberto Amato, Sonia Goncalves, Ewan Harrison, David K. Jackson, Ian Johnston, Dominic Kwiatkowski, Cordelia Langford, John Sillitoe on behalf of the Wellcome Sanger Institute COVID-19 Surveillance Team
EPI_ISL_674957	Lighthouse Lab in Cambridge	Wellcome Sanger Institute for the COVID-19 Genomics UK (COG-UK) Consortium	Rob Howes, The Lighthouse Lab in Cambridge and Alex Alderton, Roberto Amato, Sonia Goncalves, Ewan Harrison, David K. Jackson, Ian Johnston, Dominic Kwiatkowski, Cordelia Langford, John Sillitoe on behalf of the Wellcome Sanger Institute COVID-19 Surveillance Team
EPI_ISL_674958	Lighthouse Lab in Milton Keynes	Wellcome Sanger Institute for the COVID-19 Genomics UK (COG-UK) Consortium	The Lighthouse Lab in Milton Keynes and Alex Alderton, Roberto Amato, Sonia Goncalves, Ewan Harrison, David K. Jackson, Ian Johnston, Dominic Kwiatkowski, Cordelia Langford, John Sillitoe on behalf of the Wellcome Sanger Institute COVID-19 Surveillance Team
EPI_ISL_674959	Lighthouse Lab in Cambridge	Wellcome Sanger Institute for the COVID-19 Genomics UK (COG-UK) Consortium	Rob Howes, The Lighthouse Lab in Cambridge and Alex Alderton, Roberto Amato, Sonia Goncalves, Ewan Harrison, David K. Jackson, Ian Johnston, Dominic Kwiatkowski, Cordelia Langford, John Sillitoe on behalf of the Wellcome Sanger Institute COVID-19 Surveillance Team
EPI_ISL_674960, EPI_ISL_674961	Lighthouse Lab in Milton Keynes	Wellcome Sanger Institute for the COVID-19 Genomics UK (COG-UK) Consortium	The Lighthouse Lab in Milton Keynes and Alex Alderton, Roberto Amato, Sonia Goncalves, Ewan Harrison, David K. Jackson, Ian Johnston, Dominic Kwiatkowski, Cordelia Langford, John Sillitoe on behalf of the Wellcome Sanger Institute COVID-19 Surveillance Team
EPI_ISL_674962	Lighthouse Lab in Cambridge	Wellcome Sanger Institute for the COVID-19 Genomics UK (COG-UK) Consortium	Rob Howes, The Lighthouse Lab in Cambridge and Alex Alderton, Roberto Amato, Sonia Goncalves, Ewan Harrison, David K. Jackson, Ian Johnston, Dominic Kwiatkowski, Cordelia Langford, John Sillitoe on behalf of the Wellcome Sanger Institute COVID-19 Surveillance Team
EPI_ISL_674963, EPI_ISL_674964, EPI_ISL_674965, EPI_ISL_674966, EPI_ISL_674967, EPI_ISL_674968, EPI_ISL_674969, EPI_ISL_674970, EPI_ISL_674971	Lighthouse Lab in Milton Keynes	Wellcome Sanger Institute for the COVID-19 Genomics UK (COG-UK) Consortium	The Lighthouse Lab in Milton Keynes and Alex Alderton, Roberto Amato, Sonia Goncalves, Ewan Harrison, David K. Jackson, Ian Johnston, Dominic Kwiatkowski, Cordelia Langford, John Sillitoe on behalf of the Wellcome Sanger Institute COVID-19 Surveillance Team
EPI_ISL_674972, EPI_ISL_674973	Lighthouse Lab in Cambridge	Wellcome Sanger Institute for the COVID-19 Genomics UK (COG-UK) Consortium	Rob Howes, The Lighthouse Lab in Cambridge and Alex Alderton, Roberto Amato, Sonia Goncalves, Ewan Harrison, David K. Jackson, Ian Johnston, Dominic Kwiatkowski, Cordelia Langford, John Sillitoe on behalf of the Wellcome Sanger Institute COVID-19 Surveillance Team

EPI_ISL_674974, EPI_ISL_674975, EPI_ISL_674976	Lighthouse Lab in Milton Keynes	Wellcome Sanger Institute for the COVID-19 Genomics UK (COG-UK) Consortium	The Lighthouse Lab in Milton Keynes and Alex Alderton, Roberto Amato, Sonia Goncalves, Ewan Harrison, David K. Jackson, Ian Johnston, Dominic Kwiatkowski, Cordelia Langford, John Sillitoe on behalf of the Wellcome Sanger Institute COVID-19 Surveillance Team
EPI_ISL_674977	Lighthouse Lab in Cambridge	Wellcome Sanger Institute for the COVID-19 Genomics UK (COG-UK) Consortium	Rob Howes, The Lighthouse Lab in Cambridge and Alex Alderton, Roberto Amato, Sonia Goncalves, Ewan Harrison, David K. Jackson, Ian Johnston, Dominic Kwiatkowski, Cordelia Langford, John Sillitoe on behalf of the Wellcome Sanger Institute COVID-19 Surveillance Team
EPI_ISL_674978, EPI_ISL_674979, EPI_ISL_674980, EPI_ISL_674981, EPI_ISL_674982	Lighthouse Lab in Milton Keynes	Wellcome Sanger Institute for the COVID-19 Genomics UK (COG-UK) Consortium	The Lighthouse Lab in Milton Keynes and Alex Alderton, Roberto Amato, Sonia Goncalves, Ewan Harrison, David K. Jackson, Ian Johnston, Dominic Kwiatkowski, Cordelia Langford, John Sillitoe on behalf of the Wellcome Sanger Institute COVID-19 Surveillance Team
EPI_ISL_674983	Lighthouse Lab in Cambridge	Wellcome Sanger Institute for the COVID-19 Genomics UK (COG-UK) Consortium	Rob Howes, The Lighthouse Lab in Cambridge and Alex Alderton, Roberto Amato, Sonia Goncalves, Ewan Harrison, David K. Jackson, Ian Johnston, Dominic Kwiatkowski, Cordelia Langford, John Sillitoe on behalf of the Wellcome Sanger Institute COVID-19 Surveillance Team
EPI_ISL_674984, EPI_ISL_674985, EPI_ISL_674986, EPI_ISL_674987	Lighthouse Lab in Milton Keynes	Wellcome Sanger Institute for the COVID-19 Genomics UK (COG-UK) Consortium	The Lighthouse Lab in Milton Keynes and Alex Alderton, Roberto Amato, Sonia Goncalves, Ewan Harrison, David K. Jackson, Ian Johnston, Dominic Kwiatkowski, Cordelia Langford, John Sillitoe on behalf of the Wellcome Sanger Institute COVID-19 Surveillance Team
EPI_ISL_674988	Lighthouse Lab in Cambridge	Wellcome Sanger Institute for the COVID-19 Genomics UK (COG-UK) Consortium	Rob Howes, The Lighthouse Lab in Cambridge and Alex Alderton, Roberto Amato, Sonia Goncalves, Ewan Harrison, David K. Jackson, Ian Johnston, Dominic Kwiatkowski, Cordelia Langford, John Sillitoe on behalf of the Wellcome Sanger Institute COVID-19 Surveillance Team
EPI_ISL_674989, EPI_ISL_674990, EPI_ISL_674991	Lighthouse Lab in Milton Keynes	Wellcome Sanger Institute for the COVID-19 Genomics UK (COG-UK) Consortium	The Lighthouse Lab in Milton Keynes and Alex Alderton, Roberto Amato, Sonia Goncalves, Ewan Harrison, David K. Jackson, Ian Johnston, Dominic Kwiatkowski, Cordelia Langford, John Sillitoe on behalf of the Wellcome Sanger Institute COVID-19 Surveillance Team
EPI_ISL_674992	Lighthouse Lab in Cambridge	Wellcome Sanger Institute for the COVID-19 Genomics UK (COG-UK) Consortium	Rob Howes, The Lighthouse Lab in Cambridge and Alex Alderton, Roberto Amato, Sonia Goncalves, Ewan Harrison, David K. Jackson, Ian Johnston, Dominic Kwiatkowski, Cordelia Langford, John Sillitoe on behalf of the Wellcome Sanger Institute COVID-19 Surveillance Team
EPI_ISL_674993, EPI_ISL_674994	Lighthouse Lab in Milton Keynes	Wellcome Sanger Institute for the COVID-19 Genomics UK (COG-UK) Consortium	The Lighthouse Lab in Milton Keynes and Alex Alderton, Roberto Amato, Sonia Goncalves, Ewan Harrison, David K. Jackson, Ian Johnston, Dominic Kwiatkowski, Cordelia Langford, John Sillitoe on behalf of the Wellcome Sanger Institute COVID-19 Surveillance Team
EPI_ISL_674995	Lighthouse Lab in Cambridge	Wellcome Sanger Institute for the COVID-19 Genomics UK (COG-UK) Consortium	Rob Howes, The Lighthouse Lab in Cambridge and Alex Alderton, Roberto Amato, Sonia Goncalves, Ewan Harrison, David K. Jackson, Ian Johnston, Dominic Kwiatkowski, Cordelia Langford, John Sillitoe on behalf of the Wellcome Sanger Institute COVID-19 Surveillance Team
EPI_ISL_674996, EPI_ISL_674997	Lighthouse Lab in Milton Keynes	Wellcome Sanger Institute for the COVID-19 Genomics UK (COG-UK) Consortium	The Lighthouse Lab in Milton Keynes and Alex Alderton, Roberto Amato, Sonia Goncalves, Ewan Harrison, David K. Jackson, Ian Johnston, Dominic Kwiatkowski, Cordelia Langford, John Sillitoe on behalf of the Wellcome Sanger Institute COVID-19 Surveillance Team
EPI_ISL_674998	Lighthouse Lab in Cambridge	Wellcome Sanger Institute for the COVID-19 Genomics UK (COG-UK) Consortium	Rob Howes, The Lighthouse Lab in Cambridge and Alex Alderton, Roberto Amato, Sonia Goncalves, Ewan Harrison, David K. Jackson, Ian Johnston, Dominic Kwiatkowski, Cordelia Langford, John Sillitoe on behalf of the Wellcome Sanger Institute COVID-19 Surveillance Team
EPI_ISL_674999, EPI_ISL_675000, EPI_ISL_675001, EPI_ISL_675002, EPI_ISL_675003, EPI_ISL_675004, EPI_ISL_675005	Lighthouse Lab in Milton Keynes	Wellcome Sanger Institute for the COVID-19 Genomics UK (COG-UK) Consortium	The Lighthouse Lab in Milton Keynes and Alex Alderton, Roberto Amato, Sonia Goncalves, Ewan Harrison, David K. Jackson, Ian Johnston, Dominic Kwiatkowski, Cordelia Langford, John Sillitoe on behalf of the Wellcome Sanger Institute COVID-19 Surveillance Team
EPI_ISL_675006	Lighthouse Lab in Cambridge	Wellcome Sanger Institute for the COVID-19 Genomics UK (COG-UK) Consortium	Rob Howes, The Lighthouse Lab in Cambridge and Alex Alderton, Roberto Amato, Sonia Goncalves, Ewan Harrison, David K. Jackson, Ian Johnston, Dominic Kwiatkowski, Cordelia Langford, John Sillitoe on behalf of the Wellcome Sanger Institute COVID-19 Surveillance Team
EPI_ISL_675007	Lighthouse Lab in Milton Keynes	Wellcome Sanger Institute for the COVID-19 Genomics UK (COG-UK) Consortium	The Lighthouse Lab in Milton Keynes and Alex Alderton, Roberto Amato, Sonia Goncalves, Ewan Harrison, David K. Jackson, Ian Johnston, Dominic Kwiatkowski, Cordelia Langford, John Sillitoe on behalf of the Wellcome Sanger Institute COVID-19 Surveillance Team
EPI_ISL_675008	Lighthouse Lab in Cambridge	Wellcome Sanger Institute for the COVID-19 Genomics UK (COG-UK) Consortium	Rob Howes, The Lighthouse Lab in Cambridge and Alex Alderton, Roberto Amato, Sonia Goncalves, Ewan Harrison, David K. Jackson, Ian Johnston, Dominic Kwiatkowski, Cordelia Langford, John Sillitoe on behalf of the Wellcome Sanger Institute COVID-19 Surveillance Team
EPI_ISL_675010, EPI_ISL_675011	Lighthouse Lab in Milton Keynes	Wellcome Sanger Institute for the COVID-19 Genomics UK (COG-UK) Consortium	The Lighthouse Lab in Milton Keynes and Alex Alderton, Roberto Amato, Sonia Goncalves, Ewan Harrison, David K. Jackson, Ian Johnston, Dominic Kwiatkowski, Cordelia Langford, John Sillitoe on behalf of the Wellcome Sanger Institute COVID-19 Surveillance Team
EPI_ISL_675012	Lighthouse Lab in Cambridge	Wellcome Sanger Institute for the COVID-19 Genomics UK (COG-UK) Consortium	Rob Howes, The Lighthouse Lab in Cambridge and Alex Alderton, Roberto Amato, Sonia Goncalves, Ewan Harrison, David K. Jackson, Ian Johnston, Dominic Kwiatkowski, Cordelia Langford, John Sillitoe on behalf of the Wellcome Sanger Institute COVID-19 Surveillance Team
EPI_ISL_675013, EPI_ISL_675014, EPI_ISL_675015, EPI_ISL_675016, EPI_ISL_675017	Lighthouse Lab in Milton Keynes	Wellcome Sanger Institute for the COVID-19 Genomics UK (COG-UK) Consortium	The Lighthouse Lab in Milton Keynes and Alex Alderton, Roberto Amato, Sonia Goncalves, Ewan Harrison, David K. Jackson, Ian Johnston, Dominic Kwiatkowski, Cordelia Langford, John Sillitoe on behalf of the Wellcome Sanger Institute COVID-19 Surveillance Team
EPI_ISL_675018	Lighthouse Lab in Cambridge	Wellcome Sanger Institute for the COVID-19 Genomics UK (COG-UK) Consortium	Rob Howes, The Lighthouse Lab in Cambridge and Alex Alderton, Roberto Amato, Sonia Goncalves, Ewan Harrison, David K. Jackson, Ian Johnston, Dominic Kwiatkowski, Cordelia Langford, John Sillitoe on behalf of the Wellcome Sanger Institute COVID-19 Surveillance Team
EPI_ISL_675019, EPI_ISL_675020, EPI_ISL_675021, EPI_ISL_675022, EPI_ISL_675023	Lighthouse Lab in Milton Keynes	Wellcome Sanger Institute for the COVID-19 Genomics UK (COG-UK) Consortium	The Lighthouse Lab in Milton Keynes and Alex Alderton, Roberto Amato, Sonia Goncalves, Ewan Harrison, David K. Jackson, Ian Johnston, Dominic Kwiatkowski, Cordelia Langford, John Sillitoe on behalf of the Wellcome Sanger Institute COVID-19 Surveillance Team
EPI_ISL_675024	Lighthouse Lab in Cambridge	Wellcome Sanger Institute for the COVID-19 Genomics UK (COG-UK) Consortium	Rob Howes, The Lighthouse Lab in Cambridge and Alex Alderton, Roberto Amato, Sonia Goncalves, Ewan Harrison, David K. Jackson, Ian Johnston, Dominic Kwiatkowski, Cordelia Langford, John Sillitoe on behalf of the Wellcome Sanger Institute COVID-19 Surveillance Team
EPI_ISL_675025	Lighthouse Lab in Milton Keynes	Wellcome Sanger Institute for the COVID-19 Genomics UK (COG-UK) Consortium	The Lighthouse Lab in Milton Keynes and Alex Alderton, Roberto Amato, Sonia Goncalves, Ewan Harrison, David K. Jackson, Ian Johnston, Dominic Kwiatkowski, Cordelia Langford, John Sillitoe on behalf of the Wellcome Sanger Institute COVID-19 Surveillance Team
EPI_ISL_675026, EPI_ISL_675027	Lighthouse Lab in Cambridge	Wellcome Sanger Institute for the COVID-19 Genomics UK (COG-UK) Consortium	Rob Howes, The Lighthouse Lab in Cambridge and Alex Alderton, Roberto Amato, Sonia Goncalves, Ewan Harrison, David K. Jackson, Ian Johnston, Dominic Kwiatkowski, Cordelia Langford, John Sillitoe on behalf of the Wellcome Sanger Institute COVID-19 Surveillance Team
EPI_ISL_675028, EPI_ISL_675029, EPI_ISL_675030, EPI_ISL_675031, EPI_ISL_675032	Lighthouse Lab in Milton Keynes	Wellcome Sanger Institute for the COVID-19 Genomics UK (COG-UK) Consortium	The Lighthouse Lab in Milton Keynes and Alex Alderton, Roberto Amato, Sonia Goncalves, Ewan Harrison, David K. Jackson, Ian Johnston, Dominic Kwiatkowski, Cordelia Langford, John Sillitoe on behalf of the Wellcome Sanger Institute COVID-19 Surveillance Team
EPI_ISL_675033	Lighthouse Lab in Cambridge	Wellcome Sanger Institute for the COVID-19 Genomics UK (COG-UK) Consortium	Rob Howes, The Lighthouse Lab in Cambridge and Alex Alderton, Roberto Amato, Sonia Goncalves, Ewan Harrison, David K. Jackson, Ian Johnston, Dominic Kwiatkowski, Cordelia Langford, John Sillitoe on behalf of the Wellcome Sanger Institute COVID-19 Surveillance Team
EPI_ISL_675034, EPI_ISL_675035, EPI_ISL_675036, EPI_ISL_675037	Lighthouse Lab in Milton Keynes	Wellcome Sanger Institute for the COVID-19 Genomics UK (COG-UK) Consortium	The Lighthouse Lab in Milton Keynes and Alex Alderton, Roberto Amato, Sonia Goncalves, Ewan Harrison, David K. Jackson, Ian Johnston, Dominic Kwiatkowski, Cordelia Langford, John Sillitoe on behalf of the Wellcome Sanger Institute COVID-19 Surveillance Team
EPI_ISL_675038, EPI_ISL_675039, EPI_ISL_675040	Lighthouse Lab in Cambridge	Wellcome Sanger Institute for the COVID-19 Genomics UK (COG-UK) Consortium	Rob Howes, The Lighthouse Lab in Cambridge and Alex Alderton, Roberto Amato, Sonia Goncalves, Ewan Harrison, David K. Jackson, Ian Johnston, Dominic Kwiatkowski, Cordelia Langford, John Sillitoe on behalf of the Wellcome Sanger Institute COVID-19 Surveillance Team
EPI_ISL_675041	Lighthouse Lab in Milton Keynes	Wellcome Sanger Institute for the COVID-19 Genomics UK (COG-UK) Consortium	The Lighthouse Lab in Milton Keynes and Alex Alderton, Roberto Amato, Sonia Goncalves, Ewan Harrison, David K. Jackson, Ian Johnston, Dominic Kwiatkowski, Cordelia Langford, John Sillitoe on behalf of the Wellcome Sanger Institute COVID-19 Surveillance Team
EPI_ISL_675042	Lighthouse Lab in Cambridge	Wellcome Sanger Institute for the COVID-19 Genomics UK (COG-UK) Consortium	Rob Howes, The Lighthouse Lab in Cambridge and Alex Alderton, Roberto Amato, Sonia Goncalves, Ewan Harrison, David K. Jackson, Ian Johnston, Dominic Kwiatkowski, Cordelia Langford, John Sillitoe on behalf of the Wellcome Sanger Institute COVID-19 Surveillance Team
EPI_ISL_675043, EPI_ISL_675044, EPI_ISL_675045	Lighthouse Lab in Milton Keynes	Wellcome Sanger Institute for the COVID-19 Genomics UK (COG-UK) Consortium	The Lighthouse Lab in Milton Keynes and Alex Alderton, Roberto Amato, Sonia Goncalves, Ewan Harrison, David K. Jackson, Ian Johnston, Dominic Kwiatkowski, Cordelia Langford, John Sillitoe on behalf of the Wellcome Sanger Institute COVID-19 Surveillance Team
EPI_ISL_675046	Lighthouse Lab in Cambridge	Wellcome Sanger Institute for the COVID-19 Genomics UK (COG-UK) Consortium	Rob Howes, The Lighthouse Lab in Cambridge and Alex Alderton, Roberto Amato, Sonia Goncalves, Ewan Harrison, David K. Jackson, Ian Johnston, Dominic Kwiatkowski, Cordelia Langford, John Sillitoe on behalf of the Wellcome Sanger Institute COVID-19 Surveillance Team
EPI_ISL_675047, EPI_ISL_675048, EPI_ISL_675049, EPI_ISL_675050,	Lighthouse Lab in Milton Keynes	Wellcome Sanger Institute for the COVID-19 Genomics UK (COG-UK) Consortium	The Lighthouse Lab in Milton Keynes and Alex Alderton, Roberto Amato, Sonia Goncalves, Ewan Harrison, David K. Jackson, Ian Johnston, Dominic Kwiatkowski, Cordelia Langford, John Sillitoe on behalf of the Wellcome Sanger Institute COVID-19 Surveillance Team

EPI_ISL_675051, EPI_ISL_675052			
EPI_ISL_675053	Lighthouse Lab in Cambridge	Wellcome Sanger Institute for the COVID-19 Genomics UK (COG-UK) Consortium	Rob Howes, The Lighthouse Lab in Cambridge and Alex Alderton, Roberto Amato, Sonia Goncalves, Ewan Harrison, David K. Jackson, Ian Johnston, Dominic Kwiatkowski, Cordelia Langford, John Sillitoe on behalf of the Wellcome Sanger Institute COVID-19 Surveillance Team
EPI_ISL_675054, EPI_ISL_675055	Lighthouse Lab in Milton Keynes	Wellcome Sanger Institute for the COVID-19 Genomics UK (COG-UK) Consortium	The Lighthouse Lab in Milton Keynes and Alex Alderton, Roberto Amato, Sonia Goncalves, Ewan Harrison, David K. Jackson, Ian Johnston, Dominic Kwiatkowski, Cordelia Langford, John Sillitoe on behalf of the Wellcome Sanger Institute COVID-19 Surveillance Team
EPI_ISL_675056	Lighthouse Lab in Cambridge	Wellcome Sanger Institute for the COVID-19 Genomics UK (COG-UK) Consortium	Rob Howes, The Lighthouse Lab in Cambridge and Alex Alderton, Roberto Amato, Sonia Goncalves, Ewan Harrison, David K. Jackson, Ian Johnston, Dominic Kwiatkowski, Cordelia Langford, John Sillitoe on behalf of the Wellcome Sanger Institute COVID-19 Surveillance Team
EPI_ISL_675057, EPI_ISL_675058, EPI_ISL_675059, EPI_ISL_675060, EPI_ISL_675061, EPI_ISL_675062, EPI_ISL_675063	Lighthouse Lab in Milton Keynes	Wellcome Sanger Institute for the COVID-19 Genomics UK (COG-UK) Consortium	The Lighthouse Lab in Milton Keynes and Alex Alderton, Roberto Amato, Sonia Goncalves, Ewan Harrison, David K. Jackson, Ian Johnston, Dominic Kwiatkowski, Cordelia Langford, John Sillitoe on behalf of the Wellcome Sanger Institute COVID-19 Surveillance Team
EPI_ISL_675064	Lighthouse Lab in Cambridge	Wellcome Sanger Institute for the COVID-19 Genomics UK (COG-UK) Consortium	Rob Howes, The Lighthouse Lab in Cambridge and Alex Alderton, Roberto Amato, Sonia Goncalves, Ewan Harrison, David K. Jackson, Ian Johnston, Dominic Kwiatkowski, Cordelia Langford, John Sillitoe on behalf of the Wellcome Sanger Institute COVID-19 Surveillance Team
EPI_ISL_675065, EPI_ISL_675066, EPI_ISL_675067, EPI_ISL_675068, EPI_ISL_675069, EPI_ISL_675070	Lighthouse Lab in Milton Keynes	Wellcome Sanger Institute for the COVID-19 Genomics UK (COG-UK) Consortium	The Lighthouse Lab in Milton Keynes and Alex Alderton, Roberto Amato, Sonia Goncalves, Ewan Harrison, David K. Jackson, Ian Johnston, Dominic Kwiatkowski, Cordelia Langford, John Sillitoe on behalf of the Wellcome Sanger Institute COVID-19 Surveillance Team
EPI_ISL_675071	Lighthouse Lab in Cambridge	Wellcome Sanger Institute for the COVID-19 Genomics UK (COG-UK) Consortium	Rob Howes, The Lighthouse Lab in Cambridge and Alex Alderton, Roberto Amato, Sonia Goncalves, Ewan Harrison, David K. Jackson, Ian Johnston, Dominic Kwiatkowski, Cordelia Langford, John Sillitoe on behalf of the Wellcome Sanger Institute COVID-19 Surveillance Team
EPI_ISL_675072, EPI_ISL_675074	Lighthouse Lab in Milton Keynes	Wellcome Sanger Institute for the COVID-19 Genomics UK (COG-UK) Consortium	The Lighthouse Lab in Milton Keynes and Alex Alderton, Roberto Amato, Sonia Goncalves, Ewan Harrison, David K. Jackson, Ian Johnston, Dominic Kwiatkowski, Cordelia Langford, John Sillitoe on behalf of the Wellcome Sanger Institute COVID-19 Surveillance Team
EPI_ISL_675075	Lighthouse Lab in Cambridge	Wellcome Sanger Institute for the COVID-19 Genomics UK (COG-UK) Consortium	Rob Howes, The Lighthouse Lab in Cambridge and Alex Alderton, Roberto Amato, Sonia Goncalves, Ewan Harrison, David K. Jackson, Ian Johnston, Dominic Kwiatkowski, Cordelia Langford, John Sillitoe on behalf of the Wellcome Sanger Institute COVID-19 Surveillance Team
EPI_ISL_675076, EPI_ISL_675077, EPI_ISL_675078, EPI_ISL_675079	Lighthouse Lab in Milton Keynes	Wellcome Sanger Institute for the COVID-19 Genomics UK (COG-UK) Consortium	The Lighthouse Lab in Milton Keynes and Alex Alderton, Roberto Amato, Sonia Goncalves, Ewan Harrison, David K. Jackson, Ian Johnston, Dominic Kwiatkowski, Cordelia Langford, John Sillitoe on behalf of the Wellcome Sanger Institute COVID-19 Surveillance Team
EPI_ISL_675080	Lighthouse Lab in Cambridge	Wellcome Sanger Institute for the COVID-19 Genomics UK (COG-UK) Consortium	Rob Howes, The Lighthouse Lab in Cambridge and Alex Alderton, Roberto Amato, Sonia Goncalves, Ewan Harrison, David K. Jackson, Ian Johnston, Dominic Kwiatkowski, Cordelia Langford, John Sillitoe on behalf of the Wellcome Sanger Institute COVID-19 Surveillance Team
EPI_ISL_675081	Lighthouse Lab in Milton Keynes	Wellcome Sanger Institute for the COVID-19 Genomics UK (COG-UK) Consortium	The Lighthouse Lab in Milton Keynes and Alex Alderton, Roberto Amato, Sonia Goncalves, Ewan Harrison, David K. Jackson, Ian Johnston, Dominic Kwiatkowski, Cordelia Langford, John Sillitoe on behalf of the Wellcome Sanger Institute COVID-19 Surveillance Team
EPI_ISL_675082	Lighthouse Lab in Cambridge	Wellcome Sanger Institute for the COVID-19 Genomics UK (COG-UK) Consortium	Rob Howes, The Lighthouse Lab in Cambridge and Alex Alderton, Roberto Amato, Sonia Goncalves, Ewan Harrison, David K. Jackson, Ian Johnston, Dominic Kwiatkowski, Cordelia Langford, John Sillitoe on behalf of the Wellcome Sanger Institute COVID-19 Surveillance Team
EPI_ISL_675083, EPI_ISL_675084, EPI_ISL_675085, EPI_ISL_675086	Lighthouse Lab in Milton Keynes	Wellcome Sanger Institute for the COVID-19 Genomics UK (COG-UK) Consortium	The Lighthouse Lab in Milton Keynes and Alex Alderton, Roberto Amato, Sonia Goncalves, Ewan Harrison, David K. Jackson, Ian Johnston, Dominic Kwiatkowski, Cordelia Langford, John Sillitoe on behalf of the Wellcome Sanger Institute COVID-19 Surveillance Team
EPI_ISL_675087	Lighthouse Lab in Cambridge	Wellcome Sanger Institute for the COVID-19 Genomics UK (COG-UK) Consortium	Rob Howes, The Lighthouse Lab in Cambridge and Alex Alderton, Roberto Amato, Sonia Goncalves, Ewan Harrison, David K. Jackson, Ian Johnston, Dominic Kwiatkowski, Cordelia Langford, John Sillitoe on behalf of the Wellcome Sanger Institute COVID-19 Surveillance Team
EPI_ISL_675088, EPI_ISL_675089, EPI_ISL_675090, EPI_ISL_675091, EPI_ISL_675092, EPI_ISL_675093	Lighthouse Lab in Milton Keynes	Wellcome Sanger Institute for the COVID-19 Genomics UK (COG-UK) Consortium	The Lighthouse Lab in Milton Keynes and Alex Alderton, Roberto Amato, Sonia Goncalves, Ewan Harrison, David K. Jackson, Ian Johnston, Dominic Kwiatkowski, Cordelia Langford, John Sillitoe on behalf of the Wellcome Sanger Institute COVID-19 Surveillance Team
EPI_ISL_675094	Lighthouse Lab in Cambridge	Wellcome Sanger Institute for the COVID-19 Genomics UK (COG-UK) Consortium	Rob Howes, The Lighthouse Lab in Cambridge and Alex Alderton, Roberto Amato, Sonia Goncalves, Ewan Harrison, David K. Jackson, Ian Johnston, Dominic Kwiatkowski, Cordelia Langford, John Sillitoe on behalf of the Wellcome Sanger Institute COVID-19 Surveillance Team
EPI_ISL_675095, EPI_ISL_675096, EPI_ISL_675097, EPI_ISL_675098, EPI_ISL_675099, EPI_ISL_675100, EPI_ISL_675101, EPI_ISL_675102, EPI_ISL_675103	Lighthouse Lab in Milton Keynes	Wellcome Sanger Institute for the COVID-19 Genomics UK (COG-UK) Consortium	The Lighthouse Lab in Milton Keynes and Alex Alderton, Roberto Amato, Sonia Goncalves, Ewan Harrison, David K. Jackson, Ian Johnston, Dominic Kwiatkowski, Cordelia Langford, John Sillitoe on behalf of the Wellcome Sanger Institute COVID-19 Surveillance Team
EPI_ISL_675104	Lighthouse Lab in Cambridge	Wellcome Sanger Institute for the COVID-19 Genomics UK (COG-UK) Consortium	Rob Howes, The Lighthouse Lab in Cambridge and Alex Alderton, Roberto Amato, Sonia Goncalves, Ewan Harrison, David K. Jackson, Ian Johnston, Dominic Kwiatkowski, Cordelia Langford, John Sillitoe on behalf of the Wellcome Sanger Institute COVID-19 Surveillance Team
EPI_ISL_675105, EPI_ISL_675106	Lighthouse Lab in Milton Keynes	Wellcome Sanger Institute for the COVID-19 Genomics UK (COG-UK) Consortium	The Lighthouse Lab in Milton Keynes and Alex Alderton, Roberto Amato, Sonia Goncalves, Ewan Harrison, David K. Jackson, Ian Johnston, Dominic Kwiatkowski, Cordelia Langford, John Sillitoe on behalf of the Wellcome Sanger Institute COVID-19 Surveillance Team
EPI_ISL_675107	Lighthouse Lab in Cambridge	Wellcome Sanger Institute for the COVID-19 Genomics UK (COG-UK) Consortium	Rob Howes, The Lighthouse Lab in Cambridge and Alex Alderton, Roberto Amato, Sonia Goncalves, Ewan Harrison, David K. Jackson, Ian Johnston, Dominic Kwiatkowski, Cordelia Langford, John Sillitoe on behalf of the Wellcome Sanger Institute COVID-19 Surveillance Team
EPI_ISL_675108, EPI_ISL_675109	Lighthouse Lab in Milton Keynes	Wellcome Sanger Institute for the COVID-19 Genomics UK (COG-UK) Consortium	The Lighthouse Lab in Milton Keynes and Alex Alderton, Roberto Amato, Sonia Goncalves, Ewan Harrison, David K. Jackson, Ian Johnston, Dominic Kwiatkowski, Cordelia Langford, John Sillitoe on behalf of the Wellcome Sanger Institute COVID-19 Surveillance Team
EPI_ISL_675110	Lighthouse Lab in Cambridge	Wellcome Sanger Institute for the COVID-19 Genomics UK (COG-UK) Consortium	Rob Howes, The Lighthouse Lab in Cambridge and Alex Alderton, Roberto Amato, Sonia Goncalves, Ewan Harrison, David K. Jackson, Ian Johnston, Dominic Kwiatkowski, Cordelia Langford, John Sillitoe on behalf of the Wellcome Sanger Institute COVID-19 Surveillance Team
EPI_ISL_675111, EPI_ISL_675113, EPI_ISL_675114, EPI_ISL_675115, EPI_ISL_675116	Lighthouse Lab in Milton Keynes	Wellcome Sanger Institute for the COVID-19 Genomics UK (COG-UK) Consortium	The Lighthouse Lab in Milton Keynes and Alex Alderton, Roberto Amato, Sonia Goncalves, Ewan Harrison, David K. Jackson, Ian Johnston, Dominic Kwiatkowski, Cordelia Langford, John Sillitoe on behalf of the Wellcome Sanger Institute COVID-19 Surveillance Team
EPI_ISL_675117	Lighthouse Lab in Cambridge	Wellcome Sanger Institute for the COVID-19 Genomics UK (COG-UK) Consortium	Rob Howes, The Lighthouse Lab in Cambridge and Alex Alderton, Roberto Amato, Sonia Goncalves, Ewan Harrison, David K. Jackson, Ian Johnston, Dominic Kwiatkowski, Cordelia Langford, John Sillitoe on behalf of the Wellcome Sanger Institute COVID-19 Surveillance Team
EPI_ISL_675119	Lighthouse Lab in Milton Keynes	Wellcome Sanger Institute for the COVID-19 Genomics UK (COG-UK) Consortium	The Lighthouse Lab in Milton Keynes and Alex Alderton, Roberto Amato, Sonia Goncalves, Ewan Harrison, David K. Jackson, Ian Johnston, Dominic Kwiatkowski, Cordelia Langford, John Sillitoe on behalf of the Wellcome Sanger Institute COVID-19 Surveillance Team
EPI_ISL_675120	Lighthouse Lab in Cambridge	Wellcome Sanger Institute for the COVID-19 Genomics UK (COG-UK) Consortium	Rob Howes, The Lighthouse Lab in Cambridge and Alex Alderton, Roberto Amato, Sonia Goncalves, Ewan Harrison, David K. Jackson, Ian Johnston, Dominic Kwiatkowski, Cordelia Langford, John Sillitoe on behalf of the Wellcome Sanger Institute COVID-19 Surveillance Team
EPI_ISL_675121, EPI_ISL_675122, EPI_ISL_675123, EPI_ISL_675124, EPI_ISL_675125	Lighthouse Lab in Milton Keynes	Wellcome Sanger Institute for the COVID-19 Genomics UK (COG-UK) Consortium	The Lighthouse Lab in Milton Keynes and Alex Alderton, Roberto Amato, Sonia Goncalves, Ewan Harrison, David K. Jackson, Ian Johnston, Dominic Kwiatkowski, Cordelia Langford, John Sillitoe on behalf of the Wellcome Sanger Institute COVID-19 Surveillance Team
EPI_ISL_675126	Lighthouse Lab in Cambridge	Wellcome Sanger Institute for the COVID-19 Genomics UK (COG-UK) Consortium	Rob Howes, The Lighthouse Lab in Cambridge and Alex Alderton, Roberto Amato, Sonia Goncalves, Ewan Harrison, David K. Jackson, Ian Johnston, Dominic Kwiatkowski, Cordelia Langford, John Sillitoe on behalf of the Wellcome Sanger Institute COVID-19 Surveillance Team
EPI_ISL_675127, EPI_ISL_675128, EPI_ISL_675129	Lighthouse Lab in Milton Keynes	Wellcome Sanger Institute for the COVID-19 Genomics UK (COG-UK) Consortium	The Lighthouse Lab in Milton Keynes and Alex Alderton, Roberto Amato, Sonia Goncalves, Ewan Harrison, David K. Jackson, Ian Johnston, Dominic Kwiatkowski, Cordelia Langford, John Sillitoe on behalf of the Wellcome Sanger Institute COVID-19 Surveillance Team
EPI_ISL_675130	Lighthouse Lab in Alderley Park	Wellcome Sanger Institute for the COVID-19 Genomics UK (COG-UK) Consortium	Jacquelyn Wynn, Mairead Hyland, The Lighthouse Lab in Alderley Park and Alex Alderton, Roberto Amato, Sonia Goncalves, Ewan Harrison, David K. Jackson, Ian Johnston, Dominic Kwiatkowski, Cordelia Langford, John Sillitoe on behalf of the Wellcome Sanger Institute COVID-19 Surveillance Team

EPI_ISL_675131, EPI_ISL_675132	Lighthouse Lab in Milton Keynes	Wellcome Sanger Institute for the COVID-19 Genomics UK (COG-UK) Consortium	The Lighthouse Lab in Milton Keynes and Alex Alderton, Roberto Amato, Sonia Goncalves, Ewan Harrison, David K. Jackson, Ian Johnston, Dominic Kwiatkowski, Cordelia Langford, John Sillitoe on behalf of the Wellcome Sanger Institute COVID-19 Surveillance Team
EPI_ISL_675133	Lighthouse Lab in Alderley Park	Wellcome Sanger Institute for the COVID-19 Genomics UK (COG-UK) Consortium	Jacquelyn Wynn, Mairead Hyland, The Lighthouse Lab in Alderley Park and Alex Alderton, Roberto Amato, Sonia Goncalves, Ewan Harrison, David K. Jackson, Ian Johnston, Dominic Kwiatkowski, Cordelia Langford, John Sillitoe on behalf of the Wellcome Sanger Institute COVID-19 Surveillance Team
EPI_ISL_675134	Lighthouse Lab in Milton Keynes	Wellcome Sanger Institute for the COVID-19 Genomics UK (COG-UK) Consortium	The Lighthouse Lab in Milton Keynes and Alex Alderton, Roberto Amato, Sonia Goncalves, Ewan Harrison, David K. Jackson, Ian Johnston, Dominic Kwiatkowski, Cordelia Langford, John Sillitoe on behalf of the Wellcome Sanger Institute COVID-19 Surveillance Team
EPI_ISL_675135, EPI_ISL_675136, EPI_ISL_675137, EPI_ISL_675138, EPI_ISL_675139, EPI_ISL_675140, EPI_ISL_675141, EPI_ISL_675142, EPI_ISL_675143, EPI_ISL_675144	Lighthouse Lab in Alderley Park	Wellcome Sanger Institute for the COVID-19 Genomics UK (COG-UK) Consortium	Jacquelyn Wynn, Mairead Hyland, The Lighthouse Lab in Alderley Park and Alex Alderton, Roberto Amato, Sonia Goncalves, Ewan Harrison, David K. Jackson, Ian Johnston, Dominic Kwiatkowski, Cordelia Langford, John Sillitoe on behalf of the Wellcome Sanger Institute COVID-19 Surveillance Team
EPI_ISL_675145	Lighthouse Lab in Milton Keynes	Wellcome Sanger Institute for the COVID-19 Genomics UK (COG-UK) Consortium	The Lighthouse Lab in Milton Keynes and Alex Alderton, Roberto Amato, Sonia Goncalves, Ewan Harrison, David K. Jackson, Ian Johnston, Dominic Kwiatkowski, Cordelia Langford, John Sillitoe on behalf of the Wellcome Sanger Institute COVID-19 Surveillance Team
EPI_ISL_675146, EPI_ISL_675147, EPI_ISL_675148, EPI_ISL_675149, EPI_ISL_675150, EPI_ISL_675151, EPI_ISL_675152	Lighthouse Lab in Alderley Park	Wellcome Sanger Institute for the COVID-19 Genomics UK (COG-UK) Consortium	Jacquelyn Wynn, Mairead Hyland, The Lighthouse Lab in Alderley Park and Alex Alderton, Roberto Amato, Sonia Goncalves, Ewan Harrison, David K. Jackson, Ian Johnston, Dominic Kwiatkowski, Cordelia Langford, John Sillitoe on behalf of the Wellcome Sanger Institute COVID-19 Surveillance Team
EPI_ISL_675153	Lighthouse Lab in Milton Keynes	Wellcome Sanger Institute for the COVID-19 Genomics UK (COG-UK) Consortium	The Lighthouse Lab in Milton Keynes and Alex Alderton, Roberto Amato, Sonia Goncalves, Ewan Harrison, David K. Jackson, Ian Johnston, Dominic Kwiatkowski, Cordelia Langford, John Sillitoe on behalf of the Wellcome Sanger Institute COVID-19 Surveillance Team
EPI_ISL_675154, EPI_ISL_675155, EPI_ISL_675156, EPI_ISL_675157, EPI_ISL_675158, EPI_ISL_675159	Lighthouse Lab in Alderley Park	Wellcome Sanger Institute for the COVID-19 Genomics UK (COG-UK) Consortium	Jacquelyn Wynn, Mairead Hyland, The Lighthouse Lab in Alderley Park and Alex Alderton, Roberto Amato, Sonia Goncalves, Ewan Harrison, David K. Jackson, Ian Johnston, Dominic Kwiatkowski, Cordelia Langford, John Sillitoe on behalf of the Wellcome Sanger Institute COVID-19 Surveillance Team
EPI_ISL_675160	Lighthouse Lab in Milton Keynes	Wellcome Sanger Institute for the COVID-19 Genomics UK (COG-UK) Consortium	The Lighthouse Lab in Milton Keynes and Alex Alderton, Roberto Amato, Sonia Goncalves, Ewan Harrison, David K. Jackson, Ian Johnston, Dominic Kwiatkowski, Cordelia Langford, John Sillitoe on behalf of the Wellcome Sanger Institute COVID-19 Surveillance Team
EPI_ISL_675161, EPI_ISL_675162, EPI_ISL_675163, EPI_ISL_675164	Lighthouse Lab in Alderley Park	Wellcome Sanger Institute for the COVID-19 Genomics UK (COG-UK) Consortium	Jacquelyn Wynn, Mairead Hyland, The Lighthouse Lab in Alderley Park and Alex Alderton, Roberto Amato, Sonia Goncalves, Ewan Harrison, David K. Jackson, Ian Johnston, Dominic Kwiatkowski, Cordelia Langford, John Sillitoe on behalf of the Wellcome Sanger Institute COVID-19 Surveillance Team
EPI_ISL_675165	Lighthouse Lab in Milton Keynes	Wellcome Sanger Institute for the COVID-19 Genomics UK (COG-UK) Consortium	The Lighthouse Lab in Milton Keynes and Alex Alderton, Roberto Amato, Sonia Goncalves, Ewan Harrison, David K. Jackson, Ian Johnston, Dominic Kwiatkowski, Cordelia Langford, John Sillitoe on behalf of the Wellcome Sanger Institute COVID-19 Surveillance Team
EPI_ISL_675166, EPI_ISL_675167, EPI_ISL_675168, EPI_ISL_675169	Lighthouse Lab in Alderley Park	Wellcome Sanger Institute for the COVID-19 Genomics UK (COG-UK) Consortium	Jacquelyn Wynn, Mairead Hyland, The Lighthouse Lab in Alderley Park and Alex Alderton, Roberto Amato, Sonia Goncalves, Ewan Harrison, David K. Jackson, Ian Johnston, Dominic Kwiatkowski, Cordelia Langford, John Sillitoe on behalf of the Wellcome Sanger Institute COVID-19 Surveillance Team
EPI_ISL_675170	Lighthouse Lab in Milton Keynes	Wellcome Sanger Institute for the COVID-19 Genomics UK (COG-UK) Consortium	The Lighthouse Lab in Milton Keynes and Alex Alderton, Roberto Amato, Sonia Goncalves, Ewan Harrison, David K. Jackson, Ian Johnston, Dominic Kwiatkowski, Cordelia Langford, John Sillitoe on behalf of the Wellcome Sanger Institute COVID-19 Surveillance Team
EPI_ISL_675171, EPI_ISL_675172, EPI_ISL_675173, EPI_ISL_675174, EPI_ISL_675175, EPI_ISL_675176, EPI_ISL_675177	Lighthouse Lab in Alderley Park	Wellcome Sanger Institute for the COVID-19 Genomics UK (COG-UK) Consortium	Jacquelyn Wynn, Mairead Hyland, The Lighthouse Lab in Alderley Park and Alex Alderton, Roberto Amato, Sonia Goncalves, Ewan Harrison, David K. Jackson, Ian Johnston, Dominic Kwiatkowski, Cordelia Langford, John Sillitoe on behalf of the Wellcome Sanger Institute COVID-19 Surveillance Team
EPI_ISL_675178, EPI_ISL_675179	Lighthouse Lab in Milton Keynes	Wellcome Sanger Institute for the COVID-19 Genomics UK (COG-UK) Consortium	The Lighthouse Lab in Milton Keynes and Alex Alderton, Roberto Amato, Sonia Goncalves, Ewan Harrison, David K. Jackson, Ian Johnston, Dominic Kwiatkowski, Cordelia Langford, John Sillitoe on behalf of the Wellcome Sanger Institute COVID-19 Surveillance Team
EPI_ISL_675180, EPI_ISL_675181, EPI_ISL_675182	Lighthouse Lab in Alderley Park	Wellcome Sanger Institute for the COVID-19 Genomics UK (COG-UK) Consortium	Jacquelyn Wynn, Mairead Hyland, The Lighthouse Lab in Alderley Park and Alex Alderton, Roberto Amato, Sonia Goncalves, Ewan Harrison, David K. Jackson, Ian Johnston, Dominic Kwiatkowski, Cordelia Langford, John Sillitoe on behalf of the Wellcome Sanger Institute COVID-19 Surveillance Team
EPI_ISL_675183	Lighthouse Lab in Milton Keynes	Wellcome Sanger Institute for the COVID-19 Genomics UK (COG-UK) Consortium	The Lighthouse Lab in Milton Keynes and Alex Alderton, Roberto Amato, Sonia Goncalves, Ewan Harrison, David K. Jackson, Ian Johnston, Dominic Kwiatkowski, Cordelia Langford, John Sillitoe on behalf of the Wellcome Sanger Institute COVID-19 Surveillance Team
EPI_ISL_675184	Lighthouse Lab in Alderley Park	Wellcome Sanger Institute for the COVID-19 Genomics UK (COG-UK) Consortium	Jacquelyn Wynn, Mairead Hyland, The Lighthouse Lab in Alderley Park and Alex Alderton, Roberto Amato, Sonia Goncalves, Ewan Harrison, David K. Jackson, Ian Johnston, Dominic Kwiatkowski, Cordelia Langford, John Sillitoe on behalf of the Wellcome Sanger Institute COVID-19 Surveillance Team
EPI_ISL_675185	Lighthouse Lab in Milton Keynes	Wellcome Sanger Institute for the COVID-19 Genomics UK (COG-UK) Consortium	The Lighthouse Lab in Milton Keynes and Alex Alderton, Roberto Amato, Sonia Goncalves, Ewan Harrison, David K. Jackson, Ian Johnston, Dominic Kwiatkowski, Cordelia Langford, John Sillitoe on behalf of the Wellcome Sanger Institute COVID-19 Surveillance Team
EPI_ISL_675186, EPI_ISL_675187	Lighthouse Lab in Alderley Park	Wellcome Sanger Institute for the COVID-19 Genomics UK (COG-UK) Consortium	Jacquelyn Wynn, Mairead Hyland, The Lighthouse Lab in Alderley Park and Alex Alderton, Roberto Amato, Sonia Goncalves, Ewan Harrison, David K. Jackson, Ian Johnston, Dominic Kwiatkowski, Cordelia Langford, John Sillitoe on behalf of the Wellcome Sanger Institute COVID-19 Surveillance Team
EPI_ISL_675188	Lighthouse Lab in Milton Keynes	Wellcome Sanger Institute for the COVID-19 Genomics UK (COG-UK) Consortium	The Lighthouse Lab in Milton Keynes and Alex Alderton, Roberto Amato, Sonia Goncalves, Ewan Harrison, David K. Jackson, Ian Johnston, Dominic Kwiatkowski, Cordelia Langford, John Sillitoe on behalf of the Wellcome Sanger Institute COVID-19 Surveillance Team

EPI_ISL_675189, EPI_ISL_675190, EPI_ISL_675191, EPI_ISL_675192, EPI_ISL_675193, EPI_ISL_675194, EPI_ISL_675195, EPI_ISL_675196, EPI_ISL_675197, EPI_ISL_675198, EPI_ISL_675199, EPI_ISL_675200, EPI_ISL_675201, EPI_ISL_675202, EPI_ISL_675203, EPI_ISL_675204, EPI_ISL_675205, EPI_ISL_675206, EPI_ISL_675207, EPI_ISL_675208, EPI_ISL_675209

see above	Lighthouse Lab in Alderley Park	Wellcome Sanger Institute for the COVID-19 Genomics UK (COG-UK) Consortium	Jacquelyn Wynn, Mairead Hyland, The Lighthouse Lab in Alderley Park and Alex Alderton, Roberto Amato, Sonia Goncalves, Ewan Harrison, David K. Jackson, Ian Johnston, Dominic Kwiatkowski, Cordelia Langford, John Sillitoe on behalf of the Wellcome Sanger Institute COVID-19 Surveillance Team
EPI_ISL_675210	Lighthouse Lab in Milton Keynes	Wellcome Sanger Institute for the COVID-19 Genomics UK (COG-UK) Consortium	The Lighthouse Lab in Milton Keynes and Alex Alderton, Roberto Amato, Sonia Goncalves, Ewan Harrison, David K. Jackson, Ian Johnston, Dominic Kwiatkowski, Cordelia Langford, John Sillitoe on behalf of the Wellcome Sanger Institute COVID-19 Surveillance Team
EPI_ISL_675211	Lighthouse Lab in Alderley Park	Wellcome Sanger Institute for the COVID-19 Genomics UK (COG-UK) Consortium	Jacquelyn Wynn, Mairead Hyland, The Lighthouse Lab in Alderley Park and Alex Alderton, Roberto Amato, Sonia Goncalves, Ewan Harrison, David K. Jackson, Ian Johnston, Dominic Kwiatkowski, Cordelia Langford, John Sillitoe on behalf of the Wellcome Sanger Institute COVID-19 Surveillance Team
EPI_ISL_675212	Lighthouse Lab in Milton Keynes	Wellcome Sanger Institute for the COVID-19 Genomics UK (COG-UK) Consortium	The Lighthouse Lab in Milton Keynes and Alex Alderton, Roberto Amato, Sonia Goncalves, Ewan Harrison, David K. Jackson, Ian Johnston, Dominic Kwiatkowski, Cordelia Langford, John Sillitoe on behalf of the Wellcome Sanger Institute COVID-19 Surveillance Team
EPI_ISL_675213, EPI_ISL_675214, EPI_ISL_675215, EPI_ISL_675216	Lighthouse Lab in Alderley Park	Wellcome Sanger Institute for the COVID-19 Genomics UK (COG-UK) Consortium	Jacquelyn Wynn, Mairead Hyland, The Lighthouse Lab in Alderley Park and Alex Alderton, Roberto Amato, Sonia Goncalves, Ewan Harrison, David K. Jackson, Ian Johnston, Dominic Kwiatkowski, Cordelia Langford, John Sillitoe on behalf of the Wellcome Sanger Institute COVID-19 Surveillance Team
EPI_ISL_675217	Lighthouse Lab in Milton Keynes	Wellcome Sanger Institute for the COVID-19 Genomics UK (COG-UK) Consortium	The Lighthouse Lab in Milton Keynes and Alex Alderton, Roberto Amato, Sonia Goncalves, Ewan Harrison, David K. Jackson, Ian Johnston, Dominic Kwiatkowski, Cordelia Langford, John Sillitoe on behalf of the Wellcome Sanger Institute COVID-19 Surveillance Team
EPI_ISL_675218	Lighthouse Lab in Alderley Park	Wellcome Sanger Institute for the COVID-19 Genomics UK (COG-UK) Consortium	Jacquelyn Wynn, Mairead Hyland, The Lighthouse Lab in Alderley Park and Alex Alderton, Roberto Amato, Sonia Goncalves, Ewan Harrison, David K. Jackson, Ian Johnston, Dominic Kwiatkowski, Cordelia Langford, John Sillitoe on behalf of the Wellcome Sanger Institute COVID-19 Surveillance Team
EPI_ISL_675219	Lighthouse Lab in Milton Keynes	Wellcome Sanger Institute for the COVID-19 Genomics UK (COG-UK) Consortium	The Lighthouse Lab in Milton Keynes and Alex Alderton, Roberto Amato, Sonia Goncalves, Ewan Harrison, David K. Jackson, Ian Johnston, Dominic Kwiatkowski, Cordelia Langford, John Sillitoe on behalf of the Wellcome Sanger Institute COVID-19 Surveillance Team
EPI_ISL_675220, EPI_ISL_675221, EPI_ISL_675222, EPI_ISL_675223	Lighthouse Lab in Alderley Park	Wellcome Sanger Institute for the COVID-19 Genomics UK (COG-UK) Consortium	Jacquelyn Wynn, Mairead Hyland, The Lighthouse Lab in Alderley Park and Alex Alderton, Roberto Amato, Sonia Goncalves, Ewan Harrison, David K. Jackson, Ian Johnston, Dominic Kwiatkowski, Cordelia Langford, John Sillitoe on behalf of the Wellcome Sanger Institute COVID-19 Surveillance Team
EPI_ISL_675224	Lighthouse Lab in Milton Keynes	Wellcome Sanger Institute for the COVID-19 Genomics UK (COG-UK) Consortium	The Lighthouse Lab in Milton Keynes and Alex Alderton, Roberto Amato, Sonia Goncalves, Ewan Harrison, David K. Jackson, Ian Johnston, Dominic Kwiatkowski, Cordelia Langford, John Sillitoe on behalf of the Wellcome Sanger Institute COVID-19 Surveillance Team

EPI_ISL_675225, EPI_ISL_675226	Lighthouse Lab in Alderley Park	Wellcome Sanger Institute for the COVID-19 Genomics UK (COG-UK) Consortium	Jacquelyn Wynn, Mairead Hyland, The Lighthouse Lab in Alderley Park and Alex Alderton, Roberto Amato, Sonia Goncalves, Ewan Harrison, David K. Jackson, Ian Johnston, Dominic Kwiatkowski, Cordelia Langford, John Sillitoe on behalf of the Wellcome Sanger Institute COVID-19 Surveillance Team
EPI_ISL_675227, EPI_ISL_675228	Lighthouse Lab in Milton Keynes	Wellcome Sanger Institute for the COVID-19 Genomics UK (COG-UK) Consortium	The Lighthouse Lab in Milton Keynes and Alex Alderton, Roberto Amato, Sonia Goncalves, Ewan Harrison, David K. Jackson, Ian Johnston, Dominic Kwiatkowski, Cordelia Langford, John Sillitoe on behalf of the Wellcome Sanger Institute COVID-19 Surveillance Team
EPI_ISL_675229, EPI_ISL_675230, EPI_ISL_675231, EPI_ISL_675232, EPI_ISL_675233, EPI_ISL_675234, EPI_ISL_675235	Lighthouse Lab in Alderley Park	Wellcome Sanger Institute for the COVID-19 Genomics UK (COG-UK) Consortium	Jacquelyn Wynn, Mairead Hyland, The Lighthouse Lab in Alderley Park and Alex Alderton, Roberto Amato, Sonia Goncalves, Ewan Harrison, David K. Jackson, Ian Johnston, Dominic Kwiatkowski, Cordelia Langford, John Sillitoe on behalf of the Wellcome Sanger Institute COVID-19 Surveillance Team
EPI_ISL_675236	Lighthouse Lab in Milton Keynes	Wellcome Sanger Institute for the COVID-19 Genomics UK (COG-UK) Consortium	The Lighthouse Lab in Milton Keynes and Alex Alderton, Roberto Amato, Sonia Goncalves, Ewan Harrison, David K. Jackson, Ian Johnston, Dominic Kwiatkowski, Cordelia Langford, John Sillitoe on behalf of the Wellcome Sanger Institute COVID-19 Surveillance Team
EPI_ISL_675237	Lighthouse Lab in Alderley Park	Wellcome Sanger Institute for the COVID-19 Genomics UK (COG-UK) Consortium	Jacquelyn Wynn, Mairead Hyland, The Lighthouse Lab in Alderley Park and Alex Alderton, Roberto Amato, Sonia Goncalves, Ewan Harrison, David K. Jackson, Ian Johnston, Dominic Kwiatkowski, Cordelia Langford, John Sillitoe on behalf of the Wellcome Sanger Institute COVID-19 Surveillance Team
EPI_ISL_675238	Lighthouse Lab in Milton Keynes	Wellcome Sanger Institute for the COVID-19 Genomics UK (COG-UK) Consortium	The Lighthouse Lab in Milton Keynes and Alex Alderton, Roberto Amato, Sonia Goncalves, Ewan Harrison, David K. Jackson, Ian Johnston, Dominic Kwiatkowski, Cordelia Langford, John Sillitoe on behalf of the Wellcome Sanger Institute COVID-19 Surveillance Team
EPI_ISL_675239, EPI_ISL_675240	Lighthouse Lab in Alderley Park	Wellcome Sanger Institute for the COVID-19 Genomics UK (COG-UK) Consortium	Jacquelyn Wynn, Mairead Hyland, The Lighthouse Lab in Alderley Park and Alex Alderton, Roberto Amato, Sonia Goncalves, Ewan Harrison, David K. Jackson, Ian Johnston, Dominic Kwiatkowski, Cordelia Langford, John Sillitoe on behalf of the Wellcome Sanger Institute COVID-19 Surveillance Team
EPI_ISL_675241	Lighthouse Lab in Milton Keynes	Wellcome Sanger Institute for the COVID-19 Genomics UK (COG-UK) Consortium	The Lighthouse Lab in Milton Keynes and Alex Alderton, Roberto Amato, Sonia Goncalves, Ewan Harrison, David K. Jackson, Ian Johnston, Dominic Kwiatkowski, Cordelia Langford, John Sillitoe on behalf of the Wellcome Sanger Institute COVID-19 Surveillance Team
EPI_ISL_675242	Lighthouse Lab in Alderley Park	Wellcome Sanger Institute for the COVID-19 Genomics UK (COG-UK) Consortium	Jacquelyn Wynn, Mairead Hyland, The Lighthouse Lab in Alderley Park and Alex Alderton, Roberto Amato, Sonia Goncalves, Ewan Harrison, David K. Jackson, Ian Johnston, Dominic Kwiatkowski, Cordelia Langford, John Sillitoe on behalf of the Wellcome Sanger Institute COVID-19 Surveillance Team
EPI_ISL_675243, EPI_ISL_675244	Lighthouse Lab in Milton Keynes	Wellcome Sanger Institute for the COVID-19 Genomics UK (COG-UK) Consortium	The Lighthouse Lab in Milton Keynes and Alex Alderton, Roberto Amato, Sonia Goncalves, Ewan Harrison, David K. Jackson, Ian Johnston, Dominic Kwiatkowski, Cordelia Langford, John Sillitoe on behalf of the Wellcome Sanger Institute COVID-19 Surveillance Team
EPI_ISL_675245, EPI_ISL_675246, EPI_ISL_675247, EPI_ISL_675248, EPI_ISL_675249, EPI_ISL_675250, EPI_ISL_675251, EPI_ISL_675252, EPI_ISL_675253, EPI_ISL_675254, EPI_ISL_675255, EPI_ISL_675256, EPI_ISL_675257, EPI_ISL_675258, EPI_ISL_675259			
see above	Lighthouse Lab in Alderley Park	Wellcome Sanger Institute for the COVID-19 Genomics UK (COG-UK) Consortium	Jacquelyn Wynn, Mairead Hyland, The Lighthouse Lab in Alderley Park and Alex Alderton, Roberto Amato, Sonia Goncalves, Ewan Harrison, David K. Jackson, Ian Johnston, Dominic Kwiatkowski, Cordelia Langford, John Sillitoe on behalf of the Wellcome Sanger Institute COVID-19 Surveillance Team
EPI_ISL_675260	Lighthouse Lab in Milton Keynes	Wellcome Sanger Institute for the COVID-19 Genomics UK (COG-UK) Consortium	The Lighthouse Lab in Milton Keynes and Alex Alderton, Roberto Amato, Sonia Goncalves, Ewan Harrison, David K. Jackson, Ian Johnston, Dominic Kwiatkowski, Cordelia Langford, John Sillitoe on behalf of the Wellcome Sanger Institute COVID-19 Surveillance Team
EPI_ISL_675261, EPI_ISL_675262, EPI_ISL_675263	Lighthouse Lab in Alderley Park	Wellcome Sanger Institute for the COVID-19 Genomics UK (COG-UK) Consortium	Jacquelyn Wynn, Mairead Hyland, The Lighthouse Lab in Alderley Park and Alex Alderton, Roberto Amato, Sonia Goncalves, Ewan Harrison, David K. Jackson, Ian Johnston, Dominic Kwiatkowski, Cordelia Langford, John Sillitoe on behalf of the Wellcome Sanger Institute COVID-19 Surveillance Team
EPI_ISL_675264	Lighthouse Lab in Milton Keynes	Wellcome Sanger Institute for the COVID-19 Genomics UK (COG-UK) Consortium	The Lighthouse Lab in Milton Keynes and Alex Alderton, Roberto Amato, Sonia Goncalves, Ewan Harrison, David K. Jackson, Ian Johnston, Dominic Kwiatkowski, Cordelia Langford, John Sillitoe on behalf of the Wellcome Sanger Institute COVID-19 Surveillance Team
EPI_ISL_675265, EPI_ISL_675266, EPI_ISL_675268, EPI_ISL_675269, EPI_ISL_675270, EPI_ISL_675271, EPI_ISL_675272, EPI_ISL_675273, EPI_ISL_675274	Lighthouse Lab in Alderley Park	Wellcome Sanger Institute for the COVID-19 Genomics UK (COG-UK) Consortium	Jacquelyn Wynn, Mairead Hyland, The Lighthouse Lab in Alderley Park and Alex Alderton, Roberto Amato, Sonia Goncalves, Ewan Harrison, David K. Jackson, Ian Johnston, Dominic Kwiatkowski, Cordelia Langford, John Sillitoe on behalf of the Wellcome Sanger Institute COVID-19 Surveillance Team
EPI_ISL_675275, EPI_ISL_675276, EPI_ISL_675277	Lighthouse Lab in Milton Keynes	Wellcome Sanger Institute for the COVID-19 Genomics UK (COG-UK) Consortium	The Lighthouse Lab in Milton Keynes and Alex Alderton, Roberto Amato, Sonia Goncalves, Ewan Harrison, David K. Jackson, Ian Johnston, Dominic Kwiatkowski, Cordelia Langford, John Sillitoe on behalf of the Wellcome Sanger Institute COVID-19 Surveillance Team
EPI_ISL_675278, EPI_ISL_675279, EPI_ISL_675280, EPI_ISL_675281	Lighthouse Lab in Alderley Park	Wellcome Sanger Institute for the COVID-19 Genomics UK (COG-UK) Consortium	Jacquelyn Wynn, Mairead Hyland, The Lighthouse Lab in Alderley Park and Alex Alderton, Roberto Amato, Sonia Goncalves, Ewan Harrison, David K. Jackson, Ian Johnston, Dominic Kwiatkowski, Cordelia Langford, John Sillitoe on behalf of the Wellcome Sanger Institute COVID-19 Surveillance Team
EPI_ISL_675282	Lighthouse Lab in Milton Keynes	Wellcome Sanger Institute for the COVID-19 Genomics UK (COG-UK) Consortium	The Lighthouse Lab in Milton Keynes and Alex Alderton, Roberto Amato, Sonia Goncalves, Ewan Harrison, David K. Jackson, Ian Johnston, Dominic Kwiatkowski, Cordelia Langford, John Sillitoe on behalf of the Wellcome Sanger Institute COVID-19 Surveillance Team
EPI_ISL_675283	Lighthouse Lab in Alderley Park	Wellcome Sanger Institute for the COVID-19 Genomics UK (COG-UK) Consortium	Jacquelyn Wynn, Mairead Hyland, The Lighthouse Lab in Alderley Park and Alex Alderton, Roberto Amato, Sonia Goncalves, Ewan Harrison, David K. Jackson, Ian Johnston, Dominic Kwiatkowski, Cordelia Langford, John Sillitoe on behalf of the Wellcome Sanger Institute COVID-19 Surveillance Team
EPI_ISL_675284, EPI_ISL_675285, EPI_ISL_675286	Lighthouse Lab in Milton Keynes	Wellcome Sanger Institute for the COVID-19 Genomics UK (COG-UK) Consortium	The Lighthouse Lab in Milton Keynes and Alex Alderton, Roberto Amato, Sonia Goncalves, Ewan Harrison, David K. Jackson, Ian Johnston, Dominic Kwiatkowski, Cordelia Langford, John Sillitoe on behalf of the Wellcome Sanger Institute COVID-19 Surveillance Team
EPI_ISL_675287, EPI_ISL_675288, EPI_ISL_675289, EPI_ISL_675290, EPI_ISL_675291	Lighthouse Lab in Alderley Park	Wellcome Sanger Institute for the COVID-19 Genomics UK (COG-UK) Consortium	Jacquelyn Wynn, Mairead Hyland, The Lighthouse Lab in Alderley Park and Alex Alderton, Roberto Amato, Sonia Goncalves, Ewan Harrison, David K. Jackson, Ian Johnston, Dominic Kwiatkowski, Cordelia Langford, John Sillitoe on behalf of the Wellcome Sanger Institute COVID-19 Surveillance Team
EPI_ISL_675292	Lighthouse Lab in Milton Keynes	Wellcome Sanger Institute for the COVID-19 Genomics UK (COG-UK) Consortium	The Lighthouse Lab in Milton Keynes and Alex Alderton, Roberto Amato, Sonia Goncalves, Ewan Harrison, David K. Jackson, Ian Johnston, Dominic Kwiatkowski, Cordelia Langford, John Sillitoe on behalf of the Wellcome Sanger Institute COVID-19 Surveillance Team
EPI_ISL_675293, EPI_ISL_675294, EPI_ISL_675295, EPI_ISL_675296, EPI_ISL_675297, EPI_ISL_675298, EPI_ISL_675299, EPI_ISL_675300	Lighthouse Lab in Alderley Park	Wellcome Sanger Institute for the COVID-19 Genomics UK (COG-UK) Consortium	Jacquelyn Wynn, Mairead Hyland, The Lighthouse Lab in Alderley Park and Alex Alderton, Roberto Amato, Sonia Goncalves, Ewan Harrison, David K. Jackson, Ian Johnston, Dominic Kwiatkowski, Cordelia Langford, John Sillitoe on behalf of the Wellcome Sanger Institute COVID-19 Surveillance Team
EPI_ISL_675301	Lighthouse Lab in Milton Keynes	Wellcome Sanger Institute for the COVID-19 Genomics UK (COG-UK) Consortium	The Lighthouse Lab in Milton Keynes and Alex Alderton, Roberto Amato, Sonia Goncalves, Ewan Harrison, David K. Jackson, Ian Johnston, Dominic Kwiatkowski, Cordelia Langford, John Sillitoe on behalf of the Wellcome Sanger Institute COVID-19 Surveillance Team
EPI_ISL_675302, EPI_ISL_675303, EPI_ISL_675304	Lighthouse Lab in Alderley Park	Wellcome Sanger Institute for the COVID-19 Genomics UK (COG-UK) Consortium	Jacquelyn Wynn, Mairead Hyland, The Lighthouse Lab in Alderley Park and Alex Alderton, Roberto Amato, Sonia Goncalves, Ewan Harrison, David K. Jackson, Ian Johnston, Dominic Kwiatkowski, Cordelia Langford, John Sillitoe on behalf of the Wellcome Sanger Institute COVID-19 Surveillance Team
EPI_ISL_675305, EPI_ISL_675306, EPI_ISL_675307, EPI_ISL_675308, EPI_ISL_675309	Lighthouse Lab in Milton Keynes	Wellcome Sanger Institute for the COVID-19 Genomics UK (COG-UK) Consortium	The Lighthouse Lab in Milton Keynes and Alex Alderton, Roberto Amato, Sonia Goncalves, Ewan Harrison, David K. Jackson, Ian Johnston, Dominic Kwiatkowski, Cordelia Langford, John Sillitoe on behalf of the Wellcome Sanger Institute COVID-19 Surveillance Team
EPI_ISL_675310	Lighthouse Lab in Alderley Park	Wellcome Sanger Institute for the COVID-19 Genomics UK (COG-UK) Consortium	Jacquelyn Wynn, Mairead Hyland, The Lighthouse Lab in Alderley Park and Alex Alderton, Roberto Amato, Sonia Goncalves, Ewan Harrison, David K. Jackson, Ian Johnston, Dominic Kwiatkowski, Cordelia Langford, John Sillitoe on behalf of the Wellcome Sanger Institute COVID-19 Surveillance Team
EPI_ISL_675311	Lighthouse Lab in Milton Keynes	Wellcome Sanger Institute for the COVID-19 Genomics UK (COG-UK) Consortium	The Lighthouse Lab in Milton Keynes and Alex Alderton, Roberto Amato, Sonia Goncalves, Ewan Harrison, David K. Jackson, Ian Johnston, Dominic Kwiatkowski, Cordelia Langford, John Sillitoe on behalf of the Wellcome Sanger Institute COVID-19 Surveillance Team
EPI_ISL_675312	Lighthouse Lab in Alderley Park	Wellcome Sanger Institute for the COVID-19 Genomics UK (COG-UK) Consortium	Jacquelyn Wynn, Mairead Hyland, The Lighthouse Lab in Alderley Park and Alex Alderton, Roberto Amato, Sonia Goncalves, Ewan Harrison, David K. Jackson, Ian Johnston, Dominic Kwiatkowski, Cordelia Langford, John Sillitoe on behalf of the Wellcome Sanger Institute COVID-19 Surveillance Team
EPI_ISL_675313	Lighthouse Lab in Milton Keynes	Wellcome Sanger Institute for the COVID-19 Genomics UK (COG-UK) Consortium	The Lighthouse Lab in Milton Keynes and Alex Alderton, Roberto Amato, Sonia Goncalves, Ewan Harrison, David K. Jackson, Ian Johnston, Dominic Kwiatkowski, Cordelia Langford, John Sillitoe on behalf of the Wellcome Sanger Institute COVID-19 Surveillance Team
EPI_ISL_675314, EPI_ISL_675315, EPI_ISL_675316	Lighthouse Lab in Alderley Park	Wellcome Sanger Institute for the COVID-19 Genomics UK (COG-UK) Consortium	Jacquelyn Wynn, Mairead Hyland, The Lighthouse Lab in Alderley Park and Alex Alderton, Roberto Amato, Sonia Goncalves, Ewan Harrison, David K. Jackson, Ian Johnston, Dominic Kwiatkowski, Cordelia Langford, John Sillitoe on behalf of the Wellcome Sanger Institute COVID-19 Surveillance Team

EPI_ISL_675317, EPI_ISL_675318, EPI_ISL_675319, EPI_ISL_675320	Lighthouse Lab in Milton Keynes	Wellcome Sanger Institute for the COVID-19 Genomics UK (COG-UK) Consortium	The Lighthouse Lab in Milton Keynes and Alex Alderton, Roberto Amato, Sonia Goncalves, Ewan Harrison, David K. Jackson, Ian Johnston, Dominic Kwiatkowski, Cordelia Langford, John Sillitoe on behalf of the Wellcome Sanger Institute COVID-19 Surveillance Team
EPI_ISL_675321, EPI_ISL_675322, EPI_ISL_675323, EPI_ISL_675324, EPI_ISL_675325	Lighthouse Lab in Alderley Park	Wellcome Sanger Institute for the COVID-19 Genomics UK (COG-UK) Consortium	Jacquelyn Wynn, Mairead Hyland, The Lighthouse Lab in Alderley Park and Alex Alderton, Roberto Amato, Sonia Goncalves, Ewan Harrison, David K. Jackson, Ian Johnston, Dominic Kwiatkowski, Cordelia Langford, John Sillitoe on behalf of the Wellcome Sanger Institute COVID-19 Surveillance Team
EPI_ISL_675326	Lighthouse Lab in Milton Keynes	Wellcome Sanger Institute for the COVID-19 Genomics UK (COG-UK) Consortium	The Lighthouse Lab in Milton Keynes and Alex Alderton, Roberto Amato, Sonia Goncalves, Ewan Harrison, David K. Jackson, Ian Johnston, Dominic Kwiatkowski, Cordelia Langford, John Sillitoe on behalf of the Wellcome Sanger Institute COVID-19 Surveillance Team
EPI_ISL_675327, EPI_ISL_675328, EPI_ISL_675329, EPI_ISL_675330	Lighthouse Lab in Alderley Park	Wellcome Sanger Institute for the COVID-19 Genomics UK (COG-UK) Consortium	Jacquelyn Wynn, Mairead Hyland, The Lighthouse Lab in Alderley Park and Alex Alderton, Roberto Amato, Sonia Goncalves, Ewan Harrison, David K. Jackson, Ian Johnston, Dominic Kwiatkowski, Cordelia Langford, John Sillitoe on behalf of the Wellcome Sanger Institute COVID-19 Surveillance Team
EPI_ISL_675331, EPI_ISL_675332	Lighthouse Lab in Milton Keynes	Wellcome Sanger Institute for the COVID-19 Genomics UK (COG-UK) Consortium	The Lighthouse Lab in Milton Keynes and Alex Alderton, Roberto Amato, Sonia Goncalves, Ewan Harrison, David K. Jackson, Ian Johnston, Dominic Kwiatkowski, Cordelia Langford, John Sillitoe on behalf of the Wellcome Sanger Institute COVID-19 Surveillance Team
EPI_ISL_675333, EPI_ISL_675334, EPI_ISL_675335, EPI_ISL_675336, EPI_ISL_675337, EPI_ISL_675338, EPI_ISL_675339	Lighthouse Lab in Alderley Park	Wellcome Sanger Institute for the COVID-19 Genomics UK (COG-UK) Consortium	Jacquelyn Wynn, Mairead Hyland, The Lighthouse Lab in Alderley Park and Alex Alderton, Roberto Amato, Sonia Goncalves, Ewan Harrison, David K. Jackson, Ian Johnston, Dominic Kwiatkowski, Cordelia Langford, John Sillitoe on behalf of the Wellcome Sanger Institute COVID-19 Surveillance Team
EPI_ISL_675340	Lighthouse Lab in Milton Keynes	Wellcome Sanger Institute for the COVID-19 Genomics UK (COG-UK) Consortium	The Lighthouse Lab in Milton Keynes and Alex Alderton, Roberto Amato, Sonia Goncalves, Ewan Harrison, David K. Jackson, Ian Johnston, Dominic Kwiatkowski, Cordelia Langford, John Sillitoe on behalf of the Wellcome Sanger Institute COVID-19 Surveillance Team
EPI_ISL_675341, EPI_ISL_675342, EPI_ISL_675343, EPI_ISL_675344, EPI_ISL_675345, EPI_ISL_675346, EPI_ISL_675347, EPI_ISL_675348, EPI_ISL_675349, EPI_ISL_675350, EPI_ISL_675351, EPI_ISL_675352			
see above	Lighthouse Lab in Alderley Park	Wellcome Sanger Institute for the COVID-19 Genomics UK (COG-UK) Consortium	Jacquelyn Wynn, Mairead Hyland, The Lighthouse Lab in Alderley Park and Alex Alderton, Roberto Amato, Sonia Goncalves, Ewan Harrison, David K. Jackson, Ian Johnston, Dominic Kwiatkowski, Cordelia Langford, John Sillitoe on behalf of the Wellcome Sanger Institute COVID-19 Surveillance Team
EPI_ISL_675353	Lighthouse Lab in Milton Keynes	Wellcome Sanger Institute for the COVID-19 Genomics UK (COG-UK) Consortium	The Lighthouse Lab in Milton Keynes and Alex Alderton, Roberto Amato, Sonia Goncalves, Ewan Harrison, David K. Jackson, Ian Johnston, Dominic Kwiatkowski, Cordelia Langford, John Sillitoe on behalf of the Wellcome Sanger Institute COVID-19 Surveillance Team
EPI_ISL_675354, EPI_ISL_675355, EPI_ISL_675356	Lighthouse Lab in Alderley Park	Wellcome Sanger Institute for the COVID-19 Genomics UK (COG-UK) Consortium	Jacquelyn Wynn, Mairead Hyland, The Lighthouse Lab in Alderley Park and Alex Alderton, Roberto Amato, Sonia Goncalves, Ewan Harrison, David K. Jackson, Ian Johnston, Dominic Kwiatkowski, Cordelia Langford, John Sillitoe on behalf of the Wellcome Sanger Institute COVID-19 Surveillance Team
EPI_ISL_675357	Lighthouse Lab in Milton Keynes	Wellcome Sanger Institute for the COVID-19 Genomics UK (COG-UK) Consortium	The Lighthouse Lab in Milton Keynes and Alex Alderton, Roberto Amato, Sonia Goncalves, Ewan Harrison, David K. Jackson, Ian Johnston, Dominic Kwiatkowski, Cordelia Langford, John Sillitoe on behalf of the Wellcome Sanger Institute COVID-19 Surveillance Team
EPI_ISL_675358, EPI_ISL_675359, EPI_ISL_675360, EPI_ISL_675361, EPI_ISL_675362, EPI_ISL_675363	Lighthouse Lab in Alderley Park	Wellcome Sanger Institute for the COVID-19 Genomics UK (COG-UK) Consortium	Jacquelyn Wynn, Mairead Hyland, The Lighthouse Lab in Alderley Park and Alex Alderton, Roberto Amato, Sonia Goncalves, Ewan Harrison, David K. Jackson, Ian Johnston, Dominic Kwiatkowski, Cordelia Langford, John Sillitoe on behalf of the Wellcome Sanger Institute COVID-19 Surveillance Team
EPI_ISL_675364	Lighthouse Lab in Milton Keynes	Wellcome Sanger Institute for the COVID-19 Genomics UK (COG-UK) Consortium	The Lighthouse Lab in Milton Keynes and Alex Alderton, Roberto Amato, Sonia Goncalves, Ewan Harrison, David K. Jackson, Ian Johnston, Dominic Kwiatkowski, Cordelia Langford, John Sillitoe on behalf of the Wellcome Sanger Institute COVID-19 Surveillance Team
EPI_ISL_675365, EPI_ISL_675366, EPI_ISL_675367	Lighthouse Lab in Alderley Park	Wellcome Sanger Institute for the COVID-19 Genomics UK (COG-UK) Consortium	Jacquelyn Wynn, Mairead Hyland, The Lighthouse Lab in Alderley Park and Alex Alderton, Roberto Amato, Sonia Goncalves, Ewan Harrison, David K. Jackson, Ian Johnston, Dominic Kwiatkowski, Cordelia Langford, John Sillitoe on behalf of the Wellcome Sanger Institute COVID-19 Surveillance Team
EPI_ISL_675368	Lighthouse Lab in Milton Keynes	Wellcome Sanger Institute for the COVID-19 Genomics UK (COG-UK) Consortium	The Lighthouse Lab in Milton Keynes and Alex Alderton, Roberto Amato, Sonia Goncalves, Ewan Harrison, David K. Jackson, Ian Johnston, Dominic Kwiatkowski, Cordelia Langford, John Sillitoe on behalf of the Wellcome Sanger Institute COVID-19 Surveillance Team
EPI_ISL_675369, EPI_ISL_675370	Lighthouse Lab in Alderley Park	Wellcome Sanger Institute for the COVID-19 Genomics UK (COG-UK) Consortium	Jacquelyn Wynn, Mairead Hyland, The Lighthouse Lab in Alderley Park and Alex Alderton, Roberto Amato, Sonia Goncalves, Ewan Harrison, David K. Jackson, Ian Johnston, Dominic Kwiatkowski, Cordelia Langford, John Sillitoe on behalf of the Wellcome Sanger Institute COVID-19 Surveillance Team
EPI_ISL_675371	Lighthouse Lab in Milton Keynes	Wellcome Sanger Institute for the COVID-19 Genomics UK (COG-UK) Consortium	The Lighthouse Lab in Milton Keynes and Alex Alderton, Roberto Amato, Sonia Goncalves, Ewan Harrison, David K. Jackson, Ian Johnston, Dominic Kwiatkowski, Cordelia Langford, John Sillitoe on behalf of the Wellcome Sanger Institute COVID-19 Surveillance Team
EPI_ISL_675372, EPI_ISL_675373, EPI_ISL_675374, EPI_ISL_675375, EPI_ISL_675376, EPI_ISL_675377, EPI_ISL_675378, EPI_ISL_675379, EPI_ISL_675380, EPI_ISL_675381, EPI_ISL_675382, EPI_ISL_675383, EPI_ISL_675384, EPI_ISL_675385			
see above	Lighthouse Lab in Alderley Park	Wellcome Sanger Institute for the COVID-19 Genomics UK (COG-UK) Consortium	Jacquelyn Wynn, Mairead Hyland, The Lighthouse Lab in Alderley Park and Alex Alderton, Roberto Amato, Sonia Goncalves, Ewan Harrison, David K. Jackson, Ian Johnston, Dominic Kwiatkowski, Cordelia Langford, John Sillitoe on behalf of the Wellcome Sanger Institute COVID-19 Surveillance Team
EPI_ISL_675386	Lighthouse Lab in Milton Keynes	Wellcome Sanger Institute for the COVID-19 Genomics UK (COG-UK) Consortium	The Lighthouse Lab in Milton Keynes and Alex Alderton, Roberto Amato, Sonia Goncalves, Ewan Harrison, David K. Jackson, Ian Johnston, Dominic Kwiatkowski, Cordelia Langford, John Sillitoe on behalf of the Wellcome Sanger Institute COVID-19 Surveillance Team
EPI_ISL_675387, EPI_ISL_675388	Lighthouse Lab in Alderley Park	Wellcome Sanger Institute for the COVID-19 Genomics UK (COG-UK) Consortium	Jacquelyn Wynn, Mairead Hyland, The Lighthouse Lab in Alderley Park and Alex Alderton, Roberto Amato, Sonia Goncalves, Ewan Harrison, David K. Jackson, Ian Johnston, Dominic Kwiatkowski, Cordelia Langford, John Sillitoe on behalf of the Wellcome Sanger Institute COVID-19 Surveillance Team
EPI_ISL_675389	Lighthouse Lab in Milton Keynes	Wellcome Sanger Institute for the COVID-19 Genomics UK (COG-UK) Consortium	The Lighthouse Lab in Milton Keynes and Alex Alderton, Roberto Amato, Sonia Goncalves, Ewan Harrison, David K. Jackson, Ian Johnston, Dominic Kwiatkowski, Cordelia Langford, John Sillitoe on behalf of the Wellcome Sanger Institute COVID-19 Surveillance Team
EPI_ISL_675390, EPI_ISL_675391, EPI_ISL_675392	Lighthouse Lab in Alderley Park	Wellcome Sanger Institute for the COVID-19 Genomics UK (COG-UK) Consortium	Jacquelyn Wynn, Mairead Hyland, The Lighthouse Lab in Alderley Park and Alex Alderton, Roberto Amato, Sonia Goncalves, Ewan Harrison, David K. Jackson, Ian Johnston, Dominic Kwiatkowski, Cordelia Langford, John Sillitoe on behalf of the Wellcome Sanger Institute COVID-19 Surveillance Team
EPI_ISL_675393	Lighthouse Lab in Milton Keynes	Wellcome Sanger Institute for the COVID-19 Genomics UK (COG-UK) Consortium	The Lighthouse Lab in Milton Keynes and Alex Alderton, Roberto Amato, Sonia Goncalves, Ewan Harrison, David K. Jackson, Ian Johnston, Dominic Kwiatkowski, Cordelia Langford, John Sillitoe on behalf of the Wellcome Sanger Institute COVID-19 Surveillance Team
EPI_ISL_675394, EPI_ISL_675395, EPI_ISL_675396	Lighthouse Lab in Alderley Park	Wellcome Sanger Institute for the COVID-19 Genomics UK (COG-UK) Consortium	Jacquelyn Wynn, Mairead Hyland, The Lighthouse Lab in Alderley Park and Alex Alderton, Roberto Amato, Sonia Goncalves, Ewan Harrison, David K. Jackson, Ian Johnston, Dominic Kwiatkowski, Cordelia Langford, John Sillitoe on behalf of the Wellcome Sanger Institute COVID-19 Surveillance Team
EPI_ISL_675397	Lighthouse Lab in Milton Keynes	Wellcome Sanger Institute for the COVID-19 Genomics UK (COG-UK) Consortium	The Lighthouse Lab in Milton Keynes and Alex Alderton, Roberto Amato, Sonia Goncalves, Ewan Harrison, David K. Jackson, Ian Johnston, Dominic Kwiatkowski, Cordelia Langford, John Sillitoe on behalf of the Wellcome Sanger Institute COVID-19 Surveillance Team
EPI_ISL_675398	Lighthouse Lab in Alderley Park	Wellcome Sanger Institute for the COVID-19 Genomics UK (COG-UK) Consortium	Jacquelyn Wynn, Mairead Hyland, The Lighthouse Lab in Alderley Park and Alex Alderton, Roberto Amato, Sonia Goncalves, Ewan Harrison, David K. Jackson, Ian Johnston, Dominic Kwiatkowski, Cordelia Langford, John Sillitoe on behalf of the Wellcome Sanger Institute COVID-19 Surveillance Team
EPI_ISL_675399	Lighthouse Lab in Milton Keynes	Wellcome Sanger Institute for the COVID-19 Genomics UK (COG-UK) Consortium	The Lighthouse Lab in Milton Keynes and Alex Alderton, Roberto Amato, Sonia Goncalves, Ewan Harrison, David K. Jackson, Ian Johnston, Dominic Kwiatkowski, Cordelia Langford, John Sillitoe on behalf of the Wellcome Sanger Institute COVID-19 Surveillance Team
EPI_ISL_675400, EPI_ISL_675401, EPI_ISL_675402, EPI_ISL_675403	Lighthouse Lab in Alderley Park	Wellcome Sanger Institute for the COVID-19 Genomics UK (COG-UK) Consortium	Jacquelyn Wynn, Mairead Hyland, The Lighthouse Lab in Alderley Park and Alex Alderton, Roberto Amato, Sonia Goncalves, Ewan Harrison, David K. Jackson, Ian Johnston, Dominic Kwiatkowski, Cordelia Langford, John Sillitoe on behalf of the Wellcome Sanger Institute COVID-19 Surveillance Team
EPI_ISL_675404	Lighthouse Lab in Milton Keynes	Wellcome Sanger Institute for the COVID-19 Genomics UK (COG-UK) Consortium	The Lighthouse Lab in Milton Keynes and Alex Alderton, Roberto Amato, Sonia Goncalves, Ewan Harrison, David K. Jackson, Ian Johnston, Dominic Kwiatkowski, Cordelia Langford, John Sillitoe on behalf of the Wellcome Sanger Institute COVID-19 Surveillance Team
EPI_ISL_675405, EPI_ISL_675406, EPI_ISL_675407, EPI_ISL_675408, EPI_ISL_675409, EPI_ISL_675410, EPI_ISL_675411, EPI_ISL_675412, EPI_ISL_675413, EPI_ISL_675414, EPI_ISL_675415, EPI_ISL_675416			
see above	Lighthouse Lab in Alderley Park	Wellcome Sanger Institute for the COVID-19 Genomics UK (COG-UK) Consortium	Jacquelyn Wynn, Mairead Hyland, The Lighthouse Lab in Alderley Park and Alex Alderton, Roberto Amato, Sonia Goncalves, Ewan Harrison, David K. Jackson, Ian Johnston, Dominic Kwiatkowski, Cordelia Langford, John Sillitoe on behalf of the Wellcome Sanger Institute COVID-19 Surveillance Team
EPI_ISL_675417	Lighthouse Lab in Milton Keynes	Wellcome Sanger Institute for the COVID-19 Genomics UK (COG-UK) Consortium	The Lighthouse Lab in Milton Keynes and Alex Alderton, Roberto Amato, Sonia Goncalves, Ewan Harrison, David K. Jackson, Ian Johnston, Dominic Kwiatkowski, Cordelia Langford, John Sillitoe on behalf of the Wellcome Sanger Institute COVID-19 Surveillance Team
EPI_ISL_675418, EPI_ISL_675419	Lighthouse Lab in Alderley Park	Wellcome Sanger Institute for the COVID-19 Genomics UK (COG-UK) Consortium	Jacquelyn Wynn, Mairead Hyland, The Lighthouse Lab in Alderley Park and Alex Alderton, Roberto Amato, Sonia Goncalves, Ewan Harrison, David K. Jackson, Ian Johnston, Dominic Kwiatkowski, Cordelia Langford, John Sillitoe on behalf of the Wellcome Sanger Institute COVID-19 Surveillance Team

EPI_ISL_675420	Lighthouse Lab in Milton Keynes	Wellcome Sanger Institute for the COVID-19 Genomics UK (COG-UK) Consortium	The Lighthouse Lab in Milton Keynes and Alex Alderton, Roberto Amato, Sonia Goncalves, Ewan Harrison, David K. Jackson, Ian Johnston, Dominic Kwiatkowski, Cordelia Langford, John Sillitoe on behalf of the Wellcome Sanger Institute COVID-19 Surveillance Team
EPI_ISL_675421, EPI_ISL_675422	Lighthouse Lab in Alderley Park	Wellcome Sanger Institute for the COVID-19 Genomics UK (COG-UK) Consortium	Jacquelyn Wynn, Mairead Hyland, The Lighthouse Lab in Alderley Park and Alex Alderton, Roberto Amato, Sonia Goncalves, Ewan Harrison, David K. Jackson, Ian Johnston, Dominic Kwiatkowski, Cordelia Langford, John Sillitoe on behalf of the Wellcome Sanger Institute COVID-19 Surveillance Team
EPI_ISL_675423, EPI_ISL_675424	Lighthouse Lab in Milton Keynes	Wellcome Sanger Institute for the COVID-19 Genomics UK (COG-UK) Consortium	The Lighthouse Lab in Milton Keynes and Alex Alderton, Roberto Amato, Sonia Goncalves, Ewan Harrison, David K. Jackson, Ian Johnston, Dominic Kwiatkowski, Cordelia Langford, John Sillitoe on behalf of the Wellcome Sanger Institute COVID-19 Surveillance Team
EPI_ISL_675425, EPI_ISL_675426, EPI_ISL_675427, EPI_ISL_675428, EPI_ISL_675429, EPI_ISL_675430	Lighthouse Lab in Alderley Park	Wellcome Sanger Institute for the COVID-19 Genomics UK (COG-UK) Consortium	Jacquelyn Wynn, Mairead Hyland, The Lighthouse Lab in Alderley Park and Alex Alderton, Roberto Amato, Sonia Goncalves, Ewan Harrison, David K. Jackson, Ian Johnston, Dominic Kwiatkowski, Cordelia Langford, John Sillitoe on behalf of the Wellcome Sanger Institute COVID-19 Surveillance Team
EPI_ISL_675431, EPI_ISL_675432	Lighthouse Lab in Milton Keynes	Wellcome Sanger Institute for the COVID-19 Genomics UK (COG-UK) Consortium	The Lighthouse Lab in Milton Keynes and Alex Alderton, Roberto Amato, Sonia Goncalves, Ewan Harrison, David K. Jackson, Ian Johnston, Dominic Kwiatkowski, Cordelia Langford, John Sillitoe on behalf of the Wellcome Sanger Institute COVID-19 Surveillance Team
EPI_ISL_675433, EPI_ISL_675434, EPI_ISL_675435	Lighthouse Lab in Alderley Park	Wellcome Sanger Institute for the COVID-19 Genomics UK (COG-UK) Consortium	Jacquelyn Wynn, Mairead Hyland, The Lighthouse Lab in Alderley Park and Alex Alderton, Roberto Amato, Sonia Goncalves, Ewan Harrison, David K. Jackson, Ian Johnston, Dominic Kwiatkowski, Cordelia Langford, John Sillitoe on behalf of the Wellcome Sanger Institute COVID-19 Surveillance Team
EPI_ISL_675436	Lighthouse Lab in Milton Keynes	Wellcome Sanger Institute for the COVID-19 Genomics UK (COG-UK) Consortium	The Lighthouse Lab in Milton Keynes and Alex Alderton, Roberto Amato, Sonia Goncalves, Ewan Harrison, David K. Jackson, Ian Johnston, Dominic Kwiatkowski, Cordelia Langford, John Sillitoe on behalf of the Wellcome Sanger Institute COVID-19 Surveillance Team
EPI_ISL_675437, EPI_ISL_675438	Lighthouse Lab in Alderley Park	Wellcome Sanger Institute for the COVID-19 Genomics UK (COG-UK) Consortium	Jacquelyn Wynn, Mairead Hyland, The Lighthouse Lab in Alderley Park and Alex Alderton, Roberto Amato, Sonia Goncalves, Ewan Harrison, David K. Jackson, Ian Johnston, Dominic Kwiatkowski, Cordelia Langford, John Sillitoe on behalf of the Wellcome Sanger Institute COVID-19 Surveillance Team
EPI_ISL_675439	Lighthouse Lab in Milton Keynes	Wellcome Sanger Institute for the COVID-19 Genomics UK (COG-UK) Consortium	The Lighthouse Lab in Milton Keynes and Alex Alderton, Roberto Amato, Sonia Goncalves, Ewan Harrison, David K. Jackson, Ian Johnston, Dominic Kwiatkowski, Cordelia Langford, John Sillitoe on behalf of the Wellcome Sanger Institute COVID-19 Surveillance Team
EPI_ISL_675440, EPI_ISL_675441, EPI_ISL_675442	Lighthouse Lab in Alderley Park	Wellcome Sanger Institute for the COVID-19 Genomics UK (COG-UK) Consortium	Jacquelyn Wynn, Mairead Hyland, The Lighthouse Lab in Alderley Park and Alex Alderton, Roberto Amato, Sonia Goncalves, Ewan Harrison, David K. Jackson, Ian Johnston, Dominic Kwiatkowski, Cordelia Langford, John Sillitoe on behalf of the Wellcome Sanger Institute COVID-19 Surveillance Team
EPI_ISL_675443, EPI_ISL_675444	Lighthouse Lab in Milton Keynes	Wellcome Sanger Institute for the COVID-19 Genomics UK (COG-UK) Consortium	The Lighthouse Lab in Milton Keynes and Alex Alderton, Roberto Amato, Sonia Goncalves, Ewan Harrison, David K. Jackson, Ian Johnston, Dominic Kwiatkowski, Cordelia Langford, John Sillitoe on behalf of the Wellcome Sanger Institute COVID-19 Surveillance Team
EPI_ISL_675445	Lighthouse Lab in Alderley Park	Wellcome Sanger Institute for the COVID-19 Genomics UK (COG-UK) Consortium	Jacquelyn Wynn, Mairead Hyland, The Lighthouse Lab in Alderley Park and Alex Alderton, Roberto Amato, Sonia Goncalves, Ewan Harrison, David K. Jackson, Ian Johnston, Dominic Kwiatkowski, Cordelia Langford, John Sillitoe on behalf of the Wellcome Sanger Institute COVID-19 Surveillance Team
EPI_ISL_675446	Lighthouse Lab in Milton Keynes	Wellcome Sanger Institute for the COVID-19 Genomics UK (COG-UK) Consortium	The Lighthouse Lab in Milton Keynes and Alex Alderton, Roberto Amato, Sonia Goncalves, Ewan Harrison, David K. Jackson, Ian Johnston, Dominic Kwiatkowski, Cordelia Langford, John Sillitoe on behalf of the Wellcome Sanger Institute COVID-19 Surveillance Team
EPI_ISL_675447, EPI_ISL_675448, EPI_ISL_675449, EPI_ISL_675450, EPI_ISL_675451, EPI_ISL_675452, EPI_ISL_675453, EPI_ISL_675454, EPI_ISL_675455, EPI_ISL_675456, EPI_ISL_675457, EPI_ISL_675458, EPI_ISL_675459, EPI_ISL_675460, EPI_ISL_675461			
see above	Lighthouse Lab in Alderley Park	Wellcome Sanger Institute for the COVID-19 Genomics UK (COG-UK) Consortium	Jacquelyn Wynn, Mairead Hyland, The Lighthouse Lab in Alderley Park and Alex Alderton, Roberto Amato, Sonia Goncalves, Ewan Harrison, David K. Jackson, Ian Johnston, Dominic Kwiatkowski, Cordelia Langford, John Sillitoe on behalf of the Wellcome Sanger Institute COVID-19 Surveillance Team
EPI_ISL_675462	Lighthouse Lab in Milton Keynes	Wellcome Sanger Institute for the COVID-19 Genomics UK (COG-UK) Consortium	The Lighthouse Lab in Milton Keynes and Alex Alderton, Roberto Amato, Sonia Goncalves, Ewan Harrison, David K. Jackson, Ian Johnston, Dominic Kwiatkowski, Cordelia Langford, John Sillitoe on behalf of the Wellcome Sanger Institute COVID-19 Surveillance Team
EPI_ISL_675463, EPI_ISL_675464, EPI_ISL_675465, EPI_ISL_675466	Lighthouse Lab in Alderley Park	Wellcome Sanger Institute for the COVID-19 Genomics UK (COG-UK) Consortium	Jacquelyn Wynn, Mairead Hyland, The Lighthouse Lab in Alderley Park and Alex Alderton, Roberto Amato, Sonia Goncalves, Ewan Harrison, David K. Jackson, Ian Johnston, Dominic Kwiatkowski, Cordelia Langford, John Sillitoe on behalf of the Wellcome Sanger Institute COVID-19 Surveillance Team
EPI_ISL_675467	Lighthouse Lab in Milton Keynes	Wellcome Sanger Institute for the COVID-19 Genomics UK (COG-UK) Consortium	The Lighthouse Lab in Milton Keynes and Alex Alderton, Roberto Amato, Sonia Goncalves, Ewan Harrison, David K. Jackson, Ian Johnston, Dominic Kwiatkowski, Cordelia Langford, John Sillitoe on behalf of the Wellcome Sanger Institute COVID-19 Surveillance Team
EPI_ISL_675468, EPI_ISL_675469, EPI_ISL_675470, EPI_ISL_675471	Lighthouse Lab in Alderley Park	Wellcome Sanger Institute for the COVID-19 Genomics UK (COG-UK) Consortium	Jacquelyn Wynn, Mairead Hyland, The Lighthouse Lab in Alderley Park and Alex Alderton, Roberto Amato, Sonia Goncalves, Ewan Harrison, David K. Jackson, Ian Johnston, Dominic Kwiatkowski, Cordelia Langford, John Sillitoe on behalf of the Wellcome Sanger Institute COVID-19 Surveillance Team
EPI_ISL_675472	Lighthouse Lab in Milton Keynes	Wellcome Sanger Institute for the COVID-19 Genomics UK (COG-UK) Consortium	The Lighthouse Lab in Milton Keynes and Alex Alderton, Roberto Amato, Sonia Goncalves, Ewan Harrison, David K. Jackson, Ian Johnston, Dominic Kwiatkowski, Cordelia Langford, John Sillitoe on behalf of the Wellcome Sanger Institute COVID-19 Surveillance Team
EPI_ISL_675473, EPI_ISL_675474	Lighthouse Lab in Alderley Park	Wellcome Sanger Institute for the COVID-19 Genomics UK (COG-UK) Consortium	Jacquelyn Wynn, Mairead Hyland, The Lighthouse Lab in Alderley Park and Alex Alderton, Roberto Amato, Sonia Goncalves, Ewan Harrison, David K. Jackson, Ian Johnston, Dominic Kwiatkowski, Cordelia Langford, John Sillitoe on behalf of the Wellcome Sanger Institute COVID-19 Surveillance Team
EPI_ISL_675475	Lighthouse Lab in Milton Keynes	Wellcome Sanger Institute for the COVID-19 Genomics UK (COG-UK) Consortium	The Lighthouse Lab in Milton Keynes and Alex Alderton, Roberto Amato, Sonia Goncalves, Ewan Harrison, David K. Jackson, Ian Johnston, Dominic Kwiatkowski, Cordelia Langford, John Sillitoe on behalf of the Wellcome Sanger Institute COVID-19 Surveillance Team
EPI_ISL_675476, EPI_ISL_675477, EPI_ISL_675478, EPI_ISL_675479	Lighthouse Lab in Alderley Park	Wellcome Sanger Institute for the COVID-19 Genomics UK (COG-UK) Consortium	Jacquelyn Wynn, Mairead Hyland, The Lighthouse Lab in Alderley Park and Alex Alderton, Roberto Amato, Sonia Goncalves, Ewan Harrison, David K. Jackson, Ian Johnston, Dominic Kwiatkowski, Cordelia Langford, John Sillitoe on behalf of the Wellcome Sanger Institute COVID-19 Surveillance Team
EPI_ISL_675480	Lighthouse Lab in Milton Keynes	Wellcome Sanger Institute for the COVID-19 Genomics UK (COG-UK) Consortium	The Lighthouse Lab in Milton Keynes and Alex Alderton, Roberto Amato, Sonia Goncalves, Ewan Harrison, David K. Jackson, Ian Johnston, Dominic Kwiatkowski, Cordelia Langford, John Sillitoe on behalf of the Wellcome Sanger Institute COVID-19 Surveillance Team
EPI_ISL_675481, EPI_ISL_675482, EPI_ISL_675483, EPI_ISL_675484	Lighthouse Lab in Alderley Park	Wellcome Sanger Institute for the COVID-19 Genomics UK (COG-UK) Consortium	Jacquelyn Wynn, Mairead Hyland, The Lighthouse Lab in Alderley Park and Alex Alderton, Roberto Amato, Sonia Goncalves, Ewan Harrison, David K. Jackson, Ian Johnston, Dominic Kwiatkowski, Cordelia Langford, John Sillitoe on behalf of the Wellcome Sanger Institute COVID-19 Surveillance Team
EPI_ISL_675485, EPI_ISL_675486	Lighthouse Lab in Milton Keynes	Wellcome Sanger Institute for the COVID-19 Genomics UK (COG-UK) Consortium	The Lighthouse Lab in Milton Keynes and Alex Alderton, Roberto Amato, Sonia Goncalves, Ewan Harrison, David K. Jackson, Ian Johnston, Dominic Kwiatkowski, Cordelia Langford, John Sillitoe on behalf of the Wellcome Sanger Institute COVID-19 Surveillance Team
EPI_ISL_675487, EPI_ISL_675488, EPI_ISL_675489	Lighthouse Lab in Alderley Park	Wellcome Sanger Institute for the COVID-19 Genomics UK (COG-UK) Consortium	Jacquelyn Wynn, Mairead Hyland, The Lighthouse Lab in Alderley Park and Alex Alderton, Roberto Amato, Sonia Goncalves, Ewan Harrison, David K. Jackson, Ian Johnston, Dominic Kwiatkowski, Cordelia Langford, John Sillitoe on behalf of the Wellcome Sanger Institute COVID-19 Surveillance Team
EPI_ISL_675490	Lighthouse Lab in Milton Keynes	Wellcome Sanger Institute for the COVID-19 Genomics UK (COG-UK) Consortium	The Lighthouse Lab in Milton Keynes and Alex Alderton, Roberto Amato, Sonia Goncalves, Ewan Harrison, David K. Jackson, Ian Johnston, Dominic Kwiatkowski, Cordelia Langford, John Sillitoe on behalf of the Wellcome Sanger Institute COVID-19 Surveillance Team
EPI_ISL_675491, EPI_ISL_675492, EPI_ISL_675493, EPI_ISL_675494	Lighthouse Lab in Alderley Park	Wellcome Sanger Institute for the COVID-19 Genomics UK (COG-UK) Consortium	Jacquelyn Wynn, Mairead Hyland, The Lighthouse Lab in Alderley Park and Alex Alderton, Roberto Amato, Sonia Goncalves, Ewan Harrison, David K. Jackson, Ian Johnston, Dominic Kwiatkowski, Cordelia Langford, John Sillitoe on behalf of the Wellcome Sanger Institute COVID-19 Surveillance Team
EPI_ISL_675495, EPI_ISL_675496	Lighthouse Lab in Milton Keynes	Wellcome Sanger Institute for the COVID-19 Genomics UK (COG-UK) Consortium	The Lighthouse Lab in Milton Keynes and Alex Alderton, Roberto Amato, Sonia Goncalves, Ewan Harrison, David K. Jackson, Ian Johnston, Dominic Kwiatkowski, Cordelia Langford, John Sillitoe on behalf of the Wellcome Sanger Institute COVID-19 Surveillance Team
EPI_ISL_675497, EPI_ISL_675498	Lighthouse Lab in Alderley Park	Wellcome Sanger Institute for the COVID-19 Genomics UK (COG-UK) Consortium	Jacquelyn Wynn, Mairead Hyland, The Lighthouse Lab in Alderley Park and Alex Alderton, Roberto Amato, Sonia Goncalves, Ewan Harrison, David K. Jackson, Ian Johnston, Dominic Kwiatkowski, Cordelia Langford, John Sillitoe on behalf of the Wellcome Sanger Institute COVID-19 Surveillance Team
EPI_ISL_675499, EPI_ISL_675500	Lighthouse Lab in Milton Keynes	Wellcome Sanger Institute for the COVID-19 Genomics UK (COG-UK) Consortium	The Lighthouse Lab in Milton Keynes and Alex Alderton, Roberto Amato, Sonia Goncalves, Ewan Harrison, David K. Jackson, Ian Johnston, Dominic Kwiatkowski, Cordelia Langford, John Sillitoe on behalf of the Wellcome Sanger Institute COVID-19 Surveillance Team
EPI_ISL_675501, EPI_ISL_675502	Lighthouse Lab in Alderley Park	Wellcome Sanger Institute for the COVID-19 Genomics UK (COG-UK) Consortium	Jacquelyn Wynn, Mairead Hyland, The Lighthouse Lab in Alderley Park and Alex Alderton, Roberto Amato, Sonia Goncalves, Ewan Harrison, David K. Jackson, Ian Johnston, Dominic Kwiatkowski, Cordelia Langford, John Sillitoe on behalf of the Wellcome Sanger Institute COVID-19 Surveillance Team
EPI_ISL_675503, EPI_ISL_675504, EPI_ISL_675505, EPI_ISL_675506, EPI_ISL_675507	Lighthouse Lab in Milton Keynes	Wellcome Sanger Institute for the COVID-19 Genomics UK (COG-UK) Consortium	The Lighthouse Lab in Milton Keynes and Alex Alderton, Roberto Amato, Sonia Goncalves, Ewan Harrison, David K. Jackson, Ian Johnston, Dominic Kwiatkowski, Cordelia Langford, John Sillitoe on behalf of the Wellcome Sanger Institute COVID-19 Surveillance Team
EPI_ISL_675508, EPI_ISL_675509,	Lighthouse Lab in Alderley Park	Wellcome Sanger Institute for the COVID-19 Genomics UK	Jacquelyn Wynn, Mairead Hyland, The Lighthouse Lab in Alderley Park and Alex Alderton, Roberto Amato, Sonia Goncalves, Ewan Harrison, David K.

EPI_ISL_675510, EPI_ISL_675511, EPI_ISL_675512		(COG-UK) Consortium	Jackson, Ian Johnston, Dominic Kwiatkowski, Cordelia Langford, John Sillitoe on behalf of the Wellcome Sanger Institute COVID-19 Surveillance Team
EPI_ISL_675513	Lighthouse Lab in Milton Keynes	Wellcome Sanger Institute for the COVID-19 Genomics UK (COG-UK) Consortium	The Lighthouse Lab in Milton Keynes and Alex Alderton, Roberto Amato, Sonia Goncalves, Ewan Harrison, David K. Jackson, Ian Johnston, Dominic Kwiatkowski, Cordelia Langford, John Sillitoe on behalf of the Wellcome Sanger Institute COVID-19 Surveillance Team
EPI_ISL_675514, EPI_ISL_675515, EPI_ISL_675517	Lighthouse Lab in Alderley Park	Wellcome Sanger Institute for the COVID-19 Genomics UK (COG-UK) Consortium	Jacquelyn Wynn, Mairead Hyland, The Lighthouse Lab in Alderley Park and Alex Alderton, Roberto Amato, Sonia Goncalves, Ewan Harrison, David K. Jackson, Ian Johnston, Dominic Kwiatkowski, Cordelia Langford, John Sillitoe on behalf of the Wellcome Sanger Institute COVID-19 Surveillance Team
EPI_ISL_675518	Lighthouse Lab in Milton Keynes	Wellcome Sanger Institute for the COVID-19 Genomics UK (COG-UK) Consortium	The Lighthouse Lab in Milton Keynes and Alex Alderton, Roberto Amato, Sonia Goncalves, Ewan Harrison, David K. Jackson, Ian Johnston, Dominic Kwiatkowski, Cordelia Langford, John Sillitoe on behalf of the Wellcome Sanger Institute COVID-19 Surveillance Team
EPI_ISL_675519, EPI_ISL_675520, EPI_ISL_675521, EPI_ISL_675522, EPI_ISL_675523, EPI_ISL_675524, EPI_ISL_675525, EPI_ISL_675526, EPI_ISL_675527, EPI_ISL_675529, EPI_ISL_675530, EPI_ISL_675532			
see above	Lighthouse Lab in Alderley Park	Wellcome Sanger Institute for the COVID-19 Genomics UK (COG-UK) Consortium	Jacquelyn Wynn, Mairead Hyland, The Lighthouse Lab in Alderley Park and Alex Alderton, Roberto Amato, Sonia Goncalves, Ewan Harrison, David K. Jackson, Ian Johnston, Dominic Kwiatkowski, Cordelia Langford, John Sillitoe on behalf of the Wellcome Sanger Institute COVID-19 Surveillance Team
EPI_ISL_675533	Lighthouse Lab in Milton Keynes	Wellcome Sanger Institute for the COVID-19 Genomics UK (COG-UK) Consortium	The Lighthouse Lab in Milton Keynes and Alex Alderton, Roberto Amato, Sonia Goncalves, Ewan Harrison, David K. Jackson, Ian Johnston, Dominic Kwiatkowski, Cordelia Langford, John Sillitoe on behalf of the Wellcome Sanger Institute COVID-19 Surveillance Team
EPI_ISL_675534	Lighthouse Lab in Alderley Park	Wellcome Sanger Institute for the COVID-19 Genomics UK (COG-UK) Consortium	Jacquelyn Wynn, Mairead Hyland, The Lighthouse Lab in Alderley Park and Alex Alderton, Roberto Amato, Sonia Goncalves, Ewan Harrison, David K. Jackson, Ian Johnston, Dominic Kwiatkowski, Cordelia Langford, John Sillitoe on behalf of the Wellcome Sanger Institute COVID-19 Surveillance Team
EPI_ISL_675535	Lighthouse Lab in Milton Keynes	Wellcome Sanger Institute for the COVID-19 Genomics UK (COG-UK) Consortium	The Lighthouse Lab in Milton Keynes and Alex Alderton, Roberto Amato, Sonia Goncalves, Ewan Harrison, David K. Jackson, Ian Johnston, Dominic Kwiatkowski, Cordelia Langford, John Sillitoe on behalf of the Wellcome Sanger Institute COVID-19 Surveillance Team
EPI_ISL_675536, EPI_ISL_675537	Lighthouse Lab in Alderley Park	Wellcome Sanger Institute for the COVID-19 Genomics UK (COG-UK) Consortium	Jacquelyn Wynn, Mairead Hyland, The Lighthouse Lab in Alderley Park and Alex Alderton, Roberto Amato, Sonia Goncalves, Ewan Harrison, David K. Jackson, Ian Johnston, Dominic Kwiatkowski, Cordelia Langford, John Sillitoe on behalf of the Wellcome Sanger Institute COVID-19 Surveillance Team
EPI_ISL_675538	Lighthouse Lab in Milton Keynes	Wellcome Sanger Institute for the COVID-19 Genomics UK (COG-UK) Consortium	The Lighthouse Lab in Milton Keynes and Alex Alderton, Roberto Amato, Sonia Goncalves, Ewan Harrison, David K. Jackson, Ian Johnston, Dominic Kwiatkowski, Cordelia Langford, John Sillitoe on behalf of the Wellcome Sanger Institute COVID-19 Surveillance Team
EPI_ISL_675539	Lighthouse Lab in Alderley Park	Wellcome Sanger Institute for the COVID-19 Genomics UK (COG-UK) Consortium	Jacquelyn Wynn, Mairead Hyland, The Lighthouse Lab in Alderley Park and Alex Alderton, Roberto Amato, Sonia Goncalves, Ewan Harrison, David K. Jackson, Ian Johnston, Dominic Kwiatkowski, Cordelia Langford, John Sillitoe on behalf of the Wellcome Sanger Institute COVID-19 Surveillance Team
EPI_ISL_675540	Lighthouse Lab in Milton Keynes	Wellcome Sanger Institute for the COVID-19 Genomics UK (COG-UK) Consortium	The Lighthouse Lab in Milton Keynes and Alex Alderton, Roberto Amato, Sonia Goncalves, Ewan Harrison, David K. Jackson, Ian Johnston, Dominic Kwiatkowski, Cordelia Langford, John Sillitoe on behalf of the Wellcome Sanger Institute COVID-19 Surveillance Team
EPI_ISL_675541	Lighthouse Lab in Alderley Park	Wellcome Sanger Institute for the COVID-19 Genomics UK (COG-UK) Consortium	Jacquelyn Wynn, Mairead Hyland, The Lighthouse Lab in Alderley Park and Alex Alderton, Roberto Amato, Sonia Goncalves, Ewan Harrison, David K. Jackson, Ian Johnston, Dominic Kwiatkowski, Cordelia Langford, John Sillitoe on behalf of the Wellcome Sanger Institute COVID-19 Surveillance Team
EPI_ISL_675542	Lighthouse Lab in Milton Keynes	Wellcome Sanger Institute for the COVID-19 Genomics UK (COG-UK) Consortium	The Lighthouse Lab in Milton Keynes and Alex Alderton, Roberto Amato, Sonia Goncalves, Ewan Harrison, David K. Jackson, Ian Johnston, Dominic Kwiatkowski, Cordelia Langford, John Sillitoe on behalf of the Wellcome Sanger Institute COVID-19 Surveillance Team
EPI_ISL_675543, EPI_ISL_675544, EPI_ISL_675545, EPI_ISL_675546	Lighthouse Lab in Alderley Park	Wellcome Sanger Institute for the COVID-19 Genomics UK (COG-UK) Consortium	Jacquelyn Wynn, Mairead Hyland, The Lighthouse Lab in Alderley Park and Alex Alderton, Roberto Amato, Sonia Goncalves, Ewan Harrison, David K. Jackson, Ian Johnston, Dominic Kwiatkowski, Cordelia Langford, John Sillitoe on behalf of the Wellcome Sanger Institute COVID-19 Surveillance Team
EPI_ISL_675547	Lighthouse Lab in Milton Keynes	Wellcome Sanger Institute for the COVID-19 Genomics UK (COG-UK) Consortium	The Lighthouse Lab in Milton Keynes and Alex Alderton, Roberto Amato, Sonia Goncalves, Ewan Harrison, David K. Jackson, Ian Johnston, Dominic Kwiatkowski, Cordelia Langford, John Sillitoe on behalf of the Wellcome Sanger Institute COVID-19 Surveillance Team
EPI_ISL_675548, EPI_ISL_675549	Lighthouse Lab in Alderley Park	Wellcome Sanger Institute for the COVID-19 Genomics UK (COG-UK) Consortium	Jacquelyn Wynn, Mairead Hyland, The Lighthouse Lab in Alderley Park and Alex Alderton, Roberto Amato, Sonia Goncalves, Ewan Harrison, David K. Jackson, Ian Johnston, Dominic Kwiatkowski, Cordelia Langford, John Sillitoe on behalf of the Wellcome Sanger Institute COVID-19 Surveillance Team
EPI_ISL_675550	Lighthouse Lab in Milton Keynes	Wellcome Sanger Institute for the COVID-19 Genomics UK (COG-UK) Consortium	The Lighthouse Lab in Milton Keynes and Alex Alderton, Roberto Amato, Sonia Goncalves, Ewan Harrison, David K. Jackson, Ian Johnston, Dominic Kwiatkowski, Cordelia Langford, John Sillitoe on behalf of the Wellcome Sanger Institute COVID-19 Surveillance Team
EPI_ISL_675551, EPI_ISL_675552, EPI_ISL_675553, EPI_ISL_675554	Lighthouse Lab in Alderley Park	Wellcome Sanger Institute for the COVID-19 Genomics UK (COG-UK) Consortium	Jacquelyn Wynn, Mairead Hyland, The Lighthouse Lab in Alderley Park and Alex Alderton, Roberto Amato, Sonia Goncalves, Ewan Harrison, David K. Jackson, Ian Johnston, Dominic Kwiatkowski, Cordelia Langford, John Sillitoe on behalf of the Wellcome Sanger Institute COVID-19 Surveillance Team
EPI_ISL_675555, EPI_ISL_675556	Lighthouse Lab in Milton Keynes	Wellcome Sanger Institute for the COVID-19 Genomics UK (COG-UK) Consortium	The Lighthouse Lab in Milton Keynes and Alex Alderton, Roberto Amato, Sonia Goncalves, Ewan Harrison, David K. Jackson, Ian Johnston, Dominic Kwiatkowski, Cordelia Langford, John Sillitoe on behalf of the Wellcome Sanger Institute COVID-19 Surveillance Team
EPI_ISL_675557	Lighthouse Lab in Alderley Park	Wellcome Sanger Institute for the COVID-19 Genomics UK (COG-UK) Consortium	Jacquelyn Wynn, Mairead Hyland, The Lighthouse Lab in Alderley Park and Alex Alderton, Roberto Amato, Sonia Goncalves, Ewan Harrison, David K. Jackson, Ian Johnston, Dominic Kwiatkowski, Cordelia Langford, John Sillitoe on behalf of the Wellcome Sanger Institute COVID-19 Surveillance Team
EPI_ISL_675558	Lighthouse Lab in Milton Keynes	Wellcome Sanger Institute for the COVID-19 Genomics UK (COG-UK) Consortium	The Lighthouse Lab in Milton Keynes and Alex Alderton, Roberto Amato, Sonia Goncalves, Ewan Harrison, David K. Jackson, Ian Johnston, Dominic Kwiatkowski, Cordelia Langford, John Sillitoe on behalf of the Wellcome Sanger Institute COVID-19 Surveillance Team
EPI_ISL_675559, EPI_ISL_675561, EPI_ISL_675563	Lighthouse Lab in Alderley Park	Wellcome Sanger Institute for the COVID-19 Genomics UK (COG-UK) Consortium	Jacquelyn Wynn, Mairead Hyland, The Lighthouse Lab in Alderley Park and Alex Alderton, Roberto Amato, Sonia Goncalves, Ewan Harrison, David K. Jackson, Ian Johnston, Dominic Kwiatkowski, Cordelia Langford, John Sillitoe on behalf of the Wellcome Sanger Institute COVID-19 Surveillance Team
EPI_ISL_675564, EPI_ISL_675565, EPI_ISL_675566	Lighthouse Lab in Milton Keynes	Wellcome Sanger Institute for the COVID-19 Genomics UK (COG-UK) Consortium	The Lighthouse Lab in Milton Keynes and Alex Alderton, Roberto Amato, Sonia Goncalves, Ewan Harrison, David K. Jackson, Ian Johnston, Dominic Kwiatkowski, Cordelia Langford, John Sillitoe on behalf of the Wellcome Sanger Institute COVID-19 Surveillance Team
EPI_ISL_675567, EPI_ISL_675568	Lighthouse Lab in Alderley Park	Wellcome Sanger Institute for the COVID-19 Genomics UK (COG-UK) Consortium	Jacquelyn Wynn, Mairead Hyland, The Lighthouse Lab in Alderley Park and Alex Alderton, Roberto Amato, Sonia Goncalves, Ewan Harrison, David K. Jackson, Ian Johnston, Dominic Kwiatkowski, Cordelia Langford, John Sillitoe on behalf of the Wellcome Sanger Institute COVID-19 Surveillance Team
EPI_ISL_675569	Lighthouse Lab in Milton Keynes	Wellcome Sanger Institute for the COVID-19 Genomics UK (COG-UK) Consortium	The Lighthouse Lab in Milton Keynes and Alex Alderton, Roberto Amato, Sonia Goncalves, Ewan Harrison, David K. Jackson, Ian Johnston, Dominic Kwiatkowski, Cordelia Langford, John Sillitoe on behalf of the Wellcome Sanger Institute COVID-19 Surveillance Team
EPI_ISL_675570, EPI_ISL_675571, EPI_ISL_675573, EPI_ISL_675574, EPI_ISL_675575	Lighthouse Lab in Alderley Park	Wellcome Sanger Institute for the COVID-19 Genomics UK (COG-UK) Consortium	Jacquelyn Wynn, Mairead Hyland, The Lighthouse Lab in Alderley Park and Alex Alderton, Roberto Amato, Sonia Goncalves, Ewan Harrison, David K. Jackson, Ian Johnston, Dominic Kwiatkowski, Cordelia Langford, John Sillitoe on behalf of the Wellcome Sanger Institute COVID-19 Surveillance Team
EPI_ISL_675576	Lighthouse Lab in Milton Keynes	Wellcome Sanger Institute for the COVID-19 Genomics UK (COG-UK) Consortium	The Lighthouse Lab in Milton Keynes and Alex Alderton, Roberto Amato, Sonia Goncalves, Ewan Harrison, David K. Jackson, Ian Johnston, Dominic Kwiatkowski, Cordelia Langford, John Sillitoe on behalf of the Wellcome Sanger Institute COVID-19 Surveillance Team
EPI_ISL_675577	Lighthouse Lab in Alderley Park	Wellcome Sanger Institute for the COVID-19 Genomics UK (COG-UK) Consortium	Jacquelyn Wynn, Mairead Hyland, The Lighthouse Lab in Alderley Park and Alex Alderton, Roberto Amato, Sonia Goncalves, Ewan Harrison, David K. Jackson, Ian Johnston, Dominic Kwiatkowski, Cordelia Langford, John Sillitoe on behalf of the Wellcome Sanger Institute COVID-19 Surveillance Team
EPI_ISL_675578	Lighthouse Lab in Milton Keynes	Wellcome Sanger Institute for the COVID-19 Genomics UK (COG-UK) Consortium	The Lighthouse Lab in Milton Keynes and Alex Alderton, Roberto Amato, Sonia Goncalves, Ewan Harrison, David K. Jackson, Ian Johnston, Dominic Kwiatkowski, Cordelia Langford, John Sillitoe on behalf of the Wellcome Sanger Institute COVID-19 Surveillance Team
EPI_ISL_675579, EPI_ISL_675580, EPI_ISL_675581, EPI_ISL_675582, EPI_ISL_675583, EPI_ISL_675584, EPI_ISL_675585, EPI_ISL_675586, EPI_ISL_675587, EPI_ISL_675588, EPI_ISL_675589, EPI_ISL_675590, EPI_ISL_675591, EPI_ISL_675592, EPI_ISL_675593, EPI_ISL_675594, EPI_ISL_675595			
see above	Lighthouse Lab in Alderley Park	Wellcome Sanger Institute for the COVID-19 Genomics UK (COG-UK) Consortium	Jacquelyn Wynn, Mairead Hyland, The Lighthouse Lab in Alderley Park and Alex Alderton, Roberto Amato, Sonia Goncalves, Ewan Harrison, David K. Jackson, Ian Johnston, Dominic Kwiatkowski, Cordelia Langford, John Sillitoe on behalf of the Wellcome Sanger Institute COVID-19 Surveillance Team
EPI_ISL_675596	Lighthouse Lab in Milton Keynes	Wellcome Sanger Institute for the COVID-19 Genomics UK (COG-UK) Consortium	The Lighthouse Lab in Milton Keynes and Alex Alderton, Roberto Amato, Sonia Goncalves, Ewan Harrison, David K. Jackson, Ian Johnston, Dominic Kwiatkowski, Cordelia Langford, John Sillitoe on behalf of the Wellcome Sanger Institute COVID-19 Surveillance Team
EPI_ISL_675597, EPI_ISL_675598, EPI_ISL_675599, EPI_ISL_675600	Lighthouse Lab in Alderley Park	Wellcome Sanger Institute for the COVID-19 Genomics UK (COG-UK) Consortium	Jacquelyn Wynn, Mairead Hyland, The Lighthouse Lab in Alderley Park and Alex Alderton, Roberto Amato, Sonia Goncalves, Ewan Harrison, David K. Jackson, Ian Johnston, Dominic Kwiatkowski, Cordelia Langford, John Sillitoe on behalf of the Wellcome Sanger Institute COVID-19 Surveillance Team
EPI_ISL_675601, EPI_ISL_675602	Lighthouse Lab in Milton Keynes	Wellcome Sanger Institute for the COVID-19 Genomics UK	The Lighthouse Lab in Milton Keynes and Alex Alderton, Roberto Amato, Sonia Goncalves, Ewan Harrison, David K. Jackson, Ian Johnston, Dominic

		(COG-UK) Consortium	Kwiatkowski, Cordelia Langford, John Sillitoe on behalf of the Wellcome Sanger Institute COVID-19 Surveillance Team
EPI_ISL_675603, EPI_ISL_675604, EPI_ISL_675605	Lighthouse Lab in Alderley Park	Wellcome Sanger Institute for the COVID-19 Genomics UK (COG-UK) Consortium	Jacquelyn Wynn, Mairead Hyland, The Lighthouse Lab in Alderley Park and Alex Alderton, Roberto Amato, Sonia Goncalves, Ewan Harrison, David K. Jackson, Ian Johnston, Dominic Kwiatkowski, Cordelia Langford, John Sillitoe on behalf of the Wellcome Sanger Institute COVID-19 Surveillance Team
EPI_ISL_675606	Lighthouse Lab in Milton Keynes	Wellcome Sanger Institute for the COVID-19 Genomics UK (COG-UK) Consortium	The Lighthouse Lab in Milton Keynes and Alex Alderton, Roberto Amato, Sonia Goncalves, Ewan Harrison, David K. Jackson, Ian Johnston, Dominic Kwiatkowski, Cordelia Langford, John Sillitoe on behalf of the Wellcome Sanger Institute COVID-19 Surveillance Team
EPI_ISL_675607, EPI_ISL_675608, EPI_ISL_675609, EPI_ISL_675610, EPI_ISL_675611, EPI_ISL_675612, EPI_ISL_675613, EPI_ISL_675614, EPI_ISL_675615, EPI_ISL_675616, EPI_ISL_675617, EPI_ISL_675618, EPI_ISL_675619			
see above	Lighthouse Lab in Alderley Park	Wellcome Sanger Institute for the COVID-19 Genomics UK (COG-UK) Consortium	Jacquelyn Wynn, Mairead Hyland, The Lighthouse Lab in Alderley Park and Alex Alderton, Roberto Amato, Sonia Goncalves, Ewan Harrison, David K. Jackson, Ian Johnston, Dominic Kwiatkowski, Cordelia Langford, John Sillitoe on behalf of the Wellcome Sanger Institute COVID-19 Surveillance Team
EPI_ISL_675621	Lighthouse Lab in Milton Keynes	Wellcome Sanger Institute for the COVID-19 Genomics UK (COG-UK) Consortium	The Lighthouse Lab in Milton Keynes and Alex Alderton, Roberto Amato, Sonia Goncalves, Ewan Harrison, David K. Jackson, Ian Johnston, Dominic Kwiatkowski, Cordelia Langford, John Sillitoe on behalf of the Wellcome Sanger Institute COVID-19 Surveillance Team
EPI_ISL_675622, EPI_ISL_675623, EPI_ISL_675624, EPI_ISL_675625, EPI_ISL_675626, EPI_ISL_675627, EPI_ISL_675628, EPI_ISL_675629, EPI_ISL_675630	Lighthouse Lab in Alderley Park	Wellcome Sanger Institute for the COVID-19 Genomics UK (COG-UK) Consortium	Jacquelyn Wynn, Mairead Hyland, The Lighthouse Lab in Alderley Park and Alex Alderton, Roberto Amato, Sonia Goncalves, Ewan Harrison, David K. Jackson, Ian Johnston, Dominic Kwiatkowski, Cordelia Langford, John Sillitoe on behalf of the Wellcome Sanger Institute COVID-19 Surveillance Team
EPI_ISL_675631	Lighthouse Lab in Milton Keynes	Wellcome Sanger Institute for the COVID-19 Genomics UK (COG-UK) Consortium	The Lighthouse Lab in Milton Keynes and Alex Alderton, Roberto Amato, Sonia Goncalves, Ewan Harrison, David K. Jackson, Ian Johnston, Dominic Kwiatkowski, Cordelia Langford, John Sillitoe on behalf of the Wellcome Sanger Institute COVID-19 Surveillance Team
EPI_ISL_675632, EPI_ISL_675633	Lighthouse Lab in Alderley Park	Wellcome Sanger Institute for the COVID-19 Genomics UK (COG-UK) Consortium	Jacquelyn Wynn, Mairead Hyland, The Lighthouse Lab in Alderley Park and Alex Alderton, Roberto Amato, Sonia Goncalves, Ewan Harrison, David K. Jackson, Ian Johnston, Dominic Kwiatkowski, Cordelia Langford, John Sillitoe on behalf of the Wellcome Sanger Institute COVID-19 Surveillance Team
EPI_ISL_675634	Lighthouse Lab in Milton Keynes	Wellcome Sanger Institute for the COVID-19 Genomics UK (COG-UK) Consortium	The Lighthouse Lab in Milton Keynes and Alex Alderton, Roberto Amato, Sonia Goncalves, Ewan Harrison, David K. Jackson, Ian Johnston, Dominic Kwiatkowski, Cordelia Langford, John Sillitoe on behalf of the Wellcome Sanger Institute COVID-19 Surveillance Team
EPI_ISL_675635, EPI_ISL_675636, EPI_ISL_675637, EPI_ISL_675638, EPI_ISL_675639, EPI_ISL_675640, EPI_ISL_675641, EPI_ISL_675642, EPI_ISL_675643	Lighthouse Lab in Alderley Park	Wellcome Sanger Institute for the COVID-19 Genomics UK (COG-UK) Consortium	Jacquelyn Wynn, Mairead Hyland, The Lighthouse Lab in Alderley Park and Alex Alderton, Roberto Amato, Sonia Goncalves, Ewan Harrison, David K. Jackson, Ian Johnston, Dominic Kwiatkowski, Cordelia Langford, John Sillitoe on behalf of the Wellcome Sanger Institute COVID-19 Surveillance Team
EPI_ISL_675644	Lighthouse Lab in Milton Keynes	Wellcome Sanger Institute for the COVID-19 Genomics UK (COG-UK) Consortium	The Lighthouse Lab in Milton Keynes and Alex Alderton, Roberto Amato, Sonia Goncalves, Ewan Harrison, David K. Jackson, Ian Johnston, Dominic Kwiatkowski, Cordelia Langford, John Sillitoe on behalf of the Wellcome Sanger Institute COVID-19 Surveillance Team
EPI_ISL_675645, EPI_ISL_675647, EPI_ISL_675648, EPI_ISL_675649, EPI_ISL_675650, EPI_ISL_675651	Lighthouse Lab in Alderley Park	Wellcome Sanger Institute for the COVID-19 Genomics UK (COG-UK) Consortium	Jacquelyn Wynn, Mairead Hyland, The Lighthouse Lab in Alderley Park and Alex Alderton, Roberto Amato, Sonia Goncalves, Ewan Harrison, David K. Jackson, Ian Johnston, Dominic Kwiatkowski, Cordelia Langford, John Sillitoe on behalf of the Wellcome Sanger Institute COVID-19 Surveillance Team
EPI_ISL_675652	Lighthouse Lab in Milton Keynes	Wellcome Sanger Institute for the COVID-19 Genomics UK (COG-UK) Consortium	The Lighthouse Lab in Milton Keynes and Alex Alderton, Roberto Amato, Sonia Goncalves, Ewan Harrison, David K. Jackson, Ian Johnston, Dominic Kwiatkowski, Cordelia Langford, John Sillitoe on behalf of the Wellcome Sanger Institute COVID-19 Surveillance Team
EPI_ISL_675653, EPI_ISL_675654, EPI_ISL_675655, EPI_ISL_675656, EPI_ISL_675657, EPI_ISL_675658, EPI_ISL_675659	Lighthouse Lab in Alderley Park	Wellcome Sanger Institute for the COVID-19 Genomics UK (COG-UK) Consortium	Jacquelyn Wynn, Mairead Hyland, The Lighthouse Lab in Alderley Park and Alex Alderton, Roberto Amato, Sonia Goncalves, Ewan Harrison, David K. Jackson, Ian Johnston, Dominic Kwiatkowski, Cordelia Langford, John Sillitoe on behalf of the Wellcome Sanger Institute COVID-19 Surveillance Team
EPI_ISL_675660, EPI_ISL_675661	Lighthouse Lab in Milton Keynes	Wellcome Sanger Institute for the COVID-19 Genomics UK (COG-UK) Consortium	The Lighthouse Lab in Milton Keynes and Alex Alderton, Roberto Amato, Sonia Goncalves, Ewan Harrison, David K. Jackson, Ian Johnston, Dominic Kwiatkowski, Cordelia Langford, John Sillitoe on behalf of the Wellcome Sanger Institute COVID-19 Surveillance Team
EPI_ISL_675662	Lighthouse Lab in Alderley Park	Wellcome Sanger Institute for the COVID-19 Genomics UK (COG-UK) Consortium	Jacquelyn Wynn, Mairead Hyland, The Lighthouse Lab in Alderley Park and Alex Alderton, Roberto Amato, Sonia Goncalves, Ewan Harrison, David K. Jackson, Ian Johnston, Dominic Kwiatkowski, Cordelia Langford, John Sillitoe on behalf of the Wellcome Sanger Institute COVID-19 Surveillance Team
EPI_ISL_675663	Lighthouse Lab in Milton Keynes	Wellcome Sanger Institute for the COVID-19 Genomics UK (COG-UK) Consortium	The Lighthouse Lab in Milton Keynes and Alex Alderton, Roberto Amato, Sonia Goncalves, Ewan Harrison, David K. Jackson, Ian Johnston, Dominic Kwiatkowski, Cordelia Langford, John Sillitoe on behalf of the Wellcome Sanger Institute COVID-19 Surveillance Team
EPI_ISL_675665, EPI_ISL_675666	Lighthouse Lab in Alderley Park	Wellcome Sanger Institute for the COVID-19 Genomics UK (COG-UK) Consortium	Jacquelyn Wynn, Mairead Hyland, The Lighthouse Lab in Alderley Park and Alex Alderton, Roberto Amato, Sonia Goncalves, Ewan Harrison, David K. Jackson, Ian Johnston, Dominic Kwiatkowski, Cordelia Langford, John Sillitoe on behalf of the Wellcome Sanger Institute COVID-19 Surveillance Team
EPI_ISL_675667	Lighthouse Lab in Milton Keynes	Wellcome Sanger Institute for the COVID-19 Genomics UK (COG-UK) Consortium	The Lighthouse Lab in Milton Keynes and Alex Alderton, Roberto Amato, Sonia Goncalves, Ewan Harrison, David K. Jackson, Ian Johnston, Dominic Kwiatkowski, Cordelia Langford, John Sillitoe on behalf of the Wellcome Sanger Institute COVID-19 Surveillance Team
EPI_ISL_675668, EPI_ISL_675669, EPI_ISL_675670, EPI_ISL_675671, EPI_ISL_675673, EPI_ISL_675674, EPI_ISL_675675	Lighthouse Lab in Alderley Park	Wellcome Sanger Institute for the COVID-19 Genomics UK (COG-UK) Consortium	Jacquelyn Wynn, Mairead Hyland, The Lighthouse Lab in Alderley Park and Alex Alderton, Roberto Amato, Sonia Goncalves, Ewan Harrison, David K. Jackson, Ian Johnston, Dominic Kwiatkowski, Cordelia Langford, John Sillitoe on behalf of the Wellcome Sanger Institute COVID-19 Surveillance Team
EPI_ISL_675676	Lighthouse Lab in Milton Keynes	Wellcome Sanger Institute for the COVID-19 Genomics UK (COG-UK) Consortium	The Lighthouse Lab in Milton Keynes and Alex Alderton, Roberto Amato, Sonia Goncalves, Ewan Harrison, David K. Jackson, Ian Johnston, Dominic Kwiatkowski, Cordelia Langford, John Sillitoe on behalf of the Wellcome Sanger Institute COVID-19 Surveillance Team
EPI_ISL_675677, EPI_ISL_675678, EPI_ISL_675680, EPI_ISL_675681	Lighthouse Lab in Alderley Park	Wellcome Sanger Institute for the COVID-19 Genomics UK (COG-UK) Consortium	Jacquelyn Wynn, Mairead Hyland, The Lighthouse Lab in Alderley Park and Alex Alderton, Roberto Amato, Sonia Goncalves, Ewan Harrison, David K. Jackson, Ian Johnston, Dominic Kwiatkowski, Cordelia Langford, John Sillitoe on behalf of the Wellcome Sanger Institute COVID-19 Surveillance Team
EPI_ISL_675682	Lighthouse Lab in Milton Keynes	Wellcome Sanger Institute for the COVID-19 Genomics UK (COG-UK) Consortium	The Lighthouse Lab in Milton Keynes and Alex Alderton, Roberto Amato, Sonia Goncalves, Ewan Harrison, David K. Jackson, Ian Johnston, Dominic Kwiatkowski, Cordelia Langford, John Sillitoe on behalf of the Wellcome Sanger Institute COVID-19 Surveillance Team
EPI_ISL_675683, EPI_ISL_675684, EPI_ISL_675685, EPI_ISL_675686, EPI_ISL_675687, EPI_ISL_675688, EPI_ISL_675689	Lighthouse Lab in Alderley Park	Wellcome Sanger Institute for the COVID-19 Genomics UK (COG-UK) Consortium	Jacquelyn Wynn, Mairead Hyland, The Lighthouse Lab in Alderley Park and Alex Alderton, Roberto Amato, Sonia Goncalves, Ewan Harrison, David K. Jackson, Ian Johnston, Dominic Kwiatkowski, Cordelia Langford, John Sillitoe on behalf of the Wellcome Sanger Institute COVID-19 Surveillance Team
EPI_ISL_675690	Lighthouse Lab in Milton Keynes	Wellcome Sanger Institute for the COVID-19 Genomics UK (COG-UK) Consortium	The Lighthouse Lab in Milton Keynes and Alex Alderton, Roberto Amato, Sonia Goncalves, Ewan Harrison, David K. Jackson, Ian Johnston, Dominic Kwiatkowski, Cordelia Langford, John Sillitoe on behalf of the Wellcome Sanger Institute COVID-19 Surveillance Team
EPI_ISL_675691, EPI_ISL_675692	Lighthouse Lab in Alderley Park	Wellcome Sanger Institute for the COVID-19 Genomics UK (COG-UK) Consortium	Jacquelyn Wynn, Mairead Hyland, The Lighthouse Lab in Alderley Park and Alex Alderton, Roberto Amato, Sonia Goncalves, Ewan Harrison, David K. Jackson, Ian Johnston, Dominic Kwiatkowski, Cordelia Langford, John Sillitoe on behalf of the Wellcome Sanger Institute COVID-19 Surveillance Team
EPI_ISL_675693, EPI_ISL_675694, EPI_ISL_675695	Lighthouse Lab in Milton Keynes	Wellcome Sanger Institute for the COVID-19 Genomics UK (COG-UK) Consortium	The Lighthouse Lab in Milton Keynes and Alex Alderton, Roberto Amato, Sonia Goncalves, Ewan Harrison, David K. Jackson, Ian Johnston, Dominic Kwiatkowski, Cordelia Langford, John Sillitoe on behalf of the Wellcome Sanger Institute COVID-19 Surveillance Team
EPI_ISL_675696, EPI_ISL_675697	Lighthouse Lab in Alderley Park	Wellcome Sanger Institute for the COVID-19 Genomics UK (COG-UK) Consortium	Jacquelyn Wynn, Mairead Hyland, The Lighthouse Lab in Alderley Park and Alex Alderton, Roberto Amato, Sonia Goncalves, Ewan Harrison, David K. Jackson, Ian Johnston, Dominic Kwiatkowski, Cordelia Langford, John Sillitoe on behalf of the Wellcome Sanger Institute COVID-19 Surveillance Team
EPI_ISL_675698	Lighthouse Lab in Milton Keynes	Wellcome Sanger Institute for the COVID-19 Genomics UK (COG-UK) Consortium	The Lighthouse Lab in Milton Keynes and Alex Alderton, Roberto Amato, Sonia Goncalves, Ewan Harrison, David K. Jackson, Ian Johnston, Dominic Kwiatkowski, Cordelia Langford, John Sillitoe on behalf of the Wellcome Sanger Institute COVID-19 Surveillance Team
EPI_ISL_675699, EPI_ISL_675700, EPI_ISL_675701, EPI_ISL_675702,	Lighthouse Lab in Alderley Park	Wellcome Sanger Institute for the COVID-19 Genomics UK (COG-UK) Consortium	Jacquelyn Wynn, Mairead Hyland, The Lighthouse Lab in Alderley Park and Alex Alderton, Roberto Amato, Sonia Goncalves, Ewan Harrison, David K. Jackson, Ian Johnston, Dominic Kwiatkowski, Cordelia Langford, John Sillitoe on behalf of the Wellcome Sanger Institute COVID-19 Surveillance Team

EPI_ISL_675703			
EPI_ISL_675704	Lighthouse Lab in Milton Keynes	Wellcome Sanger Institute for the COVID-19 Genomics UK (COG-UK) Consortium	The Lighthouse Lab in Milton Keynes and Alex Alderton, Roberto Amato, Sonia Goncalves, Ewan Harrison, David K. Jackson, Ian Johnston, Dominic Kwiatkowski, Cordelia Langford, John Sillitoe on behalf of the Wellcome Sanger Institute COVID-19 Surveillance Team
EPI_ISL_675705, EPI_ISL_675706, EPI_ISL_675707, EPI_ISL_675708	Lighthouse Lab in Alderley Park	Wellcome Sanger Institute for the COVID-19 Genomics UK (COG-UK) Consortium	Jacquelyn Wynn, Mairead Hyland, The Lighthouse Lab in Alderley Park and Alex Alderton, Roberto Amato, Sonia Goncalves, Ewan Harrison, David K. Jackson, Ian Johnston, Dominic Kwiatkowski, Cordelia Langford, John Sillitoe on behalf of the Wellcome Sanger Institute COVID-19 Surveillance Team
EPI_ISL_675709	Lighthouse Lab in Milton Keynes	Wellcome Sanger Institute for the COVID-19 Genomics UK (COG-UK) Consortium	The Lighthouse Lab in Milton Keynes and Alex Alderton, Roberto Amato, Sonia Goncalves, Ewan Harrison, David K. Jackson, Ian Johnston, Dominic Kwiatkowski, Cordelia Langford, John Sillitoe on behalf of the Wellcome Sanger Institute COVID-19 Surveillance Team
EPI_ISL_675710	Lighthouse Lab in Alderley Park	Wellcome Sanger Institute for the COVID-19 Genomics UK (COG-UK) Consortium	Jacquelyn Wynn, Mairead Hyland, The Lighthouse Lab in Alderley Park and Alex Alderton, Roberto Amato, Sonia Goncalves, Ewan Harrison, David K. Jackson, Ian Johnston, Dominic Kwiatkowski, Cordelia Langford, John Sillitoe on behalf of the Wellcome Sanger Institute COVID-19 Surveillance Team
EPI_ISL_675711, EPI_ISL_675712, EPI_ISL_675713, EPI_ISL_675714	Lighthouse Lab in Milton Keynes	Wellcome Sanger Institute for the COVID-19 Genomics UK (COG-UK) Consortium	The Lighthouse Lab in Milton Keynes and Alex Alderton, Roberto Amato, Sonia Goncalves, Ewan Harrison, David K. Jackson, Ian Johnston, Dominic Kwiatkowski, Cordelia Langford, John Sillitoe on behalf of the Wellcome Sanger Institute COVID-19 Surveillance Team
EPI_ISL_675715, EPI_ISL_675716	Lighthouse Lab in Alderley Park	Wellcome Sanger Institute for the COVID-19 Genomics UK (COG-UK) Consortium	Jacquelyn Wynn, Mairead Hyland, The Lighthouse Lab in Alderley Park and Alex Alderton, Roberto Amato, Sonia Goncalves, Ewan Harrison, David K. Jackson, Ian Johnston, Dominic Kwiatkowski, Cordelia Langford, John Sillitoe on behalf of the Wellcome Sanger Institute COVID-19 Surveillance Team
EPI_ISL_675717, EPI_ISL_675718	Lighthouse Lab in Milton Keynes	Wellcome Sanger Institute for the COVID-19 Genomics UK (COG-UK) Consortium	The Lighthouse Lab in Milton Keynes and Alex Alderton, Roberto Amato, Sonia Goncalves, Ewan Harrison, David K. Jackson, Ian Johnston, Dominic Kwiatkowski, Cordelia Langford, John Sillitoe on behalf of the Wellcome Sanger Institute COVID-19 Surveillance Team
EPI_ISL_675719, EPI_ISL_675720, EPI_ISL_675721	Lighthouse Lab in Alderley Park	Wellcome Sanger Institute for the COVID-19 Genomics UK (COG-UK) Consortium	Jacquelyn Wynn, Mairead Hyland, The Lighthouse Lab in Alderley Park and Alex Alderton, Roberto Amato, Sonia Goncalves, Ewan Harrison, David K. Jackson, Ian Johnston, Dominic Kwiatkowski, Cordelia Langford, John Sillitoe on behalf of the Wellcome Sanger Institute COVID-19 Surveillance Team
EPI_ISL_675722, EPI_ISL_675723, EPI_ISL_675724, EPI_ISL_675725, EPI_ISL_675726, EPI_ISL_675728, EPI_ISL_675729, EPI_ISL_675730, EPI_ISL_675731, EPI_ISL_675732, EPI_ISL_675733, EPI_ISL_675734			
see above	Lighthouse Lab in Milton Keynes	Wellcome Sanger Institute for the COVID-19 Genomics UK (COG-UK) Consortium	The Lighthouse Lab in Milton Keynes and Alex Alderton, Roberto Amato, Sonia Goncalves, Ewan Harrison, David K. Jackson, Ian Johnston, Dominic Kwiatkowski, Cordelia Langford, John Sillitoe on behalf of the Wellcome Sanger Institute COVID-19 Surveillance Team
EPI_ISL_675735	Lighthouse Lab in Alderley Park	Wellcome Sanger Institute for the COVID-19 Genomics UK (COG-UK) Consortium	Jacquelyn Wynn, Mairead Hyland, The Lighthouse Lab in Alderley Park and Alex Alderton, Roberto Amato, Sonia Goncalves, Ewan Harrison, David K. Jackson, Ian Johnston, Dominic Kwiatkowski, Cordelia Langford, John Sillitoe on behalf of the Wellcome Sanger Institute COVID-19 Surveillance Team
EPI_ISL_675736, EPI_ISL_675737, EPI_ISL_675738	Lighthouse Lab in Milton Keynes	Wellcome Sanger Institute for the COVID-19 Genomics UK (COG-UK) Consortium	The Lighthouse Lab in Milton Keynes and Alex Alderton, Roberto Amato, Sonia Goncalves, Ewan Harrison, David K. Jackson, Ian Johnston, Dominic Kwiatkowski, Cordelia Langford, John Sillitoe on behalf of the Wellcome Sanger Institute COVID-19 Surveillance Team
EPI_ISL_675739, EPI_ISL_675740	Lighthouse Lab in Alderley Park	Wellcome Sanger Institute for the COVID-19 Genomics UK (COG-UK) Consortium	Jacquelyn Wynn, Mairead Hyland, The Lighthouse Lab in Alderley Park and Alex Alderton, Roberto Amato, Sonia Goncalves, Ewan Harrison, David K. Jackson, Ian Johnston, Dominic Kwiatkowski, Cordelia Langford, John Sillitoe on behalf of the Wellcome Sanger Institute COVID-19 Surveillance Team
EPI_ISL_675741, EPI_ISL_675742, EPI_ISL_675743, EPI_ISL_675744, EPI_ISL_675745	Lighthouse Lab in Milton Keynes	Wellcome Sanger Institute for the COVID-19 Genomics UK (COG-UK) Consortium	The Lighthouse Lab in Milton Keynes and Alex Alderton, Roberto Amato, Sonia Goncalves, Ewan Harrison, David K. Jackson, Ian Johnston, Dominic Kwiatkowski, Cordelia Langford, John Sillitoe on behalf of the Wellcome Sanger Institute COVID-19 Surveillance Team
EPI_ISL_675746	Lighthouse Lab in Alderley Park	Wellcome Sanger Institute for the COVID-19 Genomics UK (COG-UK) Consortium	Jacquelyn Wynn, Mairead Hyland, The Lighthouse Lab in Alderley Park and Alex Alderton, Roberto Amato, Sonia Goncalves, Ewan Harrison, David K. Jackson, Ian Johnston, Dominic Kwiatkowski, Cordelia Langford, John Sillitoe on behalf of the Wellcome Sanger Institute COVID-19 Surveillance Team
EPI_ISL_675747, EPI_ISL_675748, EPI_ISL_675749	Lighthouse Lab in Milton Keynes	Wellcome Sanger Institute for the COVID-19 Genomics UK (COG-UK) Consortium	The Lighthouse Lab in Milton Keynes and Alex Alderton, Roberto Amato, Sonia Goncalves, Ewan Harrison, David K. Jackson, Ian Johnston, Dominic Kwiatkowski, Cordelia Langford, John Sillitoe on behalf of the Wellcome Sanger Institute COVID-19 Surveillance Team
EPI_ISL_675750, EPI_ISL_675751	Lighthouse Lab in Alderley Park	Wellcome Sanger Institute for the COVID-19 Genomics UK (COG-UK) Consortium	Jacquelyn Wynn, Mairead Hyland, The Lighthouse Lab in Alderley Park and Alex Alderton, Roberto Amato, Sonia Goncalves, Ewan Harrison, David K. Jackson, Ian Johnston, Dominic Kwiatkowski, Cordelia Langford, John Sillitoe on behalf of the Wellcome Sanger Institute COVID-19 Surveillance Team
EPI_ISL_675752, EPI_ISL_675753, EPI_ISL_675754, EPI_ISL_675755	Lighthouse Lab in Milton Keynes	Wellcome Sanger Institute for the COVID-19 Genomics UK (COG-UK) Consortium	The Lighthouse Lab in Milton Keynes and Alex Alderton, Roberto Amato, Sonia Goncalves, Ewan Harrison, David K. Jackson, Ian Johnston, Dominic Kwiatkowski, Cordelia Langford, John Sillitoe on behalf of the Wellcome Sanger Institute COVID-19 Surveillance Team
EPI_ISL_675756, EPI_ISL_675757	Lighthouse Lab in Alderley Park	Wellcome Sanger Institute for the COVID-19 Genomics UK (COG-UK) Consortium	Jacquelyn Wynn, Mairead Hyland, The Lighthouse Lab in Alderley Park and Alex Alderton, Roberto Amato, Sonia Goncalves, Ewan Harrison, David K. Jackson, Ian Johnston, Dominic Kwiatkowski, Cordelia Langford, John Sillitoe on behalf of the Wellcome Sanger Institute COVID-19 Surveillance Team
EPI_ISL_675758	Lighthouse Lab in Milton Keynes	Wellcome Sanger Institute for the COVID-19 Genomics UK (COG-UK) Consortium	The Lighthouse Lab in Milton Keynes and Alex Alderton, Roberto Amato, Sonia Goncalves, Ewan Harrison, David K. Jackson, Ian Johnston, Dominic Kwiatkowski, Cordelia Langford, John Sillitoe on behalf of the Wellcome Sanger Institute COVID-19 Surveillance Team
EPI_ISL_675759, EPI_ISL_675760	Lighthouse Lab in Alderley Park	Wellcome Sanger Institute for the COVID-19 Genomics UK (COG-UK) Consortium	Jacquelyn Wynn, Mairead Hyland, The Lighthouse Lab in Alderley Park and Alex Alderton, Roberto Amato, Sonia Goncalves, Ewan Harrison, David K. Jackson, Ian Johnston, Dominic Kwiatkowski, Cordelia Langford, John Sillitoe on behalf of the Wellcome Sanger Institute COVID-19 Surveillance Team
EPI_ISL_675761, EPI_ISL_675762, EPI_ISL_675763, EPI_ISL_675764, EPI_ISL_675765	Lighthouse Lab in Milton Keynes	Wellcome Sanger Institute for the COVID-19 Genomics UK (COG-UK) Consortium	The Lighthouse Lab in Milton Keynes and Alex Alderton, Roberto Amato, Sonia Goncalves, Ewan Harrison, David K. Jackson, Ian Johnston, Dominic Kwiatkowski, Cordelia Langford, John Sillitoe on behalf of the Wellcome Sanger Institute COVID-19 Surveillance Team
EPI_ISL_675766	Lighthouse Lab in Alderley Park	Wellcome Sanger Institute for the COVID-19 Genomics UK (COG-UK) Consortium	Jacquelyn Wynn, Mairead Hyland, The Lighthouse Lab in Alderley Park and Alex Alderton, Roberto Amato, Sonia Goncalves, Ewan Harrison, David K. Jackson, Ian Johnston, Dominic Kwiatkowski, Cordelia Langford, John Sillitoe on behalf of the Wellcome Sanger Institute COVID-19 Surveillance Team
EPI_ISL_675767, EPI_ISL_675768, EPI_ISL_675769	Lighthouse Lab in Milton Keynes	Wellcome Sanger Institute for the COVID-19 Genomics UK (COG-UK) Consortium	The Lighthouse Lab in Milton Keynes and Alex Alderton, Roberto Amato, Sonia Goncalves, Ewan Harrison, David K. Jackson, Ian Johnston, Dominic Kwiatkowski, Cordelia Langford, John Sillitoe on behalf of the Wellcome Sanger Institute COVID-19 Surveillance Team
EPI_ISL_675771	Lighthouse Lab in Alderley Park	Wellcome Sanger Institute for the COVID-19 Genomics UK (COG-UK) Consortium	Jacquelyn Wynn, Mairead Hyland, The Lighthouse Lab in Alderley Park and Alex Alderton, Roberto Amato, Sonia Goncalves, Ewan Harrison, David K. Jackson, Ian Johnston, Dominic Kwiatkowski, Cordelia Langford, John Sillitoe on behalf of the Wellcome Sanger Institute COVID-19 Surveillance Team
EPI_ISL_675772, EPI_ISL_675773	Lighthouse Lab in Milton Keynes	Wellcome Sanger Institute for the COVID-19 Genomics UK (COG-UK) Consortium	The Lighthouse Lab in Milton Keynes and Alex Alderton, Roberto Amato, Sonia Goncalves, Ewan Harrison, David K. Jackson, Ian Johnston, Dominic Kwiatkowski, Cordelia Langford, John Sillitoe on behalf of the Wellcome Sanger Institute COVID-19 Surveillance Team
EPI_ISL_675774, EPI_ISL_675775, EPI_ISL_675776, EPI_ISL_675777	Lighthouse Lab in Alderley Park	Wellcome Sanger Institute for the COVID-19 Genomics UK (COG-UK) Consortium	Jacquelyn Wynn, Mairead Hyland, The Lighthouse Lab in Alderley Park and Alex Alderton, Roberto Amato, Sonia Goncalves, Ewan Harrison, David K. Jackson, Ian Johnston, Dominic Kwiatkowski, Cordelia Langford, John Sillitoe on behalf of the Wellcome Sanger Institute COVID-19 Surveillance Team
EPI_ISL_675778	Lighthouse Lab in Milton Keynes	Wellcome Sanger Institute for the COVID-19 Genomics UK (COG-UK) Consortium	The Lighthouse Lab in Milton Keynes and Alex Alderton, Roberto Amato, Sonia Goncalves, Ewan Harrison, David K. Jackson, Ian Johnston, Dominic Kwiatkowski, Cordelia Langford, John Sillitoe on behalf of the Wellcome Sanger Institute COVID-19 Surveillance Team
EPI_ISL_675779	Lighthouse Lab in Alderley Park	Wellcome Sanger Institute for the COVID-19 Genomics UK (COG-UK) Consortium	Jacquelyn Wynn, Mairead Hyland, The Lighthouse Lab in Alderley Park and Alex Alderton, Roberto Amato, Sonia Goncalves, Ewan Harrison, David K. Jackson, Ian Johnston, Dominic Kwiatkowski, Cordelia Langford, John Sillitoe on behalf of the Wellcome Sanger Institute COVID-19 Surveillance Team
EPI_ISL_675780, EPI_ISL_675781, EPI_ISL_675782, EPI_ISL_675783, EPI_ISL_675784	Lighthouse Lab in Milton Keynes	Wellcome Sanger Institute for the COVID-19 Genomics UK (COG-UK) Consortium	The Lighthouse Lab in Milton Keynes and Alex Alderton, Roberto Amato, Sonia Goncalves, Ewan Harrison, David K. Jackson, Ian Johnston, Dominic Kwiatkowski, Cordelia Langford, John Sillitoe on behalf of the Wellcome Sanger Institute COVID-19 Surveillance Team
EPI_ISL_675785	Lighthouse Lab in Alderley Park	Wellcome Sanger Institute for the COVID-19 Genomics UK (COG-UK) Consortium	Jacquelyn Wynn, Mairead Hyland, The Lighthouse Lab in Alderley Park and Alex Alderton, Roberto Amato, Sonia Goncalves, Ewan Harrison, David K. Jackson, Ian Johnston, Dominic Kwiatkowski, Cordelia Langford, John Sillitoe on behalf of the Wellcome Sanger Institute COVID-19 Surveillance Team
EPI_ISL_675786, EPI_ISL_675787	Lighthouse Lab in Milton Keynes	Wellcome Sanger Institute for the COVID-19 Genomics UK (COG-UK) Consortium	The Lighthouse Lab in Milton Keynes and Alex Alderton, Roberto Amato, Sonia Goncalves, Ewan Harrison, David K. Jackson, Ian Johnston, Dominic Kwiatkowski, Cordelia Langford, John Sillitoe on behalf of the Wellcome Sanger Institute COVID-19 Surveillance Team
EPI_ISL_675788	Lighthouse Lab in Alderley Park	Wellcome Sanger Institute for the COVID-19 Genomics UK (COG-UK) Consortium	Jacquelyn Wynn, Mairead Hyland, The Lighthouse Lab in Alderley Park and Alex Alderton, Roberto Amato, Sonia Goncalves, Ewan Harrison, David K. Jackson, Ian Johnston, Dominic Kwiatkowski, Cordelia Langford, John Sillitoe on behalf of the Wellcome Sanger Institute COVID-19 Surveillance Team
EPI_ISL_675789, EPI_ISL_675790, EPI_ISL_675791, EPI_ISL_675792,	Lighthouse Lab in Milton Keynes	Wellcome Sanger Institute for the COVID-19 Genomics UK (COG-UK) Consortium	The Lighthouse Lab in Milton Keynes and Alex Alderton, Roberto Amato, Sonia Goncalves, Ewan Harrison, David K. Jackson, Ian Johnston, Dominic Kwiatkowski, Cordelia Langford, John Sillitoe on behalf of the Wellcome Sanger Institute COVID-19 Surveillance Team

EPI_ISL_675793			
EPI_ISL_675794	Lighthouse Lab in Alderley Park	Wellcome Sanger Institute for the COVID-19 Genomics UK (COG-UK) Consortium	Jacquelyn Wynn, Mairead Hyland, The Lighthouse Lab in Alderley Park and Alex Alderton, Roberto Amato, Sonia Goncalves, Ewan Harrison, David K. Jackson, Ian Johnston, Dominic Kwiatkowski, Cordelia Langford, John Sillitoe on behalf of the Wellcome Sanger Institute COVID-19 Surveillance Team
EPI_ISL_675795, EPI_ISL_675796, EPI_ISL_675797	Lighthouse Lab in Milton Keynes	Wellcome Sanger Institute for the COVID-19 Genomics UK (COG-UK) Consortium	The Lighthouse Lab in Milton Keynes and Alex Alderton, Roberto Amato, Sonia Goncalves, Ewan Harrison, David K. Jackson, Ian Johnston, Dominic Kwiatkowski, Cordelia Langford, John Sillitoe on behalf of the Wellcome Sanger Institute COVID-19 Surveillance Team
EPI_ISL_675798, EPI_ISL_675799, EPI_ISL_675800	Lighthouse Lab in Alderley Park	Wellcome Sanger Institute for the COVID-19 Genomics UK (COG-UK) Consortium	Jacquelyn Wynn, Mairead Hyland, The Lighthouse Lab in Alderley Park and Alex Alderton, Roberto Amato, Sonia Goncalves, Ewan Harrison, David K. Jackson, Ian Johnston, Dominic Kwiatkowski, Cordelia Langford, John Sillitoe on behalf of the Wellcome Sanger Institute COVID-19 Surveillance Team
EPI_ISL_675801	Lighthouse Lab in Milton Keynes	Wellcome Sanger Institute for the COVID-19 Genomics UK (COG-UK) Consortium	The Lighthouse Lab in Milton Keynes and Alex Alderton, Roberto Amato, Sonia Goncalves, Ewan Harrison, David K. Jackson, Ian Johnston, Dominic Kwiatkowski, Cordelia Langford, John Sillitoe on behalf of the Wellcome Sanger Institute COVID-19 Surveillance Team
EPI_ISL_675802, EPI_ISL_675803	Lighthouse Lab in Alderley Park	Wellcome Sanger Institute for the COVID-19 Genomics UK (COG-UK) Consortium	Jacquelyn Wynn, Mairead Hyland, The Lighthouse Lab in Alderley Park and Alex Alderton, Roberto Amato, Sonia Goncalves, Ewan Harrison, David K. Jackson, Ian Johnston, Dominic Kwiatkowski, Cordelia Langford, John Sillitoe on behalf of the Wellcome Sanger Institute COVID-19 Surveillance Team
EPI_ISL_675804, EPI_ISL_675805	Lighthouse Lab in Milton Keynes	Wellcome Sanger Institute for the COVID-19 Genomics UK (COG-UK) Consortium	The Lighthouse Lab in Milton Keynes and Alex Alderton, Roberto Amato, Sonia Goncalves, Ewan Harrison, David K. Jackson, Ian Johnston, Dominic Kwiatkowski, Cordelia Langford, John Sillitoe on behalf of the Wellcome Sanger Institute COVID-19 Surveillance Team
EPI_ISL_675806	Lighthouse Lab in Alderley Park	Wellcome Sanger Institute for the COVID-19 Genomics UK (COG-UK) Consortium	Jacquelyn Wynn, Mairead Hyland, The Lighthouse Lab in Alderley Park and Alex Alderton, Roberto Amato, Sonia Goncalves, Ewan Harrison, David K. Jackson, Ian Johnston, Dominic Kwiatkowski, Cordelia Langford, John Sillitoe on behalf of the Wellcome Sanger Institute COVID-19 Surveillance Team
EPI_ISL_675807, EPI_ISL_675808, EPI_ISL_675809	Lighthouse Lab in Milton Keynes	Wellcome Sanger Institute for the COVID-19 Genomics UK (COG-UK) Consortium	The Lighthouse Lab in Milton Keynes and Alex Alderton, Roberto Amato, Sonia Goncalves, Ewan Harrison, David K. Jackson, Ian Johnston, Dominic Kwiatkowski, Cordelia Langford, John Sillitoe on behalf of the Wellcome Sanger Institute COVID-19 Surveillance Team
EPI_ISL_675810	Lighthouse Lab in Alderley Park	Wellcome Sanger Institute for the COVID-19 Genomics UK (COG-UK) Consortium	Jacquelyn Wynn, Mairead Hyland, The Lighthouse Lab in Alderley Park and Alex Alderton, Roberto Amato, Sonia Goncalves, Ewan Harrison, David K. Jackson, Ian Johnston, Dominic Kwiatkowski, Cordelia Langford, John Sillitoe on behalf of the Wellcome Sanger Institute COVID-19 Surveillance Team
EPI_ISL_675811	Lighthouse Lab in Milton Keynes	Wellcome Sanger Institute for the COVID-19 Genomics UK (COG-UK) Consortium	The Lighthouse Lab in Milton Keynes and Alex Alderton, Roberto Amato, Sonia Goncalves, Ewan Harrison, David K. Jackson, Ian Johnston, Dominic Kwiatkowski, Cordelia Langford, John Sillitoe on behalf of the Wellcome Sanger Institute COVID-19 Surveillance Team
EPI_ISL_675812, EPI_ISL_675813	Lighthouse Lab in Alderley Park	Wellcome Sanger Institute for the COVID-19 Genomics UK (COG-UK) Consortium	Jacquelyn Wynn, Mairead Hyland, The Lighthouse Lab in Alderley Park and Alex Alderton, Roberto Amato, Sonia Goncalves, Ewan Harrison, David K. Jackson, Ian Johnston, Dominic Kwiatkowski, Cordelia Langford, John Sillitoe on behalf of the Wellcome Sanger Institute COVID-19 Surveillance Team
EPI_ISL_675814, EPI_ISL_675815, EPI_ISL_675816	Lighthouse Lab in Milton Keynes	Wellcome Sanger Institute for the COVID-19 Genomics UK (COG-UK) Consortium	The Lighthouse Lab in Milton Keynes and Alex Alderton, Roberto Amato, Sonia Goncalves, Ewan Harrison, David K. Jackson, Ian Johnston, Dominic Kwiatkowski, Cordelia Langford, John Sillitoe on behalf of the Wellcome Sanger Institute COVID-19 Surveillance Team
EPI_ISL_675817, EPI_ISL_675818	Lighthouse Lab in Alderley Park	Wellcome Sanger Institute for the COVID-19 Genomics UK (COG-UK) Consortium	Jacquelyn Wynn, Mairead Hyland, The Lighthouse Lab in Alderley Park and Alex Alderton, Roberto Amato, Sonia Goncalves, Ewan Harrison, David K. Jackson, Ian Johnston, Dominic Kwiatkowski, Cordelia Langford, John Sillitoe on behalf of the Wellcome Sanger Institute COVID-19 Surveillance Team
EPI_ISL_675819	Lighthouse Lab in Milton Keynes	Wellcome Sanger Institute for the COVID-19 Genomics UK (COG-UK) Consortium	The Lighthouse Lab in Milton Keynes and Alex Alderton, Roberto Amato, Sonia Goncalves, Ewan Harrison, David K. Jackson, Ian Johnston, Dominic Kwiatkowski, Cordelia Langford, John Sillitoe on behalf of the Wellcome Sanger Institute COVID-19 Surveillance Team
EPI_ISL_675820	Lighthouse Lab in Alderley Park	Wellcome Sanger Institute for the COVID-19 Genomics UK (COG-UK) Consortium	Jacquelyn Wynn, Mairead Hyland, The Lighthouse Lab in Alderley Park and Alex Alderton, Roberto Amato, Sonia Goncalves, Ewan Harrison, David K. Jackson, Ian Johnston, Dominic Kwiatkowski, Cordelia Langford, John Sillitoe on behalf of the Wellcome Sanger Institute COVID-19 Surveillance Team
EPI_ISL_675821	Lighthouse Lab in Milton Keynes	Wellcome Sanger Institute for the COVID-19 Genomics UK (COG-UK) Consortium	The Lighthouse Lab in Milton Keynes and Alex Alderton, Roberto Amato, Sonia Goncalves, Ewan Harrison, David K. Jackson, Ian Johnston, Dominic Kwiatkowski, Cordelia Langford, John Sillitoe on behalf of the Wellcome Sanger Institute COVID-19 Surveillance Team
EPI_ISL_675822, EPI_ISL_675823	Lighthouse Lab in Alderley Park	Wellcome Sanger Institute for the COVID-19 Genomics UK (COG-UK) Consortium	Jacquelyn Wynn, Mairead Hyland, The Lighthouse Lab in Alderley Park and Alex Alderton, Roberto Amato, Sonia Goncalves, Ewan Harrison, David K. Jackson, Ian Johnston, Dominic Kwiatkowski, Cordelia Langford, John Sillitoe on behalf of the Wellcome Sanger Institute COVID-19 Surveillance Team
EPI_ISL_675824	Lighthouse Lab in Milton Keynes	Wellcome Sanger Institute for the COVID-19 Genomics UK (COG-UK) Consortium	The Lighthouse Lab in Milton Keynes and Alex Alderton, Roberto Amato, Sonia Goncalves, Ewan Harrison, David K. Jackson, Ian Johnston, Dominic Kwiatkowski, Cordelia Langford, John Sillitoe on behalf of the Wellcome Sanger Institute COVID-19 Surveillance Team
EPI_ISL_675825, EPI_ISL_675826	Lighthouse Lab in Alderley Park	Wellcome Sanger Institute for the COVID-19 Genomics UK (COG-UK) Consortium	Jacquelyn Wynn, Mairead Hyland, The Lighthouse Lab in Alderley Park and Alex Alderton, Roberto Amato, Sonia Goncalves, Ewan Harrison, David K. Jackson, Ian Johnston, Dominic Kwiatkowski, Cordelia Langford, John Sillitoe on behalf of the Wellcome Sanger Institute COVID-19 Surveillance Team
EPI_ISL_675827, EPI_ISL_675828	Lighthouse Lab in Milton Keynes	Wellcome Sanger Institute for the COVID-19 Genomics UK (COG-UK) Consortium	The Lighthouse Lab in Milton Keynes and Alex Alderton, Roberto Amato, Sonia Goncalves, Ewan Harrison, David K. Jackson, Ian Johnston, Dominic Kwiatkowski, Cordelia Langford, John Sillitoe on behalf of the Wellcome Sanger Institute COVID-19 Surveillance Team
EPI_ISL_675829	Lighthouse Lab in Alderley Park	Wellcome Sanger Institute for the COVID-19 Genomics UK (COG-UK) Consortium	Jacquelyn Wynn, Mairead Hyland, The Lighthouse Lab in Alderley Park and Alex Alderton, Roberto Amato, Sonia Goncalves, Ewan Harrison, David K. Jackson, Ian Johnston, Dominic Kwiatkowski, Cordelia Langford, John Sillitoe on behalf of the Wellcome Sanger Institute COVID-19 Surveillance Team
EPI_ISL_675830, EPI_ISL_675832	Lighthouse Lab in Milton Keynes	Wellcome Sanger Institute for the COVID-19 Genomics UK (COG-UK) Consortium	The Lighthouse Lab in Milton Keynes and Alex Alderton, Roberto Amato, Sonia Goncalves, Ewan Harrison, David K. Jackson, Ian Johnston, Dominic Kwiatkowski, Cordelia Langford, John Sillitoe on behalf of the Wellcome Sanger Institute COVID-19 Surveillance Team
EPI_ISL_675833	Lighthouse Lab in Alderley Park	Wellcome Sanger Institute for the COVID-19 Genomics UK (COG-UK) Consortium	Jacquelyn Wynn, Mairead Hyland, The Lighthouse Lab in Alderley Park and Alex Alderton, Roberto Amato, Sonia Goncalves, Ewan Harrison, David K. Jackson, Ian Johnston, Dominic Kwiatkowski, Cordelia Langford, John Sillitoe on behalf of the Wellcome Sanger Institute COVID-19 Surveillance Team
EPI_ISL_675834, EPI_ISL_675835, EPI_ISL_675836, EPI_ISL_675837, EPI_ISL_675838	Lighthouse Lab in Milton Keynes	Wellcome Sanger Institute for the COVID-19 Genomics UK (COG-UK) Consortium	The Lighthouse Lab in Milton Keynes and Alex Alderton, Roberto Amato, Sonia Goncalves, Ewan Harrison, David K. Jackson, Ian Johnston, Dominic Kwiatkowski, Cordelia Langford, John Sillitoe on behalf of the Wellcome Sanger Institute COVID-19 Surveillance Team
EPI_ISL_675839	Lighthouse Lab in Alderley Park	Wellcome Sanger Institute for the COVID-19 Genomics UK (COG-UK) Consortium	Jacquelyn Wynn, Mairead Hyland, The Lighthouse Lab in Alderley Park and Alex Alderton, Roberto Amato, Sonia Goncalves, Ewan Harrison, David K. Jackson, Ian Johnston, Dominic Kwiatkowski, Cordelia Langford, John Sillitoe on behalf of the Wellcome Sanger Institute COVID-19 Surveillance Team
EPI_ISL_675840, EPI_ISL_675841, EPI_ISL_675843, EPI_ISL_675844, EPI_ISL_675845, EPI_ISL_675846	Lighthouse Lab in Milton Keynes	Wellcome Sanger Institute for the COVID-19 Genomics UK (COG-UK) Consortium	The Lighthouse Lab in Milton Keynes and Alex Alderton, Roberto Amato, Sonia Goncalves, Ewan Harrison, David K. Jackson, Ian Johnston, Dominic Kwiatkowski, Cordelia Langford, John Sillitoe on behalf of the Wellcome Sanger Institute COVID-19 Surveillance Team
EPI_ISL_675847	Lighthouse Lab in Alderley Park	Wellcome Sanger Institute for the COVID-19 Genomics UK (COG-UK) Consortium	Jacquelyn Wynn, Mairead Hyland, The Lighthouse Lab in Alderley Park and Alex Alderton, Roberto Amato, Sonia Goncalves, Ewan Harrison, David K. Jackson, Ian Johnston, Dominic Kwiatkowski, Cordelia Langford, John Sillitoe on behalf of the Wellcome Sanger Institute COVID-19 Surveillance Team
EPI_ISL_675849, EPI_ISL_675850, EPI_ISL_675851	Lighthouse Lab in Milton Keynes	Wellcome Sanger Institute for the COVID-19 Genomics UK (COG-UK) Consortium	The Lighthouse Lab in Milton Keynes and Alex Alderton, Roberto Amato, Sonia Goncalves, Ewan Harrison, David K. Jackson, Ian Johnston, Dominic Kwiatkowski, Cordelia Langford, John Sillitoe on behalf of the Wellcome Sanger Institute COVID-19 Surveillance Team
EPI_ISL_675852	Lighthouse Lab in Alderley Park	Wellcome Sanger Institute for the COVID-19 Genomics UK (COG-UK) Consortium	Jacquelyn Wynn, Mairead Hyland, The Lighthouse Lab in Alderley Park and Alex Alderton, Roberto Amato, Sonia Goncalves, Ewan Harrison, David K. Jackson, Ian Johnston, Dominic Kwiatkowski, Cordelia Langford, John Sillitoe on behalf of the Wellcome Sanger Institute COVID-19 Surveillance Team
EPI_ISL_675853, EPI_ISL_675854	Lighthouse Lab in Milton Keynes	Wellcome Sanger Institute for the COVID-19 Genomics UK (COG-UK) Consortium	The Lighthouse Lab in Milton Keynes and Alex Alderton, Roberto Amato, Sonia Goncalves, Ewan Harrison, David K. Jackson, Ian Johnston, Dominic Kwiatkowski, Cordelia Langford, John Sillitoe on behalf of the Wellcome Sanger Institute COVID-19 Surveillance Team
EPI_ISL_675855	Lighthouse Lab in Alderley Park	Wellcome Sanger Institute for the COVID-19 Genomics UK (COG-UK) Consortium	Jacquelyn Wynn, Mairead Hyland, The Lighthouse Lab in Alderley Park and Alex Alderton, Roberto Amato, Sonia Goncalves, Ewan Harrison, David K. Jackson, Ian Johnston, Dominic Kwiatkowski, Cordelia Langford, John Sillitoe on behalf of the Wellcome Sanger Institute COVID-19 Surveillance Team
EPI_ISL_675856, EPI_ISL_675857, EPI_ISL_675858, EPI_ISL_675859, EPI_ISL_675860, EPI_ISL_675861, EPI_ISL_675862, EPI_ISL_675863, EPI_ISL_675864, EPI_ISL_675865, EPI_ISL_675866, EPI_ISL_675867			
see above	Lighthouse Lab in Milton Keynes	Wellcome Sanger Institute for the COVID-19 Genomics UK (COG-UK) Consortium	The Lighthouse Lab in Milton Keynes and Alex Alderton, Roberto Amato, Sonia Goncalves, Ewan Harrison, David K. Jackson, Ian Johnston, Dominic Kwiatkowski, Cordelia Langford, John Sillitoe on behalf of the Wellcome Sanger Institute COVID-19 Surveillance Team
EPI_ISL_675868	Lighthouse Lab in Alderley Park	Wellcome Sanger Institute for the COVID-19 Genomics UK (COG-UK) Consortium	Jacquelyn Wynn, Mairead Hyland, The Lighthouse Lab in Alderley Park and Alex Alderton, Roberto Amato, Sonia Goncalves, Ewan Harrison, David K. Jackson, Ian Johnston, Dominic Kwiatkowski, Cordelia Langford, John Sillitoe on behalf of the Wellcome Sanger Institute COVID-19 Surveillance Team

EPI_ISL_675869, EPI_ISL_675870, EPI_ISL_675871, EPI_ISL_675872, EPI_ISL_675873, EPI_ISL_675874, EPI_ISL_675875, EPI_ISL_675876, EPI_ISL_675877	Lighthouse Lab in Milton Keynes	Wellcome Sanger Institute for the COVID-19 Genomics UK (COG-UK) Consortium	The Lighthouse Lab in Milton Keynes and Alex Alderton, Roberto Amato, Sonia Goncalves, Ewan Harrison, David K. Jackson, Ian Johnston, Dominic Kwiatkowski, Cordelia Langford, John Sillitoe on behalf of the Wellcome Sanger Institute COVID-19 Surveillance Team
EPI_ISL_675878	Lighthouse Lab in Alderley Park	Wellcome Sanger Institute for the COVID-19 Genomics UK (COG-UK) Consortium	Jacquelyn Wynn, Mairead Hyland, The Lighthouse Lab in Alderley Park and Alex Alderton, Roberto Amato, Sonia Goncalves, Ewan Harrison, David K. Jackson, Ian Johnston, Dominic Kwiatkowski, Cordelia Langford, John Sillitoe on behalf of the Wellcome Sanger Institute COVID-19 Surveillance Team
EPI_ISL_675879, EPI_ISL_675881	Lighthouse Lab in Milton Keynes	Wellcome Sanger Institute for the COVID-19 Genomics UK (COG-UK) Consortium	The Lighthouse Lab in Milton Keynes and Alex Alderton, Roberto Amato, Sonia Goncalves, Ewan Harrison, David K. Jackson, Ian Johnston, Dominic Kwiatkowski, Cordelia Langford, John Sillitoe on behalf of the Wellcome Sanger Institute COVID-19 Surveillance Team
EPI_ISL_675882	Lighthouse Lab in Alderley Park	Wellcome Sanger Institute for the COVID-19 Genomics UK (COG-UK) Consortium	Jacquelyn Wynn, Mairead Hyland, The Lighthouse Lab in Alderley Park and Alex Alderton, Roberto Amato, Sonia Goncalves, Ewan Harrison, David K. Jackson, Ian Johnston, Dominic Kwiatkowski, Cordelia Langford, John Sillitoe on behalf of the Wellcome Sanger Institute COVID-19 Surveillance Team
EPI_ISL_675884, EPI_ISL_675885, EPI_ISL_675886, EPI_ISL_675887, EPI_ISL_675888, EPI_ISL_675889	Lighthouse Lab in Milton Keynes	Wellcome Sanger Institute for the COVID-19 Genomics UK (COG-UK) Consortium	The Lighthouse Lab in Milton Keynes and Alex Alderton, Roberto Amato, Sonia Goncalves, Ewan Harrison, David K. Jackson, Ian Johnston, Dominic Kwiatkowski, Cordelia Langford, John Sillitoe on behalf of the Wellcome Sanger Institute COVID-19 Surveillance Team
EPI_ISL_675890	Lighthouse Lab in Alderley Park	Wellcome Sanger Institute for the COVID-19 Genomics UK (COG-UK) Consortium	Jacquelyn Wynn, Mairead Hyland, The Lighthouse Lab in Alderley Park and Alex Alderton, Roberto Amato, Sonia Goncalves, Ewan Harrison, David K. Jackson, Ian Johnston, Dominic Kwiatkowski, Cordelia Langford, John Sillitoe on behalf of the Wellcome Sanger Institute COVID-19 Surveillance Team
EPI_ISL_675891	Lighthouse Lab in Milton Keynes	Wellcome Sanger Institute for the COVID-19 Genomics UK (COG-UK) Consortium	The Lighthouse Lab in Milton Keynes and Alex Alderton, Roberto Amato, Sonia Goncalves, Ewan Harrison, David K. Jackson, Ian Johnston, Dominic Kwiatkowski, Cordelia Langford, John Sillitoe on behalf of the Wellcome Sanger Institute COVID-19 Surveillance Team
EPI_ISL_675892	Lighthouse Lab in Alderley Park	Wellcome Sanger Institute for the COVID-19 Genomics UK (COG-UK) Consortium	Jacquelyn Wynn, Mairead Hyland, The Lighthouse Lab in Alderley Park and Alex Alderton, Roberto Amato, Sonia Goncalves, Ewan Harrison, David K. Jackson, Ian Johnston, Dominic Kwiatkowski, Cordelia Langford, John Sillitoe on behalf of the Wellcome Sanger Institute COVID-19 Surveillance Team
EPI_ISL_675893	Lighthouse Lab in Milton Keynes	Wellcome Sanger Institute for the COVID-19 Genomics UK (COG-UK) Consortium	The Lighthouse Lab in Milton Keynes and Alex Alderton, Roberto Amato, Sonia Goncalves, Ewan Harrison, David K. Jackson, Ian Johnston, Dominic Kwiatkowski, Cordelia Langford, John Sillitoe on behalf of the Wellcome Sanger Institute COVID-19 Surveillance Team
EPI_ISL_675894	Lighthouse Lab in Alderley Park	Wellcome Sanger Institute for the COVID-19 Genomics UK (COG-UK) Consortium	Jacquelyn Wynn, Mairead Hyland, The Lighthouse Lab in Alderley Park and Alex Alderton, Roberto Amato, Sonia Goncalves, Ewan Harrison, David K. Jackson, Ian Johnston, Dominic Kwiatkowski, Cordelia Langford, John Sillitoe on behalf of the Wellcome Sanger Institute COVID-19 Surveillance Team
EPI_ISL_675895, EPI_ISL_675896	Lighthouse Lab in Milton Keynes	Wellcome Sanger Institute for the COVID-19 Genomics UK (COG-UK) Consortium	The Lighthouse Lab in Milton Keynes and Alex Alderton, Roberto Amato, Sonia Goncalves, Ewan Harrison, David K. Jackson, Ian Johnston, Dominic Kwiatkowski, Cordelia Langford, John Sillitoe on behalf of the Wellcome Sanger Institute COVID-19 Surveillance Team
EPI_ISL_675897	Lighthouse Lab in Alderley Park	Wellcome Sanger Institute for the COVID-19 Genomics UK (COG-UK) Consortium	Jacquelyn Wynn, Mairead Hyland, The Lighthouse Lab in Alderley Park and Alex Alderton, Roberto Amato, Sonia Goncalves, Ewan Harrison, David K. Jackson, Ian Johnston, Dominic Kwiatkowski, Cordelia Langford, John Sillitoe on behalf of the Wellcome Sanger Institute COVID-19 Surveillance Team
EPI_ISL_675898, EPI_ISL_675899, EPI_ISL_675900	Lighthouse Lab in Milton Keynes	Wellcome Sanger Institute for the COVID-19 Genomics UK (COG-UK) Consortium	The Lighthouse Lab in Milton Keynes and Alex Alderton, Roberto Amato, Sonia Goncalves, Ewan Harrison, David K. Jackson, Ian Johnston, Dominic Kwiatkowski, Cordelia Langford, John Sillitoe on behalf of the Wellcome Sanger Institute COVID-19 Surveillance Team
EPI_ISL_675901, EPI_ISL_675902, EPI_ISL_675903	Lighthouse Lab in Alderley Park	Wellcome Sanger Institute for the COVID-19 Genomics UK (COG-UK) Consortium	Jacquelyn Wynn, Mairead Hyland, The Lighthouse Lab in Alderley Park and Alex Alderton, Roberto Amato, Sonia Goncalves, Ewan Harrison, David K. Jackson, Ian Johnston, Dominic Kwiatkowski, Cordelia Langford, John Sillitoe on behalf of the Wellcome Sanger Institute COVID-19 Surveillance Team
EPI_ISL_675904, EPI_ISL_675905, EPI_ISL_675906, EPI_ISL_675907	Lighthouse Lab in Milton Keynes	Wellcome Sanger Institute for the COVID-19 Genomics UK (COG-UK) Consortium	The Lighthouse Lab in Milton Keynes and Alex Alderton, Roberto Amato, Sonia Goncalves, Ewan Harrison, David K. Jackson, Ian Johnston, Dominic Kwiatkowski, Cordelia Langford, John Sillitoe on behalf of the Wellcome Sanger Institute COVID-19 Surveillance Team
EPI_ISL_675908	Lighthouse Lab in Alderley Park	Wellcome Sanger Institute for the COVID-19 Genomics UK (COG-UK) Consortium	Jacquelyn Wynn, Mairead Hyland, The Lighthouse Lab in Alderley Park and Alex Alderton, Roberto Amato, Sonia Goncalves, Ewan Harrison, David K. Jackson, Ian Johnston, Dominic Kwiatkowski, Cordelia Langford, John Sillitoe on behalf of the Wellcome Sanger Institute COVID-19 Surveillance Team
EPI_ISL_675909, EPI_ISL_675910, EPI_ISL_675911, EPI_ISL_675912	Lighthouse Lab in Milton Keynes	Wellcome Sanger Institute for the COVID-19 Genomics UK (COG-UK) Consortium	The Lighthouse Lab in Milton Keynes and Alex Alderton, Roberto Amato, Sonia Goncalves, Ewan Harrison, David K. Jackson, Ian Johnston, Dominic Kwiatkowski, Cordelia Langford, John Sillitoe on behalf of the Wellcome Sanger Institute COVID-19 Surveillance Team
EPI_ISL_675913	Lighthouse Lab in Alderley Park	Wellcome Sanger Institute for the COVID-19 Genomics UK (COG-UK) Consortium	Jacquelyn Wynn, Mairead Hyland, The Lighthouse Lab in Alderley Park and Alex Alderton, Roberto Amato, Sonia Goncalves, Ewan Harrison, David K. Jackson, Ian Johnston, Dominic Kwiatkowski, Cordelia Langford, John Sillitoe on behalf of the Wellcome Sanger Institute COVID-19 Surveillance Team
EPI_ISL_675914, EPI_ISL_675915	Lighthouse Lab in Milton Keynes	Wellcome Sanger Institute for the COVID-19 Genomics UK (COG-UK) Consortium	The Lighthouse Lab in Milton Keynes and Alex Alderton, Roberto Amato, Sonia Goncalves, Ewan Harrison, David K. Jackson, Ian Johnston, Dominic Kwiatkowski, Cordelia Langford, John Sillitoe on behalf of the Wellcome Sanger Institute COVID-19 Surveillance Team
EPI_ISL_675916	Lighthouse Lab in Alderley Park	Wellcome Sanger Institute for the COVID-19 Genomics UK (COG-UK) Consortium	Jacquelyn Wynn, Mairead Hyland, The Lighthouse Lab in Alderley Park and Alex Alderton, Roberto Amato, Sonia Goncalves, Ewan Harrison, David K. Jackson, Ian Johnston, Dominic Kwiatkowski, Cordelia Langford, John Sillitoe on behalf of the Wellcome Sanger Institute COVID-19 Surveillance Team
EPI_ISL_675917, EPI_ISL_675918, EPI_ISL_675919	Lighthouse Lab in Milton Keynes	Wellcome Sanger Institute for the COVID-19 Genomics UK (COG-UK) Consortium	The Lighthouse Lab in Milton Keynes and Alex Alderton, Roberto Amato, Sonia Goncalves, Ewan Harrison, David K. Jackson, Ian Johnston, Dominic Kwiatkowski, Cordelia Langford, John Sillitoe on behalf of the Wellcome Sanger Institute COVID-19 Surveillance Team
EPI_ISL_675920	Lighthouse Lab in Alderley Park	Wellcome Sanger Institute for the COVID-19 Genomics UK (COG-UK) Consortium	Jacquelyn Wynn, Mairead Hyland, The Lighthouse Lab in Alderley Park and Alex Alderton, Roberto Amato, Sonia Goncalves, Ewan Harrison, David K. Jackson, Ian Johnston, Dominic Kwiatkowski, Cordelia Langford, John Sillitoe on behalf of the Wellcome Sanger Institute COVID-19 Surveillance Team
EPI_ISL_675921, EPI_ISL_675922	Lighthouse Lab in Milton Keynes	Wellcome Sanger Institute for the COVID-19 Genomics UK (COG-UK) Consortium	The Lighthouse Lab in Milton Keynes and Alex Alderton, Roberto Amato, Sonia Goncalves, Ewan Harrison, David K. Jackson, Ian Johnston, Dominic Kwiatkowski, Cordelia Langford, John Sillitoe on behalf of the Wellcome Sanger Institute COVID-19 Surveillance Team
EPI_ISL_675923, EPI_ISL_675924, EPI_ISL_675925	Lighthouse Lab in Alderley Park	Wellcome Sanger Institute for the COVID-19 Genomics UK (COG-UK) Consortium	Jacquelyn Wynn, Mairead Hyland, The Lighthouse Lab in Alderley Park and Alex Alderton, Roberto Amato, Sonia Goncalves, Ewan Harrison, David K. Jackson, Ian Johnston, Dominic Kwiatkowski, Cordelia Langford, John Sillitoe on behalf of the Wellcome Sanger Institute COVID-19 Surveillance Team
EPI_ISL_675926, EPI_ISL_675927, EPI_ISL_675928	Lighthouse Lab in Milton Keynes	Wellcome Sanger Institute for the COVID-19 Genomics UK (COG-UK) Consortium	The Lighthouse Lab in Milton Keynes and Alex Alderton, Roberto Amato, Sonia Goncalves, Ewan Harrison, David K. Jackson, Ian Johnston, Dominic Kwiatkowski, Cordelia Langford, John Sillitoe on behalf of the Wellcome Sanger Institute COVID-19 Surveillance Team
EPI_ISL_675929, EPI_ISL_675930	Lighthouse Lab in Alderley Park	Wellcome Sanger Institute for the COVID-19 Genomics UK (COG-UK) Consortium	Jacquelyn Wynn, Mairead Hyland, The Lighthouse Lab in Alderley Park and Alex Alderton, Roberto Amato, Sonia Goncalves, Ewan Harrison, David K. Jackson, Ian Johnston, Dominic Kwiatkowski, Cordelia Langford, John Sillitoe on behalf of the Wellcome Sanger Institute COVID-19 Surveillance Team
EPI_ISL_675931, EPI_ISL_675932, EPI_ISL_675933	Lighthouse Lab in Milton Keynes	Wellcome Sanger Institute for the COVID-19 Genomics UK (COG-UK) Consortium	The Lighthouse Lab in Milton Keynes and Alex Alderton, Roberto Amato, Sonia Goncalves, Ewan Harrison, David K. Jackson, Ian Johnston, Dominic Kwiatkowski, Cordelia Langford, John Sillitoe on behalf of the Wellcome Sanger Institute COVID-19 Surveillance Team
EPI_ISL_675934	Lighthouse Lab in Alderley Park	Wellcome Sanger Institute for the COVID-19 Genomics UK (COG-UK) Consortium	Jacquelyn Wynn, Mairead Hyland, The Lighthouse Lab in Alderley Park and Alex Alderton, Roberto Amato, Sonia Goncalves, Ewan Harrison, David K. Jackson, Ian Johnston, Dominic Kwiatkowski, Cordelia Langford, John Sillitoe on behalf of the Wellcome Sanger Institute COVID-19 Surveillance Team
EPI_ISL_675935, EPI_ISL_675936	Lighthouse Lab in Milton Keynes	Wellcome Sanger Institute for the COVID-19 Genomics UK (COG-UK) Consortium	The Lighthouse Lab in Milton Keynes and Alex Alderton, Roberto Amato, Sonia Goncalves, Ewan Harrison, David K. Jackson, Ian Johnston, Dominic Kwiatkowski, Cordelia Langford, John Sillitoe on behalf of the Wellcome Sanger Institute COVID-19 Surveillance Team
EPI_ISL_675937	Lighthouse Lab in Alderley Park	Wellcome Sanger Institute for the COVID-19 Genomics UK (COG-UK) Consortium	Jacquelyn Wynn, Mairead Hyland, The Lighthouse Lab in Alderley Park and Alex Alderton, Roberto Amato, Sonia Goncalves, Ewan Harrison, David K. Jackson, Ian Johnston, Dominic Kwiatkowski, Cordelia Langford, John Sillitoe on behalf of the Wellcome Sanger Institute COVID-19 Surveillance Team
EPI_ISL_675938	Lighthouse Lab in Milton Keynes	Wellcome Sanger Institute for the COVID-19 Genomics UK (COG-UK) Consortium	The Lighthouse Lab in Milton Keynes and Alex Alderton, Roberto Amato, Sonia Goncalves, Ewan Harrison, David K. Jackson, Ian Johnston, Dominic Kwiatkowski, Cordelia Langford, John Sillitoe on behalf of the Wellcome Sanger Institute COVID-19 Surveillance Team
EPI_ISL_675939	Lighthouse Lab in Alderley Park	Wellcome Sanger Institute for the COVID-19 Genomics UK (COG-UK) Consortium	Jacquelyn Wynn, Mairead Hyland, The Lighthouse Lab in Alderley Park and Alex Alderton, Roberto Amato, Sonia Goncalves, Ewan Harrison, David K. Jackson, Ian Johnston, Dominic Kwiatkowski, Cordelia Langford, John Sillitoe on behalf of the Wellcome Sanger Institute COVID-19 Surveillance Team
EPI_ISL_675940, EPI_ISL_675941	Lighthouse Lab in Milton Keynes	Wellcome Sanger Institute for the COVID-19 Genomics UK (COG-UK) Consortium	The Lighthouse Lab in Milton Keynes and Alex Alderton, Roberto Amato, Sonia Goncalves, Ewan Harrison, David K. Jackson, Ian Johnston, Dominic Kwiatkowski, Cordelia Langford, John Sillitoe on behalf of the Wellcome Sanger Institute COVID-19 Surveillance Team
EPI_ISL_675942	Lighthouse Lab in Alderley Park	Wellcome Sanger Institute for the COVID-19 Genomics UK	Jacquelyn Wynn, Mairead Hyland, The Lighthouse Lab in Alderley Park and Alex Alderton, Roberto Amato, Sonia Goncalves, Ewan Harrison, David K.

		(COG-UK) Consortium	Jackson, Ian Johnston, Dominic Kwiatkowski, Cordelia Langford, John Sillitoe on behalf of the Wellcome Sanger Institute COVID-19 Surveillance Team
EPI_ISL_675943	Lighthouse Lab in Milton Keynes	Wellcome Sanger Institute for the COVID-19 Genomics UK (COG-UK) Consortium	The Lighthouse Lab in Milton Keynes and Alex Alderton, Roberto Amato, Sonia Goncalves, Ewan Harrison, David K. Jackson, Ian Johnston, Dominic Kwiatkowski, Cordelia Langford, John Sillitoe on behalf of the Wellcome Sanger Institute COVID-19 Surveillance Team
EPI_ISL_675944	Lighthouse Lab in Alderley Park	Wellcome Sanger Institute for the COVID-19 Genomics UK (COG-UK) Consortium	Jacquelyn Wynn, Mairead Hyland, The Lighthouse Lab in Alderley Park and Alex Alderton, Roberto Amato, Sonia Goncalves, Ewan Harrison, David K. Jackson, Ian Johnston, Dominic Kwiatkowski, Cordelia Langford, John Sillitoe on behalf of the Wellcome Sanger Institute COVID-19 Surveillance Team
EPI_ISL_675945, EPI_ISL_675946, EPI_ISL_675947, EPI_ISL_675948, EPI_ISL_675949, EPI_ISL_675950, EPI_ISL_675951	Lighthouse Lab in Milton Keynes	Wellcome Sanger Institute for the COVID-19 Genomics UK (COG-UK) Consortium	The Lighthouse Lab in Milton Keynes and Alex Alderton, Roberto Amato, Sonia Goncalves, Ewan Harrison, David K. Jackson, Ian Johnston, Dominic Kwiatkowski, Cordelia Langford, John Sillitoe on behalf of the Wellcome Sanger Institute COVID-19 Surveillance Team
EPI_ISL_675952	Lighthouse Lab in Alderley Park	Wellcome Sanger Institute for the COVID-19 Genomics UK (COG-UK) Consortium	Jacquelyn Wynn, Mairead Hyland, The Lighthouse Lab in Alderley Park and Alex Alderton, Roberto Amato, Sonia Goncalves, Ewan Harrison, David K. Jackson, Ian Johnston, Dominic Kwiatkowski, Cordelia Langford, John Sillitoe on behalf of the Wellcome Sanger Institute COVID-19 Surveillance Team
EPI_ISL_675953	Lighthouse Lab in Milton Keynes	Wellcome Sanger Institute for the COVID-19 Genomics UK (COG-UK) Consortium	The Lighthouse Lab in Milton Keynes and Alex Alderton, Roberto Amato, Sonia Goncalves, Ewan Harrison, David K. Jackson, Ian Johnston, Dominic Kwiatkowski, Cordelia Langford, John Sillitoe on behalf of the Wellcome Sanger Institute COVID-19 Surveillance Team
EPI_ISL_675954	Lighthouse Lab in Alderley Park	Wellcome Sanger Institute for the COVID-19 Genomics UK (COG-UK) Consortium	Jacquelyn Wynn, Mairead Hyland, The Lighthouse Lab in Alderley Park and Alex Alderton, Roberto Amato, Sonia Goncalves, Ewan Harrison, David K. Jackson, Ian Johnston, Dominic Kwiatkowski, Cordelia Langford, John Sillitoe on behalf of the Wellcome Sanger Institute COVID-19 Surveillance Team
EPI_ISL_675955, EPI_ISL_675957, EPI_ISL_675958, EPI_ISL_675959, EPI_ISL_675960, EPI_ISL_675961	Lighthouse Lab in Milton Keynes	Wellcome Sanger Institute for the COVID-19 Genomics UK (COG-UK) Consortium	The Lighthouse Lab in Milton Keynes and Alex Alderton, Roberto Amato, Sonia Goncalves, Ewan Harrison, David K. Jackson, Ian Johnston, Dominic Kwiatkowski, Cordelia Langford, John Sillitoe on behalf of the Wellcome Sanger Institute COVID-19 Surveillance Team
EPI_ISL_675962	Lighthouse Lab in Alderley Park	Wellcome Sanger Institute for the COVID-19 Genomics UK (COG-UK) Consortium	Jacquelyn Wynn, Mairead Hyland, The Lighthouse Lab in Alderley Park and Alex Alderton, Roberto Amato, Sonia Goncalves, Ewan Harrison, David K. Jackson, Ian Johnston, Dominic Kwiatkowski, Cordelia Langford, John Sillitoe on behalf of the Wellcome Sanger Institute COVID-19 Surveillance Team
EPI_ISL_675963, EPI_ISL_675964	Lighthouse Lab in Milton Keynes	Wellcome Sanger Institute for the COVID-19 Genomics UK (COG-UK) Consortium	The Lighthouse Lab in Milton Keynes and Alex Alderton, Roberto Amato, Sonia Goncalves, Ewan Harrison, David K. Jackson, Ian Johnston, Dominic Kwiatkowski, Cordelia Langford, John Sillitoe on behalf of the Wellcome Sanger Institute COVID-19 Surveillance Team
EPI_ISL_675965	Lighthouse Lab in Alderley Park	Wellcome Sanger Institute for the COVID-19 Genomics UK (COG-UK) Consortium	Jacquelyn Wynn, Mairead Hyland, The Lighthouse Lab in Alderley Park and Alex Alderton, Roberto Amato, Sonia Goncalves, Ewan Harrison, David K. Jackson, Ian Johnston, Dominic Kwiatkowski, Cordelia Langford, John Sillitoe on behalf of the Wellcome Sanger Institute COVID-19 Surveillance Team
EPI_ISL_675966, EPI_ISL_675967	Lighthouse Lab in Milton Keynes	Wellcome Sanger Institute for the COVID-19 Genomics UK (COG-UK) Consortium	The Lighthouse Lab in Milton Keynes and Alex Alderton, Roberto Amato, Sonia Goncalves, Ewan Harrison, David K. Jackson, Ian Johnston, Dominic Kwiatkowski, Cordelia Langford, John Sillitoe on behalf of the Wellcome Sanger Institute COVID-19 Surveillance Team
EPI_ISL_675968	Lighthouse Lab in Alderley Park	Wellcome Sanger Institute for the COVID-19 Genomics UK (COG-UK) Consortium	Jacquelyn Wynn, Mairead Hyland, The Lighthouse Lab in Alderley Park and Alex Alderton, Roberto Amato, Sonia Goncalves, Ewan Harrison, David K. Jackson, Ian Johnston, Dominic Kwiatkowski, Cordelia Langford, John Sillitoe on behalf of the Wellcome Sanger Institute COVID-19 Surveillance Team
EPI_ISL_675969, EPI_ISL_675970, EPI_ISL_675971	Lighthouse Lab in Milton Keynes	Wellcome Sanger Institute for the COVID-19 Genomics UK (COG-UK) Consortium	The Lighthouse Lab in Milton Keynes and Alex Alderton, Roberto Amato, Sonia Goncalves, Ewan Harrison, David K. Jackson, Ian Johnston, Dominic Kwiatkowski, Cordelia Langford, John Sillitoe on behalf of the Wellcome Sanger Institute COVID-19 Surveillance Team
EPI_ISL_675973, EPI_ISL_675974	Lighthouse Lab in Alderley Park	Wellcome Sanger Institute for the COVID-19 Genomics UK (COG-UK) Consortium	Jacquelyn Wynn, Mairead Hyland, The Lighthouse Lab in Alderley Park and Alex Alderton, Roberto Amato, Sonia Goncalves, Ewan Harrison, David K. Jackson, Ian Johnston, Dominic Kwiatkowski, Cordelia Langford, John Sillitoe on behalf of the Wellcome Sanger Institute COVID-19 Surveillance Team
EPI_ISL_675975	Lighthouse Lab in Milton Keynes	Wellcome Sanger Institute for the COVID-19 Genomics UK (COG-UK) Consortium	The Lighthouse Lab in Milton Keynes and Alex Alderton, Roberto Amato, Sonia Goncalves, Ewan Harrison, David K. Jackson, Ian Johnston, Dominic Kwiatkowski, Cordelia Langford, John Sillitoe on behalf of the Wellcome Sanger Institute COVID-19 Surveillance Team
EPI_ISL_675976	Lighthouse Lab in Alderley Park	Wellcome Sanger Institute for the COVID-19 Genomics UK (COG-UK) Consortium	Jacquelyn Wynn, Mairead Hyland, The Lighthouse Lab in Alderley Park and Alex Alderton, Roberto Amato, Sonia Goncalves, Ewan Harrison, David K. Jackson, Ian Johnston, Dominic Kwiatkowski, Cordelia Langford, John Sillitoe on behalf of the Wellcome Sanger Institute COVID-19 Surveillance Team
EPI_ISL_675977	Lighthouse Lab in Milton Keynes	Wellcome Sanger Institute for the COVID-19 Genomics UK (COG-UK) Consortium	The Lighthouse Lab in Milton Keynes and Alex Alderton, Roberto Amato, Sonia Goncalves, Ewan Harrison, David K. Jackson, Ian Johnston, Dominic Kwiatkowski, Cordelia Langford, John Sillitoe on behalf of the Wellcome Sanger Institute COVID-19 Surveillance Team
EPI_ISL_675978	Lighthouse Lab in Alderley Park	Wellcome Sanger Institute for the COVID-19 Genomics UK (COG-UK) Consortium	Jacquelyn Wynn, Mairead Hyland, The Lighthouse Lab in Alderley Park and Alex Alderton, Roberto Amato, Sonia Goncalves, Ewan Harrison, David K. Jackson, Ian Johnston, Dominic Kwiatkowski, Cordelia Langford, John Sillitoe on behalf of the Wellcome Sanger Institute COVID-19 Surveillance Team
EPI_ISL_675979	Lighthouse Lab in Milton Keynes	Wellcome Sanger Institute for the COVID-19 Genomics UK (COG-UK) Consortium	The Lighthouse Lab in Milton Keynes and Alex Alderton, Roberto Amato, Sonia Goncalves, Ewan Harrison, David K. Jackson, Ian Johnston, Dominic Kwiatkowski, Cordelia Langford, John Sillitoe on behalf of the Wellcome Sanger Institute COVID-19 Surveillance Team
EPI_ISL_675980	Lighthouse Lab in Alderley Park	Wellcome Sanger Institute for the COVID-19 Genomics UK (COG-UK) Consortium	Jacquelyn Wynn, Mairead Hyland, The Lighthouse Lab in Alderley Park and Alex Alderton, Roberto Amato, Sonia Goncalves, Ewan Harrison, David K. Jackson, Ian Johnston, Dominic Kwiatkowski, Cordelia Langford, John Sillitoe on behalf of the Wellcome Sanger Institute COVID-19 Surveillance Team
EPI_ISL_675981	Lighthouse Lab in Milton Keynes	Wellcome Sanger Institute for the COVID-19 Genomics UK (COG-UK) Consortium	The Lighthouse Lab in Milton Keynes and Alex Alderton, Roberto Amato, Sonia Goncalves, Ewan Harrison, David K. Jackson, Ian Johnston, Dominic Kwiatkowski, Cordelia Langford, John Sillitoe on behalf of the Wellcome Sanger Institute COVID-19 Surveillance Team
EPI_ISL_675982	Lighthouse Lab in Alderley Park	Wellcome Sanger Institute for the COVID-19 Genomics UK (COG-UK) Consortium	Jacquelyn Wynn, Mairead Hyland, The Lighthouse Lab in Alderley Park and Alex Alderton, Roberto Amato, Sonia Goncalves, Ewan Harrison, David K. Jackson, Ian Johnston, Dominic Kwiatkowski, Cordelia Langford, John Sillitoe on behalf of the Wellcome Sanger Institute COVID-19 Surveillance Team
EPI_ISL_675983, EPI_ISL_675984, EPI_ISL_675985	Lighthouse Lab in Milton Keynes	Wellcome Sanger Institute for the COVID-19 Genomics UK (COG-UK) Consortium	The Lighthouse Lab in Milton Keynes and Alex Alderton, Roberto Amato, Sonia Goncalves, Ewan Harrison, David K. Jackson, Ian Johnston, Dominic Kwiatkowski, Cordelia Langford, John Sillitoe on behalf of the Wellcome Sanger Institute COVID-19 Surveillance Team
EPI_ISL_675986, EPI_ISL_675987, EPI_ISL_675988	Lighthouse Lab in Alderley Park	Wellcome Sanger Institute for the COVID-19 Genomics UK (COG-UK) Consortium	Jacquelyn Wynn, Mairead Hyland, The Lighthouse Lab in Alderley Park and Alex Alderton, Roberto Amato, Sonia Goncalves, Ewan Harrison, David K. Jackson, Ian Johnston, Dominic Kwiatkowski, Cordelia Langford, John Sillitoe on behalf of the Wellcome Sanger Institute COVID-19 Surveillance Team
EPI_ISL_675989, EPI_ISL_675990, EPI_ISL_675991, EPI_ISL_675992	Lighthouse Lab in Milton Keynes	Wellcome Sanger Institute for the COVID-19 Genomics UK (COG-UK) Consortium	The Lighthouse Lab in Milton Keynes and Alex Alderton, Roberto Amato, Sonia Goncalves, Ewan Harrison, David K. Jackson, Ian Johnston, Dominic Kwiatkowski, Cordelia Langford, John Sillitoe on behalf of the Wellcome Sanger Institute COVID-19 Surveillance Team
EPI_ISL_675993	Lighthouse Lab in Alderley Park	Wellcome Sanger Institute for the COVID-19 Genomics UK (COG-UK) Consortium	Jacquelyn Wynn, Mairead Hyland, The Lighthouse Lab in Alderley Park and Alex Alderton, Roberto Amato, Sonia Goncalves, Ewan Harrison, David K. Jackson, Ian Johnston, Dominic Kwiatkowski, Cordelia Langford, John Sillitoe on behalf of the Wellcome Sanger Institute COVID-19 Surveillance Team
EPI_ISL_675994, EPI_ISL_675995, EPI_ISL_675996, EPI_ISL_675997, EPI_ISL_675998, EPI_ISL_675999	Lighthouse Lab in Milton Keynes	Wellcome Sanger Institute for the COVID-19 Genomics UK (COG-UK) Consortium	The Lighthouse Lab in Milton Keynes and Alex Alderton, Roberto Amato, Sonia Goncalves, Ewan Harrison, David K. Jackson, Ian Johnston, Dominic Kwiatkowski, Cordelia Langford, John Sillitoe on behalf of the Wellcome Sanger Institute COVID-19 Surveillance Team
EPI_ISL_676000	Lighthouse Lab in Alderley Park	Wellcome Sanger Institute for the COVID-19 Genomics UK (COG-UK) Consortium	Jacquelyn Wynn, Mairead Hyland, The Lighthouse Lab in Alderley Park and Alex Alderton, Roberto Amato, Sonia Goncalves, Ewan Harrison, David K. Jackson, Ian Johnston, Dominic Kwiatkowski, Cordelia Langford, John Sillitoe on behalf of the Wellcome Sanger Institute COVID-19 Surveillance Team
EPI_ISL_676001	Lighthouse Lab in Milton Keynes	Wellcome Sanger Institute for the COVID-19 Genomics UK (COG-UK) Consortium	The Lighthouse Lab in Milton Keynes and Alex Alderton, Roberto Amato, Sonia Goncalves, Ewan Harrison, David K. Jackson, Ian Johnston, Dominic Kwiatkowski, Cordelia Langford, John Sillitoe on behalf of the Wellcome Sanger Institute COVID-19 Surveillance Team
EPI_ISL_676002	Lighthouse Lab in Alderley Park	Wellcome Sanger Institute for the COVID-19 Genomics UK (COG-UK) Consortium	Jacquelyn Wynn, Mairead Hyland, The Lighthouse Lab in Alderley Park and Alex Alderton, Roberto Amato, Sonia Goncalves, Ewan Harrison, David K. Jackson, Ian Johnston, Dominic Kwiatkowski, Cordelia Langford, John Sillitoe on behalf of the Wellcome Sanger Institute COVID-19 Surveillance Team
EPI_ISL_676003, EPI_ISL_676004, EPI_ISL_676005	Lighthouse Lab in Milton Keynes	Wellcome Sanger Institute for the COVID-19 Genomics UK (COG-UK) Consortium	The Lighthouse Lab in Milton Keynes and Alex Alderton, Roberto Amato, Sonia Goncalves, Ewan Harrison, David K. Jackson, Ian Johnston, Dominic Kwiatkowski, Cordelia Langford, John Sillitoe on behalf of the Wellcome Sanger Institute COVID-19 Surveillance Team
EPI_ISL_676006	Lighthouse Lab in Alderley Park	Wellcome Sanger Institute for the COVID-19 Genomics UK (COG-UK) Consortium	Jacquelyn Wynn, Mairead Hyland, The Lighthouse Lab in Alderley Park and Alex Alderton, Roberto Amato, Sonia Goncalves, Ewan Harrison, David K. Jackson, Ian Johnston, Dominic Kwiatkowski, Cordelia Langford, John Sillitoe on behalf of the Wellcome Sanger Institute COVID-19 Surveillance Team
EPI_ISL_676007, EPI_ISL_676008, EPI_ISL_676009	Lighthouse Lab in Milton Keynes	Wellcome Sanger Institute for the COVID-19 Genomics UK (COG-UK) Consortium	The Lighthouse Lab in Milton Keynes and Alex Alderton, Roberto Amato, Sonia Goncalves, Ewan Harrison, David K. Jackson, Ian Johnston, Dominic Kwiatkowski, Cordelia Langford, John Sillitoe on behalf of the Wellcome Sanger Institute COVID-19 Surveillance Team

EPI_ISL_676010	Lighthouse Lab in Alderley Park	Wellcome Sanger Institute for the COVID-19 Genomics UK (COG-UK) Consortium	Jacquelyn Wynn, Mairead Hyland, The Lighthouse Lab in Alderley Park and Alex Alderton, Roberto Amato, Sonia Goncalves, Ewan Harrison, David K. Jackson, Ian Johnston, Dominic Kwiatkowski, Cordelia Langford, John Sillitoe on behalf of the Wellcome Sanger Institute COVID-19 Surveillance Team
EPI_ISL_676011, EPI_ISL_676012, EPI_ISL_676013, EPI_ISL_676014, EPI_ISL_676015, EPI_ISL_676016, EPI_ISL_676017, EPI_ISL_676018, EPI_ISL_676019, EPI_ISL_676020, EPI_ISL_676021, EPI_ISL_676022, EPI_ISL_676023, EPI_ISL_676024, EPI_ISL_676025, EPI_ISL_676026, EPI_ISL_676027, EPI_ISL_676028, EPI_ISL_676029, EPI_ISL_676030, EPI_ISL_676031, EPI_ISL_676032, EPI_ISL_676033, EPI_ISL_676034, EPI_ISL_676035, EPI_ISL_676036, EPI_ISL_676037, EPI_ISL_676038, EPI_ISL_676039, EPI_ISL_676040, EPI_ISL_676041, EPI_ISL_676042, EPI_ISL_676043, EPI_ISL_676044, EPI_ISL_676045, EPI_ISL_676046, EPI_ISL_676047, EPI_ISL_676048, EPI_ISL_676049, EPI_ISL_676050, EPI_ISL_676051, EPI_ISL_676052, EPI_ISL_676053, EPI_ISL_676054, EPI_ISL_676055, EPI_ISL_676056, EPI_ISL_676057, EPI_ISL_676058, EPI_ISL_676059, EPI_ISL_676060, EPI_ISL_676061, EPI_ISL_676062, EPI_ISL_676063, EPI_ISL_676064, EPI_ISL_676065, EPI_ISL_676066, EPI_ISL_676067, EPI_ISL_676068, EPI_ISL_676069, EPI_ISL_676070, EPI_ISL_676071, EPI_ISL_676072, EPI_ISL_676073, EPI_ISL_676074, EPI_ISL_676075, EPI_ISL_676076, EPI_ISL_676077, EPI_ISL_676078, EPI_ISL_676079, EPI_ISL_676080, EPI_ISL_676081, EPI_ISL_676082, EPI_ISL_676083, EPI_ISL_676084, EPI_ISL_676085, EPI_ISL_676086, EPI_ISL_676087, EPI_ISL_676088, EPI_ISL_676089, EPI_ISL_676090, EPI_ISL_676091, EPI_ISL_676092, EPI_ISL_676093, EPI_ISL_676094, EPI_ISL_676095, EPI_ISL_676096, EPI_ISL_676097, EPI_ISL_676098, EPI_ISL_676099, EPI_ISL_676100, EPI_ISL_676101, EPI_ISL_676102, EPI_ISL_676103, EPI_ISL_676104, EPI_ISL_676105, EPI_ISL_676106, EPI_ISL_676107, EPI_ISL_676108, EPI_ISL_676109, EPI_ISL_676110, EPI_ISL_676111, EPI_ISL_676112, EPI_ISL_676113, EPI_ISL_676114, EPI_ISL_676115, EPI_ISL_676116, EPI_ISL_676117, EPI_ISL_676118, EPI_ISL_676119, EPI_ISL_676120, EPI_ISL_676121, EPI_ISL_676122, EPI_ISL_676123, EPI_ISL_676124, EPI_ISL_676125, EPI_ISL_676126, EPI_ISL_676127, EPI_ISL_676128, EPI_ISL_676129, EPI_ISL_676130, EPI_ISL_676131, EPI_ISL_676132, EPI_ISL_676133, EPI_ISL_676134, EPI_ISL_676135, EPI_ISL_676136, EPI_ISL_676137, EPI_ISL_676138, EPI_ISL_676139, EPI_ISL_676140, EPI_ISL_676141, EPI_ISL_676142, EPI_ISL_676143, EPI_ISL_676144, EPI_ISL_676145, EPI_ISL_676146, EPI_ISL_676147, EPI_ISL_676148, EPI_ISL_676149, EPI_ISL_676150, EPI_ISL_676151, EPI_ISL_676152, EPI_ISL_676153, EPI_ISL_676154, EPI_ISL_676155, EPI_ISL_676156, EPI_ISL_676157, EPI_ISL_676158, EPI_ISL_676159, EPI_ISL_676160, EPI_ISL_676161, EPI_ISL_676162, EPI_ISL_676163, EPI_ISL_676164, EPI_ISL_676165, EPI_ISL_676166, EPI_ISL_676167, EPI_ISL_676168, EPI_ISL_676169, EPI_ISL_676170, EPI_ISL_676171, EPI_ISL_676172, EPI_ISL_676173, EPI_ISL_676174, EPI_ISL_676175, EPI_ISL_676176, EPI_ISL_676177, EPI_ISL_676178, EPI_ISL_676179, EPI_ISL_676180, EPI_ISL_676181, EPI_ISL_676182, EPI_ISL_676183, EPI_ISL_676184, EPI_ISL_676185, EPI_ISL_676186, EPI_ISL_676187, EPI_ISL_676188, EPI_ISL_676189, EPI_ISL_676190, EPI_ISL_676191, EPI_ISL_676192, EPI_ISL_676193, EPI_ISL_676194, EPI_ISL_676195, EPI_ISL_676196, EPI_ISL_676197, EPI_ISL_676198, EPI_ISL_676199, EPI_ISL_676200, EPI_ISL_676201, EPI_ISL_676202, EPI_ISL_676203, EPI_ISL_676204, EPI_ISL_676205, EPI_ISL_676206, EPI_ISL_676207, EPI_ISL_676208, EPI_ISL_676209, EPI_ISL_676210, EPI_ISL_676211, EPI_ISL_676212, EPI_ISL_676213, EPI_ISL_676214, EPI_ISL_676215, EPI_ISL_676216, EPI_ISL_676217, EPI_ISL_676218, EPI_ISL_676219, EPI_ISL_676220, EPI_ISL_676221, EPI_ISL_676222, EPI_ISL_676223, EPI_ISL_676224, EPI_ISL_676225, EPI_ISL_676226, EPI_ISL_676227, EPI_ISL_676228, EPI_ISL_676229, EPI_ISL_676230, EPI_ISL_676231, EPI_ISL_676232, EPI_ISL_676233, EPI_ISL_676234, EPI_ISL_676235, EPI_ISL_676236, EPI_ISL_676237, EPI_ISL_676238, EPI_ISL_676239, EPI_ISL_676240, EPI_ISL_676241, EPI_ISL_676242, EPI_ISL_676243, EPI_ISL_676244, EPI_ISL_676245, EPI_ISL_676246, EPI_ISL_676247, EPI_ISL_676248, EPI_ISL_676249, EPI_ISL_676250, EPI_ISL_676251, EPI_ISL_676252, EPI_ISL_676253, EPI_ISL_676254, EPI_ISL_676255, EPI_ISL_676256, EPI_ISL_676257, EPI_ISL_676258, EPI_ISL_676259, EPI_ISL_676260, EPI_ISL_676261, EPI_ISL_676262, EPI_ISL_676263, EPI_ISL_676264, EPI_ISL_676265, EPI_ISL_676266, EPI_ISL_676267, EPI_ISL_676268, EPI_ISL_676269, EPI_ISL_676270, EPI_ISL_676271, EPI_ISL_676272, EPI_ISL_676273, EPI_ISL_676274, EPI_ISL_676275, EPI_ISL_676276, EPI_ISL_676277, EPI_ISL_676278, EPI_ISL_676279, EPI_ISL_676280, EPI_ISL_676281, EPI_ISL_676282, EPI_ISL_676283, EPI_ISL_676284, EPI_ISL_676285, EPI_ISL_676286, EPI_ISL_676287, EPI_ISL_676288, EPI_ISL_676289, EPI_ISL_676290, EPI_ISL_676291, EPI_ISL_676292, EPI_ISL_676293, EPI_ISL_676294, EPI_ISL_676295, EPI_ISL_676296, EPI_ISL_676297, EPI_ISL_676298, EPI_ISL_676299, EPI_ISL_676300, EPI_ISL_676301, EPI_ISL_676302, EPI_ISL_676303, EPI_ISL_676304, EPI_ISL_676305, EPI_ISL_676306, EPI_ISL_676308, EPI_ISL_676309, EPI_ISL_676310, EPI_ISL_676311, EPI_ISL_676312			
see above	Lighthouse Lab in Milton Keynes	Wellcome Sanger Institute for the COVID-19 Genomics UK (COG-UK) Consortium	The Lighthouse Lab in Milton Keynes and Alex Alderton, Roberto Amato, Sonia Goncalves, Ewan Harrison, David K. Jackson, Ian Johnston, Dominic Kwiatkowski, Cordelia Langford, John Sillitoe on behalf of the Wellcome Sanger Institute COVID-19 Surveillance Team
EPI_ISL_676313, EPI_ISL_676314, EPI_ISL_676315, EPI_ISL_676316, EPI_ISL_676317, EPI_ISL_676318, EPI_ISL_676319, EPI_ISL_676320, EPI_ISL_676321, EPI_ISL_676322, EPI_ISL_676323, EPI_ISL_676324, EPI_ISL_676325, EPI_ISL_676326, EPI_ISL_676327, EPI_ISL_676328, EPI_ISL_676329, EPI_ISL_676330, EPI_ISL_676331, EPI_ISL_676332, EPI_ISL_676333, EPI_ISL_676334, EPI_ISL_676335, EPI_ISL_676336, EPI_ISL_676337, EPI_ISL_676338, EPI_ISL_676339, EPI_ISL_676340, EPI_ISL_676341, EPI_ISL_676342, EPI_ISL_676343, EPI_ISL_676344, EPI_ISL_676345, EPI_ISL_676346, EPI_ISL_676347, EPI_ISL_676348, EPI_ISL_676349, EPI_ISL_676350, EPI_ISL_676351, EPI_ISL_676352, EPI_ISL_676353, EPI_ISL_676354, EPI_ISL_676355, EPI_ISL_676356, EPI_ISL_676357, EPI_ISL_676358, EPI_ISL_676359, EPI_ISL_676360, EPI_ISL_676361, EPI_ISL_676362, EPI_ISL_676363, EPI_ISL_676364, EPI_ISL_676365, EPI_ISL_676366, EPI_ISL_676368, EPI_ISL_676369, EPI_ISL_676370, EPI_ISL_676371, EPI_ISL_676372, EPI_ISL_676373, EPI_ISL_676374, EPI_ISL_676375, EPI_ISL_676376, EPI_ISL_676377, EPI_ISL_676378, EPI_ISL_676379, EPI_ISL_676380, EPI_ISL_676381, EPI_ISL_676382			
see above	Lighthouse Lab in Glasgow	Wellcome Sanger Institute for the COVID-19 Genomics UK (COG-UK) Consortium	Harper VanSteenhouse, Yumi Kasai, David Gray, Carol Clugston, Anna Dominiczak and Alex Alderton, Roberto Amato, Sonia Goncalves, Ewan Harrison, David K. Jackson, Ian Johnston, Dominic Kwiatkowski, Cordelia Langford, John Sillitoe on behalf of the Wellcome Sanger Institute COVID-19 Surveillance Team
EPI_ISL_676383	Lighthouse Lab in Cambridge	Wellcome Sanger Institute for the COVID-19 Genomics UK (COG-UK) Consortium	Rob Howes, The Lighthouse Lab in Cambridge and Alex Alderton, Roberto Amato, Sonia Goncalves, Ewan Harrison, David K. Jackson, Ian Johnston, Dominic Kwiatkowski, Cordelia Langford, John Sillitoe on behalf of the Wellcome Sanger Institute COVID-19 Surveillance Team
EPI_ISL_676384, EPI_ISL_676385, EPI_ISL_676386, EPI_ISL_676387, EPI_ISL_676388, EPI_ISL_676389, EPI_ISL_676390, EPI_ISL_676391, EPI_ISL_676392, EPI_ISL_676393, EPI_ISL_676394, EPI_ISL_676395, EPI_ISL_676396, EPI_ISL_676397, EPI_ISL_676398, EPI_ISL_676399, EPI_ISL_676400, EPI_ISL_676401, EPI_ISL_676402, EPI_ISL_676403, EPI_ISL_676404, EPI_ISL_676405, EPI_ISL_676406, EPI_ISL_676408, EPI_ISL_676409, EPI_ISL_676410, EPI_ISL_676411, EPI_ISL_676412, EPI_ISL_676413, EPI_ISL_676414, EPI_ISL_676415, EPI_ISL_676416, EPI_ISL_676417, EPI_ISL_676418, EPI_ISL_676419, EPI_ISL_676420, EPI_ISL_676421, EPI_ISL_676422, EPI_ISL_676423, EPI_ISL_676424, EPI_ISL_676425, EPI_ISL_676426, EPI_ISL_676427, EPI_ISL_676428, EPI_ISL_676429, EPI_ISL_676430, EPI_ISL_676431, EPI_ISL_676432, EPI_ISL_676433, EPI_ISL_676434, EPI_ISL_676435, EPI_ISL_676436, EPI_ISL_676437, EPI_ISL_676438, EPI_ISL_676439, EPI_ISL_676440, EPI_ISL_676442, EPI_ISL_676443, EPI_ISL_676444, EPI_ISL_676446, EPI_ISL_676447, EPI_ISL_676448, EPI_ISL_676449, EPI_ISL_676451, EPI_ISL_676452, EPI_ISL_676453, EPI_ISL_676454, EPI_ISL_676455, EPI_ISL_676456, EPI_ISL_676457, EPI_ISL_676458, EPI_ISL_676459, EPI_ISL_676460, EPI_ISL_676461, EPI_ISL_676462, EPI_ISL_676463, EPI_ISL_676464, EPI_ISL_676465, EPI_ISL_676466, EPI_ISL_676467, EPI_ISL_676468, EPI_ISL_676469, EPI_ISL_676470, EPI_ISL_676471, EPI_ISL_676472, EPI_ISL_676473, EPI_ISL_676474, EPI_ISL_676475, EPI_ISL_676476, EPI_ISL_676477, EPI_ISL_676478, EPI_ISL_676479, EPI_ISL_676480, EPI_ISL_676481, EPI_ISL_676482, EPI_ISL_676483, EPI_ISL_676485, EPI_ISL_676486, EPI_ISL_676487, EPI_ISL_676488			
see above	Lighthouse Lab in Glasgow	Wellcome Sanger Institute for the COVID-19 Genomics UK (COG-UK) Consortium	Harper VanSteenhouse, Yumi Kasai, David Gray, Carol Clugston, Anna Dominiczak and Alex Alderton, Roberto Amato, Sonia Goncalves, Ewan Harrison, David K. Jackson, Ian Johnston, Dominic Kwiatkowski, Cordelia Langford, John Sillitoe on behalf of the Wellcome Sanger Institute COVID-19 Surveillance Team
EPI_ISL_676489, EPI_ISL_676490, EPI_ISL_676491	Klinisk mikrobiologi	The Public Health Agency of Sweden	Department of Microbiology, The Public Health Agency of Sweden
EPI_ISL_676492	Klinisk Mikrobiologi	The Public Health Agency of Sweden	Department of Microbiology, The Public Health Agency of Sweden
EPI_ISL_676493	Orebro klinisk mikrobiologi	The Public Health Agency of Sweden	Department of Microbiology, The Public Health Agency of Sweden
EPI_ISL_676494, EPI_ISL_676495, EPI_ISL_676496	Klinisk mikrobiologi	The Public Health Agency of Sweden	Department of Microbiology, The Public Health Agency of Sweden
EPI_ISL_676497, EPI_ISL_676498, EPI_ISL_676499, EPI_ISL_676500, EPI_ISL_676501	Klinisk Mikrobiologi	The Public Health Agency of Sweden	Department of Microbiology, The Public Health Agency of Sweden
EPI_ISL_676502, EPI_ISL_676503, EPI_ISL_676504, EPI_ISL_676505, EPI_ISL_676506, EPI_ISL_676507, EPI_ISL_676508	Klinisk mikrobiologi	The Public Health Agency of Sweden	Department of Microbiology, The Public Health Agency of Sweden
EPI_ISL_676510, EPI_ISL_676511	Respiratory Virus Unit, Microbiology Services Colindale, Public Health England	COVID-19 Genomics UK (COG-UK) Consortium	PHE Covid Sequencing Team
EPI_ISL_676513	Orebro klinisk mikrobiologi	The Public Health Agency of Sweden	Department of Microbiology, The Public Health Agency of Sweden
EPI_ISL_676514	Gavleborg	The Public Health Agency of Sweden	Department of Microbiology, The Public Health Agency of Sweden
EPI_ISL_676515	Klinisk mikrobiologi, Viruslab	The Public Health Agency of Sweden	Department of Microbiology, The Public Health Agency of Sweden
EPI_ISL_676516, EPI_ISL_676517	Laboratoriemedicin, Klinisk mikrobiologi	The Public Health Agency of Sweden	Department of Microbiology, The Public Health Agency of Sweden
EPI_ISL_676518	Klinisk Mikrobiologi	The Public Health Agency of Sweden	Department of Microbiology, The Public Health Agency of Sweden
EPI_ISL_676519	Klinsisk mikrobiologi Linkoping	The Public Health Agency of Sweden	Department of Microbiology, The Public Health Agency of Sweden
EPI_ISL_676520	Klinisk mikrobiologi	The Public Health Agency of Sweden	Department of Microbiology, The Public Health Agency of Sweden
EPI_ISL_676527, EPI_ISL_676528, EPI_ISL_676529, EPI_ISL_676530	Uppsala klinisk mikrobiologi	The Public Health Agency of Sweden	Department of Microbiology, The Public Health Agency of Sweden
EPI_ISL_676531, EPI_ISL_676532	Klinisk mikrobiologi	The Public Health Agency of Sweden	Department of Microbiology, The Public Health Agency of Sweden
EPI_ISL_676534	Kalmar klinisk mikrobiologi	The Public Health Agency of Sweden	Department of Microbiology, The Public Health Agency of Sweden
EPI_ISL_676535, EPI_ISL_676536, EPI_ISL_676537, EPI_ISL_676538, EPI_ISL_676539, EPI_ISL_676540	Laboratoriemedicin, Klinisk mikrobiologi	The Public Health Agency of Sweden	Department of Microbiology, The Public Health Agency of Sweden
EPI_ISL_676575, EPI_ISL_676579, EPI_ISL_676582, EPI_ISL_676583, EPI_ISL_676584, EPI_ISL_676592, EPI_ISL_676601	Scientific Veterinary Institute Novi Sad	Veterinary Specialized Institute "Kraljevo", Serbia	Vidanovic,D., Tesovic,B., Knezevic,A., Jovanovic,T., Jankovic,M., Sekler,M., Banovic Djeri,B., Petrovic,T., Volkening,J., Afonso,C.
EPI_ISL_676604, EPI_ISL_676605, EPI_ISL_676606, EPI_ISL_676607, EPI_ISL_676608, EPI_ISL_676609, EPI_ISL_676610, EPI_ISL_676611, EPI_ISL_676612, EPI_ISL_676614, EPI_ISL_676615, EPI_ISL_676616, EPI_ISL_676617, EPI_ISL_676618, EPI_ISL_676619, EPI_ISL_676620, EPI_ISL_676621, EPI_ISL_676622, EPI_ISL_676623, EPI_ISL_676624, EPI_ISL_676625, EPI_ISL_676626, EPI_ISL_676627, EPI_ISL_676628, EPI_ISL_676629, EPI_ISL_676630, EPI_ISL_676631, EPI_ISL_676632, EPI_ISL_676633, EPI_ISL_676634, EPI_ISL_676635, EPI_ISL_676636, EPI_ISL_676637, EPI_ISL_676638, EPI_ISL_676642, EPI_ISL_676643,			

EPI_ISL_676644, EPI_ISL_676645, EPI_ISL_676646			
see above	Texas Department of State Health Services	Texas Department of State Health Services	Rashmi Tuladhar, Bonnie Oh, Jenny Zhang, Maliha Rahman, Anita Pokharel, Myong Koag, Chung Wang, Rachel Lee, Grace Kubin, Mayela Pedrueza, James Daniel Bonser
EPI_ISL_676651	Wadsworth Center, New York State Department.of Health	Wadsworth Center, New York State Department.of Health	Kirsten St. George, Daryl M. Lamson, Alexis Russel, Jonathan Plitnick, Navjot Singh, John Kelly, Sara Griesemer, Erasmus Schneider, Erica Lasek-Nesselquist
EPI_ISL_676652	Masonic Medical Research Institute	Wadsworth Center, New York State Department.of Health	Nathan Tucker, Kirsten St. George, Daryl M. Lamson, Alexis Russel, Jonathan Plitnick, Navjot Singh, John Kelly, Sara Griesemer, Erasmus Schneider, Erica Lasek-Nesselquist
EPI_ISL_676653, EPI_ISL_676657	Wadsworth Center, New York State Department.of Health	Wadsworth Center, New York State Department.of Health	Kirsten St. George, Daryl M. Lamson, Alexis Russel, Jonathan Plitnick, Navjot Singh, John Kelly, Sara Griesemer, Erasmus Schneider, Erica Lasek-Nesselquist
EPI_ISL_676659	Masonic Medical Research Institute	Wadsworth Center, New York State Department.of Health	Nathan Tucker, Kirsten St. George, Daryl M. Lamson, Alexis Russel, Jonathan Plitnick, Navjot Singh, John Kelly, Sara Griesemer, Erasmus Schneider, Erica Lasek-Nesselquist
EPI_ISL_676660	Wadsworth Center, New York State Department.of Health	Wadsworth Center, New York State Department.of Health	Kirsten St. George, Daryl M. Lamson, Alexis Russel, Jonathan Plitnick, Navjot Singh, John Kelly, Sara Griesemer, Erasmus Schneider, Erica Lasek-Nesselquist
EPI_ISL_676661	Masonic Medical Research Institute	Wadsworth Center, New York State Department.of Health	Nathan Tucker, Kirsten St. George, Daryl M. Lamson, Alexis Russel, Jonathan Plitnick, Navjot Singh, John Kelly, Sara Griesemer, Erasmus Schneider, Erica Lasek-Nesselquist
EPI_ISL_676662	Wadsworth Center, New York State Department.of Health	Wadsworth Center, New York State Department.of Health	Kirsten St. George, Daryl M. Lamson, Alexis Russel, Jonathan Plitnick, Navjot Singh, John Kelly, Sara Griesemer, Erasmus Schneider, Erica Lasek-Nesselquist
EPI_ISL_676663, EPI_ISL_676665	Masonic Medical Research Institute	Wadsworth Center, New York State Department.of Health	Nathan Tucker, Kirsten St. George, Daryl M. Lamson, Alexis Russel, Jonathan Plitnick, Navjot Singh, John Kelly, Sara Griesemer, Erasmus Schneider, Erica Lasek-Nesselquist
EPI_ISL_676666	Wadsworth Center, New York State Department.of Health	Wadsworth Center, New York State Department.of Health	Kirsten St. George, Daryl M. Lamson, Alexis Russel, Jonathan Plitnick, Navjot Singh, John Kelly, Sara Griesemer, Erasmus Schneider, Erica Lasek-Nesselquist
EPI_ISL_676668	Masonic Medical Research Institute	Wadsworth Center, New York State Department.of Health	Nathan Tucker, Kirsten St. George, Daryl M. Lamson, Alexis Russel, Jonathan Plitnick, Navjot Singh, John Kelly, Sara Griesemer, Erasmus Schneider, Erica Lasek-Nesselquist
EPI_ISL_676671	Wadsworth Center, New York State Department.of Health	Wadsworth Center, New York State Department.of Health	Kirsten St. George, Daryl M. Lamson, Alexis Russel, Jonathan Plitnick, Navjot Singh, John Kelly, Sara Griesemer, Erasmus Schneider, Erica Lasek-Nesselquist
EPI_ISL_676673	Masonic Medical Research Institute	Wadsworth Center, New York State Department.of Health	Nathan Tucker, Kirsten St. George, Daryl M. Lamson, Alexis Russel, Jonathan Plitnick, Navjot Singh, John Kelly, Sara Griesemer, Erasmus Schneider, Erica Lasek-Nesselquist
EPI_ISL_676676	Wadsworth Center, New York State Department.of Health	Wadsworth Center, New York State Department.of Health	Kirsten St. George, Daryl M. Lamson, Alexis Russel, Jonathan Plitnick, Navjot Singh, John Kelly, Sara Griesemer, Erasmus Schneider, Erica Lasek-Nesselquist
EPI_ISL_676680	Masonic Medical Research Institute	Wadsworth Center, New York State Department.of Health	Nathan Tucker, Kirsten St. George, Daryl M. Lamson, Alexis Russel, Jonathan Plitnick, Navjot Singh, John Kelly, Sara Griesemer, Erasmus Schneider, Erica Lasek-Nesselquist
EPI_ISL_676694, EPI_ISL_676700, EPI_ISL_676701, EPI_ISL_676706, EPI_ISL_676707	Wadsworth Center, New York State Department.of Health	Wadsworth Center, New York State Department.of Health	Kirsten St. George, Daryl M. Lamson, Alexis Russel, Jonathan Plitnick, Navjot Singh, John Kelly, Sara Griesemer, Erasmus Schneider, Erica Lasek-Nesselquist
EPI_ISL_676709, EPI_ISL_676713	Masonic Medical Research Institute	Wadsworth Center, New York State Department.of Health	Nathan Tucker, Kirsten St. George, Daryl M. Lamson, Alexis Russel, Jonathan Plitnick, Navjot Singh, John Kelly, Sara Griesemer, Erasmus Schneider, Erica Lasek-Nesselquist
EPI_ISL_676716, EPI_ISL_676718	Wadsworth Center, New York State Department.of Health	Wadsworth Center, New York State Department.of Health	Kirsten St. George, Daryl M. Lamson, Alexis Russel, Jonathan Plitnick, Navjot Singh, John Kelly, Sara Griesemer, Erasmus Schneider, Erica Lasek-Nesselquist
EPI_ISL_676726	Masonic Medical Research Institute	Wadsworth Center, New York State Department.of Health	Nathan Tucker, Kirsten St. George, Daryl M. Lamson, Alexis Russel, Jonathan Plitnick, Navjot Singh, John Kelly, Sara Griesemer, Erasmus Schneider, Erica Lasek-Nesselquist
EPI_ISL_676959, EPI_ISL_676979, EPI_ISL_676982, EPI_ISL_676983, EPI_ISL_676984, EPI_ISL_676985, EPI_ISL_676986, EPI_ISL_676987, EPI_ISL_676988, EPI_ISL_676989, EPI_ISL_676990, EPI_ISL_676991, EPI_ISL_676992, EPI_ISL_676993, EPI_ISL_676994			
see above	Wadsworth Center, New York State Department.of Health	Wadsworth Center, New York State Department.of Health	Kirsten St. George, Daryl M. Lamson, Alexis Russel, Jonathan Plitnick, Navjot Singh, John Kelly, Sara Griesemer, Erasmus Schneider, Erica Lasek-Nesselquist
EPI_ISL_676995, EPI_ISL_676996, EPI_ISL_676997, EPI_ISL_676998, EPI_ISL_676999, EPI_ISL_677000, EPI_ISL_677001, EPI_ISL_677002, EPI_ISL_677003, EPI_ISL_677004, EPI_ISL_677005, EPI_ISL_677006, EPI_ISL_677007, EPI_ISL_677008, EPI_ISL_677009, EPI_ISL_677010, EPI_ISL_677011, EPI_ISL_677012, EPI_ISL_677013, EPI_ISL_677014, EPI_ISL_677015, EPI_ISL_677016, EPI_ISL_677017, EPI_ISL_677018, EPI_ISL_677019, EPI_ISL_677020, EPI_ISL_677021, EPI_ISL_677022, EPI_ISL_677023, EPI_ISL_677024, EPI_ISL_677025			
see above	Masonic Medical Research Institute	Wadsworth Center, New York State Department.of Health	Nathan Tucker, Kirsten St. George, Daryl M. Lamson, Alexis Russel, Jonathan Plitnick, Navjot Singh, John Kelly, Sara Griesemer, Erasmus Schneider, Erica Lasek-Nesselquist
EPI_ISL_677026, EPI_ISL_677027, EPI_ISL_677028, EPI_ISL_677029, EPI_ISL_677030, EPI_ISL_677031, EPI_ISL_677032, EPI_ISL_677033, EPI_ISL_677034, EPI_ISL_677035, EPI_ISL_677036, EPI_ISL_677037, EPI_ISL_677038, EPI_ISL_677039, EPI_ISL_677040, EPI_ISL_677041, EPI_ISL_677042, EPI_ISL_677043, EPI_ISL_677044, EPI_ISL_677045, EPI_ISL_677046, EPI_ISL_677047, EPI_ISL_677048			
see above	Wadsworth Center, New York State Department.of Health	Wadsworth Center, New York State Department.of Health	Kirsten St. George, Daryl M. Lamson, Alexis Russel, Jonathan Plitnick, Navjot Singh, John Kelly, Sara Griesemer, Erasmus Schneider, Erica Lasek-Nesselquist
EPI_ISL_677049, EPI_ISL_677050, EPI_ISL_677051, EPI_ISL_677052, EPI_ISL_677053, EPI_ISL_677054, EPI_ISL_677055, EPI_ISL_677056, EPI_ISL_677057, EPI_ISL_677058, EPI_ISL_677059, EPI_ISL_677060, EPI_ISL_677061, EPI_ISL_677062, EPI_ISL_677063, EPI_ISL_677064, EPI_ISL_677065, EPI_ISL_677066, EPI_ISL_677067, EPI_ISL_677068, EPI_ISL_677069, EPI_ISL_677070, EPI_ISL_677071, EPI_ISL_677072, EPI_ISL_677073, EPI_ISL_677074, EPI_ISL_677075, EPI_ISL_677076			
see above	Masonic Medical Research Institute	Wadsworth Center, New York State Department.of Health	Nathan Tucker, Kirsten St. George, Daryl M. Lamson, Alexis Russel, Jonathan Plitnick, Navjot Singh, John Kelly, Sara Griesemer, Erasmus Schneider, Erica Lasek-Nesselquist
EPI_ISL_677077, EPI_ISL_677078, EPI_ISL_677079, EPI_ISL_677080, EPI_ISL_677081, EPI_ISL_677082, EPI_ISL_677083, EPI_ISL_677084, EPI_ISL_677085, EPI_ISL_677086, EPI_ISL_677087, EPI_ISL_677088, EPI_ISL_677089, EPI_ISL_677090, EPI_ISL_677091, EPI_ISL_677092, EPI_ISL_677093, EPI_ISL_677094, EPI_ISL_677095, EPI_ISL_677096, EPI_ISL_677097, EPI_ISL_677098, EPI_ISL_677099, EPI_ISL_677100, EPI_ISL_677101, EPI_ISL_677102, EPI_ISL_677103, EPI_ISL_677104, EPI_ISL_677105, EPI_ISL_677106, EPI_ISL_677107, EPI_ISL_677108, EPI_ISL_677109, EPI_ISL_677110			
see above	Wadsworth Center, New York State Department.of Health	Wadsworth Center, New York State Department.of Health	Kirsten St. George, Daryl M. Lamson, Alexis Russel, Jonathan Plitnick, Navjot Singh, John Kelly, Sara Griesemer, Erasmus Schneider, Erica Lasek-Nesselquist
EPI_ISL_677111, EPI_ISL_677112, EPI_ISL_677113, EPI_ISL_677114, EPI_ISL_677115, EPI_ISL_677116, EPI_ISL_677117, EPI_ISL_677118, EPI_ISL_677119, EPI_ISL_677120, EPI_ISL_677121, EPI_ISL_677122, EPI_ISL_677123, EPI_ISL_677124, EPI_ISL_677125, EPI_ISL_677126, EPI_ISL_677127, EPI_ISL_677128, EPI_ISL_677129			
see above	Masonic Medical Research Institute	Wadsworth Center, New York State Department.of Health	Nathan Tucker, Kirsten St. George, Daryl M. Lamson, Alexis Russel, Jonathan Plitnick, Navjot Singh, John Kelly, Sara Griesemer, Erasmus Schneider, Erica Lasek-Nesselquist
EPI_ISL_677130, EPI_ISL_677131, EPI_ISL_677132, EPI_ISL_677133	Wadsworth Center, New York State Department.of Health	Wadsworth Center, New York State Department.of Health	Kirsten St. George, Daryl M. Lamson, Alexis Russel, Jonathan Plitnick, Navjot Singh, John Kelly, Sara Griesemer, Erasmus Schneider, Erica Lasek-Nesselquist
EPI_ISL_677134, EPI_ISL_677135, EPI_ISL_677136, EPI_ISL_677137, EPI_ISL_677138, EPI_ISL_677139, EPI_ISL_677140, EPI_ISL_677141, EPI_ISL_677142, EPI_ISL_677143, EPI_ISL_677144, EPI_ISL_677145, EPI_ISL_677146, EPI_ISL_677147, EPI_ISL_677148, EPI_ISL_677149, EPI_ISL_677150, EPI_ISL_677151, EPI_ISL_677152, EPI_ISL_677153, EPI_ISL_677154, EPI_ISL_677155, EPI_ISL_677156, EPI_ISL_677157, EPI_ISL_677158, EPI_ISL_677159, EPI_ISL_677160, EPI_ISL_677161, EPI_ISL_677162, EPI_ISL_677163, EPI_ISL_677164, EPI_ISL_677165, EPI_ISL_677166, EPI_ISL_677167, EPI_ISL_677168, EPI_ISL_677169, EPI_ISL_677170, EPI_ISL_677171, EPI_ISL_677172, EPI_ISL_677173, EPI_ISL_677174, EPI_ISL_677175, EPI_ISL_677176, EPI_ISL_677177, EPI_ISL_677178, EPI_ISL_677179, EPI_ISL_677180, EPI_ISL_677181, EPI_ISL_677182, EPI_ISL_677183, EPI_ISL_677184, EPI_ISL_677185, EPI_ISL_677186, EPI_ISL_677187, EPI_ISL_677188, EPI_ISL_677189, EPI_ISL_677190, EPI_ISL_677191, EPI_ISL_677192, EPI_ISL_677193, EPI_ISL_677194, EPI_ISL_677195, EPI_ISL_677196, EPI_ISL_677197, EPI_ISL_677198, EPI_ISL_677199, EPI_ISL_677200, EPI_ISL_677201, EPI_ISL_677202, EPI_ISL_677203, EPI_ISL_677204, EPI_ISL_677205, EPI_ISL_677206, EPI_ISL_677207, EPI_ISL_677208, EPI_ISL_677209, EPI_ISL_677210, EPI_ISL_677211, EPI_ISL_677212, EPI_ISL_677213, EPI_ISL_677214, EPI_ISL_677215, EPI_ISL_677216, EPI_ISL_677217, EPI_ISL_677218, EPI_ISL_677219, EPI_ISL_677220, EPI_ISL_677221, EPI_ISL_677222, EPI_ISL_677223, EPI_ISL_677224, EPI_ISL_677225, EPI_ISL_677226, EPI_ISL_677227, EPI_ISL_677228, EPI_ISL_677229, EPI_ISL_677230, EPI_ISL_677231, EPI_ISL_677232, EPI_ISL_677233, EPI_ISL_677234, EPI_ISL_677235, EPI_ISL_677236, EPI_ISL_677237, EPI_ISL_677238, EPI_ISL_677239, EPI_ISL_677240			

see above	Virginia Division of Consolidated Laboratory Services	Virginia Division of Consolidated Laboratory Services	Virginia DCLS
EPI_ISL_677243, EPI_ISL_677244, EPI_ISL_677245, EPI_ISL_677247, EPI_ISL_677248, EPI_ISL_677249, EPI_ISL_677250, EPI_ISL_677251, EPI_ISL_677256, EPI_ISL_677257, EPI_ISL_677262, EPI_ISL_677263, EPI_ISL_677265, EPI_ISL_677285, EPI_ISL_677293, EPI_ISL_677294, EPI_ISL_677295, EPI_ISL_677296, EPI_ISL_677297, EPI_ISL_677298, EPI_ISL_677299, EPI_ISL_677300, EPI_ISL_677301, EPI_ISL_677302, EPI_ISL_677303, EPI_ISL_677304, EPI_ISL_677305, EPI_ISL_677306, EPI_ISL_677307, EPI_ISL_677308, EPI_ISL_677309, EPI_ISL_677310, EPI_ISL_677311, EPI_ISL_677312, EPI_ISL_677313, EPI_ISL_677314, EPI_ISL_677315, EPI_ISL_677316, EPI_ISL_677317, EPI_ISL_677318, EPI_ISL_677319			
see above	Colorado Department of Public Health and Environment	Colorado Department of Puplic Health and Environment	Laura Bankers, Molly Hetherington-Rauth, Shannon Ely, Shannon R. Matzinger, Sarah Elizabeth Totten, Emily A. Travanty
EPI_ISL_677320, EPI_ISL_677321, EPI_ISL_677322, EPI_ISL_677323, EPI_ISL_677324, EPI_ISL_677325, EPI_ISL_677326, EPI_ISL_677327, EPI_ISL_677328, EPI_ISL_677329, EPI_ISL_677330, EPI_ISL_677331, EPI_ISL_677332, EPI_ISL_677333, EPI_ISL_677334, EPI_ISL_677335, EPI_ISL_677336, EPI_ISL_677338, EPI_ISL_677339, EPI_ISL_677340, EPI_ISL_677341, EPI_ISL_677342, EPI_ISL_677343, EPI_ISL_677344, EPI_ISL_677345, EPI_ISL_677346, EPI_ISL_677347, EPI_ISL_677348, EPI_ISL_677349, EPI_ISL_677350, EPI_ISL_677351, EPI_ISL_677352, EPI_ISL_677353, EPI_ISL_677354, EPI_ISL_677355, EPI_ISL_677356, EPI_ISL_677357, EPI_ISL_677358, EPI_ISL_677359, EPI_ISL_677360, EPI_ISL_677361, EPI_ISL_677362, EPI_ISL_677363, EPI_ISL_677364, EPI_ISL_677365, EPI_ISL_677366, EPI_ISL_677367, EPI_ISL_677368, EPI_ISL_677369, EPI_ISL_677370, EPI_ISL_677371, EPI_ISL_677372, EPI_ISL_677373, EPI_ISL_677374, EPI_ISL_677375, EPI_ISL_677376, EPI_ISL_677377, EPI_ISL_677378, EPI_ISL_677379, EPI_ISL_677380, EPI_ISL_677381, EPI_ISL_677382, EPI_ISL_677383, EPI_ISL_677384, EPI_ISL_677385, EPI_ISL_677387, EPI_ISL_677388, EPI_ISL_677389, EPI_ISL_677390, EPI_ISL_677391, EPI_ISL_677392, EPI_ISL_677393, EPI_ISL_677394, EPI_ISL_677395, EPI_ISL_677396, EPI_ISL_677397, EPI_ISL_677398, EPI_ISL_677399, EPI_ISL_677400, EPI_ISL_677401, EPI_ISL_677402, EPI_ISL_677403, EPI_ISL_677404, EPI_ISL_677405, EPI_ISL_677406, EPI_ISL_677407, EPI_ISL_677408, EPI_ISL_677409, EPI_ISL_677410, EPI_ISL_677411, EPI_ISL_677412, EPI_ISL_677413, EPI_ISL_677414, EPI_ISL_677415, EPI_ISL_677416, EPI_ISL_677417, EPI_ISL_677418, EPI_ISL_677419, EPI_ISL_677420, EPI_ISL_677421, EPI_ISL_677422, EPI_ISL_677423, EPI_ISL_677424, EPI_ISL_677425, EPI_ISL_677426, EPI_ISL_677427, EPI_ISL_677428, EPI_ISL_677429, EPI_ISL_677430, EPI_ISL_677431, EPI_ISL_677432, EPI_ISL_677433, EPI_ISL_677434, EPI_ISL_677435, EPI_ISL_677436, EPI_ISL_677437, EPI_ISL_677438, EPI_ISL_677439, EPI_ISL_677440, EPI_ISL_677441, EPI_ISL_677442, EPI_ISL_677443, EPI_ISL_677444, EPI_ISL_677445, EPI_ISL_677446, EPI_ISL_677447, EPI_ISL_677448, EPI_ISL_677449, EPI_ISL_677450, EPI_ISL_677451, EPI_ISL_677452, EPI_ISL_677453, EPI_ISL_677454, EPI_ISL_677455, EPI_ISL_677456, EPI_ISL_677457, EPI_ISL_677458, EPI_ISL_677459, EPI_ISL_677460, EPI_ISL_677461, EPI_ISL_677462, EPI_ISL_677463, EPI_ISL_677464, EPI_ISL_677465, EPI_ISL_677466, EPI_ISL_677467, EPI_ISL_677468, EPI_ISL_677469, EPI_ISL_677470, EPI_ISL_677471, EPI_ISL_677472, EPI_ISL_677473, EPI_ISL_677474, EPI_ISL_677475, EPI_ISL_677476, EPI_ISL_677477, EPI_ISL_677478, EPI_ISL_677479, EPI_ISL_677480, EPI_ISL_677481, EPI_ISL_677482, EPI_ISL_677483, EPI_ISL_677484, EPI_ISL_677485, EPI_ISL_677486, EPI_ISL_677487, EPI_ISL_677488, EPI_ISL_677489, EPI_ISL_677490, EPI_ISL_677491, EPI_ISL_677492, EPI_ISL_677493, EPI_ISL_677494, EPI_ISL_677495, EPI_ISL_677496, EPI_ISL_677497, EPI_ISL_677498, EPI_ISL_677499, EPI_ISL_677500, EPI_ISL_677501, EPI_ISL_677502, EPI_ISL_677503, EPI_ISL_677504, EPI_ISL_677505, EPI_ISL_677506, EPI_ISL_677507, EPI_ISL_677508, EPI_ISL_677509, EPI_ISL_677510, EPI_ISL_677511, EPI_ISL_677512, EPI_ISL_677513, EPI_ISL_677514, EPI_ISL_677515, EPI_ISL_677516, EPI_ISL_677517, EPI_ISL_677518, EPI_ISL_677519, EPI_ISL_677520, EPI_ISL_677521, EPI_ISL_677522, EPI_ISL_677523, EPI_ISL_677524, EPI_ISL_677525, EPI_ISL_677526, EPI_ISL_677527, EPI_ISL_677528, EPI_ISL_677529, EPI_ISL_677530, EPI_ISL_677531, EPI_ISL_677532, EPI_ISL_677533, EPI_ISL_677534, EPI_ISL_677535, EPI_ISL_677536, EPI_ISL_677537, EPI_ISL_677538, EPI_ISL_677539, EPI_ISL_677540, EPI_ISL_677542, EPI_ISL_677543, EPI_ISL_677544, EPI_ISL_677545, EPI_ISL_677546, EPI_ISL_677547, EPI_ISL_677548, EPI_ISL_677549, EPI_ISL_677550, EPI_ISL_677551, EPI_ISL_677552, EPI_ISL_677554, EPI_ISL_677555, EPI_ISL_677556, EPI_ISL_677557, EPI_ISL_677558, EPI_ISL_677559, EPI_ISL_677560, EPI_ISL_677561, EPI_ISL_677562, EPI_ISL_677564, EPI_ISL_677565, EPI_ISL_677566, EPI_ISL_677567, EPI_ISL_677568, EPI_ISL_677570, EPI_ISL_677571, EPI_ISL_677572, EPI_ISL_677573, EPI_ISL_677574, EPI_ISL_677576, EPI_ISL_677577, EPI_ISL_677578, EPI_ISL_677579, EPI_ISL_677580, EPI_ISL_677581, EPI_ISL_677582, EPI_ISL_677583, EPI_ISL_677585, EPI_ISL_677586, EPI_ISL_677587, EPI_ISL_677588, EPI_ISL_677589, EPI_ISL_677591, EPI_ISL_677592, EPI_ISL_677593, EPI_ISL_677594, EPI_ISL_677595, EPI_ISL_677596, EPI_ISL_677597, EPI_ISL_677598, EPI_ISL_677599, EPI_ISL_677600, EPI_ISL_677601, EPI_ISL_677602, EPI_ISL_677603, EPI_ISL_677604, EPI_ISL_677605, EPI_ISL_677606, EPI_ISL_677607, EPI_ISL_677608, EPI_ISL_677609, EPI_ISL_677610, EPI_ISL_677611, EPI_ISL_677612, EPI_ISL_677613, EPI_ISL_677614, EPI_ISL_677615, EPI_ISL_677616, EPI_ISL_677617, EPI_ISL_677618, EPI_ISL_677619, EPI_ISL_677620, EPI_ISL_677621, EPI_ISL_677622, EPI_ISL_677623, EPI_ISL_677624, EPI_ISL_677625, EPI_ISL_677626, EPI_ISL_677627, EPI_ISL_677628, EPI_ISL_677629, EPI_ISL_677630, EPI_ISL_677631, EPI_ISL_677632, EPI_ISL_677633			
see above	University of Wisconsin-Madison AIDS Vaccine Research Laboratories	University of Wisconsin-Madison AIDS Vaccine Research Laboratories	Gage Moreno, Katarina Braun, et al. AIDS Vaccine Research Laboratories
EPI_ISL_677634	Virology Unit, Institut Pasteur de Madagascar	Virology Unit, Institut Pasteur de Madagascar	Christian Ranaivoson, Cara E. Brook, Vida Ahyong, Soa Fy Andriamandimby, Vololoniaina Raharinosy, Tsiry Randriambolamanantsoa, Helisoa Razafimanjato, Norosoa Razanajatovo, Michelle Tan, Cristina M. Tato, Joseph L. DeRisi, Jean-Michel Heraud, Philippe Dussart
EPI_ISL_677638, EPI_ISL_677639, EPI_ISL_677640, EPI_ISL_677641, EPI_ISL_677642, EPI_ISL_677643, EPI_ISL_677644, EPI_ISL_677645, EPI_ISL_677646, EPI_ISL_677647, EPI_ISL_677648, EPI_ISL_677649, EPI_ISL_677650, EPI_ISL_677652, EPI_ISL_677653, EPI_ISL_677654, EPI_ISL_677655, EPI_ISL_677656, EPI_ISL_677657			
see above	Colorado Department of Public Health and Environment	Colorado Department of Puplic Health and Environment	Laura Bankers, Molly C. Hetherington-Rauth, Shannon Ely, Shannon R. Matzinger, Sarah Elizabeth Totten, Emily A. Travanty
EPI_ISL_677658, EPI_ISL_677659, EPI_ISL_677660, EPI_ISL_677661, EPI_ISL_677662, EPI_ISL_677663	University of Wisconsin-Madison AIDS Vaccine Research Laboratories	University of Wisconsin-Madison AIDS Vaccine Research Laboratories	Gage Moreno, Katarina Braun, et al. AIDS Vaccine Research Laboratories
EPI_ISL_677668	Wadsworth Center, New York State Department.of Health	Wadsworth Center, New York State Department.of Health	Kirsten St. George, Daryl M. Lamson, Alexis Russel, Jonathan Plitnick, Navjot Singh, John Kelly, Sara Griesemer, Erasmus Schneider, Erica Lasek-Nesselquist
EPI_ISL_677669	Masonic Medical Research Institute	Wadsworth Center, New York State Department.of Health	Nathan Tucker, Kirsten St. George, Daryl M. Lamson, Alexis Russel, Jonathan Plitnick, Navjot Singh, John Kelly, Sara Griesemer, Erasmus Schneider, Erica Lasek-Nesselquist
EPI_ISL_677670, EPI_ISL_677671	Wadsworth Center, New York State Department.of Health	Wadsworth Center, New York State Department.of Health	Kirsten St. George, Daryl M. Lamson, Alexis Russel, Jonathan Plitnick, Navjot Singh, John Kelly, Sara Griesemer, Erasmus Schneider, Erica Lasek-Nesselquist
EPI_ISL_677675	General Hospital - Veles	Research Center for Genetic Engineering and Biotechnology "Georgi D. Efremov" , Macedonian Academy of Sciences and Arts	RCGEB - MASA
EPI_ISL_677676, EPI_ISL_677677	Clinical Hospital - Shtip	Research Center for Genetic Engineering and Biotechnology "Georgi D. Efremov" , Macedonian Academy of Sciences and Arts	RCGEB - MASA
EPI_ISL_677695, EPI_ISL_677696, EPI_ISL_677699	Vanda Pharmaceuticals Clinical Site	Vanda Pharmaceuticals	Vanda Pharmaceuticals
EPI_ISL_677700, EPI_ISL_677701, EPI_ISL_677702	Center for public health - Skopje	Research Center for Genetic Engineering and Biotechnology "Georgi D. Efremov" , Macedonian Academy of Sciences and Arts	RCGEB - MASA
EPI_ISL_677703	General Hospital - Prilep	Research Center for Genetic Engineering and Biotechnology "Georgi D. Efremov" , Macedonian Academy of Sciences and Arts	RCGEB - MASA
EPI_ISL_677704	General Hospital - Ohrid	Research Center for Genetic Engineering and Biotechnology "Georgi D. Efremov" , Macedonian Academy of Sciences and Arts	RCGEB - MASA
EPI_ISL_677705	General Hospital - Kumanovo	Research Center for Genetic Engineering and Biotechnology "Georgi D. Efremov" , Macedonian Academy of Sciences and Arts	RCGEB - MASA
EPI_ISL_677706	Center for public health - Skopje	Research Center for Genetic Engineering and Biotechnology "Georgi D. Efremov" , Macedonian Academy of Sciences and Arts	RCGEB - MASA
EPI_ISL_677707	General Hospital - Kumanovo	Research Center for Genetic Engineering and Biotechnology "Georgi D. Efremov" , Macedonian Academy of Sciences and Arts	RCGEB - MASA
EPI_ISL_677708	Center for public health - Skopje	Research Center for Genetic Engineering and Biotechnology "Georgi D. Efremov" , Macedonian Academy of Sciences and Arts	RCGEB - MASA
EPI_ISL_677709	General Hospital - Ohrid	Research Center for Genetic Engineering and Biotechnology "Georgi D. Efremov" , Macedonian Academy of Sciences and Arts	RCGEB - MASA
EPI_ISL_677710	General Hospital - Veles	Research Center for Genetic Engineering and Biotechnology "Georgi D. Efremov" , Macedonian Academy of Sciences and Arts	RCGEB - MASA
EPI_ISL_677711	General Hospital - Ohrid	Research Center for Genetic Engineering and Biotechnology "Georgi D. Efremov" , Macedonian Academy of Sciences and	RCGEB - MASA

		Arts	
EPI_ISL_677712, EPI_ISL_677713, EPI_ISL_677714	General Hospital - Prilep	Research Center for Genetic Engineering and Biotechnology "Georgi D. Efremov" , Macedonian Academy of Sciences and Arts	RCGEB - MASA
EPI_ISL_677715	Clinical Hospital - Bitola	Research Center for Genetic Engineering and Biotechnology "Georgi D. Efremov" , Macedonian Academy of Sciences and Arts	RCGEB - MASA
EPI_ISL_677716, EPI_ISL_677717	Clinical Hospital - Shtip	Research Center for Genetic Engineering and Biotechnology "Georgi D. Efremov" , Macedonian Academy of Sciences and Arts	RCGEB - MASA
EPI_ISL_677718	Clinical Hospital - Bitola	Research Center for Genetic Engineering and Biotechnology "Georgi D. Efremov" , Macedonian Academy of Sciences and Arts	RCGEB - MASA
EPI_ISL_677719	General Hospital - Struga	Research Center for Genetic Engineering and Biotechnology "Georgi D. Efremov" , Macedonian Academy of Sciences and Arts	RCGEB - MASA
EPI_ISL_677720, EPI_ISL_677721	Clinical Hospital - Shtip	Research Center for Genetic Engineering and Biotechnology "Georgi D. Efremov" , Macedonian Academy of Sciences and Arts	RCGEB - MASA
EPI_ISL_677722	General Hospital - Ohrid	Research Center for Genetic Engineering and Biotechnology "Georgi D. Efremov" , Macedonian Academy of Sciences and Arts	RCGEB - MASA
EPI_ISL_677723	Institute for Lung Diseases in Children - Skopje	Research Center for Genetic Engineering and Biotechnology "Georgi D. Efremov" , Macedonian Academy of Sciences and Arts	RCGEB - MASA
EPI_ISL_677724	Clinical Hospital - Shtip	Research Center for Genetic Engineering and Biotechnology "Georgi D. Efremov" , Macedonian Academy of Sciences and Arts	RCGEB - MASA
EPI_ISL_677725, EPI_ISL_677726	General Hospital - Ohrid	Research Center for Genetic Engineering and Biotechnology "Georgi D. Efremov" , Macedonian Academy of Sciences and Arts	RCGEB - MASA
EPI_ISL_677727, EPI_ISL_677728, EPI_ISL_677729, EPI_ISL_677730, EPI_ISL_677731, EPI_ISL_677732, EPI_ISL_677733, EPI_ISL_677734, EPI_ISL_677735, EPI_ISL_677736, EPI_ISL_677737, EPI_ISL_677738, EPI_ISL_677739, EPI_ISL_677740, EPI_ISL_677741, EPI_ISL_677742, EPI_ISL_677743, EPI_ISL_677744, EPI_ISL_677745, EPI_ISL_677746, EPI_ISL_677747, EPI_ISL_677748, EPI_ISL_677749, EPI_ISL_677750, EPI_ISL_677751, EPI_ISL_677752, EPI_ISL_677753, EPI_ISL_677754, EPI_ISL_677755, EPI_ISL_677756, EPI_ISL_677757, EPI_ISL_677758, EPI_ISL_677759, EPI_ISL_677760, EPI_ISL_677761, EPI_ISL_677762, EPI_ISL_677763, EPI_ISL_677764, EPI_ISL_677765, EPI_ISL_677766, EPI_ISL_677767, EPI_ISL_677768, EPI_ISL_677769, EPI_ISL_677770, EPI_ISL_677771, EPI_ISL_677772, EPI_ISL_677773, EPI_ISL_677774, EPI_ISL_677775, EPI_ISL_677776, EPI_ISL_677777, EPI_ISL_677778, EPI_ISL_677779, EPI_ISL_677780, EPI_ISL_677781, EPI_ISL_677782, EPI_ISL_677783, EPI_ISL_677784, EPI_ISL_677785, EPI_ISL_677786, EPI_ISL_677787, EPI_ISL_677788, EPI_ISL_677789, EPI_ISL_677790, EPI_ISL_677791, EPI_ISL_677792, EPI_ISL_677793, EPI_ISL_677794, EPI_ISL_677795, EPI_ISL_677796, EPI_ISL_677797, EPI_ISL_677798, EPI_ISL_677799, EPI_ISL_677800, EPI_ISL_677801, EPI_ISL_677802, EPI_ISL_677803, EPI_ISL_677804, EPI_ISL_677805, EPI_ISL_677806, EPI_ISL_677807, EPI_ISL_677808, EPI_ISL_677809, EPI_ISL_677810, EPI_ISL_677811, EPI_ISL_677812, EPI_ISL_677813, EPI_ISL_677814, EPI_ISL_677815, EPI_ISL_677816, EPI_ISL_677817, EPI_ISL_677818, EPI_ISL_677819, EPI_ISL_677820			
see above	University of Szeged, Institute of Clinical Microbiology	National Laboratory of Virology, Szentágothai Research Centre	Endre Gábor Tóth, Balázs Somogyi, Brigitta, Gabriella Terhes, Ferenc Jakab, Gábor Kemenesi
EPI_ISL_677821, EPI_ISL_677823, EPI_ISL_677824, EPI_ISL_677825, EPI_ISL_677826, EPI_ISL_677827, EPI_ISL_677828, EPI_ISL_677829, EPI_ISL_677830, EPI_ISL_677831, EPI_ISL_677832, EPI_ISL_677833, EPI_ISL_677834, EPI_ISL_677835, EPI_ISL_677836, EPI_ISL_677837, EPI_ISL_677838, EPI_ISL_677839, EPI_ISL_677840, EPI_ISL_677841, EPI_ISL_677842, EPI_ISL_677843, EPI_ISL_677844, EPI_ISL_677845, EPI_ISL_677846, EPI_ISL_677847, EPI_ISL_677848, EPI_ISL_677849, EPI_ISL_677850, EPI_ISL_677851, EPI_ISL_677852, EPI_ISL_677853, EPI_ISL_677854, EPI_ISL_677855, EPI_ISL_677856, EPI_ISL_677857, EPI_ISL_677858, EPI_ISL_677859, EPI_ISL_677860, EPI_ISL_677861, EPI_ISL_677862, EPI_ISL_677863, EPI_ISL_677864, EPI_ISL_677865, EPI_ISL_677866, EPI_ISL_677867, EPI_ISL_677868, EPI_ISL_677869, EPI_ISL_677870, EPI_ISL_677871, EPI_ISL_677872, EPI_ISL_677873, EPI_ISL_677874, EPI_ISL_677875, EPI_ISL_677876, EPI_ISL_677877, EPI_ISL_677878, EPI_ISL_677879, EPI_ISL_677880, EPI_ISL_677881, EPI_ISL_677882, EPI_ISL_677883, EPI_ISL_677884, EPI_ISL_677885, EPI_ISL_677886, EPI_ISL_677887, EPI_ISL_677889, EPI_ISL_677890, EPI_ISL_677891, EPI_ISL_677892, EPI_ISL_677893, EPI_ISL_677894, EPI_ISL_677896, EPI_ISL_677897, EPI_ISL_677898, EPI_ISL_677899, EPI_ISL_677900, EPI_ISL_677901, EPI_ISL_677904, EPI_ISL_677905, EPI_ISL_677906, EPI_ISL_677907			
see above	Innovative Genomics Institute, UC Berkeley	Innovative Genomics Institute, UC Berkeley	Stacia Wyman, Haridha Shivram, Phil Frankino, Liana Lareau, Shana McDevitt, Justin Choi
EPI_ISL_677908, EPI_ISL_677909	Pathogen Genomics Lab King Abdullah University of Science and Technology(KAUST)	Pathogen Genomics Lab King Abdullah University of Science and Technology(KAUST)	Sara Mfarrej, Raeece Naeem, Raushan Nugmanova, Olga Douvropoulou, Luke Esau, Amanda Ooi, Sharif Hala, Afrah Alsomali, Asim Khogeer, Fadwa Alofi,Jumana Taha, Abdulaziz Alahmadi, Kahled Algithami, Anwar Hashem, Naif Almontashiri, Arnab Pain
EPI_ISL_677910	Pathogen Genomics Lab King Abdullah University of Science and Technology(KAUST)	Pathogen Genomics Lab King Abdullah University of Science and Technology(KAUST)	Sara Mfarrej, Sharif Hala, Olga Douvropoulou, Raushan Nugmanova, Raeece Naeem, Afrah Alsomali, Asim Khogeer, Fadwa Alofi,Jumana Taha, Abdulaziz Alahmadi, Kahled Algithami, Anwar Hashem, Naif Almontashiri, Arnab Pain
EPI_ISL_677911	Pathogen Genomics Lab King Abdullah University of Science and Technology(KAUST)	Pathogen Genomics Lab King Abdullah University of Science and Technology(KAUST)	Muhammad Shuaib, Raeece Naeem, Sara Mfarrej, Raushan Nugmanova, Olga Douvropoulou, Luke Esau, Amanda Ooi, Sharif Hala, Afrah Alsomali, Asim Khogeer, Fadwa Alofi,Jumana Taha, Abdulaziz Alahmadi, Kahled Algithami, Anwar Hashem, Naif Almontashiri, Arnab Pain
EPI_ISL_677912	Pathogen Genomics Lab King Abdullah University of Science and Technology(KAUST)	Pathogen Genomics Lab King Abdullah University of Science and Technology(KAUST)	Sara Mfarrej, Raeece Naeem, Amanda Ooi, Luke Esau, Sharif Hala, Afrah Alsomali, Asim Khogeer, Fadwa Alofi,Jumana Taha, Abdulaziz Alahmadi, Kahled Algithami, Anwar Hashem, Naif Almontashiri, Arnab Pain
EPI_ISL_677913	Pathogen Genomics Lab King Abdullah University of Science and Technology(KAUST)	Pathogen Genomics Lab King Abdullah University of Science and Technology(KAUST)	Sara Mfarrej, Raeece Naeem, Raushan Nugmanova, Olga Douvropoulou, Luke Esau, Amanda Ooi, Sharif Hala, Afrah Alsomali, Asim Khogeer, Fadwa Alofi,Jumana Taha, Abdulaziz Alahmadi, Kahled Algithami, Anwar Hashem, Naif Almontashiri, Arnab Pain
EPI_ISL_677914	Pathogen Genomics Lab King Abdullah University of Science and Technology(KAUST)	Pathogen Genomics Lab King Abdullah University of Science and Technology(KAUST)	Muhammad Shuaib, Raeece Naeem, Sara Mfarrej, Raushan Nugmanova, Olga Douvropoulou, Luke Esau, Amanda Ooi, Sharif Hala, Afrah Alsomali, Asim Khogeer, Fadwa Alofi,Jumana Taha, Abdulaziz Alahmadi, Kahled Algithami, Anwar Hashem, Naif Almontashiri, Arnab Pain
EPI_ISL_677915	Pathogen Genomics Lab King Abdullah University of Science and Technology(KAUST)	Pathogen Genomics Lab King Abdullah University of Science and Technology(KAUST)	Sara Mfarrej, Raeece Naeem, Amanda Ooi, Luke Esau, Sharif Hala, Afrah Alsomali, Asim Khogeer, Fadwa Alofi,Jumana Taha, Abdulaziz Alahmadi, Kahled Algithami, Anwar Hashem, Naif Almontashiri, Arnab Pain
EPI_ISL_677916	Pathogen Genomics Lab King Abdullah University of Science and Technology(KAUST)	Pathogen Genomics Lab King Abdullah University of Science and Technology(KAUST)	Sara Mfarrej, Sharif Hala, Olga Douvropoulou, Raushan Nugmanova, Raeece Naeem, Afrah Alsomali, Asim Khogeer, Fadwa Alofi,Jumana Taha, Abdulaziz Alahmadi, Kahled Algithami, Anwar Hashem, Naif Almontashiri, Arnab Pain
EPI_ISL_677917	Pathogen Genomics Lab King Abdullah University of Science and Technology(KAUST)	Pathogen Genomics Lab King Abdullah University of Science and Technology(KAUST)	Sara Mfarrej, Raeece Naeem, Amanda Ooi, Luke Esau, Sharif Hala, Afrah Alsomali, Asim Khogeer, Fadwa Alofi,Jumana Taha, Abdulaziz Alahmadi, Kahled Algithami, Anwar Hashem, Naif Almontashiri, Arnab Pain
EPI_ISL_677918, EPI_ISL_677919, EPI_ISL_677921	Pathogen Genomics Lab King Abdullah University of Science and Technology(KAUST)	Pathogen Genomics Lab King Abdullah University of Science and Technology(KAUST)	Sara Mfarrej, Sharif Hala, Olga Douvropoulou, Raushan Nugmanova, Raeece Naeem, Afrah Alsomali, Asim Khogeer, Fadwa Alofi,Jumana Taha, Abdulaziz Alahmadi, Kahled Algithami, Anwar Hashem, Naif Almontashiri, Arnab Pain
EPI_ISL_677922	Pathogen Genomics Lab King Abdullah University of Science and Technology(KAUST)	Pathogen Genomics Lab King Abdullah University of Science and Technology(KAUST)	Muhammad Shuaib, Raeece Naeem, Raushan Nugmanova, Olga Douvropoulou, Sharif Hala, Sara Mfarrej, Afrah Alsomali, Asim Khogeer, Fadwa Alofi,Jumana Taha, Abdulaziz Alahmadi, Kahled Algithami, Anwar Hashem, Naif Almontashiri, Arnab Pain
EPI_ISL_677923	Pathogen Genomics Lab King Abdullah University of Science and Technology(KAUST)	Pathogen Genomics Lab King Abdullah University of Science and Technology(KAUST)	Muhammad Shuaib, Sharif Hala, Sara Mfarrej, Luke Esau, Amanda Ooi, Raeece Naeem, Afrah Alsomali, Asim Khogeer, Fadwa Alofi,Jumana Taha, Abdulaziz Alahmadi, Kahled Algithami, Anwar Hashem, Naif Almontashiri, Arnab Pain
EPI_ISL_677924	Pathogen Genomics Lab King Abdullah University of Science and Technology(KAUST)	Pathogen Genomics Lab King Abdullah University of Science and Technology(KAUST)	Muhammad Shuaib, Raeece Naeem, Sara Mfarrej, Raushan Nugmanova, Olga Douvropoulou, Luke Esau, Amanda Ooi, Sharif Hala, Afrah Alsomali, Asim Khogeer, Fadwa Alofi,Jumana Taha, Abdulaziz Alahmadi, Kahled Algithami, Anwar Hashem, Naif Almontashiri, Arnab Pain
EPI_ISL_677925, EPI_ISL_677926	Pathogen Genomics Lab King Abdullah University of Science and Technology(KAUST)	Pathogen Genomics Lab King Abdullah University of Science and Technology(KAUST)	Muhammad Shuaib, Sharif Hala, Sara Mfarrej, Luke Esau, Amanda Ooi, Raeece Naeem, Afrah Alsomali, Asim Khogeer, Fadwa Alofi,Jumana Taha, Abdulaziz Alahmadi, Kahled Algithami, Anwar Hashem, Naif Almontashiri, Arnab Pain

EPI_ISL_677927	Pathogen Genomics Lab King Abdullah University of Science and Technology(KAUST)	Pathogen Genomics Lab King Abdullah University of Science and Technology(KAUST)	Muhammad Shuaib, Raeece Naeem, Raushan Nugmanova, Olga Douvropoulou, Sharif Hala, Sara Mfarrej, Afrah Alsomali, Asim Khogeer, Fadwa Alofi,Jumana Taha, Abdulaziz Alahmadi, Kahled Algithami, Anwar Hashem, Naif Almontashiri, Arnab Pain
EPI_ISL_677928	Pathogen Genomics Lab King Abdullah University of Science and Technology(KAUST)	Pathogen Genomics Lab King Abdullah University of Science and Technology(KAUST)	Sara Mfarrej, Sharif Hala, Olga Douvropoulou, Raushan Nugmanova, Raeece Naeem, Afrah Alsomali, Asim Khogeer, Fadwa Alofi,Jumana Taha, Abdulaziz Alahmadi, Kahled Algithami, Anwar Hashem, Naif Almontashiri, Arnab Pain
EPI_ISL_677929	Pathogen Genomics Lab King Abdullah University of Science and Technology(KAUST)	Pathogen Genomics Lab King Abdullah University of Science and Technology(KAUST)	Sara Mfarrej, Raeece Naeem, Amanda Ooi, Luke Esau, Sharif Hala, Afrah Alsomali, Asim Khogeer, Fadwa Alofi,Jumana Taha, Abdulaziz Alahmadi, Kahled Algithami, Anwar Hashem, Naif Almontashiri, Arnab Pain
EPI_ISL_677930, EPI_ISL_677931	Pathogen Genomics Lab King Abdullah University of Science and Technology(KAUST)	Pathogen Genomics Lab King Abdullah University of Science and Technology(KAUST)	Sara Mfarrej, Sharif Hala, Olga Douvropoulou, Raushan Nugmanova, Raeece Naeem, Afrah Alsomali, Asim Khogeer, Fadwa Alofi,Jumana Taha, Abdulaziz Alahmadi, Kahled Algithami, Anwar Hashem, Naif Almontashiri, Arnab Pain
EPI_ISL_677932, EPI_ISL_677933, EPI_ISL_677934	Pathogen Genomics Lab King Abdullah University of Science and Technology(KAUST)	Pathogen Genomics Lab King Abdullah University of Science and Technology(KAUST)	Sara Mfarrej, Raeece Naeem, Amanda Ooi, Luke Esau, Sharif Hala, Afrah Alsomali, Asim Khogeer, Fadwa Alofi,Jumana Taha, Abdulaziz Alahmadi, Kahled Algithami, Anwar Hashem, Naif Almontashiri, Arnab Pain
EPI_ISL_677935, EPI_ISL_677936	Pathogen Genomics Lab King Abdullah University of Science and Technology(KAUST)	Pathogen Genomics Lab King Abdullah University of Science and Technology(KAUST)	Sara Mfarrej, Sharif Hala, Olga Douvropoulou, Raushan Nugmanova, Raeece Naeem, Afrah Alsomali, Asim Khogeer, Fadwa Alofi,Jumana Taha, Abdulaziz Alahmadi, Kahled Algithami, Anwar Hashem, Naif Almontashiri, Arnab Pain
EPI_ISL_677948	Pathogen Genomics Lab King Abdullah University of Science and Technology(KAUST)	Pathogen Genomics Lab King Abdullah University of Science and Technology(KAUST)	Sara Mfarrej, Olga Douvropoulou, Raushan Nugmanova, Sharif Hala, Raeece Naeem, Amanda Ooi, Luke Esau, Fadwa Alofi, Afrah Alsomali, Asim Khogeer, Jumana Taha, Abdulaziz Alahmadi, Kahled Algithami, Anwar Hashem, Naif Almontashiri, Arnab Pain
EPI_ISL_677949	Pathogen Genomics Lab King Abdullah University of Science and Technology(KAUST)	Pathogen Genomics Lab King Abdullah University of Science and Technology(KAUST)	Muhammad Shuaib, Raeece Naeem, Sharif Hala, Sara Mfarrej, Olga Douvropoulou, Raushan Nugmanova, Luke Esau, Amanda Ooi, Fadwa Alofi, Afrah Alsomali, Asim Khogeer, Jumana Taha, Abdulaziz Alahmadi, Kahled Algithami, Anwar Hashem, Naif Almontashiri, Arnab Pain
EPI_ISL_677950	Pathogen Genomics Lab King Abdullah University of Science and Technology(KAUST)	Pathogen Genomics Lab King Abdullah University of Science and Technology(KAUST)	Sara Mfarrej, Sharif Hala, Luke Esau, Amanda Ooi, Raeece Naeem, Fadwa Alofi, Afrah Alsomali, Asim Khogeer, Jumana Taha, Abdulaziz Alahmadi, Kahled Algithami, Anwar Hashem, Naif Almontashiri, Arnab Pain
EPI_ISL_677951	Pathogen Genomics Lab King Abdullah University of Science and Technology(KAUST)	Pathogen Genomics Lab King Abdullah University of Science and Technology(KAUST)	Olga Douvropoulou, Sara Mfarrej, Raushan Nugmanova, Sharif Hala, Raeece Naeem, Amanda Ooi, Luke Esau, Fadwa Alofi, Afrah Alsomali, Asim Khogeer, Jumana Taha, Abdulaziz Alahmadi, Kahled Algithami, Anwar Hashem, Naif Almontashiri, Arnab Pain
EPI_ISL_677952	Pathogen Genomics Lab King Abdullah University of Science and Technology(KAUST)	Pathogen Genomics Lab King Abdullah University of Science and Technology(KAUST)	Sara Mfarrej, Raushan Nugmanova, Olga Douvropoulou, Raeece Naeem, Fadwa Alofi, Afrah Alsomali, Asim Khogeer, Jumana Taha, Abdulaziz Alahmadi, Kahled Algithami, Anwar Hashem, Naif Almontashiri, Sharif Hala, Arnab Pain
EPI_ISL_677953	Pathogen Genomics Lab King Abdullah University of Science and Technology(KAUST)	Pathogen Genomics Lab King Abdullah University of Science and Technology(KAUST)	Sara Mfarrej, Sharif Hala, Raeece Naeem, Amanda Ooi, Luke Esau, Fadwa Alofi, Afrah Alsomali, Asim Khogeer, Jumana Taha, Abdulaziz Alahmadi, Kahled Algithami, Anwar Hashem, Naif Almontashiri, Arnab Pain
EPI_ISL_677954	Pathogen Genomics Lab King Abdullah University of Science and Technology(KAUST)	Pathogen Genomics Lab King Abdullah University of Science and Technology(KAUST)	Muhammad Shuaib, Raeece Naeem, Amanda Ooi, Luke Esau, Sharif Hala, Sara Mfarrej, Fadwa Alofi, Afrah Alsomali, Asim Khogeer, Jumana Taha, Abdulaziz Alahmadi, Kahled Algithami, Anwar Hashem, Naif Almontashiri, Arnab Pain
EPI_ISL_677956, EPI_ISL_677957	Pathogen Genomics Lab King Abdullah University of Science and Technology(KAUST)	Pathogen Genomics Lab King Abdullah University of Science and Technology(KAUST)	Sara Mfarrej, Sharif Hala, Raeece Naeem, Amanda Ooi, Luke Esau, Fadwa Alofi, Afrah Alsomali, Asim Khogeer, Jumana Taha, Abdulaziz Alahmadi, Kahled Algithami, Anwar Hashem, Naif Almontashiri, Arnab Pain
EPI_ISL_677959, EPI_ISL_677961	Pathogen Genomics Lab King Abdullah University of Science and Technology(KAUST)	Pathogen Genomics Lab King Abdullah University of Science and Technology(KAUST)	Sara Mfarrej, Olga Douvropoulou, Raushan Nugmanova, Sharif Hala, Raeece Naeem, Amanda Ooi, Luke Esau, Fadwa Alofi, Afrah Alsomali, Asim Khogeer, Jumana Taha, Abdulaziz Alahmadi, Kahled Algithami, Anwar Hashem, Naif Almontashiri, Arnab Pain
EPI_ISL_677962	Pathogen Genomics Lab King Abdullah University of Science and Technology(KAUST)	Pathogen Genomics Lab King Abdullah University of Science and Technology(KAUST)	Sara Mfarrej, Sharif Hala, Raeece Naeem, Amanda Ooi, Luke Esau, Fadwa Alofi, Afrah Alsomali, Asim Khogeer, Jumana Taha, Abdulaziz Alahmadi, Kahled Algithami, Anwar Hashem, Naif Almontashiri, Arnab Pain
EPI_ISL_677967	Pathogen Genomics Lab King Abdullah University of Science and Technology(KAUST)	Pathogen Genomics Lab King Abdullah University of Science and Technology(KAUST)	Sara Mfarrej, Olga Douvropoulou, Raushan Nugmanova, Sharif Hala, Raeece Naeem, Amanda Ooi, Luke Esau, Fadwa Alofi, Afrah Alsomali, Asim Khogeer, Jumana Taha, Abdulaziz Alahmadi, Kahled Algithami, Anwar Hashem, Naif Almontashiri, Arnab Pain
EPI_ISL_677969	Pathogen Genomics Lab King Abdullah University of Science and Technology(KAUST)	Pathogen Genomics Lab King Abdullah University of Science and Technology(KAUST)	Sara Mfarrej, Sharif Hala, Raeece Naeem, Amanda Ooi, Luke Esau, Fadwa Alofi, Afrah Alsomali, Asim Khogeer, Jumana Taha, Abdulaziz Alahmadi, Kahled Algithami, Anwar Hashem, Naif Almontashiri, Arnab Pain
EPI_ISL_677972	Pathogen Genomics Lab King Abdullah University of Science and Technology(KAUST)	Pathogen Genomics Lab King Abdullah University of Science and Technology(KAUST)	Olga Douvropoulou, Sara Mfarrej, Raushan Nugmanova, Sharif Hala, Raeece Naeem, Amanda Ooi, Luke Esau, Fadwa Alofi, Afrah Alsomali, Asim Khogeer, Jumana Taha, Abdulaziz Alahmadi, Kahled Algithami, Anwar Hashem, Naif Almontashiri, Arnab Pain
EPI_ISL_677974	Pathogen Genomics Lab King Abdullah University of Science and Technology(KAUST)	Pathogen Genomics Lab King Abdullah University of Science and Technology(KAUST)	Raushan Nugmanova, Sara Mfarrej, Olga Douvropoulou, Sharif Hala, Raeece Naeem, Fadwa Alofi, Afrah Alsomali, Asim Khogeer, Jumana Taha, Abdulaziz Alahmadi, Kahled Algithami, Anwar Hashem, Naif Almontashiri, Arnab Pain
EPI_ISL_677975	Pathogen Genomics Lab King Abdullah University of Science and Technology(KAUST)	Pathogen Genomics Lab King Abdullah University of Science and Technology(KAUST)	Amit Kumar Subudhi, Sharif Hala, Raeece Naeem, Sara Mfarrej, Olga Douvropoulou, Raushan Nugmanova, Fadwa Alofi, Afrah Alsomali, Asim Khogeer, Jumana Taha, Abdulaziz Alahmadi, Kahled Algithami, Anwar Hashem, Naif Almontashiri, Arnab Pain
EPI_ISL_677977, EPI_ISL_677978, EPI_ISL_677979, EPI_ISL_677980	Pathogen Genomics Lab King Abdullah University of Science and Technology(KAUST)	Pathogen Genomics Lab King Abdullah University of Science and Technology(KAUST)	Amit Kumar Subudhi, Sharif Hala, Raeece Naeem, Sara Mfarrej, Amanda Ooi, Luke Esau, Fadwa Alofi, Afrah Alsomali, Asim Khogeer, Jumana Taha, Abdulaziz Alahmadi, Kahled Algithami, Anwar Hashem, Naif Almontashiri, Arnab Pain
EPI_ISL_677981, EPI_ISL_677982, EPI_ISL_677983, EPI_ISL_677984, EPI_ISL_677985	Pathogen Genomics Lab King Abdullah University of Science and Technology(KAUST)	Pathogen Genomics Lab King Abdullah University of Science and Technology(KAUST)	Amit Kumar Subudhi, Sharif Hala, Raeece Naeem, Sara Mfarrej, Olga Douvropoulou, Raushan Nugmanova, Fadwa Alofi, Afrah Alsomali, Asim Khogeer, Jumana Taha, Abdulaziz Alahmadi, Kahled Algithami, Anwar Hashem, Naif Almontashiri, Arnab Pain
EPI_ISL_677986, EPI_ISL_677987	Pathogen Genomics Lab King Abdullah University of Science and Technology(KAUST)	Pathogen Genomics Lab King Abdullah University of Science and Technology(KAUST)	Amit Kumar Subudhi, Sara Mfarrej, Amanda Ooi, Luke Esau, Sharif Hala, Raeece Naeem, Fadwa Alofi, Afrah Alsomali, Asim Khogeer, Jumana Taha, Abdulaziz Alahmadi, Kahled Algithami, Anwar Hashem, Naif Almontashiri, Arnab Pain
EPI_ISL_677988, EPI_ISL_677989	Pathogen Genomics Lab King Abdullah University of Science and Technology(KAUST)	Pathogen Genomics Lab King Abdullah University of Science and Technology(KAUST)	Sara Mfarrej, Olga Douvropoulou, Raushan Nugmanova, Sharif Hala, Raeece Naeem, Amanda Ooi, Luke Esau, Fadwa Alofi, Afrah Alsomali, Asim Khogeer, Jumana Taha, Abdulaziz Alahmadi, Kahled Algithami, Anwar Hashem, Naif Almontashiri, Arnab Pain
EPI_ISL_677990	Pathogen Genomics Lab King Abdullah University of Science and Technology(KAUST)	Pathogen Genomics Lab King Abdullah University of Science and Technology(KAUST)	Amit Kumar Subudhi, Sara Mfarrej, Amanda Ooi, Luke Esau, Sharif Hala, Raeece Naeem, Fadwa Alofi, Afrah Alsomali, Asim Khogeer, Jumana Taha, Abdulaziz Alahmadi, Kahled Algithami, Anwar Hashem, Naif Almontashiri, Arnab Pain
EPI_ISL_677991	Pathogen Genomics Lab King Abdullah University of Science and Technology(KAUST)	Pathogen Genomics Lab King Abdullah University of Science and Technology(KAUST)	Olga Douvropoulou, Sara Mfarrej, Raushan Nugmanova, Sharif Hala, Raeece Naeem, Amanda Ooi, Luke Esau, Fadwa Alofi, Afrah Alsomali, Asim Khogeer, Jumana Taha, Abdulaziz Alahmadi, Kahled Algithami, Anwar Hashem, Naif Almontashiri, Arnab Pain
EPI_ISL_677992, EPI_ISL_677993, EPI_ISL_677994, EPI_ISL_677995, EPI_ISL_677996, EPI_ISL_677997, EPI_ISL_677998, EPI_ISL_677999	Pathogen Genomics Lab King Abdullah University of Science and Technology(KAUST)	Pathogen Genomics Lab King Abdullah University of Science and Technology(KAUST)	Raeece Naeem, Sara Mfarrej, Sharif Hala, Olga Douvropoulou, Raushan Nugmanova, Fadwa Alofi, Asim Khogeer, Afrah Alsomali, Jumana Taha, Abdulaziz Alahmadi, Kahled Algithami, Anwar Hashem, Naif Almontashiri, Arnab Pain
EPI_ISL_678000, EPI_ISL_678001, EPI_ISL_678002, EPI_ISL_678003	Pathogen Genomics Lab King Abdullah University of Science and Technology(KAUST)	Pathogen Genomics Lab King Abdullah University of Science and Technology(KAUST)	Raeece Naeem, Sara Mfarrej, Amanda Ooi, Luke Esau, Sharif Hala, Fadwa Alofi, Afrah Alsomali, Asim Khogeer, Jumana Taha, Abdulaziz Alahmadi, Kahled Algithami, Anwar Hashem, Naif Almontashiri, Arnab Pain
EPI_ISL_678004, EPI_ISL_678005, EPI_ISL_678006, EPI_ISL_678007, EPI_ISL_678008, EPI_ISL_678009	Pathogen Genomics Lab King Abdullah University of Science and Technology(KAUST)	Pathogen Genomics Lab King Abdullah University of Science and Technology(KAUST)	Sara Mfarrej, Raushan Nugmanova, Olga Douvropoulou, Raeece Naeem, Fadwa Alofi, Afrah Alsomali, Asim Khogeer, Jumana Taha, Abdulaziz Alahmadi, Kahled Algithami, Anwar Hashem, Naif Almontashiri, Sharif Hala, Arnab Pain
EPI_ISL_678010	Pathogen Genomics Lab King Abdullah University of Science and Technology(KAUST)	Pathogen Genomics Lab King Abdullah University of Science and Technology(KAUST)	Muhammad Shuaib, Raeece Naeem, Amanda Ooi, Luke Esau, Sharif Hala, Sara Mfarrej, Fadwa Alofi, Afrah Alsomali, Asim Khogeer, Jumana Taha, Abdulaziz Alahmadi, Kahled Algithami, Anwar Hashem, Naif Almontashiri, Arnab Pain
EPI_ISL_678011, EPI_ISL_678012, EPI_ISL_678013, EPI_ISL_678014	Pathogen Genomics Lab King Abdullah University of Science and Technology(KAUST)	Pathogen Genomics Lab King Abdullah University of Science and Technology(KAUST)	Muhammad Shuaib, Raeece Naeem, Sharif Hala, Sara Mfarrej, Olga Douvropoulou, Raushan Nugmanova, Fadwa Alofi, Afrah Alsomali, Asim Khogeer, Jumana Taha, Abdulaziz Alahmadi, Kahled Algithami, Anwar Hashem, Naif Almontashiri, Arnab Pain
EPI_ISL_678015	Pathogen Genomics Lab King Abdullah University of Science and Technology(KAUST)	Pathogen Genomics Lab King Abdullah University of Science and Technology(KAUST)	Muhammad Shuaib, Amanda Ooi, Luke Esau, Sharif Hala, Raeece Naeem, Sara Mfarrej, Fadwa Alofi, Afrah Alsomali, Asim Khogeer, Jumana Taha, Abdulaziz Alahmadi, Kahled Algithami, Anwar Hashem, Naif Almontashiri, Arnab Pain
EPI_ISL_678016	Pathogen Genomics Lab King Abdullah University of Science	Pathogen Genomics Lab King Abdullah University of Science	Muhammad Shuaib, Raeece Naeem, Sharif Hala, Sara Mfarrej, Olga Douvropoulou, Raushan Nugmanova, Fadwa Alofi, Afrah Alsomali, Asim Khogeer,

	and Technology(KAUST)	and Technology(KAUST)	Jumana Taha, Abdulaziz Alahmadi, Kahled Algithami, Anwar Hashem, Naif Almontashiri, Arnab Pain
EPI_ISL_678017	Pathogen Genomics Lab King Abdullah University of Science and Technology(KAUST)	Pathogen Genomics Lab King Abdullah University of Science and Technology(KAUST)	Muhammad Shuaib, Raeece Naeem, Amanda Ooi, Luke Esau, Sharif Hala, Sara Mfarrej, Fadwa Alofi, Afrah Alsomali, Asim Khogeer, Jumana Taha, Abdulaziz Alahmadi, Kahled Algithami, Anwar Hashem, Naif Almontashiri, Arnab Pain
EPI_ISL_678018	Pathogen Genomics Lab King Abdullah University of Science and Technology(KAUST)	Pathogen Genomics Lab King Abdullah University of Science and Technology(KAUST)	Muhammad Shuaib, Amanda Ooi, Luke Esau, Sharif Hala, Raeece Naeem, Sara Mfarrej, Fadwa Alofi, Afrah Alsomali, Asim Khogeer, Jumana Taha, Abdulaziz Alahmadi, Kahled Algithami, Anwar Hashem, Naif Almontashiri, Arnab Pain
EPI_ISL_678019	Pathogen Genomics Lab King Abdullah University of Science and Technology(KAUST)	Pathogen Genomics Lab King Abdullah University of Science and Technology(KAUST)	Muhammad Shuaib, Raeece Naeem, Sharif Hala, Sara Mfarrej, Olga Douvropoulou, Raushan Nugmanova, Fadwa Alofi, Afrah Alsomali, Asim Khogeer, Jumana Taha, Abdulaziz Alahmadi, Kahled Algithami, Anwar Hashem, Naif Almontashiri, Arnab Pain
EPI_ISL_678020, EPI_ISL_678021	Pathogen Genomics Lab King Abdullah University of Science and Technology(KAUST)	Pathogen Genomics Lab King Abdullah University of Science and Technology(KAUST)	Raushan Nugmanova, Sara Mfarrej, Olga Douvropoulou, Sharif Hala, Raeece Naeem, Fadwa Alofi, Afrah Alsomali, Asim Khogeer, Jumana Taha, Abdulaziz Alahmadi, Kahled Algithami, Anwar Hashem, Naif Almontashiri, Arnab Pain
EPI_ISL_678022, EPI_ISL_678023	Pathogen Genomics Lab King Abdullah University of Science and Technology(KAUST)	Pathogen Genomics Lab King Abdullah University of Science and Technology(KAUST)	Sara Mfarrej, Sharif Hala, Raeece Naeem, Amanda Ooi, Luke Esau, Fadwa Alofi, Afrah Alsomali, Asim Khogeer, Jumana Taha, Abdulaziz Alahmadi, Kahled Algithami, Anwar Hashem, Naif Almontashiri, Arnab Pain
EPI_ISL_678024, EPI_ISL_678025	Pathogen Genomics Lab King Abdullah University of Science and Technology(KAUST)	Pathogen Genomics Lab King Abdullah University of Science and Technology(KAUST)	Raushan Nugmanova, Sara Mfarrej, Olga Douvropoulou, Sharif Hala, Raeece Naeem, Fadwa Alofi, Afrah Alsomali, Asim Khogeer, Jumana Taha, Abdulaziz Alahmadi, Kahled Algithami, Anwar Hashem, Naif Almontashiri, Arnab Pain
EPI_ISL_678049	Pathogen Genomics Lab King Abdullah University of Science and Technology(KAUST)	Pathogen Genomics Lab King Abdullah University of Science and Technology(KAUST)	Muhammad Shuaib, Sara Mfarrej, Raushan Nugmanova, Olga Douvropoulou, Raeece Naeem, Sharif Hala, Luke Esau, Amanda Ooi, Asim Khogeer, Fadwa Alofi, Afrah Alsomali,Jumana Taha, Abdulaziz Alahmadi, Kahled Algithami, Anwar Hashem, Naif Almontashiri, Arnab Pain
EPI_ISL_678064, EPI_ISL_678070, EPI_ISL_678083, EPI_ISL_678084, EPI_ISL_678085, EPI_ISL_678086, EPI_ISL_678087, EPI_ISL_678088	Pathogen Genomics Lab King Abdullah University of Science and Technology(KAUST)	Pathogen Genomics Lab King Abdullah University of Science and Technology(KAUST)	Muhammad Shuaib, Raeece Naeem, Sharif Hala, Sara Mfarrej, Olga Douvropoulou, Raushan Nugmanova, Asim Khogeer, Fadwa Alofi, Afrah Alsomali, Jumana Taha, Abdulaziz Alahmadi, Kahled Algithami, Anwar Hashem, Naif Almontashiri, Arnab Pain
EPI_ISL_678089	Pathogen Genomics Lab King Abdullah University of Science and Technology(KAUST)	Pathogen Genomics Lab King Abdullah University of Science and Technology(KAUST)	Muhammad Shuaib, Amanda Ooi, Luke Esau, Sharif Hala, Raeece Naeem, Sara Mfarrej, Asim Khogeer, Fadwa Alofi, Afrah Alsomali,Jumana Taha, Abdulaziz Alahmadi, Kahled Algithami, Anwar Hashem, Naif Almontashiri, Arnab Pain
EPI_ISL_678090, EPI_ISL_678091	Pathogen Genomics Lab King Abdullah University of Science and Technology(KAUST)	Pathogen Genomics Lab King Abdullah University of Science and Technology(KAUST)	Muhammad Shuaib, Raeece Naeem, Sharif Hala, Sara Mfarrej, Olga Douvropoulou, Raushan Nugmanova, Asim Khogeer, Fadwa Alofi, Afrah Alsomali, Jumana Taha, Abdulaziz Alahmadi, Kahled Algithami, Anwar Hashem, Naif Almontashiri, Arnab Pain
EPI_ISL_678092	Pathogen Genomics Lab King Abdullah University of Science and Technology(KAUST)	Pathogen Genomics Lab King Abdullah University of Science and Technology(KAUST)	Muhammad Shuaib, Raeece Naeem, Raushan Nugmanova, Olga Douvropoulou, Sara Mfarrej, Sharif Hala, Asim Khogeer, Fadwa Alofi, Afrah Alsomali, Jumana Taha, Abdulaziz Alahmadi, Kahled Algithami, Anwar Hashem, Naif Almontashiri, Arnab Pain
EPI_ISL_678093	Pathogen Genomics Lab King Abdullah University of Science and Technology(KAUST)	Pathogen Genomics Lab King Abdullah University of Science and Technology(KAUST)	Muhammad Shuaib, Sara Mfarrej, Raeece Naeem,Raushan Nugmanova, Olga Douvropoulou, Sharif Hala, Asim Khogeer, Fadwa Alofi, Afrah Alsomali, Jumana Taha, Abdulaziz Alahmadi, Kahled Algithami, Anwar Hashem, Naif Almontashiri, Arnab Pain
EPI_ISL_678094, EPI_ISL_678095	Pathogen Genomics Lab King Abdullah University of Science and Technology(KAUST)	Pathogen Genomics Lab King Abdullah University of Science and Technology(KAUST)	Olga Douvropoulou, Sara Mfarrej, Raushan Nugmanova, Sharif Hala, Raeece Naeem, Asim Khogeer, Fadwa Alofi, Afrah Alsomali, Jumana Taha, Abdulaziz Alahmadi, Kahled Algithami, Anwar Hashem, Naif Almontashiri, Arnab Pain
EPI_ISL_678147	Pathogen Genomics Lab King Abdullah University of Science and Technology(KAUST)	Pathogen Genomics Lab King Abdullah University of Science and Technology(KAUST)	Sara Mfarrej, Raushan Nugmanova, Olga Douvropoulou, Raeece Naeem, Sharif Hala, Luke Esau, Amanda Ooi, Awad Al-Omari, Samer Salih, Abbas Al Mutair, Arnab Pain
EPI_ISL_678148	Pathogen Genomics Lab King Abdullah University of Science and Technology(KAUST)	Pathogen Genomics Lab King Abdullah University of Science and Technology(KAUST)	Sara Mfarrej, Raeece Naeem, Amanda Ooi, Luke Esau, Sharif Hala, Awad Al-Omari, Samer Salih, Abbas Al Mutair, Arnab Pain
EPI_ISL_678149, EPI_ISL_678150	Pathogen Genomics Lab King Abdullah University of Science and Technology(KAUST)	Pathogen Genomics Lab King Abdullah University of Science and Technology(KAUST)	Sara Mfarrej, Luke Esau, Amanda Ooi, Sharif Hala, Raeece Naeem, Awad Al-Omari, Samer Salih, Abbas Al Mutair, Arnab Pain
EPI_ISL_678152, EPI_ISL_678153	Pathogen Genomics Lab King Abdullah University of Science and Technology(KAUST)	Pathogen Genomics Lab King Abdullah University of Science and Technology(KAUST)	Sara Mfarrej, Raushan Nugmanova, Olga Douvropoulou, Raeece Naeem, Sharif Hala, Luke Esau, Amanda Ooi, Awad Al-Omari, Samer Salih, Abbas Al Mutair, Arnab Pain
EPI_ISL_678154	Pathogen Genomics Lab King Abdullah University of Science and Technology(KAUST)	Pathogen Genomics Lab King Abdullah University of Science and Technology(KAUST)	Sara Mfarrej, Olga Douvropoulou, Raushan Nugmanova, Raeece Naeem, Sharif Hala, Awad Al-Omari, Samer Salih, Abbas Al Mutair, Arnab Pain
EPI_ISL_678155, EPI_ISL_678156	Pathogen Genomics Lab King Abdullah University of Science and Technology(KAUST)	Pathogen Genomics Lab King Abdullah University of Science and Technology(KAUST)	Sara Mfarrej, Raeece Naeem, Luke Esau, Amanda Ooi, Sharif Hala, Awad Al-Omari, Samer Salih, Abbas Al Mutair, Arnab Pain
EPI_ISL_678157, EPI_ISL_678158, EPI_ISL_678159	Pathogen Genomics Lab King Abdullah University of Science and Technology(KAUST)	Pathogen Genomics Lab King Abdullah University of Science and Technology(KAUST)	Sara Mfarrej, Luke Esau, Amanda Ooi, Sharif Hala, Raeece Naeem, Awad Al-Omari, Samer Salih, Abbas Al Mutair, Arnab Pain
EPI_ISL_678160	Pathogen Genomics Lab King Abdullah University of Science and Technology(KAUST)	Pathogen Genomics Lab King Abdullah University of Science and Technology(KAUST)	Raeece Naeem, Sara Mfarrej, Amanda Ooi, Luke Esau, Sharif Hala, Awad Al-Omari, Samer Salih, Abbas Al Mutair, Arnab Pain
EPI_ISL_678161	Pathogen Genomics Lab King Abdullah University of Science and Technology(KAUST)	Pathogen Genomics Lab King Abdullah University of Science and Technology(KAUST)	Raeece Naeem, Sara Mfarrej, Luke Esau, Amanda Ooi, Sharif Hala, Awad Al-Omari, Samer Salih, Abbas Al Mutair, Arnab Pain
EPI_ISL_678162	Pathogen Genomics Lab King Abdullah University of Science and Technology(KAUST)	Pathogen Genomics Lab King Abdullah University of Science and Technology(KAUST)	Sara Mfarrej, Raushan Nugmanova, Olga Douvropoulou, Raeece Naeem, Sharif Hala, Luke Esau, Amanda Ooi, Awad Al-Omari, Samer Salih, Abbas Al Mutair, Arnab Pain
EPI_ISL_678163	Pathogen Genomics Lab King Abdullah University of Science and Technology(KAUST)	Pathogen Genomics Lab King Abdullah University of Science and Technology(KAUST)	Sara Mfarrej, Luke Esau, Amanda Ooi, Sharif Hala, Raeece Naeem, Awad Al-Omari, Samer Salih, Abbas Al Mutair, Arnab Pain
EPI_ISL_678164, EPI_ISL_678165	Pathogen Genomics Lab King Abdullah University of Science and Technology(KAUST)	Pathogen Genomics Lab King Abdullah University of Science and Technology(KAUST)	Sara Mfarrej, Raushan Nugmanova, Olga Douvropoulou, Raeece Naeem, Sharif Hala, Luke Esau, Amanda Ooi, Awad Al-Omari, Samer Salih, Abbas Al Mutair, Arnab Pain
EPI_ISL_678166	Pathogen Genomics Lab King Abdullah University of Science and Technology(KAUST)	Pathogen Genomics Lab King Abdullah University of Science and Technology(KAUST)	Raeece Naeem, Sara Mfarrej, Luke Esau, Amanda Ooi, Sharif Hala, Awad Al-Omari, Samer Salih, Abbas Al Mutair, Arnab Pain
EPI_ISL_678167	Pathogen Genomics Lab King Abdullah University of Science and Technology(KAUST)	Pathogen Genomics Lab King Abdullah University of Science and Technology(KAUST)	Olga Douvropoulou, Sara Mfarrej, Raushan Nugmanova, Raeece Naeem, Sharif Hala, Luke Esau, Amanda Ooi, Awad Al-Omari, Samer Salih, Abbas Al Mutair, Arnab Pain
EPI_ISL_678168	Pathogen Genomics Lab King Abdullah University of Science and Technology(KAUST)	Pathogen Genomics Lab King Abdullah University of Science and Technology(KAUST)	Sara Mfarrej, Luke Esau, Amanda Ooi, Sharif Hala, Raeece Naeem, Awad Al-Omari, Samer Salih, Abbas Al Mutair, Arnab Pain
EPI_ISL_678169	Pathogen Genomics Lab King Abdullah University of Science and Technology(KAUST)	Pathogen Genomics Lab King Abdullah University of Science and Technology(KAUST)	Raeece Naeem, Sara Mfarrej, Amanda Ooi, Luke Esau, Sharif Hala, Awad Al-Omari, Samer Salih, Abbas Al Mutair, Arnab Pain
EPI_ISL_678170	Pathogen Genomics Lab King Abdullah University of Science and Technology(KAUST)	Pathogen Genomics Lab King Abdullah University of Science and Technology(KAUST)	Muhammad Shuaib, Sara Mfarrej, Raushan Nugmanova, Olga Douvropoulou, Raeece Naeem, Sharif Hala, Luke Esau, Amanda Ooi, Awad Al-Omari, Samer Salih, Abbas Al Mutair, Arnab Pain
EPI_ISL_678171	Pathogen Genomics Lab King Abdullah University of Science and Technology(KAUST)	Pathogen Genomics Lab King Abdullah University of Science and Technology(KAUST)	Sara Mfarrej, Sharif Hala, Luke Esau, Amanda Ooi, Raeece Naeem, Awad Al-Omari, Samer Salih, Abbas Al Mutair, Arnab Pain
EPI_ISL_678172	Pathogen Genomics Lab King Abdullah University of Science and Technology(KAUST)	Pathogen Genomics Lab King Abdullah University of Science and Technology(KAUST)	Olga Douvropoulou, Sara Mfarrej, Raushan Nugmanova, Sharif Hala, Raeece Naeem, Asim Khogeer, Fadwa Alofi, Afrah Alsomali, Jumana Taha, Abdulaziz Alahmadi, Kahled Algithami, Anwar Hashem, Naif Almontashiri, Arnab Pain
EPI_ISL_678173	Pathogen Genomics Lab King Abdullah University of Science and Technology(KAUST)	Pathogen Genomics Lab King Abdullah University of Science and Technology(KAUST)	Raeece Naeem, Sara Mfarrej, Luke Esau, Amanda Ooi, Sharif Hala, Awad Al-Omari, Samer Salih, Abbas Al Mutair, Arnab Pain
EPI_ISL_678174	Pathogen Genomics Lab King Abdullah University of Science and Technology(KAUST)	Pathogen Genomics Lab King Abdullah University of Science and Technology(KAUST)	Muhammad Shuaib, Sara Mfarrej, Amanda Ooi, Luke Esau, Sharif Hala, Raeece Naeem, Awad Al-Omari, Samer Salih, Abbas Al Mutair, Arnab Pain

EPI_ISL_678175	Pathogen Genomics Lab King Abdullah University of Science and Technology(KAUST)	Pathogen Genomics Lab King Abdullah University of Science and Technology(KAUST)	Raeece Naeem, Sara Mfarrej, Amanda Ooi, Luke Esau, Sharif Hala, Awad Al-Omari, Samer Salih, Abbas Al Mutair, Arnab Pain
EPI_ISL_678176	Pathogen Genomics Lab King Abdullah University of Science and Technology(KAUST)	Pathogen Genomics Lab King Abdullah University of Science and Technology(KAUST)	Sara Mfarrej, Luke Esau, Amanda Ooi, Sharif Hala, Raeece Naeem, Awad Al-Omari, Samer Salih, Abbas Al Mutair, Arnab Pain
EPI_ISL_678177, EPI_ISL_678178, EPI_ISL_678179	Pathogen Genomics Lab King Abdullah University of Science and Technology(KAUST)	Pathogen Genomics Lab King Abdullah University of Science and Technology(KAUST)	Sara Mfarrej, Sharif Hala, Luke Esau, Amanda Ooi, Raeece Naeem, Awad Al-Omari, Samer Salih, Abbas Al Mutair, Arnab Pain
EPI_ISL_678180	Pathogen Genomics Lab King Abdullah University of Science and Technology(KAUST)	Pathogen Genomics Lab King Abdullah University of Science and Technology(KAUST)	Olga Douvropoulou, Sara Mfarrej, Raushan Nugmanova, Sharif Hala, Raeece Naeem, Asim Khogeer, Fadwa Alofi, Afrah Alsomali, Jumana Taha, Abdulaziz Alahmadi, Kahled Algithami, Anwar Hashem, Naif Almontashiri, Arnab Pain
EPI_ISL_678181	Pathogen Genomics Lab King Abdullah University of Science and Technology(KAUST)	Pathogen Genomics Lab King Abdullah University of Science and Technology(KAUST)	Raeece Naeem, Sara Mfarrej, Amanda Ooi, Luke Esau, Sharif Hala, Awad Al-Omari, Samer Salih, Abbas Al Mutair, Arnab Pain
EPI_ISL_678182	Pathogen Genomics Lab King Abdullah University of Science and Technology(KAUST)	Pathogen Genomics Lab King Abdullah University of Science and Technology(KAUST)	Sara Mfarrej, Olga Douvropoulou, Raushan Nugmanova, Raeece Naeem, Sharif Hala, Awad Al-Omari, Samer Salih, Abbas Al Mutair, Arnab Pain
EPI_ISL_678183	Pathogen Genomics Lab King Abdullah University of Science and Technology(KAUST)	Pathogen Genomics Lab King Abdullah University of Science and Technology(KAUST)	Sara Mfarrej, Luke Esau, Amanda Ooi, Sharif Hala, Raeece Naeem, Awad Al-Omari, Samer Salih, Abbas Al Mutair, Arnab Pain
EPI_ISL_678184	Pathogen Genomics Lab King Abdullah University of Science and Technology(KAUST)	Pathogen Genomics Lab King Abdullah University of Science and Technology(KAUST)	Muhammad Shuaib, Raeece Naeem, Sara Mfarrej, Olga Douvropoulou, Raushan Nugmanova, Sharif Hala, Awad Al-Omari, Samer Salih, Abbas Al Mutair, Arnab Pain
EPI_ISL_678187	Pathogen Genomics Lab King Abdullah University of Science and Technology(KAUST)	Pathogen Genomics Lab King Abdullah University of Science and Technology(KAUST)	Raushan Nugmanova, Sharif Hala, Sara Mfarrej, Olga Douvropoulou, Raeece Naeem, Awad Al-Omari, Samer Salih, Abbas Al Mutair, Arnab Pain
EPI_ISL_678188	Pathogen Genomics Lab King Abdullah University of Science and Technology(KAUST)	Pathogen Genomics Lab King Abdullah University of Science and Technology(KAUST)	Raeece Naeem, Sara Mfarrej, Amanda Ooi, Luke Esau, Sharif Hala, Awad Al-Omari, Samer Salih, Abbas Al Mutair, Arnab Pain
EPI_ISL_678189	Pathogen Genomics Lab King Abdullah University of Science and Technology(KAUST)	Pathogen Genomics Lab King Abdullah University of Science and Technology(KAUST)	Raeece Naeem, Sara Mfarrej, Luke Esau, Amanda Ooi, Sharif Hala, Awad Al-Omari, Samer Salih, Abbas Al Mutair, Arnab Pain
EPI_ISL_678190	Pathogen Genomics Lab King Abdullah University of Science and Technology(KAUST)	Pathogen Genomics Lab King Abdullah University of Science and Technology(KAUST)	Olga Douvropoulou, Sharif Hala, Sara Mfarrej, Raushan Nugmanova, Raeece Naeem, Awad Al-Omari, Samer Salih, Abbas Al Mutair, Arnab Pain
EPI_ISL_678191	Pathogen Genomics Lab King Abdullah University of Science and Technology(KAUST)	Pathogen Genomics Lab King Abdullah University of Science and Technology(KAUST)	Raeece Naeem, Sara Mfarrej, Amanda Ooi, Luke Esau, Sharif Hala, Awad Al-Omari, Samer Salih, Abbas Al Mutair, Arnab Pain
EPI_ISL_678192	Pathogen Genomics Lab King Abdullah University of Science and Technology(KAUST)	Pathogen Genomics Lab King Abdullah University of Science and Technology(KAUST)	Raeece Naeem, Sharif Hala, Sara Mfarrej, Raushan Nugmanova, Olga Douvropoulou, Awad Al-Omari, Samer Salih, Abbas Al Mutair, Arnab Pain
EPI_ISL_678193	Pathogen Genomics Lab King Abdullah University of Science and Technology(KAUST)	Pathogen Genomics Lab King Abdullah University of Science and Technology(KAUST)	Raeece Naeem, Sara Mfarrej, Luke Esau, Amanda Ooi, Sharif Hala, Awad Al-Omari, Samer Salih, Abbas Al Mutair, Arnab Pain
EPI_ISL_678194	Pathogen Genomics Lab King Abdullah University of Science and Technology(KAUST)	Pathogen Genomics Lab King Abdullah University of Science and Technology(KAUST)	Raeece Naeem, Sharif Hala, Sara Mfarrej, Raushan Nugmanova, Olga Douvropoulou, Awad Al-Omari, Samer Salih, Abbas Al Mutair, Arnab Pain
EPI_ISL_678195	Pathogen Genomics Lab King Abdullah University of Science and Technology(KAUST)	Pathogen Genomics Lab King Abdullah University of Science and Technology(KAUST)	Raeece Naeem, Sara Mfarrej, Luke Esau, Amanda Ooi, Sharif Hala, Awad A-Omari, Samer Salih, Abbas Al Mutair, Arnab Pain
EPI_ISL_678196, EPI_ISL_678197	Pathogen Genomics Lab King Abdullah University of Science and Technology(KAUST)	Pathogen Genomics Lab King Abdullah University of Science and Technology(KAUST)	Raeece Naeem, Sara Mfarrej, Luke Esau, Amanda Ooi, Sharif Hala, Awad Al-Omari, Samer Salih, Abbas Al Mutair, Arnab Pain
EPI_ISL_678198, EPI_ISL_678199	Pathogen Genomics Lab King Abdullah University of Science and Technology(KAUST)	Pathogen Genomics Lab King Abdullah University of Science and Technology(KAUST)	Raeece Naeem, Sara Mfarrej, Luke Esau, Amanda Ooi, Sharif Hala, Awad Al-Omari, Samer Salih, Abbas Al Mutair, Arnab Pain
EPI_ISL_678200, EPI_ISL_678201, EPI_ISL_678202	Pathogen Genomics Lab King Abdullah University of Science and Technology(KAUST)	Pathogen Genomics Lab King Abdullah University of Science and Technology(KAUST)	Raushan Nugmanova, Sharif Hala, Sara Mfarrej, Olga Douvropoulou, Raeece Naeem, Awad Al-Omari, Samer Salih, Abbas Al Mutair, Arnab Pain
EPI_ISL_678203, EPI_ISL_678204	Pathogen Genomics Lab King Abdullah University of Science and Technology(KAUST)	Pathogen Genomics Lab King Abdullah University of Science and Technology(KAUST)	Sara Mfarrej, Luke Esau, Amanda Ooi, Sharif Hala, Raeece Naeem, Awad Al-Omari, Samer Salih, Abbas Al Mutair, Arnab Pain
EPI_ISL_678205	Pathogen Genomics Lab King Abdullah University of Science and Technology(KAUST)	Pathogen Genomics Lab King Abdullah University of Science and Technology(KAUST)	Raushan Nugmanova, Sharif Hala, Sara Mfarrej, Olga Douvropoulou, Raeece Naeem, Awad Al-Omari, Samer Salih, Abbas Al Mutair, Arnab Pain
EPI_ISL_678206, EPI_ISL_678207, EPI_ISL_678208, EPI_ISL_678209	Pathogen Genomics Lab King Abdullah University of Science and Technology(KAUST)	Pathogen Genomics Lab King Abdullah University of Science and Technology(KAUST)	Muhammad Shuaib, Sara Mfarrej, Amanda Ooi, Luke Esau, Sharif Hala, Raeece Naeem, Awad Al-Omari, Samer Salih, Abbas Al Mutair, Arnab Pain
EPI_ISL_678210	Pathogen Genomics Lab King Abdullah University of Science and Technology(KAUST)	Pathogen Genomics Lab King Abdullah University of Science and Technology(KAUST)	Muhammad Shuaib, Raeece Naeem, Sara Mfarrej, Olga Douvropoulou, Raushan Nugmanova, Sharif Hala, Awad Al-Omari, Samer Salih, Abbas Al Mutair, Arnab Pain
EPI_ISL_678211, EPI_ISL_678212	Pathogen Genomics Lab King Abdullah University of Science and Technology(KAUST)	Pathogen Genomics Lab King Abdullah University of Science and Technology(KAUST)	Muhammad Shuaib, Sara Mfarrej, Amanda Ooi, Luke Esau, Sharif Hala, Raeece Naeem, Awad Al-Omari, Samer Salih, Abbas Al Mutair, Arnab Pain
EPI_ISL_678213	Pathogen Genomics Lab King Abdullah University of Science and Technology(KAUST)	Pathogen Genomics Lab King Abdullah University of Science and Technology(KAUST)	Muhammad Shuaib, Raeece Naeem, Sara Mfarrej, Olga Douvropoulou, Raushan Nugmanova, Sharif Hala, Awad Al-Omari, Samer Salih, Abbas Al Mutair, Arnab Pain
EPI_ISL_678214	Pathogen Genomics Lab King Abdullah University of Science and Technology(KAUST)	Pathogen Genomics Lab King Abdullah University of Science and Technology(KAUST)	Muhammad Shuaib, Raeece Naeem, Sara Mfarrej, Olga Douvropoulou, Raushan Nugmanova, Sharif Hala, Awad Al-Omari, Samer Salih, Abbas Al Mutair, Arnab Pain
EPI_ISL_678215	Pathogen Genomics Lab King Abdullah University of Science and Technology(KAUST)	Pathogen Genomics Lab King Abdullah University of Science and Technology(KAUST)	Sara Mfarrej, Olga Douvropoulou, Raushan Nugmanova, Raeece Naeem, Sharif Hala, Awad Al-Omari, Samer Salih, Abbas Al Mutair, Arnab Pain
EPI_ISL_678216, EPI_ISL_678217, EPI_ISL_678218	Pathogen Genomics Lab King Abdullah University of Science and Technology(KAUST)	Pathogen Genomics Lab King Abdullah University of Science and Technology(KAUST)	Sara Mfarrej, Sharif Hala, Luke Esau, Amanda Ooi, Raeece Naeem, Awad Al-Omari, Samer Salih, Abbas Al Mutair, Arnab Pain
EPI_ISL_678219	Pathogen Genomics Lab King Abdullah University of Science and Technology(KAUST)	Pathogen Genomics Lab King Abdullah University of Science and Technology(KAUST)	Sara Mfarrej, Raeece Naeem, Luke Esau, Amanda Ooi, Sharif Hala, Awad Al-Omari, Samer Salih, Abbas Al Mutair, Arnab Pain
EPI_ISL_678220	Pathogen Genomics Lab King Abdullah University of Science and Technology(KAUST)	Pathogen Genomics Lab King Abdullah University of Science and Technology(KAUST)	Sara Mfarrej, Sharif Hala, Luke Esau, Amanda Ooi, Raeece Naeem, Awad Al-Omari, Samer Salih, Abbas Al Mutair, Arnab Pain
EPI_ISL_678221, EPI_ISL_678222, EPI_ISL_678223	Pathogen Genomics Lab King Abdullah University of Science and Technology(KAUST)	Pathogen Genomics Lab King Abdullah University of Science and Technology(KAUST)	Sara Mfarrej, Olga Douvropoulou, Raushan Nugmanova, Raeece Naeem, Sharif Hala, Awad Al-Omari, Samer Salih, Abbas Al Mutair, Arnab Pain
EPI_ISL_678224	Pathogen Genomics Lab King Abdullah University of Science and Technology(KAUST)	Pathogen Genomics Lab King Abdullah University of Science and Technology(KAUST)	Sara Mfarrej, Sharif Hala, Luke Esau, Amanda Ooi, Raeece Naeem, Awad Al-Omari, Samer Salih, Abbas Al Mutair, Arnab Pain
EPI_ISL_678225	Pathogen Genomics Lab King Abdullah University of Science and Technology(KAUST)	Pathogen Genomics Lab King Abdullah University of Science and Technology(KAUST)	Sara Mfarrej, Olga Douvropoulou, Raushan Nugmanova, Raeece Naeem, Sharif Hala, Awad Al-Omari, Samer Salih, Abbas Al Mutair, Arnab Pain
EPI_ISL_678226	Pathogen Genomics Lab King Abdullah University of Science and Technology(KAUST)	Pathogen Genomics Lab King Abdullah University of Science and Technology(KAUST)	Olga Douvropoulou, Sara Mfarrej, Raushan Nugmanova, Sharif Hala, Raeece Naeem, Asim Khogeer, Fadwa Alofi, Afrah Alsomali, Jumana Taha, Abdulaziz Alahmadi, Kahled Algithami, Anwar Hashem, Naif Almontashiri, Arnab Pain

EPI_ISL_678236	Pathogen Genomics Lab King Abdullah University of Science and Technology(KAUST)	Pathogen Genomics Lab King Abdullah University of Science and Technology(KAUST)	Sara Mfarrej, Luke Esau, Amanda Ooi, Sharif Hala, Raeece Naeem, Afrah Alsomali, Fadwa Alofi, Asim Khogeer, Jumana Taha, Abdulaziz Alahmadi, Kahled Algithami, Anwar Hashem, Naif Almontashiri, Arnab Pain
EPI_ISL_678237	Pathogen Genomics Lab King Abdullah University of Science and Technology(KAUST)	Pathogen Genomics Lab King Abdullah University of Science and Technology(KAUST)	Sara Mfarrej, Olga Douvropoulou, Raushan Nugmanova, Sharif Hala, Raeece Naeem, Amanda Ooi, Luke Esau, Fadwa Alofi, Afrah Alsomali, Asim Khogeer, Jumana Taha, Abdulaziz Alahmadi, Kahled Algithami, Anwar Hashem, Naif Almontashiri, Arnab Pain
EPI_ISL_678238	Pathogen Genomics Lab King Abdullah University of Science and Technology(KAUST)	Pathogen Genomics Lab King Abdullah University of Science and Technology(KAUST)	Muhammad Shuaib, Sara Mfarrej, Amanda Ooi, Luke Esau, Sharif Hala, Raeece Naeem, Awad Al-Omari, Samer Salih, Abbas Al Mutair, Arnab Pain
EPI_ISL_678239	Pathogen Genomics Lab King Abdullah University of Science and Technology(KAUST)	Pathogen Genomics Lab King Abdullah University of Science and Technology(KAUST)	Muhammad Shuaib, Raeece Naeem, Sara Mfarrej, Olga Douvropoulou, Raushan Nugmanova, Sharif Hala, Awad Al-Omari, Samer Salih, Abbas Al Mutair, Arnab Pain
EPI_ISL_678240	Pathogen Genomics Lab King Abdullah University of Science and Technology(KAUST)	Pathogen Genomics Lab King Abdullah University of Science and Technology(KAUST)	Sara Mfarrej, Raushan Nugmanova, Olga Douvropoulou, Raeece Naeem, Sharif Hala, Luke Esau, Amanda Ooi, Awad Al-Omari, Samer Salih, Abbas Al Mutair, Arnab Pain
EPI_ISL_678241	Pathogen Genomics Lab King Abdullah University of Science and Technology(KAUST)	Pathogen Genomics Lab King Abdullah University of Science and Technology(KAUST)	Muhammad Shuaib, Raeece Naeem, Raushan Nugmanova, Olga Douvropoulou, Sharif Hala, Sara Mfarrej, Afrah Alsomali, Asim Khogeer, Fadwa Alofi,Jumana Taha, Abdulaziz Alahmadi, Kahled Algithami, Anwar Hashem, Naif Almontashiri, Arnab Pain
EPI_ISL_678242	Pathogen Genomics Lab King Abdullah University of Science and Technology(KAUST)	Pathogen Genomics Lab King Abdullah University of Science and Technology(KAUST)	Muhammad Shuaib, Sharif Hala, Sara Mfarrej, Luke Esau, Amanda Ooi, Raeece Naeem, Afrah Alsomali, Asim Khogeer, Fadwa Alofi,Jumana Taha, Abdulaziz Alahmadi, Kahled Algithami, Anwar Hashem, Naif Almontashiri, Arnab Pain
EPI_ISL_678243	Pathogen Genomics Lab King Abdullah University of Science and Technology(KAUST)	Pathogen Genomics Lab King Abdullah University of Science and Technology(KAUST)	Sara Mfarrej, Raeece Naeem, Raushan Nugmanova, Olga Douvropoulou, Luke Esau, Amanda Ooi, Sharif Hala, Afrah Alsomali, Asim Khogeer, Fadwa Alofi,Jumana Taha, Abdulaziz Alahmadi, Kahled Algithami, Anwar Hashem, Naif Almontashiri, Arnab Pain
EPI_ISL_678244	Pathogen Genomics Lab King Abdullah University of Science and Technology(KAUST)	Pathogen Genomics Lab King Abdullah University of Science and Technology(KAUST)	Sara Mfarrej, Olga Douvropoulou, Raushan Nugmanova, Raeece Naeem, Sharif Hala, Awad Al-Omari, Samer Salih, Abbas Al Mutair, Arnab Pain
EPI_ISL_678245	Pathogen Genomics Lab King Abdullah University of Science and Technology(KAUST)	Pathogen Genomics Lab King Abdullah University of Science and Technology(KAUST)	Sara Mfarrej, Luke Esau, Amanda Ooi, Sharif Hala, Raeece Naeem, Awad Al-Omari, Samer Salih, Abbas Al Mutair, Arnab Pain
EPI_ISL_678247	Pathogen Genomics Lab King Abdullah University of Science and Technology (KAUST)	Pathogen Genomics Lab King Abdullah University of Science and Technology (KAUST)	Muhammad Shuaib, Raeece Naeem, Sara Mfarrej, Olga Douvropoulou, Raushan Nugmanova, Sharif Hala, Awad Al-Omari, Samer Salih, Abbas Al Mutair, Arnab Pain
EPI_ISL_678248, EPI_ISL_678249	Center for public health - Skopje	Research Center for Genetic Engineering and Biotechnology "Georgi D. Efremov" , Macedonian Academy of Sciences and Arts	RCGEB - MASA
EPI_ISL_678250	Clinical Hospital - Shtip	Research Center for Genetic Engineering and Biotechnology "Georgi D. Efremov" , Macedonian Academy of Sciences and Arts	RCGEB - MASA
EPI_ISL_678251	Institute for Lung Diseases in Children - Skopje	Research Center for Genetic Engineering and Biotechnology "Georgi D. Efremov" , Macedonian Academy of Sciences and Arts	RCGEB - MASA
EPI_ISL_678252	General Hospital - Kumanovo	Research Center for Genetic Engineering and Biotechnology "Georgi D. Efremov" , Macedonian Academy of Sciences and Arts	RCGEB - MASA
EPI_ISL_678253	Institute for Lung Diseases in Children - Skopje	Research Center for Genetic Engineering and Biotechnology "Georgi D. Efremov" , Macedonian Academy of Sciences and Arts	RCGEB - MASA
EPI_ISL_678254	General Hospital - Strumica	Research Center for Genetic Engineering and Biotechnology "Georgi D. Efremov" , Macedonian Academy of Sciences and Arts	RCGEB - MASA
EPI_ISL_678255, EPI_ISL_678256	General Hospital - Ohrid	Research Center for Genetic Engineering and Biotechnology "Georgi D. Efremov" , Macedonian Academy of Sciences and Arts	RCGEB - MASA
EPI_ISL_678257	PHI Institute of Nephrology - Struga	Research Center for Genetic Engineering and Biotechnology "Georgi D. Efremov" , Macedonian Academy of Sciences and Arts	RCGEB - MASA
EPI_ISL_678258	General Hospital - Prilep	Research Center for Genetic Engineering and Biotechnology "Georgi D. Efremov" , Macedonian Academy of Sciences and Arts	RCGEB - MASA
EPI_ISL_678259	Clinical Hospital - Shtip	Research Center for Genetic Engineering and Biotechnology "Georgi D. Efremov" , Macedonian Academy of Sciences and Arts	RCGEB - MASA
EPI_ISL_678260	General Hospital - Struga	Research Center for Genetic Engineering and Biotechnology "Georgi D. Efremov" , Macedonian Academy of Sciences and Arts	RCGEB - MASA
EPI_ISL_678261, EPI_ISL_678262, EPI_ISL_678263, EPI_ISL_678264, EPI_ISL_678265, EPI_ISL_678266, EPI_ISL_678267, EPI_ISL_678268, EPI_ISL_678269, EPI_ISL_678270, EPI_ISL_678271, EPI_ISL_678272			
see above	Communicable Disease Laboratory, Public Health Directorate	Communicable Disease Laboratory, Public Health Directorate	Alwasti,H., Altaif,Z., AlHujairi,Z., AlAbbas,Z.
EPI_ISL_678274, EPI_ISL_678281, EPI_ISL_678282, EPI_ISL_678284, EPI_ISL_678285, EPI_ISL_678286, EPI_ISL_678289	Mikrobiologie, RARI	Mikrobiologie, RARI	Krasnov,Y.M., Naryshkina,E.A., Guseva,N.P., Sosedova,E.A., Fedorov,A.V., Badanin,D.V., Sharapova,N.A., Portenko,S.A., Shcherbakova,S.A., Kutyrev,V.V.
EPI_ISL_678290, EPI_ISL_678291	University of Szeged, Institute of Clinical Microbiology	National Laboratory of Virology, Szentágothai Research Centre	Endre Gábor Tóth, Balázs Somogyi, Brigitta, Gabriella Terhes, Ferenc Jakab, Gábor Kemenesi
EPI_ISL_678318, EPI_ISL_678320, EPI_ISL_678321, EPI_ISL_678325, EPI_ISL_678326, EPI_ISL_678327, EPI_ISL_678328, EPI_ISL_678330, EPI_ISL_678331, EPI_ISL_678332, EPI_ISL_678336, EPI_ISL_678337, EPI_ISL_678340, EPI_ISL_678342, EPI_ISL_678343, EPI_ISL_678345, EPI_ISL_678346, EPI_ISL_678348, EPI_ISL_678351, EPI_ISL_678358, EPI_ISL_678363, EPI_ISL_678364, EPI_ISL_678365, EPI_ISL_678366, EPI_ISL_678367, EPI_ISL_678368, EPI_ISL_678370, EPI_ISL_678371, EPI_ISL_678372, EPI_ISL_678373, EPI_ISL_678374, EPI_ISL_678375, EPI_ISL_678376, EPI_ISL_678377, EPI_ISL_678378, EPI_ISL_678379, EPI_ISL_678380, EPI_ISL_678381, EPI_ISL_678383, EPI_ISL_678386			
see above	Area of Virology, Serology and Virology Division (SAViD), New South Wales Health Pathology Randwick	Virology Research Laboratory; Area of Virology, Serology and Virology Division (SAViD), New South Wales Health Pathology Randwick	Foster, C.; Au, J.; Ruiz Silva, M.; Deveson, I.; Bull, R.; Van Hal, S.; Rawlinson, W.
EPI_ISL_678387, EPI_ISL_678388, EPI_ISL_678389, EPI_ISL_678390,	University of Debrecen, Department of Medical Microbiology	National Laboratory of Virology, Szentágothai Research Centre	Endre Gábor Tóth, Balázs Somogyi, Brigitta Zana, Eszter Csoma, Ferenc Jakab, Gábor Kemenesi

EPI_ISL_678391, EPI_ISL_678392, EPI_ISL_678393, EPI_ISL_678394, EPI_ISL_678395, EPI_ISL_678396			
EPI_ISL_678399, EPI_ISL_678400, EPI_ISL_678401, EPI_ISL_678402, EPI_ISL_678403, EPI_ISL_678404, EPI_ISL_678405, EPI_ISL_678406, EPI_ISL_678407, EPI_ISL_678408, EPI_ISL_678409, EPI_ISL_678410, EPI_ISL_678411, EPI_ISL_678412, EPI_ISL_678413, EPI_ISL_678414, EPI_ISL_678415, EPI_ISL_678416, EPI_ISL_678417, EPI_ISL_678418, EPI_ISL_678419, EPI_ISL_678420, EPI_ISL_678421, EPI_ISL_678422, EPI_ISL_678423, EPI_ISL_678424, EPI_ISL_678425, EPI_ISL_678426, EPI_ISL_678427, EPI_ISL_678428, EPI_ISL_678429, EPI_ISL_678430, EPI_ISL_678431, EPI_ISL_678432, EPI_ISL_678433, EPI_ISL_678434, EPI_ISL_678435, EPI_ISL_678436, EPI_ISL_678437, EPI_ISL_678438, EPI_ISL_678439, EPI_ISL_678440, EPI_ISL_678441, EPI_ISL_678442, EPI_ISL_678443, EPI_ISL_678444, EPI_ISL_678445, EPI_ISL_678446, EPI_ISL_678447, EPI_ISL_678448, EPI_ISL_678449, EPI_ISL_678450, EPI_ISL_678451, EPI_ISL_678452, EPI_ISL_678453, EPI_ISL_678454, EPI_ISL_678455, EPI_ISL_678456, EPI_ISL_678457, EPI_ISL_678458, EPI_ISL_678459, EPI_ISL_678460, EPI_ISL_678461, EPI_ISL_678462, EPI_ISL_678463, EPI_ISL_678464, EPI_ISL_678465, EPI_ISL_678466, EPI_ISL_678467, EPI_ISL_678468, EPI_ISL_678469, EPI_ISL_678470, EPI_ISL_678471, EPI_ISL_678472, EPI_ISL_678473, EPI_ISL_678474, EPI_ISL_678475, EPI_ISL_678476, EPI_ISL_678477, EPI_ISL_678478, EPI_ISL_678479, EPI_ISL_678480, EPI_ISL_678481, EPI_ISL_678482, EPI_ISL_678483, EPI_ISL_678484, EPI_ISL_678485			
see above	Department of Clinical Microbiology	GIGA Medical Genomics	Keith Durkin, Maria Artesi, Sébastien Bontems, Raphaël Boreux, Bouchra Boujemla, Cécile Meex, Pierrette Melin, Marie-Pierre Hayette, Vincent Bours
EPI_ISL_678486, EPI_ISL_678489, EPI_ISL_678491, EPI_ISL_678492, EPI_ISL_678493	Veterinary Specialized Institute "Sabac", Serbia	Veterinary Specialized Institute "Kraljevo", Serbia	Vidanovic,D., Tesovic,B., Knezevic,A., Jovanovic,T., Jankovic,M., Sekler,M., Banovic Djeri,B., Petrovic,T., Mrkovacki, S., Volkening,J., Afonso,C.
EPI_ISL_678548, EPI_ISL_678549, EPI_ISL_678550, EPI_ISL_678551, EPI_ISL_678552, EPI_ISL_678553, EPI_ISL_678554, EPI_ISL_678555, EPI_ISL_678556, EPI_ISL_678557, EPI_ISL_678558, EPI_ISL_678559, EPI_ISL_678560, EPI_ISL_678561, EPI_ISL_678562, EPI_ISL_678563, EPI_ISL_678564, EPI_ISL_678565			
see above	Netcare	KRISP, KZN Research Innovation and Sequencing Platform	Giandhari J, Pillay S, Lessells R, ChimukangaraB, Mdlalose K, York D, Khan S, Tegally H, Wilkinson E, de Oliveira T
EPI_ISL_678566	NHLS-IALCH	KRISP, KZN Research Innovation and Sequencing Platform	Giandhari J, Pillay S, Lessells R, ChimukangaraB, Mdlalose K, York D, Khan S, Tegally H, Wilkinson E, de Oliveira T
EPI_ISL_678567, EPI_ISL_678568	Netcare	KRISP, KZN Research Innovation and Sequencing Platform	Giandhari J, Pillay S, Lessells R, ChimukangaraB, Mdlalose K, York D, Khan S, Tegally H, Wilkinson E, de Oliveira T
EPI_ISL_678569, EPI_ISL_678570	NHLS-IALCH	KRISP, KZN Research Innovation and Sequencing Platform	Giandhari J, Pillay S, Lessells R, ChimukangaraB, Mdlalose K, York D, Khan S, Tegally H, Wilkinson E, de Oliveira T
EPI_ISL_678571, EPI_ISL_678572, EPI_ISL_678573	Netcare	KRISP, KZN Research Innovation and Sequencing Platform	Giandhari J, Pillay S, Lessells R, ChimukangaraB, Mdlalose K, York D, Khan S, Tegally H, Wilkinson E, de Oliveira T
EPI_ISL_678574, EPI_ISL_678575	NHLS-IALCH	KRISP, KZN Research Innovation and Sequencing Platform	Giandhari J, Pillay S, Lessells R, ChimukangaraB, Mdlalose K, York D, Khan S, Tegally H, Wilkinson E, de Oliveira T
EPI_ISL_678595, EPI_ISL_678596, EPI_ISL_678597	Netcare	KRISP, KZN Research Innovation and Sequencing Platform	Giandhari J, Pillay S, Lessells R, ChimukangaraB, Mdlalose K, York D, Khan S, Tegally H, Wilkinson E, de Oliveira T
EPI_ISL_678598, EPI_ISL_678599, EPI_ISL_678600, EPI_ISL_678601, EPI_ISL_678602, EPI_ISL_678603, EPI_ISL_678604, EPI_ISL_678605, EPI_ISL_678606, EPI_ISL_678607, EPI_ISL_678608, EPI_ISL_678609, EPI_ISL_678610, EPI_ISL_678611, EPI_ISL_678612, EPI_ISL_678613, EPI_ISL_678614, EPI_ISL_678615, EPI_ISL_678616, EPI_ISL_678617, EPI_ISL_678618, EPI_ISL_678619, EPI_ISL_678620, EPI_ISL_678621, EPI_ISL_678622, EPI_ISL_678623, EPI_ISL_678624, EPI_ISL_678625, EPI_ISL_678626, EPI_ISL_678627, EPI_ISL_678628, EPI_ISL_678629, EPI_ISL_678630, EPI_ISL_678631, EPI_ISL_678632, EPI_ISL_678633, EPI_ISL_678634, EPI_ISL_678635, EPI_ISL_678636, EPI_ISL_678637, EPI_ISL_678638, EPI_ISL_678639, EPI_ISL_678640, EPI_ISL_678641, EPI_ISL_678642, EPI_ISL_678643			
see above	NHLS-IALCH	KRISP, KZN Research Innovation and Sequencing Platform	Giandhari J, Pillay S, Lessells R, ChimukangaraB, Mdlalose K, York D, Khan S, Tegally H, Wilkinson E, de Oliveira T
EPI_ISL_678644, EPI_ISL_678645, EPI_ISL_678646, EPI_ISL_678647, EPI_ISL_678648, EPI_ISL_678649, EPI_ISL_678650, EPI_ISL_678651, EPI_ISL_678652, EPI_ISL_678653, EPI_ISL_678654, EPI_ISL_678655, EPI_ISL_678656, EPI_ISL_678657, EPI_ISL_678658, EPI_ISL_678659, EPI_ISL_678660, EPI_ISL_678661, EPI_ISL_678662, EPI_ISL_678663, EPI_ISL_678664, EPI_ISL_678665, EPI_ISL_678666, EPI_ISL_678667, EPI_ISL_678668, EPI_ISL_678669, EPI_ISL_678670, EPI_ISL_678671, EPI_ISL_678672, EPI_ISL_678673, EPI_ISL_678674, EPI_ISL_678675, EPI_ISL_678676, EPI_ISL_678677, EPI_ISL_678678, EPI_ISL_678679, EPI_ISL_678680, EPI_ISL_678681, EPI_ISL_678682, EPI_ISL_678683, EPI_ISL_678684, EPI_ISL_678685, EPI_ISL_678686, EPI_ISL_678687, EPI_ISL_678688, EPI_ISL_678689, EPI_ISL_678690, EPI_ISL_678691, EPI_ISL_678692, EPI_ISL_678693, EPI_ISL_678694, EPI_ISL_678695, EPI_ISL_678696, EPI_ISL_678697, EPI_ISL_678698, EPI_ISL_678699, EPI_ISL_678700, EPI_ISL_678701, EPI_ISL_678702, EPI_ISL_678703, EPI_ISL_678704, EPI_ISL_678706, EPI_ISL_678707, EPI_ISL_678708, EPI_ISL_678709, EPI_ISL_678710, EPI_ISL_678711, EPI_ISL_678712, EPI_ISL_678713, EPI_ISL_678714, EPI_ISL_678715, EPI_ISL_678716, EPI_ISL_678717, EPI_ISL_678719, EPI_ISL_678720, EPI_ISL_678722, EPI_ISL_678723, EPI_ISL_678724, EPI_ISL_678728, EPI_ISL_678730, EPI_ISL_678731, EPI_ISL_678732, EPI_ISL_678734, EPI_ISL_678735, EPI_ISL_678738, EPI_ISL_678744, EPI_ISL_678745, EPI_ISL_678748, EPI_ISL_678750, EPI_ISL_678751, EPI_ISL_678752, EPI_ISL_678753, EPI_ISL_678754, EPI_ISL_678755, EPI_ISL_678756, EPI_ISL_678759, EPI_ISL_678761, EPI_ISL_678764, EPI_ISL_678765, EPI_ISL_678766, EPI_ISL_678768, EPI_ISL_678769, EPI_ISL_678770, EPI_ISL_678771, EPI_ISL_678772, EPI_ISL_678773, EPI_ISL_678774, EPI_ISL_678775, EPI_ISL_678776, EPI_ISL_678777, EPI_ISL_678778, EPI_ISL_678779, EPI_ISL_678780, EPI_ISL_678781, EPI_ISL_678785, EPI_ISL_678828, EPI_ISL_678830			
see above	Respiratory Virus Unit, Microbiology Services Colindale, Public Health England	COVID-19 Genomics UK (COG-UK) Consortium	PHE Covid Sequencing Team
EPI_ISL_678831	Centre for Enzyme Innovation, University of Portsmouth / Translational Research Laboratory, Portsmouth Hospitals NHS Trust	COVID-19 Genomics UK (COG-UK) Consortium	Angela Beckett,Yann Bourgeois,Garry Scarlett,Sharon Glaysher,Scott Elliott,Kelly Bicknell,Robert Impey,Allyson Lloyd,Sarah Wyllie,Ethan Butcher,Anoop Chauhan,Samuel Robson
EPI_ISL_678832, EPI_ISL_678834, EPI_ISL_678835, EPI_ISL_678836, EPI_ISL_678837, EPI_ISL_678838, EPI_ISL_678839, EPI_ISL_678840, EPI_ISL_678841, EPI_ISL_678842, EPI_ISL_678843, EPI_ISL_678844, EPI_ISL_678845, EPI_ISL_678846, EPI_ISL_678847, EPI_ISL_678848, EPI_ISL_678849, EPI_ISL_678850, EPI_ISL_678851, EPI_ISL_678852, EPI_ISL_678853, EPI_ISL_678854, EPI_ISL_678855, EPI_ISL_678856, EPI_ISL_678857, EPI_ISL_678858, EPI_ISL_678859, EPI_ISL_678860, EPI_ISL_678862, EPI_ISL_678863, EPI_ISL_678864, EPI_ISL_678865, EPI_ISL_678866, EPI_ISL_678867, EPI_ISL_678868, EPI_ISL_678869, EPI_ISL_678870, EPI_ISL_678871, EPI_ISL_678872, EPI_ISL_678873, EPI_ISL_678874, EPI_ISL_678875, EPI_ISL_678876, EPI_ISL_678877, EPI_ISL_678878, EPI_ISL_678879, EPI_ISL_678880, EPI_ISL_678881, EPI_ISL_678882, EPI_ISL_678883, EPI_ISL_678884, EPI_ISL_678885, EPI_ISL_678886, EPI_ISL_678887, EPI_ISL_678888, EPI_ISL_678889, EPI_ISL_678890, EPI_ISL_678891, EPI_ISL_678892, EPI_ISL_678893, EPI_ISL_678894, EPI_ISL_678895, EPI_ISL_678896, EPI_ISL_678897, EPI_ISL_678898, EPI_ISL_678899, EPI_ISL_678900, EPI_ISL_678901, EPI_ISL_678902, EPI_ISL_678903, EPI_ISL_678904, EPI_ISL_678905, EPI_ISL_678906, EPI_ISL_678907, EPI_ISL_678908, EPI_ISL_678909, EPI_ISL_678910, EPI_ISL_678911, EPI_ISL_678912, EPI_ISL_678913, EPI_ISL_678914, EPI_ISL_678915			
see above	Department of Pathology, University of Cambridge	COVID-19 Genomics UK (COG-UK) Consortium	Aminu S. Jahun, Yasmin Chaudhry, Grant Hall, Iliana Georgana, Myra Hosmillo, Martin D. Curran, Malte Pinckert, Surendra Parmar, Ian Goodfellow
EPI_ISL_678916, EPI_ISL_678917, EPI_ISL_678918, EPI_ISL_678919	Quadram Institute Bioscience	COVID-19 Genomics UK (COG-UK) Consortium	Dave J. Baker, Gemma L. Kay, Alp Aydin, Thanh Le-Viet, Steven Rudder, Ana P. Tedim, Anastasia Kolyva, Maria Diaz, Leonardo de Oliveira Martins, Nabil-Fareed Alikhan, Lizzie Meadows, Rachael Stanley, Ngozi Elumogo, Muhammed Yasir, Nicholas M. Thomson, Alexander J Trotter, Rachel Gilroy, Samuel Bloomfield, Claire Stuart, Andrew Bell, Reenesh Prakash, Samir Dervisevic, Alison E. Mather, John Wain, Mark Webber, Andrew J. Page, Justin O'Grady
EPI_ISL_678920, EPI_ISL_678921, EPI_ISL_678922, EPI_ISL_678923, EPI_ISL_678924, EPI_ISL_678925, EPI_ISL_678926, EPI_ISL_678927, EPI_ISL_678928, EPI_ISL_678929, EPI_ISL_678930, EPI_ISL_678932, EPI_ISL_678933, EPI_ISL_678934, EPI_ISL_678935, EPI_ISL_678936, EPI_ISL_678937, EPI_ISL_678938, EPI_ISL_678939, EPI_ISL_678940, EPI_ISL_678941, EPI_ISL_678942, EPI_ISL_678943, EPI_ISL_678944, EPI_ISL_678945, EPI_ISL_678946, EPI_ISL_678947, EPI_ISL_678948, EPI_ISL_678949, EPI_ISL_678950, EPI_ISL_678951, EPI_ISL_678952, EPI_ISL_678953, EPI_ISL_678954, EPI_ISL_678955, EPI_ISL_678956, EPI_ISL_678957, EPI_ISL_678958, EPI_ISL_678959, EPI_ISL_678960, EPI_ISL_678961, EPI_ISL_678962, EPI_ISL_678963, EPI_ISL_678964, EPI_ISL_678965, EPI_ISL_678966, EPI_ISL_678967, EPI_ISL_678968, EPI_ISL_678969, EPI_ISL_678970, EPI_ISL_678971, EPI_ISL_678972, EPI_ISL_678973, EPI_ISL_678974, EPI_ISL_678975, EPI_ISL_678976, EPI_ISL_678977, EPI_ISL_678978, EPI_ISL_678979, EPI_ISL_678980, EPI_ISL_678981, EPI_ISL_678982, EPI_ISL_678984, EPI_ISL_678985, EPI_ISL_678986, EPI_ISL_678987, EPI_ISL_678988, EPI_ISL_678989, EPI_ISL_678990, EPI_ISL_678991, EPI_ISL_678992, EPI_ISL_678993, EPI_ISL_678994, EPI_ISL_678995, EPI_ISL_678997, EPI_ISL_678998, EPI_ISL_678999, EPI_ISL_679000, EPI_ISL_679001, EPI_ISL_679002, EPI_ISL_679003, EPI_ISL_679004, EPI_ISL_679005, EPI_ISL_679006, EPI_ISL_679007			
see above	Queens Medical Centre, Clinical Microbiology Department / DeepSeq Nottingham	COVID-19 Genomics UK (COG-UK) Consortium	Gemma Clark, Wendy Smith, Manjinder Khakh, Vicki M Fleming, Michelle M Lister, Hannah Howson-Wells, Jonathan Ball, Patrick McClure, Joseph Chappell, Theocharis Tsoleridis, Nadine Holmes, Matthew Carlisle, Christopher Moore, Fei Sang, Johnny Debebe, Victoria Wright, Matthew Loose
EPI_ISL_679008, EPI_ISL_679009, EPI_ISL_679010, EPI_ISL_679011, EPI_ISL_679013, EPI_ISL_679014, EPI_ISL_679015, EPI_ISL_679016, EPI_ISL_679017, EPI_ISL_679018, EPI_ISL_679019, EPI_ISL_679020, EPI_ISL_679021, EPI_ISL_679022, EPI_ISL_679023, EPI_ISL_679024, EPI_ISL_679025, EPI_ISL_679026, EPI_ISL_679027, EPI_ISL_679028, EPI_ISL_679029, EPI_ISL_679030, EPI_ISL_679031, EPI_ISL_679032, EPI_ISL_679033, EPI_ISL_679034, EPI_ISL_679035, EPI_ISL_679036, EPI_ISL_679037, EPI_ISL_679038, EPI_ISL_679039, EPI_ISL_679040, EPI_ISL_679041, EPI_ISL_679042, EPI_ISL_679043, EPI_ISL_679044, EPI_ISL_679045, EPI_ISL_679046, EPI_ISL_679047, EPI_ISL_679048, EPI_ISL_679049, EPI_ISL_679050, EPI_ISL_679053, EPI_ISL_679054, EPI_ISL_679055, EPI_ISL_679056, EPI_ISL_679057, EPI_ISL_679058, EPI_ISL_679059, EPI_ISL_679060, EPI_ISL_679061, EPI_ISL_679062, EPI_ISL_679063, EPI_ISL_679064, EPI_ISL_679065, EPI_ISL_679066, EPI_ISL_679067, EPI_ISL_679069, EPI_ISL_679072, EPI_ISL_679073, EPI_ISL_679074, EPI_ISL_679075, EPI_ISL_679076, EPI_ISL_679077, EPI_ISL_679078, EPI_ISL_679079, EPI_ISL_679080, EPI_ISL_679081, EPI_ISL_679082, EPI_ISL_679083, EPI_ISL_679084, EPI_ISL_679085, EPI_ISL_679086, EPI_ISL_679088, EPI_ISL_679089, EPI_ISL_679090, EPI_ISL_679091, EPI_ISL_679092, EPI_ISL_679093, EPI_ISL_679094, EPI_ISL_679095, EPI_ISL_679096, EPI_ISL_679097, EPI_ISL_679098, EPI_ISL_679099, EPI_ISL_679100, EPI_ISL_679101, EPI_ISL_679102, EPI_ISL_679104, EPI_ISL_679105, EPI_ISL_679107, EPI_ISL_679108, EPI_ISL_679112, EPI_ISL_679113, EPI_ISL_679115, EPI_ISL_679117, EPI_ISL_679118, EPI_ISL_679119, EPI_ISL_679121, EPI_ISL_679124, EPI_ISL_679125, EPI_ISL_679128, EPI_ISL_679129, EPI_ISL_679130, EPI_ISL_679131, EPI_ISL_679132, EPI_ISL_679133			
see above	University of Birmingham	COVID-19 Genomics UK (COG-UK) Consortium	Institute of Microbiology, University of Birmingham: Claire McMurray, Joanne Stockton, Samuel Nicholls, Radoslaw Poplawski, Will Rowe, Josh Quick, Nicholas Loman. University of Birmingham Testing Laboratory: Celina M Whalley, Andrew Bosworth, Charlotte Poxon, Kasun Wanigasooriya, Oliver Pickles, Mike Kidd, Alex Richter, Andrew D Beggs PHE Heartlands Lab: Husam Osman, Andrew Bosworth. Queen Elizabeth Hospital: Anna Casey
EPI_ISL_679134, EPI_ISL_679135, EPI_ISL_679136, EPI_ISL_679137, EPI_ISL_679138, EPI_ISL_679139, EPI_ISL_679140, EPI_ISL_679142, EPI_ISL_679143, EPI_ISL_679145, EPI_ISL_679146, EPI_ISL_679147, EPI_ISL_679148, EPI_ISL_679149, EPI_ISL_679150, EPI_ISL_679151, EPI_ISL_679152, EPI_ISL_679153, EPI_ISL_679154, EPI_ISL_679155, EPI_ISL_679156, EPI_ISL_679157, EPI_ISL_679158, EPI_ISL_679160, EPI_ISL_679161, EPI_ISL_679162, EPI_ISL_679163, EPI_ISL_679164, EPI_ISL_679165, EPI_ISL_679166, EPI_ISL_679167, EPI_ISL_679168, EPI_ISL_679169, EPI_ISL_679170, EPI_ISL_679171, EPI_ISL_679172, EPI_ISL_679173, EPI_ISL_679174, EPI_ISL_679175, EPI_ISL_679176, EPI_ISL_679177, EPI_ISL_679178, EPI_ISL_679179, EPI_ISL_679180, EPI_ISL_679181, EPI_ISL_679182, EPI_ISL_679183, EPI_ISL_679184, EPI_ISL_679185, EPI_ISL_679186, EPI_ISL_679187, EPI_ISL_679188, EPI_ISL_679189, EPI_ISL_679190, EPI_ISL_679191, EPI_ISL_679192, EPI_ISL_679193, EPI_ISL_679194, EPI_ISL_679195, EPI_ISL_679196, EPI_ISL_679197, EPI_ISL_679198, EPI_ISL_679199, EPI_ISL_679200, EPI_ISL_679201, EPI_ISL_679202, EPI_ISL_679203, EPI_ISL_679204, EPI_ISL_679205, EPI_ISL_679206, EPI_ISL_679207, EPI_ISL_679208, EPI_ISL_679209, EPI_ISL_679210, EPI_ISL_679211, EPI_ISL_679212, EPI_ISL_679213, EPI_ISL_679214, EPI_ISL_679215, EPI_ISL_679216, EPI_ISL_679217, EPI_ISL_679218, EPI_ISL_679219, EPI_ISL_679220, EPI_ISL_679221, EPI_ISL_679223, EPI_ISL_679224, EPI_ISL_679226, EPI_ISL_679228, EPI_ISL_679230, EPI_ISL_679232, EPI_ISL_679234, EPI_ISL_679236, EPI_ISL_679238, EPI_ISL_679240, EPI_ISL_679242, EPI_ISL_679244, EPI_ISL_679246, EPI_ISL_679248			
see above	Department of Pathology, University of Cambridge	COVID-19 Genomics UK (COG-UK) Consortium	Aminu S. Jahun, Yasmin Chaudhry, Grant Hall, Iliana Georgana, Myra Hosmillo, Martin D. Curran, Malte Pinckert, Surendra Parmar, Ian Goodfellow
EPI_ISL_679249, EPI_ISL_679250, EPI_ISL_679251, EPI_ISL_679252, EPI_ISL_679253, EPI_ISL_679254, EPI_ISL_679255, EPI_ISL_679256, EPI_ISL_679257, EPI_ISL_679258, EPI_ISL_679259, EPI_ISL_679260, EPI_ISL_679261, EPI_ISL_679262, EPI_ISL_679263, EPI_ISL_679264, EPI_ISL_679265, EPI_ISL_679266, EPI_ISL_679267, EPI_ISL_679268, EPI_ISL_679269, EPI_ISL_679270, EPI_ISL_679272, EPI_ISL_679273, EPI_ISL_679274, EPI_ISL_679275, EPI_ISL_679276, EPI_ISL_679277, EPI_ISL_679278, EPI_ISL_679279, EPI_ISL_679280, EPI_ISL_679281, EPI_ISL_679282, EPI_ISL_679283, EPI_ISL_679284, EPI_ISL_679286, EPI_ISL_679287, EPI_ISL_679288, EPI_ISL_679289, EPI_ISL_679290, EPI_ISL_679291, EPI_ISL_679292, EPI_ISL_679293, EPI_ISL_679294, EPI_ISL_679295, EPI_ISL_679296, EPI_ISL_679297, EPI_ISL_679298, EPI_ISL_679299, EPI_ISL_679300, EPI_ISL_679301, EPI_ISL_679302, EPI_ISL_679303, EPI_ISL_679304,			

EPI_ISL_679305, EPI_ISL_679306, EPI_ISL_679307, EPI_ISL_679308, EPI_ISL_679309, EPI_ISL_679310, EPI_ISL_679311, EPI_ISL_679312, EPI_ISL_679313, EPI_ISL_679314			
see above	Queens Medical Centre, Clinical Microbiology Department / DeepSeq Nottingham	COVID-19 Genomics UK (COG-UK) Consortium	Gemma Clark, Wendy Smith, Manjinder Khakh, Vicki M Fleming, Michelle M Lister, Hannah Howson-Wells, Jonathan Ball, Patrick McClure, Joseph Chappell, Theocharis Tsoleridis, Nadine Holmes, Matthew Carlisle, Christopher Moore, Fei Sang, Johnny Debebe, Victoria Wright, Matthew Loose
EPI_ISL_679381, EPI_ISL_679384, EPI_ISL_679386, EPI_ISL_679390, EPI_ISL_679391, EPI_ISL_679392, EPI_ISL_679394, EPI_ISL_679395, EPI_ISL_679402	Liverpool Clinical Laboratories	COVID-19 Genomics UK (COG-UK) Consortium	Sam Haldenby, Anita Lucaci, Steve Paterson, Julian Hiscox, Alistair Darby, M Almsaud, A Alrezaihi, Muhannad Alruwaili, Stuart D Armstrong, Jones Benjamin, Eleanor G Bentley, Anu Chawla, Jordan J Clark, Angela Cowell, Richard Eccles, Isabel García-Dorival, Matthew Gemmell, Alessandro Gerada, PKF Gilmore, Richard Gregory, Ximeng Han, Catherine Hartley, Margaret Hughes, Miren Iturriza-Gomara, James Johnson, L Luu, Jenifer Manson, Charlotte Nelson, Elaine O'Toole, Cassie Olateju, Rebekah Penrice-Randal , Lucille Rainbow, N.P Randle, Trevor Ian Robinson, Parul Sharma, Ghada T Shawli, James P Stewart, Neil Swainston, Ecaterina Vamos, Joanne Watts, Mark Whitehead
EPI_ISL_679406, EPI_ISL_679407, EPI_ISL_679408, EPI_ISL_679409, EPI_ISL_679410, EPI_ISL_679411, EPI_ISL_679412, EPI_ISL_679413, EPI_ISL_679415, EPI_ISL_679416, EPI_ISL_679417, EPI_ISL_679418, EPI_ISL_679419, EPI_ISL_679420, EPI_ISL_679421, EPI_ISL_679422, EPI_ISL_679423, EPI_ISL_679424, EPI_ISL_679425, EPI_ISL_679426, EPI_ISL_679427, EPI_ISL_679428, EPI_ISL_679429, EPI_ISL_679430, EPI_ISL_679431, EPI_ISL_679432, EPI_ISL_679433, EPI_ISL_679434, EPI_ISL_679435, EPI_ISL_679436, EPI_ISL_679437, EPI_ISL_679438, EPI_ISL_679440, EPI_ISL_679441, EPI_ISL_679442, EPI_ISL_679443, EPI_ISL_679445, EPI_ISL_679446			
see above	University College London Hospital	COVID-19 Genomics UK (COG-UK) Consortium	Judith Heaney, Matthew Byott, Catherine Houlihan, Dan Frampton, Stuart Kirk, Moira Spyer and Eleni Nastouli
EPI_ISL_679447, EPI_ISL_679448, EPI_ISL_679449, EPI_ISL_679450, EPI_ISL_679452, EPI_ISL_679453, EPI_ISL_679454, EPI_ISL_679455, EPI_ISL_679456, EPI_ISL_679458, EPI_ISL_679459, EPI_ISL_679460, EPI_ISL_679461, EPI_ISL_679462, EPI_ISL_679463, EPI_ISL_679464, EPI_ISL_679465, EPI_ISL_679466, EPI_ISL_679467, EPI_ISL_679469, EPI_ISL_679470, EPI_ISL_679471, EPI_ISL_679473, EPI_ISL_679475, EPI_ISL_679476, EPI_ISL_679477, EPI_ISL_679484, EPI_ISL_679485, EPI_ISL_679488, EPI_ISL_679489, EPI_ISL_679492, EPI_ISL_679493, EPI_ISL_679494, EPI_ISL_679496, EPI_ISL_679497, EPI_ISL_679500, EPI_ISL_679502, EPI_ISL_679505, EPI_ISL_679506, EPI_ISL_679507, EPI_ISL_679508, EPI_ISL_679509, EPI_ISL_679510, EPI_ISL_679511, EPI_ISL_679512, EPI_ISL_679513, EPI_ISL_679514, EPI_ISL_679515, EPI_ISL_679516, EPI_ISL_679517, EPI_ISL_679518, EPI_ISL_679520, EPI_ISL_679521, EPI_ISL_679523, EPI_ISL_679524, EPI_ISL_679525, EPI_ISL_679526, EPI_ISL_679527, EPI_ISL_679528, EPI_ISL_679529, EPI_ISL_679534, EPI_ISL_679536, EPI_ISL_679539, EPI_ISL_679541, EPI_ISL_679546, EPI_ISL_679547, EPI_ISL_679549, EPI_ISL_679550, EPI_ISL_679551, EPI_ISL_679552, EPI_ISL_679553, EPI_ISL_679554, EPI_ISL_679555, EPI_ISL_679556, EPI_ISL_679557, EPI_ISL_679558, EPI_ISL_679559, EPI_ISL_679560, EPI_ISL_679561, EPI_ISL_679563, EPI_ISL_679565, EPI_ISL_679569, EPI_ISL_679570, EPI_ISL_679572, EPI_ISL_679574, EPI_ISL_679575, EPI_ISL_679576, EPI_ISL_679578, EPI_ISL_679579, EPI_ISL_679580, EPI_ISL_679581, EPI_ISL_679586, EPI_ISL_679588, EPI_ISL_679590, EPI_ISL_679593, EPI_ISL_679594, EPI_ISL_679598, EPI_ISL_679600, EPI_ISL_679601, EPI_ISL_679603, EPI_ISL_679605, EPI_ISL_679609, EPI_ISL_679611, EPI_ISL_679612, EPI_ISL_679613, EPI_ISL_679620, EPI_ISL_679621, EPI_ISL_679622, EPI_ISL_679623, EPI_ISL_679624, EPI_ISL_679625, EPI_ISL_679627, EPI_ISL_679628			
see above	University College London, Great Ormond Street Hospital for Children NHS Foundation Trust, Imperial College Healthcare NHS Trust	COVID-19 Genomics UK (COG-UK) Consortium	Sergi Castellano, Rachel Williams, Mark Kristiansen, Paola Resende Silva, Sunando Roy, Tony Brooks, Helena Tutill, Paola Niola, Patricia Dyal, Charlotte Williams, Leysa Forrest, Yasmin Panchbhaya, Jacqueline Findlay, Samuel Weeks, Julianne Brown, Kathryn Harris, Paul Randell, James Price, Alison Holmes, Judith Breuer
EPI_ISL_679629, EPI_ISL_679630, EPI_ISL_679631, EPI_ISL_679632, EPI_ISL_679633, EPI_ISL_679634, EPI_ISL_679635, EPI_ISL_679636, EPI_ISL_679637, EPI_ISL_679638, EPI_ISL_679639, EPI_ISL_679640, EPI_ISL_679641, EPI_ISL_679642, EPI_ISL_679643, EPI_ISL_679644, EPI_ISL_679645, EPI_ISL_679646, EPI_ISL_679647, EPI_ISL_679648, EPI_ISL_679649, EPI_ISL_679650, EPI_ISL_679651, EPI_ISL_679652, EPI_ISL_679653, EPI_ISL_679654, EPI_ISL_679655, EPI_ISL_679656, EPI_ISL_679657, EPI_ISL_679658, EPI_ISL_679659, EPI_ISL_679660, EPI_ISL_679661, EPI_ISL_679662, EPI_ISL_679663, EPI_ISL_679664, EPI_ISL_679665, EPI_ISL_679666, EPI_ISL_679667, EPI_ISL_679668, EPI_ISL_679669, EPI_ISL_679670, EPI_ISL_679671, EPI_ISL_679672, EPI_ISL_679673, EPI_ISL_679674, EPI_ISL_679675, EPI_ISL_679676, EPI_ISL_679677, EPI_ISL_679678, EPI_ISL_679679, EPI_ISL_679680, EPI_ISL_679681, EPI_ISL_679682, EPI_ISL_679683, EPI_ISL_679684, EPI_ISL_679685, EPI_ISL_679686, EPI_ISL_679687, EPI_ISL_679688, EPI_ISL_679689, EPI_ISL_679690, EPI_ISL_679691, EPI_ISL_679692, EPI_ISL_679693, EPI_ISL_679694, EPI_ISL_679695, EPI_ISL_679696, EPI_ISL_679697, EPI_ISL_679698, EPI_ISL_679699, EPI_ISL_679700, EPI_ISL_679701, EPI_ISL_679702, EPI_ISL_679703, EPI_ISL_679704, EPI_ISL_679705, EPI_ISL_679706, EPI_ISL_679707, EPI_ISL_679708, EPI_ISL_679709, EPI_ISL_679710, EPI_ISL_679711, EPI_ISL_679712, EPI_ISL_679713, EPI_ISL_679714, EPI_ISL_679715, EPI_ISL_679716, EPI_ISL_679717, EPI_ISL_679718, EPI_ISL_679719, EPI_ISL_679720, EPI_ISL_679721, EPI_ISL_679722, EPI_ISL_679723, EPI_ISL_679724, EPI_ISL_679725, EPI_ISL_679726, EPI_ISL_679727, EPI_ISL_679728, EPI_ISL_679729, EPI_ISL_679730, EPI_ISL_679731, EPI_ISL_679732, EPI_ISL_679733, EPI_ISL_679734, EPI_ISL_679735, EPI_ISL_679736, EPI_ISL_679737, EPI_ISL_679738, EPI_ISL_679739, EPI_ISL_679740, EPI_ISL_679741, EPI_ISL_679742, EPI_ISL_679743, EPI_ISL_679744, EPI_ISL_679745, EPI_ISL_679746, EPI_ISL_679747, EPI_ISL_679748, EPI_ISL_679749, EPI_ISL_679750, EPI_ISL_679751, EPI_ISL_679752, EPI_ISL_679753, EPI_ISL_679754, EPI_ISL_679755, EPI_ISL_679756, EPI_ISL_679757, EPI_ISL_679758, EPI_ISL_679759, EPI_ISL_679760, EPI_ISL_679761, EPI_ISL_679762, EPI_ISL_679763, EPI_ISL_679764, EPI_ISL_679765, EPI_ISL_679766, EPI_ISL_679767, EPI_ISL_679768, EPI_ISL_679769, EPI_ISL_679770, EPI_ISL_679771, EPI_ISL_679772, EPI_ISL_679773, EPI_ISL_679774, EPI_ISL_679775, EPI_ISL_679776, EPI_ISL_679777, EPI_ISL_679778, EPI_ISL_679779, EPI_ISL_679780, EPI_ISL_679781, EPI_ISL_679782, EPI_ISL_679783, EPI_ISL_679784, EPI_ISL_679785, EPI_ISL_679786, EPI_ISL_679787, EPI_ISL_679788, EPI_ISL_679789, EPI_ISL_679790, EPI_ISL_679791, EPI_ISL_679792, EPI_ISL_679793, EPI_ISL_679794, EPI_ISL_679795, EPI_ISL_679796, EPI_ISL_679797, EPI_ISL_679798, EPI_ISL_679799, EPI_ISL_679800, EPI_ISL_679801, EPI_ISL_679802, EPI_ISL_679803, EPI_ISL_679804, EPI_ISL_679805, EPI_ISL_679806, EPI_ISL_679807, EPI_ISL_679808, EPI_ISL_679809, EPI_ISL_679810, EPI_ISL_679811, EPI_ISL_679812, EPI_ISL_679813, EPI_ISL_679814, EPI_ISL_679815, EPI_ISL_679816, EPI_ISL_679817, EPI_ISL_679818, EPI_ISL_679819, EPI_ISL_679820, EPI_ISL_679821, EPI_ISL_679822, EPI_ISL_679823, EPI_ISL_679824, EPI_ISL_679825, EPI_ISL_679826, EPI_ISL_679827, EPI_ISL_679828, EPI_ISL_679829, EPI_ISL_679830, EPI_ISL_679831, EPI_ISL_679832, EPI_ISL_679833, EPI_ISL_679834, EPI_ISL_679835, EPI_ISL_679836, EPI_ISL_679837, EPI_ISL_679838, EPI_ISL_679839, EPI_ISL_679840, EPI_ISL_679841, EPI_ISL_679842, EPI_ISL_679843, EPI_ISL_679844, EPI_ISL_679845, EPI_ISL_679846, EPI_ISL_679847, EPI_ISL_679848, EPI_ISL_679849, EPI_ISL_679850, EPI_ISL_679851			
see above	Oxford Viromics, NDM, University of Oxford; Oxford University Hospitals; Basingstoke and North Hampshire Hospital	COVID-19 Genomics UK (COG-UK) Consortium	Tanya Golubchik, David Bonsall, George Macintyre, Amy Trebes, Mariateresa de Cesare, Catrin Moore, Alex Mobbs, Anita Justice, Robert Shaw, Monique Andersson, Timothy Peto, Emma Wise, Nathan Moore, Jessica Lynch, Nick Cortes, Matilde Mori, Stephen Kidd, David Buck, John Todd, Christophe Fraser
EPI_ISL_679852, EPI_ISL_679853, EPI_ISL_679854, EPI_ISL_679856, EPI_ISL_679857, EPI_ISL_679858, EPI_ISL_679859, EPI_ISL_679860, EPI_ISL_679861, EPI_ISL_679862, EPI_ISL_679863, EPI_ISL_679864, EPI_ISL_679865, EPI_ISL_679866, EPI_ISL_679867, EPI_ISL_679868, EPI_ISL_679869, EPI_ISL_679870, EPI_ISL_679871, EPI_ISL_679872, EPI_ISL_679873, EPI_ISL_679874, EPI_ISL_679875, EPI_ISL_679876, EPI_ISL_679877, EPI_ISL_679878, EPI_ISL_679879, EPI_ISL_679880, EPI_ISL_679881, EPI_ISL_679882, EPI_ISL_679883, EPI_ISL_679884, EPI_ISL_679885, EPI_ISL_679886, EPI_ISL_679887, EPI_ISL_679888, EPI_ISL_679889, EPI_ISL_679890, EPI_ISL_679891, EPI_ISL_679892, EPI_ISL_679893, EPI_ISL_679894, EPI_ISL_679895, EPI_ISL_679896, EPI_ISL_679897, EPI_ISL_679898, EPI_ISL_679899, EPI_ISL_679901, EPI_ISL_679902, EPI_ISL_679903, EPI_ISL_679904, EPI_ISL_679907, EPI_ISL_679908, EPI_ISL_679909, EPI_ISL_679910, EPI_ISL_679912, EPI_ISL_679913, EPI_ISL_679914, EPI_ISL_679915, EPI_ISL_679916, EPI_ISL_679917, EPI_ISL_679918, EPI_ISL_679919, EPI_ISL_679921, EPI_ISL_679922			
see above	Virology Department, Sheffield Teaching Hospitals NHS Foundation Trust/Department of Infection, Immunity and Cardiovascular Disease, The Medical School, University of Sheffield	COVID-19 Genomics UK (COG-UK) Consortium	Thushan de Silva, Matthew Parker, Nikki Smith, Adri Angyal, Rebecca Brown, Luke Green, Rachel Tucker, Paul Parsons, Danielle Groves, Katie Johnson, Laura Carrilero, Alex Keeley, Dave Partridge, Matthew Wyles, Benjamin Lindsey, Mehmet Yavuz, Mohammad Raza, Cariad Evans
EPI_ISL_679924, EPI_ISL_679925, EPI_ISL_679926, EPI_ISL_679927, EPI_ISL_679928, EPI_ISL_679929, EPI_ISL_679930, EPI_ISL_679931, EPI_ISL_679932, EPI_ISL_679933, EPI_ISL_679934, EPI_ISL_679935, EPI_ISL_679936, EPI_ISL_679937, EPI_ISL_679938, EPI_ISL_679939, EPI_ISL_679940, EPI_ISL_679941, EPI_ISL_679942, EPI_ISL_679943, EPI_ISL_679944, EPI_ISL_679945			
see above	Queens Medical Centre, Clinical Microbiology Department / DeepSeq Nottingham	COVID-19 Genomics UK (COG-UK) Consortium	Gemma Clark, Wendy Smith, Manjinder Khakh, Vicki M Fleming, Michelle M Lister, Hannah Howson-Wells, Jonathan Ball, Patrick McClure, Joseph Chappell, Theocharis Tsoleridis, Nadine Holmes, Matthew Carlisle, Christopher Moore, Fei Sang, Johnny Debebe, Victoria Wright, Matthew Loose
EPI_ISL_679946, EPI_ISL_679947, EPI_ISL_679948, EPI_ISL_679949, EPI_ISL_679951, EPI_ISL_679952, EPI_ISL_679953, EPI_ISL_679954, EPI_ISL_679955, EPI_ISL_679957, EPI_ISL_679958, EPI_ISL_679959, EPI_ISL_679960, EPI_ISL_679961, EPI_ISL_679962, EPI_ISL_679963, EPI_ISL_679964, EPI_ISL_679965, EPI_ISL_679966			
see above	Lincolnshire Hospitals and DeepSeq Nottingham	COVID-19 Genomics UK (COG-UK) Consortium	Nichola Duckworth, Tim Sloan, Sarah Walsh, Jonathan Ball, Patrick McClure, Joeseph Chappell, Nadine Holmes, Matthew Carlisle, Christopher Moore, Fei Sang, Johnny Debebe, Victoria Wright, Matthew Loose
EPI_ISL_679987, EPI_ISL_679988, EPI_ISL_679989, EPI_ISL_679990, EPI_ISL_679991, EPI_ISL_679992, EPI_ISL_679993, EPI_ISL_679994, EPI_ISL_679995, EPI_ISL_679996, EPI_ISL_679997, EPI_ISL_679998, EPI_ISL_679999, EPI_ISL_680000, EPI_ISL_680002, EPI_ISL_680003, EPI_ISL_680004, EPI_ISL_680005, EPI_ISL_680006, EPI_ISL_680007, EPI_ISL_680008, EPI_ISL_680009, EPI_ISL_680010, EPI_ISL_680011, EPI_ISL_680012, EPI_ISL_680013, EPI_ISL_680014, EPI_ISL_680015			
see above	Virology Department, Sheffield Teaching Hospitals NHS Foundation Trust/Department of Infection, Immunity and Cardiovascular Disease, The Medical School, University of Sheffield	COVID-19 Genomics UK (COG-UK) Consortium	Thushan de Silva, Matthew Parker, Nikki Smith, Adri Angyal, Rebecca Brown, Luke Green, Rachel Tucker, Paul Parsons, Danielle Groves, Katie Johnson, Laura Carrilero, Alex Keeley, Dave Partridge, Matthew Wyles, Benjamin Lindsey, Mehmet Yavuz, Mohammad Raza, Cariad Evans
EPI_ISL_680016, EPI_ISL_680017, EPI_ISL_680018, EPI_ISL_680019, EPI_ISL_680020, EPI_ISL_680021, EPI_ISL_680025, EPI_ISL_680026, EPI_ISL_680027, EPI_ISL_680028, EPI_ISL_680029, EPI_ISL_680030, EPI_ISL_680033, EPI_ISL_680034, EPI_ISL_680035			
see above	Centre for Enzyme Innovation, University of Portsmouth / Translational Research Laboratory, Portsmouth Hospitals NHS Trust	COVID-19 Genomics UK (COG-UK) Consortium	Angela Beckett,Yann Bourgeois,Garry Scarlett,Sharon Glaysher,Scott Elliott,Kelly Bicknell,Robert Impey,Allyson Lloyd,Sarah Wyllie,Ethan Butcher,Anoop Chauhan,Samuel Robson
EPI_ISL_680037, EPI_ISL_680038, EPI_ISL_680039, EPI_ISL_680040, EPI_ISL_680041, EPI_ISL_680042, EPI_ISL_680043, EPI_ISL_680044, EPI_ISL_680045, EPI_ISL_680046, EPI_ISL_680047, EPI_ISL_680048, EPI_ISL_680049, EPI_ISL_680050, EPI_ISL_680051, EPI_ISL_680053, EPI_ISL_680054, EPI_ISL_680055, EPI_ISL_680056, EPI_ISL_680057, EPI_ISL_680058, EPI_ISL_680059, EPI_ISL_680060, EPI_ISL_680061, EPI_ISL_680062, EPI_ISL_680063, EPI_ISL_680064, EPI_ISL_680065, EPI_ISL_680066, EPI_ISL_680067, EPI_ISL_680068, EPI_ISL_680069, EPI_ISL_680070, EPI_ISL_680071, EPI_ISL_680072, EPI_ISL_680074, EPI_ISL_680075, EPI_ISL_680076, EPI_ISL_680077, EPI_ISL_680078, EPI_ISL_680079, EPI_ISL_680080, EPI_ISL_680082, EPI_ISL_680083, EPI_ISL_680086, EPI_ISL_680087, EPI_ISL_680088, EPI_ISL_680089, EPI_ISL_680090, EPI_ISL_680091, EPI_ISL_680092, EPI_ISL_680093, EPI_ISL_680094, EPI_ISL_680095, EPI_ISL_680096, EPI_ISL_680097, EPI_ISL_680098, EPI_ISL_680099, EPI_ISL_680100			
see above	Department of Pathology, University of Cambridge	COVID-19 Genomics UK (COG-UK) Consortium	Aminu S. Jahun, Yasmin Chaudhry, Grant Hall, Iliana Georgana, Myra Hosmillo, Martin D. Curran, Malte Pinckert, Surendra Parmar, Ian Goodfellow
EPI_ISL_680101, EPI_ISL_680103, EPI_ISL_680104, EPI_ISL_680105, EPI_ISL_680106, EPI_ISL_680108, EPI_ISL_680110, EPI_ISL_680111, EPI_ISL_680112, EPI_ISL_680113, EPI_ISL_680115, EPI_ISL_680116, EPI_ISL_680117, EPI_ISL_680118, EPI_ISL_680119, EPI_ISL_680120, EPI_ISL_680121, EPI_ISL_680122, EPI_ISL_680123, EPI_ISL_680126, EPI_ISL_680127, EPI_ISL_680129, EPI_ISL_680132, EPI_ISL_680133, EPI_ISL_680134, EPI_ISL_680135, EPI_ISL_680136, EPI_ISL_680137, EPI_ISL_680142, EPI_ISL_680143, EPI_ISL_680144, EPI_ISL_680145, EPI_ISL_680147, EPI_ISL_680148, EPI_ISL_680149, EPI_ISL_680150, EPI_ISL_680153, EPI_ISL_680154, EPI_ISL_680155, EPI_ISL_680156, EPI_ISL_680157, EPI_ISL_680158, EPI_ISL_680160, EPI_ISL_680162, EPI_ISL_680163, EPI_ISL_680164, EPI_ISL_680165, EPI_ISL_680166, EPI_ISL_680167, EPI_ISL_680168, EPI_ISL_680169, EPI_ISL_680170, EPI_ISL_680172, EPI_ISL_680174, EPI_ISL_680175, EPI_ISL_680176, EPI_ISL_680179, EPI_ISL_680180, EPI_ISL_680181, EPI_ISL_680182, EPI_ISL_680183, EPI_ISL_680184, EPI_ISL_680185, EPI_ISL_680186, EPI_ISL_680187, EPI_ISL_680188, EPI_ISL_680189, EPI_ISL_680190, EPI_ISL_680191, EPI_ISL_680192, EPI_ISL_680193, EPI_ISL_680195, EPI_ISL_680196, EPI_ISL_680197			
see above	Virology Department, Sheffield Teaching Hospitals NHS Foundation Trust/Department of Infection, Immunity and Cardiovascular Disease, The Medical School, University of	COVID-19 Genomics UK (COG-UK) Consortium	Thushan de Silva, Matthew Parker, Nikki Smith, Adri Angyal, Rebecca Brown, Luke Green, Rachel Tucker, Paul Parsons, Danielle Groves, Katie Johnson, Laura Carrilero, Alex Keeley, Dave Partridge, Matthew Wyles, Benjamin Lindsey, Mehmet Yavuz, Mohammad Raza, Cariad Evans

	Sheffield		
EPI_ISL_680198, EPI_ISL_680199, EPI_ISL_680200, EPI_ISL_680201, EPI_ISL_680202, EPI_ISL_680203, EPI_ISL_680204, EPI_ISL_680205, EPI_ISL_680206, EPI_ISL_680207, EPI_ISL_680209, EPI_ISL_680210, EPI_ISL_680211, EPI_ISL_680212, EPI_ISL_680213, EPI_ISL_680214, EPI_ISL_680215, EPI_ISL_680216, EPI_ISL_680217, EPI_ISL_680218, EPI_ISL_680219, EPI_ISL_680220, EPI_ISL_680221, EPI_ISL_680223, EPI_ISL_680224, EPI_ISL_680225, EPI_ISL_680226, EPI_ISL_680227, EPI_ISL_680228, EPI_ISL_680229, EPI_ISL_680230, EPI_ISL_680231, EPI_ISL_680232, EPI_ISL_680233, EPI_ISL_680234, EPI_ISL_680235, EPI_ISL_680239, EPI_ISL_680240, EPI_ISL_680241, EPI_ISL_680243, EPI_ISL_680244, EPI_ISL_680245, EPI_ISL_680246, EPI_ISL_680247, EPI_ISL_680248, EPI_ISL_680249, EPI_ISL_680250, EPI_ISL_680251, EPI_ISL_680252, EPI_ISL_680254, EPI_ISL_680255, EPI_ISL_680256, EPI_ISL_680257, EPI_ISL_680258, EPI_ISL_680259, EPI_ISL_680260, EPI_ISL_680261, EPI_ISL_680262, EPI_ISL_680264, EPI_ISL_680265, EPI_ISL_680266, EPI_ISL_680267, EPI_ISL_680268, EPI_ISL_680269, EPI_ISL_680270, EPI_ISL_680271, EPI_ISL_680272, EPI_ISL_680273, EPI_ISL_680274, EPI_ISL_680275, EPI_ISL_680277, EPI_ISL_680278, EPI_ISL_680279, EPI_ISL_680280, EPI_ISL_680281, EPI_ISL_680282, EPI_ISL_680289, EPI_ISL_680290, EPI_ISL_680292, EPI_ISL_680293, EPI_ISL_680294, EPI_ISL_680295, EPI_ISL_680296, EPI_ISL_680297, EPI_ISL_680298, EPI_ISL_680300, EPI_ISL_680301, EPI_ISL_680302, EPI_ISL_680304, EPI_ISL_680305, EPI_ISL_680306, EPI_ISL_680307, EPI_ISL_680309, EPI_ISL_680310, EPI_ISL_680311, EPI_ISL_680312, EPI_ISL_680313, EPI_ISL_680314, EPI_ISL_680315, EPI_ISL_680316, EPI_ISL_680317, EPI_ISL_680319, EPI_ISL_680320, EPI_ISL_680321, EPI_ISL_680322, EPI_ISL_680323, EPI_ISL_680324, EPI_ISL_680325, EPI_ISL_680326, EPI_ISL_680327, EPI_ISL_680329, EPI_ISL_680330, EPI_ISL_680331, EPI_ISL_680332, EPI_ISL_680333, EPI_ISL_680334, EPI_ISL_680335, EPI_ISL_680336, EPI_ISL_680337, EPI_ISL_680338, EPI_ISL_680339, EPI_ISL_680340, EPI_ISL_680341, EPI_ISL_680342, EPI_ISL_680343, EPI_ISL_680344, EPI_ISL_680345, EPI_ISL_680346, EPI_ISL_680348, EPI_ISL_680349, EPI_ISL_680351, EPI_ISL_680352, EPI_ISL_680353, EPI_ISL_680354, EPI_ISL_680355, EPI_ISL_680356, EPI_ISL_680358, EPI_ISL_680359, EPI_ISL_680360, EPI_ISL_680361, EPI_ISL_680362, EPI_ISL_680363, EPI_ISL_680364, EPI_ISL_680365, EPI_ISL_680366, EPI_ISL_680367, EPI_ISL_680368, EPI_ISL_680369, EPI_ISL_680371, EPI_ISL_680372, EPI_ISL_680373, EPI_ISL_680374, EPI_ISL_680375, EPI_ISL_680376, EPI_ISL_680377, EPI_ISL_680378, EPI_ISL_680379, EPI_ISL_680380, EPI_ISL_680381, EPI_ISL_680382, EPI_ISL_680383, EPI_ISL_680384, EPI_ISL_680385, EPI_ISL_680386, EPI_ISL_680387, EPI_ISL_680388, EPI_ISL_680389, EPI_ISL_680390, EPI_ISL_680391, EPI_ISL_680392, EPI_ISL_680393, EPI_ISL_680394, EPI_ISL_680395, EPI_ISL_680396, EPI_ISL_680397, EPI_ISL_680398, EPI_ISL_680399, EPI_ISL_680400, EPI_ISL_680401, EPI_ISL_680402, EPI_ISL_680403, EPI_ISL_680404, EPI_ISL_680405, EPI_ISL_680406, EPI_ISL_680407, EPI_ISL_680408, EPI_ISL_680409, EPI_ISL_680410, EPI_ISL_680412, EPI_ISL_680414, EPI_ISL_680415, EPI_ISL_680416, EPI_ISL_680417, EPI_ISL_680418, EPI_ISL_680419, EPI_ISL_680420, EPI_ISL_680421, EPI_ISL_680423, EPI_ISL_680424, EPI_ISL_680425, EPI_ISL_680426, EPI_ISL_680427, EPI_ISL_680428, EPI_ISL_680429, EPI_ISL_680430, EPI_ISL_680431, EPI_ISL_680432, EPI_ISL_680435, EPI_ISL_680436, EPI_ISL_680437, EPI_ISL_680438, EPI_ISL_680439, EPI_ISL_680441			
see above	Regional Virus Laboratory, Belfast Health and Social Care Trust	COVID-19 Genomics UK (COG-UK) Consortium	Conall McCaughey, James McKenna, Tanya Curran, Susan Feeney, Alison Watt, Ciara Cox, Mairead Connor, Zoltan Molnar, David Simpson, Derek Fairley
EPI_ISL_680442, EPI_ISL_680445, EPI_ISL_680446, EPI_ISL_680447, EPI_ISL_680448, EPI_ISL_680449, EPI_ISL_680450, EPI_ISL_680451, EPI_ISL_680452, EPI_ISL_680453, EPI_ISL_680454, EPI_ISL_680455, EPI_ISL_680456, EPI_ISL_680457, EPI_ISL_680458, EPI_ISL_680459, EPI_ISL_680460			
see above	West of Scotland Specialist Virology Centre, NHSGGC / MRC-University of Glasgow Centre for Virus Research	COVID-19 Genomics UK (COG-UK) Consortium	Ana da Silva Filipe, Natasha Johnson, Kathy Smollett, Daniel Mair, Stephen Carmichael, Alice Broos, Lily Tong, Jenna Nichols, Kyriaki Nomikou; Sarah McDonald; Richard Orton, Joseph Hughes, Sreenu Vattipally, David L Robertson; Alasdair MacLean, Rory Gunson; Sharif Shaaban, Matthew Holden; Rachel Blacow, Guy Mollett, Kathy Li, James Shepherd, Antonia Ho, Emma Thomson
EPI_ISL_680461, EPI_ISL_680462, EPI_ISL_680463, EPI_ISL_680464, EPI_ISL_680465, EPI_ISL_680466, EPI_ISL_680467, EPI_ISL_680468, EPI_ISL_680469, EPI_ISL_680470, EPI_ISL_680471, EPI_ISL_680472, EPI_ISL_680473, EPI_ISL_680474, EPI_ISL_680475, EPI_ISL_680476, EPI_ISL_680477, EPI_ISL_680478, EPI_ISL_680480, EPI_ISL_680481, EPI_ISL_680482, EPI_ISL_680483, EPI_ISL_680484, EPI_ISL_680485, EPI_ISL_680486, EPI_ISL_680487, EPI_ISL_680488, EPI_ISL_680489, EPI_ISL_680490, EPI_ISL_680491, EPI_ISL_680492, EPI_ISL_680493, EPI_ISL_680494, EPI_ISL_680495, EPI_ISL_680496, EPI_ISL_680497, EPI_ISL_680498, EPI_ISL_680499, EPI_ISL_680500, EPI_ISL_680501, EPI_ISL_680502, EPI_ISL_680506, EPI_ISL_680507, EPI_ISL_680508, EPI_ISL_680509, EPI_ISL_680510, EPI_ISL_680511, EPI_ISL_680512, EPI_ISL_680513, EPI_ISL_680514, EPI_ISL_680515, EPI_ISL_680516, EPI_ISL_680517, EPI_ISL_680518, EPI_ISL_680519, EPI_ISL_680520, EPI_ISL_680521, EPI_ISL_680522, EPI_ISL_680523, EPI_ISL_680524, EPI_ISL_680525, EPI_ISL_680526, EPI_ISL_680527, EPI_ISL_680528, EPI_ISL_680529, EPI_ISL_680530, EPI_ISL_680531, EPI_ISL_680532, EPI_ISL_680533, EPI_ISL_680534, EPI_ISL_680535, EPI_ISL_680536, EPI_ISL_680537, EPI_ISL_680538, EPI_ISL_680539, EPI_ISL_680540, EPI_ISL_680541, EPI_ISL_680542, EPI_ISL_680543, EPI_ISL_680544, EPI_ISL_680545, EPI_ISL_680546, EPI_ISL_680547, EPI_ISL_680548, EPI_ISL_680549, EPI_ISL_680550, EPI_ISL_680551, EPI_ISL_680552, EPI_ISL_680553, EPI_ISL_680554, EPI_ISL_680555			
see above	Virology Department, Royal Infirmary of Edinburgh, NHS Lothian / School of Biological Sciences, University of Edinburgh / Institute of Genetics and Molecular Medicine, University of Edinburgh	COVID-19 Genomics UK (COG-UK) Consortium	McHugh M, Dewar R, Rooke S, Gallagher M, Balcaza C, O'Toole Á, Scher E, Hill V, McCrone JT, Colquhoun R, Yu X, Jackson B, Rambaut A, Williams TC, Templeton K
EPI_ISL_680557, EPI_ISL_680558, EPI_ISL_680559, EPI_ISL_680560, EPI_ISL_680561	Virology Department, Sheffield Teaching Hospitals NHS Foundation Trust/Department of Infection, Immunity and Cardiovascular Disease, The Medical School, University of Sheffield	COVID-19 Genomics UK (COG-UK) Consortium	Thushan de Silva, Matthew Parker, Nikki Smith, Adri Angyal, Rebecca Brown, Luke Green, Rachel Tucker, Paul Parsons, Danielle Groves, Katie Johnson, Laura Carrilero, Alex Keeley, Dave Partridge, Matthew Wyles, Benjamin Lindsey, Mehmet Yavuz, Mohammad Raza, Cariad Evans
EPI_ISL_680562, EPI_ISL_680564, EPI_ISL_680565, EPI_ISL_680566, EPI_ISL_680568, EPI_ISL_680569	Centre for Enzyme Innovation, University of Portsmouth / Translational Research Laboratory, Portsmouth Hospitals NHS Trust	COVID-19 Genomics UK (COG-UK) Consortium	Angela Beckett,Yann Bourgeois,Garry Scarlett,Sharon Glaysher,Scott Elliott,Kelly Bicknell,Robert Impey,Allyson Lloyd,Sarah Wyllie,Ethan Butcher,Anoop Chauhan,Samuel Robson
EPI_ISL_680571, EPI_ISL_680572, EPI_ISL_680573, EPI_ISL_680574, EPI_ISL_680575, EPI_ISL_680576, EPI_ISL_680577	Department of Pathology, University of Cambridge	COVID-19 Genomics UK (COG-UK) Consortium	Aminu S. Jahun, Yasmin Chaudhry, Grant Hall, Iliana Georgana, Myra Hosmillo, Martin D. Curran, Malte Pinckert, Surendra Parmar, Ian Goodfellow
EPI_ISL_680578	Centre for Enzyme Innovation, University of Portsmouth / Translational Research Laboratory, Portsmouth Hospitals NHS Trust	COVID-19 Genomics UK (COG-UK) Consortium	Angela Beckett,Yann Bourgeois,Garry Scarlett,Sharon Glaysher,Scott Elliott,Kelly Bicknell,Robert Impey,Allyson Lloyd,Sarah Wyllie,Ethan Butcher,Anoop Chauhan,Samuel Robson
EPI_ISL_680579	Oxford Viromics, NDM, University of Oxford; Oxford University Hospitals; Basingstoke and North Hampshire Hospital	COVID-19 Genomics UK (COG-UK) Consortium	Tanya Golubchik, David Bonsall, George Macintyre, Amy Trebes, Mariateresa de Cesare, Catrin Moore, Alex Mobbs, Anita Justice, Robert Shaw, Monique Andersson, Timothy Peto, Emma Wise, Nathan Moore, Jessica Lynch, Nick Cortes, Matilde Mori, Stephen Kidd, David Buck, John Todd, Christophe Fraser
EPI_ISL_680580, EPI_ISL_680581, EPI_ISL_680582, EPI_ISL_680583, EPI_ISL_680584, EPI_ISL_680585, EPI_ISL_680586, EPI_ISL_680587, EPI_ISL_680588, EPI_ISL_680589, EPI_ISL_680590, EPI_ISL_680591, EPI_ISL_680592, EPI_ISL_680593, EPI_ISL_680594, EPI_ISL_680595, EPI_ISL_680596, EPI_ISL_680597, EPI_ISL_680598, EPI_ISL_680599, EPI_ISL_680600, EPI_ISL_680601, EPI_ISL_680602, EPI_ISL_680603, EPI_ISL_680604, EPI_ISL_680605, EPI_ISL_680606, EPI_ISL_680607, EPI_ISL_680608, EPI_ISL_680609, EPI_ISL_680610, EPI_ISL_680611, EPI_ISL_680612, EPI_ISL_680613, EPI_ISL_680614, EPI_ISL_680615, EPI_ISL_680616, EPI_ISL_680617, EPI_ISL_680618, EPI_ISL_680619, EPI_ISL_680620, EPI_ISL_680621, EPI_ISL_680622, EPI_ISL_680623, EPI_ISL_680624, EPI_ISL_680625, EPI_ISL_680627, EPI_ISL_680628, EPI_ISL_680629, EPI_ISL_680630, EPI_ISL_680631, EPI_ISL_680632, EPI_ISL_680633, EPI_ISL_680634, EPI_ISL_680635, EPI_ISL_680636, EPI_ISL_680637, EPI_ISL_680639, EPI_ISL_680640, EPI_ISL_680641, EPI_ISL_680642, EPI_ISL_680643, EPI_ISL_680644, EPI_ISL_680645, EPI_ISL_680647, EPI_ISL_680648, EPI_ISL_680649, EPI_ISL_680650, EPI_ISL_680651, EPI_ISL_680652, EPI_ISL_680653, EPI_ISL_680654, EPI_ISL_680655, EPI_ISL_680656, EPI_ISL_680657, EPI_ISL_680658, EPI_ISL_680659, EPI_ISL_680660, EPI_ISL_680662, EPI_ISL_680664, EPI_ISL_680665, EPI_ISL_680666, EPI_ISL_680667, EPI_ISL_680668, EPI_ISL_680669, EPI_ISL_680670, EPI_ISL_680671, EPI_ISL_680672, EPI_ISL_680673, EPI_ISL_680675, EPI_ISL_680676, EPI_ISL_680677, EPI_ISL_680678, EPI_ISL_680679, EPI_ISL_680680, EPI_ISL_680681, EPI_ISL_680682, EPI_ISL_680683, EPI_ISL_680684, EPI_ISL_680685, EPI_ISL_680686, EPI_ISL_680687, EPI_ISL_680688, EPI_ISL_680689, EPI_ISL_680690, EPI_ISL_680691, EPI_ISL_680692, EPI_ISL_680693, EPI_ISL_680694, EPI_ISL_680695, EPI_ISL_680696, EPI_ISL_680697, EPI_ISL_680698, EPI_ISL_680699, EPI_ISL_680700, EPI_ISL_680701, EPI_ISL_680702, EPI_ISL_680703, EPI_ISL_680704, EPI_ISL_680705, EPI_ISL_680706, EPI_ISL_680707, EPI_ISL_680708, EPI_ISL_680709, EPI_ISL_680710, EPI_ISL_680711, EPI_ISL_680712, EPI_ISL_680713, EPI_ISL_680714, EPI_ISL_680715, EPI_ISL_680716, EPI_ISL_680717, EPI_ISL_680718, EPI_ISL_680719, EPI_ISL_680720, EPI_ISL_680721, EPI_ISL_680722, EPI_ISL_680723, EPI_ISL_680724, EPI_ISL_680725, EPI_ISL_680726, EPI_ISL_680727, EPI_ISL_680728, EPI_ISL_680729, EPI_ISL_680730, EPI_ISL_680731, EPI_ISL_680732, EPI_ISL_680733, EPI_ISL_680734, EPI_ISL_680735, EPI_ISL_680736, EPI_ISL_680737, EPI_ISL_680738, EPI_ISL_680739, EPI_ISL_680740, EPI_ISL_680741, EPI_ISL_680742, EPI_ISL_680743, EPI_ISL_680745, EPI_ISL_680746, EPI_ISL_680747, EPI_ISL_680748, EPI_ISL_680749, EPI_ISL_680750, EPI_ISL_680751, EPI_ISL_680752, EPI_ISL_680753, EPI_ISL_680754, EPI_ISL_680755, EPI_ISL_680756, EPI_ISL_680757, EPI_ISL_680758, EPI_ISL_680759, EPI_ISL_680760, EPI_ISL_680761, EPI_ISL_680762, EPI_ISL_680763, EPI_ISL_680765, EPI_ISL_680766, EPI_ISL_680767, EPI_ISL_680768, EPI_ISL_680769, EPI_ISL_680770, EPI_ISL_680771, EPI_ISL_680772, EPI_ISL_680773, EPI_ISL_680774, EPI_ISL_680775, EPI_ISL_680776, EPI_ISL_680777, EPI_ISL_680778, EPI_ISL_680779, EPI_ISL_680780, EPI_ISL_680781, EPI_ISL_680782, EPI_ISL_680783, EPI_ISL_680784, EPI_ISL_680785, EPI_ISL_680786, EPI_ISL_680787, EPI_ISL_680788, EPI_ISL_680789, EPI_ISL_680790, EPI_ISL_680791, EPI_ISL_680792, EPI_ISL_680793, EPI_ISL_680794, EPI_ISL_680795, EPI_ISL_680796, EPI_ISL_680797, EPI_ISL_680798, EPI_ISL_680799, EPI_ISL_680800, EPI_ISL_680801, EPI_ISL_680802, EPI_ISL_680803, EPI_ISL_680804, EPI_ISL_680805, EPI_ISL_680806, EPI_ISL_680807, EPI_ISL_680809, EPI_ISL_680810, EPI_ISL_680811, EPI_ISL_680812, EPI_ISL_680813, EPI_ISL_680814, EPI_ISL_680815, EPI_ISL_680816, EPI_ISL_680817, EPI_ISL_680818, EPI_ISL_680819, EPI_ISL_680820, EPI_ISL_680821, EPI_ISL_680822, EPI_ISL_680823, EPI_ISL_680824, EPI_ISL_680825, EPI_ISL_680826, EPI_ISL_680827, EPI_ISL_680829, EPI_ISL_680830, EPI_ISL_680831, EPI_ISL_680832, EPI_ISL_680833, EPI_ISL_680834, EPI_ISL_680835, EPI_ISL_680836, EPI_ISL_680838, EPI_ISL_680839, EPI_ISL_680840, EPI_ISL_680841, EPI_ISL_680842, EPI_ISL_680843, EPI_ISL_680844, EPI_ISL_680845, EPI_ISL_680846, EPI_ISL_680847, EPI_ISL_680848, EPI_ISL_680849, EPI_ISL_680850, EPI_ISL_680851, EPI_ISL_680852, EPI_ISL_680853, EPI_ISL_680854, EPI_ISL_680855, EPI_ISL_680856, EPI_ISL_680857, EPI_ISL_680858, EPI_ISL_680859, EPI_ISL_680860, EPI_ISL_680861, EPI_ISL_680862, EPI_ISL_680863, EPI_ISL_680864, EPI_ISL_680866, EPI_ISL_680867, EPI_ISL_680868, EPI_ISL_680869, EPI_ISL_680870, EPI_ISL_680871, EPI_ISL_680872, EPI_ISL_680873, EPI_ISL_680874, EPI_ISL_680875, EPI_ISL_680876, EPI_ISL_680877, EPI_ISL_680878, EPI_ISL_680879, EPI_ISL_680880, EPI_ISL_680881, EPI_ISL_680882, EPI_ISL_680883, EPI_ISL_680884, EPI_ISL_680885, EPI_ISL_680886, EPI_ISL_680887, EPI_ISL_680888, EPI_ISL_680889, EPI_ISL_680890, EPI_ISL_680891, EPI_ISL_680892, EPI_ISL_680893, EPI_ISL_680894, EPI_ISL_680895, EPI_ISL_680896, EPI_ISL_680897, EPI_ISL_680898, EPI_ISL_680899, EPI_ISL_680900, EPI_ISL_680901, EPI_ISL_680902, EPI_ISL_680903, EPI_ISL_680904, EPI_ISL_680905, EPI_ISL_680906, EPI_ISL_680907, EPI_ISL_680908, EPI_ISL_680909, EPI_ISL_680910, EPI_ISL_680911, EPI_ISL_680912, EPI_ISL_680913, EPI_ISL_680914, EPI_ISL_680915, EPI_ISL_680916, EPI_ISL_680917, EPI_ISL_680919, EPI_ISL_680920, EPI_ISL_680921, EPI_ISL_680922, EPI_ISL_680923, EPI_ISL_680924, EPI_ISL_680925, EPI_ISL_680926, EPI_ISL_680927, EPI_ISL_680928, EPI_ISL_680929, EPI_ISL_680930, EPI_ISL_680931, EPI_ISL_680932, EPI_ISL_680933, EPI_ISL_680935, EPI_ISL_680936, EPI_ISL_680937, EPI_ISL_680938, EPI_ISL_680939, EPI_ISL_680940, EPI_ISL_680941, EPI_ISL_680942, EPI_ISL_680943, EPI_ISL_680944, EPI_ISL_680945, EPI_ISL_680946, EPI_ISL_680947, EPI_ISL_680948, EPI_ISL_680949, EPI_ISL_680950, EPI_ISL_680951, EPI_ISL_680952, EPI_ISL_680953, EPI_ISL_680954, EPI_ISL_680955, EPI_ISL_680956, EPI_ISL_680957, EPI_ISL_680958, EPI_ISL_680959, EPI_ISL_680960, EPI_ISL_680961, EPI_ISL_680962, EPI_ISL_680963, EPI_ISL_680964, EPI_ISL_680965, EPI_ISL_680966, EPI_ISL_680967, EPI_ISL_680968, EPI_ISL_680969, EPI_ISL_680970, EPI_ISL_680971, EPI_ISL_680972, EPI_ISL_680973, EPI_ISL_680974, EPI_ISL_680975, EPI_ISL_680976, EPI_ISL_680977, EPI_ISL_680978, EPI_ISL_680979, EPI_ISL_680980, EPI_ISL_680981, EPI_ISL_680982, EPI_ISL_680983, EPI_ISL_680984, EPI_ISL_680985, EPI_ISL_680986, EPI_ISL_680987, EPI_ISL_680988, EPI_ISL_680989, EPI_ISL_680990, EPI_ISL_680991, EPI_ISL_680992, EPI_ISL_680993, EPI_ISL_680994, EPI_ISL_680995, EPI_ISL_680996, EPI_ISL_680997, EPI_ISL_680998, EPI_ISL_680999, EPI_ISL_681000, EPI_ISL_681001, EPI_ISL_681002, EPI_ISL_681003, EPI_ISL_681004, EPI_ISL_681005, EPI_ISL_681006, EPI_ISL_681007, EPI_ISL_681008, EPI_ISL_681009, EPI_ISL_681010, EPI_ISL_681011, EPI_ISL_681012, EPI_ISL_681013, EPI_ISL_681014, EPI_ISL_681015, EPI_ISL_681016, EPI_ISL_681017, EPI_ISL_681018, EPI_ISL_681019, EPI_ISL_681020, EPI_ISL_681021, EPI_ISL_681022, EPI_ISL_681023, EPI_ISL_681025, EPI_ISL_681026, EPI_ISL_681027, EPI_ISL_681028, EPI_ISL_681029, EPI_ISL_681030, EPI_ISL_681031, EPI_ISL_681032, EPI_ISL_681033, EPI_ISL_681034, EPI_ISL_681035, EPI_ISL_681036, EPI_ISL_681037, EPI_ISL_681038, EPI_ISL_681039, EPI_ISL_681040, EPI_ISL_681041, EPI_ISL_681042, EPI_ISL_681043, EPI_ISL_681044, EPI_ISL_681045, EPI_ISL_681046, EPI_ISL_681047, EPI_ISL_681048, EPI_ISL_681049, EPI_ISL_681050, EPI_ISL_681051, EPI_ISL_681052, EPI_ISL_681053, EPI_ISL_681054, EPI_ISL_681055, EPI_ISL_681056, EPI_ISL_681057, EPI_ISL_681058, EPI_ISL_681059, EPI_ISL_681060, EPI_ISL_681061, EPI_ISL_681062, EPI_ISL_681063, EPI_ISL_681064, EPI_ISL_681065, EPI_ISL_681066, EPI_ISL_681068, EPI_ISL_681069, EPI_ISL_681070, EPI_ISL_681071, EPI_ISL_681072, EPI_ISL_681073, EPI_ISL_681074, EPI_ISL_681075, EPI_ISL_681076, EPI_ISL_681078, EPI_ISL_681079, EPI_ISL_681080, EPI_ISL_681081, EPI_ISL_681082, EPI_ISL_681083, EPI_ISL_681084, EPI_ISL_681085, EPI_ISL_681086, EPI_ISL_681087, EPI_ISL_681088, EPI_ISL_681089, EPI_ISL_681090, EPI_ISL_681091, EPI_ISL_681093, EPI_ISL_681094, EPI_ISL_681095, EPI_ISL_681096, EPI_ISL_681097, EPI_ISL_681098, EPI_ISL_681099, EPI_ISL_681100, EPI_ISL_681101, EPI_ISL_681102, EPI_ISL_681103, EPI_ISL_681104, EPI_ISL_681105, EPI_ISL_681106, EPI_ISL_681107, EPI_ISL_681108, EPI_ISL_681109, EPI_ISL_681111, EPI_ISL_681112, EPI_ISL_681113, EPI_ISL_681114, EPI_ISL_681115, EPI_ISL_681116, EPI_ISL_681117, EPI_ISL_681118, EPI_ISL_681119, EPI_ISL_681120, EPI_ISL_681121, EPI_ISL_681122, EPI_ISL_681123, EPI_ISL_681124, EPI_ISL_681125, EPI_ISL_681126, EPI_ISL_681127, EPI_ISL_681128, EPI_ISL_681129, EPI_ISL_681130, EPI_ISL_681132, EPI_ISL_681133, EPI_ISL_681134, EPI_ISL_681135, EPI_ISL_681136, EPI_ISL_681137, EPI_ISL_681138, EPI_ISL_681139, EPI_ISL_681140, EPI_ISL_681141, EPI_ISL_681142, EPI_ISL_681143, EPI_ISL_681144, EPI_ISL_681146, EPI_ISL_681147, EPI_ISL_681148, EPI_ISL_681149, EPI_ISL_681150, EPI_ISL_681151, EPI_ISL_681152, EPI_ISL_681153, EPI_ISL_681154, EPI_ISL_681155, EPI_ISL_681156, EPI_ISL_681157, EPI_ISL_681158, EPI_ISL_681159, EPI_ISL_681160, EPI_ISL_681161, EPI_ISL_681163, EPI_ISL_681164, EPI_ISL_681165, EPI_ISL_681166, EPI_ISL_681167, EPI_ISL_681168, EPI_ISL_681169, EPI_ISL_681170, EPI_ISL_681171, EPI_ISL_681172, EPI_ISL_681173, EPI_ISL_681174, EPI_ISL_681175, EPI_ISL_681176, EPI_ISL_681177, EPI_ISL_681179, EPI_ISL_681180, EPI_ISL_681181, EPI_ISL_681182, EPI_ISL_681184, EPI_ISL_681185, EPI_ISL_681186, EPI_ISL_681187, EPI_ISL_681188, EPI_ISL_681189, EPI_ISL_681190, EPI_ISL_681191, EPI_ISL_681192, EPI_ISL_681193, EPI_ISL_681194, EPI_ISL_681195, EPI_ISL_681196, EPI_ISL_681197, EPI_ISL_681198, EPI_ISL_681199, EPI_ISL_681200, EPI_ISL_681201, EPI_ISL_681202, EPI_ISL_681203, EPI_ISL_681204, EPI_ISL_681205, EPI_ISL_681206, EPI_ISL_681208, EPI_ISL_681209, EPI_ISL_681210, EPI_ISL_681211, EPI_ISL_681212, EPI_ISL_681213, EPI_ISL_681214, EPI_ISL_681215, EPI_ISL_681216,			

EPI_ISL_681217, EPI_ISL_681218, EPI_ISL_681219, EPI_ISL_681220, EPI_ISL_681221, EPI_ISL_681222, EPI_ISL_681223, EPI_ISL_681224, EPI_ISL_681225, EPI_ISL_681226, EPI_ISL_681227, EPI_ISL_681228, EPI_ISL_681229, EPI_ISL_681230, EPI_ISL_681231, EPI_ISL_681232, EPI_ISL_681233, EPI_ISL_681234, EPI_ISL_681235, EPI_ISL_681236, EPI_ISL_681237, EPI_ISL_681238, EPI_ISL_681239, EPI_ISL_681240, EPI_ISL_681241, EPI_ISL_681242, EPI_ISL_681243, EPI_ISL_681244, EPI_ISL_681245, EPI_ISL_681246, EPI_ISL_681247, EPI_ISL_681248, EPI_ISL_681249, EPI_ISL_681250, EPI_ISL_681251, EPI_ISL_681252, EPI_ISL_681253, EPI_ISL_681254, EPI_ISL_681255, EPI_ISL_681256, EPI_ISL_681257, EPI_ISL_681258, EPI_ISL_681259			
see above	Wales Specialist Virology Centre Sequencing lab: Pathogen Genomics Unit	COVID-19 Genomics UK (COG-UK) Consortium	Catherine Moore, Johnathan Evans, Laura Gifford, Malorie Perry, Simon Cottrell, Angela Marchbank, Alec Birchley, Alexander Adams, Amy Gaskin, Bree Gatica-Wilcox, Jason Coombes, Joel Southgate, Lauren Gilbert, Lee Graham, Nicole Pacchiarini, Sara Kumziene-Summerhayes, Sarah Taylor, Sophie Jones, Sara Rey, Matthew Bull, Joanne Watkins, Sally Corden, Tom Connor
EPI_ISL_681260	Department of Pathology, University of Cambridge	COVID-19 Genomics UK (COG-UK) Consortium	Aminu S. Jahun, Yasmin Chaudhry, Grant Hall, Iliana Georgana, Myra Hosmillo, Martin D. Curran, Malte Pinckert, Surendra Parmar, Ian Goodfellow
EPI_ISL_681264, EPI_ISL_681265, EPI_ISL_681266, EPI_ISL_681268, EPI_ISL_681269, EPI_ISL_681270	Department of Clinical Microbiology	GIGA Medical Genomics	Keith Durkin, Maria Artesi, Sébastien Bontems, Raphaël Boreux, Bouchra Boujemla, Cécile Meex, Pierrette Melin, Marie-Pierre Hayette, Vincent Bours
EPI_ISL_681298, EPI_ISL_681299, EPI_ISL_681300, EPI_ISL_681301, EPI_ISL_681302, EPI_ISL_681303, EPI_ISL_681304, EPI_ISL_681305, EPI_ISL_681306, EPI_ISL_681307, EPI_ISL_681308, EPI_ISL_681309, EPI_ISL_681310, EPI_ISL_681311, EPI_ISL_681312, EPI_ISL_681313, EPI_ISL_681314, EPI_ISL_681315, EPI_ISL_681316, EPI_ISL_681317, EPI_ISL_681318, EPI_ISL_681319			
see above	Communicable Disease Laboratory, Public Health Directorate	Communicable Disease Laboratory, Public Health Directorate	Alwasti,H., Altaif,Z., AlHujairi,Z., AlAbbas,Z.
EPI_ISL_681320, EPI_ISL_681321	Pathology and Laboratory Medicine, UW-Madison	Pathology and Laboratory Medicine, UW-Madison	Moreno,G., Braun,K., Baczenas,J.J., Baker,D.
EPI_ISL_681322	Environmental and Global Health, University of Florida	Environmental and Global Health, University of Florida	Loeb,J.C., Stephenson,C.J., Merck,L., Morris,J.G. and Lednicky,J.A.
EPI_ISL_681325, EPI_ISL_681326, EPI_ISL_681327, EPI_ISL_681328, EPI_ISL_681329, EPI_ISL_681330, EPI_ISL_681331, EPI_ISL_681332, EPI_ISL_681333, EPI_ISL_681334, EPI_ISL_681335, EPI_ISL_681336, EPI_ISL_681337, EPI_ISL_681338, EPI_ISL_681339, EPI_ISL_681340, EPI_ISL_681341, EPI_ISL_681342, EPI_ISL_681343, EPI_ISL_681344, EPI_ISL_681346, EPI_ISL_681347, EPI_ISL_681348, EPI_ISL_681349, EPI_ISL_681350, EPI_ISL_681351, EPI_ISL_681352, EPI_ISL_681353, EPI_ISL_681354, EPI_ISL_681355, EPI_ISL_681356, EPI_ISL_681357, EPI_ISL_681358, EPI_ISL_681359, EPI_ISL_681360, EPI_ISL_681361, EPI_ISL_681362, EPI_ISL_681363, EPI_ISL_681364, EPI_ISL_681365, EPI_ISL_681366, EPI_ISL_681367, EPI_ISL_681368, EPI_ISL_681369, EPI_ISL_681370, EPI_ISL_681371, EPI_ISL_681372, EPI_ISL_681373, EPI_ISL_681374, EPI_ISL_681375, EPI_ISL_681376, EPI_ISL_681377, EPI_ISL_681378, EPI_ISL_681379, EPI_ISL_681380, EPI_ISL_681381, EPI_ISL_681382, EPI_ISL_681383, EPI_ISL_681384, EPI_ISL_681385, EPI_ISL_681386, EPI_ISL_681387, EPI_ISL_681389, EPI_ISL_681390, EPI_ISL_681391, EPI_ISL_681392, EPI_ISL_681393, EPI_ISL_681394, EPI_ISL_681395, EPI_ISL_681396, EPI_ISL_681397, EPI_ISL_681398, EPI_ISL_681399, EPI_ISL_681400, EPI_ISL_681401, EPI_ISL_681402, EPI_ISL_681403, EPI_ISL_681404, EPI_ISL_681405, EPI_ISL_681406, EPI_ISL_681407, EPI_ISL_681408, EPI_ISL_681409, EPI_ISL_681410, EPI_ISL_681411, EPI_ISL_681412, EPI_ISL_681413, EPI_ISL_681414, EPI_ISL_681415, EPI_ISL_681416, EPI_ISL_681417, EPI_ISL_681418, EPI_ISL_681419, EPI_ISL_681420, EPI_ISL_681421, EPI_ISL_681422, EPI_ISL_681423, EPI_ISL_681424, EPI_ISL_681425, EPI_ISL_681426, EPI_ISL_681427, EPI_ISL_681428, EPI_ISL_681429, EPI_ISL_681430, EPI_ISL_681431, EPI_ISL_681432, EPI_ISL_681433, EPI_ISL_681434, EPI_ISL_681435, EPI_ISL_681436, EPI_ISL_681437, EPI_ISL_681438, EPI_ISL_681440, EPI_ISL_681441, EPI_ISL_681442, EPI_ISL_681443, EPI_ISL_681444, EPI_ISL_681445, EPI_ISL_681446, EPI_ISL_681447, EPI_ISL_681448, EPI_ISL_681449, EPI_ISL_681450, EPI_ISL_681451, EPI_ISL_681452, EPI_ISL_681453, EPI_ISL_681454, EPI_ISL_681455, EPI_ISL_681456, EPI_ISL_681457, EPI_ISL_681459, EPI_ISL_681460, EPI_ISL_681461, EPI_ISL_681462, EPI_ISL_681464, EPI_ISL_681465, EPI_ISL_681467, EPI_ISL_681468, EPI_ISL_681469, EPI_ISL_681470, EPI_ISL_681471, EPI_ISL_681472, EPI_ISL_681473, EPI_ISL_681474, EPI_ISL_681475, EPI_ISL_681476, EPI_ISL_681477, EPI_ISL_681478, EPI_ISL_681479, EPI_ISL_681480, EPI_ISL_681481, EPI_ISL_681482, EPI_ISL_681483, EPI_ISL_681484, EPI_ISL_681485, EPI_ISL_681486, EPI_ISL_681487, EPI_ISL_681488, EPI_ISL_681489, EPI_ISL_681490, EPI_ISL_681491, EPI_ISL_681492, EPI_ISL_681493, EPI_ISL_681494, EPI_ISL_681495, EPI_ISL_681496, EPI_ISL_681497, EPI_ISL_681498, EPI_ISL_681499, EPI_ISL_681500, EPI_ISL_681501, EPI_ISL_681502, EPI_ISL_681503, EPI_ISL_681504, EPI_ISL_681505, EPI_ISL_681506, EPI_ISL_681507, EPI_ISL_681508, EPI_ISL_681509, EPI_ISL_681510, EPI_ISL_681511, EPI_ISL_681512, EPI_ISL_681514, EPI_ISL_681515, EPI_ISL_681516, EPI_ISL_681517, EPI_ISL_681518, EPI_ISL_681519, EPI_ISL_681520, EPI_ISL_681521, EPI_ISL_681522, EPI_ISL_681523, EPI_ISL_681524, EPI_ISL_681525, EPI_ISL_681526, EPI_ISL_681527, EPI_ISL_681528, EPI_ISL_681529, EPI_ISL_681530, EPI_ISL_681531, EPI_ISL_681532, EPI_ISL_681533, EPI_ISL_681534, EPI_ISL_681535, EPI_ISL_681536, EPI_ISL_681537, EPI_ISL_681538, EPI_ISL_681539, EPI_ISL_681540, EPI_ISL_681541, EPI_ISL_681542, EPI_ISL_681543, EPI_ISL_681544, EPI_ISL_681545, EPI_ISL_681546, EPI_ISL_681548, EPI_ISL_681549, EPI_ISL_681550, EPI_ISL_681551, EPI_ISL_681552, EPI_ISL_681553, EPI_ISL_681554, EPI_ISL_681555, EPI_ISL_681556, EPI_ISL_681557, EPI_ISL_681558, EPI_ISL_681559, EPI_ISL_681560, EPI_ISL_681561, EPI_ISL_681562, EPI_ISL_681563, EPI_ISL_681564, EPI_ISL_681565, EPI_ISL_681566, EPI_ISL_681567, EPI_ISL_681568, EPI_ISL_681569, EPI_ISL_681570, EPI_ISL_681571, EPI_ISL_681572, EPI_ISL_681573, EPI_ISL_681574, EPI_ISL_681575, EPI_ISL_681576, EPI_ISL_681577, EPI_ISL_681578, EPI_ISL_681579, EPI_ISL_681580, EPI_ISL_681581, EPI_ISL_681582, EPI_ISL_681583, EPI_ISL_681584, EPI_ISL_681586, EPI_ISL_681587, EPI_ISL_681588, EPI_ISL_681589, EPI_ISL_681590, EPI_ISL_681591, EPI_ISL_681592, EPI_ISL_681593, EPI_ISL_681594, EPI_ISL_681595, EPI_ISL_681596, EPI_ISL_681597, EPI_ISL_681598, EPI_ISL_681600, EPI_ISL_681601, EPI_ISL_681602, EPI_ISL_681603, EPI_ISL_681604, EPI_ISL_681605, EPI_ISL_681606, EPI_ISL_681607, EPI_ISL_681608, EPI_ISL_681609, EPI_ISL_681610, EPI_ISL_681611, EPI_ISL_681612, EPI_ISL_681613, EPI_ISL_681614, EPI_ISL_681615, EPI_ISL_681616, EPI_ISL_681617, EPI_ISL_681618, EPI_ISL_681619, EPI_ISL_681620, EPI_ISL_681621, EPI_ISL_681622, EPI_ISL_681623, EPI_ISL_681624, EPI_ISL_681625, EPI_ISL_681626, EPI_ISL_681627, EPI_ISL_681628, EPI_ISL_681629, EPI_ISL_681630, EPI_ISL_681631, EPI_ISL_681632, EPI_ISL_681633, EPI_ISL_681634, EPI_ISL_681635, EPI_ISL_681636, EPI_ISL_681637, EPI_ISL_681638, EPI_ISL_681639, EPI_ISL_681640, EPI_ISL_681641, EPI_ISL_681642, EPI_ISL_681644, EPI_ISL_681645, EPI_ISL_681646, EPI_ISL_681647, EPI_ISL_681648, EPI_ISL_681649, EPI_ISL_681650, EPI_ISL_681651, EPI_ISL_681652, EPI_ISL_681653, EPI_ISL_681654, EPI_ISL_681655			
see above	Lighthouse Lab in Milton Keynes	Wellcome Sanger Institute for the COVID-19 Genomics UK (COG-UK) Consortium	The Lighthouse Lab in Milton Keynes and Alex Alderton, Roberto Amato, Sonia Goncalves, Ewan Harrison, David K. Jackson, Ian Johnston, Dominic Kwiatkowski, Cordelia Langford, John Sillitoe on behalf of the Wellcome Sanger Institute COVID-19 Surveillance Team
EPI_ISL_681656, EPI_ISL_681657, EPI_ISL_681658, EPI_ISL_681659	Lighthouse Lab in Cambridge	Wellcome Sanger Institute for the COVID-19 Genomics UK (COG-UK) Consortium	Rob Howes, The Lighthouse Lab in Cambridge and Alex Alderton, Roberto Amato, Sonia Goncalves, Ewan Harrison, David K. Jackson, Ian Johnston, Dominic Kwiatkowski, Cordelia Langford, John Sillitoe on behalf of the Wellcome Sanger Institute COVID-19 Surveillance Team
EPI_ISL_681660	Lighthouse Lab in Milton Keynes	Wellcome Sanger Institute for the COVID-19 Genomics UK (COG-UK) Consortium	The Lighthouse Lab in Milton Keynes and Alex Alderton, Roberto Amato, Sonia Goncalves, Ewan Harrison, David K. Jackson, Ian Johnston, Dominic Kwiatkowski, Cordelia Langford, John Sillitoe on behalf of the Wellcome Sanger Institute COVID-19 Surveillance Team
EPI_ISL_681661, EPI_ISL_681662	Lighthouse Lab in Alderley Park	Wellcome Sanger Institute for the COVID-19 Genomics UK (COG-UK) Consortium	Jacquelyn Wynn, Mairead Hyland, The Lighthouse Lab in Alderley Park and Alex Alderton, Roberto Amato, Sonia Goncalves, Ewan Harrison, David K. Jackson, Ian Johnston, Dominic Kwiatkowski, Cordelia Langford, John Sillitoe on behalf of the Wellcome Sanger Institute COVID-19 Surveillance Team
EPI_ISL_681664	Lighthouse Lab in Milton Keynes	Wellcome Sanger Institute for the COVID-19 Genomics UK (COG-UK) Consortium	The Lighthouse Lab in Milton Keynes and Alex Alderton, Roberto Amato, Sonia Goncalves, Ewan Harrison, David K. Jackson, Ian Johnston, Dominic Kwiatkowski, Cordelia Langford, John Sillitoe on behalf of the Wellcome Sanger Institute COVID-19 Surveillance Team
EPI_ISL_681665	Lighthouse Lab in Alderley Park	Wellcome Sanger Institute for the COVID-19 Genomics UK (COG-UK) Consortium	Jacquelyn Wynn, Mairead Hyland, The Lighthouse Lab in Alderley Park and Alex Alderton, Roberto Amato, Sonia Goncalves, Ewan Harrison, David K. Jackson, Ian Johnston, Dominic Kwiatkowski, Cordelia Langford, John Sillitoe on behalf of the Wellcome Sanger Institute COVID-19 Surveillance Team
EPI_ISL_681666, EPI_ISL_681667, EPI_ISL_681668, EPI_ISL_681669, EPI_ISL_681670	Lighthouse Lab in Milton Keynes	Wellcome Sanger Institute for the COVID-19 Genomics UK (COG-UK) Consortium	The Lighthouse Lab in Milton Keynes and Alex Alderton, Roberto Amato, Sonia Goncalves, Ewan Harrison, David K. Jackson, Ian Johnston, Dominic Kwiatkowski, Cordelia Langford, John Sillitoe on behalf of the Wellcome Sanger Institute COVID-19 Surveillance Team
EPI_ISL_681671, EPI_ISL_681672	Lighthouse Lab in Cambridge	Wellcome Sanger Institute for the COVID-19 Genomics UK (COG-UK) Consortium	Rob Howes, The Lighthouse Lab in Cambridge and Alex Alderton, Roberto Amato, Sonia Goncalves, Ewan Harrison, David K. Jackson, Ian Johnston, Dominic Kwiatkowski, Cordelia Langford, John Sillitoe on behalf of the Wellcome Sanger Institute COVID-19 Surveillance Team
EPI_ISL_681685, EPI_ISL_681686, EPI_ISL_681687, EPI_ISL_681688, EPI_ISL_681689, EPI_ISL_681690, EPI_ISL_681691, EPI_ISL_681692, EPI_ISL_681693, EPI_ISL_681694, EPI_ISL_681695, EPI_ISL_681696, EPI_ISL_681697, EPI_ISL_681698, EPI_ISL_681699, EPI_ISL_681700, EPI_ISL_681701			
see above	Molecular Medicine Laboratory, University of Magallanes	Centro Asistencial Docente y de Investigacion, Universidad de Magallanes	Jorge González, Jacqueline Aldridge, Diego Alvarez, Marco Montes de Oca, Hermy Alvarez, Roberto Uribe-Paredes, Marcelo Navarrete
EPI_ISL_681776, EPI_ISL_681777, EPI_ISL_681778, EPI_ISL_681779, EPI_ISL_681780, EPI_ISL_681781, EPI_ISL_681782, EPI_ISL_681783, EPI_ISL_681784, EPI_ISL_681785, EPI_ISL_681786, EPI_ISL_681787, EPI_ISL_681788, EPI_ISL_681789, EPI_ISL_681790, EPI_ISL_681791, EPI_ISL_681792, EPI_ISL_681793, EPI_ISL_681794, EPI_ISL_681795, EPI_ISL_681796, EPI_ISL_681797, EPI_ISL_681798, EPI_ISL_681799, EPI_ISL_681800, EPI_ISL_681801, EPI_ISL_681802, EPI_ISL_681803, EPI_ISL_681804, EPI_ISL_681805, EPI_ISL_681806, EPI_ISL_681807, EPI_ISL_681808, EPI_ISL_681809, EPI_ISL_681810, EPI_ISL_681811, EPI_ISL_681812, EPI_ISL_681813, EPI_ISL_681814, EPI_ISL_681815, EPI_ISL_681816, EPI_ISL_681817, EPI_ISL_681818, EPI_ISL_681819, EPI_ISL_681820, EPI_ISL_681821, EPI_ISL_681822, EPI_ISL_681823, EPI_ISL_681824, EPI_ISL_681825, EPI_ISL_681826, EPI_ISL_681827, EPI_ISL_681828			
see above	GA Department of Public Health Laboratory	Pathogen Discovery, Respiratory Viruses Branch, Division of Viral Diseases, Centers for Disease Control and Prevention	Yan Li, Ying Tao, Jing Zhang, Krista Queen, Anna Uehara, Peter Cook, Clinton R. Paden, Haibin Wang, Suxiang Tong
EPI_ISL_681829, EPI_ISL_681830, EPI_ISL_681832, EPI_ISL_681833, EPI_ISL_681834, EPI_ISL_681835, EPI_ISL_681836, EPI_ISL_681838, EPI_ISL_681840, EPI_ISL_681841, EPI_ISL_681842			
see above	Molecular diagnostic unit for viral haemorrhagic fevers and emerging viruses, Bouaké CHU Laboratory	Project group Epidemology of Highly Pathogenic Microorganisms, Robert Koch-Institute	Chantal Akoua-Koffi, Diané Bamourou, Etilé Anoh, Essia Belarbi, Safiatou Karidioula, Grit Schubert, Adjaratou Traoré, Soundélé Maïté, Monemo Pacome, Coulibaly Mbegnan, Bamba Fatoumata Touré, Kra Ouffoué, Fabian Leendertz
EPI_ISL_681843, EPI_ISL_681844, EPI_ISL_681845, EPI_ISL_681846, EPI_ISL_681847, EPI_ISL_681848, EPI_ISL_681849, EPI_ISL_681850, EPI_ISL_681851, EPI_ISL_681852, EPI_ISL_681853, EPI_ISL_681854, EPI_ISL_681855, EPI_ISL_681856, EPI_ISL_681857, EPI_ISL_681858, EPI_ISL_681859, EPI_ISL_681860, EPI_ISL_681861, EPI_ISL_681862, EPI_ISL_681863, EPI_ISL_681864, EPI_ISL_681865, EPI_ISL_681866, EPI_ISL_681867, EPI_ISL_681876, EPI_ISL_681877, EPI_ISL_681878, EPI_ISL_681879, EPI_ISL_681880, EPI_ISL_681881, EPI_ISL_681882, EPI_ISL_681883			
see above	Texas Department of State Health Services	Texas Department of State Health Services	Rashmi Tuladhar, Bonnie Oh, Jenny Zhang, Maliha Rahman, Anita Pokharel, Myong Koag, Chung Wang, Rachel Lee, Grace Kubin, Mayela Pedrueza, James Daniel Bonser
EPI_ISL_681930, EPI_ISL_681931, EPI_ISL_681932, EPI_ISL_681933, EPI_ISL_681934, EPI_ISL_681935, EPI_ISL_681936, EPI_ISL_681937, EPI_ISL_681938, EPI_ISL_682005, EPI_ISL_682006, EPI_ISL_682007, EPI_ISL_682008, EPI_ISL_682009, EPI_ISL_682010, EPI_ISL_682011, EPI_ISL_682012, EPI_ISL_682013, EPI_ISL_682014, EPI_ISL_682015, EPI_ISL_682016, EPI_ISL_682017, EPI_ISL_682018, EPI_ISL_682019, EPI_ISL_682020, EPI_ISL_682021, EPI_ISL_682022, EPI_ISL_682023, EPI_ISL_682024, EPI_ISL_682025, EPI_ISL_682026, EPI_ISL_682027, EPI_ISL_682028, EPI_ISL_682029, EPI_ISL_682030, EPI_ISL_682031, EPI_ISL_682032, EPI_ISL_682033, EPI_ISL_682034, EPI_ISL_682035, EPI_ISL_682036, EPI_ISL_682037, EPI_ISL_682038, EPI_ISL_682039, EPI_ISL_682040, EPI_ISL_682041, EPI_ISL_682042, EPI_ISL_682043, EPI_ISL_682044, EPI_ISL_682045, EPI_ISL_682046, EPI_ISL_682047, EPI_ISL_682048, EPI_ISL_682049, EPI_ISL_682050, EPI_ISL_682051, EPI_ISL_682052, EPI_ISL_682053, EPI_ISL_682054, EPI_ISL_682055, EPI_ISL_682056			
see above	UPMC Clinical Microbiology Laboratory	Microbial Genomic Epidemiology Laboratory, University of Pittsburgh	Mustapha M. Mustapha, Jane W. Marsh, Dan Snyder, Marissa P. Griffith, Stephanie L. Mitchell, Vatsala R. Srinivasa, Kady D. Waggle, Chinelo Ezeonwuku, Vaughn S. Cooper, Lee H. Harrison
EPI_ISL_682058	Molecular diagnostic unit for viral haemorrhagic fevers and emerging viruses, Bouaké CHU Laboratory	Project group Epidemology of Highly Pathogenic Microorganisms, Robert Koch-Institute	Chantal Akoua-Koffi, Diané Bamourou, Etilé Anoh, Essia Belarbi, Safiatou Karidioula, Grit Schubert, Adjaratou Traoré, Soundélé Maïté, Monemo Pacome, Coulibaly Mbegnan, Bamba Fatoumata Touré, Kra Ouffoué, Fabian Leendertz

EPI_ISL_682060, EPI_ISL_682061, EPI_ISL_682062, EPI_ISL_682063, EPI_ISL_682064, EPI_ISL_682065, EPI_ISL_682066, EPI_ISL_682067, EPI_ISL_682068, EPI_ISL_682069, EPI_ISL_682070, EPI_ISL_682071, EPI_ISL_682072, EPI_ISL_682073, EPI_ISL_682074, EPI_ISL_682075, EPI_ISL_682076, EPI_ISL_682077, EPI_ISL_682078			
see above	Maryland Public Health Laboratory	Maryland Public Health Laboratory	Maryland Department of Health Laboratories Administration
EPI_ISL_682079, EPI_ISL_682080, EPI_ISL_682081, EPI_ISL_682082, EPI_ISL_682083, EPI_ISL_682084, EPI_ISL_682085, EPI_ISL_682086, EPI_ISL_682087, EPI_ISL_682088, EPI_ISL_682089, EPI_ISL_682090, EPI_ISL_682091, EPI_ISL_682092, EPI_ISL_682093, EPI_ISL_682094, EPI_ISL_682095, EPI_ISL_682096, EPI_ISL_682097, EPI_ISL_682098, EPI_ISL_682099, EPI_ISL_682100, EPI_ISL_682101, EPI_ISL_682102, EPI_ISL_682103, EPI_ISL_682104, EPI_ISL_682105, EPI_ISL_682106, EPI_ISL_682107, EPI_ISL_682108, EPI_ISL_682109, EPI_ISL_682110, EPI_ISL_682111, EPI_ISL_682112, EPI_ISL_682113, EPI_ISL_682114, EPI_ISL_682115, EPI_ISL_682116, EPI_ISL_682117, EPI_ISL_682118, EPI_ISL_682119, EPI_ISL_682120, EPI_ISL_682121, EPI_ISL_682122, EPI_ISL_682123, EPI_ISL_682124, EPI_ISL_682125, EPI_ISL_682126, EPI_ISL_682127, EPI_ISL_682128, EPI_ISL_682129, EPI_ISL_682130, EPI_ISL_682131, EPI_ISL_682132, EPI_ISL_682133, EPI_ISL_682134, EPI_ISL_682135, EPI_ISL_682136, EPI_ISL_682137, EPI_ISL_682138, EPI_ISL_682139, EPI_ISL_682140, EPI_ISL_682141, EPI_ISL_682142, EPI_ISL_682143, EPI_ISL_682144, EPI_ISL_682145, EPI_ISL_682146, EPI_ISL_682147, EPI_ISL_682148, EPI_ISL_682149, EPI_ISL_682150, EPI_ISL_682151, EPI_ISL_682152, EPI_ISL_682153, EPI_ISL_682154, EPI_ISL_682155, EPI_ISL_682156, EPI_ISL_682157, EPI_ISL_682158, EPI_ISL_682159, EPI_ISL_682160, EPI_ISL_682161, EPI_ISL_682162, EPI_ISL_682163, EPI_ISL_682164, EPI_ISL_682165, EPI_ISL_682166, EPI_ISL_682167, EPI_ISL_682168, EPI_ISL_682169, EPI_ISL_682170, EPI_ISL_682171, EPI_ISL_682172, EPI_ISL_682173, EPI_ISL_682174, EPI_ISL_682175, EPI_ISL_682176, EPI_ISL_682177, EPI_ISL_682178, EPI_ISL_682179, EPI_ISL_682180, EPI_ISL_682181, EPI_ISL_682182, EPI_ISL_682183, EPI_ISL_682184, EPI_ISL_682185, EPI_ISL_682186, EPI_ISL_682187, EPI_ISL_682188, EPI_ISL_682189, EPI_ISL_682190, EPI_ISL_682191, EPI_ISL_682192, EPI_ISL_682193, EPI_ISL_682194, EPI_ISL_682195, EPI_ISL_682196, EPI_ISL_682197, EPI_ISL_682198, EPI_ISL_682199, EPI_ISL_682200, EPI_ISL_682201, EPI_ISL_682202, EPI_ISL_682203, EPI_ISL_682204, EPI_ISL_682205, EPI_ISL_682206, EPI_ISL_682207, EPI_ISL_682208, EPI_ISL_682209, EPI_ISL_682210, EPI_ISL_682211, EPI_ISL_682212, EPI_ISL_682213, EPI_ISL_682214, EPI_ISL_682215, EPI_ISL_682216, EPI_ISL_682217, EPI_ISL_682218, EPI_ISL_682219, EPI_ISL_682220, EPI_ISL_682221, EPI_ISL_682222, EPI_ISL_682223, EPI_ISL_682224, EPI_ISL_682225, EPI_ISL_682226, EPI_ISL_682227, EPI_ISL_682228, EPI_ISL_682229, EPI_ISL_682230, EPI_ISL_682231, EPI_ISL_682232, EPI_ISL_682233, EPI_ISL_682234			
see above	University of Michigan Clinical Microbiology Laboratory	Lauring Lab, University of Michigan, Department of Microbiology and Immunology	Valesano
EPI_ISL_682235	AREA DE SALUD ALAJUELA NORTE - CLINICA DR. MARCIAL RODRIGUEZ	Inciensa, Instituto Costarricense de Investigación y Enseñanza en Nutrición y Salud	Francisco Duarte, Hebleen Porras, Claudio Soto-Garita, Estela Cordero, Adriana Godinez & Melany Calderon
EPI_ISL_682236	AREA DE SALUD LOS CHILES	Inciensa, Instituto Costarricense de Investigación y Enseñanza en Nutrición y Salud	Francisco Duarte, Hebleen Porras, Claudio Soto-Garita, Estela Cordero, Adriana Godinez, Melany Calderon & Mariel López
EPI_ISL_682237	AREA DE SALUD CORREDORES	Inciensa, Instituto Costarricense de Investigación y Enseñanza en Nutrición y Salud	Francisco Duarte, Hebleen Porras, Claudio Soto-Garita, Estela Cordero, Adriana Godinez & Melany Calderon
EPI_ISL_682238	AREA DE SALUD LA CRUZ	Inciensa, Instituto Costarricense de Investigación y Enseñanza en Nutrición y Salud	Francisco Duarte, Hebleen Porras, Claudio Soto-Garita, Estela Cordero, Adriana Godinez & Melany Calderon
EPI_ISL_682239	HOSPITAL CIUDAD NEILY	Inciensa, Instituto Costarricense de Investigación y Enseñanza en Nutrición y Salud	Francisco Duarte, Hebleen Porras, Claudio Soto-Garita, Estela Cordero, Adriana Godinez & Melany Calderon
EPI_ISL_682240	AREA DE SALUD GOICOCHEA 1	Inciensa, Instituto Costarricense de Investigación y Enseñanza en Nutrición y Salud	Francisco Duarte, Hebleen Porras, Claudio Soto-Garita, Estela Cordero, Adriana Godinez & Melany Calderon
EPI_ISL_682241	HOSPITAL SAN JUAN DE DIOS	Inciensa, Instituto Costarricense de Investigación y Enseñanza en Nutrición y Salud	Francisco Duarte, Hebleen Porras, Claudio Soto-Garita, Estela Cordero, Adriana Godinez & Melany Calderon
EPI_ISL_682242	HOSPITAL SAN VICENTE DE PAUL	Inciensa, Instituto Costarricense de Investigación y Enseñanza en Nutrición y Salud	Francisco Duarte, Hebleen Porras, Claudio Soto-Garita, Estela Cordero, Adriana Godinez, Melany Calderon & Mariel López
EPI_ISL_682243	HOSPITAL SAN JUAN DE DIOS	Inciensa, Instituto Costarricense de Investigación y Enseñanza en Nutrición y Salud	Francisco Duarte, Hebleen Porras, Claudio Soto-Garita, Estela Cordero, Adriana Godinez & Melany Calderon
EPI_ISL_682244	AREA DE SALUD LA CRUZ	Inciensa, Instituto Costarricense de Investigación y Enseñanza en Nutrición y Salud	Francisco Duarte, Hebleen Porras, Claudio Soto-Garita, Estela Cordero, Adriana Godinez, Melany Calderon & Mariel López
EPI_ISL_682245, EPI_ISL_682246	HOSPITAL SAN JUAN DE DIOS	Inciensa, Instituto Costarricense de Investigación y Enseñanza en Nutrición y Salud	Francisco Duarte, Hebleen Porras, Claudio Soto-Garita, Estela Cordero, Adriana Godinez & Melany Calderon
EPI_ISL_682247	AREA DE SALUD CIUDAD QUESADA	Inciensa, Instituto Costarricense de Investigación y Enseñanza en Nutrición y Salud	Francisco Duarte, Hebleen Porras, Claudio Soto-Garita, Estela Cordero, Adriana Godinez & Melany Calderon
EPI_ISL_682248	AREA DE SALUD CIUDAD QUESADA	Inciensa, Instituto Costarricense de Investigación y Enseñanza en Nutrición y Salud	Francisco Duarte, Hebleen Porras, Claudio Soto-Garita, Estela Cordero, Adriana Godinez, Melany Calderon & Mariel López
EPI_ISL_682249	AREA DE SALUD LA UNION	Inciensa, Instituto Costarricense de Investigación y Enseñanza en Nutrición y Salud	Francisco Duarte, Hebleen Porras, Claudio Soto-Garita, Estela Cordero, Adriana Godinez, Melany Calderon & Mariel López
EPI_ISL_682250, EPI_ISL_682251, EPI_ISL_682252	AREA DE SALUD ESCAZU (COOPESANA)	Inciensa, Instituto Costarricense de Investigación y Enseñanza en Nutrición y Salud	Francisco Duarte, Hebleen Porras, Claudio Soto-Garita, Estela Cordero, Adriana Godinez & Melany Calderon
EPI_ISL_682253, EPI_ISL_682254	HOSPITAL SAN JUAN DE DIOS	Inciensa, Instituto Costarricense de Investigación y Enseñanza en Nutrición y Salud	Francisco Duarte, Hebleen Porras, Claudio Soto-Garita, Estela Cordero, Adriana Godinez & Melany Calderon
EPI_ISL_682255	HOSPITAL SAN VICENTE DE PAUL	Inciensa, Instituto Costarricense de Investigación y Enseñanza en Nutrición y Salud	Francisco Duarte, Hebleen Porras, Claudio Soto-Garita, Estela Cordero, Adriana Godinez & Melany Calderon
EPI_ISL_682256	AREA DE SALUD CORREDORES	Inciensa, Instituto Costarricense de Investigación y Enseñanza en Nutrición y Salud	Francisco Duarte, Hebleen Porras, Claudio Soto-Garita, Estela Cordero, Adriana Godinez, Melany Calderon & Mariel López
EPI_ISL_682257	HOSPITAL SAN JUAN DE DIOS	Inciensa, Instituto Costarricense de Investigación y Enseñanza en Nutrición y Salud	Francisco Duarte, Hebleen Porras, Claudio Soto-Garita, Estela Cordero, Adriana Godinez & Melany Calderon
EPI_ISL_682258	AREA DE SALUD ALAJUELA NORTE - CLINICA DR. MARCIAL RODRIGUEZ	Inciensa, Instituto Costarricense de Investigación y Enseñanza en Nutrición y Salud	Francisco Duarte, Hebleen Porras, Claudio Soto-Garita, Estela Cordero, Adriana Godinez & Melany Calderon
EPI_ISL_682259	AREA DE SALUD ALAJUELA NORTE - CLINICA DR. MARCIAL RODRIGUEZ	Inciensa, Instituto Costarricense de Investigación y Enseñanza en Nutrición y Salud	Francisco Duarte, Hebleen Porras, Claudio Soto-Garita, Estela Cordero, Adriana Godinez, Melany Calderon & Mariel López
EPI_ISL_682260	AREA DE SALUD CATEDRAL NORESTE	Inciensa, Instituto Costarricense de Investigación y Enseñanza en Nutrición y Salud	Francisco Duarte, Hebleen Porras, Claudio Soto-Garita, Estela Cordero, Adriana Godinez, Melany Calderon & Mariel López
EPI_ISL_682261	TAMIZAJE COMUNITARIO- PASO CANOAS	Inciensa, Instituto Costarricense de Investigación y Enseñanza en Nutrición y Salud	Francisco Duarte, Hebleen Porras, Claudio Soto-Garita, Estela Cordero, Adriana Godinez & Melany Calderon
EPI_ISL_682262, EPI_ISL_682263, EPI_ISL_682264	HOSPITAL SAN JUAN DE DIOS	Inciensa, Instituto Costarricense de Investigación y Enseñanza en Nutrición y Salud	Francisco Duarte, Hebleen Porras, Claudio Soto-Garita, Estela Cordero, Adriana Godinez & Melany Calderon
EPI_ISL_682265	HOSPITAL UPALA	Inciensa, Instituto Costarricense de Investigación y Enseñanza en Nutrición y Salud	Francisco Duarte, Hebleen Porras, Claudio Soto-Garita, Estela Cordero, Adriana Godinez & Melany Calderon
EPI_ISL_682266, EPI_ISL_682267, EPI_ISL_682268, EPI_ISL_682269, EPI_ISL_682270, EPI_ISL_682271, EPI_ISL_682272, EPI_ISL_682273, EPI_ISL_682274	HOSPITAL SAN JUAN DE DIOS	Inciensa, Instituto Costarricense de Investigación y Enseñanza en Nutrición y Salud	Francisco Duarte, Hebleen Porras, Claudio Soto-Garita, Estela Cordero, Adriana Godinez & Melany Calderon
EPI_ISL_682275, EPI_ISL_682276, EPI_ISL_682277	LABORATORIOS LABIN	Inciensa, Instituto Costarricense de Investigación y Enseñanza en Nutrición y Salud	Francisco Duarte, Hebleen Porras, Claudio Soto-Garita, Estela Cordero, Adriana Godinez, Melany Calderon & Pei Ling Chan
EPI_ISL_682278, EPI_ISL_682279,	Middlemore Hospital	Institute of Environmental Science and Research (ESR)	Xiaoyun Ren, Matt Storey, Nikki Freed, Muhammad Faisal, Jing Wang, Hermes Perez, Anja Werno, Antje van der Linden, Arlo Upton, Chris Mansell, David

EPI_ISL_682280, EPI_ISL_682281, EPI_ISL_682282			Hammer, Dragana Drinkovic, Gary McAuliffe, Hana Sofia Andersson, James Ussher, Jill Sherwood, Josh Freeman, Julia Howard, Juliet Elvy, Mary DeAlmeida, Matt Blakiston, Matthew Rogers, Max Bloomfield, Michael Addidle, Michelle Balm, Sally Roberts, Sarah Jefferies, Sharmini Muttaiyah, Susan Morpeth, Susan Taylor, Timothy Blackmore, Vani Sathyendran, Veronica Playle, Virginia Hope, Erasmus Smit, Lauren Jelly, Olin Silander, Joep de Ligt
EPI_ISL_682283, EPI_ISL_682284, EPI_ISL_682286	Canterbury Health Laboratories	Institute of Environmental Science and Research (ESR)	Xiaoyun Ren, Matt Storey, Nikki Freed, Muhammad Faisal, Jing Wang, Hermes Perez, Anja Werno, Antje van der Linden, Arlo Upton, Chris Mansell, David Hammer, Dragana Drinkovic, Gary McAuliffe, Hana Sofia Andersson, James Ussher, Jill Sherwood, Josh Freeman, Julia Howard, Juliet Elvy, Mary DeAlmeida, Matt Blakiston, Matthew Rogers, Max Bloomfield, Michael Addidle, Michelle Balm, Sally Roberts, Sarah Jefferies, Sharmini Muttaiyah, Susan Morpeth, Susan Taylor, Timothy Blackmore, Vani Sathyendran, Veronica Playle, Virginia Hope, Erasmus Smit, Lauren Jelly, Olin Silander, Joep de Ligt
EPI_ISL_682287, EPI_ISL_682288, EPI_ISL_682289, EPI_ISL_682290	Middlemore Hospital	Institute of Environmental Science and Research (ESR)	Xiaoyun Ren, Matt Storey, Nikki Freed, Muhammad Faisal, Jing Wang, Hermes Perez, Anja Werno, Antje van der Linden, Arlo Upton, Chris Mansell, David Hammer, Dragana Drinkovic, Gary McAuliffe, Hana Sofia Andersson, James Ussher, Jill Sherwood, Josh Freeman, Julia Howard, Juliet Elvy, Mary DeAlmeida, Matt Blakiston, Matthew Rogers, Max Bloomfield, Michael Addidle, Michelle Balm, Sally Roberts, Sarah Jefferies, Sharmini Muttaiyah, Susan Morpeth, Susan Taylor, Timothy Blackmore, Vani Sathyendran, Veronica Playle, Virginia Hope, Erasmus Smit, Lauren Jelly, Olin Silander, Joep de Ligt
EPI_ISL_682291, EPI_ISL_682292, EPI_ISL_682293, EPI_ISL_682294	LabPLUS	Institute of Environmental Science and Research (ESR)	Xiaoyun Ren, Matt Storey, Nikki Freed, Muhammad Faisal, Jing Wang, Hermes Perez, Anja Werno, Antje van der Linden, Arlo Upton, Chris Mansell, David Hammer, Dragana Drinkovic, Gary McAuliffe, Hana Sofia Andersson, James Ussher, Jill Sherwood, Josh Freeman, Julia Howard, Juliet Elvy, Mary DeAlmeida, Matt Blakiston, Matthew Rogers, Max Bloomfield, Michael Addidle, Michelle Balm, Sally Roberts, Sarah Jefferies, Sharmini Muttaiyah, Susan Morpeth, Susan Taylor, Timothy Blackmore, Vani Sathyendran, Veronica Playle, Virginia Hope, Erasmus Smit, Lauren Jelly, Olin Silander, Joep de Ligt
EPI_ISL_682295, EPI_ISL_682296	Canterbury Health Laboratories	Institute of Environmental Science and Research (ESR)	Xiaoyun Ren, Matt Storey, Nikki Freed, Muhammad Faisal, Jing Wang, Hermes Perez, Anja Werno, Antje van der Linden, Arlo Upton, Chris Mansell, David Hammer, Dragana Drinkovic, Gary McAuliffe, Hana Sofia Andersson, James Ussher, Jill Sherwood, Josh Freeman, Julia Howard, Juliet Elvy, Mary DeAlmeida, Matt Blakiston, Matthew Rogers, Max Bloomfield, Michael Addidle, Michelle Balm, Sally Roberts, Sarah Jefferies, Sharmini Muttaiyah, Susan Morpeth, Susan Taylor, Timothy Blackmore, Vani Sathyendran, Veronica Playle, Virginia Hope, Erasmus Smit, Lauren Jelly, Olin Silander, Joep de Ligt
EPI_ISL_682299, EPI_ISL_682300, EPI_ISL_682301, EPI_ISL_682302, EPI_ISL_682303, EPI_ISL_682304, EPI_ISL_682305, EPI_ISL_682306, EPI_ISL_682307, EPI_ISL_682308, EPI_ISL_682309, EPI_ISL_682310, EPI_ISL_682311, EPI_ISL_682312, EPI_ISL_682313, EPI_ISL_682314, EPI_ISL_682315, EPI_ISL_682316, EPI_ISL_682317, EPI_ISL_682318, EPI_ISL_682319, EPI_ISL_682320, EPI_ISL_682321, EPI_ISL_682322			
see above	Communicable Disease Laboratory, Public Health Directorate	Communicable Disease Laboratory, Public Health Directorate	Alwasti,H., Altaif,Z., AlHujairi,Z., AlAbbas,Z.
EPI_ISL_682323, EPI_ISL_682324, EPI_ISL_682325, EPI_ISL_682326, EPI_ISL_682327, EPI_ISL_682328, EPI_ISL_682329, EPI_ISL_682330, EPI_ISL_682331, EPI_ISL_682332, EPI_ISL_682333, EPI_ISL_682334, EPI_ISL_682335, EPI_ISL_682336, EPI_ISL_682337, EPI_ISL_682338, EPI_ISL_682339, EPI_ISL_682341, EPI_ISL_682342, EPI_ISL_682343, EPI_ISL_682344, EPI_ISL_682345, EPI_ISL_682346, EPI_ISL_682347, EPI_ISL_682350, EPI_ISL_682351, EPI_ISL_682352			
see above	NHLS Universitas Academic	UFS Virology	PA Bester, MM Nyaga, P Nthiga, MT Mogotsi, D Goedhals, T de Oliveira
EPI_ISL_682353, EPI_ISL_682354, EPI_ISL_682355, EPI_ISL_682356, EPI_ISL_682357, EPI_ISL_682358, EPI_ISL_682359, EPI_ISL_682360, EPI_ISL_682361, EPI_ISL_682362, EPI_ISL_682363, EPI_ISL_682364, EPI_ISL_682365, EPI_ISL_682366, EPI_ISL_682367, EPI_ISL_682368, EPI_ISL_682369, EPI_ISL_682370, EPI_ISL_682371, EPI_ISL_682372, EPI_ISL_682373, EPI_ISL_682374, EPI_ISL_682375, EPI_ISL_682376, EPI_ISL_682377, EPI_ISL_682378, EPI_ISL_682379, EPI_ISL_682380, EPI_ISL_682381, EPI_ISL_682382, EPI_ISL_682383, EPI_ISL_682384, EPI_ISL_682385, EPI_ISL_682386, EPI_ISL_682387, EPI_ISL_682388, EPI_ISL_682389, EPI_ISL_682390, EPI_ISL_682391, EPI_ISL_682392, EPI_ISL_682393, EPI_ISL_682394, EPI_ISL_682395, EPI_ISL_682396, EPI_ISL_682397, EPI_ISL_682398, EPI_ISL_682400, EPI_ISL_682401, EPI_ISL_682402, EPI_ISL_682403, EPI_ISL_682404, EPI_ISL_682405, EPI_ISL_682406, EPI_ISL_682407, EPI_ISL_682408, EPI_ISL_682409, EPI_ISL_682410, EPI_ISL_682411, EPI_ISL_682412, EPI_ISL_682413, EPI_ISL_682414, EPI_ISL_682415, EPI_ISL_682416, EPI_ISL_682417, EPI_ISL_682418, EPI_ISL_682419, EPI_ISL_682420, EPI_ISL_682421, EPI_ISL_682422, EPI_ISL_682423, EPI_ISL_682424, EPI_ISL_682425, EPI_ISL_682426, EPI_ISL_682427, EPI_ISL_682428, EPI_ISL_682429, EPI_ISL_682430, EPI_ISL_682431, EPI_ISL_682432, EPI_ISL_682433, EPI_ISL_682434, EPI_ISL_682435, EPI_ISL_682436, EPI_ISL_682437, EPI_ISL_682438, EPI_ISL_682439, EPI_ISL_682440, EPI_ISL_682441, EPI_ISL_682442, EPI_ISL_682443, EPI_ISL_682444, EPI_ISL_682445, EPI_ISL_682446, EPI_ISL_682447, EPI_ISL_682448, EPI_ISL_682449, EPI_ISL_682450, EPI_ISL_682451, EPI_ISL_682452, EPI_ISL_682453, EPI_ISL_682455, EPI_ISL_682456, EPI_ISL_682457, EPI_ISL_682458, EPI_ISL_682459, EPI_ISL_682460, EPI_ISL_682461, EPI_ISL_682462, EPI_ISL_682463, EPI_ISL_682464, EPI_ISL_682465, EPI_ISL_682466, EPI_ISL_682467, EPI_ISL_682468, EPI_ISL_682469, EPI_ISL_682470, EPI_ISL_682471, EPI_ISL_682472, EPI_ISL_682473, EPI_ISL_682474, EPI_ISL_682475, EPI_ISL_682476, EPI_ISL_682477, EPI_ISL_682478, EPI_ISL_682479, EPI_ISL_682480, EPI_ISL_682481, EPI_ISL_682482, EPI_ISL_682483, EPI_ISL_682484, EPI_ISL_682485, EPI_ISL_682486, EPI_ISL_682487, EPI_ISL_682488, EPI_ISL_682489, EPI_ISL_682490, EPI_ISL_682491, EPI_ISL_682492, EPI_ISL_682494, EPI_ISL_682495, EPI_ISL_682496, EPI_ISL_682497, EPI_ISL_682498, EPI_ISL_682499, EPI_ISL_682500, EPI_ISL_682501, EPI_ISL_682502, EPI_ISL_682503, EPI_ISL_682504, EPI_ISL_682505, EPI_ISL_682506, EPI_ISL_682508, EPI_ISL_682509, EPI_ISL_682510, EPI_ISL_682511, EPI_ISL_682512, EPI_ISL_682513, EPI_ISL_682514, EPI_ISL_682515, EPI_ISL_682516, EPI_ISL_682517, EPI_ISL_682518, EPI_ISL_682519, EPI_ISL_682520, EPI_ISL_682521, EPI_ISL_682522, EPI_ISL_682523, EPI_ISL_682524, EPI_ISL_682525, EPI_ISL_682526, EPI_ISL_682527, EPI_ISL_682528, EPI_ISL_682529, EPI_ISL_682530, EPI_ISL_682531, EPI_ISL_682532, EPI_ISL_682533, EPI_ISL_682534, EPI_ISL_682535, EPI_ISL_682536, EPI_ISL_682537, EPI_ISL_682538, EPI_ISL_682539, EPI_ISL_682540, EPI_ISL_682541, EPI_ISL_682542, EPI_ISL_682543, EPI_ISL_682544, EPI_ISL_682545, EPI_ISL_682546, EPI_ISL_682547, EPI_ISL_682548, EPI_ISL_682549, EPI_ISL_682550, EPI_ISL_682551, EPI_ISL_682552, EPI_ISL_682553, EPI_ISL_682554, EPI_ISL_682555, EPI_ISL_682556, EPI_ISL_682557, EPI_ISL_682558, EPI_ISL_682559, EPI_ISL_682560, EPI_ISL_682561, EPI_ISL_682562, EPI_ISL_682563, EPI_ISL_682564, EPI_ISL_682565, EPI_ISL_682566, EPI_ISL_682567, EPI_ISL_682568, EPI_ISL_682569, EPI_ISL_682570, EPI_ISL_682571, EPI_ISL_682572, EPI_ISL_682573, EPI_ISL_682574, EPI_ISL_682576, EPI_ISL_682577, EPI_ISL_682578, EPI_ISL_682579, EPI_ISL_682580, EPI_ISL_682581, EPI_ISL_682582, EPI_ISL_682583, EPI_ISL_682584, EPI_ISL_682585, EPI_ISL_682586, EPI_ISL_682587, EPI_ISL_682588, EPI_ISL_682589, EPI_ISL_682591, EPI_ISL_682592, EPI_ISL_682593, EPI_ISL_682594, EPI_ISL_682595, EPI_ISL_682596, EPI_ISL_682597, EPI_ISL_682598, EPI_ISL_682599, EPI_ISL_682600, EPI_ISL_682601, EPI_ISL_682602, EPI_ISL_682603, EPI_ISL_682604, EPI_ISL_682605, EPI_ISL_682606, EPI_ISL_682607, EPI_ISL_682608, EPI_ISL_682609, EPI_ISL_682610, EPI_ISL_682611, EPI_ISL_682612, EPI_ISL_682613, EPI_ISL_682614, EPI_ISL_682615, EPI_ISL_682616, EPI_ISL_682617, EPI_ISL_682618, EPI_ISL_682619, EPI_ISL_682620, EPI_ISL_682621, EPI_ISL_682622, EPI_ISL_682623, EPI_ISL_682624, EPI_ISL_682625, EPI_ISL_682626, EPI_ISL_682627, EPI_ISL_682628, EPI_ISL_682629, EPI_ISL_682630, EPI_ISL_682631, EPI_ISL_682632, EPI_ISL_682633, EPI_ISL_682634, EPI_ISL_682635, EPI_ISL_682636, EPI_ISL_682637, EPI_ISL_682638, EPI_ISL_682639, EPI_ISL_682640, EPI_ISL_682641, EPI_ISL_682642, EPI_ISL_682643, EPI_ISL_682644, EPI_ISL_682645, EPI_ISL_682646, EPI_ISL_682647, EPI_ISL_682648, EPI_ISL_682649, EPI_ISL_682650, EPI_ISL_682651, EPI_ISL_682652, EPI_ISL_682654, EPI_ISL_682655, EPI_ISL_682656, EPI_ISL_682657, EPI_ISL_682658, EPI_ISL_682659, EPI_ISL_682660, EPI_ISL_682661, EPI_ISL_682662, EPI_ISL_682663, EPI_ISL_682664, EPI_ISL_682665, EPI_ISL_682666, EPI_ISL_682667, EPI_ISL_682669, EPI_ISL_682670, EPI_ISL_682671, EPI_ISL_682672, EPI_ISL_682673, EPI_ISL_682674, EPI_ISL_682675, EPI_ISL_682676, EPI_ISL_682677, EPI_ISL_682678, EPI_ISL_682679, EPI_ISL_682680, EPI_ISL_682681, EPI_ISL_682682, EPI_ISL_682683, EPI_ISL_682685, EPI_ISL_682686, EPI_ISL_682687, EPI_ISL_682689, EPI_ISL_682690, EPI_ISL_682691, EPI_ISL_682692, EPI_ISL_682694, EPI_ISL_682695, EPI_ISL_682696, EPI_ISL_682697, EPI_ISL_682698, EPI_ISL_682699, EPI_ISL_682700, EPI_ISL_682701, EPI_ISL_682702, EPI_ISL_682703, EPI_ISL_682704, EPI_ISL_682705, EPI_ISL_682706, EPI_ISL_682707, EPI_ISL_682708, EPI_ISL_682709, EPI_ISL_682710, EPI_ISL_682711, EPI_ISL_682712, EPI_ISL_682713, EPI_ISL_682714, EPI_ISL_682715, EPI_ISL_682716, EPI_ISL_682717, EPI_ISL_682718, EPI_ISL_682719, EPI_ISL_682720, EPI_ISL_682721, EPI_ISL_682722, EPI_ISL_682723, EPI_ISL_682724, EPI_ISL_682725, EPI_ISL_682726, EPI_ISL_682727, EPI_ISL_682728, EPI_ISL_682729, EPI_ISL_682730, EPI_ISL_682731, EPI_ISL_682732, EPI_ISL_682733, EPI_ISL_682734, EPI_ISL_682736, EPI_ISL_682737, EPI_ISL_682739, EPI_ISL_682740, EPI_ISL_682741, EPI_ISL_682742, EPI_ISL_682743, EPI_ISL_682744, EPI_ISL_682746, EPI_ISL_682747, EPI_ISL_682748, EPI_ISL_682749, EPI_ISL_682750, EPI_ISL_682751, EPI_ISL_682752, EPI_ISL_682753, EPI_ISL_682754, EPI_ISL_682755, EPI_ISL_682756, EPI_ISL_682757, EPI_ISL_682758, EPI_ISL_682759, EPI_ISL_682760, EPI_ISL_682761, EPI_ISL_682762, EPI_ISL_682763, EPI_ISL_682764, EPI_ISL_682765, EPI_ISL_682766, EPI_ISL_682767, EPI_ISL_682768, EPI_ISL_682769, EPI_ISL_682770, EPI_ISL_682772, EPI_ISL_682773, EPI_ISL_682774, EPI_ISL_682775, EPI_ISL_682776, EPI_ISL_682777, EPI_ISL_682778, EPI_ISL_682779, EPI_ISL_682780, EPI_ISL_682781, EPI_ISL_682782, EPI_ISL_682783, EPI_ISL_682784, EPI_ISL_682785, EPI_ISL_682786, EPI_ISL_682787, EPI_ISL_682788, EPI_ISL_682789, EPI_ISL_682790, EPI_ISL_682791, EPI_ISL_682792, EPI_ISL_682793, EPI_ISL_682794, EPI_ISL_682795, EPI_ISL_682796, EPI_ISL_682797, EPI_ISL_682798, EPI_ISL_682799, EPI_ISL_682800, EPI_ISL_682801, EPI_ISL_682802, EPI_ISL_682803, EPI_ISL_682804, EPI_ISL_682805, EPI_ISL_682806, EPI_ISL_682807, EPI_ISL_682808, EPI_ISL_682809, EPI_ISL_682810, EPI_ISL_682811, EPI_ISL_682812, EPI_ISL_682813, EPI_ISL_682814, EPI_ISL_682815, EPI_ISL_682816, EPI_ISL_682817, EPI_ISL_682818, EPI_ISL_682819, EPI_ISL_682820, EPI_ISL_682821, EPI_ISL_682822, EPI_ISL_682823, EPI_ISL_682824, EPI_ISL_682825, EPI_ISL_682826, EPI_ISL_682827, EPI_ISL_682828, EPI_ISL_682829, EPI_ISL_682830, EPI_ISL_682831, EPI_ISL_682833, EPI_ISL_682834, EPI_ISL_682835, EPI_ISL_682836, EPI_ISL_682837, EPI_ISL_682838, EPI_ISL_682839, EPI_ISL_682840, EPI_ISL_682841, EPI_ISL_682842, EPI_ISL_682844, EPI_ISL_682845, EPI_ISL_682846, EPI_ISL_682847, EPI_ISL_682848, EPI_ISL_682849, EPI_ISL_682850, EPI_ISL_682851, EPI_ISL_682852, EPI_ISL_682853, EPI_ISL_682854, EPI_ISL_682855, EPI_ISL_682856, EPI_ISL_682857, EPI_ISL_682858, EPI_ISL_682859, EPI_ISL_682860, EPI_ISL_682862, EPI_ISL_682863, EPI_ISL_682864, EPI_ISL_682865, EPI_ISL_682866, EPI_ISL_682867, EPI_ISL_682868, EPI_ISL_682869, EPI_ISL_682870, EPI_ISL_682871, EPI_ISL_682872, EPI_ISL_682873, EPI_ISL_682874, EPI_ISL_682875, EPI_ISL_682876, EPI_ISL_682878, EPI_ISL_682879, EPI_ISL_682880, EPI_ISL_682881, EPI_ISL_682882, EPI_ISL_682883, EPI_ISL_682884, EPI_ISL_682885, EPI_ISL_682886, EPI_ISL_682887, EPI_ISL_682888, EPI_ISL_682890, EPI_ISL_682892, EPI_ISL_682893, EPI_ISL_682894, EPI_ISL_682895, EPI_ISL_682896, EPI_ISL_682897, EPI_ISL_682898, EPI_ISL_682899, EPI_ISL_682900, EPI_ISL_682901, EPI_ISL_682902, EPI_ISL_682903, EPI_ISL_682904, EPI_ISL_682905, EPI_ISL_682906, EPI_ISL_682907, EPI_ISL_682908, EPI_ISL_682909, EPI_ISL_682910, EPI_ISL_682911, EPI_ISL_682912, EPI_ISL_682913, EPI_ISL_682914, EPI_ISL_682915, EPI_ISL_682916, EPI_ISL_682917, EPI_ISL_682918, EPI_ISL_682919, EPI_ISL_682920, EPI_ISL_682921, EPI_ISL_682922, EPI_ISL_682924, EPI_ISL_682925, EPI_ISL_682927, EPI_ISL_682928, EPI_ISL_682929, EPI_ISL_682930, EPI_ISL_682932, EPI_ISL_682933, EPI_ISL_682934, EPI_ISL_682935, EPI_ISL_682936, EPI_ISL_682939, EPI_ISL_682940, EPI_ISL_682941, EPI_ISL_682942, EPI_ISL_682943, EPI_ISL_682944, EPI_ISL_682945, EPI_ISL_682946, EPI_ISL_682947, EPI_ISL_682948, EPI_ISL_682950, EPI_ISL_682951, EPI_ISL_682952, EPI_ISL_682953, EPI_ISL_682954, EPI_ISL_682955, EPI_ISL_682956, EPI_ISL_682958, EPI_ISL_682959, EPI_ISL_682960, EPI_ISL_682961, EPI_ISL_682962, EPI_ISL_682963, EPI_ISL_682964, EPI_ISL_682965, EPI_ISL_682966, EPI_ISL_682967, EPI_ISL_682968, EPI_ISL_682969, EPI_ISL_682970, EPI_ISL_682971, EPI_ISL_682972, EPI_ISL_682973, EPI_ISL_682974, EPI_ISL_682975, EPI_ISL_682976, EPI_ISL_682977, EPI_ISL_682979, EPI_ISL_682980, EPI_ISL_682981, EPI_ISL_682982, EPI_ISL_682983, EPI_ISL_682984, EPI_ISL_682985, EPI_ISL_682986, EPI_ISL_682987, EPI_ISL_682988, EPI_ISL_682989, EPI_ISL_682990, EPI_ISL_682991, EPI_ISL_682992, EPI_ISL_682993, EPI_ISL_682994, EPI_ISL_682995, EPI_ISL_682996, EPI_ISL_682997, EPI_ISL_682998, EPI_ISL_682999, EPI_ISL_683000, EPI_ISL_683001, EPI_ISL_683002, EPI_ISL_683003, EPI_ISL_683004			
see above	Department of Virus and Microbiological Special Diagnostics, Statens Serum Institut, Copenhagen, Denmark	Albertsen Lab, Department of Chemistry and Bioscience, Aalborg University, Denmark	Danish Covid-19 Genome Consortium
EPI_ISL_683334, EPI_ISL_683335, EPI_ISL_683336, EPI_ISL_683337, EPI_ISL_683338, EPI_ISL_683339, EPI_ISL_683340, EPI_ISL_683341, EPI_ISL_683342, EPI_ISL_683343, EPI_ISL_683344, EPI_ISL_683345, EPI_ISL_683346, EPI_ISL_683347, EPI_ISL_683348, EPI_ISL_683349, EPI_ISL_683350, EPI_ISL_683351, EPI_ISL_683363, EPI_ISL_683364, EPI_ISL_683365, EPI_ISL_683366, EPI_ISL_683367, EPI_ISL_683368, EPI_ISL_683369, EPI_ISL_683370, EPI_ISL_683371, EPI_ISL_683372, EPI_ISL_683373, EPI_ISL_683374, EPI_ISL_683375, EPI_ISL_683376, EPI_ISL_683377, EPI_ISL_683378, EPI_ISL_683379, EPI_ISL_683380, EPI_ISL_683381, EPI_ISL_683382, EPI_ISL_683383, EPI_ISL_683384, EPI_ISL_683385, EPI_ISL_683386, EPI_ISL_683387, EPI_ISL_683388, EPI_ISL_683389, EPI_ISL_683390, EPI_ISL_683391, EPI_ISL_683392, EPI_ISL_683393, EPI_ISL_683394, EPI_ISL_683395, EPI_ISL_683396, EPI_ISL_683397, EPI_ISL_683398, EPI_ISL_683399, EPI_ISL_683400, EPI_ISL_683401, EPI_ISL_683402			
see above	CNR Virus des Infections Respiratoires - France SUD	CNR Virus des Infections Respiratoires - France SUD	Antonin Bal, Gregory Destras, Gwendolyne Burfin, Quentin Semanas, Martine Valette, Bruno Lina, Laurence Josset
EPI_ISL_683404, EPI_ISL_683407, EPI_ISL_683408, EPI_ISL_683409, EPI_ISL_683410, EPI_ISL_683411, EPI_ISL_683412, EPI_ISL_683413, EPI_ISL_683415, EPI_ISL_683417, EPI_ISL_683418, EPI_ISL_683419, EPI_ISL_683420, EPI_ISL_683421, EPI_ISL_683422, EPI_ISL_683423, EPI_ISL_683424, EPI_ISL_683425, EPI_ISL_683426, EPI_ISL_683427, EPI_ISL_683430, EPI_ISL_683433, EPI_ISL_683434, EPI_ISL_683435, EPI_ISL_683436, EPI_ISL_683439, EPI_ISL_683440, EPI_ISL_683441, EPI_ISL_683442, EPI_ISL_683443, EPI_ISL_683444, EPI_ISL_683445			
see above	Texas Department of State Health Services	Texas Department of State Health Services	Rashmi Tuladhar, Bonnie Oh, Jenny Zhang, Maliha Rahman, Anita Pokharel, Myong Koag, Chung Wang, Rachel Lee, Grace Kubin, Mayela Pedrueza, James Daniel Bonser
EPI_ISL_683452, EPI_ISL_683453, EPI_ISL_683454, EPI_ISL_683455, EPI_ISL_683457, EPI_ISL_683458, EPI_ISL_683459, EPI_ISL_683460, EPI_ISL_683461, EPI_ISL_683462, EPI_ISL_683463, EPI_ISL_683464, EPI_ISL_683465, EPI_ISL_683466, EPI_ISL_683467, EPI_ISL_683468, EPI_ISL_683469, EPI_ISL_683470, EPI_ISL_683471, EPI_ISL_683472, EPI_ISL_683473, EPI_ISL_683478, EPI_ISL_683479, EPI_ISL_683480, EPI_ISL_683481, EPI_ISL_683482, EPI_ISL_683483, EPI_ISL_683484, EPI_ISL_683485, EPI_ISL_683486, EPI_ISL_683487, EPI_ISL_683488, EPI_ISL_683489, EPI_ISL_683490, EPI_ISL_683491, EPI_ISL_683492, EPI_ISL_683493, EPI_ISL_683494, EPI_ISL_683495, EPI_ISL_683496, EPI_ISL_683497, EPI_ISL_683498, EPI_ISL_683499, EPI_ISL_683500, EPI_ISL_683501, EPI_ISL_683502, EPI_ISL_683503, EPI_ISL_683504, EPI_ISL_683505, EPI_ISL_683506, EPI_ISL_683507, EPI_ISL_683508, EPI_ISL_683509, EPI_ISL_683510, EPI_ISL_683511, EPI_ISL_683512, EPI_ISL_683513, EPI_ISL_683514, EPI_ISL_683515, EPI_ISL_683516, EPI_ISL_683517, EPI_ISL_683518, EPI_ISL_683519, EPI_ISL_683520, EPI_ISL_683521, EPI_ISL_683522, EPI_ISL_683523, EPI_ISL_683524, EPI_ISL_683525, EPI_ISL_683526, EPI_ISL_683527, EPI_ISL_683528, EPI_ISL_683529, EPI_ISL_683530, EPI_ISL_683531, EPI_ISL_683532, EPI_ISL_683533, EPI_ISL_683534, EPI_ISL_683535, EPI_ISL_683536, EPI_ISL_683537, EPI_ISL_683538, EPI_ISL_683539, EPI_ISL_683540, EPI_ISL_683541, EPI_ISL_683542, EPI_ISL_683543, EPI_ISL_683544, EPI_ISL_683545, EPI_ISL_683546, EPI_ISL_683547, EPI_ISL_683548, EPI_ISL_683549, EPI_ISL_683550, EPI_ISL_683551, EPI_ISL_683552, EPI_ISL_683553, EPI_ISL_683554, EPI_ISL_683555, EPI_ISL_683556, EPI_ISL_683557, EPI_ISL_683558, EPI_ISL_683559, EPI_ISL_683560, EPI_ISL_683561, EPI_ISL_683562, EPI_ISL_683563, EPI_ISL_683564, EPI_ISL_683565, EPI_ISL_683566, EPI_ISL_683567, EPI_ISL_683568, EPI_ISL_683569, EPI_ISL_683570, EPI_ISL_683571, EPI_ISL_683572, EPI_ISL_683573, EPI_ISL_683574, EPI_ISL_683575, EPI_ISL_683576, EPI_ISL_683577, EPI_ISL_683578, EPI_ISL_683579, EPI_ISL_683580, EPI_ISL_683581, EPI_ISL_683582, EPI_ISL_683583, EPI_ISL_683584, EPI_ISL_683585, EPI_ISL_683586, EPI_ISL_683587, EPI_ISL_683588, EPI_ISL_683589, EPI_ISL_683590, EPI_ISL_683591			

see above	Respiratory Virus Unit, Microbiology Services Colindale, Public Health England	COVID-19 Genomics UK (COG-UK) Consortium	PHE Covid Sequencing Team
EPI_ISL_683597	Hospital Clínico Universitario Lozano Blesa de Zaragoza (España)	SeqCOVID-SPAIN consortium/IBV(CSIC)	Rafael Benito, Sonia Algarate, Jessica Bueno and SeqCOVID-SPAIN consortium
EPI_ISL_683598, EPI_ISL_683599, EPI_ISL_683600, EPI_ISL_683601, EPI_ISL_683602, EPI_ISL_683603, EPI_ISL_683604, EPI_ISL_683605, EPI_ISL_683606, EPI_ISL_683607, EPI_ISL_683608, EPI_ISL_683609, EPI_ISL_683610, EPI_ISL_683611, EPI_ISL_683612, EPI_ISL_683613, EPI_ISL_683614, EPI_ISL_683615, EPI_ISL_683616, EPI_ISL_683617, EPI_ISL_683618, EPI_ISL_683619, EPI_ISL_683620, EPI_ISL_683621, EPI_ISL_683622, EPI_ISL_683623, EPI_ISL_683624, EPI_ISL_683625, EPI_ISL_683626, EPI_ISL_683627, EPI_ISL_683628, EPI_ISL_683629, EPI_ISL_683630, EPI_ISL_683631, EPI_ISL_683632, EPI_ISL_683633, EPI_ISL_683634, EPI_ISL_683635, EPI_ISL_683636, EPI_ISL_683637, EPI_ISL_683638, EPI_ISL_683639, EPI_ISL_683640, EPI_ISL_683641, EPI_ISL_683642, EPI_ISL_683643, EPI_ISL_683644, EPI_ISL_683645, EPI_ISL_683646, EPI_ISL_683647, EPI_ISL_683648, EPI_ISL_683649, EPI_ISL_683650, EPI_ISL_683651, EPI_ISL_683652, EPI_ISL_683653, EPI_ISL_683654, EPI_ISL_683655, EPI_ISL_683656			
see above	Servicio de Microbiología, Laboratori Clínic Metropolitana Nord. Hospital Universitari Germans Trias i Pujol. Institut d'Investigació en Ciències de la Salut Germans Trias i Pujol (IGTP)	SeqCOVID-SPAIN consortium/IBV(CSIC)	Elisa Martró, Antoni E. Bordoy, Anna Not, Adrián Antuori, Anabel Fernández, Nona Romaní, Verónica Saludes, Cristina Casañ and SeqCOVID-SPAIN consortium
EPI_ISL_683689	Hennepin County Medical Center	Minnesota Department of Health, Public Health Laboratory	Alexandra Lorentz, Jacob Garfin, Matt Plumb, and Xiong Wang
EPI_ISL_683690, EPI_ISL_683691, EPI_ISL_683692, EPI_ISL_683693, EPI_ISL_683694	Essentia Health-St. Mary's Medical Center	Minnesota Department of Health, Public Health Laboratory	Alexandra Lorentz, Jacob Garfin, Matt Plumb, and Xiong Wang
EPI_ISL_683695	Mayo Clinic & Mayo Clinic Laboratories	Minnesota Department of Health, Public Health Laboratory	Alexandra Lorentz, Jacob Garfin, Matt Plumb, and Xiong Wang
EPI_ISL_683696, EPI_ISL_683697, EPI_ISL_683698, EPI_ISL_683699, EPI_ISL_683700, EPI_ISL_683701, EPI_ISL_683702, EPI_ISL_683703, EPI_ISL_683704, EPI_ISL_683705, EPI_ISL_683706, EPI_ISL_683707, EPI_ISL_683708, EPI_ISL_683709, EPI_ISL_683710, EPI_ISL_683711, EPI_ISL_683712, EPI_ISL_683713			
see above	Essentia Health-St. Mary's Medical Center	Minnesota Department of Health, Public Health Laboratory	Alexandra Lorentz, Jacob Garfin, Matt Plumb, and Xiong Wang
EPI_ISL_683714, EPI_ISL_683715, EPI_ISL_683716, EPI_ISL_683717, EPI_ISL_683718, EPI_ISL_683719, EPI_ISL_683720, EPI_ISL_683721, EPI_ISL_683722, EPI_ISL_683723, EPI_ISL_683724, EPI_ISL_683725, EPI_ISL_683726, EPI_ISL_683727, EPI_ISL_683728			
see above	Mayo Clinic & Mayo Clinic Laboratories	Minnesota Department of Health, Public Health Laboratory	Alexandra Lorentz, Jacob Garfin, Matt Plumb, and Xiong Wang
EPI_ISL_683729, EPI_ISL_683730, EPI_ISL_683731, EPI_ISL_683732, EPI_ISL_683733, EPI_ISL_683734, EPI_ISL_683735, EPI_ISL_683736, EPI_ISL_683737, EPI_ISL_683738, EPI_ISL_683739, EPI_ISL_683740, EPI_ISL_683741, EPI_ISL_683742, EPI_ISL_683743, EPI_ISL_683744, EPI_ISL_683745, EPI_ISL_683746, EPI_ISL_683747, EPI_ISL_683748, EPI_ISL_683749, EPI_ISL_683750			
see above	Minnesota Department of Health, Public Health Laboratory	Minnesota Department of Health, Public Health Laboratory	Alexandra Lorentz, Jacob Garfin, Matt Plumb, and Xiong Wang
EPI_ISL_683753	DOHMH Corona	New York City Public Health Laboratory	Jade Wang, et al.
EPI_ISL_683754	DOHMH Morrisania	New York City Public Health Laboratory	Jade Wang, et al.
EPI_ISL_683755	DOHMH PHL	New York City Public Health Laboratory	Jade Wang, et al.
EPI_ISL_683756	DOHMH Central Harlem	New York City Public Health Laboratory	Jade Wang, et al.
EPI_ISL_683757, EPI_ISL_683758	DOHMH Corona	New York City Public Health Laboratory	Jade Wang, et al.
EPI_ISL_683759	DOHMH PHL	New York City Public Health Laboratory	Jade Wang, et al.
EPI_ISL_683760	DOHMH Morrisania	New York City Public Health Laboratory	Jade Wang, et al.
EPI_ISL_683761	DOHMH Corona	New York City Public Health Laboratory	Jade Wang, et al.
EPI_ISL_683762, EPI_ISL_683763	DOHMH Morrisania	New York City Public Health Laboratory	Jade Wang, et al.
EPI_ISL_683764	DOHMH Corona	New York City Public Health Laboratory	Jade Wang, et al.
EPI_ISL_683765	DOHMH Chelsea	New York City Public Health Laboratory	Jade Wang, et al.
EPI_ISL_683766	DOHMH Corona	New York City Public Health Laboratory	Jade Wang, et al.
EPI_ISL_683767, EPI_ISL_683768, EPI_ISL_683769	DOHMH Riverside	New York City Public Health Laboratory	Jade Wang, et al.
EPI_ISL_683770	DOHMH Corona	New York City Public Health Laboratory	Jade Wang, et al.
EPI_ISL_683771	DOHMH Fort Greene	New York City Public Health Laboratory	Jade Wang, et al.
EPI_ISL_683772	DOHMH Morrisania	New York City Public Health Laboratory	Jade Wang, et al.
EPI_ISL_683773	DOHMH Corona	New York City Public Health Laboratory	Jade Wang, et al.
EPI_ISL_683774	DOHMH PHL	New York City Public Health Laboratory	Jade Wang, et al.
EPI_ISL_683775, EPI_ISL_683776	DOHMH Jamaica	New York City Public Health Laboratory	Jade Wang, et al.
EPI_ISL_683777	DOHMH Corona	New York City Public Health Laboratory	Jade Wang, et al.
EPI_ISL_683778	DOHMH Morrisania	New York City Public Health Laboratory	Jade Wang, et al.
EPI_ISL_683779	DOHMH Corona	New York City Public Health Laboratory	Jade Wang, et al.
EPI_ISL_683780	DOHMH Chelsea	New York City Public Health Laboratory	Jade Wang, et al.
EPI_ISL_683781, EPI_ISL_683782	DOHMH Jamaica	New York City Public Health Laboratory	Jade Wang, et al.
EPI_ISL_683783	DOHMH PHL	New York City Public Health Laboratory	Jade Wang, et al.
EPI_ISL_683784	DOHMH Riverside	New York City Public Health Laboratory	Jade Wang, et al.
EPI_ISL_683785	DOHMH Corona	New York City Public Health Laboratory	Jade Wang, et al.
EPI_ISL_683786	DOHMH Jamaica	New York City Public Health Laboratory	Jade Wang, et al.
EPI_ISL_683787	DOHMH PHL	New York City Public Health Laboratory	Jade Wang, et al.
EPI_ISL_683788	DOHMH Chelsea	New York City Public Health Laboratory	Jade Wang, et al.
EPI_ISL_683789	DOHMH PHL	New York City Public Health Laboratory	Jade Wang, et al.
EPI_ISL_683790	DOHMH Morrisania	New York City Public Health Laboratory	Jade Wang, et al.
EPI_ISL_683791	DOHMH Jamaica	New York City Public Health Laboratory	Jade Wang, et al.
EPI_ISL_683792	DOHMH Chelsea	New York City Public Health Laboratory	Jade Wang, et al.
EPI_ISL_683793, EPI_ISL_683794	DOHMH Corona	New York City Public Health Laboratory	Jade Wang, et al.
EPI_ISL_683795	DOHMH Central Harlem	New York City Public Health Laboratory	Jade Wang, et al.
EPI_ISL_683796	DOHMH Corona	New York City Public Health Laboratory	Jade Wang, et al.
EPI_ISL_683797	DOHMH PHL	New York City Public Health Laboratory	Jade Wang, et al.

EPI_ISL_683798, EPI_ISL_683799, EPI_ISL_683800	DOHMH Corona	New York City Public Health Laboratory	Jade Wang, et al.
EPI_ISL_683801	DOHMH Morrisania	New York City Public Health Laboratory	Jade Wang, et al.
EPI_ISL_683802	DOHMH Corona	New York City Public Health Laboratory	Jade Wang, et al.
EPI_ISL_683803, EPI_ISL_683804, EPI_ISL_683805, EPI_ISL_683806	DOHMH Morrisania	New York City Public Health Laboratory	Jade Wang, et al.
EPI_ISL_683807, EPI_ISL_683808	DOHMH Chelsea	New York City Public Health Laboratory	Jade Wang, et al.
EPI_ISL_683809	DOHMH Corona	New York City Public Health Laboratory	Jade Wang, et al.
EPI_ISL_683810	DOHMH PHL	New York City Public Health Laboratory	Jade Wang, et al.
EPI_ISL_683811, EPI_ISL_683812	DOHMH Corona	New York City Public Health Laboratory	Jade Wang, et al.
EPI_ISL_683813	DOHMH Central Harlem	New York City Public Health Laboratory	Jade Wang, et al.
EPI_ISL_683814	DOHMH Morrisania	New York City Public Health Laboratory	Jade Wang, et al.
EPI_ISL_683815	DOHMH Corona	New York City Public Health Laboratory	Jade Wang, et al.
EPI_ISL_683816	DOHMH Fort Greene	New York City Public Health Laboratory	Jade Wang, et al.
EPI_ISL_683817, EPI_ISL_683818	DOHMH Corona	New York City Public Health Laboratory	Jade Wang, et al.
EPI_ISL_683819, EPI_ISL_683820	DOHMH Riverside	New York City Public Health Laboratory	Jade Wang, et al.
EPI_ISL_683821	DOHMH Crown Heights	New York City Public Health Laboratory	Jade Wang, et al.
EPI_ISL_683822	DOHMH Jamaica	New York City Public Health Laboratory	Jade Wang, et al.
EPI_ISL_683823	DOHMH Morrisania	New York City Public Health Laboratory	Jade Wang, et al.
EPI_ISL_683824	DOHMH Corona	New York City Public Health Laboratory	Jade Wang, et al.
EPI_ISL_683825, EPI_ISL_683826	DOHMH Morrisania	New York City Public Health Laboratory	Jade Wang, et al.
EPI_ISL_683827	DOHMH Crown Heights	New York City Public Health Laboratory	Jade Wang, et al.
EPI_ISL_683828	DOHMH Chelsea	New York City Public Health Laboratory	Jade Wang, et al.
EPI_ISL_683829	DOHMH Central Harlem	New York City Public Health Laboratory	Jade Wang, et al.
EPI_ISL_683830	DOHMH Chelsea	New York City Public Health Laboratory	Jade Wang, et al.
EPI_ISL_683831	DOHMH Corona	New York City Public Health Laboratory	Jade Wang, et al.
EPI_ISL_683832	DOHMH Fort Greene	New York City Public Health Laboratory	Jade Wang, et al.
EPI_ISL_683833	DOHMH Corona	New York City Public Health Laboratory	Jade Wang, et al.
EPI_ISL_683834	DOHMH Central Harlem	New York City Public Health Laboratory	Jade Wang, et al.
EPI_ISL_683835	CICM	Malaria Research and Training Center (MRTC-Parasito)	Antoine Dara, Abdoulaye Djimde
EPI_ISL_683836	DOHMH PHL	New York City Public Health Laboratory	Jade Wang, et al.
EPI_ISL_683837, EPI_ISL_683838	DOHMH Jamaica	New York City Public Health Laboratory	Jade Wang, et al.
EPI_ISL_683839, EPI_ISL_683840	DOHMH Morrisania	New York City Public Health Laboratory	Jade Wang, et al.
EPI_ISL_683841	DOHMH Corona	New York City Public Health Laboratory	Jade Wang, et al.
EPI_ISL_683842	DOHMH Central Harlem	New York City Public Health Laboratory	Jade Wang, et al.
EPI_ISL_683843, EPI_ISL_683844, EPI_ISL_683845	DOHMH Morrisania	New York City Public Health Laboratory	Jade Wang, et al.
EPI_ISL_683846	DOHMH PHL	New York City Public Health Laboratory	Jade Wang, et al.
EPI_ISL_683847	DOHMH Corona	New York City Public Health Laboratory	Jade Wang, et al.
EPI_ISL_683848	DOHMH Central Harlem	New York City Public Health Laboratory	Jade Wang, et al.
EPI_ISL_683849	DOHMH Corona	New York City Public Health Laboratory	Jade Wang, et al.
EPI_ISL_683850	DOHMH Jamaica	New York City Public Health Laboratory	Jade Wang, et al.
EPI_ISL_683851	DOHMH Corona	New York City Public Health Laboratory	Jade Wang, et al.
EPI_ISL_683852	DOHMH PHL	New York City Public Health Laboratory	Jade Wang, et al.
EPI_ISL_683853, EPI_ISL_683854	DOHMH Morrisania	New York City Public Health Laboratory	Jade Wang, et al.
EPI_ISL_683855	DOHMH Corona	New York City Public Health Laboratory	Jade Wang, et al.
EPI_ISL_683856	DOHMH Crown Heights	New York City Public Health Laboratory	Jade Wang, et al.
EPI_ISL_683857	DOHMH Corona	New York City Public Health Laboratory	Jade Wang, et al.
EPI_ISL_683858	DOHMH Jamaica	New York City Public Health Laboratory	Jade Wang, et al.
EPI_ISL_683859	DOHMH Central Harlem	New York City Public Health Laboratory	Jade Wang, et al.
EPI_ISL_683860	DOHMH Corona	New York City Public Health Laboratory	Jade Wang, et al.
EPI_ISL_683861, EPI_ISL_683862	DOHMH Jamaica	New York City Public Health Laboratory	Jade Wang, et al.
EPI_ISL_683863	DOHMH Morrisania	New York City Public Health Laboratory	Jade Wang, et al.
EPI_ISL_683864	DOHMH Jamaica	New York City Public Health Laboratory	Jade Wang, et al.
EPI_ISL_683865	DOHMH Chelsea	New York City Public Health Laboratory	Jade Wang, et al.
EPI_ISL_683866	DOHMH Riverside	New York City Public Health Laboratory	Jade Wang, et al.
EPI_ISL_683867	DOHMH Corona	New York City Public Health Laboratory	Jade Wang, et al.
EPI_ISL_683868	DOHMH Fort Greene	New York City Public Health Laboratory	Jade Wang, et al.
EPI_ISL_683869	DOHMH Morrisania	New York City Public Health Laboratory	Jade Wang, et al.

EPI_ISL_683870	DOHMH Corona	New York City Public Health Laboratory	Jade Wang, et al.
EPI_ISL_683871	DOHMH PHL	New York City Public Health Laboratory	Jade Wang, et al.
EPI_ISL_683872, EPI_ISL_683873	DOHMH Corona	New York City Public Health Laboratory	Jade Wang, et al.
EPI_ISL_683874	DOHMH Jamaica	New York City Public Health Laboratory	Jade Wang, et al.
EPI_ISL_683875	DOHMH PHL	New York City Public Health Laboratory	Jade Wang, et al.
EPI_ISL_683876	DOHMH Corona	New York City Public Health Laboratory	Jade Wang, et al.
EPI_ISL_683877	DOHMH Central Harlem	New York City Public Health Laboratory	Jade Wang, et al.
EPI_ISL_683878	DOHMH Jamaica	New York City Public Health Laboratory	Jade Wang, et al.
EPI_ISL_683879, EPI_ISL_683880	DOHMH Morrisania	New York City Public Health Laboratory	Jade Wang, et al.
EPI_ISL_683881	DOHMH Corona	New York City Public Health Laboratory	Jade Wang, et al.
EPI_ISL_683882, EPI_ISL_683883	DOHMH Central Harlem	New York City Public Health Laboratory	Jade Wang, et al.
EPI_ISL_683884	DOHMH Morrisania	New York City Public Health Laboratory	Jade Wang, et al.
EPI_ISL_683885	DOHMH Jamaica	New York City Public Health Laboratory	Jade Wang, et al.
EPI_ISL_683886	DOHMH Morrisania	New York City Public Health Laboratory	Jade Wang, et al.
EPI_ISL_683887, EPI_ISL_683888	DOHMH Fort Greene	New York City Public Health Laboratory	Jade Wang, et al.
EPI_ISL_683889	DOHMH Morrisania	New York City Public Health Laboratory	Jade Wang, et al.
EPI_ISL_683890	DOHMH PHL	New York City Public Health Laboratory	Jade Wang, et al.
EPI_ISL_683891	DOHMH Crown Heights	New York City Public Health Laboratory	Jade Wang, et al.
EPI_ISL_683892	DOHMH Jamaica	New York City Public Health Laboratory	Jade Wang, et al.
EPI_ISL_683893	DOHMH Corona	New York City Public Health Laboratory	Jade Wang, et al.
EPI_ISL_683894, EPI_ISL_683895	DOHMH Riverside	New York City Public Health Laboratory	Jade Wang, et al.
EPI_ISL_683896, EPI_ISL_683897, EPI_ISL_683898, EPI_ISL_683899, EPI_ISL_683900	DOHMH Corona	New York City Public Health Laboratory	Jade Wang, et al.
EPI_ISL_683901	DOHMH Jamaica	New York City Public Health Laboratory	Jade Wang, et al.
EPI_ISL_683902	DOHMH Riverside	New York City Public Health Laboratory	Jade Wang, et al.
EPI_ISL_683903	DOHMH Jamaica	New York City Public Health Laboratory	Jade Wang, et al.
EPI_ISL_683904	DOHMH Crown Heights	New York City Public Health Laboratory	Jade Wang, et al.
EPI_ISL_683905	DOHMH Morrisania	New York City Public Health Laboratory	Jade Wang, et al.
EPI_ISL_683906	DOHMH Fort Greene	New York City Public Health Laboratory	Jade Wang, et al.
EPI_ISL_683907	DOHMH Riverside	New York City Public Health Laboratory	Jade Wang, et al.
EPI_ISL_683908, EPI_ISL_683909	DOHMH Corona	New York City Public Health Laboratory	Jade Wang, et al.
EPI_ISL_683910	DOHMH Crown Heights	New York City Public Health Laboratory	Jade Wang, et al.
EPI_ISL_683911, EPI_ISL_683912	DOHMH Corona	New York City Public Health Laboratory	Jade Wang, et al.
EPI_ISL_683913	DOHMH Fort Greene	New York City Public Health Laboratory	Jade Wang, et al.
EPI_ISL_683914, EPI_ISL_683915, EPI_ISL_683916	DOHMH Jamaica	New York City Public Health Laboratory	Jade Wang, et al.
EPI_ISL_683917, EPI_ISL_683918, EPI_ISL_683919	DOHMH Morrisania	New York City Public Health Laboratory	Jade Wang, et al.
EPI_ISL_683920, EPI_ISL_683921	DOHMH Jamaica	New York City Public Health Laboratory	Jade Wang, et al.
EPI_ISL_683922	DOHMH Fort Greene	New York City Public Health Laboratory	Jade Wang, et al.
EPI_ISL_683923	DOHMH Chelsea	New York City Public Health Laboratory	Jade Wang, et al.
EPI_ISL_683924	DOHMH Central Harlem	New York City Public Health Laboratory	Jade Wang, et al.
EPI_ISL_683925	DOHMH Jamaica	New York City Public Health Laboratory	Jade Wang, et al.
EPI_ISL_683926	DOHMH Corona	New York City Public Health Laboratory	Jade Wang, et al.
EPI_ISL_683927, EPI_ISL_683928	DOHMH Central Harlem	New York City Public Health Laboratory	Jade Wang, et al.
EPI_ISL_683929	DOHMH Corona	New York City Public Health Laboratory	Jade Wang, et al.
EPI_ISL_683930	DOHMH Jamaica	New York City Public Health Laboratory	Jade Wang, et al.
EPI_ISL_683931	DOHMH Chelsea	New York City Public Health Laboratory	Jade Wang, et al.
EPI_ISL_683932	DOHMH Corona	New York City Public Health Laboratory	Jade Wang, et al.
EPI_ISL_683933	DOHMH Central Harlem	New York City Public Health Laboratory	Jade Wang, et al.
EPI_ISL_683934	DOHMH Corona	New York City Public Health Laboratory	Jade Wang, et al.
EPI_ISL_683935	DOHMH Crown Heights	New York City Public Health Laboratory	Jade Wang, et al.
EPI_ISL_683936	DOHMH Chelsea	New York City Public Health Laboratory	Jade Wang, et al.
EPI_ISL_683937	DOHMH Central Harlem	New York City Public Health Laboratory	Jade Wang, et al.
EPI_ISL_683938	DOHMH Morrisania	New York City Public Health Laboratory	Jade Wang, et al.
EPI_ISL_683939	DOHMH Jamaica	New York City Public Health Laboratory	Jade Wang, et al.
EPI_ISL_683940	DOHMH Morrisania	New York City Public Health Laboratory	Jade Wang, et al.
EPI_ISL_683941	DOHMH Chelsea	New York City Public Health Laboratory	Jade Wang, et al.
EPI_ISL_683942	DOHMH Riverside	New York City Public Health Laboratory	Jade Wang, et al.

EPI_ISL_683943	DOHMH PHL	New York City Public Health Laboratory	Jade Wang, et al.
EPI_ISL_683944, EPI_ISL_683945	DOHMH Jamaica	New York City Public Health Laboratory	Jade Wang, et al.
EPI_ISL_683946, EPI_ISL_683947	DOHMH Morrisania	New York City Public Health Laboratory	Jade Wang, et al.
EPI_ISL_683948	DOHMH Jamaica	New York City Public Health Laboratory	Jade Wang, et al.
EPI_ISL_683949, EPI_ISL_683950	DOHMH Riverside	New York City Public Health Laboratory	Jade Wang, et al.
EPI_ISL_683951	DOHMH Jamaica	New York City Public Health Laboratory	Jade Wang, et al.
EPI_ISL_683952, EPI_ISL_683953	DOHMH Corona	New York City Public Health Laboratory	Jade Wang, et al.
EPI_ISL_683954	DOHMH PHL	New York City Public Health Laboratory	Jade Wang, et al.
EPI_ISL_683955	DOHMH Riverside	New York City Public Health Laboratory	Jade Wang, et al.
EPI_ISL_683956	DOHMH Jamaica	New York City Public Health Laboratory	Jade Wang, et al.
EPI_ISL_683957	DOHMH Chelsea	New York City Public Health Laboratory	Jade Wang, et al.
EPI_ISL_683958	DOHMH Jamaica	New York City Public Health Laboratory	Jade Wang, et al.
EPI_ISL_683959	DOHMH Morrisania	New York City Public Health Laboratory	Jade Wang, et al.
EPI_ISL_683960	DOHMH Corona	New York City Public Health Laboratory	Jade Wang, et al.
EPI_ISL_683961	DOHMH Chelsea	New York City Public Health Laboratory	Jade Wang, et al.
EPI_ISL_683962	DOHMH Central Harlem	New York City Public Health Laboratory	Jade Wang, et al.
EPI_ISL_683963	DOHMH Fort Greene	New York City Public Health Laboratory	Jade Wang, et al.
EPI_ISL_683964	DOHMH Central Harlem	New York City Public Health Laboratory	Jade Wang, et al.
EPI_ISL_683965, EPI_ISL_683966	DOHMH Morrisania	New York City Public Health Laboratory	Jade Wang, et al.
EPI_ISL_683967	DOHMH Chelsea	New York City Public Health Laboratory	Jade Wang, et al.
EPI_ISL_683968	DOHMH Central Harlem	New York City Public Health Laboratory	Jade Wang, et al.
EPI_ISL_683969	DOHMH Morrisania	New York City Public Health Laboratory	Jade Wang, et al.
EPI_ISL_683970	DOHMH Crown Heights	New York City Public Health Laboratory	Jade Wang, et al.
EPI_ISL_683971, EPI_ISL_683972	DOHMH Chelsea	New York City Public Health Laboratory	Jade Wang, et al.
EPI_ISL_683973	DOHMH Fort Greene	New York City Public Health Laboratory	Jade Wang, et al.
EPI_ISL_683974	DOHMH Central Harlem	New York City Public Health Laboratory	Jade Wang, et al.
EPI_ISL_683975	DOHMH Jamaica	New York City Public Health Laboratory	Jade Wang, et al.
EPI_ISL_683976, EPI_ISL_683977	DOHMH Central Harlem	New York City Public Health Laboratory	Jade Wang, et al.
EPI_ISL_683978, EPI_ISL_683979	DOHMH Corona	New York City Public Health Laboratory	Jade Wang, et al.
EPI_ISL_683980, EPI_ISL_683981	DOHMH Chelsea	New York City Public Health Laboratory	Jade Wang, et al.
EPI_ISL_683982, EPI_ISL_683983, EPI_ISL_683984, EPI_ISL_683985	DOHMH Central Harlem	New York City Public Health Laboratory	Jade Wang, et al.
EPI_ISL_683986	DOHMH Jamaica	New York City Public Health Laboratory	Jade Wang, et al.
EPI_ISL_683987, EPI_ISL_683988	DOHMH PHL	New York City Public Health Laboratory	Jade Wang, et al.
EPI_ISL_683989	DOHMH Chelsea	New York City Public Health Laboratory	Jade Wang, et al.
EPI_ISL_683990	DOHMH Jamaica	New York City Public Health Laboratory	Jade Wang, et al.
EPI_ISL_683991	DOHMH Central Harlem	New York City Public Health Laboratory	Jade Wang, et al.
EPI_ISL_683992	DOHMH Morrisania	New York City Public Health Laboratory	Jade Wang, et al.
EPI_ISL_683993	DOHMH PHL	New York City Public Health Laboratory	Jade Wang, et al.
EPI_ISL_683994	DOHMH Riverside	New York City Public Health Laboratory	Jade Wang, et al.
EPI_ISL_683995	DOHMH PHL	New York City Public Health Laboratory	Jade Wang, et al.
EPI_ISL_683996	DOHMH Fort Greene	New York City Public Health Laboratory	Jade Wang, et al.
EPI_ISL_684000, EPI_ISL_684002, EPI_ISL_684003, EPI_ISL_684004, EPI_ISL_684005, EPI_ISL_684006, EPI_ISL_684007, EPI_ISL_684008, EPI_ISL_684010, EPI_ISL_684011, EPI_ISL_684012, EPI_ISL_684013, EPI_ISL_684015, EPI_ISL_684016, EPI_ISL_684017, EPI_ISL_684018, EPI_ISL_684020, EPI_ISL_684021, EPI_ISL_684023, EPI_ISL_684024, EPI_ISL_684026, EPI_ISL_684027			
see above	Utah Public Health Laboratory	Utah Public Health Laboratory	Erin Young, Kelly Oakeson
EPI_ISL_684028, EPI_ISL_684029, EPI_ISL_684030, EPI_ISL_684031, EPI_ISL_684032, EPI_ISL_684033, EPI_ISL_684034, EPI_ISL_684035, EPI_ISL_684036	Communicable Disease Laboratory, Public Health Directorate	Communicable Disease Laboratory, Public Health Directorate	Alwasti,H., Altaif,Z., AlHujairi,Z., AlAbbas,Z.
EPI_ISL_684047	NHLS Universitas Academic	UFS Virology	PA Bester, MM Nyaga, P Nthiga, MT Mogotsi, D Goedhals, T de Oliveira
EPI_ISL_686108, EPI_ISL_686536, EPI_ISL_686537, EPI_ISL_686538, EPI_ISL_686539, EPI_ISL_686540, EPI_ISL_686541, EPI_ISL_686542, EPI_ISL_686543, EPI_ISL_686544, EPI_ISL_686545, EPI_ISL_686547, EPI_ISL_686548, EPI_ISL_686549, EPI_ISL_686550, EPI_ISL_686551, EPI_ISL_686552, EPI_ISL_686553, EPI_ISL_686554, EPI_ISL_686555, EPI_ISL_686556, EPI_ISL_686557, EPI_ISL_686558, EPI_ISL_686559, EPI_ISL_686560, EPI_ISL_686561, EPI_ISL_686562, EPI_ISL_686563, EPI_ISL_686564, EPI_ISL_686565, EPI_ISL_686566, EPI_ISL_686567, EPI_ISL_686568, EPI_ISL_686569, EPI_ISL_686570, EPI_ISL_686571, EPI_ISL_686572, EPI_ISL_686573, EPI_ISL_686574, EPI_ISL_686575, EPI_ISL_686576, EPI_ISL_686577, EPI_ISL_686578, EPI_ISL_686579, EPI_ISL_686580, EPI_ISL_686581, EPI_ISL_686582, EPI_ISL_686583, EPI_ISL_686584, EPI_ISL_686585, EPI_ISL_686586, EPI_ISL_686587, EPI_ISL_686588, EPI_ISL_686589, EPI_ISL_686590, EPI_ISL_686591, EPI_ISL_686592, EPI_ISL_686593, EPI_ISL_686594, EPI_ISL_686595, EPI_ISL_686596, EPI_ISL_686598, EPI_ISL_686599, EPI_ISL_686600, EPI_ISL_686601, EPI_ISL_686602, EPI_ISL_686603, EPI_ISL_686604, EPI_ISL_686605, EPI_ISL_686606, EPI_ISL_686607, EPI_ISL_686608, EPI_ISL_686609, EPI_ISL_686611			
see above	Respiratory Virus Unit, Microbiology Services Colindale, Public Health England	COVID-19 Genomics UK (COG-UK) Consortium	PHE Covid Sequencing Team
EPI_ISL_691612, EPI_ISL_691613, EPI_ISL_691614, EPI_ISL_691615, EPI_ISL_691616, EPI_ISL_691617, EPI_ISL_691618, EPI_ISL_691620, EPI_ISL_691621, EPI_ISL_691622, EPI_ISL_691624, EPI_ISL_691625, EPI_ISL_691626, EPI_ISL_691627, EPI_ISL_691628, EPI_ISL_691629, EPI_ISL_691630, EPI_ISL_691631, EPI_ISL_691632, EPI_ISL_691633, EPI_ISL_691634, EPI_ISL_691635, EPI_ISL_691636, EPI_ISL_691637, EPI_ISL_691638, EPI_ISL_691641, EPI_ISL_691663, EPI_ISL_691664, EPI_ISL_691665, EPI_ISL_691667, EPI_ISL_691668, EPI_ISL_691669, EPI_ISL_691670, EPI_ISL_691671, EPI_ISL_691672, EPI_ISL_691673, EPI_ISL_691674, EPI_ISL_691675, EPI_ISL_691676, EPI_ISL_691678, EPI_ISL_691679, EPI_ISL_691680			
see above	Servicio de Microbiología, Hospital Universitario Son Espases	SeqCOVID-SPAIN consortium/IBV(CSIC)	Carla López-Causapé, Jordi Reina, Antonio Oliver and SeqCOVID-SPAIN consortium
EPI_ISL_691685	Hospital de Leon	Instituto de Salud Carlos III	Iglesias-Caballero, M. Camarero, S. Molinero Calamita, M. González-Esguevillas, M. Pozo, F. Casas, I. Jiménez, P. Jiménez, M. Zaballos, A. Monzón, S.

			Varona, S. Juliá, M. Cuesta, I. Vidan, J.
EPI_ISL_691686, EPI_ISL_691687, EPI_ISL_691688, EPI_ISL_691689, EPI_ISL_691690, EPI_ISL_691691, EPI_ISL_691692, EPI_ISL_691693, EPI_ISL_691694, EPI_ISL_691695, EPI_ISL_691696, EPI_ISL_691697, EPI_ISL_691698, EPI_ISL_691699			
see above	Hospital Universitario de Ceuta	Instituto de Salud Carlos III	Iglesias-Caballero, M. Camarero, S. Molinero Calamita, M. González-Esguevillas, M. Pozo, F. Casas, I. Jiménez, P. Jiménez, M. Zaballos, A. Monzón, S. Varona, S. Juliá, M. Cuesta, I. Hijano, S.
EPI_ISL_691700, EPI_ISL_691701, EPI_ISL_691702, EPI_ISL_691703, EPI_ISL_691704, EPI_ISL_691705, EPI_ISL_691706, EPI_ISL_691707, EPI_ISL_691708, EPI_ISL_691709, EPI_ISL_691710, EPI_ISL_691711, EPI_ISL_691712, EPI_ISL_691713, EPI_ISL_691714, EPI_ISL_691715, EPI_ISL_691716, EPI_ISL_691717, EPI_ISL_691718, EPI_ISL_691719, EPI_ISL_691720, EPI_ISL_691721, EPI_ISL_691722, EPI_ISL_691723, EPI_ISL_691724, EPI_ISL_691725, EPI_ISL_691726, EPI_ISL_691727, EPI_ISL_691728			
see above	Hospital Clínico San Carlos	Instituto de Salud Carlos III	Iglesias-Caballero, M. Camarero, S. Molinero Calamita, M. González-Esguevillas, M. Pozo, F. Casas, I. Jiménez, P. Jiménez, M. Zaballos, A. Monzón, S. Varona, S. Juliá, M. Cuesta, I. Rodríguez, I.
EPI_ISL_691729, EPI_ISL_691730	Hospital Universitario Severo Ochoa	Instituto de Salud Carlos III	Iglesias-Caballero, M. Camarero, S. Molinero Calamita, M. González-Esguevillas, M. Pozo, F. Casas, I. Jiménez, P. Jiménez, M. Zaballos, A. Monzón, S. Varona, S. Juliá, M. Cuesta, I. García, M.
EPI_ISL_691731	Hospital Universitario Puerta de Hierro	Instituto de Salud Carlos III	Iglesias-Caballero, M. Camarero, S. Molinero Calamita, M. González-Esguevillas, M. Pozo, F. Casas, I. Jiménez, P. Jiménez, M. Zaballos, A. Monzón, S. Varona, S. Juliá, M. Cuesta, I. Velasco, A.
EPI_ISL_691732	Hospital de Madrid	Instituto de Salud Carlos III	Iglesias-Caballero, M. Camarero, S. Molinero Calamita, M. González-Esguevillas, M. Pozo, F. Casas, I. Jiménez, P. Jiménez, M. Zaballos, A. Monzón, S. Varona, S. Juliá, M. Cuesta, I. Alhambra, A.
EPI_ISL_692733, EPI_ISL_692734, EPI_ISL_692735, EPI_ISL_692736, EPI_ISL_692737, EPI_ISL_692738, EPI_ISL_692739, EPI_ISL_692740, EPI_ISL_692741, EPI_ISL_692742, EPI_ISL_692743, EPI_ISL_692744, EPI_ISL_692745, EPI_ISL_692746, EPI_ISL_692747, EPI_ISL_692748, EPI_ISL_692749, EPI_ISL_692750, EPI_ISL_692751, EPI_ISL_692752, EPI_ISL_692753, EPI_ISL_692754, EPI_ISL_692755, EPI_ISL_692756, EPI_ISL_692757, EPI_ISL_692758, EPI_ISL_692759, EPI_ISL_692760, EPI_ISL_692761, EPI_ISL_692762, EPI_ISL_692763, EPI_ISL_692764, EPI_ISL_692765, EPI_ISL_692766, EPI_ISL_692767, EPI_ISL_692768			
see above	CNR Virus des Infections Respiratoires - France SUD	CNR Virus des Infections Respiratoires - France SUD	Antonin Bal, Gregory Destras, Gwendolyne Burfin, Solenne Brun, Martine Valette, Bruno Lina, Laurence Josset
EPI_ISL_692769, EPI_ISL_692770, EPI_ISL_692771, EPI_ISL_692772, EPI_ISL_692773, EPI_ISL_692774, EPI_ISL_692776, EPI_ISL_692778, EPI_ISL_692780, EPI_ISL_692782, EPI_ISL_692783, EPI_ISL_692784, EPI_ISL_692785, EPI_ISL_692786, EPI_ISL_692787, EPI_ISL_692788, EPI_ISL_692789, EPI_ISL_692790, EPI_ISL_692791, EPI_ISL_692792, EPI_ISL_692794, EPI_ISL_692795, EPI_ISL_692796, EPI_ISL_692798, EPI_ISL_692799, EPI_ISL_692801, EPI_ISL_692803, EPI_ISL_692804, EPI_ISL_692809, EPI_ISL_692810, EPI_ISL_692811, EPI_ISL_692812, EPI_ISL_692813, EPI_ISL_692814, EPI_ISL_692815, EPI_ISL_692816, EPI_ISL_692817, EPI_ISL_692818, EPI_ISL_692819, EPI_ISL_692820, EPI_ISL_692822, EPI_ISL_692834, EPI_ISL_692835, EPI_ISL_692836, EPI_ISL_692837, EPI_ISL_692838, EPI_ISL_692839, EPI_ISL_692840, EPI_ISL_692841, EPI_ISL_692842, EPI_ISL_692843, EPI_ISL_692844, EPI_ISL_692845, EPI_ISL_692846, EPI_ISL_692847, EPI_ISL_692848, EPI_ISL_692849, EPI_ISL_692850, EPI_ISL_692851, EPI_ISL_692852, EPI_ISL_692853, EPI_ISL_692854, EPI_ISL_692855, EPI_ISL_692863, EPI_ISL_692865, EPI_ISL_692866, EPI_ISL_692867, EPI_ISL_692868, EPI_ISL_692869, EPI_ISL_692870, EPI_ISL_692871, EPI_ISL_692873, EPI_ISL_692874, EPI_ISL_692875, EPI_ISL_692876, EPI_ISL_692901, EPI_ISL_692902, EPI_ISL_692903, EPI_ISL_692904, EPI_ISL_692905, EPI_ISL_692908, EPI_ISL_692909, EPI_ISL_692910, EPI_ISL_692911, EPI_ISL_692912, EPI_ISL_692913, EPI_ISL_692915, EPI_ISL_692916, EPI_ISL_692917, EPI_ISL_692918, EPI_ISL_692919, EPI_ISL_692920, EPI_ISL_692921, EPI_ISL_692922, EPI_ISL_692923, EPI_ISL_692924, EPI_ISL_692925, EPI_ISL_692926, EPI_ISL_692927, EPI_ISL_692928, EPI_ISL_692929, EPI_ISL_692930, EPI_ISL_692931, EPI_ISL_692932, EPI_ISL_692933, EPI_ISL_692934, EPI_ISL_692935, EPI_ISL_692936, EPI_ISL_692937, EPI_ISL_692938, EPI_ISL_692939, EPI_ISL_692940, EPI_ISL_692941, EPI_ISL_692942, EPI_ISL_692943, EPI_ISL_692944, EPI_ISL_692945, EPI_ISL_692946, EPI_ISL_693159, EPI_ISL_693170			
see above	Massachusetts State Public Health Laboratory	Massachusetts State Public Health Laboratory	Andrew Lang, Timelia Fink, Glen Gallagher, Sandra Smole
EPI_ISL_693204	Pronto Socorro Dr. Conrado Cesarino Nuvolini	Instituto Adolfo Lutz, Interdiciplinary Procedures Center, Strategic Laboratory	Claudio Tavares Sacchi, Claudia Regina Gonçalves, Erica Valessa Ramos Gomes, Karoline Rodrigues Campos
EPI_ISL_693205	Hospital de Campanha Covid-19 Assis	Instituto Adolfo Lutz, Interdiciplinary Procedures Center, Strategic Laboratory	Claudio Tavares Sacchi, Claudia Regina Gonçalves, Erica Valessa Ramos Gomes, Karoline Rodrigues Campos
EPI_ISL_693206	Hospital Municipal Mario Gatti	Instituto Adolfo Lutz, Interdiciplinary Procedures Center, Strategic Laboratory	Claudio Tavares Sacchi, Claudia Regina Gonçalves, Erica Valessa Ramos Gomes, Karoline Rodrigues Campos
EPI_ISL_693207	Cs II Doutor Antonio Vicoso Moreira de Rezende	Instituto Adolfo Lutz, Interdiciplinary Procedures Center, Strategic Laboratory	Claudio Tavares Sacchi, Claudia Regina Gonçalves, Erica Valessa Ramos Gomes, Karoline Rodrigues Campos
EPI_ISL_693208, EPI_ISL_693209	Hospital Municipal Antonio Giglio	Instituto Adolfo Lutz, Interdiciplinary Procedures Center, Strategic Laboratory	Claudio Tavares Sacchi, Claudia Regina Gonçalves, Erica Valessa Ramos Gomes, Karoline Rodrigues Campos
EPI_ISL_693210	Pronto-Socorro Dr. Osmar Mesquita	Instituto Adolfo Lutz, Interdiciplinary Procedures Center, Strategic Laboratory	Claudio Tavares Sacchi, Claudia Regina Gonçalves, Erica Valessa Ramos Gomes, Karoline Rodrigues Campos
EPI_ISL_693211	Santa Casa de Misericordia e Maternidade	Instituto Adolfo Lutz, Interdiciplinary Procedures Center, Strategic Laboratory	Claudio Tavares Sacchi, Claudia Regina Gonçalves, Erica Valessa Ramos Gomes, Karoline Rodrigues Campos
EPI_ISL_693212	Santa Casa de Misericordia de Braganca Paulista	Instituto Adolfo Lutz, Interdiciplinary Procedures Center, Strategic Laboratory	Claudio Tavares Sacchi, Claudia Regina Gonçalves, Erica Valessa Ramos Gomes, Karoline Rodrigues Campos
EPI_ISL_693213	Hospital E Maternidade Municipal Governador Mario Covas	Instituto Adolfo Lutz, Interdiciplinary Procedures Center, Strategic Laboratory	Claudio Tavares Sacchi, Claudia Regina Gonçalves, Erica Valessa Ramos Gomes, Karoline Rodrigues Campos
EPI_ISL_693214	Unidade de Pronto Atendimento Central de Caraguatatuba	Instituto Adolfo Lutz, Interdiciplinary Procedures Center, Strategic Laboratory	Claudio Tavares Sacchi, Claudia Regina Gonçalves, Erica Valessa Ramos Gomes, Karoline Rodrigues Campos
EPI_ISL_693215	Secretaria Municipal de Saúde de Iracemapolis	Instituto Adolfo Lutz, Interdiciplinary Procedures Center, Strategic Laboratory	Claudio Tavares Sacchi, Claudia Regina Gonçalves, Erica Valessa Ramos Gomes, Karoline Rodrigues Campos
EPI_ISL_693216, EPI_ISL_693217	Unidade de Vigilância Epiudemiológica de Araras	Instituto Adolfo Lutz, Interdiciplinary Procedures Center, Strategic Laboratory	Claudio Tavares Sacchi, Claudia Regina Gonçalves, Erica Valessa Ramos Gomes, Karoline Rodrigues Campos
EPI_ISL_693218	Hospital Domingos Leonardo Ceravolo Presidente Prudente	Instituto Adolfo Lutz, Interdiciplinary Procedures Center, Strategic Laboratory	Claudio Tavares Sacchi, Claudia Regina Gonçalves, Erica Valessa Ramos Gomes, Karoline Rodrigues Campos
EPI_ISL_693219	Santa Casa da Misericórdia de Presidente Prudente	Instituto Adolfo Lutz, Interdiciplinary Procedures Center, Strategic Laboratory	Claudio Tavares Sacchi, Claudia Regina Gonçalves, Erica Valessa Ramos Gomes, Karoline Rodrigues Campos
EPI_ISL_693220	Laboratório Municipal de Piracicaba	Instituto Adolfo Lutz, Interdiciplinary Procedures Center, Strategic Laboratory	Claudio Tavares Sacchi, Claudia Regina Gonçalves, Erica Valessa Ramos Gomes, Karoline Rodrigues Campos
EPI_ISL_693221, EPI_ISL_693222	Secretaria Municipal de Saúde de Birigui	Instituto Adolfo Lutz, Interdiciplinary Procedures Center, Strategic Laboratory	Claudio Tavares Sacchi, Claudia Regina Gonçalves, Erica Valessa Ramos Gomes, Karoline Rodrigues Campos
EPI_ISL_693223, EPI_ISL_693224	Laboratório Municipal de Piracicaba	Instituto Adolfo Lutz, Interdiciplinary Procedures Center, Strategic Laboratory	Claudio Tavares Sacchi, Claudia Regina Gonçalves, Erica Valessa Ramos Gomes, Karoline Rodrigues Campos
EPI_ISL_693225	Ubs Vila Rosa - Olímpia Gomes De Almeida	Instituto Adolfo Lutz, Interdiciplinary Procedures Center, Strategic Laboratory	Claudio Tavares Sacchi, Claudia Regina Gonçalves, Erica Valessa Ramos Gomes, Karoline Rodrigues Campos
EPI_ISL_693226	Unidade de Pronto Atendimento Sao José	Instituto Adolfo Lutz, Interdiciplinary Procedures Center, Strategic Laboratory	Claudio Tavares Sacchi, Claudia Regina Gonçalves, Erica Valessa Ramos Gomes, Karoline Rodrigues Campos
EPI_ISL_693227	UBS Vila Marchi	Instituto Adolfo Lutz, Interdiciplinary Procedures Center, Strategic Laboratory	Claudio Tavares Sacchi, Claudia Regina Gonçalves, Erica Valessa Ramos Gomes, Karoline Rodrigues Campos
EPI_ISL_693228	Secretaria Municipal de Sorocaba	Instituto Adolfo Lutz, Interdiciplinary Procedures Center, Strategic Laboratory	Claudio Tavares Sacchi, Claudia Regina Gonçalves, Erica Valessa Ramos Gomes, Karoline Rodrigues Campos
EPI_ISL_693229	Hospital 8 de Maio	Instituto Adolfo Lutz, Interdiciplinary Procedures Center, Strategic Laboratory	Claudio Tavares Sacchi, Claudia Regina Gonçalves, Erica Valessa Ramos Gomes, Karoline Rodrigues Campos
EPI_ISL_693230	Hospital e Pronto Socorro Portinari	Instituto Adolfo Lutz, Interdiciplinary Procedures Center, Strategic Laboratory	Claudio Tavares Sacchi, Claudia Regina Gonçalves, Erica Valessa Ramos Gomes, Karoline Rodrigues Campos

EPI_ISL_693231	Pronto Socorro Municipal de Santa Branca	Instituto Adolfo Lutz, Interdiciplinary Procedures Center, Strategic Laboratory	Claudio Tavares Sacchi, Claudia Regina Gonçalves, Erica Valessa Ramos Gomes, Karoline Rodrigues Campos
EPI_ISL_693233	Hospital Santa Cruz	Instituto Adolfo Lutz, Interdiciplinary Procedures Center, Strategic Laboratory	Claudio Tavares Sacchi, Claudia Regina Gonçalves, Erica Valessa Ramos Gomes, Karoline Rodrigues Campos
EPI_ISL_693234	Upa Vereador Jose Da Rocha Goncalves	Instituto Adolfo Lutz, Interdiciplinary Procedures Center, Strategic Laboratory	Claudio Tavares Sacchi, Claudia Regina Gonçalves, Erica Valessa Ramos Gomes, Karoline Rodrigues Campos
EPI_ISL_693235	Casmi Centro Atendimento Saude da Mulher e Infancia	Instituto Adolfo Lutz, Interdiciplinary Procedures Center, Strategic Laboratory	Claudio Tavares Sacchi, Claudia Regina Gonçalves, Erica Valessa Ramos Gomes, Karoline Rodrigues Campos
EPI_ISL_693236	Hospital Santa Marcelina Sao Paulo	Instituto Adolfo Lutz, Interdiciplinary Procedures Center, Strategic Laboratory	Claudio Tavares Sacchi, Claudia Regina Gonçalves, Erica Valessa Ramos Gomes, Karoline Rodrigues Campos
EPI_ISL_693237	UPA Santa Isabel	Instituto Adolfo Lutz, Interdiciplinary Procedures Center, Strategic Laboratory	Claudio Tavares Sacchi, Claudia Regina Gonçalves, Erica Valessa Ramos Gomes, Karoline Rodrigues Campos
EPI_ISL_693238, EPI_ISL_693239	Secao Centro de Diagnostico Secedi	Instituto Adolfo Lutz, Interdiciplinary Procedures Center, Strategic Laboratory	Claudio Tavares Sacchi, Claudia Regina Gonçalves, Erica Valessa Ramos Gomes, Karoline Rodrigues Campos
EPI_ISL_693240	Centro de Vigilância a Saude de Diadema	Instituto Adolfo Lutz, Interdiciplinary Procedures Center, Strategic Laboratory	Claudio Tavares Sacchi, Claudia Regina Gonçalves, Erica Valessa Ramos Gomes, Karoline Rodrigues Campos
EPI_ISL_693241	Hospital e Maternidade Sao Lucas	Instituto Adolfo Lutz, Interdiciplinary Procedures Center, Strategic Laboratory	Claudio Tavares Sacchi, Claudia Regina Gonçalves, Erica Valessa Ramos Gomes, Karoline Rodrigues Campos
EPI_ISL_693242	Centro de Vigilância a Saude de Diadema	Instituto Adolfo Lutz, Interdiciplinary Procedures Center, Strategic Laboratory	Claudio Tavares Sacchi, Claudia Regina Gonçalves, Erica Valessa Ramos Gomes, Karoline Rodrigues Campos
EPI_ISL_693243	Laboratório Municipal de Piracicaba	Instituto Adolfo Lutz, Interdiciplinary Procedures Center, Strategic Laboratory	Claudio Tavares Sacchi, Claudia Regina Gonçalves, Erica Valessa Ramos Gomes, Karoline Rodrigues Campos
EPI_ISL_693244	Centro Médico da Polícia Militar do Estado de Sao Paulo	Instituto Adolfo Lutz, Interdiciplinary Procedures Center, Strategic Laboratory	Claudio Tavares Sacchi, Claudia Regina Gonçalves, Erica Valessa Ramos Gomes, Karoline Rodrigues Campos
EPI_ISL_693245	UPA Santa Isabel	Instituto Adolfo Lutz, Interdiciplinary Procedures Center, Strategic Laboratory	Claudio Tavares Sacchi, Claudia Regina Gonçalves, Erica Valessa Ramos Gomes, Karoline Rodrigues Campos
EPI_ISL_693246	Laboratorio Municipal de Rio Grande da Serra	Instituto Adolfo Lutz, Interdiciplinary Procedures Center, Strategic Laboratory	Claudio Tavares Sacchi, Claudia Regina Gonçalves, Erica Valessa Ramos Gomes, Karoline Rodrigues Campos
EPI_ISL_693247	Secao Centro de Diagnostico Secedi	Instituto Adolfo Lutz, Interdiciplinary Procedures Center, Strategic Laboratory	Claudio Tavares Sacchi, Claudia Regina Gonçalves, Erica Valessa Ramos Gomes, Karoline Rodrigues Campos
EPI_ISL_693248	Centro Municipal de Epidemiologia e Imunizações	Instituto Adolfo Lutz, Interdiciplinary Procedures Center, Strategic Laboratory	Claudio Tavares Sacchi, Claudia Regina Gonçalves, Erica Valessa Ramos Gomes, Karoline Rodrigues Campos
EPI_ISL_693269, EPI_ISL_693282, EPI_ISL_693283, EPI_ISL_693284, EPI_ISL_693285, EPI_ISL_693297	unknown	Public Health Virology Laboratory, Forensic and Scientific Services (PHV-FSS)	Son Nguyen et al.
EPI_ISL_693298, EPI_ISL_693299	Department of Microbiology, Yokohama City University School of Medicine	Department of Microbiology, Yokohama City University School of Medicine	Kei Miyakawa, Ryo Saji, Kazuya Sakai, Reo Matsumura, Mototsugu Nishii, Ichiro Takeuchi, Akihide Ryo
EPI_ISL_693302, EPI_ISL_693303, EPI_ISL_693304, EPI_ISL_693305, EPI_ISL_693306	Department of Laboratory Medicine, National Taiwan University Hospital	Microbial Genomics Core Lab, National Taiwan University Centers of Genomic and Precision Medicine	Shiou-Hwei Yeh, You-Yu Lin, Ya-Yun Lai, Chiao-Ling Li, Shan-Chwen Chang, Pei-Jer Chen, Sui-Yuan Chang
EPI_ISL_693307, EPI_ISL_693308, EPI_ISL_693309, EPI_ISL_693310, EPI_ISL_693311, EPI_ISL_693312, EPI_ISL_693313, EPI_ISL_693314, EPI_ISL_693315, EPI_ISL_693316, EPI_ISL_693317, EPI_ISL_693318, EPI_ISL_693319, EPI_ISL_693320, EPI_ISL_693321, EPI_ISL_693322, EPI_ISL_693323, EPI_ISL_693324, EPI_ISL_693325, EPI_ISL_693326, EPI_ISL_693327, EPI_ISL_693328, EPI_ISL_693329, EPI_ISL_693330, EPI_ISL_693331			
see above	National Public Health Laboratory, National Centre for Infectious Diseases	National Public Health Laboratory, National Centre for Infectious Diseases	Tze Minn Mak, Sophie Octavia, Zhenyang Zhou, Lin Cui, Raymond Tzer Pin Lin
EPI_ISL_693332, EPI_ISL_693333	Lighthouse Lab in Alderley Park	Wellcome Sanger Institute for the COVID-19 Genomics UK (COG-UK) Consortium	Jacquelyn Wynn, Mairead Hyland, The Lighthouse Lab in Alderley Park and Alex Alderton, Roberto Amato, Sonia Goncalves, Ewan Harrison, David K. Jackson, Ian Johnston, Dominic Kwiatkowski, Cordelia Langford, John Sillitoe on behalf of the Wellcome Sanger Institute COVID-19 Surveillance Team
EPI_ISL_693392, EPI_ISL_693393, EPI_ISL_693394, EPI_ISL_693395, EPI_ISL_693396, EPI_ISL_693397, EPI_ISL_693398, EPI_ISL_693399, EPI_ISL_693400, EPI_ISL_693401, EPI_ISL_693402, EPI_ISL_693403, EPI_ISL_693404, EPI_ISL_693406, EPI_ISL_693407, EPI_ISL_693408, EPI_ISL_693409, EPI_ISL_693410, EPI_ISL_693411, EPI_ISL_693412, EPI_ISL_693413, EPI_ISL_693414, EPI_ISL_693415, EPI_ISL_693416, EPI_ISL_693417, EPI_ISL_693418, EPI_ISL_693419, EPI_ISL_693420, EPI_ISL_693421, EPI_ISL_693422, EPI_ISL_693423, EPI_ISL_693424, EPI_ISL_693425, EPI_ISL_693426, EPI_ISL_693427, EPI_ISL_693428, EPI_ISL_693429, EPI_ISL_693430, EPI_ISL_693431, EPI_ISL_693432, EPI_ISL_693433, EPI_ISL_693434, EPI_ISL_693435, EPI_ISL_693436, EPI_ISL_693437, EPI_ISL_693438, EPI_ISL_693439, EPI_ISL_693440, EPI_ISL_693441, EPI_ISL_693442, EPI_ISL_693443, EPI_ISL_693444, EPI_ISL_693445, EPI_ISL_693446, EPI_ISL_693447, EPI_ISL_693448, EPI_ISL_693449, EPI_ISL_693450, EPI_ISL_693451, EPI_ISL_693452, EPI_ISL_693453, EPI_ISL_693454, EPI_ISL_693455, EPI_ISL_693456, EPI_ISL_693457, EPI_ISL_693458, EPI_ISL_693459, EPI_ISL_693460, EPI_ISL_693461, EPI_ISL_693462, EPI_ISL_693463, EPI_ISL_693464, EPI_ISL_693465, EPI_ISL_693466, EPI_ISL_693467, EPI_ISL_693468, EPI_ISL_693469, EPI_ISL_693470			
see above	Respiratory Virus Unit, Microbiology Services Colindale, Public Health England	COVID-19 Genomics UK (COG-UK) Consortium	PHE Covid Sequencing Team
EPI_ISL_693471, EPI_ISL_693472, EPI_ISL_693473, EPI_ISL_693474, EPI_ISL_693475, EPI_ISL_693476, EPI_ISL_693477, EPI_ISL_693478, EPI_ISL_693479, EPI_ISL_693480, EPI_ISL_693481, EPI_ISL_693482, EPI_ISL_693483			
see above	Central Public Health Laboratory	National Public Health Laboratory, National Centre for Infectious Diseases	Tze Minn Mak, Sophie Octavia, Zhenyang Zhou, Esorom Daoni, Theresa Palou, Lin Cui, Raymond Tzer Pin Lin
EPI_ISL_693484, EPI_ISL_693485, EPI_ISL_693487	Respiratory Virus Unit, Microbiology Services Colindale, Public Health England	COVID-19 Genomics UK (COG-UK) Consortium	PHE Covid Sequencing Team
EPI_ISL_693488, EPI_ISL_693489, EPI_ISL_693490, EPI_ISL_693491, EPI_ISL_693492, EPI_ISL_693493, EPI_ISL_693494, EPI_ISL_693495, EPI_ISL_693496, EPI_ISL_693497, EPI_ISL_693498, EPI_ISL_693499, EPI_ISL_693500, EPI_ISL_693501, EPI_ISL_693502, EPI_ISL_693503, EPI_ISL_693504, EPI_ISL_693505, EPI_ISL_693506, EPI_ISL_693507, EPI_ISL_693508, EPI_ISL_693509, EPI_ISL_693510, EPI_ISL_693511, EPI_ISL_693512, EPI_ISL_693513, EPI_ISL_693514			
see above	CNR Virus des Infections Respiratoires - France SUD	CNR Virus des Infections Respiratoires - France SUD	Antonin Bal, Gregory Destras, Gwendolyne Burfin, Quentin Semanas, Martine Valette, Bruno Lina, Laurence Josset
EPI_ISL_693516, EPI_ISL_693518, EPI_ISL_693519, EPI_ISL_693520	Hospital Vila Franca de Xira	Instituto Nacional de Saude (INSA)	Borges et al
EPI_ISL_693521	Instituto Nacional de Saude (INSA)	Instituto Nacional de Saude (INSA)	Borges et al
EPI_ISL_693523, EPI_ISL_693524	Hospital Vila Franca de Xira	Instituto Nacional de Saude (INSA)	Borges et al
EPI_ISL_693525, EPI_ISL_693527, EPI_ISL_693528, EPI_ISL_693529, EPI_ISL_693530, EPI_ISL_693531, EPI_ISL_693532, EPI_ISL_693533, EPI_ISL_693540, EPI_ISL_693541, EPI_ISL_693542, EPI_ISL_693554, EPI_ISL_693555, EPI_ISL_693556, EPI_ISL_693557, EPI_ISL_693558, EPI_ISL_693559, EPI_ISL_693560, EPI_ISL_693561, EPI_ISL_693562, EPI_ISL_693563, EPI_ISL_693564, EPI_ISL_693565, EPI_ISL_693566, EPI_ISL_693567, EPI_ISL_693568, EPI_ISL_693569, EPI_ISL_693570, EPI_ISL_693571, EPI_ISL_693572, EPI_ISL_693573, EPI_ISL_693574, EPI_ISL_693575, EPI_ISL_693576, EPI_ISL_693577, EPI_ISL_693578, EPI_ISL_693579, EPI_ISL_693580, EPI_ISL_693581, EPI_ISL_693582, EPI_ISL_693583, EPI_ISL_693584, EPI_ISL_693585, EPI_ISL_693586, EPI_ISL_693587, EPI_ISL_693588, EPI_ISL_693589, EPI_ISL_693590, EPI_ISL_693591, EPI_ISL_693592, EPI_ISL_693593, EPI_ISL_693594, EPI_ISL_693595, EPI_ISL_693596, EPI_ISL_693597, EPI_ISL_693598, EPI_ISL_693599, EPI_ISL_693600, EPI_ISL_693601, EPI_ISL_693602, EPI_ISL_693603, EPI_ISL_693604, EPI_ISL_693605, EPI_ISL_693606, EPI_ISL_693607, EPI_ISL_693608, EPI_ISL_693609, EPI_ISL_693610, EPI_ISL_693611, EPI_ISL_693612, EPI_ISL_693613, EPI_ISL_693614, EPI_ISL_693615, EPI_ISL_693616, EPI_ISL_693617, EPI_ISL_693618, EPI_ISL_693619, EPI_ISL_693620, EPI_ISL_693621, EPI_ISL_693622, EPI_ISL_693623, EPI_ISL_693624, EPI_ISL_693625, EPI_ISL_693626, EPI_ISL_693627, EPI_ISL_693628, EPI_ISL_693629			
see above	Instituto Nacional de Saude (INSA)	Instituto Nacional de Saude (INSA)	Borges et al

EPI_ISL_693630, EPI_ISL_693631, EPI_ISL_693632, EPI_ISL_693633, EPI_ISL_693634, EPI_ISL_693635, EPI_ISL_693636, EPI_ISL_693637, EPI_ISL_693638, EPI_ISL_693639, EPI_ISL_693640, EPI_ISL_693641, EPI_ISL_693642, EPI_ISL_693643, EPI_ISL_693644, EPI_ISL_693645, EPI_ISL_693646, EPI_ISL_693647, EPI_ISL_693648, EPI_ISL_693649, EPI_ISL_693650, EPI_ISL_693651, EPI_ISL_693652, EPI_ISL_693653, EPI_ISL_693654, EPI_ISL_693655, EPI_ISL_693656, EPI_ISL_693657			
see above	Hospital Vila Franca de Xira	Instituto Nacional de Saude (INSA)	Borges et al
EPI_ISL_693658, EPI_ISL_693659	Washington University in St. Louis	Washington University in St. Louis	David Wang, Carey-Ann Burnham, Bijal Parikh, Scott Handley, Lindsay Droit, Stephen Tahan
EPI_ISL_693660, EPI_ISL_693661, EPI_ISL_693662, EPI_ISL_693663, EPI_ISL_693664, EPI_ISL_693665, EPI_ISL_693666, EPI_ISL_693667, EPI_ISL_693668, EPI_ISL_693669, EPI_ISL_693670, EPI_ISL_693671, EPI_ISL_693672, EPI_ISL_693673, EPI_ISL_693674, EPI_ISL_693675, EPI_ISL_693676, EPI_ISL_693677, EPI_ISL_693678, EPI_ISL_693679, EPI_ISL_693680, EPI_ISL_693681, EPI_ISL_693682, EPI_ISL_693683, EPI_ISL_693684, EPI_ISL_693685, EPI_ISL_693686, EPI_ISL_693687			
see above	The National Institute of Public Health	State Veterinary Institute Prague	Nagy,A;Jirincova,H;Trnka,D;Vecerova,J
EPI_ISL_693688, EPI_ISL_693689, EPI_ISL_693690, EPI_ISL_693691, EPI_ISL_693692, EPI_ISL_693693, EPI_ISL_693694, EPI_ISL_693697, EPI_ISL_693698, EPI_ISL_693700, EPI_ISL_693701, EPI_ISL_693702, EPI_ISL_693704, EPI_ISL_693705, EPI_ISL_693707, EPI_ISL_693708, EPI_ISL_693709, EPI_ISL_693710, EPI_ISL_693711, EPI_ISL_693712, EPI_ISL_693713, EPI_ISL_693714, EPI_ISL_693715, EPI_ISL_693728, EPI_ISL_693729, EPI_ISL_693741, EPI_ISL_693742, EPI_ISL_693743, EPI_ISL_693744, EPI_ISL_693745, EPI_ISL_693746, EPI_ISL_693748, EPI_ISL_693749, EPI_ISL_693750, EPI_ISL_693751, EPI_ISL_693752, EPI_ISL_693754, EPI_ISL_693755, EPI_ISL_693756, EPI_ISL_693757			
see above	Delaware Public Health Laboratory	Delaware Public Health Laboratory	Gregory Hovan
EPI_ISL_693758, EPI_ISL_693759, EPI_ISL_693760, EPI_ISL_693761, EPI_ISL_693762, EPI_ISL_693763, EPI_ISL_693764	Hospital	National Reference Center for Viruses of Respiratory Infections, Institut Pasteur, Paris	Marion Barbet, Sylvie Behillil, Méline Bizard, Angela Brisebarre, Camille Capel, Etienne Simon-Lorière, Vincent Enouf, Maud Vanpeene, Sylvie van der Werf, Gisèle Lagathu
EPI_ISL_693765	hospital	National Reference Center for Viruses of Respiratory Infections, Institut Pasteur, Paris	Marion Barbet, Sylvie Behillil, Méline Bizard, Angela Brisebarre, Camille Capel, Etienne Simon-Lorière, Vincent Enouf, Maud Vanpeene, Sylvie van der Werf, Gisèle Lagathu
EPI_ISL_693766, EPI_ISL_693767, EPI_ISL_693768, EPI_ISL_693769, EPI_ISL_693770, EPI_ISL_693771, EPI_ISL_693772, EPI_ISL_693773, EPI_ISL_693774	General practitioner	National Reference Center for Viruses of Respiratory Infections, Institut Pasteur, Paris	Marion Barbet, Sylvie Behillil, Méline Bizard, Angela Brisebarre, Camille Capel, Etienne Simon-Lorière, Vincent Enouf, Maud Vanpeene, Sylvie van der Werf
EPI_ISL_693775, EPI_ISL_693776, EPI_ISL_693777, EPI_ISL_693778, EPI_ISL_693779, EPI_ISL_693780, EPI_ISL_693781, EPI_ISL_693782, EPI_ISL_693783, EPI_ISL_693784, EPI_ISL_693785, EPI_ISL_693786, EPI_ISL_693787, EPI_ISL_693788, EPI_ISL_693789, EPI_ISL_693790, EPI_ISL_693791, EPI_ISL_693792, EPI_ISL_693793, EPI_ISL_693794, EPI_ISL_693795, EPI_ISL_693796, EPI_ISL_693797, EPI_ISL_693798, EPI_ISL_693799, EPI_ISL_693800, EPI_ISL_693801, EPI_ISL_693802, EPI_ISL_693803, EPI_ISL_693804, EPI_ISL_693805, EPI_ISL_693806, EPI_ISL_693807, EPI_ISL_693808, EPI_ISL_693809, EPI_ISL_693810, EPI_ISL_693811, EPI_ISL_693812, EPI_ISL_693813, EPI_ISL_693814, EPI_ISL_693815, EPI_ISL_693816, EPI_ISL_693817, EPI_ISL_693818, EPI_ISL_693819, EPI_ISL_693820, EPI_ISL_693821, EPI_ISL_693822, EPI_ISL_693823, EPI_ISL_693824, EPI_ISL_693825, EPI_ISL_693826, EPI_ISL_693827, EPI_ISL_693828, EPI_ISL_693829, EPI_ISL_693830, EPI_ISL_693831, EPI_ISL_693832, EPI_ISL_693833, EPI_ISL_693834, EPI_ISL_693835, EPI_ISL_693836, EPI_ISL_693837, EPI_ISL_693838, EPI_ISL_693839, EPI_ISL_693840, EPI_ISL_693841, EPI_ISL_693842, EPI_ISL_693843, EPI_ISL_693844, EPI_ISL_693845, EPI_ISL_693846, EPI_ISL_693847, EPI_ISL_693848, EPI_ISL_693849, EPI_ISL_693850, EPI_ISL_693851, EPI_ISL_693852, EPI_ISL_693853, EPI_ISL_693854, EPI_ISL_693855, EPI_ISL_693856, EPI_ISL_693857, EPI_ISL_693858, EPI_ISL_693859, EPI_ISL_693860, EPI_ISL_693861, EPI_ISL_693862, EPI_ISL_693863, EPI_ISL_693864, EPI_ISL_693865, EPI_ISL_693866, EPI_ISL_693867, EPI_ISL_693868, EPI_ISL_693869, EPI_ISL_693870, EPI_ISL_693871, EPI_ISL_693872, EPI_ISL_693873, EPI_ISL_693874, EPI_ISL_693875, EPI_ISL_693876, EPI_ISL_693877, EPI_ISL_693878, EPI_ISL_693879, EPI_ISL_693880, EPI_ISL_693881, EPI_ISL_693882, EPI_ISL_693883, EPI_ISL_693884, EPI_ISL_693885, EPI_ISL_693886, EPI_ISL_693887, EPI_ISL_693888, EPI_ISL_693889, EPI_ISL_693890, EPI_ISL_693891, EPI_ISL_693892, EPI_ISL_693893, EPI_ISL_693894, EPI_ISL_693896, EPI_ISL_693897, EPI_ISL_693898, EPI_ISL_693899, EPI_ISL_693900, EPI_ISL_693901, EPI_ISL_693902, EPI_ISL_693903, EPI_ISL_693904, EPI_ISL_693905, EPI_ISL_693906, EPI_ISL_693907, EPI_ISL_693908, EPI_ISL_693909, EPI_ISL_693910, EPI_ISL_693911, EPI_ISL_693912, EPI_ISL_693913, EPI_ISL_693914, EPI_ISL_693915, EPI_ISL_693916, EPI_ISL_693917, EPI_ISL_693918, EPI_ISL_693919, EPI_ISL_693920, EPI_ISL_693921, EPI_ISL_693922, EPI_ISL_693923, EPI_ISL_693924, EPI_ISL_693925, EPI_ISL_693926, EPI_ISL_693927, EPI_ISL_693928, EPI_ISL_693929, EPI_ISL_693930, EPI_ISL_693931, EPI_ISL_693932, EPI_ISL_693933, EPI_ISL_693934, EPI_ISL_693935, EPI_ISL_693936, EPI_ISL_693937, EPI_ISL_693938, EPI_ISL_693939, EPI_ISL_693940, EPI_ISL_693941, EPI_ISL_693942, EPI_ISL_693943, EPI_ISL_693944, EPI_ISL_693945, EPI_ISL_693946, EPI_ISL_693947, EPI_ISL_693948, EPI_ISL_693949, EPI_ISL_693950, EPI_ISL_693951, EPI_ISL_693952, EPI_ISL_693953, EPI_ISL_693954, EPI_ISL_693955, EPI_ISL_693956, EPI_ISL_693957, EPI_ISL_693958, EPI_ISL_693959, EPI_ISL_693960, EPI_ISL_693961, EPI_ISL_693962, EPI_ISL_693963, EPI_ISL_693964, EPI_ISL_693965, EPI_ISL_693966, EPI_ISL_693967, EPI_ISL_693968, EPI_ISL_693969, EPI_ISL_693970, EPI_ISL_693971, EPI_ISL_693972, EPI_ISL_693973, EPI_ISL_693974, EPI_ISL_693975, EPI_ISL_693976, EPI_ISL_693977, EPI_ISL_693978, EPI_ISL_693979, EPI_ISL_693980, EPI_ISL_693981, EPI_ISL_693982, EPI_ISL_693983, EPI_ISL_693984, EPI_ISL_693985, EPI_ISL_693986, EPI_ISL_693987, EPI_ISL_693988, EPI_ISL_693989, EPI_ISL_693990, EPI_ISL_693991, EPI_ISL_693992, EPI_ISL_693993, EPI_ISL_693994, EPI_ISL_693995, EPI_ISL_693996, EPI_ISL_693997, EPI_ISL_693998, EPI_ISL_693999, EPI_ISL_694000, EPI_ISL_694001, EPI_ISL_694002, EPI_ISL_694003, EPI_ISL_694004, EPI_ISL_694005, EPI_ISL_694006, EPI_ISL_694007, EPI_ISL_694008			
see above	Viollier AG	Department of Biosystems Science and Engineering, ETH Zürich	Christian Beisel, Sarah Nadeau, Chaoran Chen, Ivan Topolsky, Pedro Ferreira, Philipp Jablonski, Susana Posada-Céspedes, Tobias Schär, Ina Nissen, Natascha Santacroce, Elodie Burcklen, Christiane Beckmann, Maurice Redondo, Olivier Kobel, Christoph Noppen, Sophie Seidel, Noemie Santamaria de Souza, Niko Beerenwinkel, Tanja Stadler
EPI_ISL_694037, EPI_ISL_694039	TGen North	TGen North	Hayley D Yaglom, Marette Gebhardt, Ashlyn Pfeiffer, Mary Ellen Ormsby, Daniel Jasso-Selles, Darrin Lemmer, Megan Folkerts, Chris French, Matthew Maurer, Jolene R Bowers, David M Engelthaler
EPI_ISL_694046	Los Angeles County Public Health Laboratory	Los Angeles County Public Health Laboratory	Peera Hemarajata et al.
EPI_ISL_694377, EPI_ISL_694378, EPI_ISL_694379, EPI_ISL_694385, EPI_ISL_694386, EPI_ISL_694387, EPI_ISL_694389, EPI_ISL_694390, EPI_ISL_694391, EPI_ISL_694393, EPI_ISL_694394, EPI_ISL_694396, EPI_ISL_694397, EPI_ISL_694398, EPI_ISL_694399, EPI_ISL_694400, EPI_ISL_694401, EPI_ISL_694403, EPI_ISL_694404, EPI_ISL_694428, EPI_ISL_694429, EPI_ISL_694430, EPI_ISL_694431, EPI_ISL_694432, EPI_ISL_694433, EPI_ISL_694434, EPI_ISL_694435, EPI_ISL_694436, EPI_ISL_694437, EPI_ISL_694438, EPI_ISL_694439, EPI_ISL_694440, EPI_ISL_694441, EPI_ISL_694442, EPI_ISL_694443, EPI_ISL_694445, EPI_ISL_694446, EPI_ISL_694448, EPI_ISL_694449, EPI_ISL_694450, EPI_ISL_694451, EPI_ISL_694452, EPI_ISL_694453, EPI_ISL_694454, EPI_ISL_694455, EPI_ISL_694456, EPI_ISL_694457			
see above	TGen North	TGen North	Jolene Bowers, Megan Folkerts, Chris French, Hayley Yaglom, Ashlyn Pfeiffer, Darrin Lemmer, Dave Engelthaler, The Arizona COVID Genomics Union (ACGU)
EPI_ISL_694462, EPI_ISL_694464, EPI_ISL_694465, EPI_ISL_694467, EPI_ISL_694468, EPI_ISL_694469, EPI_ISL_694470, EPI_ISL_694471, EPI_ISL_694472, EPI_ISL_694473, EPI_ISL_694474, EPI_ISL_694475, EPI_ISL_694476, EPI_ISL_694477, EPI_ISL_694479, EPI_ISL_694480, EPI_ISL_694481, EPI_ISL_694483, EPI_ISL_694484, EPI_ISL_694485, EPI_ISL_694486, EPI_ISL_694487, EPI_ISL_694488, EPI_ISL_694489, EPI_ISL_694490, EPI_ISL_694491, EPI_ISL_694492, EPI_ISL_694493, EPI_ISL_694494, EPI_ISL_694495, EPI_ISL_694496, EPI_ISL_694497, EPI_ISL_694498, EPI_ISL_694499, EPI_ISL_694500, EPI_ISL_694501, EPI_ISL_694502, EPI_ISL_694503, EPI_ISL_694504, EPI_ISL_694505, EPI_ISL_694506, EPI_ISL_694508, EPI_ISL_694509, EPI_ISL_694510, EPI_ISL_694511, EPI_ISL_694512, EPI_ISL_694513, EPI_ISL_694514, EPI_ISL_694515, EPI_ISL_694516, EPI_ISL_694517, EPI_ISL_694518, EPI_ISL_694519, EPI_ISL_694520, EPI_ISL_694521, EPI_ISL_694522			
see above	AZ SPHL, Arizona Department of Health Services	TGen North	Jolene Bowers, Megan Folkerts, Chris French, Hayley Yaglom, Ashlyn Pfeiffer, Darrin Lemmer, Dave Engelthaler, The Arizona COVID Genomics Union (ACGU)
EPI_ISL_694601, EPI_ISL_694602, EPI_ISL_694603, EPI_ISL_694604, EPI_ISL_694605, EPI_ISL_694606, EPI_ISL_694607, EPI_ISL_694608, EPI_ISL_694609, EPI_ISL_694610, EPI_ISL_694611, EPI_ISL_694612, EPI_ISL_694614, EPI_ISL_694615, EPI_ISL_694616, EPI_ISL_694617, EPI_ISL_694618, EPI_ISL_694619, EPI_ISL_694620, EPI_ISL_694621, EPI_ISL_694622, EPI_ISL_694623, EPI_ISL_694624, EPI_ISL_694625, EPI_ISL_694626, EPI_ISL_694627, EPI_ISL_694628, EPI_ISL_694629, EPI_ISL_694630, EPI_ISL_694631, EPI_ISL_694632, EPI_ISL_694635, EPI_ISL_694636, EPI_ISL_694637, EPI_ISL_694638, EPI_ISL_694639, EPI_ISL_694640, EPI_ISL_694641, EPI_ISL_694642, EPI_ISL_694643, EPI_ISL_694644, EPI_ISL_694645, EPI_ISL_694646, EPI_ISL_694647, EPI_ISL_694648, EPI_ISL_694649, EPI_ISL_694650, EPI_ISL_694651, EPI_ISL_694652, EPI_ISL_694653, EPI_ISL_694654, EPI_ISL_694655, EPI_ISL_694656, EPI_ISL_694657, EPI_ISL_694658, EPI_ISL_694659, EPI_ISL_694660, EPI_ISL_694661, EPI_ISL_694662, EPI_ISL_694663, EPI_ISL_694664, EPI_ISL_694665, EPI_ISL_694666, EPI_ISL_694667, EPI_ISL_694668, EPI_ISL_694669, EPI_ISL_694670, EPI_ISL_694671, EPI_ISL_694672, EPI_ISL_694673, EPI_ISL_694674, EPI_ISL_694675, EPI_ISL_694676, EPI_ISL_694677, EPI_ISL_694678, EPI_ISL_694679, EPI_ISL_694680, EPI_ISL_694681, EPI_ISL_694682, EPI_ISL_694683, EPI_ISL_694684, EPI_ISL_694685, EPI_ISL_694686, EPI_ISL_694687, EPI_ISL_694688, EPI_ISL_694689, EPI_ISL_694690, EPI_ISL_694691, EPI_ISL_694692, EPI_ISL_694693, EPI_ISL_694695, EPI_ISL_694696, EPI_ISL_694697, EPI_ISL_694698, EPI_ISL_694699, EPI_ISL_694700, EPI_ISL_694701, EPI_ISL_694702, EPI_ISL_694703, EPI_ISL_694704, EPI_ISL_694706, EPI_ISL_694707, EPI_ISL_694708, EPI_ISL_694709, EPI_ISL_694710, EPI_ISL_694711, EPI_ISL_694712, EPI_ISL_694713, EPI_ISL_694714, EPI_ISL_694716, EPI_ISL_694717, EPI_ISL_694718, EPI_ISL_694719, EPI_ISL_694720, EPI_ISL_694721, EPI_ISL_694722, EPI_ISL_694723			
see above	TGen North	TGen North	Jolene Bowers, Megan Folkerts, Chris French, Hayley Yaglom, Ashlyn Pfeiffer, Darrin Lemmer, Dave Engelthaler, The Arizona COVID Genomics Union (ACGU)
EPI_ISL_694724, EPI_ISL_694726, EPI_ISL_694727, EPI_ISL_694728, EPI_ISL_694729, EPI_ISL_694730, EPI_ISL_694731, EPI_ISL_694732, EPI_ISL_694733, EPI_ISL_694734, EPI_ISL_694736, EPI_ISL_694737, EPI_ISL_694738, EPI_ISL_694739, EPI_ISL_694740, EPI_ISL_694741, EPI_ISL_694742, EPI_ISL_694743, EPI_ISL_694744, EPI_ISL_694745, EPI_ISL_694746, EPI_ISL_694747, EPI_ISL_694748, EPI_ISL_694750, EPI_ISL_694751, EPI_ISL_694752, EPI_ISL_694753, EPI_ISL_694754, EPI_ISL_694755, EPI_ISL_694756, EPI_ISL_694757, EPI_ISL_694758, EPI_ISL_694759, EPI_ISL_694761, EPI_ISL_694762, EPI_ISL_694763, EPI_ISL_694764, EPI_ISL_694765, EPI_ISL_694766, EPI_ISL_694767, EPI_ISL_694768, EPI_ISL_694769, EPI_ISL_694771, EPI_ISL_694772, EPI_ISL_694773, EPI_ISL_694774, EPI_ISL_694775, EPI_ISL_694776, EPI_ISL_694779, EPI_ISL_694780, EPI_ISL_694781, EPI_ISL_694782, EPI_ISL_694783, EPI_ISL_694784, EPI_ISL_694785, EPI_ISL_694786, EPI_ISL_694787, EPI_ISL_694788, EPI_ISL_694789, EPI_ISL_694790, EPI_ISL_694791, EPI_ISL_694792, EPI_ISL_694793, EPI_ISL_694794, EPI_ISL_694796			
see above	AZ SPHL, Arizona Department of Health Services	TGen North	Jolene Bowers, Megan Folkerts, Chris French, Hayley Yaglom, Ashlyn Pfeiffer, Darrin Lemmer, Dave Engelthaler, The Arizona COVID Genomics Union (ACGU)
EPI_ISL_694797, EPI_ISL_694798	TGen North	TGen North	Jolene Bowers, Megan Folkerts, Chris French, Hayley Yaglom, Ashlyn Pfeiffer, Darrin Lemmer, Dave Engelthaler, The Arizona COVID Genomics Union (ACGU)
EPI_ISL_694799, EPI_ISL_694800, EPI_ISL_694801, EPI_ISL_694802, EPI_ISL_694805, EPI_ISL_694806, EPI_ISL_694808, EPI_ISL_694809, EPI_ISL_694810, EPI_ISL_694811, EPI_ISL_694812, EPI_ISL_694813, EPI_ISL_694814, EPI_ISL_694816, EPI_ISL_694818, EPI_ISL_694819, EPI_ISL_694821, EPI_ISL_694822, EPI_ISL_694823, EPI_ISL_694824, EPI_ISL_694825, EPI_ISL_694826, EPI_ISL_694827, EPI_ISL_694828, EPI_ISL_694829, EPI_ISL_694830, EPI_ISL_694831, EPI_ISL_694832, EPI_ISL_694833, EPI_ISL_694834, EPI_ISL_694835, EPI_ISL_694836, EPI_ISL_694837, EPI_ISL_694838, EPI_ISL_694839, EPI_ISL_694840, EPI_ISL_694841, EPI_ISL_694842, EPI_ISL_694844			
see above	AZ SPHL, Arizona Department of Health Services	TGen North	Jolene Bowers, Megan Folkerts, Chris French, Hayley Yaglom, Ashlyn Pfeiffer, Darrin Lemmer, Dave Engelthaler, The Arizona COVID Genomics Union (ACGU)
EPI_ISL_694845, EPI_ISL_694846, EPI_ISL_694847, EPI_ISL_694848, EPI_ISL_694849, EPI_ISL_694850, EPI_ISL_694851, EPI_ISL_694853, EPI_ISL_694855, EPI_ISL_694856, EPI_ISL_694857, EPI_ISL_694858, EPI_ISL_694859, EPI_ISL_694860, EPI_ISL_694861, EPI_ISL_694862, EPI_ISL_694864, EPI_ISL_694865,			

EPI_ISL_694866, EPI_ISL_694867, EPI_ISL_694868, EPI_ISL_694869, EPI_ISL_694870, EPI_ISL_694871, EPI_ISL_694872, EPI_ISL_694873, EPI_ISL_694876, EPI_ISL_694879, EPI_ISL_694880, EPI_ISL_694881, EPI_ISL_694882, EPI_ISL_694883, EPI_ISL_694884

see above | TGen North | TGen North | Jolene Bowers, Megan Folkerts, Chris French, Hayley Yaglom, Ashlyn Pfeiffer, Darrin Lemmer, Dave Engelthaler, The Arizona COVID Genomics Union (ACGU)

EPI_ISL_694885, EPI_ISL_694886, EPI_ISL_694887, EPI_ISL_694888, EPI_ISL_694889, EPI_ISL_694890, EPI_ISL_694891, EPI_ISL_694892, EPI_ISL_694893, EPI_ISL_694894, EPI_ISL_694895, EPI_ISL_694896, EPI_ISL_694897, EPI_ISL_694898, EPI_ISL_694899, EPI_ISL_694900, EPI_ISL_694901, EPI_ISL_694902, EPI_ISL_694903, EPI_ISL_694905, EPI_ISL_694906, EPI_ISL_694907, EPI_ISL_694908, EPI_ISL_694909, EPI_ISL_694910, EPI_ISL_694911, EPI_ISL_694912, EPI_ISL_694913, EPI_ISL_694914, EPI_ISL_694916, EPI_ISL_694917, EPI_ISL_694918, EPI_ISL_694919, EPI_ISL_694920, EPI_ISL_694921, EPI_ISL_694922, EPI_ISL_694923, EPI_ISL_694924, EPI_ISL_694925, EPI_ISL_694926, EPI_ISL_694927, EPI_ISL_694928, EPI_ISL_694929, EPI_ISL_694930, EPI_ISL_694931, EPI_ISL_694932, EPI_ISL_694933, EPI_ISL_694934, EPI_ISL_694935, EPI_ISL_694936, EPI_ISL_694937, EPI_ISL_694938, EPI_ISL_694939, EPI_ISL_694940, EPI_ISL_694941, EPI_ISL_694942, EPI_ISL_694943, EPI_ISL_694945, EPI_ISL_694946, EPI_ISL_694947, EPI_ISL_694948, EPI_ISL_694949, EPI_ISL_694950

see above | AZ SPHL, Arizona Department of Health Services | TGen North | Jolene Bowers, Megan Folkerts, Chris French, Hayley Yaglom, Ashlyn Pfeiffer, Darrin Lemmer, Dave Engelthaler, The Arizona COVID Genomics Union (ACGU)

EPI_ISL_694951, EPI_ISL_694953, EPI_ISL_694956, EPI_ISL_694957, EPI_ISL_694959, EPI_ISL_694960, EPI_ISL_694961, EPI_ISL_694962, EPI_ISL_694963, EPI_ISL_694964, EPI_ISL_694965, EPI_ISL_694966, EPI_ISL_694969, EPI_ISL_694970, EPI_ISL_694971, EPI_ISL_694972, EPI_ISL_694973, EPI_ISL_694974, EPI_ISL_694975, EPI_ISL_694976, EPI_ISL_694977, EPI_ISL_694978, EPI_ISL_694980, EPI_ISL_694981, EPI_ISL_694982, EPI_ISL_694983, EPI_ISL_694984, EPI_ISL_694987, EPI_ISL_694988, EPI_ISL_694989, EPI_ISL_694991, EPI_ISL_694994, EPI_ISL_694995, EPI_ISL_694996, EPI_ISL_694997, EPI_ISL_694998, EPI_ISL_694999, EPI_ISL_695000, EPI_ISL_695001, EPI_ISL_695002, EPI_ISL_695003, EPI_ISL_695004, EPI_ISL_695005, EPI_ISL_695006, EPI_ISL_695007, EPI_ISL_695009, EPI_ISL_695011, EPI_ISL_695012, EPI_ISL_695013, EPI_ISL_695014, EPI_ISL_695015, EPI_ISL_695017, EPI_ISL_695018, EPI_ISL_695019, EPI_ISL_695020, EPI_ISL_695021, EPI_ISL_695022, EPI_ISL_695024, EPI_ISL_695025, EPI_ISL_695026, EPI_ISL_695027, EPI_ISL_695028, EPI_ISL_695029, EPI_ISL_695030, EPI_ISL_695031, EPI_ISL_695032, EPI_ISL_695033, EPI_ISL_695034, EPI_ISL_695035, EPI_ISL_695036, EPI_ISL_695037, EPI_ISL_695039, EPI_ISL_695041, EPI_ISL_695042, EPI_ISL_695043, EPI_ISL_695045, EPI_ISL_695047, EPI_ISL_695049, EPI_ISL_695050, EPI_ISL_695051, EPI_ISL_695053, EPI_ISL_695054, EPI_ISL_695055, EPI_ISL_695056, EPI_ISL_695057, EPI_ISL_695061, EPI_ISL_695062, EPI_ISL_695063, EPI_ISL_695064, EPI_ISL_695065, EPI_ISL_695066, EPI_ISL_695068, EPI_ISL_695069, EPI_ISL_695071, EPI_ISL_695072, EPI_ISL_695073, EPI_ISL_695074, EPI_ISL_695075, EPI_ISL_695076, EPI_ISL_695077, EPI_ISL_695078, EPI_ISL_695080, EPI_ISL_695081, EPI_ISL_695082, EPI_ISL_695083, EPI_ISL_695084, EPI_ISL_695085, EPI_ISL_695086, EPI_ISL_695087, EPI_ISL_695088, EPI_ISL_695089, EPI_ISL_695091, EPI_ISL_695092, EPI_ISL_695093, EPI_ISL_695095, EPI_ISL_695096, EPI_ISL_695097, EPI_ISL_695099, EPI_ISL_695100, EPI_ISL_695102

see above | TGen North | TGen North | Jolene Bowers, Megan Folkerts, Chris French, Hayley Yaglom, Ashlyn Pfeiffer, Darrin Lemmer, Dave Engelthaler, The Arizona COVID Genomics Union (ACGU)

EPI_ISL_695103, EPI_ISL_695104, EPI_ISL_695105, EPI_ISL_695106, EPI_ISL_695107, EPI_ISL_695108, EPI_ISL_695109, EPI_ISL_695110, EPI_ISL_695111, EPI_ISL_695112, EPI_ISL_695113, EPI_ISL_695114, EPI_ISL_695115, EPI_ISL_695116, EPI_ISL_695117, EPI_ISL_695118, EPI_ISL_695119, EPI_ISL_695120, EPI_ISL_695121, EPI_ISL_695122, EPI_ISL_695124, EPI_ISL_695126, EPI_ISL_695127, EPI_ISL_695128, EPI_ISL_695129, EPI_ISL_695130, EPI_ISL_695131, EPI_ISL_695132, EPI_ISL_695133, EPI_ISL_695134, EPI_ISL_695135, EPI_ISL_695136, EPI_ISL_695137, EPI_ISL_695138, EPI_ISL_695139, EPI_ISL_695140, EPI_ISL_695143, EPI_ISL_695144, EPI_ISL_695145, EPI_ISL_695146, EPI_ISL_695147, EPI_ISL_695148, EPI_ISL_695149, EPI_ISL_695150, EPI_ISL_695151, EPI_ISL_695152, EPI_ISL_695153, EPI_ISL_695154, EPI_ISL_695155, EPI_ISL_695156, EPI_ISL_695157, EPI_ISL_695158, EPI_ISL_695159, EPI_ISL_695160, EPI_ISL_695161, EPI_ISL_695162, EPI_ISL_695163, EPI_ISL_695164, EPI_ISL_695165, EPI_ISL_695166, EPI_ISL_695167, EPI_ISL_695168, EPI_ISL_695169, EPI_ISL_695170, EPI_ISL_695171, EPI_ISL_695172, EPI_ISL_695173, EPI_ISL_695176, EPI_ISL_695177, EPI_ISL_695178, EPI_ISL_695181, EPI_ISL_695184, EPI_ISL_695185, EPI_ISL_695186, EPI_ISL_695189, EPI_ISL_695190, EPI_ISL_695191, EPI_ISL_695192, EPI_ISL_695193, EPI_ISL_695194, EPI_ISL_695198, EPI_ISL_695202, EPI_ISL_695203, EPI_ISL_695205, EPI_ISL_695206, EPI_ISL_695207, EPI_ISL_695209, EPI_ISL_695211, EPI_ISL_695212, EPI_ISL_695214, EPI_ISL_695215, EPI_ISL_695220, EPI_ISL_695221, EPI_ISL_695222, EPI_ISL_695223, EPI_ISL_695224, EPI_ISL_695225, EPI_ISL_695226, EPI_ISL_695228

see above | AZ SPHL, Arizona Department of Health Services | TGen North | Jolene Bowers, Megan Folkerts, Chris French, Hayley Yaglom, Ashlyn Pfeiffer, Darrin Lemmer, Dave Engelthaler, The Arizona COVID Genomics Union (ACGU)

EPI_ISL_695230, EPI_ISL_695231, EPI_ISL_695232, EPI_ISL_695233, EPI_ISL_695235, EPI_ISL_695236, EPI_ISL_695237, EPI_ISL_695238, EPI_ISL_695239, EPI_ISL_695240, EPI_ISL_695241, EPI_ISL_695242, EPI_ISL_695243, EPI_ISL_695244, EPI_ISL_695245, EPI_ISL_695246, EPI_ISL_695247, EPI_ISL_695248, EPI_ISL_695249, EPI_ISL_695250, EPI_ISL_695251, EPI_ISL_695252, EPI_ISL_695253, EPI_ISL_695254, EPI_ISL_695255, EPI_ISL_695256, EPI_ISL_695257, EPI_ISL_695258, EPI_ISL_695259, EPI_ISL_695260, EPI_ISL_695261, EPI_ISL_695262, EPI_ISL_695263, EPI_ISL_695264, EPI_ISL_695265, EPI_ISL_695267, EPI_ISL_695268, EPI_ISL_695269, EPI_ISL_695270, EPI_ISL_695271, EPI_ISL_695272, EPI_ISL_695273, EPI_ISL_695274, EPI_ISL_695275, EPI_ISL_695276, EPI_ISL_695277, EPI_ISL_695278, EPI_ISL_695279, EPI_ISL_695280, EPI_ISL_695281, EPI_ISL_695282, EPI_ISL_695283, EPI_ISL_695284, EPI_ISL_695285, EPI_ISL_695286, EPI_ISL_695287, EPI_ISL_695288, EPI_ISL_695289, EPI_ISL_695290, EPI_ISL_695291, EPI_ISL_695293, EPI_ISL_695294, EPI_ISL_695295, EPI_ISL_695296, EPI_ISL_695297, EPI_ISL_695298, EPI_ISL_695299, EPI_ISL_695300, EPI_ISL_695301, EPI_ISL_695302, EPI_ISL_695303, EPI_ISL_695304, EPI_ISL_695305, EPI_ISL_695306, EPI_ISL_695307, EPI_ISL_695308, EPI_ISL_695309, EPI_ISL_695310, EPI_ISL_695311, EPI_ISL_695312, EPI_ISL_695313, EPI_ISL_695315

see above | TGen North | TGen North | Jolene Bowers, Megan Folkerts, Chris French, Hayley Yaglom, Ashlyn Pfeiffer, Darrin Lemmer, Dave Engelthaler, The Arizona COVID Genomics Union (ACGU)

EPI_ISL_695318, EPI_ISL_695319, EPI_ISL_695322, EPI_ISL_695323, EPI_ISL_695325, EPI_ISL_695326, EPI_ISL_695328, EPI_ISL_695330, EPI_ISL_695333, EPI_ISL_695334, EPI_ISL_695335, EPI_ISL_695336, EPI_ISL_695338, EPI_ISL_695339, EPI_ISL_695340, EPI_ISL_695342, EPI_ISL_695343, EPI_ISL_695345, EPI_ISL_695346, EPI_ISL_695350, EPI_ISL_695351, EPI_ISL_695352, EPI_ISL_695354, EPI_ISL_695355, EPI_ISL_695356, EPI_ISL_695359, EPI_ISL_695360, EPI_ISL_695361, EPI_ISL_695362, EPI_ISL_695364, EPI_ISL_695365, EPI_ISL_695367, EPI_ISL_695368, EPI_ISL_695369, EPI_ISL_695370

see above | AZ SPHL, Arizona Department of Health Services | TGen North | Jolene Bowers, Megan Folkerts, Chris French, Hayley Yaglom, Ashlyn Pfeiffer, Darrin Lemmer, Dave Engelthaler, The Arizona COVID Genomics Union (ACGU)

EPI_ISL_695372, EPI_ISL_695373, EPI_ISL_695374, EPI_ISL_695376, EPI_ISL_695377, EPI_ISL_695378, EPI_ISL_695379, EPI_ISL_695380, EPI_ISL_695381, EPI_ISL_695383, EPI_ISL_695384, EPI_ISL_695386, EPI_ISL_695387, EPI_ISL_695389, EPI_ISL_695392

see above | TGen North | TGen North | Jolene Bowers, Megan Folkerts, Chris French, Hayley Yaglom, Ashlyn Pfeiffer, Darrin Lemmer, Dave Engelthaler, The Arizona COVID Genomics Union (ACGU)

EPI_ISL_695394, EPI_ISL_695395, EPI_ISL_695396, EPI_ISL_695397, EPI_ISL_695398, EPI_ISL_695399, EPI_ISL_695401, EPI_ISL_695402, EPI_ISL_695403, EPI_ISL_695406, EPI_ISL_695408, EPI_ISL_695410, EPI_ISL_695411, EPI_ISL_695412, EPI_ISL_695415, EPI_ISL_695416, EPI_ISL_695417, EPI_ISL_695419, EPI_ISL_695422, EPI_ISL_695423, EPI_ISL_695425, EPI_ISL_695426, EPI_ISL_695427, EPI_ISL_695428, EPI_ISL_695429, EPI_ISL_695430, EPI_ISL_695431, EPI_ISL_695432, EPI_ISL_695433, EPI_ISL_695434, EPI_ISL_695435, EPI_ISL_695436, EPI_ISL_695437, EPI_ISL_695438, EPI_ISL_695440, EPI_ISL_695442, EPI_ISL_695445, EPI_ISL_695446, EPI_ISL_695447, EPI_ISL_695448, EPI_ISL_695449, EPI_ISL_695450, EPI_ISL_695451, EPI_ISL_695452, EPI_ISL_695453, EPI_ISL_695454, EPI_ISL_695455, EPI_ISL_695456, EPI_ISL_695458, EPI_ISL_695459, EPI_ISL_695460, EPI_ISL_695461, EPI_ISL_695462, EPI_ISL_695463, EPI_ISL_695465, EPI_ISL_695466, EPI_ISL_695467, EPI_ISL_695468, EPI_ISL_695469, EPI_ISL_695470, EPI_ISL_695472, EPI_ISL_695473, EPI_ISL_695474, EPI_ISL_695475, EPI_ISL_695476, EPI_ISL_695477, EPI_ISL_695478, EPI_ISL_695479, EPI_ISL_695480, EPI_ISL_695481, EPI_ISL_695482, EPI_ISL_695483, EPI_ISL_695484, EPI_ISL_695485, EPI_ISL_695486, EPI_ISL_695487, EPI_ISL_695488, EPI_ISL_695489, EPI_ISL_695490, EPI_ISL_695491, EPI_ISL_695492, EPI_ISL_695493, EPI_ISL_695494, EPI_ISL_695495, EPI_ISL_695496, EPI_ISL_695497, EPI_ISL_695498, EPI_ISL_695500, EPI_ISL_695501, EPI_ISL_695502, EPI_ISL_695503, EPI_ISL_695506, EPI_ISL_695508, EPI_ISL_695509, EPI_ISL_695510, EPI_ISL_695512, EPI_ISL_695513, EPI_ISL_695514, EPI_ISL_695515, EPI_ISL_695516, EPI_ISL_695517, EPI_ISL_695519

see above | AZ SPHL, Arizona Department of Health Services | TGen North | Jolene Bowers, Megan Folkerts, Chris French, Hayley Yaglom, Ashlyn Pfeiffer, Darrin Lemmer, Dave Engelthaler, The Arizona COVID Genomics Union (ACGU)

EPI_ISL_695521, EPI_ISL_695522, EPI_ISL_695523, EPI_ISL_695524, EPI_ISL_695525, EPI_ISL_695526, EPI_ISL_695527, EPI_ISL_695528, EPI_ISL_695529, EPI_ISL_695531, EPI_ISL_695532, EPI_ISL_695533, EPI_ISL_695535, EPI_ISL_695536, EPI_ISL_695537, EPI_ISL_695538, EPI_ISL_695539, EPI_ISL_695541, EPI_ISL_695542, EPI_ISL_695543, EPI_ISL_695544, EPI_ISL_695545, EPI_ISL_695546, EPI_ISL_695547, EPI_ISL_695548, EPI_ISL_695549, EPI_ISL_695550, EPI_ISL_695552, EPI_ISL_695553, EPI_ISL_695555, EPI_ISL_695556, EPI_ISL_695557, EPI_ISL_695558, EPI_ISL_695559, EPI_ISL_695560, EPI_ISL_695561, EPI_ISL_695562

see above | TGen North | TGen North | Jolene Bowers, Megan Folkerts, Chris French, Hayley Yaglom, Ashlyn Pfeiffer, Darrin Lemmer, Dave Engelthaler, The Arizona COVID Genomics Union (ACGU)

EPI_ISL_695563, EPI_ISL_695564, EPI_ISL_695565, EPI_ISL_695566, EPI_ISL_695567, EPI_ISL_695568, EPI_ISL_695570, EPI_ISL_695571, EPI_ISL_695573, EPI_ISL_695574, EPI_ISL_695575, EPI_ISL_695576, EPI_ISL_695577, EPI_ISL_695578, EPI_ISL_695579, EPI_ISL_695581, EPI_ISL_695582, EPI_ISL_695583, EPI_ISL_695584, EPI_ISL_695585, EPI_ISL_695586, EPI_ISL_695587, EPI_ISL_695588, EPI_ISL_695589, EPI_ISL_695590, EPI_ISL_695591, EPI_ISL_695592, EPI_ISL_695593, EPI_ISL_695594, EPI_ISL_695595, EPI_ISL_695596, EPI_ISL_695598, EPI_ISL_695599, EPI_ISL_695600, EPI_ISL_695601, EPI_ISL_695602, EPI_ISL_695603, EPI_ISL_695605, EPI_ISL_695606, EPI_ISL_695607, EPI_ISL_695608, EPI_ISL_695610, EPI_ISL_695611, EPI_ISL_695612, EPI_ISL_695613

see above | AZ SPHL, Arizona Department of Health Services | TGen North | Jolene Bowers, Megan Folkerts, Chris French, Hayley Yaglom, Ashlyn Pfeiffer, Darrin Lemmer, Dave Engelthaler, The Arizona COVID Genomics Union (ACGU)

EPI_ISL_695616, EPI_ISL_695618, EPI_ISL_695621, EPI_ISL_695622, EPI_ISL_695623, EPI_ISL_695624, EPI_ISL_695626, EPI_ISL_695627, EPI_ISL_695628, EPI_ISL_695629, EPI_ISL_695632, EPI_ISL_695633, EPI_ISL_695634, EPI_ISL_695635, EPI_ISL_695637, EPI_ISL_695638, EPI_ISL_695639

see above | TGen North | TGen North | Jolene Bowers, Megan Folkerts, Chris French, Hayley Yaglom, Ashlyn Pfeiffer, Darrin Lemmer, Dave Engelthaler, The Arizona COVID Genomics Union (ACGU)

EPI_ISL_695642, EPI_ISL_695644, EPI_ISL_695646, EPI_ISL_695647, EPI_ISL_695649, EPI_ISL_695650, EPI_ISL_695653, EPI_ISL_695654, EPI_ISL_695655 | AZ SPHL, Arizona Department of Health Services | TGen North | Jolene Bowers, Megan Folkerts, Chris French, Hayley Yaglom, Ashlyn Pfeiffer, Darrin Lemmer, Dave Engelthaler, The Arizona COVID Genomics Union (ACGU)

EPI_ISL_695656, EPI_ISL_695657, EPI_ISL_695659, EPI_ISL_695660, EPI_ISL_695662, EPI_ISL_695663, EPI_ISL_695664 | TGen North | TGen North | Jolene Bowers, Megan Folkerts, Chris French, Hayley Yaglom, Ashlyn Pfeiffer, Darrin Lemmer, Dave Engelthaler, The Arizona COVID Genomics Union (ACGU)

EPI_ISL_695665, EPI_ISL_695666, EPI_ISL_695667, EPI_ISL_695668, EPI_ISL_695669, EPI_ISL_695671, EPI_ISL_695672, EPI_ISL_695673, EPI_ISL_695675, EPI_ISL_695676, EPI_ISL_695679, EPI_ISL_695680, EPI_ISL_695683, EPI_ISL_695684, EPI_ISL_695685, EPI_ISL_695686, EPI_ISL_695687, EPI_ISL_695688, EPI_ISL_695689, EPI_ISL_695690, EPI_ISL_695691, EPI_ISL_695692, EPI_ISL_695693, EPI_ISL_695694, EPI_ISL_695696, EPI_ISL_695697, EPI_ISL_695700, EPI_ISL_695701

see above | AZ SPHL, Arizona Department of Health Services | TGen North | Jolene Bowers, Megan Folkerts, Chris French, Hayley Yaglom, Ashlyn Pfeiffer, Darrin Lemmer, Dave Engelthaler, The Arizona COVID Genomics Union (ACGU)

EPI_ISL_695702, EPI_ISL_695705, EPI_ISL_695706, EPI_ISL_695707, EPI_ISL_695708, EPI_ISL_695710	TGen North	TGen North	Jolene Bowers, Megan Folkerts, Chris French, Hayley Yaglom, Ashlyn Pfeiffer, Darrin Lemmer, Dave Engelthaler, The Arizona COVID Genomics Union (ACGU)
EPI_ISL_695712, EPI_ISL_695713, EPI_ISL_695714, EPI_ISL_695715, EPI_ISL_695716, EPI_ISL_695717, EPI_ISL_695718, EPI_ISL_695719, EPI_ISL_695720, EPI_ISL_695721, EPI_ISL_695722, EPI_ISL_695723, EPI_ISL_695724, EPI_ISL_695725, EPI_ISL_695726, EPI_ISL_695727, EPI_ISL_695728, EPI_ISL_695730, EPI_ISL_695731, EPI_ISL_695735, EPI_ISL_695736, EPI_ISL_695737, EPI_ISL_695738, EPI_ISL_695739, EPI_ISL_695740, EPI_ISL_695741, EPI_ISL_695742, EPI_ISL_695743, EPI_ISL_695744, EPI_ISL_695745, EPI_ISL_695746, EPI_ISL_695747, EPI_ISL_695749, EPI_ISL_695752			
see above	AZ SPHL, Arizona Department of Health Services	TGen North	Jolene Bowers, Megan Folkerts, Chris French, Hayley Yaglom, Ashlyn Pfeiffer, Darrin Lemmer, Dave Engelthaler, The Arizona COVID Genomics Union (ACGU)
EPI_ISL_695753, EPI_ISL_695754	TGen North	TGen North	Jolene Bowers, Megan Folkerts, Chris French, Hayley Yaglom, Ashlyn Pfeiffer, Darrin Lemmer, Dave Engelthaler, The Arizona COVID Genomics Union (ACGU)
EPI_ISL_695830, EPI_ISL_695831, EPI_ISL_695832, EPI_ISL_695833, EPI_ISL_695834, EPI_ISL_695835, EPI_ISL_695836, EPI_ISL_695837, EPI_ISL_695838	AZ SPHL, Arizona Department of Health Services	TGen North	Jolene Bowers, Megan Folkerts, Chris French, Hayley Yaglom, Ashlyn Pfeiffer, Darrin Lemmer, Dave Engelthaler, The Arizona COVID Genomics Union (ACGU)
EPI_ISL_696452	Thembalethu CDC wc THC & NHLS/UCT	KRISP, KZN Research Innovation and Sequencing Platform	Arash Iranzadeh, Deelan Doolabh, Lynn Tyers, Bruna Galvao, Innocent Mudau, Marvin Hsiao, Kruger Marais, Jennifer Giandhari, Sureshnee Pillay, Houriiyah Tegally, Emanuel James San, Tulio de Oliveira, Diana Hardie, Stephen Korsman, Carolyn Williamson
EPI_ISL_696453	Pacaltsdorp Clinic wc PAC & NHLS/UCT	KRISP, KZN Research Innovation and Sequencing Platform	Arash Iranzadeh, Deelan Doolabh, Lynn Tyers, Bruna Galvao, Innocent Mudau, Marvin Hsiao, Kruger Marais, Jennifer Giandhari, Sureshnee Pillay, Houriiyah Tegally, Emanuel James San, Tulio de Oliveira, Diana Hardie, Stephen Korsman, Carolyn Williamson
EPI_ISL_696454	Plettenberg Bay Clinic wc PLC & NHLS/UCT	KRISP, KZN Research Innovation and Sequencing Platform	Arash Iranzadeh, Deelan Doolabh, Lynn Tyers, Bruna Galvao, Innocent Mudau, Marvin Hsiao, Kruger Marais, Jennifer Giandhari, Sureshnee Pillay, Houriiyah Tegally, Emanuel James San, Tulio de Oliveira, Diana Hardie, Stephen Korsman, Carolyn Williamson
EPI_ISL_696455	Thembalethu CDC wc THC & NHLS/UCT	KRISP, KZN Research Innovation and Sequencing Platform	Arash Iranzadeh, Deelan Doolabh, Lynn Tyers, Bruna Galvao, Innocent Mudau, Marvin Hsiao, Kruger Marais, Jennifer Giandhari, Sureshnee Pillay, Houriiyah Tegally, Emanuel James San, Tulio de Oliveira, Diana Hardie, Stephen Korsman, Carolyn Williamson
EPI_ISL_696456	Pacaltsdorp Clinic wc PAC & NHLS/UCT	KRISP, KZN Research Innovation and Sequencing Platform	Arash Iranzadeh, Deelan Doolabh, Lynn Tyers, Bruna Galvao, Innocent Mudau, Marvin Hsiao, Kruger Marais, Jennifer Giandhari, Sureshnee Pillay, Houriiyah Tegally, Emanuel James San, Tulio de Oliveira, Diana Hardie, Stephen Korsman, Carolyn Williamson
EPI_ISL_696457	Hornlee Clinic wc HLC & NHLS/UCT	KRISP, KZN Research Innovation and Sequencing Platform	Arash Iranzadeh, Deelan Doolabh, Lynn Tyers, Bruna Galvao, Innocent Mudau, Marvin Hsiao, Kruger Marais, Jennifer Giandhari, Sureshnee Pillay, Houriiyah Tegally, Emanuel James San, Tulio de Oliveira, Diana Hardie, Stephen Korsman, Carolyn Williamson
EPI_ISL_696458	George Hospital wc GRH & NHLS/UCT	KRISP, KZN Research Innovation and Sequencing Platform	Arash Iranzadeh, Deelan Doolabh, Lynn Tyers, Bruna Galvao, Innocent Mudau, Marvin Hsiao, Kruger Marais, Jennifer Giandhari, Sureshnee Pillay, Houriiyah Tegally, Emanuel James San, Tulio de Oliveira, Diana Hardie, Stephen Korsman, Carolyn Williamson
EPI_ISL_696459	Pacaltsdorp Clinic wc PAC & NHLS/UCT	KRISP, KZN Research Innovation and Sequencing Platform	Arash Iranzadeh, Deelan Doolabh, Lynn Tyers, Bruna Galvao, Innocent Mudau, Marvin Hsiao, Kruger Marais, Jennifer Giandhari, Sureshnee Pillay, Houriiyah Tegally, Emanuel James San, Tulio de Oliveira, Diana Hardie, Stephen Korsman, Carolyn Williamson
EPI_ISL_696460	Kranshoek Clinic wc KSH & NHLS/UCT	KRISP, KZN Research Innovation and Sequencing Platform	Arash Iranzadeh, Deelan Doolabh, Lynn Tyers, Bruna Galvao, Innocent Mudau, Marvin Hsiao, Kruger Marais, Jennifer Giandhari, Sureshnee Pillay, Houriiyah Tegally, Emanuel James San, Tulio de Oliveira, Diana Hardie, Stephen Korsman, Carolyn Williamson
EPI_ISL_696461	Thembalethu CDC wc THC & NHLS/UCT	KRISP, KZN Research Innovation and Sequencing Platform	Arash Iranzadeh, Deelan Doolabh, Lynn Tyers, Bruna Galvao, Innocent Mudau, Marvin Hsiao, Kruger Marais, Jennifer Giandhari, Sureshnee Pillay, Houriiyah Tegally, Emanuel James San, Tulio de Oliveira, Diana Hardie, Stephen Korsman, Carolyn Williamson
EPI_ISL_696462	Sedgefield Clinic wc SGE & NHLS/UCT	KRISP, KZN Research Innovation and Sequencing Platform	Arash Iranzadeh, Deelan Doolabh, Lynn Tyers, Bruna Galvao, Innocent Mudau, Marvin Hsiao, Kruger Marais, Jennifer Giandhari, Sureshnee Pillay, Houriiyah Tegally, Emanuel James San, Tulio de Oliveira, Diana Hardie, Stephen Korsman, Carolyn Williamson
EPI_ISL_696463	Knysna Hospital wc KNY & NHLS/UCT	KRISP, KZN Research Innovation and Sequencing Platform	Arash Iranzadeh, Deelan Doolabh, Lynn Tyers, Bruna Galvao, Innocent Mudau, Marvin Hsiao, Kruger Marais, Jennifer Giandhari, Sureshnee Pillay, Houriiyah Tegally, Emanuel James San, Tulio de Oliveira, Diana Hardie, Stephen Korsman, Carolyn Williamson
EPI_ISL_696464	Groote Schuur Hospital wc GSH & NHLS/UCT	KRISP, KZN Research Innovation and Sequencing Platform	Arash Iranzadeh, Deelan Doolabh, Lynn Tyers, Bruna Galvao, Innocent Mudau, Marvin Hsiao, Kruger Marais, Jennifer Giandhari, Sureshnee Pillay, Houriiyah Tegally, Emanuel James San, Tulio de Oliveira, Diana Hardie, Stephen Korsman, Carolyn Williamson
EPI_ISL_696465	Kwanokuthula CDC wc KWA & NHLS/UCT	KRISP, KZN Research Innovation and Sequencing Platform	Arash Iranzadeh, Deelan Doolabh, Lynn Tyers, Bruna Galvao, Innocent Mudau, Marvin Hsiao, Kruger Marais, Jennifer Giandhari, Sureshnee Pillay, Houriiyah Tegally, Emanuel James San, Tulio de Oliveira, Diana Hardie, Stephen Korsman, Carolyn Williamson
EPI_ISL_696466	Great Brak River Clinic wc GBC & NHLS/UCT	KRISP, KZN Research Innovation and Sequencing Platform	Arash Iranzadeh, Deelan Doolabh, Lynn Tyers, Bruna Galvao, Innocent Mudau, Marvin Hsiao, Kruger Marais, Jennifer Giandhari, Sureshnee Pillay, Houriiyah Tegally, Emanuel James San, Tulio de Oliveira, Diana Hardie, Stephen Korsman, Carolyn Williamson
EPI_ISL_696467	Knysna CDC wc WLC & NHLS/UCT	KRISP, KZN Research Innovation and Sequencing Platform	Arash Iranzadeh, Deelan Doolabh, Lynn Tyers, Bruna Galvao, Innocent Mudau, Marvin Hsiao, Kruger Marais, Jennifer Giandhari, Sureshnee Pillay, Houriiyah Tegally, Emanuel James San, Tulio de Oliveira, Diana Hardie, Stephen Korsman, Carolyn Williamson
EPI_ISL_696468	New Horizon Clinic wc NZC & NHLS/UCT	KRISP, KZN Research Innovation and Sequencing Platform	Arash Iranzadeh, Deelan Doolabh, Lynn Tyers, Bruna Galvao, Innocent Mudau, Marvin Hsiao, Kruger Marais, Jennifer Giandhari, Sureshnee Pillay, Houriiyah Tegally, Emanuel James San, Tulio de Oliveira, Diana Hardie, Stephen Korsman, Carolyn Williamson
EPI_ISL_696469	Knysna Hospital wc KNY & NHLS/UCT	KRISP, KZN Research Innovation and Sequencing Platform	Arash Iranzadeh, Deelan Doolabh, Lynn Tyers, Bruna Galvao, Innocent Mudau, Marvin Hsiao, Kruger Marais, Jennifer Giandhari, Sureshnee Pillay, Houriiyah Tegally, Emanuel James San, Tulio de Oliveira, Diana Hardie, Stephen Korsman, Carolyn Williamson
EPI_ISL_696470	Pacaltsdorp Clinic wc PAC & NHLS/UCT	KRISP, KZN Research Innovation and Sequencing Platform	Arash Iranzadeh, Deelan Doolabh, Lynn Tyers, Bruna Galvao, Innocent Mudau, Marvin Hsiao, Kruger Marais, Jennifer Giandhari, Sureshnee Pillay, Houriiyah Tegally, Emanuel James San, Tulio de Oliveira, Diana Hardie, Stephen Korsman, Carolyn Williamson
EPI_ISL_696471	George Hospital wc GRH & NHLS/UCT	KRISP, KZN Research Innovation and Sequencing Platform	Arash Iranzadeh, Deelan Doolabh, Lynn Tyers, Bruna Galvao, Innocent Mudau, Marvin Hsiao, Kruger Marais, Jennifer Giandhari, Sureshnee Pillay, Houriiyah Tegally, Emanuel James San, Tulio de Oliveira, Diana Hardie, Stephen Korsman, Carolyn Williamson
EPI_ISL_696472	Conville CDC wc CVC & NHLS/UCT	KRISP, KZN Research Innovation and Sequencing Platform	Arash Iranzadeh, Deelan Doolabh, Lynn Tyers, Bruna Galvao, Innocent Mudau, Marvin Hsiao, Kruger Marais, Jennifer Giandhari, Sureshnee Pillay, Houriiyah Tegally, Emanuel James San, Tulio de Oliveira, Diana Hardie, Stephen Korsman, Carolyn Williamson
EPI_ISL_696473	George Hospital wc GRH & NHLS/UCT	KRISP, KZN Research Innovation and Sequencing Platform	Arash Iranzadeh, Deelan Doolabh, Lynn Tyers, Bruna Galvao, Innocent Mudau, Marvin Hsiao, Kruger Marais, Jennifer Giandhari, Sureshnee Pillay, Houriiyah Tegally, Emanuel James San, Tulio de Oliveira, Diana Hardie, Stephen Korsman, Carolyn Williamson
EPI_ISL_696474	Pacaltsdorp Clinic wc PAC & NHLS/UCT	KRISP, KZN Research Innovation and Sequencing Platform	Arash Iranzadeh, Deelan Doolabh, Lynn Tyers, Bruna Galvao, Innocent Mudau, Marvin Hsiao, Kruger Marais, Jennifer Giandhari, Sureshnee Pillay, Houriiyah Tegally, Emanuel James San, Tulio de Oliveira, Diana Hardie, Stephen Korsman, Carolyn Williamson
EPI_ISL_696475	Sedgefield Clinic wc SGE & NHLS/UCT	KRISP, KZN Research Innovation and Sequencing Platform	Arash Iranzadeh, Deelan Doolabh, Lynn Tyers, Bruna Galvao, Innocent Mudau, Marvin Hsiao, Kruger Marais, Jennifer Giandhari, Sureshnee Pillay, Houriiyah Tegally, Emanuel James San, Tulio de Oliveira, Diana Hardie, Stephen Korsman, Carolyn Williamson
EPI_ISL_696476	Plettenberg Bay Clinic wc PLC & NHLS/UCT	KRISP, KZN Research Innovation and Sequencing Platform	Arash Iranzadeh, Deelan Doolabh, Lynn Tyers, Bruna Galvao, Innocent Mudau, Marvin Hsiao, Kruger Marais, Jennifer Giandhari, Sureshnee Pillay, Houriiyah Tegally, Emanuel James San, Tulio de Oliveira, Diana Hardie, Stephen Korsman, Carolyn Williamson
EPI_ISL_696477, EPI_ISL_696478	Knysna Hospital wc KNY & NHLS/UCT	KRISP, KZN Research Innovation and Sequencing Platform	Arash Iranzadeh, Deelan Doolabh, Lynn Tyers, Bruna Galvao, Innocent Mudau, Marvin Hsiao, Kruger Marais, Jennifer Giandhari, Sureshnee Pillay, Houriiyah Tegally, Emanuel James San, Tulio de Oliveira, Diana Hardie, Stephen Korsman, Carolyn Williamson
EPI_ISL_696479	Hornlee Clinic wc HLC & NHLS/UCT	KRISP, KZN Research Innovation and Sequencing Platform	Arash Iranzadeh, Deelan Doolabh, Lynn Tyers, Bruna Galvao, Innocent Mudau, Marvin Hsiao, Kruger Marais, Jennifer Giandhari, Sureshnee Pillay, Houriiyah Tegally, Emanuel James San, Tulio de Oliveira, Diana Hardie, Stephen Korsman, Carolyn Williamson
EPI_ISL_696481	George Hospital wc GRH & NHLS/UCT	KRISP, KZN Research Innovation and Sequencing Platform	Arash Iranzadeh, Deelan Doolabh, Lynn Tyers, Bruna Galvao, Innocent Mudau, Marvin Hsiao, Kruger Marais, Jennifer Giandhari, Sureshnee Pillay, Houriiyah Tegally, Emanuel James San, Tulio de Oliveira, Diana Hardie, Stephen Korsman, Carolyn Williamson
EPI_ISL_696482	Knysna Hospital wc KNY & NHLS/UCT	KRISP, KZN Research Innovation and Sequencing Platform	Arash Iranzadeh, Deelan Doolabh, Lynn Tyers, Bruna Galvao, Innocent Mudau, Marvin Hsiao, Kruger Marais, Jennifer Giandhari, Sureshnee Pillay,

			Houriiyah Tegally, Emanuel James San, Tulio de Oliveira, Diana Hardie, Stephen Korsman, Carolyn Williamson
EPI_ISL_696483	Hornlee Clinic wc HLC & NHLS/UCT	KRISP, KZN Research Innovation and Sequencing Platform	Arash Iranzadeh, Deelan Doolabh, Lynn Tyers, Bruna Galvao, Innocent Mudau, Marvin Hsiao, Kruger Marais, Jennifer Giandhari, Sureshnee Pillay, Houriiyah Tegally, Emanuel James San, Tulio de Oliveira, Diana Hardie, Stephen Korsman, Carolyn Williamson
EPI_ISL_696484	Khayelethu Clinic wc KLC & NHLS/UCT	KRISP, KZN Research Innovation and Sequencing Platform	Arash Iranzadeh, Deelan Doolabh, Lynn Tyers, Bruna Galvao, Innocent Mudau, Marvin Hsiao, Kruger Marais, Jennifer Giandhari, Sureshnee Pillay, Houriiyah Tegally, Emanuel James San, Tulio de Oliveira, Diana Hardie, Stephen Korsman, Carolyn Williamson
EPI_ISL_696485	Touwsranten Clinic wc TST & NHLS/UCT	KRISP, KZN Research Innovation and Sequencing Platform	Arash Iranzadeh, Deelan Doolabh, Lynn Tyers, Bruna Galvao, Innocent Mudau, Marvin Hsiao, Kruger Marais, Jennifer Giandhari, Sureshnee Pillay, Houriiyah Tegally, Emanuel James San, Tulio de Oliveira, Diana Hardie, Stephen Korsman, Carolyn Williamson
EPI_ISL_696486	Hornlee Clinic wc HLC & NHLS/UCT	KRISP, KZN Research Innovation and Sequencing Platform	Arash Iranzadeh, Deelan Doolabh, Lynn Tyers, Bruna Galvao, Innocent Mudau, Marvin Hsiao, Kruger Marais, Jennifer Giandhari, Sureshnee Pillay, Houriiyah Tegally, Emanuel James San, Tulio de Oliveira, Diana Hardie, Stephen Korsman, Carolyn Williamson
EPI_ISL_696487, EPI_ISL_696488	Sedgefield Clinic wc SGE & NHLS/UCT	KRISP, KZN Research Innovation and Sequencing Platform	Arash Iranzadeh, Deelan Doolabh, Lynn Tyers, Bruna Galvao, Innocent Mudau, Marvin Hsiao, Kruger Marais, Jennifer Giandhari, Sureshnee Pillay, Houriiyah Tegally, Emanuel James San, Tulio de Oliveira, Diana Hardie, Stephen Korsman, Carolyn Williamson
EPI_ISL_696489	New Horizon Clinic wc NZC & NHLS/UCT	KRISP, KZN Research Innovation and Sequencing Platform	Arash Iranzadeh, Deelan Doolabh, Lynn Tyers, Bruna Galvao, Innocent Mudau, Marvin Hsiao, Kruger Marais, Jennifer Giandhari, Sureshnee Pillay, Houriiyah Tegally, Emanuel James San, Tulio de Oliveira, Diana Hardie, Stephen Korsman, Carolyn Williamson
EPI_ISL_696490	Touwsranten Clinic wc TST & NHLS/UCT	KRISP, KZN Research Innovation and Sequencing Platform	Arash Iranzadeh, Deelan Doolabh, Lynn Tyers, Bruna Galvao, Innocent Mudau, Marvin Hsiao, Kruger Marais, Jennifer Giandhari, Sureshnee Pillay, Houriiyah Tegally, Emanuel James San, Tulio de Oliveira, Diana Hardie, Stephen Korsman, Carolyn Williamson
EPI_ISL_696491	Knysna Hospital wc KNY & NHLS/UCT	KRISP, KZN Research Innovation and Sequencing Platform	Arash Iranzadeh, Deelan Doolabh, Lynn Tyers, Bruna Galvao, Innocent Mudau, Marvin Hsiao, Kruger Marais, Jennifer Giandhari, Sureshnee Pillay, Houriiyah Tegally, Emanuel James San, Tulio de Oliveira, Diana Hardie, Stephen Korsman, Carolyn Williamson
EPI_ISL_696492	Conville CDC wc CVC & NHLS/UCT	KRISP, KZN Research Innovation and Sequencing Platform	Arash Iranzadeh, Deelan Doolabh, Lynn Tyers, Bruna Galvao, Innocent Mudau, Marvin Hsiao, Kruger Marais, Jennifer Giandhari, Sureshnee Pillay, Houriiyah Tegally, Emanuel James San, Tulio de Oliveira, Diana Hardie, Stephen Korsman, Carolyn Williamson
EPI_ISL_696493, EPI_ISL_696494	Thembalethu CDC wc THC & NHLS/UCT	KRISP, KZN Research Innovation and Sequencing Platform	Arash Iranzadeh, Deelan Doolabh, Lynn Tyers, Bruna Galvao, Innocent Mudau, Marvin Hsiao, Kruger Marais, Jennifer Giandhari, Sureshnee Pillay, Houriiyah Tegally, Emanuel James San, Tulio de Oliveira, Diana Hardie, Stephen Korsman, Carolyn Williamson
EPI_ISL_696495	Knysna CDC wc WLC & NHLS/UCT	KRISP, KZN Research Innovation and Sequencing Platform	Arash Iranzadeh, Deelan Doolabh, Lynn Tyers, Bruna Galvao, Innocent Mudau, Marvin Hsiao, Kruger Marais, Jennifer Giandhari, Sureshnee Pillay, Houriiyah Tegally, Emanuel James San, Tulio de Oliveira, Diana Hardie, Stephen Korsman, Carolyn Williamson
EPI_ISL_696496	Thembalethu CDC wc THC & NHLS/UCT	KRISP, KZN Research Innovation and Sequencing Platform	Arash Iranzadeh, Deelan Doolabh, Lynn Tyers, Bruna Galvao, Innocent Mudau, Marvin Hsiao, Kruger Marais, Jennifer Giandhari, Sureshnee Pillay, Houriiyah Tegally, Emanuel James San, Tulio de Oliveira, Diana Hardie, Stephen Korsman, Carolyn Williamson
EPI_ISL_696497	Great Brak River Clinic wc GBC & NHLS/UCT	KRISP, KZN Research Innovation and Sequencing Platform	Arash Iranzadeh, Deelan Doolabh, Lynn Tyers, Bruna Galvao, Innocent Mudau, Marvin Hsiao, Kruger Marais, Jennifer Giandhari, Sureshnee Pillay, Houriiyah Tegally, Emanuel James San, Tulio de Oliveira, Diana Hardie, Stephen Korsman, Carolyn Williamson
EPI_ISL_696498	Kranshoek Clinic wc KSH & NHLS/UCT	KRISP, KZN Research Innovation and Sequencing Platform	Arash Iranzadeh, Deelan Doolabh, Lynn Tyers, Bruna Galvao, Innocent Mudau, Marvin Hsiao, Kruger Marais, Jennifer Giandhari, Sureshnee Pillay, Houriiyah Tegally, Emanuel James San, Tulio de Oliveira, Diana Hardie, Stephen Korsman, Carolyn Williamson
EPI_ISL_696499, EPI_ISL_696500	Thembalethu CDC wc THC & NHLS/UCT	KRISP, KZN Research Innovation and Sequencing Platform	Arash Iranzadeh, Deelan Doolabh, Lynn Tyers, Bruna Galvao, Innocent Mudau, Marvin Hsiao, Kruger Marais, Jennifer Giandhari, Sureshnee Pillay, Houriiyah Tegally, Emanuel James San, Tulio de Oliveira, Diana Hardie, Stephen Korsman, Carolyn Williamson
EPI_ISL_696501	Great Brak River Clinic wc GBC & NHLS/UCT	KRISP, KZN Research Innovation and Sequencing Platform	Arash Iranzadeh, Deelan Doolabh, Lynn Tyers, Bruna Galvao, Innocent Mudau, Marvin Hsiao, Kruger Marais, Jennifer Giandhari, Sureshnee Pillay, Houriiyah Tegally, Emanuel James San, Tulio de Oliveira, Diana Hardie, Stephen Korsman, Carolyn Williamson
EPI_ISL_696502	Hornlee Clinic wc HLC & NHLS/UCT	KRISP, KZN Research Innovation and Sequencing Platform	Arash Iranzadeh, Deelan Doolabh, Lynn Tyers, Bruna Galvao, Innocent Mudau, Marvin Hsiao, Kruger Marais, Jennifer Giandhari, Sureshnee Pillay, Houriiyah Tegally, Emanuel James San, Tulio de Oliveira, Diana Hardie, Stephen Korsman, Carolyn Williamson
EPI_ISL_696503	George Hospital wc GRH & NHLS/UCT	KRISP, KZN Research Innovation and Sequencing Platform	Arash Iranzadeh, Deelan Doolabh, Lynn Tyers, Bruna Galvao, Innocent Mudau, Marvin Hsiao, Kruger Marais, Jennifer Giandhari, Sureshnee Pillay, Houriiyah Tegally, Emanuel James San, Tulio de Oliveira, Diana Hardie, Stephen Korsman, Carolyn Williamson
EPI_ISL_696504	Pacaltsdorp Clinic wc PAC & NHLS/UCT	KRISP, KZN Research Innovation and Sequencing Platform	Arash Iranzadeh, Deelan Doolabh, Lynn Tyers, Bruna Galvao, Innocent Mudau, Marvin Hsiao, Kruger Marais, Jennifer Giandhari, Sureshnee Pillay, Houriiyah Tegally, Emanuel James San, Tulio de Oliveira, Diana Hardie, Stephen Korsman, Carolyn Williamson
EPI_ISL_696505	Khayelethu Clinic wc KLC & NHLS/UCT	KRISP, KZN Research Innovation and Sequencing Platform	Arash Iranzadeh, Deelan Doolabh, Lynn Tyers, Bruna Galvao, Innocent Mudau, Marvin Hsiao, Kruger Marais, Jennifer Giandhari, Sureshnee Pillay, Houriiyah Tegally, Emanuel James San, Tulio de Oliveira, Diana Hardie, Stephen Korsman, Carolyn Williamson
EPI_ISL_696506	Knysna CDC wc WLC & NHLS/UCT	KRISP, KZN Research Innovation and Sequencing Platform	Arash Iranzadeh, Deelan Doolabh, Lynn Tyers, Bruna Galvao, Innocent Mudau, Marvin Hsiao, Kruger Marais, Jennifer Giandhari, Sureshnee Pillay, Houriiyah Tegally, Emanuel James San, Tulio de Oliveira, Diana Hardie, Stephen Korsman, Carolyn Williamson
EPI_ISL_696507	Great Brak River Clinic wc GBC & NHLS/UCT	KRISP, KZN Research Innovation and Sequencing Platform	Arash Iranzadeh, Deelan Doolabh, Lynn Tyers, Bruna Galvao, Innocent Mudau, Marvin Hsiao, Kruger Marais, Jennifer Giandhari, Sureshnee Pillay, Houriiyah Tegally, Emanuel James San, Tulio de Oliveira, Diana Hardie, Stephen Korsman, Carolyn Williamson
EPI_ISL_696508, EPI_ISL_696509	Conville CDC wc CVC & NHLS/UCT	KRISP, KZN Research Innovation and Sequencing Platform	Arash Iranzadeh, Deelan Doolabh, Lynn Tyers, Bruna Galvao, Innocent Mudau, Marvin Hsiao, Kruger Marais, Jennifer Giandhari, Sureshnee Pillay, Houriiyah Tegally, Emanuel James San, Tulio de Oliveira, Diana Hardie, Stephen Korsman, Carolyn Williamson
EPI_ISL_696510	Sedgefield Clinic wc SGE & NHLS/UCT	KRISP, KZN Research Innovation and Sequencing Platform	Arash Iranzadeh, Deelan Doolabh, Lynn Tyers, Bruna Galvao, Innocent Mudau, Marvin Hsiao, Kruger Marais, Jennifer Giandhari, Sureshnee Pillay, Houriiyah Tegally, Emanuel James San, Tulio de Oliveira, Diana Hardie, Stephen Korsman, Carolyn Williamson
EPI_ISL_696511	Keurhoek Clinic wc KTT & NHLS/UCT	KRISP, KZN Research Innovation and Sequencing Platform	Arash Iranzadeh, Deelan Doolabh, Lynn Tyers, Bruna Galvao, Innocent Mudau, Marvin Hsiao, Kruger Marais, Jennifer Giandhari, Sureshnee Pillay, Houriiyah Tegally, Emanuel James San, Tulio de Oliveira, Diana Hardie, Stephen Korsman, Carolyn Williamson
EPI_ISL_696512	Conville CDC wc CVC & NHLS/UCT	KRISP, KZN Research Innovation and Sequencing Platform	Arash Iranzadeh, Deelan Doolabh, Lynn Tyers, Bruna Galvao, Innocent Mudau, Marvin Hsiao, Kruger Marais, Jennifer Giandhari, Sureshnee Pillay, Houriiyah Tegally, Emanuel James San, Tulio de Oliveira, Diana Hardie, Stephen Korsman, Carolyn Williamson
EPI_ISL_696513	Pacaltsdorp Clinic wc PAC & NHLS/UCT	KRISP, KZN Research Innovation and Sequencing Platform	Arash Iranzadeh, Deelan Doolabh, Lynn Tyers, Bruna Galvao, Innocent Mudau, Marvin Hsiao, Kruger Marais, Jennifer Giandhari, Sureshnee Pillay, Houriiyah Tegally, Emanuel James San, Tulio de Oliveira, Diana Hardie, Stephen Korsman, Carolyn Williamson
EPI_ISL_696514, EPI_ISL_696515, EPI_ISL_696516, EPI_ISL_696517, EPI_ISL_696518	Sedgefield Clinic wc SGE & NHLS/UCT	KRISP, KZN Research Innovation and Sequencing Platform	Arash Iranzadeh, Deelan Doolabh, Lynn Tyers, Bruna Galvao, Innocent Mudau, Marvin Hsiao, Kruger Marais, Jennifer Giandhari, Sureshnee Pillay, Houriiyah Tegally, Emanuel James San, Tulio de Oliveira, Diana Hardie, Stephen Korsman, Carolyn Williamson
EPI_ISL_696519, EPI_ISL_696520	Thembalethu CDC wc THC & NHLS/UCT	KRISP, KZN Research Innovation and Sequencing Platform	Arash Iranzadeh, Deelan Doolabh, Lynn Tyers, Bruna Galvao, Innocent Mudau, Marvin Hsiao, Kruger Marais, Jennifer Giandhari, Sureshnee Pillay, Houriiyah Tegally, Emanuel James San, Tulio de Oliveira, Diana Hardie, Stephen Korsman, Carolyn Williamson
EPI_ISL_696521	Thembalethu CDC wc THC & NHLS/UC	KRISP, KZN Research Innovation and Sequencing Platform	Arash Iranzadeh, Deelan Doolabh, Lynn Tyers, Bruna Galvao, Innocent Mudau, Marvin Hsiao, Kruger Marais, Jennifer Giandhari, Sureshnee Pillay, Houriiyah Tegally, Emanuel James San, Tulio de Oliveira, Diana Hardie, Stephen Korsman, Carolyn Williamson
EPI_ISL_697783, EPI_ISL_697784, EPI_ISL_697785, EPI_ISL_697786	Institute of Microbiology, Universidad San Francisco de Quito	Institute of Microbiology, Universidad San Francisco de Quito	Belén Prado-Vivar, Sully Márquez, Juan José Guadalupe, Monica Becerra-Wong, Bernardo Gutiérrez, Jonathan Araujo, Verónica Barragán, Patricio Rojas-Silva, Gabriel Trueba, Michelle Grunauer, Paúl Cárdenas
EPI_ISL_697787	Institute of Microbiology, Universidad San Francisco de Quito	Institute of Microbiology, Universidad San Francisco de Quito	Andrea Macias, Belén Prado-Vivar, Sully Márquez, Juan José Guadalupe, Monica Becerra-Wong, Bernardo Gutiérrez, Verónica Barragán, Patricio Rojas-Silva, Gabriel Trueba, Michelle Grunauer, Paúl Cárdenas
EPI_ISL_697788, EPI_ISL_697789, EPI_ISL_697790, EPI_ISL_697791, EPI_ISL_697792, EPI_ISL_697793	Institute of Microbiology, Universidad San Francisco de Quito	Institute of Microbiology, Universidad San Francisco de Quito	Belén Prado-Vivar, Sully Márquez, Juan José Guadalupe, Monica Becerra-Wong, Bernardo Gutiérrez, Jonathan Araujo, Verónica Barragán, Patricio Rojas-Silva, Gabriel Trueba, Michelle Grunauer, Paúl Cárdenas
EPI_ISL_697794, EPI_ISL_697795	Institute of Microbiology, Universidad San Francisco de Quito	Institute of Microbiology, Universidad San Francisco de Quito	Andrea Macias, Belén Prado-Vivar, Sully Márquez, Juan José Guadalupe, Monica Becerra-Wong, Bernardo Gutiérrez, Verónica Barragán, Patricio Rojas-Silva, Gabriel Trueba, Michelle Grunauer, Paúl Cárdenas

EPI_ISL_697797	Centro de Investigaciones, Universidad de Especialidades Espíritu Santo	Institute of Microbiology, Universidad San Francisco de Quito	Derly Andrade, Juan Carlos Fernandez, Belén Prado-Vivar, Sully Márquez, Juan José Guadalupe, Monica Becerra-Wong, Bernardo Gutiérrez, Gabriel Morey, Ruben Armas, Jose Pedro Barberan, Fernando Espinoza, Edith Lopez, Verónica Barragán, Patricio Rojas-Silva, Gabriel Trueba, Michelle Grunauer, Paúl Cárdenas
EPI_ISL_697798, EPI_ISL_697799	Institute of Microbiology, Universidad San Francisco de Quito	Institute of Microbiology, Universidad San Francisco de Quito	Belén Prado-Vivar, Sully Márquez, Juan José Guadalupe, Monica Becerra-Wong, Diana Zambrano, Fredy Loor, Juan Zuñiga, Edison Chavez, Bernardo Gutiérrez, Verónica Barragán, Patricio Rojas-Silva, Gabriel Trueba, Michelle Grunauer, Paúl Cárdenas
EPI_ISL_697800	Institute of Microbiology, Universidad San Francisco de Quito	Institute of Microbiology, Universidad San Francisco de Quito	Belén Prado-Vivar, Sully Márquez, Juan José Guadalupe, Monica Becerra-Wong, Maureen Mosquera, Bernardo Gutiérrez, Verónica Barragán, Patricio Rojas-Silva, Gabriel Trueba, Michelle Grunauer, Paúl Cárdenas
EPI_ISL_698079, EPI_ISL_698080, EPI_ISL_698081, EPI_ISL_698082, EPI_ISL_698083, EPI_ISL_698084, EPI_ISL_698085, EPI_ISL_698086, EPI_ISL_698088, EPI_ISL_698089, EPI_ISL_698090, EPI_ISL_698091, EPI_ISL_698092, EPI_ISL_698093, EPI_ISL_698094, EPI_ISL_698095, EPI_ISL_698096, EPI_ISL_698097, EPI_ISL_698098, EPI_ISL_698099, EPI_ISL_698100, EPI_ISL_698101, EPI_ISL_698102, EPI_ISL_698103, EPI_ISL_698104			
see above	Unity Health Toronto	Ontario Institute for Cancer Research	Ramzi Fattouh, Larissa M. Matukas, Yan Chen,Mark Downing, Trina Otterman, Karel Boissinot, Le Luu, Samira Mubareka, TIBDN, Ilinca Lungu, Bernard Lam, Jeremy Johns, Paul Krzyzanowski, Richard de Borja, Felicia Vincelli, Philip Zuzarte, Jared T. Simpson
EPI_ISL_698105, EPI_ISL_698106, EPI_ISL_698107, EPI_ISL_698108, EPI_ISL_698109, EPI_ISL_698110, EPI_ISL_698111, EPI_ISL_698112, EPI_ISL_698113, EPI_ISL_698114, EPI_ISL_698115, EPI_ISL_698116, EPI_ISL_698117, EPI_ISL_698118, EPI_ISL_698119, EPI_ISL_698120, EPI_ISL_698121, EPI_ISL_698122, EPI_ISL_698123, EPI_ISL_698124, EPI_ISL_698125, EPI_ISL_698126, EPI_ISL_698127, EPI_ISL_698128, EPI_ISL_698129, EPI_ISL_698130, EPI_ISL_698131, EPI_ISL_698132, EPI_ISL_698133, EPI_ISL_698134, EPI_ISL_698135, EPI_ISL_698136, EPI_ISL_698137, EPI_ISL_698138, EPI_ISL_698139, EPI_ISL_698140, EPI_ISL_698141, EPI_ISL_698142, EPI_ISL_698143, EPI_ISL_698144, EPI_ISL_698145, EPI_ISL_698146, EPI_ISL_698147, EPI_ISL_698148, EPI_ISL_698149, EPI_ISL_698150, EPI_ISL_698151, EPI_ISL_698152, EPI_ISL_698153, EPI_ISL_698154, EPI_ISL_698191, EPI_ISL_698201, EPI_ISL_698212, EPI_ISL_699027, EPI_ISL_699028, EPI_ISL_699029, EPI_ISL_699030, EPI_ISL_699031, EPI_ISL_699032, EPI_ISL_699033, EPI_ISL_699034, EPI_ISL_699035, EPI_ISL_699036, EPI_ISL_699037, EPI_ISL_699038, EPI_ISL_699039, EPI_ISL_699040, EPI_ISL_699041, EPI_ISL_699042, EPI_ISL_699043, EPI_ISL_699044, EPI_ISL_699045, EPI_ISL_699046, EPI_ISL_699047, EPI_ISL_699048, EPI_ISL_699049, EPI_ISL_699050, EPI_ISL_699051, EPI_ISL_699052, EPI_ISL_699053, EPI_ISL_699054, EPI_ISL_699055, EPI_ISL_699056, EPI_ISL_699057, EPI_ISL_699058, EPI_ISL_699059, EPI_ISL_699060, EPI_ISL_699061, EPI_ISL_699062, EPI_ISL_699063, EPI_ISL_699064, EPI_ISL_699065, EPI_ISL_699066, EPI_ISL_699067, EPI_ISL_699068, EPI_ISL_699069, EPI_ISL_699070, EPI_ISL_699071, EPI_ISL_699072, EPI_ISL_699073, EPI_ISL_699074, EPI_ISL_699075, EPI_ISL_699076, EPI_ISL_699077, EPI_ISL_699078, EPI_ISL_699079, EPI_ISL_699080, EPI_ISL_699081, EPI_ISL_699082, EPI_ISL_699083, EPI_ISL_699084, EPI_ISL_699085, EPI_ISL_699086, EPI_ISL_699087, EPI_ISL_699088, EPI_ISL_699089, EPI_ISL_699090, EPI_ISL_699091, EPI_ISL_699092, EPI_ISL_699093, EPI_ISL_699094, EPI_ISL_699095, EPI_ISL_699096, EPI_ISL_699097, EPI_ISL_699098, EPI_ISL_699099, EPI_ISL_699100, EPI_ISL_699101, EPI_ISL_699102, EPI_ISL_699103, EPI_ISL_699104, EPI_ISL_699105, EPI_ISL_699106, EPI_ISL_699107, EPI_ISL_699108, EPI_ISL_699109, EPI_ISL_699110, EPI_ISL_699111, EPI_ISL_699112, EPI_ISL_699113, EPI_ISL_699114, EPI_ISL_699115, EPI_ISL_699116, EPI_ISL_699117, EPI_ISL_699118, EPI_ISL_699119, EPI_ISL_699120, EPI_ISL_699121, EPI_ISL_699122, EPI_ISL_699123, EPI_ISL_699124, EPI_ISL_699125, EPI_ISL_699126, EPI_ISL_699127, EPI_ISL_699128, EPI_ISL_699129, EPI_ISL_699130, EPI_ISL_699131, EPI_ISL_699132, EPI_ISL_699133, EPI_ISL_699134, EPI_ISL_699135, EPI_ISL_699136, EPI_ISL_699137, EPI_ISL_699138, EPI_ISL_699139, EPI_ISL_699140, EPI_ISL_699141, EPI_ISL_699142, EPI_ISL_699143, EPI_ISL_699144, EPI_ISL_699145, EPI_ISL_699146, EPI_ISL_699147, EPI_ISL_699148, EPI_ISL_699149, EPI_ISL_699150, EPI_ISL_699151, EPI_ISL_699152, EPI_ISL_699153, EPI_ISL_699154, EPI_ISL_699155, EPI_ISL_699156, EPI_ISL_699157, EPI_ISL_699158, EPI_ISL_699159, EPI_ISL_699160, EPI_ISL_699161			
see above	Group 42 (G42) Healthcare, Abu Dhabi, United Arab Emirates; Department of Health, The United Arab Emirates	G42 Healthcare	Rong Liu, Pei Wu, Sally Mahmoud, Ke Liang, Pauline Ogrodzki, Pengjuan Liu, Stephen S. Francis, Tao Ma, Hanif Khalak, Fang Chen, Denghui Liu, Junhua Li, Weibin Liu, Wenjun He, Xinyu Huang, Zhaorong Yuan, Long Lin, Nan Qiao, Xin Meng, Budoor Alqarni, Javier Quilez, Vinay Kusuma, Xin Jin, Xavier Anton, Ashish Koshy, Huanming Yang, Xun Xu, Jian Wang, Peng Xiao, Nawal Ahmed Mohamed Al Kaabi, Mohammed Saifuddin Fasihuddin, Siyang Liu, Walid Abbas Zaher
EPI_ISL_699642	South Eastern Area Laboratory Services (SEALS)	NSW Health Pathology - Institute of Clinical Pathology and Medical Research; Westmead Hospital; University of Sydney	CIDM-PH et al.
EPI_ISL_699643	Medlab Pathology	NSW Health Pathology - Institute of Clinical Pathology and Medical Research; Westmead Hospital; University of Sydney	CIDM-PH et al.
EPI_ISL_699650	Douglas Hanly Moir	NSW Health Pathology - Institute of Clinical Pathology and Medical Research; Westmead Hospital; University of Sydney	CIDM-PH et al.
EPI_ISL_699653, EPI_ISL_699654	South Eastern Area Laboratory Services (SEALS)	NSW Health Pathology - Institute of Clinical Pathology and Medical Research; Westmead Hospital; University of Sydney	CIDM-PH et al.
EPI_ISL_699655	1-Laboratory of Microbiology, National Reference Lab, Charles Nicolle Hospital; 2-University of Tunis ElManar, Faculty of Medicine of Tunis, LR99ES09, Tunis, Tunisia	1-Clinical and Experimental Pharmacology Lab, LR16SP02, National Center of Pharmacovigilance, University of Tunis El Manar, Tunis, Tunisia. 2-Neurodegenerative diseases and psychiatric troubles, LR18SP03, Razi Hospital, University of Tunis El Manar, Tunis, Tunisia. 3- Ministry of Health, National Observatory of New and Emerging Diseases, 1006, Tunis, Tunisia	Ilhem Boutiba-Ben Boubaker, Sameh Trabelsi, Nissaf Ben Alaya, Maher Kharrat, Alia Ben Kahla, Jalila Ben Khelil, Salma Abid, Sana Ferjani, Asma Ferjani, Mouna Ben Sassi, Mouna Safer, Guedi Berrabeh,Salwa Mrabet, Hanen ElJebari, Gaies Emna, Riadh Daghfous, Riadh Gouider.
EPI_ISL_699807, EPI_ISL_699808, EPI_ISL_699809, EPI_ISL_699811, EPI_ISL_699812, EPI_ISL_699813, EPI_ISL_699815, EPI_ISL_699816, EPI_ISL_699817, EPI_ISL_699818, EPI_ISL_699819, EPI_ISL_699820, EPI_ISL_699821, EPI_ISL_699822, EPI_ISL_699823, EPI_ISL_699824, EPI_ISL_699826, EPI_ISL_699828, EPI_ISL_699829, EPI_ISL_699830, EPI_ISL_699831, EPI_ISL_699832, EPI_ISL_699833, EPI_ISL_699834, EPI_ISL_699835, EPI_ISL_699836, EPI_ISL_699837, EPI_ISL_699838, EPI_ISL_699839, EPI_ISL_699840, EPI_ISL_699841, EPI_ISL_699843, EPI_ISL_699844, EPI_ISL_699845, EPI_ISL_699846, EPI_ISL_699847, EPI_ISL_699848, EPI_ISL_699849, EPI_ISL_699850, EPI_ISL_699851, EPI_ISL_699852, EPI_ISL_699853, EPI_ISL_699854, EPI_ISL_699855, EPI_ISL_699856, EPI_ISL_699857, EPI_ISL_699858, EPI_ISL_699859, EPI_ISL_699860, EPI_ISL_699861, EPI_ISL_699862, EPI_ISL_699863, EPI_ISL_699864, EPI_ISL_699865, EPI_ISL_699866, EPI_ISL_699867, EPI_ISL_699868, EPI_ISL_699869, EPI_ISL_699870, EPI_ISL_699871, EPI_ISL_699872, EPI_ISL_699873, EPI_ISL_699874, EPI_ISL_699875, EPI_ISL_699876, EPI_ISL_699877, EPI_ISL_699878, EPI_ISL_699879, EPI_ISL_699880, EPI_ISL_699881, EPI_ISL_699882, EPI_ISL_699883, EPI_ISL_699884, EPI_ISL_699885, EPI_ISL_699886, EPI_ISL_699887, EPI_ISL_699888, EPI_ISL_699889, EPI_ISL_699890, EPI_ISL_699891, EPI_ISL_699892, EPI_ISL_699893, EPI_ISL_699894, EPI_ISL_699895, EPI_ISL_699896, EPI_ISL_699897, EPI_ISL_699898, EPI_ISL_699899, EPI_ISL_699900, EPI_ISL_699901, EPI_ISL_699902, EPI_ISL_699903, EPI_ISL_699904, EPI_ISL_699905, EPI_ISL_699906, EPI_ISL_699907, EPI_ISL_699908, EPI_ISL_699909, EPI_ISL_699910, EPI_ISL_699911, EPI_ISL_699912, EPI_ISL_699913, EPI_ISL_699914, EPI_ISL_699915, EPI_ISL_699916, EPI_ISL_699917, EPI_ISL_699918, EPI_ISL_699919, EPI_ISL_699920, EPI_ISL_699921, EPI_ISL_699922, EPI_ISL_699923, EPI_ISL_699924, EPI_ISL_699925, EPI_ISL_699926, EPI_ISL_699927, EPI_ISL_699928, EPI_ISL_699929, EPI_ISL_699930, EPI_ISL_699931, EPI_ISL_699932, EPI_ISL_699933, EPI_ISL_699934, EPI_ISL_699935, EPI_ISL_699936, EPI_ISL_699937, EPI_ISL_699938, EPI_ISL_699939, EPI_ISL_699940, EPI_ISL_699941, EPI_ISL_699942, EPI_ISL_699943, EPI_ISL_699944, EPI_ISL_699945, EPI_ISL_699946, EPI_ISL_699947, EPI_ISL_699948, EPI_ISL_699949, EPI_ISL_699950, EPI_ISL_699951, EPI_ISL_699952, EPI_ISL_699953, EPI_ISL_699954, EPI_ISL_699955, EPI_ISL_699956, EPI_ISL_699957, EPI_ISL_699958, EPI_ISL_699959, EPI_ISL_699960, EPI_ISL_699961, EPI_ISL_699962, EPI_ISL_699963, EPI_ISL_699964, EPI_ISL_699965, EPI_ISL_699966, EPI_ISL_699967, EPI_ISL_699968, EPI_ISL_699969, EPI_ISL_699970, EPI_ISL_699971, EPI_ISL_699972, EPI_ISL_699973, EPI_ISL_699974, EPI_ISL_699975, EPI_ISL_699976, EPI_ISL_699977, EPI_ISL_699978, EPI_ISL_699979, EPI_ISL_699980, EPI_ISL_699981, EPI_ISL_699982, EPI_ISL_699983, EPI_ISL_699984, EPI_ISL_699985, EPI_ISL_699986, EPI_ISL_699987, EPI_ISL_699988, EPI_ISL_699989, EPI_ISL_699990, EPI_ISL_699991, EPI_ISL_699992, EPI_ISL_699993, EPI_ISL_699994, EPI_ISL_699995, EPI_ISL_699996, EPI_ISL_699997, EPI_ISL_699998, EPI_ISL_699999, EPI_ISL_700000, EPI_ISL_700001, EPI_ISL_700002, EPI_ISL_700003, EPI_ISL_700004, EPI_ISL_700005, EPI_ISL_700006, EPI_ISL_700007, EPI_ISL_700008, EPI_ISL_700009, EPI_ISL_700010, EPI_ISL_700011, EPI_ISL_700012, EPI_ISL_700013, EPI_ISL_700014, EPI_ISL_700015, EPI_ISL_700016, EPI_ISL_700017, EPI_ISL_700018, EPI_ISL_700019, EPI_ISL_700020, EPI_ISL_700021, EPI_ISL_700022, EPI_ISL_700023, EPI_ISL_700024, EPI_ISL_700025, EPI_ISL_700026, EPI_ISL_700027, EPI_ISL_700028, EPI_ISL_700029, EPI_ISL_700030, EPI_ISL_700031, EPI_ISL_700032, EPI_ISL_700033, EPI_ISL_700034, EPI_ISL_700035, EPI_ISL_700036, EPI_ISL_700037, EPI_ISL_700038, EPI_ISL_700039, EPI_ISL_700040, EPI_ISL_700041			
see above	Hematopathology Laboratory, ACTREC, TMC	Hematopathology Laboratory, ACTREC, TMC	Hematopathology Laboratory, ACTREC
