# Supplementary material for: Deepening of In Silico Evaluation of SARS-CoV-2 Detection RT-qPCR Assays in the Context of New Variants
Source: Genes (Basel). 2021 Apr 13;12(4):565. doi: 10.3390/genes12040565 (PMC8069896; doi:10.3390/genes12040565)
Supplement: Supplementary file 1 [file genes-12-00565-s001.zip › Supplementary file S9.zip!/Acknowledgement tables/gisaid_hcov-19_acknowledgement_table_16.pdf]

We gratefully acknowledge the following Authors from the Originating laboratories responsible for obtaining the specimens, as well as the Submitting laboratories where the genome data were generated and shared via GISAID, on which this research is based.

All Submitters of data may be contacted directly via www.gisaid.org

Authors are sorted alphabetically.

Accession ID	Originating Laboratory	Submitting Laboratory	Authors

EPI_ISL_712978, EPI_ISL_712979, EPI_ISL_712980, EPI_ISL_712981, EPI_ISL_712982, EPI_ISL_712983, EPI_ISL_712984, EPI_ISL_712985, EPI_ISL_712986, EPI_ISL_712987, EPI_ISL_712988, EPI_ISL_712989, EPI_ISL_712990, EPI_ISL_712991, EPI_ISL_712992, EPI_ISL_712993, EPI_ISL_712994, EPI_ISL_712995,
EPI_ISL_712996, EPI_ISL_712997, EPI_ISL_712998, EPI_ISL_712999, EPI_ISL_713000, EPI_ISL_713001, EPI_ISL_713002, EPI_ISL_713003, EPI_ISL_713004, EPI_ISL_713005, EPI_ISL_713006, EPI_ISL_713007, EPI_ISL_713008, EPI_ISL_713009, EPI_ISL_713010, EPI_ISL_713011, EPI_ISL_713012, EPI_ISL_713013,
EPI_ISL_713014, EPI_ISL_713015, EPI_ISL_713016, EPI_ISL_713017, EPI_ISL_713018, EPI_ISL_713019, EPI_ISL_713020, EPI_ISL_713021, EPI_ISL_713022, EPI_ISL_713023, EPI_ISL_713024, EPI_ISL_713025, EPI_ISL_713026, EPI_ISL_713027, EPI_ISL_713028, EPI_ISL_713029, EPI_ISL_713030, EPI_ISL_713031,
EPI_ISL_713032, EPI_ISL_713033, EPI_ISL_713034, EPI_ISL_713035, EPI_ISL_713036, EPI_ISL_713037, EPI_ISL_713038, EPI_ISL_713039, EPI_ISL_713040, EPI_ISL_713041, EPI_ISL_713042, EPI_ISL_713043, EPI_ISL_713044, EPI_ISL_713045, EPI_ISL_713046, EPI_ISL_713047, EPI_ISL_713048, EPI_ISL_713049,
EPI_ISL_713050, EPI_ISL_713051, EPI_ISL_713052, EPI_ISL_713053, EPI_ISL_713054, EPI_ISL_713055, EPI_ISL_713056, EPI_ISL_713057, EPI_ISL_713058, EPI_ISL_713059, EPI_ISL_713060, EPI_ISL_713061, EPI_ISL_713062, EPI_ISL_713063, EPI_ISL_713064, EPI_ISL_713065, EPI_ISL_713066, EPI_ISL_713067,
EPI_ISL_713068, EPI_ISL_713069, EPI_ISL_713070, EPI_ISL_713071, EPI_ISL_713072, EPI_ISL_713073, EPI_ISL_713074, EPI_ISL_713075, EPI_ISL_713076, EPI_ISL_713077, EPI_ISL_713078, EPI_ISL_713079, EPI_ISL_713080, EPI_ISL_713081, EPI_ISL_713082, EPI_ISL_713083, EPI_ISL_713084, EPI_ISL_713085,
EPI_ISL_713086, EPI_ISL_713087, EPI_ISL_713088, EPI_ISL_713089, EPI_ISL_713090, EPI_ISL_713091, EPI_ISL_713092, EPI_ISL_713093, EPI_ISL_713094, EPI_ISL_713095, EPI_ISL_713096, EPI_ISL_713097, EPI_ISL_713098, EPI_ISL_713099, EPI_ISL_713100, EPI_ISL_713101, EPI_ISL_713102, EPI_ISL_713103,
EPI_ISL_713104, EPI_ISL_713105, EPI_ISL_713106, EPI_ISL_713107, EPI_ISL_713108, EPI_ISL_713109, EPI_ISL_713110, EPI_ISL_713111, EPI_ISL_713112, EPI_ISL_713113, EPI_ISL_713114, EPI_ISL_713115, EPI_ISL_713116, EPI_ISL_713117, EPI_ISL_713118, EPI_ISL_713119, EPI_ISL_713120, EPI_ISL_713121,
EPI_ISL_713122, EPI_ISL_713123, EPI_ISL_713124, EPI_ISL_713125, EPI_ISL_713126, EPI_ISL_713128, EPI_ISL_713129, EPI_ISL_713130, EPI_ISL_713131, EPI_ISL_713132, EPI_ISL_713133, EPI_ISL_713134, EPI_ISL_713135, EPI_ISL_713136, EPI_ISL_713137, EPI_ISL_713138, EPI_ISL_713139, EPI_ISL_713140,
EPI_ISL_713141, EPI_ISL_713142, EPI_ISL_713143, EPI_ISL_713144, EPI_ISL_713145, EPI_ISL_713146, EPI_ISL_713147, EPI_ISL_713148, EPI_ISL_713149, EPI_ISL_713150, EPI_ISL_713151, EPI_ISL_713152, EPI_ISL_713153, EPI_ISL_713154, EPI_ISL_713155, EPI_ISL_713156, EPI_ISL_713157, EPI_ISL_713158,
EPI_ISL_713159, EPI_ISL_713160, EPI_ISL_713161, EPI_ISL_713162, EPI_ISL_713163, EPI_ISL_713164, EPI_ISL_713165, EPI_ISL_713166, EPI_ISL_713167, EPI_ISL_713168, EPI_ISL_713169, EPI_ISL_713170, EPI_ISL_713171, EPI_ISL_713172, EPI_ISL_713173, EPI_ISL_713174, EPI_ISL_713175, EPI_ISL_713176,
EPI_ISL_713177, EPI_ISL_713178, EPI_ISL_713179, EPI_ISL_713180, EPI_ISL_713181, EPI_ISL_713182, EPI_ISL_713183, EPI_ISL_713184, EPI_ISL_713185, EPI_ISL_713186, EPI_ISL_713187, EPI_ISL_713188, EPI_ISL_713189, EPI_ISL_713190, EPI_ISL_713191, EPI_ISL_713192, EPI_ISL_713193, EPI_ISL_713194,
EPI_ISL_713195, EPI_ISL_713196, EPI_ISL_713197, EPI_ISL_713198, EPI_ISL_713199, EPI_ISL_713200, EPI_ISL_713201, EPI_ISL_713202, EPI_ISL_713203, EPI_ISL_713204, EPI_ISL_713205, EPI_ISL_713206, EPI_ISL_713207, EPI_ISL_713208, EPI_ISL_713209, EPI_ISL_713210, EPI_ISL_713211, EPI_ISL_713212,
EPI_ISL_713213, EPI_ISL_713214, EPI_ISL_713215, EPI_ISL_713216, EPI_ISL_713217, EPI_ISL_713218, EPI_ISL_713219, EPI_ISL_713220, EPI_ISL_713221, EPI_ISL_713222, EPI_ISL_713223, EPI_ISL_713224, EPI_ISL_713225, EPI_ISL_713226, EPI_ISL_713227, EPI_ISL_713228, EPI_ISL_713229, EPI_ISL_713230,
EPI_ISL_713231, EPI_ISL_713232, EPI_ISL_713233, EPI_ISL_713234, EPI_ISL_713235, EPI_ISL_713236, EPI_ISL_713237, EPI_ISL_713238, EPI_ISL_713239, EPI_ISL_713240, EPI_ISL_713241, EPI_ISL_713242, EPI_ISL_713243, EPI_ISL_713244, EPI_ISL_713245, EPI_ISL_713246, EPI_ISL_713247, EPI_ISL_713248,
EPI_ISL_713249, EPI_ISL_713250, EPI_ISL_713251, EPI_ISL_713252, EPI_ISL_713253, EPI_ISL_713254, EPI_ISL_713255, EPI_ISL_713256, EPI_ISL_713257, EPI_ISL_713258, EPI_ISL_713259, EPI_ISL_713260, EPI_ISL_713261, EPI_ISL_713262, EPI_ISL_713263, EPI_ISL_713264, EPI_ISL_713265, EPI_ISL_713266,
EPI_ISL_713267, EPI_ISL_713268, EPI_ISL_713269, EPI_ISL_713270, EPI_ISL_713271, EPI_ISL_713272, EPI_ISL_713273, EPI_ISL_713274, EPI_ISL_713275, EPI_ISL_713276, EPI_ISL_713277, EPI_ISL_713278, EPI_ISL_713279, EPI_ISL_713280, EPI_ISL_713281, EPI_ISL_713282, EPI_ISL_713283, EPI_ISL_713284,
EPI_ISL_713285, EPI_ISL_713286, EPI_ISL_713287, EPI_ISL_713288, EPI_ISL_713289, EPI_ISL_713290, EPI_ISL_713291, EPI_ISL_713292, EPI_ISL_713293, EPI_ISL_713294, EPI_ISL_713315, EPI_ISL_713316, EPI_ISL_713317, EPI_ISL_713318, EPI_ISL_713319, EPI_ISL_713320, EPI_ISL_713321, EPI_ISL_713322,
EPI_ISL_713323, EPI_ISL_713324, EPI_ISL_713325, EPI_ISL_713326, EPI_ISL_713327, EPI_ISL_713328, EPI_ISL_713329, EPI_ISL_713330, EPI_ISL_713331, EPI_ISL_713332, EPI_ISL_713333, EPI_ISL_713334, EPI_ISL_713335, EPI_ISL_713336, EPI_ISL_713337, EPI_ISL_713338, EPI_ISL_713339, EPI_ISL_713340,
EPI_ISL_713341, EPI_ISL_713342, EPI_ISL_713343, EPI_ISL_713344, EPI_ISL_713345, EPI_ISL_713346, EPI_ISL_713347, EPI_ISL_713348, EPI_ISL_713349, EPI_ISL_713350, EPI_ISL_713351, EPI_ISL_713352, EPI_ISL_713353, EPI_ISL_713354, EPI_ISL_713355, EPI_ISL_713356, EPI_ISL_713357, EPI_ISL_713358,
EPI_ISL_713359, EPI_ISL_713360, EPI_ISL_713361, EPI_ISL_713362, EPI_ISL_713363, EPI_ISL_713364, EPI_ISL_713365, EPI_ISL_713366, EPI_ISL_713367, EPI_ISL_713368, EPI_ISL_713369, EPI_ISL_713370, EPI_ISL_713371, EPI_ISL_713372, EPI_ISL_713373, EPI_ISL_713374, EPI_ISL_713375, EPI_ISL_713376,
EPI_ISL_713377, EPI_ISL_713378, EPI_ISL_713379, EPI_ISL_713380, EPI_ISL_713381, EPI_ISL_713382, EPI_ISL_713383, EPI_ISL_713384, EPI_ISL_713385, EPI_ISL_713386, EPI_ISL_713387, EPI_ISL_713388, EPI_ISL_713389, EPI_ISL_713390, EPI_ISL_713391, EPI_ISL_713392, EPI_ISL_713393, EPI_ISL_713394,
EPI_ISL_713395, EPI_ISL_713396, EPI_ISL_713397, EPI_ISL_713398, EPI_ISL_713399, EPI_ISL_713400, EPI_ISL_713401, EPI_ISL_713402, EPI_ISL_713403, EPI_ISL_713404, EPI_ISL_713405, EPI_ISL_713406, EPI_ISL_713407, EPI_ISL_713408, EPI_ISL_713409, EPI_ISL_713410, EPI_ISL_713411, EPI_ISL_713412,
EPI_ISL_713413, EPI_ISL_713414, EPI_ISL_713415, EPI_ISL_713416, EPI_ISL_713417, EPI_ISL_713418, EPI_ISL_713419, EPI_ISL_713420, EPI_ISL_713421, EPI_ISL_713422, EPI_ISL_713423, EPI_ISL_713424, EPI_ISL_713425, EPI_ISL_713426, EPI_ISL_713427, EPI_ISL_713428, EPI_ISL_713429, EPI_ISL_713430,
EPI_ISL_713431, EPI_ISL_713432, EPI_ISL_713433, EPI_ISL_713434, EPI_ISL_713435, EPI_ISL_713436, EPI_ISL_713437, EPI_ISL_713438, EPI_ISL_713439, EPI_ISL_713440, EPI_ISL_713441, EPI_ISL_713442, EPI_ISL_713443, EPI_ISL_713444, EPI_ISL_713445, EPI_ISL_713446, EPI_ISL_713447, EPI_ISL_713448,
EPI_ISL_713449, EPI_ISL_713450, EPI_ISL_713451, EPI_ISL_713452, EPI_ISL_713453, EPI_ISL_713454, EPI_ISL_713455, EPI_ISL_713456, EPI_ISL_713457, EPI_ISL_713458, EPI_ISL_713459, EPI_ISL_713460, EPI_ISL_713461, EPI_ISL_713464, EPI_ISL_713465, EPI_ISL_713466, EPI_ISL_713467, EPI_ISL_713468,
EPI_ISL_713469, EPI_ISL_713470, EPI_ISL_713471, EPI_ISL_713472, EPI_ISL_713473, EPI_ISL_713474, EPI_ISL_713475, EPI_ISL_713476, EPI_ISL_713477, EPI_ISL_713478, EPI_ISL_713479, EPI_ISL_713480, EPI_ISL_713481, EPI_ISL_713482, EPI_ISL_713483, EPI_ISL_713484, EPI_ISL_713485, EPI_ISL_713486,
EPI_ISL_713487, EPI_ISL_713488, EPI_ISL_713489, EPI_ISL_713490, EPI_ISL_713491, EPI_ISL_713492, EPI_ISL_713493, EPI_ISL_713494, EPI_ISL_713495, EPI_ISL_713496, EPI_ISL_713497, EPI_ISL_713498, EPI_ISL_713499, EPI_ISL_713500, EPI_ISL_713501, EPI_ISL_713502, EPI_ISL_713503, EPI_ISL_713504,
EPI_ISL_713505, EPI_ISL_713506, EPI_ISL_713507, EPI_ISL_713508, EPI_ISL_713509, EPI_ISL_713510, EPI_ISL_713511, EPI_ISL_713512, EPI_ISL_713513, EPI_ISL_713514, EPI_ISL_713515, EPI_ISL_713516, EPI_ISL_713517, EPI_ISL_713518, EPI_ISL_713519, EPI_ISL_713520, EPI_ISL_713521, EPI_ISL_713522,
EPI_ISL_713523, EPI_ISL_713524, EPI_ISL_713525, EPI_ISL_713526, EPI_ISL_713527, EPI_ISL_713528, EPI_ISL_713529, EPI_ISL_713530, EPI_ISL_713531, EPI_ISL_713532, EPI_ISL_713533, EPI_ISL_713534, EPI_ISL_713535, EPI_ISL_713536, EPI_ISL_713537, EPI_ISL_713538, EPI_ISL_713539, EPI_ISL_713540,
EPI_ISL_713541, EPI_ISL_713542, EPI_ISL_713543, EPI_ISL_713544, EPI_ISL_713545, EPI_ISL_713546, EPI_ISL_713547, EPI_ISL_713548, EPI_ISL_713549, EPI_ISL_713550, EPI_ISL_713551, EPI_ISL_713552, EPI_ISL_713553, EPI_ISL_713554, EPI_ISL_713555, EPI_ISL_713556, EPI_ISL_713557, EPI_ISL_713558,
EPI_ISL_713559, EPI_ISL_713560, EPI_ISL_713561, EPI_ISL_713562, EPI_ISL_713563, EPI_ISL_713564, EPI_ISL_713565, EPI_ISL_713566, EPI_ISL_713567, EPI_ISL_713568, EPI_ISL_713569, EPI_ISL_713570, EPI_ISL_713571, EPI_ISL_713572, EPI_ISL_713573, EPI_ISL_713574, EPI_ISL_713575, EPI_ISL_713576,
EPI_ISL_713577, EPI_ISL_713578, EPI_ISL_713579, EPI_ISL_713580, EPI_ISL_713581, EPI_ISL_713582, EPI_ISL_713583, EPI_ISL_713584, EPI_ISL_713585, EPI_ISL_713586, EPI_ISL_713587, EPI_ISL_713588, EPI_ISL_713589, EPI_ISL_713590, EPI_ISL_713591, EPI_ISL_713592, EPI_ISL_713593, EPI_ISL_713594,
EPI_ISL_713595, EPI_ISL_713596, EPI_ISL_713597, EPI_ISL_713598, EPI_ISL_713599, EPI_ISL_713600, EPI_ISL_713601, EPI_ISL_713602, EPI_ISL_713603, EPI_ISL_713604, EPI_ISL_713605, EPI_ISL_713606, EPI_ISL_713607, EPI_ISL_713608, EPI_ISL_713609, EPI_ISL_713610, EPI_ISL_713611, EPI_ISL_713612,
EPI_ISL_713613, EPI_ISL_713614, EPI_ISL_713615, EPI_ISL_713616, EPI_ISL_713617, EPI_ISL_713618, EPI_ISL_713619, EPI_ISL_713620, EPI_ISL_713621, EPI_ISL_713622, EPI_ISL_713623, EPI_ISL_713624, EPI_ISL_713625, EPI_ISL_713626, EPI_ISL_713627, EPI_ISL_713628, EPI_ISL_713629, EPI_ISL_713630,
EPI_ISL_713631, EPI_ISL_713632, EPI_ISL_713633, EPI_ISL_713634, EPI_ISL_713635, EPI_ISL_713636, EPI_ISL_713637, EPI_ISL_713638, EPI_ISL_713639, EPI_ISL_713640, EPI_ISL_713641, EPI_ISL_713642, EPI_ISL_713643, EPI_ISL_713644, EPI_ISL_713645, EPI_ISL_713646, EPI_ISL_713647, EPI_ISL_713648,
EPI_ISL_713649, EPI_ISL_713650, EPI_ISL_713651, EPI_ISL_713652, EPI_ISL_713653, EPI_ISL_713654, EPI_ISL_713655, EPI_ISL_713656, EPI_ISL_713657, EPI_ISL_713658, EPI_ISL_713659, EPI_ISL_713660, EPI_ISL_713661, EPI_ISL_713662, EPI_ISL_713663, EPI_ISL_713664, EPI_ISL_713665, EPI_ISL_713666,
EPI_ISL_713667, EPI_ISL_713668, EPI_ISL_713669, EPI_ISL_713670, EPI_ISL_713671, EPI_ISL_713672, EPI_ISL_713673, EPI_ISL_713674, EPI_ISL_713675, EPI_ISL_713676, EPI_ISL_713677, EPI_ISL_713678, EPI_ISL_713679, EPI_ISL_713680, EPI_ISL_713681, EPI_ISL_713682, EPI_ISL_713683, EPI_ISL_713684,
EPI_ISL_713685, EPI_ISL_713686, EPI_ISL_713687, EPI_ISL_713688, EPI_ISL_713689, EPI_ISL_713690, EPI_ISL_713691, EPI_ISL_713692, EPI_ISL_713693, EPI_ISL_713694, EPI_ISL_713695, EPI_ISL_713696, EPI_ISL_713697, EPI_ISL_713698, EPI_ISL_713699, EPI_ISL_713700, EPI_ISL_713701, EPI_ISL_713702,
EPI_ISL_713703, EPI_ISL_713704, EPI_ISL_713705, EPI_ISL_713706, EPI_ISL_713707, EPI_ISL_713708, EPI_ISL_713709, EPI_ISL_713710, EPI_ISL_713711, EPI_ISL_713712, EPI_ISL_713713, EPI_ISL_713714, EPI_ISL_713715, EPI_ISL_713716, EPI_ISL_713717, EPI_ISL_713718, EPI_ISL_713719, EPI_ISL_713720,
EPI_ISL_713721, EPI_ISL_713722, EPI_ISL_713723, EPI_ISL_713724, EPI_ISL_713725, EPI_ISL_713726, EPI_ISL_713727, EPI_ISL_713728, EPI_ISL_713729, EPI_ISL_713730, EPI_ISL_713731, EPI_ISL_713732, EPI_ISL_713733, EPI_ISL_713734, EPI_ISL_713735, EPI_ISL_713736, EPI_ISL_713737, EPI_ISL_713738,
EPI_ISL_713739, EPI_ISL_713740, EPI_ISL_713741, EPI_ISL_713742, EPI_ISL_713743, EPI_ISL_713744, EPI_ISL_713745, EPI_ISL_713746, EPI_ISL_713747, EPI_ISL_713748, EPI_ISL_713749, EPI_ISL_713750, EPI_ISL_713751, EPI_ISL_713752, EPI_ISL_713753, EPI_ISL_713754, EPI_ISL_713755, EPI_ISL_713756,
EPI_ISL_713757, EPI_ISL_713758, EPI_ISL_713759, EPI_ISL_713760, EPI_ISL_713761, EPI_ISL_713762, EPI_ISL_713763, EPI_ISL_713764, EPI_ISL_713765, EPI_ISL_713766, EPI_ISL_713767, EPI_ISL_713768, EPI_ISL_713769, EPI_ISL_713770, EPI_ISL_713771, EPI_ISL_713772, EPI_ISL_713773, EPI_ISL_713774,
EPI_ISL_713775, EPI_ISL_713776, EPI_ISL_713777, EPI_ISL_713778, EPI_ISL_713779, EPI_ISL_713780, EPI_ISL_713781, EPI_ISL_713782, EPI_ISL_713783, EPI_ISL_713784, EPI_ISL_713785, EPI_ISL_713786, EPI_ISL_713787, EPI_ISL_713788, EPI_ISL_713789, EPI_ISL_713790, EPI_ISL_713791, EPI_ISL_713792,
EPI_ISL_713793, EPI_ISL_713794, EPI_ISL_713795, EPI_ISL_713796, EPI_ISL_713797, EPI_ISL_713798, EPI_ISL_713799, EPI_ISL_713800, EPI_ISL_713801, EPI_ISL_713802, EPI_ISL_713803, EPI_ISL_713804, EPI_ISL_713805, EPI_ISL_713806, EPI_ISL_713807, EPI_ISL_713808, EPI_ISL_713809, EPI_ISL_713810,
EPI_ISL_713811, EPI_ISL_713812, EPI_ISL_713813, EPI_ISL_713814, EPI_ISL_713815, EPI_ISL_713816, EPI_ISL_713817, EPI_ISL_713818, EPI_ISL_713819, EPI_ISL_713820, EPI_ISL_713821, EPI_ISL_713822, EPI_ISL_713823, EPI_ISL_713824, EPI_ISL_713825, EPI_ISL_713826, EPI_ISL_713827, EPI_ISL_713828,
EPI_ISL_713829, EPI_ISL_713830, EPI_ISL_713831, EPI_ISL_713832, EPI_ISL_713833, EPI_ISL_713834, EPI_ISL_713835, EPI_ISL_713836, EPI_ISL_713837, EPI_ISL_713838, EPI_ISL_713839, EPI_ISL_713840, EPI_ISL_713841, EPI_ISL_713842, EPI_ISL_713843, EPI_ISL_713844, EPI_ISL_713845, EPI_ISL_713846,
EPI_ISL_713847, EPI_ISL_713848, EPI_ISL_713849, EPI_ISL_713850, EPI_ISL_713851, EPI_ISL_713852, EPI_ISL_713853, EPI_ISL_713854, EPI_ISL_713855, EPI_ISL_713856, EPI_ISL_713857, EPI_ISL_713858, EPI_ISL_713859, EPI_ISL_713860, EPI_ISL_713861, EPI_ISL_713862, EPI_ISL_713863, EPI_ISL_713864,
EPI_ISL_713865, EPI_ISL_713866, EPI_ISL_713867, EPI_ISL_713868, EPI_ISL_713869, EPI_ISL_713870, EPI_ISL_713871, EPI_ISL_713872, EPI_ISL_713873, EPI_ISL_713874, EPI_ISL_713875, EPI_ISL_713876, EPI_ISL_713877, EPI_ISL_713878, EPI_ISL_713879, EPI_ISL_713880, EPI_ISL_713881, EPI_ISL_713882,
EPI_ISL_713883, EPI_ISL_713884, EPI_ISL_713885, EPI_ISL_713886, EPI_ISL_713887, EPI_ISL_713888, EPI_ISL_713889, EPI_ISL_713890, EPI_ISL_713891, EPI_ISL_713892, EPI_ISL_713893, EPI_ISL_713894, EPI_ISL_713895, EPI_ISL_713896, EPI_ISL_713897, EPI_ISL_713898, EPI_ISL_713899, EPI_ISL_713900,
EPI_ISL_713901, EPI_ISL_713902, EPI_ISL_713903, EPI_ISL_713904, EPI_ISL_713905, EPI_ISL_713906, EPI_ISL_713907, EPI_ISL_713908, EPI_ISL_713909, EPI_ISL_713910, EPI_ISL_713911, EPI_ISL_713912, EPI_ISL_713913, EPI_ISL_713914, EPI_ISL_713915, EPI_ISL_713916, EPI_ISL_713917, EPI_ISL_713918,
EPI_ISL_713919, EPI_ISL_713920, EPI_ISL_713921, EPI_ISL_713922, EPI_ISL_713923, EPI_ISL_713924, EPI_ISL_713925, EPI_ISL_713926, EPI_ISL_713927, EPI_ISL_713928, EPI_ISL_713929, EPI_ISL_713930, EPI_ISL_713931, EPI_ISL_713932, EPI_ISL_713933, EPI_ISL_713934, EPI_ISL_713935, EPI_ISL_713936,
EPI_ISL_713937, EPI_ISL_713938, EPI_ISL_713939, EPI_ISL_713940, EPI_ISL_713941, EPI_ISL_713942, EPI_ISL_713943, EPI_ISL_713944, EPI_ISL_713945, EPI_ISL_713946, EPI_ISL_713947, EPI_ISL_713948, EPI_ISL_713949, EPI_ISL_713950, EPI_ISL_713951, EPI_ISL_713952, EPI_ISL_713953, EPI_ISL_713954,
EPI_ISL_713955, EPI_ISL_713956, EPI_ISL_713957, EPI_ISL_713958, EPI_ISL_713959, EPI_ISL_713960, EPI_ISL_713961, EPI_ISL_713962, EPI_ISL_713963, EPI_ISL_713964, EPI_ISL_713965, EPI_ISL_713966, EPI_ISL_713967, EPI_ISL_713968, EPI_ISL_713969, EPI_ISL_713970, EPI_ISL_713971, EPI_ISL_713972,
EPI_ISL_713973, EPI_ISL_713974, EPI_ISL_713975, EPI_ISL_713976, EPI_ISL_713977, EPI_ISL_713978, EPI_ISL_713979, EPI_ISL_713980, EPI_ISL_713981, EPI_ISL_713982, EPI_ISL_713983, EPI_ISL_713984, EPI_ISL_713985, EPI_ISL_713986, EPI_ISL_713987, EPI_ISL_713988, EPI_ISL_713989, EPI_ISL_713990,
EPI_ISL_713991, EPI_ISL_713992, EPI_ISL_713993, EPI_ISL_713994, EPI_ISL_713995, EPI_ISL_713996, EPI_ISL_713997, EPI_ISL_713998, EPI_ISL_713999, EPI_ISL_714000, EPI_ISL_714001, EPI_ISL_714002, EPI_ISL_714003, EPI_ISL_714004, EPI_ISL_714005, EPI_ISL_714006, EPI_ISL_714007, EPI_ISL_714008,
EPI_ISL_714009, EPI_ISL_714010, EPI_ISL_714011, EPI_ISL_714012, EPI_ISL_714013, EPI_ISL_714014, EPI_ISL_714015, EPI_ISL_714016, EPI_ISL_714017, EPI_ISL_714018, EPI_ISL_714019, EPI_ISL_714020, EPI_ISL_714021, EPI_ISL_714022, EPI_ISL_714023, EPI_ISL_714024, EPI_ISL_714025, EPI_ISL_714026,
EPI_ISL_714027, EPI_ISL_714028, EPI_ISL_714029, EPI_ISL_714030, EPI_ISL_714031, EPI_ISL_714032, EPI_ISL_714033, EPI_ISL_714034, EPI_ISL_714035, EPI_ISL_714036, EPI_ISL_714037, EPI_ISL_714038, EPI_ISL_714039, EPI_ISL_714040, EPI_ISL_714041, EPI_ISL_714042, EPI_ISL_714043, EPI_ISL_714044,
EPI_ISL_714045, EPI_ISL_714046, EPI_ISL_714047, EPI_ISL_714048, EPI_ISL_714049, EPI_ISL_714050, EPI_ISL_714051, EPI_ISL_714052, EPI_ISL_714053, EPI_ISL_714054, EPI_ISL_714055, EPI_ISL_714056, EPI_ISL_714057, EPI_ISL_714058, EPI_ISL_714059, EPI_ISL_714060, EPI_ISL_714061, EPI_ISL_714062,
EPI_ISL_714063, EPI_ISL_714064, EPI_ISL_714065, EPI_ISL_714066, EPI_ISL_714067, EPI_ISL_714068, EPI_ISL_714069, EPI_ISL_714070, EPI_ISL_714071, EPI_ISL_714072, EPI_ISL_714073, EPI_ISL_714074, EPI_ISL_714075, EPI_ISL_714076, EPI_ISL_714077, EPI_ISL_714078, EPI_ISL_714079, EPI_ISL_714080,
EPI_ISL_714081, EPI_ISL_714082, EPI_ISL_714083, EPI_ISL_714084, EPI_ISL_714085, EPI_ISL_714086, EPI_ISL_714087, EPI_ISL_714088, EPI_ISL_714089, EPI_ISL_714090, EPI_ISL_714091, EPI_ISL_714092, EPI_ISL_714093, EPI_ISL_714094, EPI_ISL_714095, EPI_ISL_714096, EPI_ISL_714097, EPI_ISL_714098,
EPI_ISL_714099, EPI_ISL_714100, EPI_ISL_714101, EPI_ISL_714102, EPI_ISL_714103, EPI_ISL_714104, EPI_ISL_714105, EPI_ISL_714106, EPI_ISL_714107, EPI_ISL_714108, EPI_ISL_714109, EPI_ISL_714110, EPI_ISL_714111, EPI_ISL_714112, EPI_ISL_714113, EPI_ISL_714114, EPI_ISL_714115, EPI_ISL_714116,
EPI_ISL_714117, EPI_ISL_714118, EPI_ISL_714119, EPI_ISL_714120, EPI_ISL_714121, EPI_ISL_714122, EPI_ISL_714123, EPI_ISL_714124, EPI_ISL_714125, EPI_ISL_714126, EPI_ISL_714127, EPI_ISL_714128, EPI_ISL_714129, EPI_ISL_714130, EPI_ISL_714131, EPI_ISL_714132, EPI_ISL_714133, EPI_ISL_714134,
EPI_ISL_714135, EPI_ISL_714136, EPI_ISL_714137, EPI_ISL_714138, EPI_ISL_714139, EPI_ISL_714140, EPI_ISL_714141, EPI_ISL_714142, EPI_ISL_714143, EPI_ISL_714144, EPI_ISL_714145, EPI_ISL_714146, EPI_ISL_714147, EPI_ISL_714148, EPI_ISL_714149, EPI_ISL_714150, EPI_ISL_714151, EPI_ISL_714152,
EPI_ISL_714153, EPI_ISL_714154, EPI_ISL_714155, EPI_ISL_714156, EPI_ISL_714157, EPI_ISL_714158, EPI_ISL_714159, EPI_ISL_714160, EPI_ISL_714161, EPI_ISL_714162, EPI_ISL_714163, EPI_ISL_714164, EPI_ISL_714165, EPI_ISL_714166, EPI_ISL_714167, EPI_ISL_714168, EPI_ISL_714169, EPI_ISL_714170,
EPI_ISL_714171, EPI_ISL_714172, EPI_ISL_714173, EPI_ISL_714174, EPI_ISL_714175, EPI_ISL_714176, EPI_ISL_714177, EPI_ISL_714178, EPI_ISL_714179, EPI_ISL_714180, EPI_ISL_714181, EPI_ISL_714182, EPI_ISL_714183, EPI_ISL_714184, EPI_ISL_714185, EPI_ISL_714186, EPI_ISL_714187, EPI_ISL_714188,
EPI_ISL_714189, EPI_ISL_714190, EPI_ISL_714192, EPI_ISL_714193, EPI_ISL_714194, EPI_ISL_714195, EPI_ISL_714196, EPI_ISL_714197, EPI_ISL_714198, EPI_ISL_714199, EPI_ISL_714200, EPI_ISL_714201, EPI_ISL_714202, EPI_ISL_714203, EPI_ISL_714204, EPI_ISL_714205, EPI_ISL_714206, EPI_ISL_714207,
EPI_ISL_714208, EPI_ISL_714209, EPI_ISL_714210, EPI_ISL_714211, EPI_ISL_714212, EPI_ISL_714213, EPI_ISL_714214, EPI_ISL_714215, EPI_ISL_714216, EPI_ISL_714217, EPI_ISL_714218, EPI_ISL_714219, EPI_ISL_714220, EPI_ISL_714222, EPI_ISL_714223, EPI_ISL_714224, EPI_ISL_714225, EPI_ISL_714226,
EPI_ISL_714227, EPI_ISL_714228, EPI_ISL_714229, EPI_ISL_714230, EPI_ISL_714231, EPI_ISL_714232, EPI_ISL_714233, EPI_ISL_714234, EPI_ISL_714235, EPI_ISL_714236, EPI_ISL_714237, EPI_ISL_714238, EPI_ISL_714239, EPI_ISL_714240, EPI_ISL_714241, EPI_ISL_714242, EPI_ISL_714243, EPI_ISL_714244,
EPI_ISL_714245, EPI_ISL_714246, EPI_ISL_714247, EPI_ISL_714248, EPI_ISL_714249, EPI_ISL_714250, EPI_ISL_714251, EPI_ISL_714252, EPI_ISL_714254, EPI_ISL_714255, EPI_ISL_714256, EPI_ISL_714257, EPI_ISL_714258, EPI_ISL_714259, EPI_ISL_714260, EPI_ISL_714261, EPI_ISL_714262, EPI_ISL_714263,
EPI_ISL_714264, EPI_ISL_714265, EPI_ISL_714266, EPI_ISL_714267, EPI_ISL_714268, EPI_ISL_714269, EPI_ISL_714270, EPI_ISL_714271, EPI_ISL_714289, EPI_ISL_714290, EPI_ISL_714291, EPI_ISL_714292, EPI_ISL_714293, EPI_ISL_714294, EPI_ISL_714295, EPI_ISL_714296, EPI_ISL_714297, EPI_ISL_714298,
EPI_ISL_714299, EPI_ISL_714300, EPI_ISL_714301, EPI_ISL_714302, EPI_ISL_714303, EPI_ISL_714304, EPI_ISL_714305, EPI_ISL_714306, EPI_ISL_714307, EPI_ISL_714308, EPI_ISL_714309, EPI_ISL_714310, EPI_ISL_714311, EPI_ISL_714312, EPI_ISL_714313, EPI_ISL_714314, EPI_ISL_714315, EPI_ISL_714316,
EPI_ISL_714317, EPI_ISL_714318, EPI_ISL_714319, EPI_ISL_714320, EPI_ISL_714321, EPI_ISL_714322, EPI_ISL_714323, EPI_ISL_714324, EPI_ISL_714325, EPI_ISL_714326, EPI_ISL_714327, EPI_ISL_714328, EPI_ISL_714329, EPI_ISL_714330, EPI_ISL_714331, EPI_ISL_714332, EPI_ISL_714333, EPI_ISL_714334,
EPI_ISL_714335, EPI_ISL_714336, EPI_ISL_714337, EPI_ISL_714338, EPI_ISL_714339, EPI_ISL_714340, EPI_ISL_714341, EPI_ISL_714342, EPI_ISL_714343, EPI_ISL_714344, EPI_ISL_714345, EPI_ISL_714346, EPI_ISL_714347, EPI_ISL_714348, EPI_ISL_714349, EPI_ISL_714350, EPI_ISL_714351, EPI_ISL_714352,
EPI_ISL_714353, EPI_ISL_714354, EPI_ISL_714355, EPI_ISL_714356, EPI_ISL_714357, EPI_ISL_714358, EPI_ISL_714359, EPI_ISL_714360, EPI_ISL_714361, EPI_ISL_714362, EPI_ISL_714363, EPI_ISL_714364, EPI_ISL_714365, EPI_ISL_714366, EPI_ISL_714367, EPI_ISL_714368, EPI_ISL_714369, EPI_ISL_714370,

EPI_ISL_714371, EPI_ISL_714372, EPI_ISL_714373, EPI_ISL_714374, EPI_ISL_714375, EPI_ISL_714376, EPI_ISL_714377, EPI_ISL_714378, EPI_ISL_714379, EPI_ISL_714380, EPI_ISL_714381, EPI_ISL_714382, EPI_ISL_714383, EPI_ISL_714384, EPI_ISL_714385, EPI_ISL_714386, EPI_ISL_714387, EPI_ISL_714388, EPI_ISL_714389, EPI_ISL_714390, EPI_ISL_714391, EPI_ISL_714392, EPI_ISL_714393, EPI_ISL_714394, EPI_ISL_714395, EPI_ISL_714396, EPI_ISL_714397, EPI_ISL_714398, EPI_ISL_714399, EPI_ISL_714400, EPI_ISL_714401, EPI_ISL_714402, EPI_ISL_714403, EPI_ISL_714404, EPI_ISL_714405, EPI_ISL_714406, EPI_ISL_714407, EPI_ISL_714408, EPI_ISL_714409, EPI_ISL_714410, EPI_ISL_714411, EPI_ISL_714412, EPI_ISL_714413, EPI_ISL_714414, EPI_ISL_714415, EPI_ISL_714416, EPI_ISL_714417, EPI_ISL_714418, EPI_ISL_714419, EPI_ISL_714420, EPI_ISL_714421, EPI_ISL_714422, EPI_ISL_714423, EPI_ISL_714424, EPI_ISL_714425, EPI_ISL_714426, EPI_ISL_714427, EPI_ISL_714428, EPI_ISL_714429, EPI_ISL_714430, EPI_ISL_714431, EPI_ISL_714432, EPI_ISL_714433, EPI_ISL_714434, EPI_ISL_714435, EPI_ISL_714436, EPI_ISL_714437, EPI_ISL_714438, EPI_ISL_714439, EPI_ISL_714440, EPI_ISL_714441, EPI_ISL_714442, EPI_ISL_714443, EPI_ISL_714444, EPI_ISL_714445, EPI_ISL_714446, EPI_ISL_714447, EPI_ISL_714448, EPI_ISL_714449, EPI_ISL_714450, EPI_ISL_714451, EPI_ISL_714452, EPI_ISL_714453, EPI_ISL_714454, EPI_ISL_714455, EPI_ISL_714456, EPI_ISL_714457, EPI_ISL_714458, EPI_ISL_714459, EPI_ISL_714460, EPI_ISL_714461, EPI_ISL_714462, EPI_ISL_714463, EPI_ISL_714464, EPI_ISL_714465, EPI_ISL_714466, EPI_ISL_714467, EPI_ISL_714468, EPI_ISL_714469, EPI_ISL_714470, EPI_ISL_714471, EPI_ISL_714472, EPI_ISL_714473, EPI_ISL_714474, EPI_ISL_714475, EPI_ISL_714476, EPI_ISL_714477, EPI_ISL_714478, EPI_ISL_714479, EPI_ISL_714480, EPI_ISL_714481, EPI_ISL_714482, EPI_ISL_714483, EPI_ISL_714484, EPI_ISL_714485, EPI_ISL_714486, EPI_ISL_714487, EPI_ISL_714488, EPI_ISL_714489, EPI_ISL_714490, EPI_ISL_714491, EPI_ISL_714492, EPI_ISL_714493, EPI_ISL_714494, EPI_ISL_714495, EPI_ISL_714496, EPI_ISL_714497, EPI_ISL_714498, EPI_ISL_714499, EPI_ISL_714500, EPI_ISL_714501, EPI_ISL_714502, EPI_ISL_714503, EPI_ISL_714504, EPI_ISL_714505, EPI_ISL_714506, EPI_ISL_714507, EPI_ISL_714508, EPI_ISL_714509, EPI_ISL_714510, EPI_ISL_714511, EPI_ISL_714512, EPI_ISL_714513, EPI_ISL_714514, EPI_ISL_714515, EPI_ISL_714516, EPI_ISL_714517, EPI_ISL_714518, EPI_ISL_714519, EPI_ISL_714520, EPI_ISL_714521, EPI_ISL_714522, EPI_ISL_714523, EPI_ISL_714524, EPI_ISL_714525, EPI_ISL_714526, EPI_ISL_714527, EPI_ISL_714528, EPI_ISL_714529, EPI_ISL_714530, EPI_ISL_714531, EPI_ISL_714532, EPI_ISL_714533, EPI_ISL_714534, EPI_ISL_714535, EPI_ISL_714536, EPI_ISL_714537, EPI_ISL_714538, EPI_ISL_714539, EPI_ISL_714540, EPI_ISL_714541, EPI_ISL_714542, EPI_ISL_714543, EPI_ISL_714544, EPI_ISL_714545, EPI_ISL_714546, EPI_ISL_714547, EPI_ISL_714548, EPI_ISL_714549, EPI_ISL_714550, EPI_ISL_714551, EPI_ISL_714552, EPI_ISL_714553, EPI_ISL_714554, EPI_ISL_714555, EPI_ISL_714556, EPI_ISL_714557, EPI_ISL_714558, EPI_ISL_714559, EPI_ISL_714560, EPI_ISL_714561, EPI_ISL_714562, EPI_ISL_714563, EPI_ISL_714564, EPI_ISL_714565, EPI_ISL_714566, EPI_ISL_714567, EPI_ISL_714568, EPI_ISL_714569, EPI_ISL_714570, EPI_ISL_714571, EPI_ISL_714572, EPI_ISL_714573, EPI_ISL_714574, EPI_ISL_714575, EPI_ISL_714576, EPI_ISL_714577, EPI_ISL_714578, EPI_ISL_714579, EPI_ISL_714580, EPI_ISL_714581, EPI_ISL_714582, EPI_ISL_714583, EPI_ISL_714584, EPI_ISL_714585, EPI_ISL_714586, EPI_ISL_714587, EPI_ISL_714588, EPI_ISL_714589, EPI_ISL_714590, EPI_ISL_714591, EPI_ISL_714592, EPI_ISL_714593, EPI_ISL_714594, EPI_ISL_714595, EPI_ISL_714596, EPI_ISL_714597, EPI_ISL_714598, EPI_ISL_714599, EPI_ISL_714600, EPI_ISL_714601, EPI_ISL_714602, EPI_ISL_714603, EPI_ISL_714604, EPI_ISL_714605, EPI_ISL_714606, EPI_ISL_714607, EPI_ISL_714608, EPI_ISL_714609, EPI_ISL_714610, EPI_ISL_714611, EPI_ISL_714612, EPI_ISL_714613, EPI_ISL_714614, EPI_ISL_714615, EPI_ISL_714616, EPI_ISL_714617, EPI_ISL_714618, EPI_ISL_714619, EPI_ISL_714620, EPI_ISL_714621, EPI_ISL_714622, EPI_ISL_714623, EPI_ISL_714624, EPI_ISL_714625, EPI_ISL_714626, EPI_ISL_714627, EPI_ISL_714628, EPI_ISL_714629, EPI_ISL_714630, EPI_ISL_714631, EPI_ISL_714632, EPI_ISL_714633, EPI_ISL_714634, EPI_ISL_714635, EPI_ISL_714636, EPI_ISL_714637, EPI_ISL_714638, EPI_ISL_714639, EPI_ISL_714640, EPI_ISL_714641, EPI_ISL_714642, EPI_ISL_714643, EPI_ISL_714644, EPI_ISL_714645, EPI_ISL_714646, EPI_ISL_714647, EPI_ISL_714648, EPI_ISL_714649, EPI_ISL_714650, EPI_ISL_714651, EPI_ISL_714652, EPI_ISL_714653, EPI_ISL_714654, EPI_ISL_714655, EPI_ISL_714656, EPI_ISL_714657, EPI_ISL_714658, EPI_ISL_714659, EPI_ISL_714660, EPI_ISL_714661, EPI_ISL_714662, EPI_ISL_714663, EPI_ISL_714664, EPI_ISL_714665, EPI_ISL_714666, EPI_ISL_714667, EPI_ISL_714668, EPI_ISL_714669, EPI_ISL_714670, EPI_ISL_714671, EPI_ISL_714672, EPI_ISL_714673, EPI_ISL_714674, EPI_ISL_714675, EPI_ISL_714676, EPI_ISL_714677, EPI_ISL_714678, EPI_ISL_714679, EPI_ISL_714680, EPI_ISL_714681, EPI_ISL_714682, EPI_ISL_714683, EPI_ISL_714684, EPI_ISL_714685, EPI_ISL_714686, EPI_ISL_714687, EPI_ISL_714688, EPI_ISL_714689, EPI_ISL_714690, EPI_ISL_714691, EPI_ISL_714692, EPI_ISL_714693, EPI_ISL_714694, EPI_ISL_714695, EPI_ISL_714696, EPI_ISL_714697, EPI_ISL_714698, EPI_ISL_714699, EPI_ISL_714700, EPI_ISL_714701, EPI_ISL_714702, EPI_ISL_714703, EPI_ISL_714704, EPI_ISL_714705, EPI_ISL_714706, EPI_ISL_714707, EPI_ISL_714708, EPI_ISL_714709, EPI_ISL_714710, EPI_ISL_714711, EPI_ISL_714712, EPI_ISL_714713, EPI_ISL_714714, EPI_ISL_714715, EPI_ISL_714716, EPI_ISL_714717, EPI_ISL_714718, EPI_ISL_714719, EPI_ISL_714720, EPI_ISL_714721, EPI_ISL_714722, EPI_ISL_714723, EPI_ISL_714724, EPI_ISL_714725, EPI_ISL_714726, EPI_ISL_714727, EPI_ISL_714728, EPI_ISL_714729, EPI_ISL_714730, EPI_ISL_714731, EPI_ISL_714732, EPI_ISL_714733, EPI_ISL_714734, EPI_ISL_714735, EPI_ISL_714736, EPI_ISL_714737, EPI_ISL_714738, EPI_ISL_714739, EPI_ISL_714740, EPI_ISL_714741, EPI_ISL_714742, EPI_ISL_714743, EPI_ISL_714744, EPI_ISL_714745, EPI_ISL_714746, EPI_ISL_714747, EPI_ISL_714748, EPI_ISL_714749, EPI_ISL_714750, EPI_ISL_714751, EPI_ISL_714752, EPI_ISL_714753, EPI_ISL_714754, EPI_ISL_714755, EPI_ISL_714756, EPI_ISL_714757, EPI_ISL_714758, EPI_ISL_714759, EPI_ISL_714760, EPI_ISL_714761, EPI_ISL_714762, EPI_ISL_714763, EPI_ISL_714764, EPI_ISL_714765, EPI_ISL_714766, EPI_ISL_714767, EPI_ISL_714768, EPI_ISL_714769, EPI_ISL_714770, EPI_ISL_714771, EPI_ISL_714772, EPI_ISL_714773, EPI_ISL_714774, EPI_ISL_714775, EPI_ISL_714776, EPI_ISL_714777, EPI_ISL_714778, EPI_ISL_714779, EPI_ISL_714780, EPI_ISL_714781, EPI_ISL_714782, EPI_ISL_714783, EPI_ISL_714784, EPI_ISL_714785, EPI_ISL_714786, EPI_ISL_714787, EPI_ISL_714788, EPI_ISL_714789, EPI_ISL_714790, EPI_ISL_714791, EPI_ISL_714792, EPI_ISL_714793, EPI_ISL_714794, EPI_ISL_714795, EPI_ISL_714796, EPI_ISL_714797, EPI_ISL_714798, EPI_ISL_714799, EPI_ISL_714800, EPI_ISL_714801, EPI_ISL_714802, EPI_ISL_714803, EPI_ISL_714804, EPI_ISL_714805, EPI_ISL_714806, EPI_ISL_714807, EPI_ISL_714808, EPI_ISL_714809, EPI_ISL_714810, EPI_ISL_714811, EPI_ISL_714812, EPI_ISL_714813, EPI_ISL_714814, EPI_ISL_714815, EPI_ISL_714816, EPI_ISL_714817, EPI_ISL_714818, EPI_ISL_714819, EPI_ISL_714820, EPI_ISL_714821, EPI_ISL_714822, EPI_ISL_714823, EPI_ISL_714824, EPI_ISL_714825, EPI_ISL_714826, EPI_ISL_714827, EPI_ISL_714828, EPI_ISL_714829, EPI_ISL_714830, EPI_ISL_714831, EPI_ISL_714832, EPI_ISL_714833, EPI_ISL_714834, EPI_ISL_714835, EPI_ISL_714836, EPI_ISL_714837, EPI_ISL_714838, EPI_ISL_714839, EPI_ISL_714840, EPI_ISL_714841, EPI_ISL_714842, EPI_ISL_714843, EPI_ISL_714844, EPI_ISL_714845, EPI_ISL_714846, EPI_ISL_714847, EPI_ISL_714848, EPI_ISL_714849, EPI_ISL_714850, EPI_ISL_714851, EPI_ISL_714852, EPI_ISL_714853, EPI_ISL_714854, EPI_ISL_714855, EPI_ISL_714856, EPI_ISL_714857, EPI_ISL_714858, EPI_ISL_714859, EPI_ISL_714860, EPI_ISL_714861, EPI_ISL_714862, EPI_ISL_714863, EPI_ISL_714864, EPI_ISL_714865, EPI_ISL_714866, EPI_ISL_714867, EPI_ISL_714868, EPI_ISL_714869, EPI_ISL_714870, EPI_ISL_714871, EPI_ISL_714872, EPI_ISL_714873, EPI_ISL_714874, EPI_ISL_714875, EPI_ISL_714876, EPI_ISL_714877, EPI_ISL_714878, EPI_ISL_714879, EPI_ISL_714880, EPI_ISL_714881, EPI_ISL_714882, EPI_ISL_714883, EPI_ISL_714884, EPI_ISL_714885, EPI_ISL_714886, EPI_ISL_714887, EPI_ISL_714888, EPI_ISL_714889, EPI_ISL_714890, EPI_ISL_714891, EPI_ISL_714892, EPI_ISL_714893, EPI_ISL_714894, EPI_ISL_714895, EPI_ISL_714896, EPI_ISL_714897, EPI_ISL_714898, EPI_ISL_714899, EPI_ISL_714900, EPI_ISL_714901, EPI_ISL_714902, EPI_ISL_714903, EPI_ISL_714904, EPI_ISL_714905, EPI_ISL_714906, EPI_ISL_714907, EPI_ISL_714908, EPI_ISL_714909, EPI_ISL_714910, EPI_ISL_714911, EPI_ISL_714912, EPI_ISL_714913, EPI_ISL_714914, EPI_ISL_714915, EPI_ISL_714916, EPI_ISL_714917, EPI_ISL_714918, EPI_ISL_714919, EPI_ISL_714920, EPI_ISL_714921, EPI_ISL_714922, EPI_ISL_714923, EPI_ISL_714924, EPI_ISL_714925, EPI_ISL_714926, EPI_ISL_714927, EPI_ISL_714928, EPI_ISL_714929, EPI_ISL_714930, EPI_ISL_714931, EPI_ISL_714932, EPI_ISL_714933, EPI_ISL_714934, EPI_ISL_714935, EPI_ISL_714936			
see above	Department of Virus and Microbiological Special Diagnostics, Statens Serum Institut, Copenhagen, Denmark	Albertsen Lab, Department of Chemistry and Bioscience, Aalborg University, Denmark	Danish Covid-19 Genome Consortium
EPI_ISL_717573	Lab voor klinische biologie	Onderzoeksgroep Virologie	Nick Vereecke, Laurens Lambrechts, Marthe Pauwels, Bruno Verhasselt, Linos Vandekerckhove, Hans Nauwynck, Sebastiaan Theuns
EPI_ISL_717574, EPI_ISL_717575, EPI_ISL_717576	Lab voor klinische biologie	Onderzoeksgroep Virologie	Laurens Lambrechts, Nick Vereecke, Marthe Pauwels, Bruno Verhasselt, Linos Vandekerckhove, Hans Nauwynck, Sebastiaan Theuns
EPI_ISL_717577	Lab voor klinische biologie	Onderzoeksgroep Virologie	Nick Vereecke, Laurens Lambrechts, Marthe Pauwels, Bruno Verhasselt, Linos Vandekerckhove, Hans Nauwynck, Sebastiaan Theuns
EPI_ISL_717578, EPI_ISL_717579	Lab voor klinische biologie	Onderzoeksgroep Virologie	Laurens Lambrechts, Nick Vereecke, Marthe Pauwels, Bruno Verhasselt, Linos Vandekerckhove, Hans Nauwynck, Sebastiaan Theuns
EPI_ISL_717580, EPI_ISL_717581	Lab voor klinische biologie	Onderzoeksgroep Virologie	Nick Vereecke, Laurens Lambrechts, Marthe Pauwels, Bruno Verhasselt, Linos Vandekerckhove, Hans Nauwynck, Sebastiaan Theuns
EPI_ISL_717582, EPI_ISL_717583, EPI_ISL_717584, EPI_ISL_717585	Lab voor klinische biologie	Onderzoeksgroep Virologie	Laurens Lambrechts, Nick Vereecke, Marthe Pauwels, Bruno Verhasselt, Linos Vandekerckhove, Hans Nauwynck, Sebastiaan Theuns
EPI_ISL_717586	Lab voor klinische biologie	Onderzoeksgroep Virologie	Nick Vereecke, Laurens Lambrechts, Marthe Pauwels, Bruno Verhasselt, Linos Vandekerckhove, Hans Nauwynck, Sebastiaan Theuns
EPI_ISL_717603	Lab voor klinische biologie	Onderzoeksgroep Virologie	Laurens Lambrechts, Nick Vereecke, Marthe Pauwels, Bruno Verhasselt, Linos Vandekerckhove, Hans Nauwynck, Sebastiaan Theuns
EPI_ISL_717604, EPI_ISL_717605	Lab voor klinische biologie	Onderzoeksgroep Virologie	Nick Vereecke, Laurens Lambrechts, Marthe Pauwels, Bruno Verhasselt, Linos Vandekerckhove, Hans Nauwynck, Sebastiaan Theuns
EPI_ISL_717606, EPI_ISL_717607, EPI_ISL_717608, EPI_ISL_717609	Lab voor klinische biologie	Onderzoeksgroep Virologie	Laurens Lambrechts, Nick Vereecke, Marthe Pauwels, Bruno Verhasselt, Linos Vandekerckhove, Hans Nauwynck, Sebastiaan Theuns
EPI_ISL_717610, EPI_ISL_717611	Lab voor klinische biologie	Onderzoeksgroep Virologie	Nick Vereecke, Laurens Lambrechts, Marthe Pauwels, Bruno Verhasselt, Linos Vandekerckhove, Hans Nauwynck, Sebastiaan Theuns
EPI_ISL_717612, EPI_ISL_717613, EPI_ISL_717614, EPI_ISL_717615, EPI_ISL_717616	Lab voor klinische biologie	Onderzoeksgroep Virologie	Laurens Lambrechts, Nick Vereecke, Marthe Pauwels, Bruno Verhasselt, Linos Vandekerckhove, Hans Nauwynck, Sebastiaan Theuns
EPI_ISL_717617, EPI_ISL_717618, EPI_ISL_717619, EPI_ISL_717620, EPI_ISL_717621, EPI_ISL_717622	Lab voor klinische biologie	Onderzoeksgroep Virologie	Nick Vereecke, Laurens Lambrechts, Marthe Pauwels, Bruno Verhasselt, Linos Vandekerckhove, Hans Nauwynck, Sebastiaan Theuns
EPI_ISL_717623, EPI_ISL_717624, EPI_ISL_717625	Lab voor klinische biologie	Onderzoeksgroep Virologie	Laurens Lambrechts, Nick Vereecke, Marthe Pauwels, Bruno Verhasselt, Linos Vandekerckhove, Hans Nauwynck, Sebastiaan Theuns
EPI_ISL_717626, EPI_ISL_717627, EPI_ISL_717628, EPI_ISL_717629, EPI_ISL_717630, EPI_ISL_717631	Lab voor klinische biologie	Onderzoeksgroep Virologie	Nick Vereecke, Laurens Lambrechts, Marthe Pauwels, Bruno Verhasselt, Linos Vandekerckhove, Hans Nauwynck, Sebastiaan Theuns
EPI_ISL_717632	Lab voor klinische biologie	Onderzoeksgroep Virologie	Laurens Lambrechts, Nick Vereecke, Marthe Pauwels, Bruno Verhasselt, Linos Vandekerckhove, Hans Nauwynck, Sebastiaan Theuns
EPI_ISL_717633	Lab voor klinische biologie	Onderzoeksgroep Virologie	Nick Vereecke, Laurens Lambrechts, Marthe Pauwels, Bruno Verhasselt, Linos Vandekerckhove, Hans Nauwynck, Sebastiaan Theuns
EPI_ISL_717692, EPI_ISL_717694, EPI_ISL_717695, EPI_ISL_717696, EPI_ISL_717697, EPI_ISL_717698, EPI_ISL_717699, EPI_ISL_717700	Trinidad Public Health Laboratory	Carrington Lab, Department of PreClinical Sciences, Faculty of Medical Sciences, The University of the West Indies	Nikita S. D. Sahadeo, Arianne Brown-Jordan, Sarah Hill, Vernie Ramkissoon, Naresh Nandram, Avery Hinds, Jerome Foster, Stanley Giddings, Karla Georges, Marsha Ivey, Rahul Naidu, Risha Singh, SueMin Nathaniel, Rajini Haraksingh, Jaya Jayaraman, Chinna Chinnadurai, Adesh Ramsubhag, Nuno Faria, Oliver Pybus, Christopher Oura, Gabriel Escobar, Christine V. F. Carrington
EPI_ISL_717701, EPI_ISL_717702, EPI_ISL_717703, EPI_ISL_717704, EPI_ISL_717705, EPI_ISL_717706, EPI_ISL_717707, EPI_ISL_717708, EPI_ISL_717710, EPI_ISL_717711, EPI_ISL_717712, EPI_ISL_717713			
see above	Area of Virology, Serology and Virology Division (SAViD), New South Wales Health Pathology Randwick	Virology Research Laboratory; Area of Virology, Serology and Virology Division (SAViD), New South Wales Health Pathology Randwick	Foster, C.; Au, J.; Ruiz Silva, M.; Deveson, I.; Bull, R.; Van Hal, S.; Rawlinson, W.
EPI_ISL_717734, EPI_ISL_717737, EPI_ISL_717741, EPI_ISL_717752	Kingston Health Sciences Centre and Queen's University	Ontario Institute for Cancer Research	Prameet M. Sheth,Calvin Sjaarda,Robert Colautti,Katya Douchant,Ilinca Lungu,Bernard Lam,Paul Krzyzanowski,Michael Laszloffy,Lawrence E. Heisler,Richard de Borja,Jared T. Simpson
EPI_ISL_717766, EPI_ISL_717767, EPI_ISL_717769, EPI_ISL_717770, EPI_ISL_717771, EPI_ISL_717772, EPI_ISL_717773, EPI_ISL_717774, EPI_ISL_717775, EPI_ISL_717776, EPI_ISL_717777, EPI_ISL_717778, EPI_ISL_717779, EPI_ISL_717780, EPI_ISL_717781, EPI_ISL_717782			
see above	UW Virology Lab	UW Virology Lab	Pavitra Roychoudhury, Hong Xie, Lasata Shrestha, Michelle Lin, Meei-Li Huang, Keith R Jerome, Alexander Greninger
EPI_ISL_717785, EPI_ISL_717786, EPI_ISL_717787, EPI_ISL_717788,	LACEN RJ - Noel Nutels	Bioinformatics Laboratory / LNCC	Carolina M Voloch, Ronaldo da Silva F Jr, Luiz G P de Almeida, Cynthia C Cardoso, Otavio Bustrolini, Alexandra L Gerber, Ana Paula de C Guimarães, Diana Mariani, Andréa Cony Cavalcanti, Claudia dos Santos Rodrigues, Terezinha M P P Castiñeira, Amilcar Tanuri, Ana Tereza R de Vasconcelos

EPI_ISL_717789, EPI_ISL_717790			
EPI_ISL_717791	Laboratorio de Virologia Molecular / UFRJ	Bioinformatics Laboratory / LNCC	Carolina M Voloch, Ronaldo da Silva F Jr, Luiz G P de Almeida, Cynthia C Cardoso, Otavio Bustrolini, Alexandra L Gerber, Ana Paula de C Guimarães, Diana Mariani, Andréa Cony Cavalcanti, Claudia dos Santos Rodrigues, Terezinha M P P Castiñeira, Amilcar Tanuri, Ana Tereza R de Vasconcelos
EPI_ISL_717792	LACEN RJ - Noel Nutels	Bioinformatics Laboratory / LNCC	Carolina M Voloch, Ronaldo da Silva F Jr, Luiz G P de Almeida, Cynthia C Cardoso, Otavio Bustrolini, Alexandra L Gerber, Ana Paula de C Guimarães, Diana Mariani, Andréa Cony Cavalcanti, Claudia dos Santos Rodrigues, Terezinha M P P Castiñeira, Amilcar Tanuri, Ana Tereza R de Vasconcelos
EPI_ISL_717793	Laboratorio de Virologia Molecular / UFRJ	Bioinformatics Laboratory / LNCC	Carolina M Voloch, Ronaldo da Silva F Jr, Luiz G P de Almeida, Cynthia C Cardoso, Otavio Bustrolini, Alexandra L Gerber, Ana Paula de C Guimarães, Diana Mariani, Andréa Cony Cavalcanti, Claudia dos Santos Rodrigues, Terezinha M P P Castiñeira, Amilcar Tanuri, Ana Tereza R de Vasconcelos
EPI_ISL_717794	LACEN RJ - Noel Nutels	Bioinformatics Laboratory / LNCC	Carolina M Voloch, Ronaldo da Silva F Jr, Luiz G P de Almeida, Cynthia C Cardoso, Otavio Bustrolini, Alexandra L Gerber, Ana Paula de C Guimarães, Diana Mariani, Andréa Cony Cavalcanti, Claudia dos Santos Rodrigues, Terezinha M P P Castiñeira, Amilcar Tanuri, Ana Tereza R de Vasconcelos
EPI_ISL_717806, EPI_ISL_717807, EPI_ISL_717808	Laboratorio de Virologia Molecular / UFRJ	Bioinformatics Laboratory / LNCC	Carolina M Voloch, Ronaldo da Silva F Jr, Luiz G P de Almeida, Cynthia C Cardoso, Otavio Bustrolini, Alexandra L Gerber, Ana Paula de C Guimarães, Diana Mariani, Andréa Cony Cavalcanti, Claudia dos Santos Rodrigues, Terezinha M P P Castiñeira, Amilcar Tanuri, Ana Tereza R de Vasconcelos
EPI_ISL_717809	LACEN Dr. Francisco Rimolo Neto	Bioinformatics Laboratory / LNCC	Carolina M Voloch, Ronaldo da Silva F Jr, Luiz G P de Almeida, Cynthia C Cardoso, Otavio Bustrolini, Alexandra L Gerber, Ana Paula de C Guimarães, Diana Mariani, Andréa Cony Cavalcanti, Claudia dos Santos Rodrigues, Terezinha M P P Castiñeira, Amilcar Tanuri, Ana Tereza R de Vasconcelos
EPI_ISL_717812, EPI_ISL_717813, EPI_ISL_717814, EPI_ISL_717815, EPI_ISL_717816, EPI_ISL_717817, EPI_ISL_717818, EPI_ISL_717819, EPI_ISL_717820, EPI_ISL_717821, EPI_ISL_717822, EPI_ISL_717823, EPI_ISL_717824, EPI_ISL_717825, EPI_ISL_717826, EPI_ISL_717827, EPI_ISL_717828, EPI_ISL_717829, EPI_ISL_717830, EPI_ISL_717831			
see above	Laboratorio de Virologia Molecular / UFRJ	Bioinformatics Laboratory / LNCC	Carolina M Voloch, Ronaldo da Silva F Jr, Luiz G P de Almeida, Cynthia C Cardoso, Otavio Bustrolini, Alexandra L Gerber, Ana Paula de C Guimarães, Diana Mariani, Andréa Cony Cavalcanti, Claudia dos Santos Rodrigues, Terezinha M P P Castiñeira, Amilcar Tanuri, Ana Tereza R de Vasconcelos
EPI_ISL_717832, EPI_ISL_717833, EPI_ISL_717834, EPI_ISL_717835, EPI_ISL_717836, EPI_ISL_717841	LACEN Dr. Francisco Rimolo Neto	Bioinformatics Laboratory / LNCC	Carolina M Voloch, Ronaldo da Silva F Jr, Luiz G P de Almeida, Cynthia C Cardoso, Otavio Bustrolini, Alexandra L Gerber, Ana Paula de C Guimarães, Diana Mariani, Andréa Cony Cavalcanti, Claudia dos Santos Rodrigues, Terezinha M P P Castiñeira, Amilcar Tanuri, Ana Tereza R de Vasconcelos
EPI_ISL_717845, EPI_ISL_717846, EPI_ISL_717847, EPI_ISL_717848, EPI_ISL_717849, EPI_ISL_717850, EPI_ISL_717851, EPI_ISL_717852, EPI_ISL_717853, EPI_ISL_717854, EPI_ISL_717855, EPI_ISL_717856, EPI_ISL_717857, EPI_ISL_717858, EPI_ISL_717859, EPI_ISL_717860, EPI_ISL_717861, EPI_ISL_717862, EPI_ISL_717863, EPI_ISL_717864, EPI_ISL_717865, EPI_ISL_717866, EPI_ISL_717867, EPI_ISL_717868, EPI_ISL_717869, EPI_ISL_717870, EPI_ISL_717871, EPI_ISL_717872, EPI_ISL_717873, EPI_ISL_717874, EPI_ISL_717875, EPI_ISL_717876, EPI_ISL_717877, EPI_ISL_717878, EPI_ISL_717879, EPI_ISL_717880, EPI_ISL_717881, EPI_ISL_717882, EPI_ISL_717883, EPI_ISL_717884, EPI_ISL_717885, EPI_ISL_717886, EPI_ISL_717887, EPI_ISL_717888, EPI_ISL_717889, EPI_ISL_717890, EPI_ISL_717891, EPI_ISL_717892, EPI_ISL_717893, EPI_ISL_717894, EPI_ISL_717895, EPI_ISL_717896, EPI_ISL_717897, EPI_ISL_717898			
see above	Laboratorio de Virologia Molecular / UFRJ	Bioinformatics Laboratory / LNCC	Carolina M Voloch, Ronaldo da Silva F Jr, Luiz G P de Almeida, Cynthia C Cardoso, Otavio Bustrolini, Alexandra L Gerber, Ana Paula de C Guimarães, Diana Mariani, Andréa Cony Cavalcanti, Claudia dos Santos Rodrigues, Terezinha M P P Castiñeira, Amilcar Tanuri, Ana Tereza R de Vasconcelos
EPI_ISL_717899, EPI_ISL_717900, EPI_ISL_717901, EPI_ISL_717902, EPI_ISL_717903, EPI_ISL_717904, EPI_ISL_717905, EPI_ISL_717906, EPI_ISL_717907, EPI_ISL_717908, EPI_ISL_717909			
see above	LACEN RJ - Noel Nutels	Bioinformatics Laboratory / LNCC	Carolina M Voloch, Ronaldo da Silva F Jr, Luiz G P de Almeida, Cynthia C Cardoso, Otavio Bustrolini, Alexandra L Gerber, Ana Paula de C Guimarães, Diana Mariani, Andréa Cony Cavalcanti, Claudia dos Santos Rodrigues, Terezinha M P P Castiñeira, Amilcar Tanuri, Ana Tereza R de Vasconcelos
EPI_ISL_717911, EPI_ISL_717912, EPI_ISL_717913, EPI_ISL_717914, EPI_ISL_717915, EPI_ISL_717916, EPI_ISL_717917, EPI_ISL_717918, EPI_ISL_717919	LACEN Dr. Francisco Rimolo Neto	Bioinformatics Laboratory / LNCC	Carolina M Voloch, Ronaldo da Silva F Jr, Luiz G P de Almeida, Cynthia C Cardoso, Otavio Bustrolini, Alexandra L Gerber, Ana Paula de C Guimarães, Diana Mariani, Andréa Cony Cavalcanti, Claudia dos Santos Rodrigues, Terezinha M P P Castiñeira, Amilcar Tanuri, Ana Tereza R de Vasconcelos
EPI_ISL_717920, EPI_ISL_717921, EPI_ISL_717922, EPI_ISL_717923, EPI_ISL_717924, EPI_ISL_717925, EPI_ISL_717926, EPI_ISL_717927, EPI_ISL_717928, EPI_ISL_717929, EPI_ISL_717930, EPI_ISL_717931, EPI_ISL_717932, EPI_ISL_717933, EPI_ISL_717934, EPI_ISL_717935, EPI_ISL_717936, EPI_ISL_717937, EPI_ISL_717938, EPI_ISL_717939, EPI_ISL_717940, EPI_ISL_717941, EPI_ISL_717942, EPI_ISL_717943, EPI_ISL_717944, EPI_ISL_717945, EPI_ISL_717946, EPI_ISL_717947, EPI_ISL_717948, EPI_ISL_717949, EPI_ISL_717950, EPI_ISL_717951, EPI_ISL_717952, EPI_ISL_717953, EPI_ISL_717954, EPI_ISL_717955, EPI_ISL_717956, EPI_ISL_717957			
see above	Laboratorio de Virologia Molecular / UFRJ	Bioinformatics Laboratory / LNCC	Carolina M Voloch, Ronaldo da Silva F Jr, Luiz G P de Almeida, Cynthia C Cardoso, Otavio Bustrolini, Alexandra L Gerber, Ana Paula de C Guimarães, Diana Mariani, Andréa Cony Cavalcanti, Claudia dos Santos Rodrigues, Terezinha M P P Castiñeira, Amilcar Tanuri, Ana Tereza R de Vasconcelos
EPI_ISL_717958	LACEN Dr. Francisco Rimolo Neto	Bioinformatics Laboratory / LNCC	Carolina M Voloch, Ronaldo da Silva F Jr, Luiz G P de Almeida, Cynthia C Cardoso, Otavio Bustrolini, Alexandra L Gerber, Ana Paula de C Guimarães, Diana Mariani, Andréa Cony Cavalcanti, Claudia dos Santos Rodrigues, Terezinha M P P Castiñeira, Amilcar Tanuri, Ana Tereza R de Vasconcelos
EPI_ISL_717959, EPI_ISL_717960, EPI_ISL_717961	Laboratorio de Virologia Molecular / UFRJ	Bioinformatics Laboratory / LNCC	Carolina M Voloch, Ronaldo da Silva F Jr, Luiz G P de Almeida, Cynthia C Cardoso, Otavio Bustrolini, Alexandra L Gerber, Ana Paula de C Guimarães, Diana Mariani, Andréa Cony Cavalcanti, Claudia dos Santos Rodrigues, Terezinha M P P Castiñeira, Amilcar Tanuri, Ana Tereza R de Vasconcelos
EPI_ISL_717962	LACEN RJ - Noel Nutels	Bioinformatics Laboratory / LNCC	Carolina M Voloch, Ronaldo da Silva F Jr, Luiz G P de Almeida, Cynthia C Cardoso, Otavio Bustrolini, Alexandra L Gerber, Ana Paula de C Guimarães, Diana Mariani, Andréa Cony Cavalcanti, Claudia dos Santos Rodrigues, Terezinha M P P Castiñeira, Amilcar Tanuri, Ana Tereza R de Vasconcelos
EPI_ISL_717963, EPI_ISL_717964	LACEN Dr. Francisco Rimolo Neto	Bioinformatics Laboratory / LNCC	Carolina M Voloch, Ronaldo da Silva F Jr, Luiz G P de Almeida, Cynthia C Cardoso, Otavio Bustrolini, Alexandra L Gerber, Ana Paula de C Guimarães, Diana Mariani, Andréa Cony Cavalcanti, Claudia dos Santos Rodrigues, Terezinha M P P Castiñeira, Amilcar Tanuri, Ana Tereza R de Vasconcelos
EPI_ISL_717965, EPI_ISL_717966, EPI_ISL_717967, EPI_ISL_717968, EPI_ISL_717969, EPI_ISL_717970, EPI_ISL_717971, EPI_ISL_717972, EPI_ISL_717973, EPI_ISL_717974	Molecular diagnostic laboratory of Federal Budget Institution of Science "Central Research Institute of Epidemiology" of The Federal Service on Customers' Rights Protection and Human Well-being Surveillance	Group of Genomics and Postgenomic Technologies of Central Research Institute of Epidemiology	Samoilov AE, Kaptelova VV, Dudorova AV, Korneenko EV, Speranskaya AS, Tivanova EV, Shipulina OY, Akimkin VG
EPI_ISL_717975	Institute of Virology, Biomedical Research Center of the Slovak Academy of Sciences, Bratislava	Faculty of Natural Sciences, Comenius University, Bratislava	Broa Brejová, Viktória Hodorová, Kristína Boršová, Viktória abanová, Dominika Friová, Sabina Fumaová Havlíková, Juraj Kopáek, Martina Liková, ubomíra Lukáiková, Martina Neboháová, Monika Sláviková, Edita Staroová, Elena Tichá, Tomáš Vina, Jozef Nosek, Boris Klempa
EPI_ISL_717978	Army Medical Center, Scientific Department, Virology Laboratory	Army Medical Center, Scientific Department, Virology Laboratory	Silvia Fillo, Giovanni Faggioni, Riccardo De Santis, Antonella Fortunato, Anna Anselmo, Vanessa Vera Fain, Francesco Giordani, Nino D'Amore, Anella Monte, Marzia Cavalli, Alessandra Amoroso, Stella Lia, Roberta Sorrentino, Rossella Tirelli, Federica Galeano, Annalisa Pelo, Margherita De Santis, Giulia Campoli, Andrea Ciammaruconi, Florigio Lista.
EPI_ISL_717980, EPI_ISL_717981	Lab voor klinische biologie	Onderzoeksgroep Virologie	Laurens Lambrechts, Nick Vereecke, Marthe Pauwels, Bruno Verhasselt, Linos Vandekerckhove, Hans Nauwynck, Sebastiaan Theuns
EPI_ISL_717982, EPI_ISL_717983	Lab voor klinische biologie	Onderzoeksgroep Virologie	Nick Vereecke, Laurens Lambrechts, Marthe Pauwels, Bruno Verhasselt, Linos Vandekerckhove, Hans Nauwynck, Sebastiaan Theuns
EPI_ISL_717991, EPI_ISL_717992, EPI_ISL_717993, EPI_ISL_717994, EPI_ISL_717995, EPI_ISL_717996, EPI_ISL_717997, EPI_ISL_717998, EPI_ISL_717999, EPI_ISL_718000	Lab voor klinische biologie	Onderzoeksgroep Virologie	Laurens Lambrechts, Nick Vereecke, Marthe Pauwels, Bruno Verhasselt, Linos Vandekerckhove, Hans Nauwynck, Sebastiaan Theuns
EPI_ISL_718001, EPI_ISL_718002, EPI_ISL_718003, EPI_ISL_718004, EPI_ISL_718005, EPI_ISL_718006, EPI_ISL_718007, EPI_ISL_718008	Lab voor klinische biologie	Onderzoeksgroep Virologie	Nick Vereecke, Laurens Lambrechts, Marthe Pauwels, Bruno Verhasselt, Linos Vandekerckhove, Hans Nauwynck, Sebastiaan Theuns
EPI_ISL_718009, EPI_ISL_718010, EPI_ISL_718011, EPI_ISL_718012, EPI_ISL_718013, EPI_ISL_718014, EPI_ISL_718015	Lab voor klinische biologie	Onderzoeksgroep Virologie	Laurens Lambrechts, Nick Vereecke, Marthe Pauwels, Bruno Verhasselt, Linos Vandekerckhove, Hans Nauwynck, Sebastiaan Theuns
EPI_ISL_718016, EPI_ISL_718017, EPI_ISL_718018, EPI_ISL_718019,	Lab voor klinische biologie	Onderzoeksgroep Virologie	Nick Vereecke, Laurens Lambrechts, Marthe Pauwels, Bruno Verhasselt, Linos Vandekerckhove, Hans Nauwynck, Sebastiaan Theuns

EPI_ISL_718020, EPI_ISL_718021, EPI_ISL_718022			
EPI_ISL_718023, EPI_ISL_718024	Lab voor klinische biologie	Onderzoeksgroep Virologie	Laurens Lambrechts, Nick Vereecke, Marthe Pauwels, Bruno Verhasselt, Linos Vandekerckhove, Hans Nauwynck, Sebastiaan Theuns
EPI_ISL_718025, EPI_ISL_718026	Lab voor klinische biologie	Onderzoeksgroep Virologie	Nick Vereecke, Laurens Lambrechts, Marthe Pauwels, Bruno Verhasselt, Linos Vandekerckhove, Hans Nauwynck, Sebastiaan Theuns
EPI_ISL_718027, EPI_ISL_718028, EPI_ISL_718029, EPI_ISL_718030, EPI_ISL_718031, EPI_ISL_718032, EPI_ISL_718033, EPI_ISL_718034, EPI_ISL_718035, EPI_ISL_718036, EPI_ISL_718037, EPI_ISL_718038, EPI_ISL_718039, EPI_ISL_718040, EPI_ISL_718041, EPI_ISL_718042, EPI_ISL_718043, EPI_ISL_718044, EPI_ISL_718045, EPI_ISL_718046, EPI_ISL_718047, EPI_ISL_718048, EPI_ISL_718049, EPI_ISL_718050, EPI_ISL_718051, EPI_ISL_718052, EPI_ISL_718054, EPI_ISL_718055, EPI_ISL_718056, EPI_ISL_718057, EPI_ISL_718058, EPI_ISL_718059, EPI_ISL_718060, EPI_ISL_718061, EPI_ISL_718062, EPI_ISL_718063, EPI_ISL_718064, EPI_ISL_718065, EPI_ISL_718066, EPI_ISL_718067, EPI_ISL_718068, EPI_ISL_718069, EPI_ISL_718070, EPI_ISL_718071, EPI_ISL_718072, EPI_ISL_718073, EPI_ISL_718074, EPI_ISL_718075, EPI_ISL_718076, EPI_ISL_718077, EPI_ISL_718078, EPI_ISL_718079, EPI_ISL_718080, EPI_ISL_718081, EPI_ISL_718082, EPI_ISL_718083, EPI_ISL_718084, EPI_ISL_718085, EPI_ISL_718086, EPI_ISL_718087, EPI_ISL_718088, EPI_ISL_718089, EPI_ISL_718090, EPI_ISL_718091, EPI_ISL_718092, EPI_ISL_718093, EPI_ISL_718094, EPI_ISL_718095, EPI_ISL_718096, EPI_ISL_718097, EPI_ISL_718098, EPI_ISL_718099, EPI_ISL_718100, EPI_ISL_718101, EPI_ISL_718103, EPI_ISL_718104			
see above	ZOTZ KLIMAS MVZ Düsseldorf-Centrum GbR ÜBAG für Labormedizin, Genetik, Zytologie, Pathologie	Center of Medical Microbiology, Virology, and Hospital Hygiene, University of Duesseldorf	Maximilian Damagnez, Alexander Dilthey, Ashley-Jane Duplessis, Patrick Finzer, Katrin Hoffmann, Torsten Houwaart, Lisanna Hülse, Malte Kohns Vasconcelos, Marek Korencak, Nadine Lübke, Jessica Nicolai, Klaus Pfeffer, Daniel Strelow, Jörg Timm, Andreas Walker, Tobias Wienemann, Rainer Zotz
EPI_ISL_718143, EPI_ISL_718146	Ministry of Health Hospitals	Institute of Health and Community Medicine	David Perera, Ooi Mong How, Chua Hock Hin, Tonnii Sia Loong Loong, Wong Jyn Shan, Wong Kiing Aik, Chan Chia Jui
EPI_ISL_718167	Borneo Medical Centre	Institute of Health and Community Medicine	David Perera, Ooi Mong How, Chua Hock Hin, Tonnii Sia Loong Loong, Wong Jyn Shan, Wong Kiing Aik, Chan Chia Jui
EPI_ISL_718168, EPI_ISL_718169, EPI_ISL_718170, EPI_ISL_718171, EPI_ISL_718172, EPI_ISL_718173	Ministry of Health Hospitals	Institute of Health and Community Medicine	David Perera, Ooi Mong How, Chua Hock Hin, Tonnii Sia Loong Loong, Wong Jyn Shan, Wong Kiing Aik, Chan Chia Jui
EPI_ISL_718174	Borneo Medical Centre	Institute of Health and Community Medicine	David Perera, Ooi Mong How, Chua Hock Hin, Tonnii Sia Loong Loong, Wong Jyn Shan, Wong Kiing Aik, Chan Chia Jui
EPI_ISL_718175	Ministry of Health Hospitals	Institute of Health and Community Medicine	David Perera, Ooi Mong How, Chua Hock Hin, Tonnii Sia Loong Loong, Wong Jyn Shan, Wong Kiing Aik, Chan Chia Jui
EPI_ISL_718176	Borneo Medical Centre	Institute of Health and Community Medicine	David Perera, Ooi Mong How, Chua Hock Hin, Tonnii Sia Loong Loong, Wong Jyn Shan, Wong Kiing Aik, Chan Chia Jui
EPI_ISL_718177	Ministry of Health Hospitals	Institute of Health and Community Medicine	David Perera, Ooi Mong How, Chua Hock Hin, Tonnii Sia Loong Loong, Wong Jyn Shan, Wong Kiing Aik, Chan Chia Jui
EPI_ISL_718178	Borneo Medical Centre	Institute of Health and Community Medicine	David Perera, Ooi Mong How, Chua Hock Hin, Tonnii Sia Loong Loong, Wong Jyn Shan, Wong Kiing Aik, Chan Chia Jui
EPI_ISL_718179, EPI_ISL_718180, EPI_ISL_718181	Ministry of Health Hospitals	Institute of Health and Community Medicine	David Perera, Ooi Mong How, Chua Hock Hin, Tonnii Sia Loong Loong, Wong Jyn Shan, Wong Kiing Aik, Chan Chia Jui
EPI_ISL_718182	Borneo Medical Centre	Institute of Health and Community Medicine	David Perera, Ooi Mong How, Chua Hock Hin, Tonnii Sia Loong Loong, Wong Jyn Shan, Wong Kiing Aik, Chan Chia Jui
EPI_ISL_718183, EPI_ISL_718184	Ministry of Health Hospitals	Institute of Health and Community Medicine	David Perera, Ooi Mong How, Chua Hock Hin, Tonnii Sia Loong Loong, Wong Jyn Shan, Wong Kiing Aik, Chan Chia Jui
EPI_ISL_718185	Borneo Medical Centre	Institute of Health and Community Medicine	David Perera, Ooi Mong How, Chua Hock Hin, Tonnii Sia Loong Loong, Wong Jyn Shan, Wong Kiing Aik, Chan Chia Jui
EPI_ISL_718186, EPI_ISL_718187, EPI_ISL_718188, EPI_ISL_718189, EPI_ISL_718190	Ministry of Health Hospitals	Institute of Health and Community Medicine	David Perera, Ooi Mong How, Chua Hock Hin, Tonnii Sia Loong Loong, Wong Jyn Shan, Wong Kiing Aik, Chan Chia Jui
EPI_ISL_718191, EPI_ISL_718192	Borneo Medical Centre	Institute of Health and Community Medicine	David Perera, Ooi Mong How, Chua Hock Hin, Tonnii Sia Loong Loong, Wong Jyn Shan, Wong Kiing Aik, Chan Chia Jui
EPI_ISL_718193, EPI_ISL_718194, EPI_ISL_718195, EPI_ISL_718196	Ministry of Health Hospitals	Institute of Health and Community Medicine	David Perera, Ooi Mong How, Chua Hock Hin, Tonnii Sia Loong Loong, Wong Jyn Shan, Wong Kiing Aik, Chan Chia Jui
EPI_ISL_718197, EPI_ISL_718198	Borneo Medical Centre	Institute of Health and Community Medicine	David Perera, Ooi Mong How, Chua Hock Hin, Tonnii Sia Loong Loong, Wong Jyn Shan, Wong Kiing Aik, Chan Chia Jui
EPI_ISL_718199, EPI_ISL_718200, EPI_ISL_718201, EPI_ISL_718202	Ministry of Health Hospitals	Institute of Health and Community Medicine	David Perera, Ooi Mong How, Chua Hock Hin, Tonnii Sia Loong Loong, Wong Jyn Shan, Wong Kiing Aik, Chan Chia Jui
EPI_ISL_718203, EPI_ISL_718204, EPI_ISL_718205	Borneo Medical Centre	Institute of Health and Community Medicine	David Perera, Ooi Mong How, Chua Hock Hin, Tonnii Sia Loong Loong, Wong Jyn Shan, Wong Kiing Aik, Chan Chia Jui
EPI_ISL_718206, EPI_ISL_718207, EPI_ISL_718208, EPI_ISL_718209, EPI_ISL_718210, EPI_ISL_718211, EPI_ISL_718212, EPI_ISL_718213, EPI_ISL_718214, EPI_ISL_718215	Ministry of Health Hospitals	Institute of Health and Community Medicine	David Perera, Ooi Mong How, Chua Hock Hin, Tonnii Sia Loong Loong, Wong Jyn Shan, Wong Kiing Aik, Chan Chia Jui
EPI_ISL_718216	Borneo Medical Centre	Institute of Health and Community Medicine	David Perera, Ooi Mong How, Chua Hock Hin, Tonnii Sia Loong Loong, Wong Jyn Shan, Wong Kiing Aik, Chan Chia Jui
EPI_ISL_718217	Ministry of Health Hospitals	Institute of Health and Community Medicine	David Perera, Ooi Mong How, Chua Hock Hin, Tonnii Sia Loong Loong, Wong Jyn Shan, Wong Kiing Aik, Chan Chia Jui
EPI_ISL_718218	Borneo Medical Centre	Institute of Health and Community Medicine	David Perera, Ooi Mong How, Chua Hock Hin, Tonnii Sia Loong Loong, Wong Jyn Shan, Wong Kiing Aik, Chan Chia Jui
EPI_ISL_718219, EPI_ISL_718220	Ministry of Health Hospitals	Institute of Health and Community Medicine	David Perera, Ooi Mong How, Chua Hock Hin, Tonnii Sia Loong Loong, Wong Jyn Shan, Wong Kiing Aik, Chan Chia Jui
EPI_ISL_718222	Borneo Medical Centre	Institute of Health and Community Medicine	David Perera, Ooi Mong How, Chua Hock Hin, Tonnii Sia Loong Loong, Wong Jyn Shan, Wong Kiing Aik, Chan Chia Jui
EPI_ISL_718226, EPI_ISL_718227, EPI_ISL_718228, EPI_ISL_718229, EPI_ISL_718230, EPI_ISL_718231, EPI_ISL_718232, EPI_ISL_718233, EPI_ISL_718234, EPI_ISL_718235, EPI_ISL_718236, EPI_ISL_718237, EPI_ISL_718238, EPI_ISL_718239, EPI_ISL_718240, EPI_ISL_718241, EPI_ISL_718242, EPI_ISL_718243, EPI_ISL_718244, EPI_ISL_718245, EPI_ISL_718246, EPI_ISL_718247, EPI_ISL_718248, EPI_ISL_718249			
see above	Hospital	National Reference Center for Viruses of Respiratory Infections, Institut Pasteur, Paris	Marion Barbet, Sylvie Behillil, Méline Bizard, Angela Brisebarre, Camille Capel, Etienne Simon-Lorière, Vincent Enouf, Maud Vanpeene, Sylvie van der Werf, Gisèle Lagathu
EPI_ISL_718250	Institute of Virology, Biomedical Research Center of the Slovak Academy of Sciences, Bratislava	Faculty of Natural Sciences, Comenius University, Bratislava	Kristína Boršová, Viktória Hodorová, Broa Brejová, Viktória abanová, Sabina Fumaová Havlíková, Juraj Kopáek, Martina Liková, ubomíra Lukáiková, Martina Neboháová, Monika Sláviková, Tomáš Vina, Boris Klempa, Jozef Nosek
EPI_ISL_718251	Institute of Virology, Biomedical Research Center of the Slovak Academy of Sciences, Bratislava	Faculty of Natural Sciences, Comenius University, Bratislava	Viktória Hodorová, Kristína Boršová, Broa Brejová, Viktória abanová, Sabina Fumaová Havlíková, Juraj Kopáek, Martina Liková, ubomíra Lukáiková, Martina Neboháová, Monika Sláviková, Tomáš Vina, Jozef Nosek, Boris Klempa
EPI_ISL_718252	Institute of Virology, Biomedical Research Center of the Slovak Academy of Sciences, Bratislava	Faculty of Natural Sciences, Comenius University, Bratislava	Kristína Boršová, Viktória Hodorová, Broa Brejová, Viktória abanová, Sabina Fumaová Havlíková, Juraj Kopáek, Martina Liková, ubomíra Lukáiková, Martina Neboháová, Monika Sláviková, Tomáš Vina, Boris Klempa, Jozef Nosek
EPI_ISL_718253	Institute of Virology, Biomedical Research Center of the Slovak Academy of Sciences, Bratislava	Faculty of Natural Sciences, Comenius University, Bratislava	Viktória Hodorová, Kristína Boršová, Broa Brejová, Viktória abanová, Sabina Fumaová Havlíková, Juraj Kopáek, Martina Liková, ubomíra Lukáiková, Martina Neboháová, Monika Sláviková, Tomáš Vina, Jozef Nosek, Boris Klempa
EPI_ISL_718254	Institute of Virology, Biomedical Research Center of the Slovak Academy of Sciences, Bratislava	Faculty of Natural Sciences, Comenius University, Bratislava	Kristína Boršová, Viktória Hodorová, Broa Brejová, Viktória abanová, Sabina Fumaová Havlíková, Juraj Kopáek, Martina Liková, ubomíra Lukáiková, Martina Neboháová, Monika Sláviková, Tomáš Vina, Boris Klempa, Jozef Nosek
EPI_ISL_718255, EPI_ISL_718256, EPI_ISL_718257	Institute of Virology, Biomedical Research Center of the Slovak Academy of Sciences, Bratislava	Faculty of Natural Sciences, Comenius University, Bratislava	Broa Brejová, Viktória Hodorová, Kristína Boršová, Viktória abanová, Sabina Fumaová Havlíková, Juraj Kopáek, Martina Liková, ubomíra Lukáiková, Martina Neboháová, Monika Sláviková, Tomáš Vina, Jozef Nosek, Boris Klempa
EPI_ISL_718258	Institute of Virology, Biomedical Research Center of the Slovak Academy of Sciences, Bratislava	Faculty of Natural Sciences, Comenius University, Bratislava	Viktória Hodorová, Kristína Boršová, Broa Brejová, Viktória abanová, Sabina Fumaová Havlíková, Juraj Kopáek, Martina Liková, ubomíra Lukáiková, Martina Neboháová, Monika Sláviková, Tomáš Vina, Jozef Nosek, Boris Klempa
EPI_ISL_718259	Institute of Virology, Biomedical Research Center of the Slovak Academy of Sciences, Bratislava	Faculty of Natural Sciences, Comenius University, Bratislava	Broa Brejová, Viktória Hodorová, Kristína Boršová, Viktória abanová, Sabina Fumaová Havlíková, Juraj Kopáek, Martina Liková, ubomíra Lukáiková, Martina Neboháová, Monika Sláviková, Tomáš Vina, Jozef Nosek, Boris Klempa
EPI_ISL_718260	Institute of Virology, Biomedical Research Center of the	Faculty of Natural Sciences, Comenius University, Bratislava	Viktória Hodorová, Kristína Boršová, Broa Brejová, Viktória abanová, Sabina Fumaová Havlíková, Juraj Kopáek, Martina Liková, ubomíra Lukáiková,

	Slovak Academy of Sciences, Bratislava		Martina Neboháová, Monika Sláviková, Tomáš Vina, Jozef Nosek, Boris Klempa
EPI_ISL_718262	National Institute for Infectious Diseases, INMI, "L. Spallanzani" IRCCS	National Institute for Infectious Diseases, INMI, "L. Spallanzani" IRCCS	C.E.M Gruber, F Messina, M Rueca, B Bartolini, E Giombini, MR Capobianchi, A Di Caro
EPI_ISL_718263	National Institute for Infectious Diseases, INMI, "L. Spallanzani" IRCCS	National Institute for Infectious Diseases, INMI, "L. Spallanzani" IRCCS	E Giombini, C.E.M Gruber, M Rueca, B Bartolini, F Messina, A Di Caro, MR Capobianchi
EPI_ISL_718264	National Institute for Infectious Diseases, INMI, "L. Spallanzani" IRCCS	National Institute for Infectious Diseases, INMI, "L. Spallanzani" IRCCS	F Messina, M Rueca, B Bartolini, C.E.M Gruber, E Giombini, MR Capobianchi, A Di Caro
EPI_ISL_718265	National Institute for Infectious Diseases, INMI, "L. Spallanzani" IRCCS	National Institute for Infectious Diseases, INMI, "L. Spallanzani" IRCCS	B Bartolini, M Rueca, C.E.M Gruber, F Messina, E Giombini, A Di Caro, MR Capobianchi
EPI_ISL_718267, EPI_ISL_718268, EPI_ISL_718269, EPI_ISL_718270, EPI_ISL_718271, EPI_ISL_718272, EPI_ISL_718274, EPI_ISL_718275, EPI_ISL_718276, EPI_ISL_718277, EPI_ISL_718278, EPI_ISL_718279, EPI_ISL_718280, EPI_ISL_718281, EPI_ISL_718282, EPI_ISL_718283, EPI_ISL_718284, EPI_ISL_718285, EPI_ISL_718286, EPI_ISL_718287, EPI_ISL_718288, EPI_ISL_718289, EPI_ISL_718290, EPI_ISL_718291, EPI_ISL_718292, EPI_ISL_718293, EPI_ISL_718294, EPI_ISL_718295, EPI_ISL_718296, EPI_ISL_718297, EPI_ISL_718298, EPI_ISL_718299, EPI_ISL_718300, EPI_ISL_718301, EPI_ISL_718302, EPI_ISL_718303, EPI_ISL_718304, EPI_ISL_718305, EPI_ISL_718306, EPI_ISL_718307, EPI_ISL_718308, EPI_ISL_718309, EPI_ISL_718310, EPI_ISL_718311, EPI_ISL_718312, EPI_ISL_718313, EPI_ISL_718314, EPI_ISL_718315			
see above	Institute for Medical Research, Infectious Disease Research Centre, National Institutes of Health, Ministry of Health Malaysia	Institute for Medical Research, Infectious Disease Research Centre, National Institutes of Health, Ministry of Health Malaysia	Suppiah J, Kamel K, Mohd-Zawawi Z, Thayan R
EPI_ISL_718318, EPI_ISL_718319, EPI_ISL_718320, EPI_ISL_718321, EPI_ISL_718322, EPI_ISL_718323, EPI_ISL_718324, EPI_ISL_718325, EPI_ISL_718326, EPI_ISL_718327, EPI_ISL_718328, EPI_ISL_718329, EPI_ISL_718330, EPI_ISL_718331, EPI_ISL_718332, EPI_ISL_718333, EPI_ISL_718334, EPI_ISL_718335, EPI_ISL_718336, EPI_ISL_718338, EPI_ISL_718339, EPI_ISL_718340, EPI_ISL_718341, EPI_ISL_718342, EPI_ISL_718343, EPI_ISL_718344, EPI_ISL_718345, EPI_ISL_718346, EPI_ISL_718347, EPI_ISL_718348, EPI_ISL_718349, EPI_ISL_718350, EPI_ISL_718351, EPI_ISL_718352, EPI_ISL_718353, EPI_ISL_718354, EPI_ISL_718355, EPI_ISL_718356, EPI_ISL_718357, EPI_ISL_718358, EPI_ISL_718359, EPI_ISL_718360, EPI_ISL_718361, EPI_ISL_718362, EPI_ISL_718363, EPI_ISL_718364, EPI_ISL_718365, EPI_ISL_718366, EPI_ISL_718367, EPI_ISL_718368, EPI_ISL_718369, EPI_ISL_718370, EPI_ISL_718371, EPI_ISL_718372, EPI_ISL_718373, EPI_ISL_718374, EPI_ISL_718375, EPI_ISL_718376, EPI_ISL_718377, EPI_ISL_718378, EPI_ISL_718379, EPI_ISL_718380, EPI_ISL_718381, EPI_ISL_718382, EPI_ISL_718383, EPI_ISL_718384, EPI_ISL_718385, EPI_ISL_718386, EPI_ISL_718387, EPI_ISL_718388, EPI_ISL_718389, EPI_ISL_718390, EPI_ISL_718391, EPI_ISL_718392, EPI_ISL_718393, EPI_ISL_718394, EPI_ISL_718395, EPI_ISL_718396, EPI_ISL_718397, EPI_ISL_718398, EPI_ISL_718399, EPI_ISL_718400, EPI_ISL_718401, EPI_ISL_718402, EPI_ISL_718403, EPI_ISL_718404, EPI_ISL_718405, EPI_ISL_718406, EPI_ISL_718407, EPI_ISL_718408, EPI_ISL_718409, EPI_ISL_718410, EPI_ISL_718411, EPI_ISL_718412, EPI_ISL_718413, EPI_ISL_718414, EPI_ISL_718415, EPI_ISL_718416, EPI_ISL_718417, EPI_ISL_718418, EPI_ISL_718419, EPI_ISL_718420, EPI_ISL_718421, EPI_ISL_718422, EPI_ISL_718423, EPI_ISL_718424, EPI_ISL_718425, EPI_ISL_718426, EPI_ISL_718427, EPI_ISL_718428, EPI_ISL_718429, EPI_ISL_718430, EPI_ISL_718431, EPI_ISL_718432, EPI_ISL_718433, EPI_ISL_718434, EPI_ISL_718435, EPI_ISL_718436, EPI_ISL_718437, EPI_ISL_718438, EPI_ISL_718439, EPI_ISL_718440, EPI_ISL_718441, EPI_ISL_718442, EPI_ISL_718443, EPI_ISL_718444, EPI_ISL_718445, EPI_ISL_718446, EPI_ISL_718447, EPI_ISL_718448, EPI_ISL_718449, EPI_ISL_718450, EPI_ISL_718451, EPI_ISL_718452, EPI_ISL_718453, EPI_ISL_718454, EPI_ISL_718455, EPI_ISL_718456, EPI_ISL_718457, EPI_ISL_718458, EPI_ISL_718459, EPI_ISL_718460, EPI_ISL_718461, EPI_ISL_718462, EPI_ISL_718463, EPI_ISL_718464, EPI_ISL_718465, EPI_ISL_718466, EPI_ISL_718467, EPI_ISL_718468, EPI_ISL_718469, EPI_ISL_718470, EPI_ISL_718471, EPI_ISL_718472, EPI_ISL_718473, EPI_ISL_718474, EPI_ISL_718475, EPI_ISL_718476, EPI_ISL_718477, EPI_ISL_718478, EPI_ISL_718479, EPI_ISL_718480, EPI_ISL_718481, EPI_ISL_718482, EPI_ISL_718483, EPI_ISL_718484, EPI_ISL_718485, EPI_ISL_718486, EPI_ISL_718487, EPI_ISL_718488, EPI_ISL_718489, EPI_ISL_718490, EPI_ISL_718491, EPI_ISL_718492, EPI_ISL_718493, EPI_ISL_718494, EPI_ISL_718495, EPI_ISL_718496, EPI_ISL_718497, EPI_ISL_718498, EPI_ISL_718499, EPI_ISL_718500, EPI_ISL_718501, EPI_ISL_718502, EPI_ISL_718503, EPI_ISL_718504, EPI_ISL_718505, EPI_ISL_718506, EPI_ISL_718507, EPI_ISL_718508, EPI_ISL_718509, EPI_ISL_718510, EPI_ISL_718511, EPI_ISL_718512, EPI_ISL_718513, EPI_ISL_718514, EPI_ISL_718515, EPI_ISL_718516, EPI_ISL_718517, EPI_ISL_718518, EPI_ISL_718519, EPI_ISL_718520, EPI_ISL_718521, EPI_ISL_718522, EPI_ISL_718523, EPI_ISL_718524, EPI_ISL_718525, EPI_ISL_718526, EPI_ISL_718527, EPI_ISL_718528, EPI_ISL_718529, EPI_ISL_718530, EPI_ISL_718531, EPI_ISL_718532, EPI_ISL_718533, EPI_ISL_718534, EPI_ISL_718535, EPI_ISL_718536, EPI_ISL_718537, EPI_ISL_718538, EPI_ISL_718539, EPI_ISL_718540, EPI_ISL_718541, EPI_ISL_718542, EPI_ISL_718543, EPI_ISL_718544, EPI_ISL_718545, EPI_ISL_718546, EPI_ISL_718547, EPI_ISL_718548, EPI_ISL_718549, EPI_ISL_718550, EPI_ISL_718551, EPI_ISL_718552, EPI_ISL_718553, EPI_ISL_718554, EPI_ISL_718555, EPI_ISL_718556, EPI_ISL_718557, EPI_ISL_718558, EPI_ISL_718559, EPI_ISL_718560, EPI_ISL_718561, EPI_ISL_718562, EPI_ISL_718563, EPI_ISL_718564, EPI_ISL_718565, EPI_ISL_718566, EPI_ISL_718567, EPI_ISL_718568, EPI_ISL_718569, EPI_ISL_718570, EPI_ISL_718571, EPI_ISL_718572, EPI_ISL_718573, EPI_ISL_718574, EPI_ISL_718575, EPI_ISL_718576, EPI_ISL_718577, EPI_ISL_718578, EPI_ISL_718579, EPI_ISL_718580, EPI_ISL_718581, EPI_ISL_718582, EPI_ISL_718583, EPI_ISL_718584, EPI_ISL_718585, EPI_ISL_718586, EPI_ISL_718587, EPI_ISL_718588, EPI_ISL_718589, EPI_ISL_718590, EPI_ISL_718591, EPI_ISL_718592, EPI_ISL_718593, EPI_ISL_718594, EPI_ISL_718595, EPI_ISL_718596, EPI_ISL_718597, EPI_ISL_718598, EPI_ISL_718599, EPI_ISL_718600, EPI_ISL_718601, EPI_ISL_718602, EPI_ISL_718603, EPI_ISL_718604, EPI_ISL_718605, EPI_ISL_718606, EPI_ISL_718607, EPI_ISL_718608, EPI_ISL_718609, EPI_ISL_718610, EPI_ISL_718611, EPI_ISL_718612, EPI_ISL_718613, EPI_ISL_718614, EPI_ISL_718615, EPI_ISL_718616, EPI_ISL_718617, EPI_ISL_718619, EPI_ISL_718620, EPI_ISL_718621, EPI_ISL_718622, EPI_ISL_718623, EPI_ISL_718624, EPI_ISL_718625, EPI_ISL_718626, EPI_ISL_718627, EPI_ISL_718628			
see above	Lighthouse Lab in Cambridge	Wellcome Sanger Institute for the COVID-19 Genomics UK (COG-UK) Consortium	Rob Howes, The Lighthouse Lab in Cambridge and Alex Alderton, Roberto Amato, Sonia Goncalves, Ewan Harrison, David K. Jackson, Ian Johnston, Dominic Kwiatkowski, Cordelia Langford, John Sillitoe on behalf of the Wellcome Sanger Institute COVID-19 Surveillance Team
EPI_ISL_718629	Lighthouse Lab in Alderley Park	Wellcome Sanger Institute for the COVID-19 Genomics UK (COG-UK) Consortium	Jacquelyn Wynn, Mairead Hyland, The Lighthouse Lab in Alderley Park and Alex Alderton, Roberto Amato, Sonia Goncalves, Ewan Harrison, David K. Jackson, Ian Johnston, Dominic Kwiatkowski, Cordelia Langford, John Sillitoe on behalf of the Wellcome Sanger Institute COVID-19 Surveillance Team
EPI_ISL_718630	Lighthouse Lab in Cambridge	Wellcome Sanger Institute for the COVID-19 Genomics UK (COG-UK) Consortium	Rob Howes, The Lighthouse Lab in Cambridge and Alex Alderton, Roberto Amato, Sonia Goncalves, Ewan Harrison, David K. Jackson, Ian Johnston, Dominic Kwiatkowski, Cordelia Langford, John Sillitoe on behalf of the Wellcome Sanger Institute COVID-19 Surveillance Team
EPI_ISL_718631	Lighthouse Lab in Alderley Park	Wellcome Sanger Institute for the COVID-19 Genomics UK (COG-UK) Consortium	Jacquelyn Wynn, Mairead Hyland, The Lighthouse Lab in Alderley Park and Alex Alderton, Roberto Amato, Sonia Goncalves, Ewan Harrison, David K. Jackson, Ian Johnston, Dominic Kwiatkowski, Cordelia Langford, John Sillitoe on behalf of the Wellcome Sanger Institute COVID-19 Surveillance Team
EPI_ISL_718632	Lighthouse Lab in Cambridge	Wellcome Sanger Institute for the COVID-19 Genomics UK (COG-UK) Consortium	Rob Howes, The Lighthouse Lab in Cambridge and Alex Alderton, Roberto Amato, Sonia Goncalves, Ewan Harrison, David K. Jackson, Ian Johnston, Dominic Kwiatkowski, Cordelia Langford, John Sillitoe on behalf of the Wellcome Sanger Institute COVID-19 Surveillance Team
EPI_ISL_718633	Lighthouse Lab in Alderley Park	Wellcome Sanger Institute for the COVID-19 Genomics UK (COG-UK) Consortium	Jacquelyn Wynn, Mairead Hyland, The Lighthouse Lab in Alderley Park and Alex Alderton, Roberto Amato, Sonia Goncalves, Ewan Harrison, David K. Jackson, Ian Johnston, Dominic Kwiatkowski, Cordelia Langford, John Sillitoe on behalf of the Wellcome Sanger Institute COVID-19 Surveillance Team
EPI_ISL_718634	Lighthouse Lab in Cambridge	Wellcome Sanger Institute for the COVID-19 Genomics UK (COG-UK) Consortium	Rob Howes, The Lighthouse Lab in Cambridge and Alex Alderton, Roberto Amato, Sonia Goncalves, Ewan Harrison, David K. Jackson, Ian Johnston, Dominic Kwiatkowski, Cordelia Langford, John Sillitoe on behalf of the Wellcome Sanger Institute COVID-19 Surveillance Team
EPI_ISL_718635	Lighthouse Lab in Alderley Park	Wellcome Sanger Institute for the COVID-19 Genomics UK (COG-UK) Consortium	Jacquelyn Wynn, Mairead Hyland, The Lighthouse Lab in Alderley Park and Alex Alderton, Roberto Amato, Sonia Goncalves, Ewan Harrison, David K. Jackson, Ian Johnston, Dominic Kwiatkowski, Cordelia Langford, John Sillitoe on behalf of the Wellcome Sanger Institute COVID-19 Surveillance Team
EPI_ISL_718636	Lighthouse Lab in Cambridge	Wellcome Sanger Institute for the COVID-19 Genomics UK (COG-UK) Consortium	Rob Howes, The Lighthouse Lab in Cambridge and Alex Alderton, Roberto Amato, Sonia Goncalves, Ewan Harrison, David K. Jackson, Ian Johnston, Dominic Kwiatkowski, Cordelia Langford, John Sillitoe on behalf of the Wellcome Sanger Institute COVID-19 Surveillance Team
EPI_ISL_718637	Lighthouse Lab in Alderley Park	Wellcome Sanger Institute for the COVID-19 Genomics UK (COG-UK) Consortium	Jacquelyn Wynn, Mairead Hyland, The Lighthouse Lab in Alderley Park and Alex Alderton, Roberto Amato, Sonia Goncalves, Ewan Harrison, David K. Jackson, Ian Johnston, Dominic Kwiatkowski, Cordelia Langford, John Sillitoe on behalf of the Wellcome Sanger Institute COVID-19 Surveillance Team
EPI_ISL_718638	Lighthouse Lab in Cambridge	Wellcome Sanger Institute for the COVID-19 Genomics UK (COG-UK) Consortium	Rob Howes, The Lighthouse Lab in Cambridge and Alex Alderton, Roberto Amato, Sonia Goncalves, Ewan Harrison, David K. Jackson, Ian Johnston, Dominic Kwiatkowski, Cordelia Langford, John Sillitoe on behalf of the Wellcome Sanger Institute COVID-19 Surveillance Team
EPI_ISL_718639, EPI_ISL_718640, EPI_ISL_718641	Lighthouse Lab in Alderley Park	Wellcome Sanger Institute for the COVID-19 Genomics UK (COG-UK) Consortium	Jacquelyn Wynn, Mairead Hyland, The Lighthouse Lab in Alderley Park and Alex Alderton, Roberto Amato, Sonia Goncalves, Ewan Harrison, David K. Jackson, Ian Johnston, Dominic Kwiatkowski, Cordelia Langford, John Sillitoe on behalf of the Wellcome Sanger Institute COVID-19 Surveillance Team
EPI_ISL_718642	Lighthouse Lab in Cambridge	Wellcome Sanger Institute for the COVID-19 Genomics UK (COG-UK) Consortium	Rob Howes, The Lighthouse Lab in Cambridge and Alex Alderton, Roberto Amato, Sonia Goncalves, Ewan Harrison, David K. Jackson, Ian Johnston, Dominic Kwiatkowski, Cordelia Langford, John Sillitoe on behalf of the Wellcome Sanger Institute COVID-19 Surveillance Team
EPI_ISL_718643, EPI_ISL_718644	Lighthouse Lab in Alderley Park	Wellcome Sanger Institute for the COVID-19 Genomics UK (COG-UK) Consortium	Jacquelyn Wynn, Mairead Hyland, The Lighthouse Lab in Alderley Park and Alex Alderton, Roberto Amato, Sonia Goncalves, Ewan Harrison, David K. Jackson, Ian Johnston, Dominic Kwiatkowski, Cordelia Langford, John Sillitoe on behalf of the Wellcome Sanger Institute COVID-19 Surveillance Team
EPI_ISL_718645, EPI_ISL_718646	Lighthouse Lab in Cambridge	Wellcome Sanger Institute for the COVID-19 Genomics UK (COG-UK) Consortium	Rob Howes, The Lighthouse Lab in Cambridge and Alex Alderton, Roberto Amato, Sonia Goncalves, Ewan Harrison, David K. Jackson, Ian Johnston, Dominic Kwiatkowski, Cordelia Langford, John Sillitoe on behalf of the Wellcome Sanger Institute COVID-19 Surveillance Team
EPI_ISL_718647	Lighthouse Lab in Alderley Park	Wellcome Sanger Institute for the COVID-19 Genomics UK (COG-UK) Consortium	Jacquelyn Wynn, Mairead Hyland, The Lighthouse Lab in Alderley Park and Alex Alderton, Roberto Amato, Sonia Goncalves, Ewan Harrison, David K. Jackson, Ian Johnston, Dominic Kwiatkowski, Cordelia Langford, John Sillitoe on behalf of the Wellcome Sanger Institute COVID-19 Surveillance Team
EPI_ISL_718648, EPI_ISL_718649	Lighthouse Lab in Cambridge	Wellcome Sanger Institute for the COVID-19 Genomics UK (COG-UK) Consortium	Rob Howes, The Lighthouse Lab in Cambridge and Alex Alderton, Roberto Amato, Sonia Goncalves, Ewan Harrison, David K. Jackson, Ian Johnston, Dominic Kwiatkowski, Cordelia Langford, John Sillitoe on behalf of the Wellcome Sanger Institute COVID-19 Surveillance Team
EPI_ISL_718650	Lighthouse Lab in Alderley Park	Wellcome Sanger Institute for the COVID-19 Genomics UK (COG-UK) Consortium	Jacquelyn Wynn, Mairead Hyland, The Lighthouse Lab in Alderley Park and Alex Alderton, Roberto Amato, Sonia Goncalves, Ewan Harrison, David K. Jackson, Ian Johnston, Dominic Kwiatkowski, Cordelia Langford, John Sillitoe on behalf of the Wellcome Sanger Institute COVID-19 Surveillance Team
EPI_ISL_718651, EPI_ISL_718652	Lighthouse Lab in Cambridge	Wellcome Sanger Institute for the COVID-19 Genomics UK (COG-UK) Consortium	Rob Howes, The Lighthouse Lab in Cambridge and Alex Alderton, Roberto Amato, Sonia Goncalves, Ewan Harrison, David K. Jackson, Ian Johnston, Dominic Kwiatkowski, Cordelia Langford, John Sillitoe on behalf of the Wellcome Sanger Institute COVID-19 Surveillance Team
EPI_ISL_718653	Lighthouse Lab in Alderley Park	Wellcome Sanger Institute for the COVID-19 Genomics UK (COG-UK) Consortium	Jacquelyn Wynn, Mairead Hyland, The Lighthouse Lab in Alderley Park and Alex Alderton, Roberto Amato, Sonia Goncalves, Ewan Harrison, David K. Jackson, Ian Johnston, Dominic Kwiatkowski, Cordelia Langford, John Sillitoe on behalf of the Wellcome Sanger Institute COVID-19 Surveillance Team

EPI_ISL_718654, EPI_ISL_718655, EPI_ISL_718656	Lighthouse Lab in Cambridge	Wellcome Sanger Institute for the COVID-19 Genomics UK (COG-UK) Consortium	Rob Howes, The Lighthouse Lab in Cambridge and Alex Alderton, Roberto Amato, Sonia Goncalves, Ewan Harrison, David K. Jackson, Ian Johnston, Dominic Kwiatkowski, Cordelia Langford, John Sillitoe on behalf of the Wellcome Sanger Institute COVID-19 Surveillance Team
EPI_ISL_718657, EPI_ISL_718658	Lighthouse Lab in Alderley Park	Wellcome Sanger Institute for the COVID-19 Genomics UK (COG-UK) Consortium	Jacquelyn Wynn, Mairead Hyland, The Lighthouse Lab in Alderley Park and Alex Alderton, Roberto Amato, Sonia Goncalves, Ewan Harrison, David K. Jackson, Ian Johnston, Dominic Kwiatkowski, Cordelia Langford, John Sillitoe on behalf of the Wellcome Sanger Institute COVID-19 Surveillance Team
EPI_ISL_718659, EPI_ISL_718660, EPI_ISL_718661	Lighthouse Lab in Cambridge	Wellcome Sanger Institute for the COVID-19 Genomics UK (COG-UK) Consortium	Rob Howes, The Lighthouse Lab in Cambridge and Alex Alderton, Roberto Amato, Sonia Goncalves, Ewan Harrison, David K. Jackson, Ian Johnston, Dominic Kwiatkowski, Cordelia Langford, John Sillitoe on behalf of the Wellcome Sanger Institute COVID-19 Surveillance Team
EPI_ISL_718662, EPI_ISL_718663, EPI_ISL_718664, EPI_ISL_718665, EPI_ISL_718666	Lighthouse Lab in Alderley Park	Wellcome Sanger Institute for the COVID-19 Genomics UK (COG-UK) Consortium	Jacquelyn Wynn, Mairead Hyland, The Lighthouse Lab in Alderley Park and Alex Alderton, Roberto Amato, Sonia Goncalves, Ewan Harrison, David K. Jackson, Ian Johnston, Dominic Kwiatkowski, Cordelia Langford, John Sillitoe on behalf of the Wellcome Sanger Institute COVID-19 Surveillance Team
EPI_ISL_718667, EPI_ISL_718668, EPI_ISL_718669	Lighthouse Lab in Cambridge	Wellcome Sanger Institute for the COVID-19 Genomics UK (COG-UK) Consortium	Rob Howes, The Lighthouse Lab in Cambridge and Alex Alderton, Roberto Amato, Sonia Goncalves, Ewan Harrison, David K. Jackson, Ian Johnston, Dominic Kwiatkowski, Cordelia Langford, John Sillitoe on behalf of the Wellcome Sanger Institute COVID-19 Surveillance Team
EPI_ISL_718670	Lighthouse Lab in Alderley Park	Wellcome Sanger Institute for the COVID-19 Genomics UK (COG-UK) Consortium	Jacquelyn Wynn, Mairead Hyland, The Lighthouse Lab in Alderley Park and Alex Alderton, Roberto Amato, Sonia Goncalves, Ewan Harrison, David K. Jackson, Ian Johnston, Dominic Kwiatkowski, Cordelia Langford, John Sillitoe on behalf of the Wellcome Sanger Institute COVID-19 Surveillance Team
EPI_ISL_718671	Lighthouse Lab in Cambridge	Wellcome Sanger Institute for the COVID-19 Genomics UK (COG-UK) Consortium	Rob Howes, The Lighthouse Lab in Cambridge and Alex Alderton, Roberto Amato, Sonia Goncalves, Ewan Harrison, David K. Jackson, Ian Johnston, Dominic Kwiatkowski, Cordelia Langford, John Sillitoe on behalf of the Wellcome Sanger Institute COVID-19 Surveillance Team
EPI_ISL_718672	Lighthouse Lab in Alderley Park	Wellcome Sanger Institute for the COVID-19 Genomics UK (COG-UK) Consortium	Jacquelyn Wynn, Mairead Hyland, The Lighthouse Lab in Alderley Park and Alex Alderton, Roberto Amato, Sonia Goncalves, Ewan Harrison, David K. Jackson, Ian Johnston, Dominic Kwiatkowski, Cordelia Langford, John Sillitoe on behalf of the Wellcome Sanger Institute COVID-19 Surveillance Team
EPI_ISL_718673	Lighthouse Lab in Cambridge	Wellcome Sanger Institute for the COVID-19 Genomics UK (COG-UK) Consortium	Rob Howes, The Lighthouse Lab in Cambridge and Alex Alderton, Roberto Amato, Sonia Goncalves, Ewan Harrison, David K. Jackson, Ian Johnston, Dominic Kwiatkowski, Cordelia Langford, John Sillitoe on behalf of the Wellcome Sanger Institute COVID-19 Surveillance Team
EPI_ISL_718674, EPI_ISL_718675, EPI_ISL_718676	Lighthouse Lab in Alderley Park	Wellcome Sanger Institute for the COVID-19 Genomics UK (COG-UK) Consortium	Jacquelyn Wynn, Mairead Hyland, The Lighthouse Lab in Alderley Park and Alex Alderton, Roberto Amato, Sonia Goncalves, Ewan Harrison, David K. Jackson, Ian Johnston, Dominic Kwiatkowski, Cordelia Langford, John Sillitoe on behalf of the Wellcome Sanger Institute COVID-19 Surveillance Team
EPI_ISL_718677	Lighthouse Lab in Cambridge	Wellcome Sanger Institute for the COVID-19 Genomics UK (COG-UK) Consortium	Rob Howes, The Lighthouse Lab in Cambridge and Alex Alderton, Roberto Amato, Sonia Goncalves, Ewan Harrison, David K. Jackson, Ian Johnston, Dominic Kwiatkowski, Cordelia Langford, John Sillitoe on behalf of the Wellcome Sanger Institute COVID-19 Surveillance Team
EPI_ISL_718678	Lighthouse Lab in Alderley Park	Wellcome Sanger Institute for the COVID-19 Genomics UK (COG-UK) Consortium	Jacquelyn Wynn, Mairead Hyland, The Lighthouse Lab in Alderley Park and Alex Alderton, Roberto Amato, Sonia Goncalves, Ewan Harrison, David K. Jackson, Ian Johnston, Dominic Kwiatkowski, Cordelia Langford, John Sillitoe on behalf of the Wellcome Sanger Institute COVID-19 Surveillance Team
EPI_ISL_718679, EPI_ISL_718680, EPI_ISL_718681	Lighthouse Lab in Cambridge	Wellcome Sanger Institute for the COVID-19 Genomics UK (COG-UK) Consortium	Rob Howes, The Lighthouse Lab in Cambridge and Alex Alderton, Roberto Amato, Sonia Goncalves, Ewan Harrison, David K. Jackson, Ian Johnston, Dominic Kwiatkowski, Cordelia Langford, John Sillitoe on behalf of the Wellcome Sanger Institute COVID-19 Surveillance Team
EPI_ISL_718682	Lighthouse Lab in Alderley Park	Wellcome Sanger Institute for the COVID-19 Genomics UK (COG-UK) Consortium	Jacquelyn Wynn, Mairead Hyland, The Lighthouse Lab in Alderley Park and Alex Alderton, Roberto Amato, Sonia Goncalves, Ewan Harrison, David K. Jackson, Ian Johnston, Dominic Kwiatkowski, Cordelia Langford, John Sillitoe on behalf of the Wellcome Sanger Institute COVID-19 Surveillance Team
EPI_ISL_718683, EPI_ISL_718684, EPI_ISL_718685	Lighthouse Lab in Cambridge	Wellcome Sanger Institute for the COVID-19 Genomics UK (COG-UK) Consortium	Rob Howes, The Lighthouse Lab in Cambridge and Alex Alderton, Roberto Amato, Sonia Goncalves, Ewan Harrison, David K. Jackson, Ian Johnston, Dominic Kwiatkowski, Cordelia Langford, John Sillitoe on behalf of the Wellcome Sanger Institute COVID-19 Surveillance Team
EPI_ISL_718686, EPI_ISL_718687	Lighthouse Lab in Alderley Park	Wellcome Sanger Institute for the COVID-19 Genomics UK (COG-UK) Consortium	Jacquelyn Wynn, Mairead Hyland, The Lighthouse Lab in Alderley Park and Alex Alderton, Roberto Amato, Sonia Goncalves, Ewan Harrison, David K. Jackson, Ian Johnston, Dominic Kwiatkowski, Cordelia Langford, John Sillitoe on behalf of the Wellcome Sanger Institute COVID-19 Surveillance Team
EPI_ISL_718688, EPI_ISL_718689, EPI_ISL_718690	Lighthouse Lab in Cambridge	Wellcome Sanger Institute for the COVID-19 Genomics UK (COG-UK) Consortium	Rob Howes, The Lighthouse Lab in Cambridge and Alex Alderton, Roberto Amato, Sonia Goncalves, Ewan Harrison, David K. Jackson, Ian Johnston, Dominic Kwiatkowski, Cordelia Langford, John Sillitoe on behalf of the Wellcome Sanger Institute COVID-19 Surveillance Team
EPI_ISL_718691	Lighthouse Lab in Alderley Park	Wellcome Sanger Institute for the COVID-19 Genomics UK (COG-UK) Consortium	Jacquelyn Wynn, Mairead Hyland, The Lighthouse Lab in Alderley Park and Alex Alderton, Roberto Amato, Sonia Goncalves, Ewan Harrison, David K. Jackson, Ian Johnston, Dominic Kwiatkowski, Cordelia Langford, John Sillitoe on behalf of the Wellcome Sanger Institute COVID-19 Surveillance Team
EPI_ISL_718692, EPI_ISL_718693	Lighthouse Lab in Cambridge	Wellcome Sanger Institute for the COVID-19 Genomics UK (COG-UK) Consortium	Rob Howes, The Lighthouse Lab in Cambridge and Alex Alderton, Roberto Amato, Sonia Goncalves, Ewan Harrison, David K. Jackson, Ian Johnston, Dominic Kwiatkowski, Cordelia Langford, John Sillitoe on behalf of the Wellcome Sanger Institute COVID-19 Surveillance Team
EPI_ISL_718694, EPI_ISL_718695	Lighthouse Lab in Alderley Park	Wellcome Sanger Institute for the COVID-19 Genomics UK (COG-UK) Consortium	Jacquelyn Wynn, Mairead Hyland, The Lighthouse Lab in Alderley Park and Alex Alderton, Roberto Amato, Sonia Goncalves, Ewan Harrison, David K. Jackson, Ian Johnston, Dominic Kwiatkowski, Cordelia Langford, John Sillitoe on behalf of the Wellcome Sanger Institute COVID-19 Surveillance Team
EPI_ISL_718696, EPI_ISL_718697, EPI_ISL_718698, EPI_ISL_718699	Lighthouse Lab in Cambridge	Wellcome Sanger Institute for the COVID-19 Genomics UK (COG-UK) Consortium	Rob Howes, The Lighthouse Lab in Cambridge and Alex Alderton, Roberto Amato, Sonia Goncalves, Ewan Harrison, David K. Jackson, Ian Johnston, Dominic Kwiatkowski, Cordelia Langford, John Sillitoe on behalf of the Wellcome Sanger Institute COVID-19 Surveillance Team
EPI_ISL_718700	Lighthouse Lab in Alderley Park	Wellcome Sanger Institute for the COVID-19 Genomics UK (COG-UK) Consortium	Jacquelyn Wynn, Mairead Hyland, The Lighthouse Lab in Alderley Park and Alex Alderton, Roberto Amato, Sonia Goncalves, Ewan Harrison, David K. Jackson, Ian Johnston, Dominic Kwiatkowski, Cordelia Langford, John Sillitoe on behalf of the Wellcome Sanger Institute COVID-19 Surveillance Team
EPI_ISL_718701, EPI_ISL_718702, EPI_ISL_718703, EPI_ISL_718704	Lighthouse Lab in Cambridge	Wellcome Sanger Institute for the COVID-19 Genomics UK (COG-UK) Consortium	Rob Howes, The Lighthouse Lab in Cambridge and Alex Alderton, Roberto Amato, Sonia Goncalves, Ewan Harrison, David K. Jackson, Ian Johnston, Dominic Kwiatkowski, Cordelia Langford, John Sillitoe on behalf of the Wellcome Sanger Institute COVID-19 Surveillance Team
EPI_ISL_718705	Lighthouse Lab in Alderley Park	Wellcome Sanger Institute for the COVID-19 Genomics UK (COG-UK) Consortium	Jacquelyn Wynn, Mairead Hyland, The Lighthouse Lab in Alderley Park and Alex Alderton, Roberto Amato, Sonia Goncalves, Ewan Harrison, David K. Jackson, Ian Johnston, Dominic Kwiatkowski, Cordelia Langford, John Sillitoe on behalf of the Wellcome Sanger Institute COVID-19 Surveillance Team
EPI_ISL_718706	Lighthouse Lab in Cambridge	Wellcome Sanger Institute for the COVID-19 Genomics UK (COG-UK) Consortium	Rob Howes, The Lighthouse Lab in Cambridge and Alex Alderton, Roberto Amato, Sonia Goncalves, Ewan Harrison, David K. Jackson, Ian Johnston, Dominic Kwiatkowski, Cordelia Langford, John Sillitoe on behalf of the Wellcome Sanger Institute COVID-19 Surveillance Team
EPI_ISL_718707	Lighthouse Lab in Alderley Park	Wellcome Sanger Institute for the COVID-19 Genomics UK (COG-UK) Consortium	Jacquelyn Wynn, Mairead Hyland, The Lighthouse Lab in Alderley Park and Alex Alderton, Roberto Amato, Sonia Goncalves, Ewan Harrison, David K. Jackson, Ian Johnston, Dominic Kwiatkowski, Cordelia Langford, John Sillitoe on behalf of the Wellcome Sanger Institute COVID-19 Surveillance Team
EPI_ISL_718708, EPI_ISL_718709	Lighthouse Lab in Cambridge	Wellcome Sanger Institute for the COVID-19 Genomics UK (COG-UK) Consortium	Rob Howes, The Lighthouse Lab in Cambridge and Alex Alderton, Roberto Amato, Sonia Goncalves, Ewan Harrison, David K. Jackson, Ian Johnston, Dominic Kwiatkowski, Cordelia Langford, John Sillitoe on behalf of the Wellcome Sanger Institute COVID-19 Surveillance Team
EPI_ISL_718710, EPI_ISL_718711, EPI_ISL_718712, EPI_ISL_718713, EPI_ISL_718714, EPI_ISL_718715, EPI_ISL_718716	Lighthouse Lab in Alderley Park	Wellcome Sanger Institute for the COVID-19 Genomics UK (COG-UK) Consortium	Jacquelyn Wynn, Mairead Hyland, The Lighthouse Lab in Alderley Park and Alex Alderton, Roberto Amato, Sonia Goncalves, Ewan Harrison, David K. Jackson, Ian Johnston, Dominic Kwiatkowski, Cordelia Langford, John Sillitoe on behalf of the Wellcome Sanger Institute COVID-19 Surveillance Team
EPI_ISL_718717	Lighthouse Lab in Cambridge	Wellcome Sanger Institute for the COVID-19 Genomics UK (COG-UK) Consortium	Rob Howes, The Lighthouse Lab in Cambridge and Alex Alderton, Roberto Amato, Sonia Goncalves, Ewan Harrison, David K. Jackson, Ian Johnston, Dominic Kwiatkowski, Cordelia Langford, John Sillitoe on behalf of the Wellcome Sanger Institute COVID-19 Surveillance Team
EPI_ISL_718718, EPI_ISL_718719	Lighthouse Lab in Alderley Park	Wellcome Sanger Institute for the COVID-19 Genomics UK (COG-UK) Consortium	Jacquelyn Wynn, Mairead Hyland, The Lighthouse Lab in Alderley Park and Alex Alderton, Roberto Amato, Sonia Goncalves, Ewan Harrison, David K. Jackson, Ian Johnston, Dominic Kwiatkowski, Cordelia Langford, John Sillitoe on behalf of the Wellcome Sanger Institute COVID-19 Surveillance Team
EPI_ISL_718720	Lighthouse Lab in Cambridge	Wellcome Sanger Institute for the COVID-19 Genomics UK (COG-UK) Consortium	Rob Howes, The Lighthouse Lab in Cambridge and Alex Alderton, Roberto Amato, Sonia Goncalves, Ewan Harrison, David K. Jackson, Ian Johnston, Dominic Kwiatkowski, Cordelia Langford, John Sillitoe on behalf of the Wellcome Sanger Institute COVID-19 Surveillance Team
EPI_ISL_718721	Lighthouse Lab in Alderley Park	Wellcome Sanger Institute for the COVID-19 Genomics UK (COG-UK) Consortium	Jacquelyn Wynn, Mairead Hyland, The Lighthouse Lab in Alderley Park and Alex Alderton, Roberto Amato, Sonia Goncalves, Ewan Harrison, David K. Jackson, Ian Johnston, Dominic Kwiatkowski, Cordelia Langford, John Sillitoe on behalf of the Wellcome Sanger Institute COVID-19 Surveillance Team
EPI_ISL_718722	Lighthouse Lab in Cambridge	Wellcome Sanger Institute for the COVID-19 Genomics UK (COG-UK) Consortium	Rob Howes, The Lighthouse Lab in Cambridge and Alex Alderton, Roberto Amato, Sonia Goncalves, Ewan Harrison, David K. Jackson, Ian Johnston, Dominic Kwiatkowski, Cordelia Langford, John Sillitoe on behalf of the Wellcome Sanger Institute COVID-19 Surveillance Team
EPI_ISL_718723, EPI_ISL_718724, EPI_ISL_718725	Lighthouse Lab in Alderley Park	Wellcome Sanger Institute for the COVID-19 Genomics UK (COG-UK) Consortium	Jacquelyn Wynn, Mairead Hyland, The Lighthouse Lab in Alderley Park and Alex Alderton, Roberto Amato, Sonia Goncalves, Ewan Harrison, David K. Jackson, Ian Johnston, Dominic Kwiatkowski, Cordelia Langford, John Sillitoe on behalf of the Wellcome Sanger Institute COVID-19 Surveillance Team

EPI_ISL_718726, EPI_ISL_718727	Lighthouse Lab in Cambridge	Wellcome Sanger Institute for the COVID-19 Genomics UK (COG-UK) Consortium	Rob Howes, The Lighthouse Lab in Cambridge and Alex Alderton, Roberto Amato, Sonia Goncalves, Ewan Harrison, David K. Jackson, Ian Johnston, Dominic Kwiatkowski, Cordelia Langford, John Sillitoe on behalf of the Wellcome Sanger Institute COVID-19 Surveillance Team
EPI_ISL_718728, EPI_ISL_718729	Lighthouse Lab in Alderley Park	Wellcome Sanger Institute for the COVID-19 Genomics UK (COG-UK) Consortium	Jacquelyn Wynn, Mairead Hyland, The Lighthouse Lab in Alderley Park and Alex Alderton, Roberto Amato, Sonia Goncalves, Ewan Harrison, David K. Jackson, Ian Johnston, Dominic Kwiatkowski, Cordelia Langford, John Sillitoe on behalf of the Wellcome Sanger Institute COVID-19 Surveillance Team
EPI_ISL_718730, EPI_ISL_718731, EPI_ISL_718732, EPI_ISL_718733, EPI_ISL_718734	Lighthouse Lab in Cambridge	Wellcome Sanger Institute for the COVID-19 Genomics UK (COG-UK) Consortium	Rob Howes, The Lighthouse Lab in Cambridge and Alex Alderton, Roberto Amato, Sonia Goncalves, Ewan Harrison, David K. Jackson, Ian Johnston, Dominic Kwiatkowski, Cordelia Langford, John Sillitoe on behalf of the Wellcome Sanger Institute COVID-19 Surveillance Team
EPI_ISL_718735	Lighthouse Lab in Alderley Park	Wellcome Sanger Institute for the COVID-19 Genomics UK (COG-UK) Consortium	Jacquelyn Wynn, Mairead Hyland, The Lighthouse Lab in Alderley Park and Alex Alderton, Roberto Amato, Sonia Goncalves, Ewan Harrison, David K. Jackson, Ian Johnston, Dominic Kwiatkowski, Cordelia Langford, John Sillitoe on behalf of the Wellcome Sanger Institute COVID-19 Surveillance Team
EPI_ISL_718736, EPI_ISL_718737	Lighthouse Lab in Cambridge	Wellcome Sanger Institute for the COVID-19 Genomics UK (COG-UK) Consortium	Rob Howes, The Lighthouse Lab in Cambridge and Alex Alderton, Roberto Amato, Sonia Goncalves, Ewan Harrison, David K. Jackson, Ian Johnston, Dominic Kwiatkowski, Cordelia Langford, John Sillitoe on behalf of the Wellcome Sanger Institute COVID-19 Surveillance Team
EPI_ISL_718738, EPI_ISL_718739, EPI_ISL_718740	Lighthouse Lab in Alderley Park	Wellcome Sanger Institute for the COVID-19 Genomics UK (COG-UK) Consortium	Jacquelyn Wynn, Mairead Hyland, The Lighthouse Lab in Alderley Park and Alex Alderton, Roberto Amato, Sonia Goncalves, Ewan Harrison, David K. Jackson, Ian Johnston, Dominic Kwiatkowski, Cordelia Langford, John Sillitoe on behalf of the Wellcome Sanger Institute COVID-19 Surveillance Team
EPI_ISL_718741	Lighthouse Lab in Cambridge	Wellcome Sanger Institute for the COVID-19 Genomics UK (COG-UK) Consortium	Rob Howes, The Lighthouse Lab in Cambridge and Alex Alderton, Roberto Amato, Sonia Goncalves, Ewan Harrison, David K. Jackson, Ian Johnston, Dominic Kwiatkowski, Cordelia Langford, John Sillitoe on behalf of the Wellcome Sanger Institute COVID-19 Surveillance Team
EPI_ISL_718742	Lighthouse Lab in Alderley Park	Wellcome Sanger Institute for the COVID-19 Genomics UK (COG-UK) Consortium	Jacquelyn Wynn, Mairead Hyland, The Lighthouse Lab in Alderley Park and Alex Alderton, Roberto Amato, Sonia Goncalves, Ewan Harrison, David K. Jackson, Ian Johnston, Dominic Kwiatkowski, Cordelia Langford, John Sillitoe on behalf of the Wellcome Sanger Institute COVID-19 Surveillance Team
EPI_ISL_718743	Lighthouse Lab in Cambridge	Wellcome Sanger Institute for the COVID-19 Genomics UK (COG-UK) Consortium	Rob Howes, The Lighthouse Lab in Cambridge and Alex Alderton, Roberto Amato, Sonia Goncalves, Ewan Harrison, David K. Jackson, Ian Johnston, Dominic Kwiatkowski, Cordelia Langford, John Sillitoe on behalf of the Wellcome Sanger Institute COVID-19 Surveillance Team
EPI_ISL_718744, EPI_ISL_718745, EPI_ISL_718746, EPI_ISL_718747	Lighthouse Lab in Alderley Park	Wellcome Sanger Institute for the COVID-19 Genomics UK (COG-UK) Consortium	Jacquelyn Wynn, Mairead Hyland, The Lighthouse Lab in Alderley Park and Alex Alderton, Roberto Amato, Sonia Goncalves, Ewan Harrison, David K. Jackson, Ian Johnston, Dominic Kwiatkowski, Cordelia Langford, John Sillitoe on behalf of the Wellcome Sanger Institute COVID-19 Surveillance Team
EPI_ISL_718748, EPI_ISL_718749	Lighthouse Lab in Cambridge	Wellcome Sanger Institute for the COVID-19 Genomics UK (COG-UK) Consortium	Rob Howes, The Lighthouse Lab in Cambridge and Alex Alderton, Roberto Amato, Sonia Goncalves, Ewan Harrison, David K. Jackson, Ian Johnston, Dominic Kwiatkowski, Cordelia Langford, John Sillitoe on behalf of the Wellcome Sanger Institute COVID-19 Surveillance Team
EPI_ISL_718750	Lighthouse Lab in Alderley Park	Wellcome Sanger Institute for the COVID-19 Genomics UK (COG-UK) Consortium	Jacquelyn Wynn, Mairead Hyland, The Lighthouse Lab in Alderley Park and Alex Alderton, Roberto Amato, Sonia Goncalves, Ewan Harrison, David K. Jackson, Ian Johnston, Dominic Kwiatkowski, Cordelia Langford, John Sillitoe on behalf of the Wellcome Sanger Institute COVID-19 Surveillance Team
EPI_ISL_718751, EPI_ISL_718752, EPI_ISL_718753	Lighthouse Lab in Cambridge	Wellcome Sanger Institute for the COVID-19 Genomics UK (COG-UK) Consortium	Rob Howes, The Lighthouse Lab in Cambridge and Alex Alderton, Roberto Amato, Sonia Goncalves, Ewan Harrison, David K. Jackson, Ian Johnston, Dominic Kwiatkowski, Cordelia Langford, John Sillitoe on behalf of the Wellcome Sanger Institute COVID-19 Surveillance Team
EPI_ISL_718754, EPI_ISL_718755	Lighthouse Lab in Alderley Park	Wellcome Sanger Institute for the COVID-19 Genomics UK (COG-UK) Consortium	Jacquelyn Wynn, Mairead Hyland, The Lighthouse Lab in Alderley Park and Alex Alderton, Roberto Amato, Sonia Goncalves, Ewan Harrison, David K. Jackson, Ian Johnston, Dominic Kwiatkowski, Cordelia Langford, John Sillitoe on behalf of the Wellcome Sanger Institute COVID-19 Surveillance Team
EPI_ISL_718756, EPI_ISL_718757, EPI_ISL_718758	Lighthouse Lab in Cambridge	Wellcome Sanger Institute for the COVID-19 Genomics UK (COG-UK) Consortium	Rob Howes, The Lighthouse Lab in Cambridge and Alex Alderton, Roberto Amato, Sonia Goncalves, Ewan Harrison, David K. Jackson, Ian Johnston, Dominic Kwiatkowski, Cordelia Langford, John Sillitoe on behalf of the Wellcome Sanger Institute COVID-19 Surveillance Team
EPI_ISL_718759	Lighthouse Lab in Alderley Park	Wellcome Sanger Institute for the COVID-19 Genomics UK (COG-UK) Consortium	Jacquelyn Wynn, Mairead Hyland, The Lighthouse Lab in Alderley Park and Alex Alderton, Roberto Amato, Sonia Goncalves, Ewan Harrison, David K. Jackson, Ian Johnston, Dominic Kwiatkowski, Cordelia Langford, John Sillitoe on behalf of the Wellcome Sanger Institute COVID-19 Surveillance Team
EPI_ISL_718760	Lighthouse Lab in Cambridge	Wellcome Sanger Institute for the COVID-19 Genomics UK (COG-UK) Consortium	Rob Howes, The Lighthouse Lab in Cambridge and Alex Alderton, Roberto Amato, Sonia Goncalves, Ewan Harrison, David K. Jackson, Ian Johnston, Dominic Kwiatkowski, Cordelia Langford, John Sillitoe on behalf of the Wellcome Sanger Institute COVID-19 Surveillance Team
EPI_ISL_718761, EPI_ISL_718762	Lighthouse Lab in Alderley Park	Wellcome Sanger Institute for the COVID-19 Genomics UK (COG-UK) Consortium	Jacquelyn Wynn, Mairead Hyland, The Lighthouse Lab in Alderley Park and Alex Alderton, Roberto Amato, Sonia Goncalves, Ewan Harrison, David K. Jackson, Ian Johnston, Dominic Kwiatkowski, Cordelia Langford, John Sillitoe on behalf of the Wellcome Sanger Institute COVID-19 Surveillance Team
EPI_ISL_718763	Lighthouse Lab in Cambridge	Wellcome Sanger Institute for the COVID-19 Genomics UK (COG-UK) Consortium	Rob Howes, The Lighthouse Lab in Cambridge and Alex Alderton, Roberto Amato, Sonia Goncalves, Ewan Harrison, David K. Jackson, Ian Johnston, Dominic Kwiatkowski, Cordelia Langford, John Sillitoe on behalf of the Wellcome Sanger Institute COVID-19 Surveillance Team
EPI_ISL_718764, EPI_ISL_718765, EPI_ISL_718766, EPI_ISL_718767	Lighthouse Lab in Alderley Park	Wellcome Sanger Institute for the COVID-19 Genomics UK (COG-UK) Consortium	Jacquelyn Wynn, Mairead Hyland, The Lighthouse Lab in Alderley Park and Alex Alderton, Roberto Amato, Sonia Goncalves, Ewan Harrison, David K. Jackson, Ian Johnston, Dominic Kwiatkowski, Cordelia Langford, John Sillitoe on behalf of the Wellcome Sanger Institute COVID-19 Surveillance Team
EPI_ISL_718768	Lighthouse Lab in Cambridge	Wellcome Sanger Institute for the COVID-19 Genomics UK (COG-UK) Consortium	Rob Howes, The Lighthouse Lab in Cambridge and Alex Alderton, Roberto Amato, Sonia Goncalves, Ewan Harrison, David K. Jackson, Ian Johnston, Dominic Kwiatkowski, Cordelia Langford, John Sillitoe on behalf of the Wellcome Sanger Institute COVID-19 Surveillance Team
EPI_ISL_718769	Lighthouse Lab in Alderley Park	Wellcome Sanger Institute for the COVID-19 Genomics UK (COG-UK) Consortium	Jacquelyn Wynn, Mairead Hyland, The Lighthouse Lab in Alderley Park and Alex Alderton, Roberto Amato, Sonia Goncalves, Ewan Harrison, David K. Jackson, Ian Johnston, Dominic Kwiatkowski, Cordelia Langford, John Sillitoe on behalf of the Wellcome Sanger Institute COVID-19 Surveillance Team
EPI_ISL_718770, EPI_ISL_718771	Lighthouse Lab in Cambridge	Wellcome Sanger Institute for the COVID-19 Genomics UK (COG-UK) Consortium	Rob Howes, The Lighthouse Lab in Cambridge and Alex Alderton, Roberto Amato, Sonia Goncalves, Ewan Harrison, David K. Jackson, Ian Johnston, Dominic Kwiatkowski, Cordelia Langford, John Sillitoe on behalf of the Wellcome Sanger Institute COVID-19 Surveillance Team
EPI_ISL_718772, EPI_ISL_718773, EPI_ISL_718774	Lighthouse Lab in Alderley Park	Wellcome Sanger Institute for the COVID-19 Genomics UK (COG-UK) Consortium	Jacquelyn Wynn, Mairead Hyland, The Lighthouse Lab in Alderley Park and Alex Alderton, Roberto Amato, Sonia Goncalves, Ewan Harrison, David K. Jackson, Ian Johnston, Dominic Kwiatkowski, Cordelia Langford, John Sillitoe on behalf of the Wellcome Sanger Institute COVID-19 Surveillance Team
EPI_ISL_718775, EPI_ISL_718776	Lighthouse Lab in Cambridge	Wellcome Sanger Institute for the COVID-19 Genomics UK (COG-UK) Consortium	Rob Howes, The Lighthouse Lab in Cambridge and Alex Alderton, Roberto Amato, Sonia Goncalves, Ewan Harrison, David K. Jackson, Ian Johnston, Dominic Kwiatkowski, Cordelia Langford, John Sillitoe on behalf of the Wellcome Sanger Institute COVID-19 Surveillance Team
EPI_ISL_718777	Lighthouse Lab in Alderley Park	Wellcome Sanger Institute for the COVID-19 Genomics UK (COG-UK) Consortium	Jacquelyn Wynn, Mairead Hyland, The Lighthouse Lab in Alderley Park and Alex Alderton, Roberto Amato, Sonia Goncalves, Ewan Harrison, David K. Jackson, Ian Johnston, Dominic Kwiatkowski, Cordelia Langford, John Sillitoe on behalf of the Wellcome Sanger Institute COVID-19 Surveillance Team
EPI_ISL_718778	Lighthouse Lab in Cambridge	Wellcome Sanger Institute for the COVID-19 Genomics UK (COG-UK) Consortium	Rob Howes, The Lighthouse Lab in Cambridge and Alex Alderton, Roberto Amato, Sonia Goncalves, Ewan Harrison, David K. Jackson, Ian Johnston, Dominic Kwiatkowski, Cordelia Langford, John Sillitoe on behalf of the Wellcome Sanger Institute COVID-19 Surveillance Team
EPI_ISL_718779, EPI_ISL_718780, EPI_ISL_718781, EPI_ISL_718782, EPI_ISL_718783	Lighthouse Lab in Alderley Park	Wellcome Sanger Institute for the COVID-19 Genomics UK (COG-UK) Consortium	Jacquelyn Wynn, Mairead Hyland, The Lighthouse Lab in Alderley Park and Alex Alderton, Roberto Amato, Sonia Goncalves, Ewan Harrison, David K. Jackson, Ian Johnston, Dominic Kwiatkowski, Cordelia Langford, John Sillitoe on behalf of the Wellcome Sanger Institute COVID-19 Surveillance Team
EPI_ISL_718784, EPI_ISL_718785, EPI_ISL_718786	Lighthouse Lab in Cambridge	Wellcome Sanger Institute for the COVID-19 Genomics UK (COG-UK) Consortium	Rob Howes, The Lighthouse Lab in Cambridge and Alex Alderton, Roberto Amato, Sonia Goncalves, Ewan Harrison, David K. Jackson, Ian Johnston, Dominic Kwiatkowski, Cordelia Langford, John Sillitoe on behalf of the Wellcome Sanger Institute COVID-19 Surveillance Team
EPI_ISL_718787	Lighthouse Lab in Alderley Park	Wellcome Sanger Institute for the COVID-19 Genomics UK (COG-UK) Consortium	Jacquelyn Wynn, Mairead Hyland, The Lighthouse Lab in Alderley Park and Alex Alderton, Roberto Amato, Sonia Goncalves, Ewan Harrison, David K. Jackson, Ian Johnston, Dominic Kwiatkowski, Cordelia Langford, John Sillitoe on behalf of the Wellcome Sanger Institute COVID-19 Surveillance Team
EPI_ISL_718788	Lighthouse Lab in Cambridge	Wellcome Sanger Institute for the COVID-19 Genomics UK (COG-UK) Consortium	Rob Howes, The Lighthouse Lab in Cambridge and Alex Alderton, Roberto Amato, Sonia Goncalves, Ewan Harrison, David K. Jackson, Ian Johnston, Dominic Kwiatkowski, Cordelia Langford, John Sillitoe on behalf of the Wellcome Sanger Institute COVID-19 Surveillance Team
EPI_ISL_718789	Lighthouse Lab in Alderley Park	Wellcome Sanger Institute for the COVID-19 Genomics UK (COG-UK) Consortium	Jacquelyn Wynn, Mairead Hyland, The Lighthouse Lab in Alderley Park and Alex Alderton, Roberto Amato, Sonia Goncalves, Ewan Harrison, David K. Jackson, Ian Johnston, Dominic Kwiatkowski, Cordelia Langford, John Sillitoe on behalf of the Wellcome Sanger Institute COVID-19 Surveillance Team
EPI_ISL_718790	Lighthouse Lab in Cambridge	Wellcome Sanger Institute for the COVID-19 Genomics UK (COG-UK) Consortium	Rob Howes, The Lighthouse Lab in Cambridge and Alex Alderton, Roberto Amato, Sonia Goncalves, Ewan Harrison, David K. Jackson, Ian Johnston, Dominic Kwiatkowski, Cordelia Langford, John Sillitoe on behalf of the Wellcome Sanger Institute COVID-19 Surveillance Team
EPI_ISL_718791	Lighthouse Lab in Alderley Park	Wellcome Sanger Institute for the COVID-19 Genomics UK (COG-UK) Consortium	Jacquelyn Wynn, Mairead Hyland, The Lighthouse Lab in Alderley Park and Alex Alderton, Roberto Amato, Sonia Goncalves, Ewan Harrison, David K. Jackson, Ian Johnston, Dominic Kwiatkowski, Cordelia Langford, John Sillitoe on behalf of the Wellcome Sanger Institute COVID-19 Surveillance Team
EPI_ISL_718792, EPI_ISL_718793,	Lighthouse Lab in Cambridge	Wellcome Sanger Institute for the COVID-19 Genomics UK	Rob Howes, The Lighthouse Lab in Cambridge and Alex Alderton, Roberto Amato, Sonia Goncalves, Ewan Harrison, David K. Jackson, Ian Johnston,

EPI_ISL_718794		(COG-UK) Consortium	Dominic Kwiatkowski, Cordelia Langford, John Sillitoe on behalf of the Wellcome Sanger Institute COVID-19 Surveillance Team
EPI_ISL_718795, EPI_ISL_718796	Lighthouse Lab in Alderley Park	Wellcome Sanger Institute for the COVID-19 Genomics UK (COG-UK) Consortium	Jacquelyn Wynn, Mairead Hyland, The Lighthouse Lab in Alderley Park and Alex Alderton, Roberto Amato, Sonia Goncalves, Ewan Harrison, David K. Jackson, Ian Johnston, Dominic Kwiatkowski, Cordelia Langford, John Sillitoe on behalf of the Wellcome Sanger Institute COVID-19 Surveillance Team
EPI_ISL_718797	Lighthouse Lab in Cambridge	Wellcome Sanger Institute for the COVID-19 Genomics UK (COG-UK) Consortium	Rob Howes, The Lighthouse Lab in Cambridge and Alex Alderton, Roberto Amato, Sonia Goncalves, Ewan Harrison, David K. Jackson, Ian Johnston, Dominic Kwiatkowski, Cordelia Langford, John Sillitoe on behalf of the Wellcome Sanger Institute COVID-19 Surveillance Team
EPI_ISL_718798, EPI_ISL_718799	Lighthouse Lab in Alderley Park	Wellcome Sanger Institute for the COVID-19 Genomics UK (COG-UK) Consortium	Jacquelyn Wynn, Mairead Hyland, The Lighthouse Lab in Alderley Park and Alex Alderton, Roberto Amato, Sonia Goncalves, Ewan Harrison, David K. Jackson, Ian Johnston, Dominic Kwiatkowski, Cordelia Langford, John Sillitoe on behalf of the Wellcome Sanger Institute COVID-19 Surveillance Team
EPI_ISL_718800	Lighthouse Lab in Cambridge	Wellcome Sanger Institute for the COVID-19 Genomics UK (COG-UK) Consortium	Rob Howes, The Lighthouse Lab in Cambridge and Alex Alderton, Roberto Amato, Sonia Goncalves, Ewan Harrison, David K. Jackson, Ian Johnston, Dominic Kwiatkowski, Cordelia Langford, John Sillitoe on behalf of the Wellcome Sanger Institute COVID-19 Surveillance Team
EPI_ISL_718801, EPI_ISL_718802, EPI_ISL_718803	Lighthouse Lab in Alderley Park	Wellcome Sanger Institute for the COVID-19 Genomics UK (COG-UK) Consortium	Jacquelyn Wynn, Mairead Hyland, The Lighthouse Lab in Alderley Park and Alex Alderton, Roberto Amato, Sonia Goncalves, Ewan Harrison, David K. Jackson, Ian Johnston, Dominic Kwiatkowski, Cordelia Langford, John Sillitoe on behalf of the Wellcome Sanger Institute COVID-19 Surveillance Team
EPI_ISL_718804, EPI_ISL_718805	Lighthouse Lab in Cambridge	Wellcome Sanger Institute for the COVID-19 Genomics UK (COG-UK) Consortium	Rob Howes, The Lighthouse Lab in Cambridge and Alex Alderton, Roberto Amato, Sonia Goncalves, Ewan Harrison, David K. Jackson, Ian Johnston, Dominic Kwiatkowski, Cordelia Langford, John Sillitoe on behalf of the Wellcome Sanger Institute COVID-19 Surveillance Team
EPI_ISL_718806, EPI_ISL_718807, EPI_ISL_718808	Lighthouse Lab in Alderley Park	Wellcome Sanger Institute for the COVID-19 Genomics UK (COG-UK) Consortium	Jacquelyn Wynn, Mairead Hyland, The Lighthouse Lab in Alderley Park and Alex Alderton, Roberto Amato, Sonia Goncalves, Ewan Harrison, David K. Jackson, Ian Johnston, Dominic Kwiatkowski, Cordelia Langford, John Sillitoe on behalf of the Wellcome Sanger Institute COVID-19 Surveillance Team
EPI_ISL_718809, EPI_ISL_718810	Lighthouse Lab in Cambridge	Wellcome Sanger Institute for the COVID-19 Genomics UK (COG-UK) Consortium	Rob Howes, The Lighthouse Lab in Cambridge and Alex Alderton, Roberto Amato, Sonia Goncalves, Ewan Harrison, David K. Jackson, Ian Johnston, Dominic Kwiatkowski, Cordelia Langford, John Sillitoe on behalf of the Wellcome Sanger Institute COVID-19 Surveillance Team
EPI_ISL_718811, EPI_ISL_718812, EPI_ISL_718813	Lighthouse Lab in Alderley Park	Wellcome Sanger Institute for the COVID-19 Genomics UK (COG-UK) Consortium	Jacquelyn Wynn, Mairead Hyland, The Lighthouse Lab in Alderley Park and Alex Alderton, Roberto Amato, Sonia Goncalves, Ewan Harrison, David K. Jackson, Ian Johnston, Dominic Kwiatkowski, Cordelia Langford, John Sillitoe on behalf of the Wellcome Sanger Institute COVID-19 Surveillance Team
EPI_ISL_718814, EPI_ISL_718815, EPI_ISL_718816, EPI_ISL_718817, EPI_ISL_718818	Lighthouse Lab in Cambridge	Wellcome Sanger Institute for the COVID-19 Genomics UK (COG-UK) Consortium	Rob Howes, The Lighthouse Lab in Cambridge and Alex Alderton, Roberto Amato, Sonia Goncalves, Ewan Harrison, David K. Jackson, Ian Johnston, Dominic Kwiatkowski, Cordelia Langford, John Sillitoe on behalf of the Wellcome Sanger Institute COVID-19 Surveillance Team
EPI_ISL_718819	Lighthouse Lab in Alderley Park	Wellcome Sanger Institute for the COVID-19 Genomics UK (COG-UK) Consortium	Jacquelyn Wynn, Mairead Hyland, The Lighthouse Lab in Alderley Park and Alex Alderton, Roberto Amato, Sonia Goncalves, Ewan Harrison, David K. Jackson, Ian Johnston, Dominic Kwiatkowski, Cordelia Langford, John Sillitoe on behalf of the Wellcome Sanger Institute COVID-19 Surveillance Team
EPI_ISL_718820	Lighthouse Lab in Cambridge	Wellcome Sanger Institute for the COVID-19 Genomics UK (COG-UK) Consortium	Rob Howes, The Lighthouse Lab in Cambridge and Alex Alderton, Roberto Amato, Sonia Goncalves, Ewan Harrison, David K. Jackson, Ian Johnston, Dominic Kwiatkowski, Cordelia Langford, John Sillitoe on behalf of the Wellcome Sanger Institute COVID-19 Surveillance Team
EPI_ISL_718821	Lighthouse Lab in Alderley Park	Wellcome Sanger Institute for the COVID-19 Genomics UK (COG-UK) Consortium	Jacquelyn Wynn, Mairead Hyland, The Lighthouse Lab in Alderley Park and Alex Alderton, Roberto Amato, Sonia Goncalves, Ewan Harrison, David K. Jackson, Ian Johnston, Dominic Kwiatkowski, Cordelia Langford, John Sillitoe on behalf of the Wellcome Sanger Institute COVID-19 Surveillance Team
EPI_ISL_718822	Lighthouse Lab in Cambridge	Wellcome Sanger Institute for the COVID-19 Genomics UK (COG-UK) Consortium	Rob Howes, The Lighthouse Lab in Cambridge and Alex Alderton, Roberto Amato, Sonia Goncalves, Ewan Harrison, David K. Jackson, Ian Johnston, Dominic Kwiatkowski, Cordelia Langford, John Sillitoe on behalf of the Wellcome Sanger Institute COVID-19 Surveillance Team
EPI_ISL_718823	Lighthouse Lab in Alderley Park	Wellcome Sanger Institute for the COVID-19 Genomics UK (COG-UK) Consortium	Jacquelyn Wynn, Mairead Hyland, The Lighthouse Lab in Alderley Park and Alex Alderton, Roberto Amato, Sonia Goncalves, Ewan Harrison, David K. Jackson, Ian Johnston, Dominic Kwiatkowski, Cordelia Langford, John Sillitoe on behalf of the Wellcome Sanger Institute COVID-19 Surveillance Team
EPI_ISL_718824, EPI_ISL_718825, EPI_ISL_718826	Lighthouse Lab in Cambridge	Wellcome Sanger Institute for the COVID-19 Genomics UK (COG-UK) Consortium	Rob Howes, The Lighthouse Lab in Cambridge and Alex Alderton, Roberto Amato, Sonia Goncalves, Ewan Harrison, David K. Jackson, Ian Johnston, Dominic Kwiatkowski, Cordelia Langford, John Sillitoe on behalf of the Wellcome Sanger Institute COVID-19 Surveillance Team
EPI_ISL_718827, EPI_ISL_718828, EPI_ISL_718829, EPI_ISL_718830, EPI_ISL_718831, EPI_ISL_718832, EPI_ISL_718833	Lighthouse Lab in Alderley Park	Wellcome Sanger Institute for the COVID-19 Genomics UK (COG-UK) Consortium	Jacquelyn Wynn, Mairead Hyland, The Lighthouse Lab in Alderley Park and Alex Alderton, Roberto Amato, Sonia Goncalves, Ewan Harrison, David K. Jackson, Ian Johnston, Dominic Kwiatkowski, Cordelia Langford, John Sillitoe on behalf of the Wellcome Sanger Institute COVID-19 Surveillance Team
EPI_ISL_718834, EPI_ISL_718835, EPI_ISL_718836	Lighthouse Lab in Cambridge	Wellcome Sanger Institute for the COVID-19 Genomics UK (COG-UK) Consortium	Rob Howes, The Lighthouse Lab in Cambridge and Alex Alderton, Roberto Amato, Sonia Goncalves, Ewan Harrison, David K. Jackson, Ian Johnston, Dominic Kwiatkowski, Cordelia Langford, John Sillitoe on behalf of the Wellcome Sanger Institute COVID-19 Surveillance Team
EPI_ISL_718837, EPI_ISL_718838, EPI_ISL_718839	Lighthouse Lab in Alderley Park	Wellcome Sanger Institute for the COVID-19 Genomics UK (COG-UK) Consortium	Jacquelyn Wynn, Mairead Hyland, The Lighthouse Lab in Alderley Park and Alex Alderton, Roberto Amato, Sonia Goncalves, Ewan Harrison, David K. Jackson, Ian Johnston, Dominic Kwiatkowski, Cordelia Langford, John Sillitoe on behalf of the Wellcome Sanger Institute COVID-19 Surveillance Team
EPI_ISL_718840	Lighthouse Lab in Cambridge	Wellcome Sanger Institute for the COVID-19 Genomics UK (COG-UK) Consortium	Rob Howes, The Lighthouse Lab in Cambridge and Alex Alderton, Roberto Amato, Sonia Goncalves, Ewan Harrison, David K. Jackson, Ian Johnston, Dominic Kwiatkowski, Cordelia Langford, John Sillitoe on behalf of the Wellcome Sanger Institute COVID-19 Surveillance Team
EPI_ISL_718841	Lighthouse Lab in Alderley Park	Wellcome Sanger Institute for the COVID-19 Genomics UK (COG-UK) Consortium	Jacquelyn Wynn, Mairead Hyland, The Lighthouse Lab in Alderley Park and Alex Alderton, Roberto Amato, Sonia Goncalves, Ewan Harrison, David K. Jackson, Ian Johnston, Dominic Kwiatkowski, Cordelia Langford, John Sillitoe on behalf of the Wellcome Sanger Institute COVID-19 Surveillance Team
EPI_ISL_718842, EPI_ISL_718843	Lighthouse Lab in Cambridge	Wellcome Sanger Institute for the COVID-19 Genomics UK (COG-UK) Consortium	Rob Howes, The Lighthouse Lab in Cambridge and Alex Alderton, Roberto Amato, Sonia Goncalves, Ewan Harrison, David K. Jackson, Ian Johnston, Dominic Kwiatkowski, Cordelia Langford, John Sillitoe on behalf of the Wellcome Sanger Institute COVID-19 Surveillance Team
EPI_ISL_718844	Lighthouse Lab in Alderley Park	Wellcome Sanger Institute for the COVID-19 Genomics UK (COG-UK) Consortium	Jacquelyn Wynn, Mairead Hyland, The Lighthouse Lab in Alderley Park and Alex Alderton, Roberto Amato, Sonia Goncalves, Ewan Harrison, David K. Jackson, Ian Johnston, Dominic Kwiatkowski, Cordelia Langford, John Sillitoe on behalf of the Wellcome Sanger Institute COVID-19 Surveillance Team
EPI_ISL_718845, EPI_ISL_718846	Lighthouse Lab in Cambridge	Wellcome Sanger Institute for the COVID-19 Genomics UK (COG-UK) Consortium	Rob Howes, The Lighthouse Lab in Cambridge and Alex Alderton, Roberto Amato, Sonia Goncalves, Ewan Harrison, David K. Jackson, Ian Johnston, Dominic Kwiatkowski, Cordelia Langford, John Sillitoe on behalf of the Wellcome Sanger Institute COVID-19 Surveillance Team
EPI_ISL_718847	Lighthouse Lab in Alderley Park	Wellcome Sanger Institute for the COVID-19 Genomics UK (COG-UK) Consortium	Jacquelyn Wynn, Mairead Hyland, The Lighthouse Lab in Alderley Park and Alex Alderton, Roberto Amato, Sonia Goncalves, Ewan Harrison, David K. Jackson, Ian Johnston, Dominic Kwiatkowski, Cordelia Langford, John Sillitoe on behalf of the Wellcome Sanger Institute COVID-19 Surveillance Team
EPI_ISL_718848	Lighthouse Lab in Cambridge	Wellcome Sanger Institute for the COVID-19 Genomics UK (COG-UK) Consortium	Rob Howes, The Lighthouse Lab in Cambridge and Alex Alderton, Roberto Amato, Sonia Goncalves, Ewan Harrison, David K. Jackson, Ian Johnston, Dominic Kwiatkowski, Cordelia Langford, John Sillitoe on behalf of the Wellcome Sanger Institute COVID-19 Surveillance Team
EPI_ISL_718849, EPI_ISL_718850	Lighthouse Lab in Alderley Park	Wellcome Sanger Institute for the COVID-19 Genomics UK (COG-UK) Consortium	Jacquelyn Wynn, Mairead Hyland, The Lighthouse Lab in Alderley Park and Alex Alderton, Roberto Amato, Sonia Goncalves, Ewan Harrison, David K. Jackson, Ian Johnston, Dominic Kwiatkowski, Cordelia Langford, John Sillitoe on behalf of the Wellcome Sanger Institute COVID-19 Surveillance Team
EPI_ISL_718851, EPI_ISL_718852	Lighthouse Lab in Cambridge	Wellcome Sanger Institute for the COVID-19 Genomics UK (COG-UK) Consortium	Rob Howes, The Lighthouse Lab in Cambridge and Alex Alderton, Roberto Amato, Sonia Goncalves, Ewan Harrison, David K. Jackson, Ian Johnston, Dominic Kwiatkowski, Cordelia Langford, John Sillitoe on behalf of the Wellcome Sanger Institute COVID-19 Surveillance Team
EPI_ISL_718853, EPI_ISL_718854	Lighthouse Lab in Alderley Park	Wellcome Sanger Institute for the COVID-19 Genomics UK (COG-UK) Consortium	Jacquelyn Wynn, Mairead Hyland, The Lighthouse Lab in Alderley Park and Alex Alderton, Roberto Amato, Sonia Goncalves, Ewan Harrison, David K. Jackson, Ian Johnston, Dominic Kwiatkowski, Cordelia Langford, John Sillitoe on behalf of the Wellcome Sanger Institute COVID-19 Surveillance Team
EPI_ISL_718855, EPI_ISL_718856, EPI_ISL_718857	Lighthouse Lab in Cambridge	Wellcome Sanger Institute for the COVID-19 Genomics UK (COG-UK) Consortium	Rob Howes, The Lighthouse Lab in Cambridge and Alex Alderton, Roberto Amato, Sonia Goncalves, Ewan Harrison, David K. Jackson, Ian Johnston, Dominic Kwiatkowski, Cordelia Langford, John Sillitoe on behalf of the Wellcome Sanger Institute COVID-19 Surveillance Team
EPI_ISL_718858, EPI_ISL_718859	Lighthouse Lab in Alderley Park	Wellcome Sanger Institute for the COVID-19 Genomics UK (COG-UK) Consortium	Jacquelyn Wynn, Mairead Hyland, The Lighthouse Lab in Alderley Park and Alex Alderton, Roberto Amato, Sonia Goncalves, Ewan Harrison, David K. Jackson, Ian Johnston, Dominic Kwiatkowski, Cordelia Langford, John Sillitoe on behalf of the Wellcome Sanger Institute COVID-19 Surveillance Team
EPI_ISL_718860, EPI_ISL_718861, EPI_ISL_718862, EPI_ISL_718863	Lighthouse Lab in Cambridge	Wellcome Sanger Institute for the COVID-19 Genomics UK (COG-UK) Consortium	Rob Howes, The Lighthouse Lab in Cambridge and Alex Alderton, Roberto Amato, Sonia Goncalves, Ewan Harrison, David K. Jackson, Ian Johnston, Dominic Kwiatkowski, Cordelia Langford, John Sillitoe on behalf of the Wellcome Sanger Institute COVID-19 Surveillance Team
EPI_ISL_718864, EPI_ISL_718865, EPI_ISL_718866	Lighthouse Lab in Alderley Park	Wellcome Sanger Institute for the COVID-19 Genomics UK (COG-UK) Consortium	Jacquelyn Wynn, Mairead Hyland, The Lighthouse Lab in Alderley Park and Alex Alderton, Roberto Amato, Sonia Goncalves, Ewan Harrison, David K. Jackson, Ian Johnston, Dominic Kwiatkowski, Cordelia Langford, John Sillitoe on behalf of the Wellcome Sanger Institute COVID-19 Surveillance Team
EPI_ISL_718867, EPI_ISL_718868,	Lighthouse Lab in Cambridge	Wellcome Sanger Institute for the COVID-19 Genomics UK	Rob Howes, The Lighthouse Lab in Cambridge and Alex Alderton, Roberto Amato, Sonia Goncalves, Ewan Harrison, David K. Jackson, Ian Johnston,

EPI_ISL_718869		(COG-UK) Consortium	Dominic Kwiatkowski, Cordelia Langford, John Sillitoe on behalf of the Wellcome Sanger Institute COVID-19 Surveillance Team
EPI_ISL_718870, EPI_ISL_718871, EPI_ISL_718872, EPI_ISL_718873	Lighthouse Lab in Alderley Park	Wellcome Sanger Institute for the COVID-19 Genomics UK (COG-UK) Consortium	Jacquelyn Wynn, Mairead Hyland, The Lighthouse Lab in Alderley Park and Alex Alderton, Roberto Amato, Sonia Goncalves, Ewan Harrison, David K. Jackson, Ian Johnston, Dominic Kwiatkowski, Cordelia Langford, John Sillitoe on behalf of the Wellcome Sanger Institute COVID-19 Surveillance Team
EPI_ISL_718874, EPI_ISL_718875, EPI_ISL_718876	Lighthouse Lab in Cambridge	Wellcome Sanger Institute for the COVID-19 Genomics UK (COG-UK) Consortium	Rob Howes, The Lighthouse Lab in Cambridge and Alex Alderton, Roberto Amato, Sonia Goncalves, Ewan Harrison, David K. Jackson, Ian Johnston, Dominic Kwiatkowski, Cordelia Langford, John Sillitoe on behalf of the Wellcome Sanger Institute COVID-19 Surveillance Team
EPI_ISL_718877, EPI_ISL_718878	Lighthouse Lab in Alderley Park	Wellcome Sanger Institute for the COVID-19 Genomics UK (COG-UK) Consortium	Jacquelyn Wynn, Mairead Hyland, The Lighthouse Lab in Alderley Park and Alex Alderton, Roberto Amato, Sonia Goncalves, Ewan Harrison, David K. Jackson, Ian Johnston, Dominic Kwiatkowski, Cordelia Langford, John Sillitoe on behalf of the Wellcome Sanger Institute COVID-19 Surveillance Team
EPI_ISL_718879	Lighthouse Lab in Cambridge	Wellcome Sanger Institute for the COVID-19 Genomics UK (COG-UK) Consortium	Rob Howes, The Lighthouse Lab in Cambridge and Alex Alderton, Roberto Amato, Sonia Goncalves, Ewan Harrison, David K. Jackson, Ian Johnston, Dominic Kwiatkowski, Cordelia Langford, John Sillitoe on behalf of the Wellcome Sanger Institute COVID-19 Surveillance Team
EPI_ISL_718880, EPI_ISL_718881	Lighthouse Lab in Alderley Park	Wellcome Sanger Institute for the COVID-19 Genomics UK (COG-UK) Consortium	Jacquelyn Wynn, Mairead Hyland, The Lighthouse Lab in Alderley Park and Alex Alderton, Roberto Amato, Sonia Goncalves, Ewan Harrison, David K. Jackson, Ian Johnston, Dominic Kwiatkowski, Cordelia Langford, John Sillitoe on behalf of the Wellcome Sanger Institute COVID-19 Surveillance Team
EPI_ISL_718882, EPI_ISL_718883, EPI_ISL_718884	Lighthouse Lab in Cambridge	Wellcome Sanger Institute for the COVID-19 Genomics UK (COG-UK) Consortium	Rob Howes, The Lighthouse Lab in Cambridge and Alex Alderton, Roberto Amato, Sonia Goncalves, Ewan Harrison, David K. Jackson, Ian Johnston, Dominic Kwiatkowski, Cordelia Langford, John Sillitoe on behalf of the Wellcome Sanger Institute COVID-19 Surveillance Team
EPI_ISL_718885, EPI_ISL_718886, EPI_ISL_718887, EPI_ISL_718888, EPI_ISL_718889, EPI_ISL_718890, EPI_ISL_718891	Lighthouse Lab in Alderley Park	Wellcome Sanger Institute for the COVID-19 Genomics UK (COG-UK) Consortium	Jacquelyn Wynn, Mairead Hyland, The Lighthouse Lab in Alderley Park and Alex Alderton, Roberto Amato, Sonia Goncalves, Ewan Harrison, David K. Jackson, Ian Johnston, Dominic Kwiatkowski, Cordelia Langford, John Sillitoe on behalf of the Wellcome Sanger Institute COVID-19 Surveillance Team
EPI_ISL_718892, EPI_ISL_718893	Lighthouse Lab in Cambridge	Wellcome Sanger Institute for the COVID-19 Genomics UK (COG-UK) Consortium	Rob Howes, The Lighthouse Lab in Cambridge and Alex Alderton, Roberto Amato, Sonia Goncalves, Ewan Harrison, David K. Jackson, Ian Johnston, Dominic Kwiatkowski, Cordelia Langford, John Sillitoe on behalf of the Wellcome Sanger Institute COVID-19 Surveillance Team
EPI_ISL_718894, EPI_ISL_718895	Lighthouse Lab in Alderley Park	Wellcome Sanger Institute for the COVID-19 Genomics UK (COG-UK) Consortium	Jacquelyn Wynn, Mairead Hyland, The Lighthouse Lab in Alderley Park and Alex Alderton, Roberto Amato, Sonia Goncalves, Ewan Harrison, David K. Jackson, Ian Johnston, Dominic Kwiatkowski, Cordelia Langford, John Sillitoe on behalf of the Wellcome Sanger Institute COVID-19 Surveillance Team
EPI_ISL_718896, EPI_ISL_718897, EPI_ISL_718898, EPI_ISL_718899, EPI_ISL_718900	Lighthouse Lab in Cambridge	Wellcome Sanger Institute for the COVID-19 Genomics UK (COG-UK) Consortium	Rob Howes, The Lighthouse Lab in Cambridge and Alex Alderton, Roberto Amato, Sonia Goncalves, Ewan Harrison, David K. Jackson, Ian Johnston, Dominic Kwiatkowski, Cordelia Langford, John Sillitoe on behalf of the Wellcome Sanger Institute COVID-19 Surveillance Team
EPI_ISL_718901, EPI_ISL_718902, EPI_ISL_718903	Lighthouse Lab in Alderley Park	Wellcome Sanger Institute for the COVID-19 Genomics UK (COG-UK) Consortium	Jacquelyn Wynn, Mairead Hyland, The Lighthouse Lab in Alderley Park and Alex Alderton, Roberto Amato, Sonia Goncalves, Ewan Harrison, David K. Jackson, Ian Johnston, Dominic Kwiatkowski, Cordelia Langford, John Sillitoe on behalf of the Wellcome Sanger Institute COVID-19 Surveillance Team
EPI_ISL_718904, EPI_ISL_718905, EPI_ISL_718906	Lighthouse Lab in Cambridge	Wellcome Sanger Institute for the COVID-19 Genomics UK (COG-UK) Consortium	Rob Howes, The Lighthouse Lab in Cambridge and Alex Alderton, Roberto Amato, Sonia Goncalves, Ewan Harrison, David K. Jackson, Ian Johnston, Dominic Kwiatkowski, Cordelia Langford, John Sillitoe on behalf of the Wellcome Sanger Institute COVID-19 Surveillance Team
EPI_ISL_718907, EPI_ISL_718908	Lighthouse Lab in Alderley Park	Wellcome Sanger Institute for the COVID-19 Genomics UK (COG-UK) Consortium	Jacquelyn Wynn, Mairead Hyland, The Lighthouse Lab in Alderley Park and Alex Alderton, Roberto Amato, Sonia Goncalves, Ewan Harrison, David K. Jackson, Ian Johnston, Dominic Kwiatkowski, Cordelia Langford, John Sillitoe on behalf of the Wellcome Sanger Institute COVID-19 Surveillance Team
EPI_ISL_718909, EPI_ISL_718910	Lighthouse Lab in Cambridge	Wellcome Sanger Institute for the COVID-19 Genomics UK (COG-UK) Consortium	Rob Howes, The Lighthouse Lab in Cambridge and Alex Alderton, Roberto Amato, Sonia Goncalves, Ewan Harrison, David K. Jackson, Ian Johnston, Dominic Kwiatkowski, Cordelia Langford, John Sillitoe on behalf of the Wellcome Sanger Institute COVID-19 Surveillance Team
EPI_ISL_718911	Lighthouse Lab in Alderley Park	Wellcome Sanger Institute for the COVID-19 Genomics UK (COG-UK) Consortium	Jacquelyn Wynn, Mairead Hyland, The Lighthouse Lab in Alderley Park and Alex Alderton, Roberto Amato, Sonia Goncalves, Ewan Harrison, David K. Jackson, Ian Johnston, Dominic Kwiatkowski, Cordelia Langford, John Sillitoe on behalf of the Wellcome Sanger Institute COVID-19 Surveillance Team
EPI_ISL_718912, EPI_ISL_718913	Lighthouse Lab in Cambridge	Wellcome Sanger Institute for the COVID-19 Genomics UK (COG-UK) Consortium	Rob Howes, The Lighthouse Lab in Cambridge and Alex Alderton, Roberto Amato, Sonia Goncalves, Ewan Harrison, David K. Jackson, Ian Johnston, Dominic Kwiatkowski, Cordelia Langford, John Sillitoe on behalf of the Wellcome Sanger Institute COVID-19 Surveillance Team
EPI_ISL_718914, EPI_ISL_718915	Lighthouse Lab in Alderley Park	Wellcome Sanger Institute for the COVID-19 Genomics UK (COG-UK) Consortium	Jacquelyn Wynn, Mairead Hyland, The Lighthouse Lab in Alderley Park and Alex Alderton, Roberto Amato, Sonia Goncalves, Ewan Harrison, David K. Jackson, Ian Johnston, Dominic Kwiatkowski, Cordelia Langford, John Sillitoe on behalf of the Wellcome Sanger Institute COVID-19 Surveillance Team
EPI_ISL_718916	Lighthouse Lab in Cambridge	Wellcome Sanger Institute for the COVID-19 Genomics UK (COG-UK) Consortium	Rob Howes, The Lighthouse Lab in Cambridge and Alex Alderton, Roberto Amato, Sonia Goncalves, Ewan Harrison, David K. Jackson, Ian Johnston, Dominic Kwiatkowski, Cordelia Langford, John Sillitoe on behalf of the Wellcome Sanger Institute COVID-19 Surveillance Team
EPI_ISL_718917	Lighthouse Lab in Alderley Park	Wellcome Sanger Institute for the COVID-19 Genomics UK (COG-UK) Consortium	Jacquelyn Wynn, Mairead Hyland, The Lighthouse Lab in Alderley Park and Alex Alderton, Roberto Amato, Sonia Goncalves, Ewan Harrison, David K. Jackson, Ian Johnston, Dominic Kwiatkowski, Cordelia Langford, John Sillitoe on behalf of the Wellcome Sanger Institute COVID-19 Surveillance Team
EPI_ISL_718918	Lighthouse Lab in Cambridge	Wellcome Sanger Institute for the COVID-19 Genomics UK (COG-UK) Consortium	Rob Howes, The Lighthouse Lab in Cambridge and Alex Alderton, Roberto Amato, Sonia Goncalves, Ewan Harrison, David K. Jackson, Ian Johnston, Dominic Kwiatkowski, Cordelia Langford, John Sillitoe on behalf of the Wellcome Sanger Institute COVID-19 Surveillance Team
EPI_ISL_718919	Lighthouse Lab in Alderley Park	Wellcome Sanger Institute for the COVID-19 Genomics UK (COG-UK) Consortium	Jacquelyn Wynn, Mairead Hyland, The Lighthouse Lab in Alderley Park and Alex Alderton, Roberto Amato, Sonia Goncalves, Ewan Harrison, David K. Jackson, Ian Johnston, Dominic Kwiatkowski, Cordelia Langford, John Sillitoe on behalf of the Wellcome Sanger Institute COVID-19 Surveillance Team
EPI_ISL_718920, EPI_ISL_718921	Lighthouse Lab in Cambridge	Wellcome Sanger Institute for the COVID-19 Genomics UK (COG-UK) Consortium	Rob Howes, The Lighthouse Lab in Cambridge and Alex Alderton, Roberto Amato, Sonia Goncalves, Ewan Harrison, David K. Jackson, Ian Johnston, Dominic Kwiatkowski, Cordelia Langford, John Sillitoe on behalf of the Wellcome Sanger Institute COVID-19 Surveillance Team
EPI_ISL_718922, EPI_ISL_718923	Lighthouse Lab in Alderley Park	Wellcome Sanger Institute for the COVID-19 Genomics UK (COG-UK) Consortium	Jacquelyn Wynn, Mairead Hyland, The Lighthouse Lab in Alderley Park and Alex Alderton, Roberto Amato, Sonia Goncalves, Ewan Harrison, David K. Jackson, Ian Johnston, Dominic Kwiatkowski, Cordelia Langford, John Sillitoe on behalf of the Wellcome Sanger Institute COVID-19 Surveillance Team
EPI_ISL_718924, EPI_ISL_718925	Lighthouse Lab in Cambridge	Wellcome Sanger Institute for the COVID-19 Genomics UK (COG-UK) Consortium	Rob Howes, The Lighthouse Lab in Cambridge and Alex Alderton, Roberto Amato, Sonia Goncalves, Ewan Harrison, David K. Jackson, Ian Johnston, Dominic Kwiatkowski, Cordelia Langford, John Sillitoe on behalf of the Wellcome Sanger Institute COVID-19 Surveillance Team
EPI_ISL_718926	Lighthouse Lab in Alderley Park	Wellcome Sanger Institute for the COVID-19 Genomics UK (COG-UK) Consortium	Jacquelyn Wynn, Mairead Hyland, The Lighthouse Lab in Alderley Park and Alex Alderton, Roberto Amato, Sonia Goncalves, Ewan Harrison, David K. Jackson, Ian Johnston, Dominic Kwiatkowski, Cordelia Langford, John Sillitoe on behalf of the Wellcome Sanger Institute COVID-19 Surveillance Team
EPI_ISL_718927, EPI_ISL_718928	Lighthouse Lab in Cambridge	Wellcome Sanger Institute for the COVID-19 Genomics UK (COG-UK) Consortium	Rob Howes, The Lighthouse Lab in Cambridge and Alex Alderton, Roberto Amato, Sonia Goncalves, Ewan Harrison, David K. Jackson, Ian Johnston, Dominic Kwiatkowski, Cordelia Langford, John Sillitoe on behalf of the Wellcome Sanger Institute COVID-19 Surveillance Team
EPI_ISL_718929, EPI_ISL_718930	Lighthouse Lab in Alderley Park	Wellcome Sanger Institute for the COVID-19 Genomics UK (COG-UK) Consortium	Jacquelyn Wynn, Mairead Hyland, The Lighthouse Lab in Alderley Park and Alex Alderton, Roberto Amato, Sonia Goncalves, Ewan Harrison, David K. Jackson, Ian Johnston, Dominic Kwiatkowski, Cordelia Langford, John Sillitoe on behalf of the Wellcome Sanger Institute COVID-19 Surveillance Team
EPI_ISL_718931	Lighthouse Lab in Cambridge	Wellcome Sanger Institute for the COVID-19 Genomics UK (COG-UK) Consortium	Rob Howes, The Lighthouse Lab in Cambridge and Alex Alderton, Roberto Amato, Sonia Goncalves, Ewan Harrison, David K. Jackson, Ian Johnston, Dominic Kwiatkowski, Cordelia Langford, John Sillitoe on behalf of the Wellcome Sanger Institute COVID-19 Surveillance Team
EPI_ISL_718932	Lighthouse Lab in Alderley Park	Wellcome Sanger Institute for the COVID-19 Genomics UK (COG-UK) Consortium	Jacquelyn Wynn, Mairead Hyland, The Lighthouse Lab in Alderley Park and Alex Alderton, Roberto Amato, Sonia Goncalves, Ewan Harrison, David K. Jackson, Ian Johnston, Dominic Kwiatkowski, Cordelia Langford, John Sillitoe on behalf of the Wellcome Sanger Institute COVID-19 Surveillance Team
EPI_ISL_718933, EPI_ISL_718934	Lighthouse Lab in Cambridge	Wellcome Sanger Institute for the COVID-19 Genomics UK (COG-UK) Consortium	Rob Howes, The Lighthouse Lab in Cambridge and Alex Alderton, Roberto Amato, Sonia Goncalves, Ewan Harrison, David K. Jackson, Ian Johnston, Dominic Kwiatkowski, Cordelia Langford, John Sillitoe on behalf of the Wellcome Sanger Institute COVID-19 Surveillance Team
EPI_ISL_718935, EPI_ISL_718936	Lighthouse Lab in Alderley Park	Wellcome Sanger Institute for the COVID-19 Genomics UK (COG-UK) Consortium	Jacquelyn Wynn, Mairead Hyland, The Lighthouse Lab in Alderley Park and Alex Alderton, Roberto Amato, Sonia Goncalves, Ewan Harrison, David K. Jackson, Ian Johnston, Dominic Kwiatkowski, Cordelia Langford, John Sillitoe on behalf of the Wellcome Sanger Institute COVID-19 Surveillance Team
EPI_ISL_718937, EPI_ISL_718938	Lighthouse Lab in Cambridge	Wellcome Sanger Institute for the COVID-19 Genomics UK (COG-UK) Consortium	Rob Howes, The Lighthouse Lab in Cambridge and Alex Alderton, Roberto Amato, Sonia Goncalves, Ewan Harrison, David K. Jackson, Ian Johnston, Dominic Kwiatkowski, Cordelia Langford, John Sillitoe on behalf of the Wellcome Sanger Institute COVID-19 Surveillance Team
EPI_ISL_718939	Lighthouse Lab in Alderley Park	Wellcome Sanger Institute for the COVID-19 Genomics UK (COG-UK) Consortium	Jacquelyn Wynn, Mairead Hyland, The Lighthouse Lab in Alderley Park and Alex Alderton, Roberto Amato, Sonia Goncalves, Ewan Harrison, David K. Jackson, Ian Johnston, Dominic Kwiatkowski, Cordelia Langford, John Sillitoe on behalf of the Wellcome Sanger Institute COVID-19 Surveillance Team
EPI_ISL_718940, EPI_ISL_718941,	Lighthouse Lab in Cambridge	Wellcome Sanger Institute for the COVID-19 Genomics UK	Rob Howes, The Lighthouse Lab in Cambridge and Alex Alderton, Roberto Amato, Sonia Goncalves, Ewan Harrison, David K. Jackson, Ian Johnston,

EPI_ISL_718942		(COG-UK) Consortium	Dominic Kwiatkowski, Cordelia Langford, John Sillitoe on behalf of the Wellcome Sanger Institute COVID-19 Surveillance Team
EPI_ISL_718943	Lighthouse Lab in Alderley Park	Wellcome Sanger Institute for the COVID-19 Genomics UK (COG-UK) Consortium	Jacquelyn Wynn, Mairead Hyland, The Lighthouse Lab in Alderley Park and Alex Alderton, Roberto Amato, Sonia Goncalves, Ewan Harrison, David K. Jackson, Ian Johnston, Dominic Kwiatkowski, Cordelia Langford, John Sillitoe on behalf of the Wellcome Sanger Institute COVID-19 Surveillance Team
EPI_ISL_718944	Lighthouse Lab in Cambridge	Wellcome Sanger Institute for the COVID-19 Genomics UK (COG-UK) Consortium	Rob Howes, The Lighthouse Lab in Cambridge and Alex Alderton, Roberto Amato, Sonia Goncalves, Ewan Harrison, David K. Jackson, Ian Johnston, Dominic Kwiatkowski, Cordelia Langford, John Sillitoe on behalf of the Wellcome Sanger Institute COVID-19 Surveillance Team
EPI_ISL_718945	Lighthouse Lab in Alderley Park	Wellcome Sanger Institute for the COVID-19 Genomics UK (COG-UK) Consortium	Jacquelyn Wynn, Mairead Hyland, The Lighthouse Lab in Alderley Park and Alex Alderton, Roberto Amato, Sonia Goncalves, Ewan Harrison, David K. Jackson, Ian Johnston, Dominic Kwiatkowski, Cordelia Langford, John Sillitoe on behalf of the Wellcome Sanger Institute COVID-19 Surveillance Team
EPI_ISL_718946, EPI_ISL_718947, EPI_ISL_718948, EPI_ISL_718949	Lighthouse Lab in Cambridge	Wellcome Sanger Institute for the COVID-19 Genomics UK (COG-UK) Consortium	Rob Howes, The Lighthouse Lab in Cambridge and Alex Alderton, Roberto Amato, Sonia Goncalves, Ewan Harrison, David K. Jackson, Ian Johnston, Dominic Kwiatkowski, Cordelia Langford, John Sillitoe on behalf of the Wellcome Sanger Institute COVID-19 Surveillance Team
EPI_ISL_718950	Lighthouse Lab in Alderley Park	Wellcome Sanger Institute for the COVID-19 Genomics UK (COG-UK) Consortium	Jacquelyn Wynn, Mairead Hyland, The Lighthouse Lab in Alderley Park and Alex Alderton, Roberto Amato, Sonia Goncalves, Ewan Harrison, David K. Jackson, Ian Johnston, Dominic Kwiatkowski, Cordelia Langford, John Sillitoe on behalf of the Wellcome Sanger Institute COVID-19 Surveillance Team
EPI_ISL_718951	Lighthouse Lab in Cambridge	Wellcome Sanger Institute for the COVID-19 Genomics UK (COG-UK) Consortium	Rob Howes, The Lighthouse Lab in Cambridge and Alex Alderton, Roberto Amato, Sonia Goncalves, Ewan Harrison, David K. Jackson, Ian Johnston, Dominic Kwiatkowski, Cordelia Langford, John Sillitoe on behalf of the Wellcome Sanger Institute COVID-19 Surveillance Team
EPI_ISL_718952, EPI_ISL_718953	Lighthouse Lab in Alderley Park	Wellcome Sanger Institute for the COVID-19 Genomics UK (COG-UK) Consortium	Jacquelyn Wynn, Mairead Hyland, The Lighthouse Lab in Alderley Park and Alex Alderton, Roberto Amato, Sonia Goncalves, Ewan Harrison, David K. Jackson, Ian Johnston, Dominic Kwiatkowski, Cordelia Langford, John Sillitoe on behalf of the Wellcome Sanger Institute COVID-19 Surveillance Team
EPI_ISL_718954	Lighthouse Lab in Cambridge	Wellcome Sanger Institute for the COVID-19 Genomics UK (COG-UK) Consortium	Rob Howes, The Lighthouse Lab in Cambridge and Alex Alderton, Roberto Amato, Sonia Goncalves, Ewan Harrison, David K. Jackson, Ian Johnston, Dominic Kwiatkowski, Cordelia Langford, John Sillitoe on behalf of the Wellcome Sanger Institute COVID-19 Surveillance Team
EPI_ISL_718955	Lighthouse Lab in Alderley Park	Wellcome Sanger Institute for the COVID-19 Genomics UK (COG-UK) Consortium	Jacquelyn Wynn, Mairead Hyland, The Lighthouse Lab in Alderley Park and Alex Alderton, Roberto Amato, Sonia Goncalves, Ewan Harrison, David K. Jackson, Ian Johnston, Dominic Kwiatkowski, Cordelia Langford, John Sillitoe on behalf of the Wellcome Sanger Institute COVID-19 Surveillance Team
EPI_ISL_718956	Lighthouse Lab in Cambridge	Wellcome Sanger Institute for the COVID-19 Genomics UK (COG-UK) Consortium	Rob Howes, The Lighthouse Lab in Cambridge and Alex Alderton, Roberto Amato, Sonia Goncalves, Ewan Harrison, David K. Jackson, Ian Johnston, Dominic Kwiatkowski, Cordelia Langford, John Sillitoe on behalf of the Wellcome Sanger Institute COVID-19 Surveillance Team
EPI_ISL_718957	Lighthouse Lab in Alderley Park	Wellcome Sanger Institute for the COVID-19 Genomics UK (COG-UK) Consortium	Jacquelyn Wynn, Mairead Hyland, The Lighthouse Lab in Alderley Park and Alex Alderton, Roberto Amato, Sonia Goncalves, Ewan Harrison, David K. Jackson, Ian Johnston, Dominic Kwiatkowski, Cordelia Langford, John Sillitoe on behalf of the Wellcome Sanger Institute COVID-19 Surveillance Team
EPI_ISL_718958	Lighthouse Lab in Cambridge	Wellcome Sanger Institute for the COVID-19 Genomics UK (COG-UK) Consortium	Rob Howes, The Lighthouse Lab in Cambridge and Alex Alderton, Roberto Amato, Sonia Goncalves, Ewan Harrison, David K. Jackson, Ian Johnston, Dominic Kwiatkowski, Cordelia Langford, John Sillitoe on behalf of the Wellcome Sanger Institute COVID-19 Surveillance Team
EPI_ISL_718959	Lighthouse Lab in Alderley Park	Wellcome Sanger Institute for the COVID-19 Genomics UK (COG-UK) Consortium	Jacquelyn Wynn, Mairead Hyland, The Lighthouse Lab in Alderley Park and Alex Alderton, Roberto Amato, Sonia Goncalves, Ewan Harrison, David K. Jackson, Ian Johnston, Dominic Kwiatkowski, Cordelia Langford, John Sillitoe on behalf of the Wellcome Sanger Institute COVID-19 Surveillance Team
EPI_ISL_718960, EPI_ISL_718961	Lighthouse Lab in Cambridge	Wellcome Sanger Institute for the COVID-19 Genomics UK (COG-UK) Consortium	Rob Howes, The Lighthouse Lab in Cambridge and Alex Alderton, Roberto Amato, Sonia Goncalves, Ewan Harrison, David K. Jackson, Ian Johnston, Dominic Kwiatkowski, Cordelia Langford, John Sillitoe on behalf of the Wellcome Sanger Institute COVID-19 Surveillance Team
EPI_ISL_718962, EPI_ISL_718963, EPI_ISL_718964	Lighthouse Lab in Alderley Park	Wellcome Sanger Institute for the COVID-19 Genomics UK (COG-UK) Consortium	Jacquelyn Wynn, Mairead Hyland, The Lighthouse Lab in Alderley Park and Alex Alderton, Roberto Amato, Sonia Goncalves, Ewan Harrison, David K. Jackson, Ian Johnston, Dominic Kwiatkowski, Cordelia Langford, John Sillitoe on behalf of the Wellcome Sanger Institute COVID-19 Surveillance Team
EPI_ISL_718965	Lighthouse Lab in Cambridge	Wellcome Sanger Institute for the COVID-19 Genomics UK (COG-UK) Consortium	Rob Howes, The Lighthouse Lab in Cambridge and Alex Alderton, Roberto Amato, Sonia Goncalves, Ewan Harrison, David K. Jackson, Ian Johnston, Dominic Kwiatkowski, Cordelia Langford, John Sillitoe on behalf of the Wellcome Sanger Institute COVID-19 Surveillance Team
EPI_ISL_718966, EPI_ISL_718967	Lighthouse Lab in Alderley Park	Wellcome Sanger Institute for the COVID-19 Genomics UK (COG-UK) Consortium	Jacquelyn Wynn, Mairead Hyland, The Lighthouse Lab in Alderley Park and Alex Alderton, Roberto Amato, Sonia Goncalves, Ewan Harrison, David K. Jackson, Ian Johnston, Dominic Kwiatkowski, Cordelia Langford, John Sillitoe on behalf of the Wellcome Sanger Institute COVID-19 Surveillance Team
EPI_ISL_718968, EPI_ISL_718969	Lighthouse Lab in Cambridge	Wellcome Sanger Institute for the COVID-19 Genomics UK (COG-UK) Consortium	Rob Howes, The Lighthouse Lab in Cambridge and Alex Alderton, Roberto Amato, Sonia Goncalves, Ewan Harrison, David K. Jackson, Ian Johnston, Dominic Kwiatkowski, Cordelia Langford, John Sillitoe on behalf of the Wellcome Sanger Institute COVID-19 Surveillance Team
EPI_ISL_718970	Lighthouse Lab in Alderley Park	Wellcome Sanger Institute for the COVID-19 Genomics UK (COG-UK) Consortium	Jacquelyn Wynn, Mairead Hyland, The Lighthouse Lab in Alderley Park and Alex Alderton, Roberto Amato, Sonia Goncalves, Ewan Harrison, David K. Jackson, Ian Johnston, Dominic Kwiatkowski, Cordelia Langford, John Sillitoe on behalf of the Wellcome Sanger Institute COVID-19 Surveillance Team
EPI_ISL_718971, EPI_ISL_718972, EPI_ISL_718973	Lighthouse Lab in Cambridge	Wellcome Sanger Institute for the COVID-19 Genomics UK (COG-UK) Consortium	Rob Howes, The Lighthouse Lab in Cambridge and Alex Alderton, Roberto Amato, Sonia Goncalves, Ewan Harrison, David K. Jackson, Ian Johnston, Dominic Kwiatkowski, Cordelia Langford, John Sillitoe on behalf of the Wellcome Sanger Institute COVID-19 Surveillance Team
EPI_ISL_718974, EPI_ISL_718975, EPI_ISL_718976, EPI_ISL_718977, EPI_ISL_718978, EPI_ISL_718979	Lighthouse Lab in Alderley Park	Wellcome Sanger Institute for the COVID-19 Genomics UK (COG-UK) Consortium	Jacquelyn Wynn, Mairead Hyland, The Lighthouse Lab in Alderley Park and Alex Alderton, Roberto Amato, Sonia Goncalves, Ewan Harrison, David K. Jackson, Ian Johnston, Dominic Kwiatkowski, Cordelia Langford, John Sillitoe on behalf of the Wellcome Sanger Institute COVID-19 Surveillance Team

EPI_ISL_718980, EPI_ISL_718981, EPI_ISL_718982, EPI_ISL_718983, EPI_ISL_718984, EPI_ISL_718985, EPI_ISL_718986, EPI_ISL_718987, EPI_ISL_718988, EPI_ISL_718989, EPI_ISL_718990, EPI_ISL_718991, EPI_ISL_718992, EPI_ISL_718993, EPI_ISL_718994, EPI_ISL_718995, EPI_ISL_718996, EPI_ISL_718997, EPI_ISL_718998, EPI_ISL_718999, EPI_ISL_719000, EPI_ISL_719001, EPI_ISL_719002, EPI_ISL_719003, EPI_ISL_719004, EPI_ISL_719005, EPI_ISL_719006, EPI_ISL_719007, EPI_ISL_719008, EPI_ISL_719009, EPI_ISL_719010, EPI_ISL_719011, EPI_ISL_719012, EPI_ISL_719013, EPI_ISL_719014, EPI_ISL_719015, EPI_ISL_719016, EPI_ISL_719017, EPI_ISL_719018, EPI_ISL_719019, EPI_ISL_719020, EPI_ISL_719021, EPI_ISL_719022, EPI_ISL_719023, EPI_ISL_719024, EPI_ISL_719025, EPI_ISL_719026, EPI_ISL_719027, EPI_ISL_719028, EPI_ISL_719029, EPI_ISL_719030, EPI_ISL_719031, EPI_ISL_719032, EPI_ISL_719033, EPI_ISL_719034, EPI_ISL_719035, EPI_ISL_719036, EPI_ISL_719038, EPI_ISL_719039, EPI_ISL_719040, EPI_ISL_719041, EPI_ISL_719043, EPI_ISL_719044, EPI_ISL_719045, EPI_ISL_719046, EPI_ISL_719047, EPI_ISL_719048, EPI_ISL_719049, EPI_ISL_719050, EPI_ISL_719051, EPI_ISL_719052, EPI_ISL_719053, EPI_ISL_719054, EPI_ISL_719055, EPI_ISL_719056, EPI_ISL_719057, EPI_ISL_719058, EPI_ISL_719059, EPI_ISL_719060, EPI_ISL_719061, EPI_ISL_719062, EPI_ISL_719063, EPI_ISL_719064, EPI_ISL_719065, EPI_ISL_719066, EPI_ISL_719067, EPI_ISL_719068, EPI_ISL_719069, EPI_ISL_719070, EPI_ISL_719071, EPI_ISL_719072, EPI_ISL_719073, EPI_ISL_719074, EPI_ISL_719075, EPI_ISL_719076, EPI_ISL_719077, EPI_ISL_719078, EPI_ISL_719079, EPI_ISL_719080, EPI_ISL_719081, EPI_ISL_719082, EPI_ISL_719083, EPI_ISL_719084, EPI_ISL_719085, EPI_ISL_719086, EPI_ISL_719087, EPI_ISL_719088, EPI_ISL_719089, EPI_ISL_719090, EPI_ISL_719091, EPI_ISL_719092, EPI_ISL_719093, EPI_ISL_719094, EPI_ISL_719095, EPI_ISL_719096, EPI_ISL_719097, EPI_ISL_719098, EPI_ISL_719099, EPI_ISL_719100, EPI_ISL_719101, EPI_ISL_719102, EPI_ISL_719103, EPI_ISL_719104, EPI_ISL_719105, EPI_ISL_719106, EPI_ISL_719107, EPI_ISL_719108, EPI_ISL_719109, EPI_ISL_719110, EPI_ISL_719111, EPI_ISL_719112, EPI_ISL_719113, EPI_ISL_719114, EPI_ISL_719115, EPI_ISL_719116, EPI_ISL_719117, EPI_ISL_719118, EPI_ISL_719119, EPI_ISL_719120, EPI_ISL_719121, EPI_ISL_719122, EPI_ISL_719123, EPI_ISL_719124, EPI_ISL_719125, EPI_ISL_719126, EPI_ISL_719127, EPI_ISL_719128, EPI_ISL_719129, EPI_ISL_719130, EPI_ISL_719131, EPI_ISL_719132, EPI_ISL_719133, EPI_ISL_719134, EPI_ISL_719135, EPI_ISL_719136, EPI_ISL_719137, EPI_ISL_719138, EPI_ISL_719139, EPI_ISL_719140, EPI_ISL_719141, EPI_ISL_719142, EPI_ISL_719143, EPI_ISL_719145, EPI_ISL_719146, EPI_ISL_719147, EPI_ISL_719148, EPI_ISL_719149, EPI_ISL_719150, EPI_ISL_719151, EPI_ISL_719152, EPI_ISL_719153, EPI_ISL_719154, EPI_ISL_719155, EPI_ISL_719156, EPI_ISL_719157, EPI_ISL_719158, EPI_ISL_719159, EPI_ISL_719160, EPI_ISL_719161, EPI_ISL_719162, EPI_ISL_719163, EPI_ISL_719164, EPI_ISL_719165, EPI_ISL_719166, EPI_ISL_719167, EPI_ISL_719168, EPI_ISL_719169, EPI_ISL_719170, EPI_ISL_719171, EPI_ISL_719172, EPI_ISL_719173, EPI_ISL_719174, EPI_ISL_719175, EPI_ISL_719176, EPI_ISL_719177, EPI_ISL_719178, EPI_ISL_719179, EPI_ISL_719180, EPI_ISL_719181, EPI_ISL_719182, EPI_ISL_719183, EPI_ISL_719184, EPI_ISL_719185, EPI_ISL_719186, EPI_ISL_719187, EPI_ISL_719188, EPI_ISL_719189, EPI_ISL_719190, EPI_ISL_719191, EPI_ISL_719192, EPI_ISL_719193, EPI_ISL_719194, EPI_ISL_719195, EPI_ISL_719196, EPI_ISL_719197, EPI_ISL_719198, EPI_ISL_719199, EPI_ISL_719200, EPI_ISL_719201, EPI_ISL_719202, EPI_ISL_719203, EPI_ISL_719204, EPI_ISL_719205, EPI_ISL_719206, EPI_ISL_719207, EPI_ISL_719208, EPI_ISL_719209, EPI_ISL_719210, EPI_ISL_719211, EPI_ISL_719212, EPI_ISL_719213, EPI_ISL_719214, EPI_ISL_719215, EPI_ISL_719216, EPI_ISL_719217, EPI_ISL_719218, EPI_ISL_719219, EPI_ISL_719220, EPI_ISL_719221, EPI_ISL_719222, EPI_ISL_719223, EPI_ISL_719224, EPI_ISL_719225, EPI_ISL_719226, EPI_ISL_719227, EPI_ISL_719228, EPI_ISL_719229, EPI_ISL_719230, EPI_ISL_719231, EPI_ISL_719232, EPI_ISL_719233, EPI_ISL_719234, EPI_ISL_719235, EPI_ISL_719236, EPI_ISL_719237, EPI_ISL_719238, EPI_ISL_719239, EPI_ISL_719240, EPI_ISL_719241, EPI_ISL_719242, EPI_ISL_719243, EPI_ISL_719244, EPI_ISL_719245, EPI_ISL_719246, EPI_ISL_719247, EPI_ISL_719248, EPI_ISL_719249, EPI_ISL_719250, EPI_ISL_719251, EPI_ISL_719252, EPI_ISL_719253, EPI_ISL_719254, EPI_ISL_719255, EPI_ISL_719256, EPI_ISL_719257, EPI_ISL_719258, EPI_ISL_719259, EPI_ISL_719260, EPI_ISL_719261, EPI_ISL_719262, EPI_ISL_719263, EPI_ISL_719264, EPI_ISL_719265, EPI_ISL_719266, EPI_ISL_719267, EPI_ISL_719268, EPI_ISL_719269, EPI_ISL_719270, EPI_ISL_719271, EPI_ISL_719272, EPI_ISL_719273, EPI_ISL_719274, EPI_ISL_719275, EPI_ISL_719276, EPI_ISL_719277, EPI_ISL_719278, EPI_ISL_719279, EPI_ISL_719280, EPI_ISL_719281, EPI_ISL_719282, EPI_ISL_719283, EPI_ISL_719284, EPI_ISL_719285, EPI_ISL_719286, EPI_ISL_719287, EPI_ISL_719288, EPI_ISL_719289, EPI_ISL_719290, EPI_ISL_719291, EPI_ISL_719292, EPI_ISL_719293, EPI_ISL_719294, EPI_ISL_719295, EPI_ISL_719296, EPI_ISL_719297, EPI_ISL_719298, EPI_ISL_719299, EPI_ISL_719300, EPI_ISL_719301, EPI_ISL_719302, EPI_ISL_719303, EPI_ISL_719304, EPI_ISL_719305, EPI_ISL_719306, EPI_ISL_719307, EPI_ISL_719308, EPI_ISL_719309, EPI_ISL_719310, EPI_ISL_719312, EPI_ISL_719313, EPI_ISL_719314, EPI_ISL_719315, EPI_ISL_719316, EPI_ISL_719317, EPI_ISL_719318, EPI_ISL_719319, EPI_ISL_719320, EPI_ISL_719321, EPI_ISL_719322, EPI_ISL_719323, EPI_ISL_719324, EPI_ISL_719325, EPI_ISL_719326, EPI_ISL_719327, EPI_ISL_719328, EPI_ISL_719329, EPI_ISL_719330, EPI_ISL_719331, EPI_ISL_719332, EPI_ISL_719333, EPI_ISL_719334, EPI_ISL_719335, EPI_ISL_719336, EPI_ISL_719337, EPI_ISL_719338, EPI_ISL_719339, EPI_ISL_719340, EPI_ISL_719341, EPI_ISL_719343, EPI_ISL_719344, EPI_ISL_719346, EPI_ISL_719348, EPI_ISL_719349, EPI_ISL_719350, EPI_ISL_719351, EPI_ISL_719352, EPI_ISL_719353, EPI_ISL_719354, EPI_ISL_719355, EPI_ISL_719356, EPI_ISL_719357, EPI_ISL_719358, EPI_ISL_719359, EPI_ISL_719360, EPI_ISL_719361, EPI_ISL_719362, EPI_ISL_719363, EPI_ISL_719365, EPI_ISL_719366, EPI_ISL_719367, EPI_ISL_719368, EPI_ISL_719369, EPI_ISL_719370, EPI_ISL_719371, EPI_ISL_719372, EPI_ISL_719373, EPI_ISL_719374, EPI_ISL_719375, EPI_ISL_719376, EPI_ISL_719377, EPI_ISL_719378, EPI_ISL_719379, EPI_ISL_719380, EPI_ISL_719381, EPI_ISL_719382, EPI_ISL_719383, EPI_ISL_719384, EPI_ISL_719385, EPI_ISL_719386, EPI_ISL_719387, EPI_ISL_719388, EPI_ISL_719389, EPI_ISL_719390, EPI_ISL_719391, EPI_ISL_719392, EPI_ISL_719393, EPI_ISL_719394, EPI_ISL_719395, EPI_ISL_719396, EPI_ISL_719397, EPI_ISL_719398, EPI_ISL_719399, EPI_ISL_719400, EPI_ISL_719401, EPI_ISL_719402, EPI_ISL_719403, EPI_ISL_719404, EPI_ISL_719406, EPI_ISL_719407, EPI_ISL_719408, EPI_ISL_719409, EPI_ISL_719410, EPI_ISL_719411, EPI_ISL_719412, EPI_ISL_719413, EPI_ISL_719414, EPI_ISL_719415, EPI_ISL_719416, EPI_ISL_719417, EPI_ISL_719418, EPI_ISL_719419, EPI_ISL_719420, EPI_ISL_719421, EPI_ISL_719422, EPI_ISL_719423, EPI_ISL_719424, EPI_ISL_719425, EPI_ISL_719426, EPI_ISL_719427, EPI_ISL_719428, EPI_ISL_719429, EPI_ISL_719430, EPI_ISL_719431, EPI_ISL_719432, EPI_ISL_719433, EPI_ISL_719434, EPI_ISL_719435, EPI_ISL_719436, EPI_ISL_719437, EPI_ISL_719438, EPI_ISL_719439, EPI_ISL_719440, EPI_ISL_719441, EPI_ISL_719442, EPI_ISL_719443, EPI_ISL_719444, EPI_ISL_719445, EPI_ISL_719446, EPI_ISL_719447, EPI_ISL_719448, EPI_ISL_719449, EPI_ISL_719450, EPI_ISL_719451, EPI_ISL_719452, EPI_ISL_719453, EPI_ISL_719454, EPI_ISL_719455, EPI_ISL_719456, EPI_ISL_719457, EPI_ISL_719458, EPI_ISL_719459, EPI_ISL_719460, EPI_ISL_719461, EPI_ISL_719462, EPI_ISL_719463, EPI_ISL_719464, EPI_ISL_719465, EPI_ISL_719466, EPI_ISL_719467, EPI_ISL_719468, EPI_ISL_719469, EPI_ISL_719470, EPI_ISL_719471, EPI_ISL_719472, EPI_ISL_719473, EPI_ISL_719474, EPI_ISL_719475, EPI_ISL_719476, EPI_ISL_719477, EPI_ISL_719478, EPI_ISL_719479, EPI_ISL_719480, EPI_ISL_719481, EPI_ISL_719482, EPI_ISL_719483, EPI_ISL_719484, EPI_ISL_719485, EPI_ISL_719486, EPI_ISL_719487, EPI_ISL_719488, EPI_ISL_719489, EPI_ISL_719490, EPI_ISL_719491, EPI_ISL_719493, EPI_ISL_719494, EPI_ISL_719495, EPI_ISL_719496, EPI_ISL_719497, EPI_ISL_719498, EPI_ISL_719500, EPI_ISL_719501, EPI_ISL_719502, EPI_ISL_719503, EPI_ISL_719504, EPI_ISL_719505, EPI_ISL_719506, EPI_ISL_719507, EPI_ISL_719508, EPI_ISL_719509, EPI_ISL_719510, EPI_ISL_719511, EPI_ISL_719512, EPI_ISL_719513, EPI_ISL_719514, EPI_ISL_719515, EPI_ISL_719516, EPI_ISL_719517, EPI_ISL_719518, EPI_ISL_719519, EPI_ISL_719520, EPI_ISL_719522, EPI_ISL_719523, EPI_ISL_719524, EPI_ISL_719525, EPI_ISL_719526, EPI_ISL_719527, EPI_ISL_719528, EPI_ISL_719529, EPI_ISL_719530, EPI_ISL_719531, EPI_ISL_719532, EPI_ISL_719533, EPI_ISL_719534, EPI_ISL_719535, EPI_ISL_719537, EPI_ISL_719538, EPI_ISL_719539, EPI_ISL_719540, EPI_ISL_719541, EPI_ISL_719542, EPI_ISL_719543, EPI_ISL_719544, EPI_ISL_719545, EPI_ISL_719546, EPI_ISL_719547, EPI_ISL_719548, EPI_ISL_719549, EPI_ISL_719550, EPI_ISL_719551, EPI_ISL_719552, EPI_ISL_719553, EPI_ISL_719554, EPI_ISL_719555, EPI_ISL_719556, EPI_ISL_719557, EPI_ISL_719558, EPI_ISL_719559, EPI_ISL_719560, EPI_ISL_719561, EPI_ISL_719562, EPI_ISL_719563, EPI_ISL_719564, EPI_ISL_719565, EPI_ISL_719566, EPI_ISL_719567, EPI_ISL_719568,

EPI_ISL_719569, EPI_ISL_719570, EPI_ISL_719571, EPI_ISL_719572, EPI_ISL_719573, EPI_ISL_719574, EPI_ISL_719575, EPI_ISL_719576, EPI_ISL_719577, EPI_ISL_719578, EPI_ISL_719579, EPI_ISL_719580, EPI_ISL_719581, EPI_ISL_719582, EPI_ISL_719583, EPI_ISL_719584, EPI_ISL_719585, EPI_ISL_719586, EPI_ISL_719587, EPI_ISL_719588, EPI_ISL_719589, EPI_ISL_719590, EPI_ISL_719591, EPI_ISL_719592, EPI_ISL_719594, EPI_ISL_719595, EPI_ISL_719596, EPI_ISL_719597, EPI_ISL_719598, EPI_ISL_719599, EPI_ISL_719600, EPI_ISL_719601, EPI_ISL_719602, EPI_ISL_719603, EPI_ISL_719604			
see above	Lighthouse Lab in Cambridge	Wellcome Sanger Institute for the COVID-19 Genomics UK (COG-UK) Consortium	Rob Howes, The Lighthouse Lab in Cambridge and Alex Alderton, Roberto Amato, Sonia Goncalves, Ewan Harrison, David K. Jackson, Ian Johnston, Dominic Kwiatkowski, Cordelia Langford, John Sillitoe on behalf of the Wellcome Sanger Institute COVID-19 Surveillance Team
EPI_ISL_719605, EPI_ISL_719606, EPI_ISL_719607, EPI_ISL_719608, EPI_ISL_719609, EPI_ISL_719610, EPI_ISL_719611, EPI_ISL_719612, EPI_ISL_719613, EPI_ISL_719615, EPI_ISL_719616, EPI_ISL_719617, EPI_ISL_719618, EPI_ISL_719619, EPI_ISL_719620, EPI_ISL_719621, EPI_ISL_719622, EPI_ISL_719623, EPI_ISL_719624, EPI_ISL_719625, EPI_ISL_719626, EPI_ISL_719627, EPI_ISL_719628, EPI_ISL_719629, EPI_ISL_719630, EPI_ISL_719631, EPI_ISL_719632, EPI_ISL_719633, EPI_ISL_719634, EPI_ISL_719635, EPI_ISL_719636, EPI_ISL_719637, EPI_ISL_719640, EPI_ISL_719641, EPI_ISL_719642, EPI_ISL_719643, EPI_ISL_719644, EPI_ISL_719645, EPI_ISL_719646, EPI_ISL_719647, EPI_ISL_719648, EPI_ISL_719649, EPI_ISL_719650, EPI_ISL_719651, EPI_ISL_719652, EPI_ISL_719653, EPI_ISL_719654, EPI_ISL_719655, EPI_ISL_719656, EPI_ISL_719657, EPI_ISL_719658, EPI_ISL_719659, EPI_ISL_719660, EPI_ISL_719661, EPI_ISL_719662, EPI_ISL_719663, EPI_ISL_719664, EPI_ISL_719665, EPI_ISL_719666, EPI_ISL_719667, EPI_ISL_719668, EPI_ISL_719669, EPI_ISL_719670, EPI_ISL_719671, EPI_ISL_719672, EPI_ISL_719673, EPI_ISL_719674, EPI_ISL_719675, EPI_ISL_719677, EPI_ISL_719678, EPI_ISL_719679, EPI_ISL_719680, EPI_ISL_719681, EPI_ISL_719682, EPI_ISL_719683, EPI_ISL_719684, EPI_ISL_719685, EPI_ISL_719686, EPI_ISL_719687, EPI_ISL_719688, EPI_ISL_719689, EPI_ISL_719690, EPI_ISL_719691, EPI_ISL_719692, EPI_ISL_719693, EPI_ISL_719694, EPI_ISL_719695, EPI_ISL_719696, EPI_ISL_719697, EPI_ISL_719698, EPI_ISL_719699, EPI_ISL_719700, EPI_ISL_719701, EPI_ISL_719702, EPI_ISL_719703, EPI_ISL_719704, EPI_ISL_719705, EPI_ISL_719706, EPI_ISL_719707, EPI_ISL_719708, EPI_ISL_719709, EPI_ISL_719710, EPI_ISL_719711, EPI_ISL_719712, EPI_ISL_719713, EPI_ISL_719714, EPI_ISL_719715, EPI_ISL_719716, EPI_ISL_719717, EPI_ISL_719719, EPI_ISL_719720, EPI_ISL_719721, EPI_ISL_719722, EPI_ISL_719723, EPI_ISL_719724, EPI_ISL_719725, EPI_ISL_719726, EPI_ISL_719727, EPI_ISL_719728, EPI_ISL_719729, EPI_ISL_719730, EPI_ISL_719731, EPI_ISL_719732, EPI_ISL_719733, EPI_ISL_719734, EPI_ISL_719735, EPI_ISL_719736, EPI_ISL_719737, EPI_ISL_719738, EPI_ISL_719739, EPI_ISL_719740, EPI_ISL_719742, EPI_ISL_719744, EPI_ISL_719745, EPI_ISL_719746, EPI_ISL_719747, EPI_ISL_719748, EPI_ISL_719749, EPI_ISL_719750, EPI_ISL_719751, EPI_ISL_719752, EPI_ISL_719753, EPI_ISL_719755, EPI_ISL_719756, EPI_ISL_719758, EPI_ISL_719759, EPI_ISL_719760, EPI_ISL_719761, EPI_ISL_719762, EPI_ISL_719763, EPI_ISL_719764, EPI_ISL_719765, EPI_ISL_719766, EPI_ISL_719768, EPI_ISL_719769, EPI_ISL_719770, EPI_ISL_719771, EPI_ISL_719772, EPI_ISL_719773, EPI_ISL_719774, EPI_ISL_719775, EPI_ISL_719776, EPI_ISL_719777, EPI_ISL_719778, EPI_ISL_719779, EPI_ISL_719780, EPI_ISL_719781, EPI_ISL_719782, EPI_ISL_719783, EPI_ISL_719784, EPI_ISL_719785, EPI_ISL_719786, EPI_ISL_719787, EPI_ISL_719788, EPI_ISL_719789, EPI_ISL_719790, EPI_ISL_719791, EPI_ISL_719792, EPI_ISL_719793, EPI_ISL_719795, EPI_ISL_719796, EPI_ISL_719797, EPI_ISL_719798, EPI_ISL_719799, EPI_ISL_719800, EPI_ISL_719801, EPI_ISL_719802, EPI_ISL_719803, EPI_ISL_719804, EPI_ISL_719805, EPI_ISL_719806, EPI_ISL_719807, EPI_ISL_719808, EPI_ISL_719809, EPI_ISL_719810, EPI_ISL_719811, EPI_ISL_719812, EPI_ISL_719813, EPI_ISL_719814, EPI_ISL_719815, EPI_ISL_719816, EPI_ISL_719817, EPI_ISL_719818, EPI_ISL_719819, EPI_ISL_719820, EPI_ISL_719821, EPI_ISL_719822, EPI_ISL_719823, EPI_ISL_719824, EPI_ISL_719825, EPI_ISL_719826, EPI_ISL_719827, EPI_ISL_719828, EPI_ISL_719829, EPI_ISL_719830, EPI_ISL_719831, EPI_ISL_719832, EPI_ISL_719833, EPI_ISL_719834, EPI_ISL_719835, EPI_ISL_719836, EPI_ISL_719839, EPI_ISL_719840, EPI_ISL_719841, EPI_ISL_719843, EPI_ISL_719844, EPI_ISL_719846, EPI_ISL_719847, EPI_ISL_719848, EPI_ISL_719849, EPI_ISL_719850, EPI_ISL_719851, EPI_ISL_719852, EPI_ISL_719853, EPI_ISL_719854, EPI_ISL_719855, EPI_ISL_719856, EPI_ISL_719857, EPI_ISL_719858, EPI_ISL_719859, EPI_ISL_719860, EPI_ISL_719861, EPI_ISL_719862, EPI_ISL_719863, EPI_ISL_719864, EPI_ISL_719865, EPI_ISL_719866, EPI_ISL_719867, EPI_ISL_719868, EPI_ISL_719870, EPI_ISL_719871, EPI_ISL_719872, EPI_ISL_719873, EPI_ISL_719874, EPI_ISL_719875, EPI_ISL_719876, EPI_ISL_719877, EPI_ISL_719878, EPI_ISL_719879, EPI_ISL_719880, EPI_ISL_719881, EPI_ISL_719883, EPI_ISL_719884, EPI_ISL_719885, EPI_ISL_719887, EPI_ISL_719888, EPI_ISL_719889, EPI_ISL_719890, EPI_ISL_719891, EPI_ISL_719892, EPI_ISL_719894, EPI_ISL_719895, EPI_ISL_719896, EPI_ISL_719897, EPI_ISL_719898, EPI_ISL_719899, EPI_ISL_719900, EPI_ISL_719901, EPI_ISL_719902, EPI_ISL_719903, EPI_ISL_719904, EPI_ISL_719905, EPI_ISL_719906, EPI_ISL_719907, EPI_ISL_719908, EPI_ISL_719909, EPI_ISL_719910, EPI_ISL_719911, EPI_ISL_719912, EPI_ISL_719913, EPI_ISL_719914, EPI_ISL_719915, EPI_ISL_719916, EPI_ISL_719917, EPI_ISL_719918, EPI_ISL_719919, EPI_ISL_719920, EPI_ISL_719921, EPI_ISL_719922, EPI_ISL_719923, EPI_ISL_719924, EPI_ISL_719925, EPI_ISL_719926, EPI_ISL_719927, EPI_ISL_719928, EPI_ISL_719929, EPI_ISL_719930, EPI_ISL_719931, EPI_ISL_719932, EPI_ISL_719933, EPI_ISL_719934, EPI_ISL_719935, EPI_ISL_719936, EPI_ISL_719937, EPI_ISL_719938, EPI_ISL_719939, EPI_ISL_719940, EPI_ISL_719941, EPI_ISL_719942, EPI_ISL_719943, EPI_ISL_719944, EPI_ISL_719945, EPI_ISL_719946, EPI_ISL_719947, EPI_ISL_719948, EPI_ISL_719950, EPI_ISL_719951, EPI_ISL_719953, EPI_ISL_719954, EPI_ISL_719956, EPI_ISL_719957, EPI_ISL_719958, EPI_ISL_719959, EPI_ISL_719960, EPI_ISL_719961, EPI_ISL_719962, EPI_ISL_719963, EPI_ISL_719964, EPI_ISL_719965, EPI_ISL_719966, EPI_ISL_719967, EPI_ISL_719968, EPI_ISL_719969, EPI_ISL_719970, EPI_ISL_719971, EPI_ISL_719972, EPI_ISL_719973, EPI_ISL_719974, EPI_ISL_719975, EPI_ISL_719976, EPI_ISL_719977, EPI_ISL_719978, EPI_ISL_719979, EPI_ISL_719980, EPI_ISL_719981, EPI_ISL_719982, EPI_ISL_719983, EPI_ISL_719984, EPI_ISL_719985, EPI_ISL_719986			
see above	Lighthouse Lab in Glasgow	Wellcome Sanger Institute for the COVID-19 Genomics UK (COG-UK) Consortium	Harper VanSteenhouse, Yumi Kasai, David Gray, Carol Clugston, Anna Dominiczak and Alex Alderton, Roberto Amato, Sonia Goncalves, Ewan Harrison, David K. Jackson, Ian Johnston, Dominic Kwiatkowski, Cordelia Langford, John Sillitoe on behalf of the Wellcome Sanger Institute COVID-19 Surveillance Team
EPI_ISL_719988, EPI_ISL_719989, EPI_ISL_719990, EPI_ISL_719991, EPI_ISL_719992, EPI_ISL_719993, EPI_ISL_719994, EPI_ISL_719995, EPI_ISL_719996, EPI_ISL_719997, EPI_ISL_719998, EPI_ISL_719999, EPI_ISL_720000, EPI_ISL_720001, EPI_ISL_720002, EPI_ISL_720003, EPI_ISL_720004, EPI_ISL_720005, EPI_ISL_720006, EPI_ISL_720007, EPI_ISL_720008, EPI_ISL_720009, EPI_ISL_720010, EPI_ISL_720011, EPI_ISL_720012, EPI_ISL_720013, EPI_ISL_720014, EPI_ISL_720015, EPI_ISL_720016, EPI_ISL_720017, EPI_ISL_720018, EPI_ISL_720019, EPI_ISL_720020, EPI_ISL_720021, EPI_ISL_720022, EPI_ISL_720023, EPI_ISL_720024, EPI_ISL_720025, EPI_ISL_720026, EPI_ISL_720027, EPI_ISL_720028, EPI_ISL_720029, EPI_ISL_720030, EPI_ISL_720031, EPI_ISL_720032, EPI_ISL_720033, EPI_ISL_720034, EPI_ISL_720035, EPI_ISL_720036, EPI_ISL_720037, EPI_ISL_720038, EPI_ISL_720039, EPI_ISL_720040, EPI_ISL_720041, EPI_ISL_720042, EPI_ISL_720043, EPI_ISL_720044, EPI_ISL_720045, EPI_ISL_720046, EPI_ISL_720047, EPI_ISL_720048, EPI_ISL_720049, EPI_ISL_720050, EPI_ISL_720051, EPI_ISL_720052, EPI_ISL_720053, EPI_ISL_720054, EPI_ISL_720055, EPI_ISL_720056, EPI_ISL_720057, EPI_ISL_720058, EPI_ISL_720059, EPI_ISL_720060, EPI_ISL_720061, EPI_ISL_720062, EPI_ISL_720063, EPI_ISL_720064, EPI_ISL_720065, EPI_ISL_720066, EPI_ISL_720067, EPI_ISL_720068, EPI_ISL_720069, EPI_ISL_720070, EPI_ISL_720071, EPI_ISL_720072, EPI_ISL_720073, EPI_ISL_720074, EPI_ISL_720075, EPI_ISL_720076, EPI_ISL_720077, EPI_ISL_720078, EPI_ISL_720079, EPI_ISL_720080, EPI_ISL_720081, EPI_ISL_720082, EPI_ISL_720083, EPI_ISL_720084, EPI_ISL_720085, EPI_ISL_720086, EPI_ISL_720087, EPI_ISL_720088, EPI_ISL_720089, EPI_ISL_720090, EPI_ISL_720091, EPI_ISL_720092, EPI_ISL_720093, EPI_ISL_720094, EPI_ISL_720095, EPI_ISL_720096, EPI_ISL_720097, EPI_ISL_720098, EPI_ISL_720099, EPI_ISL_720100, EPI_ISL_720101, EPI_ISL_720102, EPI_ISL_720103, EPI_ISL_720104, EPI_ISL_720105, EPI_ISL_720106, EPI_ISL_720107, EPI_ISL_720108, EPI_ISL_720109, EPI_ISL_720110, EPI_ISL_720111, EPI_ISL_720112, EPI_ISL_720113, EPI_ISL_720114, EPI_ISL_720115, EPI_ISL_720116, EPI_ISL_720117, EPI_ISL_720118, EPI_ISL_720119, EPI_ISL_720120, EPI_ISL_720121, EPI_ISL_720122, EPI_ISL_720123, EPI_ISL_720124, EPI_ISL_720125, EPI_ISL_720126, EPI_ISL_720127, EPI_ISL_720128, EPI_ISL_720129, EPI_ISL_720130, EPI_ISL_720131, EPI_ISL_720132, EPI_ISL_720133, EPI_ISL_720134, EPI_ISL_720135, EPI_ISL_720136, EPI_ISL_720137, EPI_ISL_720138, EPI_ISL_720139, EPI_ISL_720140, EPI_ISL_720141, EPI_ISL_720142, EPI_ISL_720143, EPI_ISL_720144, EPI_ISL_720145, EPI_ISL_720147, EPI_ISL_720148, EPI_ISL_720149, EPI_ISL_720150, EPI_ISL_720151, EPI_ISL_720152, EPI_ISL_720153, EPI_ISL_720154, EPI_ISL_720155, EPI_ISL_720156, EPI_ISL_720157, EPI_ISL_720158, EPI_ISL_720159, EPI_ISL_720160, EPI_ISL_720161, EPI_ISL_720162, EPI_ISL_720163, EPI_ISL_720164, EPI_ISL_720165, EPI_ISL_720166, EPI_ISL_720167, EPI_ISL_720168, EPI_ISL_720169, EPI_ISL_720170, EPI_ISL_720171, EPI_ISL_720172, EPI_ISL_720173, EPI_ISL_720174, EPI_ISL_720175, EPI_ISL_720176, EPI_ISL_720177, EPI_ISL_720178, EPI_ISL_720179, EPI_ISL_720180, EPI_ISL_720181, EPI_ISL_720182, EPI_ISL_720183, EPI_ISL_720184, EPI_ISL_720185, EPI_ISL_720186, EPI_ISL_720187, EPI_ISL_720188, EPI_ISL_720189, EPI_ISL_720190, EPI_ISL_720191, EPI_ISL_720192, EPI_ISL_720193, EPI_ISL_720194, EPI_ISL_720195, EPI_ISL_720196, EPI_ISL_720197, EPI_ISL_720198, EPI_ISL_720199, EPI_ISL_720200, EPI_ISL_720201, EPI_ISL_720202, EPI_ISL_720203, EPI_ISL_720204, EPI_ISL_720205, EPI_ISL_720206, EPI_ISL_720207, EPI_ISL_720208, EPI_ISL_720209, EPI_ISL_720210, EPI_ISL_720211, EPI_ISL_720212, EPI_ISL_720213, EPI_ISL_720214, EPI_ISL_720215, EPI_ISL_720216, EPI_ISL_720217, EPI_ISL_720218, EPI_ISL_720219, EPI_ISL_720220, EPI_ISL_720221, EPI_ISL_720222, EPI_ISL_720223, EPI_ISL_720224, EPI_ISL_720225, EPI_ISL_720226, EPI_ISL_720227, EPI_ISL_720228, EPI_ISL_720229, EPI_ISL_720230, EPI_ISL_720231, EPI_ISL_720232, EPI_ISL_720233, EPI_ISL_720234, EPI_ISL_720235, EPI_ISL_720236, EPI_ISL_720237, EPI_ISL_720238, EPI_ISL_720239, EPI_ISL_720240, EPI_ISL_720241, EPI_ISL_720242, EPI_ISL_720243, EPI_ISL_720244, EPI_ISL_720245, EPI_ISL_720246, EPI_ISL_720247, EPI_ISL_720248, EPI_ISL_720249, EPI_ISL_720250, EPI_ISL_720251, EPI_ISL_720252, EPI_ISL_720253, EPI_ISL_720254, EPI_ISL_720255, EPI_ISL_720256, EPI_ISL_720257, EPI_ISL_720258, EPI_ISL_720259, EPI_ISL_720261, EPI_ISL_720262, EPI_ISL_720263, EPI_ISL_720264, EPI_ISL_720265, EPI_ISL_720266, EPI_ISL_720267, EPI_ISL_720268, EPI_ISL_720269, EPI_ISL_720270, EPI_ISL_720271, EPI_ISL_720272, EPI_ISL_720273, EPI_ISL_720274, EPI_ISL_720275, EPI_ISL_720276, EPI_ISL_720277, EPI_ISL_720278, EPI_ISL_720279, EPI_ISL_720280, EPI_ISL_720281, EPI_ISL_720282, EPI_ISL_720283, EPI_ISL_720284, EPI_ISL_720285, EPI_ISL_720286, EPI_ISL_720287, EPI_ISL_720288, EPI_ISL_720289, EPI_ISL_720290, EPI_ISL_720291, EPI_ISL_720292, EPI_ISL_720293, EPI_ISL_720294, EPI_ISL_720295, EPI_ISL_720296, EPI_ISL_720297, EPI_ISL_720298, EPI_ISL_720299, EPI_ISL_720300, EPI_ISL_720303, EPI_ISL_720304, EPI_ISL_720305, EPI_ISL_720306, EPI_ISL_720307, EPI_ISL_720308, EPI_ISL_720309, EPI_ISL_720310, EPI_ISL_720311, EPI_ISL_720312, EPI_ISL_720313, EPI_ISL_720314, EPI_ISL_720315, EPI_ISL_720316, EPI_ISL_720317, EPI_ISL_720318, EPI_ISL_720319, EPI_ISL_720320, EPI_ISL_720321, EPI_ISL_720322, EPI_ISL_720323, EPI_ISL_720324, EPI_ISL_720325, EPI_ISL_720326, EPI_ISL_720327, EPI_ISL_720328, EPI_ISL_720329, EPI_ISL_720330, EPI_ISL_720331, EPI_ISL_720332, EPI_ISL_720334, EPI_ISL_720335, EPI_ISL_720336, EPI_ISL_720337, EPI_ISL_720338, EPI_ISL_720339, EPI_ISL_720340, EPI_ISL_720341, EPI_ISL_720342, EPI_ISL_720343, EPI_ISL_720344, EPI_ISL_720345, EPI_ISL_720346, EPI_ISL_720347, EPI_ISL_720348, EPI_ISL_720349, EPI_ISL_720350, EPI_ISL_720351, EPI_ISL_720352, EPI_ISL_720353, EPI_ISL_720354, EPI_ISL_720355, EPI_ISL_720356, EPI_ISL_720357, EPI_ISL_720358, EPI_ISL_720359, EPI_ISL_720360, EPI_ISL_720361, EPI_ISL_720362, EPI_ISL_720363, EPI_ISL_720364, EPI_ISL_720365, EPI_ISL_720366, EPI_ISL_720367, EPI_ISL_720368, EPI_ISL_720369, EPI_ISL_720370, EPI_ISL_720371, EPI_ISL_720372, EPI_ISL_720373, EPI_ISL_720374, EPI_ISL_720375, EPI_ISL_720376, EPI_ISL_720377, EPI_ISL_720378, EPI_ISL_720379, EPI_ISL_720380, EPI_ISL_720381, EPI_ISL_720382, EPI_ISL_720383, EPI_ISL_720384, EPI_ISL_720385, EPI_ISL_720386, EPI_ISL_720387, EPI_ISL_720388, EPI_ISL_720389, EPI_ISL_720390, EPI_ISL_720391, EPI_ISL_720392, EPI_ISL_720393, EPI_ISL_720394, EPI_ISL_720395, EPI_ISL_720396, EPI_ISL_720397, EPI_ISL_720398, EPI_ISL_720399, EPI_ISL_720400, EPI_ISL_720401, EPI_ISL_720402, EPI_ISL_720403, EPI_ISL_720404, EPI_ISL_720405, EPI_ISL_720406, EPI_ISL_720407, EPI_ISL_720408, EPI_ISL_720409, EPI_ISL_720410, EPI_ISL_720411, EPI_ISL_720412, EPI_ISL_720413, EPI_ISL_720414, EPI_ISL_720415, EPI_ISL_720416, EPI_ISL_720417, EPI_ISL_720418, EPI_ISL_720420, EPI_ISL_720421, EPI_ISL_720422, EPI_ISL_720423, EPI_ISL_720424, EPI_ISL_720425, EPI_ISL_720426, EPI_ISL_720427, EPI_ISL_720428, EPI_ISL_720429, EPI_ISL_720430, EPI_ISL_720431, EPI_ISL_720432, EPI_ISL_720433, EPI_ISL_720434, EPI_ISL_720435, EPI_ISL_720436, EPI_ISL_720437, EPI_ISL_720438, EPI_ISL_720439, EPI_ISL_720440, EPI_ISL_720441, EPI_ISL_720442, EPI_ISL_720443, EPI_ISL_720444, EPI_ISL_720445, EPI_ISL_720446, EPI_ISL_720447, EPI_ISL_720448, EPI_ISL_720449, EPI_ISL_720450, EPI_ISL_720451, EPI_ISL_720452, EPI_ISL_720453, EPI_ISL_720454, EPI_ISL_720455, EPI_ISL_720456, EPI_ISL_720457, EPI_ISL_720458, EPI_ISL_720459, EPI_ISL_720460, EPI_ISL_720461, EPI_ISL_720462, EPI_ISL_720463, EPI_ISL_720464, EPI_ISL_720465, EPI_ISL_720466, EPI_ISL_720467, EPI_ISL_720468, EPI_ISL_720469, EPI_ISL_720470, EPI_ISL_720471, EPI_ISL_720472, EPI_ISL_720473, EPI_ISL_720474, EPI_ISL_720475, EPI_ISL_720476, EPI_ISL_720477, EPI_ISL_720478, EPI_ISL_720479, EPI_ISL_720480, EPI_ISL_720481, EPI_ISL_720482, EPI_ISL_720483, EPI_ISL_720484, EPI_ISL_720485, EPI_ISL_720486, EPI_ISL_720487, EPI_ISL_720488, EPI_ISL_720489, EPI_ISL_720490, EPI_ISL_720491, EPI_ISL_720492, EPI_ISL_720493, EPI_ISL_720494, EPI_ISL_720495, EPI_ISL_720496, EPI_ISL_720497, EPI_ISL_720498, EPI_ISL_720499, EPI_ISL_720500, EPI_ISL_720501, EPI_ISL_720503, EPI_ISL_720504, EPI_ISL_720505, EPI_ISL_720506, EPI_ISL_720507, EPI_ISL_720508, EPI_ISL_720509, EPI_ISL_720510, EPI_ISL_720511, EPI_ISL_720512, EPI_ISL_720513, EPI_ISL_720514, EPI_ISL_720515, EPI_ISL_720516, EPI_ISL_720517, EPI_ISL_720518, EPI_ISL_720519, EPI_ISL_720520, EPI_ISL_720521, EPI_ISL_720522, EPI_ISL_720523, EPI_ISL_720524, EPI_ISL_720525, EPI_ISL_720526, EPI_ISL_720527, EPI_ISL_720528, EPI_ISL_720529, EPI_ISL_720530, EPI_ISL_720531, EPI_ISL_720532, EPI_ISL_720533, EPI_ISL_720534, EPI_ISL_720535, EPI_ISL_720536, EPI_ISL_720537, EPI_ISL_720538, EPI_ISL_720539, EPI_ISL_720540, EPI_ISL_720541, EPI_ISL_720542, EPI_ISL_720543, EPI_ISL_720544, EPI_ISL_720545, EPI_ISL_720546, EPI_ISL_720547, EPI_ISL_720548, EPI_ISL_720549, EPI_ISL_720550, EPI_ISL_720551, EPI_ISL_720552, EPI_ISL_720553, EPI_ISL_720554, EPI_ISL_720555, EPI_ISL_720556, EPI_ISL_720557, EPI_ISL_720558, EPI_ISL_720559, EPI_ISL_720560, EPI_ISL_720561, EPI_ISL_720562, EPI_ISL_720563, EPI_ISL_720564, EPI_ISL_720565, EPI_ISL_720566, EPI_ISL_720567, EPI_ISL_720568, EPI_ISL_720569, EPI_ISL_720570, EPI_ISL_720571, EPI_ISL_720572, EPI_ISL_720573, EPI_ISL_720574, EPI_ISL_720575, EPI_ISL_720576, EPI_ISL_720577, EPI_ISL_720578, EPI_ISL_720579, EPI_ISL_720580, EPI_ISL_720581, EPI_ISL_720582, EPI_ISL_720583, EPI_ISL_720584, EPI_ISL_720585, EPI_ISL_720586, EPI_ISL_720587, EPI_ISL_720588, EPI_ISL_720589, EPI_ISL_720590, EPI_ISL_720591, EPI_ISL_720592, EPI_ISL_720593, EPI_ISL_720594, EPI_ISL_720595, EPI_ISL_720596, EPI_ISL_720597, EPI_ISL_720598, EPI_ISL_720599, EPI_ISL_720600, EPI_ISL_720601, EPI_ISL_720602, EPI_ISL_720603, EPI_ISL_720604, EPI_ISL_720605, EPI_ISL_720606, EPI_ISL_720607, EPI_ISL_720608, EPI_ISL_720609, EPI_ISL_720610, EPI_ISL_720611, EPI_ISL_720612, EPI_ISL_720613, EPI_ISL_720614, EPI_ISL_720615, EPI_ISL_720616, EPI_ISL_720617, EPI_ISL_720618, EPI_ISL_720619, EPI_ISL_720620, EPI_ISL_720621, EPI_ISL_720622, EPI_ISL_720623, EPI_ISL_720624, EPI_ISL_720625, EPI_ISL_720626, EPI_ISL_720627, EPI_ISL_720628, EPI_ISL_720630, EPI_ISL_720631, EPI_ISL_720632, EPI_ISL_720633, EPI_ISL_720634, EPI_ISL_720635, EPI_ISL_720636, EPI_ISL_720637, EPI_ISL_720638, EPI_ISL_720639, EPI_ISL_720640, EPI_ISL_720641, EPI_ISL_720642, EPI_ISL_720643, EPI_ISL_720644, EPI_ISL_720645, EPI_ISL_720646, EPI_ISL_720647, EPI_ISL_720648, EPI_ISL_720649, EPI_ISL_720650, EPI_ISL_720651, EPI_ISL_720652, EPI_ISL_720653, EPI_ISL_720654, EPI_ISL_720655, EPI_ISL_720656, EPI_ISL_720657, EPI_ISL_720658, EPI_ISL_720659, EPI_ISL_720660, EPI_ISL_720661, EPI_ISL_720662, EPI_ISL_720663, EPI_ISL_720664, EPI_ISL_720665, EPI_ISL_720666, EPI_ISL_720667			
see above	Lighthouse Lab in Milton Keynes	Wellcome Sanger Institute for the COVID-19 Genomics UK (COG-UK) Consortium	The Lighthouse Lab in Milton Keynes and Alex Alderton, Roberto Amato, Sonia Goncalves, Ewan Harrison, David K. Jackson, Ian Johnston, Dominic Kwiatkowski, Cordelia Langford, John Sillitoe on behalf of the Wellcome Sanger Institute COVID-19 Surveillance Team
EPI_ISL_720668	Lighthouse Lab in Glasgow	Wellcome Sanger Institute for the COVID-19 Genomics UK (COG-UK) Consortium	Harper VanSteenhouse, Yumi Kasai, David Gray, Carol Clugston, Anna Dominiczak and Alex Alderton, Roberto Amato, Sonia Goncalves, Ewan Harrison, David K. Jackson, Ian Johnston, Dominic Kwiatkowski, Cordelia Langford, John Sillitoe on behalf of the Wellcome Sanger Institute COVID-19 Surveillance Team
EPI_ISL_720669, EPI_ISL_720671	Lighthouse Lab in Milton Keynes	Wellcome Sanger Institute for the COVID-19 Genomics UK (COG-UK) Consortium	The Lighthouse Lab in Milton Keynes and Alex Alderton, Roberto Amato, Sonia Goncalves, Ewan Harrison, David K. Jackson, Ian Johnston, Dominic Kwiatkowski, Cordelia Langford, John Sillitoe on behalf of the Wellcome Sanger Institute COVID-19 Surveillance Team
EPI_ISL_720672	Lighthouse Lab in Glasgow	Wellcome Sanger Institute for the COVID-19 Genomics UK (COG-UK) Consortium	Harper VanSteenhouse, Yumi Kasai, David Gray, Carol Clugston, Anna Dominiczak and Alex Alderton, Roberto Amato, Sonia Goncalves, Ewan Harrison, David K. Jackson, Ian Johnston, Dominic Kwiatkowski, Cordelia Langford, John Sillitoe on behalf of the Wellcome Sanger Institute COVID-19 Surveillance Team
EPI_ISL_720673, EPI_ISL_720674, EPI_ISL_720675, EPI_ISL_720676, EPI_ISL_720677, EPI_ISL_720678, EPI_ISL_720679, EPI_ISL_720680, EPI_ISL_720681, EPI_ISL_720682	Lighthouse Lab in Milton Keynes	Wellcome Sanger Institute for the COVID-19 Genomics UK (COG-UK) Consortium	The Lighthouse Lab in Milton Keynes and Alex Alderton, Roberto Amato, Sonia Goncalves, Ewan Harrison, David K. Jackson, Ian Johnston, Dominic Kwiatkowski, Cordelia Langford, John Sillitoe on behalf of the Wellcome Sanger Institute COVID-19 Surveillance Team
EPI_ISL_720683	Lighthouse Lab in Alderley Park	Wellcome Sanger Institute for the COVID-19 Genomics UK (COG-UK) Consortium	Jacquelyn Wynn, Mairead Hyland, The Lighthouse Lab in Alderley Park and Alex Alderton, Roberto Amato, Sonia Goncalves, Ewan Harrison, David K. Jackson, Ian Johnston, Dominic Kwiatkowski, Cordelia Langford, John Sillitoe on behalf of the Wellcome Sanger Institute COVID-19 Surveillance Team

EPI_ISL_720684	Lighthouse Lab in Glasgow	Wellcome Sanger Institute for the COVID-19 Genomics UK (COG-UK) Consortium	Harper VanSteenhouse, Yumi Kasai, David Gray, Carol Clugston, Anna Dominiczak and Alex Alderton, Roberto Amato, Sonia Goncalves, Ewan Harrison, David K. Jackson, Ian Johnston, Dominic Kwiatkowski, Cordelia Langford, John Sillitoe on behalf of the Wellcome Sanger Institute COVID-19 Surveillance Team
EPI_ISL_720685, EPI_ISL_720686	Lighthouse Lab in Alderley Park	Wellcome Sanger Institute for the COVID-19 Genomics UK (COG-UK) Consortium	Jacquelyn Wynn, Mairead Hyland, The Lighthouse Lab in Alderley Park and Alex Alderton, Roberto Amato, Sonia Goncalves, Ewan Harrison, David K. Jackson, Ian Johnston, Dominic Kwiatkowski, Cordelia Langford, John Sillitoe on behalf of the Wellcome Sanger Institute COVID-19 Surveillance Team
EPI_ISL_720687	Lighthouse Lab in Milton Keynes	Wellcome Sanger Institute for the COVID-19 Genomics UK (COG-UK) Consortium	The Lighthouse Lab in Milton Keynes and Alex Alderton, Roberto Amato, Sonia Goncalves, Ewan Harrison, David K. Jackson, Ian Johnston, Dominic Kwiatkowski, Cordelia Langford, John Sillitoe on behalf of the Wellcome Sanger Institute COVID-19 Surveillance Team
EPI_ISL_720688	Lighthouse Lab in Alderley Park	Wellcome Sanger Institute for the COVID-19 Genomics UK (COG-UK) Consortium	Jacquelyn Wynn, Mairead Hyland, The Lighthouse Lab in Alderley Park and Alex Alderton, Roberto Amato, Sonia Goncalves, Ewan Harrison, David K. Jackson, Ian Johnston, Dominic Kwiatkowski, Cordelia Langford, John Sillitoe on behalf of the Wellcome Sanger Institute COVID-19 Surveillance Team
EPI_ISL_720689, EPI_ISL_720691, EPI_ISL_720692, EPI_ISL_720693	Lighthouse Lab in Milton Keynes	Wellcome Sanger Institute for the COVID-19 Genomics UK (COG-UK) Consortium	The Lighthouse Lab in Milton Keynes and Alex Alderton, Roberto Amato, Sonia Goncalves, Ewan Harrison, David K. Jackson, Ian Johnston, Dominic Kwiatkowski, Cordelia Langford, John Sillitoe on behalf of the Wellcome Sanger Institute COVID-19 Surveillance Team
EPI_ISL_720694, EPI_ISL_720695	Lighthouse Lab in Alderley Park	Wellcome Sanger Institute for the COVID-19 Genomics UK (COG-UK) Consortium	Jacquelyn Wynn, Mairead Hyland, The Lighthouse Lab in Alderley Park and Alex Alderton, Roberto Amato, Sonia Goncalves, Ewan Harrison, David K. Jackson, Ian Johnston, Dominic Kwiatkowski, Cordelia Langford, John Sillitoe on behalf of the Wellcome Sanger Institute COVID-19 Surveillance Team
EPI_ISL_720696	Lighthouse Lab in Milton Keynes	Wellcome Sanger Institute for the COVID-19 Genomics UK (COG-UK) Consortium	The Lighthouse Lab in Milton Keynes and Alex Alderton, Roberto Amato, Sonia Goncalves, Ewan Harrison, David K. Jackson, Ian Johnston, Dominic Kwiatkowski, Cordelia Langford, John Sillitoe on behalf of the Wellcome Sanger Institute COVID-19 Surveillance Team
EPI_ISL_720697	Lighthouse Lab in Alderley Park	Wellcome Sanger Institute for the COVID-19 Genomics UK (COG-UK) Consortium	Jacquelyn Wynn, Mairead Hyland, The Lighthouse Lab in Alderley Park and Alex Alderton, Roberto Amato, Sonia Goncalves, Ewan Harrison, David K. Jackson, Ian Johnston, Dominic Kwiatkowski, Cordelia Langford, John Sillitoe on behalf of the Wellcome Sanger Institute COVID-19 Surveillance Team
EPI_ISL_720698, EPI_ISL_720699, EPI_ISL_720700, EPI_ISL_720701	Lighthouse Lab in Milton Keynes	Wellcome Sanger Institute for the COVID-19 Genomics UK (COG-UK) Consortium	The Lighthouse Lab in Milton Keynes and Alex Alderton, Roberto Amato, Sonia Goncalves, Ewan Harrison, David K. Jackson, Ian Johnston, Dominic Kwiatkowski, Cordelia Langford, John Sillitoe on behalf of the Wellcome Sanger Institute COVID-19 Surveillance Team
EPI_ISL_720702	Lighthouse Lab in Alderley Park	Wellcome Sanger Institute for the COVID-19 Genomics UK (COG-UK) Consortium	Jacquelyn Wynn, Mairead Hyland, The Lighthouse Lab in Alderley Park and Alex Alderton, Roberto Amato, Sonia Goncalves, Ewan Harrison, David K. Jackson, Ian Johnston, Dominic Kwiatkowski, Cordelia Langford, John Sillitoe on behalf of the Wellcome Sanger Institute COVID-19 Surveillance Team
EPI_ISL_720703	Lighthouse Lab in Milton Keynes	Wellcome Sanger Institute for the COVID-19 Genomics UK (COG-UK) Consortium	The Lighthouse Lab in Milton Keynes and Alex Alderton, Roberto Amato, Sonia Goncalves, Ewan Harrison, David K. Jackson, Ian Johnston, Dominic Kwiatkowski, Cordelia Langford, John Sillitoe on behalf of the Wellcome Sanger Institute COVID-19 Surveillance Team
EPI_ISL_720704	Lighthouse Lab in Glasgow	Wellcome Sanger Institute for the COVID-19 Genomics UK (COG-UK) Consortium	Harper VanSteenhouse, Yumi Kasai, David Gray, Carol Clugston, Anna Dominiczak and Alex Alderton, Roberto Amato, Sonia Goncalves, Ewan Harrison, David K. Jackson, Ian Johnston, Dominic Kwiatkowski, Cordelia Langford, John Sillitoe on behalf of the Wellcome Sanger Institute COVID-19 Surveillance Team
EPI_ISL_720705, EPI_ISL_720706, EPI_ISL_720707, EPI_ISL_720708	Lighthouse Lab in Milton Keynes	Wellcome Sanger Institute for the COVID-19 Genomics UK (COG-UK) Consortium	The Lighthouse Lab in Milton Keynes and Alex Alderton, Roberto Amato, Sonia Goncalves, Ewan Harrison, David K. Jackson, Ian Johnston, Dominic Kwiatkowski, Cordelia Langford, John Sillitoe on behalf of the Wellcome Sanger Institute COVID-19 Surveillance Team
EPI_ISL_720709	Lighthouse Lab in Alderley Park	Wellcome Sanger Institute for the COVID-19 Genomics UK (COG-UK) Consortium	Jacquelyn Wynn, Mairead Hyland, The Lighthouse Lab in Alderley Park and Alex Alderton, Roberto Amato, Sonia Goncalves, Ewan Harrison, David K. Jackson, Ian Johnston, Dominic Kwiatkowski, Cordelia Langford, John Sillitoe on behalf of the Wellcome Sanger Institute COVID-19 Surveillance Team
EPI_ISL_720710	Lighthouse Lab in Milton Keynes	Wellcome Sanger Institute for the COVID-19 Genomics UK (COG-UK) Consortium	The Lighthouse Lab in Milton Keynes and Alex Alderton, Roberto Amato, Sonia Goncalves, Ewan Harrison, David K. Jackson, Ian Johnston, Dominic Kwiatkowski, Cordelia Langford, John Sillitoe on behalf of the Wellcome Sanger Institute COVID-19 Surveillance Team
EPI_ISL_720711	Lighthouse Lab in Alderley Park	Wellcome Sanger Institute for the COVID-19 Genomics UK (COG-UK) Consortium	Jacquelyn Wynn, Mairead Hyland, The Lighthouse Lab in Alderley Park and Alex Alderton, Roberto Amato, Sonia Goncalves, Ewan Harrison, David K. Jackson, Ian Johnston, Dominic Kwiatkowski, Cordelia Langford, John Sillitoe on behalf of the Wellcome Sanger Institute COVID-19 Surveillance Team
EPI_ISL_720712	Lighthouse Lab in Milton Keynes	Wellcome Sanger Institute for the COVID-19 Genomics UK (COG-UK) Consortium	The Lighthouse Lab in Milton Keynes and Alex Alderton, Roberto Amato, Sonia Goncalves, Ewan Harrison, David K. Jackson, Ian Johnston, Dominic Kwiatkowski, Cordelia Langford, John Sillitoe on behalf of the Wellcome Sanger Institute COVID-19 Surveillance Team
EPI_ISL_720713	Lighthouse Lab in Alderley Park	Wellcome Sanger Institute for the COVID-19 Genomics UK (COG-UK) Consortium	Jacquelyn Wynn, Mairead Hyland, The Lighthouse Lab in Alderley Park and Alex Alderton, Roberto Amato, Sonia Goncalves, Ewan Harrison, David K. Jackson, Ian Johnston, Dominic Kwiatkowski, Cordelia Langford, John Sillitoe on behalf of the Wellcome Sanger Institute COVID-19 Surveillance Team
EPI_ISL_720714	Lighthouse Lab in Milton Keynes	Wellcome Sanger Institute for the COVID-19 Genomics UK (COG-UK) Consortium	The Lighthouse Lab in Milton Keynes and Alex Alderton, Roberto Amato, Sonia Goncalves, Ewan Harrison, David K. Jackson, Ian Johnston, Dominic Kwiatkowski, Cordelia Langford, John Sillitoe on behalf of the Wellcome Sanger Institute COVID-19 Surveillance Team
EPI_ISL_720715	Lighthouse Lab in Alderley Park	Wellcome Sanger Institute for the COVID-19 Genomics UK (COG-UK) Consortium	Jacquelyn Wynn, Mairead Hyland, The Lighthouse Lab in Alderley Park and Alex Alderton, Roberto Amato, Sonia Goncalves, Ewan Harrison, David K. Jackson, Ian Johnston, Dominic Kwiatkowski, Cordelia Langford, John Sillitoe on behalf of the Wellcome Sanger Institute COVID-19 Surveillance Team
EPI_ISL_720716, EPI_ISL_720717, EPI_ISL_720718, EPI_ISL_720719, EPI_ISL_720720, EPI_ISL_720721	Lighthouse Lab in Milton Keynes	Wellcome Sanger Institute for the COVID-19 Genomics UK (COG-UK) Consortium	The Lighthouse Lab in Milton Keynes and Alex Alderton, Roberto Amato, Sonia Goncalves, Ewan Harrison, David K. Jackson, Ian Johnston, Dominic Kwiatkowski, Cordelia Langford, John Sillitoe on behalf of the Wellcome Sanger Institute COVID-19 Surveillance Team
EPI_ISL_720722	Lighthouse Lab in Alderley Park	Wellcome Sanger Institute for the COVID-19 Genomics UK (COG-UK) Consortium	Jacquelyn Wynn, Mairead Hyland, The Lighthouse Lab in Alderley Park and Alex Alderton, Roberto Amato, Sonia Goncalves, Ewan Harrison, David K. Jackson, Ian Johnston, Dominic Kwiatkowski, Cordelia Langford, John Sillitoe on behalf of the Wellcome Sanger Institute COVID-19 Surveillance Team
EPI_ISL_720723, EPI_ISL_720724, EPI_ISL_720725	Lighthouse Lab in Milton Keynes	Wellcome Sanger Institute for the COVID-19 Genomics UK (COG-UK) Consortium	The Lighthouse Lab in Milton Keynes and Alex Alderton, Roberto Amato, Sonia Goncalves, Ewan Harrison, David K. Jackson, Ian Johnston, Dominic Kwiatkowski, Cordelia Langford, John Sillitoe on behalf of the Wellcome Sanger Institute COVID-19 Surveillance Team
EPI_ISL_720726	Lighthouse Lab in Glasgow	Wellcome Sanger Institute for the COVID-19 Genomics UK (COG-UK) Consortium	Harper VanSteenhouse, Yumi Kasai, David Gray, Carol Clugston, Anna Dominiczak and Alex Alderton, Roberto Amato, Sonia Goncalves, Ewan Harrison, David K. Jackson, Ian Johnston, Dominic Kwiatkowski, Cordelia Langford, John Sillitoe on behalf of the Wellcome Sanger Institute COVID-19 Surveillance Team
EPI_ISL_720727	Lighthouse Lab in Milton Keynes	Wellcome Sanger Institute for the COVID-19 Genomics UK (COG-UK) Consortium	The Lighthouse Lab in Milton Keynes and Alex Alderton, Roberto Amato, Sonia Goncalves, Ewan Harrison, David K. Jackson, Ian Johnston, Dominic Kwiatkowski, Cordelia Langford, John Sillitoe on behalf of the Wellcome Sanger Institute COVID-19 Surveillance Team
EPI_ISL_720728	Lighthouse Lab in Glasgow	Wellcome Sanger Institute for the COVID-19 Genomics UK (COG-UK) Consortium	Harper VanSteenhouse, Yumi Kasai, David Gray, Carol Clugston, Anna Dominiczak and Alex Alderton, Roberto Amato, Sonia Goncalves, Ewan Harrison, David K. Jackson, Ian Johnston, Dominic Kwiatkowski, Cordelia Langford, John Sillitoe on behalf of the Wellcome Sanger Institute COVID-19 Surveillance Team
EPI_ISL_720729, EPI_ISL_720730, EPI_ISL_720731	Lighthouse Lab in Milton Keynes	Wellcome Sanger Institute for the COVID-19 Genomics UK (COG-UK) Consortium	The Lighthouse Lab in Milton Keynes and Alex Alderton, Roberto Amato, Sonia Goncalves, Ewan Harrison, David K. Jackson, Ian Johnston, Dominic Kwiatkowski, Cordelia Langford, John Sillitoe on behalf of the Wellcome Sanger Institute COVID-19 Surveillance Team
EPI_ISL_720732, EPI_ISL_720733	Lighthouse Lab in Glasgow	Wellcome Sanger Institute for the COVID-19 Genomics UK (COG-UK) Consortium	Harper VanSteenhouse, Yumi Kasai, David Gray, Carol Clugston, Anna Dominiczak and Alex Alderton, Roberto Amato, Sonia Goncalves, Ewan Harrison, David K. Jackson, Ian Johnston, Dominic Kwiatkowski, Cordelia Langford, John Sillitoe on behalf of the Wellcome Sanger Institute COVID-19 Surveillance Team
EPI_ISL_720734	Lighthouse Lab in Alderley Park	Wellcome Sanger Institute for the COVID-19 Genomics UK (COG-UK) Consortium	Jacquelyn Wynn, Mairead Hyland, The Lighthouse Lab in Alderley Park and Alex Alderton, Roberto Amato, Sonia Goncalves, Ewan Harrison, David K. Jackson, Ian Johnston, Dominic Kwiatkowski, Cordelia Langford, John Sillitoe on behalf of the Wellcome Sanger Institute COVID-19 Surveillance Team
EPI_ISL_720735, EPI_ISL_720736, EPI_ISL_720737	Lighthouse Lab in Milton Keynes	Wellcome Sanger Institute for the COVID-19 Genomics UK (COG-UK) Consortium	The Lighthouse Lab in Milton Keynes and Alex Alderton, Roberto Amato, Sonia Goncalves, Ewan Harrison, David K. Jackson, Ian Johnston, Dominic Kwiatkowski, Cordelia Langford, John Sillitoe on behalf of the Wellcome Sanger Institute COVID-19 Surveillance Team
EPI_ISL_720738	Lighthouse Lab in Glasgow	Wellcome Sanger Institute for the COVID-19 Genomics UK (COG-UK) Consortium	Harper VanSteenhouse, Yumi Kasai, David Gray, Carol Clugston, Anna Dominiczak and Alex Alderton, Roberto Amato, Sonia Goncalves, Ewan Harrison, David K. Jackson, Ian Johnston, Dominic Kwiatkowski, Cordelia Langford, John Sillitoe on behalf of the Wellcome Sanger Institute COVID-19 Surveillance Team
EPI_ISL_720739	Lighthouse Lab in Milton Keynes	Wellcome Sanger Institute for the COVID-19 Genomics UK (COG-UK) Consortium	The Lighthouse Lab in Milton Keynes and Alex Alderton, Roberto Amato, Sonia Goncalves, Ewan Harrison, David K. Jackson, Ian Johnston, Dominic Kwiatkowski, Cordelia Langford, John Sillitoe on behalf of the Wellcome Sanger Institute COVID-19 Surveillance Team
EPI_ISL_720740	Lighthouse Lab in Glasgow	Wellcome Sanger Institute for the COVID-19 Genomics UK	Harper VanSteenhouse, Yumi Kasai, David Gray, Carol Clugston, Anna Dominiczak and Alex Alderton, Roberto Amato, Sonia Goncalves, Ewan Harrison,

		(COG-UK) Consortium	David K. Jackson, Ian Johnston, Dominic Kwiatkowski, Cordelia Langford, John Sillitoe on behalf of the Wellcome Sanger Institute COVID-19 Surveillance Team
EPI_ISL_720741	Lighthouse Lab in Alderley Park	Wellcome Sanger Institute for the COVID-19 Genomics UK (COG-UK) Consortium	Jacquelyn Wynn, Mairead Hyland, The Lighthouse Lab in Alderley Park and Alex Alderton, Roberto Amato, Sonia Goncalves, Ewan Harrison, David K. Jackson, Ian Johnston, Dominic Kwiatkowski, Cordelia Langford, John Sillitoe on behalf of the Wellcome Sanger Institute COVID-19 Surveillance Team
EPI_ISL_720742, EPI_ISL_720743, EPI_ISL_720744	Lighthouse Lab in Milton Keynes	Wellcome Sanger Institute for the COVID-19 Genomics UK (COG-UK) Consortium	The Lighthouse Lab in Milton Keynes and Alex Alderton, Roberto Amato, Sonia Goncalves, Ewan Harrison, David K. Jackson, Ian Johnston, Dominic Kwiatkowski, Cordelia Langford, John Sillitoe on behalf of the Wellcome Sanger Institute COVID-19 Surveillance Team
EPI_ISL_720745	Lighthouse Lab in Alderley Park	Wellcome Sanger Institute for the COVID-19 Genomics UK (COG-UK) Consortium	Jacquelyn Wynn, Mairead Hyland, The Lighthouse Lab in Alderley Park and Alex Alderton, Roberto Amato, Sonia Goncalves, Ewan Harrison, David K. Jackson, Ian Johnston, Dominic Kwiatkowski, Cordelia Langford, John Sillitoe on behalf of the Wellcome Sanger Institute COVID-19 Surveillance Team
EPI_ISL_720746, EPI_ISL_720747, EPI_ISL_720748	Lighthouse Lab in Milton Keynes	Wellcome Sanger Institute for the COVID-19 Genomics UK (COG-UK) Consortium	The Lighthouse Lab in Milton Keynes and Alex Alderton, Roberto Amato, Sonia Goncalves, Ewan Harrison, David K. Jackson, Ian Johnston, Dominic Kwiatkowski, Cordelia Langford, John Sillitoe on behalf of the Wellcome Sanger Institute COVID-19 Surveillance Team
EPI_ISL_720749	Lighthouse Lab in Glasgow	Wellcome Sanger Institute for the COVID-19 Genomics UK (COG-UK) Consortium	Harper VanSteenhouse, Yumi Kasai, David Gray, Carol Clugston, Anna Dominiczak and Alex Alderton, Roberto Amato, Sonia Goncalves, Ewan Harrison, David K. Jackson, Ian Johnston, Dominic Kwiatkowski, Cordelia Langford, John Sillitoe on behalf of the Wellcome Sanger Institute COVID-19 Surveillance Team
EPI_ISL_720750	Lighthouse Lab in Alderley Park	Wellcome Sanger Institute for the COVID-19 Genomics UK (COG-UK) Consortium	Jacquelyn Wynn, Mairead Hyland, The Lighthouse Lab in Alderley Park and Alex Alderton, Roberto Amato, Sonia Goncalves, Ewan Harrison, David K. Jackson, Ian Johnston, Dominic Kwiatkowski, Cordelia Langford, John Sillitoe on behalf of the Wellcome Sanger Institute COVID-19 Surveillance Team
EPI_ISL_720751	Lighthouse Lab in Milton Keynes	Wellcome Sanger Institute for the COVID-19 Genomics UK (COG-UK) Consortium	The Lighthouse Lab in Milton Keynes and Alex Alderton, Roberto Amato, Sonia Goncalves, Ewan Harrison, David K. Jackson, Ian Johnston, Dominic Kwiatkowski, Cordelia Langford, John Sillitoe on behalf of the Wellcome Sanger Institute COVID-19 Surveillance Team
EPI_ISL_720752	Lighthouse Lab in Glasgow	Wellcome Sanger Institute for the COVID-19 Genomics UK (COG-UK) Consortium	Harper VanSteenhouse, Yumi Kasai, David Gray, Carol Clugston, Anna Dominiczak and Alex Alderton, Roberto Amato, Sonia Goncalves, Ewan Harrison, David K. Jackson, Ian Johnston, Dominic Kwiatkowski, Cordelia Langford, John Sillitoe on behalf of the Wellcome Sanger Institute COVID-19 Surveillance Team
EPI_ISL_720753, EPI_ISL_720754	Lighthouse Lab in Milton Keynes	Wellcome Sanger Institute for the COVID-19 Genomics UK (COG-UK) Consortium	The Lighthouse Lab in Milton Keynes and Alex Alderton, Roberto Amato, Sonia Goncalves, Ewan Harrison, David K. Jackson, Ian Johnston, Dominic Kwiatkowski, Cordelia Langford, John Sillitoe on behalf of the Wellcome Sanger Institute COVID-19 Surveillance Team
EPI_ISL_720755	Lighthouse Lab in Glasgow	Wellcome Sanger Institute for the COVID-19 Genomics UK (COG-UK) Consortium	Harper VanSteenhouse, Yumi Kasai, David Gray, Carol Clugston, Anna Dominiczak and Alex Alderton, Roberto Amato, Sonia Goncalves, Ewan Harrison, David K. Jackson, Ian Johnston, Dominic Kwiatkowski, Cordelia Langford, John Sillitoe on behalf of the Wellcome Sanger Institute COVID-19 Surveillance Team
EPI_ISL_720756	Lighthouse Lab in Alderley Park	Wellcome Sanger Institute for the COVID-19 Genomics UK (COG-UK) Consortium	Jacquelyn Wynn, Mairead Hyland, The Lighthouse Lab in Alderley Park and Alex Alderton, Roberto Amato, Sonia Goncalves, Ewan Harrison, David K. Jackson, Ian Johnston, Dominic Kwiatkowski, Cordelia Langford, John Sillitoe on behalf of the Wellcome Sanger Institute COVID-19 Surveillance Team
EPI_ISL_720757, EPI_ISL_720758, EPI_ISL_720759, EPI_ISL_720760	Lighthouse Lab in Milton Keynes	Wellcome Sanger Institute for the COVID-19 Genomics UK (COG-UK) Consortium	The Lighthouse Lab in Milton Keynes and Alex Alderton, Roberto Amato, Sonia Goncalves, Ewan Harrison, David K. Jackson, Ian Johnston, Dominic Kwiatkowski, Cordelia Langford, John Sillitoe on behalf of the Wellcome Sanger Institute COVID-19 Surveillance Team
EPI_ISL_720761	Lighthouse Lab in Glasgow	Wellcome Sanger Institute for the COVID-19 Genomics UK (COG-UK) Consortium	Harper VanSteenhouse, Yumi Kasai, David Gray, Carol Clugston, Anna Dominiczak and Alex Alderton, Roberto Amato, Sonia Goncalves, Ewan Harrison, David K. Jackson, Ian Johnston, Dominic Kwiatkowski, Cordelia Langford, John Sillitoe on behalf of the Wellcome Sanger Institute COVID-19 Surveillance Team
EPI_ISL_720763	Lighthouse Lab in Milton Keynes	Wellcome Sanger Institute for the COVID-19 Genomics UK (COG-UK) Consortium	The Lighthouse Lab in Milton Keynes and Alex Alderton, Roberto Amato, Sonia Goncalves, Ewan Harrison, David K. Jackson, Ian Johnston, Dominic Kwiatkowski, Cordelia Langford, John Sillitoe on behalf of the Wellcome Sanger Institute COVID-19 Surveillance Team
EPI_ISL_720764, EPI_ISL_720765	Lighthouse Lab in Alderley Park	Wellcome Sanger Institute for the COVID-19 Genomics UK (COG-UK) Consortium	Jacquelyn Wynn, Mairead Hyland, The Lighthouse Lab in Alderley Park and Alex Alderton, Roberto Amato, Sonia Goncalves, Ewan Harrison, David K. Jackson, Ian Johnston, Dominic Kwiatkowski, Cordelia Langford, John Sillitoe on behalf of the Wellcome Sanger Institute COVID-19 Surveillance Team
EPI_ISL_720766	Lighthouse Lab in Milton Keynes	Wellcome Sanger Institute for the COVID-19 Genomics UK (COG-UK) Consortium	The Lighthouse Lab in Milton Keynes and Alex Alderton, Roberto Amato, Sonia Goncalves, Ewan Harrison, David K. Jackson, Ian Johnston, Dominic Kwiatkowski, Cordelia Langford, John Sillitoe on behalf of the Wellcome Sanger Institute COVID-19 Surveillance Team
EPI_ISL_720767	Lighthouse Lab in Glasgow	Wellcome Sanger Institute for the COVID-19 Genomics UK (COG-UK) Consortium	Harper VanSteenhouse, Yumi Kasai, David Gray, Carol Clugston, Anna Dominiczak and Alex Alderton, Roberto Amato, Sonia Goncalves, Ewan Harrison, David K. Jackson, Ian Johnston, Dominic Kwiatkowski, Cordelia Langford, John Sillitoe on behalf of the Wellcome Sanger Institute COVID-19 Surveillance Team
EPI_ISL_720768, EPI_ISL_720769	Lighthouse Lab in Milton Keynes	Wellcome Sanger Institute for the COVID-19 Genomics UK (COG-UK) Consortium	The Lighthouse Lab in Milton Keynes and Alex Alderton, Roberto Amato, Sonia Goncalves, Ewan Harrison, David K. Jackson, Ian Johnston, Dominic Kwiatkowski, Cordelia Langford, John Sillitoe on behalf of the Wellcome Sanger Institute COVID-19 Surveillance Team
EPI_ISL_720770	Lighthouse Lab in Alderley Park	Wellcome Sanger Institute for the COVID-19 Genomics UK (COG-UK) Consortium	Jacquelyn Wynn, Mairead Hyland, The Lighthouse Lab in Alderley Park and Alex Alderton, Roberto Amato, Sonia Goncalves, Ewan Harrison, David K. Jackson, Ian Johnston, Dominic Kwiatkowski, Cordelia Langford, John Sillitoe on behalf of the Wellcome Sanger Institute COVID-19 Surveillance Team
EPI_ISL_720771, EPI_ISL_720772	Lighthouse Lab in Milton Keynes	Wellcome Sanger Institute for the COVID-19 Genomics UK (COG-UK) Consortium	The Lighthouse Lab in Milton Keynes and Alex Alderton, Roberto Amato, Sonia Goncalves, Ewan Harrison, David K. Jackson, Ian Johnston, Dominic Kwiatkowski, Cordelia Langford, John Sillitoe on behalf of the Wellcome Sanger Institute COVID-19 Surveillance Team
EPI_ISL_720773	Lighthouse Lab in Alderley Park	Wellcome Sanger Institute for the COVID-19 Genomics UK (COG-UK) Consortium	Jacquelyn Wynn, Mairead Hyland, The Lighthouse Lab in Alderley Park and Alex Alderton, Roberto Amato, Sonia Goncalves, Ewan Harrison, David K. Jackson, Ian Johnston, Dominic Kwiatkowski, Cordelia Langford, John Sillitoe on behalf of the Wellcome Sanger Institute COVID-19 Surveillance Team
EPI_ISL_720774, EPI_ISL_720775	Lighthouse Lab in Milton Keynes	Wellcome Sanger Institute for the COVID-19 Genomics UK (COG-UK) Consortium	The Lighthouse Lab in Milton Keynes and Alex Alderton, Roberto Amato, Sonia Goncalves, Ewan Harrison, David K. Jackson, Ian Johnston, Dominic Kwiatkowski, Cordelia Langford, John Sillitoe on behalf of the Wellcome Sanger Institute COVID-19 Surveillance Team
EPI_ISL_720776	Lighthouse Lab in Glasgow	Wellcome Sanger Institute for the COVID-19 Genomics UK (COG-UK) Consortium	Harper VanSteenhouse, Yumi Kasai, David Gray, Carol Clugston, Anna Dominiczak and Alex Alderton, Roberto Amato, Sonia Goncalves, Ewan Harrison, David K. Jackson, Ian Johnston, Dominic Kwiatkowski, Cordelia Langford, John Sillitoe on behalf of the Wellcome Sanger Institute COVID-19 Surveillance Team
EPI_ISL_720777, EPI_ISL_720778	Lighthouse Lab in Milton Keynes	Wellcome Sanger Institute for the COVID-19 Genomics UK (COG-UK) Consortium	The Lighthouse Lab in Milton Keynes and Alex Alderton, Roberto Amato, Sonia Goncalves, Ewan Harrison, David K. Jackson, Ian Johnston, Dominic Kwiatkowski, Cordelia Langford, John Sillitoe on behalf of the Wellcome Sanger Institute COVID-19 Surveillance Team
EPI_ISL_720779, EPI_ISL_720780	Lighthouse Lab in Alderley Park	Wellcome Sanger Institute for the COVID-19 Genomics UK (COG-UK) Consortium	Jacquelyn Wynn, Mairead Hyland, The Lighthouse Lab in Alderley Park and Alex Alderton, Roberto Amato, Sonia Goncalves, Ewan Harrison, David K. Jackson, Ian Johnston, Dominic Kwiatkowski, Cordelia Langford, John Sillitoe on behalf of the Wellcome Sanger Institute COVID-19 Surveillance Team
EPI_ISL_720781, EPI_ISL_720782, EPI_ISL_720783, EPI_ISL_720784, EPI_ISL_720785	Lighthouse Lab in Milton Keynes	Wellcome Sanger Institute for the COVID-19 Genomics UK (COG-UK) Consortium	The Lighthouse Lab in Milton Keynes and Alex Alderton, Roberto Amato, Sonia Goncalves, Ewan Harrison, David K. Jackson, Ian Johnston, Dominic Kwiatkowski, Cordelia Langford, John Sillitoe on behalf of the Wellcome Sanger Institute COVID-19 Surveillance Team
EPI_ISL_720786, EPI_ISL_720787	Lighthouse Lab in Glasgow	Wellcome Sanger Institute for the COVID-19 Genomics UK (COG-UK) Consortium	Harper VanSteenhouse, Yumi Kasai, David Gray, Carol Clugston, Anna Dominiczak and Alex Alderton, Roberto Amato, Sonia Goncalves, Ewan Harrison, David K. Jackson, Ian Johnston, Dominic Kwiatkowski, Cordelia Langford, John Sillitoe on behalf of the Wellcome Sanger Institute COVID-19 Surveillance Team
EPI_ISL_720788, EPI_ISL_720789, EPI_ISL_720791	Lighthouse Lab in Milton Keynes	Wellcome Sanger Institute for the COVID-19 Genomics UK (COG-UK) Consortium	The Lighthouse Lab in Milton Keynes and Alex Alderton, Roberto Amato, Sonia Goncalves, Ewan Harrison, David K. Jackson, Ian Johnston, Dominic Kwiatkowski, Cordelia Langford, John Sillitoe on behalf of the Wellcome Sanger Institute COVID-19 Surveillance Team
EPI_ISL_720792	Lighthouse Lab in Glasgow	Wellcome Sanger Institute for the COVID-19 Genomics UK (COG-UK) Consortium	Harper VanSteenhouse, Yumi Kasai, David Gray, Carol Clugston, Anna Dominiczak and Alex Alderton, Roberto Amato, Sonia Goncalves, Ewan Harrison, David K. Jackson, Ian Johnston, Dominic Kwiatkowski, Cordelia Langford, John Sillitoe on behalf of the Wellcome Sanger Institute COVID-19 Surveillance Team
EPI_ISL_720793, EPI_ISL_720794, EPI_ISL_720795	Lighthouse Lab in Milton Keynes	Wellcome Sanger Institute for the COVID-19 Genomics UK (COG-UK) Consortium	The Lighthouse Lab in Milton Keynes and Alex Alderton, Roberto Amato, Sonia Goncalves, Ewan Harrison, David K. Jackson, Ian Johnston, Dominic Kwiatkowski, Cordelia Langford, John Sillitoe on behalf of the Wellcome Sanger Institute COVID-19 Surveillance Team
EPI_ISL_720796	Lighthouse Lab in Alderley Park	Wellcome Sanger Institute for the COVID-19 Genomics UK	Jacquelyn Wynn, Mairead Hyland, The Lighthouse Lab in Alderley Park and Alex Alderton, Roberto Amato, Sonia Goncalves, Ewan Harrison, David K.

		(COG-UK) Consortium	Jackson, Ian Johnston, Dominic Kwiatkowski, Cordelia Langford, John Sillitoe on behalf of the Wellcome Sanger Institute COVID-19 Surveillance Team
EPI_ISL_720797, EPI_ISL_720798, EPI_ISL_720799, EPI_ISL_720800, EPI_ISL_720801	Lighthouse Lab in Milton Keynes	Wellcome Sanger Institute for the COVID-19 Genomics UK (COG-UK) Consortium	The Lighthouse Lab in Milton Keynes and Alex Alderton, Roberto Amato, Sonia Goncalves, Ewan Harrison, David K. Jackson, Ian Johnston, Dominic Kwiatkowski, Cordelia Langford, John Sillitoe on behalf of the Wellcome Sanger Institute COVID-19 Surveillance Team
EPI_ISL_720802	Lighthouse Lab in Glasgow	Wellcome Sanger Institute for the COVID-19 Genomics UK (COG-UK) Consortium	Harper VanSteenhouse, Yumi Kasai, David Gray, Carol Clugston, Anna Dominiczak and Alex Alderton, Roberto Amato, Sonia Goncalves, Ewan Harrison, David K. Jackson, Ian Johnston, Dominic Kwiatkowski, Cordelia Langford, John Sillitoe on behalf of the Wellcome Sanger Institute COVID-19 Surveillance Team
EPI_ISL_720803, EPI_ISL_720804	Lighthouse Lab in Milton Keynes	Wellcome Sanger Institute for the COVID-19 Genomics UK (COG-UK) Consortium	The Lighthouse Lab in Milton Keynes and Alex Alderton, Roberto Amato, Sonia Goncalves, Ewan Harrison, David K. Jackson, Ian Johnston, Dominic Kwiatkowski, Cordelia Langford, John Sillitoe on behalf of the Wellcome Sanger Institute COVID-19 Surveillance Team
EPI_ISL_720805	Lighthouse Lab in Glasgow	Wellcome Sanger Institute for the COVID-19 Genomics UK (COG-UK) Consortium	Harper VanSteenhouse, Yumi Kasai, David Gray, Carol Clugston, Anna Dominiczak and Alex Alderton, Roberto Amato, Sonia Goncalves, Ewan Harrison, David K. Jackson, Ian Johnston, Dominic Kwiatkowski, Cordelia Langford, John Sillitoe on behalf of the Wellcome Sanger Institute COVID-19 Surveillance Team
EPI_ISL_720806, EPI_ISL_720807, EPI_ISL_720808, EPI_ISL_720809	Lighthouse Lab in Milton Keynes	Wellcome Sanger Institute for the COVID-19 Genomics UK (COG-UK) Consortium	The Lighthouse Lab in Milton Keynes and Alex Alderton, Roberto Amato, Sonia Goncalves, Ewan Harrison, David K. Jackson, Ian Johnston, Dominic Kwiatkowski, Cordelia Langford, John Sillitoe on behalf of the Wellcome Sanger Institute COVID-19 Surveillance Team
EPI_ISL_720810	Lighthouse Lab in Alderley Park	Wellcome Sanger Institute for the COVID-19 Genomics UK (COG-UK) Consortium	Jacquelyn Wynn, Mairead Hyland, The Lighthouse Lab in Alderley Park and Alex Alderton, Roberto Amato, Sonia Goncalves, Ewan Harrison, David K. Jackson, Ian Johnston, Dominic Kwiatkowski, Cordelia Langford, John Sillitoe on behalf of the Wellcome Sanger Institute COVID-19 Surveillance Team
EPI_ISL_720811	Lighthouse Lab in Glasgow	Wellcome Sanger Institute for the COVID-19 Genomics UK (COG-UK) Consortium	Harper VanSteenhouse, Yumi Kasai, David Gray, Carol Clugston, Anna Dominiczak and Alex Alderton, Roberto Amato, Sonia Goncalves, Ewan Harrison, David K. Jackson, Ian Johnston, Dominic Kwiatkowski, Cordelia Langford, John Sillitoe on behalf of the Wellcome Sanger Institute COVID-19 Surveillance Team
EPI_ISL_720812, EPI_ISL_720813	Lighthouse Lab in Milton Keynes	Wellcome Sanger Institute for the COVID-19 Genomics UK (COG-UK) Consortium	The Lighthouse Lab in Milton Keynes and Alex Alderton, Roberto Amato, Sonia Goncalves, Ewan Harrison, David K. Jackson, Ian Johnston, Dominic Kwiatkowski, Cordelia Langford, John Sillitoe on behalf of the Wellcome Sanger Institute COVID-19 Surveillance Team
EPI_ISL_720814, EPI_ISL_720816	Lighthouse Lab in Alderley Park	Wellcome Sanger Institute for the COVID-19 Genomics UK (COG-UK) Consortium	Jacquelyn Wynn, Mairead Hyland, The Lighthouse Lab in Alderley Park and Alex Alderton, Roberto Amato, Sonia Goncalves, Ewan Harrison, David K. Jackson, Ian Johnston, Dominic Kwiatkowski, Cordelia Langford, John Sillitoe on behalf of the Wellcome Sanger Institute COVID-19 Surveillance Team
EPI_ISL_720817, EPI_ISL_720818	Lighthouse Lab in Milton Keynes	Wellcome Sanger Institute for the COVID-19 Genomics UK (COG-UK) Consortium	The Lighthouse Lab in Milton Keynes and Alex Alderton, Roberto Amato, Sonia Goncalves, Ewan Harrison, David K. Jackson, Ian Johnston, Dominic Kwiatkowski, Cordelia Langford, John Sillitoe on behalf of the Wellcome Sanger Institute COVID-19 Surveillance Team
EPI_ISL_720819	Lighthouse Lab in Glasgow	Wellcome Sanger Institute for the COVID-19 Genomics UK (COG-UK) Consortium	Harper VanSteenhouse, Yumi Kasai, David Gray, Carol Clugston, Anna Dominiczak and Alex Alderton, Roberto Amato, Sonia Goncalves, Ewan Harrison, David K. Jackson, Ian Johnston, Dominic Kwiatkowski, Cordelia Langford, John Sillitoe on behalf of the Wellcome Sanger Institute COVID-19 Surveillance Team
EPI_ISL_720820, EPI_ISL_720821, EPI_ISL_720822	Lighthouse Lab in Milton Keynes	Wellcome Sanger Institute for the COVID-19 Genomics UK (COG-UK) Consortium	The Lighthouse Lab in Milton Keynes and Alex Alderton, Roberto Amato, Sonia Goncalves, Ewan Harrison, David K. Jackson, Ian Johnston, Dominic Kwiatkowski, Cordelia Langford, John Sillitoe on behalf of the Wellcome Sanger Institute COVID-19 Surveillance Team
EPI_ISL_720823	Lighthouse Lab in Alderley Park	Wellcome Sanger Institute for the COVID-19 Genomics UK (COG-UK) Consortium	Jacquelyn Wynn, Mairead Hyland, The Lighthouse Lab in Alderley Park and Alex Alderton, Roberto Amato, Sonia Goncalves, Ewan Harrison, David K. Jackson, Ian Johnston, Dominic Kwiatkowski, Cordelia Langford, John Sillitoe on behalf of the Wellcome Sanger Institute COVID-19 Surveillance Team
EPI_ISL_720824	Lighthouse Lab in Milton Keynes	Wellcome Sanger Institute for the COVID-19 Genomics UK (COG-UK) Consortium	The Lighthouse Lab in Milton Keynes and Alex Alderton, Roberto Amato, Sonia Goncalves, Ewan Harrison, David K. Jackson, Ian Johnston, Dominic Kwiatkowski, Cordelia Langford, John Sillitoe on behalf of the Wellcome Sanger Institute COVID-19 Surveillance Team
EPI_ISL_720825	Lighthouse Lab in Alderley Park	Wellcome Sanger Institute for the COVID-19 Genomics UK (COG-UK) Consortium	Jacquelyn Wynn, Mairead Hyland, The Lighthouse Lab in Alderley Park and Alex Alderton, Roberto Amato, Sonia Goncalves, Ewan Harrison, David K. Jackson, Ian Johnston, Dominic Kwiatkowski, Cordelia Langford, John Sillitoe on behalf of the Wellcome Sanger Institute COVID-19 Surveillance Team
EPI_ISL_720826	Lighthouse Lab in Milton Keynes	Wellcome Sanger Institute for the COVID-19 Genomics UK (COG-UK) Consortium	The Lighthouse Lab in Milton Keynes and Alex Alderton, Roberto Amato, Sonia Goncalves, Ewan Harrison, David K. Jackson, Ian Johnston, Dominic Kwiatkowski, Cordelia Langford, John Sillitoe on behalf of the Wellcome Sanger Institute COVID-19 Surveillance Team
EPI_ISL_720827	Lighthouse Lab in Alderley Park	Wellcome Sanger Institute for the COVID-19 Genomics UK (COG-UK) Consortium	Jacquelyn Wynn, Mairead Hyland, The Lighthouse Lab in Alderley Park and Alex Alderton, Roberto Amato, Sonia Goncalves, Ewan Harrison, David K. Jackson, Ian Johnston, Dominic Kwiatkowski, Cordelia Langford, John Sillitoe on behalf of the Wellcome Sanger Institute COVID-19 Surveillance Team
EPI_ISL_720828, EPI_ISL_720829, EPI_ISL_720830	Lighthouse Lab in Milton Keynes	Wellcome Sanger Institute for the COVID-19 Genomics UK (COG-UK) Consortium	The Lighthouse Lab in Milton Keynes and Alex Alderton, Roberto Amato, Sonia Goncalves, Ewan Harrison, David K. Jackson, Ian Johnston, Dominic Kwiatkowski, Cordelia Langford, John Sillitoe on behalf of the Wellcome Sanger Institute COVID-19 Surveillance Team
EPI_ISL_720831	Lighthouse Lab in Glasgow	Wellcome Sanger Institute for the COVID-19 Genomics UK (COG-UK) Consortium	Harper VanSteenhouse, Yumi Kasai, David Gray, Carol Clugston, Anna Dominiczak and Alex Alderton, Roberto Amato, Sonia Goncalves, Ewan Harrison, David K. Jackson, Ian Johnston, Dominic Kwiatkowski, Cordelia Langford, John Sillitoe on behalf of the Wellcome Sanger Institute COVID-19 Surveillance Team
EPI_ISL_720832, EPI_ISL_720833	Lighthouse Lab in Milton Keynes	Wellcome Sanger Institute for the COVID-19 Genomics UK (COG-UK) Consortium	The Lighthouse Lab in Milton Keynes and Alex Alderton, Roberto Amato, Sonia Goncalves, Ewan Harrison, David K. Jackson, Ian Johnston, Dominic Kwiatkowski, Cordelia Langford, John Sillitoe on behalf of the Wellcome Sanger Institute COVID-19 Surveillance Team
EPI_ISL_720834	Lighthouse Lab in Glasgow	Wellcome Sanger Institute for the COVID-19 Genomics UK (COG-UK) Consortium	Harper VanSteenhouse, Yumi Kasai, David Gray, Carol Clugston, Anna Dominiczak and Alex Alderton, Roberto Amato, Sonia Goncalves, Ewan Harrison, David K. Jackson, Ian Johnston, Dominic Kwiatkowski, Cordelia Langford, John Sillitoe on behalf of the Wellcome Sanger Institute COVID-19 Surveillance Team
EPI_ISL_720835	Lighthouse Lab in Milton Keynes	Wellcome Sanger Institute for the COVID-19 Genomics UK (COG-UK) Consortium	The Lighthouse Lab in Milton Keynes and Alex Alderton, Roberto Amato, Sonia Goncalves, Ewan Harrison, David K. Jackson, Ian Johnston, Dominic Kwiatkowski, Cordelia Langford, John Sillitoe on behalf of the Wellcome Sanger Institute COVID-19 Surveillance Team
EPI_ISL_720836	Lighthouse Lab in Alderley Park	Wellcome Sanger Institute for the COVID-19 Genomics UK (COG-UK) Consortium	Jacquelyn Wynn, Mairead Hyland, The Lighthouse Lab in Alderley Park and Alex Alderton, Roberto Amato, Sonia Goncalves, Ewan Harrison, David K. Jackson, Ian Johnston, Dominic Kwiatkowski, Cordelia Langford, John Sillitoe on behalf of the Wellcome Sanger Institute COVID-19 Surveillance Team
EPI_ISL_720838, EPI_ISL_720839, EPI_ISL_720840	Lighthouse Lab in Milton Keynes	Wellcome Sanger Institute for the COVID-19 Genomics UK (COG-UK) Consortium	The Lighthouse Lab in Milton Keynes and Alex Alderton, Roberto Amato, Sonia Goncalves, Ewan Harrison, David K. Jackson, Ian Johnston, Dominic Kwiatkowski, Cordelia Langford, John Sillitoe on behalf of the Wellcome Sanger Institute COVID-19 Surveillance Team
EPI_ISL_720841	Lighthouse Lab in Glasgow	Wellcome Sanger Institute for the COVID-19 Genomics UK (COG-UK) Consortium	Harper VanSteenhouse, Yumi Kasai, David Gray, Carol Clugston, Anna Dominiczak and Alex Alderton, Roberto Amato, Sonia Goncalves, Ewan Harrison, David K. Jackson, Ian Johnston, Dominic Kwiatkowski, Cordelia Langford, John Sillitoe on behalf of the Wellcome Sanger Institute COVID-19 Surveillance Team
EPI_ISL_720842	Lighthouse Lab in Alderley Park	Wellcome Sanger Institute for the COVID-19 Genomics UK (COG-UK) Consortium	Jacquelyn Wynn, Mairead Hyland, The Lighthouse Lab in Alderley Park and Alex Alderton, Roberto Amato, Sonia Goncalves, Ewan Harrison, David K. Jackson, Ian Johnston, Dominic Kwiatkowski, Cordelia Langford, John Sillitoe on behalf of the Wellcome Sanger Institute COVID-19 Surveillance Team
EPI_ISL_720843, EPI_ISL_720844	Lighthouse Lab in Milton Keynes	Wellcome Sanger Institute for the COVID-19 Genomics UK (COG-UK) Consortium	The Lighthouse Lab in Milton Keynes and Alex Alderton, Roberto Amato, Sonia Goncalves, Ewan Harrison, David K. Jackson, Ian Johnston, Dominic Kwiatkowski, Cordelia Langford, John Sillitoe on behalf of the Wellcome Sanger Institute COVID-19 Surveillance Team
EPI_ISL_720845, EPI_ISL_720846	Lighthouse Lab in Alderley Park	Wellcome Sanger Institute for the COVID-19 Genomics UK (COG-UK) Consortium	Jacquelyn Wynn, Mairead Hyland, The Lighthouse Lab in Alderley Park and Alex Alderton, Roberto Amato, Sonia Goncalves, Ewan Harrison, David K. Jackson, Ian Johnston, Dominic Kwiatkowski, Cordelia Langford, John Sillitoe on behalf of the Wellcome Sanger Institute COVID-19 Surveillance Team
EPI_ISL_720847	Lighthouse Lab in Milton Keynes	Wellcome Sanger Institute for the COVID-19 Genomics UK (COG-UK) Consortium	The Lighthouse Lab in Milton Keynes and Alex Alderton, Roberto Amato, Sonia Goncalves, Ewan Harrison, David K. Jackson, Ian Johnston, Dominic Kwiatkowski, Cordelia Langford, John Sillitoe on behalf of the Wellcome Sanger Institute COVID-19 Surveillance Team
EPI_ISL_720848, EPI_ISL_720849	Lighthouse Lab in Alderley Park	Wellcome Sanger Institute for the COVID-19 Genomics UK (COG-UK) Consortium	Jacquelyn Wynn, Mairead Hyland, The Lighthouse Lab in Alderley Park and Alex Alderton, Roberto Amato, Sonia Goncalves, Ewan Harrison, David K. Jackson, Ian Johnston, Dominic Kwiatkowski, Cordelia Langford, John Sillitoe on behalf of the Wellcome Sanger Institute COVID-19 Surveillance Team
EPI_ISL_720850	Lighthouse Lab in Glasgow	Wellcome Sanger Institute for the COVID-19 Genomics UK (COG-UK) Consortium	Harper VanSteenhouse, Yumi Kasai, David Gray, Carol Clugston, Anna Dominiczak and Alex Alderton, Roberto Amato, Sonia Goncalves, Ewan Harrison, David K. Jackson, Ian Johnston, Dominic Kwiatkowski, Cordelia Langford, John Sillitoe on behalf of the Wellcome Sanger Institute COVID-19 Surveillance Team

EPI_ISL_720851, EPI_ISL_720852	Lighthouse Lab in Milton Keynes	Wellcome Sanger Institute for the COVID-19 Genomics UK (COG-UK) Consortium	The Lighthouse Lab in Milton Keynes and Alex Alderton, Roberto Amato, Sonia Goncalves, Ewan Harrison, David K. Jackson, Ian Johnston, Dominic Kwiatkowski, Cordelia Langford, John Sillitoe on behalf of the Wellcome Sanger Institute COVID-19 Surveillance Team
EPI_ISL_720853	Lighthouse Lab in Alderley Park	Wellcome Sanger Institute for the COVID-19 Genomics UK (COG-UK) Consortium	Jacquelyn Wynn, Mairead Hyland, The Lighthouse Lab in Alderley Park and Alex Alderton, Roberto Amato, Sonia Goncalves, Ewan Harrison, David K. Jackson, Ian Johnston, Dominic Kwiatkowski, Cordelia Langford, John Sillitoe on behalf of the Wellcome Sanger Institute COVID-19 Surveillance Team
EPI_ISL_720854, EPI_ISL_720855	Lighthouse Lab in Milton Keynes	Wellcome Sanger Institute for the COVID-19 Genomics UK (COG-UK) Consortium	The Lighthouse Lab in Milton Keynes and Alex Alderton, Roberto Amato, Sonia Goncalves, Ewan Harrison, David K. Jackson, Ian Johnston, Dominic Kwiatkowski, Cordelia Langford, John Sillitoe on behalf of the Wellcome Sanger Institute COVID-19 Surveillance Team
EPI_ISL_720856	Lighthouse Lab in Glasgow	Wellcome Sanger Institute for the COVID-19 Genomics UK (COG-UK) Consortium	Harper VanSteenhouse, Yumi Kasai, David Gray, Carol Clugston, Anna Dominiczak and Alex Alderton, Roberto Amato, Sonia Goncalves, Ewan Harrison, David K. Jackson, Ian Johnston, Dominic Kwiatkowski, Cordelia Langford, John Sillitoe on behalf of the Wellcome Sanger Institute COVID-19 Surveillance Team
EPI_ISL_720857, EPI_ISL_720858	Lighthouse Lab in Alderley Park	Wellcome Sanger Institute for the COVID-19 Genomics UK (COG-UK) Consortium	Jacquelyn Wynn, Mairead Hyland, The Lighthouse Lab in Alderley Park and Alex Alderton, Roberto Amato, Sonia Goncalves, Ewan Harrison, David K. Jackson, Ian Johnston, Dominic Kwiatkowski, Cordelia Langford, John Sillitoe on behalf of the Wellcome Sanger Institute COVID-19 Surveillance Team
EPI_ISL_720859, EPI_ISL_720860, EPI_ISL_720861, EPI_ISL_720862, EPI_ISL_720863	Lighthouse Lab in Milton Keynes	Wellcome Sanger Institute for the COVID-19 Genomics UK (COG-UK) Consortium	The Lighthouse Lab in Milton Keynes and Alex Alderton, Roberto Amato, Sonia Goncalves, Ewan Harrison, David K. Jackson, Ian Johnston, Dominic Kwiatkowski, Cordelia Langford, John Sillitoe on behalf of the Wellcome Sanger Institute COVID-19 Surveillance Team
EPI_ISL_720864	Lighthouse Lab in Alderley Park	Wellcome Sanger Institute for the COVID-19 Genomics UK (COG-UK) Consortium	Jacquelyn Wynn, Mairead Hyland, The Lighthouse Lab in Alderley Park and Alex Alderton, Roberto Amato, Sonia Goncalves, Ewan Harrison, David K. Jackson, Ian Johnston, Dominic Kwiatkowski, Cordelia Langford, John Sillitoe on behalf of the Wellcome Sanger Institute COVID-19 Surveillance Team
EPI_ISL_720865, EPI_ISL_720866	Lighthouse Lab in Milton Keynes	Wellcome Sanger Institute for the COVID-19 Genomics UK (COG-UK) Consortium	The Lighthouse Lab in Milton Keynes and Alex Alderton, Roberto Amato, Sonia Goncalves, Ewan Harrison, David K. Jackson, Ian Johnston, Dominic Kwiatkowski, Cordelia Langford, John Sillitoe on behalf of the Wellcome Sanger Institute COVID-19 Surveillance Team
EPI_ISL_720867	Lighthouse Lab in Glasgow	Wellcome Sanger Institute for the COVID-19 Genomics UK (COG-UK) Consortium	Harper VanSteenhouse, Yumi Kasai, David Gray, Carol Clugston, Anna Dominiczak and Alex Alderton, Roberto Amato, Sonia Goncalves, Ewan Harrison, David K. Jackson, Ian Johnston, Dominic Kwiatkowski, Cordelia Langford, John Sillitoe on behalf of the Wellcome Sanger Institute COVID-19 Surveillance Team
EPI_ISL_720868	Lighthouse Lab in Milton Keynes	Wellcome Sanger Institute for the COVID-19 Genomics UK (COG-UK) Consortium	The Lighthouse Lab in Milton Keynes and Alex Alderton, Roberto Amato, Sonia Goncalves, Ewan Harrison, David K. Jackson, Ian Johnston, Dominic Kwiatkowski, Cordelia Langford, John Sillitoe on behalf of the Wellcome Sanger Institute COVID-19 Surveillance Team
EPI_ISL_720869	Lighthouse Lab in Alderley Park	Wellcome Sanger Institute for the COVID-19 Genomics UK (COG-UK) Consortium	Jacquelyn Wynn, Mairead Hyland, The Lighthouse Lab in Alderley Park and Alex Alderton, Roberto Amato, Sonia Goncalves, Ewan Harrison, David K. Jackson, Ian Johnston, Dominic Kwiatkowski, Cordelia Langford, John Sillitoe on behalf of the Wellcome Sanger Institute COVID-19 Surveillance Team
EPI_ISL_720870	Lighthouse Lab in Glasgow	Wellcome Sanger Institute for the COVID-19 Genomics UK (COG-UK) Consortium	Harper VanSteenhouse, Yumi Kasai, David Gray, Carol Clugston, Anna Dominiczak and Alex Alderton, Roberto Amato, Sonia Goncalves, Ewan Harrison, David K. Jackson, Ian Johnston, Dominic Kwiatkowski, Cordelia Langford, John Sillitoe on behalf of the Wellcome Sanger Institute COVID-19 Surveillance Team
EPI_ISL_720871	Lighthouse Lab in Milton Keynes	Wellcome Sanger Institute for the COVID-19 Genomics UK (COG-UK) Consortium	The Lighthouse Lab in Milton Keynes and Alex Alderton, Roberto Amato, Sonia Goncalves, Ewan Harrison, David K. Jackson, Ian Johnston, Dominic Kwiatkowski, Cordelia Langford, John Sillitoe on behalf of the Wellcome Sanger Institute COVID-19 Surveillance Team
EPI_ISL_720872	Lighthouse Lab in Alderley Park	Wellcome Sanger Institute for the COVID-19 Genomics UK (COG-UK) Consortium	Jacquelyn Wynn, Mairead Hyland, The Lighthouse Lab in Alderley Park and Alex Alderton, Roberto Amato, Sonia Goncalves, Ewan Harrison, David K. Jackson, Ian Johnston, Dominic Kwiatkowski, Cordelia Langford, John Sillitoe on behalf of the Wellcome Sanger Institute COVID-19 Surveillance Team
EPI_ISL_720873, EPI_ISL_720874	Lighthouse Lab in Milton Keynes	Wellcome Sanger Institute for the COVID-19 Genomics UK (COG-UK) Consortium	The Lighthouse Lab in Milton Keynes and Alex Alderton, Roberto Amato, Sonia Goncalves, Ewan Harrison, David K. Jackson, Ian Johnston, Dominic Kwiatkowski, Cordelia Langford, John Sillitoe on behalf of the Wellcome Sanger Institute COVID-19 Surveillance Team
EPI_ISL_720875	Lighthouse Lab in Alderley Park	Wellcome Sanger Institute for the COVID-19 Genomics UK (COG-UK) Consortium	Jacquelyn Wynn, Mairead Hyland, The Lighthouse Lab in Alderley Park and Alex Alderton, Roberto Amato, Sonia Goncalves, Ewan Harrison, David K. Jackson, Ian Johnston, Dominic Kwiatkowski, Cordelia Langford, John Sillitoe on behalf of the Wellcome Sanger Institute COVID-19 Surveillance Team
EPI_ISL_720876	Lighthouse Lab in Milton Keynes	Wellcome Sanger Institute for the COVID-19 Genomics UK (COG-UK) Consortium	The Lighthouse Lab in Milton Keynes and Alex Alderton, Roberto Amato, Sonia Goncalves, Ewan Harrison, David K. Jackson, Ian Johnston, Dominic Kwiatkowski, Cordelia Langford, John Sillitoe on behalf of the Wellcome Sanger Institute COVID-19 Surveillance Team
EPI_ISL_720877	Lighthouse Lab in Glasgow	Wellcome Sanger Institute for the COVID-19 Genomics UK (COG-UK) Consortium	Harper VanSteenhouse, Yumi Kasai, David Gray, Carol Clugston, Anna Dominiczak and Alex Alderton, Roberto Amato, Sonia Goncalves, Ewan Harrison, David K. Jackson, Ian Johnston, Dominic Kwiatkowski, Cordelia Langford, John Sillitoe on behalf of the Wellcome Sanger Institute COVID-19 Surveillance Team
EPI_ISL_720878, EPI_ISL_720879	Lighthouse Lab in Milton Keynes	Wellcome Sanger Institute for the COVID-19 Genomics UK (COG-UK) Consortium	The Lighthouse Lab in Milton Keynes and Alex Alderton, Roberto Amato, Sonia Goncalves, Ewan Harrison, David K. Jackson, Ian Johnston, Dominic Kwiatkowski, Cordelia Langford, John Sillitoe on behalf of the Wellcome Sanger Institute COVID-19 Surveillance Team
EPI_ISL_720880	Lighthouse Lab in Alderley Park	Wellcome Sanger Institute for the COVID-19 Genomics UK (COG-UK) Consortium	Jacquelyn Wynn, Mairead Hyland, The Lighthouse Lab in Alderley Park and Alex Alderton, Roberto Amato, Sonia Goncalves, Ewan Harrison, David K. Jackson, Ian Johnston, Dominic Kwiatkowski, Cordelia Langford, John Sillitoe on behalf of the Wellcome Sanger Institute COVID-19 Surveillance Team
EPI_ISL_720881, EPI_ISL_720882, EPI_ISL_720883, EPI_ISL_720884	Lighthouse Lab in Milton Keynes	Wellcome Sanger Institute for the COVID-19 Genomics UK (COG-UK) Consortium	The Lighthouse Lab in Milton Keynes and Alex Alderton, Roberto Amato, Sonia Goncalves, Ewan Harrison, David K. Jackson, Ian Johnston, Dominic Kwiatkowski, Cordelia Langford, John Sillitoe on behalf of the Wellcome Sanger Institute COVID-19 Surveillance Team
EPI_ISL_720885, EPI_ISL_720886	Lighthouse Lab in Alderley Park	Wellcome Sanger Institute for the COVID-19 Genomics UK (COG-UK) Consortium	Jacquelyn Wynn, Mairead Hyland, The Lighthouse Lab in Alderley Park and Alex Alderton, Roberto Amato, Sonia Goncalves, Ewan Harrison, David K. Jackson, Ian Johnston, Dominic Kwiatkowski, Cordelia Langford, John Sillitoe on behalf of the Wellcome Sanger Institute COVID-19 Surveillance Team
EPI_ISL_720887, EPI_ISL_720888	Lighthouse Lab in Milton Keynes	Wellcome Sanger Institute for the COVID-19 Genomics UK (COG-UK) Consortium	The Lighthouse Lab in Milton Keynes and Alex Alderton, Roberto Amato, Sonia Goncalves, Ewan Harrison, David K. Jackson, Ian Johnston, Dominic Kwiatkowski, Cordelia Langford, John Sillitoe on behalf of the Wellcome Sanger Institute COVID-19 Surveillance Team
EPI_ISL_720889	Lighthouse Lab in Glasgow	Wellcome Sanger Institute for the COVID-19 Genomics UK (COG-UK) Consortium	Harper VanSteenhouse, Yumi Kasai, David Gray, Carol Clugston, Anna Dominiczak and Alex Alderton, Roberto Amato, Sonia Goncalves, Ewan Harrison, David K. Jackson, Ian Johnston, Dominic Kwiatkowski, Cordelia Langford, John Sillitoe on behalf of the Wellcome Sanger Institute COVID-19 Surveillance Team
EPI_ISL_720890, EPI_ISL_720892	Lighthouse Lab in Milton Keynes	Wellcome Sanger Institute for the COVID-19 Genomics UK (COG-UK) Consortium	The Lighthouse Lab in Milton Keynes and Alex Alderton, Roberto Amato, Sonia Goncalves, Ewan Harrison, David K. Jackson, Ian Johnston, Dominic Kwiatkowski, Cordelia Langford, John Sillitoe on behalf of the Wellcome Sanger Institute COVID-19 Surveillance Team
EPI_ISL_720893, EPI_ISL_720894	Lighthouse Lab in Alderley Park	Wellcome Sanger Institute for the COVID-19 Genomics UK (COG-UK) Consortium	Jacquelyn Wynn, Mairead Hyland, The Lighthouse Lab in Alderley Park and Alex Alderton, Roberto Amato, Sonia Goncalves, Ewan Harrison, David K. Jackson, Ian Johnston, Dominic Kwiatkowski, Cordelia Langford, John Sillitoe on behalf of the Wellcome Sanger Institute COVID-19 Surveillance Team
EPI_ISL_720895, EPI_ISL_720896	Lighthouse Lab in Milton Keynes	Wellcome Sanger Institute for the COVID-19 Genomics UK (COG-UK) Consortium	The Lighthouse Lab in Milton Keynes and Alex Alderton, Roberto Amato, Sonia Goncalves, Ewan Harrison, David K. Jackson, Ian Johnston, Dominic Kwiatkowski, Cordelia Langford, John Sillitoe on behalf of the Wellcome Sanger Institute COVID-19 Surveillance Team
EPI_ISL_720897	Lighthouse Lab in Glasgow	Wellcome Sanger Institute for the COVID-19 Genomics UK (COG-UK) Consortium	Harper VanSteenhouse, Yumi Kasai, David Gray, Carol Clugston, Anna Dominiczak and Alex Alderton, Roberto Amato, Sonia Goncalves, Ewan Harrison, David K. Jackson, Ian Johnston, Dominic Kwiatkowski, Cordelia Langford, John Sillitoe on behalf of the Wellcome Sanger Institute COVID-19 Surveillance Team
EPI_ISL_720898	Lighthouse Lab in Alderley Park	Wellcome Sanger Institute for the COVID-19 Genomics UK (COG-UK) Consortium	Jacquelyn Wynn, Mairead Hyland, The Lighthouse Lab in Alderley Park and Alex Alderton, Roberto Amato, Sonia Goncalves, Ewan Harrison, David K. Jackson, Ian Johnston, Dominic Kwiatkowski, Cordelia Langford, John Sillitoe on behalf of the Wellcome Sanger Institute COVID-19 Surveillance Team
EPI_ISL_720899	Lighthouse Lab in Milton Keynes	Wellcome Sanger Institute for the COVID-19 Genomics UK (COG-UK) Consortium	The Lighthouse Lab in Milton Keynes and Alex Alderton, Roberto Amato, Sonia Goncalves, Ewan Harrison, David K. Jackson, Ian Johnston, Dominic Kwiatkowski, Cordelia Langford, John Sillitoe on behalf of the Wellcome Sanger Institute COVID-19 Surveillance Team
EPI_ISL_720900	Lighthouse Lab in Alderley Park	Wellcome Sanger Institute for the COVID-19 Genomics UK (COG-UK) Consortium	Jacquelyn Wynn, Mairead Hyland, The Lighthouse Lab in Alderley Park and Alex Alderton, Roberto Amato, Sonia Goncalves, Ewan Harrison, David K. Jackson, Ian Johnston, Dominic Kwiatkowski, Cordelia Langford, John Sillitoe on behalf of the Wellcome Sanger Institute COVID-19 Surveillance Team
EPI_ISL_720901, EPI_ISL_720902, EPI_ISL_720903	Lighthouse Lab in Milton Keynes	Wellcome Sanger Institute for the COVID-19 Genomics UK (COG-UK) Consortium	The Lighthouse Lab in Milton Keynes and Alex Alderton, Roberto Amato, Sonia Goncalves, Ewan Harrison, David K. Jackson, Ian Johnston, Dominic Kwiatkowski, Cordelia Langford, John Sillitoe on behalf of the Wellcome Sanger Institute COVID-19 Surveillance Team
EPI_ISL_720904	Lighthouse Lab in Glasgow	Wellcome Sanger Institute for the COVID-19 Genomics UK	Harper VanSteenhouse, Yumi Kasai, David Gray, Carol Clugston, Anna Dominiczak and Alex Alderton, Roberto Amato, Sonia Goncalves, Ewan Harrison,

		Wellcome Sanger Institute for the COVID-19 Genomics UK (COG-UK) Consortium	David K. Jackson, Ian Johnston, Dominic Kwiatkowski, Cordelia Langford, John Sillitoe on behalf of the Wellcome Sanger Institute COVID-19 Surveillance Team
EPI_ISL_720905, EPI_ISL_720906	Lighthouse Lab in Milton Keynes	Wellcome Sanger Institute for the COVID-19 Genomics UK (COG-UK) Consortium	The Lighthouse Lab in Milton Keynes and Alex Alderton, Roberto Amato, Sonia Goncalves, Ewan Harrison, David K. Jackson, Ian Johnston, Dominic Kwiatkowski, Cordelia Langford, John Sillitoe on behalf of the Wellcome Sanger Institute COVID-19 Surveillance Team
EPI_ISL_720907	Lighthouse Lab in Alderley Park	Wellcome Sanger Institute for the COVID-19 Genomics UK (COG-UK) Consortium	Jacquelyn Wynn, Mairead Hyland, The Lighthouse Lab in Alderley Park and Alex Alderton, Roberto Amato, Sonia Goncalves, Ewan Harrison, David K. Jackson, Ian Johnston, Dominic Kwiatkowski, Cordelia Langford, John Sillitoe on behalf of the Wellcome Sanger Institute COVID-19 Surveillance Team
EPI_ISL_720908	Lighthouse Lab in Milton Keynes	Wellcome Sanger Institute for the COVID-19 Genomics UK (COG-UK) Consortium	The Lighthouse Lab in Milton Keynes and Alex Alderton, Roberto Amato, Sonia Goncalves, Ewan Harrison, David K. Jackson, Ian Johnston, Dominic Kwiatkowski, Cordelia Langford, John Sillitoe on behalf of the Wellcome Sanger Institute COVID-19 Surveillance Team
EPI_ISL_720909, EPI_ISL_720910, EPI_ISL_720911, EPI_ISL_720912, EPI_ISL_720913, EPI_ISL_720914, EPI_ISL_720915, EPI_ISL_720916, EPI_ISL_720917, EPI_ISL_720918, EPI_ISL_720919, EPI_ISL_720920, EPI_ISL_720921, EPI_ISL_720922, EPI_ISL_720923, EPI_ISL_720924, EPI_ISL_720925, EPI_ISL_720926, EPI_ISL_720927, EPI_ISL_720928, EPI_ISL_720929, EPI_ISL_720930, EPI_ISL_720931, EPI_ISL_720932, EPI_ISL_720933, EPI_ISL_720934, EPI_ISL_720935, EPI_ISL_720936, EPI_ISL_720937, EPI_ISL_720938, EPI_ISL_720939, EPI_ISL_720940, EPI_ISL_720941, EPI_ISL_720942, EPI_ISL_720943, EPI_ISL_720944, EPI_ISL_720945, EPI_ISL_720946, EPI_ISL_720947, EPI_ISL_720948, EPI_ISL_720949, EPI_ISL_720950, EPI_ISL_720951, EPI_ISL_720952, EPI_ISL_720953, EPI_ISL_720954, EPI_ISL_720955, EPI_ISL_720956, EPI_ISL_720957, EPI_ISL_720958, EPI_ISL_720959, EPI_ISL_720960, EPI_ISL_720961, EPI_ISL_720962, EPI_ISL_720963, EPI_ISL_720964, EPI_ISL_720965, EPI_ISL_720966, EPI_ISL_720967, EPI_ISL_720968, EPI_ISL_720969, EPI_ISL_720970, EPI_ISL_720971, EPI_ISL_720972, EPI_ISL_720973, EPI_ISL_720974, EPI_ISL_720975, EPI_ISL_720976, EPI_ISL_720977, EPI_ISL_720978, EPI_ISL_720979, EPI_ISL_720980, EPI_ISL_720981, EPI_ISL_720982, EPI_ISL_720983, EPI_ISL_720984, EPI_ISL_720985, EPI_ISL_720986, EPI_ISL_720987, EPI_ISL_720988, EPI_ISL_720989, EPI_ISL_720990, EPI_ISL_720991, EPI_ISL_720992, EPI_ISL_720993, EPI_ISL_720994, EPI_ISL_720995, EPI_ISL_720996, EPI_ISL_720997, EPI_ISL_720998, EPI_ISL_720999, EPI_ISL_721000, EPI_ISL_721001, EPI_ISL_721002, EPI_ISL_721003, EPI_ISL_721004, EPI_ISL_721005, EPI_ISL_721006, EPI_ISL_721007, EPI_ISL_721008, EPI_ISL_721009, EPI_ISL_721010, EPI_ISL_721011, EPI_ISL_721012, EPI_ISL_721013, EPI_ISL_721014, EPI_ISL_721015, EPI_ISL_721016, EPI_ISL_721017, EPI_ISL_721018, EPI_ISL_721019, EPI_ISL_721020, EPI_ISL_721021, EPI_ISL_721022, EPI_ISL_721023, EPI_ISL_721024, EPI_ISL_721025, EPI_ISL_721026, EPI_ISL_721027, EPI_ISL_721028, EPI_ISL_721029, EPI_ISL_721030, EPI_ISL_721031, EPI_ISL_721033, EPI_ISL_721034, EPI_ISL_721035, EPI_ISL_721036, EPI_ISL_721037, EPI_ISL_721038, EPI_ISL_721039, EPI_ISL_721040, EPI_ISL_721041, EPI_ISL_721042, EPI_ISL_721043, EPI_ISL_721044, EPI_ISL_721045, EPI_ISL_721046, EPI_ISL_721047, EPI_ISL_721048, EPI_ISL_721049, EPI_ISL_721050, EPI_ISL_721051, EPI_ISL_721052, EPI_ISL_721053, EPI_ISL_721054, EPI_ISL_721055, EPI_ISL_721056, EPI_ISL_721057, EPI_ISL_721058, EPI_ISL_721059, EPI_ISL_721060, EPI_ISL_721062, EPI_ISL_721063, EPI_ISL_721064, EPI_ISL_721065, EPI_ISL_721066, EPI_ISL_721067, EPI_ISL_721068, EPI_ISL_721069, EPI_ISL_721070, EPI_ISL_721071, EPI_ISL_721072, EPI_ISL_721073, EPI_ISL_721074, EPI_ISL_721075, EPI_ISL_721076, EPI_ISL_721077, EPI_ISL_721078, EPI_ISL_721079, EPI_ISL_721080, EPI_ISL_721081, EPI_ISL_721082, EPI_ISL_721083, EPI_ISL_721084, EPI_ISL_721085, EPI_ISL_721086, EPI_ISL_721087, EPI_ISL_721088, EPI_ISL_721089, EPI_ISL_721090, EPI_ISL_721091, EPI_ISL_721092, EPI_ISL_721093, EPI_ISL_721094, EPI_ISL_721095, EPI_ISL_721096, EPI_ISL_721097, EPI_ISL_721098, EPI_ISL_721099, EPI_ISL_721100, EPI_ISL_721101, EPI_ISL_721102, EPI_ISL_721103, EPI_ISL_721104, EPI_ISL_721105, EPI_ISL_721106, EPI_ISL_721107, EPI_ISL_721108, EPI_ISL_721109, EPI_ISL_721110, EPI_ISL_721111, EPI_ISL_721112, EPI_ISL_721113, EPI_ISL_721114, EPI_ISL_721115, EPI_ISL_721116, EPI_ISL_721117, EPI_ISL_721118, EPI_ISL_721119, EPI_ISL_721120, EPI_ISL_721121, EPI_ISL_721122, EPI_ISL_721123, EPI_ISL_721124, EPI_ISL_721125, EPI_ISL_721126, EPI_ISL_721127, EPI_ISL_721128, EPI_ISL_721129, EPI_ISL_721130, EPI_ISL_721131, EPI_ISL_721132, EPI_ISL_721133, EPI_ISL_721134, EPI_ISL_721136, EPI_ISL_721137, EPI_ISL_721138, EPI_ISL_721139, EPI_ISL_721140, EPI_ISL_721141, EPI_ISL_721142, EPI_ISL_721143, EPI_ISL_721144, EPI_ISL_721145, EPI_ISL_721146, EPI_ISL_721147, EPI_ISL_721148, EPI_ISL_721149, EPI_ISL_721150, EPI_ISL_721151, EPI_ISL_721152, EPI_ISL_721153, EPI_ISL_721154, EPI_ISL_721155, EPI_ISL_721156, EPI_ISL_721157, EPI_ISL_721158, EPI_ISL_721159, EPI_ISL_721161, EPI_ISL_721162, EPI_ISL_721163, EPI_ISL_721164, EPI_ISL_721165, EPI_ISL_721166, EPI_ISL_721167, EPI_ISL_721168, EPI_ISL_721169, EPI_ISL_721170, EPI_ISL_721171, EPI_ISL_721172, EPI_ISL_721173, EPI_ISL_721174, EPI_ISL_721175, EPI_ISL_721176, EPI_ISL_721177, EPI_ISL_721178, EPI_ISL_721179, EPI_ISL_721180, EPI_ISL_721181, EPI_ISL_721182, EPI_ISL_721183, EPI_ISL_721184			
see above	Lighthouse Lab in Glasgow	Wellcome Sanger Institute for the COVID-19 Genomics UK (COG-UK) Consortium	Harper VanSteenhouse, Yumi Kasai, David Gray, Carol Clugston, Anna Dominiczak and Alex Alderton, Roberto Amato, Sonia Goncalves, Ewan Harrison, David K. Jackson, Ian Johnston, Dominic Kwiatkowski, Cordelia Langford, John Sillitoe on behalf of the Wellcome Sanger Institute COVID-19 Surveillance Team
EPI_ISL_721185, EPI_ISL_721186, EPI_ISL_721187, EPI_ISL_721189, EPI_ISL_721190, EPI_ISL_721191, EPI_ISL_721192, EPI_ISL_721193, EPI_ISL_721194, EPI_ISL_721195, EPI_ISL_721196, EPI_ISL_721197, EPI_ISL_721198, EPI_ISL_721199, EPI_ISL_721200, EPI_ISL_721201, EPI_ISL_721202, EPI_ISL_721203, EPI_ISL_721204, EPI_ISL_721205, EPI_ISL_721206, EPI_ISL_721207, EPI_ISL_721208, EPI_ISL_721209, EPI_ISL_721210, EPI_ISL_721211, EPI_ISL_721212, EPI_ISL_721213, EPI_ISL_721214, EPI_ISL_721215, EPI_ISL_721216, EPI_ISL_721217, EPI_ISL_721218, EPI_ISL_721219, EPI_ISL_721220, EPI_ISL_721221, EPI_ISL_721222, EPI_ISL_721223, EPI_ISL_721224, EPI_ISL_721225, EPI_ISL_721226, EPI_ISL_721227, EPI_ISL_721228, EPI_ISL_721229, EPI_ISL_721230, EPI_ISL_721231, EPI_ISL_721232, EPI_ISL_721233, EPI_ISL_721234, EPI_ISL_721235, EPI_ISL_721236, EPI_ISL_721237, EPI_ISL_721238, EPI_ISL_721239, EPI_ISL_721240, EPI_ISL_721241, EPI_ISL_721242, EPI_ISL_721243, EPI_ISL_721244, EPI_ISL_721245, EPI_ISL_721246, EPI_ISL_721247, EPI_ISL_721248, EPI_ISL_721249, EPI_ISL_721250, EPI_ISL_721251, EPI_ISL_721252, EPI_ISL_721253, EPI_ISL_721254, EPI_ISL_721255, EPI_ISL_721256, EPI_ISL_721257, EPI_ISL_721258, EPI_ISL_721259, EPI_ISL_721260, EPI_ISL_721261, EPI_ISL_721262, EPI_ISL_721263, EPI_ISL_721264, EPI_ISL_721265, EPI_ISL_721266, EPI_ISL_721267, EPI_ISL_721268, EPI_ISL_721269, EPI_ISL_721270, EPI_ISL_721271, EPI_ISL_721272, EPI_ISL_721273, EPI_ISL_721274, EPI_ISL_721275, EPI_ISL_721276, EPI_ISL_721277, EPI_ISL_721278, EPI_ISL_721279, EPI_ISL_721280, EPI_ISL_721281, EPI_ISL_721282, EPI_ISL_721283, EPI_ISL_721284, EPI_ISL_721285, EPI_ISL_721286, EPI_ISL_721287, EPI_ISL_721288, EPI_ISL_721289, EPI_ISL_721290, EPI_ISL_721291, EPI_ISL_721292, EPI_ISL_721293, EPI_ISL_721294, EPI_ISL_721295, EPI_ISL_721296, EPI_ISL_721297, EPI_ISL_721298, EPI_ISL_721299, EPI_ISL_721300, EPI_ISL_721301, EPI_ISL_721302, EPI_ISL_721303, EPI_ISL_721304, EPI_ISL_721305, EPI_ISL_721306, EPI_ISL_721307, EPI_ISL_721308, EPI_ISL_721309, EPI_ISL_721310, EPI_ISL_721311, EPI_ISL_721312, EPI_ISL_721313, EPI_ISL_721314, EPI_ISL_721315, EPI_ISL_721316, EPI_ISL_721317, EPI_ISL_721318, EPI_ISL_721319, EPI_ISL_721320, EPI_ISL_721321, EPI_ISL_721322, EPI_ISL_721323, EPI_ISL_721324, EPI_ISL_721325, EPI_ISL_721326, EPI_ISL_721327, EPI_ISL_721328, EPI_ISL_721329, EPI_ISL_721330, EPI_ISL_721331, EPI_ISL_721332, EPI_ISL_721333, EPI_ISL_721334, EPI_ISL_721336, EPI_ISL_721337, EPI_ISL_721338, EPI_ISL_721339, EPI_ISL_721340, EPI_ISL_721341, EPI_ISL_721342, EPI_ISL_721343, EPI_ISL_721344, EPI_ISL_721345, EPI_ISL_721346, EPI_ISL_721347, EPI_ISL_721348, EPI_ISL_721349, EPI_ISL_721350, EPI_ISL_721351, EPI_ISL_721352, EPI_ISL_721353, EPI_ISL_721354, EPI_ISL_721355, EPI_ISL_721356, EPI_ISL_721357, EPI_ISL_721358, EPI_ISL_721359, EPI_ISL_721360, EPI_ISL_721361, EPI_ISL_721362, EPI_ISL_721363, EPI_ISL_721364, EPI_ISL_721365, EPI_ISL_721366, EPI_ISL_721367, EPI_ISL_721368, EPI_ISL_721369, EPI_ISL_721370, EPI_ISL_721371, EPI_ISL_721372, EPI_ISL_721373, EPI_ISL_721374, EPI_ISL_721375, EPI_ISL_721376, EPI_ISL_721377, EPI_ISL_721378, EPI_ISL_721379, EPI_ISL_721380, EPI_ISL_721381, EPI_ISL_721382, EPI_ISL_721383, EPI_ISL_721384, EPI_ISL_721385, EPI_ISL_721386, EPI_ISL_721387, EPI_ISL_721388, EPI_ISL_721389, EPI_ISL_721390, EPI_ISL_721391, EPI_ISL_721392, EPI_ISL_721393, EPI_ISL_721394, EPI_ISL_721395, EPI_ISL_721396, EPI_ISL_721397, EPI_ISL_721398, EPI_ISL_721399, EPI_ISL_721400, EPI_ISL_721401, EPI_ISL_721402, EPI_ISL_721403, EPI_ISL_721404, EPI_ISL_721405, EPI_ISL_721406, EPI_ISL_721407, EPI_ISL_721408, EPI_ISL_721409, EPI_ISL_721410, EPI_ISL_721411, EPI_ISL_721412, EPI_ISL_721413, EPI_ISL_721414, EPI_ISL_721415, EPI_ISL_721416, EPI_ISL_721417, EPI_ISL_721418, EPI_ISL_721419, EPI_ISL_721420, EPI_ISL_721421, EPI_ISL_721422, EPI_ISL_721423, EPI_ISL_721424, EPI_ISL_721425, EPI_ISL_721426, EPI_ISL_721427, EPI_ISL_721428, EPI_ISL_721429, EPI_ISL_721430, EPI_ISL_721431, EPI_ISL_721432, EPI_ISL_721433, EPI_ISL_721434, EPI_ISL_721435, EPI_ISL_721436, EPI_ISL_721437, EPI_ISL_721438, EPI_ISL_721439, EPI_ISL_721440, EPI_ISL_721441, EPI_ISL_721442, EPI_ISL_721443, EPI_ISL_721444, EPI_ISL_721445, EPI_ISL_721446, EPI_ISL_721447, EPI_ISL_721448, EPI_ISL_721449, EPI_ISL_721450, EPI_ISL_721451, EPI_ISL_721452, EPI_ISL_721453, EPI_ISL_721454, EPI_ISL_721455, EPI_ISL_721456, EPI_ISL_721458, EPI_ISL_721459, EPI_ISL_721460, EPI_ISL_721461, EPI_ISL_721462, EPI_ISL_721463, EPI_ISL_721464, EPI_ISL_721465, EPI_ISL_721466, EPI_ISL_721467, EPI_ISL_721468, EPI_ISL_721469, EPI_ISL_721470, EPI_ISL_721471, EPI_ISL_721472, EPI_ISL_721473, EPI_ISL_721474, EPI_ISL_721475, EPI_ISL_721477, EPI_ISL_721478, EPI_ISL_721479, EPI_ISL_721480, EPI_ISL_721481, EPI_ISL_721482, EPI_ISL_721483, EPI_ISL_721484, EPI_ISL_721485, EPI_ISL_721486, EPI_ISL_721487, EPI_ISL_721488, EPI_ISL_721489, EPI_ISL_721490, EPI_ISL_721491, EPI_ISL_721492, EPI_ISL_721493, EPI_ISL_721494, EPI_ISL_721495, EPI_ISL_721496, EPI_ISL_721497, EPI_ISL_721498, EPI_ISL_721499, EPI_ISL_721500, EPI_ISL_721501, EPI_ISL_721502, EPI_ISL_721503, EPI_ISL_721504, EPI_ISL_721505, EPI_ISL_721506, EPI_ISL_721507, EPI_ISL_721508, EPI_ISL_721509, EPI_ISL_721510, EPI_ISL_721512, EPI_ISL_721513, EPI_ISL_721514, EPI_ISL_721515, EPI_ISL_721516, EPI_ISL_721517, EPI_ISL_721518			
see above	Lighthouse Lab in Alderley Park	Wellcome Sanger Institute for the COVID-19 Genomics UK (COG-UK) Consortium	Jacquelyn Wynn, Mairead Hyland, The Lighthouse Lab in Alderley Park and Alex Alderton, Roberto Amato, Sonia Goncalves, Ewan Harrison, David K. Jackson, Ian Johnston, Dominic Kwiatkowski, Cordelia Langford, John Sillitoe on behalf of the Wellcome Sanger Institute COVID-19 Surveillance Team
EPI_ISL_721519	Lighthouse Lab in Glasgow	Wellcome Sanger Institute for the COVID-19 Genomics UK (COG-UK) Consortium	Harper VanSteenhouse, Yumi Kasai, David Gray, Carol Clugston, Anna Dominiczak and Alex Alderton, Roberto Amato, Sonia Goncalves, Ewan Harrison, David K. Jackson, Ian Johnston, Dominic Kwiatkowski, Cordelia Langford, John Sillitoe on behalf of the Wellcome Sanger Institute COVID-19 Surveillance Team
EPI_ISL_721520	Lighthouse Lab in Alderley Park	Wellcome Sanger Institute for the COVID-19 Genomics UK (COG-UK) Consortium	Jacquelyn Wynn, Mairead Hyland, The Lighthouse Lab in Alderley Park and Alex Alderton, Roberto Amato, Sonia Goncalves, Ewan Harrison, David K. Jackson, Ian Johnston, Dominic Kwiatkowski, Cordelia Langford, John Sillitoe on behalf of the Wellcome Sanger Institute COVID-19 Surveillance Team
EPI_ISL_721521	Lighthouse Lab in Glasgow	Wellcome Sanger Institute for the COVID-19 Genomics UK (COG-UK) Consortium	Harper VanSteenhouse, Yumi Kasai, David Gray, Carol Clugston, Anna Dominiczak and Alex Alderton, Roberto Amato, Sonia Goncalves, Ewan Harrison, David K. Jackson, Ian Johnston, Dominic Kwiatkowski, Cordelia Langford, John Sillitoe on behalf of the Wellcome Sanger Institute COVID-19 Surveillance Team
EPI_ISL_721522, EPI_ISL_721523, EPI_ISL_721525	Lighthouse Lab in Alderley Park	Wellcome Sanger Institute for the COVID-19 Genomics UK (COG-UK) Consortium	Jacquelyn Wynn, Mairead Hyland, The Lighthouse Lab in Alderley Park and Alex Alderton, Roberto Amato, Sonia Goncalves, Ewan Harrison, David K. Jackson, Ian Johnston, Dominic Kwiatkowski, Cordelia Langford, John Sillitoe on behalf of the Wellcome Sanger Institute COVID-19 Surveillance Team
EPI_ISL_721526, EPI_ISL_721527, EPI_ISL_721529, EPI_ISL_721530, EPI_ISL_721531, EPI_ISL_721532	Lighthouse Lab in Milton Keynes	Wellcome Sanger Institute for the COVID-19 Genomics UK (COG-UK) Consortium	The Lighthouse Lab in Milton Keynes and Alex Alderton, Roberto Amato, Sonia Goncalves, Ewan Harrison, David K. Jackson, Ian Johnston, Dominic Kwiatkowski, Cordelia Langford, John Sillitoe on behalf of the Wellcome Sanger Institute COVID-19 Surveillance Team
EPI_ISL_721533, EPI_ISL_721534, EPI_ISL_721535, EPI_ISL_721536, EPI_ISL_721537, EPI_ISL_721538, EPI_ISL_721539, EPI_ISL_721540, EPI_ISL_721541, EPI_ISL_721543, EPI_ISL_721544, EPI_ISL_721545, EPI_ISL_721546, EPI_ISL_721548, EPI_ISL_721549, EPI_ISL_721550, EPI_ISL_721551, EPI_ISL_721552, EPI_ISL_721553, EPI_ISL_721554, EPI_ISL_721556, EPI_ISL_721557, EPI_ISL_721558, EPI_ISL_721559, EPI_ISL_721560, EPI_ISL_721561, EPI_ISL_721563, EPI_ISL_721564, EPI_ISL_721565, EPI_ISL_721566, EPI_ISL_721567			
see above	Lighthouse Lab in Glasgow	Wellcome Sanger Institute for the COVID-19 Genomics UK (COG-UK) Consortium	Harper VanSteenhouse, Yumi Kasai, David Gray, Carol Clugston, Anna Dominiczak and Alex Alderton, Roberto Amato, Sonia Goncalves, Ewan Harrison, David K. Jackson, Ian Johnston, Dominic Kwiatkowski, Cordelia Langford, John Sillitoe on behalf of the Wellcome Sanger Institute COVID-19 Surveillance Team
EPI_ISL_721624	National Institute for Infectious Diseases, INMI, "L. Spallanzani" IRCCS	National Institute for Infectious Diseases, INMI, "L. Spallanzani" IRCCS	C.E.M Gruber, B Bartolini, M Rueca, F Messina, E Giombini, A Di Caro, MR Capobianchi
EPI_ISL_721625	National Institute for Infectious Diseases, INMI, "L. Spallanzani" IRCCS	National Institute for Infectious Diseases, INMI, "L. Spallanzani" IRCCS	B Bartolini, C.E.M Gruber, M Rueca, F Messina, E Giombini, MR Capobianchi, A Di Caro
EPI_ISL_721626	National Institute for Infectious Diseases, INMI, "L. Spallanzani" IRCCS	National Institute for Infectious Diseases, INMI, "L. Spallanzani" IRCCS	M Rueca, B Bartolini, C.E.M Gruber, F Messina, E Giombini, A Di Caro, MR Capobianchi
EPI_ISL_721627	National Institute for Infectious Diseases, INMI, "L. Spallanzani" IRCCS	National Institute for Infectious Diseases, INMI, "L. Spallanzani" IRCCS	F Messina, E Giombini, M Rueca, B Bartolini, C.E.M Gruber, MR Capobianchi, A Di Caro
EPI_ISL_721628	National Institute for Infectious Diseases, INMI, "L. Spallanzani" IRCCS	National Institute for Infectious Diseases, INMI, "L. Spallanzani" IRCCS	E Giombini, M Rueca, B Bartolini, C.E.M Gruber, F Messina, A Di Caro, MR Capobianchi
EPI_ISL_721629, EPI_ISL_721630, EPI_ISL_721631, EPI_ISL_721632, EPI_ISL_721633, EPI_ISL_721634, EPI_ISL_721635, EPI_ISL_721636, EPI_ISL_721637, EPI_ISL_721638, EPI_ISL_721639, EPI_ISL_721640, EPI_ISL_721641, EPI_ISL_721642, EPI_ISL_721643, EPI_ISL_721644, EPI_ISL_721645			

see above	Armed Forces Medical College	National Centre For Cell Science	Dhiraj Paul, Kunal Jani, Radha Chauhan, Janesh Kumar, Vasudevan Seshadri, Girdhari Lal, Rajesh Karyakarte, Suvarna Joshi, Murlidhar Tambe, Sourav Sen, Santosh Karade, Kavita Bala Anand, Shelinder Pal Singh Shergill, Rajiv Mohan Gupta, Manoj Kumar Bhat, Arvind Sahu, Yogesh S Shouche
EPI_ISL_721646, EPI_ISL_721647, EPI_ISL_721648, EPI_ISL_721649, EPI_ISL_721650, EPI_ISL_721651, EPI_ISL_721653, EPI_ISL_721654, EPI_ISL_721655, EPI_ISL_721656, EPI_ISL_721657, EPI_ISL_721658, EPI_ISL_721659, EPI_ISL_721660, EPI_ISL_721661, EPI_ISL_721662, EPI_ISL_721663, EPI_ISL_721664, EPI_ISL_721665, EPI_ISL_721666, EPI_ISL_721667			
see above	National Centre For Cell Science	National Centre For Cell Science	Dhiraj Paul, Kunal Jani, Radha Chauhan, Janesh Kumar, Vasudevan Seshadri, Girdhari Lal, Rajesh Karyakarte, Suvarna Joshi, Murlidhar Tambe, Sourav Sen, Santosh Karade, Kavita Bala Anand, Shelinder Pal Singh Shergill, Rajiv Mohan Gupta, Manoj Kumar Bhat, Arvind Sahu, Yogesh S Shouche
EPI_ISL_721670, EPI_ISL_721671, EPI_ISL_721672, EPI_ISL_721673, EPI_ISL_721674, EPI_ISL_721675, EPI_ISL_721676, EPI_ISL_721677, EPI_ISL_721678, EPI_ISL_721679, EPI_ISL_721680, EPI_ISL_721681, EPI_ISL_721688, EPI_ISL_721689, EPI_ISL_721690, EPI_ISL_721691, EPI_ISL_721692, EPI_ISL_721693, EPI_ISL_721694, EPI_ISL_721695, EPI_ISL_721696, EPI_ISL_721697, EPI_ISL_721698, EPI_ISL_721699, EPI_ISL_721700, EPI_ISL_721701, EPI_ISL_721702, EPI_ISL_721703, EPI_ISL_721704, EPI_ISL_721705, EPI_ISL_721706, EPI_ISL_721707, EPI_ISL_721708, EPI_ISL_721709, EPI_ISL_721710, EPI_ISL_721711, EPI_ISL_721712, EPI_ISL_721713, EPI_ISL_721714, EPI_ISL_721715, EPI_ISL_721716, EPI_ISL_721717, EPI_ISL_721718, EPI_ISL_721719, EPI_ISL_721720, EPI_ISL_721721, EPI_ISL_721722, EPI_ISL_721723, EPI_ISL_721724, EPI_ISL_721725, EPI_ISL_721726, EPI_ISL_721727, EPI_ISL_721728, EPI_ISL_721729, EPI_ISL_721730, EPI_ISL_721731, EPI_ISL_721732, EPI_ISL_721733, EPI_ISL_721734, EPI_ISL_721735, EPI_ISL_721736, EPI_ISL_721737, EPI_ISL_721738, EPI_ISL_721739, EPI_ISL_721740, EPI_ISL_721741, EPI_ISL_721742, EPI_ISL_721743, EPI_ISL_721744, EPI_ISL_721745, EPI_ISL_721746, EPI_ISL_721747, EPI_ISL_721748, EPI_ISL_721749, EPI_ISL_721750, EPI_ISL_721751, EPI_ISL_721752, EPI_ISL_721753, EPI_ISL_721754, EPI_ISL_721755, EPI_ISL_721756, EPI_ISL_721757, EPI_ISL_721758, EPI_ISL_721759, EPI_ISL_721760, EPI_ISL_721761, EPI_ISL_721762, EPI_ISL_721763, EPI_ISL_721764, EPI_ISL_721765, EPI_ISL_721766, EPI_ISL_721767, EPI_ISL_721768, EPI_ISL_721769, EPI_ISL_721770, EPI_ISL_721771, EPI_ISL_721772, EPI_ISL_721773, EPI_ISL_721774, EPI_ISL_721775, EPI_ISL_721776, EPI_ISL_721777, EPI_ISL_721778, EPI_ISL_721779, EPI_ISL_721780, EPI_ISL_721781, EPI_ISL_721782, EPI_ISL_721783, EPI_ISL_721784, EPI_ISL_721785, EPI_ISL_721786, EPI_ISL_721787, EPI_ISL_721788, EPI_ISL_721789, EPI_ISL_721790, EPI_ISL_721791, EPI_ISL_721792, EPI_ISL_721793, EPI_ISL_721794, EPI_ISL_721795, EPI_ISL_721796, EPI_ISL_721797, EPI_ISL_721798, EPI_ISL_721799, EPI_ISL_721800, EPI_ISL_721801, EPI_ISL_721802, EPI_ISL_721803, EPI_ISL_721804, EPI_ISL_721805, EPI_ISL_721806, EPI_ISL_721807, EPI_ISL_721808, EPI_ISL_721809, EPI_ISL_721810, EPI_ISL_721811, EPI_ISL_721812, EPI_ISL_721813, EPI_ISL_721814, EPI_ISL_721815, EPI_ISL_721816, EPI_ISL_721817, EPI_ISL_721818, EPI_ISL_721819, EPI_ISL_721820, EPI_ISL_721821, EPI_ISL_721822, EPI_ISL_721823, EPI_ISL_721824, EPI_ISL_721825, EPI_ISL_721826, EPI_ISL_721827, EPI_ISL_721828, EPI_ISL_721829, EPI_ISL_721830, EPI_ISL_721831, EPI_ISL_721832, EPI_ISL_721833, EPI_ISL_721834, EPI_ISL_721835, EPI_ISL_721836, EPI_ISL_721837, EPI_ISL_721838, EPI_ISL_721839, EPI_ISL_721840, EPI_ISL_721841, EPI_ISL_721842, EPI_ISL_721843, EPI_ISL_721844, EPI_ISL_721845, EPI_ISL_721846, EPI_ISL_721847, EPI_ISL_721848, EPI_ISL_721849, EPI_ISL_721850, EPI_ISL_721851, EPI_ISL_721852, EPI_ISL_721853, EPI_ISL_721854, EPI_ISL_721855, EPI_ISL_721856, EPI_ISL_721857, EPI_ISL_721858, EPI_ISL_721859, EPI_ISL_721860, EPI_ISL_721861, EPI_ISL_721862, EPI_ISL_721863, EPI_ISL_721864, EPI_ISL_721865, EPI_ISL_721866, EPI_ISL_721867, EPI_ISL_721868, EPI_ISL_721869, EPI_ISL_721870, EPI_ISL_721871, EPI_ISL_721872, EPI_ISL_721873, EPI_ISL_721874, EPI_ISL_721875, EPI_ISL_721876, EPI_ISL_721877, EPI_ISL_721878, EPI_ISL_721879, EPI_ISL_721880, EPI_ISL_721881, EPI_ISL_721882, EPI_ISL_721883, EPI_ISL_721884, EPI_ISL_721885, EPI_ISL_721886, EPI_ISL_721887, EPI_ISL_721888, EPI_ISL_721889, EPI_ISL_721890, EPI_ISL_721891, EPI_ISL_721892, EPI_ISL_721893, EPI_ISL_721894, EPI_ISL_721895, EPI_ISL_721896, EPI_ISL_721897, EPI_ISL_721898, EPI_ISL_721899, EPI_ISL_721900, EPI_ISL_721901, EPI_ISL_721902, EPI_ISL_721903, EPI_ISL_721904, EPI_ISL_721905, EPI_ISL_721906, EPI_ISL_721907, EPI_ISL_721908, EPI_ISL_721909, EPI_ISL_721910, EPI_ISL_721911, EPI_ISL_721912, EPI_ISL_721913, EPI_ISL_721914, EPI_ISL_721915, EPI_ISL_721916, EPI_ISL_721917, EPI_ISL_721918, EPI_ISL_721919, EPI_ISL_721920, EPI_ISL_721921, EPI_ISL_721922, EPI_ISL_721923, EPI_ISL_721924, EPI_ISL_721925, EPI_ISL_721926, EPI_ISL_721927, EPI_ISL_721928, EPI_ISL_721929, EPI_ISL_721930, EPI_ISL_721931, EPI_ISL_721932, EPI_ISL_721933, EPI_ISL_721934, EPI_ISL_721935, EPI_ISL_721936, EPI_ISL_721937, EPI_ISL_721938, EPI_ISL_721939, EPI_ISL_721940, EPI_ISL_721941, EPI_ISL_721942, EPI_ISL_721943, EPI_ISL_721944, EPI_ISL_721945, EPI_ISL_721946, EPI_ISL_721947, EPI_ISL_721948, EPI_ISL_721949, EPI_ISL_721950, EPI_ISL_721951, EPI_ISL_721952, EPI_ISL_721953, EPI_ISL_721954, EPI_ISL_721955, EPI_ISL_721956, EPI_ISL_721957, EPI_ISL_721958, EPI_ISL_721959, EPI_ISL_721960, EPI_ISL_721961, EPI_ISL_721962, EPI_ISL_721963, EPI_ISL_721964, EPI_ISL_721965, EPI_ISL_721966, EPI_ISL_721967, EPI_ISL_721968, EPI_ISL_721969, EPI_ISL_721970, EPI_ISL_721971, EPI_ISL_721972, EPI_ISL_721973, EPI_ISL_721974, EPI_ISL_721975, EPI_ISL_721976, EPI_ISL_721977, EPI_ISL_721978, EPI_ISL_721979			
see above	Viollier AG	Department of Biosystems Science and Engineering, ETH Zürich	Christian Beisel
EPI_ISL_722177, EPI_ISL_722178, EPI_ISL_722179, EPI_ISL_722180, EPI_ISL_722181, EPI_ISL_722182, EPI_ISL_722183, EPI_ISL_722184, EPI_ISL_722185, EPI_ISL_722186	National Centre For Cell Science	National Centre For Cell Science	Dhiraj Paul, Kunal Jani, Radha Chauhan, Janesh Kumar, Vasudevan Seshadri, Girdhari Lal, Rajesh Karyakarte, Suvarna Joshi, Murlidhar Tambe, Sourav Sen, Santosh Karade, Kavita Bala Anand, Shelinder Pal Singh Shergill, Rajiv Mohan Gupta, Manoj Kumar Bhat, Arvind Sahu, Yogesh S Shouche
EPI_ISL_722187, EPI_ISL_722188, EPI_ISL_722189, EPI_ISL_722190, EPI_ISL_722191, EPI_ISL_722192, EPI_ISL_722193, EPI_ISL_722194, EPI_ISL_722195, EPI_ISL_722196, EPI_ISL_722197, EPI_ISL_722198, EPI_ISL_722199, EPI_ISL_722200			
see above	Armed Forces Medical College	National Centre For Cell Science	Dhiraj Paul, Kunal Jani, Radha Chauhan, Janesh Kumar, Vasudevan Seshadri, Girdhari Lal, Rajesh Karyakarte, Suvarna Joshi, Murlidhar Tambe, Sourav Sen, Santosh Karade, Kavita Bala Anand, Shelinder Pal Singh Shergill, Rajiv Mohan Gupta, Manoj Kumar Bhat, Arvind Sahu, Yogesh S Shouche
EPI_ISL_722201	Vitalis Mostar	Alea Genetic Center	Salihefendic L., Pecar D., Konjhodzic R.
EPI_ISL_722202, EPI_ISL_722203, EPI_ISL_722204, EPI_ISL_722205, EPI_ISL_722206, EPI_ISL_722207	University Hospital Zurich	Institute of Medical Virology, University of Zurich	Stefan Schmutz, Verena Kufner, Maryam Zaheri, Gabriela Ziltener, Aline Wolfensberger, Thomas Scheier, Jürg Böni, Michael Huber, Alexandra Trkola
EPI_ISL_722209	Dom Zdravlja Sarajevo	Alea Genetic Center	Salihefendic L., Pecar D., Konjhodzic R.
EPI_ISL_722210, EPI_ISL_722212, EPI_ISL_722213, EPI_ISL_722214, EPI_ISL_722215, EPI_ISL_722217, EPI_ISL_722218, EPI_ISL_722219, EPI_ISL_722220, EPI_ISL_722221, EPI_ISL_722222, EPI_ISL_722223, EPI_ISL_722224, EPI_ISL_722225, EPI_ISL_722226, EPI_ISL_722227, EPI_ISL_722228, EPI_ISL_722229, EPI_ISL_722230, EPI_ISL_722231, EPI_ISL_722232, EPI_ISL_722242, EPI_ISL_722243, EPI_ISL_722244, EPI_ISL_722245, EPI_ISL_722246, EPI_ISL_722247, EPI_ISL_722248, EPI_ISL_722249, EPI_ISL_722250, EPI_ISL_722251, EPI_ISL_722252, EPI_ISL_722253, EPI_ISL_722254, EPI_ISL_722255, EPI_ISL_722256, EPI_ISL_722257, EPI_ISL_722258, EPI_ISL_722259, EPI_ISL_722260, EPI_ISL_722261, EPI_ISL_722262, EPI_ISL_722263, EPI_ISL_722264, EPI_ISL_722265, EPI_ISL_722266, EPI_ISL_722267, EPI_ISL_722268, EPI_ISL_722269, EPI_ISL_722270, EPI_ISL_722271, EPI_ISL_722272			
see above	Servicio de Microbiología, Hospital Miguel Servet, Zaragoza	SeqCOVID-SPAIN consortium/IBV(CSIC)	Antonio Rezusta López, Alexander Tristancho Baró, Ana Milagro, Yolanda Gracia Grataloup, Nieves Martínez Cameo and SeqCOVID-SPAIN consortium
EPI_ISL_722273, EPI_ISL_722274, EPI_ISL_722275, EPI_ISL_722276, EPI_ISL_722277, EPI_ISL_722278, EPI_ISL_722279, EPI_ISL_722280, EPI_ISL_722281, EPI_ISL_722282, EPI_ISL_722283, EPI_ISL_722284, EPI_ISL_722285, EPI_ISL_722286, EPI_ISL_722287, EPI_ISL_722288, EPI_ISL_722289, EPI_ISL_722290, EPI_ISL_722291, EPI_ISL_722292, EPI_ISL_722293, EPI_ISL_722294, EPI_ISL_722295, EPI_ISL_722296, EPI_ISL_722297, EPI_ISL_722298, EPI_ISL_722299, EPI_ISL_722302, EPI_ISL_722303, EPI_ISL_722304, EPI_ISL_722305, EPI_ISL_722306, EPI_ISL_722307, EPI_ISL_722308, EPI_ISL_722309, EPI_ISL_722310, EPI_ISL_722311, EPI_ISL_722312, EPI_ISL_722314, EPI_ISL_722315, EPI_ISL_722316, EPI_ISL_722317, EPI_ISL_722318, EPI_ISL_722319, EPI_ISL_722321, EPI_ISL_722322, EPI_ISL_722323, EPI_ISL_722324, EPI_ISL_722326, EPI_ISL_722327, EPI_ISL_722328, EPI_ISL_722329, EPI_ISL_722330, EPI_ISL_722331, EPI_ISL_722332, EPI_ISL_722333, EPI_ISL_722334, EPI_ISL_722335, EPI_ISL_722336, EPI_ISL_722337, EPI_ISL_722338, EPI_ISL_722339, EPI_ISL_722340, EPI_ISL_722341, EPI_ISL_722342, EPI_ISL_722343, EPI_ISL_722344, EPI_ISL_722345, EPI_ISL_722346, EPI_ISL_722347, EPI_ISL_722348, EPI_ISL_722349, EPI_ISL_722350, EPI_ISL_722351, EPI_ISL_722352, EPI_ISL_722353, EPI_ISL_722354, EPI_ISL_722355, EPI_ISL_722356, EPI_ISL_722357, EPI_ISL_722359, EPI_ISL_722360, EPI_ISL_722361, EPI_ISL_722362, EPI_ISL_722363, EPI_ISL_722364, EPI_ISL_722365, EPI_ISL_722366, EPI_ISL_722367, EPI_ISL_722368, EPI_ISL_722369, EPI_ISL_722370, EPI_ISL_722371, EPI_ISL_722413, EPI_ISL_722419, EPI_ISL_722420, EPI_ISL_722421, EPI_ISL_722422, EPI_ISL_722423, EPI_ISL_722424, EPI_ISL_722425, EPI_ISL_722426, EPI_ISL_722427, EPI_ISL_722428, EPI_ISL_722429, EPI_ISL_722430, EPI_ISL_722431, EPI_ISL_722432, EPI_ISL_722433, EPI_ISL_722434, EPI_ISL_722435, EPI_ISL_722436, EPI_ISL_722437, EPI_ISL_722438, EPI_ISL_722439, EPI_ISL_722440, EPI_ISL_722441, EPI_ISL_722442, EPI_ISL_722443, EPI_ISL_722444, EPI_ISL_722445, EPI_ISL_722446, EPI_ISL_722447, EPI_ISL_722452, EPI_ISL_722453, EPI_ISL_722454, EPI_ISL_722455, EPI_ISL_722456, EPI_ISL_722457, EPI_ISL_722458, EPI_ISL_722459, EPI_ISL_722460, EPI_ISL_722461, EPI_ISL_722462, EPI_ISL_722463, EPI_ISL_722464, EPI_ISL_722465, EPI_ISL_722492, EPI_ISL_722493, EPI_ISL_722494, EPI_ISL_722495, EPI_ISL_722496, EPI_ISL_722497, EPI_ISL_722498, EPI_ISL_722499, EPI_ISL_722500, EPI_ISL_722501, EPI_ISL_722502, EPI_ISL_722503, EPI_ISL_722504, EPI_ISL_722505, EPI_ISL_722506, EPI_ISL_722507, EPI_ISL_722508, EPI_ISL_722509, EPI_ISL_722510, EPI_ISL_722511, EPI_ISL_722512, EPI_ISL_722513, EPI_ISL_722514, EPI_ISL_722515, EPI_ISL_722516, EPI_ISL_722517, EPI_ISL_722518, EPI_ISL_722519, EPI_ISL_722520, EPI_ISL_722521, EPI_ISL_722522, EPI_ISL_722523, EPI_ISL_722524, EPI_ISL_722525, EPI_ISL_722526, EPI_ISL_722527, EPI_ISL_722528, EPI_ISL_722529, EPI_ISL_722530, EPI_ISL_722531, EPI_ISL_722532, EPI_ISL_722533, EPI_ISL_722534, EPI_ISL_722535, EPI_ISL_722536, EPI_ISL_722537, EPI_ISL_722538, EPI_ISL_722539, EPI_ISL_722540, EPI_ISL_722541, EPI_ISL_722542, EPI_ISL_722543, EPI_ISL_722544, EPI_ISL_722545, EPI_ISL_722546, EPI_ISL_722547, EPI_ISL_722548, EPI_ISL_722549, EPI_ISL_722550, EPI_ISL_722551, EPI_ISL_722552, EPI_ISL_722553, EPI_ISL_722554, EPI_ISL_722555, EPI_ISL_722556, EPI_ISL_722557, EPI_ISL_722558, EPI_ISL_722559, EPI_ISL_722560, EPI_ISL_722561, EPI_ISL_722562, EPI_ISL_722563, EPI_ISL_722564, EPI_ISL_722565, EPI_ISL_722566, EPI_ISL_722567, EPI_ISL_722568, EPI_ISL_722569, EPI_ISL_722570, EPI_ISL_722571, EPI_ISL_722572, EPI_ISL_722573, EPI_ISL_722574, EPI_ISL_722575, EPI_ISL_722576, EPI_ISL_722577, EPI_ISL_722578, EPI_ISL_722579, EPI_ISL_722580, EPI_ISL_722581, EPI_ISL_722582, EPI_ISL_722583, EPI_ISL_722584, EPI_ISL_722585, EPI_ISL_722586, EPI_ISL_722587, EPI_ISL_722588, EPI_ISL_722589, EPI_ISL_722590, EPI_ISL_722591, EPI_ISL_722592, EPI_ISL_722593, EPI_ISL_722594, EPI_ISL_722595, EPI_ISL_722596, EPI_ISL_722597, EPI_ISL_722598, EPI_ISL_722599, EPI_ISL_722600, EPI_ISL_722601, EPI_ISL_722602, EPI_ISL_722603, EPI_ISL_722604, EPI_ISL_722605, EPI_ISL_722606, EPI_ISL_722607, EPI_ISL_722608, EPI_ISL_722609, EPI_ISL_722610, EPI_ISL_722611, EPI_ISL_722612, EPI_ISL_722613, EPI_ISL_722614, EPI_ISL_722615, EPI_ISL_722616, EPI_ISL_722617, EPI_ISL_722618, EPI_ISL_722619, EPI_ISL_722620, EPI_ISL_722621, EPI_ISL_722622, EPI_ISL_722623, EPI_ISL_722624, EPI_ISL_722625, EPI_ISL_722626, EPI_ISL_722627, EPI_ISL_722628, EPI_ISL_722629, EPI_ISL_722630, EPI_ISL_722631, EPI_ISL_722632, EPI_ISL_722633, EPI_ISL_722634, EPI_ISL_722635, EPI_ISL_722636, EPI_ISL_722637, EPI_ISL_722638, EPI_ISL_722639, EPI_ISL_722640, EPI_ISL_722641, EPI_ISL_722642, EPI_ISL_722643, EPI_ISL_722644, EPI_ISL_722645, EPI_ISL_722646, EPI_ISL_722647, EPI_ISL_722648, EPI_ISL_722649, EPI_ISL_722650, EPI_ISL_722651, EPI_ISL_722652, EPI_ISL_722653, EPI_ISL_722654, EPI_ISL_722655, EPI_ISL_722656, EPI_ISL_722657, EPI_ISL_722658, EPI_ISL_722659, EPI_ISL_722660, EPI_ISL_722661, EPI_ISL_722662, EPI_ISL_722663, EPI_ISL_722664, EPI_ISL_722665, EPI_ISL_722666, EPI_ISL_722667, EPI_ISL_722668, EPI_ISL_722669, EPI_ISL_722670, EPI_ISL_722671, EPI_ISL_722672, EPI_ISL_722673, EPI_ISL_722674, EPI_ISL_722675, EPI_ISL_722676, EPI_ISL_722677, EPI_ISL_722678, EPI_ISL_722679, EPI_ISL_722680, EPI_ISL_722681, EPI_ISL_722682, EPI_ISL_722683, EPI_ISL_722684, EPI_ISL_722685, EPI_ISL_722686, EPI_ISL_722687, EPI_ISL_722688, EPI_ISL_722689, EPI_ISL_722690, EPI_ISL_722691, EPI_ISL_722692, EPI_ISL_722693, EPI_ISL_722694, EPI_ISL_722695, EPI_ISL_722696, EPI_ISL_722697, EPI_ISL_722698, EPI_ISL_722699, EPI_ISL_722700, EPI_ISL_722701, EPI_ISL_722702, EPI_ISL_722703, EPI_ISL_722704, EPI_ISL_722705, EPI_ISL_722706, EPI_ISL_722707, EPI_ISL_722708, EPI_ISL_722709, EPI_ISL_722710, EPI_ISL_722711, EPI_ISL_722712, EPI_ISL_722713, EPI_ISL_722714, EPI_ISL_722715, EPI_ISL_722716, EPI_ISL_722717, EPI_ISL_722718, EPI_ISL_722719, EPI_ISL_722720, EPI_ISL_722721, EPI_ISL_722722, EPI_ISL_722723, EPI_ISL_722724, EPI_ISL_722725, EPI_ISL_722726, EPI_ISL_722727, EPI_ISL_722728, EPI_ISL_722729, EPI_ISL_722730, EPI_ISL_722731, EPI_ISL_722732, EPI_ISL_722733, EPI_ISL_722734, EPI_ISL_722735, EPI_ISL_722736, EPI_ISL_722737, EPI_ISL_722738, EPI_ISL_722739, EPI_ISL_722740, EPI_ISL_722741, EPI_ISL_722742, EPI_ISL_722743, EPI_ISL_722744, EPI_ISL_722745, EPI_ISL_722746, EPI_ISL_722747, EPI_ISL_722748, EPI_ISL_722749, EPI_ISL_722750, EPI_ISL_722751, EPI_ISL_722752, EPI_ISL_722753, EPI_ISL_722754, EPI_ISL_722755, EPI_ISL_722756, EPI_ISL_722757, EPI_ISL_722758, EPI_ISL_722759, EPI_ISL_722760, EPI_ISL_722761, EPI_ISL_722762, EPI_ISL_722763, EPI_ISL_722764, EPI_ISL_722765, EPI_ISL_722766, EPI_ISL_722767, EPI_ISL_722768, EPI_ISL_722769, EPI_ISL_722770, EPI_ISL_722771, EPI_ISL_722772, EPI_ISL_722773, EPI_ISL_722774, EPI_ISL_722775, EPI_ISL_722776, EPI_ISL_722777, EPI_ISL_722778, EPI_ISL_722779, EPI_ISL_722780, EPI_ISL_722781, EPI_ISL_722782, EPI_ISL_722783, EPI_ISL_722784, EPI_ISL_722785, EPI_ISL_722786, EPI_ISL_722787, EPI_ISL_722788, EPI_ISL_722789, EPI_ISL_722790, EPI_ISL_722791, EPI_ISL_722792, EPI_ISL_722793, EPI_ISL_722794, EPI_ISL_722795, EPI_ISL_722796, EPI_ISL_722797, EPI_ISL_722798, EPI_ISL_722799, EPI_ISL_722800, EPI_ISL_722801, EPI_ISL_722802, EPI_ISL_722803, EPI_ISL_722804, EPI_ISL_722805, EPI_ISL_722806, EPI_ISL_722807, EPI_ISL_722808, EPI_ISL_722809, EPI_ISL_722810, EPI_ISL_722811, EPI_ISL_722812, EPI_ISL_722813, EPI_ISL_722814, EPI_ISL_722815, EPI_ISL_722816, EPI_ISL_722817, EPI_ISL_722818, EPI_ISL_722819, EPI_ISL_722820, EPI_ISL_722821, EPI_ISL_722822, EPI_ISL_722823, EPI_ISL_722824, EPI_ISL_722825, EPI_ISL_722826, EPI_ISL_722827, EPI_ISL_722828, EPI_ISL_722829, EPI_ISL_722830, EPI_ISL_722831, EPI_ISL_722832, EPI_ISL_722833, EPI_ISL_722834, EPI_ISL_722835, EPI_ISL_722836, EPI_ISL_722837, EPI_ISL_722838, EPI_ISL_722839, EPI_ISL_722840, EPI_ISL_722841, EPI_ISL_722842, EPI_ISL_722843, EPI_ISL_722844, EPI_ISL_722845, EPI_ISL_722846, EPI_ISL_722847, EPI_ISL_722848, EPI_ISL_722849, EPI_ISL_722850			
see above	Dutch COVID-19 response team	Erasmus Medical Center	Bas Oude Munnink, Reina Sikkema, David Nieuwenhuijse, Irina Chestakova, Anne van der Linden, Marjan Boter, Emmanuelle Munger, Corine GeurtsvanKessel, Annemiek van der Eijk, Richard Molenkamp, Marion Koopmans, on behalf of the Dutch national COVID-19 response team.
EPI_ISL_722851	I.R.C.C.S. "S. De Bellis" - Ente Ospedaliero	Istituto Zooprofilattico Sperimentale della Puglia e della Basilicata	Parisi A., Bianco A., Capozzi L., Del Sambro L., Lippolis A., Notarnicola M., Manzulli V, Rondinone V., Pace L.
EPI_ISL_722852	I.R.C.C.S. "S. De Bellis" - Ente Ospedaliero	Istituto Zooprofilattico Sperimentale della Puglia e della Basilicata	Parisi A., Bianco A., Capozzi L., Del Sambro L., Lippolis A., Notarnicola M., Cipolletta D., Galante D.
EPI_ISL_722853	I.R.C.C.S. "S. De Bellis" - Ente Ospedaliero	Istituto Zooprofilattico Sperimentale della Puglia e della Basilicata	Parisi A., Bianco A., Capozzi L., Del Sambro L., Lippolis A., Notarnicola M., Manzulli V, Rondinone V., Pace L.

EPI_ISL_722854	I.R.C.C.S. "S. De Bellis" - Ente Ospedaliero	Istituto Zooprofilattico Sperimentale della Puglia e della Basilicata	Parisi A., Bianco A., Capozzi L., Del Sambro L., Lippolis A., Notarnicola M., Cipolletta D., Galante D.
EPI_ISL_722855, EPI_ISL_722856, EPI_ISL_722857, EPI_ISL_722858	Dipartimento di Scienze Biomediche e Oncologia Umana - Azienda Ospedaliero Universitaria Consorziale Policlinico	Istituto Zooprofilattico Sperimentale della Puglia e della Basilicata	Parisi A., Bianco A., Capozzi L., Del Sambro L., Chironna M., Loconsole D.
EPI_ISL_722859, EPI_ISL_722860	I.R.C.C.S. "S. De Bellis" - Ente Ospedaliero	Istituto Zooprofilattico Sperimentale della Puglia e della Basilicata	Parisi A., Bianco A., Capozzi L., Del Sambro L., Lippolis A., Notarnicola M., Manzulli V, Rondinone V., Pace L.
EPI_ISL_722861	I.R.C.C.S. "S. De Bellis" - Ente Ospedaliero	Istituto Zooprofilattico Sperimentale della Puglia e della Basilicata	Parisi A., Bianco A., Capozzi L., Del Sambro L., Lippolis A., Notarnicola M., Cipolletta D., Galante D.
EPI_ISL_722862	I.R.C.C.S. "S. De Bellis" - Ente Ospedaliero	Istituto Zooprofilattico Sperimentale della Puglia e della Basilicata	Parisi A., Bianco A., Capozzi L., Del Sambro L., Lippolis A., Notarnicola M., Manzulli V, Rondinone V., Pace L.
EPI_ISL_722863	I.R.C.C.S. "S. De Bellis" - Ente Ospedaliero	Istituto Zooprofilattico Sperimentale della Puglia e della Basilicata	Parisi A., Bianco A., Capozzi L., Del Sambro L., Lippolis A., Notarnicola M., Cipolletta D., Galante D.
EPI_ISL_722864, EPI_ISL_722865	I.R.C.C.S. "S. De Bellis" - Ente Ospedaliero	Istituto Zooprofilattico Sperimentale della Puglia e della Basilicata	Parisi A., Bianco A., Capozzi L., Del Sambro L., Lippolis A., Notarnicola M., Manzulli V, Rondinone V., Pace L.
EPI_ISL_722866	I.R.C.C.S. "S. De Bellis" - Ente Ospedaliero	Istituto Zooprofilattico Sperimentale della Puglia e della Basilicata	Parisi A., Bianco A., Capozzi L., Del Sambro L., Lippolis A., Notarnicola M., Cipolletta D., Galante D.
EPI_ISL_722867, EPI_ISL_722868	I.R.C.C.S. "S. De Bellis" - Ente Ospedaliero	Istituto Zooprofilattico Sperimentale della Puglia e della Basilicata	Parisi A., Bianco A., Capozzi L., Del Sambro L., Lippolis A., Notarnicola M., Manzulli V, Rondinone V., Pace L.
EPI_ISL_722869	I.R.C.C.S. "S. De Bellis" - Ente Ospedaliero	Istituto Zooprofilattico Sperimentale della Puglia e della Basilicata	Parisi A., Bianco A., Capozzi L., Del Sambro L., Lippolis A., Notarnicola M., Cipolletta D., Galante D.
EPI_ISL_722870	I.R.C.C.S. "S. De Bellis" - Ente Ospedaliero	Istituto Zooprofilattico Sperimentale della Puglia e della Basilicata	Parisi A., Bianco A., Capozzi L., Del Sambro L., Lippolis A., Notarnicola M., Manzulli V, Rondinone V., Pace L.
EPI_ISL_722871	I.R.C.C.S. "S. De Bellis" - Ente Ospedaliero	Istituto Zooprofilattico Sperimentale della Puglia e della Basilicata	Parisi A., Bianco A., Capozzi L., Del Sambro L., Lippolis A., Notarnicola M., Cipolletta D., Galante D.
EPI_ISL_722872	Dipartimento di Scienze Biomediche e Oncologia Umana - Azienda Ospedaliero Universitaria Consorziale Policlinico	Istituto Zooprofilattico Sperimentale della Puglia e della Basilicata	Parisi A., Bianco A., Capozzi L., Del Sambro L., Chironna M., Loconsole D.
EPI_ISL_722873, EPI_ISL_722874, EPI_ISL_722875, EPI_ISL_722876, EPI_ISL_722877, EPI_ISL_722878, EPI_ISL_722879, EPI_ISL_722880, EPI_ISL_722881, EPI_ISL_722882, EPI_ISL_722883, EPI_ISL_722884, EPI_ISL_722885, EPI_ISL_722886, EPI_ISL_722887, EPI_ISL_722888, EPI_ISL_722889, EPI_ISL_722890, EPI_ISL_722891, EPI_ISL_722892, EPI_ISL_722893, EPI_ISL_722894			
see above	Istituto Zooprofilattico Sperimentale della Puglia e della Basilicata	Istituto Zooprofilattico Sperimentale della Puglia e della Basilicata	Parisi A., Bianco A., Capozzi L., Del Sambro L., Manzulli V, Rondinone V., Pace L., Cipolletta D., Galante D.
EPI_ISL_722895	I.R.C.C.S. "S. De Bellis" - Ente Ospedaliero	Istituto Zooprofilattico Sperimentale della Puglia e della Basilicata	Parisi A., Bianco A., Capozzi L., Del Sambro L., Lippolis A., Notarnicola M., Cipolletta D., Galante D.
EPI_ISL_722896, EPI_ISL_722897	Dipartimento di Scienze Biomediche e Oncologia Umana - Azienda Ospedaliero Universitaria Consorziale Policlinico	Istituto Zooprofilattico Sperimentale della Puglia e della Basilicata	Parisi A., Bianco A., Capozzi L., Del Sambro L., Chironna M., Loconsole D.
EPI_ISL_722898, EPI_ISL_722900	I.R.C.C.S. "S. De Bellis" - Ente Ospedaliero	Istituto Zooprofilattico Sperimentale della Puglia e della Basilicata	Parisi A., Bianco A., Capozzi L., Del Sambro L., Lippolis A., Notarnicola M., Cipolletta D., Galante D.
EPI_ISL_722901, EPI_ISL_722902, EPI_ISL_722903, EPI_ISL_722904, EPI_ISL_722905, EPI_ISL_722906, EPI_ISL_722907, EPI_ISL_722908, EPI_ISL_722909, EPI_ISL_722910, EPI_ISL_722911, EPI_ISL_722913, EPI_ISL_722914, EPI_ISL_722915, EPI_ISL_722916, EPI_ISL_722917, EPI_ISL_722918, EPI_ISL_722919, EPI_ISL_722920, EPI_ISL_722921, EPI_ISL_722922, EPI_ISL_722923, EPI_ISL_722924, EPI_ISL_722925			
see above	Istituto Zooprofilattico Sperimentale della Puglia e della Basilicata	Istituto Zooprofilattico Sperimentale della Puglia e della Basilicata	Parisi A., Bianco A., Capozzi L., Del Sambro L., Manzulli V, Rondinone V., Pace L., Cipolletta D., Galante D.
EPI_ISL_722926, EPI_ISL_722927, EPI_ISL_722928, EPI_ISL_722929, EPI_ISL_722930, EPI_ISL_722931, EPI_ISL_722932, EPI_ISL_722933, EPI_ISL_722934, EPI_ISL_722935, EPI_ISL_722936, EPI_ISL_722937, EPI_ISL_722938, EPI_ISL_722939, EPI_ISL_722940, EPI_ISL_722941, EPI_ISL_722942, EPI_ISL_722943, EPI_ISL_722944, EPI_ISL_722945, EPI_ISL_722946, EPI_ISL_722947, EPI_ISL_722948, EPI_ISL_722949, EPI_ISL_722950, EPI_ISL_722951, EPI_ISL_722952, EPI_ISL_722953, EPI_ISL_722954, EPI_ISL_722955, EPI_ISL_722956, EPI_ISL_722957, EPI_ISL_722958, EPI_ISL_722959, EPI_ISL_722960, EPI_ISL_722961, EPI_ISL_722962, EPI_ISL_722963, EPI_ISL_722964, EPI_ISL_722965, EPI_ISL_722966			
see above	Department of Clinical Microbiology	GIGA Medical Genomics	Keith Durkin, Maria Artesi, Sébastien Bontems, Raphaël Boreux, Bouchra Boujemla, Cécile Meex, Pierrette Melin, Marie-Pierre Hayette, Vincent Bours
EPI_ISL_722967, EPI_ISL_722968, EPI_ISL_722969, EPI_ISL_722970, EPI_ISL_722971, EPI_ISL_722972, EPI_ISL_722973, EPI_ISL_722974, EPI_ISL_722975, EPI_ISL_722976, EPI_ISL_722977, EPI_ISL_722978, EPI_ISL_722979, EPI_ISL_722980, EPI_ISL_722981, EPI_ISL_722982, EPI_ISL_722983, EPI_ISL_722984, EPI_ISL_722985, EPI_ISL_722986, EPI_ISL_722987, EPI_ISL_722988, EPI_ISL_722989, EPI_ISL_722990, EPI_ISL_722991, EPI_ISL_722992, EPI_ISL_722993, EPI_ISL_722994, EPI_ISL_722995, EPI_ISL_722996, EPI_ISL_722997, EPI_ISL_722998, EPI_ISL_722999, EPI_ISL_723000, EPI_ISL_723001, EPI_ISL_723002, EPI_ISL_723003, EPI_ISL_723004, EPI_ISL_723005, EPI_ISL_723006, EPI_ISL_723007, EPI_ISL_723008, EPI_ISL_723009, EPI_ISL_723010, EPI_ISL_723011, EPI_ISL_723012, EPI_ISL_723013, EPI_ISL_723014, EPI_ISL_723015, EPI_ISL_723016, EPI_ISL_723017, EPI_ISL_723018, EPI_ISL_723019, EPI_ISL_723020, EPI_ISL_723021, EPI_ISL_723022, EPI_ISL_723023, EPI_ISL_723024, EPI_ISL_723025, EPI_ISL_723026, EPI_ISL_723027, EPI_ISL_723028, EPI_ISL_723029, EPI_ISL_723030, EPI_ISL_723031, EPI_ISL_723032, EPI_ISL_723033, EPI_ISL_723034, EPI_ISL_723035, EPI_ISL_723036, EPI_ISL_723037, EPI_ISL_723038, EPI_ISL_723039, EPI_ISL_723040, EPI_ISL_723041, EPI_ISL_723042, EPI_ISL_723043, EPI_ISL_723044			
see above	Respiratory Virus Unit, National Infection Service, Public Health England	COVID-19 Genomics UK (COG-UK) Consortium	PHE Covid Sequencing Team
EPI_ISL_723048, EPI_ISL_723049, EPI_ISL_723050, EPI_ISL_723051, EPI_ISL_723052, EPI_ISL_723053, EPI_ISL_723054, EPI_ISL_723055, EPI_ISL_723056, EPI_ISL_723057, EPI_ISL_723058, EPI_ISL_723059, EPI_ISL_723060, EPI_ISL_723061, EPI_ISL_723062, EPI_ISL_723063, EPI_ISL_723064, EPI_ISL_723065, EPI_ISL_723066, EPI_ISL_723067, EPI_ISL_723068, EPI_ISL_723069, EPI_ISL_723070, EPI_ISL_723071, EPI_ISL_723072, EPI_ISL_723073, EPI_ISL_723074, EPI_ISL_723075, EPI_ISL_723076			
see above	Hematopathology Laboratory, ACTREC, TMC	Hematopathology Laboratory, ACTREC, TMC	Hematopathology Laboratory, ACTREC
EPI_ISL_723119, EPI_ISL_723120, EPI_ISL_723121, EPI_ISL_723122, EPI_ISL_723123, EPI_ISL_723124, EPI_ISL_723125, EPI_ISL_723126, EPI_ISL_723127, EPI_ISL_723128, EPI_ISL_723129, EPI_ISL_723130, EPI_ISL_723131, EPI_ISL_723132, EPI_ISL_723133, EPI_ISL_723134, EPI_ISL_723135, EPI_ISL_723136, EPI_ISL_723137, EPI_ISL_723138, EPI_ISL_723139, EPI_ISL_723140			
see above	Minnesota Department of Health, Public Health Laboratory	Minnesota Department of Health, Public Health Laboratory	Alexandra Lorentz, Jacob Garfin, Matt Plumb, and Xiong Wang
EPI_ISL_723141, EPI_ISL_723142, EPI_ISL_723143, EPI_ISL_723144, EPI_ISL_723145	Mayo Clinic & Mayo Clinic Laboratories	Minnesota Department of Health, Public Health Laboratory	Alexandra Lorentz, Jacob Garfin, Matt Plumb, and Xiong Wang
EPI_ISL_723146	Minnesota Department of Health, Public Health Laboratory	Minnesota Department of Health, Public Health Laboratory	Alexandra Lorentz, Jacob Garfin, Matt Plumb, and Xiong Wang
EPI_ISL_723147, EPI_ISL_723148, EPI_ISL_723149, EPI_ISL_723150, EPI_ISL_723151, EPI_ISL_723152, EPI_ISL_723153, EPI_ISL_723154	Vault Health	Minnesota Department of Health, Public Health Laboratory	Alexandra Lorentz, Jacob Garfin, Matt Plumb, and Xiong Wang
EPI_ISL_723155, EPI_ISL_723156, EPI_ISL_723157, EPI_ISL_723158, EPI_ISL_723159, EPI_ISL_723160, EPI_ISL_723161, EPI_ISL_723162, EPI_ISL_723163, EPI_ISL_723164, EPI_ISL_723165, EPI_ISL_723166, EPI_ISL_723167, EPI_ISL_723168, EPI_ISL_723169, EPI_ISL_723170, EPI_ISL_723171, EPI_ISL_723172, EPI_ISL_723173, EPI_ISL_723174, EPI_ISL_723175, EPI_ISL_723176, EPI_ISL_723177, EPI_ISL_723178, EPI_ISL_723179, EPI_ISL_723180, EPI_ISL_723181, EPI_ISL_723182, EPI_ISL_723183, EPI_ISL_723184, EPI_ISL_723185, EPI_ISL_723186, EPI_ISL_723187, EPI_ISL_723188, EPI_ISL_723189, EPI_ISL_723190, EPI_ISL_723191, EPI_ISL_723192, EPI_ISL_723193, EPI_ISL_723194, EPI_ISL_723195, EPI_ISL_723196, EPI_ISL_723197, EPI_ISL_723198, EPI_ISL_723199, EPI_ISL_723200, EPI_ISL_723201, EPI_ISL_723202, EPI_ISL_723203, EPI_ISL_723204, EPI_ISL_723205, EPI_ISL_723206, EPI_ISL_723207, EPI_ISL_723208, EPI_ISL_723209, EPI_ISL_723210, EPI_ISL_723211, EPI_ISL_723212, EPI_ISL_723213, EPI_ISL_723214, EPI_ISL_723215, EPI_ISL_723216, EPI_ISL_723217, EPI_ISL_723218, EPI_ISL_723219, EPI_ISL_723220, EPI_ISL_723221, EPI_ISL_723222, EPI_ISL_723223, EPI_ISL_723224, EPI_ISL_723225, EPI_ISL_723226, EPI_ISL_723227, EPI_ISL_723228, EPI_ISL_723229, EPI_ISL_723230, EPI_ISL_723231, EPI_ISL_723232, EPI_ISL_723233, EPI_ISL_723234, EPI_ISL_723235, EPI_ISL_723236, EPI_ISL_723237, EPI_ISL_723238, EPI_ISL_723239, EPI_ISL_723240, EPI_ISL_723241, EPI_ISL_723242, EPI_ISL_723243, EPI_ISL_723244, EPI_ISL_723245, EPI_ISL_723246, EPI_ISL_723247, EPI_ISL_723248, EPI_ISL_723249, EPI_ISL_723250, EPI_ISL_723251, EPI_ISL_723252, EPI_ISL_723253, EPI_ISL_723254, EPI_ISL_723255, EPI_ISL_723256, EPI_ISL_723257, EPI_ISL_723258, EPI_ISL_723259, EPI_ISL_723260, EPI_ISL_723261, EPI_ISL_723262, EPI_ISL_723263, EPI_ISL_723264, EPI_ISL_723265, EPI_ISL_723266, EPI_ISL_723267, EPI_ISL_723268, EPI_ISL_723269, EPI_ISL_723270, EPI_ISL_723271, EPI_ISL_723272, EPI_ISL_723273, EPI_ISL_723274, EPI_ISL_723275, EPI_ISL_723276, EPI_ISL_723277, EPI_ISL_723278, EPI_ISL_723279, EPI_ISL_723280,			

EPI_ISL_723281, EPI_ISL_723282, EPI_ISL_723283, EPI_ISL_723284, EPI_ISL_723285, EPI_ISL_723286, EPI_ISL_723287, EPI_ISL_723288, EPI_ISL_723289, EPI_ISL_723290, EPI_ISL_723291, EPI_ISL_723292, EPI_ISL_723293, EPI_ISL_723294, EPI_ISL_723295, EPI_ISL_723296, EPI_ISL_723297, EPI_ISL_723298, EPI_ISL_723299, EPI_ISL_723300, EPI_ISL_723301, EPI_ISL_723302, EPI_ISL_723303, EPI_ISL_723304, EPI_ISL_723305, EPI_ISL_723306, EPI_ISL_723307, EPI_ISL_723308, EPI_ISL_723309, EPI_ISL_723310, EPI_ISL_723311, EPI_ISL_723312, EPI_ISL_723313, EPI_ISL_723314, EPI_ISL_723315, EPI_ISL_723316, EPI_ISL_723317, EPI_ISL_723318, EPI_ISL_723319, EPI_ISL_723320, EPI_ISL_723321, EPI_ISL_723322, EPI_ISL_723323, EPI_ISL_723324, EPI_ISL_723325, EPI_ISL_723326, EPI_ISL_723327, EPI_ISL_723328, EPI_ISL_723329, EPI_ISL_723330, EPI_ISL_723331, EPI_ISL_723332, EPI_ISL_723333, EPI_ISL_723334, EPI_ISL_723335, EPI_ISL_723336, EPI_ISL_723337, EPI_ISL_723338, EPI_ISL_723339, EPI_ISL_723340, EPI_ISL_723341, EPI_ISL_723342, EPI_ISL_723343, EPI_ISL_723344, EPI_ISL_723345, EPI_ISL_723346, EPI_ISL_723347, EPI_ISL_723348, EPI_ISL_723349, EPI_ISL_723350, EPI_ISL_723351, EPI_ISL_723352, EPI_ISL_723353, EPI_ISL_723354, EPI_ISL_723355, EPI_ISL_723356, EPI_ISL_723357, EPI_ISL_723358, EPI_ISL_723359, EPI_ISL_723360, EPI_ISL_723361, EPI_ISL_723362, EPI_ISL_723363, EPI_ISL_723364, EPI_ISL_723365, EPI_ISL_723366, EPI_ISL_723367, EPI_ISL_723368, EPI_ISL_723369, EPI_ISL_723370, EPI_ISL_723371, EPI_ISL_723372, EPI_ISL_723373, EPI_ISL_723374, EPI_ISL_723375, EPI_ISL_723376, EPI_ISL_723377, EPI_ISL_723378, EPI_ISL_723379, EPI_ISL_723380, EPI_ISL_723381, EPI_ISL_723382, EPI_ISL_723383, EPI_ISL_723384, EPI_ISL_723385, EPI_ISL_723386, EPI_ISL_723387, EPI_ISL_723388, EPI_ISL_723389, EPI_ISL_723390, EPI_ISL_723391, EPI_ISL_723392, EPI_ISL_723393, EPI_ISL_723394, EPI_ISL_723395, EPI_ISL_723396, EPI_ISL_723397, EPI_ISL_723398, EPI_ISL_723399, EPI_ISL_723400, EPI_ISL_723401, EPI_ISL_723402, EPI_ISL_723403, EPI_ISL_723404, EPI_ISL_723405, EPI_ISL_723406, EPI_ISL_723407, EPI_ISL_723408, EPI_ISL_723409, EPI_ISL_723410, EPI_ISL_723411, EPI_ISL_723412, EPI_ISL_723413, EPI_ISL_723414, EPI_ISL_723415, EPI_ISL_723416, EPI_ISL_723417, EPI_ISL_723418, EPI_ISL_723419, EPI_ISL_723420, EPI_ISL_723421, EPI_ISL_723422, EPI_ISL_723423, EPI_ISL_723424, EPI_ISL_723425, EPI_ISL_723426, EPI_ISL_723427, EPI_ISL_723428, EPI_ISL_723429, EPI_ISL_723430, EPI_ISL_723431, EPI_ISL_723432, EPI_ISL_723433, EPI_ISL_723434, EPI_ISL_723435, EPI_ISL_723436, EPI_ISL_723437, EPI_ISL_723438, EPI_ISL_723439, EPI_ISL_723440, EPI_ISL_723441, EPI_ISL_723442, EPI_ISL_723443, EPI_ISL_723444, EPI_ISL_723445, EPI_ISL_723446, EPI_ISL_723447, EPI_ISL_723448, EPI_ISL_723449, EPI_ISL_723450, EPI_ISL_723451, EPI_ISL_723452, EPI_ISL_723453, EPI_ISL_723454, EPI_ISL_723455, EPI_ISL_723456, EPI_ISL_723457, EPI_ISL_723458, EPI_ISL_723459, EPI_ISL_723460, EPI_ISL_723461, EPI_ISL_723462, EPI_ISL_723463, EPI_ISL_723464, EPI_ISL_723465, EPI_ISL_723466, EPI_ISL_723467			
see above	Dutch COVID-19 response team	National Institute for Public Health and the Environment (RIVM)	Adam Meijer, Harry Vennema, Jeroen Cremer, Sharon van den Brink, Bas van der Veer, AnneMarie van den Brandt, Florian Zwagemaker, Dennis Schmitz, Chantal Reusken, on behalf of the national COVID-19 response team
EPI_ISL_723470, EPI_ISL_723471, EPI_ISL_723472, EPI_ISL_723473, EPI_ISL_723474, EPI_ISL_723475, EPI_ISL_723476, EPI_ISL_723477, EPI_ISL_723478, EPI_ISL_723479, EPI_ISL_723480, EPI_ISL_723481, EPI_ISL_723482, EPI_ISL_723483, EPI_ISL_723484, EPI_ISL_723485, EPI_ISL_723486, EPI_ISL_723487, EPI_ISL_723488, EPI_ISL_723489, EPI_ISL_723490, EPI_ISL_723491			
see above	Virginia DCLS	Virginia DCLS	Virginia DCLS
EPI_ISL_723492, EPI_ISL_723493, EPI_ISL_723494, EPI_ISL_723495, EPI_ISL_723496, EPI_ISL_723497, EPI_ISL_723498, EPI_ISL_723499, EPI_ISL_723500, EPI_ISL_723501, EPI_ISL_723502, EPI_ISL_723503, EPI_ISL_723504, EPI_ISL_723505, EPI_ISL_723506, EPI_ISL_723507, EPI_ISL_723508, EPI_ISL_723509, EPI_ISL_723510, EPI_ISL_723511, EPI_ISL_723512, EPI_ISL_723513, EPI_ISL_723514, EPI_ISL_723515, EPI_ISL_723516, EPI_ISL_723517, EPI_ISL_723518, EPI_ISL_723519, EPI_ISL_723520			
see above	Virginia Division of Consolidated Laboratory Services (DCLS)	Virginia Division of Consolidated Laboratory Services (DCLS)	Virginia DCLS
EPI_ISL_723521, EPI_ISL_723522, EPI_ISL_723523, EPI_ISL_723524, EPI_ISL_723525, EPI_ISL_723526, EPI_ISL_723527, EPI_ISL_723528, EPI_ISL_723529, EPI_ISL_723530, EPI_ISL_723531, EPI_ISL_723532, EPI_ISL_723533, EPI_ISL_723534, EPI_ISL_723535, EPI_ISL_723536, EPI_ISL_723537, EPI_ISL_723538, EPI_ISL_723539, EPI_ISL_723540, EPI_ISL_723541, EPI_ISL_723542, EPI_ISL_723543, EPI_ISL_723544, EPI_ISL_723545, EPI_ISL_723546, EPI_ISL_723547, EPI_ISL_723548, EPI_ISL_723549, EPI_ISL_723550, EPI_ISL_723551, EPI_ISL_723552, EPI_ISL_723553, EPI_ISL_723554, EPI_ISL_723555, EPI_ISL_723556, EPI_ISL_723557			
see above	Centre for Enzyme Innovation, University of Portsmouth / Translational Research Laboratory, Portsmouth Hospitals NHS Trust	COVID-19 Genomics UK (COG-UK) Consortium	Angela Beckett,Yann Bourgeois,Garry Scarlett,Sharon Glaysher,Scott Elliott,Kelly Bicknell,Robert Impey,Allyson Lloyd,Sarah Wyllie,Ethan Butcher,Anoop Chauhan,Samuel Robson
EPI_ISL_723558, EPI_ISL_723559, EPI_ISL_723560, EPI_ISL_723561, EPI_ISL_723562, EPI_ISL_723563, EPI_ISL_723564, EPI_ISL_723565, EPI_ISL_723566, EPI_ISL_723567, EPI_ISL_723568, EPI_ISL_723569, EPI_ISL_723570, EPI_ISL_723571, EPI_ISL_723572, EPI_ISL_723574, EPI_ISL_723575, EPI_ISL_723576, EPI_ISL_723577, EPI_ISL_723578, EPI_ISL_723579, EPI_ISL_723580, EPI_ISL_723581, EPI_ISL_723582, EPI_ISL_723583, EPI_ISL_723584, EPI_ISL_723585, EPI_ISL_723586, EPI_ISL_723587, EPI_ISL_723588, EPI_ISL_723589, EPI_ISL_723590, EPI_ISL_723591, EPI_ISL_723592, EPI_ISL_723593, EPI_ISL_723594, EPI_ISL_723595, EPI_ISL_723596, EPI_ISL_723597, EPI_ISL_723598, EPI_ISL_723599, EPI_ISL_723600, EPI_ISL_723601, EPI_ISL_723602, EPI_ISL_723603, EPI_ISL_723604, EPI_ISL_723605, EPI_ISL_723606, EPI_ISL_723607, EPI_ISL_723608, EPI_ISL_723609, EPI_ISL_723610, EPI_ISL_723611, EPI_ISL_723612, EPI_ISL_723614, EPI_ISL_723615, EPI_ISL_723616, EPI_ISL_723617, EPI_ISL_723618, EPI_ISL_723619, EPI_ISL_723620, EPI_ISL_723621, EPI_ISL_723622, EPI_ISL_723624, EPI_ISL_723625, EPI_ISL_723626, EPI_ISL_723628, EPI_ISL_723629, EPI_ISL_723630			
see above	Northumbria University / South Tees Hospitals NHS Foundation Trust / North Cumbria Integrated Care NHS Foundation Trust / North Tees and Hartlepool NHS Foundation Trust / Newcastle Hospitals NHS Foundation Trust	COVID-19 Genomics UK (COG-UK) Consortium	Darren L Smith,Andrew Nelson,Matthew Bashton,Greg R Young,Joshua Loh,John Allan,Mohammad A Tariq,Giles S Holt,Gary Black,Wen C Yew,Lynn Dover,Paul Baker,Steve Liggett,Sarah Essex,Jane Greenaway,Debra Padgett,Clive Graham,Garren Scott,Edward Barton,Emma Swindells,Brendan Payne,Jennifer Collins,Yusri Taha,Gary Eltringham
EPI_ISL_723631, EPI_ISL_723632, EPI_ISL_723633, EPI_ISL_723634, EPI_ISL_723635, EPI_ISL_723636, EPI_ISL_723637, EPI_ISL_723638, EPI_ISL_723639, EPI_ISL_723640, EPI_ISL_723641, EPI_ISL_723642, EPI_ISL_723643, EPI_ISL_723644, EPI_ISL_723645, EPI_ISL_723646, EPI_ISL_723647, EPI_ISL_723648, EPI_ISL_723649, EPI_ISL_723650, EPI_ISL_723651, EPI_ISL_723652, EPI_ISL_723653, EPI_ISL_723654, EPI_ISL_723655, EPI_ISL_723656, EPI_ISL_723657, EPI_ISL_723658, EPI_ISL_723659, EPI_ISL_723660, EPI_ISL_723661, EPI_ISL_723662, EPI_ISL_723663, EPI_ISL_723664, EPI_ISL_723665, EPI_ISL_723666, EPI_ISL_723667, EPI_ISL_723668, EPI_ISL_723669, EPI_ISL_723670, EPI_ISL_723671, EPI_ISL_723672			
see above	Oxford Viromics, NDM, University of Oxford; Oxford University Hospitals; Basingstoke and North Hampshire Hospital	COVID-19 Genomics UK (COG-UK) Consortium	Tanya Golubchik, David Bonsall, George Macintyre, Amy Trebes, Mariateresa de Cesare, Catrin Moore, Alex Mobbs, Anita Justice, Robert Shaw, Monique Andersson, Timothy Peto, Emma Wise, Nathan Moore, Jessica Lynch, Nick Cortes, Matilde Mori, Stephen Kidd, David Buck, John Todd, Christophe Fraser
EPI_ISL_723673	Quadram Institute Bioscience	COVID-19 Genomics UK (COG-UK) Consortium	Dave J. Baker, Gemma L. Kay, Alp Aydin, Thanh Le-Viet, Steven Rudder, Ana P. Tedim, Anastasia Kolyva, Maria Diaz, Leonardo de Oliveira Martins, Nabil-Fareed Alikhan, Lizzie Meadows, Rachael Stanley, Ngozi Elumogo, Muhammed Yasir, Nicholas M. Thomson, Alexander J Trotter, Rachel Gilroy, Samuel Bloomfield, Claire Stuart, Andrew Bell, Reenesh Prakash, Samir Dervisevic, Alison E. Mather, John Wain, Mark Webber, Andrew J. Page, Justin O'Grady
EPI_ISL_723674, EPI_ISL_723675, EPI_ISL_723676, EPI_ISL_723677, EPI_ISL_723678, EPI_ISL_723679, EPI_ISL_723680, EPI_ISL_723681, EPI_ISL_723682, EPI_ISL_723683, EPI_ISL_723684, EPI_ISL_723685, EPI_ISL_723686, EPI_ISL_723687, EPI_ISL_723688, EPI_ISL_723689, EPI_ISL_723690, EPI_ISL_723691, EPI_ISL_723692, EPI_ISL_723693, EPI_ISL_723694, EPI_ISL_723695, EPI_ISL_723696, EPI_ISL_723697, EPI_ISL_723698, EPI_ISL_723699, EPI_ISL_723700, EPI_ISL_723701, EPI_ISL_723702, EPI_ISL_723703, EPI_ISL_723704, EPI_ISL_723705, EPI_ISL_723706, EPI_ISL_723707, EPI_ISL_723708, EPI_ISL_723709, EPI_ISL_723710, EPI_ISL_723711, EPI_ISL_723712, EPI_ISL_723713, EPI_ISL_723714, EPI_ISL_723715, EPI_ISL_723716, EPI_ISL_723717, EPI_ISL_723718, EPI_ISL_723719, EPI_ISL_723720, EPI_ISL_723721, EPI_ISL_723722, EPI_ISL_723723, EPI_ISL_723724, EPI_ISL_723725, EPI_ISL_723726, EPI_ISL_723727, EPI_ISL_723728, EPI_ISL_723729, EPI_ISL_723730, EPI_ISL_723731, EPI_ISL_723732, EPI_ISL_723733, EPI_ISL_723734, EPI_ISL_723735, EPI_ISL_723736, EPI_ISL_723737, EPI_ISL_723738, EPI_ISL_723739, EPI_ISL_723740, EPI_ISL_723741, EPI_ISL_723742, EPI_ISL_723743, EPI_ISL_723744, EPI_ISL_723745, EPI_ISL_723746, EPI_ISL_723747, EPI_ISL_723748, EPI_ISL_723749, EPI_ISL_723750, EPI_ISL_723751, EPI_ISL_723752, EPI_ISL_723753, EPI_ISL_723754, EPI_ISL_723755, EPI_ISL_723756, EPI_ISL_723757, EPI_ISL_723758, EPI_ISL_723759, EPI_ISL_723760, EPI_ISL_723761, EPI_ISL_723762, EPI_ISL_723763, EPI_ISL_723764, EPI_ISL_723765, EPI_ISL_723766, EPI_ISL_723767, EPI_ISL_723768, EPI_ISL_723769, EPI_ISL_723770, EPI_ISL_723771, EPI_ISL_723772, EPI_ISL_723773, EPI_ISL_723774, EPI_ISL_723775, EPI_ISL_723776, EPI_ISL_723777, EPI_ISL_723778, EPI_ISL_723779, EPI_ISL_723780, EPI_ISL_723781, EPI_ISL_723782, EPI_ISL_723783, EPI_ISL_723784, EPI_ISL_723785, EPI_ISL_723786, EPI_ISL_723787, EPI_ISL_723788, EPI_ISL_723789, EPI_ISL_723790, EPI_ISL_723791, EPI_ISL_723792, EPI_ISL_723793, EPI_ISL_723794, EPI_ISL_723795, EPI_ISL_723796, EPI_ISL_723797, EPI_ISL_723798, EPI_ISL_723799, EPI_ISL_723800, EPI_ISL_723801, EPI_ISL_723802, EPI_ISL_723803, EPI_ISL_723804, EPI_ISL_723805, EPI_ISL_723806, EPI_ISL_723807, EPI_ISL_723808, EPI_ISL_723809, EPI_ISL_723810, EPI_ISL_723811, EPI_ISL_723812, EPI_ISL_723813, EPI_ISL_723814, EPI_ISL_723815, EPI_ISL_723816, EPI_ISL_723817, EPI_ISL_723818, EPI_ISL_723819, EPI_ISL_723820, EPI_ISL_723821, EPI_ISL_723822, EPI_ISL_723823, EPI_ISL_723824, EPI_ISL_723825, EPI_ISL_723826, EPI_ISL_723827, EPI_ISL_723828, EPI_ISL_723829, EPI_ISL_723830, EPI_ISL_723831, EPI_ISL_723832, EPI_ISL_723833, EPI_ISL_723834, EPI_ISL_723835, EPI_ISL_723836, EPI_ISL_723837, EPI_ISL_723838, EPI_ISL_723839, EPI_ISL_723840, EPI_ISL_723841, EPI_ISL_723842, EPI_ISL_723843, EPI_ISL_723844, EPI_ISL_723845, EPI_ISL_723846, EPI_ISL_723847, EPI_ISL_723848, EPI_ISL_723849, EPI_ISL_723850			
see above	Oxford Viromics, NDM, University of Oxford; Oxford University Hospitals; Basingstoke and North Hampshire Hospital	COVID-19 Genomics UK (COG-UK) Consortium	Tanya Golubchik, David Bonsall, George Macintyre, Amy Trebes, Mariateresa de Cesare, Catrin Moore, Alex Mobbs, Anita Justice, Robert Shaw, Monique Andersson, Timothy Peto, Emma Wise, Nathan Moore, Jessica Lynch, Nick Cortes, Matilde Mori, Stephen Kidd, David Buck, John Todd, Christophe Fraser
EPI_ISL_723852, EPI_ISL_723853, EPI_ISL_723854, EPI_ISL_723856, EPI_ISL_723857, EPI_ISL_723858, EPI_ISL_723859, EPI_ISL_723860, EPI_ISL_723861, EPI_ISL_723862, EPI_ISL_723863, EPI_ISL_723864, EPI_ISL_723867, EPI_ISL_723869, EPI_ISL_723870, EPI_ISL_723871, EPI_ISL_723872, EPI_ISL_723873, EPI_ISL_723874, EPI_ISL_723875, EPI_ISL_723876, EPI_ISL_723877, EPI_ISL_723878, EPI_ISL_723879, EPI_ISL_723880, EPI_ISL_723882, EPI_ISL_723885, EPI_ISL_723886, EPI_ISL_723887, EPI_ISL_723888, EPI_ISL_723889, EPI_ISL_723890, EPI_ISL_723891, EPI_ISL_723892, EPI_ISL_723893, EPI_ISL_723894, EPI_ISL_723895, EPI_ISL_723896, EPI_ISL_723897, EPI_ISL_723898, EPI_ISL_723899, EPI_ISL_723901, EPI_ISL_723902, EPI_ISL_723903, EPI_ISL_723904, EPI_ISL_723905, EPI_ISL_723906, EPI_ISL_723907, EPI_ISL_723908, EPI_ISL_723909, EPI_ISL_723910, EPI_ISL_723911, EPI_ISL_723912, EPI_ISL_723913, EPI_ISL_723914, EPI_ISL_723915, EPI_ISL_723916, EPI_ISL_723919, EPI_ISL_723920, EPI_ISL_723921, EPI_ISL_723922, EPI_ISL_723923, EPI_ISL_723924, EPI_ISL_723925, EPI_ISL_723926, EPI_ISL_723927, EPI_ISL_723928, EPI_ISL_723929, EPI_ISL_723930, EPI_ISL_723931, EPI_ISL_723932, EPI_ISL_723933, EPI_ISL_723934, EPI_ISL_723935, EPI_ISL_723936, EPI_ISL_723937, EPI_ISL_723939, EPI_ISL_723940, EPI_ISL_723941, EPI_ISL_723942, EPI_ISL_723943, EPI_ISL_723944, EPI_ISL_723945, EPI_ISL_723946, EPI_ISL_723947			
see above	Department of Pathology, University of Cambridge	COVID-19 Genomics UK (COG-UK) Consortium	Aminu S. Jahun, Yasmin Chaudhry, Grant Hall, Iliana Georgana, Myra Hosmillo, Martin D. Curran, Malte Pinckert, Surendra Parmar, Ian Goodfellow
EPI_ISL_723949, EPI_ISL_723950, EPI_ISL_723951, EPI_ISL_723952, EPI_ISL_723953, EPI_ISL_723954, EPI_ISL_723955, EPI_ISL_723956, EPI_ISL_723957, EPI_ISL_723958, EPI_ISL_723959, EPI_ISL_723960, EPI_ISL_723961, EPI_ISL_723962, EPI_ISL_723963, EPI_ISL_723964, EPI_ISL_723965, EPI_ISL_723966, EPI_ISL_723967, EPI_ISL_723968, EPI_ISL_723969, EPI_ISL_723970, EPI_ISL_723971, EPI_ISL_723972, EPI_ISL_723973, EPI_ISL_723974, EPI_ISL_723975, EPI_ISL_723976, EPI_ISL_723977, EPI_ISL_723978, EPI_ISL_723979, EPI_ISL_723980, EPI_ISL_723981, EPI_ISL_723982, EPI_ISL_723983, EPI_ISL_723984, EPI_ISL_723985, EPI_ISL_723986, EPI_ISL_723987, EPI_ISL_723988, EPI_ISL_723989, EPI_ISL_723990, EPI_ISL_723991, EPI_ISL_723992, EPI_ISL_723993, EPI_ISL_723994, EPI_ISL_723995, EPI_ISL_723996, EPI_ISL_723997, EPI_ISL_723998, EPI_ISL_723999, EPI_ISL_724000, EPI_ISL_724001, EPI_ISL_724002, EPI_ISL_724003, EPI_ISL_724004, EPI_ISL_724005, EPI_ISL_724006, EPI_ISL_724007, EPI_ISL_724008, EPI_ISL_724009, EPI_ISL_724010, EPI_ISL_724011, EPI_ISL_724012, EPI_ISL_724013, EPI_ISL_724014, EPI_ISL_724015, EPI_ISL_724016, EPI_ISL_724017, EPI_ISL_724018, EPI_ISL_724019, EPI_ISL_724020, EPI_ISL_724021, EPI_ISL_724022, EPI_ISL_724023, EPI_ISL_724024, EPI_ISL_724025, EPI_ISL_724026, EPI_ISL_724027, EPI_ISL_724028, EPI_ISL_724029, EPI_ISL_724030, EPI_ISL_724031, EPI_ISL_724032, EPI_ISL_724033, EPI_ISL_724034, EPI_ISL_724035, EPI_ISL_724036, EPI_ISL_724037, EPI_ISL_724038, EPI_ISL_724039, EPI_ISL_724040, EPI_ISL_724041, EPI_ISL_724042, EPI_ISL_724043, EPI_ISL_724044, EPI_ISL_724045, EPI_ISL_724046, EPI_ISL_724047, EPI_ISL_724048, EPI_ISL_724049, EPI_ISL_724050, EPI_ISL_724051, EPI_ISL_724052, EPI_ISL_724053, EPI_ISL_724054, EPI_ISL_724055, EPI_ISL_724056, EPI_ISL_724057, EPI_ISL_724058, EPI_ISL_724059, EPI_ISL_724060, EPI_ISL_724061, EPI_ISL_724062			
see above	Oxford Viromics, NDM, University of Oxford; Oxford University Hospitals; Basingstoke and North Hampshire Hospital	COVID-19 Genomics UK (COG-UK) Consortium	Tanya Golubchik, David Bonsall, George Macintyre, Amy Trebes, Mariateresa de Cesare, Catrin Moore, Alex Mobbs, Anita Justice, Robert Shaw, Monique Andersson, Timothy Peto, Emma Wise, Nathan Moore, Jessica Lynch, Nick Cortes, Matilde Mori, Stephen Kidd, David Buck, John Todd, Christophe Fraser
EPI_ISL_724063, EPI_ISL_724064, EPI_ISL_724065, EPI_ISL_724066, EPI_ISL_724067, EPI_ISL_724068, EPI_ISL_724069, EPI_ISL_724070, EPI_ISL_724071, EPI_ISL_724072, EPI_ISL_724073, EPI_ISL_724074, EPI_ISL_724075, EPI_ISL_724076, EPI_ISL_724077, EPI_ISL_724078, EPI_ISL_724079, EPI_ISL_724081, EPI_ISL_724082, EPI_ISL_724083, EPI_ISL_724084, EPI_ISL_724085, EPI_ISL_724086, EPI_ISL_724087, EPI_ISL_724088, EPI_ISL_724089, EPI_ISL_724090, EPI_ISL_724091, EPI_ISL_724092, EPI_ISL_724093, EPI_ISL_724094, EPI_ISL_724096, EPI_ISL_724097, EPI_ISL_724100, EPI_ISL_724101, EPI_ISL_724102, EPI_ISL_724103, EPI_ISL_724104, EPI_ISL_724105, EPI_ISL_724106, EPI_ISL_724107, EPI_ISL_724108, EPI_ISL_724109, EPI_ISL_724110, EPI_ISL_724111, EPI_ISL_724112, EPI_ISL_724113, EPI_ISL_724114, EPI_ISL_724115, EPI_ISL_724116, EPI_ISL_724117, EPI_ISL_724118, EPI_ISL_724119, EPI_ISL_724120, EPI_ISL_724121, EPI_ISL_724122, EPI_ISL_724123			
see above	Northumbria University / South Tees Hospitals NHS Foundation Trust / North Cumbria Integrated Care NHS Foundation Trust / North Tees and Hartlepool NHS Foundation Trust / Newcastle Hospitals NHS Foundation Trust	COVID-19 Genomics UK (COG-UK) Consortium	Darren L Smith,Andrew Nelson,Matthew Bashton,Greg R Young,Joshua Loh,John Allan,Mohammad A Tariq,Giles S Holt,Gary Black,Wen C Yew,Lynn Dover,Paul Baker,Steve Liggett,Sarah Essex,Jane Greenaway,Debra Padgett,Clive Graham,Garren Scott,Edward Barton,Emma Swindells,Brendan Payne,Jennifer Collins,Yusri Taha,Gary Eltringham

EPI_ISL_724124, EPI_ISL_724125, EPI_ISL_724126, EPI_ISL_724127, EPI_ISL_724128, EPI_ISL_724129, EPI_ISL_724130, EPI_ISL_724131, EPI_ISL_724132, EPI_ISL_724133, EPI_ISL_724135, EPI_ISL_724136, EPI_ISL_724137, EPI_ISL_724138, EPI_ISL_724139, EPI_ISL_724140, EPI_ISL_724141, EPI_ISL_724142, EPI_ISL_724143, EPI_ISL_724144, EPI_ISL_724145, EPI_ISL_724146, EPI_ISL_724147, EPI_ISL_724148, EPI_ISL_724149, EPI_ISL_724150, EPI_ISL_724151, EPI_ISL_724152, EPI_ISL_724153, EPI_ISL_724154, EPI_ISL_724155, EPI_ISL_724156, EPI_ISL_724157, EPI_ISL_724158, EPI_ISL_724159, EPI_ISL_724160, EPI_ISL_724161, EPI_ISL_724162, EPI_ISL_724163, EPI_ISL_724164, EPI_ISL_724165, EPI_ISL_724166, EPI_ISL_724167, EPI_ISL_724168, EPI_ISL_724169, EPI_ISL_724170, EPI_ISL_724171, EPI_ISL_724172, EPI_ISL_724173, EPI_ISL_724174, EPI_ISL_724175, EPI_ISL_724176, EPI_ISL_724177, EPI_ISL_724178, EPI_ISL_724179, EPI_ISL_724180, EPI_ISL_724181, EPI_ISL_724182, EPI_ISL_724183, EPI_ISL_724184, EPI_ISL_724185, EPI_ISL_724186, EPI_ISL_724187, EPI_ISL_724188, EPI_ISL_724189, EPI_ISL_724190, EPI_ISL_724191, EPI_ISL_724192, EPI_ISL_724193, EPI_ISL_724194, EPI_ISL_724195, EPI_ISL_724197, EPI_ISL_724198, EPI_ISL_724199, EPI_ISL_724200, EPI_ISL_724201, EPI_ISL_724202, EPI_ISL_724203, EPI_ISL_724204, EPI_ISL_724205, EPI_ISL_724206, EPI_ISL_724207, EPI_ISL_724208, EPI_ISL_724209			
see above	Quadram Institute Bioscience	COVID-19 Genomics UK (COG-UK) Consortium	Dave J. Baker, Gemma L. Kay, Alp Aydin, Thanh Le-Viet, Steven Rudder, Ana P. Tedim, Anastasia Kolyva, Maria Diaz, Leonardo de Oliveira Martins, Nabil-Fareed Alikhan, Lizzie Meadows, Rachael Stanley, Ngozi Elumogo, Muhammed Yasir, Nicholas M. Thomson, Alexander J Trotter, Rachel Gilroy, Samuel Bloomfield, Claire Stuart, Andrew Bell, Reenesh Prakash, Samir Dervisevic, Alison E. Mather, John Wain, Mark Webber, Andrew J. Page, Justin O'Grady
EPI_ISL_724210, EPI_ISL_724211, EPI_ISL_724212, EPI_ISL_724213, EPI_ISL_724214, EPI_ISL_724215, EPI_ISL_724216, EPI_ISL_724217, EPI_ISL_724218, EPI_ISL_724219, EPI_ISL_724220, EPI_ISL_724221			
see above	West of Scotland Specialist Virology Centre, NHSGGC / MRC-University of Glasgow Centre for Virus Research	COVID-19 Genomics UK (COG-UK) Consortium	Ana da Silva Filipe, Natasha Johnson, Kathy Smollett, Daniel Mair, Stephen Carmichael, Alice Broos, Lily Tong, Jenna Nichols, Kyriaki Nomikou; Sarah McDonald; Richard Orton, Joseph Hughes, Sreenu Vattipally, David L Robertson; Alasdair MacLean, Rory Gunson; Sharif Shaaban, Matthew Holden; Rachel Blacow, Guy Mollett, Kathy Li, James Shepherd, Antonia Ho, Emma Thomson
EPI_ISL_724223, EPI_ISL_724224, EPI_ISL_724225, EPI_ISL_724226, EPI_ISL_724227, EPI_ISL_724228, EPI_ISL_724229, EPI_ISL_724230, EPI_ISL_724231, EPI_ISL_724232, EPI_ISL_724233, EPI_ISL_724234, EPI_ISL_724235, EPI_ISL_724236, EPI_ISL_724237, EPI_ISL_724238, EPI_ISL_724239, EPI_ISL_724240, EPI_ISL_724241, EPI_ISL_724242, EPI_ISL_724243, EPI_ISL_724244, EPI_ISL_724245, EPI_ISL_724246, EPI_ISL_724247, EPI_ISL_724248, EPI_ISL_724249, EPI_ISL_724250, EPI_ISL_724251, EPI_ISL_724252			
see above	Virology Department, Royal Infirmary of Edinburgh, NHS Lothian / School of Biological Sciences, University of Edinburgh / Institute of Genetics and Molecular Medicine, University of Edinburgh	COVID-19 Genomics UK (COG-UK) Consortium	McHugh M, Dewar R, Rooke S, Gallagher M, Balcaza C, O'Toole Á, Scher E, Hill V, McCrone JT, Colquhoun R, Yu X, Jackson B, Rambaut A, Williams TC, Templeton K
EPI_ISL_724268, EPI_ISL_724276, EPI_ISL_724278, EPI_ISL_724279, EPI_ISL_724283, EPI_ISL_724287, EPI_ISL_724290, EPI_ISL_724291, EPI_ISL_724294, EPI_ISL_724295, EPI_ISL_724300, EPI_ISL_724307, EPI_ISL_724318, EPI_ISL_724319			
see above	University of Exeter	COVID-19 Genomics UK (COG-UK) Consortium	Ben Temperton,Aaron Jeffries,Michelle Michelsen,Joanna Warwick-Dugdale,Audrey Farbos,Robyn Manley,Stephen Michell,Jane Masoli
EPI_ISL_724349, EPI_ISL_724351, EPI_ISL_724353, EPI_ISL_724355, EPI_ISL_724356, EPI_ISL_724357, EPI_ISL_724358, EPI_ISL_724359, EPI_ISL_724360, EPI_ISL_724361, EPI_ISL_724362, EPI_ISL_724363, EPI_ISL_724364, EPI_ISL_724365, EPI_ISL_724366, EPI_ISL_724367, EPI_ISL_724368, EPI_ISL_724369, EPI_ISL_724370, EPI_ISL_724372, EPI_ISL_724374, EPI_ISL_724375, EPI_ISL_724377, EPI_ISL_724378, EPI_ISL_724379, EPI_ISL_724380, EPI_ISL_724382, EPI_ISL_724383, EPI_ISL_724384, EPI_ISL_724386, EPI_ISL_724387, EPI_ISL_724388, EPI_ISL_724389, EPI_ISL_724390, EPI_ISL_724391, EPI_ISL_724394, EPI_ISL_724396, EPI_ISL_724399, EPI_ISL_724401, EPI_ISL_724404, EPI_ISL_724405, EPI_ISL_724406, EPI_ISL_724408, EPI_ISL_724409, EPI_ISL_724410, EPI_ISL_724411, EPI_ISL_724412, EPI_ISL_724413, EPI_ISL_724415, EPI_ISL_724417, EPI_ISL_724418, EPI_ISL_724419, EPI_ISL_724420, EPI_ISL_724421, EPI_ISL_724422, EPI_ISL_724423, EPI_ISL_724425, EPI_ISL_724427, EPI_ISL_724428, EPI_ISL_724429, EPI_ISL_724430, EPI_ISL_724431, EPI_ISL_724432, EPI_ISL_724433, EPI_ISL_724434, EPI_ISL_724435, EPI_ISL_724436, EPI_ISL_724437, EPI_ISL_724438, EPI_ISL_724439, EPI_ISL_724440, EPI_ISL_724441, EPI_ISL_724442, EPI_ISL_724443, EPI_ISL_724444, EPI_ISL_724445, EPI_ISL_724446, EPI_ISL_724447, EPI_ISL_724448, EPI_ISL_724449, EPI_ISL_724450, EPI_ISL_724451, EPI_ISL_724452, EPI_ISL_724453, EPI_ISL_724454, EPI_ISL_724455, EPI_ISL_724457, EPI_ISL_724458, EPI_ISL_724459, EPI_ISL_724460, EPI_ISL_724461, EPI_ISL_724462, EPI_ISL_724463, EPI_ISL_724464, EPI_ISL_724465, EPI_ISL_724466, EPI_ISL_724467, EPI_ISL_724468, EPI_ISL_724469, EPI_ISL_724470, EPI_ISL_724471, EPI_ISL_724472, EPI_ISL_724473, EPI_ISL_724474, EPI_ISL_724475, EPI_ISL_724477, EPI_ISL_724478, EPI_ISL_724479, EPI_ISL_724480, EPI_ISL_724481, EPI_ISL_724482, EPI_ISL_724483, EPI_ISL_724484, EPI_ISL_724485, EPI_ISL_724486, EPI_ISL_724487, EPI_ISL_724488, EPI_ISL_724489, EPI_ISL_724490, EPI_ISL_724491, EPI_ISL_724492, EPI_ISL_724493, EPI_ISL_724495, EPI_ISL_724496, EPI_ISL_724498, EPI_ISL_724499, EPI_ISL_724500, EPI_ISL_724501, EPI_ISL_724502, EPI_ISL_724503, EPI_ISL_724505, EPI_ISL_724506, EPI_ISL_724507, EPI_ISL_724509, EPI_ISL_724510, EPI_ISL_724511, EPI_ISL_724513, EPI_ISL_724514, EPI_ISL_724516, EPI_ISL_724517, EPI_ISL_724518, EPI_ISL_724519, EPI_ISL_724520, EPI_ISL_724521, EPI_ISL_724522, EPI_ISL_724523, EPI_ISL_724524, EPI_ISL_724525, EPI_ISL_724529, EPI_ISL_724530, EPI_ISL_724531, EPI_ISL_724534, EPI_ISL_724536, EPI_ISL_724537			
see above	Liverpool Clinical Laboratories	COVID-19 Genomics UK (COG-UK) Consortium	Sam Haldenby, Anita Lucaci, Steve Paterson, Julian Hiscox, Alistair Darby, M Almsaud, A Alrezaihi, Muhannad Alruwaili, Stuart D Armstrong, Jones Benjamin, Eleanor G Bentley, Anu Chawla, Jordan J Clark, Angela Cowell, Richard Eccles, Isabel García-Dorival, Matthew Gemmell, Alessandro Gerada, PKF Gilmore, Richard Gregory, Ximeng Han, Catherine Hartley, Margaret Hughes, Miren Iturriza-Gomara, James Johnson, L Luu, Jenifer Manson, Charlotte Nelson, Elaine O'Toole, Cassie Olateju, Rebekah Penrice-Randal , Lucille Rainbow, N.P Randle, Trevor Ian Robinson, Parul Sharma, Ghada T Shawli, James P Stewart, Neil Swainston, Ecaterina Vamos, Joanne Watts, Mark Whitehead
EPI_ISL_724541, EPI_ISL_724542, EPI_ISL_724544, EPI_ISL_724545, EPI_ISL_724546, EPI_ISL_724547, EPI_ISL_724548, EPI_ISL_724549, EPI_ISL_724550, EPI_ISL_724551, EPI_ISL_724552, EPI_ISL_724553, EPI_ISL_724555, EPI_ISL_724556, EPI_ISL_724557, EPI_ISL_724558, EPI_ISL_724560, EPI_ISL_724562, EPI_ISL_724563, EPI_ISL_724564, EPI_ISL_724565, EPI_ISL_724567, EPI_ISL_724568, EPI_ISL_724569, EPI_ISL_724570, EPI_ISL_724571, EPI_ISL_724573, EPI_ISL_724574, EPI_ISL_724576, EPI_ISL_724578, EPI_ISL_724580, EPI_ISL_724582, EPI_ISL_724584, EPI_ISL_724588, EPI_ISL_724589, EPI_ISL_724590, EPI_ISL_724592, EPI_ISL_724594, EPI_ISL_724595, EPI_ISL_724596, EPI_ISL_724598, EPI_ISL_724599, EPI_ISL_724600, EPI_ISL_724602, EPI_ISL_724603, EPI_ISL_724604, EPI_ISL_724610, EPI_ISL_724614, EPI_ISL_724616, EPI_ISL_724617, EPI_ISL_724618, EPI_ISL_724619, EPI_ISL_724621, EPI_ISL_724623, EPI_ISL_724624, EPI_ISL_724625, EPI_ISL_724626, EPI_ISL_724627, EPI_ISL_724629, EPI_ISL_724630, EPI_ISL_724631, EPI_ISL_724633, EPI_ISL_724636, EPI_ISL_724638, EPI_ISL_724639, EPI_ISL_724640, EPI_ISL_724641, EPI_ISL_724642, EPI_ISL_724643, EPI_ISL_724644, EPI_ISL_724645, EPI_ISL_724646, EPI_ISL_724647, EPI_ISL_724649, EPI_ISL_724650, EPI_ISL_724651, EPI_ISL_724652, EPI_ISL_724653, EPI_ISL_724654, EPI_ISL_724655, EPI_ISL_724656, EPI_ISL_724657, EPI_ISL_724658, EPI_ISL_724659, EPI_ISL_724660, EPI_ISL_724661, EPI_ISL_724662, EPI_ISL_724663, EPI_ISL_724664, EPI_ISL_724665, EPI_ISL_724666, EPI_ISL_724668, EPI_ISL_724670, EPI_ISL_724671, EPI_ISL_724673, EPI_ISL_724674, EPI_ISL_724675, EPI_ISL_724676, EPI_ISL_724677, EPI_ISL_724679, EPI_ISL_724681, EPI_ISL_724682, EPI_ISL_724683, EPI_ISL_724685, EPI_ISL_724686, EPI_ISL_724687, EPI_ISL_724688, EPI_ISL_724689, EPI_ISL_724690, EPI_ISL_724691, EPI_ISL_724693, EPI_ISL_724694, EPI_ISL_724695, EPI_ISL_724696, EPI_ISL_724697, EPI_ISL_724698, EPI_ISL_724699, EPI_ISL_724700, EPI_ISL_724701, EPI_ISL_724703, EPI_ISL_724704, EPI_ISL_724705, EPI_ISL_724708, EPI_ISL_724710, EPI_ISL_724711, EPI_ISL_724712, EPI_ISL_724713, EPI_ISL_724714, EPI_ISL_724715, EPI_ISL_724716, EPI_ISL_724718, EPI_ISL_724719, EPI_ISL_724721, EPI_ISL_724722, EPI_ISL_724723, EPI_ISL_724724, EPI_ISL_724725, EPI_ISL_724726, EPI_ISL_724727, EPI_ISL_724728, EPI_ISL_724729, EPI_ISL_724730, EPI_ISL_724731, EPI_ISL_724732, EPI_ISL_724733, EPI_ISL_724735, EPI_ISL_724736, EPI_ISL_724739, EPI_ISL_724740, EPI_ISL_724741, EPI_ISL_724742, EPI_ISL_724743, EPI_ISL_724744, EPI_ISL_724746, EPI_ISL_724747, EPI_ISL_724748, EPI_ISL_724749, EPI_ISL_724750, EPI_ISL_724751, EPI_ISL_724752, EPI_ISL_724753, EPI_ISL_724754, EPI_ISL_724755, EPI_ISL_724757, EPI_ISL_724758, EPI_ISL_724759, EPI_ISL_724760, EPI_ISL_724761, EPI_ISL_724762, EPI_ISL_724763, EPI_ISL_724764, EPI_ISL_724767, EPI_ISL_724768, EPI_ISL_724769, EPI_ISL_724771, EPI_ISL_724772, EPI_ISL_724774, EPI_ISL_724775, EPI_ISL_724776, EPI_ISL_724777			
see above	University College London, Great Ormond Street Hospital for Children NHS Foundation Trust, Imperial College Healthcare NHS Trust	COVID-19 Genomics UK (COG-UK) Consortium	Sergi Castellano, Rachel Williams, Mark Kristiansen, Paola Resende Silva, Sunando Roy, Tony Brooks, Helena Tutill, Paola Niola, Patricia Dyal, Charlotte Williams, Leysa Forrest, Yasmin Panchbhaya, Jacqueline Findlay, Samuel Weeks, Julianne Brown, Kathryn Harris, Paul Randell, James Price, Alison Holmes, Judith Breuer
EPI_ISL_724778, EPI_ISL_724779, EPI_ISL_724780, EPI_ISL_724781, EPI_ISL_724782, EPI_ISL_724783, EPI_ISL_724784, EPI_ISL_724785, EPI_ISL_724786, EPI_ISL_724787, EPI_ISL_724788, EPI_ISL_724789, EPI_ISL_724790, EPI_ISL_724791, EPI_ISL_724792, EPI_ISL_724793, EPI_ISL_724794, EPI_ISL_724795, EPI_ISL_724796, EPI_ISL_724797, EPI_ISL_724798, EPI_ISL_724799, EPI_ISL_724800, EPI_ISL_724801, EPI_ISL_724802, EPI_ISL_724803, EPI_ISL_724804, EPI_ISL_724805, EPI_ISL_724806, EPI_ISL_724807, EPI_ISL_724808, EPI_ISL_724809, EPI_ISL_724810, EPI_ISL_724811, EPI_ISL_724812, EPI_ISL_724813, EPI_ISL_724814, EPI_ISL_724815, EPI_ISL_724816, EPI_ISL_724817, EPI_ISL_724818, EPI_ISL_724819, EPI_ISL_724820, EPI_ISL_724821, EPI_ISL_724822, EPI_ISL_724823, EPI_ISL_724824, EPI_ISL_724825, EPI_ISL_724826, EPI_ISL_724827, EPI_ISL_724828, EPI_ISL_724829, EPI_ISL_724830, EPI_ISL_724831, EPI_ISL_724832, EPI_ISL_724833, EPI_ISL_724834, EPI_ISL_724835			
see above	Oxford Viromics, NDM, University of Oxford; Oxford University Hospitals; Basingstoke and North Hampshire Hospital	COVID-19 Genomics UK (COG-UK) Consortium	Tanya Golubchik, David Bonsall, George Macintyre, Amy Trebes, Mariateresa de Cesare, Catrin Moore, Alex Mobbs, Anita Justice, Robert Shaw, Monique Andersson, Timothy Peto, Emma Wise, Nathan Moore, Jessica Lynch, Nick Cortes, Matilde Mori, Stephen Kidd, David Buck, John Todd, Christophe Fraser
EPI_ISL_724842, EPI_ISL_724845, EPI_ISL_724848, EPI_ISL_724850, EPI_ISL_724852, EPI_ISL_724853, EPI_ISL_724854, EPI_ISL_724855, EPI_ISL_724856, EPI_ISL_724857, EPI_ISL_724858, EPI_ISL_724859, EPI_ISL_724860, EPI_ISL_724861, EPI_ISL_724862, EPI_ISL_724863, EPI_ISL_724864, EPI_ISL_724865, EPI_ISL_724866, EPI_ISL_724868, EPI_ISL_724869, EPI_ISL_724870, EPI_ISL_724872, EPI_ISL_724873, EPI_ISL_724874, EPI_ISL_724875, EPI_ISL_724876, EPI_ISL_724877, EPI_ISL_724878, EPI_ISL_724879, EPI_ISL_724880, EPI_ISL_724881, EPI_ISL_724882, EPI_ISL_724883, EPI_ISL_724884, EPI_ISL_724885, EPI_ISL_724886, EPI_ISL_724887, EPI_ISL_724888, EPI_ISL_724889, EPI_ISL_724890, EPI_ISL_724891, EPI_ISL_724892, EPI_ISL_724893, EPI_ISL_724894, EPI_ISL_724895, EPI_ISL_724896, EPI_ISL_724897, EPI_ISL_724898, EPI_ISL_724899, EPI_ISL_724900, EPI_ISL_724901, EPI_ISL_724902, EPI_ISL_724903, EPI_ISL_724904, EPI_ISL_724905, EPI_ISL_724906, EPI_ISL_724907, EPI_ISL_724908, EPI_ISL_724909, EPI_ISL_724910, EPI_ISL_724911, EPI_ISL_724912, EPI_ISL_724913, EPI_ISL_724914, EPI_ISL_724915, EPI_ISL_724916, EPI_ISL_724917, EPI_ISL_724918, EPI_ISL_724919, EPI_ISL_724921, EPI_ISL_724922, EPI_ISL_724923, EPI_ISL_724924, EPI_ISL_724925, EPI_ISL_724926, EPI_ISL_724927, EPI_ISL_724928, EPI_ISL_724929, EPI_ISL_724930, EPI_ISL_724931, EPI_ISL_724932, EPI_ISL_724934, EPI_ISL_724935, EPI_ISL_724936, EPI_ISL_724937, EPI_ISL_724938, EPI_ISL_724939, EPI_ISL_724940, EPI_ISL_724941, EPI_ISL_724942, EPI_ISL_724944, EPI_ISL_724945, EPI_ISL_724946, EPI_ISL_724947, EPI_ISL_724948, EPI_ISL_724949, EPI_ISL_724950, EPI_ISL_724951, EPI_ISL_724952, EPI_ISL_724953, EPI_ISL_724954, EPI_ISL_724955, EPI_ISL_724956, EPI_ISL_724957, EPI_ISL_724958, EPI_ISL_724959, EPI_ISL_724961, EPI_ISL_724963, EPI_ISL_724964, EPI_ISL_724967, EPI_ISL_724968, EPI_ISL_724969, EPI_ISL_724972, EPI_ISL_724973, EPI_ISL_724974, EPI_ISL_724976, EPI_ISL_724977, EPI_ISL_724978, EPI_ISL_724979, EPI_ISL_724980, EPI_ISL_724983, EPI_ISL_724984, EPI_ISL_724986, EPI_ISL_724987, EPI_ISL_724988, EPI_ISL_724989, EPI_ISL_724991, EPI_ISL_724992, EPI_ISL_724993, EPI_ISL_724994, EPI_ISL_724995, EPI_ISL_724996, EPI_ISL_724997, EPI_ISL_724998, EPI_ISL_724999, EPI_ISL_725001, EPI_ISL_725002, EPI_ISL_725003, EPI_ISL_725004, EPI_ISL_725005, EPI_ISL_725006, EPI_ISL_725007, EPI_ISL_725008, EPI_ISL_725009, EPI_ISL_725010, EPI_ISL_725011, EPI_ISL_725012, EPI_ISL_725013, EPI_ISL_725014, EPI_ISL_725015, EPI_ISL_725016, EPI_ISL_725017, EPI_ISL_725018, EPI_ISL_725019, EPI_ISL_725020, EPI_ISL_725021, EPI_ISL_725022, EPI_ISL_725023, EPI_ISL_725024, EPI_ISL_725025, EPI_ISL_725026, EPI_ISL_725027, EPI_ISL_725028			
see above	Northumbria University / South Tees Hospitals NHS Foundation Trust / North Cumbria Integrated Care NHS Foundation Trust / North Tees and Hartlepool NHS Foundation Trust / Newcastle Hospitals NHS Foundation Trust	COVID-19 Genomics UK (COG-UK) Consortium	Darren L Smith,Andrew Nelson,Matthew Bashton,Greg R Young,Joshua Loh,John Allan,Mohammad A Tariq,Giles S Holt,Gary Black,Wen C Yew,Lynn Dover,Paul Baker,Steve Liggett,Sarah Essex,Jane Greenaway,Debra Padgett,Clive Graham,Garren Scott,Edward Barton,Emma Swindells,Brendan Payne,Jennifer Collins,Yusri Taha,Gary Eltringham
EPI_ISL_725029, EPI_ISL_725030, EPI_ISL_725031, EPI_ISL_725032, EPI_ISL_725033, EPI_ISL_725034, EPI_ISL_725035, EPI_ISL_725036, EPI_ISL_725037, EPI_ISL_725038, EPI_ISL_725039, EPI_ISL_725040, EPI_ISL_725041, EPI_ISL_725042, EPI_ISL_725043, EPI_ISL_725044, EPI_ISL_725045, EPI_ISL_725046, EPI_ISL_725047, EPI_ISL_725048, EPI_ISL_725049, EPI_ISL_725050, EPI_ISL_725051, EPI_ISL_725052, EPI_ISL_725054, EPI_ISL_725055, EPI_ISL_725056, EPI_ISL_725057, EPI_ISL_725058, EPI_ISL_725059, EPI_ISL_725060, EPI_ISL_725061, EPI_ISL_725062, EPI_ISL_725063, EPI_ISL_725064, EPI_ISL_725066, EPI_ISL_725067, EPI_ISL_725068, EPI_ISL_725069, EPI_ISL_725070, EPI_ISL_725071, EPI_ISL_725072, EPI_ISL_725073, EPI_ISL_725074, EPI_ISL_725075, EPI_ISL_725076, EPI_ISL_725077, EPI_ISL_725078, EPI_ISL_725079, EPI_ISL_725080, EPI_ISL_725081, EPI_ISL_725082, EPI_ISL_725083, EPI_ISL_725084, EPI_ISL_725085, EPI_ISL_725086, EPI_ISL_725087, EPI_ISL_725088, EPI_ISL_725089, EPI_ISL_725090, EPI_ISL_725091, EPI_ISL_725092, EPI_ISL_725093, EPI_ISL_725094, EPI_ISL_725095, EPI_ISL_725096, EPI_ISL_725097, EPI_ISL_725098, EPI_ISL_725099, EPI_ISL_725101, EPI_ISL_725102, EPI_ISL_725103, EPI_ISL_725104, EPI_ISL_725105, EPI_ISL_725106, EPI_ISL_725107, EPI_ISL_725108, EPI_ISL_725109, EPI_ISL_725110, EPI_ISL_725111, EPI_ISL_725112, EPI_ISL_725113, EPI_ISL_725114, EPI_ISL_725115, EPI_ISL_725116, EPI_ISL_725117, EPI_ISL_725119, EPI_ISL_725120, EPI_ISL_725122, EPI_ISL_725123, EPI_ISL_725125, EPI_ISL_725126, EPI_ISL_725127, EPI_ISL_725128, EPI_ISL_725129, EPI_ISL_725130, EPI_ISL_725131, EPI_ISL_725132, EPI_ISL_725133, EPI_ISL_725134, EPI_ISL_725137, EPI_ISL_725138, EPI_ISL_725139, EPI_ISL_725140, EPI_ISL_725141, EPI_ISL_725142, EPI_ISL_725143, EPI_ISL_725144, EPI_ISL_725145, EPI_ISL_725146, EPI_ISL_725147, EPI_ISL_725148, EPI_ISL_725150, EPI_ISL_725151, EPI_ISL_725153, EPI_ISL_725154, EPI_ISL_725155, EPI_ISL_725156, EPI_ISL_725157, EPI_ISL_725158, EPI_ISL_725160, EPI_ISL_725161, EPI_ISL_725162, EPI_ISL_725163, EPI_ISL_725164, EPI_ISL_725166, EPI_ISL_725167, EPI_ISL_725168, EPI_ISL_725169, EPI_ISL_725170, EPI_ISL_725172, EPI_ISL_725174, EPI_ISL_725175, EPI_ISL_725176, EPI_ISL_725177, EPI_ISL_725178, EPI_ISL_725179, EPI_ISL_725180, EPI_ISL_725181, EPI_ISL_725182, EPI_ISL_725183, EPI_ISL_725184, EPI_ISL_725186, EPI_ISL_725187, EPI_ISL_725188, EPI_ISL_725189, EPI_ISL_725190, EPI_ISL_725191, EPI_ISL_725192, EPI_ISL_725193, EPI_ISL_725194, EPI_ISL_725195, EPI_ISL_725196, EPI_ISL_725197, EPI_ISL_725198, EPI_ISL_725199, EPI_ISL_725200, EPI_ISL_725202, EPI_ISL_725204, EPI_ISL_725205, EPI_ISL_725207, EPI_ISL_725208, EPI_ISL_725209, EPI_ISL_725210, EPI_ISL_725211, EPI_ISL_725212, EPI_ISL_725213, EPI_ISL_725214, EPI_ISL_725216, EPI_ISL_725217, EPI_ISL_725218, EPI_ISL_725219, EPI_ISL_725220, EPI_ISL_725221, EPI_ISL_725222, EPI_ISL_725223, EPI_ISL_725224, EPI_ISL_725226, EPI_ISL_725227, EPI_ISL_725228, EPI_ISL_725229, EPI_ISL_725230, EPI_ISL_725231, EPI_ISL_725232, EPI_ISL_725233, EPI_ISL_725236, EPI_ISL_725237, EPI_ISL_725238, EPI_ISL_725239, EPI_ISL_725240, EPI_ISL_725241, EPI_ISL_725243, EPI_ISL_725245, EPI_ISL_725246, EPI_ISL_725247, EPI_ISL_725248, EPI_ISL_725249, EPI_ISL_725250,			

EPI_ISL_725252, EPI_ISL_725253, EPI_ISL_725255, EPI_ISL_725256, EPI_ISL_725258, EPI_ISL_725259, EPI_ISL_725260, EPI_ISL_725261, EPI_ISL_725262, EPI_ISL_725263, EPI_ISL_725264, EPI_ISL_725265, EPI_ISL_725266, EPI_ISL_725267, EPI_ISL_725268, EPI_ISL_725269, EPI_ISL_725270, EPI_ISL_725271, EPI_ISL_725273, EPI_ISL_725274, EPI_ISL_725275, EPI_ISL_725276, EPI_ISL_725278, EPI_ISL_725279, EPI_ISL_725281, EPI_ISL_725282, EPI_ISL_725283, EPI_ISL_725285, EPI_ISL_725286, EPI_ISL_725287, EPI_ISL_725288, EPI_ISL_725289, EPI_ISL_725290, EPI_ISL_725291, EPI_ISL_725292, EPI_ISL_725293, EPI_ISL_725294, EPI_ISL_725295, EPI_ISL_725296, EPI_ISL_725297, EPI_ISL_725298, EPI_ISL_725299, EPI_ISL_725300, EPI_ISL_725301, EPI_ISL_725302, EPI_ISL_725303, EPI_ISL_725304, EPI_ISL_725305, EPI_ISL_725306, EPI_ISL_725307, EPI_ISL_725308, EPI_ISL_725309, EPI_ISL_725311, EPI_ISL_725312, EPI_ISL_725313, EPI_ISL_725314, EPI_ISL_725315, EPI_ISL_725316, EPI_ISL_725317, EPI_ISL_725318, EPI_ISL_725319, EPI_ISL_725320, EPI_ISL_725321, EPI_ISL_725322, EPI_ISL_725323, EPI_ISL_725324, EPI_ISL_725325, EPI_ISL_725326, EPI_ISL_725327, EPI_ISL_725328, EPI_ISL_725329, EPI_ISL_725330, EPI_ISL_725331, EPI_ISL_725332, EPI_ISL_725333, EPI_ISL_725334, EPI_ISL_725335, EPI_ISL_725336, EPI_ISL_725337, EPI_ISL_725338, EPI_ISL_725339, EPI_ISL_725340, EPI_ISL_725341, EPI_ISL_725342, EPI_ISL_725343, EPI_ISL_725344, EPI_ISL_725345, EPI_ISL_725346, EPI_ISL_725347, EPI_ISL_725348, EPI_ISL_725349, EPI_ISL_725350, EPI_ISL_725351, EPI_ISL_725352, EPI_ISL_725353, EPI_ISL_725354, EPI_ISL_725355, EPI_ISL_725356, EPI_ISL_725357, EPI_ISL_725358, EPI_ISL_725359, EPI_ISL_725360, EPI_ISL_725362, EPI_ISL_725363, EPI_ISL_725365, EPI_ISL_725366, EPI_ISL_725368, EPI_ISL_725370, EPI_ISL_725371, EPI_ISL_725373, EPI_ISL_725374, EPI_ISL_725375, EPI_ISL_725376, EPI_ISL_725377, EPI_ISL_725378, EPI_ISL_725379, EPI_ISL_725380, EPI_ISL_725381, EPI_ISL_725382, EPI_ISL_725383, EPI_ISL_725384, EPI_ISL_725385, EPI_ISL_725386, EPI_ISL_725387, EPI_ISL_725388, EPI_ISL_725389, EPI_ISL_725391, EPI_ISL_725392, EPI_ISL_725393, EPI_ISL_725394, EPI_ISL_725395, EPI_ISL_725396, EPI_ISL_725397, EPI_ISL_725398, EPI_ISL_725399, EPI_ISL_725400, EPI_ISL_725401, EPI_ISL_725402, EPI_ISL_725403, EPI_ISL_725404, EPI_ISL_725405, EPI_ISL_725407, EPI_ISL_725408, EPI_ISL_725409, EPI_ISL_725410, EPI_ISL_725411, EPI_ISL_725412, EPI_ISL_725413, EPI_ISL_725414, EPI_ISL_725415, EPI_ISL_725416, EPI_ISL_725417, EPI_ISL_725418, EPI_ISL_725420, EPI_ISL_725421, EPI_ISL_725422, EPI_ISL_725423, EPI_ISL_725424, EPI_ISL_725425, EPI_ISL_725426, EPI_ISL_725428, EPI_ISL_725429, EPI_ISL_725430, EPI_ISL_725431, EPI_ISL_725432, EPI_ISL_725433, EPI_ISL_725434, EPI_ISL_725435, EPI_ISL_725436, EPI_ISL_725437, EPI_ISL_725438, EPI_ISL_725439, EPI_ISL_725440, EPI_ISL_725441, EPI_ISL_725442, EPI_ISL_725443, EPI_ISL_725444, EPI_ISL_725445, EPI_ISL_725446, EPI_ISL_725447, EPI_ISL_725448, EPI_ISL_725449, EPI_ISL_725450, EPI_ISL_725451, EPI_ISL_725452, EPI_ISL_725453, EPI_ISL_725454, EPI_ISL_725455, EPI_ISL_725456, EPI_ISL_725457, EPI_ISL_725458, EPI_ISL_725459, EPI_ISL_725460, EPI_ISL_725461, EPI_ISL_725462, EPI_ISL_725463, EPI_ISL_725464, EPI_ISL_725465, EPI_ISL_725466, EPI_ISL_725467, EPI_ISL_725468, EPI_ISL_725469, EPI_ISL_725470, EPI_ISL_725471, EPI_ISL_725472, EPI_ISL_725473, EPI_ISL_725474, EPI_ISL_725475, EPI_ISL_725476, EPI_ISL_725477, EPI_ISL_725478, EPI_ISL_725479, EPI_ISL_725481, EPI_ISL_725482, EPI_ISL_725483, EPI_ISL_725484, EPI_ISL_725485, EPI_ISL_725486, EPI_ISL_725487, EPI_ISL_725489, EPI_ISL_725490, EPI_ISL_725491, EPI_ISL_725492, EPI_ISL_725493, EPI_ISL_725494, EPI_ISL_725495, EPI_ISL_725496, EPI_ISL_725497, EPI_ISL_725498, EPI_ISL_725499, EPI_ISL_725500, EPI_ISL_725501, EPI_ISL_725502, EPI_ISL_725505, EPI_ISL_725506, EPI_ISL_725507, EPI_ISL_725508, EPI_ISL_725509, EPI_ISL_725510, EPI_ISL_725511, EPI_ISL_725513, EPI_ISL_725514, EPI_ISL_725515, EPI_ISL_725516, EPI_ISL_725518

see above	Quadram Institute Bioscience	COVID-19 Genomics UK (COG-UK) Consortium	Dave J. Baker, Gemma L. Kay, Alp Aydin, Thanh Le-Viet, Steven Rudder, Ana P. Tedim, Anastasia Kolyva, Maria Diaz, Leonardo de Oliveira Martins, Nabil-Fareed Alikhan, Lizzie Meadows, Rachael Stanley, Ngozi Elumogo, Muhammed Yasir, Nicholas M. Thomson, Alexander J Trotter, Rachel Gilroy, Samuel Bloomfield, Claire Stuart, Andrew Bell, Reenesh Prakash, Samir Dervisevic, Alison E. Mather, John Wain, Mark Webber, Andrew J. Page, Justin O'Grady

EPI_ISL_725519, EPI_ISL_725520, EPI_ISL_725521, EPI_ISL_725522, EPI_ISL_725523, EPI_ISL_725524, EPI_ISL_725525, EPI_ISL_725527, EPI_ISL_725528, EPI_ISL_725529, EPI_ISL_725530, EPI_ISL_725531, EPI_ISL_725533, EPI_ISL_725534, EPI_ISL_725535, EPI_ISL_725536, EPI_ISL_725537, EPI_ISL_725538

see above	Queens Medical Centre, Clinical Microbiology Department / DeepSeq Nottingham	COVID-19 Genomics UK (COG-UK) Consortium	Gemma Clark, Wendy Smith, Manjinder Khakh, Vicki M Fleming, Michelle M Lister, Hannah Howson-Wells, Jonathan Ball, Patrick McClure, Joseph Chappell, Theocharis Tsoleridis, Nadine Holmes, Matthew Carlisle, Christopher Moore, Fei Sang, Johnny Debebe, Victoria Wright, Matthew Loose

EPI_ISL_725540, EPI_ISL_725541, EPI_ISL_725542, EPI_ISL_725543, EPI_ISL_725544, EPI_ISL_725545, EPI_ISL_725546, EPI_ISL_725547, EPI_ISL_725548, EPI_ISL_725549, EPI_ISL_725550, EPI_ISL_725551, EPI_ISL_725552, EPI_ISL_725553, EPI_ISL_725554, EPI_ISL_725555, EPI_ISL_725556, EPI_ISL_725557, EPI_ISL_725558, EPI_ISL_725559, EPI_ISL_725560, EPI_ISL_725561, EPI_ISL_725562, EPI_ISL_725563, EPI_ISL_725564, EPI_ISL_725566, EPI_ISL_725567, EPI_ISL_725568, EPI_ISL_725569, EPI_ISL_725570, EPI_ISL_725571, EPI_ISL_725572, EPI_ISL_725573, EPI_ISL_725574, EPI_ISL_725575, EPI_ISL_725577, EPI_ISL_725578, EPI_ISL_725579, EPI_ISL_725580, EPI_ISL_725581, EPI_ISL_725582, EPI_ISL_725583, EPI_ISL_725584, EPI_ISL_725585, EPI_ISL_725586, EPI_ISL_725587, EPI_ISL_725588, EPI_ISL_725589, EPI_ISL_725590, EPI_ISL_725591, EPI_ISL_725592, EPI_ISL_725593, EPI_ISL_725594, EPI_ISL_725595, EPI_ISL_725596, EPI_ISL_725597, EPI_ISL_725598, EPI_ISL_725599, EPI_ISL_725600, EPI_ISL_725602, EPI_ISL_725603, EPI_ISL_725604

see above	Lincolnshire Hospitals and DeepSeq Nottingham	COVID-19 Genomics UK (COG-UK) Consortium	Nichola Duckworth, Tim Sloan, Sarah Walsh, Jonathan Ball, Patrick McClure, Joeseph Chappell, Nadine Holmes, Matthew Carlisle, Christopher Moore, Fei Sang, Johnny Debebe, Victoria Wright, Matthew Loose

EPI_ISL_725605, EPI_ISL_725606, EPI_ISL_725607, EPI_ISL_725608, EPI_ISL_725609, EPI_ISL_725610, EPI_ISL_725612, EPI_ISL_725613, EPI_ISL_725614, EPI_ISL_725615, EPI_ISL_725616, EPI_ISL_725617, EPI_ISL_725619, EPI_ISL_725620, EPI_ISL_725622, EPI_ISL_725623, EPI_ISL_725624, EPI_ISL_725625, EPI_ISL_725626, EPI_ISL_725627, EPI_ISL_725628, EPI_ISL_725629, EPI_ISL_725630, EPI_ISL_725631, EPI_ISL_725632, EPI_ISL_725633, EPI_ISL_725634, EPI_ISL_725635, EPI_ISL_725636, EPI_ISL_725637, EPI_ISL_725638, EPI_ISL_725639, EPI_ISL_725641, EPI_ISL_725642, EPI_ISL_725643, EPI_ISL_725644, EPI_ISL_725645, EPI_ISL_725646, EPI_ISL_725647, EPI_ISL_725648, EPI_ISL_725649, EPI_ISL_725650, EPI_ISL_725652, EPI_ISL_725653, EPI_ISL_725654, EPI_ISL_725656, EPI_ISL_725658, EPI_ISL_725659, EPI_ISL_725660, EPI_ISL_725667, EPI_ISL_725669

see above	Queens Medical Centre, Clinical Microbiology Department / DeepSeq Nottingham	COVID-19 Genomics UK (COG-UK) Consortium	Gemma Clark, Wendy Smith, Manjinder Khakh, Vicki M Fleming, Michelle M Lister, Hannah Howson-Wells, Jonathan Ball, Patrick McClure, Joseph Chappell, Theocharis Tsoleridis, Nadine Holmes, Matthew Carlisle, Christopher Moore, Fei Sang, Johnny Debebe, Victoria Wright, Matthew Loose

EPI_ISL_725670, EPI_ISL_725671, EPI_ISL_725672, EPI_ISL_725673, EPI_ISL_725674, EPI_ISL_725675, EPI_ISL_725676, EPI_ISL_725677, EPI_ISL_725678, EPI_ISL_725679, EPI_ISL_725680, EPI_ISL_725681, EPI_ISL_725682, EPI_ISL_725683, EPI_ISL_725684, EPI_ISL_725685, EPI_ISL_725686, EPI_ISL_725687, EPI_ISL_725688, EPI_ISL_725690, EPI_ISL_725691, EPI_ISL_725692, EPI_ISL_725693, EPI_ISL_725694, EPI_ISL_725695, EPI_ISL_725696, EPI_ISL_725697, EPI_ISL_725698, EPI_ISL_725699, EPI_ISL_725700, EPI_ISL_725701, EPI_ISL_725702, EPI_ISL_725703, EPI_ISL_725704, EPI_ISL_725705, EPI_ISL_725706, EPI_ISL_725707, EPI_ISL_725708, EPI_ISL_725709, EPI_ISL_725710, EPI_ISL_725711, EPI_ISL_725712, EPI_ISL_725713, EPI_ISL_725714, EPI_ISL_725715, EPI_ISL_725716, EPI_ISL_725717, EPI_ISL_725718, EPI_ISL_725719, EPI_ISL_725720, EPI_ISL_725721, EPI_ISL_725722, EPI_ISL_725723, EPI_ISL_725724, EPI_ISL_725725, EPI_ISL_725726, EPI_ISL_725727, EPI_ISL_725728, EPI_ISL_725729, EPI_ISL_725730, EPI_ISL_725731, EPI_ISL_725732, EPI_ISL_725733, EPI_ISL_725734, EPI_ISL_725735, EPI_ISL_725736, EPI_ISL_725737, EPI_ISL_725738, EPI_ISL_725739, EPI_ISL_725740, EPI_ISL_725741, EPI_ISL_725742, EPI_ISL_725743, EPI_ISL_725744, EPI_ISL_725745, EPI_ISL_725746, EPI_ISL_725747, EPI_ISL_725748, EPI_ISL_725749, EPI_ISL_725750, EPI_ISL_725751, EPI_ISL_725752, EPI_ISL_725753, EPI_ISL_725756, EPI_ISL_725757, EPI_ISL_725758, EPI_ISL_725759, EPI_ISL_725760, EPI_ISL_725761, EPI_ISL_725762, EPI_ISL_725763, EPI_ISL_725764, EPI_ISL_725765, EPI_ISL_725766, EPI_ISL_725767, EPI_ISL_725768, EPI_ISL_725769, EPI_ISL_725770, EPI_ISL_725771, EPI_ISL_725772, EPI_ISL_725773, EPI_ISL_725774, EPI_ISL_725775, EPI_ISL_725776, EPI_ISL_725777, EPI_ISL_725778, EPI_ISL_725779, EPI_ISL_725780, EPI_ISL_725781, EPI_ISL_725782, EPI_ISL_725783, EPI_ISL_725784, EPI_ISL_725785, EPI_ISL_725786, EPI_ISL_725787, EPI_ISL_725788, EPI_ISL_725789, EPI_ISL_725790, EPI_ISL_725791, EPI_ISL_725792, EPI_ISL_725793, EPI_ISL_725794, EPI_ISL_725795, EPI_ISL_725796, EPI_ISL_725797, EPI_ISL_725798, EPI_ISL_725799, EPI_ISL_725800, EPI_ISL_725801, EPI_ISL_725802, EPI_ISL_725803, EPI_ISL_725804, EPI_ISL_725805, EPI_ISL_725806, EPI_ISL_725807, EPI_ISL_725808, EPI_ISL_725809, EPI_ISL_725810, EPI_ISL_725811, EPI_ISL_725812, EPI_ISL_725813, EPI_ISL_725814, EPI_ISL_725815, EPI_ISL_725816, EPI_ISL_725817, EPI_ISL_725818, EPI_ISL_725819, EPI_ISL_725820, EPI_ISL_725821, EPI_ISL_725822, EPI_ISL_725823, EPI_ISL_725824, EPI_ISL_725825, EPI_ISL_725826, EPI_ISL_725827, EPI_ISL_725828, EPI_ISL_725829, EPI_ISL_725830, EPI_ISL_725831, EPI_ISL_725832, EPI_ISL_725833, EPI_ISL_725834, EPI_ISL_725835, EPI_ISL_725836, EPI_ISL_725837, EPI_ISL_725838, EPI_ISL_725839, EPI_ISL_725840, EPI_ISL_725841, EPI_ISL_725842, EPI_ISL_725843, EPI_ISL_725844, EPI_ISL_725845, EPI_ISL_725846, EPI_ISL_725847, EPI_ISL_725848, EPI_ISL_725849, EPI_ISL_725850, EPI_ISL_725851, EPI_ISL_725852, EPI_ISL_725853, EPI_ISL_725854, EPI_ISL_725855, EPI_ISL_725856, EPI_ISL_725857, EPI_ISL_725858, EPI_ISL_725859, EPI_ISL_725860, EPI_ISL_725861, EPI_ISL_725862, EPI_ISL_725863, EPI_ISL_725864, EPI_ISL_725865, EPI_ISL_725866, EPI_ISL_725867, EPI_ISL_725868, EPI_ISL_725869, EPI_ISL_725870, EPI_ISL_725871, EPI_ISL_725872, EPI_ISL_725873, EPI_ISL_725874, EPI_ISL_725875, EPI_ISL_725876, EPI_ISL_725877, EPI_ISL_725878, EPI_ISL_725879, EPI_ISL_725880, EPI_ISL_725881, EPI_ISL_725882, EPI_ISL_725883, EPI_ISL_725884, EPI_ISL_725885, EPI_ISL_725886, EPI_ISL_725887, EPI_ISL_725888, EPI_ISL_725889, EPI_ISL_725890, EPI_ISL_725891, EPI_ISL_725892, EPI_ISL_725893, EPI_ISL_725894, EPI_ISL_725895, EPI_ISL_725896, EPI_ISL_725897, EPI_ISL_725898, EPI_ISL_725899, EPI_ISL_725900, EPI_ISL_725901, EPI_ISL_725902, EPI_ISL_725903, EPI_ISL_725904, EPI_ISL_725906, EPI_ISL_725907, EPI_ISL_725908, EPI_ISL_725909, EPI_ISL_725910, EPI_ISL_725911, EPI_ISL_725912, EPI_ISL_725913, EPI_ISL_725914, EPI_ISL_725915, EPI_ISL_725916, EPI_ISL_725917, EPI_ISL_725918, EPI_ISL_725919, EPI_ISL_725920, EPI_ISL_725921, EPI_ISL_725922, EPI_ISL_725923, EPI_ISL_725924, EPI_ISL_725925, EPI_ISL_725926, EPI_ISL_725927, EPI_ISL_725928, EPI_ISL_725929, EPI_ISL_725930, EPI_ISL_725931, EPI_ISL_725932, EPI_ISL_725933, EPI_ISL_725934, EPI_ISL_725935, EPI_ISL_725936, EPI_ISL_725937, EPI_ISL_725938, EPI_ISL_725939, EPI_ISL_725940, EPI_ISL_725941, EPI_ISL_725942, EPI_ISL_725943, EPI_ISL_725944, EPI_ISL_725945, EPI_ISL_725946, EPI_ISL_725947, EPI_ISL_725948, EPI_ISL_725949, EPI_ISL_725950, EPI_ISL_725951, EPI_ISL_725952, EPI_ISL_725953, EPI_ISL_725954, EPI_ISL_725955, EPI_ISL_725956, EPI_ISL_725957, EPI_ISL_725958, EPI_ISL_725959, EPI_ISL_725960, EPI_ISL_725961, EPI_ISL_725962, EPI_ISL_725963, EPI_ISL_725964, EPI_ISL_725965, EPI_ISL_725966, EPI_ISL_725967, EPI_ISL_725968, EPI_ISL_725969, EPI_ISL_725970, EPI_ISL_725971, EPI_ISL_725972, EPI_ISL_725973, EPI_ISL_725974, EPI_ISL_725975, EPI_ISL_725976, EPI_ISL_725977, EPI_ISL_725978, EPI_ISL_725979, EPI_ISL_725980, EPI_ISL_725981, EPI_ISL_725982, EPI_ISL_725983, EPI_ISL_725984, EPI_ISL_725985, EPI_ISL_725986, EPI_ISL_725987, EPI_ISL_725988, EPI_ISL_725989, EPI_ISL_725990, EPI_ISL_725991, EPI_ISL_725992, EPI_ISL_725993, EPI_ISL_725994, EPI_ISL_725995, EPI_ISL_725996, EPI_ISL_725997, EPI_ISL_725998, EPI_ISL_725999, EPI_ISL_726000, EPI_ISL_726001, EPI_ISL_726002, EPI_ISL_726003, EPI_ISL_726004, EPI_ISL_726005, EPI_ISL_726006, EPI_ISL_726007, EPI_ISL_726008, EPI_ISL_726009, EPI_ISL_726010, EPI_ISL_726011, EPI_ISL_726012, EPI_ISL_726013, EPI_ISL_726014, EPI_ISL_726015, EPI_ISL_726016, EPI_ISL_726017, EPI_ISL_726018, EPI_ISL_726019, EPI_ISL_726020, EPI_ISL_726021, EPI_ISL_726022, EPI_ISL_726023, EPI_ISL_726024, EPI_ISL_726025, EPI_ISL_726026, EPI_ISL_726027, EPI_ISL_726028, EPI_ISL_726029, EPI_ISL_726030, EPI_ISL_726031, EPI_ISL_726032, EPI_ISL_726033, EPI_ISL_726034, EPI_ISL_726036, EPI_ISL_726037, EPI_ISL_726038, EPI_ISL_726039, EPI_ISL_726040, EPI_ISL_726041, EPI_ISL_726042, EPI_ISL_726043, EPI_ISL_726044, EPI_ISL_726045, EPI_ISL_726046, EPI_ISL_726047, EPI_ISL_726048, EPI_ISL_726049, EPI_ISL_726050, EPI_ISL_726051, EPI_ISL_726052, EPI_ISL_726053, EPI_ISL_726054, EPI_ISL_726055, EPI_ISL_726056, EPI_ISL_726057, EPI_ISL_726058, EPI_ISL_726059, EPI_ISL_726060, EPI_ISL_726061, EPI_ISL_726062, EPI_ISL_726063, EPI_ISL_726064, EPI_ISL_726066, EPI_ISL_726067, EPI_ISL_726068, EPI_ISL_726069, EPI_ISL_726070, EPI_ISL_726071, EPI_ISL_726072, EPI_ISL_726073, EPI_ISL_726074, EPI_ISL_726075, EPI_ISL_726076, EPI_ISL_726077, EPI_ISL_726078, EPI_ISL_726079, EPI_ISL_726080, EPI_ISL_726081, EPI_ISL_726082, EPI_ISL_726083, EPI_ISL_726084, EPI_ISL_726085, EPI_ISL_726086, EPI_ISL_726087, EPI_ISL_726088, EPI_ISL_726089, EPI_ISL_726090, EPI_ISL_726091, EPI_ISL_726092, EPI_ISL_726093, EPI_ISL_726094, EPI_ISL_726095, EPI_ISL_726096, EPI_ISL_726097, EPI_ISL_726098, EPI_ISL_726099, EPI_ISL_726100, EPI_ISL_726101, EPI_ISL_726102, EPI_ISL_726103, EPI_ISL_726104, EPI_ISL_726105, EPI_ISL_726106, EPI_ISL_726107, EPI_ISL_726108, EPI_ISL_726109, EPI_ISL_726110, EPI_ISL_726111, EPI_ISL_726112, EPI_ISL_726113, EPI_ISL_726114, EPI_ISL_726115, EPI_ISL_726116, EPI_ISL_726117, EPI_ISL_726118, EPI_ISL_726119, EPI_ISL_726120, EPI_ISL_726121, EPI_ISL_726122, EPI_ISL_726123, EPI_ISL_726124, EPI_ISL_726125, EPI_ISL_726126, EPI_ISL_726127, EPI_ISL_726128, EPI_ISL_726129, EPI_ISL_726130, EPI_ISL_726131, EPI_ISL_726132, EPI_ISL_726133, EPI_ISL_726134, EPI_ISL_726135, EPI_ISL_726136, EPI_ISL_726137, EPI_ISL_726138, EPI_ISL_726139, EPI_ISL_726140, EPI_ISL_726141, EPI_ISL_726142, EPI_ISL_726143, EPI_ISL_726144, EPI_ISL_726145, EPI_ISL_726146, EPI_ISL_726147, EPI_ISL_726148, EPI_ISL_726149, EPI_ISL_726150, EPI_ISL_726151, EPI_ISL_726152, EPI_ISL_726153, EPI_ISL_726154, EPI_ISL_726155, EPI_ISL_726156, EPI_ISL_726157, EPI_ISL_726158, EPI_ISL_726159, EPI_ISL_726160, EPI_ISL_726161, EPI_ISL_726162, EPI_ISL_726163, EPI_ISL_726164, EPI_ISL_726165, EPI_ISL_726166, EPI_ISL_726167, EPI_ISL_726168, EPI_ISL_726169, EPI_ISL_726170, EPI_ISL_726171, EPI_ISL_726172, EPI_ISL_726173, EPI_ISL_726174, EPI_ISL_726175, EPI_ISL_726176, EPI_ISL_726177, EPI_ISL_726178, EPI_ISL_726179, EPI_ISL_726180, EPI_ISL_726181, EPI_ISL_726182, EPI_ISL_726183, EPI_ISL_726185, EPI_ISL_726186, EPI_ISL_726187, EPI_ISL_726188, EPI_ISL_726189, EPI_ISL_726190, EPI_ISL_726191, EPI_ISL_726192, EPI_ISL_726193, EPI_ISL_726194, EPI_ISL_726195, EPI_ISL_726196, EPI_ISL_726197, EPI_ISL_726198, EPI_ISL_726199, EPI_ISL_726200, EPI_ISL_726201, EPI_ISL_726202, EPI_ISL_726203, EPI_ISL_726204, EPI_ISL_726205, EPI_ISL_726206, EPI_ISL_726207, EPI_ISL_726208, EPI_ISL_726209, EPI_ISL_726210, EPI_ISL_726211, EPI_ISL_726212, EPI_ISL_726213, EPI_ISL_726214, EPI_ISL_726215, EPI_ISL_726216, EPI_ISL_726217, EPI_ISL_726218, EPI_ISL_726219, EPI_ISL_726220, EPI_ISL_726221, EPI_ISL_726222, EPI_ISL_726223, EPI_ISL_726224, EPI_ISL_726225, EPI_ISL_726226, EPI_ISL_726227, EPI_ISL_726228, EPI_ISL_726229, EPI_ISL_726230, EPI_ISL_726231, EPI_ISL_726232, EPI_ISL_726233, EPI_ISL_726234, EPI_ISL_726235, EPI_ISL_726236, EPI_ISL_726237, EPI_ISL_726238, EPI_ISL_726239, EPI_ISL_726240, EPI_ISL_726241, EPI_ISL_726242, EPI_ISL_726243, EPI_ISL_726244, EPI_ISL_726245, EPI_ISL_726246, EPI_ISL_726247, EPI_ISL_726248, EPI_ISL_726249, EPI_ISL_726250, EPI_ISL_726251, EPI_ISL_726252, EPI_ISL_726253, EPI_ISL_726254, EPI_ISL_726255, EPI_ISL_726256, EPI_ISL_726257, EPI_ISL_726258, EPI_ISL_726259, EPI_ISL_726260, EPI_ISL_726261, EPI_ISL_726262, EPI_ISL_726263, EPI_ISL_726264, EPI_ISL_726265, EPI_ISL_726266, EPI_ISL_726267, EPI_ISL_726268, EPI_ISL_726269, EPI_ISL_726270, EPI_ISL_726271, EPI_ISL_726272, EPI_ISL_726273, EPI_ISL_726274, EPI_ISL_726275, EPI_ISL_726276, EPI_ISL_726278, EPI_ISL_726280, EPI_ISL_726281, EPI_ISL_726282, EPI_ISL_726283, EPI_ISL_726284, EPI_ISL_726285, EPI_ISL_726286, EPI_ISL_726287, EPI_ISL_726288, EPI_ISL_726289, EPI_ISL_726290, EPI_ISL_726291, EPI_ISL_726292, EPI_ISL_726293, EPI_ISL_726294, EPI_ISL_726295, EPI_ISL_726296, EPI_ISL_726297, EPI_ISL_726298, EPI_ISL_726299, EPI_ISL_726300, EPI_ISL_726301, EPI_ISL_726302, EPI_ISL_726303, EPI_ISL_726304, EPI_ISL_726305, EPI_ISL_726306, EPI_ISL_726307, EPI_ISL_726308, EPI_ISL_726309, EPI_ISL_726310, EPI_ISL_726311, EPI_ISL_726312, EPI_ISL_726313, EPI_ISL_726314, EPI_ISL_726315, EPI_ISL_726316, EPI_ISL_726317, EPI_ISL_726318, EPI_ISL_726319, EPI_ISL_726320, EPI_ISL_726321, EPI_ISL_726322, EPI_ISL_726323, EPI_ISL_726324, EPI_ISL_726325, EPI_ISL_726326, EPI_ISL_726327, EPI_ISL_726328, EPI_ISL_726329, EPI_ISL_726330, EPI_ISL_726331, EPI_ISL_726332, EPI_ISL_726333, EPI_ISL_726334, EPI_ISL_726335, EPI_ISL_726336, EPI_ISL_726337, EPI_ISL_726338, EPI_ISL_726339, EPI_ISL_726340, EPI_ISL_726341, EPI_ISL_726342, EPI_ISL_726343, EPI_ISL_726344, EPI_ISL_726345, EPI_ISL_726346, EPI_ISL_726347, EPI_ISL_726348, EPI_ISL_726349, EPI_ISL_726350, EPI_ISL_726351, EPI_ISL_726352, EPI_ISL_726353, EPI_ISL_726354, EPI_ISL_726355, EPI_ISL_726356, EPI_ISL_726357, EPI_ISL_726358, EPI_ISL_726359, EPI_ISL_726360, EPI_ISL_726361, EPI_ISL_726363, EPI_ISL_726365, EPI_ISL_726366, EPI_ISL_726367, EPI_ISL_726368, EPI_ISL_726369, EPI_ISL_726370, EPI_ISL_726371, EPI_ISL_726372, EPI_ISL_726373, EPI_ISL_726374, EPI_ISL_726375, EPI_ISL_726376, EPI_ISL_726377, EPI_ISL_726378, EPI_ISL_726379, EPI_ISL_726380, EPI_ISL_726381, EPI_ISL_726382, EPI_ISL_726384, EPI_ISL_726385, EPI_ISL_726386, EPI_ISL_726387, EPI_ISL_726388, EPI_ISL_726389, EPI_ISL_726390, EPI_ISL_726391, EPI_ISL_726392, EPI_ISL_726393, EPI_ISL_726394, EPI_ISL_726395, EPI_ISL_726396, EPI_ISL_726397, EPI_ISL_726398, EPI_ISL_726399, EPI_ISL_726400, EPI_ISL_726401, EPI_ISL_726402, EPI_ISL_726403, EPI_ISL_726404, EPI_ISL_726405, EPI_ISL_726406, EPI_ISL_726407, EPI_ISL_726408, EPI_ISL_726409, EPI_ISL_726410, EPI_ISL_726411, EPI_ISL_726412, EPI_ISL_726413, EPI_ISL_726414, EPI_ISL_726415, EPI_ISL_726416, EPI_ISL_726417, EPI_ISL_726418, EPI_ISL_726419, EPI_ISL_726420, EPI_ISL_726421, EPI_ISL_726422, EPI_ISL_726423, EPI_ISL_726424, EPI_ISL_726425, EPI_ISL_726426, EPI_ISL_726427, EPI_ISL_726428, EPI_ISL_726429, EPI_ISL_726430, EPI_ISL_726433, EPI_ISL_726434, EPI_ISL_726435, EPI_ISL_726436, EPI_ISL_726437, EPI_ISL_726438, EPI_ISL_726439, EPI_ISL_726440, EPI_ISL_726441, EPI_ISL_726442, EPI_ISL_726443, EPI_ISL_726444, EPI_ISL_726445, EPI_ISL_726446, EPI_ISL_726447, EPI_ISL_726448, EPI_ISL_726449, EPI_ISL_726450, EPI_ISL_726451, EPI_ISL_726452, EPI_ISL_726453, EPI_ISL_726454, EPI_ISL_726455, EPI_ISL_726456, EPI_ISL_726457, EPI_ISL_726458, EPI_ISL_726459, EPI_ISL_726460, EPI_ISL_726461, EPI_ISL_726462, EPI_ISL_726463, EPI_ISL_726464, EPI_ISL_726465, EPI_ISL_726466, EPI_ISL_726467, EPI_ISL_726468, EPI_ISL_726469, EPI_ISL_726470, EPI_ISL_726471, EPI_ISL_726472, EPI_ISL_726473, EPI_ISL_726474, EPI_ISL_726475, EPI_ISL_726476, EPI_ISL_726477, EPI_ISL_726478, EPI_ISL_726479, EPI_ISL_726480, EPI_ISL_726481, EPI_ISL_726482, EPI_ISL_726483, EPI_ISL_726484, EPI_ISL_726485, EPI_ISL_726486, EPI_ISL_726487, EPI_ISL_726488, EPI_ISL_726489, EPI_ISL_726490, EPI_ISL_726491, EPI_ISL_726492, EPI_ISL_726493, EPI_ISL_726494, EPI_ISL_726495, EPI_ISL_726496, EPI_ISL_726497, EPI_ISL_726498, EPI_ISL_726499, EPI_ISL_726500, EPI_ISL_726501, EPI_ISL_726502, EPI_ISL_726503, EPI_ISL_726504, EPI_ISL_726505, EPI_ISL_726506, EPI_ISL_726507, EPI_ISL_726508, EPI_ISL_726509, EPI_ISL_726510, EPI_ISL_726512, EPI_ISL_726513, EPI_ISL_726514, EPI_ISL_726515, EPI_ISL_726516, EPI_ISL_726517, EPI_ISL_726518, EPI_ISL_726519, EPI_ISL_726520, EPI_ISL_726522, EPI_ISL_726523, EPI_ISL_726524, EPI_ISL_726525, EPI_ISL_726526, EPI_ISL_726527, EPI_ISL_726528, EPI_ISL_726529, EPI_ISL_726530, EPI_ISL_726531, EPI_ISL_726532, EPI_ISL_726533, EPI_ISL_726534, EPI_ISL_726535, EPI_ISL_726536, EPI_ISL_726537, EPI_ISL_726538, EPI_ISL_726539, EPI_ISL_726540, EPI_ISL_726541, EPI_ISL_726542, EPI_ISL_726543, EPI_ISL_726544, EPI_ISL_726545, EPI_ISL_726546, EPI_ISL_726547, EPI_ISL_726548, EPI_ISL_726549, EPI_ISL_726550, EPI_ISL_726551, EPI_ISL_726552, EPI_ISL_726553, EPI_ISL_726554, EPI_ISL_726555, EPI_ISL_726556, EPI_ISL_726557, EPI_ISL_726558, EPI_ISL_726560, EPI_ISL_726561, EPI_ISL_726562, EPI_ISL_726563, EPI_ISL_726564, EPI_ISL_726565, EPI_ISL_726566, EPI_ISL_726567, EPI_ISL_726568, EPI_ISL_726569, EPI_ISL_726570, EPI_ISL_726571, EPI_ISL_726572, EPI_ISL_726573, EPI_ISL_726574, EPI_ISL_726575, EPI_ISL_726576, EPI_ISL_726577, EPI_ISL_726578, EPI_ISL_726579, EPI_ISL_726580, EPI_ISL_726581, EPI_ISL_726582, EPI_ISL_726583, EPI_ISL_726584, EPI_ISL_726585, EPI_ISL_726586, EPI_ISL_726587, EPI_ISL_726588, EPI_ISL_726589, EPI_ISL_726590, EPI_ISL_726591, EPI_ISL_726592

see above	Wales Specialist Virology Centre Sequencing lab: Pathogen Genomics Unit	COVID-19 Genomics UK (COG-UK) Consortium	Catherine Moore, Johnathan Evans, Laura Gifford, Malorie Perry, Simon Cottrell, Angela Marchbank, Alec Birchley, Alexander Adams, Amy Gaskin, Bree Gatica-Wilcox, Jason Coombes, Joel Southgate, Lauren Gilbert, Lee Graham, Nicole Pacchiarini, Sara Kumziene-Summerhayes, Sarah Taylor, Sophie Jones, Sara Rey, Matthew Bull, Joanne Watkins, Sally Corden, Tom Connor
