# Supplementary material for: Deepening of In Silico Evaluation of SARS-CoV-2 Detection RT-qPCR Assays in the Context of New Variants
Source: Genes (Basel). 2021 Apr 13;12(4):565. doi: 10.3390/genes12040565 (PMC8069896; doi:10.3390/genes12040565)
Supplement: Supplementary file 1 [file genes-12-00565-s001.zip › Supplementary file S9.zip!/Acknowledgement tables/gisaid_hcov-19_acknowledgement_table_18.pdf]

We gratefully acknowledge the following Authors from the Originating laboratories responsible for obtaining the specimens, as well as the Submitting laboratories where the genome data were generated and shared via GISAID, on which this research is based.

All Submitters of data may be contacted directly via www.gisaid.org

Authors are sorted alphabetically.

Accession ID	Originating Laboratory	Submitting Laboratory	Authors
EPI_ISL_737603, EPI_ISL_737604, EPI_ISL_737605, EPI_ISL_737606	Viollier AG	Department of Biosystems Science and Engineering, ETH Zürich	Chaoran Chen, Sarah Nadeau, Catharine Aquino, Ivan Topolsky, Philipp Jablonski, Lara Fuhrmann, David Dreifuss, Katharina Jahn, Andreia Cabral de Gouvea, Maria Domenica Moccia, Simon Grüter, Timothy Sykes, Lennart Opitz, Griffin White, Laura Neff, Doris Popovic, Andrea Patrignani, Jay Tracy, Ralph Schlapbach, Christiane Beckmann, Maurice Redondo, Olivier Kobel, Christoph Noppen, Sophie Seidel, Noemie Santamaria de Souza, Niko Beerenwinkel, Tanja Stadler
EPI_ISL_737607, EPI_ISL_737608	Viollier AG	Department of Biosystems Science and Engineering, ETH Zürich	Chaoran Chen, Sarah Nadeau, Ivan Topolsky, Emmanouil Dermitzakis, Keith Harshman, Ioannis Xenarios, Henri Pegeot, Lorenzo Cerutti, Deborah Penet, Philipp Jablonski, Lara Fuhrmann, David Dreifuss, Katharina Jahn, Christiane Beckmann, Maurice Redondo, Olivier Kobel, Christoph Noppen, Sophie Seidel, Noemie Santamaria de Souza, Niko Beerenwinkel, Tanja Stadler
EPI_ISL_737609, EPI_ISL_737625, EPI_ISL_737626, EPI_ISL_737627	Viollier AG	Department of Biosystems Science and Engineering, ETH Zürich	Chaoran Chen, Sarah Nadeau, Catharine Aquino, Ivan Topolsky, Philipp Jablonski, Lara Fuhrmann, David Dreifuss, Katharina Jahn, Andreia Cabral de Gouvea, Maria Domenica Moccia, Simon Grüter, Timothy Sykes, Lennart Opitz, Griffin White, Laura Neff, Doris Popovic, Andrea Patrignani, Jay Tracy, Ralph Schlapbach, Christiane Beckmann, Maurice Redondo, Olivier Kobel, Christoph Noppen, Sophie Seidel, Noemie Santamaria de Souza, Niko Beerenwinkel, Tanja Stadler
EPI_ISL_737628	Viollier AG	Department of Biosystems Science and Engineering, ETH Zürich	Chaoran Chen, Sarah Nadeau, Ivan Topolsky, Emmanouil Dermitzakis, Keith Harshman, Ioannis Xenarios, Henri Pegeot, Lorenzo Cerutti, Deborah Penet, Philipp Jablonski, Lara Fuhrmann, David Dreifuss, Katharina Jahn, Christiane Beckmann, Maurice Redondo, Olivier Kobel, Christoph Noppen, Sophie Seidel, Noemie Santamaria de Souza, Niko Beerenwinkel, Tanja Stadler
EPI_ISL_737629, EPI_ISL_737630, EPI_ISL_737631, EPI_ISL_737632, EPI_ISL_737633, EPI_ISL_737634, EPI_ISL_737635, EPI_ISL_737636, EPI_ISL_737637, EPI_ISL_737638, EPI_ISL_737639, EPI_ISL_737640, EPI_ISL_737641, EPI_ISL_737642			
see above	Viollier AG	Department of Biosystems Science and Engineering, ETH Zürich	Chaoran Chen, Sarah Nadeau, Catharine Aquino, Ivan Topolsky, Philipp Jablonski, Lara Fuhrmann, David Dreifuss, Katharina Jahn, Andreia Cabral de Gouvea, Maria Domenica Moccia, Simon Grüter, Timothy Sykes, Lennart Opitz, Griffin White, Laura Neff, Doris Popovic, Andrea Patrignani, Jay Tracy, Ralph Schlapbach, Christiane Beckmann, Maurice Redondo, Olivier Kobel, Christoph Noppen, Sophie Seidel, Noemie Santamaria de Souza, Niko Beerenwinkel, Tanja Stadler
EPI_ISL_737643	Viollier AG	Department of Biosystems Science and Engineering, ETH Zürich	Chaoran Chen, Sarah Nadeau, Ivan Topolsky, Emmanouil Dermitzakis, Keith Harshman, Ioannis Xenarios, Henri Pegeot, Lorenzo Cerutti, Deborah Penet, Philipp Jablonski, Lara Fuhrmann, David Dreifuss, Katharina Jahn, Christiane Beckmann, Maurice Redondo, Olivier Kobel, Christoph Noppen, Sophie Seidel, Noemie Santamaria de Souza, Niko Beerenwinkel, Tanja Stadler
EPI_ISL_737644, EPI_ISL_737645, EPI_ISL_737646, EPI_ISL_737647, EPI_ISL_737648, EPI_ISL_737649, EPI_ISL_737650, EPI_ISL_737651, EPI_ISL_737652, EPI_ISL_737653, EPI_ISL_737654, EPI_ISL_737655, EPI_ISL_737656, EPI_ISL_737657, EPI_ISL_737658, EPI_ISL_737659, EPI_ISL_737660, EPI_ISL_737661, EPI_ISL_737662, EPI_ISL_737663, EPI_ISL_737664, EPI_ISL_737665, EPI_ISL_737666, EPI_ISL_737667, EPI_ISL_737668, EPI_ISL_737669, EPI_ISL_737670, EPI_ISL_737671, EPI_ISL_737672, EPI_ISL_737673, EPI_ISL_737674, EPI_ISL_737675, EPI_ISL_737676, EPI_ISL_737677, EPI_ISL_737678, EPI_ISL_737679, EPI_ISL_737680, EPI_ISL_737681			
see above	Viollier AG	Department of Biosystems Science and Engineering, ETH Zürich	Chaoran Chen, Sarah Nadeau, Catharine Aquino, Ivan Topolsky, Philipp Jablonski, Lara Fuhrmann, David Dreifuss, Katharina Jahn, Andreia Cabral de Gouvea, Maria Domenica Moccia, Simon Grüter, Timothy Sykes, Lennart Opitz, Griffin White, Laura Neff, Doris Popovic, Andrea Patrignani, Jay Tracy, Ralph Schlapbach, Christiane Beckmann, Maurice Redondo, Olivier Kobel, Christoph Noppen, Sophie Seidel, Noemie Santamaria de Souza, Niko Beerenwinkel, Tanja Stadler
EPI_ISL_737682, EPI_ISL_737683, EPI_ISL_737684, EPI_ISL_737685, EPI_ISL_737686, EPI_ISL_737687, EPI_ISL_737688, EPI_ISL_737689, EPI_ISL_737690, EPI_ISL_737691, EPI_ISL_737692, EPI_ISL_737693, EPI_ISL_737694, EPI_ISL_737695, EPI_ISL_737696, EPI_ISL_737697, EPI_ISL_737698, EPI_ISL_737699, EPI_ISL_737700, EPI_ISL_737701, EPI_ISL_737702, EPI_ISL_737703, EPI_ISL_737704, EPI_ISL_737705, EPI_ISL_737706, EPI_ISL_737707			
see above	Viollier AG	Department of Biosystems Science and Engineering, ETH Zürich	Chaoran Chen, Sarah Nadeau, Ivan Topolsky, Emmanouil Dermitzakis, Keith Harshman, Ioannis Xenarios, Henri Pegeot, Lorenzo Cerutti, Deborah Penet, Philipp Jablonski, Lara Fuhrmann, David Dreifuss, Katharina Jahn, Christiane Beckmann, Maurice Redondo, Olivier Kobel, Christoph Noppen, Sophie Seidel, Noemie Santamaria de Souza, Niko Beerenwinkel, Tanja Stadler
EPI_ISL_737708, EPI_ISL_737709, EPI_ISL_737710, EPI_ISL_737711, EPI_ISL_737712, EPI_ISL_737713, EPI_ISL_737714, EPI_ISL_737715, EPI_ISL_737716, EPI_ISL_737717, EPI_ISL_737718, EPI_ISL_737719, EPI_ISL_737720, EPI_ISL_737721, EPI_ISL_737722, EPI_ISL_737723, EPI_ISL_737724, EPI_ISL_737725, EPI_ISL_737726, EPI_ISL_737727, EPI_ISL_737728, EPI_ISL_737729, EPI_ISL_737730, EPI_ISL_737731, EPI_ISL_737732, EPI_ISL_737733, EPI_ISL_737734, EPI_ISL_737735, EPI_ISL_737736, EPI_ISL_737737, EPI_ISL_737738, EPI_ISL_737739, EPI_ISL_737740, EPI_ISL_737741, EPI_ISL_737742, EPI_ISL_737743, EPI_ISL_737744, EPI_ISL_737745, EPI_ISL_737746, EPI_ISL_737747, EPI_ISL_737748, EPI_ISL_737749, EPI_ISL_737750			
see above	Viollier AG	Department of Biosystems Science and Engineering, ETH Zürich	Chaoran Chen, Sarah Nadeau, Catharine Aquino, Ivan Topolsky, Philipp Jablonski, Lara Fuhrmann, David Dreifuss, Katharina Jahn, Andreia Cabral de Gouvea, Maria Domenica Moccia, Simon Grüter, Timothy Sykes, Lennart Opitz, Griffin White, Laura Neff, Doris Popovic, Andrea Patrignani, Jay Tracy, Ralph Schlapbach, Christiane Beckmann, Maurice Redondo, Olivier Kobel, Christoph Noppen, Sophie Seidel, Noemie Santamaria de Souza, Niko Beerenwinkel, Tanja Stadler
EPI_ISL_737751, EPI_ISL_737752, EPI_ISL_737753, EPI_ISL_737754, EPI_ISL_737755, EPI_ISL_737756, EPI_ISL_737757	Viollier AG	Department of Biosystems Science and Engineering, ETH Zürich	Chaoran Chen, Sarah Nadeau, Ivan Topolsky, Emmanouil Dermitzakis, Keith Harshman, Ioannis Xenarios, Henri Pegeot, Lorenzo Cerutti, Deborah Penet, Philipp Jablonski, Lara Fuhrmann, David Dreifuss, Katharina Jahn, Christiane Beckmann, Maurice Redondo, Olivier Kobel, Christoph Noppen, Sophie Seidel, Noemie Santamaria de Souza, Niko Beerenwinkel, Tanja Stadler
EPI_ISL_737758, EPI_ISL_737759, EPI_ISL_737760, EPI_ISL_737761, EPI_ISL_737762	Viollier AG	Department of Biosystems Science and Engineering, ETH Zürich	Chaoran Chen, Sarah Nadeau, Catharine Aquino, Ivan Topolsky, Philipp Jablonski, Lara Fuhrmann, David Dreifuss, Katharina Jahn, Andreia Cabral de Gouvea, Maria Domenica Moccia, Simon Grüter, Timothy Sykes, Lennart Opitz, Griffin White, Laura Neff, Doris Popovic, Andrea Patrignani, Jay Tracy, Ralph Schlapbach, Christiane Beckmann, Maurice Redondo, Olivier Kobel, Christoph Noppen, Sophie Seidel, Noemie Santamaria de Souza, Niko Beerenwinkel, Tanja Stadler
EPI_ISL_737763, EPI_ISL_737764	Viollier AG	Department of Biosystems Science and Engineering, ETH Zürich	Chaoran Chen, Sarah Nadeau, Ivan Topolsky, Emmanouil Dermitzakis, Keith Harshman, Ioannis Xenarios, Henri Pegeot, Lorenzo Cerutti, Deborah Penet, Philipp Jablonski, Lara Fuhrmann, David Dreifuss, Katharina Jahn, Christiane Beckmann, Maurice Redondo, Olivier Kobel, Christoph Noppen, Sophie Seidel, Noemie Santamaria de Souza, Niko Beerenwinkel, Tanja Stadler
EPI_ISL_737765, EPI_ISL_737766, EPI_ISL_737767, EPI_ISL_737768, EPI_ISL_737769, EPI_ISL_737770, EPI_ISL_737771, EPI_ISL_737772, EPI_ISL_737773, EPI_ISL_737774, EPI_ISL_737775, EPI_ISL_737776, EPI_ISL_737777, EPI_ISL_737778, EPI_ISL_737779, EPI_ISL_737780, EPI_ISL_737781, EPI_ISL_737782, EPI_ISL_737783			
see above	Viollier AG	Department of Biosystems Science and Engineering, ETH Zürich	Chaoran Chen, Sarah Nadeau, Catharine Aquino, Ivan Topolsky, Philipp Jablonski, Lara Fuhrmann, David Dreifuss, Katharina Jahn, Andreia Cabral de Gouvea, Maria Domenica Moccia, Simon Grüter, Timothy Sykes, Lennart Opitz, Griffin White, Laura Neff, Doris Popovic, Andrea Patrignani, Jay Tracy, Ralph Schlapbach, Christiane Beckmann, Maurice Redondo, Olivier Kobel, Christoph Noppen, Sophie Seidel, Noemie Santamaria de Souza, Niko Beerenwinkel, Tanja Stadler
EPI_ISL_737784	Viollier AG	Department of Biosystems Science and Engineering, ETH Zürich	Chaoran Chen, Sarah Nadeau, Ivan Topolsky, Emmanouil Dermitzakis, Keith Harshman, Ioannis Xenarios, Henri Pegeot, Lorenzo Cerutti, Deborah Penet, Philipp Jablonski, Lara Fuhrmann, David Dreifuss, Katharina Jahn, Christiane Beckmann, Maurice Redondo, Olivier Kobel, Christoph Noppen, Sophie Seidel, Noemie Santamaria de Souza, Niko Beerenwinkel, Tanja Stadler
EPI_ISL_737785, EPI_ISL_737786, EPI_ISL_737787, EPI_ISL_737788, EPI_ISL_737789, EPI_ISL_737790, EPI_ISL_737791, EPI_ISL_737792, EPI_ISL_737793, EPI_ISL_737794, EPI_ISL_737795, EPI_ISL_737796, EPI_ISL_737797, EPI_ISL_737798, EPI_ISL_737799, EPI_ISL_737800, EPI_ISL_737801, EPI_ISL_737802, EPI_ISL_737803, EPI_ISL_737804, EPI_ISL_737805, EPI_ISL_737806, EPI_ISL_737807, EPI_ISL_737808, EPI_ISL_737809, EPI_ISL_737810, EPI_ISL_737811, EPI_ISL_737812, EPI_ISL_737813, EPI_ISL_737814, EPI_ISL_737815, EPI_ISL_737816, EPI_ISL_737817, EPI_ISL_737818, EPI_ISL_737819, EPI_ISL_737820, EPI_ISL_737821			
see above	Viollier AG	Department of Biosystems Science and Engineering, ETH	Chaoran Chen, Sarah Nadeau, Catharine Aquino, Ivan Topolsky, Philipp Jablonski, Lara Fuhrmann, David Dreifuss, Katharina Jahn, Andreia Cabral de

		Zürich	Gouvea, Maria Domenica Moccia, Simon Grüter, Timothy Sykes, Lennart Opitz, Griffin White, Laura Neff, Doris Popovic, Andrea Patrignani, Jay Tracy, Ralph Schlapbach, Christiane Beckmann, Maurice Redondo, Olivier Kobel, Christoph Noppen, Sophie Seidel, Noemie Santamaria de Souza, Niko Beerenwinkel, Tanja Stadler
EPI_ISL_737822, EPI_ISL_737823, EPI_ISL_737824, EPI_ISL_737825, EPI_ISL_737826, EPI_ISL_737827, EPI_ISL_737828, EPI_ISL_737829, EPI_ISL_737830, EPI_ISL_737831, EPI_ISL_737832, EPI_ISL_737833, EPI_ISL_737834, EPI_ISL_737835, EPI_ISL_737836, EPI_ISL_737837, EPI_ISL_737838, EPI_ISL_737839			
see above	Viollier AG	Department of Biosystems Science and Engineering, ETH Zürich	Chaoran Chen, Sarah Nadeau, Ivan Topolsky, Emmanouil Dermitzakis, Keith Harshman, Ioannis Xenarios, Henri Pegeot, Lorenzo Cerutti, Deborah Penet, Philipp Jablonski, Lara Fuhrmann, David Dreifuss, Katharina Jahn, Christiane Beckmann, Maurice Redondo, Olivier Kobel, Christoph Noppen, Sophie Seidel, Noemie Santamaria de Souza, Niko Beerenwinkel, Tanja Stadler
EPI_ISL_737840, EPI_ISL_737841, EPI_ISL_737842, EPI_ISL_737843, EPI_ISL_737844, EPI_ISL_737845, EPI_ISL_737846, EPI_ISL_737847, EPI_ISL_737848, EPI_ISL_737849, EPI_ISL_737850, EPI_ISL_737851, EPI_ISL_737852, EPI_ISL_737853, EPI_ISL_737854, EPI_ISL_737855, EPI_ISL_737856, EPI_ISL_737857, EPI_ISL_737858, EPI_ISL_737859, EPI_ISL_737860, EPI_ISL_737861, EPI_ISL_737862, EPI_ISL_737863, EPI_ISL_737864, EPI_ISL_737865, EPI_ISL_737866, EPI_ISL_737867, EPI_ISL_737868, EPI_ISL_737869, EPI_ISL_737870, EPI_ISL_737871, EPI_ISL_737872, EPI_ISL_737873, EPI_ISL_737874, EPI_ISL_737875, EPI_ISL_737876, EPI_ISL_737877, EPI_ISL_737878			
see above	Viollier AG	Department of Biosystems Science and Engineering, ETH Zürich	Chaoran Chen, Sarah Nadeau, Catharine Aquino, Ivan Topolsky, Philipp Jablonski, Lara Fuhrmann, David Dreifuss, Katharina Jahn, Andreia Cabral de Gouvea, Maria Domenica Moccia, Simon Grüter, Timothy Sykes, Lennart Opitz, Griffin White, Laura Neff, Doris Popovic, Andrea Patrignani, Jay Tracy, Ralph Schlapbach, Christiane Beckmann, Maurice Redondo, Olivier Kobel, Christoph Noppen, Sophie Seidel, Noemie Santamaria de Souza, Niko Beerenwinkel, Tanja Stadler
EPI_ISL_737879, EPI_ISL_737880, EPI_ISL_737881, EPI_ISL_737882, EPI_ISL_737883, EPI_ISL_737884, EPI_ISL_737885, EPI_ISL_737886, EPI_ISL_737887, EPI_ISL_737888, EPI_ISL_737889, EPI_ISL_737890, EPI_ISL_737891, EPI_ISL_737892, EPI_ISL_737893, EPI_ISL_737894, EPI_ISL_737895, EPI_ISL_737896, EPI_ISL_737897, EPI_ISL_737898, EPI_ISL_737899, EPI_ISL_737900			
see above	Viollier AG	Department of Biosystems Science and Engineering, ETH Zürich	Chaoran Chen, Sarah Nadeau, Ivan Topolsky, Emmanouil Dermitzakis, Keith Harshman, Ioannis Xenarios, Henri Pegeot, Lorenzo Cerutti, Deborah Penet, Philipp Jablonski, Lara Fuhrmann, David Dreifuss, Katharina Jahn, Christiane Beckmann, Maurice Redondo, Olivier Kobel, Christoph Noppen, Sophie Seidel, Noemie Santamaria de Souza, Niko Beerenwinkel, Tanja Stadler
EPI_ISL_737901, EPI_ISL_737902, EPI_ISL_737903, EPI_ISL_737904, EPI_ISL_737905, EPI_ISL_737906, EPI_ISL_737907, EPI_ISL_737908, EPI_ISL_737909, EPI_ISL_737910, EPI_ISL_737911, EPI_ISL_737912, EPI_ISL_737913, EPI_ISL_737914, EPI_ISL_737915, EPI_ISL_737916, EPI_ISL_737917, EPI_ISL_737918, EPI_ISL_737919, EPI_ISL_737920, EPI_ISL_737921, EPI_ISL_737922, EPI_ISL_737923, EPI_ISL_737924, EPI_ISL_737925, EPI_ISL_737926, EPI_ISL_737927, EPI_ISL_737928, EPI_ISL_737929			
see above	Viollier AG	Department of Biosystems Science and Engineering, ETH Zürich	Chaoran Chen, Sarah Nadeau, Catharine Aquino, Ivan Topolsky, Philipp Jablonski, Lara Fuhrmann, David Dreifuss, Katharina Jahn, Andreia Cabral de Gouvea, Maria Domenica Moccia, Simon Grüter, Timothy Sykes, Lennart Opitz, Griffin White, Laura Neff, Doris Popovic, Andrea Patrignani, Jay Tracy, Ralph Schlapbach, Christiane Beckmann, Maurice Redondo, Olivier Kobel, Christoph Noppen, Sophie Seidel, Noemie Santamaria de Souza, Niko Beerenwinkel, Tanja Stadler
EPI_ISL_737930	Hospital Universitario 12 de Octubre	Hospital Universitario 12 de Octubre	Raul Recio, Esther Viedma, Sara González, Irene Múñoz-Gallego, Mª Dolores Folgueira, Rafael Delgado
EPI_ISL_737931, EPI_ISL_737939, EPI_ISL_737940, EPI_ISL_737941, EPI_ISL_737942, EPI_ISL_737943, EPI_ISL_737944, EPI_ISL_737945, EPI_ISL_737965, EPI_ISL_737971, EPI_ISL_737972, EPI_ISL_737973, EPI_ISL_737974, EPI_ISL_737975, EPI_ISL_737976, EPI_ISL_737977, EPI_ISL_737978, EPI_ISL_737979, EPI_ISL_737980, EPI_ISL_737981, EPI_ISL_737982, EPI_ISL_737983, EPI_ISL_737985, EPI_ISL_737986, EPI_ISL_737987, EPI_ISL_737988, EPI_ISL_737989, EPI_ISL_737990, EPI_ISL_737991, EPI_ISL_737992, EPI_ISL_737993, EPI_ISL_737994, EPI_ISL_737995, EPI_ISL_737997, EPI_ISL_737998, EPI_ISL_737999, EPI_ISL_738000, EPI_ISL_738001, EPI_ISL_738002, EPI_ISL_738003, EPI_ISL_738004, EPI_ISL_738005, EPI_ISL_738006, EPI_ISL_738007, EPI_ISL_738008, EPI_ISL_738009, EPI_ISL_738010, EPI_ISL_738011, EPI_ISL_738012, EPI_ISL_738013, EPI_ISL_738014, EPI_ISL_738015, EPI_ISL_738016, EPI_ISL_738017, EPI_ISL_738018, EPI_ISL_738019, EPI_ISL_738020, EPI_ISL_738021, EPI_ISL_738022, EPI_ISL_738023, EPI_ISL_738025, EPI_ISL_738026, EPI_ISL_738027, EPI_ISL_738028, EPI_ISL_738029, EPI_ISL_738030, EPI_ISL_738031, EPI_ISL_738032, EPI_ISL_738033, EPI_ISL_738034, EPI_ISL_738035, EPI_ISL_738036, EPI_ISL_738037, EPI_ISL_738038, EPI_ISL_738039, EPI_ISL_738040, EPI_ISL_738041, EPI_ISL_738042, EPI_ISL_738043			
see above	Uganda Central Public Health Lab and Uganda Virus Research Institute	MRC/UVRI & LSHTM Uganda Research Unit	Matthew Cotten, Dan Lule Bugembe, My V.T. Phan, Pontiano Kaleebu et al.
EPI_ISL_738044	SIESP CHIETI - DRIVE IN LANCIANO	Istituto Zooprofilattico Sperimentale dell'Abruzzo e Molise "G. Caporale"	Lorusso A, Marcacci M, Di Domenico M, Ancora M, Curini V, Mangone I, Rinaldi A, Di Pasquale A, Cammà C, Puglia I, Savini G
EPI_ISL_738045	SIESP DIPARTIMENTO DI PREVENZIONE CHIETI	Istituto Zooprofilattico Sperimentale dell'Abruzzo e Molise "G. Caporale"	Lorusso A, Marcacci M, Di Domenico M, Ancora M, Curini V, Mangone I, Rinaldi A, Di Pasquale A, Cammà C, Puglia I, Savini G
EPI_ISL_738046, EPI_ISL_738047, EPI_ISL_738048	SIESP CHIETI - DRIVE IN ORTONA	Istituto Zooprofilattico Sperimentale dell'Abruzzo e Molise "G. Caporale"	Lorusso A, Marcacci M, Di Domenico M, Ancora M, Curini V, Mangone I, Rinaldi A, Di Pasquale A, Cammà C, Puglia I, Savini G
EPI_ISL_738064, EPI_ISL_738065	Department of Laboratory Medicine, National Taiwan University Hospital	Microbial Genomics Core Lab, National Taiwan University Centers of Genomic and Precision Medicine	Shiou-Hwei Yeh, You-Yu Lin, Ya-Yun Lai, Chiao-Ling Li, Shan-Chwen Chang, Pei-Jer Chen, Sui-Yuan Chang
EPI_ISL_738080	Laboratory Services Section, Texas Department of State Health Services	Laboratory Services Section, Texas Department of State Health Services	Pokharel,A., Oh,B., Bonser,J.D., Tuladhar,R., Pedrueza,M., Zhang,J., Rahman,M., Koag,M., Wang,C., Lee,R. and Kubin,G.
EPI_ISL_738088, EPI_ISL_738089, EPI_ISL_738090, EPI_ISL_738091, EPI_ISL_738092, EPI_ISL_738093, EPI_ISL_738094, EPI_ISL_738095, EPI_ISL_738096, EPI_ISL_738097, EPI_ISL_738098, EPI_ISL_738099, EPI_ISL_738100, EPI_ISL_738101, EPI_ISL_738102, EPI_ISL_738103, EPI_ISL_738104, EPI_ISL_738105, EPI_ISL_738106, EPI_ISL_738107, EPI_ISL_738108, EPI_ISL_738109, EPI_ISL_738110, EPI_ISL_738112, EPI_ISL_738113, EPI_ISL_738114, EPI_ISL_738115, EPI_ISL_738116, EPI_ISL_738117, EPI_ISL_738118, EPI_ISL_738119, EPI_ISL_738120			
see above	Instituto Nacional de Saude (INSA)	Instituto Nacional de Saude (INSA)	Borges et al
EPI_ISL_738121, EPI_ISL_738122, EPI_ISL_738123, EPI_ISL_738124, EPI_ISL_738125, EPI_ISL_738126, EPI_ISL_738127, EPI_ISL_738128, EPI_ISL_738129, EPI_ISL_738130, EPI_ISL_738131, EPI_ISL_738132			
see above	IZSM-U.O.C. Virologia	Istituto Zooprofilattico Sperimentale del Mezzogiorno	Maurizio Viscardi, Lorena Cardillo, Giovanna Fusco
EPI_ISL_738133	The Public Health Agency of Sweden	The Public Health Agency of Sweden	Oskar Karlsson Lindsjo, Maria Lind Karlberg, Anna-Malin Linde, Sofia Stamouli, Olov Svartstrom, Anna Risberg, Shaman Muradrasoli, Karin Tegmark-Wisell
EPI_ISL_738134, EPI_ISL_738135, EPI_ISL_738136	Department of Virology and Immunology, University of Helsinki and Helsinki University Hospital, Huslab Finland	Department of Virology, Faculty of Medicine, University of Helsinki, Helsinki, Finland	Teemu Smura, Olli Vapalahti, Maija Lappalainen, Satu Kurkela
EPI_ISL_738137	Area of Virology, Serology and Virology Division (SAViD), New South Wales Health Pathology Randwick	Area of Virology, Serology and Virology Division (SAViD), New South Wales Health Pathology Randwick	Rawlinson, W., Deveson, I., Van Haal, S., Foster, C.
EPI_ISL_738139, EPI_ISL_738141, EPI_ISL_738142	Division of Emerging Infectious Diseases, Bureau of Infectious Diseases Diagnosis Control, Korea Disease Control and Prevention Agency	Division of Emerging Infectious Diseases, Bureau of Infectious Diseases Diagnosis Control, Korea Disease Control and Prevention Agency	Jeong-Min Kim, Il-Hwan Kim, Ae Kyung Park, Namjoo Lee, Sang Hee Woo, Heui Man Kim, Eun-Jin Kim
EPI_ISL_738144	University of Bari Biomedical Sciences and Human Oncology	University of Bari Biomedical Sciences and Human Oncology	Maria Chironna, Anna Sallustio, Daniela Loconsole, Marisa Accogli
EPI_ISL_738146	Instituto Nacional de Saude (INSA)	Instituto Nacional de Saude (INSA)	Borges et al
EPI_ISL_738148, EPI_ISL_738149, EPI_ISL_738150, EPI_ISL_738151, EPI_ISL_738152, EPI_ISL_738153, EPI_ISL_738154, EPI_ISL_738155, EPI_ISL_738156, EPI_ISL_738157, EPI_ISL_738158, EPI_ISL_738159, EPI_ISL_738160, EPI_ISL_738161, EPI_ISL_738162, EPI_ISL_738163, EPI_ISL_738164, EPI_ISL_738165, EPI_ISL_738166, EPI_ISL_738167, EPI_ISL_738168, EPI_ISL_738169, EPI_ISL_738170, EPI_ISL_738171, EPI_ISL_738172, EPI_ISL_738173, EPI_ISL_738174, EPI_ISL_738175, EPI_ISL_738176, EPI_ISL_738177, EPI_ISL_738178, EPI_ISL_738179, EPI_ISL_738180, EPI_ISL_738181, EPI_ISL_738182, EPI_ISL_738183, EPI_ISL_738184, EPI_ISL_738185, EPI_ISL_738186, EPI_ISL_738187, EPI_ISL_738188, EPI_ISL_738189, EPI_ISL_738190, EPI_ISL_738191, EPI_ISL_738192			
see above	Texas Department of State Health Services	Texas Department of State Health Services	Anita Pokharel, Bonnie Oh, James Daniel Bonser, Rashmi Tuladhar, Mayela Pedrueza, Jenny Zhang, Maliha Rahman, Myong Koag, Chung Wang, Rachel Lee, Grace Kubin
EPI_ISL_738195, EPI_ISL_738218, EPI_ISL_738219, EPI_ISL_738220, EPI_ISL_738221, EPI_ISL_738222, EPI_ISL_738223, EPI_ISL_738224, EPI_ISL_738225, EPI_ISL_738226, EPI_ISL_738227, EPI_ISL_738228, EPI_ISL_738229, EPI_ISL_738230, EPI_ISL_738231, EPI_ISL_738232, EPI_ISL_738233, EPI_ISL_738234, EPI_ISL_738235, EPI_ISL_738236, EPI_ISL_738237, EPI_ISL_738238, EPI_ISL_738239, EPI_ISL_738240			
see above	UZ Leuven, National Reference Laboratory for Coronaviruses, Laboratory Medicine, Leuven, Belgium	KU Leuven, Rega Institute, Clinical and Epidemiological Virology	Tony Wawina-Bokalanga, Joan Marti-Carerras, Bert Vanmechelen, Piet Maes
EPI_ISL_738313	Foerde Hospital, Department of Microbiology	Norwegian Institute of Public Health, Department of Virology	Kathrine Stene-Johansen, Kamilla Heddeland Instefjord, Hilde Elshaug, Atiya R Ali,Marie Paulsen Madsen, Rasmus Riis Kopperud, Hilde Vollan, Karoline Bragstad, Olav Hungnes
EPI_ISL_738314	Unilabs Laboratory Medicine	Norwegian Institute of Public Health, Department of Virology	Kathrine Stene-Johansen, Kamilla Heddeland Instefjord, Hilde Elshaug, Atiya R Ali,Marie Paulsen Madsen, Rasmus Riis Kopperud, Hilde Vollan, Karoline Bragstad, Olav Hungnes

EPI_ISL_738316	Utah Public Health Laboratory	Utah Public Health Laboratory	Erin Young, Kelly Oakeson, Tara Gallagher
EPI_ISL_738317, EPI_ISL_738318, EPI_ISL_738319, EPI_ISL_738320, EPI_ISL_738321, EPI_ISL_738322, EPI_ISL_738326, EPI_ISL_738327, EPI_ISL_738328, EPI_ISL_738329, EPI_ISL_738330, EPI_ISL_738331, EPI_ISL_738332, EPI_ISL_738333, EPI_ISL_738334, EPI_ISL_738335, EPI_ISL_738336, EPI_ISL_738337, EPI_ISL_738338, EPI_ISL_738339, EPI_ISL_738340, EPI_ISL_738341, EPI_ISL_738342, EPI_ISL_738343, EPI_ISL_738344, EPI_ISL_738345, EPI_ISL_738346, EPI_ISL_738347, EPI_ISL_738348, EPI_ISL_738349			
see above	Landstuhl Regional Medical Center	United States Air Force School of Aerospace Medicine	Anthony Fries, Jennifer Meyer, Amanda Javorina, Sarah Purves, William Gruner, Clarise Starr, Elizabeth Macias, Fritz Castillo, Cole Anderson
EPI_ISL_738350, EPI_ISL_738351, EPI_ISL_738352, EPI_ISL_738353, EPI_ISL_738354, EPI_ISL_738355, EPI_ISL_738356, EPI_ISL_738357, EPI_ISL_738358, EPI_ISL_738359	Utah Public Health Laboratory	Utah Public Health Laboratory	Erin Young, Kelly Oakeson, Tara Gallagher
EPI_ISL_738360, EPI_ISL_738361, EPI_ISL_738362, EPI_ISL_738363, EPI_ISL_738364, EPI_ISL_738365, EPI_ISL_738366, EPI_ISL_738367, EPI_ISL_738368, EPI_ISL_738369, EPI_ISL_738370, EPI_ISL_738371, EPI_ISL_738372, EPI_ISL_738373, EPI_ISL_738374, EPI_ISL_738375, EPI_ISL_738376, EPI_ISL_738377, EPI_ISL_738378, EPI_ISL_738379, EPI_ISL_738380, EPI_ISL_738381, EPI_ISL_738382, EPI_ISL_738383, EPI_ISL_738384, EPI_ISL_738385, EPI_ISL_738386, EPI_ISL_738387, EPI_ISL_738388, EPI_ISL_738389, EPI_ISL_738390, EPI_ISL_738391, EPI_ISL_738392, EPI_ISL_738393, EPI_ISL_738394, EPI_ISL_738395, EPI_ISL_738396, EPI_ISL_738397, EPI_ISL_738398, EPI_ISL_738399, EPI_ISL_738400, EPI_ISL_738401, EPI_ISL_738402, EPI_ISL_738403, EPI_ISL_738404, EPI_ISL_738405, EPI_ISL_738406, EPI_ISL_738407, EPI_ISL_738408, EPI_ISL_738409, EPI_ISL_738410, EPI_ISL_738411, EPI_ISL_738412, EPI_ISL_738413, EPI_ISL_738414, EPI_ISL_738415, EPI_ISL_738416, EPI_ISL_738417, EPI_ISL_738418, EPI_ISL_738419, EPI_ISL_738420, EPI_ISL_738421, EPI_ISL_738422, EPI_ISL_738423, EPI_ISL_738424, EPI_ISL_738425, EPI_ISL_738426, EPI_ISL_738427, EPI_ISL_738428, EPI_ISL_738429, EPI_ISL_738430, EPI_ISL_738431, EPI_ISL_738432, EPI_ISL_738433, EPI_ISL_738434, EPI_ISL_738435, EPI_ISL_738436, EPI_ISL_738437, EPI_ISL_738438, EPI_ISL_738439, EPI_ISL_738440, EPI_ISL_738441, EPI_ISL_738442, EPI_ISL_738443, EPI_ISL_738444, EPI_ISL_738445, EPI_ISL_738446, EPI_ISL_738447, EPI_ISL_738448, EPI_ISL_738449, EPI_ISL_738450, EPI_ISL_738451, EPI_ISL_738452, EPI_ISL_738453, EPI_ISL_738454, EPI_ISL_738455, EPI_ISL_738456, EPI_ISL_738457, EPI_ISL_738458, EPI_ISL_738459, EPI_ISL_738460, EPI_ISL_738461, EPI_ISL_738462, EPI_ISL_738463, EPI_ISL_738464, EPI_ISL_738465, EPI_ISL_738466, EPI_ISL_738467, EPI_ISL_738468, EPI_ISL_738469, EPI_ISL_738470, EPI_ISL_738471, EPI_ISL_738472, EPI_ISL_738473, EPI_ISL_738474, EPI_ISL_738475, EPI_ISL_738476, EPI_ISL_738477, EPI_ISL_738478, EPI_ISL_738479, EPI_ISL_738480, EPI_ISL_738481, EPI_ISL_738482, EPI_ISL_738483, EPI_ISL_738484, EPI_ISL_738485, EPI_ISL_738486, EPI_ISL_738487, EPI_ISL_738488, EPI_ISL_738489, EPI_ISL_738490, EPI_ISL_738491, EPI_ISL_738492, EPI_ISL_738493, EPI_ISL_738494, EPI_ISL_738495, EPI_ISL_738496, EPI_ISL_738497, EPI_ISL_738498, EPI_ISL_738499			
see above	UZ Leuven, National Reference Laboratory for Coronaviruses, Laboratory Medicine, Leuven, Belgium	KU Leuven, Rega Institute, Clinical and Epidemiological Virology	Tony Wawina-Bokalanga, Joan Marti-Carerras, Bert Vanmechelen, Piet Maes
EPI_ISL_738500, EPI_ISL_738501, EPI_ISL_738502	Santa Clara County Public Health Laboratory	Chan-Zuckerberg Biohub	CZB Cliahub Consortium
EPI_ISL_738503	Orange County Public Health Lab	Chan-Zuckerberg Biohub	CZB Cliahub Consortium
EPI_ISL_738504, EPI_ISL_738505	Humboldt County Public Health Laboratory	Chan-Zuckerberg Biohub	CZB Cliahub Consortium
EPI_ISL_738506	Monterey County Public Health Lab	Chan-Zuckerberg Biohub	CZB Cliahub Consortium
EPI_ISL_738507	Orange County Public Health Lab	Chan-Zuckerberg Biohub	CZB Cliahub Consortium
EPI_ISL_738508	Santa Clara County Public Health Laboratory	Chan-Zuckerberg Biohub	CZB Cliahub Consortium
EPI_ISL_738510	Alameda County Public Health Lab	Chan-Zuckerberg Biohub	CZB Cliahub Consortium
EPI_ISL_738511	Santa Clara County Public Health Laboratory	Chan-Zuckerberg Biohub	CZB Cliahub Consortium
EPI_ISL_738512	Napa-Solano-Yolo- Marin County (NSYM) Public Health Laboratories	Chan-Zuckerberg Biohub	CZB Cliahub Consortium
EPI_ISL_738513, EPI_ISL_738514	Alameda County Public Health Lab	Chan-Zuckerberg Biohub	CZB Cliahub Consortium
EPI_ISL_738515	Orange County Public Health Lab	Chan-Zuckerberg Biohub	CZB Cliahub Consortium
EPI_ISL_738516, EPI_ISL_738517	Santa Clara County Public Health Laboratory	Chan-Zuckerberg Biohub	CZB Cliahub Consortium
EPI_ISL_738518	Orange County Public Health Lab	Chan-Zuckerberg Biohub	CZB Cliahub Consortium
EPI_ISL_738519	Alameda County Public Health Lab	Chan-Zuckerberg Biohub	CZB Cliahub Consortium
EPI_ISL_738520, EPI_ISL_738521	Santa Clara County Public Health Laboratory	Chan-Zuckerberg Biohub	CZB Cliahub Consortium
EPI_ISL_738522, EPI_ISL_738523, EPI_ISL_738524	Alameda County Public Health Lab	Chan-Zuckerberg Biohub	CZB Cliahub Consortium
EPI_ISL_738525, EPI_ISL_738526	Santa Clara County Public Health Laboratory	Chan-Zuckerberg Biohub	CZB Cliahub Consortium
EPI_ISL_738527	Alameda County Public Health Lab	Chan-Zuckerberg Biohub	CZB Cliahub Consortium
EPI_ISL_738529	Orange County Public Health Lab	Chan-Zuckerberg Biohub	CZB Cliahub Consortium
EPI_ISL_738530	Humboldt County Public Health Laboratory	Chan-Zuckerberg Biohub	CZB Cliahub Consortium
EPI_ISL_738531	Alameda County Public Health Lab	Chan-Zuckerberg Biohub	CZB Cliahub Consortium
EPI_ISL_738532, EPI_ISL_738533, EPI_ISL_738534	Santa Clara County Public Health Laboratory	Chan-Zuckerberg Biohub	CZB Cliahub Consortium
EPI_ISL_738535	Alameda County Public Health Lab	Chan-Zuckerberg Biohub	CZB Cliahub Consortium
EPI_ISL_738536	Santa Clara County Public Health Laboratory	Chan-Zuckerberg Biohub	CZB Cliahub Consortium
EPI_ISL_738537, EPI_ISL_738538	Alameda County Public Health Lab	Chan-Zuckerberg Biohub	CZB Cliahub Consortium
EPI_ISL_738539	Humboldt County Public Health Laboratory	Chan-Zuckerberg Biohub	CZB Cliahub Consortium
EPI_ISL_738540	Alameda County Public Health Lab	Chan-Zuckerberg Biohub	CZB Cliahub Consortium
EPI_ISL_738541	Napa-Solano-Yolo- Marin County (NSYM) Public Health Laboratories	Chan-Zuckerberg Biohub	CZB Cliahub Consortium
EPI_ISL_738542, EPI_ISL_738543	Santa Clara County Public Health Laboratory	Chan-Zuckerberg Biohub	CZB Cliahub Consortium
EPI_ISL_738544	UCSF Clinical Microbiology Laboratory	Chan-Zuckerberg Biohub	CZB Cliahub Consortium
EPI_ISL_738545	Alameda County Public Health Lab	Chan-Zuckerberg Biohub	CZB Cliahub Consortium
EPI_ISL_738546	Santa Clara County Public Health Laboratory	Chan-Zuckerberg Biohub	CZB Cliahub Consortium
EPI_ISL_738547	Alameda County Public Health Lab	Chan-Zuckerberg Biohub	CZB Cliahub Consortium
EPI_ISL_738548	Orange County Public Health Lab	Chan-Zuckerberg Biohub	CZB Cliahub Consortium
EPI_ISL_738549, EPI_ISL_738550, EPI_ISL_738551, EPI_ISL_738552	Santa Clara County Public Health Laboratory	Chan-Zuckerberg Biohub	CZB Cliahub Consortium
EPI_ISL_738553, EPI_ISL_738554	Alameda County Public Health Lab	Chan-Zuckerberg Biohub	CZB Cliahub Consortium
EPI_ISL_738555, EPI_ISL_738556, EPI_ISL_738557, EPI_ISL_738558	Santa Clara County Public Health Laboratory	Chan-Zuckerberg Biohub	CZB Cliahub Consortium
EPI_ISL_738559	Orange County Public Health Lab	Chan-Zuckerberg Biohub	CZB Cliahub Consortium
EPI_ISL_738560	Santa Clara County Public Health Laboratory	Chan-Zuckerberg Biohub	CZB Cliahub Consortium

EPI_ISL_738561, EPI_ISL_738562	Alameda County Public Health Lab	Chan-Zuckerberg Biohub	CZB Cliahub Consortium
EPI_ISL_738563	Humboldt County Public Health Laboratory	Chan-Zuckerberg Biohub	CZB Cliahub Consortium
EPI_ISL_738564, EPI_ISL_738565	Alameda County Public Health Lab	Chan-Zuckerberg Biohub	CZB Cliahub Consortium
EPI_ISL_738566	Santa Clara County Public Health Laboratory	Chan-Zuckerberg Biohub	CZB Cliahub Consortium
EPI_ISL_738567	Renegade	Chan-Zuckerberg Biohub	CZB Cliahub Consortium
EPI_ISL_738568	Santa Clara County Public Health Laboratory	Chan-Zuckerberg Biohub	CZB Cliahub Consortium
EPI_ISL_738569, EPI_ISL_738570, EPI_ISL_738571	Orange County Public Health Lab	Chan-Zuckerberg Biohub	CZB Cliahub Consortium
EPI_ISL_738572, EPI_ISL_738573	Alameda County Public Health Lab	Chan-Zuckerberg Biohub	CZB Cliahub Consortium
EPI_ISL_738574, EPI_ISL_738575, EPI_ISL_738576	Renegade	Chan-Zuckerberg Biohub	CZB Cliahub Consortium
EPI_ISL_738577	Alameda County Public Health Lab	Chan-Zuckerberg Biohub	CZB Cliahub Consortium
EPI_ISL_738578, EPI_ISL_738579, EPI_ISL_738581, EPI_ISL_738582, EPI_ISL_738583	Santa Clara County Public Health Laboratory	Chan-Zuckerberg Biohub	CZB Cliahub Consortium
EPI_ISL_738584, EPI_ISL_738585	Alameda County Public Health Lab	Chan-Zuckerberg Biohub	CZB Cliahub Consortium
EPI_ISL_738586	Santa Clara County Public Health Laboratory	Chan-Zuckerberg Biohub	CZB Cliahub Consortium
EPI_ISL_738588	Orange County Public Health Lab	Chan-Zuckerberg Biohub	CZB Cliahub Consortium
EPI_ISL_738589, EPI_ISL_738590	Santa Clara County Public Health Laboratory	Chan-Zuckerberg Biohub	CZB Cliahub Consortium
EPI_ISL_738591	Renegade	Chan-Zuckerberg Biohub	CZB Cliahub Consortium
EPI_ISL_738592	Alameda County Public Health Lab	Chan-Zuckerberg Biohub	CZB Cliahub Consortium
EPI_ISL_738593	Santa Clara County Public Health Laboratory	Chan-Zuckerberg Biohub	CZB Cliahub Consortium
EPI_ISL_738594	Orange County Public Health Lab	Chan-Zuckerberg Biohub	CZB Cliahub Consortium
EPI_ISL_738595, EPI_ISL_738596, EPI_ISL_738597, EPI_ISL_738598	Santa Clara County Public Health Laboratory	Chan-Zuckerberg Biohub	CZB Cliahub Consortium
EPI_ISL_738599	Orange County Public Health Lab	Chan-Zuckerberg Biohub	CZB Cliahub Consortium
EPI_ISL_738600	Humboldt County Public Health Laboratory	Chan-Zuckerberg Biohub	CZB Cliahub Consortium
EPI_ISL_738601	Santa Clara County Public Health Laboratory	Chan-Zuckerberg Biohub	CZB Cliahub Consortium
EPI_ISL_738602, EPI_ISL_738603	Orange County Public Health Lab	Chan-Zuckerberg Biohub	CZB Cliahub Consortium
EPI_ISL_738604, EPI_ISL_738605, EPI_ISL_738606, EPI_ISL_738607	Alameda County Public Health Lab	Chan-Zuckerberg Biohub	CZB Cliahub Consortium
EPI_ISL_738608	Santa Clara County Public Health Laboratory	Chan-Zuckerberg Biohub	CZB Cliahub Consortium
EPI_ISL_738609	Alameda County Public Health Lab	Chan-Zuckerberg Biohub	CZB Cliahub Consortium
EPI_ISL_738610, EPI_ISL_738611, EPI_ISL_738612	Santa Clara County Public Health Laboratory	Chan-Zuckerberg Biohub	CZB Cliahub Consortium
EPI_ISL_738613, EPI_ISL_738614, EPI_ISL_738615, EPI_ISL_738616	Alameda County Public Health Lab	Chan-Zuckerberg Biohub	CZB Cliahub Consortium
EPI_ISL_738617, EPI_ISL_738618	Santa Clara County Public Health Laboratory	Chan-Zuckerberg Biohub	CZB Cliahub Consortium
EPI_ISL_738619	Alameda County Public Health Lab	Chan-Zuckerberg Biohub	CZB Cliahub Consortium
EPI_ISL_738620, EPI_ISL_738621	Orange County Public Health Lab	Chan-Zuckerberg Biohub	CZB Cliahub Consortium
EPI_ISL_738622, EPI_ISL_738623	Alameda County Public Health Lab	Chan-Zuckerberg Biohub	CZB Cliahub Consortium
EPI_ISL_738624	Orange County Public Health Lab	Chan-Zuckerberg Biohub	CZB Cliahub Consortium
EPI_ISL_738625, EPI_ISL_738626, EPI_ISL_738627	Alameda County Public Health Lab	Chan-Zuckerberg Biohub	CZB Cliahub Consortium
EPI_ISL_738628	Santa Clara County Public Health Laboratory	Chan-Zuckerberg Biohub	CZB Cliahub Consortium
EPI_ISL_738629	Alameda County Public Health Lab	Chan-Zuckerberg Biohub	CZB Cliahub Consortium
EPI_ISL_738630	Orange County Public Health Lab	Chan-Zuckerberg Biohub	CZB Cliahub Consortium
EPI_ISL_738631	Santa Clara County Public Health Laboratory	Chan-Zuckerberg Biohub	CZB Cliahub Consortium
EPI_ISL_738632	Orange County Public Health Lab	Chan-Zuckerberg Biohub	CZB Cliahub Consortium
EPI_ISL_738633	Alameda County Public Health Lab	Chan-Zuckerberg Biohub	CZB Cliahub Consortium
EPI_ISL_738634, EPI_ISL_738635, EPI_ISL_738636, EPI_ISL_738637, EPI_ISL_738638, EPI_ISL_738639, EPI_ISL_738640, EPI_ISL_738641	Orange County Public Health Lab	Chan-Zuckerberg Biohub	CZB Cliahub Consortium
EPI_ISL_738642	Alameda County Public Health Lab	Chan-Zuckerberg Biohub	CZB Cliahub Consortium
EPI_ISL_738643, EPI_ISL_738644, EPI_ISL_738645	Santa Clara County Public Health Laboratory	Chan-Zuckerberg Biohub	CZB Cliahub Consortium
EPI_ISL_738646	Orange County Public Health Lab	Chan-Zuckerberg Biohub	CZB Cliahub Consortium
EPI_ISL_738647	Humboldt County Public Health Laboratory	Chan-Zuckerberg Biohub	CZB Cliahub Consortium
EPI_ISL_738648	Alameda County Public Health Lab	Chan-Zuckerberg Biohub	CZB Cliahub Consortium
EPI_ISL_738649	Humboldt County Public Health Laboratory	Chan-Zuckerberg Biohub	CZB Cliahub Consortium
EPI_ISL_738650	Santa Clara County Public Health Laboratory	Chan-Zuckerberg Biohub	CZB Cliahub Consortium
EPI_ISL_738651	Humboldt County Public Health Laboratory	Chan-Zuckerberg Biohub	CZB Cliahub Consortium

EPI_ISL_738652, EPI_ISL_738653	Santa Clara County Public Health Laboratory	Chan-Zuckerberg Biohub	CZB Cliahub Consortium
EPI_ISL_738654	Alameda County Public Health Lab	Chan-Zuckerberg Biohub	CZB Cliahub Consortium
EPI_ISL_738655	Orange County Public Health Lab	Chan-Zuckerberg Biohub	CZB Cliahub Consortium
EPI_ISL_738656	Alameda County Public Health Lab	Chan-Zuckerberg Biohub	CZB Cliahub Consortium
EPI_ISL_738657	Santa Clara County Public Health Laboratory	Chan-Zuckerberg Biohub	CZB Cliahub Consortium
EPI_ISL_738658	Monterey County Public Health Lab	Chan-Zuckerberg Biohub	CZB Cliahub Consortium
EPI_ISL_738659	Alameda County Public Health Lab	Chan-Zuckerberg Biohub	CZB Cliahub Consortium
EPI_ISL_738660	Renegade	Chan-Zuckerberg Biohub	CZB Cliahub Consortium
EPI_ISL_738661	Santa Clara County Public Health Laboratory	Chan-Zuckerberg Biohub	CZB Cliahub Consortium
EPI_ISL_738662	Renegade	Chan-Zuckerberg Biohub	CZB Cliahub Consortium
EPI_ISL_738663	Santa Clara County Public Health Laboratory	Chan-Zuckerberg Biohub	CZB Cliahub Consortium
EPI_ISL_738664	Orange County Public Health Lab	Chan-Zuckerberg Biohub	CZB Cliahub Consortium
EPI_ISL_738666	Madera County Department of Public Health	Chan-Zuckerberg Biohub	CZB Cliahub Consortium
EPI_ISL_738667	Santa Clara County Public Health Laboratory	Chan-Zuckerberg Biohub	CZB Cliahub Consortium
EPI_ISL_738668	Alameda County Public Health Lab	Chan-Zuckerberg Biohub	CZB Cliahub Consortium
EPI_ISL_738669	Orange County Public Health Lab	Chan-Zuckerberg Biohub	CZB Cliahub Consortium
EPI_ISL_738670	Santa Clara County Public Health Laboratory	Chan-Zuckerberg Biohub	CZB Cliahub Consortium
EPI_ISL_738671	Alameda County Public Health Lab	Chan-Zuckerberg Biohub	CZB Cliahub Consortium
EPI_ISL_738672, EPI_ISL_738673	Santa Clara County Public Health Laboratory	Chan-Zuckerberg Biohub	CZB Cliahub Consortium
EPI_ISL_738674, EPI_ISL_738675, EPI_ISL_738676, EPI_ISL_738677, EPI_ISL_738678	Alameda County Public Health Lab	Chan-Zuckerberg Biohub	CZB Cliahub Consortium
EPI_ISL_738707, EPI_ISL_738708	Madera County Department of Public Health	Chan-Zuckerberg Biohub	CZB Cliahub Consortium
EPI_ISL_738709	Orange County Public Health Lab	Chan-Zuckerberg Biohub	CZB Cliahub Consortium
EPI_ISL_738710	Santa Clara County Public Health Laboratory	Chan-Zuckerberg Biohub	CZB Cliahub Consortium
EPI_ISL_738711	Alameda County Public Health Lab	Chan-Zuckerberg Biohub	CZB Cliahub Consortium
EPI_ISL_738712, EPI_ISL_738713, EPI_ISL_738714	Renegade	Chan-Zuckerberg Biohub	CZB Cliahub Consortium
EPI_ISL_738715	Alameda County Public Health Lab	Chan-Zuckerberg Biohub	CZB Cliahub Consortium
EPI_ISL_738716, EPI_ISL_738717, EPI_ISL_738718, EPI_ISL_738719, EPI_ISL_738720	Santa Clara County Public Health Laboratory	Chan-Zuckerberg Biohub	CZB Cliahub Consortium
EPI_ISL_738721	Alameda County Public Health Lab	Chan-Zuckerberg Biohub	CZB Cliahub Consortium
EPI_ISL_738722	Orange County Public Health Lab	Chan-Zuckerberg Biohub	CZB Cliahub Consortium
EPI_ISL_738723, EPI_ISL_738725	Renegade	Chan-Zuckerberg Biohub	CZB Cliahub Consortium
EPI_ISL_738726	Santa Clara County Public Health Laboratory	Chan-Zuckerberg Biohub	CZB Cliahub Consortium
EPI_ISL_738727	Renegade	Chan-Zuckerberg Biohub	CZB Cliahub Consortium
EPI_ISL_738728	Santa Clara County Public Health Laboratory	Chan-Zuckerberg Biohub	CZB Cliahub Consortium
EPI_ISL_738729	Orange County Public Health Lab	Chan-Zuckerberg Biohub	CZB Cliahub Consortium
EPI_ISL_738730	Santa Clara County Public Health Laboratory	Chan-Zuckerberg Biohub	CZB Cliahub Consortium
EPI_ISL_738731, EPI_ISL_738732, EPI_ISL_738733, EPI_ISL_738734, EPI_ISL_738735, EPI_ISL_738736, EPI_ISL_738737, EPI_ISL_738738, EPI_ISL_738739, EPI_ISL_738740, EPI_ISL_738741			
see above	Orange County Public Health Lab	Chan-Zuckerberg Biohub	CZB Cliahub Consortium
EPI_ISL_738742	Humboldt County Public Health Laboratory	Chan-Zuckerberg Biohub	CZB Cliahub Consortium
EPI_ISL_738743, EPI_ISL_738744, EPI_ISL_738745	Alameda County Public Health Lab	Chan-Zuckerberg Biohub	CZB Cliahub Consortium
EPI_ISL_738746, EPI_ISL_738747	Santa Clara County Public Health Laboratory	Chan-Zuckerberg Biohub	CZB Cliahub Consortium
EPI_ISL_738748, EPI_ISL_738749	Alameda County Public Health Lab	Chan-Zuckerberg Biohub	CZB Cliahub Consortium
EPI_ISL_738750	Santa Clara County Public Health Laboratory	Chan-Zuckerberg Biohub	CZB Cliahub Consortium
EPI_ISL_738751	Alameda County Public Health Lab	Chan-Zuckerberg Biohub	CZB Cliahub Consortium
EPI_ISL_738752	Orange County Public Health Lab	Chan-Zuckerberg Biohub	CZB Cliahub Consortium
EPI_ISL_738754	Napa-Solano-Yolo- Marin County (NSYM) Public Health Laboratories	Chan-Zuckerberg Biohub	CZB Cliahub Consortium
EPI_ISL_738755	Orange County Public Health Lab	Chan-Zuckerberg Biohub	CZB Cliahub Consortium
EPI_ISL_738756	Humboldt County Public Health Laboratory	Chan-Zuckerberg Biohub	CZB Cliahub Consortium
EPI_ISL_738757, EPI_ISL_738758	Orange County Public Health Lab	Chan-Zuckerberg Biohub	CZB Cliahub Consortium
EPI_ISL_738759	Humboldt County Public Health Laboratory	Chan-Zuckerberg Biohub	CZB Cliahub Consortium
EPI_ISL_738760	Alameda County Public Health Lab	Chan-Zuckerberg Biohub	CZB Cliahub Consortium
EPI_ISL_738761, EPI_ISL_738762	Orange County Public Health Lab	Chan-Zuckerberg Biohub	CZB Cliahub Consortium
EPI_ISL_738763, EPI_ISL_738764, EPI_ISL_738765	Santa Clara County Public Health Laboratory	Chan-Zuckerberg Biohub	CZB Cliahub Consortium
EPI_ISL_738766, EPI_ISL_738767	Alameda County Public Health Lab	Chan-Zuckerberg Biohub	CZB Cliahub Consortium

EPI_ISL_738769	Santa Clara County Public Health Laboratory	Chan-Zuckerberg Biohub	CZB Cliahub Consortium
EPI_ISL_738770	Renegade	Chan-Zuckerberg Biohub	CZB Cliahub Consortium
EPI_ISL_738771, EPI_ISL_738772	Santa Clara County Public Health Laboratory	Chan-Zuckerberg Biohub	CZB Cliahub Consortium
EPI_ISL_738774, EPI_ISL_738775, EPI_ISL_738776	Alameda County Public Health Lab	Chan-Zuckerberg Biohub	CZB Cliahub Consortium
EPI_ISL_738778, EPI_ISL_738779	Humboldt County Public Health Laboratory	Chan-Zuckerberg Biohub	CZB Cliahub Consortium
EPI_ISL_738780	Santa Clara County Public Health Laboratory	Chan-Zuckerberg Biohub	CZB Cliahub Consortium
EPI_ISL_738781	Alameda County Public Health Lab	Chan-Zuckerberg Biohub	CZB Cliahub Consortium
EPI_ISL_738782	Orange County Public Health Lab	Chan-Zuckerberg Biohub	CZB Cliahub Consortium
EPI_ISL_738783	Alameda County Public Health Lab	Chan-Zuckerberg Biohub	CZB Cliahub Consortium
EPI_ISL_738784	Orange County Public Health Lab	Chan-Zuckerberg Biohub	CZB Cliahub Consortium
EPI_ISL_738785, EPI_ISL_738786	Humboldt County Public Health Laboratory	Chan-Zuckerberg Biohub	CZB Cliahub Consortium
EPI_ISL_738787	Orange County Public Health Lab	Chan-Zuckerberg Biohub	CZB Cliahub Consortium
EPI_ISL_738789	Santa Clara County Public Health Laboratory	Chan-Zuckerberg Biohub	CZB Cliahub Consortium
EPI_ISL_738790	Orange County Public Health Lab	Chan-Zuckerberg Biohub	CZB Cliahub Consortium
EPI_ISL_738791	Santa Clara County Public Health Laboratory	Chan-Zuckerberg Biohub	CZB Cliahub Consortium
EPI_ISL_738792, EPI_ISL_738793	Alameda County Public Health Lab	Chan-Zuckerberg Biohub	CZB Cliahub Consortium
EPI_ISL_738794	Santa Clara County Public Health Laboratory	Chan-Zuckerberg Biohub	CZB Cliahub Consortium
EPI_ISL_738795	Humboldt County Public Health Laboratory	Chan-Zuckerberg Biohub	CZB Cliahub Consortium
EPI_ISL_738796, EPI_ISL_738797	Alameda County Public Health Lab	Chan-Zuckerberg Biohub	CZB Cliahub Consortium
EPI_ISL_738798	Santa Clara County Public Health Laboratory	Chan-Zuckerberg Biohub	CZB Cliahub Consortium
EPI_ISL_738799	Orange County Public Health Lab	Chan-Zuckerberg Biohub	CZB Cliahub Consortium
EPI_ISL_738800	Alameda County Public Health Lab	Chan-Zuckerberg Biohub	CZB Cliahub Consortium
EPI_ISL_738801	Humboldt County Public Health Laboratory	Chan-Zuckerberg Biohub	CZB Cliahub Consortium
EPI_ISL_738802	Alameda County Public Health Lab	Chan-Zuckerberg Biohub	CZB Cliahub Consortium
EPI_ISL_738803	Santa Clara County Public Health Laboratory	Chan-Zuckerberg Biohub	CZB Cliahub Consortium
EPI_ISL_738804	Alameda County Public Health Lab	Chan-Zuckerberg Biohub	CZB Cliahub Consortium
EPI_ISL_738805	Santa Clara County Public Health Laboratory	Chan-Zuckerberg Biohub	CZB Cliahub Consortium
EPI_ISL_738806, EPI_ISL_738807	Humboldt County Public Health Laboratory	Chan-Zuckerberg Biohub	CZB Cliahub Consortium
EPI_ISL_738808	Orange County Public Health Lab	Chan-Zuckerberg Biohub	CZB Cliahub Consortium
EPI_ISL_738809, EPI_ISL_738810	Humboldt County Public Health Laboratory	Chan-Zuckerberg Biohub	CZB Cliahub Consortium
EPI_ISL_738811	Orange County Public Health Lab	Chan-Zuckerberg Biohub	CZB Cliahub Consortium
EPI_ISL_738812	Santa Clara County Public Health Laboratory	Chan-Zuckerberg Biohub	CZB Cliahub Consortium
EPI_ISL_738813, EPI_ISL_738814	Alameda County Public Health Lab	Chan-Zuckerberg Biohub	CZB Cliahub Consortium
EPI_ISL_738815, EPI_ISL_738816	Orange County Public Health Lab	Chan-Zuckerberg Biohub	CZB Cliahub Consortium
EPI_ISL_738817	Alameda County Public Health Lab	Chan-Zuckerberg Biohub	CZB Cliahub Consortium
EPI_ISL_738818	Orange County Public Health Lab	Chan-Zuckerberg Biohub	CZB Cliahub Consortium
EPI_ISL_738819, EPI_ISL_738821, EPI_ISL_738822	Alameda County Public Health Lab	Chan-Zuckerberg Biohub	CZB Cliahub Consortium
EPI_ISL_738823, EPI_ISL_738824	Orange County Public Health Lab	Chan-Zuckerberg Biohub	CZB Cliahub Consortium
EPI_ISL_738825	Alameda County Public Health Lab	Chan-Zuckerberg Biohub	CZB Cliahub Consortium
EPI_ISL_738826	Orange County Public Health Lab	Chan-Zuckerberg Biohub	CZB Cliahub Consortium
EPI_ISL_738827	Humboldt County Public Health Laboratory	Chan-Zuckerberg Biohub	CZB Cliahub Consortium
EPI_ISL_738828	Alameda County Public Health Lab	Chan-Zuckerberg Biohub	CZB Cliahub Consortium
EPI_ISL_738829, EPI_ISL_738830	Orange County Public Health Lab	Chan-Zuckerberg Biohub	CZB Cliahub Consortium
EPI_ISL_738831	Alameda County Public Health Lab	Chan-Zuckerberg Biohub	CZB Cliahub Consortium
EPI_ISL_738832	Humboldt County Public Health Laboratory	Chan-Zuckerberg Biohub	CZB Cliahub Consortium
EPI_ISL_738833	Alameda County Public Health Lab	Chan-Zuckerberg Biohub	CZB Cliahub Consortium
EPI_ISL_738834	Santa Clara County Public Health Laboratory	Chan-Zuckerberg Biohub	CZB Cliahub Consortium
EPI_ISL_738835	Alameda County Public Health Lab	Chan-Zuckerberg Biohub	CZB Cliahub Consortium
EPI_ISL_738836	Napa-Solano-Yolo- Marin County (NSYM) Public Health Laboratories	Chan-Zuckerberg Biohub	CZB Cliahub Consortium
EPI_ISL_738837, EPI_ISL_738838	Alameda County Public Health Lab	Chan-Zuckerberg Biohub	CZB Cliahub Consortium
EPI_ISL_738839	Santa Clara County Public Health Laboratory	Chan-Zuckerberg Biohub	CZB Cliahub Consortium
EPI_ISL_738840, EPI_ISL_738841	Orange County Public Health Lab	Chan-Zuckerberg Biohub	CZB Cliahub Consortium
EPI_ISL_738842	Santa Clara County Public Health Laboratory	Chan-Zuckerberg Biohub	CZB Cliahub Consortium
EPI_ISL_738844	Humboldt County Public Health Laboratory	Chan-Zuckerberg Biohub	CZB Cliahub Consortium
EPI_ISL_738845	Orange County Public Health Lab	Chan-Zuckerberg Biohub	CZB Cliahub Consortium
EPI_ISL_738846, EPI_ISL_738847, EPI_ISL_738848	Alameda County Public Health Lab	Chan-Zuckerberg Biohub	CZB Cliahub Consortium

EPI_ISL_738849	Humboldt County Public Health Laboratory	Chan-Zuckerberg Biohub	CZB Cliahub Consortium
EPI_ISL_738850	Orange County Public Health Lab	Chan-Zuckerberg Biohub	CZB Cliahub Consortium
EPI_ISL_738851	Renegade	Chan-Zuckerberg Biohub	CZB Cliahub Consortium
EPI_ISL_738852	Humboldt County Public Health Laboratory	Chan-Zuckerberg Biohub	CZB Cliahub Consortium
EPI_ISL_738853	Santa Clara County Public Health Laboratory	Chan-Zuckerberg Biohub	CZB Cliahub Consortium
EPI_ISL_738854	Humboldt County Public Health Laboratory	Chan-Zuckerberg Biohub	CZB Cliahub Consortium
EPI_ISL_738855	Santa Clara County Public Health Laboratory	Chan-Zuckerberg Biohub	CZB Cliahub Consortium
EPI_ISL_738856, EPI_ISL_738857	Orange County Public Health Lab	Chan-Zuckerberg Biohub	CZB Cliahub Consortium
EPI_ISL_738859	Santa Clara County Public Health Laboratory	Chan-Zuckerberg Biohub	CZB Cliahub Consortium
EPI_ISL_738860	Orange County Public Health Lab	Chan-Zuckerberg Biohub	CZB Cliahub Consortium
EPI_ISL_738861	Santa Clara County Public Health Laboratory	Chan-Zuckerberg Biohub	CZB Cliahub Consortium
EPI_ISL_738862, EPI_ISL_738863	Orange County Public Health Lab	Chan-Zuckerberg Biohub	CZB Cliahub Consortium
EPI_ISL_738864	Santa Clara County Public Health Laboratory	Chan-Zuckerberg Biohub	CZB Cliahub Consortium
EPI_ISL_738865	Humboldt County Public Health Laboratory	Chan-Zuckerberg Biohub	CZB Cliahub Consortium
EPI_ISL_738866	Orange County Public Health Lab	Chan-Zuckerberg Biohub	CZB Cliahub Consortium
EPI_ISL_738867	Santa Clara County Public Health Laboratory	Chan-Zuckerberg Biohub	CZB Cliahub Consortium
EPI_ISL_738868	Alameda County Public Health Lab	Chan-Zuckerberg Biohub	CZB Cliahub Consortium
EPI_ISL_738869	Orange County Public Health Lab	Chan-Zuckerberg Biohub	CZB Cliahub Consortium
EPI_ISL_738870	Napa-Solano-Yolo- Marin County (NSYM) Public Health Laboratories	Chan-Zuckerberg Biohub	CZB Cliahub Consortium
EPI_ISL_738871, EPI_ISL_738872, EPI_ISL_738873, EPI_ISL_738874	Orange County Public Health Lab	Chan-Zuckerberg Biohub	CZB Cliahub Consortium
EPI_ISL_738875	Humboldt County Public Health Laboratory	Chan-Zuckerberg Biohub	CZB Cliahub Consortium
EPI_ISL_738876	Alameda County Public Health Lab	Chan-Zuckerberg Biohub	CZB Cliahub Consortium
EPI_ISL_738877	Renegade	Chan-Zuckerberg Biohub	CZB Cliahub Consortium
EPI_ISL_738878	Orange County Public Health Lab	Chan-Zuckerberg Biohub	CZB Cliahub Consortium
EPI_ISL_738879	Humboldt County Public Health Laboratory	Chan-Zuckerberg Biohub	CZB Cliahub Consortium
EPI_ISL_738880	Orange County Public Health Lab	Chan-Zuckerberg Biohub	CZB Cliahub Consortium
EPI_ISL_738881	Alameda County Public Health Lab	Chan-Zuckerberg Biohub	CZB Cliahub Consortium
EPI_ISL_738882	Orange County Public Health Lab	Chan-Zuckerberg Biohub	CZB Cliahub Consortium
EPI_ISL_738883	Santa Clara County Public Health Laboratory	Chan-Zuckerberg Biohub	CZB Cliahub Consortium
EPI_ISL_738884, EPI_ISL_738885	Orange County Public Health Lab	Chan-Zuckerberg Biohub	CZB Cliahub Consortium
EPI_ISL_738886	Alameda County Public Health Lab	Chan-Zuckerberg Biohub	CZB Cliahub Consortium
EPI_ISL_738887	Santa Clara County Public Health Laboratory	Chan-Zuckerberg Biohub	CZB Cliahub Consortium
EPI_ISL_738888	Orange County Public Health Lab	Chan-Zuckerberg Biohub	CZB Cliahub Consortium
EPI_ISL_738889	Santa Clara County Public Health Laboratory	Chan-Zuckerberg Biohub	CZB Cliahub Consortium
EPI_ISL_738890	County of San Luis Obispo Public Health Laboratory	Chan-Zuckerberg Biohub	CZB Cliahub Consortium
EPI_ISL_738891, EPI_ISL_738892	Alameda County Public Health Lab	Chan-Zuckerberg Biohub	CZB Cliahub Consortium
EPI_ISL_738893	Orange County Public Health Lab	Chan-Zuckerberg Biohub	CZB Cliahub Consortium
EPI_ISL_738894	Santa Clara County Public Health Laboratory	Chan-Zuckerberg Biohub	CZB Cliahub Consortium
EPI_ISL_738895, EPI_ISL_738896	Alameda County Public Health Lab	Chan-Zuckerberg Biohub	CZB Cliahub Consortium
EPI_ISL_738898	Santa Clara County Public Health Laboratory	Chan-Zuckerberg Biohub	CZB Cliahub Consortium
EPI_ISL_738899, EPI_ISL_738900	Orange County Public Health Lab	Chan-Zuckerberg Biohub	CZB Cliahub Consortium
EPI_ISL_738901	Renegade	Chan-Zuckerberg Biohub	CZB Cliahub Consortium
EPI_ISL_738902	Humboldt County Public Health Laboratory	Chan-Zuckerberg Biohub	CZB Cliahub Consortium
EPI_ISL_738903	Santa Clara County Public Health Laboratory	Chan-Zuckerberg Biohub	CZB Cliahub Consortium
EPI_ISL_738904	Alameda County Public Health Lab	Chan-Zuckerberg Biohub	CZB Cliahub Consortium
EPI_ISL_738905	Orange County Public Health Lab	Chan-Zuckerberg Biohub	CZB Cliahub Consortium
EPI_ISL_738906	Santa Clara County Public Health Laboratory	Chan-Zuckerberg Biohub	CZB Cliahub Consortium
EPI_ISL_738907, EPI_ISL_738908	Orange County Public Health Lab	Chan-Zuckerberg Biohub	CZB Cliahub Consortium
EPI_ISL_738909, EPI_ISL_738910	Santa Clara County Public Health Laboratory	Chan-Zuckerberg Biohub	CZB Cliahub Consortium
EPI_ISL_738911	Napa-Solano-Yolo- Marin County (NSYM) Public Health Laboratories	Chan-Zuckerberg Biohub	CZB Cliahub Consortium
EPI_ISL_738912, EPI_ISL_738913	Humboldt County Public Health Laboratory	Chan-Zuckerberg Biohub	CZB Cliahub Consortium
EPI_ISL_738914	Alameda County Public Health Lab	Chan-Zuckerberg Biohub	CZB Cliahub Consortium
EPI_ISL_738915	Santa Clara County Public Health Laboratory	Chan-Zuckerberg Biohub	CZB Cliahub Consortium
EPI_ISL_738916	Madera County Department of Public Health	Chan-Zuckerberg Biohub	CZB Cliahub Consortium
EPI_ISL_738917, EPI_ISL_738918	Humboldt County Public Health Laboratory	Chan-Zuckerberg Biohub	CZB Cliahub Consortium
EPI_ISL_738919	Alameda County Public Health Lab	Chan-Zuckerberg Biohub	CZB Cliahub Consortium
EPI_ISL_738920	Santa Clara County Public Health Laboratory	Chan-Zuckerberg Biohub	CZB Cliahub Consortium

EPI_ISL_738921	Orange County Public Health Lab	Chan-Zuckerberg Biohub	CZB Cliahub Consortium
EPI_ISL_738922	Santa Clara County Public Health Laboratory	Chan-Zuckerberg Biohub	CZB Cliahub Consortium
EPI_ISL_738923	Humboldt County Public Health Laboratory	Chan-Zuckerberg Biohub	CZB Cliahub Consortium
EPI_ISL_738924	Santa Clara County Public Health Laboratory	Chan-Zuckerberg Biohub	CZB Cliahub Consortium
EPI_ISL_738925	Alameda County Public Health Lab	Chan-Zuckerberg Biohub	CZB Cliahub Consortium
EPI_ISL_738926, EPI_ISL_738927	Santa Clara County Public Health Laboratory	Chan-Zuckerberg Biohub	CZB Cliahub Consortium
EPI_ISL_738928	Orange County Public Health Lab	Chan-Zuckerberg Biohub	CZB Cliahub Consortium
EPI_ISL_738929	Humboldt County Public Health Laboratory	Chan-Zuckerberg Biohub	CZB Cliahub Consortium
EPI_ISL_738930	Orange County Public Health Lab	Chan-Zuckerberg Biohub	CZB Cliahub Consortium
EPI_ISL_738931	Santa Clara County Public Health Laboratory	Chan-Zuckerberg Biohub	CZB Cliahub Consortium
EPI_ISL_738932	Alameda County Public Health Lab	Chan-Zuckerberg Biohub	CZB Cliahub Consortium
EPI_ISL_738933	Santa Clara County Public Health Laboratory	Chan-Zuckerberg Biohub	CZB Cliahub Consortium
EPI_ISL_738934	Alameda County Public Health Lab	Chan-Zuckerberg Biohub	CZB Cliahub Consortium
EPI_ISL_738935	Orange County Public Health Lab	Chan-Zuckerberg Biohub	CZB Cliahub Consortium
EPI_ISL_738936	Santa Clara County Public Health Laboratory	Chan-Zuckerberg Biohub	CZB Cliahub Consortium
EPI_ISL_738937	Orange County Public Health Lab	Chan-Zuckerberg Biohub	CZB Cliahub Consortium
EPI_ISL_738938	Humboldt County Public Health Laboratory	Chan-Zuckerberg Biohub	CZB Cliahub Consortium
EPI_ISL_738939	Orange County Public Health Lab	Chan-Zuckerberg Biohub	CZB Cliahub Consortium
EPI_ISL_738940	Alameda County Public Health Lab	Chan-Zuckerberg Biohub	CZB Cliahub Consortium
EPI_ISL_738941, EPI_ISL_738942, EPI_ISL_738943	Santa Clara County Public Health Laboratory	Chan-Zuckerberg Biohub	CZB Cliahub Consortium
EPI_ISL_738944	Orange County Public Health Lab	Chan-Zuckerberg Biohub	CZB Cliahub Consortium
EPI_ISL_738945	Alameda County Public Health Lab	Chan-Zuckerberg Biohub	CZB Cliahub Consortium
EPI_ISL_738946	Orange County Public Health Lab	Chan-Zuckerberg Biohub	CZB Cliahub Consortium
EPI_ISL_738947	Humboldt County Public Health Laboratory	Chan-Zuckerberg Biohub	CZB Cliahub Consortium
EPI_ISL_738948	Santa Clara County Public Health Laboratory	Chan-Zuckerberg Biohub	CZB Cliahub Consortium
EPI_ISL_738949	Orange County Public Health Lab	Chan-Zuckerberg Biohub	CZB Cliahub Consortium
EPI_ISL_738951	Humboldt County Public Health Laboratory	Chan-Zuckerberg Biohub	CZB Cliahub Consortium
EPI_ISL_738952	Napa-Solano-Yolo- Marin County (NSYM) Public Health Laboratories	Chan-Zuckerberg Biohub	CZB Cliahub Consortium
EPI_ISL_738953	Humboldt County Public Health Laboratory	Chan-Zuckerberg Biohub	CZB Cliahub Consortium
EPI_ISL_738954	Orange County Public Health Lab	Chan-Zuckerberg Biohub	CZB Cliahub Consortium
EPI_ISL_738955, EPI_ISL_738956	Santa Clara County Public Health Laboratory	Chan-Zuckerberg Biohub	CZB Cliahub Consortium
EPI_ISL_738957	Alameda County Public Health Lab	Chan-Zuckerberg Biohub	CZB Cliahub Consortium
EPI_ISL_738958	Humboldt County Public Health Laboratory	Chan-Zuckerberg Biohub	CZB Cliahub Consortium
EPI_ISL_738959	Napa-Solano-Yolo- Marin County (NSYM) Public Health Laboratories	Chan-Zuckerberg Biohub	CZB Cliahub Consortium
EPI_ISL_738960	Santa Clara County Public Health Laboratory	Chan-Zuckerberg Biohub	CZB Cliahub Consortium
EPI_ISL_738961	Humboldt County Public Health Laboratory	Chan-Zuckerberg Biohub	CZB Cliahub Consortium
EPI_ISL_738962	Santa Clara County Public Health Laboratory	Chan-Zuckerberg Biohub	CZB Cliahub Consortium
EPI_ISL_738963	Alameda County Public Health Lab	Chan-Zuckerberg Biohub	CZB Cliahub Consortium
EPI_ISL_738964	Madera County Department of Public Health	Chan-Zuckerberg Biohub	CZB Cliahub Consortium
EPI_ISL_738965	Santa Clara County Public Health Laboratory	Chan-Zuckerberg Biohub	CZB Cliahub Consortium
EPI_ISL_738966	Orange County Public Health Lab	Chan-Zuckerberg Biohub	CZB Cliahub Consortium
EPI_ISL_738967	Santa Clara County Public Health Laboratory	Chan-Zuckerberg Biohub	CZB Cliahub Consortium
EPI_ISL_738968	Orange County Public Health Lab	Chan-Zuckerberg Biohub	CZB Cliahub Consortium
EPI_ISL_738969	Humboldt County Public Health Laboratory	Chan-Zuckerberg Biohub	CZB Cliahub Consortium
EPI_ISL_738970	Orange County Public Health Lab	Chan-Zuckerberg Biohub	CZB Cliahub Consortium
EPI_ISL_738971, EPI_ISL_738972	Santa Clara County Public Health Laboratory	Chan-Zuckerberg Biohub	CZB Cliahub Consortium
EPI_ISL_738973	Orange County Public Health Lab	Chan-Zuckerberg Biohub	CZB Cliahub Consortium
EPI_ISL_738974	Alameda County Public Health Lab	Chan-Zuckerberg Biohub	CZB Cliahub Consortium
EPI_ISL_738975	Orange County Public Health Lab	Chan-Zuckerberg Biohub	CZB Cliahub Consortium
EPI_ISL_738976	Alameda County Public Health Lab	Chan-Zuckerberg Biohub	CZB Cliahub Consortium
EPI_ISL_738977	Renegade	Chan-Zuckerberg Biohub	CZB Cliahub Consortium
EPI_ISL_738978	Orange County Public Health Lab	Chan-Zuckerberg Biohub	CZB Cliahub Consortium
EPI_ISL_738979	Santa Clara County Public Health Laboratory	Chan-Zuckerberg Biohub	CZB Cliahub Consortium
EPI_ISL_738980	Humboldt County Public Health Laboratory	Chan-Zuckerberg Biohub	CZB Cliahub Consortium
EPI_ISL_738981	Orange County Public Health Lab	Chan-Zuckerberg Biohub	CZB Cliahub Consortium
EPI_ISL_738982, EPI_ISL_738983	Alameda County Public Health Lab	Chan-Zuckerberg Biohub	CZB Cliahub Consortium

EPI_ISL_738984	Santa Clara County Public Health Laboratory	Chan-Zuckerberg Biohub	CZB Cliahub Consortium
EPI_ISL_738985	Humboldt County Public Health Laboratory	Chan-Zuckerberg Biohub	CZB Cliahub Consortium
EPI_ISL_738987	Santa Clara County Public Health Laboratory	Chan-Zuckerberg Biohub	CZB Cliahub Consortium
EPI_ISL_738988	Humboldt County Public Health Laboratory	Chan-Zuckerberg Biohub	CZB Cliahub Consortium
EPI_ISL_738989	Orange County Public Health Lab	Chan-Zuckerberg Biohub	CZB Cliahub Consortium
EPI_ISL_738990	County of San Luis Obispo Public Health Laboratory	Chan-Zuckerberg Biohub	CZB Cliahub Consortium
EPI_ISL_738991	Orange County Public Health Lab	Chan-Zuckerberg Biohub	CZB Cliahub Consortium
EPI_ISL_738992	Alameda County Public Health Lab	Chan-Zuckerberg Biohub	CZB Cliahub Consortium
EPI_ISL_738993	Orange County Public Health Lab	Chan-Zuckerberg Biohub	CZB Cliahub Consortium
EPI_ISL_738994	Santa Clara County Public Health Laboratory	Chan-Zuckerberg Biohub	CZB Cliahub Consortium
EPI_ISL_738995	Orange County Public Health Lab	Chan-Zuckerberg Biohub	CZB Cliahub Consortium
EPI_ISL_738996, EPI_ISL_738997, EPI_ISL_738998	Santa Clara County Public Health Laboratory	Chan-Zuckerberg Biohub	CZB Cliahub Consortium
EPI_ISL_738999, EPI_ISL_739000, EPI_ISL_739001	Humboldt County Public Health Laboratory	Chan-Zuckerberg Biohub	CZB Cliahub Consortium
EPI_ISL_739002	Orange County Public Health Lab	Chan-Zuckerberg Biohub	CZB Cliahub Consortium
EPI_ISL_739004	UCSF Clinical Microbiology Laboratory	Chan-Zuckerberg Biohub	CZB Cliahub Consortium
EPI_ISL_739005, EPI_ISL_739006	Santa Clara County Public Health Laboratory	Chan-Zuckerberg Biohub	CZB Cliahub Consortium
EPI_ISL_739007	Humboldt County Public Health Laboratory	Chan-Zuckerberg Biohub	CZB Cliahub Consortium
EPI_ISL_739008, EPI_ISL_739009, EPI_ISL_739010	Santa Clara County Public Health Laboratory	Chan-Zuckerberg Biohub	CZB Cliahub Consortium
EPI_ISL_739011	Humboldt County Public Health Laboratory	Chan-Zuckerberg Biohub	CZB Cliahub Consortium
EPI_ISL_739012	Santa Clara County Public Health Laboratory	Chan-Zuckerberg Biohub	CZB Cliahub Consortium
EPI_ISL_739013	Orange County Public Health Lab	Chan-Zuckerberg Biohub	CZB Cliahub Consortium
EPI_ISL_739014	Renegade	Chan-Zuckerberg Biohub	CZB Cliahub Consortium
EPI_ISL_739015	Santa Clara County Public Health Laboratory	Chan-Zuckerberg Biohub	CZB Cliahub Consortium
EPI_ISL_739016	Orange County Public Health Lab	Chan-Zuckerberg Biohub	CZB Cliahub Consortium
EPI_ISL_739017	County of San Luis Obispo Public Health Laboratory	Chan-Zuckerberg Biohub	CZB Cliahub Consortium
EPI_ISL_739018	Alameda County Public Health Lab	Chan-Zuckerberg Biohub	CZB Cliahub Consortium
EPI_ISL_739019, EPI_ISL_739020, EPI_ISL_739021	Santa Clara County Public Health Laboratory	Chan-Zuckerberg Biohub	CZB Cliahub Consortium
EPI_ISL_739022	Renegade	Chan-Zuckerberg Biohub	CZB Cliahub Consortium
EPI_ISL_739023	Alameda County Public Health Lab	Chan-Zuckerberg Biohub	CZB Cliahub Consortium
EPI_ISL_739025, EPI_ISL_739026, EPI_ISL_739027	Santa Clara County Public Health Laboratory	Chan-Zuckerberg Biohub	CZB Cliahub Consortium
EPI_ISL_739028	Orange County Public Health Lab	Chan-Zuckerberg Biohub	CZB Cliahub Consortium
EPI_ISL_739030, EPI_ISL_739031	Santa Clara County Public Health Laboratory	Chan-Zuckerberg Biohub	CZB Cliahub Consortium
EPI_ISL_739032	Humboldt County Public Health Laboratory	Chan-Zuckerberg Biohub	CZB Cliahub Consortium
EPI_ISL_739033	Alameda County Public Health Lab	Chan-Zuckerberg Biohub	CZB Cliahub Consortium
EPI_ISL_739034	Orange County Public Health Lab	Chan-Zuckerberg Biohub	CZB Cliahub Consortium
EPI_ISL_739035	Alameda County Public Health Lab	Chan-Zuckerberg Biohub	CZB Cliahub Consortium
EPI_ISL_739036, EPI_ISL_739037	Santa Clara County Public Health Laboratory	Chan-Zuckerberg Biohub	CZB Cliahub Consortium
EPI_ISL_739038	County of San Luis Obispo Public Health Laboratory	Chan-Zuckerberg Biohub	CZB Cliahub Consortium
EPI_ISL_739039, EPI_ISL_739040, EPI_ISL_739041	Santa Clara County Public Health Laboratory	Chan-Zuckerberg Biohub	CZB Cliahub Consortium
EPI_ISL_739042	Orange County Public Health Lab	Chan-Zuckerberg Biohub	CZB Cliahub Consortium
EPI_ISL_739043	Humboldt County Public Health Laboratory	Chan-Zuckerberg Biohub	CZB Cliahub Consortium
EPI_ISL_739044	Orange County Public Health Lab	Chan-Zuckerberg Biohub	CZB Cliahub Consortium
EPI_ISL_739045	Santa Clara County Public Health Laboratory	Chan-Zuckerberg Biohub	CZB Cliahub Consortium
EPI_ISL_739046, EPI_ISL_739047	Alameda County Public Health Lab	Chan-Zuckerberg Biohub	CZB Cliahub Consortium
EPI_ISL_739048	Orange County Public Health Lab	Chan-Zuckerberg Biohub	CZB Cliahub Consortium
EPI_ISL_739049	Alameda County Public Health Lab	Chan-Zuckerberg Biohub	CZB Cliahub Consortium
EPI_ISL_739050	Santa Clara County Public Health Laboratory	Chan-Zuckerberg Biohub	CZB Cliahub Consortium
EPI_ISL_739051, EPI_ISL_739052	Humboldt County Public Health Laboratory	Chan-Zuckerberg Biohub	CZB Cliahub Consortium
EPI_ISL_739053	Santa Clara County Public Health Laboratory	Chan-Zuckerberg Biohub	CZB Cliahub Consortium
EPI_ISL_739054	Alameda County Public Health Lab	Chan-Zuckerberg Biohub	CZB Cliahub Consortium
EPI_ISL_739055	Orange County Public Health Lab	Chan-Zuckerberg Biohub	CZB Cliahub Consortium
EPI_ISL_739056, EPI_ISL_739057	Alameda County Public Health Lab	Chan-Zuckerberg Biohub	CZB Cliahub Consortium
EPI_ISL_739058	Santa Clara County Public Health Laboratory	Chan-Zuckerberg Biohub	CZB Cliahub Consortium
EPI_ISL_739059	Alameda County Public Health Lab	Chan-Zuckerberg Biohub	CZB Cliahub Consortium
EPI_ISL_739060	Santa Clara County Public Health Laboratory	Chan-Zuckerberg Biohub	CZB Cliahub Consortium

EPI_ISL_739061	Madera County Department of Public Health	Chan-Zuckerberg Biohub	CZB Cliahub Consortium
EPI_ISL_739062	Alameda County Public Health Lab	Chan-Zuckerberg Biohub	CZB Cliahub Consortium
EPI_ISL_739063	Orange County Public Health Lab	Chan-Zuckerberg Biohub	CZB Cliahub Consortium
EPI_ISL_739064	Alameda County Public Health Lab	Chan-Zuckerberg Biohub	CZB Cliahub Consortium
EPI_ISL_739065, EPI_ISL_739066, EPI_ISL_739067, EPI_ISL_739068	Santa Clara County Public Health Laboratory	Chan-Zuckerberg Biohub	CZB Cliahub Consortium
EPI_ISL_739069	Orange County Public Health Lab	Chan-Zuckerberg Biohub	CZB Cliahub Consortium
EPI_ISL_739070	Santa Clara County Public Health Laboratory	Chan-Zuckerberg Biohub	CZB Cliahub Consortium
EPI_ISL_739071, EPI_ISL_739072	Orange County Public Health Lab	Chan-Zuckerberg Biohub	CZB Cliahub Consortium
EPI_ISL_739073	Alameda County Public Health Lab	Chan-Zuckerberg Biohub	CZB Cliahub Consortium
EPI_ISL_739074	Santa Clara County Public Health Laboratory	Chan-Zuckerberg Biohub	CZB Cliahub Consortium
EPI_ISL_739075	Alameda County Public Health Lab	Chan-Zuckerberg Biohub	CZB Cliahub Consortium
EPI_ISL_739076	Santa Clara County Public Health Laboratory	Chan-Zuckerberg Biohub	CZB Cliahub Consortium
EPI_ISL_739077	Alameda County Public Health Lab	Chan-Zuckerberg Biohub	CZB Cliahub Consortium
EPI_ISL_739078	Orange County Public Health Lab	Chan-Zuckerberg Biohub	CZB Cliahub Consortium
EPI_ISL_739079	Humboldt County Public Health Laboratory	Chan-Zuckerberg Biohub	CZB Cliahub Consortium
EPI_ISL_739080	Santa Clara County Public Health Laboratory	Chan-Zuckerberg Biohub	CZB Cliahub Consortium
EPI_ISL_739081	Orange County Public Health Lab	Chan-Zuckerberg Biohub	CZB Cliahub Consortium
EPI_ISL_739082, EPI_ISL_739083, EPI_ISL_739084	Alameda County Public Health Lab	Chan-Zuckerberg Biohub	CZB Cliahub Consortium
EPI_ISL_739085	Santa Clara County Public Health Laboratory	Chan-Zuckerberg Biohub	CZB Cliahub Consortium
EPI_ISL_739086	Humboldt County Public Health Laboratory	Chan-Zuckerberg Biohub	CZB Cliahub Consortium
EPI_ISL_739087	Santa Clara County Public Health Laboratory	Chan-Zuckerberg Biohub	CZB Cliahub Consortium
EPI_ISL_739088	Alameda County Public Health Lab	Chan-Zuckerberg Biohub	CZB Cliahub Consortium
EPI_ISL_739089	Orange County Public Health Lab	Chan-Zuckerberg Biohub	CZB Cliahub Consortium
EPI_ISL_739090	Alameda County Public Health Lab	Chan-Zuckerberg Biohub	CZB Cliahub Consortium
EPI_ISL_739091	Santa Clara County Public Health Laboratory	Chan-Zuckerberg Biohub	CZB Cliahub Consortium
EPI_ISL_739092	Orange County Public Health Lab	Chan-Zuckerberg Biohub	CZB Cliahub Consortium
EPI_ISL_739093	Alameda County Public Health Lab	Chan-Zuckerberg Biohub	CZB Cliahub Consortium
EPI_ISL_739094	Orange County Public Health Lab	Chan-Zuckerberg Biohub	CZB Cliahub Consortium
EPI_ISL_739095	County of San Luis Obispo Public Health Laboratory	Chan-Zuckerberg Biohub	CZB Cliahub Consortium
EPI_ISL_739096	Alameda County Public Health Lab	Chan-Zuckerberg Biohub	CZB Cliahub Consortium
EPI_ISL_739097	Orange County Public Health Lab	Chan-Zuckerberg Biohub	CZB Cliahub Consortium
EPI_ISL_739098	Santa Clara County Public Health Laboratory	Chan-Zuckerberg Biohub	CZB Cliahub Consortium
EPI_ISL_739099	Orange County Public Health Lab	Chan-Zuckerberg Biohub	CZB Cliahub Consortium
EPI_ISL_739100	Alameda County Public Health Lab	Chan-Zuckerberg Biohub	CZB Cliahub Consortium
EPI_ISL_739101	Monterey County Public Health Lab	Chan-Zuckerberg Biohub	CZB Cliahub Consortium
EPI_ISL_739102	County of San Luis Obispo Public Health Laboratory	Chan-Zuckerberg Biohub	CZB Cliahub Consortium
EPI_ISL_739103, EPI_ISL_739104	Santa Clara County Public Health Laboratory	Chan-Zuckerberg Biohub	CZB Cliahub Consortium
EPI_ISL_739105	Orange County Public Health Lab	Chan-Zuckerberg Biohub	CZB Cliahub Consortium
EPI_ISL_739106	Humboldt County Public Health Laboratory	Chan-Zuckerberg Biohub	CZB Cliahub Consortium
EPI_ISL_739107	Santa Clara County Public Health Laboratory	Chan-Zuckerberg Biohub	CZB Cliahub Consortium
EPI_ISL_739108	Orange County Public Health Lab	Chan-Zuckerberg Biohub	CZB Cliahub Consortium
EPI_ISL_739109, EPI_ISL_739110, EPI_ISL_739111	Alameda County Public Health Lab	Chan-Zuckerberg Biohub	CZB Cliahub Consortium
EPI_ISL_739112	Humboldt County Public Health Laboratory	Chan-Zuckerberg Biohub	CZB Cliahub Consortium
EPI_ISL_739113	Alameda County Public Health Lab	Chan-Zuckerberg Biohub	CZB Cliahub Consortium
EPI_ISL_739114	Humboldt County Public Health Laboratory	Chan-Zuckerberg Biohub	CZB Cliahub Consortium
EPI_ISL_739115	Alameda County Public Health Lab	Chan-Zuckerberg Biohub	CZB Cliahub Consortium
EPI_ISL_739116	Orange County Public Health Lab	Chan-Zuckerberg Biohub	CZB Cliahub Consortium
EPI_ISL_739117	Santa Clara County Public Health Laboratory	Chan-Zuckerberg Biohub	CZB Cliahub Consortium
EPI_ISL_739118	Alameda County Public Health Lab	Chan-Zuckerberg Biohub	CZB Cliahub Consortium
EPI_ISL_739119	Tulare County Public Health Lab	Chan-Zuckerberg Biohub	CZB Cliahub Consortium
EPI_ISL_739120	Alameda County Public Health Lab	Chan-Zuckerberg Biohub	CZB Cliahub Consortium
EPI_ISL_739121, EPI_ISL_739122	Santa Clara County Public Health Laboratory	Chan-Zuckerberg Biohub	CZB Cliahub Consortium
EPI_ISL_739123	Humboldt County Public Health Laboratory	Chan-Zuckerberg Biohub	CZB Cliahub Consortium
EPI_ISL_739124	Alameda County Public Health Lab	Chan-Zuckerberg Biohub	CZB Cliahub Consortium
EPI_ISL_739125	Orange County Public Health Lab	Chan-Zuckerberg Biohub	CZB Cliahub Consortium
EPI_ISL_739126	Humboldt County Public Health Laboratory	Chan-Zuckerberg Biohub	CZB Cliahub Consortium

EPI_ISL_739127	Alameda County Public Health Lab	Chan-Zuckerberg Biohub	CZB Cliahub Consortium
EPI_ISL_739128, EPI_ISL_739129, EPI_ISL_739130	Santa Clara County Public Health Laboratory	Chan-Zuckerberg Biohub	CZB Cliahub Consortium
EPI_ISL_739131	Humboldt County Public Health Laboratory	Chan-Zuckerberg Biohub	CZB Cliahub Consortium
EPI_ISL_739132	Orange County Public Health Lab	Chan-Zuckerberg Biohub	CZB Cliahub Consortium
EPI_ISL_739133	Madera County Department of Public Health	Chan-Zuckerberg Biohub	CZB Cliahub Consortium
EPI_ISL_739134, EPI_ISL_739135, EPI_ISL_739136, EPI_ISL_739137	Alameda County Public Health Lab	Chan-Zuckerberg Biohub	CZB Cliahub Consortium
EPI_ISL_739138	Santa Clara County Public Health Laboratory	Chan-Zuckerberg Biohub	CZB Cliahub Consortium
EPI_ISL_739139	Orange County Public Health Lab	Chan-Zuckerberg Biohub	CZB Cliahub Consortium
EPI_ISL_739140	Alameda County Public Health Lab	Chan-Zuckerberg Biohub	CZB Cliahub Consortium
EPI_ISL_739141	Santa Clara County Public Health Laboratory	Chan-Zuckerberg Biohub	CZB Cliahub Consortium
EPI_ISL_739142, EPI_ISL_739143	Orange County Public Health Lab	Chan-Zuckerberg Biohub	CZB Cliahub Consortium
EPI_ISL_739144	Alameda County Public Health Lab	Chan-Zuckerberg Biohub	CZB Cliahub Consortium
EPI_ISL_739145, EPI_ISL_739146, EPI_ISL_739147	Santa Clara County Public Health Laboratory	Chan-Zuckerberg Biohub	CZB Cliahub Consortium
EPI_ISL_739148	Humboldt County Public Health Laboratory	Chan-Zuckerberg Biohub	CZB Cliahub Consortium
EPI_ISL_739149, EPI_ISL_739150	Alameda County Public Health Lab	Chan-Zuckerberg Biohub	CZB Cliahub Consortium
EPI_ISL_739151	Orange County Public Health Lab	Chan-Zuckerberg Biohub	CZB Cliahub Consortium
EPI_ISL_739152, EPI_ISL_739153	Santa Clara County Public Health Laboratory	Chan-Zuckerberg Biohub	CZB Cliahub Consortium
EPI_ISL_739154	Orange County Public Health Lab	Chan-Zuckerberg Biohub	CZB Cliahub Consortium
EPI_ISL_739155	Humboldt County Public Health Laboratory	Chan-Zuckerberg Biohub	CZB Cliahub Consortium
EPI_ISL_739156	Orange County Public Health Lab	Chan-Zuckerberg Biohub	CZB Cliahub Consortium
EPI_ISL_739157	Santa Clara County Public Health Laboratory	Chan-Zuckerberg Biohub	CZB Cliahub Consortium
EPI_ISL_739158	Alameda County Public Health Lab	Chan-Zuckerberg Biohub	CZB Cliahub Consortium
EPI_ISL_739159	Humboldt County Public Health Laboratory	Chan-Zuckerberg Biohub	CZB Cliahub Consortium
EPI_ISL_739160	Alameda County Public Health Lab	Chan-Zuckerberg Biohub	CZB Cliahub Consortium
EPI_ISL_739162	Orange County Public Health Lab	Chan-Zuckerberg Biohub	CZB Cliahub Consortium
EPI_ISL_739163, EPI_ISL_739164	Alameda County Public Health Lab	Chan-Zuckerberg Biohub	CZB Cliahub Consortium
EPI_ISL_739165	Humboldt County Public Health Laboratory	Chan-Zuckerberg Biohub	CZB Cliahub Consortium
EPI_ISL_739166, EPI_ISL_739167, EPI_ISL_739168	Santa Clara County Public Health Laboratory	Chan-Zuckerberg Biohub	CZB Cliahub Consortium
EPI_ISL_739169	Napa-Solano-Yolo- Marin County (NSYM) Public Health Laboratories	Chan-Zuckerberg Biohub	CZB Cliahub Consortium
EPI_ISL_739170	Humboldt County Public Health Laboratory	Chan-Zuckerberg Biohub	CZB Cliahub Consortium
EPI_ISL_739172	Madera County Department of Public Health	Chan-Zuckerberg Biohub	CZB Cliahub Consortium
EPI_ISL_739173	Alameda County Public Health Lab	Chan-Zuckerberg Biohub	CZB Cliahub Consortium
EPI_ISL_739175, EPI_ISL_739176	Orange County Public Health Lab	Chan-Zuckerberg Biohub	CZB Cliahub Consortium
EPI_ISL_739177	Humboldt County Public Health Laboratory	Chan-Zuckerberg Biohub	CZB Cliahub Consortium
EPI_ISL_739179	Alameda County Public Health Lab	Chan-Zuckerberg Biohub	CZB Cliahub Consortium
EPI_ISL_739180	Santa Clara County Public Health Laboratory	Chan-Zuckerberg Biohub	CZB Cliahub Consortium
EPI_ISL_739181	Napa-Solano-Yolo- Marin County (NSYM) Public Health Laboratories	Chan-Zuckerberg Biohub	CZB Cliahub Consortium
EPI_ISL_739182	Orange County Public Health Lab	Chan-Zuckerberg Biohub	CZB Cliahub Consortium
EPI_ISL_739183	Humboldt County Public Health Laboratory	Chan-Zuckerberg Biohub	CZB Cliahub Consortium
EPI_ISL_739184	Santa Clara County Public Health Laboratory	Chan-Zuckerberg Biohub	CZB Cliahub Consortium
EPI_ISL_739185	Orange County Public Health Lab	Chan-Zuckerberg Biohub	CZB Cliahub Consortium
EPI_ISL_739186, EPI_ISL_739187, EPI_ISL_739188	Santa Clara County Public Health Laboratory	Chan-Zuckerberg Biohub	CZB Cliahub Consortium
EPI_ISL_739189, EPI_ISL_739190	Alameda County Public Health Lab	Chan-Zuckerberg Biohub	CZB Cliahub Consortium
EPI_ISL_739191	Orange County Public Health Lab	Chan-Zuckerberg Biohub	CZB Cliahub Consortium
EPI_ISL_739193	Tulare County Public Health Lab	Chan-Zuckerberg Biohub	CZB Cliahub Consortium
EPI_ISL_739194	Humboldt County Public Health Laboratory	Chan-Zuckerberg Biohub	CZB Cliahub Consortium
EPI_ISL_739195	Santa Clara County Public Health Laboratory	Chan-Zuckerberg Biohub	CZB Cliahub Consortium
EPI_ISL_739197	Madera County Department of Public Health	Chan-Zuckerberg Biohub	CZB Cliahub Consortium
EPI_ISL_739198	Humboldt County Public Health Laboratory	Chan-Zuckerberg Biohub	CZB Cliahub Consortium
EPI_ISL_739199	Orange County Public Health Lab	Chan-Zuckerberg Biohub	CZB Cliahub Consortium
EPI_ISL_739200, EPI_ISL_739201, EPI_ISL_739202	Alameda County Public Health Lab	Chan-Zuckerberg Biohub	CZB Cliahub Consortium
EPI_ISL_739203	UCSF Clinical Microbiology Laboratory	Chan-Zuckerberg Biohub	CZB Cliahub Consortium
EPI_ISL_739204	Santa Clara County Public Health Laboratory	Chan-Zuckerberg Biohub	CZB Cliahub Consortium

EPI_ISL_739205	Orange County Public Health Lab	Chan-Zuckerberg Biohub	CZB Cliahub Consortium
EPI_ISL_739206, EPI_ISL_739208, EPI_ISL_739209	Alameda County Public Health Lab	Chan-Zuckerberg Biohub	CZB Cliahub Consortium
EPI_ISL_739210	Humboldt County Public Health Laboratory	Chan-Zuckerberg Biohub	CZB Cliahub Consortium
EPI_ISL_739211	Orange County Public Health Lab	Chan-Zuckerberg Biohub	CZB Cliahub Consortium
EPI_ISL_739212, EPI_ISL_739213	Alameda County Public Health Lab	Chan-Zuckerberg Biohub	CZB Cliahub Consortium
EPI_ISL_739214	Santa Clara County Public Health Laboratory	Chan-Zuckerberg Biohub	CZB Cliahub Consortium
EPI_ISL_739215	Alameda County Public Health Lab	Chan-Zuckerberg Biohub	CZB Cliahub Consortium
EPI_ISL_739216	Orange County Public Health Lab	Chan-Zuckerberg Biohub	CZB Cliahub Consortium
EPI_ISL_739217, EPI_ISL_739218	Alameda County Public Health Lab	Chan-Zuckerberg Biohub	CZB Cliahub Consortium
EPI_ISL_739219	Santa Clara County Public Health Laboratory	Chan-Zuckerberg Biohub	CZB Cliahub Consortium
EPI_ISL_739220	Alameda County Public Health Lab	Chan-Zuckerberg Biohub	CZB Cliahub Consortium
EPI_ISL_739221	Santa Clara County Public Health Laboratory	Chan-Zuckerberg Biohub	CZB Cliahub Consortium
EPI_ISL_739222	Orange County Public Health Lab	Chan-Zuckerberg Biohub	CZB Cliahub Consortium
EPI_ISL_739223	Napa-Solano-Yolo- Marin County (NSYM) Public Health Laboratories	Chan-Zuckerberg Biohub	CZB Cliahub Consortium
EPI_ISL_739224	Santa Clara County Public Health Laboratory	Chan-Zuckerberg Biohub	CZB Cliahub Consortium
EPI_ISL_739225	Humboldt County Public Health Laboratory	Chan-Zuckerberg Biohub	CZB Cliahub Consortium
EPI_ISL_739226	Alameda County Public Health Lab	Chan-Zuckerberg Biohub	CZB Cliahub Consortium
EPI_ISL_739227	Orange County Public Health Lab	Chan-Zuckerberg Biohub	CZB Cliahub Consortium
EPI_ISL_739228	Humboldt County Public Health Laboratory	Chan-Zuckerberg Biohub	CZB Cliahub Consortium
EPI_ISL_739230	Orange County Public Health Lab	Chan-Zuckerberg Biohub	CZB Cliahub Consortium
EPI_ISL_739231	Alameda County Public Health Lab	Chan-Zuckerberg Biohub	CZB Cliahub Consortium
EPI_ISL_739232	Santa Clara County Public Health Laboratory	Chan-Zuckerberg Biohub	CZB Cliahub Consortium
EPI_ISL_739233	Alameda County Public Health Lab	Chan-Zuckerberg Biohub	CZB Cliahub Consortium
EPI_ISL_739234	Santa Clara County Public Health Laboratory	Chan-Zuckerberg Biohub	CZB Cliahub Consortium
EPI_ISL_739235	Humboldt County Public Health Laboratory	Chan-Zuckerberg Biohub	CZB Cliahub Consortium
EPI_ISL_739236	Alameda County Public Health Lab	Chan-Zuckerberg Biohub	CZB Cliahub Consortium
EPI_ISL_739237	Santa Clara County Public Health Laboratory	Chan-Zuckerberg Biohub	CZB Cliahub Consortium
EPI_ISL_739238, EPI_ISL_739239	Orange County Public Health Lab	Chan-Zuckerberg Biohub	CZB Cliahub Consortium
EPI_ISL_739240	Humboldt County Public Health Laboratory	Chan-Zuckerberg Biohub	CZB Cliahub Consortium
EPI_ISL_739241	Alameda County Public Health Lab	Chan-Zuckerberg Biohub	CZB Cliahub Consortium
EPI_ISL_739242	Humboldt County Public Health Laboratory	Chan-Zuckerberg Biohub	CZB Cliahub Consortium
EPI_ISL_739243, EPI_ISL_739244	Alameda County Public Health Lab	Chan-Zuckerberg Biohub	CZB Cliahub Consortium
EPI_ISL_739245	Orange County Public Health Lab	Chan-Zuckerberg Biohub	CZB Cliahub Consortium
EPI_ISL_739246, EPI_ISL_739247, EPI_ISL_739248	Santa Clara County Public Health Laboratory	Chan-Zuckerberg Biohub	CZB Cliahub Consortium
EPI_ISL_739250	Humboldt County Public Health Laboratory	Chan-Zuckerberg Biohub	CZB Cliahub Consortium
EPI_ISL_739252, EPI_ISL_739253, EPI_ISL_739254	Santa Clara County Public Health Laboratory	Chan-Zuckerberg Biohub	CZB Cliahub Consortium
EPI_ISL_739255	Orange County Public Health Lab	Chan-Zuckerberg Biohub	CZB Cliahub Consortium
EPI_ISL_739256	Santa Clara County Public Health Laboratory	Chan-Zuckerberg Biohub	CZB Cliahub Consortium
EPI_ISL_739257	Alameda County Public Health Lab	Chan-Zuckerberg Biohub	CZB Cliahub Consortium
EPI_ISL_739258, EPI_ISL_739259	Orange County Public Health Lab	Chan-Zuckerberg Biohub	CZB Cliahub Consortium
EPI_ISL_739260	Santa Clara County Public Health Laboratory	Chan-Zuckerberg Biohub	CZB Cliahub Consortium
EPI_ISL_739261, EPI_ISL_739262	Alameda County Public Health Lab	Chan-Zuckerberg Biohub	CZB Cliahub Consortium
EPI_ISL_739263	Santa Clara County Public Health Laboratory	Chan-Zuckerberg Biohub	CZB Cliahub Consortium
EPI_ISL_739264	Humboldt County Public Health Laboratory	Chan-Zuckerberg Biohub	CZB Cliahub Consortium
EPI_ISL_739265, EPI_ISL_739266, EPI_ISL_739267	Orange County Public Health Lab	Chan-Zuckerberg Biohub	CZB Cliahub Consortium
EPI_ISL_739269	Humboldt County Public Health Laboratory	Chan-Zuckerberg Biohub	CZB Cliahub Consortium
EPI_ISL_739270	Alameda County Public Health Lab	Chan-Zuckerberg Biohub	CZB Cliahub Consortium
EPI_ISL_739271	Orange County Public Health Lab	Chan-Zuckerberg Biohub	CZB Cliahub Consortium
EPI_ISL_739272	Humboldt County Public Health Laboratory	Chan-Zuckerberg Biohub	CZB Cliahub Consortium
EPI_ISL_739273	Santa Clara County Public Health Laboratory	Chan-Zuckerberg Biohub	CZB Cliahub Consortium
EPI_ISL_739274	Humboldt County Public Health Laboratory	Chan-Zuckerberg Biohub	CZB Cliahub Consortium
EPI_ISL_739275	Santa Clara County Public Health Laboratory	Chan-Zuckerberg Biohub	CZB Cliahub Consortium
EPI_ISL_739276	Orange County Public Health Lab	Chan-Zuckerberg Biohub	CZB Cliahub Consortium
EPI_ISL_739277, EPI_ISL_739278	Santa Clara County Public Health Laboratory	Chan-Zuckerberg Biohub	CZB Cliahub Consortium
EPI_ISL_739279	Orange County Public Health Lab	Chan-Zuckerberg Biohub	CZB Cliahub Consortium

EPI_ISL_739280	Alameda County Public Health Lab	Chan-Zuckerberg Biohub	CZB Cliahub Consortium
EPI_ISL_739281, EPI_ISL_739282	Santa Clara County Public Health Laboratory	Chan-Zuckerberg Biohub	CZB Cliahub Consortium
EPI_ISL_739283	Alameda County Public Health Lab	Chan-Zuckerberg Biohub	CZB Cliahub Consortium
EPI_ISL_739284	Santa Clara County Public Health Laboratory	Chan-Zuckerberg Biohub	CZB Cliahub Consortium
EPI_ISL_739285	Alameda County Public Health Lab	Chan-Zuckerberg Biohub	CZB Cliahub Consortium
EPI_ISL_739287	Humboldt County Public Health Laboratory	Chan-Zuckerberg Biohub	CZB Cliahub Consortium
EPI_ISL_739288	Alameda County Public Health Lab	Chan-Zuckerberg Biohub	CZB Cliahub Consortium
EPI_ISL_739289	Orange County Public Health Lab	Chan-Zuckerberg Biohub	CZB Cliahub Consortium
EPI_ISL_739290	Alameda County Public Health Lab	Chan-Zuckerberg Biohub	CZB Cliahub Consortium
EPI_ISL_739291	Orange County Public Health Lab	Chan-Zuckerberg Biohub	CZB Cliahub Consortium
EPI_ISL_739292	Humboldt County Public Health Laboratory	Chan-Zuckerberg Biohub	CZB Cliahub Consortium
EPI_ISL_739293	Santa Clara County Public Health Laboratory	Chan-Zuckerberg Biohub	CZB Cliahub Consortium
EPI_ISL_739294	Alameda County Public Health Lab	Chan-Zuckerberg Biohub	CZB Cliahub Consortium
EPI_ISL_739295	UCSF Clinical Microbiology Laboratory	Chan-Zuckerberg Biohub	CZB Cliahub Consortium
EPI_ISL_739296	Orange County Public Health Lab	Chan-Zuckerberg Biohub	CZB Cliahub Consortium
EPI_ISL_739297	Humboldt County Public Health Laboratory	Chan-Zuckerberg Biohub	CZB Cliahub Consortium
EPI_ISL_739298	Santa Clara County Public Health Laboratory	Chan-Zuckerberg Biohub	CZB Cliahub Consortium
EPI_ISL_739299	Humboldt County Public Health Laboratory	Chan-Zuckerberg Biohub	CZB Cliahub Consortium
EPI_ISL_739300, EPI_ISL_739302, EPI_ISL_739303	Alameda County Public Health Lab	Chan-Zuckerberg Biohub	CZB Cliahub Consortium
EPI_ISL_739304	Orange County Public Health Lab	Chan-Zuckerberg Biohub	CZB Cliahub Consortium
EPI_ISL_739305	Santa Clara County Public Health Laboratory	Chan-Zuckerberg Biohub	CZB Cliahub Consortium
EPI_ISL_739307, EPI_ISL_739308, EPI_ISL_739309	Alameda County Public Health Lab	Chan-Zuckerberg Biohub	CZB Cliahub Consortium
EPI_ISL_739310	Santa Clara County Public Health Laboratory	Chan-Zuckerberg Biohub	CZB Cliahub Consortium
EPI_ISL_739311	Humboldt County Public Health Laboratory	Chan-Zuckerberg Biohub	CZB Cliahub Consortium
EPI_ISL_739312	Alameda County Public Health Lab	Chan-Zuckerberg Biohub	CZB Cliahub Consortium
EPI_ISL_739313	Humboldt County Public Health Laboratory	Chan-Zuckerberg Biohub	CZB Cliahub Consortium
EPI_ISL_739314	Orange County Public Health Lab	Chan-Zuckerberg Biohub	CZB Cliahub Consortium
EPI_ISL_739315	Napa-Solano-Yolo- Marin County (NSYM) Public Health Laboratories	Chan-Zuckerberg Biohub	CZB Cliahub Consortium
EPI_ISL_739316	Renegade	Chan-Zuckerberg Biohub	CZB Cliahub Consortium
EPI_ISL_739317	Santa Clara County Public Health Laboratory	Chan-Zuckerberg Biohub	CZB Cliahub Consortium
EPI_ISL_739318	Humboldt County Public Health Laboratory	Chan-Zuckerberg Biohub	CZB Cliahub Consortium
EPI_ISL_739319, EPI_ISL_739320, EPI_ISL_739321	Santa Clara County Public Health Laboratory	Chan-Zuckerberg Biohub	CZB Cliahub Consortium
EPI_ISL_739322	Orange County Public Health Lab	Chan-Zuckerberg Biohub	CZB Cliahub Consortium
EPI_ISL_739323, EPI_ISL_739324	Alameda County Public Health Lab	Chan-Zuckerberg Biohub	CZB Cliahub Consortium
EPI_ISL_739325	Napa-Solano-Yolo- Marin County (NSYM) Public Health Laboratories	Chan-Zuckerberg Biohub	CZB Cliahub Consortium
EPI_ISL_739326	Alameda County Public Health Lab	Chan-Zuckerberg Biohub	CZB Cliahub Consortium
EPI_ISL_739327	Santa Clara County Public Health Laboratory	Chan-Zuckerberg Biohub	CZB Cliahub Consortium
EPI_ISL_739328	Alameda County Public Health Lab	Chan-Zuckerberg Biohub	CZB Cliahub Consortium
EPI_ISL_739329	Monterey County Public Health Lab	Chan-Zuckerberg Biohub	CZB Cliahub Consortium
EPI_ISL_739330	Humboldt County Public Health Laboratory	Chan-Zuckerberg Biohub	CZB Cliahub Consortium
EPI_ISL_739331	Orange County Public Health Lab	Chan-Zuckerberg Biohub	CZB Cliahub Consortium
EPI_ISL_739332	Santa Clara County Public Health Laboratory	Chan-Zuckerberg Biohub	CZB Cliahub Consortium
EPI_ISL_739334	Orange County Public Health Lab	Chan-Zuckerberg Biohub	CZB Cliahub Consortium
EPI_ISL_739335	Santa Clara County Public Health Laboratory	Chan-Zuckerberg Biohub	CZB Cliahub Consortium
EPI_ISL_739336	Monterey County Public Health Lab	Chan-Zuckerberg Biohub	CZB Cliahub Consortium
EPI_ISL_739337, EPI_ISL_739338	Alameda County Public Health Lab	Chan-Zuckerberg Biohub	CZB Cliahub Consortium
EPI_ISL_739339	Napa-Solano-Yolo- Marin County (NSYM) Public Health Laboratories	Chan-Zuckerberg Biohub	CZB Cliahub Consortium
EPI_ISL_739340	Alameda County Public Health Lab	Chan-Zuckerberg Biohub	CZB Cliahub Consortium
EPI_ISL_739341, EPI_ISL_739342	Orange County Public Health Lab	Chan-Zuckerberg Biohub	CZB Cliahub Consortium
EPI_ISL_739343, EPI_ISL_739344	Santa Clara County Public Health Laboratory	Chan-Zuckerberg Biohub	CZB Cliahub Consortium
EPI_ISL_739345	Orange County Public Health Lab	Chan-Zuckerberg Biohub	CZB Cliahub Consortium
EPI_ISL_739346	Santa Clara County Public Health Laboratory	Chan-Zuckerberg Biohub	CZB Cliahub Consortium
EPI_ISL_739347	Orange County Public Health Lab	Chan-Zuckerberg Biohub	CZB Cliahub Consortium
EPI_ISL_739348	Renegade	Chan-Zuckerberg Biohub	CZB Cliahub Consortium
EPI_ISL_739349	Alameda County Public Health Lab	Chan-Zuckerberg Biohub	CZB Cliahub Consortium

EPI_ISL_739350	Orange County Public Health Lab	Chan-Zuckerberg Biohub	CZB Cliahub Consortium
EPI_ISL_739351	Alameda County Public Health Lab	Chan-Zuckerberg Biohub	CZB Cliahub Consortium
EPI_ISL_739352	Santa Clara County Public Health Laboratory	Chan-Zuckerberg Biohub	CZB Cliahub Consortium
EPI_ISL_739353	Orange County Public Health Lab	Chan-Zuckerberg Biohub	CZB Cliahub Consortium
EPI_ISL_739354	Santa Clara County Public Health Laboratory	Chan-Zuckerberg Biohub	CZB Cliahub Consortium
EPI_ISL_739355	Renegade	Chan-Zuckerberg Biohub	CZB Cliahub Consortium
EPI_ISL_739356	Alameda County Public Health Lab	Chan-Zuckerberg Biohub	CZB Cliahub Consortium
EPI_ISL_739357	Santa Clara County Public Health Laboratory	Chan-Zuckerberg Biohub	CZB Cliahub Consortium
EPI_ISL_739358	Alameda County Public Health Lab	Chan-Zuckerberg Biohub	CZB Cliahub Consortium
EPI_ISL_739359	Orange County Public Health Lab	Chan-Zuckerberg Biohub	CZB Cliahub Consortium
EPI_ISL_739360	Santa Clara County Public Health Laboratory	Chan-Zuckerberg Biohub	CZB Cliahub Consortium
EPI_ISL_739361	Alameda County Public Health Lab	Chan-Zuckerberg Biohub	CZB Cliahub Consortium
EPI_ISL_739362	Orange County Public Health Lab	Chan-Zuckerberg Biohub	CZB Cliahub Consortium
EPI_ISL_739363	County of San Luis Obispo Public Health Laboratory	Chan-Zuckerberg Biohub	CZB Cliahub Consortium
EPI_ISL_739364	Monterey County Public Health Lab	Chan-Zuckerberg Biohub	CZB Cliahub Consortium
EPI_ISL_739365	Santa Clara County Public Health Laboratory	Chan-Zuckerberg Biohub	CZB Cliahub Consortium
EPI_ISL_739366, EPI_ISL_739367	Humboldt County Public Health Laboratory	Chan-Zuckerberg Biohub	CZB Cliahub Consortium
EPI_ISL_739368	Alameda County Public Health Lab	Chan-Zuckerberg Biohub	CZB Cliahub Consortium
EPI_ISL_739369	Orange County Public Health Lab	Chan-Zuckerberg Biohub	CZB Cliahub Consortium
EPI_ISL_739370, EPI_ISL_739371	Santa Clara County Public Health Laboratory	Chan-Zuckerberg Biohub	CZB Cliahub Consortium
EPI_ISL_739372	Monterey County Public Health Lab	Chan-Zuckerberg Biohub	CZB Cliahub Consortium
EPI_ISL_739373	Orange County Public Health Lab	Chan-Zuckerberg Biohub	CZB Cliahub Consortium
EPI_ISL_739374	Santa Clara County Public Health Laboratory	Chan-Zuckerberg Biohub	CZB Cliahub Consortium
EPI_ISL_739375	Orange County Public Health Lab	Chan-Zuckerberg Biohub	CZB Cliahub Consortium
EPI_ISL_739376, EPI_ISL_739377	Santa Clara County Public Health Laboratory	Chan-Zuckerberg Biohub	CZB Cliahub Consortium
EPI_ISL_739378	Alameda County Public Health Lab	Chan-Zuckerberg Biohub	CZB Cliahub Consortium
EPI_ISL_739379	Orange County Public Health Lab	Chan-Zuckerberg Biohub	CZB Cliahub Consortium
EPI_ISL_739380, EPI_ISL_739381, EPI_ISL_739382	Santa Clara County Public Health Laboratory	Chan-Zuckerberg Biohub	CZB Cliahub Consortium
EPI_ISL_739384	Orange County Public Health Lab	Chan-Zuckerberg Biohub	CZB Cliahub Consortium
EPI_ISL_739385	Alameda County Public Health Lab	Chan-Zuckerberg Biohub	CZB Cliahub Consortium
EPI_ISL_739386	Orange County Public Health Lab	Chan-Zuckerberg Biohub	CZB Cliahub Consortium
EPI_ISL_739387	Alameda County Public Health Lab	Chan-Zuckerberg Biohub	CZB Cliahub Consortium
EPI_ISL_739388	Santa Clara County Public Health Laboratory	Chan-Zuckerberg Biohub	CZB Cliahub Consortium
EPI_ISL_739389	Humboldt County Public Health Laboratory	Chan-Zuckerberg Biohub	CZB Cliahub Consortium
EPI_ISL_739390	Madera County Department of Public Health	Chan-Zuckerberg Biohub	CZB Cliahub Consortium
EPI_ISL_739391	Alameda County Public Health Lab	Chan-Zuckerberg Biohub	CZB Cliahub Consortium
EPI_ISL_739392	Santa Clara County Public Health Laboratory	Chan-Zuckerberg Biohub	CZB Cliahub Consortium
EPI_ISL_739393	Madera County Department of Public Health	Chan-Zuckerberg Biohub	CZB Cliahub Consortium
EPI_ISL_739394	Renegade	Chan-Zuckerberg Biohub	CZB Cliahub Consortium
EPI_ISL_739395	Alameda County Public Health Lab	Chan-Zuckerberg Biohub	CZB Cliahub Consortium
EPI_ISL_739396, EPI_ISL_739397	Orange County Public Health Lab	Chan-Zuckerberg Biohub	CZB Cliahub Consortium
EPI_ISL_739398	Santa Clara County Public Health Laboratory	Chan-Zuckerberg Biohub	CZB Cliahub Consortium
EPI_ISL_739399	County of San Luis Obispo Public Health Laboratory	Chan-Zuckerberg Biohub	CZB Cliahub Consortium
EPI_ISL_739400	Santa Clara County Public Health Laboratory	Chan-Zuckerberg Biohub	CZB Cliahub Consortium
EPI_ISL_739401	Orange County Public Health Lab	Chan-Zuckerberg Biohub	CZB Cliahub Consortium
EPI_ISL_739402	Santa Clara County Public Health Laboratory	Chan-Zuckerberg Biohub	CZB Cliahub Consortium
EPI_ISL_739403	Humboldt County Public Health Laboratory	Chan-Zuckerberg Biohub	CZB Cliahub Consortium
EPI_ISL_739404	Santa Clara County Public Health Laboratory	Chan-Zuckerberg Biohub	CZB Cliahub Consortium
EPI_ISL_739405	Humboldt County Public Health Laboratory	Chan-Zuckerberg Biohub	CZB Cliahub Consortium
EPI_ISL_739406, EPI_ISL_739407, EPI_ISL_739408	Alameda County Public Health Lab	Chan-Zuckerberg Biohub	CZB Cliahub Consortium
EPI_ISL_739409	Santa Clara County Public Health Laboratory	Chan-Zuckerberg Biohub	CZB Cliahub Consortium
EPI_ISL_739410	Orange County Public Health Lab	Chan-Zuckerberg Biohub	CZB Cliahub Consortium
EPI_ISL_739411	Humboldt County Public Health Laboratory	Chan-Zuckerberg Biohub	CZB Cliahub Consortium
EPI_ISL_739412	Orange County Public Health Lab	Chan-Zuckerberg Biohub	CZB Cliahub Consortium
EPI_ISL_739413, EPI_ISL_739415, EPI_ISL_739416	Santa Clara County Public Health Laboratory	Chan-Zuckerberg Biohub	CZB Cliahub Consortium
EPI_ISL_739417, EPI_ISL_739418	Humboldt County Public Health Laboratory	Chan-Zuckerberg Biohub	CZB Cliahub Consortium

EPI_ISL_739419	Santa Clara County Public Health Laboratory	Chan-Zuckerberg Biohub	CZB Cliahub Consortium
EPI_ISL_739420, EPI_ISL_739421	Orange County Public Health Lab	Chan-Zuckerberg Biohub	CZB Cliahub Consortium
EPI_ISL_739422	Alameda County Public Health Lab	Chan-Zuckerberg Biohub	CZB Cliahub Consortium
EPI_ISL_739423	Orange County Public Health Lab	Chan-Zuckerberg Biohub	CZB Cliahub Consortium
EPI_ISL_739425	Humboldt County Public Health Laboratory	Chan-Zuckerberg Biohub	CZB Cliahub Consortium
EPI_ISL_739426, EPI_ISL_739427	Alameda County Public Health Lab	Chan-Zuckerberg Biohub	CZB Cliahub Consortium
EPI_ISL_739428	Orange County Public Health Lab	Chan-Zuckerberg Biohub	CZB Cliahub Consortium
EPI_ISL_739429	Madera County Department of Public Health	Chan-Zuckerberg Biohub	CZB Cliahub Consortium
EPI_ISL_739430	Humboldt County Public Health Laboratory	Chan-Zuckerberg Biohub	CZB Cliahub Consortium
EPI_ISL_739431	Santa Clara County Public Health Laboratory	Chan-Zuckerberg Biohub	CZB Cliahub Consortium
EPI_ISL_739432	Orange County Public Health Lab	Chan-Zuckerberg Biohub	CZB Cliahub Consortium
EPI_ISL_739433	Humboldt County Public Health Laboratory	Chan-Zuckerberg Biohub	CZB Cliahub Consortium
EPI_ISL_739434, EPI_ISL_739435	Alameda County Public Health Lab	Chan-Zuckerberg Biohub	CZB Cliahub Consortium
EPI_ISL_739436	Humboldt County Public Health Laboratory	Chan-Zuckerberg Biohub	CZB Cliahub Consortium
EPI_ISL_739437, EPI_ISL_739438	Orange County Public Health Lab	Chan-Zuckerberg Biohub	CZB Cliahub Consortium
EPI_ISL_739439	Alameda County Public Health Lab	Chan-Zuckerberg Biohub	CZB Cliahub Consortium
EPI_ISL_739440	Santa Clara County Public Health Laboratory	Chan-Zuckerberg Biohub	CZB Cliahub Consortium
EPI_ISL_739441	Orange County Public Health Lab	Chan-Zuckerberg Biohub	CZB Cliahub Consortium
EPI_ISL_739442	Humboldt County Public Health Laboratory	Chan-Zuckerberg Biohub	CZB Cliahub Consortium
EPI_ISL_739443	Santa Clara County Public Health Laboratory	Chan-Zuckerberg Biohub	CZB Cliahub Consortium
EPI_ISL_739444	Alameda County Public Health Lab	Chan-Zuckerberg Biohub	CZB Cliahub Consortium
EPI_ISL_739445	Orange County Public Health Lab	Chan-Zuckerberg Biohub	CZB Cliahub Consortium
EPI_ISL_739446	Alameda County Public Health Lab	Chan-Zuckerberg Biohub	CZB Cliahub Consortium
EPI_ISL_739447	Humboldt County Public Health Laboratory	Chan-Zuckerberg Biohub	CZB Cliahub Consortium
EPI_ISL_739448, EPI_ISL_739449	Alameda County Public Health Lab	Chan-Zuckerberg Biohub	CZB Cliahub Consortium
EPI_ISL_739450	Orange County Public Health Lab	Chan-Zuckerberg Biohub	CZB Cliahub Consortium
EPI_ISL_739451	Santa Clara County Public Health Laboratory	Chan-Zuckerberg Biohub	CZB Cliahub Consortium
EPI_ISL_739452	Humboldt County Public Health Laboratory	Chan-Zuckerberg Biohub	CZB Cliahub Consortium
EPI_ISL_739453	Santa Clara County Public Health Laboratory	Chan-Zuckerberg Biohub	CZB Cliahub Consortium
EPI_ISL_739454	Alameda County Public Health Lab	Chan-Zuckerberg Biohub	CZB Cliahub Consortium
EPI_ISL_739455	Orange County Public Health Lab	Chan-Zuckerberg Biohub	CZB Cliahub Consortium
EPI_ISL_739456, EPI_ISL_739457, EPI_ISL_739458	Santa Clara County Public Health Laboratory	Chan-Zuckerberg Biohub	CZB Cliahub Consortium
EPI_ISL_739459	Alameda County Public Health Lab	Chan-Zuckerberg Biohub	CZB Cliahub Consortium
EPI_ISL_739460	Orange County Public Health Lab	Chan-Zuckerberg Biohub	CZB Cliahub Consortium
EPI_ISL_739461	Humboldt County Public Health Laboratory	Chan-Zuckerberg Biohub	CZB Cliahub Consortium
EPI_ISL_739462	Orange County Public Health Lab	Chan-Zuckerberg Biohub	CZB Cliahub Consortium
EPI_ISL_739463	Alameda County Public Health Lab	Chan-Zuckerberg Biohub	CZB Cliahub Consortium
EPI_ISL_739464, EPI_ISL_739465	Orange County Public Health Lab	Chan-Zuckerberg Biohub	CZB Cliahub Consortium
EPI_ISL_739466, EPI_ISL_739467	Renegade	Chan-Zuckerberg Biohub	CZB Cliahub Consortium
EPI_ISL_739469	Napa-Solano-Yolo- Marin County (NSYM) Public Health Laboratories	Chan-Zuckerberg Biohub	CZB Cliahub Consortium
EPI_ISL_739470	Alameda County Public Health Lab	Chan-Zuckerberg Biohub	CZB Cliahub Consortium
EPI_ISL_739471	Napa-Solano-Yolo- Marin County (NSYM) Public Health Laboratories	Chan-Zuckerberg Biohub	CZB Cliahub Consortium
EPI_ISL_739472, EPI_ISL_739473	Alameda County Public Health Lab	Chan-Zuckerberg Biohub	CZB Cliahub Consortium
EPI_ISL_739474	Napa-Solano-Yolo- Marin County (NSYM) Public Health Laboratories	Chan-Zuckerberg Biohub	CZB Cliahub Consortium
EPI_ISL_739475	Humboldt County Public Health Laboratory	Chan-Zuckerberg Biohub	CZB Cliahub Consortium
EPI_ISL_739476	Santa Clara County Public Health Laboratory	Chan-Zuckerberg Biohub	CZB Cliahub Consortium
EPI_ISL_739477	Orange County Public Health Lab	Chan-Zuckerberg Biohub	CZB Cliahub Consortium
EPI_ISL_739478	Renegade	Chan-Zuckerberg Biohub	CZB Cliahub Consortium
EPI_ISL_739479	Santa Clara County Public Health Laboratory	Chan-Zuckerberg Biohub	CZB Cliahub Consortium
EPI_ISL_739480	County of San Luis Obispo Public Health Laboratory	Chan-Zuckerberg Biohub	CZB Cliahub Consortium
EPI_ISL_739481	Santa Clara County Public Health Laboratory	Chan-Zuckerberg Biohub	CZB Cliahub Consortium
EPI_ISL_739482	Orange County Public Health Lab	Chan-Zuckerberg Biohub	CZB Cliahub Consortium
EPI_ISL_739483, EPI_ISL_739484	Alameda County Public Health Lab	Chan-Zuckerberg Biohub	CZB Cliahub Consortium
EPI_ISL_739485	Santa Clara County Public Health Laboratory	Chan-Zuckerberg Biohub	CZB Cliahub Consortium
EPI_ISL_739486	Orange County Public Health Lab	Chan-Zuckerberg Biohub	CZB Cliahub Consortium
EPI_ISL_739487	Santa Clara County Public Health Laboratory	Chan-Zuckerberg Biohub	CZB Cliahub Consortium

EPI_ISL_739488	Orange County Public Health Lab	Chan-Zuckerberg Biohub	CZB Cliahub Consortium
EPI_ISL_739489	Humboldt County Public Health Laboratory	Chan-Zuckerberg Biohub	CZB Cliahub Consortium
EPI_ISL_739490	Orange County Public Health Lab	Chan-Zuckerberg Biohub	CZB Cliahub Consortium
EPI_ISL_739491	Santa Clara County Public Health Laboratory	Chan-Zuckerberg Biohub	CZB Cliahub Consortium
EPI_ISL_739492	County of San Luis Obispo Public Health Laboratory	Chan-Zuckerberg Biohub	CZB Cliahub Consortium
EPI_ISL_739493, EPI_ISL_739494	Orange County Public Health Lab	Chan-Zuckerberg Biohub	CZB Cliahub Consortium
EPI_ISL_739495	Alameda County Public Health Lab	Chan-Zuckerberg Biohub	CZB Cliahub Consortium
EPI_ISL_739496, EPI_ISL_739497	Orange County Public Health Lab	Chan-Zuckerberg Biohub	CZB Cliahub Consortium
EPI_ISL_739498	Santa Clara County Public Health Laboratory	Chan-Zuckerberg Biohub	CZB Cliahub Consortium
EPI_ISL_739499	Humboldt County Public Health Laboratory	Chan-Zuckerberg Biohub	CZB Cliahub Consortium
EPI_ISL_739500	Orange County Public Health Lab	Chan-Zuckerberg Biohub	CZB Cliahub Consortium
EPI_ISL_739501, EPI_ISL_739502	Santa Clara County Public Health Laboratory	Chan-Zuckerberg Biohub	CZB Cliahub Consortium
EPI_ISL_739503	Alameda County Public Health Lab	Chan-Zuckerberg Biohub	CZB Cliahub Consortium
EPI_ISL_739504, EPI_ISL_739505	Santa Clara County Public Health Laboratory	Chan-Zuckerberg Biohub	CZB Cliahub Consortium
EPI_ISL_739506, EPI_ISL_739507	Alameda County Public Health Lab	Chan-Zuckerberg Biohub	CZB Cliahub Consortium
EPI_ISL_739508, EPI_ISL_739509, EPI_ISL_739510, EPI_ISL_739512	Santa Clara County Public Health Laboratory	Chan-Zuckerberg Biohub	CZB Cliahub Consortium
EPI_ISL_739513	Orange County Public Health Lab	Chan-Zuckerberg Biohub	CZB Cliahub Consortium
EPI_ISL_739515, EPI_ISL_739516	Alameda County Public Health Lab	Chan-Zuckerberg Biohub	CZB Cliahub Consortium
EPI_ISL_739517	Orange County Public Health Lab	Chan-Zuckerberg Biohub	CZB Cliahub Consortium
EPI_ISL_739518, EPI_ISL_739519	Alameda County Public Health Lab	Chan-Zuckerberg Biohub	CZB Cliahub Consortium
EPI_ISL_739520, EPI_ISL_739521	Santa Clara County Public Health Laboratory	Chan-Zuckerberg Biohub	CZB Cliahub Consortium
EPI_ISL_739522, EPI_ISL_739523	Alameda County Public Health Lab	Chan-Zuckerberg Biohub	CZB Cliahub Consortium
EPI_ISL_739524	Orange County Public Health Lab	Chan-Zuckerberg Biohub	CZB Cliahub Consortium
EPI_ISL_739525	Humboldt County Public Health Laboratory	Chan-Zuckerberg Biohub	CZB Cliahub Consortium
EPI_ISL_739526	Alameda County Public Health Lab	Chan-Zuckerberg Biohub	CZB Cliahub Consortium
EPI_ISL_739527	Humboldt County Public Health Laboratory	Chan-Zuckerberg Biohub	CZB Cliahub Consortium
EPI_ISL_739528	Alameda County Public Health Lab	Chan-Zuckerberg Biohub	CZB Cliahub Consortium
EPI_ISL_739529	Orange County Public Health Lab	Chan-Zuckerberg Biohub	CZB Cliahub Consortium
EPI_ISL_739530, EPI_ISL_739531	Humboldt County Public Health Laboratory	Chan-Zuckerberg Biohub	CZB Cliahub Consortium
EPI_ISL_739532	Orange County Public Health Lab	Chan-Zuckerberg Biohub	CZB Cliahub Consortium
EPI_ISL_739533	Humboldt County Public Health Laboratory	Chan-Zuckerberg Biohub	CZB Cliahub Consortium
EPI_ISL_739534	Orange County Public Health Lab	Chan-Zuckerberg Biohub	CZB Cliahub Consortium
EPI_ISL_739535	Alameda County Public Health Lab	Chan-Zuckerberg Biohub	CZB Cliahub Consortium
EPI_ISL_739536, EPI_ISL_739537	Santa Clara County Public Health Laboratory	Chan-Zuckerberg Biohub	CZB Cliahub Consortium
EPI_ISL_739539	Madera County Department of Public Health	Chan-Zuckerberg Biohub	CZB Cliahub Consortium
EPI_ISL_739540	Orange County Public Health Lab	Chan-Zuckerberg Biohub	CZB Cliahub Consortium
EPI_ISL_739541	Alameda County Public Health Lab	Chan-Zuckerberg Biohub	CZB Cliahub Consortium
EPI_ISL_739542	Humboldt County Public Health Laboratory	Chan-Zuckerberg Biohub	CZB Cliahub Consortium
EPI_ISL_739543	Santa Clara County Public Health Laboratory	Chan-Zuckerberg Biohub	CZB Cliahub Consortium
EPI_ISL_739544	Alameda County Public Health Lab	Chan-Zuckerberg Biohub	CZB Cliahub Consortium
EPI_ISL_739545	Orange County Public Health Lab	Chan-Zuckerberg Biohub	CZB Cliahub Consortium
EPI_ISL_739546	Monterey County Public Health Lab	Chan-Zuckerberg Biohub	CZB Cliahub Consortium
EPI_ISL_739547	Orange County Public Health Lab	Chan-Zuckerberg Biohub	CZB Cliahub Consortium
EPI_ISL_739548, EPI_ISL_739549, EPI_ISL_739550	Santa Clara County Public Health Laboratory	Chan-Zuckerberg Biohub	CZB Cliahub Consortium
EPI_ISL_739551, EPI_ISL_739552	Alameda County Public Health Lab	Chan-Zuckerberg Biohub	CZB Cliahub Consortium
EPI_ISL_739553	Humboldt County Public Health Laboratory	Chan-Zuckerberg Biohub	CZB Cliahub Consortium
EPI_ISL_739555	Alameda County Public Health Lab	Chan-Zuckerberg Biohub	CZB Cliahub Consortium
EPI_ISL_739556	Orange County Public Health Lab	Chan-Zuckerberg Biohub	CZB Cliahub Consortium
EPI_ISL_739557	Napa-Solano-Yolo- Marin County (NSYM) Public Health Laboratories	Chan-Zuckerberg Biohub	CZB Cliahub Consortium
EPI_ISL_739558	Humboldt County Public Health Laboratory	Chan-Zuckerberg Biohub	CZB Cliahub Consortium
EPI_ISL_739559	Orange County Public Health Lab	Chan-Zuckerberg Biohub	CZB Cliahub Consortium
EPI_ISL_739560, EPI_ISL_739561, EPI_ISL_739562	Santa Clara County Public Health Laboratory	Chan-Zuckerberg Biohub	CZB Cliahub Consortium
EPI_ISL_739563	Alameda County Public Health Lab	Chan-Zuckerberg Biohub	CZB Cliahub Consortium
EPI_ISL_739564	Renegade	Chan-Zuckerberg Biohub	CZB Cliahub Consortium
EPI_ISL_739565	Alameda County Public Health Lab	Chan-Zuckerberg Biohub	CZB Cliahub Consortium
EPI_ISL_739566	Orange County Public Health Lab	Chan-Zuckerberg Biohub	CZB Cliahub Consortium

EPI_ISL_739567	Madera County Department of Public Health	Chan-Zuckerberg Biohub	CZB Cliahub Consortium
EPI_ISL_739568	Orange County Public Health Lab	Chan-Zuckerberg Biohub	CZB Cliahub Consortium
EPI_ISL_739569	Alameda County Public Health Lab	Chan-Zuckerberg Biohub	CZB Cliahub Consortium
EPI_ISL_739570	Orange County Public Health Lab	Chan-Zuckerberg Biohub	CZB Cliahub Consortium
EPI_ISL_739571	Humboldt County Public Health Laboratory	Chan-Zuckerberg Biohub	CZB Cliahub Consortium
EPI_ISL_739572	Alameda County Public Health Lab	Chan-Zuckerberg Biohub	CZB Cliahub Consortium
EPI_ISL_739573	Orange County Public Health Lab	Chan-Zuckerberg Biohub	CZB Cliahub Consortium
EPI_ISL_739574	Santa Clara County Public Health Laboratory	Chan-Zuckerberg Biohub	CZB Cliahub Consortium
EPI_ISL_739575	Alameda County Public Health Lab	Chan-Zuckerberg Biohub	CZB Cliahub Consortium
EPI_ISL_739577	Orange County Public Health Lab	Chan-Zuckerberg Biohub	CZB Cliahub Consortium
EPI_ISL_739578	Alameda County Public Health Lab	Chan-Zuckerberg Biohub	CZB Cliahub Consortium
EPI_ISL_739579, EPI_ISL_739580	Santa Clara County Public Health Laboratory	Chan-Zuckerberg Biohub	CZB Cliahub Consortium
EPI_ISL_739581, EPI_ISL_739582	Orange County Public Health Lab	Chan-Zuckerberg Biohub	CZB Cliahub Consortium
EPI_ISL_739583	Alameda County Public Health Lab	Chan-Zuckerberg Biohub	CZB Cliahub Consortium
EPI_ISL_739584	Santa Clara County Public Health Laboratory	Chan-Zuckerberg Biohub	CZB Cliahub Consortium
EPI_ISL_739585	Humboldt County Public Health Laboratory	Chan-Zuckerberg Biohub	CZB Cliahub Consortium
EPI_ISL_739586	Orange County Public Health Lab	Chan-Zuckerberg Biohub	CZB Cliahub Consortium
EPI_ISL_739587	Alameda County Public Health Lab	Chan-Zuckerberg Biohub	CZB Cliahub Consortium
EPI_ISL_739588	County of San Luis Obispo Public Health Laboratory	Chan-Zuckerberg Biohub	CZB Cliahub Consortium
EPI_ISL_739589	Santa Clara County Public Health Laboratory	Chan-Zuckerberg Biohub	CZB Cliahub Consortium
EPI_ISL_739590	Alameda County Public Health Lab	Chan-Zuckerberg Biohub	CZB Cliahub Consortium
EPI_ISL_739591, EPI_ISL_739592	Orange County Public Health Lab	Chan-Zuckerberg Biohub	CZB Cliahub Consortium
EPI_ISL_739593, EPI_ISL_739594	Santa Clara County Public Health Laboratory	Chan-Zuckerberg Biohub	CZB Cliahub Consortium
EPI_ISL_739595	Orange County Public Health Lab	Chan-Zuckerberg Biohub	CZB Cliahub Consortium
EPI_ISL_739596, EPI_ISL_739597	Santa Clara County Public Health Laboratory	Chan-Zuckerberg Biohub	CZB Cliahub Consortium
EPI_ISL_739598	Alameda County Public Health Lab	Chan-Zuckerberg Biohub	CZB Cliahub Consortium
EPI_ISL_739599	Orange County Public Health Lab	Chan-Zuckerberg Biohub	CZB Cliahub Consortium
EPI_ISL_739600	Alameda County Public Health Lab	Chan-Zuckerberg Biohub	CZB Cliahub Consortium
EPI_ISL_739602	Santa Clara County Public Health Laboratory	Chan-Zuckerberg Biohub	CZB Cliahub Consortium
EPI_ISL_739603	San Francisco Public Health Laboratory	Chan-Zuckerberg Biohub	CZB Cliahub Consortium
EPI_ISL_739604	Alameda County Public Health Lab	Chan-Zuckerberg Biohub	CZB Cliahub Consortium
EPI_ISL_739605	Santa Clara County Public Health Laboratory	Chan-Zuckerberg Biohub	CZB Cliahub Consortium
EPI_ISL_739606	Alameda County Public Health Lab	Chan-Zuckerberg Biohub	CZB Cliahub Consortium
EPI_ISL_739607	Orange County Public Health Lab	Chan-Zuckerberg Biohub	CZB Cliahub Consortium
EPI_ISL_739608	Humboldt County Public Health Laboratory	Chan-Zuckerberg Biohub	CZB Cliahub Consortium
EPI_ISL_739609	Orange County Public Health Lab	Chan-Zuckerberg Biohub	CZB Cliahub Consortium
EPI_ISL_739610	Alameda County Public Health Lab	Chan-Zuckerberg Biohub	CZB Cliahub Consortium
EPI_ISL_739611	Humboldt County Public Health Laboratory	Chan-Zuckerberg Biohub	CZB Cliahub Consortium
EPI_ISL_739612	Santa Clara County Public Health Laboratory	Chan-Zuckerberg Biohub	CZB Cliahub Consortium
EPI_ISL_739613	UCSF Clinical Microbiology Laboratory	Chan-Zuckerberg Biohub	CZB Cliahub Consortium
EPI_ISL_739614	Orange County Public Health Lab	Chan-Zuckerberg Biohub	CZB Cliahub Consortium
EPI_ISL_739616	Alameda County Public Health Lab	Chan-Zuckerberg Biohub	CZB Cliahub Consortium
EPI_ISL_739617, EPI_ISL_739618	Santa Clara County Public Health Laboratory	Chan-Zuckerberg Biohub	CZB Cliahub Consortium
EPI_ISL_739619	Alameda County Public Health Lab	Chan-Zuckerberg Biohub	CZB Cliahub Consortium
EPI_ISL_739620	Orange County Public Health Lab	Chan-Zuckerberg Biohub	CZB Cliahub Consortium
EPI_ISL_739621	Humboldt County Public Health Laboratory	Chan-Zuckerberg Biohub	CZB Cliahub Consortium
EPI_ISL_739622, EPI_ISL_739623	Alameda County Public Health Lab	Chan-Zuckerberg Biohub	CZB Cliahub Consortium
EPI_ISL_739624	Santa Clara County Public Health Laboratory	Chan-Zuckerberg Biohub	CZB Cliahub Consortium
EPI_ISL_739625	Orange County Public Health Lab	Chan-Zuckerberg Biohub	CZB Cliahub Consortium
EPI_ISL_739626	Santa Clara County Public Health Laboratory	Chan-Zuckerberg Biohub	CZB Cliahub Consortium
EPI_ISL_739627	Orange County Public Health Lab	Chan-Zuckerberg Biohub	CZB Cliahub Consortium
EPI_ISL_739628	Napa-Solano-Yolo- Marin County (NSYM) Public Health Laboratories	Chan-Zuckerberg Biohub	CZB Cliahub Consortium
EPI_ISL_739629	Santa Clara County Public Health Laboratory	Chan-Zuckerberg Biohub	CZB Cliahub Consortium
EPI_ISL_739630	Renegade	Chan-Zuckerberg Biohub	CZB Cliahub Consortium
EPI_ISL_739631	Alameda County Public Health Lab	Chan-Zuckerberg Biohub	CZB Cliahub Consortium
EPI_ISL_739632, EPI_ISL_739633	UCSF Clinical Microbiology Laboratory	Chan-Zuckerberg Biohub	CZB Cliahub Consortium
EPI_ISL_739634	Alameda County Public Health Lab	Chan-Zuckerberg Biohub	CZB Cliahub Consortium
EPI_ISL_739636, EPI_ISL_739637	Orange County Public Health Lab	Chan-Zuckerberg Biohub	CZB Cliahub Consortium

EPI_ISL_739638	Alameda County Public Health Lab	Chan-Zuckerberg Biohub	CZB Cliahub Consortium
EPI_ISL_739639	Tulare County Public Health Lab	Chan-Zuckerberg Biohub	CZB Cliahub Consortium
EPI_ISL_739641	Orange County Public Health Lab	Chan-Zuckerberg Biohub	CZB Cliahub Consortium
EPI_ISL_739642	Santa Clara County Public Health Laboratory	Chan-Zuckerberg Biohub	CZB Cliahub Consortium
EPI_ISL_739643, EPI_ISL_739644	Alameda County Public Health Lab	Chan-Zuckerberg Biohub	CZB Cliahub Consortium
EPI_ISL_739645	Napa-Solano-Yolo- Marin County (NSYM) Public Health Laboratories	Chan-Zuckerberg Biohub	CZB Cliahub Consortium
EPI_ISL_739646	Orange County Public Health Lab	Chan-Zuckerberg Biohub	CZB Cliahub Consortium
EPI_ISL_739647	Santa Clara County Public Health Laboratory	Chan-Zuckerberg Biohub	CZB Cliahub Consortium
EPI_ISL_739648	Alameda County Public Health Lab	Chan-Zuckerberg Biohub	CZB Cliahub Consortium
EPI_ISL_739649, EPI_ISL_739650	Santa Clara County Public Health Laboratory	Chan-Zuckerberg Biohub	CZB Cliahub Consortium
EPI_ISL_739651	Humboldt County Public Health Laboratory	Chan-Zuckerberg Biohub	CZB Cliahub Consortium
EPI_ISL_739653	Santa Clara County Public Health Laboratory	Chan-Zuckerberg Biohub	CZB Cliahub Consortium
EPI_ISL_739654	Orange County Public Health Lab	Chan-Zuckerberg Biohub	CZB Cliahub Consortium
EPI_ISL_739655	Renegade	Chan-Zuckerberg Biohub	CZB Cliahub Consortium
EPI_ISL_739656	Alameda County Public Health Lab	Chan-Zuckerberg Biohub	CZB Cliahub Consortium
EPI_ISL_739657	Santa Clara County Public Health Laboratory	Chan-Zuckerberg Biohub	CZB Cliahub Consortium
EPI_ISL_739658	Orange County Public Health Lab	Chan-Zuckerberg Biohub	CZB Cliahub Consortium
EPI_ISL_739659, EPI_ISL_739660, EPI_ISL_739661	Al-Quds Nutrition and Health Research Institute, Al-Quds University	Al-Quds Nutrition and Health Research Institute, Al-Quds University	Nasereddin,A., Ereqat,S., Al-Jawabreh,A., Rishmawi,C.
EPI_ISL_739663, EPI_ISL_739671, EPI_ISL_739672, EPI_ISL_739673, EPI_ISL_739675, EPI_ISL_739680	Instituto Nacional de Salud, Bogotá, Colombia	Instituto Nacional de Salud, Bogotá, Colombia	Katherine Laiton-Donato, Diego A. Álvarez-Díaz, Carlos Franco-Muñoz, Mauricio Pacheco-Montealegre, Jonathan Reales, Diego Andrés Prada, Sheryl Corchuelo, Magdalena Weisner, Martha Lucia Ospina Martinez, Marcela Mercado-Reyes
EPI_ISL_739693, EPI_ISL_739694, EPI_ISL_739695, EPI_ISL_739696, EPI_ISL_739697, EPI_ISL_739698, EPI_ISL_739699	Laboratoire national de santé, Microbiology, Virology	Laboratoire national de santé, Microbiology, Microbial Genomics Platform	Anke Wienecke-Baldacchino, Catherine Ragimbeau,Jessica Tapp, Fatu Djabi, Lise Pignon, Raoul Salmon, Tamir Abdelrahman
EPI_ISL_739700	Laboratoire national de santé, Microbiology, Virology	Laboratoire national de santé, Microbiology, Epidemiology and Microbial Genomics	Anke Wienecke-Baldacchino, Catherine Ragimbeau, Tamir Abdelrahman, Jessica Tapp, Fatu Djabi, Trung Nguyen Nguyen
EPI_ISL_739701, EPI_ISL_739702, EPI_ISL_739703, EPI_ISL_739704, EPI_ISL_739705, EPI_ISL_739706, EPI_ISL_739707, EPI_ISL_739708, EPI_ISL_739709, EPI_ISL_739710, EPI_ISL_739711, EPI_ISL_739712, EPI_ISL_739713, EPI_ISL_739714, EPI_ISL_739715, EPI_ISL_739716, EPI_ISL_739717, EPI_ISL_739718, EPI_ISL_739719, EPI_ISL_739720, EPI_ISL_739721, EPI_ISL_739722, EPI_ISL_739723, EPI_ISL_739724, EPI_ISL_739725, EPI_ISL_739726, EPI_ISL_739727, EPI_ISL_739728, EPI_ISL_739729, EPI_ISL_739730, EPI_ISL_739731, EPI_ISL_739732, EPI_ISL_739733, EPI_ISL_739734, EPI_ISL_739735, EPI_ISL_739736, EPI_ISL_739737, EPI_ISL_739738, EPI_ISL_739739, EPI_ISL_739740, EPI_ISL_739741, EPI_ISL_739742, EPI_ISL_739743, EPI_ISL_739744, EPI_ISL_739745, EPI_ISL_739746, EPI_ISL_739747, EPI_ISL_739748, EPI_ISL_739749, EPI_ISL_739750, EPI_ISL_739751, EPI_ISL_739752			
see above	Laboratoire national de santé, Microbiology, Virology	Laboratoire national de santé, Microbiology, Microbial Genomics Platform	Anke Wienecke-Baldacchino, Catherine Ragimbeau,Jessica Tapp, Fatu Djabi, Lise Pignon, Raoul Salmon, Tamir Abdelrahman
EPI_ISL_739753	Laboratoire national de santé, Microbiology, Virology	Laboratoire national de santé, Microbiology, Microbial Genomics Platform	Anke Wienecke-Baldacchino, Catherine Ragimbeau, Tamir Abdelrahman, Jessica Tapp, Fatu Djabi
EPI_ISL_739754, EPI_ISL_739755, EPI_ISL_739756, EPI_ISL_739757, EPI_ISL_739759, EPI_ISL_739760, EPI_ISL_739761, EPI_ISL_739762, EPI_ISL_739763, EPI_ISL_739764, EPI_ISL_739765, EPI_ISL_739766, EPI_ISL_739767, EPI_ISL_739768, EPI_ISL_739769, EPI_ISL_739770, EPI_ISL_739771, EPI_ISL_739772			
see above	Laboratoire national de santé, Microbiology, Virology	Laboratoire national de santé, Microbiology, Microbial Genomics Platform	Anke Wienecke-Baldacchino, Catherine Ragimbeau,Jessica Tapp, Fatu Djabi, Lise Pignon, Raoul Salmon, Tamir Abdelrahman
EPI_ISL_739773	Laboratoire national de santé, Microbiology, Virology	Laboratoire national de santé, Microbiology, Microbial Genomics Platform	Anke Wienecke-Baldacchino, Catherine Ragimbeau, Tamir Abdelrahman, Jessica Tapp, Fatu Djabi
EPI_ISL_739774, EPI_ISL_739775, EPI_ISL_739776, EPI_ISL_739777, EPI_ISL_739778, EPI_ISL_739779, EPI_ISL_739780, EPI_ISL_739781, EPI_ISL_739782, EPI_ISL_739783, EPI_ISL_739784, EPI_ISL_739785, EPI_ISL_739786, EPI_ISL_739787, EPI_ISL_739788, EPI_ISL_739789, EPI_ISL_739790, EPI_ISL_739791			
see above	Laboratoire national de santé, Microbiology, Virology	Laboratoire national de santé, Microbiology, Microbial Genomics Platform	Anke Wienecke-Baldacchino, Catherine Ragimbeau,Jessica Tapp, Fatu Djabi, Lise Pignon, Raoul Salmon, Tamir Abdelrahman
EPI_ISL_739792	Laboratoire national de santé, Microbiology, Virology	Laboratoire national de santé, Microbiology, Microbial Genomics Platform	Anke Wienecke-Baldacchino, Catherine Ragimbeau, Tamir Abdelrahman, Jessica Tapp, Fatu Djabi
EPI_ISL_739793, EPI_ISL_739794, EPI_ISL_739795, EPI_ISL_739796, EPI_ISL_739797, EPI_ISL_739798, EPI_ISL_739799, EPI_ISL_739800, EPI_ISL_739801, EPI_ISL_739802, EPI_ISL_739803, EPI_ISL_739804, EPI_ISL_739805, EPI_ISL_739806, EPI_ISL_739807, EPI_ISL_739808, EPI_ISL_739809, EPI_ISL_739810, EPI_ISL_739811, EPI_ISL_739812, EPI_ISL_739814, EPI_ISL_739815, EPI_ISL_739816, EPI_ISL_739817, EPI_ISL_739818, EPI_ISL_739819, EPI_ISL_739820, EPI_ISL_739821, EPI_ISL_739822, EPI_ISL_739823, EPI_ISL_739824, EPI_ISL_739825, EPI_ISL_739826, EPI_ISL_739827, EPI_ISL_739828, EPI_ISL_739829, EPI_ISL_739830, EPI_ISL_739831, EPI_ISL_739832			
see above	Laboratoire national de santé, Microbiology, Virology	Laboratoire national de santé, Microbiology, Microbial Genomics Platform	Anke Wienecke-Baldacchino, Catherine Ragimbeau,Jessica Tapp, Fatu Djabi, Lise Pignon, Raoul Salmon, Tamir Abdelrahman
EPI_ISL_739833, EPI_ISL_739834, EPI_ISL_739835	Laboratoire national de santé, Microbiology, Virology	Laboratoire national de santé, Microbiology, Microbial Genomics Platform	Anke Wienecke-Baldacchino, Catherine Ragimbeau, Tamir Abdelrahman, Jessica Tapp, Fatu Djabi
EPI_ISL_739836, EPI_ISL_739837, EPI_ISL_739838, EPI_ISL_739839, EPI_ISL_739840, EPI_ISL_739841, EPI_ISL_739842, EPI_ISL_739843, EPI_ISL_739844, EPI_ISL_739845, EPI_ISL_739846, EPI_ISL_739847			
see above	Laboratoire national de santé, Microbiology, Virology	Laboratoire national de santé, Microbiology, Microbial Genomics Platform	Anke Wienecke-Baldacchino, Catherine Ragimbeau,Jessica Tapp, Fatu Djabi, Lise Pignon, Raoul Salmon, Tamir Abdelrahman
EPI_ISL_739848	Laboratoire national de santé, Microbiology, Virology	Laboratoire national de santé, Microbiology, Microbial Genomics Platform	Anke Wienecke-Baldacchino, Catherine Ragimbeau, Tamir Abdelrahman, Jessica Tapp, Fatu Djabi
EPI_ISL_739849, EPI_ISL_739850, EPI_ISL_739851, EPI_ISL_739852, EPI_ISL_739853, EPI_ISL_739854, EPI_ISL_739855, EPI_ISL_739856, EPI_ISL_739857, EPI_ISL_739858, EPI_ISL_739859, EPI_ISL_739860, EPI_ISL_739861, EPI_ISL_739862, EPI_ISL_739863, EPI_ISL_739864, EPI_ISL_739865, EPI_ISL_739866, EPI_ISL_739867, EPI_ISL_739868, EPI_ISL_739869			
see above	Laboratoire national de santé, Microbiology, Virology	Laboratoire national de santé, Microbiology, Microbial Genomics Platform	Anke Wienecke-Baldacchino, Catherine Ragimbeau,Jessica Tapp, Fatu Djabi, Lise Pignon, Raoul Salmon, Tamir Abdelrahman
EPI_ISL_739870	Laboratoire national de santé, Microbiology, Virology	Laboratoire national de santé, Microbiology, Microbial Genomics Platform	Anke Wienecke-Baldacchino, Catherine Ragimbeau, Tamir Abdelrahman, Jessica Tapp, Fatu Djabi
EPI_ISL_739871, EPI_ISL_739872, EPI_ISL_739873, EPI_ISL_739874, EPI_ISL_739875, EPI_ISL_739876, EPI_ISL_739878, EPI_ISL_739879, EPI_ISL_739881, EPI_ISL_739882, EPI_ISL_739883, EPI_ISL_739884, EPI_ISL_739885, EPI_ISL_739886, EPI_ISL_739887, EPI_ISL_739888, EPI_ISL_739889, EPI_ISL_739890, EPI_ISL_739891, EPI_ISL_739892, EPI_ISL_739893, EPI_ISL_739894, EPI_ISL_739895, EPI_ISL_739896, EPI_ISL_739898, EPI_ISL_739899, EPI_ISL_739900, EPI_ISL_739901, EPI_ISL_739902, EPI_ISL_739903, EPI_ISL_739904, EPI_ISL_739905, EPI_ISL_739906, EPI_ISL_739907, EPI_ISL_739908, EPI_ISL_739909, EPI_ISL_739910, EPI_ISL_739911, EPI_ISL_739912, EPI_ISL_739913, EPI_ISL_739914, EPI_ISL_739915, EPI_ISL_739916, EPI_ISL_739917			
see above	Laboratoire national de santé, Microbiology, Virology	Laboratoire national de santé, Microbiology, Microbial	Anke Wienecke-Baldacchino, Catherine Ragimbeau,Jessica Tapp, Fatu Djabi, Lise Pignon, Raoul Salmon, Tamir Abdelrahman

		Genomics Platform	
EPI_ISL_739918	Laboratoire national de santé, Microbiology, Virology	Laboratoire national de santé, Microbiology, Microbial Genomics Platform	Anke Wienecke-Baldacchino, Catherine Ragimbeau, Tamir Abdelrahman, Jessica Tapp, Fatu Djabi
EPI_ISL_739919, EPI_ISL_739920, EPI_ISL_739921, EPI_ISL_739922, EPI_ISL_739923, EPI_ISL_739924, EPI_ISL_739925, EPI_ISL_739926, EPI_ISL_739927, EPI_ISL_739928, EPI_ISL_739929, EPI_ISL_739930, EPI_ISL_739931, EPI_ISL_739932, EPI_ISL_739933, EPI_ISL_739934, EPI_ISL_739935, EPI_ISL_739936, EPI_ISL_739937, EPI_ISL_739938, EPI_ISL_739939, EPI_ISL_739940, EPI_ISL_739941, EPI_ISL_739942, EPI_ISL_739943, EPI_ISL_739944, EPI_ISL_739945, EPI_ISL_739946, EPI_ISL_739948, EPI_ISL_739949, EPI_ISL_739950, EPI_ISL_739951, EPI_ISL_739952, EPI_ISL_739953, EPI_ISL_739954, EPI_ISL_739955, EPI_ISL_739956, EPI_ISL_739957, EPI_ISL_739958, EPI_ISL_739959, EPI_ISL_739960, EPI_ISL_739962, EPI_ISL_739963, EPI_ISL_739964, EPI_ISL_739965, EPI_ISL_739966, EPI_ISL_739967, EPI_ISL_739968, EPI_ISL_739969, EPI_ISL_739970, EPI_ISL_739971, EPI_ISL_739972, EPI_ISL_739973, EPI_ISL_739974, EPI_ISL_739975, EPI_ISL_739976			
see above	Laboratoire national de santé, Microbiology, Virology	Laboratoire national de santé, Microbiology, Microbial Genomics Platform	Anke Wienecke-Baldacchino, Catherine Ragimbeau,Jessica Tapp, Fatu Djabi, Lise Pignon, Raoul Salmon, Tamir Abdelrahman
EPI_ISL_739977, EPI_ISL_739978	Laboratoire national de santé, Microbiology, Virology	Laboratoire national de santé, Microbiology, Microbial Genomics Platform	Anke Wienecke-Baldacchino, Catherine Ragimbeau, Tamir Abdelrahman, Jessica Tapp, Fatu Djabi
EPI_ISL_739979, EPI_ISL_739980, EPI_ISL_739981, EPI_ISL_739982, EPI_ISL_739983, EPI_ISL_739984, EPI_ISL_739985, EPI_ISL_739986, EPI_ISL_739987, EPI_ISL_739988, EPI_ISL_739989, EPI_ISL_739990, EPI_ISL_739991, EPI_ISL_739992, EPI_ISL_739993, EPI_ISL_739994			
see above	Laboratoire national de santé, Microbiology, Virology	Laboratoire national de santé, Microbiology, Microbial Genomics Platform	Anke Wienecke-Baldacchino, Catherine Ragimbeau,Jessica Tapp, Fatu Djabi, Lise Pignon, Raoul Salmon, Tamir Abdelrahman
EPI_ISL_739995	Laboratoire national de santé, Microbiology, Virology	Laboratoire national de santé, Microbiology, Microbial Genomics Platform	Anke Wienecke-Baldacchino, Catherine Ragimbeau, Tamir Abdelrahman, Jessica Tapp, Fatu Djabi
EPI_ISL_739996, EPI_ISL_739997, EPI_ISL_739998, EPI_ISL_739999, EPI_ISL_740000, EPI_ISL_740001, EPI_ISL_740002, EPI_ISL_740003, EPI_ISL_740004	Laboratoire national de santé, Microbiology, Virology	Laboratoire national de santé, Microbiology, Microbial Genomics Platform	Anke Wienecke-Baldacchino, Catherine Ragimbeau,Jessica Tapp, Fatu Djabi, Lise Pignon, Raoul Salmon, Tamir Abdelrahman
EPI_ISL_740005	Laboratoire national de santé, Microbiology, Virology	Laboratoire national de santé, Microbiology, Microbial Genomics Platform	Anke Wienecke-Baldacchino, Catherine Ragimbeau, Tamir Abdelrahman, Jessica Tapp, Fatu Djabi
EPI_ISL_740006, EPI_ISL_740007, EPI_ISL_740008, EPI_ISL_740009, EPI_ISL_740010, EPI_ISL_740011, EPI_ISL_740012, EPI_ISL_740013, EPI_ISL_740014, EPI_ISL_740015, EPI_ISL_740016, EPI_ISL_740017, EPI_ISL_740018, EPI_ISL_740019, EPI_ISL_740020, EPI_ISL_740021, EPI_ISL_740022, EPI_ISL_740023, EPI_ISL_740024, EPI_ISL_740025, EPI_ISL_740026, EPI_ISL_740027, EPI_ISL_740028, EPI_ISL_740029			
see above	Laboratoire national de santé, Microbiology, Virology	Laboratoire national de santé, Microbiology, Microbial Genomics Platform	Anke Wienecke-Baldacchino, Catherine Ragimbeau,Jessica Tapp, Fatu Djabi, Lise Pignon, Raoul Salmon, Tamir Abdelrahman
EPI_ISL_740030	Laboratoire national de santé, Microbiology, Virology	Laboratoire national de santé, Microbiology, Microbial Genomics Platform	Anke Wienecke-Baldacchino, Catherine Ragimbeau, Tamir Abdelrahman, Jessica Tapp, Fatu Djabi
EPI_ISL_740031	Laboratoire national de santé, Microbiology, Virology	Laboratoire national de santé, Microbiology, Microbial Genomics Platform	Anke Wienecke-Baldacchino, Catherine Ragimbeau,Jessica Tapp, Fatu Djabi, Lise Pignon, Raoul Salmon, Tamir Abdelrahman
EPI_ISL_740032	Laboratoire national de santé, Microbiology, Virology	Laboratoire national de santé, Microbiology, Microbial Genomics Platform	Anke Wienecke-Baldacchino, Catherine Ragimbeau, Tamir Abdelrahman, Jessica Tapp, Fatu Djabi
EPI_ISL_740033, EPI_ISL_740034, EPI_ISL_740035, EPI_ISL_740036, EPI_ISL_740037, EPI_ISL_740038, EPI_ISL_740039, EPI_ISL_740040, EPI_ISL_740041, EPI_ISL_740042, EPI_ISL_740043, EPI_ISL_740044, EPI_ISL_740045, EPI_ISL_740046, EPI_ISL_740047			
see above	Laboratoire national de santé, Microbiology, Virology	Laboratoire national de santé, Microbiology, Microbial Genomics Platform	Anke Wienecke-Baldacchino, Catherine Ragimbeau,Jessica Tapp, Fatu Djabi, Lise Pignon, Raoul Salmon, Tamir Abdelrahman
EPI_ISL_740048	Laboratoire national de santé, Microbiology, Virology	Laboratoire national de santé, Microbiology, Microbial Genomics Platform	Anke Wienecke-Baldacchino, Catherine Ragimbeau, Tamir Abdelrahman, Jessica Tapp, Fatu Djabi
EPI_ISL_740049, EPI_ISL_740050	Laboratoire national de santé, Microbiology, Virology	Laboratoire national de santé, Microbiology, Microbial Genomics Platform	Anke Wienecke-Baldacchino, Catherine Ragimbeau,Jessica Tapp, Fatu Djabi, Lise Pignon, Raoul Salmon, Tamir Abdelrahman
EPI_ISL_740051	Laboratoire national de santé, Microbiology, Virology	Laboratoire national de santé, Microbiology, Microbial Genomics Platform	Anke Wienecke-Baldacchino, Catherine Ragimbeau, Tamir Abdelrahman, Jessica Tapp, Fatu Djabi
EPI_ISL_740052, EPI_ISL_740053, EPI_ISL_740054, EPI_ISL_740055, EPI_ISL_740056, EPI_ISL_740057, EPI_ISL_740058, EPI_ISL_740059, EPI_ISL_740060, EPI_ISL_740061	Laboratoire national de santé, Microbiology, Virology	Laboratoire national de santé, Microbiology, Microbial Genomics Platform	Anke Wienecke-Baldacchino, Catherine Ragimbeau,Jessica Tapp, Fatu Djabi, Lise Pignon, Raoul Salmon, Tamir Abdelrahman
EPI_ISL_740062	Laboratoire national de santé, Microbiology, Virology	Laboratoire national de santé, Microbiology, Microbial Genomics Platform	Anke Wienecke-Baldacchino, Catherine Ragimbeau, Tamir Abdelrahman, Jessica Tapp, Fatu Djabi
EPI_ISL_740063, EPI_ISL_740064, EPI_ISL_740065, EPI_ISL_740066, EPI_ISL_740067, EPI_ISL_740068, EPI_ISL_740069, EPI_ISL_740070, EPI_ISL_740071, EPI_ISL_740072, EPI_ISL_740073, EPI_ISL_740074, EPI_ISL_740075, EPI_ISL_740076, EPI_ISL_740077, EPI_ISL_740078			
see above	Laboratoire national de santé, Microbiology, Virology	Laboratoire national de santé, Microbiology, Microbial Genomics Platform	Anke Wienecke-Baldacchino, Catherine Ragimbeau,Jessica Tapp, Fatu Djabi, Lise Pignon, Raoul Salmon, Tamir Abdelrahman
EPI_ISL_740079	Laboratoire national de santé, Microbiology, Virology	Laboratoire national de santé, Microbiology, Microbial Genomics Platform	Anke Wienecke-Baldacchino, Catherine Ragimbeau, Tamir Abdelrahman, Jessica Tapp, Fatu Djabi
EPI_ISL_740080, EPI_ISL_740081, EPI_ISL_740082, EPI_ISL_740083, EPI_ISL_740084, EPI_ISL_740085, EPI_ISL_740086, EPI_ISL_740087, EPI_ISL_740088, EPI_ISL_740089, EPI_ISL_740090, EPI_ISL_740091, EPI_ISL_740092, EPI_ISL_740093, EPI_ISL_740094, EPI_ISL_740095, EPI_ISL_740096, EPI_ISL_740097			
see above	Laboratoire national de santé, Microbiology, Virology	Laboratoire national de santé, Microbiology, Microbial Genomics Platform	Anke Wienecke-Baldacchino, Catherine Ragimbeau,Jessica Tapp, Fatu Djabi, Lise Pignon, Raoul Salmon, Tamir Abdelrahman
EPI_ISL_740098	Laboratoire national de santé, Microbiology, Virology	Laboratoire national de santé, Microbiology, Microbial Genomics Platform	Anke Wienecke-Baldacchino, Catherine Ragimbeau, Tamir Abdelrahman, Jessica Tapp, Fatu Djabi
EPI_ISL_740099, EPI_ISL_740100, EPI_ISL_740101, EPI_ISL_740102	Laboratoire national de santé, Microbiology, Virology	Laboratoire national de santé, Microbiology, Microbial Genomics Platform	Anke Wienecke-Baldacchino, Catherine Ragimbeau,Jessica Tapp, Fatu Djabi, Lise Pignon, Raoul Salmon, Tamir Abdelrahman
EPI_ISL_740103	Laboratoire national de santé, Microbiology, Virology	Laboratoire national de santé, Microbiology, Microbial Genomics Platform	Anke Wienecke-Baldacchino, Catherine Ragimbeau, Tamir Abdelrahman, Jessica Tapp, Fatu Djabi
EPI_ISL_740104, EPI_ISL_740105, EPI_ISL_740106, EPI_ISL_740107, EPI_ISL_740108, EPI_ISL_740109, EPI_ISL_740110, EPI_ISL_740111, EPI_ISL_740112, EPI_ISL_740113, EPI_ISL_740114, EPI_ISL_740115, EPI_ISL_740116, EPI_ISL_740117, EPI_ISL_740118, EPI_ISL_740119, EPI_ISL_740120, EPI_ISL_740121, EPI_ISL_740122, EPI_ISL_740123, EPI_ISL_740124, EPI_ISL_740126			
see above	Laboratoire national de santé, Microbiology, Virology	Laboratoire national de santé, Microbiology, Microbial Genomics Platform	Anke Wienecke-Baldacchino, Catherine Ragimbeau,Jessica Tapp, Fatu Djabi, Lise Pignon, Raoul Salmon, Tamir Abdelrahman
EPI_ISL_740127	Laboratoire national de santé, Microbiology, Virology	Laboratoire national de santé, Microbiology, Microbial Genomics Platform	Anke Wienecke-Baldacchino, Catherine Ragimbeau, Tamir Abdelrahman, Jessica Tapp, Fatu Djabi
EPI_ISL_740128, EPI_ISL_740130, EPI_ISL_740131	Laboratoire national de santé, Microbiology, Virology	Laboratoire national de santé, Microbiology, Microbial Genomics Platform	Anke Wienecke-Baldacchino, Catherine Ragimbeau,Jessica Tapp, Fatu Djabi, Lise Pignon, Raoul Salmon, Tamir Abdelrahman

EPI_ISL_740132	Laboratoire national de santé, Microbiology, Virology	Laboratoire national de santé, Microbiology, Microbial Genomics Platform	Anke Wienecke-Baldacchino, Catherine Ragimbeau, Tamir Abdelrahman, Jessica Tapp, Fatu Djabi
EPI_ISL_740133, EPI_ISL_740134, EPI_ISL_740135, EPI_ISL_740136, EPI_ISL_740137, EPI_ISL_740138, EPI_ISL_740139, EPI_ISL_740140, EPI_ISL_740141, EPI_ISL_740142, EPI_ISL_740143, EPI_ISL_740144, EPI_ISL_740145, EPI_ISL_740146, EPI_ISL_740147, EPI_ISL_740148, EPI_ISL_740149, EPI_ISL_740150, EPI_ISL_740151, EPI_ISL_740152, EPI_ISL_740153, EPI_ISL_740154, EPI_ISL_740155, EPI_ISL_740156, EPI_ISL_740158, EPI_ISL_740159, EPI_ISL_740160, EPI_ISL_740161, EPI_ISL_740162, EPI_ISL_740163, EPI_ISL_740164, EPI_ISL_740165, EPI_ISL_740166, EPI_ISL_740167, EPI_ISL_740168, EPI_ISL_740169, EPI_ISL_740170, EPI_ISL_740171, EPI_ISL_740172, EPI_ISL_740173, EPI_ISL_740174, EPI_ISL_740175, EPI_ISL_740176, EPI_ISL_740177, EPI_ISL_740178, EPI_ISL_740179, EPI_ISL_740180, EPI_ISL_740181, EPI_ISL_740183, EPI_ISL_740184, EPI_ISL_740185			
see above	Laboratoire national de santé, Microbiology, Virology	Laboratoire national de santé, Microbiology, Microbial Genomics Platform	Anke Wienecke-Baldacchino, Catherine Ragimbeau,Jessica Tapp, Fatu Djabi, Lise Pignon, Raoul Salmon, Tamir Abdelrahman
EPI_ISL_740186	Laboratoire national de santé, Microbiology, Virology	Laboratoire national de santé, Microbiology, Microbial Genomics Platform	Anke Wienecke-Baldacchino, Catherine Ragimbeau, Tamir Abdelrahman, Jessica Tapp, Fatu Djabi
EPI_ISL_740187, EPI_ISL_740188, EPI_ISL_740189, EPI_ISL_740190, EPI_ISL_740191, EPI_ISL_740192, EPI_ISL_740193, EPI_ISL_740194, EPI_ISL_740195, EPI_ISL_740196, EPI_ISL_740197, EPI_ISL_740198, EPI_ISL_740200, EPI_ISL_740201, EPI_ISL_740203			
see above	Laboratoire national de santé, Microbiology, Virology	Laboratoire national de santé, Microbiology, Microbial Genomics Platform	Anke Wienecke-Baldacchino, Catherine Ragimbeau,Jessica Tapp, Fatu Djabi, Lise Pignon, Raoul Salmon, Tamir Abdelrahman
EPI_ISL_740204	Laboratoire national de santé, Microbiology, Virology	Laboratoire national de santé, Microbiology, Microbial Genomics Platform	Anke Wienecke-Baldacchino, Catherine Ragimbeau, Tamir Abdelrahman, Jessica Tapp, Fatu Djabi
EPI_ISL_740205, EPI_ISL_740206, EPI_ISL_740207, EPI_ISL_740208, EPI_ISL_740209, EPI_ISL_740210, EPI_ISL_740211, EPI_ISL_740212, EPI_ISL_740213, EPI_ISL_740214, EPI_ISL_740215, EPI_ISL_740216, EPI_ISL_740217, EPI_ISL_740218, EPI_ISL_740219, EPI_ISL_740220, EPI_ISL_740221, EPI_ISL_740222, EPI_ISL_740223, EPI_ISL_740224, EPI_ISL_740225, EPI_ISL_740226			
see above	Laboratoire national de santé, Microbiology, Virology	Laboratoire national de santé, Microbiology, Microbial Genomics Platform	Anke Wienecke-Baldacchino, Catherine Ragimbeau,Jessica Tapp, Fatu Djabi, Lise Pignon, Raoul Salmon, Tamir Abdelrahman
EPI_ISL_740227	Laboratoire national de santé, Microbiology, Virology	Laboratoire national de santé, Microbiology, Microbial Genomics Platform	Anke Wienecke-Baldacchino, Catherine Ragimbeau, Tamir Abdelrahman, Jessica Tapp, Fatu Djabi
EPI_ISL_740228, EPI_ISL_740229, EPI_ISL_740230, EPI_ISL_740231, EPI_ISL_740232, EPI_ISL_740233, EPI_ISL_740234, EPI_ISL_740235, EPI_ISL_740236, EPI_ISL_740237, EPI_ISL_740238, EPI_ISL_740239, EPI_ISL_740240, EPI_ISL_740241, EPI_ISL_740243, EPI_ISL_740244, EPI_ISL_740245, EPI_ISL_740247, EPI_ISL_740248, EPI_ISL_740249			
see above	Laboratoire national de santé, Microbiology, Virology	Laboratoire national de santé, Microbiology, Microbial Genomics Platform	Anke Wienecke-Baldacchino, Catherine Ragimbeau,Jessica Tapp, Fatu Djabi, Lise Pignon, Raoul Salmon, Tamir Abdelrahman
EPI_ISL_740251	Laboratoire national de santé, Microbiology, Virology	Laboratoire national de santé, Microbiology, Microbial Genomics Platform	Anke Wienecke-Baldacchino, Catherine Ragimbeau, Tamir Abdelrahman, Jessica Tapp, Fatu Djabi
EPI_ISL_740252, EPI_ISL_740253, EPI_ISL_740254, EPI_ISL_740255, EPI_ISL_740256, EPI_ISL_740257, EPI_ISL_740258	Laboratoire national de santé, Microbiology, Virology	Laboratoire national de santé, Microbiology, Microbial Genomics Platform	Anke Wienecke-Baldacchino, Catherine Ragimbeau,Jessica Tapp, Fatu Djabi, Lise Pignon, Raoul Salmon, Tamir Abdelrahman
EPI_ISL_740259	Laboratoire national de santé, Microbiology, Virology	Laboratoire national de santé, Microbiology, Microbial Genomics Platform	Anke Wienecke-Baldacchino, Catherine Ragimbeau, Tamir Abdelrahman, Jessica Tapp, Fatu Djabi
EPI_ISL_740260, EPI_ISL_740261, EPI_ISL_740262, EPI_ISL_740263, EPI_ISL_740264, EPI_ISL_740265, EPI_ISL_740266, EPI_ISL_740267, EPI_ISL_740268, EPI_ISL_740269, EPI_ISL_740270, EPI_ISL_740271, EPI_ISL_740272, EPI_ISL_740273, EPI_ISL_740274, EPI_ISL_740275, EPI_ISL_740276, EPI_ISL_740277			
see above	Laboratoire national de santé, Microbiology, Virology	Laboratoire national de santé, Microbiology, Microbial Genomics Platform	Anke Wienecke-Baldacchino, Catherine Ragimbeau,Jessica Tapp, Fatu Djabi, Lise Pignon, Raoul Salmon, Tamir Abdelrahman
EPI_ISL_740278	Laboratoire national de santé, Microbiology, Virology	Laboratoire national de santé, Microbiology, Microbial Genomics Platform	Anke Wienecke-Baldacchino, Catherine Ragimbeau, Tamir Abdelrahman, Jessica Tapp, Fatu Djabi
EPI_ISL_740279, EPI_ISL_740280, EPI_ISL_740281, EPI_ISL_740282, EPI_ISL_740283, EPI_ISL_740284, EPI_ISL_740285, EPI_ISL_740286, EPI_ISL_740287, EPI_ISL_740288	Laboratoire national de santé, Microbiology, Virology	Laboratoire national de santé, Microbiology, Microbial Genomics Platform	Anke Wienecke-Baldacchino, Catherine Ragimbeau,Jessica Tapp, Fatu Djabi, Lise Pignon, Raoul Salmon, Tamir Abdelrahman
EPI_ISL_740289	Laboratoire national de santé, Microbiology, Virology	Laboratoire national de santé, Microbiology, Microbial Genomics Platform	Anke Wienecke-Baldacchino, Catherine Ragimbeau, Tamir Abdelrahman, Jessica Tapp, Fatu Djabi
EPI_ISL_740290, EPI_ISL_740291, EPI_ISL_740292, EPI_ISL_740293, EPI_ISL_740294, EPI_ISL_740295, EPI_ISL_740296, EPI_ISL_740297, EPI_ISL_740298, EPI_ISL_740299, EPI_ISL_740300, EPI_ISL_740301, EPI_ISL_740302, EPI_ISL_740303, EPI_ISL_740304, EPI_ISL_740305, EPI_ISL_740306, EPI_ISL_740307, EPI_ISL_740308, EPI_ISL_740309, EPI_ISL_740310, EPI_ISL_740311, EPI_ISL_740312, EPI_ISL_740313			
see above	Laboratoire national de santé, Microbiology, Virology	Laboratoire national de santé, Microbiology, Microbial Genomics Platform	Anke Wienecke-Baldacchino, Catherine Ragimbeau,Jessica Tapp, Fatu Djabi, Lise Pignon, Raoul Salmon, Tamir Abdelrahman
EPI_ISL_740314	Laboratoire national de santé, Microbiology, Virology	Laboratoire national de santé, Microbiology, Microbial Genomics Platform	Anke Wienecke-Baldacchino, Catherine Ragimbeau, Tamir Abdelrahman, Jessica Tapp, Fatu Djabi
EPI_ISL_740315, EPI_ISL_740316, EPI_ISL_740317, EPI_ISL_740318, EPI_ISL_740319, EPI_ISL_740320, EPI_ISL_740322, EPI_ISL_740323, EPI_ISL_740324, EPI_ISL_740325, EPI_ISL_740326, EPI_ISL_740327, EPI_ISL_740328, EPI_ISL_740329, EPI_ISL_740330, EPI_ISL_740331, EPI_ISL_740332, EPI_ISL_740333, EPI_ISL_740334, EPI_ISL_740335, EPI_ISL_740336, EPI_ISL_740337, EPI_ISL_740338, EPI_ISL_740339, EPI_ISL_740340, EPI_ISL_740341, EPI_ISL_740342			
see above	Laboratoire national de santé, Microbiology, Virology	Laboratoire national de santé, Microbiology, Microbial Genomics Platform	Anke Wienecke-Baldacchino, Catherine Ragimbeau,Jessica Tapp, Fatu Djabi, Lise Pignon, Raoul Salmon, Tamir Abdelrahman
EPI_ISL_740343	Laboratoire national de santé, Microbiology, Virology	Laboratoire national de santé, Microbiology, Microbial Genomics Platform	Anke Wienecke-Baldacchino, Catherine Ragimbeau, Tamir Abdelrahman, Jessica Tapp, Fatu Djabi
EPI_ISL_740344, EPI_ISL_740345, EPI_ISL_740346, EPI_ISL_740347, EPI_ISL_740348, EPI_ISL_740349, EPI_ISL_740350, EPI_ISL_740351, EPI_ISL_740352, EPI_ISL_740353, EPI_ISL_740354, EPI_ISL_740355, EPI_ISL_740356, EPI_ISL_740357, EPI_ISL_740358, EPI_ISL_740359			
see above	Laboratoire national de santé, Microbiology, Virology	Laboratoire national de santé, Microbiology, Microbial Genomics Platform	Anke Wienecke-Baldacchino, Catherine Ragimbeau,Jessica Tapp, Fatu Djabi, Lise Pignon, Raoul Salmon, Tamir Abdelrahman
EPI_ISL_740360	Laboratoire national de santé, Microbiology, Virology	Laboratoire national de santé, Microbiology, Microbial Genomics Platform	Anke Wienecke-Baldacchino, Catherine Ragimbeau, Tamir Abdelrahman, Jessica Tapp, Fatu Djabi
EPI_ISL_740361, EPI_ISL_740362, EPI_ISL_740363, EPI_ISL_740364, EPI_ISL_740365, EPI_ISL_740366, EPI_ISL_740367, EPI_ISL_740368, EPI_ISL_740369, EPI_ISL_740370, EPI_ISL_740371, EPI_ISL_740372, EPI_ISL_740373, EPI_ISL_740374, EPI_ISL_740375, EPI_ISL_740376, EPI_ISL_740377, EPI_ISL_740378, EPI_ISL_740379, EPI_ISL_740380, EPI_ISL_740381, EPI_ISL_740382, EPI_ISL_740383, EPI_ISL_740384, EPI_ISL_740385, EPI_ISL_740386, EPI_ISL_740387, EPI_ISL_740388, EPI_ISL_740389, EPI_ISL_740390, EPI_ISL_740391, EPI_ISL_740392, EPI_ISL_740393, EPI_ISL_740394, EPI_ISL_740395, EPI_ISL_740396, EPI_ISL_740397, EPI_ISL_740398, EPI_ISL_740399, EPI_ISL_740400, EPI_ISL_740401, EPI_ISL_740402, EPI_ISL_740403			
see above	Laboratoire national de santé, Microbiology, Virology	Laboratoire national de santé, Microbiology, Microbial Genomics Platform	Anke Wienecke-Baldacchino, Catherine Ragimbeau,Jessica Tapp, Fatu Djabi, Lise Pignon, Raoul Salmon, Tamir Abdelrahman
EPI_ISL_740404	Laboratoire national de santé, Microbiology, Virology	Laboratoire national de santé, Microbiology, Microbial Genomics Platform	Anke Wienecke-Baldacchino, Catherine Ragimbeau, Tamir Abdelrahman, Jessica Tapp, Fatu Djabi
EPI_ISL_740406, EPI_ISL_740407, EPI_ISL_740408, EPI_ISL_740409, EPI_ISL_740410, EPI_ISL_740411, EPI_ISL_740412, EPI_ISL_740413, EPI_ISL_740414, EPI_ISL_740415, EPI_ISL_740416, EPI_ISL_740417, EPI_ISL_740418, EPI_ISL_740419, EPI_ISL_740420, EPI_ISL_740421, EPI_ISL_740422, EPI_ISL_740423, EPI_ISL_740424, EPI_ISL_740425, EPI_ISL_740426, EPI_ISL_740427, EPI_ISL_740428			
see above	Laboratoire national de santé, Microbiology, Virology	Laboratoire national de santé, Microbiology, Microbial	Anke Wienecke-Baldacchino, Catherine Ragimbeau,Jessica Tapp, Fatu Djabi, Lise Pignon, Raoul Salmon, Tamir Abdelrahman

		Genomics Platform	
EPI_ISL_740430	Laboratoire national de santé, Microbiology, Virology	Laboratoire national de santé, Microbiology, Microbial Genomics Platform	Anke Wienecke-Baldacchino, Catherine Ragimbeau, Tamir Abdelrahman, Jessica Tapp, Fatu Djabi
EPI_ISL_740431, EPI_ISL_740432, EPI_ISL_740433, EPI_ISL_740434, EPI_ISL_740435, EPI_ISL_740436, EPI_ISL_740437	Laboratoire national de santé, Microbiology, Virology	Laboratoire national de santé, Microbiology, Microbial Genomics Platform	Anke Wienecke-Baldacchino, Catherine Ragimbeau,Jessica Tapp, Fatu Djabi, Lise Pignon, Raoul Salmon, Tamir Abdelrahman
EPI_ISL_740438	Laboratoire national de santé, Microbiology, Virology	Laboratoire national de santé, Microbiology, Microbial Genomics Platform	Anke Wienecke-Baldacchino, Catherine Ragimbeau, Tamir Abdelrahman, Jessica Tapp, Fatu Djabi
EPI_ISL_740441, EPI_ISL_740442, EPI_ISL_740443, EPI_ISL_740445, EPI_ISL_740446, EPI_ISL_740447, EPI_ISL_740448, EPI_ISL_740449, EPI_ISL_740450, EPI_ISL_740451, EPI_ISL_740452, EPI_ISL_740453, EPI_ISL_740454, EPI_ISL_740455			
see above	Laboratoire national de santé, Microbiology, Virology	Laboratoire national de santé, Microbiology, Microbial Genomics Platform	Anke Wienecke-Baldacchino, Catherine Ragimbeau,Jessica Tapp, Fatu Djabi, Lise Pignon, Raoul Salmon, Tamir Abdelrahman
EPI_ISL_740456	Laboratoire national de santé, Microbiology, Virology	Laboratoire national de santé, Microbiology, Microbial Genomics Platform	Anke Wienecke-Baldacchino, Catherine Ragimbeau, Tamir Abdelrahman, Jessica Tapp, Fatu Djabi
EPI_ISL_740457, EPI_ISL_740458, EPI_ISL_740459, EPI_ISL_740460, EPI_ISL_740461, EPI_ISL_740462, EPI_ISL_740463, EPI_ISL_740464, EPI_ISL_740466, EPI_ISL_740467, EPI_ISL_740468			
see above	Laboratoire national de santé, Microbiology, Virology	Laboratoire national de santé, Microbiology, Microbial Genomics Platform	Anke Wienecke-Baldacchino, Catherine Ragimbeau,Jessica Tapp, Fatu Djabi, Lise Pignon, Raoul Salmon, Tamir Abdelrahman
EPI_ISL_740469	Laboratoire national de santé, Microbiology, Virology	Laboratoire national de santé, Microbiology, Microbial Genomics Platform	Anke Wienecke-Baldacchino, Catherine Ragimbeau, Tamir Abdelrahman, Jessica Tapp, Fatu Djabi
EPI_ISL_740470	Laboratoire national de santé, Microbiology, Virology	Laboratoire national de santé, Microbiology, Microbial Genomics Platform	Anke Wienecke-Baldacchino, Catherine Ragimbeau,Jessica Tapp, Fatu Djabi, Lise Pignon, Raoul Salmon, Tamir Abdelrahman
EPI_ISL_740471	Laboratoire national de santé, Microbiology, Virology	Laboratoire national de santé, Microbiology, Microbial Genomics Platform	Anke Wienecke-Baldacchino, Catherine Ragimbeau, Tamir Abdelrahman, Jessica Tapp, Fatu Djabi
EPI_ISL_740472, EPI_ISL_740473, EPI_ISL_740474, EPI_ISL_740475, EPI_ISL_740476, EPI_ISL_740477, EPI_ISL_740478, EPI_ISL_740479, EPI_ISL_740480, EPI_ISL_740481, EPI_ISL_740482, EPI_ISL_740483, EPI_ISL_740484, EPI_ISL_740485			
see above	Laboratoire national de santé, Microbiology, Virology	Laboratoire national de santé, Microbiology, Microbial Genomics Platform	Anke Wienecke-Baldacchino, Catherine Ragimbeau,Jessica Tapp, Fatu Djabi, Lise Pignon, Raoul Salmon, Tamir Abdelrahman
EPI_ISL_740486	Laboratoire national de santé, Microbiology, Virology	Laboratoire national de santé, Microbiology, Microbial Genomics Platform	Anke Wienecke-Baldacchino, Catherine Ragimbeau, Tamir Abdelrahman, Jessica Tapp, Fatu Djabi
EPI_ISL_740487, EPI_ISL_740488, EPI_ISL_740489, EPI_ISL_740490, EPI_ISL_740491, EPI_ISL_740492, EPI_ISL_740493, EPI_ISL_740494, EPI_ISL_740495, EPI_ISL_740496, EPI_ISL_740497, EPI_ISL_740498, EPI_ISL_740499, EPI_ISL_740500, EPI_ISL_740501, EPI_ISL_740502, EPI_ISL_740503, EPI_ISL_740504, EPI_ISL_740505, EPI_ISL_740506, EPI_ISL_740507, EPI_ISL_740508, EPI_ISL_740509, EPI_ISL_740511, EPI_ISL_740512, EPI_ISL_740513, EPI_ISL_740514, EPI_ISL_740515, EPI_ISL_740516, EPI_ISL_740517, EPI_ISL_740518, EPI_ISL_740519, EPI_ISL_740520, EPI_ISL_740521, EPI_ISL_740522, EPI_ISL_740523, EPI_ISL_740524, EPI_ISL_740525, EPI_ISL_740526, EPI_ISL_740527, EPI_ISL_740528, EPI_ISL_740529, EPI_ISL_740530, EPI_ISL_740531, EPI_ISL_740532, EPI_ISL_740533, EPI_ISL_740534, EPI_ISL_740535, EPI_ISL_740536, EPI_ISL_740537, EPI_ISL_740538, EPI_ISL_740539, EPI_ISL_740540, EPI_ISL_740541, EPI_ISL_740542, EPI_ISL_740543, EPI_ISL_740544, EPI_ISL_740545, EPI_ISL_740546			
see above	Laboratoire national de santé, Microbiology, Virology	Laboratoire national de santé, Microbiology, Microbial Genomics Platform	Anke Wienecke-Baldacchino, Catherine Ragimbeau,Jessica Tapp, Fatu Djabi, Lise Pignon, Raoul Salmon, Tamir Abdelrahman
EPI_ISL_740547	Department of Laboratory Medicine, National Taiwan University Hospital	Microbial Genomics Core Lab, National Taiwan University Centers of Genomic and Precision Medicine	Shiou-Hwei Yeh, You-Yu Lin, Ya-Yun Lai, Chiao-Ling Li, Shan-Chwen Chang, Pei-Jer Chen, Sui-Yuan Chang
EPI_ISL_740863, EPI_ISL_740864, EPI_ISL_740865	South Eastern Area Laboratory Services (SEALS)	NSW Health Pathology - Institute of Clinical Pathology and Medical Research; Westmead Hospital; University of Sydney	CIDM-PH et al.
EPI_ISL_740866	Pathology North - Royal North Shore Hospital - NSW Health Pathology	NSW Health Pathology - Institute of Clinical Pathology and Medical Research; Westmead Hospital; University of Sydney	CIDM-PH et al.
EPI_ISL_740867, EPI_ISL_740868, EPI_ISL_740869, EPI_ISL_740870, EPI_ISL_740871, EPI_ISL_740872, EPI_ISL_740873, EPI_ISL_740874	South Eastern Area Laboratory Services (SEALS)	NSW Health Pathology - Institute of Clinical Pathology and Medical Research; Westmead Hospital; University of Sydney	CIDM-PH et al.
EPI_ISL_740875, EPI_ISL_740876, EPI_ISL_740877, EPI_ISL_740878	Pathology North - Royal North Shore Hospital - NSW Health Pathology	NSW Health Pathology - Institute of Clinical Pathology and Medical Research; Westmead Hospital; University of Sydney	CIDM-PH et al.
EPI_ISL_740879, EPI_ISL_740880	Histopath	NSW Health Pathology - Institute of Clinical Pathology and Medical Research; Westmead Hospital; University of Sydney	CIDM-PH et al.
EPI_ISL_740881, EPI_ISL_740882	Douglass Hanly Moir Pathology	NSW Health Pathology - Institute of Clinical Pathology and Medical Research; Westmead Hospital; University of Sydney	CIDM-PH et al.
EPI_ISL_740883, EPI_ISL_740884, EPI_ISL_740885, EPI_ISL_740886, EPI_ISL_740887, EPI_ISL_740888, EPI_ISL_740889	Pathology North - Royal North Shore Hospital - NSW Health Pathology	NSW Health Pathology - Institute of Clinical Pathology and Medical Research; Westmead Hospital; University of Sydney	CIDM-PH et al.
EPI_ISL_740890	Pathology West - NSW Health Pathology	NSW Health Pathology - Institute of Clinical Pathology and Medical Research; Westmead Hospital; University of Sydney	CIDM-PH et al.
EPI_ISL_740891	Pathology North - Hunter - NSW Health Pathology	NSW Health Pathology - Institute of Clinical Pathology and Medical Research; Westmead Hospital; University of Sydney	CIDM-PH et al.
EPI_ISL_740892, EPI_ISL_740893	Laverty Pathology	NSW Health Pathology - Institute of Clinical Pathology and Medical Research; Westmead Hospital; University of Sydney	CIDM-PH et al.
EPI_ISL_740894, EPI_ISL_740895	Sydney South West Pathology Service (SSWPS) - Royal Prince Alfred Hospital - NSW Health Pathology	NSW Health Pathology - Institute of Clinical Pathology and Medical Research; Westmead Hospital; University of Sydney	CIDM-PH et al.
EPI_ISL_740896	Histopath	NSW Health Pathology - Institute of Clinical Pathology and Medical Research; Westmead Hospital; University of Sydney	CIDM-PH et al.
EPI_ISL_740897, EPI_ISL_740898, EPI_ISL_740899, EPI_ISL_740900, EPI_ISL_740901, EPI_ISL_740902, EPI_ISL_740903, EPI_ISL_740904, EPI_ISL_740905, EPI_ISL_740906, EPI_ISL_740907, EPI_ISL_740908, EPI_ISL_740909, EPI_ISL_740910, EPI_ISL_740911, EPI_ISL_740912, EPI_ISL_740913, EPI_ISL_740914, EPI_ISL_740915, EPI_ISL_740916, EPI_ISL_740917, EPI_ISL_740918, EPI_ISL_740919, EPI_ISL_740920, EPI_ISL_740921, EPI_ISL_740922, EPI_ISL_740923, EPI_ISL_740924, EPI_ISL_740925, EPI_ISL_740926, EPI_ISL_740927, EPI_ISL_740928, EPI_ISL_740929, EPI_ISL_740930, EPI_ISL_740931, EPI_ISL_740932, EPI_ISL_740933, EPI_ISL_740934, EPI_ISL_740935, EPI_ISL_740936, EPI_ISL_740937, EPI_ISL_740938, EPI_ISL_740939, EPI_ISL_740940, EPI_ISL_740941, EPI_ISL_740942, EPI_ISL_740943, EPI_ISL_740944, EPI_ISL_740945, EPI_ISL_740946, EPI_ISL_740947, EPI_ISL_740948, EPI_ISL_740949, EPI_ISL_740950, EPI_ISL_740951, EPI_ISL_740952			
see above	Oxford Viromics, NDM, University of Oxford; Oxford University Hospitals; Basingstoke and North Hampshire Hospital	COVID-19 Genomics UK (COG-UK) Consortium	Tanya Golubchik, David Bonsall, George Macintyre, Amy Trebes, Mariateresa de Cesare, Catrin Moore, Alex Mobbs, Anita Justice, Robert Shaw, Monique Andersson, Timothy Peto, Emma Wise, Nathan Moore, Jessica Lynch, Nick Cortes, Matilde Mori, Stephen Kidd, David Buck, John Todd, Christophe Fraser
EPI_ISL_740953, EPI_ISL_740954, EPI_ISL_740955, EPI_ISL_740956, EPI_ISL_740957, EPI_ISL_740958, EPI_ISL_740959, EPI_ISL_740961, EPI_ISL_740962, EPI_ISL_740963, EPI_ISL_740964, EPI_ISL_740965, EPI_ISL_740966, EPI_ISL_740967, EPI_ISL_740968, EPI_ISL_740969, EPI_ISL_740970, EPI_ISL_740972,			

EPI_ISL_740973, EPI_ISL_740974, EPI_ISL_740976, EPI_ISL_740977, EPI_ISL_740978, EPI_ISL_740979, EPI_ISL_740980, EPI_ISL_740981, EPI_ISL_740982, EPI_ISL_740984, EPI_ISL_740985, EPI_ISL_740990, EPI_ISL_740991, EPI_ISL_740993, EPI_ISL_740996, EPI_ISL_740997, EPI_ISL_740999, EPI_ISL_741000, EPI_ISL_741001, EPI_ISL_741002, EPI_ISL_741005, EPI_ISL_741006, EPI_ISL_741007, EPI_ISL_741008, EPI_ISL_741009, EPI_ISL_741010, EPI_ISL_741011, EPI_ISL_741012, EPI_ISL_741013, EPI_ISL_741014, EPI_ISL_741015, EPI_ISL_741016, EPI_ISL_741017			
see above	Department of Pathology, University of Cambridge	COVID-19 Genomics UK (COG-UK) Consortium	Aminu S. Jahun, Yasmin Chaudhry, Grant Hall, Iliana Georgana, Myra Hosmillo, Martin D. Curran, Malte Pinckert, Surendra Parmar, Ian Goodfellow
EPI_ISL_741018, EPI_ISL_741019, EPI_ISL_741020, EPI_ISL_741021, EPI_ISL_741022, EPI_ISL_741023, EPI_ISL_741024, EPI_ISL_741025, EPI_ISL_741026, EPI_ISL_741027, EPI_ISL_741028, EPI_ISL_741029, EPI_ISL_741030, EPI_ISL_741031, EPI_ISL_741032, EPI_ISL_741033, EPI_ISL_741034, EPI_ISL_741035, EPI_ISL_741036, EPI_ISL_741037, EPI_ISL_741038, EPI_ISL_741039, EPI_ISL_741040, EPI_ISL_741041, EPI_ISL_741042, EPI_ISL_741043, EPI_ISL_741044, EPI_ISL_741045, EPI_ISL_741046, EPI_ISL_741047, EPI_ISL_741048, EPI_ISL_741049, EPI_ISL_741050, EPI_ISL_741051, EPI_ISL_741052, EPI_ISL_741053, EPI_ISL_741054, EPI_ISL_741055, EPI_ISL_741056, EPI_ISL_741057, EPI_ISL_741058, EPI_ISL_741059, EPI_ISL_741060, EPI_ISL_741061, EPI_ISL_741062, EPI_ISL_741063, EPI_ISL_741064, EPI_ISL_741065, EPI_ISL_741066, EPI_ISL_741067, EPI_ISL_741068, EPI_ISL_741069, EPI_ISL_741070, EPI_ISL_741071, EPI_ISL_741072, EPI_ISL_741073, EPI_ISL_741074, EPI_ISL_741075, EPI_ISL_741076, EPI_ISL_741077, EPI_ISL_741078, EPI_ISL_741079, EPI_ISL_741080, EPI_ISL_741081, EPI_ISL_741082, EPI_ISL_741083, EPI_ISL_741084, EPI_ISL_741085, EPI_ISL_741086, EPI_ISL_741087, EPI_ISL_741088, EPI_ISL_741089, EPI_ISL_741090, EPI_ISL_741091, EPI_ISL_741092, EPI_ISL_741093, EPI_ISL_741094, EPI_ISL_741095, EPI_ISL_741096, EPI_ISL_741097, EPI_ISL_741098, EPI_ISL_741099, EPI_ISL_741100, EPI_ISL_741101, EPI_ISL_741102, EPI_ISL_741103, EPI_ISL_741104, EPI_ISL_741105, EPI_ISL_741106, EPI_ISL_741107, EPI_ISL_741108, EPI_ISL_741109, EPI_ISL_741110, EPI_ISL_741111, EPI_ISL_741112, EPI_ISL_741113, EPI_ISL_741114, EPI_ISL_741115, EPI_ISL_741116, EPI_ISL_741117, EPI_ISL_741118, EPI_ISL_741119, EPI_ISL_741120, EPI_ISL_741121, EPI_ISL_741122, EPI_ISL_741123, EPI_ISL_741124, EPI_ISL_741125, EPI_ISL_741126, EPI_ISL_741127, EPI_ISL_741128, EPI_ISL_741129, EPI_ISL_741130, EPI_ISL_741131, EPI_ISL_741132, EPI_ISL_741133, EPI_ISL_741134, EPI_ISL_741135, EPI_ISL_741136, EPI_ISL_741137, EPI_ISL_741138, EPI_ISL_741139, EPI_ISL_741140, EPI_ISL_741141, EPI_ISL_741142, EPI_ISL_741143, EPI_ISL_741144, EPI_ISL_741145, EPI_ISL_741146, EPI_ISL_741147, EPI_ISL_741148, EPI_ISL_741149, EPI_ISL_741150, EPI_ISL_741151, EPI_ISL_741152, EPI_ISL_741153, EPI_ISL_741154, EPI_ISL_741155, EPI_ISL_741156, EPI_ISL_741157, EPI_ISL_741158, EPI_ISL_741159, EPI_ISL_741160, EPI_ISL_741161, EPI_ISL_741162, EPI_ISL_741163, EPI_ISL_741164, EPI_ISL_741165, EPI_ISL_741166, EPI_ISL_741167, EPI_ISL_741168, EPI_ISL_741169, EPI_ISL_741170, EPI_ISL_741171, EPI_ISL_741172, EPI_ISL_741173, EPI_ISL_741174, EPI_ISL_741175, EPI_ISL_741176, EPI_ISL_741177, EPI_ISL_741178, EPI_ISL_741179, EPI_ISL_741180, EPI_ISL_741181, EPI_ISL_741182, EPI_ISL_741183, EPI_ISL_741184, EPI_ISL_741185			
see above	Oxford Viromics, NDM, University of Oxford; Oxford University Hospitals; Basingstoke and North Hampshire Hospital	COVID-19 Genomics UK (COG-UK) Consortium	Tanya Golubchik, David Bonsall, George Macintyre, Amy Trebes, Mariateresa de Cesare, Catrin Moore, Alex Mobbs, Anita Justice, Robert Shaw, Monique Andersson, Timothy Peto, Emma Wise, Nathan Moore, Jessica Lynch, Nick Cortes, Matilde Mori, Stephen Kidd, David Buck, John Todd, Christophe Fraser
EPI_ISL_741186, EPI_ISL_741187, EPI_ISL_741188, EPI_ISL_741189, EPI_ISL_741190, EPI_ISL_741191, EPI_ISL_741192, EPI_ISL_741193, EPI_ISL_741194, EPI_ISL_741195, EPI_ISL_741196, EPI_ISL_741197, EPI_ISL_741198, EPI_ISL_741199, EPI_ISL_741200, EPI_ISL_741201, EPI_ISL_741202, EPI_ISL_741203, EPI_ISL_741204, EPI_ISL_741205, EPI_ISL_741206, EPI_ISL_741207, EPI_ISL_741208, EPI_ISL_741209, EPI_ISL_741210, EPI_ISL_741211, EPI_ISL_741212, EPI_ISL_741213, EPI_ISL_741214, EPI_ISL_741215, EPI_ISL_741216, EPI_ISL_741217, EPI_ISL_741218, EPI_ISL_741219, EPI_ISL_741220, EPI_ISL_741221, EPI_ISL_741222, EPI_ISL_741223, EPI_ISL_741224, EPI_ISL_741225, EPI_ISL_741226, EPI_ISL_741227, EPI_ISL_741228, EPI_ISL_741229, EPI_ISL_741230, EPI_ISL_741231, EPI_ISL_741232, EPI_ISL_741233, EPI_ISL_741234, EPI_ISL_741235, EPI_ISL_741236, EPI_ISL_741237, EPI_ISL_741238, EPI_ISL_741239, EPI_ISL_741240, EPI_ISL_741241, EPI_ISL_741242, EPI_ISL_741243, EPI_ISL_741244, EPI_ISL_741245, EPI_ISL_741246, EPI_ISL_741247, EPI_ISL_741248, EPI_ISL_741249, EPI_ISL_741250, EPI_ISL_741251, EPI_ISL_741252, EPI_ISL_741253, EPI_ISL_741254, EPI_ISL_741257, EPI_ISL_741258, EPI_ISL_741259, EPI_ISL_741260, EPI_ISL_741261, EPI_ISL_741263, EPI_ISL_741264, EPI_ISL_741265, EPI_ISL_741266, EPI_ISL_741267, EPI_ISL_741268, EPI_ISL_741269, EPI_ISL_741270, EPI_ISL_741271, EPI_ISL_741272, EPI_ISL_741273, EPI_ISL_741274, EPI_ISL_741275, EPI_ISL_741276, EPI_ISL_741277, EPI_ISL_741278, EPI_ISL_741279, EPI_ISL_741280, EPI_ISL_741281, EPI_ISL_741282, EPI_ISL_741283, EPI_ISL_741284, EPI_ISL_741285, EPI_ISL_741286, EPI_ISL_741287, EPI_ISL_741289, EPI_ISL_741291, EPI_ISL_741292, EPI_ISL_741293, EPI_ISL_741294, EPI_ISL_741295, EPI_ISL_741296, EPI_ISL_741297, EPI_ISL_741298, EPI_ISL_741299, EPI_ISL_741300, EPI_ISL_741301, EPI_ISL_741302, EPI_ISL_741303, EPI_ISL_741304, EPI_ISL_741305, EPI_ISL_741306, EPI_ISL_741307, EPI_ISL_741308, EPI_ISL_741309, EPI_ISL_741310, EPI_ISL_741311, EPI_ISL_741313, EPI_ISL_741314, EPI_ISL_741315, EPI_ISL_741316, EPI_ISL_741317, EPI_ISL_741318, EPI_ISL_741319, EPI_ISL_741320, EPI_ISL_741321, EPI_ISL_741322, EPI_ISL_741323, EPI_ISL_741324, EPI_ISL_741325, EPI_ISL_741326, EPI_ISL_741327, EPI_ISL_741328, EPI_ISL_741329, EPI_ISL_741330, EPI_ISL_741331, EPI_ISL_741332, EPI_ISL_741334, EPI_ISL_741335, EPI_ISL_741336, EPI_ISL_741337, EPI_ISL_741338, EPI_ISL_741339, EPI_ISL_741340, EPI_ISL_741341, EPI_ISL_741342, EPI_ISL_741343, EPI_ISL_741344, EPI_ISL_741345, EPI_ISL_741346			
see above	University College London, Great Ormond Street Hospital for Children NHS Foundation Trust, Imperial College Healthcare NHS Trust	COVID-19 Genomics UK (COG-UK) Consortium	Sergi Castellano, Rachel Williams, Mark Kristiansen, Paola Resende Silva, Sunando Roy, Tony Brooks, Helena Tutill, Paola Niola, Patricia Dyal, Charlotte Williams, Leysa Forrest, Yasmin Panchbhaya, Jacqueline Findlay, Samuel Weeks, Julianne Brown, Kathryn Harris, Paul Randell, James Price, Alison Holmes, Judith Breuer
EPI_ISL_741347	Wales Specialist Virology Centre Sequencing lab: Pathogen Genomics Unit	COVID-19 Genomics UK (COG-UK) Consortium	Catherine Moore, Johnathan Evans, Laura Gifford, Malorie Perry, Simon Cottrell, Angela Marchbank, Alec Birchley, Alexander Adams, Amy Gaskin, Bree Gatica-Wilcox, Jason Coombes, Joel Southgate, Lauren Gilbert, Lee Graham, Nicole Pacchiarini, Sara Kumziene-Summerhayes, Sarah Taylor, Sophie Jones, Sara Rey, Matthew Bull, Joanne Watkins, Sally Corden, Tom Connor
EPI_ISL_741348	Centre for Enzyme Innovation, University of Portsmouth / Translational Research Laboratory, Portsmouth Hospitals NHS Trust	COVID-19 Genomics UK (COG-UK) Consortium	Angela Beckett,Yann Bourgeois,Garry Scarlett,Sharon Glaysher,Scott Elliott,Kelly Bicknell,Robert Impey,Allyson Lloyd,Sarah Wyllie,Ethan Butcher,Anoop Chauhan,Samuel Robson
EPI_ISL_741349, EPI_ISL_741350, EPI_ISL_741351, EPI_ISL_741352, EPI_ISL_741353, EPI_ISL_741354, EPI_ISL_741355, EPI_ISL_741356	Oxford Viromics, NDM, University of Oxford; Oxford University Hospitals; Basingstoke and North Hampshire Hospital	COVID-19 Genomics UK (COG-UK) Consortium	Tanya Golubchik, David Bonsall, George Macintyre, Amy Trebes, Mariateresa de Cesare, Catrin Moore, Alex Mobbs, Anita Justice, Robert Shaw, Monique Andersson, Timothy Peto, Emma Wise, Nathan Moore, Jessica Lynch, Nick Cortes, Matilde Mori, Stephen Kidd, David Buck, John Todd, Christophe Fraser
EPI_ISL_741357, EPI_ISL_741358	Centre for Enzyme Innovation, University of Portsmouth / Translational Research Laboratory, Portsmouth Hospitals NHS Trust	COVID-19 Genomics UK (COG-UK) Consortium	Angela Beckett,Yann Bourgeois,Garry Scarlett,Sharon Glaysher,Scott Elliott,Kelly Bicknell,Robert Impey,Allyson Lloyd,Sarah Wyllie,Ethan Butcher,Anoop Chauhan,Samuel Robson
EPI_ISL_741359, EPI_ISL_741360, EPI_ISL_741361	University of Exeter	COVID-19 Genomics UK (COG-UK) Consortium	Ben Temperton,Aaron Jeffries,Michelle Michelsen,Joanna Warwick-Dugdale,Audrey Farbos,Robyn Manley,Stephen Michell,Jane Masoli
EPI_ISL_741362, EPI_ISL_741363, EPI_ISL_741364, EPI_ISL_741365, EPI_ISL_741366, EPI_ISL_741367, EPI_ISL_741368, EPI_ISL_741369, EPI_ISL_741370, EPI_ISL_741371, EPI_ISL_741372, EPI_ISL_741373, EPI_ISL_741374, EPI_ISL_741375, EPI_ISL_741376, EPI_ISL_741377, EPI_ISL_741378, EPI_ISL_741379, EPI_ISL_741380, EPI_ISL_741381, EPI_ISL_741382, EPI_ISL_741383, EPI_ISL_741384, EPI_ISL_741385, EPI_ISL_741386, EPI_ISL_741387, EPI_ISL_741388, EPI_ISL_741389, EPI_ISL_741390, EPI_ISL_741391, EPI_ISL_741392, EPI_ISL_741393, EPI_ISL_741394, EPI_ISL_741395, EPI_ISL_741396, EPI_ISL_741397, EPI_ISL_741398, EPI_ISL_741399, EPI_ISL_741400, EPI_ISL_741401, EPI_ISL_741402, EPI_ISL_741403, EPI_ISL_741404, EPI_ISL_741405, EPI_ISL_741406, EPI_ISL_741407, EPI_ISL_741408, EPI_ISL_741409, EPI_ISL_741410, EPI_ISL_741411, EPI_ISL_741412, EPI_ISL_741413, EPI_ISL_741414, EPI_ISL_741415, EPI_ISL_741416, EPI_ISL_741417, EPI_ISL_741418, EPI_ISL_741419, EPI_ISL_741420			
see above	Oxford Viromics, NDM, University of Oxford; Oxford University Hospitals; Basingstoke and North Hampshire Hospital	COVID-19 Genomics UK (COG-UK) Consortium	Tanya Golubchik, David Bonsall, George Macintyre, Amy Trebes, Mariateresa de Cesare, Catrin Moore, Alex Mobbs, Anita Justice, Robert Shaw, Monique Andersson, Timothy Peto, Emma Wise, Nathan Moore, Jessica Lynch, Nick Cortes, Matilde Mori, Stephen Kidd, David Buck, John Todd, Christophe Fraser
EPI_ISL_741425, EPI_ISL_741426, EPI_ISL_741427, EPI_ISL_741428, EPI_ISL_741429, EPI_ISL_741430, EPI_ISL_741432, EPI_ISL_741433, EPI_ISL_741434, EPI_ISL_741435, EPI_ISL_741436, EPI_ISL_741437, EPI_ISL_741438, EPI_ISL_741439, EPI_ISL_741440, EPI_ISL_741441, EPI_ISL_741442, EPI_ISL_741443, EPI_ISL_741444, EPI_ISL_741445, EPI_ISL_741446, EPI_ISL_741447, EPI_ISL_741448, EPI_ISL_741449, EPI_ISL_741450, EPI_ISL_741451, EPI_ISL_741452			
see above	Northumbria University / South Tees Hospitals NHS Foundation Trust / North Cumbria Integrated Care NHS Foundation Trust / North Tees and Hartlepool NHS Foundation Trust / Newcastle Hospitals NHS Foundation Trust	COVID-19 Genomics UK (COG-UK) Consortium	Darren L Smith,Andrew Nelson,Matthew Bashton,Greg R Young,Joshua Loh,John Allan,Mohammad A Tariq,Giles S Holt,Gary Black,Wen C Yew,Lynn Dover,Paul Baker,Steve Liggett,Sarah Essex,Jane Greenaway,Debra Padgett,Clive Graham,Garren Scott,Edward Barton,Emma Swindells,Brendan Payne,Jennifer Collins,Yusri Taha,Gary Eltringham
EPI_ISL_741453, EPI_ISL_741454, EPI_ISL_741455, EPI_ISL_741456, EPI_ISL_741457, EPI_ISL_741458, EPI_ISL_741459, EPI_ISL_741460, EPI_ISL_741461, EPI_ISL_741462, EPI_ISL_741463, EPI_ISL_741464, EPI_ISL_741465, EPI_ISL_741466, EPI_ISL_741467, EPI_ISL_741468, EPI_ISL_741469, EPI_ISL_741470, EPI_ISL_741471, EPI_ISL_741472, EPI_ISL_741473, EPI_ISL_741474, EPI_ISL_741475, EPI_ISL_741476, EPI_ISL_741477, EPI_ISL_741478, EPI_ISL_741479, EPI_ISL_741480, EPI_ISL_741481, EPI_ISL_741482, EPI_ISL_741483, EPI_ISL_741484, EPI_ISL_741485, EPI_ISL_741486, EPI_ISL_741487, EPI_ISL_741488, EPI_ISL_741489, EPI_ISL_741490, EPI_ISL_741491, EPI_ISL_741492, EPI_ISL_741493, EPI_ISL_741494, EPI_ISL_741495, EPI_ISL_741496, EPI_ISL_741497, EPI_ISL_741498, EPI_ISL_741499, EPI_ISL_741500, EPI_ISL_741501, EPI_ISL_741502, EPI_ISL_741503, EPI_ISL_741505, EPI_ISL_741506, EPI_ISL_741507, EPI_ISL_741508, EPI_ISL_741510, EPI_ISL_741511, EPI_ISL_741512, EPI_ISL_741513, EPI_ISL_741514, EPI_ISL_741515, EPI_ISL_741516, EPI_ISL_741517, EPI_ISL_741518, EPI_ISL_741519, EPI_ISL_741520, EPI_ISL_741521, EPI_ISL_741524, EPI_ISL_741525, EPI_ISL_741526, EPI_ISL_741527, EPI_ISL_741528, EPI_ISL_741529, EPI_ISL_741530, EPI_ISL_741531, EPI_ISL_741532, EPI_ISL_741533, EPI_ISL_741534, EPI_ISL_741535, EPI_ISL_741536, EPI_ISL_741537, EPI_ISL_741538, EPI_ISL_741539, EPI_ISL_741540, EPI_ISL_741541, EPI_ISL_741542, EPI_ISL_741543, EPI_ISL_741544, EPI_ISL_741545, EPI_ISL_741546, EPI_ISL_741547, EPI_ISL_741548, EPI_ISL_741549, EPI_ISL_741550, EPI_ISL_741551, EPI_ISL_741552, EPI_ISL_741553, EPI_ISL_741554, EPI_ISL_741555, EPI_ISL_741556, EPI_ISL_741557, EPI_ISL_741558, EPI_ISL_741559, EPI_ISL_741560, EPI_ISL_741561, EPI_ISL_741562, EPI_ISL_741563, EPI_ISL_741564, EPI_ISL_741565, EPI_ISL_741566, EPI_ISL_741567, EPI_ISL_741568, EPI_ISL_741570, EPI_ISL_741571, EPI_ISL_741572, EPI_ISL_741573, EPI_ISL_741574, EPI_ISL_741575, EPI_ISL_741576, EPI_ISL_741577, EPI_ISL_741578, EPI_ISL_741579, EPI_ISL_741580, EPI_ISL_741581, EPI_ISL_741582, EPI_ISL_741584, EPI_ISL_741585, EPI_ISL_741586, EPI_ISL_741587, EPI_ISL_741588, EPI_ISL_741589, EPI_ISL_741590, EPI_ISL_741591, EPI_ISL_741593, EPI_ISL_741594, EPI_ISL_741595, EPI_ISL_741596, EPI_ISL_741597, EPI_ISL_741598, EPI_ISL_741599, EPI_ISL_741600, EPI_ISL_741601, EPI_ISL_741602, EPI_ISL_741603, EPI_ISL_741604, EPI_ISL_741605, EPI_ISL_741606, EPI_ISL_741607, EPI_ISL_741608			
see above	Quadram Institute Bioscience	COVID-19 Genomics UK (COG-UK) Consortium	Dave J. Baker, Gemma L. Kay, Alp Aydin, Thanh Le-Viet, Steven Rudder, Ana P. Tedim, Anastasia Kolyva, Maria Diaz, Leonardo de Oliveira Martins, Nabil-Fareed Alikhan, Lizzie Meadows, Rachael Stanley, Ngozi Elumogo, Muhammed Yasir, Nicholas M. Thomson, Alexander J Trotter, Rachel Gilroy, Samuel Bloomfield, Claire Stuart, Andrew Bell, Reenesh Prakash, Samir Dervisevic, Alison E. Mather, John Wain, Mark Webber, Andrew J. Page, Justin O'Grady
EPI_ISL_741609, EPI_ISL_741610, EPI_ISL_741611, EPI_ISL_741612, EPI_ISL_741614, EPI_ISL_741617, EPI_ISL_741618, EPI_ISL_741619, EPI_ISL_741620, EPI_ISL_741621, EPI_ISL_741622, EPI_ISL_741623, EPI_ISL_741624, EPI_ISL_741625, EPI_ISL_741626, EPI_ISL_741627, EPI_ISL_741628, EPI_ISL_741629, EPI_ISL_741630, EPI_ISL_741631, EPI_ISL_741632, EPI_ISL_741633, EPI_ISL_741634, EPI_ISL_741635, EPI_ISL_741636, EPI_ISL_741637, EPI_ISL_741638, EPI_ISL_741639, EPI_ISL_741640, EPI_ISL_741641, EPI_ISL_741643, EPI_ISL_741644, EPI_ISL_741645, EPI_ISL_741646, EPI_ISL_741647, EPI_ISL_741648, EPI_ISL_741649, EPI_ISL_741650, EPI_ISL_741651, EPI_ISL_741652, EPI_ISL_741655, EPI_ISL_741656			
see above	Queens Medical Centre, Clinical Microbiology Department / DeepSeq Nottingham	COVID-19 Genomics UK (COG-UK) Consortium	Gemma Clark, Wendy Smith, Manjinder Khakh, Vicki M Fleming, Michelle M Lister, Hannah Howson-Wells, Jonathan Ball, Patrick McClure, Joseph Chappell, Theocharis Tsoleridis, Nadine Holmes, Matthew Carlisle, Christopher Moore, Fei Sang, Johnny Debebe, Victoria Wright, Matthew Loose
EPI_ISL_741658, EPI_ISL_741675, EPI_ISL_741693, EPI_ISL_741697, EPI_ISL_741716, EPI_ISL_741717, EPI_ISL_741718, EPI_ISL_741719, EPI_ISL_741720, EPI_ISL_741721, EPI_ISL_741722, EPI_ISL_741723, EPI_ISL_741724, EPI_ISL_741725, EPI_ISL_741726, EPI_ISL_741728, EPI_ISL_741729, EPI_ISL_741730, EPI_ISL_741731, EPI_ISL_741732, EPI_ISL_741733, EPI_ISL_741734, EPI_ISL_741735, EPI_ISL_741736, EPI_ISL_741737, EPI_ISL_741738, EPI_ISL_741739, EPI_ISL_741740, EPI_ISL_741741, EPI_ISL_741742, EPI_ISL_741743, EPI_ISL_741744, EPI_ISL_741745, EPI_ISL_741746, EPI_ISL_741747, EPI_ISL_741748, EPI_ISL_741749, EPI_ISL_741750, EPI_ISL_741751, EPI_ISL_741752, EPI_ISL_741753, EPI_ISL_741754, EPI_ISL_741755, EPI_ISL_741756, EPI_ISL_741758, EPI_ISL_741759			

see above	Centre for Enzyme Innovation, University of Portsmouth / Translational Research Laboratory, Portsmouth Hospitals NHS Trust	COVID-19 Genomics UK (COG-UK) Consortium	Angela Beckett,Yann Bourgeois,Garry Scarlett,Sharon Glaysher,Scott Elliott,Kelly Bicknell,Robert Impey,Allyson Lloyd,Sarah Wyllie,Ethan Butcher,Anoop Chauhan,Samuel Robson
EPI_ISL_741760, EPI_ISL_741761, EPI_ISL_741762, EPI_ISL_741763, EPI_ISL_741764, EPI_ISL_741765, EPI_ISL_741766, EPI_ISL_741767, EPI_ISL_741768, EPI_ISL_741769, EPI_ISL_741770, EPI_ISL_741771			
see above	Oxford Viromics, NDM, University of Oxford; Oxford University Hospitals; Basingstoke and North Hampshire Hospital	COVID-19 Genomics UK (COG-UK) Consortium	Tanya Golubchik, David Bonsall, George Macintyre, Amy Trebes, Mariateresa de Cesare, Catrin Moore, Alex Mobbs, Anita Justice, Robert Shaw, Monique Andersson, Timothy Peto, Emma Wise, Nathan Moore, Jessica Lynch, Nick Cortes, Matilde Mori, Stephen Kidd, David Buck, John Todd, Christophe Fraser
EPI_ISL_741772	Centre for Enzyme Innovation, University of Portsmouth / Translational Research Laboratory, Portsmouth Hospitals NHS Trust	COVID-19 Genomics UK (COG-UK) Consortium	Angela Beckett,Yann Bourgeois,Garry Scarlett,Sharon Glaysher,Scott Elliott,Kelly Bicknell,Robert Impey,Allyson Lloyd,Sarah Wyllie,Ethan Butcher,Anoop Chauhan,Samuel Robson
EPI_ISL_741773, EPI_ISL_741774, EPI_ISL_741775, EPI_ISL_741776, EPI_ISL_741777, EPI_ISL_741778, EPI_ISL_741779, EPI_ISL_741780, EPI_ISL_741781, EPI_ISL_741782, EPI_ISL_741783, EPI_ISL_741784, EPI_ISL_741785, EPI_ISL_741786, EPI_ISL_741787, EPI_ISL_741788, EPI_ISL_741789, EPI_ISL_741790, EPI_ISL_741791, EPI_ISL_741792, EPI_ISL_741793, EPI_ISL_741794, EPI_ISL_741795, EPI_ISL_741796, EPI_ISL_741797, EPI_ISL_741798, EPI_ISL_741799, EPI_ISL_741800, EPI_ISL_741801, EPI_ISL_741802, EPI_ISL_741803, EPI_ISL_741804, EPI_ISL_741805, EPI_ISL_741806, EPI_ISL_741807, EPI_ISL_741808, EPI_ISL_741809, EPI_ISL_741810, EPI_ISL_741811, EPI_ISL_741812, EPI_ISL_741813, EPI_ISL_741814, EPI_ISL_741815, EPI_ISL_741816, EPI_ISL_741817, EPI_ISL_741818, EPI_ISL_741819, EPI_ISL_741820, EPI_ISL_741821, EPI_ISL_741822, EPI_ISL_741823, EPI_ISL_741824, EPI_ISL_741825, EPI_ISL_741826, EPI_ISL_741827, EPI_ISL_741828, EPI_ISL_741829, EPI_ISL_741830, EPI_ISL_741831, EPI_ISL_741832, EPI_ISL_741833, EPI_ISL_741834, EPI_ISL_741835, EPI_ISL_741836, EPI_ISL_741837, EPI_ISL_741838, EPI_ISL_741839, EPI_ISL_741840, EPI_ISL_741841, EPI_ISL_741842, EPI_ISL_741843, EPI_ISL_741844, EPI_ISL_741845, EPI_ISL_741846, EPI_ISL_741847, EPI_ISL_741848, EPI_ISL_741849, EPI_ISL_741850, EPI_ISL_741851, EPI_ISL_741852, EPI_ISL_741853, EPI_ISL_741854, EPI_ISL_741855, EPI_ISL_741856, EPI_ISL_741857, EPI_ISL_741858, EPI_ISL_741859, EPI_ISL_741860, EPI_ISL_741861, EPI_ISL_741862, EPI_ISL_741863, EPI_ISL_741864, EPI_ISL_741865, EPI_ISL_741866, EPI_ISL_741867, EPI_ISL_741868, EPI_ISL_741869, EPI_ISL_741870, EPI_ISL_741871, EPI_ISL_741872, EPI_ISL_741873, EPI_ISL_741874, EPI_ISL_741875, EPI_ISL_741876, EPI_ISL_741877, EPI_ISL_741878, EPI_ISL_741879, EPI_ISL_741880, EPI_ISL_741881, EPI_ISL_741882, EPI_ISL_741883, EPI_ISL_741884, EPI_ISL_741885, EPI_ISL_741886, EPI_ISL_741887, EPI_ISL_741888, EPI_ISL_741889, EPI_ISL_741890, EPI_ISL_741891, EPI_ISL_741892, EPI_ISL_741893, EPI_ISL_741894, EPI_ISL_741895, EPI_ISL_741896, EPI_ISL_741897, EPI_ISL_741898, EPI_ISL_741899, EPI_ISL_741900, EPI_ISL_741901, EPI_ISL_741902, EPI_ISL_741903, EPI_ISL_741904, EPI_ISL_741905, EPI_ISL_741906, EPI_ISL_741907, EPI_ISL_741908, EPI_ISL_741909, EPI_ISL_741910, EPI_ISL_741911, EPI_ISL_741912, EPI_ISL_741913, EPI_ISL_741914, EPI_ISL_741915, EPI_ISL_741916, EPI_ISL_741917, EPI_ISL_741918, EPI_ISL_741919, EPI_ISL_741920, EPI_ISL_741921, EPI_ISL_741922, EPI_ISL_741923, EPI_ISL_741924, EPI_ISL_741925, EPI_ISL_741926, EPI_ISL_741927, EPI_ISL_741928, EPI_ISL_741929, EPI_ISL_741930, EPI_ISL_741931, EPI_ISL_741932, EPI_ISL_741933, EPI_ISL_741934, EPI_ISL_741935			
see above	Oxford Viromics, NDM, University of Oxford; Oxford University Hospitals; Basingstoke and North Hampshire Hospital	COVID-19 Genomics UK (COG-UK) Consortium	Tanya Golubchik, David Bonsall, George Macintyre, Amy Trebes, Mariateresa de Cesare, Catrin Moore, Alex Mobbs, Anita Justice, Robert Shaw, Monique Andersson, Timothy Peto, Emma Wise, Nathan Moore, Jessica Lynch, Nick Cortes, Matilde Mori, Stephen Kidd, David Buck, John Todd, Christophe Fraser
EPI_ISL_741936, EPI_ISL_741937, EPI_ISL_741938, EPI_ISL_741939, EPI_ISL_741940, EPI_ISL_741942, EPI_ISL_741943, EPI_ISL_741944, EPI_ISL_741945, EPI_ISL_741946, EPI_ISL_741947, EPI_ISL_741948, EPI_ISL_741949, EPI_ISL_741950, EPI_ISL_741951, EPI_ISL_741952, EPI_ISL_741953, EPI_ISL_741954, EPI_ISL_741955, EPI_ISL_741956, EPI_ISL_741957, EPI_ISL_741960, EPI_ISL_741961, EPI_ISL_741963, EPI_ISL_741964, EPI_ISL_741965, EPI_ISL_741966, EPI_ISL_741967, EPI_ISL_741968, EPI_ISL_741969, EPI_ISL_741970, EPI_ISL_741971, EPI_ISL_741972, EPI_ISL_741973, EPI_ISL_741975, EPI_ISL_741976, EPI_ISL_741977, EPI_ISL_741978, EPI_ISL_741980, EPI_ISL_741981, EPI_ISL_741982, EPI_ISL_741983, EPI_ISL_741985, EPI_ISL_741986, EPI_ISL_741987, EPI_ISL_741988, EPI_ISL_741989, EPI_ISL_741990, EPI_ISL_741991, EPI_ISL_741992, EPI_ISL_741993, EPI_ISL_741994, EPI_ISL_741995, EPI_ISL_741996, EPI_ISL_741997, EPI_ISL_741998, EPI_ISL_741999, EPI_ISL_742000, EPI_ISL_742002, EPI_ISL_742003, EPI_ISL_742005, EPI_ISL_742006, EPI_ISL_742007, EPI_ISL_742008, EPI_ISL_742009, EPI_ISL_742010, EPI_ISL_742011, EPI_ISL_742012, EPI_ISL_742014, EPI_ISL_742015, EPI_ISL_742016, EPI_ISL_742017, EPI_ISL_742018, EPI_ISL_742019, EPI_ISL_742021, EPI_ISL_742022, EPI_ISL_742023, EPI_ISL_742024, EPI_ISL_742025, EPI_ISL_742026, EPI_ISL_742027, EPI_ISL_742028, EPI_ISL_742029, EPI_ISL_742030, EPI_ISL_742031, EPI_ISL_742032, EPI_ISL_742033, EPI_ISL_742034, EPI_ISL_742036, EPI_ISL_742037, EPI_ISL_742038, EPI_ISL_742039, EPI_ISL_742040, EPI_ISL_742041, EPI_ISL_742042, EPI_ISL_742043, EPI_ISL_742044, EPI_ISL_742046, EPI_ISL_742047, EPI_ISL_742048, EPI_ISL_742049, EPI_ISL_742050, EPI_ISL_742051, EPI_ISL_742052, EPI_ISL_742053, EPI_ISL_742054, EPI_ISL_742056, EPI_ISL_742058, EPI_ISL_742059, EPI_ISL_742062, EPI_ISL_742063, EPI_ISL_742064, EPI_ISL_742065, EPI_ISL_742066, EPI_ISL_742067, EPI_ISL_742068, EPI_ISL_742069, EPI_ISL_742070, EPI_ISL_742071, EPI_ISL_742072, EPI_ISL_742073, EPI_ISL_742074, EPI_ISL_742075, EPI_ISL_742076, EPI_ISL_742077, EPI_ISL_742078, EPI_ISL_742080, EPI_ISL_742081, EPI_ISL_742082, EPI_ISL_742083, EPI_ISL_742084, EPI_ISL_742085, EPI_ISL_742086, EPI_ISL_742087, EPI_ISL_742088, EPI_ISL_742089, EPI_ISL_742090, EPI_ISL_742091, EPI_ISL_742092, EPI_ISL_742093, EPI_ISL_742094, EPI_ISL_742095, EPI_ISL_742096, EPI_ISL_742097, EPI_ISL_742098, EPI_ISL_742099, EPI_ISL_742100, EPI_ISL_742101, EPI_ISL_742102, EPI_ISL_742103, EPI_ISL_742104, EPI_ISL_742105, EPI_ISL_742106, EPI_ISL_742107, EPI_ISL_742109, EPI_ISL_742110, EPI_ISL_742111, EPI_ISL_742112, EPI_ISL_742113, EPI_ISL_742115, EPI_ISL_742116			
see above	Virology Department, Sheffield Teaching Hospitals NHS Foundation Trust/Department of Infection, Immunity and Cardiovascular Disease, The Medical School, University of Sheffield	COVID-19 Genomics UK (COG-UK) Consortium	Thushan de Silva, Matthew Parker, Nikki Smith, Adri Angyal, Rebecca Brown, Luke Green, Rachel Tucker, Paul Parsons, Danielle Groves, Katie Johnson, Laura Carrilero, Alex Keeley, Dave Partridge, Matthew Wyles, Benjamin Lindsey, Mehmet Yavuz, Mohammad Raza, Cariad Evans
EPI_ISL_742117	University of Exeter	COVID-19 Genomics UK (COG-UK) Consortium	Ben Temperton,Aaron Jeffries,Michelle Michelsen,Joanna Warwick-Dugdale,Audrey Farbos,Robyn Manley,Stephen Michell,Jane Masoli
EPI_ISL_742118, EPI_ISL_742119, EPI_ISL_742120, EPI_ISL_742121, EPI_ISL_742122, EPI_ISL_742123, EPI_ISL_742124, EPI_ISL_742125, EPI_ISL_742126, EPI_ISL_742127, EPI_ISL_742129, EPI_ISL_742130, EPI_ISL_742131, EPI_ISL_742132, EPI_ISL_742133, EPI_ISL_742134, EPI_ISL_742135, EPI_ISL_742137, EPI_ISL_742138, EPI_ISL_742139, EPI_ISL_742140, EPI_ISL_742141, EPI_ISL_742142, EPI_ISL_742143, EPI_ISL_742144, EPI_ISL_742145, EPI_ISL_742146, EPI_ISL_742147, EPI_ISL_742148, EPI_ISL_742149, EPI_ISL_742150, EPI_ISL_742151, EPI_ISL_742152, EPI_ISL_742153, EPI_ISL_742154, EPI_ISL_742155, EPI_ISL_742156, EPI_ISL_742157, EPI_ISL_742158, EPI_ISL_742159, EPI_ISL_742161, EPI_ISL_742162, EPI_ISL_742163, EPI_ISL_742164, EPI_ISL_742165, EPI_ISL_742167, EPI_ISL_742168, EPI_ISL_742169, EPI_ISL_742171, EPI_ISL_742172, EPI_ISL_742173			
see above	Regional Virus Laboratory, Belfast Health and Social Care Trust	COVID-19 Genomics UK (COG-UK) Consortium	Conall McCaughey, James McKenna, Tanya Curran, Susan Feeney, Alison Watt, Ciara Cox, Mairead Connor, Zoltan Molnar, David Simpson, Derek Fairley
EPI_ISL_742175, EPI_ISL_742176, EPI_ISL_742177, EPI_ISL_742178, EPI_ISL_742179, EPI_ISL_742180, EPI_ISL_742181, EPI_ISL_742182, EPI_ISL_742183, EPI_ISL_742184, EPI_ISL_742185, EPI_ISL_742186, EPI_ISL_742187, EPI_ISL_742188, EPI_ISL_742189, EPI_ISL_742190, EPI_ISL_742191, EPI_ISL_742192, EPI_ISL_742193, EPI_ISL_742194, EPI_ISL_742195, EPI_ISL_742196, EPI_ISL_742197, EPI_ISL_742198, EPI_ISL_742199, EPI_ISL_742200, EPI_ISL_742201, EPI_ISL_742202, EPI_ISL_742203, EPI_ISL_742204, EPI_ISL_742205, EPI_ISL_742206, EPI_ISL_742207, EPI_ISL_742208, EPI_ISL_742209, EPI_ISL_742210, EPI_ISL_742211, EPI_ISL_742212, EPI_ISL_742213, EPI_ISL_742214, EPI_ISL_742215, EPI_ISL_742216, EPI_ISL_742217, EPI_ISL_742218, EPI_ISL_742219, EPI_ISL_742220, EPI_ISL_742221, EPI_ISL_742222, EPI_ISL_742223, EPI_ISL_742224, EPI_ISL_742225, EPI_ISL_742226, EPI_ISL_742227, EPI_ISL_742228, EPI_ISL_742229, EPI_ISL_742230, EPI_ISL_742231, EPI_ISL_742232, EPI_ISL_742233, EPI_ISL_742234, EPI_ISL_742235, EPI_ISL_742236, EPI_ISL_742237, EPI_ISL_742238, EPI_ISL_742239, EPI_ISL_742240, EPI_ISL_742241, EPI_ISL_742242, EPI_ISL_742243, EPI_ISL_742244, EPI_ISL_742245, EPI_ISL_742246			
see above	Virology Department, Royal Infirmary of Edinburgh, NHS Lothian / School of Biological Sciences, University of Edinburgh / Institute of Genetics and Molecular Medicine, University of Edinburgh	COVID-19 Genomics UK (COG-UK) Consortium	McHugh M, Dewar R, Rooke S, Gallagher M, Balcaza C, O'Toole Á, Scher E, Hill V, McCrone JT, Colquhoun R, Yu X, Jackson B, Rambaut A, Williams TC, Templeton K
EPI_ISL_742247, EPI_ISL_742248, EPI_ISL_742249, EPI_ISL_742250, EPI_ISL_742251, EPI_ISL_742252, EPI_ISL_742253, EPI_ISL_742254, EPI_ISL_742255, EPI_ISL_742256, EPI_ISL_742257, EPI_ISL_742258, EPI_ISL_742259			
see above	Oxford Viromics, NDM, University of Oxford; Oxford University Hospitals; Basingstoke and North Hampshire Hospital	COVID-19 Genomics UK (COG-UK) Consortium	Tanya Golubchik, David Bonsall, George Macintyre, Amy Trebes, Mariateresa de Cesare, Catrin Moore, Alex Mobbs, Anita Justice, Robert Shaw, Monique Andersson, Timothy Peto, Emma Wise, Nathan Moore, Jessica Lynch, Nick Cortes, Matilde Mori, Stephen Kidd, David Buck, John Todd, Christophe Fraser
EPI_ISL_742260	Centre for Enzyme Innovation, University of Portsmouth / Translational Research Laboratory, Portsmouth Hospitals NHS Trust	COVID-19 Genomics UK (COG-UK) Consortium	Angela Beckett,Yann Bourgeois,Garry Scarlett,Sharon Glaysher,Scott Elliott,Kelly Bicknell,Robert Impey,Allyson Lloyd,Sarah Wyllie,Ethan Butcher,Anoop Chauhan,Samuel Robson
EPI_ISL_742261	Oxford Viromics, NDM, University of Oxford; Oxford University Hospitals; Basingstoke and North Hampshire Hospital	COVID-19 Genomics UK (COG-UK) Consortium	Tanya Golubchik, David Bonsall, George Macintyre, Amy Trebes, Mariateresa de Cesare, Catrin Moore, Alex Mobbs, Anita Justice, Robert Shaw, Monique Andersson, Timothy Peto, Emma Wise, Nathan Moore, Jessica Lynch, Nick Cortes, Matilde Mori, Stephen Kidd, David Buck, John Todd, Christophe Fraser
EPI_ISL_742262, EPI_ISL_742263, EPI_ISL_742264, EPI_ISL_742265, EPI_ISL_742266, EPI_ISL_742267, EPI_ISL_742268, EPI_ISL_742269, EPI_ISL_742270, EPI_ISL_742271, EPI_ISL_742273, EPI_ISL_742274, EPI_ISL_742276, EPI_ISL_742277, EPI_ISL_742278, EPI_ISL_742279, EPI_ISL_742280, EPI_ISL_742281, EPI_ISL_742282, EPI_ISL_742283, EPI_ISL_742284, EPI_ISL_742285, EPI_ISL_742286, EPI_ISL_742287, EPI_ISL_742289, EPI_ISL_742290, EPI_ISL_742291, EPI_ISL_742292, EPI_ISL_742293, EPI_ISL_742294, EPI_ISL_742295, EPI_ISL_742296, EPI_ISL_742297, EPI_ISL_742298, EPI_ISL_742299, EPI_ISL_742300, EPI_ISL_742301, EPI_ISL_742303, EPI_ISL_742304, EPI_ISL_742305, EPI_ISL_742306, EPI_ISL_742307, EPI_ISL_742308, EPI_ISL_742310, EPI_ISL_742311, EPI_ISL_742312, EPI_ISL_742313, EPI_ISL_742314, EPI_ISL_742315, EPI_ISL_742316, EPI_ISL_742317, EPI_ISL_742318, EPI_ISL_742319, EPI_ISL_742320, EPI_ISL_742321, EPI_ISL_742322, EPI_ISL_742323, EPI_ISL_742324, EPI_ISL_742325, EPI_ISL_742326, EPI_ISL_742327, EPI_ISL_742328, EPI_ISL_742329, EPI_ISL_742330, EPI_ISL_742331, EPI_ISL_742332, EPI_ISL_742333, EPI_ISL_742334, EPI_ISL_742335, EPI_ISL_742336, EPI_ISL_742337, EPI_ISL_742338, EPI_ISL_742339, EPI_ISL_742340, EPI_ISL_742341, EPI_ISL_742342, EPI_ISL_742343, EPI_ISL_742344, EPI_ISL_742345, EPI_ISL_742346, EPI_ISL_742347, EPI_ISL_742348, EPI_ISL_742349, EPI_ISL_742350, EPI_ISL_742351, EPI_ISL_742352, EPI_ISL_742354, EPI_ISL_742355, EPI_ISL_742356, EPI_ISL_742357, EPI_ISL_742358, EPI_ISL_742359, EPI_ISL_742360, EPI_ISL_742361, EPI_ISL_742362, EPI_ISL_742363, EPI_ISL_742364, EPI_ISL_742365, EPI_ISL_742367, EPI_ISL_742368, EPI_ISL_742369, EPI_ISL_742370, EPI_ISL_742371, EPI_ISL_742372, EPI_ISL_742373, EPI_ISL_742374, EPI_ISL_742375, EPI_ISL_742376, EPI_ISL_742377, EPI_ISL_742378, EPI_ISL_742379, EPI_ISL_742380, EPI_ISL_742381, EPI_ISL_742382, EPI_ISL_742383, EPI_ISL_742386, EPI_ISL_742387, EPI_ISL_742388, EPI_ISL_742389, EPI_ISL_742390, EPI_ISL_742391, EPI_ISL_742392, EPI_ISL_742393, EPI_ISL_742394, EPI_ISL_742395, EPI_ISL_742396, EPI_ISL_742397, EPI_ISL_742398, EPI_ISL_742399, EPI_ISL_742400, EPI_ISL_742401, EPI_ISL_742402, EPI_ISL_742403, EPI_ISL_742404, EPI_ISL_742405, EPI_ISL_742406, EPI_ISL_742407, EPI_ISL_742408, EPI_ISL_742409, EPI_ISL_742410, EPI_ISL_742411, EPI_ISL_742412, EPI_ISL_742413, EPI_ISL_742414, EPI_ISL_742415, EPI_ISL_742416, EPI_ISL_742417, EPI_ISL_742418, EPI_ISL_742419, EPI_ISL_742420, EPI_ISL_742421, EPI_ISL_742422, EPI_ISL_742423, EPI_ISL_742424, EPI_ISL_742425, EPI_ISL_742426, EPI_ISL_742427, EPI_ISL_742428, EPI_ISL_742429, EPI_ISL_742430, EPI_ISL_742431, EPI_ISL_742432, EPI_ISL_742433, EPI_ISL_742434, EPI_ISL_742435, EPI_ISL_742436, EPI_ISL_742437, EPI_ISL_742438, EPI_ISL_742439, EPI_ISL_742440, EPI_ISL_742441, EPI_ISL_742442, EPI_ISL_742443, EPI_ISL_742444, EPI_ISL_742445, EPI_ISL_742446, EPI_ISL_742447, EPI_ISL_742448, EPI_ISL_742449, EPI_ISL_742450, EPI_ISL_742451, EPI_ISL_742452, EPI_ISL_742453, EPI_ISL_742454, EPI_ISL_742455, EPI_ISL_742456, EPI_ISL_742457, EPI_ISL_742458, EPI_ISL_742459, EPI_ISL_742460, EPI_ISL_742461, EPI_ISL_742462, EPI_ISL_742463, EPI_ISL_742464, EPI_ISL_742465, EPI_ISL_742466, EPI_ISL_742467, EPI_ISL_742468, EPI_ISL_742469, EPI_ISL_742470, EPI_ISL_742471, EPI_ISL_742472, EPI_ISL_742473, EPI_ISL_742474, EPI_ISL_742475, EPI_ISL_742476, EPI_ISL_742477, EPI_ISL_742478, EPI_ISL_742479, EPI_ISL_742480, EPI_ISL_742481, EPI_ISL_742482, EPI_ISL_742483, EPI_ISL_742484, EPI_ISL_742485, EPI_ISL_742486, EPI_ISL_742487, EPI_ISL_742488, EPI_ISL_742489, EPI_ISL_742490, EPI_ISL_742491, EPI_ISL_742492, EPI_ISL_742493, EPI_ISL_742494, EPI_ISL_742495, EPI_ISL_742496, EPI_ISL_742497, EPI_ISL_742498, EPI_ISL_742499, EPI_ISL_742500, EPI_ISL_742501, EPI_ISL_742502, EPI_ISL_742503, EPI_ISL_742504, EPI_ISL_742505, EPI_ISL_742506, EPI_ISL_742507, EPI_ISL_742508, EPI_ISL_742509, EPI_ISL_742510, EPI_ISL_742511, EPI_ISL_742512, EPI_ISL_742513, EPI_ISL_742514, EPI_ISL_742515, EPI_ISL_742516, EPI_ISL_742517, EPI_ISL_742518, EPI_ISL_742519, EPI_ISL_742520, EPI_ISL_742521, EPI_ISL_742522, EPI_ISL_742523, EPI_ISL_742524, EPI_ISL_742525, EPI_ISL_742526, EPI_ISL_742527, EPI_ISL_742528, EPI_ISL_742530, EPI_ISL_742531, EPI_ISL_742532, EPI_ISL_742533, EPI_ISL_742534, EPI_ISL_742535, EPI_ISL_742536, EPI_ISL_742537, EPI_ISL_742538, EPI_ISL_742539, EPI_ISL_742540, EPI_ISL_742541, EPI_ISL_742542, EPI_ISL_742543, EPI_ISL_742544, EPI_ISL_742545, EPI_ISL_742546, EPI_ISL_742547, EPI_ISL_742548, EPI_ISL_742549, EPI_ISL_742550, EPI_ISL_742551, EPI_ISL_742552, EPI_ISL_742554, EPI_ISL_742555, EPI_ISL_742556, EPI_ISL_742557, EPI_ISL_742558, EPI_ISL_742559, EPI_ISL_742560, EPI_ISL_742561, EPI_ISL_742562, EPI_ISL_742563, EPI_ISL_742564, EPI_ISL_742565, EPI_ISL_742566, EPI_ISL_742567, EPI_ISL_742568, EPI_ISL_742569, EPI_ISL_742570, EPI_ISL_742571, EPI_ISL_742572, EPI_ISL_742573, EPI_ISL_742574, EPI_ISL_742575, EPI_ISL_742576, EPI_ISL_742577, EPI_ISL_742578, EPI_ISL_742579, EPI_ISL_742580, EPI_ISL_742581, EPI_ISL_742582, EPI_ISL_742583, EPI_ISL_742584, EPI_ISL_742585, EPI_ISL_742586, EPI_ISL_742587, EPI_ISL_742588, EPI_ISL_742589, EPI_ISL_742590, EPI_ISL_742591, EPI_ISL_742592, EPI_ISL_742594, EPI_ISL_742595, EPI_ISL_742596, EPI_ISL_742597, EPI_ISL_742598, EPI_ISL_742599, EPI_ISL_742601, EPI_ISL_742602, EPI_ISL_742603, EPI_ISL_742604, EPI_ISL_742605, EPI_ISL_742606, EPI_ISL_742607, EPI_ISL_742608, EPI_ISL_742609, EPI_ISL_742610, EPI_ISL_742611, EPI_ISL_742612, EPI_ISL_742613, EPI_ISL_742614, EPI_ISL_742615, EPI_ISL_742616,			

EPI_ISL_742617, EPI_ISL_742619, EPI_ISL_742621, EPI_ISL_742622, EPI_ISL_742623, EPI_ISL_742624, EPI_ISL_742625, EPI_ISL_742626, EPI_ISL_742627, EPI_ISL_742628, EPI_ISL_742629, EPI_ISL_742630, EPI_ISL_742631, EPI_ISL_742632, EPI_ISL_742634, EPI_ISL_742635, EPI_ISL_742636, EPI_ISL_742637, EPI_ISL_742638, EPI_ISL_742639, EPI_ISL_742640, EPI_ISL_742641, EPI_ISL_742642, EPI_ISL_742643, EPI_ISL_742644, EPI_ISL_742645, EPI_ISL_742646, EPI_ISL_742647, EPI_ISL_742648, EPI_ISL_742649, EPI_ISL_742650, EPI_ISL_742651, EPI_ISL_742652, EPI_ISL_742653, EPI_ISL_742654, EPI_ISL_742655, EPI_ISL_742656, EPI_ISL_742657, EPI_ISL_742658, EPI_ISL_742659, EPI_ISL_742660, EPI_ISL_742661, EPI_ISL_742662, EPI_ISL_742663, EPI_ISL_742664, EPI_ISL_742665, EPI_ISL_742666, EPI_ISL_742667, EPI_ISL_742668, EPI_ISL_742669, EPI_ISL_742670, EPI_ISL_742671, EPI_ISL_742672, EPI_ISL_742673, EPI_ISL_742675, EPI_ISL_742676, EPI_ISL_742677, EPI_ISL_742678, EPI_ISL_742679, EPI_ISL_742680, EPI_ISL_742681, EPI_ISL_742682, EPI_ISL_742683, EPI_ISL_742684, EPI_ISL_742685, EPI_ISL_742686, EPI_ISL_742687, EPI_ISL_742688, EPI_ISL_742689, EPI_ISL_742690, EPI_ISL_742691, EPI_ISL_742692, EPI_ISL_742693, EPI_ISL_742694, EPI_ISL_742695, EPI_ISL_742696, EPI_ISL_742697, EPI_ISL_742698, EPI_ISL_742699, EPI_ISL_742700, EPI_ISL_742701, EPI_ISL_742702, EPI_ISL_742703, EPI_ISL_742704, EPI_ISL_742705, EPI_ISL_742706, EPI_ISL_742707, EPI_ISL_742708, EPI_ISL_742709, EPI_ISL_742710, EPI_ISL_742712, EPI_ISL_742713, EPI_ISL_742714, EPI_ISL_742715, EPI_ISL_742716, EPI_ISL_742717, EPI_ISL_742718, EPI_ISL_742719, EPI_ISL_742720, EPI_ISL_742721, EPI_ISL_742722, EPI_ISL_742723, EPI_ISL_742724, EPI_ISL_742725, EPI_ISL_742726, EPI_ISL_742727, EPI_ISL_742728, EPI_ISL_742729, EPI_ISL_742730, EPI_ISL_742731, EPI_ISL_742732, EPI_ISL_742733, EPI_ISL_742734, EPI_ISL_742735, EPI_ISL_742736, EPI_ISL_742737, EPI_ISL_742738, EPI_ISL_742739, EPI_ISL_742740, EPI_ISL_742741, EPI_ISL_742742, EPI_ISL_742743, EPI_ISL_742744, EPI_ISL_742745, EPI_ISL_742746, EPI_ISL_742747, EPI_ISL_742748, EPI_ISL_742749, EPI_ISL_742750, EPI_ISL_742751, EPI_ISL_742752, EPI_ISL_742753, EPI_ISL_742754, EPI_ISL_742755, EPI_ISL_742757, EPI_ISL_742758, EPI_ISL_742759, EPI_ISL_742760, EPI_ISL_742761, EPI_ISL_742762, EPI_ISL_742763, EPI_ISL_742764, EPI_ISL_742765, EPI_ISL_742766, EPI_ISL_742767, EPI_ISL_742768, EPI_ISL_742769, EPI_ISL_742770, EPI_ISL_742771, EPI_ISL_742772, EPI_ISL_742773, EPI_ISL_742774, EPI_ISL_742775, EPI_ISL_742776, EPI_ISL_742777, EPI_ISL_742778, EPI_ISL_742779, EPI_ISL_742780, EPI_ISL_742781, EPI_ISL_742782, EPI_ISL_742783, EPI_ISL_742784, EPI_ISL_742785, EPI_ISL_742786, EPI_ISL_742787, EPI_ISL_742788, EPI_ISL_742789, EPI_ISL_742790, EPI_ISL_742791, EPI_ISL_742792, EPI_ISL_742793, EPI_ISL_742794, EPI_ISL_742795, EPI_ISL_742796, EPI_ISL_742797, EPI_ISL_742798, EPI_ISL_742799, EPI_ISL_742800, EPI_ISL_742801, EPI_ISL_742802, EPI_ISL_742803, EPI_ISL_742804, EPI_ISL_742805, EPI_ISL_742806, EPI_ISL_742807, EPI_ISL_742808, EPI_ISL_742809, EPI_ISL_742810, EPI_ISL_742811, EPI_ISL_742812, EPI_ISL_742813, EPI_ISL_742814, EPI_ISL_742815, EPI_ISL_742816, EPI_ISL_742817, EPI_ISL_742818, EPI_ISL_742819, EPI_ISL_742820, EPI_ISL_742821, EPI_ISL_742822, EPI_ISL_742824, EPI_ISL_742825, EPI_ISL_742826, EPI_ISL_742827, EPI_ISL_742828, EPI_ISL_742829, EPI_ISL_742830, EPI_ISL_742832, EPI_ISL_742833, EPI_ISL_742834, EPI_ISL_742835, EPI_ISL_742836, EPI_ISL_742837, EPI_ISL_742838, EPI_ISL_742839, EPI_ISL_742840, EPI_ISL_742841, EPI_ISL_742842, EPI_ISL_742843, EPI_ISL_742844, EPI_ISL_742845, EPI_ISL_742846, EPI_ISL_742848, EPI_ISL_742849, EPI_ISL_742850, EPI_ISL_742851, EPI_ISL_742852, EPI_ISL_742856, EPI_ISL_742854, EPI_ISL_742855, EPI_ISL_742856, EPI_ISL_742857, EPI_ISL_742858, EPI_ISL_742859, EPI_ISL_742860, EPI_ISL_742861, EPI_ISL_742862, EPI_ISL_742863, EPI_ISL_742864, EPI_ISL_742865, EPI_ISL_742866, EPI_ISL_742867, EPI_ISL_742868, EPI_ISL_742869, EPI_ISL_742870, EPI_ISL_742871, EPI_ISL_742872, EPI_ISL_742873, EPI_ISL_742874, EPI_ISL_742875, EPI_ISL_742876, EPI_ISL_742877, EPI_ISL_742878, EPI_ISL_742879, EPI_ISL_742880, EPI_ISL_742881, EPI_ISL_742882, EPI_ISL_742883, EPI_ISL_742884, EPI_ISL_742885, EPI_ISL_742886, EPI_ISL_742887, EPI_ISL_742888, EPI_ISL_742889, EPI_ISL_742890, EPI_ISL_742891, EPI_ISL_742892, EPI_ISL_742893, EPI_ISL_742894, EPI_ISL_742895, EPI_ISL_742896, EPI_ISL_742898, EPI_ISL_742899, EPI_ISL_742900, EPI_ISL_742901, EPI_ISL_742902, EPI_ISL_742903, EPI_ISL_742904, EPI_ISL_742905, EPI_ISL_742907, EPI_ISL_742908, EPI_ISL_742909, EPI_ISL_742910, EPI_ISL_742911, EPI_ISL_742912, EPI_ISL_742913, EPI_ISL_742914, EPI_ISL_742915, EPI_ISL_742916, EPI_ISL_742917, EPI_ISL_742918, EPI_ISL_742919, EPI_ISL_742920, EPI_ISL_742921, EPI_ISL_742922, EPI_ISL_742923, EPI_ISL_742924, EPI_ISL_742925, EPI_ISL_742926, EPI_ISL_742927, EPI_ISL_742928, EPI_ISL_742929, EPI_ISL_742930, EPI_ISL_742931, EPI_ISL_742932, EPI_ISL_742933, EPI_ISL_742934, EPI_ISL_742935, EPI_ISL_742936, EPI_ISL_742937, EPI_ISL_742938, EPI_ISL_742939, EPI_ISL_742940, EPI_ISL_742941, EPI_ISL_742942, EPI_ISL_742943, EPI_ISL_742944, EPI_ISL_742945, EPI_ISL_742946, EPI_ISL_742947, EPI_ISL_742948, EPI_ISL_742949, EPI_ISL_742950, EPI_ISL_742951, EPI_ISL_742952, EPI_ISL_742953, EPI_ISL_742954, EPI_ISL_742955, EPI_ISL_742956, EPI_ISL_742957, EPI_ISL_742958, EPI_ISL_742959, EPI_ISL_742960, EPI_ISL_742961, EPI_ISL_742962, EPI_ISL_742963, EPI_ISL_742964, EPI_ISL_742965, EPI_ISL_742966, EPI_ISL_742967, EPI_ISL_742968, EPI_ISL_742969, EPI_ISL_742970, EPI_ISL_742971, EPI_ISL_742972, EPI_ISL_742973, EPI_ISL_742974, EPI_ISL_742975, EPI_ISL_742976, EPI_ISL_742977, EPI_ISL_742978, EPI_ISL_742979, EPI_ISL_742980, EPI_ISL_742981, EPI_ISL_742982, EPI_ISL_742983, EPI_ISL_742984, EPI_ISL_742985, EPI_ISL_742986, EPI_ISL_742987, EPI_ISL_742988, EPI_ISL_742989, EPI_ISL_742990, EPI_ISL_742991, EPI_ISL_742992, EPI_ISL_742993, EPI_ISL_742994, EPI_ISL_742995, EPI_ISL_742996, EPI_ISL_742997, EPI_ISL_742998, EPI_ISL_742999, EPI_ISL_743000, EPI_ISL_743001, EPI_ISL_743002, EPI_ISL_743003, EPI_ISL_743004, EPI_ISL_743005, EPI_ISL_743006, EPI_ISL_743007, EPI_ISL_743008, EPI_ISL_743009, EPI_ISL_743010, EPI_ISL_743011, EPI_ISL_743012, EPI_ISL_743013, EPI_ISL_743014, EPI_ISL_743015, EPI_ISL_743016, EPI_ISL_743017, EPI_ISL_743018, EPI_ISL_743019, EPI_ISL_743020, EPI_ISL_743021, EPI_ISL_743022, EPI_ISL_743023, EPI_ISL_743024, EPI_ISL_743025, EPI_ISL_743027, EPI_ISL_743028, EPI_ISL_743029, EPI_ISL_743030, EPI_ISL_743031, EPI_ISL_743032, EPI_ISL_743033, EPI_ISL_743035, EPI_ISL_743036, EPI_ISL_743037, EPI_ISL_743039, EPI_ISL_743040, EPI_ISL_743041, EPI_ISL_743042, EPI_ISL_743043, EPI_ISL_743044, EPI_ISL_743045, EPI_ISL_743046, EPI_ISL_743047, EPI_ISL_743048, EPI_ISL_743049, EPI_ISL_743051, EPI_ISL_743052, EPI_ISL_743053, EPI_ISL_743054, EPI_ISL_743055, EPI_ISL_743056, EPI_ISL_743057, EPI_ISL_743058, EPI_ISL_743060, EPI_ISL_743061, EPI_ISL_743062, EPI_ISL_743063, EPI_ISL_743064, EPI_ISL_743065, EPI_ISL_743066, EPI_ISL_743067, EPI_ISL_743068, EPI_ISL_743069, EPI_ISL_743070, EPI_ISL_743071, EPI_ISL_743072, EPI_ISL_743073, EPI_ISL_743074, EPI_ISL_743076, EPI_ISL_743077, EPI_ISL_743078, EPI_ISL_743079, EPI_ISL_743080, EPI_ISL_743081, EPI_ISL_743082, EPI_ISL_743083, EPI_ISL_743084, EPI_ISL_743085, EPI_ISL_743086, EPI_ISL_743087, EPI_ISL_743088, EPI_ISL_743089, EPI_ISL_743091, EPI_ISL_743092, EPI_ISL_743093, EPI_ISL_743094, EPI_ISL_743095, EPI_ISL_743096, EPI_ISL_743098, EPI_ISL_743099, EPI_ISL_743101, EPI_ISL_743102, EPI_ISL_743103, EPI_ISL_743104, EPI_ISL_743105, EPI_ISL_743107, EPI_ISL_743108, EPI_ISL_743109, EPI_ISL_743110, EPI_ISL_743111, EPI_ISL_743112, EPI_ISL_743113, EPI_ISL_743114, EPI_ISL_743115, EPI_ISL_743116, EPI_ISL_743117, EPI_ISL_743118, EPI_ISL_743119, EPI_ISL_743120, EPI_ISL_743121, EPI_ISL_743122, EPI_ISL_743123, EPI_ISL_743124, EPI_ISL_743125, EPI_ISL_743126, EPI_ISL_743127, EPI_ISL_743128, EPI_ISL_743129, EPI_ISL_743130, EPI_ISL_743131, EPI_ISL_743132, EPI_ISL_743133, EPI_ISL_743134, EPI_ISL_743135, EPI_ISL_743136, EPI_ISL_743137, EPI_ISL_743138, EPI_ISL_743139, EPI_ISL_743140, EPI_ISL_743141, EPI_ISL_743142, EPI_ISL_743143, EPI_ISL_743144, EPI_ISL_743145, EPI_ISL_743146, EPI_ISL_743147, EPI_ISL_743148, EPI_ISL_743149, EPI_ISL_743150, EPI_ISL_743151, EPI_ISL_743152, EPI_ISL_743153, EPI_ISL_743154, EPI_ISL_743155, EPI_ISL_743156, EPI_ISL_743157, EPI_ISL_743158, EPI_ISL_743159, EPI_ISL_743160, EPI_ISL_743161, EPI_ISL_743162, EPI_ISL_743163, EPI_ISL_743164, EPI_ISL_743165, EPI_ISL_743166, EPI_ISL_743167, EPI_ISL_743168, EPI_ISL_743169, EPI_ISL_743170, EPI_ISL_743171, EPI_ISL_743172, EPI_ISL_743173, EPI_ISL_743174, EPI_ISL_743175, EPI_ISL_743176, EPI_ISL_743177, EPI_ISL_743178, EPI_ISL_743179, EPI_ISL_743180, EPI_ISL_743181, EPI_ISL_743182, EPI_ISL_743183, EPI_ISL_743184, EPI_ISL_743185, EPI_ISL_743186, EPI_ISL_743187, EPI_ISL_743188, EPI_ISL_743189, EPI_ISL_743190, EPI_ISL_743191, EPI_ISL_743192, EPI_ISL_743193, EPI_ISL_743195, EPI_ISL_743196, EPI_ISL_743197, EPI_ISL_743198, EPI_ISL_743199, EPI_ISL_743200, EPI_ISL_743201, EPI_ISL_743202, EPI_ISL_743203, EPI_ISL_743204, EPI_ISL_743205, EPI_ISL_743206, EPI_ISL_743207, EPI_ISL_743208, EPI_ISL_743209, EPI_ISL_743210, EPI_ISL_743212, EPI_ISL_743213, EPI_ISL_743214, EPI_ISL_743215, EPI_ISL_743216, EPI_ISL_743217, EPI_ISL_743218, EPI_ISL_743219, EPI_ISL_743220, EPI_ISL_743221, EPI_ISL_743222, EPI_ISL_743223, EPI_ISL_743224, EPI_ISL_743225, EPI_ISL_743226, EPI_ISL_743227, EPI_ISL_743228, EPI_ISL_743229, EPI_ISL_743230, EPI_ISL_743231, EPI_ISL_743232, EPI_ISL_743233, EPI_ISL_743234, EPI_ISL_743235, EPI_ISL_743236, EPI_ISL_743237, EPI_ISL_743238, EPI_ISL_743239, EPI_ISL_743240, EPI_ISL_743241, EPI_ISL_743242, EPI_ISL_743243, EPI_ISL_743245, EPI_ISL_743246, EPI_ISL_743247, EPI_ISL_743248, EPI_ISL_743249, EPI_ISL_743250, EPI_ISL_743251, EPI_ISL_743252, EPI_ISL_743253, EPI_ISL_743254, EPI_ISL_743255, EPI_ISL_743256, EPI_ISL_743257, EPI_ISL_743258, EPI_ISL_743259, EPI_ISL_743260, EPI_ISL_743261, EPI_ISL_743262, EPI_ISL_743263, EPI_ISL_743264, EPI_ISL_743265, EPI_ISL_743266, EPI_ISL_743267, EPI_ISL_743268, EPI_ISL_743269, EPI_ISL_743270, EPI_ISL_743271, EPI_ISL_743272, EPI_ISL_743273, EPI_ISL_743274, EPI_ISL_743275, EPI_ISL_743276, EPI_ISL_743277, EPI_ISL_743278, EPI_ISL_743279, EPI_ISL_743280, EPI_ISL_743281, EPI_ISL_743282, EPI_ISL_743283, EPI_ISL_743284, EPI_ISL_743285, EPI_ISL_743286, EPI_ISL_743287, EPI_ISL_743288, EPI_ISL_743289, EPI_ISL_743290, EPI_ISL_743291, EPI_ISL_743292, EPI_ISL_743293, EPI_ISL_743294, EPI_ISL_743295, EPI_ISL_743296, EPI_ISL_743297, EPI_ISL_743298, EPI_ISL_743299, EPI_ISL_743300, EPI_ISL_743301, EPI_ISL_743302, EPI_ISL_743303, EPI_ISL_743304, EPI_ISL_743305, EPI_ISL_743306, EPI_ISL_743307, EPI_ISL_743308, EPI_ISL_743309, EPI_ISL_743310, EPI_ISL_743311, EPI_ISL_743312, EPI_ISL_743313, EPI_ISL_743314, EPI_ISL_743315, EPI_ISL_743316, EPI_ISL_743317, EPI_ISL_743318, EPI_ISL_743319, EPI_ISL_743320, EPI_ISL_743321, EPI_ISL_743322, EPI_ISL_743323, EPI_ISL_743324, EPI_ISL_743325, EPI_ISL_743326, EPI_ISL_743327, EPI_ISL_743328, EPI_ISL_743329, EPI_ISL_743330, EPI_ISL_743331, EPI_ISL_743332, EPI_ISL_743333, EPI_ISL_743334, EPI_ISL_743335, EPI_ISL_743336, EPI_ISL_743337, EPI_ISL_743338, EPI_ISL_743339, EPI_ISL_743340, EPI_ISL_743341, EPI_ISL_743342, EPI_ISL_743343, EPI_ISL_743344, EPI_ISL_743345, EPI_ISL_743346, EPI_ISL_743347, EPI_ISL_743348, EPI_ISL_743349, EPI_ISL_743350, EPI_ISL_743351, EPI_ISL_743352, EPI_ISL_743353, EPI_ISL_743354, EPI_ISL_743355, EPI_ISL_743356, EPI_ISL_743357, EPI_ISL_743358, EPI_ISL_743359, EPI_ISL_743360, EPI_ISL_743361, EPI_ISL_743362, EPI_ISL_743363, EPI_ISL_743364, EPI_ISL_743365, EPI_ISL_743366, EPI_ISL_743367, EPI_ISL_743368, EPI_ISL_743369, EPI_ISL_743370, EPI_ISL_743371, EPI_ISL_743372, EPI_ISL_743373, EPI_ISL_743374, EPI_ISL_743375, EPI_ISL_743376, EPI_ISL_743377, EPI_ISL_743378, EPI_ISL_743379, EPI_ISL_743380, EPI_ISL_743381, EPI_ISL_743382, EPI_ISL_743383, EPI_ISL_743384, EPI_ISL_743385, EPI_ISL_743386, EPI_ISL_743387, EPI_ISL_743388, EPI_ISL_743389, EPI_ISL_743390, EPI_ISL_743391, EPI_ISL_743392, EPI_ISL_743393, EPI_ISL_743394, EPI_ISL_743395, EPI_ISL_743396, EPI_ISL_743397, EPI_ISL_743398, EPI_ISL_743399, EPI_ISL_743400, EPI_ISL_743401, EPI_ISL_743402, EPI_ISL_743403, EPI_ISL_743404, EPI_ISL_743405, EPI_ISL_743406, EPI_ISL_743408, EPI_ISL_743409, EPI_ISL_743410, EPI_ISL_743411, EPI_ISL_743412, EPI_ISL_743413, EPI_ISL_743414, EPI_ISL_743415, EPI_ISL_743416, EPI_ISL_743417, EPI_ISL_743418, EPI_ISL_743419, EPI_ISL_743420, EPI_ISL_743421, EPI_ISL_743422, EPI_ISL_743424, EPI_ISL_743426, EPI_ISL_743427, EPI_ISL_743428, EPI_ISL_743429, EPI_ISL_743430, EPI_ISL_743431, EPI_ISL_743432, EPI_ISL_743433, EPI_ISL_743434, EPI_ISL_743435, EPI_ISL_743436, EPI_ISL_743437, EPI_ISL_743438, EPI_ISL_743439, EPI_ISL_743440, EPI_ISL_743441, EPI_ISL_743442, EPI_ISL_743443, EPI_ISL_743445, EPI_ISL_743446, EPI_ISL_743447, EPI_ISL_743448, EPI_ISL_743449, EPI_ISL_743450, EPI_ISL_743451, EPI_ISL_743452, EPI_ISL_743453, EPI_ISL_743455, EPI_ISL_743456, EPI_ISL_743457, EPI_ISL_743458, EPI_ISL_743459, EPI_ISL_743460, EPI_ISL_743461, EPI_ISL_743462, EPI_ISL_743463, EPI_ISL_743464, EPI_ISL_743465, EPI_ISL_743466, EPI_ISL_743467, EPI_ISL_743468, EPI_ISL_743469, EPI_ISL_743470, EPI_ISL_743471, EPI_ISL_743472, EPI_ISL_743473, EPI_ISL_743474, EPI_ISL_743475, EPI_ISL_743476, EPI_ISL_743477, EPI_ISL_743478, EPI_ISL_743479, EPI_ISL_743480, EPI_ISL_743481, EPI_ISL_743482, EPI_ISL_743483, EPI_ISL_743484, EPI_ISL_743485, EPI_ISL_743486, EPI_ISL_743487, EPI_ISL_743488, EPI_ISL_743489, EPI_ISL_743490, EPI_ISL_743491, EPI_ISL_743492, EPI_ISL_743493, EPI_ISL_743494, EPI_ISL_743495, EPI_ISL_743496, EPI_ISL_743497, EPI_ISL_743498, EPI_ISL_743499, EPI_ISL_743500, EPI_ISL_743501, EPI_ISL_743502, EPI_ISL_743503, EPI_ISL_743504, EPI_ISL_743505, EPI_ISL_743506, EPI_ISL_743507, EPI_ISL_743508, EPI_ISL_743509, EPI_ISL_743510, EPI_ISL_743511, EPI_ISL_743512, EPI_ISL_743513, EPI_ISL_743515, EPI_ISL_743516, EPI_ISL_743517, EPI_ISL_743518, EPI_ISL_743519, EPI_ISL_743520, EPI_ISL_743521, EPI_ISL_743522, EPI_ISL_743523, EPI_ISL_743524, EPI_ISL_743525, EPI_ISL_743526, EPI_ISL_743527, EPI_ISL_743528, EPI_ISL_743529, EPI_ISL_743530, EPI_ISL_743531, EPI_ISL_743532, EPI_ISL_743533, EPI_ISL_743534, EPI_ISL_743535, EPI_ISL_743536, EPI_ISL_743537, EPI_ISL_743538, EPI_ISL_743539, EPI_ISL_743540, EPI_ISL_743541, EPI_ISL_743542, EPI_ISL_743543, EPI_ISL_743544, EPI_ISL_743545, EPI_ISL_743546, EPI_ISL_743547, EPI_ISL_743548, EPI_ISL_743549, EPI_ISL_743550, EPI_ISL_743551, EPI_ISL_743552, EPI_ISL_743553, EPI_ISL_743554, EPI_ISL_743555, EPI_ISL_743556, EPI_ISL_743557, EPI_ISL_743558, EPI_ISL_743559, EPI_ISL_743560, EPI_ISL_743561, EPI_ISL_743562, EPI_ISL_743563, EPI_ISL_743564, EPI_ISL_743565, EPI_ISL_743566, EPI_ISL_743567, EPI_ISL_743568, EPI_ISL_743569, EPI_ISL_743570, EPI_ISL_743571, EPI_ISL_743573, EPI_ISL_743574, EPI_ISL_743575, EPI_ISL_743576, EPI_ISL_743577, EPI_ISL_743578, EPI_ISL_743579, EPI_ISL_743580, EPI_ISL_743581, EPI_ISL_743582, EPI_ISL_743583, EPI_ISL_743584, EPI_ISL_743586, EPI_ISL_743587, EPI_ISL_743588, EPI_ISL_743589, EPI_ISL_743590, EPI_ISL_743591, EPI_ISL_743592, EPI_ISL_743593, EPI_ISL_743594, EPI_ISL_743595, EPI_ISL_743596, EPI_ISL_743597, EPI_ISL_743598, EPI_ISL_743599, EPI_ISL_743600, EPI_ISL_743601, EPI_ISL_743602, EPI_ISL_743603, EPI_ISL_743605, EPI_ISL_743606, EPI_ISL_743608, EPI_ISL_743610, EPI_ISL_743611, EPI_ISL_743613, EPI_ISL_743614, EPI_ISL_743616, EPI_ISL_743617, EPI_ISL_743618, EPI_ISL_743619, EPI_ISL_743622, EPI_ISL_743623, EPI_ISL_743626, EPI_ISL_743628, EPI_ISL_743629, EPI_ISL_743630, EPI_ISL_743631, EPI_ISL_743632, EPI_ISL_743633, EPI_ISL_743634, EPI_ISL_743635, EPI_ISL_743639, EPI_ISL_743640, EPI_ISL_743641, EPI_ISL_743642, EPI_ISL_743643, EPI_ISL_743644, EPI_ISL_743645, EPI_ISL_743646, EPI_ISL_743647, EPI_ISL_743648, EPI_ISL_743649, EPI_ISL_743650, EPI_ISL_743651, EPI_ISL_743652, EPI_ISL_743653, EPI_ISL_743654, EPI_ISL_743655, EPI_ISL_743656, EPI_ISL_743657, EPI_ISL_743658, EPI_ISL_743659, EPI_ISL_743660, EPI_ISL_743661, EPI_ISL_743662, EPI_ISL_743663, EPI_ISL_743664, EPI_ISL_743665, EPI_ISL_743666, EPI_ISL_743667, EPI_ISL_743668, EPI_ISL_743669, EPI_ISL_743670, EPI_ISL_743671, EPI_ISL_743672, EPI_ISL_743673, EPI_ISL_743674, EPI_ISL_743675, EPI_ISL_743676, EPI_ISL_743677, EPI_ISL_743678, EPI_ISL_743679, EPI_ISL_743680, EPI_ISL_743681, EPI_ISL_743682, EPI_ISL_743683, EPI_ISL_743684, EPI_ISL_743685, EPI_ISL_743686, EPI_ISL_743687, EPI_ISL_743688, EPI_ISL_743689, EPI_ISL_743690, EPI_ISL_743691, EPI_ISL_743692, EPI_ISL_743693, EPI_ISL_743694, EPI_ISL_743695, EPI_ISL_743696, EPI_ISL_743697, EPI_ISL_743698, EPI_ISL_743699, EPI_ISL_743700, EPI_ISL_743701, EPI_ISL_743702, EPI_ISL_743703, EPI_ISL_743704, EPI_ISL_743705, EPI_ISL_743706, EPI_ISL_743707, EPI_ISL_743708, EPI_ISL_743709, EPI_ISL_743710, EPI_ISL_743711, EPI_ISL_743712, EPI_ISL_743713, EPI_ISL_743714, EPI_ISL_743715, EPI_ISL_743716, EPI_ISL_743717, EPI_ISL_743718, EPI_ISL_743719, EPI_ISL_743720, EPI_ISL_743721, EPI_ISL_743722, EPI_ISL_743724, EPI_ISL_743725, EPI_ISL_743726, EPI_ISL_743727, EPI_ISL_743728, EPI_ISL_743729, EPI_ISL_743730, EPI_ISL_743731, EPI_ISL_743732, EPI_ISL_743733, EPI_ISL_743734, EPI_ISL_743735, EPI_ISL_743736, EPI_ISL_743737, EPI_ISL_743738, EPI_ISL_743739, EPI_ISL_743740, EPI_ISL_743741, EPI_ISL_743742, EPI_ISL_743743, EPI_ISL_743744, EPI_ISL_743745, EPI_ISL_743746, EPI_ISL_743747, EPI_ISL_743748, EPI_ISL_743749, EPI_ISL_743750, EPI_ISL_743751, EPI_ISL_743752, EPI_ISL_743753, EPI_ISL_743754, EPI_ISL_743755, EPI_ISL_743756, EPI_ISL_743757, EPI_ISL_743758, EPI_ISL_743759, EPI_ISL_743760, EPI_ISL_743761, EPI_ISL_743762, EPI_ISL_743763, EPI_ISL_743764, EPI_ISL_743765, EPI_ISL_743766, EPI_ISL_743767, EPI_ISL_743768, EPI_ISL_743769, EPI_ISL_743770, EPI_ISL_743771, EPI_ISL_743772, EPI_ISL_743773, EPI_ISL_743774, EPI_ISL_743775, EPI_ISL_743776, EPI_ISL_743777, EPI_ISL_743778, EPI_ISL_743779, EPI_ISL_743780, EPI_ISL_743781, EPI_ISL_743782, EPI_ISL_743783, EPI_ISL_743784, EPI_ISL_743785, EPI_ISL_743786, EPI_ISL_743787, EPI_ISL_743788, EPI_ISL_743789, EPI_ISL_743790, EPI_ISL_743791, EPI_ISL_743792, EPI_ISL_743794, EPI_ISL_743795, EPI_ISL_743796, EPI_ISL_743797, EPI_ISL_743798, EPI_ISL_743799, EPI_ISL_743800, EPI_ISL_743804, EPI_ISL_743805, EPI_ISL_743806, EPI_ISL_743807, EPI_ISL_743808, EPI_ISL_743810, EPI_ISL_743811, EPI_ISL_743812, EPI_ISL_743813, EPI_ISL_743814, EPI_ISL_743815, EPI_ISL_743816, EPI_ISL_743817, EPI_ISL_743818, EPI_ISL_743819, EPI_ISL_743822, EPI_ISL_743823, EPI_ISL_743824, EPI_ISL_743825, EPI_ISL_743826, EPI_ISL_743827, EPI_ISL_743828, EPI_ISL_743829, EPI_ISL_743830, EPI_ISL_743831, EPI_ISL_743832, EPI_ISL_743833, EPI_ISL_743834, EPI_ISL_743835, EPI_ISL_743836, EPI_ISL_743837, EPI_ISL_743838, EPI_ISL_743839, EPI_ISL_743840, EPI_ISL_743841, EPI_ISL_743842, EPI_ISL_743843, EPI_ISL_743845, EPI_ISL_743846, EPI_ISL_743847, EPI_ISL_743848, EPI_ISL_743849, EPI_ISL_743850, EPI_ISL_743851, EPI_ISL_743852, EPI_ISL_743853, EPI_ISL_743854, EPI_ISL_743855, EPI_ISL_743856, EPI_ISL_743857, EPI_ISL_743858, EPI_ISL_743859, EPI_ISL_743861, EPI_ISL_743862, EPI_ISL_743863, EPI_ISL_743864, EPI_ISL_743865, EPI_ISL_743866, EPI_ISL_743867, EPI_ISL_743868, EPI_ISL_743869, EPI_ISL_743870, EPI_ISL_743871, EPI_ISL_743872, EPI_ISL_743873, EPI_ISL_743874, EPI_ISL_743875, EPI_ISL_743876, EPI_ISL_743877, EPI_ISL_743878, EPI_ISL_743879, EPI_ISL_743880, EPI_ISL_743881, EPI_ISL_743882, EPI_ISL_743883, EPI_ISL_743884, EPI_ISL_743885, EPI_ISL_743886, EPI_ISL_743887, EPI_ISL_743888, EPI_ISL_743889, EPI_ISL_743890, EPI_ISL_743891, EPI_ISL_743892, EPI_ISL_743893, EPI_ISL_743894, EPI_ISL_743895, EPI_ISL_743896, EPI_ISL_743897, EPI_ISL_743898, EPI_ISL_743899, EPI_ISL_743900, EPI_ISL_743901, EPI_ISL_743902, EPI_ISL_743903, EPI_ISL_743904, EPI_ISL_743905, EPI_ISL_743906, EPI_ISL_743907, EPI_ISL_743908, EPI_ISL_743909, EPI_ISL_743910, EPI_ISL_743911, EPI_ISL_743912, EPI_ISL_743913, EPI_ISL_743914, EPI_ISL_743915, EPI_ISL_743916, EPI_ISL_743917, EPI_ISL_743918, EPI_ISL_743919, EPI_ISL_743920, EPI_ISL_743921, EPI_ISL_743922, EPI_ISL_743923, EPI_ISL_743924, EPI_ISL_743925, EPI_ISL_743926, EPI_ISL_743927, EPI_ISL_743928, EPI_ISL_743929, EPI_ISL_743930, EPI_ISL_743931, EPI_ISL_743932, EPI_ISL_743933, EPI_ISL_743934, EPI_ISL_743935, EPI_ISL_743936, EPI_ISL_743937, EPI_ISL_743938, EPI_ISL_743939, EPI_ISL_743940, EPI_ISL_743941, EPI_ISL_743942, EPI_ISL_743943, EPI_ISL_743944, EPI_ISL_743945, EPI_ISL_743946, EPI_ISL_743947, EPI_ISL_743948, EPI_ISL_743949, EPI_ISL_743950, EPI_ISL_743951, EPI_ISL_743952, EPI_ISL_743953, EPI_ISL_743954, EPI_ISL_743955, EPI_ISL_743956, EPI_ISL_743957, EPI_ISL_743958, EPI_ISL_743959, EPI_ISL_743960, EPI_ISL_743961, EPI_ISL_743962, EPI_ISL_743963, EPI_ISL_743965, EPI_ISL_743966, EPI_ISL_743967, EPI_ISL_743968, EPI_ISL_743969, EPI_ISL_743970, EPI_ISL_743971, EPI_ISL_743972, EPI_ISL_743973, EPI_ISL_743974, EPI_ISL_743975, EPI_ISL_743976, EPI_ISL_743977, EPI_ISL_743978, EPI_ISL_743979, EPI_ISL_743980, EPI_ISL_743982, EPI_ISL_743983, EPI_ISL_743984, EPI_ISL_743985, EPI_ISL_743986, EPI_ISL_743988, EPI_ISL_743989, EPI_ISL_743990, EPI_ISL_743991, EPI_ISL_743992, EPI_ISL_743993, EPI_ISL_743994, EPI_ISL_743995, EPI_ISL_743996, EPI_ISL_743997, EPI_ISL_743998, EPI_ISL_743999, EPI_ISL_744000, EPI_ISL_744001, EPI_ISL_744002, EPI_ISL_744003, EPI_ISL_744004, EPI_ISL_744005, EPI_ISL_744006, EPI_ISL_744007, EPI_ISL_744008, EPI_ISL_744009, EPI_ISL_744010, EPI_ISL_744011, EPI_ISL_744012, EPI_ISL_744013, EPI_ISL_744014, EPI_ISL_744015, EPI_ISL_744016, EPI_ISL_744017, EPI_ISL_744018, EPI_ISL_744019, EPI_ISL_744020, EPI_ISL_744021, EPI_ISL_744022, EPI_ISL_744023, EPI_ISL_744024, EPI_ISL_744025, EPI_ISL_744026, EPI_ISL_744027, EPI_ISL_744028, EPI_ISL_744029, EPI_ISL_744030, EPI_ISL_744031, EPI_ISL_744032, EPI_ISL_744033, EPI_ISL_744034, EPI_ISL_744035, EPI_ISL_744036, EPI_ISL_744037, EPI_ISL_744038, EPI_ISL_744039, EPI_ISL_744040, EPI_ISL_744042, EPI_ISL_744043, EPI_ISL_744044, EPI_ISL_744045, EPI_ISL_744046, EPI_ISL_744047, EPI_ISL_744048, EPI_ISL_744049, EPI_ISL_744050, EPI_ISL_744051, EPI_ISL_744052, EPI_ISL_744053, EPI_ISL_744054, EPI_ISL_744055, EPI_ISL_744056, EPI_ISL_744057, EPI_ISL_744058, EPI_ISL_744059, EPI_ISL_744060, EPI_ISL_744061, EPI_ISL_744062, EPI_ISL_744063, EPI_ISL_744064, EPI_ISL_744065, EPI_ISL_744066, EPI_ISL_744067, EPI_ISL_744068, EPI_ISL_744069, EPI_ISL_744070, EPI_ISL_744071, EPI_ISL_744072, EPI_ISL_744073, EPI_ISL_744074, EPI_ISL_744075, EPI_ISL_744076, EPI_ISL_744077, EPI_ISL_744078, EPI_ISL_744079, EPI_ISL_744080, EPI_ISL_744082, EPI_ISL_744083, EPI_ISL_744084, EPI_ISL_744085, EPI_ISL_744086, EPI_ISL_744087, EPI_ISL_744088, EPI_ISL_744089, EPI_ISL_744090, EPI_ISL_744091, EPI_ISL_744092, EPI_ISL_744093, EPI_ISL_744094, EPI_ISL_744095, EPI_ISL_744096, EPI_ISL_744097, EPI_ISL_744098, EPI_ISL_744099, EPI_ISL_744100, EPI_ISL_744101, EPI_ISL_744102, EPI_ISL_744103, EPI_ISL_744104, EPI_ISL_744105, EPI_ISL_744106, EPI_ISL_744107, EPI_ISL_744108, EPI_ISL_744109, EPI_ISL_744110, EPI_ISL_744111, EPI_ISL_744112, EPI_ISL_744113, EPI_ISL_744114, EPI_ISL_744115, EPI_ISL_744116, EPI_ISL_744117, EPI_ISL_744118, EPI_ISL_744119, EPI_ISL_744120, EPI_ISL_744121, EPI_ISL_744122, EPI_ISL_744123, EPI_ISL_744124, EPI_ISL_744125, EPI_ISL_744126, EPI_ISL_744127, EPI_ISL_744128, EPI_ISL_744129, EPI_ISL_744130, EPI_ISL_744131, EPI_ISL_744132, EPI_ISL_744133, EPI_ISL_744134

see above	Wales Specialist Virology Centre Sequencing lab: Pathogen Genomics Unit	COVID-19 Genomics UK (COG-UK) Consortium	Catherine Moore, Johnathan Evans, Laura Gifford, Malorie Perry, Simon Cottrell, Angela Marchbank, Alec Birchley, Alexander Adams, Amy Gaskin, Bree Gatica-Wilcox, Jason Coombes, Joel Southgate, Lauren Gilbert, Lee Graham, Nicole Pacchiarini, Sara Kumziene-Summerhayes, Sarah Taylor, Sophie Jones, Sara Rey, Matthew Bull, Joanne Watkins, Sally Corden, Tom Connor

EPI_ISL_744135, EPI_ISL_744136, EPI_ISL_744137, EPI_ISL_744138, EPI_ISL_744140, EPI_ISL_744141, EPI_ISL_744142, EPI_ISL_744143, EPI_ISL_744144, EPI_ISL_744145, EPI_ISL_744146

see above	Laboratoire national de santé, Microbiology, Virology	Laboratoire national de santé, Microbiology, Microbial	Anke Wienecke-Baldacchino, Catherine Ragimbeau,Jessica Tapp, Fatu Djabi, Lise Pignon, Raoul Salmon, Tamir Abdelrahman

		Genomics Platform	
EPI_ISL_744147	Laboratoire national de santé, Microbiology, Virology	Laboratoire national de santé, Microbiology, Microbial Genomics Platform	Anke Wienecke-Baldacchino, Catherine Ragimbeau, Tamir Abdelrahman, Jessica Tapp, Fatu Djabi
EPI_ISL_744148, EPI_ISL_744149, EPI_ISL_744150, EPI_ISL_744152, EPI_ISL_744153, EPI_ISL_744154, EPI_ISL_744155, EPI_ISL_744156, EPI_ISL_744157, EPI_ISL_744158, EPI_ISL_744159, EPI_ISL_744160, EPI_ISL_744161, EPI_ISL_744162, EPI_ISL_744163, EPI_ISL_744164, EPI_ISL_744165, EPI_ISL_744166, EPI_ISL_744167, EPI_ISL_744168, EPI_ISL_744169, EPI_ISL_744170, EPI_ISL_744171, EPI_ISL_744172, EPI_ISL_744173, EPI_ISL_744174, EPI_ISL_744175, EPI_ISL_744176, EPI_ISL_744177, EPI_ISL_744178, EPI_ISL_744179, EPI_ISL_744180, EPI_ISL_744181, EPI_ISL_744182, EPI_ISL_744183, EPI_ISL_744184, EPI_ISL_744185, EPI_ISL_744186, EPI_ISL_744187, EPI_ISL_744188, EPI_ISL_744189, EPI_ISL_744190, EPI_ISL_744191, EPI_ISL_744192, EPI_ISL_744193, EPI_ISL_744194, EPI_ISL_744195, EPI_ISL_744196, EPI_ISL_744197			
see above	Laboratoire national de santé, Microbiology, Virology	Laboratoire national de santé, Microbiology, Microbial Genomics Platform	Anke Wienecke-Baldacchino, Catherine Ragimbeau,Jessica Tapp, Fatu Djabi, Lise Pignon, Raoul Salmon, Tamir Abdelrahman
EPI_ISL_744198	Laboratoire national de santé, Microbiology, Virology	Laboratoire national de santé, Microbiology, Microbial Genomics Platform	Anke Wienecke-Baldacchino, Catherine Ragimbeau, Tamir Abdelrahman, Jessica Tapp, Fatu Djabi
EPI_ISL_744199, EPI_ISL_744200, EPI_ISL_744201	Laboratoire national de santé, Microbiology, Virology	Laboratoire national de santé, Microbiology, Microbial Genomics Platform	Anke Wienecke-Baldacchino, Catherine Ragimbeau,Jessica Tapp, Fatu Djabi, Lise Pignon, Raoul Salmon, Tamir Abdelrahman
EPI_ISL_744202	Laboratoire national de santé, Microbiology, Virology	Laboratoire national de santé, Microbiology, Microbial Genomics Platform	Anke Wienecke-Baldacchino, Catherine Ragimbeau, Tamir Abdelrahman, Jessica Tapp, Fatu Djabi
EPI_ISL_744203, EPI_ISL_744204, EPI_ISL_744205, EPI_ISL_744206, EPI_ISL_744207, EPI_ISL_744208, EPI_ISL_744209, EPI_ISL_744210, EPI_ISL_744211, EPI_ISL_744212, EPI_ISL_744213, EPI_ISL_744214, EPI_ISL_744215, EPI_ISL_744216, EPI_ISL_744217, EPI_ISL_744218, EPI_ISL_744219, EPI_ISL_744220, EPI_ISL_744221, EPI_ISL_744222, EPI_ISL_744223, EPI_ISL_744224, EPI_ISL_744225, EPI_ISL_744226, EPI_ISL_744227, EPI_ISL_744228, EPI_ISL_744229, EPI_ISL_744230, EPI_ISL_744231, EPI_ISL_744232, EPI_ISL_744234			
see above	Laboratoire national de santé, Microbiology, Virology	Laboratoire national de santé, Microbiology, Microbial Genomics Platform	Anke Wienecke-Baldacchino, Catherine Ragimbeau,Jessica Tapp, Fatu Djabi, Lise Pignon, Raoul Salmon, Tamir Abdelrahman
EPI_ISL_744235	Laboratoire national de santé, Microbiology, Virology	Laboratoire national de santé, Microbiology, Microbial Genomics Platform	Anke Wienecke-Baldacchino, Catherine Ragimbeau, Tamir Abdelrahman, Jessica Tapp, Fatu Djabi
EPI_ISL_744236, EPI_ISL_744237, EPI_ISL_744238, EPI_ISL_744239, EPI_ISL_744240, EPI_ISL_744241, EPI_ISL_744242, EPI_ISL_744243, EPI_ISL_744244, EPI_ISL_744245, EPI_ISL_744246, EPI_ISL_744247, EPI_ISL_744248			
see above	Laboratoire national de santé, Microbiology, Virology	Laboratoire national de santé, Microbiology, Microbial Genomics Platform	Anke Wienecke-Baldacchino, Catherine Ragimbeau,Jessica Tapp, Fatu Djabi, Lise Pignon, Raoul Salmon, Tamir Abdelrahman
EPI_ISL_744249	Laboratoire national de santé, Microbiology, Virology	Laboratoire national de santé, Microbiology, Microbial Genomics Platform	Anke Wienecke-Baldacchino, Catherine Ragimbeau, Tamir Abdelrahman, Jessica Tapp, Fatu Djabi
EPI_ISL_744250, EPI_ISL_744251, EPI_ISL_744252, EPI_ISL_744253, EPI_ISL_744254, EPI_ISL_744255, EPI_ISL_744256, EPI_ISL_744257, EPI_ISL_744258, EPI_ISL_744259, EPI_ISL_744260, EPI_ISL_744261, EPI_ISL_744262, EPI_ISL_744263, EPI_ISL_744264, EPI_ISL_744265, EPI_ISL_744266, EPI_ISL_744267, EPI_ISL_744268, EPI_ISL_744269, EPI_ISL_744270, EPI_ISL_744271, EPI_ISL_744272, EPI_ISL_744273, EPI_ISL_744275, EPI_ISL_744276, EPI_ISL_744277, EPI_ISL_744278, EPI_ISL_744279, EPI_ISL_744280, EPI_ISL_744281, EPI_ISL_744282, EPI_ISL_744283, EPI_ISL_744284, EPI_ISL_744285, EPI_ISL_744286, EPI_ISL_744287, EPI_ISL_744288, EPI_ISL_744289, EPI_ISL_744290, EPI_ISL_744291, EPI_ISL_744292, EPI_ISL_744293, EPI_ISL_744294			
see above	Laboratoire national de santé, Microbiology, Virology	Laboratoire national de santé, Microbiology, Microbial Genomics Platform	Anke Wienecke-Baldacchino, Catherine Ragimbeau,Jessica Tapp, Fatu Djabi, Lise Pignon, Raoul Salmon, Tamir Abdelrahman
EPI_ISL_744295	Laboratoire national de santé, Microbiology, Virology	Laboratoire national de santé, Microbiology, Epidemiology and Microbial Genomics	Anke Wienecke-Baldacchino, Catherine Ragimbeau, Tamir Abdelrahman, Jessica Tapp, Fatu Djabi, Trung Nguyen Nguyen
EPI_ISL_744296, EPI_ISL_744297, EPI_ISL_744298, EPI_ISL_744299, EPI_ISL_744300, EPI_ISL_744301, EPI_ISL_744302, EPI_ISL_744303, EPI_ISL_744304, EPI_ISL_744305, EPI_ISL_744306, EPI_ISL_744307, EPI_ISL_744308, EPI_ISL_744309, EPI_ISL_744310			
see above	Laboratoire national de santé, Microbiology, Virology	Laboratoire national de santé, Microbiology, Microbial Genomics Platform	Anke Wienecke-Baldacchino, Catherine Ragimbeau,Jessica Tapp, Fatu Djabi, Lise Pignon, Raoul Salmon, Tamir Abdelrahman
EPI_ISL_744311	Laboratoire national de santé, Microbiology, Virology	Laboratoire national de santé, Microbiology, Microbial Genomics Platform	Anke Wienecke-Baldacchino, Catherine Ragimbeau, Tamir Abdelrahman, Jessica Tapp, Fatu Djabi
EPI_ISL_744312, EPI_ISL_744313, EPI_ISL_744314, EPI_ISL_744315, EPI_ISL_744316, EPI_ISL_744317, EPI_ISL_744318, EPI_ISL_744319, EPI_ISL_744320, EPI_ISL_744321, EPI_ISL_744322, EPI_ISL_744323, EPI_ISL_744324, EPI_ISL_744325, EPI_ISL_744326, EPI_ISL_744327, EPI_ISL_744328, EPI_ISL_744329, EPI_ISL_744330, EPI_ISL_744331, EPI_ISL_744332, EPI_ISL_744333, EPI_ISL_744334, EPI_ISL_744335, EPI_ISL_744336, EPI_ISL_744337, EPI_ISL_744338, EPI_ISL_744339, EPI_ISL_744340, EPI_ISL_744341, EPI_ISL_744342, EPI_ISL_744343, EPI_ISL_744344, EPI_ISL_744345, EPI_ISL_744346, EPI_ISL_744347, EPI_ISL_744348, EPI_ISL_744349, EPI_ISL_744350, EPI_ISL_744351, EPI_ISL_744352, EPI_ISL_744353, EPI_ISL_744354, EPI_ISL_744355, EPI_ISL_744356, EPI_ISL_744357, EPI_ISL_744358, EPI_ISL_744359, EPI_ISL_744360, EPI_ISL_744361, EPI_ISL_744362, EPI_ISL_744363, EPI_ISL_744364, EPI_ISL_744365, EPI_ISL_744366			
see above	Laboratoire national de santé, Microbiology, Virology	Laboratoire national de santé, Microbiology, Microbial Genomics Platform	Anke Wienecke-Baldacchino, Catherine Ragimbeau,Jessica Tapp, Fatu Djabi, Lise Pignon, Raoul Salmon, Tamir Abdelrahman
EPI_ISL_744367	Laboratoire national de santé, Microbiology, Virology	Laboratoire national de santé, Microbiology, Microbial Genomics Platform	Anke Wienecke-Baldacchino, Catherine Ragimbeau, Tamir Abdelrahman, Jessica Tapp, Fatu Djabi
EPI_ISL_744368, EPI_ISL_744369, EPI_ISL_744370, EPI_ISL_744371, EPI_ISL_744372, EPI_ISL_744373, EPI_ISL_744374, EPI_ISL_744375, EPI_ISL_744376, EPI_ISL_744377, EPI_ISL_744378, EPI_ISL_744379, EPI_ISL_744380, EPI_ISL_744381, EPI_ISL_744382, EPI_ISL_744383, EPI_ISL_744384, EPI_ISL_744385, EPI_ISL_744386, EPI_ISL_744387, EPI_ISL_744388, EPI_ISL_744389, EPI_ISL_744390, EPI_ISL_744391, EPI_ISL_744392, EPI_ISL_744393, EPI_ISL_744394, EPI_ISL_744395, EPI_ISL_744396, EPI_ISL_744397, EPI_ISL_744398, EPI_ISL_744399, EPI_ISL_744400, EPI_ISL_744401, EPI_ISL_744402, EPI_ISL_744403, EPI_ISL_744404, EPI_ISL_744405			
see above	Laboratoire national de santé, Microbiology, Virology	Laboratoire national de santé, Microbiology, Microbial Genomics Platform	Anke Wienecke-Baldacchino, Catherine Ragimbeau,Jessica Tapp, Fatu Djabi, Lise Pignon, Raoul Salmon, Tamir Abdelrahman
EPI_ISL_744406	Laboratoire national de santé, Microbiology, Virology	Laboratoire national de santé, Microbiology, Microbial Genomics Platform	Anke Wienecke-Baldacchino, Catherine Ragimbeau, Tamir Abdelrahman, Jessica Tapp, Fatu Djabi
EPI_ISL_744407, EPI_ISL_744408, EPI_ISL_744409, EPI_ISL_744410, EPI_ISL_744411, EPI_ISL_744412, EPI_ISL_744413, EPI_ISL_744414, EPI_ISL_744415, EPI_ISL_744416, EPI_ISL_744417, EPI_ISL_744418, EPI_ISL_744419, EPI_ISL_744420, EPI_ISL_744422, EPI_ISL_744423, EPI_ISL_744424, EPI_ISL_744425, EPI_ISL_744426, EPI_ISL_744427, EPI_ISL_744428, EPI_ISL_744429, EPI_ISL_744430, EPI_ISL_744431, EPI_ISL_744432, EPI_ISL_744433			
see above	Laboratoire national de santé, Microbiology, Virology	Laboratoire national de santé, Microbiology, Microbial Genomics Platform	Anke Wienecke-Baldacchino, Catherine Ragimbeau,Jessica Tapp, Fatu Djabi, Lise Pignon, Raoul Salmon, Tamir Abdelrahman
EPI_ISL_744434	Laboratoire national de santé, Microbiology, Virology	Laboratoire national de santé, Microbiology, Microbial Genomics Platform	Anke Wienecke-Baldacchino, Catherine Ragimbeau, Tamir Abdelrahman, Jessica Tapp, Fatu Djabi
EPI_ISL_744435, EPI_ISL_744436, EPI_ISL_744437, EPI_ISL_744438, EPI_ISL_744439, EPI_ISL_744440, EPI_ISL_744441, EPI_ISL_744442, EPI_ISL_744443	Laboratoire national de santé, Microbiology, Virology	Laboratoire national de santé, Microbiology, Microbial Genomics Platform	Anke Wienecke-Baldacchino, Catherine Ragimbeau,Jessica Tapp, Fatu Djabi, Lise Pignon, Raoul Salmon, Tamir Abdelrahman
EPI_ISL_744444	Laboratoire national de santé, Microbiology, Virology	Laboratoire national de santé, Microbiology, Microbial Genomics Platform	Anke Wienecke-Baldacchino, Catherine Ragimbeau, Tamir Abdelrahman, Jessica Tapp, Fatu Djabi
EPI_ISL_744445, EPI_ISL_744446, EPI_ISL_744447, EPI_ISL_744448, EPI_ISL_744449, EPI_ISL_744450, EPI_ISL_744451, EPI_ISL_744452, EPI_ISL_744453, EPI_ISL_744454, EPI_ISL_744455, EPI_ISL_744456, EPI_ISL_744457, EPI_ISL_744458, EPI_ISL_744459, EPI_ISL_744460, EPI_ISL_744461, EPI_ISL_744462, EPI_ISL_744463, EPI_ISL_744465, EPI_ISL_744466, EPI_ISL_744467, EPI_ISL_744468, EPI_ISL_744469, EPI_ISL_744470, EPI_ISL_744471, EPI_ISL_744472, EPI_ISL_744473, EPI_ISL_744474, EPI_ISL_744475, EPI_ISL_744476, EPI_ISL_744479			
see above	Laboratoire national de santé, Microbiology, Virology	Laboratoire national de santé, Microbiology, Microbial Genomics Platform	Anke Wienecke-Baldacchino, Catherine Ragimbeau,Jessica Tapp, Fatu Djabi, Lise Pignon, Raoul Salmon, Tamir Abdelrahman
EPI_ISL_744480	Laboratoire national de santé, Microbiology, Virology	Laboratoire national de santé, Microbiology, Microbial Genomics Platform	Anke Wienecke-Baldacchino, Catherine Ragimbeau, Tamir Abdelrahman, Jessica Tapp, Fatu Djabi

EPI_ISL_744481, EPI_ISL_744482, EPI_ISL_744483, EPI_ISL_744484	Laboratoire national de santé, Microbiology, Virology	Laboratoire national de santé, Microbiology, Microbial Genomics Platform	Anke Wienecke-Baldacchino, Catherine Ragimbeau,Jessica Tapp, Fatu Djabi, Lise Pignon, Raoul Salmon, Tamir Abdelrahman
EPI_ISL_744486	Laboratoire national de santé, Microbiology, Virology	Laboratoire national de santé, Microbiology, Microbial Genomics Platform	Anke Wienecke-Baldacchino, Catherine Ragimbeau, Tamir Abdelrahman, Jessica Tapp, Fatu Djabi
EPI_ISL_744487, EPI_ISL_744488, EPI_ISL_744489	Laboratoire national de santé, Microbiology, Virology	Laboratoire national de santé, Microbiology, Microbial Genomics Platform	Anke Wienecke-Baldacchino, Catherine Ragimbeau,Jessica Tapp, Fatu Djabi, Lise Pignon, Raoul Salmon, Tamir Abdelrahman
EPI_ISL_744490	Laboratoire national de santé, Microbiology, Virology	Laboratoire national de santé, Microbiology, Microbial Genomics Platform	Anke Wienecke-Baldacchino, Catherine Ragimbeau, Tamir Abdelrahman, Jessica Tapp, Fatu Djabi
EPI_ISL_744491, EPI_ISL_744492, EPI_ISL_744493, EPI_ISL_744494, EPI_ISL_744495, EPI_ISL_744496, EPI_ISL_744497, EPI_ISL_744498, EPI_ISL_744499	Laboratoire national de santé, Microbiology, Virology	Laboratoire national de santé, Microbiology, Microbial Genomics Platform	Anke Wienecke-Baldacchino, Catherine Ragimbeau,Jessica Tapp, Fatu Djabi, Lise Pignon, Raoul Salmon, Tamir Abdelrahman
EPI_ISL_744500	Laboratoire national de santé, Microbiology, Virology	Laboratoire national de santé, Microbiology, Microbial Genomics Platform	Anke Wienecke-Baldacchino, Catherine Ragimbeau, Tamir Abdelrahman, Jessica Tapp, Fatu Djabi
EPI_ISL_744501, EPI_ISL_744502, EPI_ISL_744503, EPI_ISL_744504, EPI_ISL_744505, EPI_ISL_744506, EPI_ISL_744507, EPI_ISL_744508, EPI_ISL_744509, EPI_ISL_744510, EPI_ISL_744511, EPI_ISL_744512, EPI_ISL_744513, EPI_ISL_744514, EPI_ISL_744515, EPI_ISL_744516, EPI_ISL_744517, EPI_ISL_744518, EPI_ISL_744519, EPI_ISL_744520, EPI_ISL_744521, EPI_ISL_744522, EPI_ISL_744523, EPI_ISL_744524, EPI_ISL_744525, EPI_ISL_744526, EPI_ISL_744527, EPI_ISL_744528, EPI_ISL_744529, EPI_ISL_744530, EPI_ISL_744531, EPI_ISL_744532, EPI_ISL_744533, EPI_ISL_744534, EPI_ISL_744535, EPI_ISL_744536, EPI_ISL_744537, EPI_ISL_744538, EPI_ISL_744539, EPI_ISL_744540, EPI_ISL_744541, EPI_ISL_744542, EPI_ISL_744543, EPI_ISL_744544, EPI_ISL_744545, EPI_ISL_744546, EPI_ISL_744547, EPI_ISL_744548, EPI_ISL_744549, EPI_ISL_744550, EPI_ISL_744552, EPI_ISL_744553, EPI_ISL_744554, EPI_ISL_744555, EPI_ISL_744556, EPI_ISL_744557, EPI_ISL_744558, EPI_ISL_744559			
see above	Laboratoire national de santé, Microbiology, Virology	Laboratoire national de santé, Microbiology, Microbial Genomics Platform	Anke Wienecke-Baldacchino, Catherine Ragimbeau,Jessica Tapp, Fatu Djabi, Lise Pignon, Raoul Salmon, Tamir Abdelrahman
EPI_ISL_744561	Laboratoire national de santé, Microbiology, Virology	Laboratoire national de santé, Microbiology, Microbial Genomics Platform	Anke Wienecke-Baldacchino, Catherine Ragimbeau, Tamir Abdelrahman, Jessica Tapp, Fatu Djabi
EPI_ISL_744562, EPI_ISL_744563, EPI_ISL_744564, EPI_ISL_744565, EPI_ISL_744566, EPI_ISL_744567, EPI_ISL_744568, EPI_ISL_744569, EPI_ISL_744570, EPI_ISL_744571, EPI_ISL_744572, EPI_ISL_744573, EPI_ISL_744574, EPI_ISL_744575, EPI_ISL_744576, EPI_ISL_744577, EPI_ISL_744578, EPI_ISL_744579, EPI_ISL_744580, EPI_ISL_744581, EPI_ISL_744582, EPI_ISL_744583, EPI_ISL_744584, EPI_ISL_744585			
see above	Laboratoire national de santé, Microbiology, Virology	Laboratoire national de santé, Microbiology, Microbial Genomics Platform	Anke Wienecke-Baldacchino, Catherine Ragimbeau,Jessica Tapp, Fatu Djabi, Lise Pignon, Raoul Salmon, Tamir Abdelrahman
EPI_ISL_744586	Laboratoire national de santé, Microbiology, Virology	Laboratoire national de santé, Microbiology, Microbial Genomics Platform	Anke Wienecke-Baldacchino, Catherine Ragimbeau, Tamir Abdelrahman, Jessica Tapp, Fatu Djabi
EPI_ISL_744587, EPI_ISL_744588, EPI_ISL_744589, EPI_ISL_744590, EPI_ISL_744591, EPI_ISL_744592, EPI_ISL_744593, EPI_ISL_744594, EPI_ISL_744595, EPI_ISL_744596	Laboratoire national de santé, Microbiology, Virology	Laboratoire national de santé, Microbiology, Microbial Genomics Platform	Anke Wienecke-Baldacchino, Catherine Ragimbeau,Jessica Tapp, Fatu Djabi, Lise Pignon, Raoul Salmon, Tamir Abdelrahman
EPI_ISL_744598	Laboratoire national de santé, Microbiology, Virology	Laboratoire national de santé, Microbiology, Microbial Genomics Platform	Anke Wienecke-Baldacchino, Catherine Ragimbeau, Tamir Abdelrahman, Jessica Tapp, Fatu Djabi
EPI_ISL_744599, EPI_ISL_744600, EPI_ISL_744601, EPI_ISL_744602, EPI_ISL_744603, EPI_ISL_744604, EPI_ISL_744605, EPI_ISL_744606, EPI_ISL_744607, EPI_ISL_744608, EPI_ISL_744609, EPI_ISL_744610, EPI_ISL_744611, EPI_ISL_744612, EPI_ISL_744613, EPI_ISL_744614, EPI_ISL_744615, EPI_ISL_744616, EPI_ISL_744617, EPI_ISL_744618, EPI_ISL_744619, EPI_ISL_744620, EPI_ISL_744621, EPI_ISL_744622, EPI_ISL_744623, EPI_ISL_744624, EPI_ISL_744625, EPI_ISL_744626, EPI_ISL_744627, EPI_ISL_744628, EPI_ISL_744629, EPI_ISL_744630			
see above	Laboratoire national de santé, Microbiology, Virology	Laboratoire national de santé, Microbiology, Microbial Genomics Platform	Anke Wienecke-Baldacchino, Catherine Ragimbeau,Jessica Tapp, Fatu Djabi, Lise Pignon, Raoul Salmon, Tamir Abdelrahman
EPI_ISL_744631	Laboratoire national de santé, Microbiology, Virology	Laboratoire national de santé, Microbiology, Microbial Genomics Platform	Anke Wienecke-Baldacchino, Catherine Ragimbeau, Tamir Abdelrahman, Jessica Tapp, Fatu Djabi
EPI_ISL_744632, EPI_ISL_744633, EPI_ISL_744634, EPI_ISL_744635, EPI_ISL_744636, EPI_ISL_744637, EPI_ISL_744638, EPI_ISL_744639, EPI_ISL_744640, EPI_ISL_744641, EPI_ISL_744642, EPI_ISL_744643, EPI_ISL_744645, EPI_ISL_744646, EPI_ISL_744647, EPI_ISL_744648, EPI_ISL_744649, EPI_ISL_744650, EPI_ISL_744651, EPI_ISL_744653, EPI_ISL_744654, EPI_ISL_744655, EPI_ISL_744656, EPI_ISL_744657, EPI_ISL_744658, EPI_ISL_744659, EPI_ISL_744660, EPI_ISL_744661, EPI_ISL_744662, EPI_ISL_744663, EPI_ISL_744664, EPI_ISL_744665, EPI_ISL_744666, EPI_ISL_744667, EPI_ISL_744668, EPI_ISL_744669, EPI_ISL_744670, EPI_ISL_744671, EPI_ISL_744672, EPI_ISL_744673, EPI_ISL_744674, EPI_ISL_744675, EPI_ISL_744676, EPI_ISL_744677, EPI_ISL_744678, EPI_ISL_744679, EPI_ISL_744680			
see above	Laboratoire national de santé, Microbiology, Virology	Laboratoire national de santé, Microbiology, Microbial Genomics Platform	Anke Wienecke-Baldacchino, Catherine Ragimbeau,Jessica Tapp, Fatu Djabi, Lise Pignon, Raoul Salmon, Tamir Abdelrahman
EPI_ISL_744681	Laboratoire national de santé, Microbiology, Virology	Laboratoire national de santé, Microbiology, Microbial Genomics Platform	Anke Wienecke-Baldacchino, Catherine Ragimbeau, Tamir Abdelrahman, Jessica Tapp, Fatu Djabi
EPI_ISL_744682, EPI_ISL_744683, EPI_ISL_744684, EPI_ISL_744685, EPI_ISL_744686, EPI_ISL_744687, EPI_ISL_744688, EPI_ISL_744689, EPI_ISL_744690, EPI_ISL_744691, EPI_ISL_744692, EPI_ISL_744693, EPI_ISL_744694, EPI_ISL_744695			
see above	Laboratoire national de santé, Microbiology, Virology	Laboratoire national de santé, Microbiology, Microbial Genomics Platform	Anke Wienecke-Baldacchino, Catherine Ragimbeau,Jessica Tapp, Fatu Djabi, Lise Pignon, Raoul Salmon, Tamir Abdelrahman
EPI_ISL_744696	Laboratoire national de santé, Microbiology, Virology	Laboratoire national de santé, Microbiology, Microbial Genomics Platform	Anke Wienecke-Baldacchino, Catherine Ragimbeau, Tamir Abdelrahman, Jessica Tapp, Fatu Djabi
EPI_ISL_744697, EPI_ISL_744698, EPI_ISL_744699, EPI_ISL_744700, EPI_ISL_744701, EPI_ISL_744702, EPI_ISL_744703, EPI_ISL_744704, EPI_ISL_744705, EPI_ISL_744706, EPI_ISL_744707, EPI_ISL_744708, EPI_ISL_744709, EPI_ISL_744710			
see above	Laboratoire national de santé, Microbiology, Virology	Laboratoire national de santé, Microbiology, Microbial Genomics Platform	Anke Wienecke-Baldacchino, Catherine Ragimbeau,Jessica Tapp, Fatu Djabi, Lise Pignon, Raoul Salmon, Tamir Abdelrahman
EPI_ISL_744711	Laboratoire national de santé, Microbiology, Virology	Laboratoire national de santé, Microbiology, Microbial Genomics Platform	Anke Wienecke-Baldacchino, Catherine Ragimbeau, Tamir Abdelrahman, Jessica Tapp, Fatu Djabi
EPI_ISL_744712, EPI_ISL_744713, EPI_ISL_744714, EPI_ISL_744715, EPI_ISL_744716, EPI_ISL_744717, EPI_ISL_744718	Laboratoire national de santé, Microbiology, Virology	Laboratoire national de santé, Microbiology, Microbial Genomics Platform	Anke Wienecke-Baldacchino, Catherine Ragimbeau,Jessica Tapp, Fatu Djabi, Lise Pignon, Raoul Salmon, Tamir Abdelrahman
EPI_ISL_744719, EPI_ISL_744720	Laboratoire national de santé, Microbiology, Virology	Laboratoire national de santé, Microbiology, Microbial Genomics Platform	Anke Wienecke-Baldacchino, Catherine Ragimbeau, Tamir Abdelrahman, Jessica Tapp, Fatu Djabi
EPI_ISL_744721, EPI_ISL_744722	Laboratoire national de santé, Microbiology, Virology	Laboratoire national de santé, Microbiology, Microbial Genomics Platform	Anke Wienecke-Baldacchino, Catherine Ragimbeau,Jessica Tapp, Fatu Djabi, Lise Pignon, Raoul Salmon, Tamir Abdelrahman
EPI_ISL_744724	Laboratoire national de santé, Microbiology, Virology	Laboratoire national de santé, Microbiology, Microbial Genomics Platform	Anke Wienecke-Baldacchino, Catherine Ragimbeau, Tamir Abdelrahman, Jessica Tapp, Fatu Djabi
EPI_ISL_744725, EPI_ISL_744726, EPI_ISL_744727, EPI_ISL_744728, EPI_ISL_744729, EPI_ISL_744730, EPI_ISL_744731, EPI_ISL_744732, EPI_ISL_744733, EPI_ISL_744734, EPI_ISL_744735, EPI_ISL_744736, EPI_ISL_744737, EPI_ISL_744738, EPI_ISL_744739, EPI_ISL_744740, EPI_ISL_744741, EPI_ISL_744742, EPI_ISL_744743, EPI_ISL_744744, EPI_ISL_744745, EPI_ISL_744746, EPI_ISL_744747, EPI_ISL_744748, EPI_ISL_744749, EPI_ISL_744750			
see above	Laboratoire national de santé, Microbiology, Virology	Laboratoire national de santé, Microbiology, Microbial	Anke Wienecke-Baldacchino, Catherine Ragimbeau,Jessica Tapp, Fatu Djabi, Lise Pignon, Raoul Salmon, Tamir Abdelrahman

		Genomics Platform	
EPI_ISL_744751	Laboratoire national de santé, Microbiology, Virology	Laboratoire national de santé, Microbiology, Microbial Genomics Platform	Anke Wienecke-Baldacchino, Catherine Ragimbeau, Tamir Abdelrahman, Jessica Tapp, Fatu Djabi
EPI_ISL_744752, EPI_ISL_744753, EPI_ISL_744754, EPI_ISL_744755, EPI_ISL_744756, EPI_ISL_744757, EPI_ISL_744758, EPI_ISL_744759, EPI_ISL_744760, EPI_ISL_744761, EPI_ISL_744762, EPI_ISL_744763, EPI_ISL_744764, EPI_ISL_744765			
see above	Laboratoire national de santé, Microbiology, Virology	Laboratoire national de santé, Microbiology, Microbial Genomics Platform	Anke Wienecke-Baldacchino, Catherine Ragimbeau,Jessica Tapp, Fatu Djabi, Lise Pignon, Raoul Salmon, Tamir Abdelrahman
EPI_ISL_744766	Laboratoire national de santé, Microbiology, Virology	Laboratoire national de santé, Microbiology, Microbial Genomics Platform	Anke Wienecke-Baldacchino, Catherine Ragimbeau, Tamir Abdelrahman, Jessica Tapp, Fatu Djabi
EPI_ISL_744767, EPI_ISL_744768, EPI_ISL_744769, EPI_ISL_744770, EPI_ISL_744771, EPI_ISL_744772, EPI_ISL_744773, EPI_ISL_744774, EPI_ISL_744775, EPI_ISL_744776, EPI_ISL_744777, EPI_ISL_744778, EPI_ISL_744779, EPI_ISL_744780, EPI_ISL_744781, EPI_ISL_744782, EPI_ISL_744783, EPI_ISL_744784, EPI_ISL_744785, EPI_ISL_744786, EPI_ISL_744787, EPI_ISL_744788, EPI_ISL_744789, EPI_ISL_744790, EPI_ISL_744791, EPI_ISL_744792, EPI_ISL_744793, EPI_ISL_744794, EPI_ISL_744795, EPI_ISL_744796, EPI_ISL_744797, EPI_ISL_744798, EPI_ISL_744799, EPI_ISL_744800, EPI_ISL_744801, EPI_ISL_744802, EPI_ISL_744803, EPI_ISL_744804, EPI_ISL_744805, EPI_ISL_744806, EPI_ISL_744807, EPI_ISL_744808, EPI_ISL_744809, EPI_ISL_744810			
see above	Laboratoire national de santé, Microbiology, Virology	Laboratoire national de santé, Microbiology, Microbial Genomics Platform	Anke Wienecke-Baldacchino, Catherine Ragimbeau,Jessica Tapp, Fatu Djabi, Lise Pignon, Raoul Salmon, Tamir Abdelrahman
EPI_ISL_744811	Laboratoire national de santé, Microbiology, Virology	Laboratoire national de santé, Microbiology, Microbial Genomics Platform	Anke Wienecke-Baldacchino, Catherine Ragimbeau, Tamir Abdelrahman, Jessica Tapp, Fatu Djabi
EPI_ISL_744812, EPI_ISL_744813, EPI_ISL_744814, EPI_ISL_744815, EPI_ISL_744816, EPI_ISL_744817, EPI_ISL_744818, EPI_ISL_744820, EPI_ISL_744821, EPI_ISL_744822, EPI_ISL_744823, EPI_ISL_744824, EPI_ISL_744825, EPI_ISL_744826, EPI_ISL_744827, EPI_ISL_744828, EPI_ISL_744829, EPI_ISL_744831, EPI_ISL_744832, EPI_ISL_744833, EPI_ISL_744834, EPI_ISL_744835, EPI_ISL_744836, EPI_ISL_744837, EPI_ISL_744838, EPI_ISL_744839, EPI_ISL_744840, EPI_ISL_744841, EPI_ISL_744842, EPI_ISL_744844, EPI_ISL_744845, EPI_ISL_744846, EPI_ISL_744847, EPI_ISL_744848, EPI_ISL_744849, EPI_ISL_744850, EPI_ISL_744851, EPI_ISL_744852, EPI_ISL_744853, EPI_ISL_744854, EPI_ISL_744855, EPI_ISL_744856, EPI_ISL_744857, EPI_ISL_744858, EPI_ISL_744859, EPI_ISL_744860, EPI_ISL_744861, EPI_ISL_744862, EPI_ISL_744863, EPI_ISL_744864, EPI_ISL_744865, EPI_ISL_744866, EPI_ISL_744867, EPI_ISL_744868, EPI_ISL_744869, EPI_ISL_744871, EPI_ISL_744872, EPI_ISL_744873			
see above	Laboratoire national de santé, Microbiology, Virology	Laboratoire national de santé, Microbiology, Microbial Genomics Platform	Anke Wienecke-Baldacchino, Catherine Ragimbeau,Jessica Tapp, Fatu Djabi, Lise Pignon, Raoul Salmon, Tamir Abdelrahman
EPI_ISL_744874	Laboratoire national de santé, Microbiology, Virology	Laboratoire national de santé, Microbiology, Microbial Genomics Platform	Anke Wienecke-Baldacchino, Catherine Ragimbeau, Tamir Abdelrahman, Jessica Tapp, Fatu Djabi
EPI_ISL_744875, EPI_ISL_744876, EPI_ISL_744877, EPI_ISL_744878, EPI_ISL_744879, EPI_ISL_744880, EPI_ISL_744881, EPI_ISL_744882, EPI_ISL_744883, EPI_ISL_744884, EPI_ISL_744885			
see above	Laboratoire national de santé, Microbiology, Virology	Laboratoire national de santé, Microbiology, Microbial Genomics Platform	Anke Wienecke-Baldacchino, Catherine Ragimbeau,Jessica Tapp, Fatu Djabi, Lise Pignon, Raoul Salmon, Tamir Abdelrahman
EPI_ISL_744886	Laboratoire national de santé, Microbiology, Virology	Laboratoire national de santé, Microbiology, Microbial Genomics Platform	Anke Wienecke-Baldacchino, Catherine Ragimbeau, Tamir Abdelrahman, Jessica Tapp, Fatu Djabi
EPI_ISL_744887, EPI_ISL_744888, EPI_ISL_744889, EPI_ISL_744890, EPI_ISL_744891, EPI_ISL_744892, EPI_ISL_744893, EPI_ISL_744895, EPI_ISL_744896, EPI_ISL_744897, EPI_ISL_744898, EPI_ISL_744899, EPI_ISL_744900, EPI_ISL_744901, EPI_ISL_744902, EPI_ISL_744903, EPI_ISL_744904, EPI_ISL_744905, EPI_ISL_744906, EPI_ISL_744907, EPI_ISL_744909, EPI_ISL_744910, EPI_ISL_744911, EPI_ISL_744912, EPI_ISL_744913, EPI_ISL_744914, EPI_ISL_744915, EPI_ISL_744916, EPI_ISL_744917, EPI_ISL_744918, EPI_ISL_744919, EPI_ISL_744920, EPI_ISL_744921, EPI_ISL_744922, EPI_ISL_744923, EPI_ISL_744924, EPI_ISL_744925, EPI_ISL_744926, EPI_ISL_744927, EPI_ISL_744928, EPI_ISL_744929, EPI_ISL_744930, EPI_ISL_744931, EPI_ISL_744932, EPI_ISL_744933, EPI_ISL_744934, EPI_ISL_744935, EPI_ISL_744936, EPI_ISL_744937, EPI_ISL_744938, EPI_ISL_744939, EPI_ISL_744940, EPI_ISL_744941, EPI_ISL_744942, EPI_ISL_744943, EPI_ISL_744944, EPI_ISL_744945, EPI_ISL_744946, EPI_ISL_744947, EPI_ISL_744948, EPI_ISL_744949, EPI_ISL_744950, EPI_ISL_744951, EPI_ISL_744952, EPI_ISL_744953			
see above	Laboratoire national de santé, Microbiology, Virology	Laboratoire national de santé, Microbiology, Microbial Genomics Platform	Anke Wienecke-Baldacchino, Catherine Ragimbeau,Jessica Tapp, Fatu Djabi, Lise Pignon, Raoul Salmon, Tamir Abdelrahman
EPI_ISL_744954	Laboratoire national de santé, Microbiology, Virology	Laboratoire national de santé, Microbiology, Microbial Genomics Platform	Anke Wienecke-Baldacchino, Catherine Ragimbeau, Tamir Abdelrahman, Jessica Tapp, Fatu Djabi
EPI_ISL_744955, EPI_ISL_744956, EPI_ISL_744957, EPI_ISL_744959, EPI_ISL_744960, EPI_ISL_744961, EPI_ISL_744962, EPI_ISL_744963, EPI_ISL_744964, EPI_ISL_744965, EPI_ISL_744966, EPI_ISL_744967			
see above	Laboratoire national de santé, Microbiology, Virology	Laboratoire national de santé, Microbiology, Microbial Genomics Platform	Anke Wienecke-Baldacchino, Catherine Ragimbeau,Jessica Tapp, Fatu Djabi, Lise Pignon, Raoul Salmon, Tamir Abdelrahman
EPI_ISL_744968	Laboratoire national de santé, Microbiology, Virology	Laboratoire national de santé, Microbiology, Microbial Genomics Platform	Anke Wienecke-Baldacchino, Catherine Ragimbeau, Tamir Abdelrahman, Jessica Tapp, Fatu Djabi
EPI_ISL_744969, EPI_ISL_744970, EPI_ISL_744971, EPI_ISL_744972, EPI_ISL_744973, EPI_ISL_744974, EPI_ISL_744975, EPI_ISL_744976, EPI_ISL_744977, EPI_ISL_744978, EPI_ISL_744979, EPI_ISL_744980, EPI_ISL_744981, EPI_ISL_744982, EPI_ISL_744983, EPI_ISL_744984, EPI_ISL_744985, EPI_ISL_744986, EPI_ISL_744987, EPI_ISL_744988, EPI_ISL_744989, EPI_ISL_744990, EPI_ISL_744991, EPI_ISL_744992, EPI_ISL_744993, EPI_ISL_744994, EPI_ISL_744995, EPI_ISL_744996, EPI_ISL_744997, EPI_ISL_744998, EPI_ISL_744999, EPI_ISL_745000, EPI_ISL_745001, EPI_ISL_745002, EPI_ISL_745003, EPI_ISL_745004, EPI_ISL_745005, EPI_ISL_745006, EPI_ISL_745007, EPI_ISL_745008, EPI_ISL_745009, EPI_ISL_745010, EPI_ISL_745012			
see above	Laboratoire national de santé, Microbiology, Virology	Laboratoire national de santé, Microbiology, Microbial Genomics Platform	Anke Wienecke-Baldacchino, Catherine Ragimbeau,Jessica Tapp, Fatu Djabi, Lise Pignon, Raoul Salmon, Tamir Abdelrahman
EPI_ISL_745013	Laboratoire national de santé, Microbiology, Virology	Laboratoire national de santé, Microbiology, Microbial Genomics Platform	Anke Wienecke-Baldacchino, Catherine Ragimbeau, Tamir Abdelrahman, Jessica Tapp, Fatu Djabi
EPI_ISL_745014, EPI_ISL_745015, EPI_ISL_745016, EPI_ISL_745017, EPI_ISL_745018, EPI_ISL_745019, EPI_ISL_745020, EPI_ISL_745021	Laboratoire national de santé, Microbiology, Virology	Laboratoire national de santé, Microbiology, Microbial Genomics Platform	Anke Wienecke-Baldacchino, Catherine Ragimbeau,Jessica Tapp, Fatu Djabi, Lise Pignon, Raoul Salmon, Tamir Abdelrahman
EPI_ISL_745022	Laboratoire national de santé, Microbiology, Virology	Laboratoire national de santé, Microbiology, Microbial Genomics Platform	Anke Wienecke-Baldacchino, Catherine Ragimbeau, Tamir Abdelrahman, Jessica Tapp, Fatu Djabi
EPI_ISL_745023, EPI_ISL_745025, EPI_ISL_745026, EPI_ISL_745027	Laboratoire national de santé, Microbiology, Virology	Laboratoire national de santé, Microbiology, Microbial Genomics Platform	Anke Wienecke-Baldacchino, Catherine Ragimbeau,Jessica Tapp, Fatu Djabi, Lise Pignon, Raoul Salmon, Tamir Abdelrahman
EPI_ISL_745029, EPI_ISL_745030, EPI_ISL_745031	Area of Virology, Serology and Virology Division (SAViD), New South Wales Health Pathology Randwick	Area of Virology, Serology and Virology Division (SAViD), New South Wales Health Pathology Randwick	Rawlinson, W.
EPI_ISL_745033	West Java Health Laboratory	West Java Health Laboratory	Azzania Fibriani, Ema Rahmawati, Ryan Bayusantika Ristandi, Rifky Waluyajati Rachman, Cut Nur Cinthia Alamanda, Isak Solihin, Rini Robiani, Miftahul Faridl, Karimatu Khoirunnisa
EPI_ISL_745034, EPI_ISL_745035, EPI_ISL_745036, EPI_ISL_745037, EPI_ISL_745038, EPI_ISL_745039, EPI_ISL_745040, EPI_ISL_745041, EPI_ISL_745042, EPI_ISL_745043, EPI_ISL_745044, EPI_ISL_745045, EPI_ISL_745046, EPI_ISL_745047, EPI_ISL_745048, EPI_ISL_745049, EPI_ISL_745050, EPI_ISL_745051, EPI_ISL_745052, EPI_ISL_745053, EPI_ISL_745054, EPI_ISL_745056, EPI_ISL_745057, EPI_ISL_745058, EPI_ISL_745059, EPI_ISL_745061, EPI_ISL_745062, EPI_ISL_745063, EPI_ISL_745064, EPI_ISL_745065, EPI_ISL_745066, EPI_ISL_745067, EPI_ISL_745068, EPI_ISL_745069, EPI_ISL_745070, EPI_ISL_745071, EPI_ISL_745072, EPI_ISL_745073, EPI_ISL_745074, EPI_ISL_745075, EPI_ISL_745076, EPI_ISL_745077, EPI_ISL_745078, EPI_ISL_745079, EPI_ISL_745080, EPI_ISL_745081, EPI_ISL_745083, EPI_ISL_745084, EPI_ISL_745085, EPI_ISL_745086			
see above	Israel Central Virology laboratory	Israel Central Virology laboratory	Neta Zuckerman, Efrat Dahan Bucris, Oran Erster, Michal Mandelboim, Orna Mor, Ella Mendelson
EPI_ISL_745091, EPI_ISL_745092	Australian Clinical Labs	CIDM-PH, Westmead Hospital	CIDM-PH et al.
EPI_ISL_745093, EPI_ISL_745094	Histopath	CIDM-PH, Westmead Hospital	CIDM-PH et al.
EPI_ISL_745095	Laverty Pathology	CIDM-PH, Westmead Hospital	CIDM-PH et al.
EPI_ISL_745096	Pathology North - Hunter - NSW Health Pathology	CIDM-PH, Westmead Hospital	CIDM-PH et al.
EPI_ISL_745097, EPI_ISL_745098, EPI_ISL_745099, EPI_ISL_745100, EPI_ISL_745101, EPI_ISL_745102, EPI_ISL_745103, EPI_ISL_745104, EPI_ISL_745105, EPI_ISL_745106, EPI_ISL_745107			
see above	Pathology North - Royal North Shore Hospital - NSW Health	CIDM-PH, Westmead Hospital	CIDM-PH et al.

	Pathology		
EPI_ISL_745108	St Vincent's Pathology (SydPath)	CIDM-PH, Westmead Hospital	CIDM-PH et al.
EPI_ISL_745109	Kraaifontein CHC wc KFP	National Health Laboratory Service (NHLS), Tygerberg	Susan Engelbrecht, Kayla Delaney, Bronwyn Kleinhans, Houriiyah Tegally, Eduan Wilkindon, Gert van Zyl, Wolfgang Preiser, Tulio de Oliveira
EPI_ISL_745110	Elsies River CHC wc ERP	National Health Laboratory Service (NHLS), Tygerberg	Susan Engelbrecht, Kayla Delaney, Bronwyn Kleinhans, Houriiyah Tegally, Eduan Wilkindon, Gert van Zyl, Wolfgang Preiser, Tulio de Oliveira
EPI_ISL_745111	Khayelitsha (Site B) CHC wc KHC	National Health Laboratory Service (NHLS), Tygerberg	Susan Engelbrecht, Kayla Delaney, Bronwyn Kleinhans, Houriiyah Tegally, Eduan Wilkindon, Gert van Zyl, Wolfgang Preiser, Tulio de Oliveira
EPI_ISL_745112	Dr Van Niekerk Hospital	National Health Laboratory Service (NHLS), Tygerberg	Susan Engelbrecht, Kayla Delaney, Bronwyn Kleinhans, Houriiyah Tegally, Eduan Wilkindon, Gert van Zyl, Wolfgang Preiser, Tulio de Oliveira
EPI_ISL_745113	Dr Ivan Toms Clinic wc IVT	National Health Laboratory Service (NHLS), Tygerberg	Susan Engelbrecht, Kayla Delaney, Bronwyn Kleinhans, Houriiyah Tegally, Eduan Wilkindon, Gert van Zyl, Wolfgang Preiser, Tulio de Oliveira
EPI_ISL_745114, EPI_ISL_745115, EPI_ISL_745116	Khayelitsha Hospital wc KHA	National Health Laboratory Service (NHLS), Tygerberg	Susan Engelbrecht, Kayla Delaney, Bronwyn Kleinhans, Houriiyah Tegally, Eduan Wilkindon, Gert van Zyl, Wolfgang Preiser, Tulio de Oliveira
EPI_ISL_745117	Vredenburg Hospital wc VBG	National Health Laboratory Service (NHLS), Tygerberg	Susan Engelbrecht, Kayla Delaney, Bronwyn Kleinhans, Houriiyah Tegally, Eduan Wilkindon, Gert van Zyl, Wolfgang Preiser, Tulio de Oliveira
EPI_ISL_745118	Bishop Lavis CDC wc BLP	National Health Laboratory Service (NHLS), Tygerberg	Susan Engelbrecht, Kayla Delaney, Bronwyn Kleinhans, Houriiyah Tegally, Eduan Wilkindon, Gert van Zyl, Wolfgang Preiser, Tulio de Oliveira
EPI_ISL_745119	Eerste River Hospital wc ERH	National Health Laboratory Service (NHLS), Tygerberg	Susan Engelbrecht, Kayla Delaney, Bronwyn Kleinhans, Houriiyah Tegally, Eduan Wilkindon, Gert van Zyl, Wolfgang Preiser, Tulio de Oliveira
EPI_ISL_745120	Khayelitsha Hospital wc KHA	National Health Laboratory Service (NHLS), Tygerberg	Susan Engelbrecht, Kayla Delaney, Bronwyn Kleinhans, Houriiyah Tegally, Eduan Wilkindon, Gert van Zyl, Wolfgang Preiser, Tulio de Oliveira
EPI_ISL_745121	Calvinia Hospital	National Health Laboratory Service (NHLS), Tygerberg	Susan Engelbrecht, Kayla Delaney, Bronwyn Kleinhans, Houriiyah Tegally, Eduan Wilkindon, Gert van Zyl, Wolfgang Preiser, Tulio de Oliveira
EPI_ISL_745122, EPI_ISL_745123	Khayelitsha Hospital wc KHA	National Health Laboratory Service (NHLS), Tygerberg	Susan Engelbrecht, Kayla Delaney, Bronwyn Kleinhans, Houriiyah Tegally, Eduan Wilkindon, Gert van Zyl, Wolfgang Preiser, Tulio de Oliveira
EPI_ISL_745124	Laetitia Bam CHC	National Health Laboratory Service (NHLS), Tygerberg	Susan Engelbrecht, Kayla Delaney, Bronwyn Kleinhans, Houriiyah Tegally, Eduan Wilkindon, Gert van Zyl, Wolfgang Preiser, Tulio de Oliveira
EPI_ISL_745125	Elsies River CHC wc ERP	National Health Laboratory Service (NHLS), Tygerberg	Susan Engelbrecht, Kayla Delaney, Bronwyn Kleinhans, Houriiyah Tegally, Eduan Wilkindon, Gert van Zyl, Wolfgang Preiser, Tulio de Oliveira
EPI_ISL_745126	Okiep CHC	National Health Laboratory Service (NHLS), Tygerberg	Susan Engelbrecht, Kayla Delaney, Bronwyn Kleinhans, Houriiyah Tegally, Eduan Wilkindon, Gert van Zyl, Wolfgang Preiser, Tulio de Oliveira
EPI_ISL_745127	Khayelitsha (Site B) CHC wc KHC	National Health Laboratory Service (NHLS), Tygerberg	Susan Engelbrecht, Kayla Delaney, Bronwyn Kleinhans, Houriiyah Tegally, Eduan Wilkindon, Gert van Zyl, Wolfgang Preiser, Tulio de Oliveira
EPI_ISL_745128	CoVid EC Nelson Mandela Bay Metro	National Health Laboratory Service (NHLS), Tygerberg	Susan Engelbrecht, Kayla Delaney, Bronwyn Kleinhans, Houriiyah Tegally, Eduan Wilkindon, Gert van Zyl, Wolfgang Preiser, Tulio de Oliveira
EPI_ISL_745129	Port Nolloth Hospital	National Health Laboratory Service (NHLS), Tygerberg	Susan Engelbrecht, Kayla Delaney, Bronwyn Kleinhans, Houriiyah Tegally, Eduan Wilkindon, Gert van Zyl, Wolfgang Preiser, Tulio de Oliveira
EPI_ISL_745130	Langebaan Clinic wc LBC	National Health Laboratory Service (NHLS), Tygerberg	Susan Engelbrecht, Kayla Delaney, Bronwyn Kleinhans, Houriiyah Tegally, Eduan Wilkindon, Gert van Zyl, Wolfgang Preiser, Tulio de Oliveira
EPI_ISL_745131	Kraaifontein CHC wc KFP	National Health Laboratory Service (NHLS), Tygerberg	Susan Engelbrecht, Kayla Delaney, Bronwyn Kleinhans, Houriiyah Tegally, Eduan Wilkindon, Gert van Zyl, Wolfgang Preiser, Tulio de Oliveira
EPI_ISL_745132	Dr Ivan Toms Clinic wc IVT	National Health Laboratory Service (NHLS), Tygerberg	Susan Engelbrecht, Kayla Delaney, Bronwyn Kleinhans, Houriiyah Tegally, Eduan Wilkindon, Gert van Zyl, Wolfgang Preiser, Tulio de Oliveira
EPI_ISL_745133	Kraaifontein CHC wc KFP	National Health Laboratory Service (NHLS), Tygerberg	Susan Engelbrecht, Kayla Delaney, Bronwyn Kleinhans, Houriiyah Tegally, Eduan Wilkindon, Gert van Zyl, Wolfgang Preiser, Tulio de Oliveira
EPI_ISL_745134	Bishop Lavis CDC wc BLP	National Health Laboratory Service (NHLS), Tygerberg	Susan Engelbrecht, Kayla Delaney, Bronwyn Kleinhans, Houriiyah Tegally, Eduan Wilkindon, Gert van Zyl, Wolfgang Preiser, Tulio de Oliveira
EPI_ISL_745135	Joe Slovo Hospital	National Health Laboratory Service (NHLS), Tygerberg	Susan Engelbrecht, Kayla Delaney, Bronwyn Kleinhans, Houriiyah Tegally, Eduan Wilkindon, Gert van Zyl, Wolfgang Preiser, Tulio de Oliveira
EPI_ISL_745136	Kraaifontein CHC wc KFP	National Health Laboratory Service (NHLS), Tygerberg	Susan Engelbrecht, Kayla Delaney, Bronwyn Kleinhans, Houriiyah Tegally, Eduan Wilkindon, Gert van Zyl, Wolfgang Preiser, Tulio de Oliveira
EPI_ISL_745140, EPI_ISL_745141	Tygerberg Hospital wc TBH	National Health Laboratory Service (NHLS), Tygerberg	Susan Engelbrecht, Kayla Delaney, Bronwyn Kleinhans, Houriiyah Tegally, Eduan Wilkindon, Gert van Zyl, Wolfgang Preiser, Tulio de Oliveira
EPI_ISL_745142, EPI_ISL_745143	Nomangesi Jayiya Clinic	National Health Laboratory Service (NHLS), Tygerberg	Susan Engelbrecht, Kayla Delaney, Bronwyn Kleinhans, Houriiyah Tegally, Eduan Wilkindon, Gert van Zyl, Wolfgang Preiser, Tulio de Oliveira
EPI_ISL_745144	Joe Slovo Hospital	National Health Laboratory Service (NHLS), Tygerberg	Susan Engelbrecht, Kayla Delaney, Bronwyn Kleinhans, Houriiyah Tegally, Eduan Wilkindon, Gert van Zyl, Wolfgang Preiser, Tulio de Oliveira
EPI_ISL_745145	Khayelitsha Hospital wc KHA	National Health Laboratory Service (NHLS), Tygerberg	Susan Engelbrecht, Kayla Delaney, Bronwyn Kleinhans, Houriiyah Tegally, Eduan Wilkindon, Gert van Zyl, Wolfgang Preiser, Tulio de Oliveira
EPI_ISL_745146	Vredendal Hospital wc VRE	National Health Laboratory Service (NHLS), Tygerberg	Susan Engelbrecht, Kayla Delaney, Bronwyn Kleinhans, Houriiyah Tegally, Eduan Wilkindon, Gert van Zyl, Wolfgang Preiser, Tulio de Oliveira
EPI_ISL_745147, EPI_ISL_745148, EPI_ISL_745149, EPI_ISL_745150	Tygerberg Hospital wc TBH	National Health Laboratory Service (NHLS), Tygerberg	Susan Engelbrecht, Kayla Delaney, Bronwyn Kleinhans, Houriiyah Tegally, Eduan Wilkindon, Gert van Zyl, Wolfgang Preiser, Tulio de Oliveira
EPI_ISL_745151	Edameni Clinic	National Health Laboratory Service (NHLS), Tygerberg	Susan Engelbrecht, Kayla Delaney, Bronwyn Kleinhans, Houriiyah Tegally, Eduan Wilkindon, Gert van Zyl, Wolfgang Preiser, Tulio de Oliveira
EPI_ISL_745152	Laetitia Bam CHC	National Health Laboratory Service (NHLS), Tygerberg	Susan Engelbrecht, Kayla Delaney, Bronwyn Kleinhans, Houriiyah Tegally, Eduan Wilkindon, Gert van Zyl, Wolfgang Preiser, Tulio de Oliveira
EPI_ISL_745153	CoVid EC Nelson Mandela Bay Metro	National Health Laboratory Service (NHLS), Tygerberg	Susan Engelbrecht, Kayla Delaney, Bronwyn Kleinhans, Houriiyah Tegally, Eduan Wilkindon, Gert van Zyl, Wolfgang Preiser, Tulio de Oliveira
EPI_ISL_745154	Dr Ivan Toms Clinic wc IVT	National Health Laboratory Service (NHLS), Tygerberg	Susan Engelbrecht, Kayla Delaney, Bronwyn Kleinhans, Houriiyah Tegally, Eduan Wilkindon, Gert van Zyl, Wolfgang Preiser, Tulio de Oliveira
EPI_ISL_745155	Langebaan Clinic wc LBC	National Health Laboratory Service (NHLS), Tygerberg	Susan Engelbrecht, Kayla Delaney, Bronwyn Kleinhans, Houriiyah Tegally, Eduan Wilkindon, Gert van Zyl, Wolfgang Preiser, Tulio de Oliveira
EPI_ISL_745156	SAS Saldanha	National Health Laboratory Service (NHLS), Tygerberg	Susan Engelbrecht, Kayla Delaney, Bronwyn Kleinhans, Houriiyah Tegally, Eduan Wilkindon, Gert van Zyl, Wolfgang Preiser, Tulio de Oliveira
EPI_ISL_745157	SAS Saldanha VPA	National Health Laboratory Service (NHLS), Tygerberg	Susan Engelbrecht, Kayla Delaney, Bronwyn Kleinhans, Houriiyah Tegally, Eduan Wilkindon, Gert van Zyl, Wolfgang Preiser, Tulio de Oliveira
EPI_ISL_745158, EPI_ISL_745159	Bishop Lavis CDC wc BLP	National Health Laboratory Service (NHLS), Tygerberg	Susan Engelbrecht, Kayla Delaney, Bronwyn Kleinhans, Houriiyah Tegally, Eduan Wilkindon, Gert van Zyl, Wolfgang Preiser, Tulio de Oliveira
EPI_ISL_745160	Langebaan Clinic wc LBC	National Health Laboratory Service (NHLS), Tygerberg	Susan Engelbrecht, Kayla Delaney, Bronwyn Kleinhans, Houriiyah Tegally, Eduan Wilkindon, Gert van Zyl, Wolfgang Preiser, Tulio de Oliveira
EPI_ISL_745161	SAS Saldanha VPA	National Health Laboratory Service (NHLS), Tygerberg	Susan Engelbrecht, Kayla Delaney, Bronwyn Kleinhans, Houriiyah Tegally, Eduan Wilkindon, Gert van Zyl, Wolfgang Preiser, Tulio de Oliveira
EPI_ISL_745162	Elsies River CHC wc ERP	National Health Laboratory Service (NHLS), Tygerberg	Susan Engelbrecht, Kayla Delaney, Bronwyn Kleinhans, Houriiyah Tegally, Eduan Wilkindon, Gert van Zyl, Wolfgang Preiser, Tulio de Oliveira
EPI_ISL_745163	Khayelitsha (Site B) CHC wc KHC	National Health Laboratory Service (NHLS), Tygerberg	Susan Engelbrecht, Kayla Delaney, Bronwyn Kleinhans, Houriiyah Tegally, Eduan Wilkindon, Gert van Zyl, Wolfgang Preiser, Tulio de Oliveira
EPI_ISL_745164	Kraaifontein CHC wc KFP	National Health Laboratory Service (NHLS), Tygerberg	Susan Engelbrecht, Kayla Delaney, Bronwyn Kleinhans, Houriiyah Tegally, Eduan Wilkindon, Gert van Zyl, Wolfgang Preiser, Tulio de Oliveira
EPI_ISL_745165	Vredenburg Hospital wc VBG	National Health Laboratory Service (NHLS), Tygerberg	Susan Engelbrecht, Kayla Delaney, Bronwyn Kleinhans, Houriiyah Tegally, Eduan Wilkindon, Gert van Zyl, Wolfgang Preiser, Tulio de Oliveira
EPI_ISL_745166	Khayelitsha Hospital wc KHA	National Health Laboratory Service (NHLS), Tygerberg	Susan Engelbrecht, Kayla Delaney, Bronwyn Kleinhans, Houriiyah Tegally, Eduan Wilkindon, Gert van Zyl, Wolfgang Preiser, Tulio de Oliveira
EPI_ISL_745167	Vredenburg Hospital wc VBG	National Health Laboratory Service (NHLS), Tygerberg	Susan Engelbrecht, Kayla Delaney, Bronwyn Kleinhans, Houriiyah Tegally, Eduan Wilkindon, Gert van Zyl, Wolfgang Preiser, Tulio de Oliveira
EPI_ISL_745168, EPI_ISL_745169	Bishop Lavis CDC wc BLP	National Health Laboratory Service (NHLS), Tygerberg	Susan Engelbrecht, Kayla Delaney, Bronwyn Kleinhans, Houriiyah Tegally, Eduan Wilkindon, Gert van Zyl, Wolfgang Preiser, Tulio de Oliveira
EPI_ISL_745170	Brackengate Field Hospital COVID-19 wc BRG	National Health Laboratory Service (NHLS), Tygerberg	Susan Engelbrecht, Kayla Delaney, Bronwyn Kleinhans, Houriiyah Tegally, Eduan Wilkindon, Gert van Zyl, Wolfgang Preiser, Tulio de Oliveira
EPI_ISL_745171, EPI_ISL_745172	Delft CHC wc DFP	National Health Laboratory Service (NHLS), Tygerberg	Susan Engelbrecht, Kayla Delaney, Bronwyn Kleinhans, Houriiyah Tegally, Eduan Wilkindon, Gert van Zyl, Wolfgang Preiser, Tulio de Oliveira
EPI_ISL_745173, EPI_ISL_745174, EPI_ISL_745175, EPI_ISL_745176, EPI_ISL_745177, EPI_ISL_745178	Eerste River Hospital wc ERH	National Health Laboratory Service (NHLS), Tygerberg	Susan Engelbrecht, Kayla Delaney, Bronwyn Kleinhans, Houriiyah Tegally, Eduan Wilkindon, Gert van Zyl, Wolfgang Preiser, Tulio de Oliveira
EPI_ISL_745179	Kraaifontein CHC wc KFP	National Health Laboratory Service (NHLS), Tygerberg	Susan Engelbrecht, Kayla Delaney, Bronwyn Kleinhans, Houriiyah Tegally, Eduan Wilkindon, Gert van Zyl, Wolfgang Preiser, Tulio de Oliveira
EPI_ISL_745180, EPI_ISL_745181, EPI_ISL_745182, EPI_ISL_745183, EPI_ISL_745184, EPI_ISL_745185	Tygerberg Hospital wc TBH	National Health Laboratory Service (NHLS), Tygerberg	Susan Engelbrecht, Kayla Delaney, Bronwyn Kleinhans, Houriiyah Tegally, Eduan Wilkindon, Gert van Zyl, Wolfgang Preiser, Tulio de Oliveira

EPI_ISL_745186	Wallacedene Clinic wc WAL	National Health Laboratory Service (NHLS), Tygerberg	Susan Engelbrecht, Kayla Delaney, Bronwyn Kleinhans, Houriiyah Tegally, Eduan Wilkindon, Gert van Zyl, Wolfgang Preiser, Tulio de Oliveira
EPI_ISL_745187	Port Nolloth Hospital	National Health Laboratory Service (NHLS), Tygerberg	Susan Engelbrecht, Kayla Delaney, Bronwyn Kleinhans, Houriiyah Tegally, Eduan Wilkindon, Gert van Zyl, Wolfgang Preiser, Tulio de Oliveira
EPI_ISL_745188	Nababeep Hospital	National Health Laboratory Service (NHLS), Tygerberg	Susan Engelbrecht, Kayla Delaney, Bronwyn Kleinhans, Houriiyah Tegally, Eduan Wilkindon, Gert van Zyl, Wolfgang Preiser, Tulio de Oliveira
EPI_ISL_745189	Vredenburg Hospital wc VBG	National Health Laboratory Service (NHLS), Tygerberg	Susan Engelbrecht, Kayla Delaney, Bronwyn Kleinhans, Houriiyah Tegally, Eduan Wilkindon, Gert van Zyl, Wolfgang Preiser, Tulio de Oliveira
EPI_ISL_745190	Brackengate Field Hospital COVID-19 wc BRG	National Health Laboratory Service (NHLS), Tygerberg	Susan Engelbrecht, Kayla Delaney, Bronwyn Kleinhans, Houriiyah Tegally, Eduan Wilkindon, Gert van Zyl, Wolfgang Preiser, Tulio de Oliveira
EPI_ISL_745192	Ospedale Di Venere	Istituto Zooprofilattico Sperimentale della Puglia e della Basilicata	Parisi A., Capozzi L., Del Sambro L., Bianco A., Chiara M., Pesole G., De Sabato L., Iacobellis M.
EPI_ISL_745193	Ospedale Di Venere	Istituto Zooprofilattico Sperimentale della Puglia e della Basilicata	Parisi A., Bianco A., Capozzi L., Del Sambro L., Chiara M., De Sabato L., Pesole G., Iacobellis M
EPI_ISL_745194, EPI_ISL_745196	Osmania Medical College	CSIR-Centre for Cellular and Molecular Biology	Dr.V.Sudha Rani,Dr.S.Pavani,Dr.Satyaprasad,Dr.P.Shashikala Reddy,Namami Gaur,Sakshi Shambhavi,Lamuk Zaveri,Shagufta Khan,Nikhil Hajirnis,M Soujanya Reddy,Pratheusa Maccha,Tulasi Nagabandi,Purushotham Vodnala,Blessy B John,Viswagithe S L,B Himasri,Onkar Kulkarni,Payel Mukherjee,Sofia Banu,Priya Singh,Archana Bharadwaj Siva,Karthik Bharadwaj Tallapaka,Rakesh K Mishra,Divya Tej Sowpati
EPI_ISL_745197	Kakatiya Medical College	CSIR-Centre for Cellular and Molecular Biology	Dr.V.Sudha Rani,Dr.S.Pavani,Dr.Satyaprasad,Dr.P.Shashikala Reddy,Namami Gaur,Sakshi Shambhavi,Lamuk Zaveri,Shagufta Khan,Nikhil Hajirnis,M Soujanya Reddy,Pratheusa Maccha,Tulasi Nagabandi,Purushotham Vodnala,Blessy B John,Viswagithe S L,B Himasri,Onkar Kulkarni,Payel Mukherjee,Sofia Banu,Priya Singh,Archana Bharadwaj Siva,Karthik Bharadwaj Tallapaka,Rakesh K Mishra,Divya Tej Sowpati
EPI_ISL_745198	ESI Hospital	CSIR-Centre for Cellular and Molecular Biology	Dr.V.Sudha Rani,Dr.S.Pavani,Dr.Satyaprasad,Dr.P.Shashikala Reddy,Namami Gaur,Sakshi Shambhavi,Lamuk Zaveri,Shagufta Khan,Nikhil Hajirnis,M Soujanya Reddy,Pratheusa Maccha,Tulasi Nagabandi,Purushotham Vodnala,Blessy B John,Viswagithe S L,B Himasri,Onkar Kulkarni,Payel Mukherjee,Sofia Banu,Priya Singh,Archana Bharadwaj Siva,Karthik Bharadwaj Tallapaka,Rakesh K Mishra,Divya Tej Sowpati
EPI_ISL_745199	Rangaraya Medical College	CSIR-Centre for Cellular and Molecular Biology	Dr.V.Sudha Rani,Dr.S.Pavani,Dr.Satyaprasad,Dr.P.Shashikala Reddy,Namami Gaur,Sakshi Shambhavi,Lamuk Zaveri,Shagufta Khan,Nikhil Hajirnis,M Soujanya Reddy,Pratheusa Maccha,Tulasi Nagabandi,Purushotham Vodnala,Blessy B John,Viswagithe S L,B Himasri,Onkar Kulkarni,Payel Mukherjee,Sofia Banu,Priya Singh,Archana Bharadwaj Siva,Karthik Bharadwaj Tallapaka,Rakesh K Mishra,Divya Tej Sowpati
EPI_ISL_745200	Nizams Institute of Medical Sciences	CSIR-Centre for Cellular and Molecular Biology	Dr.V.Sudha Rani,Dr.S.Pavani,Dr.Satyaprasad,Dr.P.Shashikala Reddy,Namami Gaur,Sakshi Shambhavi,Lamuk Zaveri,Shagufta Khan,Nikhil Hajirnis,M Soujanya Reddy,Pratheusa Maccha,Tulasi Nagabandi,Purushotham Vodnala,Blessy B John,Viswagithe S L,B Himasri,Onkar Kulkarni,Payel Mukherjee,Sofia Banu,Priya Singh,Archana Bharadwaj Siva,Karthik Bharadwaj Tallapaka,Rakesh K Mishra,Divya Tej Sowpati
EPI_ISL_745201	Guntur General Hospital	CSIR-Centre for Cellular and Molecular Biology	Dr.V.Sudha Rani,Dr.S.Pavani,Dr.Satyaprasad,Dr.P.Shashikala Reddy,Namami Gaur,Sakshi Shambhavi,Lamuk Zaveri,Shagufta Khan,Nikhil Hajirnis,M Soujanya Reddy,Pratheusa Maccha,Tulasi Nagabandi,Purushotham Vodnala,Blessy B John,Viswagithe S L,B Himasri,Onkar Kulkarni,Payel Mukherjee,Sofia Banu,Priya Singh,Archana Bharadwaj Siva,Karthik Bharadwaj Tallapaka,Rakesh K Mishra,Divya Tej Sowpati
EPI_ISL_745202, EPI_ISL_745203, EPI_ISL_745204, EPI_ISL_745205, EPI_ISL_745206, EPI_ISL_745207, EPI_ISL_745209, EPI_ISL_745210, EPI_ISL_745211	Lab voor klinische biologie	Onderzoeksgroep Virologie	Laurens Lambrechts, Nick Vereecke, Marthe Pauwels, Bruno Verhasselt, Linos Vandekerckhove, Hans Nauwynck, Sebastiaan Theuns
EPI_ISL_745212, EPI_ISL_745214, EPI_ISL_745215, EPI_ISL_745216, EPI_ISL_745217, EPI_ISL_745218, EPI_ISL_745219, EPI_ISL_745220	Lab voor klinische biologie	Onderzoeksgroep Virologie	Nick Vereecke, Laurens Lambrechts, Marthe Pauwels, Bruno Verhasselt, Linos Vandekerckhove, Hans Nauwynck, Sebastiaan Theuns
EPI_ISL_745222	UZ Leuven, National Reference Laboratory for Coronaviruses, Laboratory Medicine, Leuven, Belgium	KU Leuven, Rega Institute, Clinical and Epidemiological Virology	Tony Wawina-Bokalanga, Joan Marti-Carerras, Bert Vanmechelen, Piet Maes
EPI_ISL_745223, EPI_ISL_745224, EPI_ISL_745225, EPI_ISL_745226, EPI_ISL_745227, EPI_ISL_745228, EPI_ISL_745229, EPI_ISL_745230, EPI_ISL_745231, EPI_ISL_745232, EPI_ISL_745233, EPI_ISL_745234, EPI_ISL_745235, EPI_ISL_745236, EPI_ISL_745237, EPI_ISL_745238, EPI_ISL_745239, EPI_ISL_745240, EPI_ISL_745241, EPI_ISL_745242, EPI_ISL_745243, EPI_ISL_745244, EPI_ISL_745245, EPI_ISL_745246, EPI_ISL_745247, EPI_ISL_745248, EPI_ISL_745249, EPI_ISL_745250, EPI_ISL_745251, EPI_ISL_745252, EPI_ISL_745253, EPI_ISL_745254, EPI_ISL_745255, EPI_ISL_745256, EPI_ISL_745257, EPI_ISL_745258, EPI_ISL_745259			
see above	Molecular diagnostic laboratory of Federal Budget Institution of Science "Central Research Institute of Epidemiology" of The Federal Service on Customers' Rights Protection and Human Well-being Surveillance	Group of Genomics and Postgenomic Technologies of Central Research Institute of Epidemilology	Samoilov AE, Kaptelova VV, Korneenko EV, Dudorova AV, Saenko SS, Speranskaya AS, Tivanova EV, Shipulina OY, Akimkin VG
EPI_ISL_745260	CHUM-Site Glen-LAB Microbiologie	Laboratoire de santé publique du Québec	Sandrine Moreira, Ioannis Ragoussis, Guillaume Bourque, Jesse Shapiro, Mark Lathrop and Michel Roger on behalf of the CoVSeQ research group (http://covseq.ca/researchgroup)
EPI_ISL_745261, EPI_ISL_745262, EPI_ISL_745263, EPI_ISL_745264, EPI_ISL_745265, EPI_ISL_745266, EPI_ISL_745267, EPI_ISL_745268, EPI_ISL_745269, EPI_ISL_745270, EPI_ISL_745271, EPI_ISL_745272, EPI_ISL_745273, EPI_ISL_745274, EPI_ISL_745276, EPI_ISL_745277, EPI_ISL_745278, EPI_ISL_745279, EPI_ISL_745280, EPI_ISL_745281, EPI_ISL_745282, EPI_ISL_745283, EPI_ISL_745284, EPI_ISL_745285, EPI_ISL_745286, EPI_ISL_745287, EPI_ISL_745288, EPI_ISL_745289, EPI_ISL_745290, EPI_ISL_745291, EPI_ISL_745292, EPI_ISL_745293, EPI_ISL_745294, EPI_ISL_745295, EPI_ISL_745296, EPI_ISL_745297, EPI_ISL_745298, EPI_ISL_745299, EPI_ISL_745300, EPI_ISL_745301, EPI_ISL_745302, EPI_ISL_745303, EPI_ISL_745304, EPI_ISL_745305, EPI_ISL_745306			
see above	Texas Department of State Health Services	Texas Department of State Health Services	Rashmi Tuladhar, Bonnie Oh, Jenny Zhang, Maliha Rahman, Anita Pokharel, Myong Koag, Chung Wang, Rachel Lee, Grace Kubin, Mayela Pedrueza, James Daniel Bonser
EPI_ISL_745307, EPI_ISL_745308, EPI_ISL_745309, EPI_ISL_745310, EPI_ISL_745311	CNR Virus des Infections Respiratoires - France SUD	CNR Virus des Infections Respiratoires - France SUD	Antonin Bal, Gregory Destras, Claudia Gonzalez, Gwendolyne Burfin, Quentin Semanas, Martine Valette, Bruno Lina, Laurence Josset
EPI_ISL_745312, EPI_ISL_745313, EPI_ISL_745314, EPI_ISL_745315	CHU Clermont-Ferrand	CNR Virus des Infections Respiratoires - France SUD	Antonin Bal, Gregory Destras, Gwendolyne Burfin, Hadrien Règue, Quentin Semanas, Martine Valette, Bruno Lina, Christine Archimbaud, Amélie Brebion, Hélène Chabrolles, Martine Chambon, Audrey Mirand, Christel Regagnon, Maxime Bisseux, Patricia Combes, Cécile Henquell, Laurence Josset
EPI_ISL_745316, EPI_ISL_745317, EPI_ISL_745318, EPI_ISL_745319, EPI_ISL_745320, EPI_ISL_745321, EPI_ISL_745322, EPI_ISL_745323, EPI_ISL_745324, EPI_ISL_745325, EPI_ISL_745326, EPI_ISL_745327, EPI_ISL_745328, EPI_ISL_745329, EPI_ISL_745330, EPI_ISL_745331, EPI_ISL_745332, EPI_ISL_745333, EPI_ISL_745334			
see above	CNR Virus des Infections Respiratoires - France SUD	CNR Virus des Infections Respiratoires - France SUD	Antonin Bal, Gregory Destras, Claudia Gonzalez, Gwendolyne Burfin, Quentin Semanas, Martine Valette, Bruno Lina, Laurence Josset
EPI_ISL_745335, EPI_ISL_745336	CHU Clermont-Ferrand	CNR Virus des Infections Respiratoires - France SUD	Antonin Bal, Gregory Destras, Gwendolyne Burfin, Hadrien Règue, Quentin Semanas, Martine Valette, Bruno Lina, Christine Archimbaud, Amélie Brebion, Hélène Chabrolles, Martine Chambon, Audrey Mirand, Christel Regagnon, Maxime Bisseux, Patricia Combes, Cécile Henquell, Laurence Josset
EPI_ISL_745337, EPI_ISL_745338, EPI_ISL_745339, EPI_ISL_745340, EPI_ISL_745341, EPI_ISL_745342, EPI_ISL_745343, EPI_ISL_745344, EPI_ISL_745345, EPI_ISL_745346, EPI_ISL_745347, EPI_ISL_745348, EPI_ISL_745349, EPI_ISL_745350, EPI_ISL_745351, EPI_ISL_745352, EPI_ISL_745353, EPI_ISL_745354, EPI_ISL_745355, EPI_ISL_745356, EPI_ISL_745357, EPI_ISL_745358, EPI_ISL_745359, EPI_ISL_745360, EPI_ISL_745361, EPI_ISL_745362, EPI_ISL_745363, EPI_ISL_745364, EPI_ISL_745365, EPI_ISL_745366, EPI_ISL_745367, EPI_ISL_745368, EPI_ISL_745369, EPI_ISL_745370, EPI_ISL_745371, EPI_ISL_745372, EPI_ISL_745373, EPI_ISL_745374, EPI_ISL_745375, EPI_ISL_745376, EPI_ISL_745377, EPI_ISL_745378, EPI_ISL_745379, EPI_ISL_745380, EPI_ISL_745381, EPI_ISL_745382, EPI_ISL_745383, EPI_ISL_745384, EPI_ISL_745385, EPI_ISL_745386, EPI_ISL_745387, EPI_ISL_745388, EPI_ISL_745389, EPI_ISL_745390, EPI_ISL_745391, EPI_ISL_745392, EPI_ISL_745393, EPI_ISL_745394, EPI_ISL_745395, EPI_ISL_745396, EPI_ISL_745397			
see above	CNR Virus des Infections Respiratoires - France SUD	CNR Virus des Infections Respiratoires - France SUD	Antonin Bal, Gregory Destras, Claudia Gonzalez, Gwendolyne Burfin, Quentin Semanas, Martine Valette, Bruno Lina, Laurence Josset
EPI_ISL_745398	DOHMH Jamaica	New York City Public Health Laboratory	Jade Wang, et al.
EPI_ISL_745399	DOHMH Morrisania	New York City Public Health Laboratory	Jade Wang, et al.
EPI_ISL_745400	DOHMH Central Harlem	New York City Public Health Laboratory	Jade Wang, et al.
EPI_ISL_745401	DOHMH Corona	New York City Public Health Laboratory	Jade Wang, et al.

EPI_ISL_745402	DOHMH Jamaica	New York City Public Health Laboratory	Jade Wang, et al.
EPI_ISL_745403	DOHMH Corona	New York City Public Health Laboratory	Jade Wang, et al.
EPI_ISL_745404	DOHMH Central Harlem	New York City Public Health Laboratory	Jade Wang, et al.
EPI_ISL_745405	DOHMH Jamaica	New York City Public Health Laboratory	Jade Wang, et al.
EPI_ISL_745406	DOHMH Morrisania	New York City Public Health Laboratory	Jade Wang, et al.
EPI_ISL_745407	DOHMH Jamaica	New York City Public Health Laboratory	Jade Wang, et al.
EPI_ISL_745408	DOHMH Riverside	New York City Public Health Laboratory	Jade Wang, et al.
EPI_ISL_745409	DOHMH Corona	New York City Public Health Laboratory	Jade Wang, et al.
EPI_ISL_745410	DOHMH Chelsea	New York City Public Health Laboratory	Jade Wang, et al.
EPI_ISL_745411	DOHMH Corona	New York City Public Health Laboratory	Jade Wang, et al.
EPI_ISL_745412	DOHMH Morrisania	New York City Public Health Laboratory	Jade Wang, et al.
EPI_ISL_745413	DOHMH Central Harlem	New York City Public Health Laboratory	Jade Wang, et al.
EPI_ISL_745414, EPI_ISL_745415, EPI_ISL_745416, EPI_ISL_745417, EPI_ISL_745418	DOHMH Jamaica	New York City Public Health Laboratory	Jade Wang, et al.
EPI_ISL_745419, EPI_ISL_745420	DOHMH Crown Heights	New York City Public Health Laboratory	Jade Wang, et al.
EPI_ISL_745421, EPI_ISL_745422	DOHMH Chelsea	New York City Public Health Laboratory	Jade Wang, et al.
EPI_ISL_745423, EPI_ISL_745424	DOHMH Corona	New York City Public Health Laboratory	Jade Wang, et al.
EPI_ISL_745425	DOHMH Central Harlem	New York City Public Health Laboratory	Jade Wang, et al.
EPI_ISL_745426, EPI_ISL_745427	DOHMH Corona	New York City Public Health Laboratory	Jade Wang, et al.
EPI_ISL_745428, EPI_ISL_745429	DOHMH Riverside	New York City Public Health Laboratory	Jade Wang, et al.
EPI_ISL_745430, EPI_ISL_745431, EPI_ISL_745432, EPI_ISL_745433	DOHMH Morrisania	New York City Public Health Laboratory	Jade Wang, et al.
EPI_ISL_745434, EPI_ISL_745435	DOHMH Corona	New York City Public Health Laboratory	Jade Wang, et al.
EPI_ISL_745436	DOHMH Chelsea	New York City Public Health Laboratory	Jade Wang, et al.
EPI_ISL_745437, EPI_ISL_745438	DOHMH PHL	New York City Public Health Laboratory	Jade Wang, et al.
EPI_ISL_745439	DOHMH Riverside	New York City Public Health Laboratory	Jade Wang, et al.
EPI_ISL_745440	DOHMH Jamaica	New York City Public Health Laboratory	Jade Wang, et al.
EPI_ISL_745441, EPI_ISL_745442, EPI_ISL_745443	DOHMH Morrisania	New York City Public Health Laboratory	Jade Wang, et al.
EPI_ISL_745444	DOHMH Jamaica	New York City Public Health Laboratory	Jade Wang, et al.
EPI_ISL_745445	DOHMH Crown Heights	New York City Public Health Laboratory	Jade Wang, et al.
EPI_ISL_745446, EPI_ISL_745447	DOHMH Corona	New York City Public Health Laboratory	Jade Wang, et al.
EPI_ISL_745448	DOHMH Crown Heights	New York City Public Health Laboratory	Jade Wang, et al.
EPI_ISL_745449, EPI_ISL_745450	DOHMH Morrisania	New York City Public Health Laboratory	Jade Wang, et al.
EPI_ISL_745451	DOHMH Central Harlem	New York City Public Health Laboratory	Jade Wang, et al.
EPI_ISL_745452	DOHMH Crown Heights	New York City Public Health Laboratory	Jade Wang, et al.
EPI_ISL_745453	DOHMH Chelsea	New York City Public Health Laboratory	Jade Wang, et al.
EPI_ISL_745454	DOHMH Crown Heights	New York City Public Health Laboratory	Jade Wang, et al.
EPI_ISL_745455	DOHMH Corona	New York City Public Health Laboratory	Jade Wang, et al.
EPI_ISL_745456	DOHMH PHL	New York City Public Health Laboratory	Jade Wang, et al.
EPI_ISL_745457	DOHMH Chelsea	New York City Public Health Laboratory	Jade Wang, et al.
EPI_ISL_745458, EPI_ISL_745459, EPI_ISL_745460, EPI_ISL_745461, EPI_ISL_745462	DOHMH Corona	New York City Public Health Laboratory	Jade Wang, et al.
EPI_ISL_745463	DOHMH Fort Greene	New York City Public Health Laboratory	Jade Wang, et al.
EPI_ISL_745464	DOHMH Corona	New York City Public Health Laboratory	Jade Wang, et al.
EPI_ISL_745465	DOHMH PHL	New York City Public Health Laboratory	Jade Wang, et al.
EPI_ISL_745466, EPI_ISL_745467	DOHMH Corona	New York City Public Health Laboratory	Jade Wang, et al.
EPI_ISL_745468	DOHMH Riverside	New York City Public Health Laboratory	Jade Wang, et al.
EPI_ISL_745469	DOHMH Central Harlem	New York City Public Health Laboratory	Jade Wang, et al.
EPI_ISL_745470	DOHMH Morrisania	New York City Public Health Laboratory	Jade Wang, et al.
EPI_ISL_745471, EPI_ISL_745472, EPI_ISL_745473, EPI_ISL_745474, EPI_ISL_745475, EPI_ISL_745476, EPI_ISL_745477, EPI_ISL_745478, EPI_ISL_745479, EPI_ISL_745480, EPI_ISL_745481, EPI_ISL_745482, EPI_ISL_745483, EPI_ISL_745484, EPI_ISL_745485, EPI_ISL_745486, EPI_ISL_745487, EPI_ISL_745488, EPI_ISL_745489, EPI_ISL_745490, EPI_ISL_745491, EPI_ISL_745492, EPI_ISL_745493, EPI_ISL_745494, EPI_ISL_745495, EPI_ISL_745496, EPI_ISL_745497, EPI_ISL_745498, EPI_ISL_745499, EPI_ISL_745500, EPI_ISL_745501, EPI_ISL_745502, EPI_ISL_745503, EPI_ISL_745504, EPI_ISL_745505, EPI_ISL_745506, EPI_ISL_745507, EPI_ISL_745508, EPI_ISL_745509, EPI_ISL_745510, EPI_ISL_745511, EPI_ISL_745512, EPI_ISL_745513, EPI_ISL_745514, EPI_ISL_745515, EPI_ISL_745516, EPI_ISL_745517, EPI_ISL_745518, EPI_ISL_745519, EPI_ISL_745520, EPI_ISL_745521, EPI_ISL_745522, EPI_ISL_745523, EPI_ISL_745524, EPI_ISL_745525, EPI_ISL_745526, EPI_ISL_745527, EPI_ISL_745528, EPI_ISL_745529, EPI_ISL_745530, EPI_ISL_745531, EPI_ISL_745532, EPI_ISL_745533, EPI_ISL_745534, EPI_ISL_745535, EPI_ISL_745536, EPI_ISL_745537, EPI_ISL_745538, EPI_ISL_745539, EPI_ISL_745540, EPI_ISL_745541, EPI_ISL_745542, EPI_ISL_745543, EPI_ISL_745544, EPI_ISL_745545, EPI_ISL_745546, EPI_ISL_745547, EPI_ISL_745548, EPI_ISL_745549, EPI_ISL_745550, EPI_ISL_745551, EPI_ISL_745552, EPI_ISL_745553, EPI_ISL_745554, EPI_ISL_745555, EPI_ISL_745556, EPI_ISL_745557, EPI_ISL_745558, EPI_ISL_745559, EPI_ISL_745560, EPI_ISL_745561, EPI_ISL_745562, EPI_ISL_745563, EPI_ISL_745564, EPI_ISL_745565, EPI_ISL_745566, EPI_ISL_745567, EPI_ISL_745568, EPI_ISL_745569, EPI_ISL_745570, EPI_ISL_745571, EPI_ISL_745572, EPI_ISL_745573, EPI_ISL_745574, EPI_ISL_745575, EPI_ISL_745576, EPI_ISL_745577, EPI_ISL_745578, EPI_ISL_745579, EPI_ISL_745580, EPI_ISL_745581, EPI_ISL_745582, EPI_ISL_745583, EPI_ISL_745584, EPI_ISL_745585, EPI_ISL_745586, EPI_ISL_745587, EPI_ISL_745588, EPI_ISL_745589, EPI_ISL_745590, EPI_ISL_745591, EPI_ISL_745592, EPI_ISL_745593, EPI_ISL_745594, EPI_ISL_745595, EPI_ISL_745596, EPI_ISL_745597, EPI_ISL_745598, EPI_ISL_745659, EPI_ISL_745660, EPI_ISL_745661, EPI_ISL_745662, EPI_ISL_745663, EPI_ISL_745664, EPI_ISL_745665, EPI_ISL_745666, EPI_ISL_745667, EPI_ISL_745668, EPI_ISL_745669, EPI_ISL_745670, EPI_ISL_745671, EPI_ISL_745672, EPI_ISL_745673, EPI_ISL_745674, EPI_ISL_745675, EPI_ISL_745676, EPI_ISL_745677, EPI_ISL_745678, EPI_ISL_745679, EPI_ISL_745680, EPI_ISL_745681, EPI_ISL_745682, EPI_ISL_745683, EPI_ISL_745684, EPI_ISL_745685, EPI_ISL_745686, EPI_ISL_745687, EPI_ISL_745688, EPI_ISL_745689, EPI_ISL_745690, EPI_ISL_745691, EPI_ISL_745692, EPI_ISL_745693, EPI_ISL_745694, EPI_ISL_745695, EPI_ISL_745696, EPI_ISL_745697, EPI_ISL_745698, EPI_ISL_745699, EPI_ISL_745700, EPI_ISL_745701, EPI_ISL_745702, EPI_ISL_745703, EPI_ISL_745704, EPI_ISL_745705, EPI_ISL_745706, EPI_ISL_745707, EPI_ISL_745708, EPI_ISL_745709, EPI_ISL_745710, EPI_ISL_745711, EPI_ISL_745712, EPI_ISL_745713, EPI_ISL_745714, EPI_ISL_745715, EPI_ISL_745716, EPI_ISL_745717, EPI_ISL_745718, EPI_ISL_745719, EPI_ISL_745720, EPI_ISL_745721, EPI_ISL_745722, EPI_ISL_745723, EPI_ISL_745724, EPI_ISL_745725, EPI_ISL_745726, EPI_ISL_745727, EPI_ISL_745728, EPI_ISL_745729, EPI_ISL_745730, EPI_ISL_745731, EPI_ISL_745732, EPI_ISL_745733, EPI_ISL_745734, EPI_ISL_745735, EPI_ISL_745736, EPI_ISL_745737, EPI_ISL_745738, EPI_ISL_745739, EPI_ISL_745740, EPI_ISL_745741, EPI_ISL_745742, EPI_ISL_745743, EPI_ISL_745744, EPI_ISL_745745, EPI_ISL_745746, EPI_ISL_745747, EPI_ISL_745748, EPI_ISL_745749, EPI_ISL_745750, EPI_ISL_745751, EPI_ISL_745752, EPI_ISL_745753, EPI_ISL_745754, EPI_ISL_745755, EPI_ISL_745756, EPI_ISL_745757, EPI_ISL_745758, EPI_ISL_745759, EPI_ISL_745760, EPI_ISL_745761, EPI_ISL_745762, EPI_ISL_745763, EPI_ISL_745764,			

EPI_ISL_745765, EPI_ISL_745766, EPI_ISL_745767, EPI_ISL_745768, EPI_ISL_745769, EPI_ISL_745770, EPI_ISL_745771, EPI_ISL_745772, EPI_ISL_745773, EPI_ISL_745774, EPI_ISL_745775, EPI_ISL_745776, EPI_ISL_745777, EPI_ISL_745778, EPI_ISL_745779, EPI_ISL_745780, EPI_ISL_745781, EPI_ISL_745782, EPI_ISL_745783, EPI_ISL_745784, EPI_ISL_745785, EPI_ISL_745786, EPI_ISL_745787, EPI_ISL_745788, EPI_ISL_745789, EPI_ISL_745790, EPI_ISL_745791, EPI_ISL_745792, EPI_ISL_745793, EPI_ISL_745794, EPI_ISL_745795, EPI_ISL_745796, EPI_ISL_745797, EPI_ISL_745798, EPI_ISL_745799, EPI_ISL_745800, EPI_ISL_745801, EPI_ISL_745802, EPI_ISL_745803, EPI_ISL_745805, EPI_ISL_745806, EPI_ISL_745807, EPI_ISL_745808, EPI_ISL_745809, EPI_ISL_745810, EPI_ISL_745811, EPI_ISL_745812, EPI_ISL_745813, EPI_ISL_745814, EPI_ISL_745815, EPI_ISL_745816, EPI_ISL_745817, EPI_ISL_745818, EPI_ISL_745819, EPI_ISL_745820, EPI_ISL_745821, EPI_ISL_745822, EPI_ISL_745823, EPI_ISL_745824, EPI_ISL_745825, EPI_ISL_745826, EPI_ISL_745827, EPI_ISL_745828, EPI_ISL_745829, EPI_ISL_745830, EPI_ISL_745831, EPI_ISL_745832, EPI_ISL_745833, EPI_ISL_745834, EPI_ISL_745835, EPI_ISL_745836, EPI_ISL_745837, EPI_ISL_745838, EPI_ISL_745839, EPI_ISL_745840, EPI_ISL_745841, EPI_ISL_745842, EPI_ISL_745843, EPI_ISL_745844, EPI_ISL_745845, EPI_ISL_745846, EPI_ISL_745847, EPI_ISL_745848, EPI_ISL_745849, EPI_ISL_745850, EPI_ISL_745851, EPI_ISL_745852, EPI_ISL_745853, EPI_ISL_745854, EPI_ISL_745855, EPI_ISL_745856, EPI_ISL_745857, EPI_ISL_745858, EPI_ISL_745859, EPI_ISL_745860, EPI_ISL_745861, EPI_ISL_745862, EPI_ISL_745863, EPI_ISL_745864, EPI_ISL_745865, EPI_ISL_745866, EPI_ISL_745867, EPI_ISL_745868, EPI_ISL_745869, EPI_ISL_745870, EPI_ISL_745871, EPI_ISL_745872, EPI_ISL_745873, EPI_ISL_745874, EPI_ISL_745875, EPI_ISL_745876, EPI_ISL_745877, EPI_ISL_745878, EPI_ISL_745879, EPI_ISL_745880, EPI_ISL_745881, EPI_ISL_745882, EPI_ISL_745883, EPI_ISL_745884, EPI_ISL_745885, EPI_ISL_745886, EPI_ISL_745887, EPI_ISL_745888, EPI_ISL_745889, EPI_ISL_745890, EPI_ISL_745891, EPI_ISL_745892, EPI_ISL_745893, EPI_ISL_745894, EPI_ISL_745895, EPI_ISL_745896, EPI_ISL_745897, EPI_ISL_745898, EPI_ISL_745899, EPI_ISL_745900, EPI_ISL_745901, EPI_ISL_745902, EPI_ISL_745903, EPI_ISL_745904, EPI_ISL_745905, EPI_ISL_745906, EPI_ISL_745907, EPI_ISL_745908, EPI_ISL_745909, EPI_ISL_745910, EPI_ISL_745911, EPI_ISL_745912, EPI_ISL_745913, EPI_ISL_745914, EPI_ISL_745915, EPI_ISL_745916, EPI_ISL_745917, EPI_ISL_745918, EPI_ISL_745919, EPI_ISL_745920, EPI_ISL_745921, EPI_ISL_745922, EPI_ISL_745923, EPI_ISL_745924, EPI_ISL_745925, EPI_ISL_745926, EPI_ISL_745927, EPI_ISL_745928, EPI_ISL_745929, EPI_ISL_745930, EPI_ISL_745931, EPI_ISL_745932, EPI_ISL_745933, EPI_ISL_745934, EPI_ISL_745935, EPI_ISL_745936, EPI_ISL_745937, EPI_ISL_745938, EPI_ISL_745939, EPI_ISL_745940, EPI_ISL_745941, EPI_ISL_745942, EPI_ISL_745943, EPI_ISL_745944, EPI_ISL_745945, EPI_ISL_745946, EPI_ISL_745947, EPI_ISL_745948, EPI_ISL_745949, EPI_ISL_745950, EPI_ISL_745951, EPI_ISL_745952, EPI_ISL_745953, EPI_ISL_745954, EPI_ISL_745955, EPI_ISL_745956, EPI_ISL_745957, EPI_ISL_745958, EPI_ISL_745959, EPI_ISL_745960, EPI_ISL_745961, EPI_ISL_745962, EPI_ISL_745963, EPI_ISL_745964, EPI_ISL_745965, EPI_ISL_745966, EPI_ISL_745967, EPI_ISL_745968, EPI_ISL_745969, EPI_ISL_745970, EPI_ISL_745971, EPI_ISL_745972, EPI_ISL_745973, EPI_ISL_745974, EPI_ISL_745975, EPI_ISL_745976, EPI_ISL_745977, EPI_ISL_745978, EPI_ISL_745979, EPI_ISL_745980, EPI_ISL_745981, EPI_ISL_745982, EPI_ISL_745983, EPI_ISL_745984, EPI_ISL_745985, EPI_ISL_745986, EPI_ISL_745987, EPI_ISL_745988, EPI_ISL_745989, EPI_ISL_745990, EPI_ISL_745991, EPI_ISL_745992, EPI_ISL_745993, EPI_ISL_745994, EPI_ISL_745995, EPI_ISL_745996, EPI_ISL_745997, EPI_ISL_745998, EPI_ISL_745999, EPI_ISL_746000, EPI_ISL_746001, EPI_ISL_746002, EPI_ISL_746003, EPI_ISL_746004, EPI_ISL_746005, EPI_ISL_746006, EPI_ISL_746007, EPI_ISL_746008, EPI_ISL_746009, EPI_ISL_746010, EPI_ISL_746011, EPI_ISL_746012, EPI_ISL_746013, EPI_ISL_746014, EPI_ISL_746015, EPI_ISL_746016, EPI_ISL_746017, EPI_ISL_746018, EPI_ISL_746019, EPI_ISL_746020, EPI_ISL_746021, EPI_ISL_746022, EPI_ISL_746023, EPI_ISL_746024, EPI_ISL_746025, EPI_ISL_746026, EPI_ISL_746027, EPI_ISL_746028, EPI_ISL_746029, EPI_ISL_746030, EPI_ISL_746031, EPI_ISL_746032, EPI_ISL_746033, EPI_ISL_746034, EPI_ISL_746035, EPI_ISL_746036, EPI_ISL_746037, EPI_ISL_746038, EPI_ISL_746039, EPI_ISL_746040, EPI_ISL_746041, EPI_ISL_746042, EPI_ISL_746043, EPI_ISL_746044, EPI_ISL_746045, EPI_ISL_746046, EPI_ISL_746047, EPI_ISL_746048, EPI_ISL_746049, EPI_ISL_746050, EPI_ISL_746051, EPI_ISL_746052, EPI_ISL_746053, EPI_ISL_746054, EPI_ISL_746055, EPI_ISL_746056, EPI_ISL_746057, EPI_ISL_746058, EPI_ISL_746059, EPI_ISL_746060, EPI_ISL_746061, EPI_ISL_746062, EPI_ISL_746063, EPI_ISL_746064, EPI_ISL_746065, EPI_ISL_746066, EPI_ISL_746067, EPI_ISL_746068, EPI_ISL_746069, EPI_ISL_746070, EPI_ISL_746071, EPI_ISL_746072, EPI_ISL_746073, EPI_ISL_746074, EPI_ISL_746075, EPI_ISL_746076, EPI_ISL_746077, EPI_ISL_746078, EPI_ISL_746079, EPI_ISL_746080, EPI_ISL_746081, EPI_ISL_746082, EPI_ISL_746083, EPI_ISL_746084, EPI_ISL_746085, EPI_ISL_746086, EPI_ISL_746087, EPI_ISL_746088, EPI_ISL_746089, EPI_ISL_746090, EPI_ISL_746091, EPI_ISL_746092, EPI_ISL_746093, EPI_ISL_746094, EPI_ISL_746095, EPI_ISL_746096, EPI_ISL_746097, EPI_ISL_746098, EPI_ISL_746099, EPI_ISL_746100, EPI_ISL_746101, EPI_ISL_746102, EPI_ISL_746103, EPI_ISL_746104, EPI_ISL_746105, EPI_ISL_746106, EPI_ISL_746107, EPI_ISL_746108, EPI_ISL_746109, EPI_ISL_746110, EPI_ISL_746111, EPI_ISL_746112, EPI_ISL_746113, EPI_ISL_746114, EPI_ISL_746115, EPI_ISL_746116, EPI_ISL_746117, EPI_ISL_746118, EPI_ISL_746119, EPI_ISL_746120, EPI_ISL_746121, EPI_ISL_746122, EPI_ISL_746123, EPI_ISL_746124, EPI_ISL_746125, EPI_ISL_746126, EPI_ISL_746127, EPI_ISL_746128, EPI_ISL_746129, EPI_ISL_746130, EPI_ISL_746131, EPI_ISL_746132, EPI_ISL_746133, EPI_ISL_746134, EPI_ISL_746135, EPI_ISL_746136, EPI_ISL_746137, EPI_ISL_746138, EPI_ISL_746139, EPI_ISL_746140, EPI_ISL_746141, EPI_ISL_746142, EPI_ISL_746143, EPI_ISL_746144, EPI_ISL_746145, EPI_ISL_746146, EPI_ISL_746147, EPI_ISL_746148, EPI_ISL_746149, EPI_ISL_746150, EPI_ISL_746151, EPI_ISL_746152, EPI_ISL_746153, EPI_ISL_746154, EPI_ISL_746155, EPI_ISL_746156, EPI_ISL_746157, EPI_ISL_746158, EPI_ISL_746159, EPI_ISL_746160, EPI_ISL_746161, EPI_ISL_746162, EPI_ISL_746163, EPI_ISL_746164, EPI_ISL_746165, EPI_ISL_746166, EPI_ISL_746167, EPI_ISL_746168, EPI_ISL_746169, EPI_ISL_746170, EPI_ISL_746171, EPI_ISL_746172, EPI_ISL_746173, EPI_ISL_746174, EPI_ISL_746175, EPI_ISL_746176, EPI_ISL_746177, EPI_ISL_746178, EPI_ISL_746179, EPI_ISL_746180, EPI_ISL_746181, EPI_ISL_746182, EPI_ISL_746183, EPI_ISL_746184, EPI_ISL_746185, EPI_ISL_746186, EPI_ISL_746187, EPI_ISL_746188, EPI_ISL_746189, EPI_ISL_746190, EPI_ISL_746191, EPI_ISL_746192, EPI_ISL_746193, EPI_ISL_746194, EPI_ISL_746195, EPI_ISL_746196, EPI_ISL_746197, EPI_ISL_746198, EPI_ISL_746199, EPI_ISL_746200, EPI_ISL_746201, EPI_ISL_746202, EPI_ISL_746203, EPI_ISL_746204, EPI_ISL_746205, EPI_ISL_746206, EPI_ISL_746207, EPI_ISL_746208, EPI_ISL_746209, EPI_ISL_746210, EPI_ISL_746211, EPI_ISL_746212, EPI_ISL_746213, EPI_ISL_746214, EPI_ISL_746215, EPI_ISL_746216, EPI_ISL_746217, EPI_ISL_746218, EPI_ISL_746219, EPI_ISL_746220, EPI_ISL_746221, EPI_ISL_746222, EPI_ISL_746223, EPI_ISL_746224, EPI_ISL_746225, EPI_ISL_746226, EPI_ISL_746227, EPI_ISL_746228, EPI_ISL_746229, EPI_ISL_746230, EPI_ISL_746231, EPI_ISL_746232, EPI_ISL_746233, EPI_ISL_746234, EPI_ISL_746235, EPI_ISL_746236, EPI_ISL_746237, EPI_ISL_746238, EPI_ISL_746239, EPI_ISL_746240, EPI_ISL_746241, EPI_ISL_746242, EPI_ISL_746243, EPI_ISL_746244, EPI_ISL_746245, EPI_ISL_746246, EPI_ISL_746247, EPI_ISL_746248, EPI_ISL_746249, EPI_ISL_746250, EPI_ISL_746251, EPI_ISL_746252, EPI_ISL_746253, EPI_ISL_746254, EPI_ISL_746255, EPI_ISL_746256, EPI_ISL_746257, EPI_ISL_746258, EPI_ISL_746259, EPI_ISL_746260, EPI_ISL_746261, EPI_ISL_746262, EPI_ISL_746263, EPI_ISL_746264, EPI_ISL_746265, EPI_ISL_746266, EPI_ISL_746267, EPI_ISL_746268, EPI_ISL_746269, EPI_ISL_746270, EPI_ISL_746271, EPI_ISL_746272, EPI_ISL_746273, EPI_ISL_746274, EPI_ISL_746275, EPI_ISL_746276, EPI_ISL_746277, EPI_ISL_746278, EPI_ISL_746279, EPI_ISL_746280, EPI_ISL_746281, EPI_ISL_746282, EPI_ISL_746283, EPI_ISL_746284, EPI_ISL_746285, EPI_ISL_746286, EPI_ISL_746287, EPI_ISL_746288, EPI_ISL_746289, EPI_ISL_746290, EPI_ISL_746291, EPI_ISL_746292, EPI_ISL_746293, EPI_ISL_746295, EPI_ISL_746296, EPI_ISL_746297, EPI_ISL_746298, EPI_ISL_746299, EPI_ISL_746300, EPI_ISL_746301, EPI_ISL_746302, EPI_ISL_746303, EPI_ISL_746304, EPI_ISL_746305, EPI_ISL_746306, EPI_ISL_746307, EPI_ISL_746308, EPI_ISL_746309, EPI_ISL_746310, EPI_ISL_746311, EPI_ISL_746312, EPI_ISL_746313, EPI_ISL_746314, EPI_ISL_746315, EPI_ISL_746316, EPI_ISL_746317			
see above	Ginkgo Bioworks Clinical Laboratory	Utah Public Health Laboratory	Erin L. Young, Kelly Oakeson, Tara Gallagher, Michael T. Pyne, E. Susan Slechta, Melanie A. Mallory, Jeffrey B. Stevenson, Salika M. Shakir, David R. Hillyard, Malaika McKenzie-Bennett, James McGann, Jim Griffin, Keith Robison, Alex Plocik, Becky Schilling, Martha Pierson, Rebecca Littlefield, Michelle Spencer, Birgitte Simen
EPI_ISL_746319	Genome Center	Genome Center	Md. Shazid Hasan, Hassan M. Al-Emran, Ovinu Kibria Islam, A. S. M. Rubayet- Ul- Alam, Selina Akter, Md. Tanvir Islam, Pravas Chandra Roy, Shovon Lal Sarkar, Najmuj Sakib, Nigar Sultana Meghla, S. M. Tanjil Shah, Shireen Nigar, Md. Iqbal Kabir Jahid, Md. Anwar Hossain
EPI_ISL_746323	Genome Center	Genome Center	Ovinu Kibria Islam, Hassan M. Al-Emran, A. S. M. Rubayet- Ul- Alam, Md. Shazid Hasan, Selina Akter, Md. Tanvir Islam, Pravas Chandra Roy, Shovon Lal Sarkar, Najmuj Sakib, Nigar Sultana Meghla, S. M. Tanjil Shah, Shireen Nigar, Md. Iqbal Kabir Jahid, Md. Anwar Hossain
EPI_ISL_746324	Genome Center	Genome Center	A. S. M. Rubayet- Ul- Alam, Ovinu Kibria Islam, Hassan M. Al-Emran, Md. Shazid Hasan, Selina Akter, Md. Tanvir Islam, Pravas Chandra Roy, Shovon Lal Sarkar, Najmuj Sakib, Nigar Sultana Meghla, S. M. Tanjil Shah, Shireen Nigar, Md. Iqbal Kabir Jahid, Md. Anwar Hossain
EPI_ISL_746325, EPI_ISL_746326, EPI_ISL_746327, EPI_ISL_746328, EPI_ISL_746329, EPI_ISL_746330, EPI_ISL_746331, EPI_ISL_746332, EPI_ISL_746333, EPI_ISL_746334, EPI_ISL_746335, EPI_ISL_746336, EPI_ISL_746337, EPI_ISL_746338, EPI_ISL_746339, EPI_ISL_746340, EPI_ISL_746341, EPI_ISL_746360, EPI_ISL_746361, EPI_ISL_746362, EPI_ISL_746363, EPI_ISL_746364, EPI_ISL_746365, EPI_ISL_746366, EPI_ISL_746367, EPI_ISL_746368, EPI_ISL_746369, EPI_ISL_746370, EPI_ISL_746371, EPI_ISL_746372, EPI_ISL_746373, EPI_ISL_746374, EPI_ISL_746375, EPI_ISL_746376, EPI_ISL_746377, EPI_ISL_746378, EPI_ISL_746379, EPI_ISL_746380, EPI_ISL_746381, EPI_ISL_746382, EPI_ISL_746383, EPI_ISL_746384, EPI_ISL_746385, EPI_ISL_746386, EPI_ISL_746387, EPI_ISL_746388, EPI_ISL_746389, EPI_ISL_746390, EPI_ISL_746391, EPI_ISL_746392, EPI_ISL_746393, EPI_ISL_746394, EPI_ISL_746395, EPI_ISL_746396, EPI_ISL_746397, EPI_ISL_746398, EPI_ISL_746399, EPI_ISL_746400, EPI_ISL_746401, EPI_ISL_746402, EPI_ISL_746403, EPI_ISL_746404, EPI_ISL_746405, EPI_ISL_746406, EPI_ISL_746424, EPI_ISL_746425, EPI_ISL_746426, EPI_ISL_746427, EPI_ISL_746428, EPI_ISL_746429, EPI_ISL_746430, EPI_ISL_746431, EPI_ISL_746432, EPI_ISL_746433, EPI_ISL_746434, EPI_ISL_746435, EPI_ISL_746436, EPI_ISL_746437, EPI_ISL_746438, EPI_ISL_746439, EPI_ISL_746440, EPI_ISL_746441, EPI_ISL_746442, EPI_ISL_746443, EPI_ISL_746444, EPI_ISL_746445, EPI_ISL_746446, EPI_ISL_746447, EPI_ISL_746448, EPI_ISL_746449, EPI_ISL_746450, EPI_ISL_746451, EPI_ISL_746452, EPI_ISL_746453, EPI_ISL_746454, EPI_ISL_746455, EPI_ISL_746456, EPI_ISL_746457, EPI_ISL_746458, EPI_ISL_746459, EPI_ISL_746460, EPI_ISL_746461, EPI_ISL_746462, EPI_ISL_746463, EPI_ISL_746464, EPI_ISL_746465, EPI_ISL_746466, EPI_ISL_746467, EPI_ISL_746468, EPI_ISL_746469, EPI_ISL_746470, EPI_ISL_746471, EPI_ISL_746472, EPI_ISL_746473, EPI_ISL_746474, EPI_ISL_746475, EPI_ISL_746476, EPI_ISL_746477			
see above	Utah Public Health Laboratory	Utah Public Health Laboratory	Erin Young, Kelly Oakeson, Tara Gallagher
EPI_ISL_746478, EPI_ISL_746479, EPI_ISL_746480, EPI_ISL_746481, EPI_ISL_746483, EPI_ISL_746484, EPI_ISL_746485, EPI_ISL_746486, EPI_ISL_746487, EPI_ISL_746488, EPI_ISL_746490, EPI_ISL_746491, EPI_ISL_746492, EPI_ISL_746494, EPI_ISL_746495, EPI_ISL_746496, EPI_ISL_746497, EPI_ISL_746498, EPI_ISL_746499, EPI_ISL_746500, EPI_ISL_746501, EPI_ISL_746502, EPI_ISL_746503, EPI_ISL_746504, EPI_ISL_746505, EPI_ISL_746506, EPI_ISL_746507, EPI_ISL_746508, EPI_ISL_746509, EPI_ISL_746510, EPI_ISL_746511, EPI_ISL_746512, EPI_ISL_746513, EPI_ISL_746514, EPI_ISL_746515, EPI_ISL_746516, EPI_ISL_746517, EPI_ISL_746518, EPI_ISL_746519, EPI_ISL_746520, EPI_ISL_746521, EPI_ISL_746522, EPI_ISL_746523, EPI_ISL_746524, EPI_ISL_746525, EPI_ISL_746526, EPI_ISL_746527, EPI_ISL_746528, EPI_ISL_746529, EPI_ISL_746530, EPI_ISL_746531, EPI_ISL_746532, EPI_ISL_746533, EPI_ISL_746534, EPI_ISL_746535, EPI_ISL_746536, EPI_ISL_746537, EPI_ISL_746538, EPI_ISL_746539, EPI_ISL_746540, EPI_ISL_746541, EPI_ISL_746542, EPI_ISL_746543, EPI_ISL_746544, EPI_ISL_746545, EPI_ISL_746546, EPI_ISL_746547, EPI_ISL_746548, EPI_ISL_746549, EPI_ISL_746550, EPI_ISL_746551, EPI_ISL_746552, EPI_ISL_746553, EPI_ISL_746554, EPI_ISL_746555, EPI_ISL_746556, EPI_ISL_746557, EPI_ISL_746558, EPI_ISL_746559, EPI_ISL_746560, EPI_ISL_746561, EPI_ISL_746562, EPI_ISL_746563, EPI_ISL_746564, EPI_ISL_746565, EPI_ISL_746566, EPI_ISL_746567, EPI_ISL_746568, EPI_ISL_746569, EPI_ISL_746570, EPI_ISL_746571, EPI_ISL_746572, EPI_ISL_746573, EPI_ISL_746574, EPI_ISL_746575, EPI_ISL_746576, EPI_ISL_746577, EPI_ISL_746578, EPI_ISL_746579, EPI_ISL_746580, EPI_ISL_746581, EPI_ISL_746582, EPI_ISL_746583, EPI_ISL_746584, EPI_ISL_746585, EPI_ISL_746586, EPI_ISL_746587, EPI_ISL_746588, EPI_ISL_746589, EPI_ISL_746590, EPI_ISL_746591, EPI_ISL_746592, EPI_ISL_746593, EPI_ISL_746594, EPI_ISL_746595, EPI_ISL_746596, EPI_ISL_746597, EPI_ISL_746598, EPI_ISL_746599, EPI_ISL_746600, EPI_ISL_746601, EPI_ISL_746602, EPI_ISL_746603, EPI_ISL_746604, EPI_ISL_746605, EPI_ISL_746606, EPI_ISL_746607, EPI_ISL_746608, EPI_ISL_746609, EPI_ISL_746610, EPI_ISL_746611, EPI_ISL_746612, EPI_ISL_746613, EPI_ISL_746614, EPI_ISL_746615, EPI_ISL_746616, EPI_ISL_746617, EPI_ISL_746618, EPI_ISL_746619, EPI_ISL_746620, EPI_ISL_746621, EPI_ISL_746622, EPI_ISL_746623, EPI_ISL_746624, EPI_ISL_746625, EPI_ISL_746626, EPI_ISL_746627, EPI_ISL_746628, EPI_ISL_746629, EPI_ISL_746630, EPI_ISL_746631, EPI_ISL_746632, EPI_ISL_746633, EPI_ISL_746634, EPI_ISL_746635, EPI_ISL_746636, EPI_ISL_746637, EPI_ISL_746638, EPI_ISL_746639, EPI_ISL_746640, EPI_ISL_746641, EPI_ISL_746642, EPI_ISL_746643, EPI_ISL_746644, EPI_ISL_746645, EPI_ISL_746646, EPI_ISL_746647, EPI_ISL_746648, EPI_ISL_746649, EPI_ISL_746650, EPI_ISL_746651, EPI_ISL_746652, EPI_ISL_746653, EPI_ISL_746654, EPI_ISL_746655, EPI_ISL_746656, EPI_ISL_746657, EPI_ISL_746658, EPI_ISL_746659, EPI_ISL_746660, EPI_ISL_746661, EPI_ISL_746662, EPI_ISL_746663, EPI_ISL_746664, EPI_ISL_746665, EPI_ISL_746666, EPI_ISL_746667, EPI_ISL_746668, EPI_ISL_746669, EPI_ISL_746670, EPI_ISL_746671, EPI_ISL_746672, EPI_ISL_746673, EPI_ISL_746674, EPI_ISL_746675, EPI_ISL_746676, EPI_ISL_746677, EPI_ISL_746678, EPI_ISL_746679, EPI_ISL_746680, EPI_ISL_746681, EPI_ISL_746682, EPI_ISL_746683, EPI_ISL_746684, EPI_ISL_746685, EPI_ISL_746686, EPI_ISL_746687, EPI_ISL_746688, EPI_ISL_746689, EPI_ISL_746690, EPI_ISL_746691, EPI_ISL_746692, EPI_ISL_746693, EPI_ISL_746694, EPI_ISL_746695, EPI_ISL_746696, EPI_ISL_746697, EPI_ISL_746698, EPI_ISL_746699, EPI_ISL_746700, EPI_ISL_746701, EPI_ISL_746702, EPI_ISL_746703, EPI_ISL_746704, EPI_ISL_746705, EPI_ISL_746706, EPI_ISL_746707, EPI_ISL_746708, EPI_ISL_746709, EPI_ISL_746710, EPI_ISL_746711, EPI_ISL_746712, EPI_ISL_746713, EPI_ISL_746714, EPI_ISL_746715, EPI_ISL_746716, EPI_ISL_746717, EPI_ISL_746718, EPI_ISL_746719, EPI_ISL_746720, EPI_ISL_746721, EPI_ISL_746722, EPI_ISL_746723, EPI_ISL_746724, EPI_ISL_746725, EPI_ISL_746726, EPI_ISL_746727, EPI_ISL_746728, EPI_ISL_746729, EPI_ISL_746730, EPI_ISL_746731, EPI_ISL_746732, EPI_ISL_746733, EPI_ISL_746734, EPI_ISL_746735, EPI_ISL_746736, EPI_ISL_746737, EPI_ISL_746738, EPI_ISL_746739, EPI_ISL_746740, EPI_ISL_746741, EPI_ISL_746742, EPI_ISL_746743, EPI_ISL_746744, EPI_ISL_746745, EPI_ISL_746746, EPI_ISL_746747, EPI_ISL_746748, EPI_ISL_746749, EPI_ISL_746750, EPI_ISL_746751, EPI_ISL_746752, EPI_ISL_746753, EPI_ISL_746754, EPI_ISL_746755, EPI_ISL_746756, EPI_ISL_746757, EPI_ISL_746758, EPI_ISL_746759, EPI_ISL_746760, EPI_ISL_746761, EPI_ISL_746762, EPI_ISL_746763, EPI_ISL_746764, EPI_ISL_746765, EPI_ISL_746766, EPI_ISL_746767, EPI_ISL_746768, EPI_ISL_746769, EPI_ISL_746770, EPI_ISL_746771, EPI_ISL_746772, EPI_ISL_746773, EPI_ISL_746811, EPI_ISL_746812, EPI_ISL_746815, EPI_ISL_746816, EPI_ISL_746817, EPI_ISL_746818, EPI_ISL_746819, EPI_ISL_746820, EPI_ISL_746821, EPI_ISL_746822, EPI_ISL_746823, EPI_ISL_746824, EPI_ISL_746825			
see above	Genetica Molecular and Subdepartamento de Virologia ISP Chile	Instituto de Salud Publica de Chile	Javier Tognarelli, Barbara Parra, Loredana Arata, Jaime Lagos, Gisselle Barra, Patricia Bustos, Rodrigo Fasce, Andres Castillo, Jorge Fernandez
EPI_ISL_746826	National Institute for Infectious Diseases, INMI, "L. Spallanzani" IRCCS	National Institute for Infectious Diseases, INMI, "L. Spallanzani" IRCCS	C.E.M Gruber, B Bartolini, M Rueca, F Messina, E Giombini, A Di Caro, MR Capobianchi
EPI_ISL_746827	National Institute for Infectious Diseases, INMI, "L. Spallanzani" IRCCS	National Institute for Infectious Diseases, INMI, "L. Spallanzani" IRCCS	B Bartolini, C.E.M Gruber, M Rueca, F Messina, E Giombini, MR Capobianchi, A Di Caro
EPI_ISL_746828	National Institute for Infectious Diseases, INMI, "L. Spallanzani" IRCCS	National Institute for Infectious Diseases, INMI, "L. Spallanzani" IRCCS	M Rueca, B Bartolini, C.E.M Gruber, F Messina, E Giombini, A Di Caro, MR Capobianchi
EPI_ISL_746829	National Institute for Infectious Diseases, INMI, "L. Spallanzani" IRCCS	National Institute for Infectious Diseases, INMI, "L. Spallanzani" IRCCS	F Messina, E Giombini, M Rueca, B Bartolini, C.E.M Gruber, MR Capobianchi, A Di Caro
EPI_ISL_746830	National Institute for Infectious Diseases, INMI, "L. Spallanzani" IRCCS	National Institute for Infectious Diseases, INMI, "L. Spallanzani" IRCCS	E Giombini, M Rueca, B Bartolini, C.E.M Gruber, F Messina, A Di Caro, MR Capobianchi
EPI_ISL_746831	National Institute for Infectious Diseases, INMI, "L. Spallanzani" IRCCS	National Institute for Infectious Diseases, INMI, "L. Spallanzani" IRCCS	C.E.M Gruber, F Messina, M Rueca, B Bartolini, E Giombini, MR Capobianchi, A Di Caro
EPI_ISL_746832	National Institute for Infectious Diseases, INMI, "L.	National Institute for Infectious Diseases, INMI, "L.	E Giombini, C.E.M Gruber, M Rueca, B Bartolini, F Messina, A Di Caro, MR Capobianchi

	Spallanzani" IRCCS	Spallanzani" IRCCS	
EPI_ISL_746833	National Institute for Infectious Diseases, INMI, "L. Spallanzani" IRCCS	National Institute for Infectious Diseases, INMI, "L. Spallanzani" IRCCS	F Messina, M Rueca, B Bartolini, C.E.M Gruber, E Giombini, MR Capobianchi, A Di Caro
EPI_ISL_746834	National Institute for Infectious Diseases, INMI, "L. Spallanzani" IRCCS	National Institute for Infectious Diseases, INMI, "L. Spallanzani" IRCCS	B Bartolini, M Rueca, C.E.M Gruber, F Messina, E Giombini, A Di Caro, MR Capobianchi
EPI_ISL_746835	National Institute for Infectious Diseases, INMI, "L. Spallanzani" IRCCS	National Institute for Infectious Diseases, INMI, "L. Spallanzani" IRCCS	M Rueca, E Giombini, B Bartolini, C.E.M Gruber, F Messina, A Di Caro, MR Capobianchi
EPI_ISL_746836, EPI_ISL_746837, EPI_ISL_746838, EPI_ISL_746839, EPI_ISL_746840, EPI_ISL_746841, EPI_ISL_746842, EPI_ISL_746843, EPI_ISL_746844, EPI_ISL_746845, EPI_ISL_746846, EPI_ISL_746847, EPI_ISL_746848, EPI_ISL_746849, EPI_ISL_746850, EPI_ISL_746851, EPI_ISL_746852, EPI_ISL_746853, EPI_ISL_746854, EPI_ISL_746855, EPI_ISL_746856, EPI_ISL_746857, EPI_ISL_746858, EPI_ISL_746859, EPI_ISL_746860, EPI_ISL_746861, EPI_ISL_746862, EPI_ISL_746863, EPI_ISL_746864, EPI_ISL_746865, EPI_ISL_746866, EPI_ISL_746867, EPI_ISL_746868, EPI_ISL_746869, EPI_ISL_746870, EPI_ISL_746871, EPI_ISL_746872, EPI_ISL_746873, EPI_ISL_746874, EPI_ISL_746875, EPI_ISL_746876, EPI_ISL_746877, EPI_ISL_746878, EPI_ISL_746879, EPI_ISL_746880, EPI_ISL_746881, EPI_ISL_746882, EPI_ISL_746883, EPI_ISL_746884, EPI_ISL_746885, EPI_ISL_746886, EPI_ISL_746887, EPI_ISL_746888, EPI_ISL_746889, EPI_ISL_746890, EPI_ISL_746948, EPI_ISL_746949, EPI_ISL_746950, EPI_ISL_746951, EPI_ISL_746952, EPI_ISL_746953, EPI_ISL_746954, EPI_ISL_746955, EPI_ISL_746956, EPI_ISL_746957, EPI_ISL_746958, EPI_ISL_746959, EPI_ISL_746960, EPI_ISL_746961, EPI_ISL_746962, EPI_ISL_746963, EPI_ISL_746964, EPI_ISL_746965, EPI_ISL_746966, EPI_ISL_746967, EPI_ISL_746968, EPI_ISL_746969, EPI_ISL_746970, EPI_ISL_746971, EPI_ISL_746972, EPI_ISL_746973, EPI_ISL_746974, EPI_ISL_746975, EPI_ISL_746976, EPI_ISL_746977, EPI_ISL_746978, EPI_ISL_746979, EPI_ISL_746980, EPI_ISL_746981, EPI_ISL_746982, EPI_ISL_746983, EPI_ISL_746984, EPI_ISL_746985, EPI_ISL_746986, EPI_ISL_746987, EPI_ISL_746988, EPI_ISL_746989, EPI_ISL_746990, EPI_ISL_746991, EPI_ISL_746992, EPI_ISL_746993, EPI_ISL_746994, EPI_ISL_746995, EPI_ISL_746996, EPI_ISL_746997, EPI_ISL_746998, EPI_ISL_746999, EPI_ISL_747000, EPI_ISL_747001, EPI_ISL_747002, EPI_ISL_747003, EPI_ISL_747004, EPI_ISL_747005, EPI_ISL_747006, EPI_ISL_747007, EPI_ISL_747008, EPI_ISL_747009, EPI_ISL_747010, EPI_ISL_747011, EPI_ISL_747012, EPI_ISL_747013, EPI_ISL_747014, EPI_ISL_747015, EPI_ISL_747016, EPI_ISL_747017, EPI_ISL_747018, EPI_ISL_747019, EPI_ISL_747020, EPI_ISL_747021, EPI_ISL_747022, EPI_ISL_747023, EPI_ISL_747024, EPI_ISL_747025, EPI_ISL_747026, EPI_ISL_747027, EPI_ISL_747028, EPI_ISL_747029, EPI_ISL_747030, EPI_ISL_747031			
see above	Utah Public Health Laboratory	Utah Public Health Laboratory	Erin Young, Kelly Oakeson, Tara Gallagher
EPI_ISL_747032, EPI_ISL_747033, EPI_ISL_747034, EPI_ISL_747035, EPI_ISL_747036, EPI_ISL_747038, EPI_ISL_747039, EPI_ISL_747040, EPI_ISL_747041, EPI_ISL_747042, EPI_ISL_747043, EPI_ISL_747044, EPI_ISL_747045, EPI_ISL_747046, EPI_ISL_747047, EPI_ISL_747048, EPI_ISL_747049, EPI_ISL_747050, EPI_ISL_747051, EPI_ISL_747052, EPI_ISL_747053, EPI_ISL_747054, EPI_ISL_747055, EPI_ISL_747056, EPI_ISL_747057, EPI_ISL_747058, EPI_ISL_747059, EPI_ISL_747060, EPI_ISL_747061, EPI_ISL_747062, EPI_ISL_747063, EPI_ISL_747064, EPI_ISL_747065, EPI_ISL_747066, EPI_ISL_747067, EPI_ISL_747068, EPI_ISL_747069, EPI_ISL_747070, EPI_ISL_747071, EPI_ISL_747072, EPI_ISL_747073, EPI_ISL_747074, EPI_ISL_747075, EPI_ISL_747076, EPI_ISL_747077, EPI_ISL_747078, EPI_ISL_747079, EPI_ISL_747080, EPI_ISL_747081, EPI_ISL_747082, EPI_ISL_747083, EPI_ISL_747084, EPI_ISL_747085, EPI_ISL_747086, EPI_ISL_747087, EPI_ISL_747088, EPI_ISL_747089, EPI_ISL_747090, EPI_ISL_747091, EPI_ISL_747092, EPI_ISL_747093, EPI_ISL_747094, EPI_ISL_747095, EPI_ISL_747096, EPI_ISL_747097, EPI_ISL_747098, EPI_ISL_747099, EPI_ISL_747100, EPI_ISL_747101, EPI_ISL_747102, EPI_ISL_747103, EPI_ISL_747104, EPI_ISL_747105, EPI_ISL_747106, EPI_ISL_747107, EPI_ISL_747108, EPI_ISL_747110, EPI_ISL_747111, EPI_ISL_747112, EPI_ISL_747113, EPI_ISL_747114, EPI_ISL_747115, EPI_ISL_747116, EPI_ISL_747117, EPI_ISL_747118, EPI_ISL_747119, EPI_ISL_747120, EPI_ISL_747121, EPI_ISL_747122, EPI_ISL_747123, EPI_ISL_747124, EPI_ISL_747125, EPI_ISL_747126, EPI_ISL_747127, EPI_ISL_747128, EPI_ISL_747129, EPI_ISL_747130, EPI_ISL_747131, EPI_ISL_747132, EPI_ISL_747133, EPI_ISL_747134, EPI_ISL_747135, EPI_ISL_747136, EPI_ISL_747137, EPI_ISL_747138, EPI_ISL_747139, EPI_ISL_747140, EPI_ISL_747141, EPI_ISL_747142, EPI_ISL_747143, EPI_ISL_747144, EPI_ISL_747145, EPI_ISL_747146, EPI_ISL_747147, EPI_ISL_747149, EPI_ISL_747150, EPI_ISL_747151, EPI_ISL_747152, EPI_ISL_747153, EPI_ISL_747154, EPI_ISL_747155, EPI_ISL_747156, EPI_ISL_747157, EPI_ISL_747158, EPI_ISL_747159, EPI_ISL_747160, EPI_ISL_747161, EPI_ISL_747162, EPI_ISL_747163, EPI_ISL_747164, EPI_ISL_747165, EPI_ISL_747166, EPI_ISL_747167, EPI_ISL_747168, EPI_ISL_747169, EPI_ISL_747170, EPI_ISL_747171, EPI_ISL_747172, EPI_ISL_747173, EPI_ISL_747174, EPI_ISL_747175, EPI_ISL_747176, EPI_ISL_747177, EPI_ISL_747178, EPI_ISL_747179, EPI_ISL_747180, EPI_ISL_747181, EPI_ISL_747182, EPI_ISL_747183, EPI_ISL_747184, EPI_ISL_747185, EPI_ISL_747186, EPI_ISL_747187, EPI_ISL_747188, EPI_ISL_747189, EPI_ISL_747190, EPI_ISL_747191, EPI_ISL_747192, EPI_ISL_747193			
see above	Respiratory Viruses Branch, Centers for Disease Control and Prevention	Respiratory Viruses Branch, Centers for Disease Control and Prevention	Queen,K., Li,Y., Tao,Y., Uehara,A., Montmayeur,A., Paden,C.R., Cook,P.W., Marine,R., Sheth,M., Wang,H., Lee,J., Tong,S.
EPI_ISL_747194	Clinical Microbiology Laboratory, Faculty of Medicine, Universitas Indonesia	Clinical Microbiology Laboratory, Faculty of Medicine, Universitas Indonesia	Fera Ibrahim, Augustine Natasha, Andi Yasmon, Pratiwi Sudarmono, Yulia Rosa Saharman, Anis Karuniawati, Fithriyah
EPI_ISL_747195, EPI_ISL_747196, EPI_ISL_747197, EPI_ISL_747198, EPI_ISL_747199, EPI_ISL_747200, EPI_ISL_747201, EPI_ISL_747202, EPI_ISL_747203, EPI_ISL_747204, EPI_ISL_747205, EPI_ISL_747206, EPI_ISL_747207, EPI_ISL_747208, EPI_ISL_747209, EPI_ISL_747210, EPI_ISL_747211, EPI_ISL_747212, EPI_ISL_747213, EPI_ISL_747214, EPI_ISL_747215, EPI_ISL_747216, EPI_ISL_747217, EPI_ISL_747218, EPI_ISL_747219, EPI_ISL_747220, EPI_ISL_747221, EPI_ISL_747222, EPI_ISL_747223, EPI_ISL_747224, EPI_ISL_747225, EPI_ISL_747226, EPI_ISL_747227, EPI_ISL_747228, EPI_ISL_747229, EPI_ISL_747230, EPI_ISL_747231, EPI_ISL_747232			
see above	UW Virology Lab	UW Virology Lab	Pavitra Roychoudhury, Hong Xie, Lasata Shrestha, Michelle Lin, Meei-Li Huang, Keith R Jerome, Alexander Greninger
EPI_ISL_747233, EPI_ISL_747234	National Institute of Health Research and Development	National Institute of Health Research and Development	Subangkit; Indrasari,ND; Wulandari,D; Monika,M; Pawestri,HA; Puspa,KD; Nugraha,AA; Ikawati,HD; Pangesti,KNA; Soekarso,T; Paisal; Setiawaty,Vivi
EPI_ISL_747235	Sukabumi Health Laboratory	West Java Health Laboratory; School of Life Sciences and Technology, Institut Teknologi Bandung	Azzania Fibriani, Ema Rahmawati, Ryan Bayusantika Ristandi, Rifky Waluyajati Rachman, Cut Nur Cinthia Alamanda, Isak Solihin, Rini Robiani, Miftahul Faridl, Karimatu Khoirunnisa
EPI_ISL_747236	Sukabumi Public Health	West Java Health Laboratory; School of Life Sciences and Technology, Institut Teknologi Bandung	Azzania Fibriani, Ema Rahmawati, Ryan Bayusantika Ristandi, Rifky Waluyajati Rachman, Cut Nur Cinthia Alamanda, Isak Solihin, Rini Robiani, Miftahul Faridl, Karimatu Khoirunnisa
EPI_ISL_747237	National Institute of Health Research and Development	National Institute of Health Research and Development	Subangkit,Nafiandi,Estaria,V;Pawestri,HA;Puspa,KD;Nugraha,AA;Ikawati,HD;Pangesti,KNA;Soekarso,T;Paisal;Puspandari,N;Setiawaty,V
EPI_ISL_747238	Bogor Public Health	West Java Health Laboratory; School of Life Sciences and Technology, Institut Teknologi Bandung	Azzania Fibriani, Ema Rahmawati, Ryan Bayusantika Ristandi, Rifky Waluyajati Rachman, Cut Nur Cinthia Alamanda, Isak Solihin, Rini Robiani, Miftahul Faridl, Karimatu Khoirunnisa
EPI_ISL_747239	Kuningan Publlic Health Office	West Java Health Laboratory; School of Life Sciences and Technology, Institut Teknologi Bandung	Azzania Fibriani, Ema Rahmawati, Ryan Bayusantika Ristandi, Rifky Waluyajati Rachman, Cut Nur Cinthia Alamanda, Isak Solihin, Rini Robiani, Miftahul Faridl, Karimatu Khoirunnisa
EPI_ISL_747240	Al Islam Hospital	West Java Health Laboratory; School of Life Sciences and Technology, Institut Teknologi Bandung	Azzania Fibriani, Ema Rahmawati, Ryan Bayusantika Ristandi, Rifky Waluyajati Rachman, Cut Nur Cinthia Alamanda, Isak Solihin, Rini Robiani, Miftahul Faridl, Karimatu Khoirunnisa
EPI_ISL_747241	Subang Public Health Office	West Java Health Laboratory; School of Life Sciences and Technology, Institut Teknologi Bandung	Azzania Fibriani, Ema Rahmawati, Ryan Bayusantika Ristandi, Rifky Waluyajati Rachman, Cut Nur Cinthia Alamanda, Isak Solihin, Rini Robiani, Miftahul Faridl, Karimatu Khoirunnisa
EPI_ISL_747244	BBMP Urban PHC	Department of Neurovirology, National Institute of Mental Health and Neurosciences (NIMHANS)	Chitra Pattabiraman, Pramada Prasad, Anita S Desai, V Ravi
EPI_ISL_747245, EPI_ISL_747246, EPI_ISL_747247, EPI_ISL_747248, EPI_ISL_747249, EPI_ISL_747250, EPI_ISL_747251, EPI_ISL_747252, EPI_ISL_747253, EPI_ISL_747254, EPI_ISL_747255, EPI_ISL_747256, EPI_ISL_747257, EPI_ISL_747258, EPI_ISL_747260, EPI_ISL_747261, EPI_ISL_747262, EPI_ISL_747263, EPI_ISL_747264, EPI_ISL_747265, EPI_ISL_747266, EPI_ISL_747267, EPI_ISL_747268, EPI_ISL_747269, EPI_ISL_747270, EPI_ISL_747271, EPI_ISL_747272, EPI_ISL_747273, EPI_ISL_747274, EPI_ISL_747275, EPI_ISL_747276, EPI_ISL_747277, EPI_ISL_747278, EPI_ISL_747279, EPI_ISL_747280, EPI_ISL_747281			
see above	Division of Emerging Infectious Diseases, Bureau of Infectious Diseases Diagnosis Control, Korea Disease Control and Prevention Agency	Division of Emerging Infectious Diseases, Bureau of Infectious Diseases Diagnosis Control, Korea Disease Control and Prevention Agency	Ae Kyung Park, Il-Hwan Kim, Heui Man Kim, Jeong-Min Kim, Namjoo Lee, Chaeyoung Lee, Sang Hee Woo, Eun-Jin Kim
EPI_ISL_747283	DNA SOLUTION LTD.	DNA SOLUTION LTD.	Md. Imran Khan, Kazi Nadim Hasan, Abu Sufian, Jannatun Naima, Abdul Khaleque, Mizanur Rahman, Firoz Kabir, Mohammad Fazle Alam Rabbi, Sharif Akhteruzzaman
EPI_ISL_747284, EPI_ISL_747285, EPI_ISL_747286, EPI_ISL_747287, EPI_ISL_747288, EPI_ISL_747289, EPI_ISL_747290, EPI_ISL_747291, EPI_ISL_747292, EPI_ISL_747293, EPI_ISL_747294, EPI_ISL_747295, EPI_ISL_747296, EPI_ISL_747297, EPI_ISL_747298, EPI_ISL_747299, EPI_ISL_747300, EPI_ISL_747301, EPI_ISL_747302, EPI_ISL_747303, EPI_ISL_747304, EPI_ISL_747305, EPI_ISL_747306, EPI_ISL_747307, EPI_ISL_747308, EPI_ISL_747309, EPI_ISL_747310, EPI_ISL_747311, EPI_ISL_747312, EPI_ISL_747313, EPI_ISL_747314, EPI_ISL_747315, EPI_ISL_747316, EPI_ISL_747317, EPI_ISL_747318, EPI_ISL_747319, EPI_ISL_747320, EPI_ISL_747321, EPI_ISL_747322, EPI_ISL_747323, EPI_ISL_747324, EPI_ISL_747325, EPI_ISL_747326, EPI_ISL_747327, EPI_ISL_747328, EPI_ISL_747329, EPI_ISL_747330, EPI_ISL_747331, EPI_ISL_747332, EPI_ISL_747333, EPI_ISL_747334, EPI_ISL_747335, EPI_ISL_747336, EPI_ISL_747337, EPI_ISL_747338, EPI_ISL_747339, EPI_ISL_747340, EPI_ISL_747341, EPI_ISL_747342, EPI_ISL_747343, EPI_ISL_747344, EPI_ISL_747345, EPI_ISL_747346, EPI_ISL_747347, EPI_ISL_747348, EPI_ISL_747349, EPI_ISL_747350, EPI_ISL_747351, EPI_ISL_747352, EPI_ISL_747353, EPI_ISL_747354, EPI_ISL_747355, EPI_ISL_747356, EPI_ISL_747357, EPI_ISL_747358, EPI_ISL_747359, EPI_ISL_747360, EPI_ISL_747361, EPI_ISL_747362, EPI_ISL_747363, EPI_ISL_747364, EPI_ISL_747365, EPI_ISL_747366, EPI_ISL_747367, EPI_ISL_747368, EPI_ISL_747369, EPI_ISL_747370, EPI_ISL_747371, EPI_ISL_747372, EPI_ISL_747373, EPI_ISL_747374, EPI_ISL_747375, EPI_ISL_747376, EPI_ISL_747377, EPI_ISL_747378, EPI_ISL_747379, EPI_ISL_747380, EPI_ISL_747381, EPI_ISL_747382, EPI_ISL_747383, EPI_ISL_747384, EPI_ISL_747385, EPI_ISL_747386, EPI_ISL_747387, EPI_ISL_747388, EPI_ISL_747389, EPI_ISL_747390, EPI_ISL_747391, EPI_ISL_747392, EPI_ISL_747393, EPI_ISL_747394, EPI_ISL_747395, EPI_ISL_747396, EPI_ISL_747397, EPI_ISL_747398, EPI_ISL_747399, EPI_ISL_747400, EPI_ISL_747401, EPI_ISL_747402, EPI_ISL_747403, EPI_ISL_747404, EPI_ISL_747405, EPI_ISL_747406, EPI_ISL_747407, EPI_ISL_747408, EPI_ISL_747409, EPI_ISL_747410, EPI_ISL_747411, EPI_ISL_747412, EPI_ISL_747413, EPI_ISL_747414, EPI_ISL_747415, EPI_ISL_747416, EPI_ISL_747417, EPI_ISL_747418, EPI_ISL_747419, EPI_ISL_747420, EPI_ISL_747421, EPI_ISL_747425, EPI_ISL_747426, EPI_ISL_747427, EPI_ISL_747428, EPI_ISL_747429, EPI_ISL_747430, EPI_ISL_747431, EPI_ISL_747432, EPI_ISL_747433, EPI_ISL_747434, EPI_ISL_747435, EPI_ISL_747436, EPI_ISL_747437, EPI_ISL_747438, EPI_ISL_747439, EPI_ISL_747440, EPI_ISL_747441, EPI_ISL_747442, EPI_ISL_747443, EPI_ISL_747444, EPI_ISL_747445, EPI_ISL_747446, EPI_ISL_747447, EPI_ISL_747448, EPI_ISL_747449, EPI_ISL_747450, EPI_ISL_747451, EPI_ISL_747452, EPI_ISL_747453, EPI_ISL_747454, EPI_ISL_747455, EPI_ISL_747456, EPI_ISL_747457, EPI_ISL_747458			
see above	Division of Emerging Infectious Diseases, Bureau of Infectious Diseases Diagnosis Control, Korea Disease Control and Prevention Agency	Division of Emerging Infectious Diseases, Bureau of Infectious Diseases Diagnosis Control, Korea Disease Control and Prevention Agency	Ae Kyung Park, Il-Hwan Kim, Heui Man Kim, Jeong-Min Kim, Namjoo Lee, Chaeyoung Lee, Sang Hee Woo, Eun-Jin Kim
EPI_ISL_747459, EPI_ISL_747460, EPI_ISL_747461, EPI_ISL_747462, EPI_ISL_747463, EPI_ISL_747464	Ospedale San Bonifacio	Istituto Zooprofilattico Sperimentale delle Venezie	Adelaide Milani, Alessia Schivo, Annalisa Salviato, Erika Giorgia Quaranta, Ambra Pastori, Bianca Zecchin, Alice Fusaro, Isabella Monne, Calogero Terregino, Antonia Ricci
EPI_ISL_747465, EPI_ISL_747466, EPI_ISL_747467, EPI_ISL_747468,	Ospedale Mater Salutis	Istituto Zooprofilattico Sperimentale delle Venezie	Adelaide Milani, Alessia Schivo, Annalisa Salviato, Erika Giorgia Quaranta, Ambra Pastori, Bianca Zecchin, Alice Fusaro, Isabella Monne, Calogero Terregino, Antonia Ricci

EPI_ISL_747469, EPI_ISL_747470			
EPI_ISL_747471, EPI_ISL_747472, EPI_ISL_747473, EPI_ISL_747474, EPI_ISL_747475, EPI_ISL_747476	ULSS9 Distretto di Bussolengo	Istituto Zooprofilattico Sperimentale delle Venezie	Adelaide Milani, Alessia Schivo, Annalisa Salviato, Erika Giorgia Quaranta, Ambra Pastori, Bianca Zecchin, Alice Fusaro, Isabella Monne, Calogero Terregino, Antonia Ricci
EPI_ISL_747477, EPI_ISL_747478, EPI_ISL_747479, EPI_ISL_747480, EPI_ISL_747481	ULSS 5 Polesana	Istituto Zooprofilattico Sperimentale delle Venezie	Adelaide Milani, Alessia Schivo, Annalisa Salviato, Erika Giorgia Quaranta, Ambra Pastori, Bianca Zecchin, Alice Fusaro, Isabella Monne, Calogero Terregino, Antonia Ricci
EPI_ISL_747482, EPI_ISL_747483	Ospedale San Bonifacio	Istituto Zooprofilattico Sperimentale delle Venezie	Adelaide Milani, Alessia Schivo, Annalisa Salviato, Erika Giorgia Quaranta, Ambra Pastori, Bianca Zecchin, Alice Fusaro, Isabella Monne, Calogero Terregino, Antonia Ricci
EPI_ISL_747484, EPI_ISL_747485	Ospedale Mater Salutis	Istituto Zooprofilattico Sperimentale delle Venezie	Adelaide Milani, Alessia Schivo, Annalisa Salviato, Erika Giorgia Quaranta, Ambra Pastori, Bianca Zecchin, Alice Fusaro, Isabella Monne, Calogero Terregino, Antonia Ricci
EPI_ISL_747486, EPI_ISL_747487, EPI_ISL_747488, EPI_ISL_747489	ULSS 5 Polesana	Istituto Zooprofilattico Sperimentale delle Venezie	Adelaide Milani, Alessia Schivo, Annalisa Salviato, Erika Giorgia Quaranta, Ambra Pastori, Bianca Zecchin, Alice Fusaro, Isabella Monne, Calogero Terregino, Antonia Ricci
EPI_ISL_747490, EPI_ISL_747491, EPI_ISL_747492, EPI_ISL_747493, EPI_ISL_747494, EPI_ISL_747495, EPI_ISL_747496, EPI_ISL_747497, EPI_ISL_747498, EPI_ISL_747499, EPI_ISL_747500, EPI_ISL_747501, EPI_ISL_747502, EPI_ISL_747503, EPI_ISL_747504, EPI_ISL_747505, EPI_ISL_747506, EPI_ISL_747507, EPI_ISL_747508, EPI_ISL_747509, EPI_ISL_747510, EPI_ISL_747511, EPI_ISL_747512, EPI_ISL_747513, EPI_ISL_747514, EPI_ISL_747515, EPI_ISL_747516, EPI_ISL_747517, EPI_ISL_747518, EPI_ISL_747519			
see above	Respiratory Virus Unit, National Infection Service, Public Health England	COVID-19 Genomics UK (COG-UK) Consortium	PHE Covid Sequencing Team
EPI_ISL_747520	ZOTZ KLIMAS MVZ Düsseldorf-Centrum GbR ÜBAG für Labormedizin, Genetik, Zytologie, Pathologie	Center of Medical Microbiology, Virology, and Hospital Hygiene, University of Duesseldorf	Maximilian Damagnez, Alexander Dilthey, Ashley-Jane Duplessis, Patrick Finzer, Katrin Hoffmann, Torsten Houwaart, Lisanna Hülse, Malte Kohns Vasconcelos, Marek Korencak, Nadine Lübke, Jessica Nicolai, Klaus Pfeffer, Daniel Strelow, Jörg Timm, Andreas Walker, Tobias Wienemann, Rainer Zotz
EPI_ISL_747521, EPI_ISL_747522, EPI_ISL_747523, EPI_ISL_747524, EPI_ISL_747525	Dutch COVID-19 response team	National Institute for Public Health and the Environment (RIVM)	Adam Meijer, Harry Vennema, Jeroen Cremer, Sharon van den Brink, Bas van der Veer, AnneMarie van den Brandt, Florian Zwagemaker, Dennis Schmitz, Chantal Reusken, on behalf of the national COVID-19 response team
EPI_ISL_747527, EPI_ISL_747528, EPI_ISL_747529, EPI_ISL_747530, EPI_ISL_747531, EPI_ISL_747532, EPI_ISL_747533, EPI_ISL_747534, EPI_ISL_747535, EPI_ISL_747536, EPI_ISL_747537, EPI_ISL_747538, EPI_ISL_747539, EPI_ISL_747540, EPI_ISL_747541, EPI_ISL_747542, EPI_ISL_747543, EPI_ISL_747544, EPI_ISL_747545, EPI_ISL_747546, EPI_ISL_747547, EPI_ISL_747548, EPI_ISL_747549, EPI_ISL_747550, EPI_ISL_747551, EPI_ISL_747552, EPI_ISL_747553, EPI_ISL_747554, EPI_ISL_747555, EPI_ISL_747556, EPI_ISL_747557, EPI_ISL_747558, EPI_ISL_747559, EPI_ISL_747560, EPI_ISL_747561, EPI_ISL_747562, EPI_ISL_747563, EPI_ISL_747564, EPI_ISL_747565, EPI_ISL_747566, EPI_ISL_747567, EPI_ISL_747568, EPI_ISL_747569, EPI_ISL_747570, EPI_ISL_747571, EPI_ISL_747572, EPI_ISL_747573, EPI_ISL_747574, EPI_ISL_747575, EPI_ISL_747576, EPI_ISL_747577, EPI_ISL_747578, EPI_ISL_747579, EPI_ISL_747580, EPI_ISL_747581, EPI_ISL_747582, EPI_ISL_747583, EPI_ISL_747584, EPI_ISL_747585, EPI_ISL_747586, EPI_ISL_747587, EPI_ISL_747588, EPI_ISL_747589, EPI_ISL_747590, EPI_ISL_747591, EPI_ISL_747592, EPI_ISL_747593, EPI_ISL_747594, EPI_ISL_747595, EPI_ISL_747596, EPI_ISL_747597, EPI_ISL_747598, EPI_ISL_747599, EPI_ISL_747600, EPI_ISL_747601, EPI_ISL_747602, EPI_ISL_747603, EPI_ISL_747604, EPI_ISL_747605, EPI_ISL_747606, EPI_ISL_747607, EPI_ISL_747608, EPI_ISL_747610, EPI_ISL_747611, EPI_ISL_747612, EPI_ISL_747613, EPI_ISL_747614, EPI_ISL_747616, EPI_ISL_747617, EPI_ISL_747618, EPI_ISL_747619, EPI_ISL_747620, EPI_ISL_747621, EPI_ISL_747622, EPI_ISL_747623, EPI_ISL_747624, EPI_ISL_747625, EPI_ISL_747626, EPI_ISL_747627, EPI_ISL_747628, EPI_ISL_747629, EPI_ISL_747630, EPI_ISL_747631, EPI_ISL_747632, EPI_ISL_747633, EPI_ISL_747634, EPI_ISL_747635, EPI_ISL_747659, EPI_ISL_747660, EPI_ISL_747661, EPI_ISL_747662, EPI_ISL_747663, EPI_ISL_747664, EPI_ISL_747665, EPI_ISL_747666, EPI_ISL_747667, EPI_ISL_747668, EPI_ISL_747669, EPI_ISL_747670, EPI_ISL_747671, EPI_ISL_747672, EPI_ISL_747673, EPI_ISL_747674, EPI_ISL_747675, EPI_ISL_747676, EPI_ISL_747677, EPI_ISL_747678, EPI_ISL_747679, EPI_ISL_747680, EPI_ISL_747681, EPI_ISL_747682, EPI_ISL_747683, EPI_ISL_747684, EPI_ISL_747685, EPI_ISL_747686, EPI_ISL_747687, EPI_ISL_747688, EPI_ISL_747689, EPI_ISL_747690, EPI_ISL_747691, EPI_ISL_747692, EPI_ISL_747693, EPI_ISL_747694, EPI_ISL_747695, EPI_ISL_747696, EPI_ISL_747697, EPI_ISL_747698, EPI_ISL_747699, EPI_ISL_747700, EPI_ISL_747701, EPI_ISL_747702, EPI_ISL_747703, EPI_ISL_747704, EPI_ISL_747705, EPI_ISL_747706, EPI_ISL_747707, EPI_ISL_747708, EPI_ISL_747709, EPI_ISL_747710, EPI_ISL_747711, EPI_ISL_747712, EPI_ISL_747713, EPI_ISL_747714, EPI_ISL_747715, EPI_ISL_747716, EPI_ISL_747717, EPI_ISL_747718, EPI_ISL_747719, EPI_ISL_747720, EPI_ISL_747721, EPI_ISL_747722, EPI_ISL_747723, EPI_ISL_747724, EPI_ISL_747725, EPI_ISL_747726, EPI_ISL_747727, EPI_ISL_747728, EPI_ISL_747729, EPI_ISL_747730, EPI_ISL_747731, EPI_ISL_747732, EPI_ISL_747733, EPI_ISL_747734, EPI_ISL_747735, EPI_ISL_747736, EPI_ISL_747737, EPI_ISL_747738, EPI_ISL_747739, EPI_ISL_747740, EPI_ISL_747741, EPI_ISL_747742, EPI_ISL_747743, EPI_ISL_747744, EPI_ISL_747745, EPI_ISL_747746, EPI_ISL_747747, EPI_ISL_747748, EPI_ISL_747749, EPI_ISL_747750, EPI_ISL_747751, EPI_ISL_747752, EPI_ISL_747753, EPI_ISL_747754, EPI_ISL_747755, EPI_ISL_747756, EPI_ISL_747757, EPI_ISL_747758, EPI_ISL_747759, EPI_ISL_747760, EPI_ISL_747761, EPI_ISL_747762, EPI_ISL_747763, EPI_ISL_747764, EPI_ISL_747765, EPI_ISL_747766, EPI_ISL_747767, EPI_ISL_747768, EPI_ISL_747769, EPI_ISL_747770, EPI_ISL_747771, EPI_ISL_747772, EPI_ISL_747773, EPI_ISL_747774, EPI_ISL_747775, EPI_ISL_747776, EPI_ISL_747777, EPI_ISL_747778, EPI_ISL_747779, EPI_ISL_747780, EPI_ISL_747781, EPI_ISL_747782, EPI_ISL_747783, EPI_ISL_747784, EPI_ISL_747785, EPI_ISL_747786, EPI_ISL_747787, EPI_ISL_747788, EPI_ISL_747789, EPI_ISL_747790, EPI_ISL_747791, EPI_ISL_747792, EPI_ISL_747793, EPI_ISL_747794, EPI_ISL_747795, EPI_ISL_747796, EPI_ISL_747797, EPI_ISL_747798, EPI_ISL_747799, EPI_ISL_747800, EPI_ISL_747801, EPI_ISL_747802, EPI_ISL_747803, EPI_ISL_747804, EPI_ISL_747805, EPI_ISL_747806, EPI_ISL_747807, EPI_ISL_747808, EPI_ISL_747809, EPI_ISL_747810, EPI_ISL_747811, EPI_ISL_747812, EPI_ISL_747813, EPI_ISL_747814, EPI_ISL_747815, EPI_ISL_747816, EPI_ISL_747817, EPI_ISL_747818, EPI_ISL_747819, EPI_ISL_747820, EPI_ISL_747821, EPI_ISL_747822, EPI_ISL_747823, EPI_ISL_747824, EPI_ISL_747825, EPI_ISL_747826, EPI_ISL_747827, EPI_ISL_747828, EPI_ISL_747829, EPI_ISL_747830, EPI_ISL_747831, EPI_ISL_747832, EPI_ISL_747833, EPI_ISL_747834, EPI_ISL_747835, EPI_ISL_747836, EPI_ISL_747837, EPI_ISL_747838, EPI_ISL_747839, EPI_ISL_747840, EPI_ISL_747841, EPI_ISL_747842, EPI_ISL_747843, EPI_ISL_747844, EPI_ISL_747845, EPI_ISL_747846, EPI_ISL_747847, EPI_ISL_747848, EPI_ISL_747849, EPI_ISL_747850, EPI_ISL_747851, EPI_ISL_747852, EPI_ISL_747853, EPI_ISL_747854, EPI_ISL_747855, EPI_ISL_747856, EPI_ISL_747857, EPI_ISL_747858, EPI_ISL_747859, EPI_ISL_747860, EPI_ISL_747861, EPI_ISL_747862, EPI_ISL_747863, EPI_ISL_747864, EPI_ISL_747865, EPI_ISL_747866, EPI_ISL_747867, EPI_ISL_747868, EPI_ISL_747869, EPI_ISL_747870, EPI_ISL_747871, EPI_ISL_747872, EPI_ISL_747873, EPI_ISL_747874, EPI_ISL_747875, EPI_ISL_747876, EPI_ISL_747877, EPI_ISL_747878, EPI_ISL_747879, EPI_ISL_747880, EPI_ISL_747881, EPI_ISL_747882, EPI_ISL_747883, EPI_ISL_747884, EPI_ISL_747885, EPI_ISL_747886, EPI_ISL_747887, EPI_ISL_747888, EPI_ISL_747889, EPI_ISL_747890, EPI_ISL_747891, EPI_ISL_747892, EPI_ISL_747893, EPI_ISL_747894, EPI_ISL_747895, EPI_ISL_747896, EPI_ISL_747897, EPI_ISL_747898, EPI_ISL_747899, EPI_ISL_747900, EPI_ISL_747901, EPI_ISL_747902, EPI_ISL_747903, EPI_ISL_747904, EPI_ISL_747905, EPI_ISL_747906, EPI_ISL_747907, EPI_ISL_747908, EPI_ISL_747909, EPI_ISL_747910, EPI_ISL_747911, EPI_ISL_747912, EPI_ISL_747913, EPI_ISL_747914, EPI_ISL_747915, EPI_ISL_747916, EPI_ISL_747917, EPI_ISL_747918, EPI_ISL_747919, EPI_ISL_747920, EPI_ISL_747921, EPI_ISL_747922, EPI_ISL_747923, EPI_ISL_747924, EPI_ISL_747925, EPI_ISL_747926, EPI_ISL_747927, EPI_ISL_747928, EPI_ISL_747929, EPI_ISL_747930, EPI_ISL_747931, EPI_ISL_747932, EPI_ISL_747933, EPI_ISL_747934, EPI_ISL_747935, EPI_ISL_747936, EPI_ISL_747937, EPI_ISL_747938, EPI_ISL_747939, EPI_ISL_747940, EPI_ISL_747941, EPI_ISL_747942, EPI_ISL_747943, EPI_ISL_747944, EPI_ISL_747945, EPI_ISL_747946, EPI_ISL_747947, EPI_ISL_747948, EPI_ISL_747949, EPI_ISL_747950, EPI_ISL_747951, EPI_ISL_747952, EPI_ISL_747953, EPI_ISL_747954, EPI_ISL_747955, EPI_ISL_747956, EPI_ISL_747957, EPI_ISL_747958, EPI_ISL_747959, EPI_ISL_747960, EPI_ISL_747961, EPI_ISL_747962, EPI_ISL_747963, EPI_ISL_747964, EPI_ISL_747965, EPI_ISL_747966, EPI_ISL_747967, EPI_ISL_747968, EPI_ISL_747969, EPI_ISL_747970, EPI_ISL_747971, EPI_ISL_747972, EPI_ISL_747973, EPI_ISL_747974, EPI_ISL_747975, EPI_ISL_747976, EPI_ISL_747977, EPI_ISL_747978, EPI_ISL_747979, EPI_ISL_747980, EPI_ISL_747981, EPI_ISL_747982, EPI_ISL_747983, EPI_ISL_747984, EPI_ISL_747985, EPI_ISL_747986, EPI_ISL_747987, EPI_ISL_747988, EPI_ISL_747989, EPI_ISL_747990, EPI_ISL_747991, EPI_ISL_747992, EPI_ISL_747993, EPI_ISL_747994, EPI_ISL_747995, EPI_ISL_747996, EPI_ISL_747997, EPI_ISL_747998, EPI_ISL_747999, EPI_ISL_748000, EPI_ISL_748001, EPI_ISL_748002, EPI_ISL_748003, EPI_ISL_748004, EPI_ISL_748005, EPI_ISL_748006, EPI_ISL_748007, EPI_ISL_748008, EPI_ISL_748009, EPI_ISL_748010, EPI_ISL_748011, EPI_ISL_748012, EPI_ISL_748013, EPI_ISL_748014, EPI_ISL_748015, EPI_ISL_748016, EPI_ISL_748017, EPI_ISL_748018, EPI_ISL_748019, EPI_ISL_748020, EPI_ISL_748021, EPI_ISL_748022, EPI_ISL_748023, EPI_ISL_748024, EPI_ISL_748025, EPI_ISL_748026, EPI_ISL_748027, EPI_ISL_748028, EPI_ISL_748029, EPI_ISL_748030, EPI_ISL_748031, EPI_ISL_748032, EPI_ISL_748033, EPI_ISL_748034, EPI_ISL_748035, EPI_ISL_748036, EPI_ISL_748037, EPI_ISL_748038, EPI_ISL_748039, EPI_ISL_748040, EPI_ISL_748041, EPI_ISL_748042, EPI_ISL_748043, EPI_ISL_748044, EPI_ISL_748045, EPI_ISL_748046, EPI_ISL_748047, EPI_ISL_748048, EPI_ISL_748049, EPI_ISL_748050, EPI_ISL_748051, EPI_ISL_748052, EPI_ISL_748053, EPI_ISL_748054, EPI_ISL_748055, EPI_ISL_748056, EPI_ISL_748057, EPI_ISL_748058, EPI_ISL_748059, EPI_ISL_748060, EPI_ISL_748061, EPI_ISL_748062, EPI_ISL_748063, EPI_ISL_748064, EPI_ISL_748065, EPI_ISL_748066, EPI_ISL_748067, EPI_ISL_748068, EPI_ISL_748069, EPI_ISL_748070, EPI_ISL_748071, EPI_ISL_748072, EPI_ISL_748073, EPI_ISL_748074, EPI_ISL_748075, EPI_ISL_748076, EPI_ISL_748077, EPI_ISL_748078, EPI_ISL_748079, EPI_ISL_748080, EPI_ISL_748081, EPI_ISL_748082, EPI_ISL_748083, EPI_ISL_748084, EPI_ISL_748085, EPI_ISL_748086, EPI_ISL_748087, EPI_ISL_748088, EPI_ISL_748089, EPI_ISL_748090, EPI_ISL_748091, EPI_ISL_748092, EPI_ISL_748093, EPI_ISL_748094, EPI_ISL_748095, EPI_ISL_748096, EPI_ISL_748097, EPI_ISL_748098, EPI_ISL_748099, EPI_ISL_748100, EPI_ISL_748101, EPI_ISL_748102, EPI_ISL_748103, EPI_ISL_748104, EPI_ISL_748105, EPI_ISL_748106, EPI_ISL_748107, EPI_ISL_748108, EPI_ISL_748109, EPI_ISL_748110, EPI_ISL_748111, EPI_ISL_748112, EPI_ISL_748113, EPI_ISL_748114, EPI_ISL_748115, EPI_ISL_748116, EPI_ISL_748117, EPI_ISL_748118, EPI_ISL_748119, EPI_ISL_748120, EPI_ISL_748121, EPI_ISL_748122, EPI_ISL_748123, EPI_ISL_748124, EPI_ISL_748125, EPI_ISL_748126, EPI_ISL_748127, EPI_ISL_748128, EPI_ISL_748129, EPI_ISL_748130, EPI_ISL_748131, EPI_ISL_748132, EPI_ISL_748133, EPI_ISL_748134, EPI_ISL_748135, EPI_ISL_748136, EPI_ISL_748137, EPI_ISL_748146, EPI_ISL_748147, EPI_ISL_748148, EPI_ISL_748149, EPI_ISL_748150, EPI_ISL_748151, EPI_ISL_748152, EPI_ISL_748153, EPI_ISL_748154, EPI_ISL_748155, EPI_ISL_748156, EPI_ISL_748157, EPI_ISL_748158, EPI_ISL_748159, EPI_ISL_748160, EPI_ISL_748161, EPI_ISL_748162, EPI_ISL_748163, EPI_ISL_748164, EPI_ISL_748165, EPI_ISL_748166, EPI_ISL_748168, EPI_ISL_748169, EPI_ISL_748170, EPI_ISL_748171, EPI_ISL_748172, EPI_ISL_748173, EPI_ISL_748174, EPI_ISL_748175, EPI_ISL_748176, EPI_ISL_748177, EPI_ISL_748178, EPI_ISL_748179, EPI_ISL_748180, EPI_ISL_748181, EPI_ISL_748182, EPI_ISL_748183, EPI_ISL_748184, EPI_ISL_748185, EPI_ISL_748186, EPI_ISL_748187, EPI_ISL_748188, EPI_ISL_748189, EPI_ISL_748190, EPI_ISL_748191, EPI_ISL_748192, EPI_ISL_748193, EPI_ISL_748194, EPI_ISL_748195, EPI_ISL_748196, EPI_ISL_748197, EPI_ISL_748198, EPI_ISL_748199, EPI_ISL_748200, EPI_ISL_748201, EPI_ISL_748202, EPI_ISL_748203, EPI_ISL_748204, EPI_ISL_748205, EPI_ISL_748206, EPI_ISL_748207, EPI_ISL_748208, EPI_ISL_748209, EPI_ISL_748210, EPI_ISL_748211, EPI_ISL_748212, EPI_ISL_748213, EPI_ISL_748214, EPI_ISL_748215, EPI_ISL_748216, EPI_ISL_748217, EPI_ISL_748218, EPI_ISL_748219, EPI_ISL_748220, EPI_ISL_748221, EPI_ISL_748222, EPI_ISL_748223, EPI_ISL_748224, EPI_ISL_748225, EPI_ISL_748226, EPI_ISL_748227, EPI_ISL_748228, EPI_ISL_748229, EPI_ISL_748230, EPI_ISL_748231, EPI_ISL_748232, EPI_ISL_748233, EPI_ISL_748234, EPI_ISL_748235, EPI_ISL_748236, EPI_ISL_748237, EPI_ISL_748238, EPI_ISL_748239, EPI_ISL_748240, EPI_ISL_748241, EPI_ISL_748242, EPI_ISL_748243, EPI_ISL_748244, EPI_ISL_748245, EPI_ISL_748246, EPI_ISL_748248, EPI_ISL_748249, EPI_ISL_748250, EPI_ISL_748251, EPI_ISL_748252, EPI_ISL_748253, EPI_ISL_748254, EPI_ISL_748255, EPI_ISL_748256, EPI_ISL_748257, EPI_ISL_748258, EPI_ISL_748259, EPI_ISL_748260, EPI_ISL_748261, EPI_ISL_748262, EPI_ISL_748263, EPI_ISL_748264, EPI_ISL_748265, EPI_ISL_748266, EPI_ISL_748267, EPI_ISL_748268, EPI_ISL_748269, EPI_ISL_748270, EPI_ISL_748271, EPI_ISL_748272, EPI_ISL_748273, EPI_ISL_748274, EPI_ISL_748275, EPI_ISL_748276, EPI_ISL_748277, EPI_ISL_748278, EPI_ISL_748279, EPI_ISL_748280, EPI_ISL_748281, EPI_ISL_748282, EPI_ISL_748283, EPI_ISL_748284, EPI_ISL_748285, EPI_ISL_748286, EPI_ISL_748287, EPI_ISL_748288, EPI_ISL_748289, EPI_ISL_748290, EPI_ISL_748291, EPI_ISL_748292, EPI_ISL_748293, EPI_ISL_748294, EPI_ISL_748295, EPI_ISL_748296, EPI_ISL_748297, EPI_ISL_748298, EPI_ISL_748299, EPI_ISL_748300, EPI_ISL_748301, EPI_ISL_748302, EPI_ISL_748303, EPI_ISL_748304, EPI_ISL_748305, EPI_ISL_748306, EPI_ISL_748307, EPI_ISL_748308, EPI_ISL_748309, EPI_ISL_748310, EPI_ISL_748311, EPI_ISL_748312, EPI_ISL_748313, EPI_ISL_748314, EPI_ISL_748315, EPI_ISL_748316, EPI_ISL_748317, EPI_ISL_748318, EPI_ISL_748319, EPI_ISL_748320, EPI_ISL_748321, EPI_ISL_748322, EPI_ISL_748323, EPI_ISL_748324, EPI_ISL_748359, EPI_ISL_748360, EPI_ISL_748361, EPI_ISL_748362, EPI_ISL_748363, EPI_ISL_748364, EPI_ISL_748365, EPI_ISL_748366, EPI_ISL_748367, EPI_ISL_748368, EPI_ISL_748369, EPI_ISL_748370, EPI_ISL_748371, EPI_ISL_748372, EPI_ISL_748373, EPI_ISL_748374, EPI_ISL_748375, EPI_ISL_748376, EPI_ISL_748377, EPI_ISL_748378, EPI_ISL_748379, EPI_ISL_748380, EPI_ISL_748381, EPI_ISL_748382, EPI_ISL_748383, EPI_ISL_748384, EPI_ISL_748385, EPI_ISL_748386, EPI_ISL_748387, EPI_ISL_748388, EPI_ISL_748389, EPI_ISL_748390, EPI_ISL_748391, EPI_ISL_748392, EPI_ISL_748393, EPI_ISL_748394, EPI_ISL_748395, EPI_ISL_748396, EPI_ISL_748397, EPI_ISL_748398, EPI_ISL_748399, EPI_ISL_748400, EPI_ISL_748401, EPI_ISL_748402, EPI_ISL_748403, EPI_ISL_748404, EPI_ISL_748405, EPI_ISL_748406, EPI_ISL_748407, EPI_ISL_748408, EPI_ISL_748409, EPI_ISL_748410, EPI_ISL_748411, EPI_ISL_748412, EPI_ISL_748413, EPI_ISL_748414, EPI_ISL_748415, EPI_ISL_748416, EPI_ISL_748417, EPI_ISL_748418, EPI_ISL_748419, EPI_ISL_748420, EPI_ISL_748421, EPI_ISL_748422, EPI_ISL_748423, EPI_ISL_748424, EPI_ISL_748425, EPI_ISL_748426, EPI_ISL_748427, EPI_ISL_748428, EPI_ISL_748429, EPI_ISL_748430, EPI_ISL_748431, EPI_ISL_748432, EPI_ISL_748433, EPI_ISL_748434, EPI_ISL_748435, EPI_ISL_748436, EPI_ISL_748437, EPI_ISL_748438, EPI_ISL_748439, EPI_ISL_748440, EPI_ISL_748441, EPI_ISL_748442, EPI_ISL_748443, EPI_ISL_748444, EPI_ISL_748445, EPI_ISL_748446, EPI_ISL_748447, EPI_ISL_748448, EPI_ISL_748449, EPI_ISL_748450, EPI_ISL_748451, EPI_ISL_748452, EPI_ISL_748453, EPI_ISL_748454, EPI_ISL_748455, EPI_ISL_748456, EPI_ISL_748457, EPI_ISL_748458, EPI_ISL_748459, EPI_ISL_748460, EPI_ISL_748461, EPI_ISL_748462, EPI_ISL_748463, EPI_ISL_748464, EPI_ISL_748465, EPI_ISL_748466, EPI_ISL_748467, EPI_ISL_748468, EPI_ISL_748469, EPI_ISL_748470, EPI_ISL_748471, EPI_ISL_748472, EPI_ISL_748473, EPI_ISL_748474, EPI_ISL_748475, EPI_ISL_748476, EPI_ISL_748477, EPI_ISL_748478, EPI_ISL_748479, EPI_ISL_748480, EPI_ISL_748481, EPI_ISL_748482, EPI_ISL_748483, EPI_ISL_748484, EPI_ISL_748485, EPI_ISL_748486, EPI_ISL_748487, EPI_ISL_748488, EPI_ISL_748489, EPI_ISL_748490, EPI_ISL_748491, EPI_ISL_748492, EPI_ISL_748493, EPI_ISL_748494, EPI_ISL_748495, EPI_ISL_748496, EPI_ISL_748497, EPI_ISL_748498, EPI_ISL_748499, EPI_ISL_748500, EPI_ISL_748501, EPI_ISL_748502, EPI_ISL_748503, EPI_ISL_748504, EPI_ISL_748507, EPI_ISL_748508, EPI_ISL_748509, EPI_ISL_748510, EPI_ISL_748511, EPI_ISL_748512, EPI_ISL_748513, EPI_ISL_748514, EPI_ISL_748515, EPI_ISL_748516, EPI_ISL_748517, EPI_ISL_748518, EPI_ISL_748519, EPI_ISL_748520, EPI_ISL_748521, EPI_ISL_748522, EPI_ISL_748523, EPI_ISL_748524, EPI_ISL_748525, EPI_ISL_748526, EPI_ISL_748527, EPI_ISL_748528, EPI_ISL_748529, EPI_ISL_748530, EPI_ISL_748531, EPI_ISL_748532, EPI_ISL_748533, EPI_ISL_748534, EPI_ISL_748535, EPI_ISL_748536, EPI_ISL_748537, EPI_ISL_748538, EPI_ISL_748539, EPI_ISL_748540, EPI_ISL_748541, EPI_ISL_748542, EPI_ISL_748543, EPI_ISL_748544, EPI_ISL_748545, EPI_ISL_748546, EPI_ISL_748547, EPI_ISL_748548, EPI_ISL_748549, EPI_ISL_748550, EPI_ISL_748551, EPI_ISL_748552, EPI_ISL_748553, EPI_ISL_748554, EPI_ISL_748555, EPI_ISL_748556, EPI_ISL_748557, EPI_ISL_748558, EPI_ISL_748559, EPI_ISL_748560, EPI_ISL_748561, EPI_ISL_748562, EPI_ISL_748563, EPI_ISL_748564			
see above	Department of Virus and Microbiological Special Diagnostics, Statens Serum Institut, Copenhagen, Denmark	Albertsen Lab, Department of Chemistry and Bioscience, Aalborg University, Denmark	Danish Covid-19 Genome Consortium
