# Supplementary material for: Deepening of In Silico Evaluation of SARS-CoV-2 Detection RT-qPCR Assays in the Context of New Variants
Source: Genes (Basel). 2021 Apr 13;12(4):565. doi: 10.3390/genes12040565 (PMC8069896; doi:10.3390/genes12040565)
Supplement: Supplementary file 1 [file genes-12-00565-s001.zip › Supplementary file S9.zip!/Acknowledgement tables/gisaid_hcov-19_acknowledgement_table_19.pdf]

We gratefully acknowledge the following Authors from the Originating laboratories responsible for obtaining the specimens, as well as the Submitting laboratories where the genome data were generated and shared via GISAID, on which this research is based.

All Submitters of data may be contacted directly via www.gisaid.org

Authors are sorted alphabetically.

Accession ID	Originating Laboratory	Submitting Laboratory	Authors
EPI_ISL_748565, EPI_ISL_748566, EPI_ISL_748567, EPI_ISL_748568, EPI_ISL_748569, EPI_ISL_748570, EPI_ISL_748571, EPI_ISL_748572, EPI_ISL_748573, EPI_ISL_748574, EPI_ISL_748575, EPI_ISL_748576, EPI_ISL_748577, EPI_ISL_748578, EPI_ISL_748579, EPI_ISL_748580, EPI_ISL_748581, EPI_ISL_748582, EPI_ISL_748583, EPI_ISL_748584, EPI_ISL_748585, EPI_ISL_748586, EPI_ISL_748587, EPI_ISL_748588, EPI_ISL_748589, EPI_ISL_748590, EPI_ISL_748591, EPI_ISL_748592, EPI_ISL_748593, EPI_ISL_748594, EPI_ISL_748595, EPI_ISL_748596, EPI_ISL_748597, EPI_ISL_748598, EPI_ISL_748599, EPI_ISL_748600, EPI_ISL_748601, EPI_ISL_748602, EPI_ISL_748603, EPI_ISL_748604, EPI_ISL_748605, EPI_ISL_748606, EPI_ISL_748607, EPI_ISL_748608, EPI_ISL_748609, EPI_ISL_748610, EPI_ISL_748611, EPI_ISL_748612, EPI_ISL_748613, EPI_ISL_748614, EPI_ISL_748615, EPI_ISL_748616, EPI_ISL_748617, EPI_ISL_748618, EPI_ISL_748619, EPI_ISL_748620, EPI_ISL_748621, EPI_ISL_748622, EPI_ISL_748623, EPI_ISL_748624, EPI_ISL_748625, EPI_ISL_748626, EPI_ISL_748627, EPI_ISL_748628, EPI_ISL_748629, EPI_ISL_748630, EPI_ISL_748631, EPI_ISL_748632, EPI_ISL_748633, EPI_ISL_748634, EPI_ISL_748635, EPI_ISL_748636, EPI_ISL_748637, EPI_ISL_748638, EPI_ISL_748639, EPI_ISL_748640, EPI_ISL_748641, EPI_ISL_748642, EPI_ISL_748643, EPI_ISL_748644, EPI_ISL_748645, EPI_ISL_748646, EPI_ISL_748647, EPI_ISL_748648, EPI_ISL_748649, EPI_ISL_748650, EPI_ISL_748651, EPI_ISL_748652, EPI_ISL_748653, EPI_ISL_748654, EPI_ISL_748655, EPI_ISL_748656, EPI_ISL_748657, EPI_ISL_748658, EPI_ISL_748659, EPI_ISL_748660, EPI_ISL_748661, EPI_ISL_748662, EPI_ISL_748663, EPI_ISL_748664, EPI_ISL_748665, EPI_ISL_748666, EPI_ISL_748668, EPI_ISL_748669, EPI_ISL_748670, EPI_ISL_748671, EPI_ISL_748672, EPI_ISL_748673, EPI_ISL_748674, EPI_ISL_748675, EPI_ISL_748676, EPI_ISL_748677, EPI_ISL_748678, EPI_ISL_748679, EPI_ISL_748680, EPI_ISL_748681, EPI_ISL_748682, EPI_ISL_748683, EPI_ISL_748684, EPI_ISL_748685, EPI_ISL_748686, EPI_ISL_748687, EPI_ISL_748688, EPI_ISL_748689, EPI_ISL_748690, EPI_ISL_748691, EPI_ISL_748692, EPI_ISL_748693, EPI_ISL_748694, EPI_ISL_748695, EPI_ISL_748696, EPI_ISL_748697, EPI_ISL_748698, EPI_ISL_748699, EPI_ISL_748700, EPI_ISL_748701, EPI_ISL_748702, EPI_ISL_748703, EPI_ISL_748704, EPI_ISL_748705, EPI_ISL_748706, EPI_ISL_748707, EPI_ISL_748708, EPI_ISL_748709, EPI_ISL_748710, EPI_ISL_748711, EPI_ISL_748712, EPI_ISL_748713, EPI_ISL_748714, EPI_ISL_748715, EPI_ISL_748716, EPI_ISL_748717, EPI_ISL_748718, EPI_ISL_748719, EPI_ISL_748720, EPI_ISL_748721, EPI_ISL_748722, EPI_ISL_748723, EPI_ISL_748724, EPI_ISL_748725, EPI_ISL_748726, EPI_ISL_748727, EPI_ISL_748728, EPI_ISL_748729, EPI_ISL_748730, EPI_ISL_748731, EPI_ISL_748732, EPI_ISL_748733, EPI_ISL_748734, EPI_ISL_748735, EPI_ISL_748736, EPI_ISL_748737, EPI_ISL_748738, EPI_ISL_748739, EPI_ISL_748740, EPI_ISL_748741, EPI_ISL_748742, EPI_ISL_748743, EPI_ISL_748744, EPI_ISL_748745, EPI_ISL_748746, EPI_ISL_748747, EPI_ISL_748748, EPI_ISL_748749, EPI_ISL_748750, EPI_ISL_748751, EPI_ISL_748752, EPI_ISL_748753, EPI_ISL_748754, EPI_ISL_748755, EPI_ISL_748756, EPI_ISL_748757, EPI_ISL_748758, EPI_ISL_748759, EPI_ISL_748760, EPI_ISL_748761, EPI_ISL_748762, EPI_ISL_748763, EPI_ISL_748764, EPI_ISL_748765, EPI_ISL_748766, EPI_ISL_748767, EPI_ISL_748768, EPI_ISL_748769, EPI_ISL_748770, EPI_ISL_748771, EPI_ISL_748772, EPI_ISL_748773, EPI_ISL_748774, EPI_ISL_748775, EPI_ISL_748776, EPI_ISL_748777, EPI_ISL_748778, EPI_ISL_748779, EPI_ISL_748780, EPI_ISL_748781, EPI_ISL_748782, EPI_ISL_748783, EPI_ISL_748784, EPI_ISL_748785, EPI_ISL_748786, EPI_ISL_748787, EPI_ISL_748788, EPI_ISL_748789, EPI_ISL_748790, EPI_ISL_748791, EPI_ISL_748792, EPI_ISL_748793, EPI_ISL_748794, EPI_ISL_748795, EPI_ISL_748796, EPI_ISL_748797, EPI_ISL_748798, EPI_ISL_748799, EPI_ISL_748800, EPI_ISL_748801, EPI_ISL_748802, EPI_ISL_748803, EPI_ISL_748804, EPI_ISL_748805, EPI_ISL_748806, EPI_ISL_748807, EPI_ISL_748808, EPI_ISL_748809, EPI_ISL_748810, EPI_ISL_748811, EPI_ISL_748812, EPI_ISL_748813, EPI_ISL_748814, EPI_ISL_748815, EPI_ISL_748816, EPI_ISL_748817, EPI_ISL_748818, EPI_ISL_748819, EPI_ISL_748820, EPI_ISL_748821, EPI_ISL_748822, EPI_ISL_748823, EPI_ISL_748824, EPI_ISL_748825, EPI_ISL_748826, EPI_ISL_748827, EPI_ISL_748828, EPI_ISL_748829, EPI_ISL_748830, EPI_ISL_748831, EPI_ISL_748832, EPI_ISL_748833, EPI_ISL_748834, EPI_ISL_748835, EPI_ISL_748836, EPI_ISL_748837, EPI_ISL_748838, EPI_ISL_748839, EPI_ISL_748840, EPI_ISL_748841, EPI_ISL_748842, EPI_ISL_748843, EPI_ISL_748844, EPI_ISL_748845, EPI_ISL_748846, EPI_ISL_748847, EPI_ISL_748848, EPI_ISL_748849, EPI_ISL_748850, EPI_ISL_748851, EPI_ISL_748852, EPI_ISL_748853, EPI_ISL_748854, EPI_ISL_748855, EPI_ISL_748856, EPI_ISL_748857, EPI_ISL_748858, EPI_ISL_748859, EPI_ISL_748860, EPI_ISL_748861, EPI_ISL_748862, EPI_ISL_748863, EPI_ISL_748864, EPI_ISL_748865, EPI_ISL_748866, EPI_ISL_748867, EPI_ISL_748868, EPI_ISL_748869, EPI_ISL_748870, EPI_ISL_748871, EPI_ISL_748872, EPI_ISL_748873, EPI_ISL_748874, EPI_ISL_748875, EPI_ISL_748876, EPI_ISL_748877, EPI_ISL_748878, EPI_ISL_748879, EPI_ISL_748880, EPI_ISL_748881, EPI_ISL_748882, EPI_ISL_748883, EPI_ISL_748884, EPI_ISL_748885, EPI_ISL_748886, EPI_ISL_748887, EPI_ISL_748888, EPI_ISL_748889, EPI_ISL_748890, EPI_ISL_748891, EPI_ISL_748892, EPI_ISL_748893, EPI_ISL_748894, EPI_ISL_748895, EPI_ISL_748896, EPI_ISL_748897, EPI_ISL_748898, EPI_ISL_748899, EPI_ISL_748900, EPI_ISL_748901, EPI_ISL_748902, EPI_ISL_748903, EPI_ISL_748904, EPI_ISL_748905, EPI_ISL_748906, EPI_ISL_748907, EPI_ISL_748908, EPI_ISL_748909, EPI_ISL_748910, EPI_ISL_748911, EPI_ISL_748912, EPI_ISL_748913, EPI_ISL_748914, EPI_ISL_748915, EPI_ISL_748916, EPI_ISL_748917, EPI_ISL_748918, EPI_ISL_748919, EPI_ISL_748920, EPI_ISL_748921, EPI_ISL_748922, EPI_ISL_748923, EPI_ISL_748924, EPI_ISL_748925, EPI_ISL_748926, EPI_ISL_748927, EPI_ISL_748928, EPI_ISL_748929, EPI_ISL_748930, EPI_ISL_748931, EPI_ISL_748932, EPI_ISL_748933, EPI_ISL_748934, EPI_ISL_748935, EPI_ISL_748936, EPI_ISL_748937, EPI_ISL_748938, EPI_ISL_748939, EPI_ISL_748940, EPI_ISL_748941, EPI_ISL_748942, EPI_ISL_748943, EPI_ISL_748944, EPI_ISL_748945, EPI_ISL_748946, EPI_ISL_748947, EPI_ISL_748948, EPI_ISL_748949, EPI_ISL_748950, EPI_ISL_748951, EPI_ISL_748952, EPI_ISL_748953, EPI_ISL_748954, EPI_ISL_748955, EPI_ISL_748956, EPI_ISL_748957, EPI_ISL_748958, EPI_ISL_748959, EPI_ISL_748960, EPI_ISL_748961, EPI_ISL_748962, EPI_ISL_748963, EPI_ISL_748964, EPI_ISL_748965, EPI_ISL_748966, EPI_ISL_748967, EPI_ISL_748968, EPI_ISL_748969, EPI_ISL_748970, EPI_ISL_748971, EPI_ISL_748972, EPI_ISL_748973, EPI_ISL_748974, EPI_ISL_748975, EPI_ISL_748976, EPI_ISL_748977, EPI_ISL_748978, EPI_ISL_748979, EPI_ISL_748980, EPI_ISL_748981, EPI_ISL_748982, EPI_ISL_748983, EPI_ISL_748984, EPI_ISL_748985, EPI_ISL_748986, EPI_ISL_748987, EPI_ISL_748988, EPI_ISL_748989, EPI_ISL_748990, EPI_ISL_748991, EPI_ISL_748992, EPI_ISL_748993, EPI_ISL_748994, EPI_ISL_748995, EPI_ISL_748996, EPI_ISL_748997, EPI_ISL_748998, EPI_ISL_748999, EPI_ISL_749000, EPI_ISL_749001, EPI_ISL_749002, EPI_ISL_749003, EPI_ISL_749004, EPI_ISL_749005, EPI_ISL_749006, EPI_ISL_749007, EPI_ISL_749008, EPI_ISL_749009, EPI_ISL_749010, EPI_ISL_749011, EPI_ISL_749012, EPI_ISL_749013, EPI_ISL_749014, EPI_ISL_749015, EPI_ISL_749016, EPI_ISL_749017, EPI_ISL_749018, EPI_ISL_749019, EPI_ISL_749020, EPI_ISL_749021, EPI_ISL_749022, EPI_ISL_749023, EPI_ISL_749024, EPI_ISL_749025, EPI_ISL_749026, EPI_ISL_749027, EPI_ISL_749028, EPI_ISL_749029, EPI_ISL_749030, EPI_ISL_749031, EPI_ISL_749032, EPI_ISL_749033, EPI_ISL_749034, EPI_ISL_749035, EPI_ISL_749037, EPI_ISL_749038, EPI_ISL_749039, EPI_ISL_749040, EPI_ISL_749041, EPI_ISL_749042, EPI_ISL_749043, EPI_ISL_749044, EPI_ISL_749045, EPI_ISL_749046, EPI_ISL_749047, EPI_ISL_749048, EPI_ISL_749049, EPI_ISL_749050, EPI_ISL_749051, EPI_ISL_749052, EPI_ISL_749053, EPI_ISL_749054, EPI_ISL_749055, EPI_ISL_749056, EPI_ISL_749057, EPI_ISL_749058, EPI_ISL_749059, EPI_ISL_749060, EPI_ISL_749061, EPI_ISL_749062, EPI_ISL_749063, EPI_ISL_749064, EPI_ISL_749065, EPI_ISL_749066, EPI_ISL_749067, EPI_ISL_749068, EPI_ISL_749069, EPI_ISL_749070, EPI_ISL_749071, EPI_ISL_749072, EPI_ISL_749073, EPI_ISL_749074, EPI_ISL_749075, EPI_ISL_749076, EPI_ISL_749077, EPI_ISL_749078, EPI_ISL_749079, EPI_ISL_749080, EPI_ISL_749081, EPI_ISL_749082, EPI_ISL_749083, EPI_ISL_749084, EPI_ISL_749085, EPI_ISL_749086, EPI_ISL_749087, EPI_ISL_749088, EPI_ISL_749089, EPI_ISL_749090, EPI_ISL_749091, EPI_ISL_749092, EPI_ISL_749093, EPI_ISL_749094, EPI_ISL_749095, EPI_ISL_749096, EPI_ISL_749097, EPI_ISL_749098, EPI_ISL_749099, EPI_ISL_749100, EPI_ISL_749101, EPI_ISL_749102, EPI_ISL_749103, EPI_ISL_749104, EPI_ISL_749105, EPI_ISL_749106, EPI_ISL_749107, EPI_ISL_749108, EPI_ISL_749109, EPI_ISL_749110, EPI_ISL_749111, EPI_ISL_749112, EPI_ISL_749113, EPI_ISL_749114, EPI_ISL_749115, EPI_ISL_749116, EPI_ISL_749117, EPI_ISL_749118, EPI_ISL_749119, EPI_ISL_749120, EPI_ISL_749121, EPI_ISL_749122, EPI_ISL_749123, EPI_ISL_749124, EPI_ISL_749125, EPI_ISL_749126, EPI_ISL_749127, EPI_ISL_749128, EPI_ISL_749129, EPI_ISL_749130, EPI_ISL_749131, EPI_ISL_749132, EPI_ISL_749133, EPI_ISL_749134, EPI_ISL_749135, EPI_ISL_749136, EPI_ISL_749137, EPI_ISL_749138, EPI_ISL_749139, EPI_ISL_749140, EPI_ISL_749141, EPI_ISL_749142, EPI_ISL_749143, EPI_ISL_749144, EPI_ISL_749145, EPI_ISL_749146, EPI_ISL_749147			
see above	Department of Virus and Microbiological Special Diagnostics, Statens Serum Institut, Copenhagen, Denmark	Albertsen Lab, Department of Chemistry and Bioscience, Aalborg University, Denmark	Danish Covid-19 Genome Consortium
EPI_ISL_749148, EPI_ISL_749151, EPI_ISL_749155	Sanatorio Americano	Institut Pasteur de Montevideo	Daiana Mir, Natalia Rego, Paola Cristina Resende, Fernando Lopez-Tort, Tamara Fernandez-Calero, Veronica Noya, Mariana Brandes, Tania Possi, Mailen Arleo, Natalia Reyes, Matias Victoria, Andres Lizasoain, Matias Castells, Leticia Maya, Matías Salvo, Tatiana Schäffer Gregianini, Marilda Tereza Mar da Rosa, Letícia Garay Martins, Cecilia Alonso, Yasser Vega, Cecilia Salazar, Ignacio Ferrés, Pablo Smircich, Jose Sotelo, Ighor Arantes, Luciana Appolinario, Ana Carolina Mendonça, Maria Jose Benitez-Galeano, Martín Graña, Camila Simoes, Fernando Motta, Marilda Mendonça Siqueira, Gonzalo Bello, Rodney Colina, Lucia Spangenberg
EPI_ISL_749156, EPI_ISL_749157, EPI_ISL_749158, EPI_ISL_749159, EPI_ISL_749160, EPI_ISL_749161, EPI_ISL_749162, EPI_ISL_749163, EPI_ISL_749164, EPI_ISL_749165, EPI_ISL_749166, EPI_ISL_749167, EPI_ISL_749168, EPI_ISL_749169, EPI_ISL_749170, EPI_ISL_749171, EPI_ISL_749172, EPI_ISL_749173, EPI_ISL_749174, EPI_ISL_749175, EPI_ISL_749176, EPI_ISL_749177, EPI_ISL_749178, EPI_ISL_749179, EPI_ISL_749180, EPI_ISL_749181, EPI_ISL_749182, EPI_ISL_749183, EPI_ISL_749184, EPI_ISL_749185, EPI_ISL_749186, EPI_ISL_749187, EPI_ISL_749188, EPI_ISL_749189, EPI_ISL_749190, EPI_ISL_749191, EPI_ISL_749192, EPI_ISL_749193, EPI_ISL_749194, EPI_ISL_749195, EPI_ISL_749196, EPI_ISL_749197, EPI_ISL_749198, EPI_ISL_749199, EPI_ISL_749200, EPI_ISL_749201, EPI_ISL_749202, EPI_ISL_749203, EPI_ISL_749204, EPI_ISL_749205, EPI_ISL_749206, EPI_ISL_749207, EPI_ISL_749208, EPI_ISL_749209, EPI_ISL_749210, EPI_ISL_749211, EPI_ISL_749212, EPI_ISL_749213, EPI_ISL_749214, EPI_ISL_749215, EPI_ISL_749216, EPI_ISL_749217, EPI_ISL_749218, EPI_ISL_749219, EPI_ISL_749220, EPI_ISL_749221, EPI_ISL_749222, EPI_ISL_749223, EPI_ISL_749224, EPI_ISL_749225, EPI_ISL_749226, EPI_ISL_749227, EPI_ISL_749228, EPI_ISL_749229, EPI_ISL_749230, EPI_ISL_749231, EPI_ISL_749232, EPI_ISL_749233, EPI_ISL_749234, EPI_ISL_749235, EPI_ISL_749236, EPI_ISL_749237			
see above	Department of Virus and Microbiological Special Diagnostics, Statens Serum Institut, Copenhagen, Denmark	Albertsen Lab, Department of Chemistry and Bioscience, Aalborg University, Denmark	Danish Covid-19 Genome Consortium
EPI_ISL_749238	Sanatorio Americano	Institut Pasteur de Montevideo	Daiana Mir, Natalia Rego, Paola Cristina Resende, Fernando Lopez-Tort, Tamara Fernandez-Calero, Veronica Noya, Mariana Brandes, Tania Possi, Mailen Arleo, Natalia Reyes, Matias Victoria, Andres Lizasoain, Matias Castells, Leticia Maya, Matías Salvo, Tatiana Schäffer Gregianini, Marilda Tereza Mar da Rosa, Letícia Garay Martins, Cecilia Alonso, Yasser Vega, Cecilia Salazar, Ignacio Ferrés, Pablo Smircich, Jose Sotelo, Ighor Arantes, Luciana Appolinario, Ana Carolina Mendonça, Maria Jose Benitez-Galeano, Martín Graña, Camila Simoes, Fernando Motta, Marilda Mendonça Siqueira, Gonzalo Bello, Rodney Colina, Lucia Spangenberg
EPI_ISL_749239, EPI_ISL_749240, EPI_ISL_749241, EPI_ISL_749242, EPI_ISL_749243, EPI_ISL_749244, EPI_ISL_749245, EPI_ISL_749246, EPI_ISL_749247, EPI_ISL_749248, EPI_ISL_749249, EPI_ISL_749250, EPI_ISL_749251, EPI_ISL_749252, EPI_ISL_749253, EPI_ISL_749254, EPI_ISL_749255, EPI_ISL_749256, EPI_ISL_749257, EPI_ISL_749258, EPI_ISL_749259, EPI_ISL_749260, EPI_ISL_749261, EPI_ISL_749262, EPI_ISL_749263, EPI_ISL_749264, EPI_ISL_749265, EPI_ISL_749266, EPI_ISL_749267, EPI_ISL_749268, EPI_ISL_749269, EPI_ISL_749270, EPI_ISL_749271, EPI_ISL_749272, EPI_ISL_749273, EPI_ISL_749274, EPI_ISL_749275, EPI_ISL_749276, EPI_ISL_749277, EPI_ISL_749278, EPI_ISL_749279, EPI_ISL_749280, EPI_ISL_749281, EPI_ISL_749282, EPI_ISL_749283, EPI_ISL_749284, EPI_ISL_749285, EPI_ISL_749287, EPI_ISL_749288, EPI_ISL_749289, EPI_ISL_749290, EPI_ISL_749291, EPI_ISL_749292, EPI_ISL_749293, EPI_ISL_749294, EPI_ISL_749295, EPI_ISL_749296, EPI_ISL_749297, EPI_ISL_749298, EPI_ISL_749299, EPI_ISL_749300, EPI_ISL_749301, EPI_ISL_749302, EPI_ISL_749303, EPI_ISL_749304, EPI_ISL_749305, EPI_ISL_749306, EPI_ISL_749307, EPI_ISL_749308, EPI_ISL_749309, EPI_ISL_749310, EPI_ISL_749311, EPI_ISL_749312, EPI_ISL_749313, EPI_ISL_749314, EPI_ISL_749315, EPI_ISL_749316, EPI_ISL_749317, EPI_ISL_749318, EPI_ISL_749319, EPI_ISL_749320, EPI_ISL_749321, EPI_ISL_749322, EPI_ISL_749323, EPI_ISL_749324, EPI_ISL_749325, EPI_ISL_749326, EPI_ISL_749327, EPI_ISL_749328, EPI_ISL_749329, EPI_ISL_749330, EPI_ISL_749331, EPI_ISL_749332, EPI_ISL_749333, EPI_ISL_749334, EPI_ISL_749335, EPI_ISL_749336, EPI_ISL_749337, EPI_ISL_749338, EPI_ISL_749339, EPI_ISL_749340, EPI_ISL_749341, EPI_ISL_749342, EPI_ISL_749343, EPI_ISL_749344, EPI_ISL_749345, EPI_ISL_749346, EPI_ISL_749347, EPI_ISL_749348, EPI_ISL_749349, EPI_ISL_749350, EPI_ISL_749351, EPI_ISL_749352, EPI_ISL_749353, EPI_ISL_749354, EPI_ISL_749355, EPI_ISL_749356, EPI_ISL_749357, EPI_ISL_749378, EPI_ISL_749379, EPI_ISL_749380, EPI_ISL_749381, EPI_ISL_749382, EPI_ISL_749383, EPI_ISL_749384, EPI_ISL_749385, EPI_ISL_749386, EPI_ISL_749387, EPI_ISL_749388, EPI_ISL_749389, EPI_ISL_749390, EPI_ISL_749391, EPI_ISL_749392, EPI_ISL_749393, EPI_ISL_749394, EPI_ISL_749395, EPI_ISL_749396, EPI_ISL_749397, EPI_ISL_749398, EPI_ISL_749399, EPI_ISL_749400, EPI_ISL_749401, EPI_ISL_749402, EPI_ISL_749403, EPI_ISL_749404, EPI_ISL_749405, EPI_ISL_749406, EPI_ISL_749407, EPI_ISL_749408, EPI_ISL_749409, EPI_ISL_749410, EPI_ISL_749411, EPI_ISL_749412, EPI_ISL_749413, EPI_ISL_749414, EPI_ISL_749415, EPI_ISL_749416, EPI_ISL_749417, EPI_ISL_749418, EPI_ISL_749419, EPI_ISL_749420, EPI_ISL_749421, EPI_ISL_749422, EPI_ISL_749423, EPI_ISL_749424, EPI_ISL_749425, EPI_ISL_749426, EPI_ISL_749427, EPI_ISL_749428, EPI_ISL_749429, EPI_ISL_749430, EPI_ISL_749431, EPI_ISL_749432, EPI_ISL_749433, EPI_ISL_749434, EPI_ISL_749435, EPI_ISL_749436, EPI_ISL_749437, EPI_ISL_749438, EPI_ISL_749439, EPI_ISL_749440, EPI_ISL_749441, EPI_ISL_749442, EPI_ISL_749443, EPI_ISL_749444, EPI_ISL_749445, EPI_ISL_749446, EPI_ISL_749447, EPI_ISL_749448, EPI_ISL_749449, EPI_ISL_749450, EPI_ISL_749451, EPI_ISL_749452, EPI_ISL_749453, EPI_ISL_749454, EPI_ISL_749455, EPI_ISL_749456, EPI_ISL_749457, EPI_ISL_749458, EPI_ISL_749459, EPI_ISL_749460, EPI_ISL_749461, EPI_ISL_749462, EPI_ISL_749463, EPI_ISL_749464, EPI_ISL_749465, EPI_ISL_749466, EPI_ISL_749467, EPI_ISL_749468, EPI_ISL_749469, EPI_ISL_749470, EPI_ISL_749471, EPI_ISL_749472, EPI_ISL_749473			
see above	Department of Virus and Microbiological Special Diagnostics, Statens Serum Institut, Copenhagen, Denmark	Albertsen Lab, Department of Chemistry and Bioscience, Aalborg University, Denmark	Danish Covid-19 Genome Consortium
EPI_ISL_749474	Sanatorio Americano	Institut Pasteur de Montevideo	Daiana Mir, Natalia Rego, Paola Cristina Resende, Fernando Lopez-Tort, Tamara Fernandez-Calero, Veronica Noya, Mariana Brandes, Tania Possi, Mailen Arleo, Natalia Reyes, Matias Victoria, Andres Lizasoain, Matias Castells, Leticia Maya, Matías Salvo, Tatiana Schäffer Gregianini, Marilda Tereza Mar da Rosa, Letícia Garay Martins, Cecilia Alonso, Yasser Vega, Cecilia Salazar, Ignacio Ferrés, Pablo Smircich, Jose Sotelo, Ighor Arantes, Luciana Appolinario, Ana Carolina Mendonça, Maria Jose Benitez-Galeano, Martín Graña, Camila Simoes, Fernando Motta, Marilda Mendonça Siqueira, Gonzalo Bello, Rodney Colina, Lucia Spangenberg

EPI_ISL_749475, EPI_ISL_749476, EPI_ISL_749477, EPI_ISL_749478, EPI_ISL_749479, EPI_ISL_749480, EPI_ISL_749481, EPI_ISL_749482, EPI_ISL_749483, EPI_ISL_749484, EPI_ISL_749485, EPI_ISL_749486, EPI_ISL_749487, EPI_ISL_749488, EPI_ISL_749489, EPI_ISL_749490, EPI_ISL_749491, EPI_ISL_749492, EPI_ISL_749493, EPI_ISL_749494, EPI_ISL_749495, EPI_ISL_749496, EPI_ISL_749497, EPI_ISL_749498, EPI_ISL_749499, EPI_ISL_749500, EPI_ISL_749501, EPI_ISL_749502, EPI_ISL_749503, EPI_ISL_749504, EPI_ISL_749505, EPI_ISL_749506, EPI_ISL_749507, EPI_ISL_749508, EPI_ISL_749509, EPI_ISL_749510, EPI_ISL_749511, EPI_ISL_749512, EPI_ISL_749513, EPI_ISL_749514, EPI_ISL_749519, EPI_ISL_749520, EPI_ISL_749521, EPI_ISL_749522, EPI_ISL_749523, EPI_ISL_749524, EPI_ISL_749525, EPI_ISL_749526, EPI_ISL_749527, EPI_ISL_749528, EPI_ISL_749529, EPI_ISL_749530, EPI_ISL_749531, EPI_ISL_749532, EPI_ISL_749533, EPI_ISL_749534, EPI_ISL_749535, EPI_ISL_749536, EPI_ISL_749537, EPI_ISL_749538, EPI_ISL_749539, EPI_ISL_749540, EPI_ISL_749541, EPI_ISL_749542, EPI_ISL_749543, EPI_ISL_749544, EPI_ISL_749545, EPI_ISL_749546, EPI_ISL_749547, EPI_ISL_749548, EPI_ISL_749549, EPI_ISL_749550, EPI_ISL_749551, EPI_ISL_749552, EPI_ISL_749553, EPI_ISL_749554, EPI_ISL_749555, EPI_ISL_749556, EPI_ISL_749557, EPI_ISL_749558, EPI_ISL_749559, EPI_ISL_749560, EPI_ISL_749561, EPI_ISL_749562, EPI_ISL_749563, EPI_ISL_749564, EPI_ISL_749565, EPI_ISL_749566, EPI_ISL_749567, EPI_ISL_749568, EPI_ISL_749569, EPI_ISL_749570, EPI_ISL_749571, EPI_ISL_749572, EPI_ISL_749573, EPI_ISL_749574, EPI_ISL_749575, EPI_ISL_749576, EPI_ISL_749577, EPI_ISL_749578, EPI_ISL_749579, EPI_ISL_749580, EPI_ISL_749581, EPI_ISL_749582, EPI_ISL_749583, EPI_ISL_749584, EPI_ISL_749585, EPI_ISL_749586, EPI_ISL_749587, EPI_ISL_749588, EPI_ISL_749589, EPI_ISL_749590, EPI_ISL_749591, EPI_ISL_749592, EPI_ISL_749593, EPI_ISL_749594, EPI_ISL_749595, EPI_ISL_749596, EPI_ISL_749597, EPI_ISL_749598, EPI_ISL_749599, EPI_ISL_749600, EPI_ISL_749601, EPI_ISL_749602, EPI_ISL_749603, EPI_ISL_749604, EPI_ISL_749605, EPI_ISL_749606, EPI_ISL_749607, EPI_ISL_749608, EPI_ISL_749609, EPI_ISL_749610, EPI_ISL_749611, EPI_ISL_749612, EPI_ISL_749613, EPI_ISL_749614, EPI_ISL_749615, EPI_ISL_749616, EPI_ISL_749617, EPI_ISL_749618, EPI_ISL_749619, EPI_ISL_749620, EPI_ISL_749621, EPI_ISL_749622, EPI_ISL_749623, EPI_ISL_749624, EPI_ISL_749625, EPI_ISL_749626, EPI_ISL_749627, EPI_ISL_749628, EPI_ISL_749629, EPI_ISL_749630, EPI_ISL_749631, EPI_ISL_749632, EPI_ISL_749633, EPI_ISL_749634, EPI_ISL_749635, EPI_ISL_749636, EPI_ISL_749637, EPI_ISL_749638, EPI_ISL_749639, EPI_ISL_749640, EPI_ISL_749641, EPI_ISL_749642, EPI_ISL_749643, EPI_ISL_749644, EPI_ISL_749645, EPI_ISL_749646, EPI_ISL_749647, EPI_ISL_749648, EPI_ISL_749649, EPI_ISL_749650, EPI_ISL_749651, EPI_ISL_749652, EPI_ISL_749653, EPI_ISL_749654, EPI_ISL_749655, EPI_ISL_749656, EPI_ISL_749657, EPI_ISL_749658, EPI_ISL_749659, EPI_ISL_749660, EPI_ISL_749661, EPI_ISL_749662, EPI_ISL_749663, EPI_ISL_749664, EPI_ISL_749665, EPI_ISL_749666, EPI_ISL_749667, EPI_ISL_749668, EPI_ISL_749669, EPI_ISL_749670, EPI_ISL_749671, EPI_ISL_749672, EPI_ISL_749673, EPI_ISL_749674, EPI_ISL_749675, EPI_ISL_749676, EPI_ISL_749677, EPI_ISL_749678, EPI_ISL_749679, EPI_ISL_749680, EPI_ISL_749681, EPI_ISL_749682, EPI_ISL_749683, EPI_ISL_749684, EPI_ISL_749685, EPI_ISL_749686, EPI_ISL_749687, EPI_ISL_749688, EPI_ISL_749689, EPI_ISL_749690, EPI_ISL_749691, EPI_ISL_749692, EPI_ISL_749693, EPI_ISL_749694, EPI_ISL_749695, EPI_ISL_749696, EPI_ISL_749697, EPI_ISL_749698, EPI_ISL_749699, EPI_ISL_749700, EPI_ISL_749701, EPI_ISL_749702, EPI_ISL_749703, EPI_ISL_749704, EPI_ISL_749705, EPI_ISL_749707, EPI_ISL_749708, EPI_ISL_749709, EPI_ISL_749710, EPI_ISL_749711, EPI_ISL_749712, EPI_ISL_749713, EPI_ISL_749714, EPI_ISL_749715, EPI_ISL_749716, EPI_ISL_749717, EPI_ISL_749718, EPI_ISL_749719, EPI_ISL_749720, EPI_ISL_749721, EPI_ISL_749722, EPI_ISL_749723, EPI_ISL_749724, EPI_ISL_749725, EPI_ISL_749726, EPI_ISL_749727, EPI_ISL_749728, EPI_ISL_749729, EPI_ISL_749730, EPI_ISL_749731, EPI_ISL_749732, EPI_ISL_749733, EPI_ISL_749734, EPI_ISL_749735, EPI_ISL_749736, EPI_ISL_749737, EPI_ISL_749738, EPI_ISL_749739, EPI_ISL_749740, EPI_ISL_749741, EPI_ISL_749742, EPI_ISL_749743, EPI_ISL_749744, EPI_ISL_749745, EPI_ISL_749746, EPI_ISL_749747, EPI_ISL_749748, EPI_ISL_749749, EPI_ISL_749750, EPI_ISL_749751, EPI_ISL_749752, EPI_ISL_749753, EPI_ISL_749754, EPI_ISL_749755, EPI_ISL_749756, EPI_ISL_749757, EPI_ISL_749758, EPI_ISL_749759, EPI_ISL_749760, EPI_ISL_749761, EPI_ISL_749762, EPI_ISL_749763, EPI_ISL_749764, EPI_ISL_749765, EPI_ISL_749766, EPI_ISL_749767, EPI_ISL_749768, EPI_ISL_749769, EPI_ISL_749770, EPI_ISL_749771, EPI_ISL_749772, EPI_ISL_749773, EPI_ISL_749774, EPI_ISL_749775, EPI_ISL_749776, EPI_ISL_749777, EPI_ISL_749778, EPI_ISL_749779, EPI_ISL_749780, EPI_ISL_749781, EPI_ISL_749782, EPI_ISL_749783, EPI_ISL_749784, EPI_ISL_749785, EPI_ISL_749786, EPI_ISL_749787, EPI_ISL_749788, EPI_ISL_749789, EPI_ISL_749790, EPI_ISL_749791, EPI_ISL_749792, EPI_ISL_749793, EPI_ISL_749794, EPI_ISL_749795, EPI_ISL_749796, EPI_ISL_749797, EPI_ISL_749798, EPI_ISL_749799, EPI_ISL_749800, EPI_ISL_749801, EPI_ISL_749802, EPI_ISL_749803, EPI_ISL_749804, EPI_ISL_749805, EPI_ISL_749806, EPI_ISL_749807, EPI_ISL_749808, EPI_ISL_749809, EPI_ISL_749810, EPI_ISL_749811, EPI_ISL_749812, EPI_ISL_749813, EPI_ISL_749814, EPI_ISL_749815, EPI_ISL_749816, EPI_ISL_749817, EPI_ISL_749818, EPI_ISL_749819, EPI_ISL_749820, EPI_ISL_749821, EPI_ISL_749822, EPI_ISL_749823, EPI_ISL_749824, EPI_ISL_749825, EPI_ISL_749826, EPI_ISL_749827, EPI_ISL_749828, EPI_ISL_749829, EPI_ISL_749830, EPI_ISL_749831, EPI_ISL_749832, EPI_ISL_749833, EPI_ISL_749834, EPI_ISL_749835, EPI_ISL_749836, EPI_ISL_749837, EPI_ISL_749838, EPI_ISL_749839, EPI_ISL_749840, EPI_ISL_749841, EPI_ISL_749842, EPI_ISL_749843, EPI_ISL_749844, EPI_ISL_749845, EPI_ISL_749846, EPI_ISL_749847, EPI_ISL_749848, EPI_ISL_749849, EPI_ISL_749850, EPI_ISL_749851, EPI_ISL_749852, EPI_ISL_749853, EPI_ISL_749854, EPI_ISL_749855, EPI_ISL_749856, EPI_ISL_749857, EPI_ISL_749858, EPI_ISL_749859, EPI_ISL_749860, EPI_ISL_749861, EPI_ISL_749862, EPI_ISL_749863, EPI_ISL_749864, EPI_ISL_749865, EPI_ISL_749866, EPI_ISL_749867, EPI_ISL_749868, EPI_ISL_749869, EPI_ISL_749870, EPI_ISL_749871, EPI_ISL_749872, EPI_ISL_749873, EPI_ISL_749874, EPI_ISL_749875, EPI_ISL_749876, EPI_ISL_749877, EPI_ISL_749878, EPI_ISL_749879, EPI_ISL_749880, EPI_ISL_749881, EPI_ISL_749882, EPI_ISL_749883, EPI_ISL_749884, EPI_ISL_749885, EPI_ISL_749886, EPI_ISL_749887, EPI_ISL_749888, EPI_ISL_749889, EPI_ISL_749890, EPI_ISL_749891, EPI_ISL_749892, EPI_ISL_749893, EPI_ISL_749894, EPI_ISL_749895, EPI_ISL_749896, EPI_ISL_749897, EPI_ISL_749898, EPI_ISL_749899, EPI_ISL_749900, EPI_ISL_749901, EPI_ISL_749902, EPI_ISL_749903, EPI_ISL_749904, EPI_ISL_749905			
see above	Department of Virus and Microbiological Special Diagnostics, Statens Serum Institut, Copenhagen, Denmark	Albertsen Lab, Department of Chemistry and Bioscience, Aalborg University, Denmark	Danish Covid-19 Genome Consortium
EPI_ISL_749906	Sanatorio Americano	Institut Pasteur de Montevideo	Daiana Mir, Natalia Rego, Paola Cristina Resende, Fernando Lopez-Tort, Tamara Fernandez-Calero, Veronica Noya, Mariana Brandes, Tania Possi, Mailen Arleo, Natalia Reyes, Matias Victoria, Andres Lizasoain, Matias Castells, Leticia Maya, Matías Salvo, Tatiana Schäffer Gregianini, Marilda Tereza Mar da Rosa, Letícia Garay Martins, Cecilia Alonso, Yasser Vega, Cecilia Salazar, Ignacio Ferrés, Pablo Smircich, Jose Sotelo, Ighor Arantes, Luciana Appolinario, Ana Carolina Mendonça, Maria Jose Benitez-Galeano, Martín Graña, Camila Simoes, Fernando Motta, Marilda Mendonça Siqueira, Gonzalo Bello, Rodney Colina, Lucia Spangenberg
EPI_ISL_749907, EPI_ISL_749908, EPI_ISL_749909, EPI_ISL_749910, EPI_ISL_749911, EPI_ISL_749912, EPI_ISL_749913, EPI_ISL_749914, EPI_ISL_749915, EPI_ISL_749916, EPI_ISL_749917, EPI_ISL_749918, EPI_ISL_749919, EPI_ISL_749920, EPI_ISL_749921, EPI_ISL_749922, EPI_ISL_749923, EPI_ISL_749924, EPI_ISL_749925, EPI_ISL_749926, EPI_ISL_749927, EPI_ISL_749928, EPI_ISL_749929, EPI_ISL_749930, EPI_ISL_749931, EPI_ISL_749932, EPI_ISL_749933, EPI_ISL_749934, EPI_ISL_749935, EPI_ISL_749936, EPI_ISL_749937, EPI_ISL_749938, EPI_ISL_749939, EPI_ISL_749940, EPI_ISL_749941, EPI_ISL_749942, EPI_ISL_749943, EPI_ISL_749944, EPI_ISL_749945, EPI_ISL_749946, EPI_ISL_749947, EPI_ISL_749948, EPI_ISL_749949, EPI_ISL_749950, EPI_ISL_749951, EPI_ISL_749952, EPI_ISL_749953, EPI_ISL_749954, EPI_ISL_749955, EPI_ISL_749956, EPI_ISL_749957, EPI_ISL_749958, EPI_ISL_749959, EPI_ISL_749960, EPI_ISL_749961, EPI_ISL_749962, EPI_ISL_749963, EPI_ISL_749964, EPI_ISL_749965, EPI_ISL_749966, EPI_ISL_749967, EPI_ISL_749968, EPI_ISL_749969, EPI_ISL_749970, EPI_ISL_749971, EPI_ISL_749972, EPI_ISL_749973, EPI_ISL_749974, EPI_ISL_749975, EPI_ISL_749976, EPI_ISL_749977, EPI_ISL_749978, EPI_ISL_749979, EPI_ISL_749980, EPI_ISL_749981, EPI_ISL_749982, EPI_ISL_749983, EPI_ISL_749984, EPI_ISL_749985, EPI_ISL_749986, EPI_ISL_749987, EPI_ISL_749988, EPI_ISL_749989, EPI_ISL_749990, EPI_ISL_749991, EPI_ISL_749992, EPI_ISL_749993, EPI_ISL_749994, EPI_ISL_749995, EPI_ISL_749996, EPI_ISL_749997, EPI_ISL_749998, EPI_ISL_749999, EPI_ISL_750000, EPI_ISL_750001, EPI_ISL_750002, EPI_ISL_750003, EPI_ISL_750004, EPI_ISL_750005, EPI_ISL_750006, EPI_ISL_750007, EPI_ISL_750008, EPI_ISL_750009, EPI_ISL_750010, EPI_ISL_750011, EPI_ISL_750012, EPI_ISL_750013, EPI_ISL_750014, EPI_ISL_750015, EPI_ISL_750016, EPI_ISL_750017, EPI_ISL_750018, EPI_ISL_750019, EPI_ISL_750020, EPI_ISL_750021, EPI_ISL_750022, EPI_ISL_750023, EPI_ISL_750024, EPI_ISL_750025, EPI_ISL_750026, EPI_ISL_750027, EPI_ISL_750028, EPI_ISL_750029, EPI_ISL_750030, EPI_ISL_750031, EPI_ISL_750032, EPI_ISL_750033, EPI_ISL_750034, EPI_ISL_750035, EPI_ISL_750036, EPI_ISL_750037, EPI_ISL_750038, EPI_ISL_750039, EPI_ISL_750040, EPI_ISL_750041, EPI_ISL_750042, EPI_ISL_750043, EPI_ISL_750044, EPI_ISL_750045, EPI_ISL_750046, EPI_ISL_750047, EPI_ISL_750048, EPI_ISL_750049, EPI_ISL_750050, EPI_ISL_750051, EPI_ISL_750052, EPI_ISL_750053, EPI_ISL_750054, EPI_ISL_750055, EPI_ISL_750056, EPI_ISL_750057, EPI_ISL_750058, EPI_ISL_750059, EPI_ISL_750060, EPI_ISL_750061, EPI_ISL_750062, EPI_ISL_750063, EPI_ISL_750064, EPI_ISL_750065, EPI_ISL_750066, EPI_ISL_750067, EPI_ISL_750068, EPI_ISL_750069, EPI_ISL_750070, EPI_ISL_750071, EPI_ISL_750072, EPI_ISL_750073, EPI_ISL_750074, EPI_ISL_750075, EPI_ISL_750076, EPI_ISL_750077, EPI_ISL_750078, EPI_ISL_750079, EPI_ISL_750080, EPI_ISL_750081, EPI_ISL_750082, EPI_ISL_750083, EPI_ISL_750084, EPI_ISL_750085, EPI_ISL_750086, EPI_ISL_750087, EPI_ISL_750088, EPI_ISL_750089, EPI_ISL_750090, EPI_ISL_750091, EPI_ISL_750092, EPI_ISL_750093, EPI_ISL_750094, EPI_ISL_750095, EPI_ISL_750096, EPI_ISL_750097, EPI_ISL_750098, EPI_ISL_750099, EPI_ISL_750100, EPI_ISL_750101, EPI_ISL_750102, EPI_ISL_750103, EPI_ISL_750104, EPI_ISL_750105, EPI_ISL_750106, EPI_ISL_750107			
see above	Department of Virus and Microbiological Special Diagnostics, Statens Serum Institut, Copenhagen, Denmark	Albertsen Lab, Department of Chemistry and Bioscience, Aalborg University, Denmark	Danish Covid-19 Genome Consortium
EPI_ISL_750108	Sanatorio Americano	Institut Pasteur de Montevideo	Daiana Mir, Natalia Rego, Paola Cristina Resende, Fernando Lopez-Tort, Tamara Fernandez-Calero, Veronica Noya, Mariana Brandes, Tania Possi, Mailen Arleo, Natalia Reyes, Matias Victoria, Andres Lizasoain, Matias Castells, Leticia Maya, Matías Salvo, Tatiana Schäffer Gregianini, Marilda Tereza Mar da Rosa, Letícia Garay Martins, Cecilia Alonso, Yasser Vega, Cecilia Salazar, Ignacio Ferrés, Pablo Smircich, Jose Sotelo, Ighor Arantes, Luciana Appolinario, Ana Carolina Mendonça, Maria Jose Benitez-Galeano, Martín Graña, Camila Simoes, Fernando Motta, Marilda Mendonça Siqueira, Gonzalo Bello, Rodney Colina, Lucia Spangenberg
EPI_ISL_750109, EPI_ISL_750110, EPI_ISL_750111, EPI_ISL_750112, EPI_ISL_750113, EPI_ISL_750114, EPI_ISL_750115, EPI_ISL_750116, EPI_ISL_750117, EPI_ISL_750118, EPI_ISL_750119, EPI_ISL_750120, EPI_ISL_750121, EPI_ISL_750122, EPI_ISL_750123, EPI_ISL_750124, EPI_ISL_750125, EPI_ISL_750126, EPI_ISL_750127, EPI_ISL_750128, EPI_ISL_750129, EPI_ISL_750130, EPI_ISL_750131, EPI_ISL_750132, EPI_ISL_750133, EPI_ISL_750134, EPI_ISL_750135, EPI_ISL_750136, EPI_ISL_750137, EPI_ISL_750138, EPI_ISL_750139, EPI_ISL_750140, EPI_ISL_750141, EPI_ISL_750142, EPI_ISL_750143, EPI_ISL_750144, EPI_ISL_750145, EPI_ISL_750146, EPI_ISL_750147, EPI_ISL_750148, EPI_ISL_750149, EPI_ISL_750150, EPI_ISL_750151, EPI_ISL_750152, EPI_ISL_750153, EPI_ISL_750154, EPI_ISL_750155, EPI_ISL_750156, EPI_ISL_750157, EPI_ISL_750158, EPI_ISL_750159, EPI_ISL_750160			
see above	Department of Virus and Microbiological Special Diagnostics, Statens Serum Institut, Copenhagen, Denmark	Albertsen Lab, Department of Chemistry and Bioscience, Aalborg University, Denmark	Danish Covid-19 Genome Consortium
EPI_ISL_750161, EPI_ISL_750162, EPI_ISL_750168, EPI_ISL_750169, EPI_ISL_750170, EPI_ISL_750171, EPI_ISL_750172, EPI_ISL_750173, EPI_ISL_750174	Sanatorio Americano	Institut Pasteur de Montevideo	Daiana Mir, Natalia Rego, Paola Cristina Resende, Fernando Lopez-Tort, Tamara Fernandez-Calero, Veronica Noya, Mariana Brandes, Tania Possi, Mailen Arleo, Natalia Reyes, Matias Victoria, Andres Lizasoain, Matias Castells, Leticia Maya, Matías Salvo, Tatiana Schäffer Gregianini, Marilda Tereza Mar da Rosa, Letícia Garay Martins, Cecilia Alonso, Yasser Vega, Cecilia Salazar, Ignacio Ferrés, Pablo Smircich, Jose Sotelo, Ighor Arantes, Luciana Appolinario, Ana Carolina Mendonça, Maria Jose Benitez-Galeano, Martín Graña, Camila Simoes, Fernando Motta, Marilda Mendonça Siqueira, Gonzalo Bello, Rodney Colina, Lucia Spangenberg
EPI_ISL_750175	CENUR Este-Sede Rocha-UdelaR	Institut Pasteur de Montevideo	Daiana Mir, Natalia Rego, Paola Cristina Resende, Fernando Lopez-Tort, Tamara Fernandez-Calero, Veronica Noya, Mariana Brandes, Tania Possi, Mailen Arleo, Natalia Reyes, Matias Victoria, Andres Lizasoain, Matias Castells, Leticia Maya, Matías Salvo, Tatiana Schäffer Gregianini, Marilda Tereza Mar da Rosa, Letícia Garay Martins, Cecilia Alonso, Yasser Vega, Cecilia Salazar, Ignacio Ferrés, Pablo Smircich, Jose Sotelo, Ighor Arantes, Luciana Appolinario, Ana Carolina Mendonça, Maria Jose Benitez-Galeano, Martín Graña, Camila Simoes, Fernando Motta, Marilda Mendonça Siqueira, Gonzalo Bello, Rodney Colina, Lucia Spangenberg
EPI_ISL_750176, EPI_ISL_750177, EPI_ISL_750178	Sanatorio Americano	Institut Pasteur de Montevideo	Daiana Mir, Natalia Rego, Paola Cristina Resende, Fernando Lopez-Tort, Tamara Fernandez-Calero, Veronica Noya, Mariana Brandes, Tania Possi, Mailen Arleo, Natalia Reyes, Matias Victoria, Andres Lizasoain, Matias Castells, Leticia Maya, Matías Salvo, Tatiana Schäffer Gregianini, Marilda Tereza Mar da Rosa, Letícia Garay Martins, Cecilia Alonso, Yasser Vega, Cecilia Salazar, Ignacio Ferrés, Pablo Smircich, Jose Sotelo, Ighor Arantes, Luciana Appolinario, Ana Carolina Mendonça, Maria Jose Benitez-Galeano, Martín Graña, Camila Simoes, Fernando Motta, Marilda Mendonça Siqueira, Gonzalo Bello, Rodney Colina, Lucia Spangenberg
EPI_ISL_750180, EPI_ISL_750181, EPI_ISL_750182, EPI_ISL_750183, EPI_ISL_750184, EPI_ISL_750185, EPI_ISL_750186, EPI_ISL_750189, EPI_ISL_750190, EPI_ISL_750191, EPI_ISL_750192, EPI_ISL_750193, EPI_ISL_750194, EPI_ISL_750195, EPI_ISL_750196, EPI_ISL_750197, EPI_ISL_750198, EPI_ISL_750199, EPI_ISL_750200, EPI_ISL_750201, EPI_ISL_750202, EPI_ISL_750203, EPI_ISL_750204, EPI_ISL_750205, EPI_ISL_750206, EPI_ISL_750207, EPI_ISL_750208, EPI_ISL_750209, EPI_ISL_750210, EPI_ISL_750211, EPI_ISL_750212, EPI_ISL_750213, EPI_ISL_750214, EPI_ISL_750215, EPI_ISL_750216, EPI_ISL_750217, EPI_ISL_750218, EPI_ISL_750219, EPI_ISL_750220, EPI_ISL_750221, EPI_ISL_750222, EPI_ISL_750223, EPI_ISL_750224, EPI_ISL_750225, EPI_ISL_750226, EPI_ISL_750227, EPI_ISL_750228, EPI_ISL_750229, EPI_ISL_750230, EPI_ISL_750231, EPI_ISL_750232, EPI_ISL_750233, EPI_ISL_750234, EPI_ISL_750235, EPI_ISL_750236, EPI_ISL_750237, EPI_ISL_750238, EPI_ISL_750239, EPI_ISL_750240, EPI_ISL_750241, EPI_ISL_750242, EPI_ISL_750243, EPI_ISL_750244, EPI_ISL_750245, EPI_ISL_750246, EPI_ISL_750247, EPI_ISL_750248, EPI_ISL_750249, EPI_ISL_750250, EPI_ISL_750251, EPI_ISL_750252, EPI_ISL_750253, EPI_ISL_750254, EPI_ISL_750255			
see above	Department of Virus and Microbiological Special Diagnostics, Statens Serum Institut, Copenhagen, Denmark	Albertsen Lab, Department of Chemistry and Bioscience, Aalborg University, Denmark	Danish Covid-19 Genome Consortium

EPI_ISL_750256	Sanatorio Americano	Institut Pasteur de Montevideo	Daiana Mir, Natalia Rego, Paola Cristina Resende, Fernando Lopez-Tort, Tamara Fernandez-Calero, Veronica Noya, Mariana Brandes, Tania Possi, Mailen Arleo, Natalia Reyes, Matias Victoria, Andres Lizasoain, Matias Castells, Leticia Maya, Matías Salvo, Tatiana Schäffer Gregianini, Marilda Tereza Mar da Rosa, Letícia Garay Martins, Cecilia Alonso, Yasser Vega, Cecilia Salazar, Ignacio Ferrés, Pablo Smircich, Jose Sotelo, Ighor Arantes, Luciana Appolinario, Ana Carolina Mendonça, Maria Jose Benitez-Galeano, Martín Graña, Camila Simoes, Fernando Motta, Marilda Mendonça Siqueira, Gonzalo Bello, Rodney Colina, Lucia Spangenberg
EPI_ISL_750257, EPI_ISL_750258, EPI_ISL_750259, EPI_ISL_750260, EPI_ISL_750261, EPI_ISL_750262, EPI_ISL_750263, EPI_ISL_750264, EPI_ISL_750265, EPI_ISL_750266, EPI_ISL_750267, EPI_ISL_750268, EPI_ISL_750269, EPI_ISL_750270, EPI_ISL_750271, EPI_ISL_750272, EPI_ISL_750273, EPI_ISL_750274, EPI_ISL_750275, EPI_ISL_750276, EPI_ISL_750277, EPI_ISL_750278, EPI_ISL_750279, EPI_ISL_750280, EPI_ISL_750281, EPI_ISL_750282, EPI_ISL_750283, EPI_ISL_750284, EPI_ISL_750287, EPI_ISL_750288, EPI_ISL_750289, EPI_ISL_750290, EPI_ISL_750291, EPI_ISL_750292, EPI_ISL_750293, EPI_ISL_750294, EPI_ISL_750295, EPI_ISL_750296, EPI_ISL_750297, EPI_ISL_750298, EPI_ISL_750299, EPI_ISL_750300, EPI_ISL_750301, EPI_ISL_750302, EPI_ISL_750303, EPI_ISL_750304, EPI_ISL_750305, EPI_ISL_750306, EPI_ISL_750307, EPI_ISL_750308, EPI_ISL_750309, EPI_ISL_750310, EPI_ISL_750311, EPI_ISL_750312, EPI_ISL_750313, EPI_ISL_750314, EPI_ISL_750315, EPI_ISL_750316, EPI_ISL_750317, EPI_ISL_750318, EPI_ISL_750319, EPI_ISL_750320, EPI_ISL_750321, EPI_ISL_750322, EPI_ISL_750323, EPI_ISL_750324, EPI_ISL_750325, EPI_ISL_750326, EPI_ISL_750327, EPI_ISL_750328, EPI_ISL_750329, EPI_ISL_750330, EPI_ISL_750331, EPI_ISL_750332, EPI_ISL_750333, EPI_ISL_750334, EPI_ISL_750335, EPI_ISL_750336, EPI_ISL_750337, EPI_ISL_750338, EPI_ISL_750339, EPI_ISL_750340, EPI_ISL_750341, EPI_ISL_750342, EPI_ISL_750343, EPI_ISL_750344, EPI_ISL_750345, EPI_ISL_750346, EPI_ISL_750347, EPI_ISL_750348, EPI_ISL_750349, EPI_ISL_750350, EPI_ISL_750351, EPI_ISL_750352, EPI_ISL_750353, EPI_ISL_750354, EPI_ISL_750355, EPI_ISL_750356, EPI_ISL_750357, EPI_ISL_750358, EPI_ISL_750359, EPI_ISL_750360, EPI_ISL_750361, EPI_ISL_750362, EPI_ISL_750363, EPI_ISL_750364, EPI_ISL_750365, EPI_ISL_750366, EPI_ISL_750367, EPI_ISL_750368, EPI_ISL_750369, EPI_ISL_750370, EPI_ISL_750371, EPI_ISL_750372, EPI_ISL_750392, EPI_ISL_750393, EPI_ISL_750394, EPI_ISL_750395, EPI_ISL_750396, EPI_ISL_750397, EPI_ISL_750398, EPI_ISL_750399, EPI_ISL_750400, EPI_ISL_750401, EPI_ISL_750402, EPI_ISL_750403, EPI_ISL_750404, EPI_ISL_750405, EPI_ISL_750406, EPI_ISL_750407, EPI_ISL_750408, EPI_ISL_750409, EPI_ISL_750410, EPI_ISL_750411, EPI_ISL_750412, EPI_ISL_750413, EPI_ISL_750414, EPI_ISL_750415, EPI_ISL_750416, EPI_ISL_750417, EPI_ISL_750418, EPI_ISL_750419, EPI_ISL_750420, EPI_ISL_750421, EPI_ISL_750422, EPI_ISL_750423, EPI_ISL_750424, EPI_ISL_750425, EPI_ISL_750426, EPI_ISL_750427, EPI_ISL_750428, EPI_ISL_750429			
see above	Department of Virus and Microbiological Special Diagnostics, Statens Serum Institut, Copenhagen, Denmark	Albertsen Lab, Department of Chemistry and Bioscience, Aalborg University, Denmark	Danish Covid-19 Genome Consortium
EPI_ISL_750430	Sanatorio Americano	Institut Pasteur de Montevideo	Daiana Mir, Natalia Rego, Paola Cristina Resende, Fernando Lopez-Tort, Tamara Fernandez-Calero, Veronica Noya, Mariana Brandes, Tania Possi, Mailen Arleo, Natalia Reyes, Matias Victoria, Andres Lizasoain, Matias Castells, Leticia Maya, Matías Salvo, Tatiana Schäffer Gregianini, Marilda Tereza Mar da Rosa, Letícia Garay Martins, Cecilia Alonso, Yasser Vega, Cecilia Salazar, Ignacio Ferrés, Pablo Smircich, Jose Sotelo, Ighor Arantes, Luciana Appolinario, Ana Carolina Mendonça, Maria Jose Benitez-Galeano, Martín Graña, Camila Simoes, Fernando Motta, Marilda Mendonça Siqueira, Gonzalo Bello, Rodney Colina, Lucia Spangenberg
EPI_ISL_750431, EPI_ISL_750432, EPI_ISL_750433, EPI_ISL_750434, EPI_ISL_750435, EPI_ISL_750436, EPI_ISL_750437, EPI_ISL_750438, EPI_ISL_750439, EPI_ISL_750440, EPI_ISL_750441, EPI_ISL_750442, EPI_ISL_750443, EPI_ISL_750444, EPI_ISL_750445, EPI_ISL_750446, EPI_ISL_750447, EPI_ISL_750448, EPI_ISL_750449, EPI_ISL_750450, EPI_ISL_750451, EPI_ISL_750452, EPI_ISL_750453, EPI_ISL_750454, EPI_ISL_750455, EPI_ISL_750456, EPI_ISL_750457, EPI_ISL_750458, EPI_ISL_750459, EPI_ISL_750460, EPI_ISL_750461, EPI_ISL_750462, EPI_ISL_750463, EPI_ISL_750464, EPI_ISL_750465, EPI_ISL_750466, EPI_ISL_750467, EPI_ISL_750468, EPI_ISL_750469, EPI_ISL_750470, EPI_ISL_750471, EPI_ISL_750472, EPI_ISL_750473, EPI_ISL_750474, EPI_ISL_750475, EPI_ISL_750476, EPI_ISL_750477, EPI_ISL_750478, EPI_ISL_750479, EPI_ISL_750480, EPI_ISL_750481, EPI_ISL_750482, EPI_ISL_750483, EPI_ISL_750484, EPI_ISL_750485, EPI_ISL_750486, EPI_ISL_750487, EPI_ISL_750488, EPI_ISL_750489, EPI_ISL_750490, EPI_ISL_750491, EPI_ISL_750492, EPI_ISL_750493, EPI_ISL_750494, EPI_ISL_750495, EPI_ISL_750496, EPI_ISL_750497, EPI_ISL_750498, EPI_ISL_750499, EPI_ISL_750500, EPI_ISL_750501, EPI_ISL_750502, EPI_ISL_750503, EPI_ISL_750504, EPI_ISL_750505, EPI_ISL_750506, EPI_ISL_750507, EPI_ISL_750508, EPI_ISL_750509, EPI_ISL_750512, EPI_ISL_750513, EPI_ISL_750514, EPI_ISL_750515, EPI_ISL_750516, EPI_ISL_750517, EPI_ISL_750518, EPI_ISL_750519, EPI_ISL_750520, EPI_ISL_750521, EPI_ISL_750522, EPI_ISL_750523, EPI_ISL_750524, EPI_ISL_750525, EPI_ISL_750526, EPI_ISL_750527, EPI_ISL_750528, EPI_ISL_750529, EPI_ISL_750530, EPI_ISL_750531, EPI_ISL_750532, EPI_ISL_750533, EPI_ISL_750534, EPI_ISL_750535, EPI_ISL_750536, EPI_ISL_750537, EPI_ISL_750538, EPI_ISL_750539, EPI_ISL_750540, EPI_ISL_750541, EPI_ISL_750542, EPI_ISL_750543, EPI_ISL_750544, EPI_ISL_750545, EPI_ISL_750546, EPI_ISL_750547, EPI_ISL_750548, EPI_ISL_750549, EPI_ISL_750550, EPI_ISL_750551, EPI_ISL_750552, EPI_ISL_750553, EPI_ISL_750554, EPI_ISL_750555, EPI_ISL_750556, EPI_ISL_750557, EPI_ISL_750558, EPI_ISL_750559, EPI_ISL_750560, EPI_ISL_750561, EPI_ISL_750562, EPI_ISL_750563, EPI_ISL_750564, EPI_ISL_750565, EPI_ISL_750566, EPI_ISL_750567, EPI_ISL_750568, EPI_ISL_750569, EPI_ISL_750570, EPI_ISL_750571, EPI_ISL_750572, EPI_ISL_750573, EPI_ISL_750574, EPI_ISL_750575, EPI_ISL_750576, EPI_ISL_750577, EPI_ISL_750578, EPI_ISL_750579, EPI_ISL_750580, EPI_ISL_750581, EPI_ISL_750582, EPI_ISL_750583, EPI_ISL_750584, EPI_ISL_750585, EPI_ISL_750586, EPI_ISL_750587, EPI_ISL_750588, EPI_ISL_750589, EPI_ISL_750590, EPI_ISL_750591, EPI_ISL_750592, EPI_ISL_750593, EPI_ISL_750594, EPI_ISL_750595, EPI_ISL_750596, EPI_ISL_750597, EPI_ISL_750598, EPI_ISL_750599, EPI_ISL_750600, EPI_ISL_750601, EPI_ISL_750602, EPI_ISL_750603, EPI_ISL_750604, EPI_ISL_750605, EPI_ISL_750606, EPI_ISL_750607, EPI_ISL_750608, EPI_ISL_750609, EPI_ISL_750610, EPI_ISL_750611, EPI_ISL_750612, EPI_ISL_750613, EPI_ISL_750614, EPI_ISL_750615, EPI_ISL_750616, EPI_ISL_750617, EPI_ISL_750618, EPI_ISL_750619, EPI_ISL_750620, EPI_ISL_750621, EPI_ISL_750622, EPI_ISL_750623, EPI_ISL_750624, EPI_ISL_750625, EPI_ISL_750626, EPI_ISL_750627, EPI_ISL_750628, EPI_ISL_750629, EPI_ISL_750630, EPI_ISL_750631, EPI_ISL_750632, EPI_ISL_750633, EPI_ISL_750634, EPI_ISL_750635, EPI_ISL_750636, EPI_ISL_750637, EPI_ISL_750638, EPI_ISL_750639, EPI_ISL_750640, EPI_ISL_750641, EPI_ISL_750642, EPI_ISL_750643, EPI_ISL_750644, EPI_ISL_750645, EPI_ISL_750646, EPI_ISL_750647, EPI_ISL_750648, EPI_ISL_750649, EPI_ISL_750650, EPI_ISL_750651, EPI_ISL_750652, EPI_ISL_750653, EPI_ISL_750654, EPI_ISL_750655, EPI_ISL_750656, EPI_ISL_750657, EPI_ISL_750658, EPI_ISL_750659, EPI_ISL_750660, EPI_ISL_750661, EPI_ISL_750662, EPI_ISL_750663, EPI_ISL_750664, EPI_ISL_750665, EPI_ISL_750666, EPI_ISL_750667, EPI_ISL_750668, EPI_ISL_750669, EPI_ISL_750670, EPI_ISL_750671, EPI_ISL_750672, EPI_ISL_750673, EPI_ISL_750674, EPI_ISL_750675, EPI_ISL_750676, EPI_ISL_750677, EPI_ISL_750678, EPI_ISL_750679, EPI_ISL_750680, EPI_ISL_750681, EPI_ISL_750682, EPI_ISL_750683, EPI_ISL_750684, EPI_ISL_750685, EPI_ISL_750686, EPI_ISL_750687, EPI_ISL_750688, EPI_ISL_750689, EPI_ISL_750690, EPI_ISL_750691, EPI_ISL_750692, EPI_ISL_750693, EPI_ISL_750694, EPI_ISL_750695, EPI_ISL_750696, EPI_ISL_750697, EPI_ISL_750698, EPI_ISL_750699, EPI_ISL_750700, EPI_ISL_750701, EPI_ISL_750702, EPI_ISL_750703, EPI_ISL_750704, EPI_ISL_750705, EPI_ISL_750706, EPI_ISL_750707, EPI_ISL_750708, EPI_ISL_750709, EPI_ISL_750710, EPI_ISL_750711, EPI_ISL_750712, EPI_ISL_750713, EPI_ISL_750714, EPI_ISL_750715, EPI_ISL_750716, EPI_ISL_750717, EPI_ISL_750718, EPI_ISL_750719, EPI_ISL_750720, EPI_ISL_750721, EPI_ISL_750722, EPI_ISL_750723, EPI_ISL_750724, EPI_ISL_750725, EPI_ISL_750726, EPI_ISL_750727, EPI_ISL_750728, EPI_ISL_750729, EPI_ISL_750730, EPI_ISL_750731, EPI_ISL_750732, EPI_ISL_750733, EPI_ISL_750734, EPI_ISL_750735, EPI_ISL_750736, EPI_ISL_750737, EPI_ISL_750738, EPI_ISL_750739, EPI_ISL_750740, EPI_ISL_750741, EPI_ISL_750742, EPI_ISL_750743, EPI_ISL_750744, EPI_ISL_750745, EPI_ISL_750746, EPI_ISL_750747, EPI_ISL_750748, EPI_ISL_750749, EPI_ISL_750750, EPI_ISL_750751, EPI_ISL_750752, EPI_ISL_750753, EPI_ISL_750754, EPI_ISL_750755, EPI_ISL_750756, EPI_ISL_750757, EPI_ISL_750758, EPI_ISL_750759, EPI_ISL_750760, EPI_ISL_750761, EPI_ISL_750762, EPI_ISL_750763, EPI_ISL_750764, EPI_ISL_750765, EPI_ISL_750766, EPI_ISL_750767, EPI_ISL_750768, EPI_ISL_750769, EPI_ISL_750770, EPI_ISL_750771, EPI_ISL_750772, EPI_ISL_750773, EPI_ISL_750774, EPI_ISL_750775, EPI_ISL_750776, EPI_ISL_750777, EPI_ISL_750778, EPI_ISL_750779, EPI_ISL_750780, EPI_ISL_750781, EPI_ISL_750782, EPI_ISL_750783, EPI_ISL_750784, EPI_ISL_750785, EPI_ISL_750786, EPI_ISL_750787, EPI_ISL_750788, EPI_ISL_750789, EPI_ISL_750790, EPI_ISL_750791, EPI_ISL_750792, EPI_ISL_750793, EPI_ISL_750794, EPI_ISL_750795, EPI_ISL_750796, EPI_ISL_750797, EPI_ISL_750798, EPI_ISL_750799, EPI_ISL_750800, EPI_ISL_750801, EPI_ISL_750802, EPI_ISL_750803, EPI_ISL_750804, EPI_ISL_750805, EPI_ISL_750806, EPI_ISL_750807, EPI_ISL_750808, EPI_ISL_750809, EPI_ISL_750810, EPI_ISL_750811, EPI_ISL_750812, EPI_ISL_750813, EPI_ISL_750814, EPI_ISL_750815, EPI_ISL_750816, EPI_ISL_750817, EPI_ISL_750818, EPI_ISL_750819			
see above	Department of Virus and Microbiological Special Diagnostics, Statens Serum Institut, Copenhagen, Denmark	Albertsen Lab, Department of Chemistry and Bioscience, Aalborg University, Denmark	Danish Covid-19 Genome Consortium
EPI_ISL_750820	Sanatorio Americano	Institut Pasteur de Montevideo	Daiana Mir, Natalia Rego, Paola Cristina Resende, Fernando Lopez-Tort, Tamara Fernandez-Calero, Veronica Noya, Mariana Brandes, Tania Possi, Mailen Arleo, Natalia Reyes, Matias Victoria, Andres Lizasoain, Matias Castells, Leticia Maya, Matías Salvo, Tatiana Schäffer Gregianini, Marilda Tereza Mar da Rosa, Letícia Garay Martins, Cecilia Alonso, Yasser Vega, Cecilia Salazar, Ignacio Ferrés, Pablo Smircich, Jose Sotelo, Ighor Arantes, Luciana Appolinario, Ana Carolina Mendonça, Maria Jose Benitez-Galeano, Martín Graña, Camila Simoes, Fernando Motta, Marilda Mendonça Siqueira, Gonzalo Bello, Rodney Colina, Lucia Spangenberg
EPI_ISL_750821, EPI_ISL_750822, EPI_ISL_750823, EPI_ISL_750824, EPI_ISL_750825, EPI_ISL_750826, EPI_ISL_750827, EPI_ISL_750828, EPI_ISL_750829, EPI_ISL_750830, EPI_ISL_750831, EPI_ISL_750832, EPI_ISL_750833, EPI_ISL_750834, EPI_ISL_750835, EPI_ISL_750836, EPI_ISL_750837, EPI_ISL_750838, EPI_ISL_750839, EPI_ISL_750840, EPI_ISL_750841, EPI_ISL_750842, EPI_ISL_750843, EPI_ISL_750844, EPI_ISL_750845, EPI_ISL_750846, EPI_ISL_750847, EPI_ISL_750848, EPI_ISL_750849, EPI_ISL_750850, EPI_ISL_750851, EPI_ISL_750852, EPI_ISL_750853, EPI_ISL_750854, EPI_ISL_750855, EPI_ISL_750856, EPI_ISL_750857, EPI_ISL_750858, EPI_ISL_750859, EPI_ISL_750860, EPI_ISL_750861, EPI_ISL_750862, EPI_ISL_750863, EPI_ISL_750864, EPI_ISL_750865, EPI_ISL_750866, EPI_ISL_750867, EPI_ISL_750868, EPI_ISL_750869, EPI_ISL_750870, EPI_ISL_750871, EPI_ISL_750872, EPI_ISL_750873, EPI_ISL_750874, EPI_ISL_750875, EPI_ISL_750876, EPI_ISL_750877, EPI_ISL_750878, EPI_ISL_750879, EPI_ISL_750880, EPI_ISL_750881, EPI_ISL_750882, EPI_ISL_750883, EPI_ISL_750884, EPI_ISL_750885, EPI_ISL_750886, EPI_ISL_750887, EPI_ISL_750888, EPI_ISL_750889, EPI_ISL_750890, EPI_ISL_750891, EPI_ISL_750892, EPI_ISL_750893, EPI_ISL_750894, EPI_ISL_750895, EPI_ISL_750896, EPI_ISL_750897, EPI_ISL_750898, EPI_ISL_750899, EPI_ISL_750900, EPI_ISL_750901, EPI_ISL_750902, EPI_ISL_750903, EPI_ISL_750904, EPI_ISL_750905, EPI_ISL_750906, EPI_ISL_750907, EPI_ISL_750908, EPI_ISL_750909, EPI_ISL_750910, EPI_ISL_750911, EPI_ISL_750912, EPI_ISL_750913, EPI_ISL_750914, EPI_ISL_750915, EPI_ISL_750916, EPI_ISL_750917, EPI_ISL_750918, EPI_ISL_750919, EPI_ISL_750920, EPI_ISL_750921, EPI_ISL_750922, EPI_ISL_750923, EPI_ISL_750924, EPI_ISL_750925, EPI_ISL_750926, EPI_ISL_750927, EPI_ISL_750928, EPI_ISL_750929, EPI_ISL_750930, EPI_ISL_750931, EPI_ISL_750932, EPI_ISL_750933, EPI_ISL_750934, EPI_ISL_750935, EPI_ISL_750936, EPI_ISL_750937, EPI_ISL_750938, EPI_ISL_750939, EPI_ISL_750940, EPI_ISL_750941, EPI_ISL_750942, EPI_ISL_750943, EPI_ISL_750944, EPI_ISL_750945, EPI_ISL_750946, EPI_ISL_750947, EPI_ISL_750948, EPI_ISL_750949, EPI_ISL_750950, EPI_ISL_750951, EPI_ISL_750952, EPI_ISL_750953, EPI_ISL_750954, EPI_ISL_750955, EPI_ISL_750956, EPI_ISL_750957, EPI_ISL_750958, EPI_ISL_750959, EPI_ISL_750960, EPI_ISL_750961, EPI_ISL_750962, EPI_ISL_750963, EPI_ISL_750964, EPI_ISL_750965, EPI_ISL_750966, EPI_ISL_750967, EPI_ISL_750968, EPI_ISL_750969, EPI_ISL_750970, EPI_ISL_750971, EPI_ISL_750972, EPI_ISL_750973, EPI_ISL_750974, EPI_ISL_750975, EPI_ISL_750976, EPI_ISL_750977, EPI_ISL_750978, EPI_ISL_750979, EPI_ISL_750980, EPI_ISL_750981, EPI_ISL_750982, EPI_ISL_750983, EPI_ISL_750984, EPI_ISL_750985, EPI_ISL_750986, EPI_ISL_750987, EPI_ISL_750988, EPI_ISL_750989, EPI_ISL_750990, EPI_ISL_750991, EPI_ISL_750992, EPI_ISL_750993, EPI_ISL_750994, EPI_ISL_750995, EPI_ISL_750996, EPI_ISL_750997, EPI_ISL_750998, EPI_ISL_750999, EPI_ISL_751000, EPI_ISL_751001, EPI_ISL_751002, EPI_ISL_751003, EPI_ISL_751004, EPI_ISL_751005, EPI_ISL_751006, EPI_ISL_751007, EPI_ISL_751008, EPI_ISL_751009, EPI_ISL_751010			
see above	Department of Virus and Microbiological Special Diagnostics, Statens Serum Institut, Copenhagen, Denmark	Albertsen Lab, Department of Chemistry and Bioscience, Aalborg University, Denmark	Danish Covid-19 Genome Consortium
EPI_ISL_751011	Sanatorio Americano	Institut Pasteur de Montevideo	Daiana Mir, Natalia Rego, Paola Cristina Resende, Fernando Lopez-Tort, Tamara Fernandez-Calero, Veronica Noya, Mariana Brandes, Tania Possi, Mailen Arleo, Natalia Reyes, Matias Victoria, Andres Lizasoain, Matias Castells, Leticia Maya, Matías Salvo, Tatiana Schäffer Gregianini, Marilda Tereza Mar da Rosa, Letícia Garay Martins, Cecilia Alonso, Yasser Vega, Cecilia Salazar, Ignacio Ferrés, Pablo Smircich, Jose Sotelo, Ighor Arantes, Luciana Appolinario, Ana Carolina Mendonça, Maria Jose Benitez-Galeano, Martín Graña, Camila Simoes, Fernando Motta, Marilda Mendonça Siqueira, Gonzalo Bello, Rodney Colina, Lucia Spangenberg
EPI_ISL_751012, EPI_ISL_751013, EPI_ISL_751014, EPI_ISL_751015, EPI_ISL_751016, EPI_ISL_751017, EPI_ISL_751018, EPI_ISL_751019, EPI_ISL_751020, EPI_ISL_751021, EPI_ISL_751022, EPI_ISL_751023, EPI_ISL_751024, EPI_ISL_751025, EPI_ISL_751026, EPI_ISL_751027, EPI_ISL_751028, EPI_ISL_751029, EPI_ISL_751030, EPI_ISL_751031, EPI_ISL_751032, EPI_ISL_751033, EPI_ISL_751034, EPI_ISL_751035, EPI_ISL_751036, EPI_ISL_751037, EPI_ISL_751038, EPI_ISL_751039, EPI_ISL_751040, EPI_ISL_751041, EPI_ISL_751042, EPI_ISL_751043, EPI_ISL_751044, EPI_ISL_751045, EPI_ISL_751046, EPI_ISL_751047, EPI_ISL_751048, EPI_ISL_751049, EPI_ISL_751050, EPI_ISL_751051, EPI_ISL_751052, EPI_ISL_751053, EPI_ISL_751054, EPI_ISL_751055, EPI_ISL_751056, EPI_ISL_751057, EPI_ISL_751058, EPI_ISL_751059, EPI_ISL_751060, EPI_ISL_751061, EPI_ISL_751062, EPI_ISL_751063, EPI_ISL_751064, EPI_ISL_751065, EPI_ISL_751066, EPI_ISL_751067, EPI_ISL_751068, EPI_ISL_751069, EPI_ISL_751070, EPI_ISL_751071, EPI_ISL_751072, EPI_ISL_751073, EPI_ISL_751074, EPI_ISL_751075, EPI_ISL_751076, EPI_ISL_751077, EPI_ISL_751078, EPI_ISL_751079, EPI_ISL_751080, EPI_ISL_751081, EPI_ISL_751082, EPI_ISL_751083, EPI_ISL_751084, EPI_ISL_751085, EPI_ISL_751086, EPI_ISL_751087, EPI_ISL_751088, EPI_ISL_751089, EPI_ISL_751090, EPI_ISL_751091, EPI_ISL_751092, EPI_ISL_751093, EPI_ISL_751094, EPI_ISL_751095, EPI_ISL_751096, EPI_ISL_751097, EPI_ISL_751098, EPI_ISL_751099, EPI_ISL_751100, EPI_ISL_751101, EPI_ISL_751102, EPI_ISL_751103, EPI_ISL_751104, EPI_ISL_751105, EPI_ISL_751106, EPI_ISL_751107, EPI_ISL_751108, EPI_ISL_751109, EPI_ISL_751110, EPI_ISL_751111, EPI_ISL_751112, EPI_ISL_751113, EPI_ISL_751114, EPI_ISL_751115, EPI_ISL_751116, EPI_ISL_751117, EPI_ISL_751118, EPI_ISL_751119, EPI_ISL_751120, EPI_ISL_751121, EPI_ISL_751122, EPI_ISL_751123, EPI_ISL_751124, EPI_ISL_751125, EPI_ISL_751126, EPI_ISL_751127, EPI_ISL_751128, EPI_ISL_751129, EPI_ISL_751130, EPI_ISL_751131, EPI_ISL_751132, EPI_ISL_751133, EPI_ISL_751134, EPI_ISL_751135, EPI_ISL_751136, EPI_ISL_751137, EPI_ISL_751138, EPI_ISL_751139, EPI_ISL_751140, EPI_ISL_751141, EPI_ISL_751142, EPI_ISL_751143, EPI_ISL_751144, EPI_ISL_751145, EPI_ISL_751146, EPI_ISL_751147, EPI_ISL_751148, EPI_ISL_751149, EPI_ISL_751150, EPI_ISL_751151, EPI_ISL_751152, EPI_ISL_751153, EPI_ISL_751154, EPI_ISL_751155, EPI_ISL_751156, EPI_ISL_751157, EPI_ISL_751158, EPI_ISL_751159, EPI_ISL_751160, EPI_ISL_751161, EPI_ISL_751162, EPI_ISL_751163, EPI_ISL_751164, EPI_ISL_751165, EPI_ISL_751166, EPI_ISL_751167, EPI_ISL_751168, EPI_ISL_751169, EPI_ISL_751170, EPI_ISL_751171, EPI_ISL_751172, EPI_ISL_751173, EPI_ISL_751174, EPI_ISL_751175, EPI_ISL_751176, EPI_ISL_751177, EPI_ISL_751178, EPI_ISL_751179, EPI_ISL_751180, EPI_ISL_751181, EPI_ISL_751182, EPI_ISL_751183			
see above	Department of Virus and Microbiological Special Diagnostics, Statens Serum Institut, Copenhagen, Denmark	Albertsen Lab, Department of Chemistry and Bioscience, Aalborg University, Denmark	Danish Covid-19 Genome Consortium
EPI_ISL_751185,	CENUR Litoral Norte - UdelaR,	Institut Pasteur de	Daiana Mir, Natalia Rego, Paola Cristina Resende, Fernando Lopez-Tort, Tamara Fernandez-Calero, Veronica Noya, Mariana Brandes, Tania Possi, Mailen Arleo, Natalia Reyes, Matias Victoria, Andres Lizasoain, Matias Castells, Leticia Maya,

EPI_ISL_751187, EPI_ISL_751188	Salto, Uruguay	Montevideo	Matías Salvo, Tatiana Schäffer Gregianini, Marilda Tereza Mar da Rosa, Letícia Garay Martins, Cecilia Alonso, Yasser Vega, Cecilia Salazar, Ignacio Ferrés, Pablo Smircich, Jose Sotelo, Ighor Arantes, Luciana Appolinario, Ana Carolina Mendonça, Maria Jose Benitez-Galeano, Martín Graña, Camila Simoes, Fernando Motta, Marilda Mendonça Siqueira, Gonzalo Bello, Rodney Colina, Lucia Spangenberg
EPI_ISL_751191, EPI_ISL_751192	Limmattal Hospital	Institute of Medical Virology, University of Zurich	Stefan Schmutz, Kevin Steiner, Verena Kufner, Maryam Zaheri, Gabriela Ziltener, Jürg Böni, Michael Huber, Alexandra Trkola, Roberto Buonomano
EPI_ISL_751193	University Hospital Zürich	Institute of Medical Virology, University of Zurich	Stefan Schmutz, Kevin Steiner, Verena Kufner, Maryam Zaheri, Gabriela Ziltener, Jürg Böni, Michael Huber, Alexandra Trkola
EPI_ISL_751194, EPI_ISL_751195, EPI_ISL_751196, EPI_ISL_751197, EPI_ISL_751198, EPI_ISL_751199, EPI_ISL_751200	Dienststelle Gesundheit und Sport Kanton Luzern	Institute of Medical Virology, University of Zurich	Stefan Schmutz, Kevin Steiner, Verena Kufner, Maryam Zaheri, Gabriela Ziltener, Jürg Böni, Michael Huber, Alexandra Trkola, Claudia Schmutz, Eva Spieler
EPI_ISL_751201	Laboratorio DILAVE/MGAP-INIA-UdelaR -Tacuarembó	Institut Pasteur de Montevideo	Daiana Mir, Natalia Rego, Paola Cristina Resende, Fernando Lopez-Tort, Tamara Fernandez-Calero, Veronica Noya, Mariana Brandes, Tania Possi, Mailen Arleo, Natalia Reyes, Matias Victoria, Andres Lizasoain, Matias Castells, Leticia Maya, Matías Salvo, Tatiana Schäffer Gregianini, Marilda Tereza Mar da Rosa, Letícia Garay Martins, Cecilia Alonso, Yasser Vega, Cecilia Salazar, Ignacio Ferrés, Pablo Smircich, Jose Sotelo, Ighor Arantes, Luciana Appolinario, Ana Carolina Mendonça, Maria Jose Benitez-Galeano, Martín Graña, Camila Simoes, Fernando Motta, Marilda Mendonça Siqueira, Gonzalo Bello, Rodney Colina, Lucia Spangenberg
EPI_ISL_751209	Pathogen Genomics Lab King Abdullah University of Science and Technology(KAUST)	Pathogen Genomics Lab King Abdullah University of Science and Technology(KAUST)	Sara Mfarrej, Olga Douvropoulou, Raushan Nugmanova, Raeece Naeem, Sharif Hala, Fadwa Alofi, Asim Khogeer, Afrah Alsomali, Jumana Taha, Abdulaziz Alahmadi, Kahled Algithami, Anwar Hashem, Naif Almontashiri, Arnab Pain
EPI_ISL_751210	Pathogen Genomics Lab King Abdullah University of Science and Technology(KAUST)	Pathogen Genomics Lab King Abdullah University of Science and Technology(KAUST)	Sara Mfarrej, Raushan Nugmanova, Olga Douvropoulou, Sharif Hala, Raeece Naeem, Fadwa Alofi, Asim Khogeer, Afrah Alsomali, Jumana Taha, Abdulaziz Alahmadi, Kahled Algithami, Anwar Hashem, Naif Almontashiri, Arnab Pain
EPI_ISL_751214	Pathogen Genomics Lab King Abdullah University of Science and Technology(KAUST)	Pathogen Genomics Lab King Abdullah University of Science and Technology(KAUST)	Sara Mfarrej, Raushan Nugmanova, Olga Douvropoulou, Sharif Hala, Raeece Naeem, Fadwa Alofi, Asim Khogeer, Afrah Alsomali, Jumana Taha, Abdulaziz Alahmadi, Kahled Algithami, Anwar Hashem, Naif Almontashiri, Arnab Pain
EPI_ISL_751215	Pathogen Genomics Lab King Abdullah University of Science and Technology(KAUST)	Pathogen Genomics Lab King Abdullah University of Science and Technology(KAUST)	Sara Mfarrej, Raushan Nugmanova, Olga Douvropoulou, Sharif Hala, Raeece Naeem, Fadwa Alofi, Asim Khogeer, Afrah Alsomali, Jumana Taha, Abdulaziz Alahmadi, Kahled Algithami, Anwar Hashem, Naif Almontashiri, Arnab Pain
EPI_ISL_751216	Pathogen Genomics Lab King Abdullah University of Science and Technology(KAUST)	Pathogen Genomics Lab King Abdullah University of Science and Technology(KAUST)	Sara Mfarrej, Olga Douvropoulou, Raushan Nugmanova, Raeece Naeem, Sharif Hala, Fadwa Alofi, Asim Khogeer, Afrah Alsomali, Jumana Taha, Abdulaziz Alahmadi, Kahled Algithami, Anwar Hashem, Naif Almontashiri, Arnab Pain
EPI_ISL_751217	Pathogen Genomics Lab King Abdullah University of Science and Technology(KAUST)	Pathogen Genomics Lab King Abdullah University of Science and Technology(KAUST)	Sara Mfarrej, Sharif Hala, Raushan Nugmanova, Olga Douvropoulou, Raeece Naeem, Fadwa Alofi, Asim Khogeer, Afrah Alsomali, Jumana Taha, Abdulaziz Alahmadi, Kahled Algithami, Anwar Hashem, Naif Almontashiri, Arnab Pain
EPI_ISL_751225	Pathogen Genomics Lab King Abdullah University of Science and Technology(KAUST)	Pathogen Genomics Lab King Abdullah University of Science and Technology(KAUST)	Raushan Nugmanova, Olga Douvropoulou, Sara Mfarrej, Raeece Naeem, Sharif Hala, Fadwa Alofi, Asim Khogeer, Afrah Alsomali, Jumana Taha, Abdulaziz Alahmadi, Kahled Algithami, Anwar Hashem, Naif Almontashiri, Arnab Pain
EPI_ISL_751226	Pathogen Genomics Lab King Abdullah University of Science and Technology(KAUST)	Pathogen Genomics Lab King Abdullah University of Science and Technology(KAUST)	Sara Mfarrej, Raushan Nugmanova, Olga Douvropoulou, Sharif Hala, Raeece Naeem, Fadwa Alofi, Asim Khogeer, Afrah Alsomali, Jumana Taha, Abdulaziz Alahmadi, Kahled Algithami, Anwar Hashem, Naif Almontashiri, Arnab Pain
EPI_ISL_751227	Pathogen Genomics Lab King Abdullah University of Science and Technology(KAUST)	Pathogen Genomics Lab King Abdullah University of Science and Technology(KAUST)	Sara Mfarrej, Sharif Hala, Raushan Nugmanova, Olga Douvropoulou, Raeece Naeem, Fadwa Alofi, Asim Khogeer, Afrah Alsomali, Jumana Taha, Abdulaziz Alahmadi, Kahled Algithami, Anwar Hashem, Naif Almontashiri, Arnab Pain
EPI_ISL_751228	Pathogen Genomics Lab King Abdullah University of Science and Technology(KAUST)	Pathogen Genomics Lab King Abdullah University of Science and Technology(KAUST)	Sara Mfarrej, Raushan Nugmanova, Olga Douvropoulou, Sharif Hala, Raeece Naeem, Fadwa Alofi, Asim Khogeer, Afrah Alsomali, Jumana Taha, Abdulaziz Alahmadi, Kahled Algithami, Anwar Hashem, Naif Almontashiri, Arnab Pain
EPI_ISL_751229	Pathogen Genomics Lab King Abdullah University of Science and Technology(KAUST)	Pathogen Genomics Lab King Abdullah University of Science and Technology(KAUST)	Sara Mfarrej, Sharif Hala, Raushan Nugmanova, Olga Douvropoulou, Raeece Naeem, Fadwa Alofi, Asim Khogeer, Afrah Alsomali, Jumana Taha, Abdulaziz Alahmadi, Kahled Algithami, Anwar Hashem, Naif Almontashiri, Arnab Pain
EPI_ISL_751230	Pathogen Genomics Lab King Abdullah University of Science and Technology(KAUST)	Pathogen Genomics Lab King Abdullah University of Science and Technology(KAUST)	Sara Mfarrej, Olga Douvropoulou, Raushan Nugmanova, Raeece Naeem, Sharif Hala, Fadwa Alofi, Asim Khogeer, Afrah Alsomali, Jumana Taha, Abdulaziz Alahmadi, Kahled Algithami, Anwar Hashem, Naif Almontashiri, Arnab Pain
EPI_ISL_751231	Pathogen Genomics Lab King Abdullah University of Science and Technology(KAUST)	Pathogen Genomics Lab King Abdullah University of Science and Technology(KAUST)	Sara Mfarrej, Raushan Nugmanova, Olga Douvropoulou, Sharif Hala, Raeece Naeem, Fadwa Alofi, Asim Khogeer, Afrah Alsomali, Jumana Taha, Abdulaziz Alahmadi, Kahled Algithami, Anwar Hashem, Naif Almontashiri, Arnab Pain
EPI_ISL_751232	Pathogen Genomics Lab King Abdullah University of Science and Technology(KAUST)	Pathogen Genomics Lab King Abdullah University of Science and Technology(KAUST)	Sara Mfarrej, Sharif Hala, Raushan Nugmanova, Olga Douvropoulou, Raeece Naeem, Fadwa Alofi, Asim Khogeer, Afrah Alsomali, Jumana Taha, Abdulaziz Alahmadi, Kahled Algithami, Anwar Hashem, Naif Almontashiri, Arnab Pain
EPI_ISL_751233	Pathogen Genomics Lab King Abdullah University of Science and Technology(KAUST)	Pathogen Genomics Lab King Abdullah University of Science and Technology(KAUST)	Olga Douvropoulou, Raushan Nugmanova, Sara Mfarrej, Raeece Naeem, Sharif Hala, Fadwa Alofi, Asim Khogeer, Afrah Alsomali, Jumana Taha, Abdulaziz Alahmadi, Kahled Algithami, Anwar Hashem, Naif Almontashiri, Arnab Pain

EPI_ISL_751234, EPI_ISL_751235, EPI_ISL_751236	Pathogen Genomics Lab King Abdullah University of Science and Technology(KAUST)	Pathogen Genomics Lab King Abdullah University of Science and Technology(KAUST)	Sara Mfarrej, Olga Douvropoulou, Raushan Nugmanova, Raeece Naeem, Sharif Hala, Fadwa Alofi, Asim Khogeer, Afrah Alsomali, Jumana Taha, Abdulaziz Alahmadi, Kahled Algithami, Anwar Hashem, Naif Almontashiri, Arnab Pain
EPI_ISL_751323, EPI_ISL_751324, EPI_ISL_751331, EPI_ISL_751332, EPI_ISL_751335, EPI_ISL_751337, EPI_ISL_751339, EPI_ISL_751340, EPI_ISL_751341, EPI_ISL_751342, EPI_ISL_751343, EPI_ISL_751344, EPI_ISL_751345, EPI_ISL_751346			
see above	IRCCS Sacro Cuore Don Calabria Hospital, Department of Infectious, Tropical Diseases & Microbiology	University of Verona, Department of Biotechnology	Antonio Mori, Michela Deiana, Elena Pomari, Chiara Piubelli; Giulia Lopatriello, Luca Marcolungo, Cristina Beltrami, Chiara Degli Esposti, Emanuela Cosentino, Massimo Delledonne
EPI_ISL_751448, EPI_ISL_751449, EPI_ISL_751450, EPI_ISL_751451, EPI_ISL_751452, EPI_ISL_751453, EPI_ISL_751454, EPI_ISL_751455, EPI_ISL_751456, EPI_ISL_751457, EPI_ISL_751458, EPI_ISL_751459, EPI_ISL_751460, EPI_ISL_751461, EPI_ISL_751462, EPI_ISL_751463, EPI_ISL_751464, EPI_ISL_751465, EPI_ISL_751466, EPI_ISL_751467, EPI_ISL_751468, EPI_ISL_751469, EPI_ISL_751470, EPI_ISL_751471, EPI_ISL_751472, EPI_ISL_751473, EPI_ISL_751474, EPI_ISL_751475, EPI_ISL_751476, EPI_ISL_751477, EPI_ISL_751478, EPI_ISL_751479, EPI_ISL_751480, EPI_ISL_751481, EPI_ISL_751482, EPI_ISL_751483, EPI_ISL_751484, EPI_ISL_751485, EPI_ISL_751486, EPI_ISL_751487, EPI_ISL_751488, EPI_ISL_751489, EPI_ISL_751490, EPI_ISL_751491, EPI_ISL_751492, EPI_ISL_751493, EPI_ISL_751494, EPI_ISL_751495, EPI_ISL_751496, EPI_ISL_751497, EPI_ISL_751498, EPI_ISL_751499			
see above	CHU Purpan - Laboratoire de Virologie - Institut Fédératif de Biologie	CHU Purpan - Laboratoire de Virologie - Institut Fédératif de Biologie	Latour J., Ranger N., Dubois M., Carcenac R., Harter A., Boyer P., Tremeaux P., Izopet J.
EPI_ISL_751501	Pathogen Genomics Lab King Abdullah University of Science and Technology(KAUST)	Pathogen Genomics Lab King Abdullah University of Science and Technology(KAUST)	Sara Mfarrej, Raushan Nugmanova, Olga Douvropoulou, Sharif Hala, Raeece Naeem, Fadwa Alofi, Asim Khogeer, Afrah Alsomali, Jumana Taha, Abdulaziz Alahmadi, Kahled Algithami, Anwar Hashem, Naif Almontashiri, Arnab Pain
EPI_ISL_751504, EPI_ISL_751505, EPI_ISL_751506, EPI_ISL_751507, EPI_ISL_751508, EPI_ISL_751509, EPI_ISL_751510, EPI_ISL_751511, EPI_ISL_751512, EPI_ISL_751513, EPI_ISL_751514, EPI_ISL_751515, EPI_ISL_751516, EPI_ISL_751517, EPI_ISL_751518, EPI_ISL_751519			
see above	Texas Department of State Health Services	Texas Department of State Health Services	Rashmi Tuladhar, Bonnie Oh, Jenny Zhang, Maliha Rahman, Anita Pokharel, Myong Koag, Chung Wang, Rachel Lee, Grace Kubin, Mayela Pedrueza, James Daniel Bonser
EPI_ISL_751542	SC Dept of Health and Env. Control-Bureau of Laboratories	Genomics and Discovery, Respiratory Viruses Branch, Division of Viral Diseases, Centers for Disease Control and Prevention	Krista Queen, Yan Li, Ying Tao, Jing Zhang, Anna Uehara, Anna Montmayeur, Clinton R. Paden, Peter W. Cook,Rachel Marine, Mili Sheth, Haibin Wang, Justin Lee, Suxiang Tong
EPI_ISL_751543	DE Public Health Laboratory	Genomics and Discovery, Respiratory Viruses Branch, Division of Viral Diseases, Centers for Disease Control and Prevention	Krista Queen, Yan Li, Ying Tao, Jing Zhang, Anna Uehara, Anna Montmayeur, Clinton R. Paden, Peter W. Cook,Rachel Marine, Mili Sheth, Haibin Wang, Justin Lee, Suxiang Tong
EPI_ISL_751544	NH Dept. of Health and Human Services Public Health Labs	Genomics and Discovery, Respiratory Viruses Branch, Division of Viral Diseases, Centers for Disease Control and Prevention	Krista Queen, Yan Li, Ying Tao, Jing Zhang, Anna Uehara, Anna Montmayeur, Clinton R. Paden, Peter W. Cook,Rachel Marine, Mili Sheth, Haibin Wang, Justin Lee, Suxiang Tong
EPI_ISL_751545	NYSDOH Wadsworth Center, Virology Lab	Genomics and Discovery, Respiratory Viruses Branch, Division of Viral Diseases, Centers for Disease Control and Prevention	Krista Queen, Yan Li, Ying Tao, Jing Zhang, Anna Uehara, Anna Montmayeur, Clinton R. Paden, Peter W. Cook,Rachel Marine, Mili Sheth, Haibin Wang, Justin Lee, Suxiang Tong
EPI_ISL_751546	VA-Division of Consolidated Laboratory Services	Genomics and Discovery, Respiratory Viruses Branch, Division of Viral Diseases, Centers for Disease Control and Prevention	Krista Queen, Yan Li, Ying Tao, Jing Zhang, Anna Uehara, Anna Montmayeur, Clinton R. Paden, Peter W. Cook,Rachel Marine, Mili Sheth, Haibin Wang, Justin Lee, Suxiang Tong
EPI_ISL_751547	DE Public Health Laboratory	Genomics and Discovery, Respiratory Viruses Branch, Division of Viral Diseases, Centers for Disease Control and Prevention	Krista Queen, Yan Li, Ying Tao, Jing Zhang, Anna Uehara, Anna Montmayeur, Clinton R. Paden, Peter W. Cook,Rachel Marine, Mili Sheth, Haibin Wang, Justin Lee, Suxiang Tong
EPI_ISL_751548	NYSDOH Wadsworth Center, Virology Lab	Genomics and Discovery, Respiratory Viruses Branch, Division of Viral Diseases, Centers for Disease Control and Prevention	Krista Queen, Yan Li, Ying Tao, Jing Zhang, Anna Uehara, Anna Montmayeur, Clinton R. Paden, Peter W. Cook,Rachel Marine, Mili Sheth, Haibin Wang, Justin Lee, Suxiang Tong
EPI_ISL_751554	CT-Dr. Katherine A. Kelley State Public Health Lab	Genomics and Discovery, Respiratory Viruses Branch, Division of Viral Diseases, Centers for Disease Control and Prevention	Krista Queen, Yan Li, Ying Tao, Jing Zhang, Anna Uehara, Anna Montmayeur, Clinton R. Paden, Peter W. Cook,Rachel Marine, Mili Sheth, Haibin Wang, Justin Lee, Suxiang Tong
EPI_ISL_751555	WY Public Health Laboratory	Genomics and Discovery, Respiratory Viruses Branch, Division of Viral Diseases, Centers for Disease Control and Prevention	Krista Queen, Yan Li, Ying Tao, Jing Zhang, Anna Uehara, Anna Montmayeur, Clinton R. Paden, Peter W. Cook,Rachel Marine, Mili Sheth, Haibin Wang, Justin Lee, Suxiang Tong
EPI_ISL_751556, EPI_ISL_751557	NV State Public Health Laboratory	Genomics and Discovery, Respiratory Viruses	Krista Queen, Yan Li, Ying Tao, Jing Zhang, Anna Uehara, Anna Montmayeur, Clinton R. Paden, Peter W. Cook,Rachel Marine, Mili Sheth, Haibin Wang, Justin Lee, Suxiang Tong

		Branch, Division of Viral Diseases, Centers for Disease Control and Prevention	
EPI_ISL_751558	NH Dept. of Health and Human Services Public Health Labs	Genomics and Discovery, Respiratory Viruses Branch, Division of Viral Diseases, Centers for Disease Control and Prevention	Krista Queen, Yan Li, Ying Tao, Jing Zhang, Anna Uehara, Anna Montmayeur, Clinton R. Paden, Peter W. Cook,Rachel Marine, Mili Sheth, Haibin Wang, Justin Lee, Suxiang Tong
EPI_ISL_751559	MD DOH Laboratories Administration	Genomics and Discovery, Respiratory Viruses Branch, Division of Viral Diseases, Centers for Disease Control and Prevention	Krista Queen, Yan Li, Ying Tao, Jing Zhang, Anna Uehara, Anna Montmayeur, Clinton R. Paden, Peter W. Cook,Rachel Marine, Mili Sheth, Haibin Wang, Justin Lee, Suxiang Tong
EPI_ISL_751560	NYSDOH Wadsworth Center, Virology Lab	Genomics and Discovery, Respiratory Viruses Branch, Division of Viral Diseases, Centers for Disease Control and Prevention	Krista Queen, Yan Li, Ying Tao, Jing Zhang, Anna Uehara, Anna Montmayeur, Clinton R. Paden, Peter W. Cook,Rachel Marine, Mili Sheth, Haibin Wang, Justin Lee, Suxiang Tong
EPI_ISL_751561	MT Public Health Laboratory	Genomics and Discovery, Respiratory Viruses Branch, Division of Viral Diseases, Centers for Disease Control and Prevention	Krista Queen, Yan Li, Ying Tao, Jing Zhang, Anna Uehara, Anna Montmayeur, Clinton R. Paden, Peter W. Cook,Rachel Marine, Mili Sheth, Haibin Wang, Justin Lee, Suxiang Tong
EPI_ISL_751562	NYSDOH Wadsworth Center, Virology Lab	Genomics and Discovery, Respiratory Viruses Branch, Division of Viral Diseases, Centers for Disease Control and Prevention	Krista Queen, Yan Li, Ying Tao, Jing Zhang, Anna Uehara, Anna Montmayeur, Clinton R. Paden, Peter W. Cook,Rachel Marine, Mili Sheth, Haibin Wang, Justin Lee, Suxiang Tong
EPI_ISL_751563	NE Public Health Laboratory	Genomics and Discovery, Respiratory Viruses Branch, Division of Viral Diseases, Centers for Disease Control and Prevention	Krista Queen, Yan Li, Ying Tao, Jing Zhang, Anna Uehara, Anna Montmayeur, Clinton R. Paden, Peter W. Cook,Rachel Marine, Mili Sheth, Haibin Wang, Justin Lee, Suxiang Tong
EPI_ISL_751564	VA-Division of Consolidated Laboratory Services	Genomics and Discovery, Respiratory Viruses Branch, Division of Viral Diseases, Centers for Disease Control and Prevention	Krista Queen, Yan Li, Ying Tao, Jing Zhang, Anna Uehara, Anna Montmayeur, Clinton R. Paden, Peter W. Cook,Rachel Marine, Mili Sheth, Haibin Wang, Justin Lee, Suxiang Tong
EPI_ISL_751565	IA State Hygienic Laboratory	Genomics and Discovery, Respiratory Viruses Branch, Division of Viral Diseases, Centers for Disease Control and Prevention	Krista Queen, Yan Li, Ying Tao, Jing Zhang, Anna Uehara, Anna Montmayeur, Clinton R. Paden, Peter W. Cook,Rachel Marine, Mili Sheth, Haibin Wang, Justin Lee, Suxiang Tong
EPI_ISL_751566	WVDHHR - Office of Laboratory Services	Genomics and Discovery, Respiratory Viruses Branch, Division of Viral Diseases, Centers for Disease Control and Prevention	Krista Queen, Yan Li, Ying Tao, Jing Zhang, Anna Uehara, Anna Montmayeur, Clinton R. Paden, Peter W. Cook,Rachel Marine, Mili Sheth, Haibin Wang, Justin Lee, Suxiang Tong
EPI_ISL_751567	ID Bureau of Laboratories	Genomics and Discovery, Respiratory Viruses Branch, Division of Viral Diseases, Centers for Disease Control and Prevention	Krista Queen, Yan Li, Ying Tao, Jing Zhang, Anna Uehara, Anna Montmayeur, Clinton R. Paden, Peter W. Cook,Rachel Marine, Mili Sheth, Haibin Wang, Justin Lee, Suxiang Tong
EPI_ISL_751568	MS Public Health Laboratory	Genomics and Discovery, Respiratory Viruses Branch, Division of Viral Diseases, Centers for Disease Control and Prevention	Krista Queen, Yan Li, Ying Tao, Jing Zhang, Anna Uehara, Anna Montmayeur, Clinton R. Paden, Peter W. Cook,Rachel Marine, Mili Sheth, Haibin Wang, Justin Lee, Suxiang Tong
EPI_ISL_751569	MI - Michigan Department of Health and Human Services - Bureau of Laboratories	Genomics and Discovery, Respiratory Viruses Branch, Division of Viral Diseases, Centers for Disease Control and Prevention	Krista Queen, Yan Li, Ying Tao, Jing Zhang, Anna Uehara, Anna Montmayeur, Clinton R. Paden, Peter W. Cook,Rachel Marine, Mili Sheth, Haibin Wang, Justin Lee, Suxiang Tong
EPI_ISL_751570	KY State Public Health Lab	Genomics and Discovery, Respiratory Viruses Branch, Division of Viral	Krista Queen, Yan Li, Ying Tao, Jing Zhang, Anna Uehara, Anna Montmayeur, Clinton R. Paden, Peter W. Cook,Rachel Marine, Mili Sheth, Haibin Wang, Justin Lee, Suxiang Tong

		Diseases, Centers for Disease Control and Prevention	
EPI_ISL_751571	TX DSHS, Lab Services Section MC 1947	Genomics and Discovery, Respiratory Viruses Branch, Division of Viral Diseases, Centers for Disease Control and Prevention	Krista Queen, Yan Li, Ying Tao, Jing Zhang, Anna Uehara, Anna Montmayeur, Clinton R. Paden, Peter W. Cook,Rachel Marine, Mili Sheth, Haibin Wang, Justin Lee, Suxiang Tong
EPI_ISL_751572	KS Health and Environmental Laboratories	Genomics and Discovery, Respiratory Viruses Branch, Division of Viral Diseases, Centers for Disease Control and Prevention	Krista Queen, Yan Li, Ying Tao, Jing Zhang, Anna Uehara, Anna Montmayeur, Clinton R. Paden, Peter W. Cook,Rachel Marine, Mili Sheth, Haibin Wang, Justin Lee, Suxiang Tong
EPI_ISL_751573	MN PHL Division, Minnesota Department of Health	Genomics and Discovery, Respiratory Viruses Branch, Division of Viral Diseases, Centers for Disease Control and Prevention	Krista Queen, Yan Li, Ying Tao, Jing Zhang, Anna Uehara, Anna Montmayeur, Clinton R. Paden, Peter W. Cook,Rachel Marine, Mili Sheth, Haibin Wang, Justin Lee, Suxiang Tong
EPI_ISL_751574	AR Dept. of Health-Public Health Lab	Genomics and Discovery, Respiratory Viruses Branch, Division of Viral Diseases, Centers for Disease Control and Prevention	Krista Queen, Yan Li, Ying Tao, Jing Zhang, Anna Uehara, Anna Montmayeur, Clinton R. Paden, Peter W. Cook,Rachel Marine, Mili Sheth, Haibin Wang, Justin Lee, Suxiang Tong
EPI_ISL_751575	CO Dept. of Public Health and Environment, Lab Services Division	Genomics and Discovery, Respiratory Viruses Branch, Division of Viral Diseases, Centers for Disease Control and Prevention	Krista Queen, Yan Li, Ying Tao, Jing Zhang, Anna Uehara, Anna Montmayeur, Clinton R. Paden, Peter W. Cook,Rachel Marine, Mili Sheth, Haibin Wang, Justin Lee, Suxiang Tong
EPI_ISL_751576	MN PHL Division, Minnesota Department of Health	Genomics and Discovery, Respiratory Viruses Branch, Division of Viral Diseases, Centers for Disease Control and Prevention	Krista Queen, Yan Li, Ying Tao, Jing Zhang, Anna Uehara, Anna Montmayeur, Clinton R. Paden, Peter W. Cook,Rachel Marine, Mili Sheth, Haibin Wang, Justin Lee, Suxiang Tong
EPI_ISL_751577	MD DOH Laboratories Administration	Genomics and Discovery, Respiratory Viruses Branch, Division of Viral Diseases, Centers for Disease Control and Prevention	Krista Queen, Yan Li, Ying Tao, Jing Zhang, Anna Uehara, Anna Montmayeur, Clinton R. Paden, Peter W. Cook,Rachel Marine, Mili Sheth, Haibin Wang, Justin Lee, Suxiang Tong
EPI_ISL_751578	HI Dept. of Health, State Laboratories Division	Genomics and Discovery, Respiratory Viruses Branch, Division of Viral Diseases, Centers for Disease Control and Prevention	Krista Queen, Yan Li, Ying Tao, Jing Zhang, Anna Uehara, Anna Montmayeur, Clinton R. Paden, Peter W. Cook,Rachel Marine, Mili Sheth, Haibin Wang, Justin Lee, Suxiang Tong
EPI_ISL_751579	IN State Department of Health Laboratory Services	Genomics and Discovery, Respiratory Viruses Branch, Division of Viral Diseases, Centers for Disease Control and Prevention	Krista Queen, Yan Li, Ying Tao, Jing Zhang, Anna Uehara, Anna Montmayeur, Clinton R. Paden, Peter W. Cook,Rachel Marine, Mili Sheth, Haibin Wang, Justin Lee, Suxiang Tong
EPI_ISL_751580, EPI_ISL_751581	NYSDOH Wadsworth Center, Virology Lab	Genomics and Discovery, Respiratory Viruses Branch, Division of Viral Diseases, Centers for Disease Control and Prevention	Krista Queen, Yan Li, Ying Tao, Jing Zhang, Anna Uehara, Anna Montmayeur, Clinton R. Paden, Peter W. Cook,Rachel Marine, Mili Sheth, Haibin Wang, Justin Lee, Suxiang Tong
EPI_ISL_751582	NH Dept. of Health and Human Services Public Health Labs	Genomics and Discovery, Respiratory Viruses Branch, Division of Viral Diseases, Centers for Disease Control and Prevention	Krista Queen, Yan Li, Ying Tao, Jing Zhang, Anna Uehara, Anna Montmayeur, Clinton R. Paden, Peter W. Cook,Rachel Marine, Mili Sheth, Haibin Wang, Justin Lee, Suxiang Tong
EPI_ISL_751583	NE Public Health Laboratory	Genomics and Discovery, Respiratory Viruses Branch, Division of Viral Diseases, Centers for Disease Control and Prevention	Krista Queen, Yan Li, Ying Tao, Jing Zhang, Anna Uehara, Anna Montmayeur, Clinton R. Paden, Peter W. Cook,Rachel Marine, Mili Sheth, Haibin Wang, Justin Lee, Suxiang Tong
EPI_ISL_751584	NH Dept. of Health and Human Services Public Health Labs	Genomics and Discovery, Respiratory Viruses Branch, Division of Viral Diseases, Centers for Disease Control and Prevention	Krista Queen, Yan Li, Ying Tao, Jing Zhang, Anna Uehara, Anna Montmayeur, Clinton R. Paden, Peter W. Cook,Rachel Marine, Mili Sheth, Haibin Wang, Justin Lee, Suxiang Tong

EPI_ISL_751585	NE Public Health Laboratory	Genomics and Discovery, Respiratory Viruses Branch, Division of Viral Diseases, Centers for Disease Control and Prevention	Krista Queen, Yan Li, Ying Tao, Jing Zhang, Anna Uehara, Anna Montmayeur, Clinton R. Paden, Peter W. Cook,Rachel Marine, Mili Sheth, Haibin Wang, Justin Lee, Suxiang Tong
EPI_ISL_751586	OH Department of Health Laboratory	Genomics and Discovery, Respiratory Viruses Branch, Division of Viral Diseases, Centers for Disease Control and Prevention	Krista Queen, Yan Li, Ying Tao, Jing Zhang, Anna Uehara, Anna Montmayeur, Clinton R. Paden, Peter W. Cook,Rachel Marine, Mili Sheth, Haibin Wang, Justin Lee, Suxiang Tong
EPI_ISL_751587	NH Dept. of Health and Human Services Public Health Labs	Genomics and Discovery, Respiratory Viruses Branch, Division of Viral Diseases, Centers for Disease Control and Prevention	Krista Queen, Yan Li, Ying Tao, Jing Zhang, Anna Uehara, Anna Montmayeur, Clinton R. Paden, Peter W. Cook,Rachel Marine, Mili Sheth, Haibin Wang, Justin Lee, Suxiang Tong
EPI_ISL_751588	VA-Division of Consolidated Laboratory Services	Genomics and Discovery, Respiratory Viruses Branch, Division of Viral Diseases, Centers for Disease Control and Prevention	Krista Queen, Yan Li, Ying Tao, Jing Zhang, Anna Uehara, Anna Montmayeur, Clinton R. Paden, Peter W. Cook,Rachel Marine, Mili Sheth, Haibin Wang, Justin Lee, Suxiang Tong
EPI_ISL_751589	WVDHHR - Office of Laboratory Services	Genomics and Discovery, Respiratory Viruses Branch, Division of Viral Diseases, Centers for Disease Control and Prevention	Krista Queen, Yan Li, Ying Tao, Jing Zhang, Anna Uehara, Anna Montmayeur, Clinton R. Paden, Peter W. Cook,Rachel Marine, Mili Sheth, Haibin Wang, Justin Lee, Suxiang Tong
EPI_ISL_751590	ID Bureau of Laboratories	Genomics and Discovery, Respiratory Viruses Branch, Division of Viral Diseases, Centers for Disease Control and Prevention	Krista Queen, Yan Li, Ying Tao, Jing Zhang, Anna Uehara, Anna Montmayeur, Clinton R. Paden, Peter W. Cook,Rachel Marine, Mili Sheth, Haibin Wang, Justin Lee, Suxiang Tong
EPI_ISL_751591	WY Public Health Laboratory	Genomics and Discovery, Respiratory Viruses Branch, Division of Viral Diseases, Centers for Disease Control and Prevention	Krista Queen, Yan Li, Ying Tao, Jing Zhang, Anna Uehara, Anna Montmayeur, Clinton R. Paden, Peter W. Cook,Rachel Marine, Mili Sheth, Haibin Wang, Justin Lee, Suxiang Tong
EPI_ISL_751592, EPI_ISL_751593	AK State Public Health Lab, State Health Department	Genomics and Discovery, Respiratory Viruses Branch, Division of Viral Diseases, Centers for Disease Control and Prevention	Krista Queen, Yan Li, Ying Tao, Jing Zhang, Anna Uehara, Anna Montmayeur, Clinton R. Paden, Peter W. Cook,Rachel Marine, Mili Sheth, Haibin Wang, Justin Lee, Suxiang Tong
EPI_ISL_751594	KY State Public Health Lab	Genomics and Discovery, Respiratory Viruses Branch, Division of Viral Diseases, Centers for Disease Control and Prevention	Krista Queen, Yan Li, Ying Tao, Jing Zhang, Anna Uehara, Anna Montmayeur, Clinton R. Paden, Peter W. Cook,Rachel Marine, Mili Sheth, Haibin Wang, Justin Lee, Suxiang Tong
EPI_ISL_751595	RI State Health Laboratories	Genomics and Discovery, Respiratory Viruses Branch, Division of Viral Diseases, Centers for Disease Control and Prevention	Krista Queen, Yan Li, Ying Tao, Jing Zhang, Anna Uehara, Anna Montmayeur, Clinton R. Paden, Peter W. Cook,Rachel Marine, Mili Sheth, Haibin Wang, Justin Lee, Suxiang Tong
EPI_ISL_751596	KY State Public Health Lab	Genomics and Discovery, Respiratory Viruses Branch, Division of Viral Diseases, Centers for Disease Control and Prevention	Krista Queen, Yan Li, Ying Tao, Jing Zhang, Anna Uehara, Anna Montmayeur, Clinton R. Paden, Peter W. Cook,Rachel Marine, Mili Sheth, Haibin Wang, Justin Lee, Suxiang Tong
EPI_ISL_751597, EPI_ISL_751598	AK State Public Health Lab, State Health Department	Genomics and Discovery, Respiratory Viruses Branch, Division of Viral Diseases, Centers for Disease Control and Prevention	Krista Queen, Yan Li, Ying Tao, Jing Zhang, Anna Uehara, Anna Montmayeur, Clinton R. Paden, Peter W. Cook,Rachel Marine, Mili Sheth, Haibin Wang, Justin Lee, Suxiang Tong
EPI_ISL_751599, EPI_ISL_751600	IN State Department of Health Laboratory Services	Genomics and Discovery, Respiratory Viruses Branch, Division of Viral Diseases, Centers for Disease Control and Prevention	Krista Queen, Yan Li, Ying Tao, Jing Zhang, Anna Uehara, Anna Montmayeur, Clinton R. Paden, Peter W. Cook,Rachel Marine, Mili Sheth, Haibin Wang, Justin Lee, Suxiang Tong
EPI_ISL_751601	MI - Michigan Department of Health and Human Services - Bureau of Laboratories	Genomics and Discovery, Respiratory Viruses Branch, Division of Viral	Krista Queen, Yan Li, Ying Tao, Jing Zhang, Anna Uehara, Anna Montmayeur, Clinton R. Paden, Peter W. Cook,Rachel Marine, Mili Sheth, Haibin Wang, Justin Lee, Suxiang Tong

		Diseases, Centers for Disease Control and Prevention	
EPI_ISL_751602	DE Public Health Laboratory	Genomics and Discovery, Respiratory Viruses Branch, Division of Viral Diseases, Centers for Disease Control and Prevention	Krista Queen, Yan Li, Ying Tao, Jing Zhang, Anna Uehara, Anna Montmayeur, Clinton R. Paden, Peter W. Cook,Rachel Marine, Mili Sheth, Haibin Wang, Justin Lee, Suxiang Tong
EPI_ISL_751603	HI Dept. of Health, State Laboratories Division	Genomics and Discovery, Respiratory Viruses Branch, Division of Viral Diseases, Centers for Disease Control and Prevention	Krista Queen, Yan Li, Ying Tao, Jing Zhang, Anna Uehara, Anna Montmayeur, Clinton R. Paden, Peter W. Cook,Rachel Marine, Mili Sheth, Haibin Wang, Justin Lee, Suxiang Tong
EPI_ISL_751604	MD DOH Laboratories Administration	Genomics and Discovery, Respiratory Viruses Branch, Division of Viral Diseases, Centers for Disease Control and Prevention	Krista Queen, Yan Li, Ying Tao, Jing Zhang, Anna Uehara, Anna Montmayeur, Clinton R. Paden, Peter W. Cook,Rachel Marine, Mili Sheth, Haibin Wang, Justin Lee, Suxiang Tong
EPI_ISL_751605	DE Public Health Laboratory	Genomics and Discovery, Respiratory Viruses Branch, Division of Viral Diseases, Centers for Disease Control and Prevention	Krista Queen, Yan Li, Ying Tao, Jing Zhang, Anna Uehara, Anna Montmayeur, Clinton R. Paden, Peter W. Cook,Rachel Marine, Mili Sheth, Haibin Wang, Justin Lee, Suxiang Tong
EPI_ISL_751606, EPI_ISL_751607	MD DOH Laboratories Administration	Genomics and Discovery, Respiratory Viruses Branch, Division of Viral Diseases, Centers for Disease Control and Prevention	Krista Queen, Yan Li, Ying Tao, Jing Zhang, Anna Uehara, Anna Montmayeur, Clinton R. Paden, Peter W. Cook,Rachel Marine, Mili Sheth, Haibin Wang, Justin Lee, Suxiang Tong
EPI_ISL_751608	NE Public Health Laboratory	Genomics and Discovery, Respiratory Viruses Branch, Division of Viral Diseases, Centers for Disease Control and Prevention	Krista Queen, Yan Li, Ying Tao, Jing Zhang, Anna Uehara, Anna Montmayeur, Clinton R. Paden, Peter W. Cook,Rachel Marine, Mili Sheth, Haibin Wang, Justin Lee, Suxiang Tong
EPI_ISL_751609, EPI_ISL_751610	OH Department of Health Laboratory	Genomics and Discovery, Respiratory Viruses Branch, Division of Viral Diseases, Centers for Disease Control and Prevention	Krista Queen, Yan Li, Ying Tao, Jing Zhang, Anna Uehara, Anna Montmayeur, Clinton R. Paden, Peter W. Cook,Rachel Marine, Mili Sheth, Haibin Wang, Justin Lee, Suxiang Tong
EPI_ISL_751611	SC Dept of Health and Env. Control-Bureau of Laboratories	Genomics and Discovery, Respiratory Viruses Branch, Division of Viral Diseases, Centers for Disease Control and Prevention	Krista Queen, Yan Li, Ying Tao, Jing Zhang, Anna Uehara, Anna Montmayeur, Clinton R. Paden, Peter W. Cook,Rachel Marine, Mili Sheth, Haibin Wang, Justin Lee, Suxiang Tong
EPI_ISL_751612	ID Bureau of Laboratories	Genomics and Discovery, Respiratory Viruses Branch, Division of Viral Diseases, Centers for Disease Control and Prevention	Krista Queen, Yan Li, Ying Tao, Jing Zhang, Anna Uehara, Anna Montmayeur, Clinton R. Paden, Peter W. Cook,Rachel Marine, Mili Sheth, Haibin Wang, Justin Lee, Suxiang Tong
EPI_ISL_751613	OH Department of Health Laboratory	Genomics and Discovery, Respiratory Viruses Branch, Division of Viral Diseases, Centers for Disease Control and Prevention	Krista Queen, Yan Li, Ying Tao, Jing Zhang, Anna Uehara, Anna Montmayeur, Clinton R. Paden, Peter W. Cook,Rachel Marine, Mili Sheth, Haibin Wang, Justin Lee, Suxiang Tong
EPI_ISL_751614	ID Bureau of Laboratories	Genomics and Discovery, Respiratory Viruses Branch, Division of Viral Diseases, Centers for Disease Control and Prevention	Krista Queen, Yan Li, Ying Tao, Jing Zhang, Anna Uehara, Anna Montmayeur, Clinton R. Paden, Peter W. Cook,Rachel Marine, Mili Sheth, Haibin Wang, Justin Lee, Suxiang Tong
EPI_ISL_751615	OR State PHL-Virology/Immunology Section	Genomics and Discovery, Respiratory Viruses Branch, Division of Viral Diseases, Centers for Disease Control and Prevention	Krista Queen, Yan Li, Ying Tao, Jing Zhang, Anna Uehara, Anna Montmayeur, Clinton R. Paden, Peter W. Cook,Rachel Marine, Mili Sheth, Haibin Wang, Justin Lee, Suxiang Tong
EPI_ISL_751616	OH Department of Health Laboratory	Genomics and Discovery, Respiratory Viruses Branch, Division of Viral Diseases, Centers for Disease Control and Prevention	Krista Queen, Yan Li, Ying Tao, Jing Zhang, Anna Uehara, Anna Montmayeur, Clinton R. Paden, Peter W. Cook,Rachel Marine, Mili Sheth, Haibin Wang, Justin Lee, Suxiang Tong

EPI_ISL_751617	TX DSHS, Lab Services Section MC 1947	Genomics and Discovery, Respiratory Viruses Branch, Division of Viral Diseases, Centers for Disease Control and Prevention	Krista Queen, Yan Li, Ying Tao, Jing Zhang, Anna Uehara, Anna Montmayeur, Clinton R. Paden, Peter W. Cook,Rachel Marine, Mili Sheth, Haibin Wang, Justin Lee, Suxiang Tong
EPI_ISL_751618	ID Bureau of Laboratories	Genomics and Discovery, Respiratory Viruses Branch, Division of Viral Diseases, Centers for Disease Control and Prevention	Krista Queen, Yan Li, Ying Tao, Jing Zhang, Anna Uehara, Anna Montmayeur, Clinton R. Paden, Peter W. Cook,Rachel Marine, Mili Sheth, Haibin Wang, Justin Lee, Suxiang Tong
EPI_ISL_751619	WY Public Health Laboratory	Genomics and Discovery, Respiratory Viruses Branch, Division of Viral Diseases, Centers for Disease Control and Prevention	Krista Queen, Yan Li, Ying Tao, Jing Zhang, Anna Uehara, Anna Montmayeur, Clinton R. Paden, Peter W. Cook,Rachel Marine, Mili Sheth, Haibin Wang, Justin Lee, Suxiang Tong
EPI_ISL_751620	LA Office of Public Health Laboratories	Genomics and Discovery, Respiratory Viruses Branch, Division of Viral Diseases, Centers for Disease Control and Prevention	Krista Queen, Yan Li, Ying Tao, Jing Zhang, Anna Uehara, Anna Montmayeur, Clinton R. Paden, Peter W. Cook,Rachel Marine, Mili Sheth, Haibin Wang, Justin Lee, Suxiang Tong
EPI_ISL_751621	DE Public Health Laboratory	Genomics and Discovery, Respiratory Viruses Branch, Division of Viral Diseases, Centers for Disease Control and Prevention	Krista Queen, Yan Li, Ying Tao, Jing Zhang, Anna Uehara, Anna Montmayeur, Clinton R. Paden, Peter W. Cook,Rachel Marine, Mili Sheth, Haibin Wang, Justin Lee, Suxiang Tong
EPI_ISL_751622	MI - Michigan Department of Health and Human Services - Bureau of Laboratories	Genomics and Discovery, Respiratory Viruses Branch, Division of Viral Diseases, Centers for Disease Control and Prevention	Krista Queen, Yan Li, Ying Tao, Jing Zhang, Anna Uehara, Anna Montmayeur, Clinton R. Paden, Peter W. Cook,Rachel Marine, Mili Sheth, Haibin Wang, Justin Lee, Suxiang Tong
EPI_ISL_751623	DE Public Health Laboratory	Genomics and Discovery, Respiratory Viruses Branch, Division of Viral Diseases, Centers for Disease Control and Prevention	Krista Queen, Yan Li, Ying Tao, Jing Zhang, Anna Uehara, Anna Montmayeur, Clinton R. Paden, Peter W. Cook,Rachel Marine, Mili Sheth, Haibin Wang, Justin Lee, Suxiang Tong
EPI_ISL_751624	CT-Dr. Katherine A. Kelley State Public Health Lab	Genomics and Discovery, Respiratory Viruses Branch, Division of Viral Diseases, Centers for Disease Control and Prevention	Krista Queen, Yan Li, Ying Tao, Jing Zhang, Anna Uehara, Anna Montmayeur, Clinton R. Paden, Peter W. Cook,Rachel Marine, Mili Sheth, Haibin Wang, Justin Lee, Suxiang Tong
EPI_ISL_751625	MD DOH Laboratories Administration	Genomics and Discovery, Respiratory Viruses Branch, Division of Viral Diseases, Centers for Disease Control and Prevention	Krista Queen, Yan Li, Ying Tao, Jing Zhang, Anna Uehara, Anna Montmayeur, Clinton R. Paden, Peter W. Cook,Rachel Marine, Mili Sheth, Haibin Wang, Justin Lee, Suxiang Tong
EPI_ISL_751626	IN State Department of Health Laboratory Services	Genomics and Discovery, Respiratory Viruses Branch, Division of Viral Diseases, Centers for Disease Control and Prevention	Krista Queen, Yan Li, Ying Tao, Jing Zhang, Anna Uehara, Anna Montmayeur, Clinton R. Paden, Peter W. Cook,Rachel Marine, Mili Sheth, Haibin Wang, Justin Lee, Suxiang Tong
EPI_ISL_751627	DE Public Health Laboratory	Genomics and Discovery, Respiratory Viruses Branch, Division of Viral Diseases, Centers for Disease Control and Prevention	Krista Queen, Yan Li, Ying Tao, Jing Zhang, Anna Uehara, Anna Montmayeur, Clinton R. Paden, Peter W. Cook,Rachel Marine, Mili Sheth, Haibin Wang, Justin Lee, Suxiang Tong
EPI_ISL_751628, EPI_ISL_751629	MD DOH Laboratories Administration	Genomics and Discovery, Respiratory Viruses Branch, Division of Viral Diseases, Centers for Disease Control and Prevention	Krista Queen, Yan Li, Ying Tao, Jing Zhang, Anna Uehara, Anna Montmayeur, Clinton R. Paden, Peter W. Cook,Rachel Marine, Mili Sheth, Haibin Wang, Justin Lee, Suxiang Tong
EPI_ISL_751630	CO Dept. of Public Health and Environment, Lab Services Division	Genomics and Discovery, Respiratory Viruses Branch, Division of Viral Diseases, Centers for Disease Control and Prevention	Krista Queen, Yan Li, Ying Tao, Jing Zhang, Anna Uehara, Anna Montmayeur, Clinton R. Paden, Peter W. Cook,Rachel Marine, Mili Sheth, Haibin Wang, Justin Lee, Suxiang Tong
EPI_ISL_751631	DE Public Health Laboratory	Genomics and Discovery, Respiratory Viruses Branch, Division of Viral	Krista Queen, Yan Li, Ying Tao, Jing Zhang, Anna Uehara, Anna Montmayeur, Clinton R. Paden, Peter W. Cook,Rachel Marine, Mili Sheth, Haibin Wang, Justin Lee, Suxiang Tong

		Diseases, Centers for Disease Control and Prevention	
EPI_ISL_751632	IN State Department of Health Laboratory Services	Genomics and Discovery, Respiratory Viruses Branch, Division of Viral Diseases, Centers for Disease Control and Prevention	Krista Queen, Yan Li, Ying Tao, Jing Zhang, Anna Uehara, Anna Montmayeur, Clinton R. Paden, Peter W. Cook,Rachel Marine, Mili Sheth, Haibin Wang, Justin Lee, Suxiang Tong
EPI_ISL_751633	MT Public Health Laboratory	Genomics and Discovery, Respiratory Viruses Branch, Division of Viral Diseases, Centers for Disease Control and Prevention	Krista Queen, Yan Li, Ying Tao, Jing Zhang, Anna Uehara, Anna Montmayeur, Clinton R. Paden, Peter W. Cook,Rachel Marine, Mili Sheth, Haibin Wang, Justin Lee, Suxiang Tong
EPI_ISL_751634, EPI_ISL_751635	MO State Public Health Laboratory	Genomics and Discovery, Respiratory Viruses Branch, Division of Viral Diseases, Centers for Disease Control and Prevention	Krista Queen, Yan Li, Ying Tao, Jing Zhang, Anna Uehara, Anna Montmayeur, Clinton R. Paden, Peter W. Cook,Rachel Marine, Mili Sheth, Haibin Wang, Justin Lee, Suxiang Tong
EPI_ISL_751636, EPI_ISL_751637	NV State Public Health Laboratory	Genomics and Discovery, Respiratory Viruses Branch, Division of Viral Diseases, Centers for Disease Control and Prevention	Krista Queen, Yan Li, Ying Tao, Jing Zhang, Anna Uehara, Anna Montmayeur, Clinton R. Paden, Peter W. Cook,Rachel Marine, Mili Sheth, Haibin Wang, Justin Lee, Suxiang Tong
EPI_ISL_751638	NE Public Health Laboratory	Genomics and Discovery, Respiratory Viruses Branch, Division of Viral Diseases, Centers for Disease Control and Prevention	Krista Queen, Yan Li, Ying Tao, Jing Zhang, Anna Uehara, Anna Montmayeur, Clinton R. Paden, Peter W. Cook,Rachel Marine, Mili Sheth, Haibin Wang, Justin Lee, Suxiang Tong
EPI_ISL_751639	NV State Public Health Laboratory	Genomics and Discovery, Respiratory Viruses Branch, Division of Viral Diseases, Centers for Disease Control and Prevention	Krista Queen, Yan Li, Ying Tao, Jing Zhang, Anna Uehara, Anna Montmayeur, Clinton R. Paden, Peter W. Cook,Rachel Marine, Mili Sheth, Haibin Wang, Justin Lee, Suxiang Tong
EPI_ISL_751640	MT Public Health Laboratory	Genomics and Discovery, Respiratory Viruses Branch, Division of Viral Diseases, Centers for Disease Control and Prevention	Krista Queen, Yan Li, Ying Tao, Jing Zhang, Anna Uehara, Anna Montmayeur, Clinton R. Paden, Peter W. Cook,Rachel Marine, Mili Sheth, Haibin Wang, Justin Lee, Suxiang Tong
EPI_ISL_751641	MN PHL Division, Minnesota Department of Health	Genomics and Discovery, Respiratory Viruses Branch, Division of Viral Diseases, Centers for Disease Control and Prevention	Krista Queen, Yan Li, Ying Tao, Jing Zhang, Anna Uehara, Anna Montmayeur, Clinton R. Paden, Peter W. Cook,Rachel Marine, Mili Sheth, Haibin Wang, Justin Lee, Suxiang Tong
EPI_ISL_751642	NH Dept. of Health and Human Services Public Health Labs	Genomics and Discovery, Respiratory Viruses Branch, Division of Viral Diseases, Centers for Disease Control and Prevention	Krista Queen, Yan Li, Ying Tao, Jing Zhang, Anna Uehara, Anna Montmayeur, Clinton R. Paden, Peter W. Cook,Rachel Marine, Mili Sheth, Haibin Wang, Justin Lee, Suxiang Tong
EPI_ISL_751643	WY Public Health Laboratory	Genomics and Discovery, Respiratory Viruses Branch, Division of Viral Diseases, Centers for Disease Control and Prevention	Krista Queen, Yan Li, Ying Tao, Jing Zhang, Anna Uehara, Anna Montmayeur, Clinton R. Paden, Peter W. Cook,Rachel Marine, Mili Sheth, Haibin Wang, Justin Lee, Suxiang Tong
EPI_ISL_751644, EPI_ISL_751645, EPI_ISL_751646	TX DSHS, Lab Services Section MC 1947	Genomics and Discovery, Respiratory Viruses Branch, Division of Viral Diseases, Centers for Disease Control and Prevention	Krista Queen, Yan Li, Ying Tao, Jing Zhang, Anna Uehara, Anna Montmayeur, Clinton R. Paden, Peter W. Cook,Rachel Marine, Mili Sheth, Haibin Wang, Justin Lee, Suxiang Tong
EPI_ISL_751647	AL Dept. of Public Health Bureau of Clinical Laboratories	Genomics and Discovery, Respiratory Viruses Branch, Division of Viral Diseases, Centers for Disease Control and Prevention	Krista Queen, Yan Li, Ying Tao, Jing Zhang, Anna Uehara, Anna Montmayeur, Clinton R. Paden, Peter W. Cook,Rachel Marine, Mili Sheth, Haibin Wang, Justin Lee, Suxiang Tong
EPI_ISL_751648	KY State Public Health Lab	Genomics and Discovery, Respiratory Viruses Branch, Division of Viral Diseases, Centers for Disease Control and Prevention	Krista Queen, Yan Li, Ying Tao, Jing Zhang, Anna Uehara, Anna Montmayeur, Clinton R. Paden, Peter W. Cook,Rachel Marine, Mili Sheth, Haibin Wang, Justin Lee, Suxiang Tong

EPI_ISL_751649, EPI_ISL_751650	CT-Dr. Katherine A. Kelley State Public Health Lab	Genomics and Discovery, Respiratory Viruses Branch, Division of Viral Diseases, Centers for Disease Control and Prevention	Krista Queen, Yan Li, Ying Tao, Jing Zhang, Anna Uehara, Anna Montmayeur, Clinton R. Paden, Peter W. Cook,Rachel Marine, Mili Sheth, Haibin Wang, Justin Lee, Suxiang Tong
EPI_ISL_751651	HI Dept. of Health, State Laboratories Division	Genomics and Discovery, Respiratory Viruses Branch, Division of Viral Diseases, Centers for Disease Control and Prevention	Krista Queen, Yan Li, Ying Tao, Jing Zhang, Anna Uehara, Anna Montmayeur, Clinton R. Paden, Peter W. Cook,Rachel Marine, Mili Sheth, Haibin Wang, Justin Lee, Suxiang Tong
EPI_ISL_751652	AR Dept. of Health-Public Health Lab	Genomics and Discovery, Respiratory Viruses Branch, Division of Viral Diseases, Centers for Disease Control and Prevention	Krista Queen, Yan Li, Ying Tao, Jing Zhang, Anna Uehara, Anna Montmayeur, Clinton R. Paden, Peter W. Cook,Rachel Marine, Mili Sheth, Haibin Wang, Justin Lee, Suxiang Tong
EPI_ISL_751653, EPI_ISL_751654, EPI_ISL_751655	MN PHL Division, Minnesota Department of Health	Genomics and Discovery, Respiratory Viruses Branch, Division of Viral Diseases, Centers for Disease Control and Prevention	Krista Queen, Yan Li, Ying Tao, Jing Zhang, Anna Uehara, Anna Montmayeur, Clinton R. Paden, Peter W. Cook,Rachel Marine, Mili Sheth, Haibin Wang, Justin Lee, Suxiang Tong
EPI_ISL_751656, EPI_ISL_751657	MO State Public Health Laboratory	Genomics and Discovery, Respiratory Viruses Branch, Division of Viral Diseases, Centers for Disease Control and Prevention	Krista Queen, Yan Li, Ying Tao, Jing Zhang, Anna Uehara, Anna Montmayeur, Clinton R. Paden, Peter W. Cook,Rachel Marine, Mili Sheth, Haibin Wang, Justin Lee, Suxiang Tong
EPI_ISL_751658	NYSDOH Wadsworth Center, Virology Lab	Genomics and Discovery, Respiratory Viruses Branch, Division of Viral Diseases, Centers for Disease Control and Prevention	Krista Queen, Yan Li, Ying Tao, Jing Zhang, Anna Uehara, Anna Montmayeur, Clinton R. Paden, Peter W. Cook,Rachel Marine, Mili Sheth, Haibin Wang, Justin Lee, Suxiang Tong
EPI_ISL_751659	NJ Public Health and Environmental Laboratories	Genomics and Discovery, Respiratory Viruses Branch, Division of Viral Diseases, Centers for Disease Control and Prevention	Krista Queen, Yan Li, Ying Tao, Jing Zhang, Anna Uehara, Anna Montmayeur, Clinton R. Paden, Peter W. Cook,Rachel Marine, Mili Sheth, Haibin Wang, Justin Lee, Suxiang Tong
EPI_ISL_751660	MN PHL Division, Minnesota Department of Health	Genomics and Discovery, Respiratory Viruses Branch, Division of Viral Diseases, Centers for Disease Control and Prevention	Krista Queen, Yan Li, Ying Tao, Jing Zhang, Anna Uehara, Anna Montmayeur, Clinton R. Paden, Peter W. Cook,Rachel Marine, Mili Sheth, Haibin Wang, Justin Lee, Suxiang Tong
EPI_ISL_751661	MS Public Health Laboratory	Genomics and Discovery, Respiratory Viruses Branch, Division of Viral Diseases, Centers for Disease Control and Prevention	Krista Queen, Yan Li, Ying Tao, Jing Zhang, Anna Uehara, Anna Montmayeur, Clinton R. Paden, Peter W. Cook,Rachel Marine, Mili Sheth, Haibin Wang, Justin Lee, Suxiang Tong
EPI_ISL_751662	NJ Public Health and Environmental Laboratories	Genomics and Discovery, Respiratory Viruses Branch, Division of Viral Diseases, Centers for Disease Control and Prevention	Krista Queen, Yan Li, Ying Tao, Jing Zhang, Anna Uehara, Anna Montmayeur, Clinton R. Paden, Peter W. Cook,Rachel Marine, Mili Sheth, Haibin Wang, Justin Lee, Suxiang Tong
EPI_ISL_751663	WY Public Health Laboratory	Genomics and Discovery, Respiratory Viruses Branch, Division of Viral Diseases, Centers for Disease Control and Prevention	Krista Queen, Yan Li, Ying Tao, Jing Zhang, Anna Uehara, Anna Montmayeur, Clinton R. Paden, Peter W. Cook,Rachel Marine, Mili Sheth, Haibin Wang, Justin Lee, Suxiang Tong
EPI_ISL_751664, EPI_ISL_751665	SC Dept of Health and Env. Control-Bureau of Laboratories	Genomics and Discovery, Respiratory Viruses Branch, Division of Viral Diseases, Centers for Disease Control and Prevention	Krista Queen, Yan Li, Ying Tao, Jing Zhang, Anna Uehara, Anna Montmayeur, Clinton R. Paden, Peter W. Cook,Rachel Marine, Mili Sheth, Haibin Wang, Justin Lee, Suxiang Tong
EPI_ISL_751666	IA State Hygienic Laboratory	Genomics and Discovery, Respiratory Viruses Branch, Division of Viral Diseases, Centers for Disease Control and Prevention	Krista Queen, Yan Li, Ying Tao, Jing Zhang, Anna Uehara, Anna Montmayeur, Clinton R. Paden, Peter W. Cook,Rachel Marine, Mili Sheth, Haibin Wang, Justin Lee, Suxiang Tong
EPI_ISL_751667	RI State Health Laboratories	Genomics and Discovery, Respiratory Viruses Branch, Division of Viral	Krista Queen, Yan Li, Ying Tao, Jing Zhang, Anna Uehara, Anna Montmayeur, Clinton R. Paden, Peter W. Cook,Rachel Marine, Mili Sheth, Haibin Wang, Justin Lee, Suxiang Tong

		Diseases, Centers for Disease Control and Prevention	
EPI_ISL_751668	AL Dept. of Public Health Bureau of Clinical Laboratories	Genomics and Discovery, Respiratory Viruses Branch, Division of Viral Diseases, Centers for Disease Control and Prevention	Krista Queen, Yan Li, Ying Tao, Jing Zhang, Anna Uehara, Anna Montmayeur, Clinton R. Paden, Peter W. Cook,Rachel Marine, Mili Sheth, Haibin Wang, Justin Lee, Suxiang Tong
EPI_ISL_751669	LA Office of Public Health Laboratories	Genomics and Discovery, Respiratory Viruses Branch, Division of Viral Diseases, Centers for Disease Control and Prevention	Krista Queen, Yan Li, Ying Tao, Jing Zhang, Anna Uehara, Anna Montmayeur, Clinton R. Paden, Peter W. Cook,Rachel Marine, Mili Sheth, Haibin Wang, Justin Lee, Suxiang Tong
EPI_ISL_751670	KY State Public Health Lab	Genomics and Discovery, Respiratory Viruses Branch, Division of Viral Diseases, Centers for Disease Control and Prevention	Krista Queen, Yan Li, Ying Tao, Jing Zhang, Anna Uehara, Anna Montmayeur, Clinton R. Paden, Peter W. Cook,Rachel Marine, Mili Sheth, Haibin Wang, Justin Lee, Suxiang Tong
EPI_ISL_751671	RI State Health Laboratories	Genomics and Discovery, Respiratory Viruses Branch, Division of Viral Diseases, Centers for Disease Control and Prevention	Krista Queen, Yan Li, Ying Tao, Jing Zhang, Anna Uehara, Anna Montmayeur, Clinton R. Paden, Peter W. Cook,Rachel Marine, Mili Sheth, Haibin Wang, Justin Lee, Suxiang Tong
EPI_ISL_751672	IN State Department of Health Laboratory Services	Genomics and Discovery, Respiratory Viruses Branch, Division of Viral Diseases, Centers for Disease Control and Prevention	Krista Queen, Yan Li, Ying Tao, Jing Zhang, Anna Uehara, Anna Montmayeur, Clinton R. Paden, Peter W. Cook,Rachel Marine, Mili Sheth, Haibin Wang, Justin Lee, Suxiang Tong
EPI_ISL_751673, EPI_ISL_751674, EPI_ISL_751675	HI Dept. of Health, State Laboratories Division	Genomics and Discovery, Respiratory Viruses Branch, Division of Viral Diseases, Centers for Disease Control and Prevention	Krista Queen, Yan Li, Ying Tao, Jing Zhang, Anna Uehara, Anna Montmayeur, Clinton R. Paden, Peter W. Cook,Rachel Marine, Mili Sheth, Haibin Wang, Justin Lee, Suxiang Tong
EPI_ISL_751676	IN State Department of Health Laboratory Services	Genomics and Discovery, Respiratory Viruses Branch, Division of Viral Diseases, Centers for Disease Control and Prevention	Krista Queen, Yan Li, Ying Tao, Jing Zhang, Anna Uehara, Anna Montmayeur, Clinton R. Paden, Peter W. Cook,Rachel Marine, Mili Sheth, Haibin Wang, Justin Lee, Suxiang Tong
EPI_ISL_751677	KS Health and Environmental Laboratories	Genomics and Discovery, Respiratory Viruses Branch, Division of Viral Diseases, Centers for Disease Control and Prevention	Krista Queen, Yan Li, Ying Tao, Jing Zhang, Anna Uehara, Anna Montmayeur, Clinton R. Paden, Peter W. Cook,Rachel Marine, Mili Sheth, Haibin Wang, Justin Lee, Suxiang Tong
EPI_ISL_751678	NH Dept. of Health and Human Services Public Health Labs	Genomics and Discovery, Respiratory Viruses Branch, Division of Viral Diseases, Centers for Disease Control and Prevention	Krista Queen, Yan Li, Ying Tao, Jing Zhang, Anna Uehara, Anna Montmayeur, Clinton R. Paden, Peter W. Cook,Rachel Marine, Mili Sheth, Haibin Wang, Justin Lee, Suxiang Tong
EPI_ISL_751679	MS Public Health Laboratory	Genomics and Discovery, Respiratory Viruses Branch, Division of Viral Diseases, Centers for Disease Control and Prevention	Krista Queen, Yan Li, Ying Tao, Jing Zhang, Anna Uehara, Anna Montmayeur, Clinton R. Paden, Peter W. Cook,Rachel Marine, Mili Sheth, Haibin Wang, Justin Lee, Suxiang Tong
EPI_ISL_751680	NJ Public Health and Environmental Laboratories	Genomics and Discovery, Respiratory Viruses Branch, Division of Viral Diseases, Centers for Disease Control and Prevention	Krista Queen, Yan Li, Ying Tao, Jing Zhang, Anna Uehara, Anna Montmayeur, Clinton R. Paden, Peter W. Cook,Rachel Marine, Mili Sheth, Haibin Wang, Justin Lee, Suxiang Tong
EPI_ISL_751681	NE Public Health Laboratory	Genomics and Discovery, Respiratory Viruses Branch, Division of Viral Diseases, Centers for Disease Control and Prevention	Krista Queen, Yan Li, Ying Tao, Jing Zhang, Anna Uehara, Anna Montmayeur, Clinton R. Paden, Peter W. Cook,Rachel Marine, Mili Sheth, Haibin Wang, Justin Lee, Suxiang Tong
EPI_ISL_751682	NYSDOH Wadsworth Center, Virology Lab	Genomics and Discovery, Respiratory Viruses Branch, Division of Viral Diseases, Centers for Disease Control and Prevention	Krista Queen, Yan Li, Ying Tao, Jing Zhang, Anna Uehara, Anna Montmayeur, Clinton R. Paden, Peter W. Cook,Rachel Marine, Mili Sheth, Haibin Wang, Justin Lee, Suxiang Tong

EPI_ISL_751683	MS Public Health Laboratory	Genomics and Discovery, Respiratory Viruses Branch, Division of Viral Diseases, Centers for Disease Control and Prevention	Krista Queen, Yan Li, Ying Tao, Jing Zhang, Anna Uehara, Anna Montmayeur, Clinton R. Paden, Peter W. Cook,Rachel Marine, Mili Sheth, Haibin Wang, Justin Lee, Suxiang Tong
EPI_ISL_751684	TX DSHS, Lab Services Section MC 1947	Genomics and Discovery, Respiratory Viruses Branch, Division of Viral Diseases, Centers for Disease Control and Prevention	Krista Queen, Yan Li, Ying Tao, Jing Zhang, Anna Uehara, Anna Montmayeur, Clinton R. Paden, Peter W. Cook,Rachel Marine, Mili Sheth, Haibin Wang, Justin Lee, Suxiang Tong
EPI_ISL_751685, EPI_ISL_751686	VA-Division of Consolidated Laboratory Services	Genomics and Discovery, Respiratory Viruses Branch, Division of Viral Diseases, Centers for Disease Control and Prevention	Krista Queen, Yan Li, Ying Tao, Jing Zhang, Anna Uehara, Anna Montmayeur, Clinton R. Paden, Peter W. Cook,Rachel Marine, Mili Sheth, Haibin Wang, Justin Lee, Suxiang Tong
EPI_ISL_751687	IA State Hygienic Laboratory	Genomics and Discovery, Respiratory Viruses Branch, Division of Viral Diseases, Centers for Disease Control and Prevention	Krista Queen, Yan Li, Ying Tao, Jing Zhang, Anna Uehara, Anna Montmayeur, Clinton R. Paden, Peter W. Cook,Rachel Marine, Mili Sheth, Haibin Wang, Justin Lee, Suxiang Tong
EPI_ISL_751688	WY Public Health Laboratory	Genomics and Discovery, Respiratory Viruses Branch, Division of Viral Diseases, Centers for Disease Control and Prevention	Krista Queen, Yan Li, Ying Tao, Jing Zhang, Anna Uehara, Anna Montmayeur, Clinton R. Paden, Peter W. Cook,Rachel Marine, Mili Sheth, Haibin Wang, Justin Lee, Suxiang Tong
EPI_ISL_751689	OR State PHL-Virology/Immunology Section	Genomics and Discovery, Respiratory Viruses Branch, Division of Viral Diseases, Centers for Disease Control and Prevention	Krista Queen, Yan Li, Ying Tao, Jing Zhang, Anna Uehara, Anna Montmayeur, Clinton R. Paden, Peter W. Cook,Rachel Marine, Mili Sheth, Haibin Wang, Justin Lee, Suxiang Tong
EPI_ISL_751690	VA-Division of Consolidated Laboratory Services	Genomics and Discovery, Respiratory Viruses Branch, Division of Viral Diseases, Centers for Disease Control and Prevention	Krista Queen, Yan Li, Ying Tao, Jing Zhang, Anna Uehara, Anna Montmayeur, Clinton R. Paden, Peter W. Cook,Rachel Marine, Mili Sheth, Haibin Wang, Justin Lee, Suxiang Tong
EPI_ISL_751691	WY Public Health Laboratory	Genomics and Discovery, Respiratory Viruses Branch, Division of Viral Diseases, Centers for Disease Control and Prevention	Krista Queen, Yan Li, Ying Tao, Jing Zhang, Anna Uehara, Anna Montmayeur, Clinton R. Paden, Peter W. Cook,Rachel Marine, Mili Sheth, Haibin Wang, Justin Lee, Suxiang Tong
EPI_ISL_751692	IA State Hygienic Laboratory	Genomics and Discovery, Respiratory Viruses Branch, Division of Viral Diseases, Centers for Disease Control and Prevention	Krista Queen, Yan Li, Ying Tao, Jing Zhang, Anna Uehara, Anna Montmayeur, Clinton R. Paden, Peter W. Cook,Rachel Marine, Mili Sheth, Haibin Wang, Justin Lee, Suxiang Tong
EPI_ISL_751693	OR State PHL-Virology/Immunology Section	Genomics and Discovery, Respiratory Viruses Branch, Division of Viral Diseases, Centers for Disease Control and Prevention	Krista Queen, Yan Li, Ying Tao, Jing Zhang, Anna Uehara, Anna Montmayeur, Clinton R. Paden, Peter W. Cook,Rachel Marine, Mili Sheth, Haibin Wang, Justin Lee, Suxiang Tong
EPI_ISL_751694	AK State Public Health Lab, State Health Department	Genomics and Discovery, Respiratory Viruses Branch, Division of Viral Diseases, Centers for Disease Control and Prevention	Krista Queen, Yan Li, Ying Tao, Jing Zhang, Anna Uehara, Anna Montmayeur, Clinton R. Paden, Peter W. Cook,Rachel Marine, Mili Sheth, Haibin Wang, Justin Lee, Suxiang Tong
EPI_ISL_751695	RI State Health Laboratories	Genomics and Discovery, Respiratory Viruses Branch, Division of Viral Diseases, Centers for Disease Control and Prevention	Krista Queen, Yan Li, Ying Tao, Jing Zhang, Anna Uehara, Anna Montmayeur, Clinton R. Paden, Peter W. Cook,Rachel Marine, Mili Sheth, Haibin Wang, Justin Lee, Suxiang Tong
EPI_ISL_751696	LA Office of Public Health Laboratories	Genomics and Discovery, Respiratory Viruses Branch, Division of Viral Diseases, Centers for Disease Control and Prevention	Krista Queen, Yan Li, Ying Tao, Jing Zhang, Anna Uehara, Anna Montmayeur, Clinton R. Paden, Peter W. Cook,Rachel Marine, Mili Sheth, Haibin Wang, Justin Lee, Suxiang Tong
EPI_ISL_751697, EPI_ISL_751698, EPI_ISL_751699	AR Dept. of Health-Public Health Lab	Genomics and Discovery, Respiratory Viruses Branch, Division of Viral	Krista Queen, Yan Li, Ying Tao, Jing Zhang, Anna Uehara, Anna Montmayeur, Clinton R. Paden, Peter W. Cook,Rachel Marine, Mili Sheth, Haibin Wang, Justin Lee, Suxiang Tong

		Diseases, Centers for Disease Control and Prevention	
EPI_ISL_751700	MD DOH Laboratories Administration	Genomics and Discovery, Respiratory Viruses Branch, Division of Viral Diseases, Centers for Disease Control and Prevention	Krista Queen, Yan Li, Ying Tao, Jing Zhang, Anna Uehara, Anna Montmayeur, Clinton R. Paden, Peter W. Cook,Rachel Marine, Mili Sheth, Haibin Wang, Justin Lee, Suxiang Tong
EPI_ISL_751701	CO Dept. of Public Health and Environment, Lab Services Division	Genomics and Discovery, Respiratory Viruses Branch, Division of Viral Diseases, Centers for Disease Control and Prevention	Krista Queen, Yan Li, Ying Tao, Jing Zhang, Anna Uehara, Anna Montmayeur, Clinton R. Paden, Peter W. Cook,Rachel Marine, Mili Sheth, Haibin Wang, Justin Lee, Suxiang Tong
EPI_ISL_751702, EPI_ISL_751703	MD DOH Laboratories Administration	Genomics and Discovery, Respiratory Viruses Branch, Division of Viral Diseases, Centers for Disease Control and Prevention	Krista Queen, Yan Li, Ying Tao, Jing Zhang, Anna Uehara, Anna Montmayeur, Clinton R. Paden, Peter W. Cook,Rachel Marine, Mili Sheth, Haibin Wang, Justin Lee, Suxiang Tong
EPI_ISL_751704, EPI_ISL_751705	CO Dept. of Public Health and Environment, Lab Services Division	Genomics and Discovery, Respiratory Viruses Branch, Division of Viral Diseases, Centers for Disease Control and Prevention	Krista Queen, Yan Li, Ying Tao, Jing Zhang, Anna Uehara, Anna Montmayeur, Clinton R. Paden, Peter W. Cook,Rachel Marine, Mili Sheth, Haibin Wang, Justin Lee, Suxiang Tong
EPI_ISL_751706	CT-Dr. Katherine A. Kelley State Public Health Lab	Genomics and Discovery, Respiratory Viruses Branch, Division of Viral Diseases, Centers for Disease Control and Prevention	Krista Queen, Yan Li, Ying Tao, Jing Zhang, Anna Uehara, Anna Montmayeur, Clinton R. Paden, Peter W. Cook,Rachel Marine, Mili Sheth, Haibin Wang, Justin Lee, Suxiang Tong
EPI_ISL_751707	KS Health and Environmental Laboratories	Genomics and Discovery, Respiratory Viruses Branch, Division of Viral Diseases, Centers for Disease Control and Prevention	Krista Queen, Yan Li, Ying Tao, Jing Zhang, Anna Uehara, Anna Montmayeur, Clinton R. Paden, Peter W. Cook,Rachel Marine, Mili Sheth, Haibin Wang, Justin Lee, Suxiang Tong
EPI_ISL_751708	NV State Public Health Laboratory	Genomics and Discovery, Respiratory Viruses Branch, Division of Viral Diseases, Centers for Disease Control and Prevention	Krista Queen, Yan Li, Ying Tao, Jing Zhang, Anna Uehara, Anna Montmayeur, Clinton R. Paden, Peter W. Cook,Rachel Marine, Mili Sheth, Haibin Wang, Justin Lee, Suxiang Tong
EPI_ISL_751709	NJ Public Health and Environmental Laboratories	Genomics and Discovery, Respiratory Viruses Branch, Division of Viral Diseases, Centers for Disease Control and Prevention	Krista Queen, Yan Li, Ying Tao, Jing Zhang, Anna Uehara, Anna Montmayeur, Clinton R. Paden, Peter W. Cook,Rachel Marine, Mili Sheth, Haibin Wang, Justin Lee, Suxiang Tong
EPI_ISL_751710, EPI_ISL_751711	MT Public Health Laboratory	Genomics and Discovery, Respiratory Viruses Branch, Division of Viral Diseases, Centers for Disease Control and Prevention	Krista Queen, Yan Li, Ying Tao, Jing Zhang, Anna Uehara, Anna Montmayeur, Clinton R. Paden, Peter W. Cook,Rachel Marine, Mili Sheth, Haibin Wang, Justin Lee, Suxiang Tong
EPI_ISL_751712	NH Dept. of Health and Human Services Public Health Labs	Genomics and Discovery, Respiratory Viruses Branch, Division of Viral Diseases, Centers for Disease Control and Prevention	Krista Queen, Yan Li, Ying Tao, Jing Zhang, Anna Uehara, Anna Montmayeur, Clinton R. Paden, Peter W. Cook,Rachel Marine, Mili Sheth, Haibin Wang, Justin Lee, Suxiang Tong
EPI_ISL_751713	MT Public Health Laboratory	Genomics and Discovery, Respiratory Viruses Branch, Division of Viral Diseases, Centers for Disease Control and Prevention	Krista Queen, Yan Li, Ying Tao, Jing Zhang, Anna Uehara, Anna Montmayeur, Clinton R. Paden, Peter W. Cook,Rachel Marine, Mili Sheth, Haibin Wang, Justin Lee, Suxiang Tong
EPI_ISL_751714	NV State Public Health Laboratory	Genomics and Discovery, Respiratory Viruses Branch, Division of Viral Diseases, Centers for Disease Control and Prevention	Krista Queen, Yan Li, Ying Tao, Jing Zhang, Anna Uehara, Anna Montmayeur, Clinton R. Paden, Peter W. Cook,Rachel Marine, Mili Sheth, Haibin Wang, Justin Lee, Suxiang Tong
EPI_ISL_751715	MO State Public Health Laboratory	Genomics and Discovery, Respiratory Viruses Branch, Division of Viral Diseases, Centers for Disease Control and Prevention	Krista Queen, Yan Li, Ying Tao, Jing Zhang, Anna Uehara, Anna Montmayeur, Clinton R. Paden, Peter W. Cook,Rachel Marine, Mili Sheth, Haibin Wang, Justin Lee, Suxiang Tong

EPI_ISL_751716	MN PHL Division, Minnesota Department of Health	Genomics and Discovery, Respiratory Viruses Branch, Division of Viral Diseases, Centers for Disease Control and Prevention	Krista Queen, Yan Li, Ying Tao, Jing Zhang, Anna Uehara, Anna Montmayeur, Clinton R. Paden, Peter W. Cook,Rachel Marine, Mili Sheth, Haibin Wang, Justin Lee, Suxiang Tong
EPI_ISL_751717	NYSDOH Wadsworth Center, Virology Lab	Genomics and Discovery, Respiratory Viruses Branch, Division of Viral Diseases, Centers for Disease Control and Prevention	Krista Queen, Yan Li, Ying Tao, Jing Zhang, Anna Uehara, Anna Montmayeur, Clinton R. Paden, Peter W. Cook,Rachel Marine, Mili Sheth, Haibin Wang, Justin Lee, Suxiang Tong
EPI_ISL_751718	SC Dept of Health and Env. Control-Bureau of Laboratories	Genomics and Discovery, Respiratory Viruses Branch, Division of Viral Diseases, Centers for Disease Control and Prevention	Krista Queen, Yan Li, Ying Tao, Jing Zhang, Anna Uehara, Anna Montmayeur, Clinton R. Paden, Peter W. Cook,Rachel Marine, Mili Sheth, Haibin Wang, Justin Lee, Suxiang Tong
EPI_ISL_751719	ID Bureau of Laboratories	Genomics and Discovery, Respiratory Viruses Branch, Division of Viral Diseases, Centers for Disease Control and Prevention	Krista Queen, Yan Li, Ying Tao, Jing Zhang, Anna Uehara, Anna Montmayeur, Clinton R. Paden, Peter W. Cook,Rachel Marine, Mili Sheth, Haibin Wang, Justin Lee, Suxiang Tong
EPI_ISL_751720	TX DSHS, Lab Services Section MC 1947	Genomics and Discovery, Respiratory Viruses Branch, Division of Viral Diseases, Centers for Disease Control and Prevention	Krista Queen, Yan Li, Ying Tao, Jing Zhang, Anna Uehara, Anna Montmayeur, Clinton R. Paden, Peter W. Cook,Rachel Marine, Mili Sheth, Haibin Wang, Justin Lee, Suxiang Tong
EPI_ISL_751721	IA State Hygienic Laboratory	Genomics and Discovery, Respiratory Viruses Branch, Division of Viral Diseases, Centers for Disease Control and Prevention	Krista Queen, Yan Li, Ying Tao, Jing Zhang, Anna Uehara, Anna Montmayeur, Clinton R. Paden, Peter W. Cook,Rachel Marine, Mili Sheth, Haibin Wang, Justin Lee, Suxiang Tong
EPI_ISL_751722	LA Office of Public Health Laboratories	Genomics and Discovery, Respiratory Viruses Branch, Division of Viral Diseases, Centers for Disease Control and Prevention	Krista Queen, Yan Li, Ying Tao, Jing Zhang, Anna Uehara, Anna Montmayeur, Clinton R. Paden, Peter W. Cook,Rachel Marine, Mili Sheth, Haibin Wang, Justin Lee, Suxiang Tong
EPI_ISL_751723	AR Dept. of Health-Public Health Lab	Genomics and Discovery, Respiratory Viruses Branch, Division of Viral Diseases, Centers for Disease Control and Prevention	Krista Queen, Yan Li, Ying Tao, Jing Zhang, Anna Uehara, Anna Montmayeur, Clinton R. Paden, Peter W. Cook,Rachel Marine, Mili Sheth, Haibin Wang, Justin Lee, Suxiang Tong
EPI_ISL_751724	KS Health and Environmental Laboratories	Genomics and Discovery, Respiratory Viruses Branch, Division of Viral Diseases, Centers for Disease Control and Prevention	Krista Queen, Yan Li, Ying Tao, Jing Zhang, Anna Uehara, Anna Montmayeur, Clinton R. Paden, Peter W. Cook,Rachel Marine, Mili Sheth, Haibin Wang, Justin Lee, Suxiang Tong
EPI_ISL_751725	MD DOH Laboratories Administration	Genomics and Discovery, Respiratory Viruses Branch, Division of Viral Diseases, Centers for Disease Control and Prevention	Krista Queen, Yan Li, Ying Tao, Jing Zhang, Anna Uehara, Anna Montmayeur, Clinton R. Paden, Peter W. Cook,Rachel Marine, Mili Sheth, Haibin Wang, Justin Lee, Suxiang Tong
EPI_ISL_751726	DE Public Health Laboratory	Genomics and Discovery, Respiratory Viruses Branch, Division of Viral Diseases, Centers for Disease Control and Prevention	Krista Queen, Yan Li, Ying Tao, Jing Zhang, Anna Uehara, Anna Montmayeur, Clinton R. Paden, Peter W. Cook,Rachel Marine, Mili Sheth, Haibin Wang, Justin Lee, Suxiang Tong
EPI_ISL_751727	IN State Department of Health Laboratory Services	Genomics and Discovery, Respiratory Viruses Branch, Division of Viral Diseases, Centers for Disease Control and Prevention	Krista Queen, Yan Li, Ying Tao, Jing Zhang, Anna Uehara, Anna Montmayeur, Clinton R. Paden, Peter W. Cook,Rachel Marine, Mili Sheth, Haibin Wang, Justin Lee, Suxiang Tong
EPI_ISL_751728	CO Dept. of Public Health and Environment, Lab Services Division	Genomics and Discovery, Respiratory Viruses Branch, Division of Viral Diseases, Centers for Disease Control and Prevention	Krista Queen, Yan Li, Ying Tao, Jing Zhang, Anna Uehara, Anna Montmayeur, Clinton R. Paden, Peter W. Cook,Rachel Marine, Mili Sheth, Haibin Wang, Justin Lee, Suxiang Tong
EPI_ISL_751729	CT-Dr. Katherine A. Kelley State Public Health Lab	Genomics and Discovery, Respiratory Viruses Branch, Division of Viral	Krista Queen, Yan Li, Ying Tao, Jing Zhang, Anna Uehara, Anna Montmayeur, Clinton R. Paden, Peter W. Cook,Rachel Marine, Mili Sheth, Haibin Wang, Justin Lee, Suxiang Tong

		Diseases, Centers for Disease Control and Prevention	
EPI_ISL_751730	KS Health and Environmental Laboratories	Genomics and Discovery, Respiratory Viruses Branch, Division of Viral Diseases, Centers for Disease Control and Prevention	Krista Queen, Yan Li, Ying Tao, Jing Zhang, Anna Uehara, Anna Montmayeur, Clinton R. Paden, Peter W. Cook,Rachel Marine, Mili Sheth, Haibin Wang, Justin Lee, Suxiang Tong
EPI_ISL_751731	AR Dept. of Health-Public Health Lab	Genomics and Discovery, Respiratory Viruses Branch, Division of Viral Diseases, Centers for Disease Control and Prevention	Krista Queen, Yan Li, Ying Tao, Jing Zhang, Anna Uehara, Anna Montmayeur, Clinton R. Paden, Peter W. Cook,Rachel Marine, Mili Sheth, Haibin Wang, Justin Lee, Suxiang Tong
EPI_ISL_751732	NE Public Health Laboratory	Genomics and Discovery, Respiratory Viruses Branch, Division of Viral Diseases, Centers for Disease Control and Prevention	Krista Queen, Yan Li, Ying Tao, Jing Zhang, Anna Uehara, Anna Montmayeur, Clinton R. Paden, Peter W. Cook,Rachel Marine, Mili Sheth, Haibin Wang, Justin Lee, Suxiang Tong
EPI_ISL_751733	MN PHL Division, Minnesota Department of Health	Genomics and Discovery, Respiratory Viruses Branch, Division of Viral Diseases, Centers for Disease Control and Prevention	Krista Queen, Yan Li, Ying Tao, Jing Zhang, Anna Uehara, Anna Montmayeur, Clinton R. Paden, Peter W. Cook,Rachel Marine, Mili Sheth, Haibin Wang, Justin Lee, Suxiang Tong
EPI_ISL_751734	NE Public Health Laboratory	Genomics and Discovery, Respiratory Viruses Branch, Division of Viral Diseases, Centers for Disease Control and Prevention	Krista Queen, Yan Li, Ying Tao, Jing Zhang, Anna Uehara, Anna Montmayeur, Clinton R. Paden, Peter W. Cook,Rachel Marine, Mili Sheth, Haibin Wang, Justin Lee, Suxiang Tong
EPI_ISL_751735	NH Dept. of Health and Human Services Public Health Labs	Genomics and Discovery, Respiratory Viruses Branch, Division of Viral Diseases, Centers for Disease Control and Prevention	Krista Queen, Yan Li, Ying Tao, Jing Zhang, Anna Uehara, Anna Montmayeur, Clinton R. Paden, Peter W. Cook,Rachel Marine, Mili Sheth, Haibin Wang, Justin Lee, Suxiang Tong
EPI_ISL_751736	NV State Public Health Laboratory	Genomics and Discovery, Respiratory Viruses Branch, Division of Viral Diseases, Centers for Disease Control and Prevention	Krista Queen, Yan Li, Ying Tao, Jing Zhang, Anna Uehara, Anna Montmayeur, Clinton R. Paden, Peter W. Cook,Rachel Marine, Mili Sheth, Haibin Wang, Justin Lee, Suxiang Tong
EPI_ISL_751737	MO State Public Health Laboratory	Genomics and Discovery, Respiratory Viruses Branch, Division of Viral Diseases, Centers for Disease Control and Prevention	Krista Queen, Yan Li, Ying Tao, Jing Zhang, Anna Uehara, Anna Montmayeur, Clinton R. Paden, Peter W. Cook,Rachel Marine, Mili Sheth, Haibin Wang, Justin Lee, Suxiang Tong
EPI_ISL_751738	MT Public Health Laboratory	Genomics and Discovery, Respiratory Viruses Branch, Division of Viral Diseases, Centers for Disease Control and Prevention	Krista Queen, Yan Li, Ying Tao, Jing Zhang, Anna Uehara, Anna Montmayeur, Clinton R. Paden, Peter W. Cook,Rachel Marine, Mili Sheth, Haibin Wang, Justin Lee, Suxiang Tong
EPI_ISL_751739	MN PHL Division, Minnesota Department of Health	Genomics and Discovery, Respiratory Viruses Branch, Division of Viral Diseases, Centers for Disease Control and Prevention	Krista Queen, Yan Li, Ying Tao, Jing Zhang, Anna Uehara, Anna Montmayeur, Clinton R. Paden, Peter W. Cook,Rachel Marine, Mili Sheth, Haibin Wang, Justin Lee, Suxiang Tong
EPI_ISL_751740	MO State Public Health Laboratory	Genomics and Discovery, Respiratory Viruses Branch, Division of Viral Diseases, Centers for Disease Control and Prevention	Krista Queen, Yan Li, Ying Tao, Jing Zhang, Anna Uehara, Anna Montmayeur, Clinton R. Paden, Peter W. Cook,Rachel Marine, Mili Sheth, Haibin Wang, Justin Lee, Suxiang Tong
EPI_ISL_751741	OH Department of Health Laboratory	Genomics and Discovery, Respiratory Viruses Branch, Division of Viral Diseases, Centers for Disease Control and Prevention	Krista Queen, Yan Li, Ying Tao, Jing Zhang, Anna Uehara, Anna Montmayeur, Clinton R. Paden, Peter W. Cook,Rachel Marine, Mili Sheth, Haibin Wang, Justin Lee, Suxiang Tong
EPI_ISL_751742	IA State Hygienic Laboratory	Genomics and Discovery, Respiratory Viruses Branch, Division of Viral Diseases, Centers for Disease Control and Prevention	Krista Queen, Yan Li, Ying Tao, Jing Zhang, Anna Uehara, Anna Montmayeur, Clinton R. Paden, Peter W. Cook,Rachel Marine, Mili Sheth, Haibin Wang, Justin Lee, Suxiang Tong

EPI_ISL_751743	WY Public Health Laboratory	Genomics and Discovery, Respiratory Viruses Branch, Division of Viral Diseases, Centers for Disease Control and Prevention	Krista Queen, Yan Li, Ying Tao, Jing Zhang, Anna Uehara, Anna Montmayeur, Clinton R. Paden, Peter W. Cook,Rachel Marine, Mili Sheth, Haibin Wang, Justin Lee, Suxiang Tong
EPI_ISL_751744	SC Dept of Health and Env. Control-Bureau of Laboratories	Genomics and Discovery, Respiratory Viruses Branch, Division of Viral Diseases, Centers for Disease Control and Prevention	Krista Queen, Yan Li, Ying Tao, Jing Zhang, Anna Uehara, Anna Montmayeur, Clinton R. Paden, Peter W. Cook,Rachel Marine, Mili Sheth, Haibin Wang, Justin Lee, Suxiang Tong
EPI_ISL_751745	VA-Division of Consolidated Laboratory Services	Genomics and Discovery, Respiratory Viruses Branch, Division of Viral Diseases, Centers for Disease Control and Prevention	Krista Queen, Yan Li, Ying Tao, Jing Zhang, Anna Uehara, Anna Montmayeur, Clinton R. Paden, Peter W. Cook,Rachel Marine, Mili Sheth, Haibin Wang, Justin Lee, Suxiang Tong
EPI_ISL_751746	WVDHHR - Office of Laboratory Services	Genomics and Discovery, Respiratory Viruses Branch, Division of Viral Diseases, Centers for Disease Control and Prevention	Krista Queen, Yan Li, Ying Tao, Jing Zhang, Anna Uehara, Anna Montmayeur, Clinton R. Paden, Peter W. Cook,Rachel Marine, Mili Sheth, Haibin Wang, Justin Lee, Suxiang Tong
EPI_ISL_751747	VA-Division of Consolidated Laboratory Services	Genomics and Discovery, Respiratory Viruses Branch, Division of Viral Diseases, Centers for Disease Control and Prevention	Krista Queen, Yan Li, Ying Tao, Jing Zhang, Anna Uehara, Anna Montmayeur, Clinton R. Paden, Peter W. Cook,Rachel Marine, Mili Sheth, Haibin Wang, Justin Lee, Suxiang Tong
EPI_ISL_751748	ID Bureau of Laboratories	Genomics and Discovery, Respiratory Viruses Branch, Division of Viral Diseases, Centers for Disease Control and Prevention	Krista Queen, Yan Li, Ying Tao, Jing Zhang, Anna Uehara, Anna Montmayeur, Clinton R. Paden, Peter W. Cook,Rachel Marine, Mili Sheth, Haibin Wang, Justin Lee, Suxiang Tong
EPI_ISL_751749	TX DSHS, Lab Services Section MC 1947	Genomics and Discovery, Respiratory Viruses Branch, Division of Viral Diseases, Centers for Disease Control and Prevention	Krista Queen, Yan Li, Ying Tao, Jing Zhang, Anna Uehara, Anna Montmayeur, Clinton R. Paden, Peter W. Cook,Rachel Marine, Mili Sheth, Haibin Wang, Justin Lee, Suxiang Tong
EPI_ISL_751750	ID Bureau of Laboratories	Genomics and Discovery, Respiratory Viruses Branch, Division of Viral Diseases, Centers for Disease Control and Prevention	Krista Queen, Yan Li, Ying Tao, Jing Zhang, Anna Uehara, Anna Montmayeur, Clinton R. Paden, Peter W. Cook,Rachel Marine, Mili Sheth, Haibin Wang, Justin Lee, Suxiang Tong
EPI_ISL_751751	RI State Health Laboratories	Genomics and Discovery, Respiratory Viruses Branch, Division of Viral Diseases, Centers for Disease Control and Prevention	Krista Queen, Yan Li, Ying Tao, Jing Zhang, Anna Uehara, Anna Montmayeur, Clinton R. Paden, Peter W. Cook,Rachel Marine, Mili Sheth, Haibin Wang, Justin Lee, Suxiang Tong
EPI_ISL_751752	LA Office of Public Health Laboratories	Genomics and Discovery, Respiratory Viruses Branch, Division of Viral Diseases, Centers for Disease Control and Prevention	Krista Queen, Yan Li, Ying Tao, Jing Zhang, Anna Uehara, Anna Montmayeur, Clinton R. Paden, Peter W. Cook,Rachel Marine, Mili Sheth, Haibin Wang, Justin Lee, Suxiang Tong
EPI_ISL_751753	AL Dept. of Public Health Bureau of Clinical Laboratories	Genomics and Discovery, Respiratory Viruses Branch, Division of Viral Diseases, Centers for Disease Control and Prevention	Krista Queen, Yan Li, Ying Tao, Jing Zhang, Anna Uehara, Anna Montmayeur, Clinton R. Paden, Peter W. Cook,Rachel Marine, Mili Sheth, Haibin Wang, Justin Lee, Suxiang Tong
EPI_ISL_751754	KY State Public Health Lab	Genomics and Discovery, Respiratory Viruses Branch, Division of Viral Diseases, Centers for Disease Control and Prevention	Krista Queen, Yan Li, Ying Tao, Jing Zhang, Anna Uehara, Anna Montmayeur, Clinton R. Paden, Peter W. Cook,Rachel Marine, Mili Sheth, Haibin Wang, Justin Lee, Suxiang Tong
EPI_ISL_751755, EPI_ISL_751756	AL Dept. of Public Health Bureau of Clinical Laboratories	Genomics and Discovery, Respiratory Viruses Branch, Division of Viral Diseases, Centers for Disease Control and Prevention	Krista Queen, Yan Li, Ying Tao, Jing Zhang, Anna Uehara, Anna Montmayeur, Clinton R. Paden, Peter W. Cook,Rachel Marine, Mili Sheth, Haibin Wang, Justin Lee, Suxiang Tong
EPI_ISL_751757	RI State Health Laboratories	Genomics and Discovery, Respiratory Viruses Branch, Division of Viral	Krista Queen, Yan Li, Ying Tao, Jing Zhang, Anna Uehara, Anna Montmayeur, Clinton R. Paden, Peter W. Cook,Rachel Marine, Mili Sheth, Haibin Wang, Justin Lee, Suxiang Tong

		Diseases, Centers for Disease Control and Prevention	
EPI_ISL_751758	HI Dept. of Health, State Laboratories Division	Genomics and Discovery, Respiratory Viruses Branch, Division of Viral Diseases, Centers for Disease Control and Prevention	Krista Queen, Yan Li, Ying Tao, Jing Zhang, Anna Uehara, Anna Montmayeur, Clinton R. Paden, Peter W. Cook,Rachel Marine, Mili Sheth, Haibin Wang, Justin Lee, Suxiang Tong
EPI_ISL_751759	CT-Dr. Katherine A. Kelley State Public Health Lab	Genomics and Discovery, Respiratory Viruses Branch, Division of Viral Diseases, Centers for Disease Control and Prevention	Krista Queen, Yan Li, Ying Tao, Jing Zhang, Anna Uehara, Anna Montmayeur, Clinton R. Paden, Peter W. Cook,Rachel Marine, Mili Sheth, Haibin Wang, Justin Lee, Suxiang Tong
EPI_ISL_751760	AR Dept. of Health-Public Health Lab	Genomics and Discovery, Respiratory Viruses Branch, Division of Viral Diseases, Centers for Disease Control and Prevention	Krista Queen, Yan Li, Ying Tao, Jing Zhang, Anna Uehara, Anna Montmayeur, Clinton R. Paden, Peter W. Cook,Rachel Marine, Mili Sheth, Haibin Wang, Justin Lee, Suxiang Tong
EPI_ISL_751761	HI Dept. of Health, State Laboratories Division	Genomics and Discovery, Respiratory Viruses Branch, Division of Viral Diseases, Centers for Disease Control and Prevention	Krista Queen, Yan Li, Ying Tao, Jing Zhang, Anna Uehara, Anna Montmayeur, Clinton R. Paden, Peter W. Cook,Rachel Marine, Mili Sheth, Haibin Wang, Justin Lee, Suxiang Tong
EPI_ISL_751762	CT-Dr. Katherine A. Kelley State Public Health Lab	Genomics and Discovery, Respiratory Viruses Branch, Division of Viral Diseases, Centers for Disease Control and Prevention	Krista Queen, Yan Li, Ying Tao, Jing Zhang, Anna Uehara, Anna Montmayeur, Clinton R. Paden, Peter W. Cook,Rachel Marine, Mili Sheth, Haibin Wang, Justin Lee, Suxiang Tong
EPI_ISL_751763	CO Dept. of Public Health and Environment, Lab Services Division	Genomics and Discovery, Respiratory Viruses Branch, Division of Viral Diseases, Centers for Disease Control and Prevention	Krista Queen, Yan Li, Ying Tao, Jing Zhang, Anna Uehara, Anna Montmayeur, Clinton R. Paden, Peter W. Cook,Rachel Marine, Mili Sheth, Haibin Wang, Justin Lee, Suxiang Tong
EPI_ISL_751764, EPI_ISL_751765	AR Dept. of Health-Public Health Lab	Genomics and Discovery, Respiratory Viruses Branch, Division of Viral Diseases, Centers for Disease Control and Prevention	Krista Queen, Yan Li, Ying Tao, Jing Zhang, Anna Uehara, Anna Montmayeur, Clinton R. Paden, Peter W. Cook,Rachel Marine, Mili Sheth, Haibin Wang, Justin Lee, Suxiang Tong
EPI_ISL_751766	CO Dept. of Public Health and Environment, Lab Services Division	Genomics and Discovery, Respiratory Viruses Branch, Division of Viral Diseases, Centers for Disease Control and Prevention	Krista Queen, Yan Li, Ying Tao, Jing Zhang, Anna Uehara, Anna Montmayeur, Clinton R. Paden, Peter W. Cook,Rachel Marine, Mili Sheth, Haibin Wang, Justin Lee, Suxiang Tong
EPI_ISL_751767	HI Dept. of Health, State Laboratories Division	Genomics and Discovery, Respiratory Viruses Branch, Division of Viral Diseases, Centers for Disease Control and Prevention	Krista Queen, Yan Li, Ying Tao, Jing Zhang, Anna Uehara, Anna Montmayeur, Clinton R. Paden, Peter W. Cook,Rachel Marine, Mili Sheth, Haibin Wang, Justin Lee, Suxiang Tong
EPI_ISL_751768	IN State Department of Health Laboratory Services	Genomics and Discovery, Respiratory Viruses Branch, Division of Viral Diseases, Centers for Disease Control and Prevention	Krista Queen, Yan Li, Ying Tao, Jing Zhang, Anna Uehara, Anna Montmayeur, Clinton R. Paden, Peter W. Cook,Rachel Marine, Mili Sheth, Haibin Wang, Justin Lee, Suxiang Tong
EPI_ISL_751769	MI - Michigan Department of Health and Human Services - Bureau of Laboratories	Genomics and Discovery, Respiratory Viruses Branch, Division of Viral Diseases, Centers for Disease Control and Prevention	Krista Queen, Yan Li, Ying Tao, Jing Zhang, Anna Uehara, Anna Montmayeur, Clinton R. Paden, Peter W. Cook,Rachel Marine, Mili Sheth, Haibin Wang, Justin Lee, Suxiang Tong
EPI_ISL_751770	IN State Department of Health Laboratory Services	Genomics and Discovery, Respiratory Viruses Branch, Division of Viral Diseases, Centers for Disease Control and Prevention	Krista Queen, Yan Li, Ying Tao, Jing Zhang, Anna Uehara, Anna Montmayeur, Clinton R. Paden, Peter W. Cook,Rachel Marine, Mili Sheth, Haibin Wang, Justin Lee, Suxiang Tong
EPI_ISL_751771	MD DOH Laboratories Administration	Genomics and Discovery, Respiratory Viruses Branch, Division of Viral Diseases, Centers for Disease Control and Prevention	Krista Queen, Yan Li, Ying Tao, Jing Zhang, Anna Uehara, Anna Montmayeur, Clinton R. Paden, Peter W. Cook,Rachel Marine, Mili Sheth, Haibin Wang, Justin Lee, Suxiang Tong

EPI_ISL_751772	HI Dept. of Health, State Laboratories Division	Genomics and Discovery, Respiratory Viruses Branch, Division of Viral Diseases, Centers for Disease Control and Prevention	Krista Queen, Yan Li, Ying Tao, Jing Zhang, Anna Uehara, Anna Montmayeur, Clinton R. Paden, Peter W. Cook,Rachel Marine, Mili Sheth, Haibin Wang, Justin Lee, Suxiang Tong
EPI_ISL_751773	NJ Public Health and Environmental Laboratories	Genomics and Discovery, Respiratory Viruses Branch, Division of Viral Diseases, Centers for Disease Control and Prevention	Krista Queen, Yan Li, Ying Tao, Jing Zhang, Anna Uehara, Anna Montmayeur, Clinton R. Paden, Peter W. Cook,Rachel Marine, Mili Sheth, Haibin Wang, Justin Lee, Suxiang Tong
EPI_ISL_751774	MO State Public Health Laboratory	Genomics and Discovery, Respiratory Viruses Branch, Division of Viral Diseases, Centers for Disease Control and Prevention	Krista Queen, Yan Li, Ying Tao, Jing Zhang, Anna Uehara, Anna Montmayeur, Clinton R. Paden, Peter W. Cook,Rachel Marine, Mili Sheth, Haibin Wang, Justin Lee, Suxiang Tong
EPI_ISL_751775	MT Public Health Laboratory	Genomics and Discovery, Respiratory Viruses Branch, Division of Viral Diseases, Centers for Disease Control and Prevention	Krista Queen, Yan Li, Ying Tao, Jing Zhang, Anna Uehara, Anna Montmayeur, Clinton R. Paden, Peter W. Cook,Rachel Marine, Mili Sheth, Haibin Wang, Justin Lee, Suxiang Tong
EPI_ISL_751776	MS Public Health Laboratory	Genomics and Discovery, Respiratory Viruses Branch, Division of Viral Diseases, Centers for Disease Control and Prevention	Krista Queen, Yan Li, Ying Tao, Jing Zhang, Anna Uehara, Anna Montmayeur, Clinton R. Paden, Peter W. Cook,Rachel Marine, Mili Sheth, Haibin Wang, Justin Lee, Suxiang Tong
EPI_ISL_751777	OH Department of Health Laboratory	Genomics and Discovery, Respiratory Viruses Branch, Division of Viral Diseases, Centers for Disease Control and Prevention	Krista Queen, Yan Li, Ying Tao, Jing Zhang, Anna Uehara, Anna Montmayeur, Clinton R. Paden, Peter W. Cook,Rachel Marine, Mili Sheth, Haibin Wang, Justin Lee, Suxiang Tong
EPI_ISL_751778	NV State Public Health Laboratory	Genomics and Discovery, Respiratory Viruses Branch, Division of Viral Diseases, Centers for Disease Control and Prevention	Krista Queen, Yan Li, Ying Tao, Jing Zhang, Anna Uehara, Anna Montmayeur, Clinton R. Paden, Peter W. Cook,Rachel Marine, Mili Sheth, Haibin Wang, Justin Lee, Suxiang Tong
EPI_ISL_751779	IA State Hygienic Laboratory	Genomics and Discovery, Respiratory Viruses Branch, Division of Viral Diseases, Centers for Disease Control and Prevention	Krista Queen, Yan Li, Ying Tao, Jing Zhang, Anna Uehara, Anna Montmayeur, Clinton R. Paden, Peter W. Cook,Rachel Marine, Mili Sheth, Haibin Wang, Justin Lee, Suxiang Tong
EPI_ISL_751780	ID Bureau of Laboratories	Genomics and Discovery, Respiratory Viruses Branch, Division of Viral Diseases, Centers for Disease Control and Prevention	Krista Queen, Yan Li, Ying Tao, Jing Zhang, Anna Uehara, Anna Montmayeur, Clinton R. Paden, Peter W. Cook,Rachel Marine, Mili Sheth, Haibin Wang, Justin Lee, Suxiang Tong
EPI_ISL_751781	WY Public Health Laboratory	Genomics and Discovery, Respiratory Viruses Branch, Division of Viral Diseases, Centers for Disease Control and Prevention	Krista Queen, Yan Li, Ying Tao, Jing Zhang, Anna Uehara, Anna Montmayeur, Clinton R. Paden, Peter W. Cook,Rachel Marine, Mili Sheth, Haibin Wang, Justin Lee, Suxiang Tong
EPI_ISL_751782	ID Bureau of Laboratories	Genomics and Discovery, Respiratory Viruses Branch, Division of Viral Diseases, Centers for Disease Control and Prevention	Krista Queen, Yan Li, Ying Tao, Jing Zhang, Anna Uehara, Anna Montmayeur, Clinton R. Paden, Peter W. Cook,Rachel Marine, Mili Sheth, Haibin Wang, Justin Lee, Suxiang Tong
EPI_ISL_751783	IA State Hygienic Laboratory	Genomics and Discovery, Respiratory Viruses Branch, Division of Viral Diseases, Centers for Disease Control and Prevention	Krista Queen, Yan Li, Ying Tao, Jing Zhang, Anna Uehara, Anna Montmayeur, Clinton R. Paden, Peter W. Cook,Rachel Marine, Mili Sheth, Haibin Wang, Justin Lee, Suxiang Tong
EPI_ISL_751784	VA-Division of Consolidated Laboratory Services	Genomics and Discovery, Respiratory Viruses Branch, Division of Viral Diseases, Centers for Disease Control and Prevention	Krista Queen, Yan Li, Ying Tao, Jing Zhang, Anna Uehara, Anna Montmayeur, Clinton R. Paden, Peter W. Cook,Rachel Marine, Mili Sheth, Haibin Wang, Justin Lee, Suxiang Tong
EPI_ISL_751785, EPI_ISL_751786	WVDHHR - Office of Laboratory Services	Genomics and Discovery, Respiratory Viruses Branch, Division of Viral	Krista Queen, Yan Li, Ying Tao, Jing Zhang, Anna Uehara, Anna Montmayeur, Clinton R. Paden, Peter W. Cook,Rachel Marine, Mili Sheth, Haibin Wang, Justin Lee, Suxiang Tong

		Diseases, Centers for Disease Control and Prevention	
EPI_ISL_751787	TX DSHS, Lab Services Section MC 1947	Genomics and Discovery, Respiratory Viruses Branch, Division of Viral Diseases, Centers for Disease Control and Prevention	Krista Queen, Yan Li, Ying Tao, Jing Zhang, Anna Uehara, Anna Montmayeur, Clinton R. Paden, Peter W. Cook,Rachel Marine, Mili Sheth, Haibin Wang, Justin Lee, Suxiang Tong
EPI_ISL_751788	OH Department of Health Laboratory	Genomics and Discovery, Respiratory Viruses Branch, Division of Viral Diseases, Centers for Disease Control and Prevention	Krista Queen, Yan Li, Ying Tao, Jing Zhang, Anna Uehara, Anna Montmayeur, Clinton R. Paden, Peter W. Cook,Rachel Marine, Mili Sheth, Haibin Wang, Justin Lee, Suxiang Tong
EPI_ISL_751789	ID Bureau of Laboratories	Genomics and Discovery, Respiratory Viruses Branch, Division of Viral Diseases, Centers for Disease Control and Prevention	Krista Queen, Yan Li, Ying Tao, Jing Zhang, Anna Uehara, Anna Montmayeur, Clinton R. Paden, Peter W. Cook,Rachel Marine, Mili Sheth, Haibin Wang, Justin Lee, Suxiang Tong
EPI_ISL_751790	RI State Health Laboratories	Genomics and Discovery, Respiratory Viruses Branch, Division of Viral Diseases, Centers for Disease Control and Prevention	Krista Queen, Yan Li, Ying Tao, Jing Zhang, Anna Uehara, Anna Montmayeur, Clinton R. Paden, Peter W. Cook,Rachel Marine, Mili Sheth, Haibin Wang, Justin Lee, Suxiang Tong
EPI_ISL_751791, EPI_ISL_751792	AL Dept. of Public Health Bureau of Clinical Laboratories	Genomics and Discovery, Respiratory Viruses Branch, Division of Viral Diseases, Centers for Disease Control and Prevention	Krista Queen, Yan Li, Ying Tao, Jing Zhang, Anna Uehara, Anna Montmayeur, Clinton R. Paden, Peter W. Cook,Rachel Marine, Mili Sheth, Haibin Wang, Justin Lee, Suxiang Tong
EPI_ISL_751793	AK State Public Health Lab, State Health Department	Genomics and Discovery, Respiratory Viruses Branch, Division of Viral Diseases, Centers for Disease Control and Prevention	Krista Queen, Yan Li, Ying Tao, Jing Zhang, Anna Uehara, Anna Montmayeur, Clinton R. Paden, Peter W. Cook,Rachel Marine, Mili Sheth, Haibin Wang, Justin Lee, Suxiang Tong
EPI_ISL_751794	KY State Public Health Lab	Genomics and Discovery, Respiratory Viruses Branch, Division of Viral Diseases, Centers for Disease Control and Prevention	Krista Queen, Yan Li, Ying Tao, Jing Zhang, Anna Uehara, Anna Montmayeur, Clinton R. Paden, Peter W. Cook,Rachel Marine, Mili Sheth, Haibin Wang, Justin Lee, Suxiang Tong
EPI_ISL_751795	RI State Health Laboratories	Genomics and Discovery, Respiratory Viruses Branch, Division of Viral Diseases, Centers for Disease Control and Prevention	Krista Queen, Yan Li, Ying Tao, Jing Zhang, Anna Uehara, Anna Montmayeur, Clinton R. Paden, Peter W. Cook,Rachel Marine, Mili Sheth, Haibin Wang, Justin Lee, Suxiang Tong
EPI_ISL_751799	Labor Dr. Wisplinghoff - Köln	Robert Koch Institute, Bioinformatics MF1, Berlin, Germany	Dr. R. Grosser, Stephan Fuchs, Stefan Kroeger, Marianne Wedde, Oliver Drechsel, Aleksandar Radonic, Rene Kmiecinski, Ralf Duerrwald, Thorsten Wolff
EPI_ISL_751800	Colorado Department of Public Health and Environment	Colorado Department of Public Health & Environment	Laura Bankers, Molly C. Hetherington-Rauth, Diana Ir, Shannon Ely, Shannon R. Matzinger, Sarah Elizabeth Totten, Emily A. Travanty
EPI_ISL_751801	UCSD EXCITE	Andersen lab at Scripps Research	SEARCH Alliance San Diego
EPI_ISL_751802, EPI_ISL_751803, EPI_ISL_751804, EPI_ISL_751805, EPI_ISL_751806, EPI_ISL_751807, EPI_ISL_751808, EPI_ISL_751809, EPI_ISL_751810, EPI_ISL_751811, EPI_ISL_751812, EPI_ISL_751813, EPI_ISL_751814, EPI_ISL_751815, EPI_ISL_751816, EPI_ISL_751817, EPI_ISL_751818, EPI_ISL_751819, EPI_ISL_751820, EPI_ISL_751821, EPI_ISL_751822, EPI_ISL_751823, EPI_ISL_751824, EPI_ISL_751825, EPI_ISL_751826, EPI_ISL_751827, EPI_ISL_751828, EPI_ISL_751829, EPI_ISL_751830, EPI_ISL_751831, EPI_ISL_751832, EPI_ISL_751833, EPI_ISL_751834, EPI_ISL_751835, EPI_ISL_751836, EPI_ISL_751837, EPI_ISL_751838, EPI_ISL_751839, EPI_ISL_751840, EPI_ISL_751841, EPI_ISL_751842, EPI_ISL_751843, EPI_ISL_751844, EPI_ISL_751845, EPI_ISL_751846			
see above	New Mexico Department of Health Scientific Laboratory	New Mexico Department of Health Scientific Laboratory	D'eldra Malone, Ellie Johnson, Anastacia Griego-Fisher
EPI_ISL_752511, EPI_ISL_752512, EPI_ISL_752513, EPI_ISL_752514, EPI_ISL_752515, EPI_ISL_752516, EPI_ISL_752517, EPI_ISL_752518, EPI_ISL_752519, EPI_ISL_752520, EPI_ISL_752521, EPI_ISL_752522, EPI_ISL_752523, EPI_ISL_752524, EPI_ISL_752525, EPI_ISL_752526, EPI_ISL_752527, EPI_ISL_752528, EPI_ISL_752529, EPI_ISL_752530, EPI_ISL_752531, EPI_ISL_752532, EPI_ISL_752533, EPI_ISL_752534, EPI_ISL_752535, EPI_ISL_752536, EPI_ISL_752537, EPI_ISL_752538, EPI_ISL_752539, EPI_ISL_752540, EPI_ISL_752541, EPI_ISL_752542, EPI_ISL_752543, EPI_ISL_752544, EPI_ISL_752545, EPI_ISL_752546, EPI_ISL_752547, EPI_ISL_752548, EPI_ISL_752549, EPI_ISL_752550, EPI_ISL_752551, EPI_ISL_752552, EPI_ISL_752553, EPI_ISL_752554, EPI_ISL_752555, EPI_ISL_752556, EPI_ISL_752557, EPI_ISL_752558, EPI_ISL_752559, EPI_ISL_752560, EPI_ISL_752561, EPI_ISL_752562, EPI_ISL_752563, EPI_ISL_752564, EPI_ISL_752565, EPI_ISL_752566, EPI_ISL_752568, EPI_ISL_752572, EPI_ISL_752573, EPI_ISL_752574, EPI_ISL_752575, EPI_ISL_752576, EPI_ISL_752577, EPI_ISL_752578, EPI_ISL_752579, EPI_ISL_752580, EPI_ISL_752582, EPI_ISL_752583, EPI_ISL_752584, EPI_ISL_752586, EPI_ISL_752587, EPI_ISL_752588, EPI_ISL_752589, EPI_ISL_752590, EPI_ISL_752591, EPI_ISL_752593, EPI_ISL_752595, EPI_ISL_752596, EPI_ISL_752597			
see above	National Virus Reference Laboratory	National Virus Reference Laboratory	Michael Carr, Gabriel Gonzalez, Jonathan Dean, Daniel Hare, Cillian F De Gascun
EPI_ISL_752598, EPI_ISL_752599	SA Pathology	SA Pathology	Lex Leong, Julien Soubrier, Chuan Kok Lim, Song Gao, Mark Turra, Karin Kassahn, Ivan Bastian, Geoff Higgins
EPI_ISL_752605	Faculty of Medicine, Al-Quds University	Faculty of Medicine, Al-Quds University	Ereqat,S., Nasereddin,A. and Al-Jawabreh,A.
EPI_ISL_752606	Yale Pathology Lab	Grubaugh Lab - Yale School of Public Health	Tara Alpert, Joseph Fauver, Anderson Brito, Chen Liu, Pei Hui, Jianhui Wang, Kien Pham, Nathan Grubaugh

EPI_ISL_752644, EPI_ISL_752646, EPI_ISL_752647, EPI_ISL_752648, EPI_ISL_752649, EPI_ISL_752650, EPI_ISL_752651, EPI_ISL_752652, EPI_ISL_752653, EPI_ISL_752654, EPI_ISL_752655, EPI_ISL_752656, EPI_ISL_752657, EPI_ISL_752658, EPI_ISL_752659, EPI_ISL_752664, EPI_ISL_752665, EPI_ISL_752666, EPI_ISL_752667, EPI_ISL_752673, EPI_ISL_752674, EPI_ISL_752675, EPI_ISL_752676, EPI_ISL_752677, EPI_ISL_752678, EPI_ISL_752679, EPI_ISL_752680, EPI_ISL_752681, EPI_ISL_752682, EPI_ISL_752683, EPI_ISL_752684, EPI_ISL_752685, EPI_ISL_752686, EPI_ISL_752687, EPI_ISL_752688, EPI_ISL_752689, EPI_ISL_752690, EPI_ISL_752691, EPI_ISL_752692, EPI_ISL_752693, EPI_ISL_752694, EPI_ISL_752695, EPI_ISL_752696, EPI_ISL_752697, EPI_ISL_752698, EPI_ISL_752699, EPI_ISL_752701, EPI_ISL_752702, EPI_ISL_752703, EPI_ISL_752704, EPI_ISL_752705, EPI_ISL_752706, EPI_ISL_752707, EPI_ISL_752708, EPI_ISL_752709, EPI_ISL_752712, EPI_ISL_752713, EPI_ISL_752714, EPI_ISL_752715, EPI_ISL_752716, EPI_ISL_752717, EPI_ISL_752718, EPI_ISL_752719, EPI_ISL_752720, EPI_ISL_752721, EPI_ISL_752722, EPI_ISL_752723, EPI_ISL_752724, EPI_ISL_752725, EPI_ISL_752726, EPI_ISL_752727, EPI_ISL_752728, EPI_ISL_752729, EPI_ISL_752730, EPI_ISL_752731, EPI_ISL_752732, EPI_ISL_752733, EPI_ISL_752734, EPI_ISL_752735, EPI_ISL_752736, EPI_ISL_752737, EPI_ISL_752738, EPI_ISL_752739, EPI_ISL_752740, EPI_ISL_752741, EPI_ISL_752742, EPI_ISL_752743, EPI_ISL_752744, EPI_ISL_752745, EPI_ISL_752746, EPI_ISL_752747, EPI_ISL_752748, EPI_ISL_752749, EPI_ISL_752750, EPI_ISL_752751, EPI_ISL_752752, EPI_ISL_752753, EPI_ISL_752754, EPI_ISL_752755, EPI_ISL_752756, EPI_ISL_752757, EPI_ISL_752758, EPI_ISL_752759, EPI_ISL_752760, EPI_ISL_752761, EPI_ISL_752762, EPI_ISL_752763, EPI_ISL_752764, EPI_ISL_752765, EPI_ISL_752766, EPI_ISL_752767, EPI_ISL_752768, EPI_ISL_752769, EPI_ISL_752770, EPI_ISL_752771, EPI_ISL_752772, EPI_ISL_752773, EPI_ISL_752774, EPI_ISL_752775, EPI_ISL_752776, EPI_ISL_752777, EPI_ISL_752778, EPI_ISL_752779, EPI_ISL_752780, EPI_ISL_752781, EPI_ISL_752782, EPI_ISL_752783, EPI_ISL_752784, EPI_ISL_752785, EPI_ISL_752786, EPI_ISL_752787, EPI_ISL_752788, EPI_ISL_752789, EPI_ISL_752790, EPI_ISL_752791, EPI_ISL_752792, EPI_ISL_752793, EPI_ISL_752794, EPI_ISL_752795, EPI_ISL_752796, EPI_ISL_752797, EPI_ISL_752798, EPI_ISL_752799, EPI_ISL_752800, EPI_ISL_752801, EPI_ISL_752802, EPI_ISL_752803, EPI_ISL_752804, EPI_ISL_752805, EPI_ISL_752806, EPI_ISL_752807, EPI_ISL_752808, EPI_ISL_752809, EPI_ISL_752810, EPI_ISL_752811, EPI_ISL_752812, EPI_ISL_752813, EPI_ISL_752814, EPI_ISL_752815, EPI_ISL_752816, EPI_ISL_752817, EPI_ISL_752818, EPI_ISL_752819, EPI_ISL_752820, EPI_ISL_752821, EPI_ISL_752822, EPI_ISL_752823, EPI_ISL_752824, EPI_ISL_752825, EPI_ISL_752826, EPI_ISL_752827, EPI_ISL_752828, EPI_ISL_752829, EPI_ISL_752830, EPI_ISL_752831, EPI_ISL_752832, EPI_ISL_752833, EPI_ISL_752834, EPI_ISL_752835, EPI_ISL_752836, EPI_ISL_752837, EPI_ISL_752838, EPI_ISL_752839, EPI_ISL_752840, EPI_ISL_752841, EPI_ISL_752842, EPI_ISL_752843, EPI_ISL_752844, EPI_ISL_752846, EPI_ISL_752847, EPI_ISL_752848, EPI_ISL_752849, EPI_ISL_752850, EPI_ISL_752851, EPI_ISL_752852, EPI_ISL_752853, EPI_ISL_752854, EPI_ISL_752855, EPI_ISL_752856, EPI_ISL_752857, EPI_ISL_752858, EPI_ISL_752859, EPI_ISL_752860, EPI_ISL_752861, EPI_ISL_752862, EPI_ISL_752863, EPI_ISL_752864, EPI_ISL_752865, EPI_ISL_752866, EPI_ISL_752867, EPI_ISL_752868, EPI_ISL_752869, EPI_ISL_752870, EPI_ISL_752871, EPI_ISL_752872, EPI_ISL_752873, EPI_ISL_752874, EPI_ISL_752875, EPI_ISL_752876, EPI_ISL_752877, EPI_ISL_752878, EPI_ISL_752879, EPI_ISL_752880, EPI_ISL_752881, EPI_ISL_752882, EPI_ISL_752883, EPI_ISL_752884, EPI_ISL_752885, EPI_ISL_752886, EPI_ISL_752887, EPI_ISL_752888, EPI_ISL_752889, EPI_ISL_752890, EPI_ISL_752891, EPI_ISL_752892, EPI_ISL_752893, EPI_ISL_752894, EPI_ISL_752895, EPI_ISL_752896, EPI_ISL_752897, EPI_ISL_752898, EPI_ISL_752899, EPI_ISL_752900, EPI_ISL_752901, EPI_ISL_752902, EPI_ISL_752903, EPI_ISL_752904, EPI_ISL_752905, EPI_ISL_752906, EPI_ISL_752907, EPI_ISL_752908, EPI_ISL_752909, EPI_ISL_752910, EPI_ISL_752911, EPI_ISL_752912, EPI_ISL_752913, EPI_ISL_752914, EPI_ISL_752915, EPI_ISL_752916, EPI_ISL_752917, EPI_ISL_752918, EPI_ISL_752919, EPI_ISL_752920, EPI_ISL_752921, EPI_ISL_752922, EPI_ISL_752923, EPI_ISL_752924, EPI_ISL_752925, EPI_ISL_752926, EPI_ISL_752927, EPI_ISL_752928, EPI_ISL_752929, EPI_ISL_752930, EPI_ISL_752931, EPI_ISL_752932, EPI_ISL_752933, EPI_ISL_752934, EPI_ISL_752935, EPI_ISL_752936, EPI_ISL_752937, EPI_ISL_752938, EPI_ISL_752939, EPI_ISL_752940, EPI_ISL_752941, EPI_ISL_752942, EPI_ISL_752943, EPI_ISL_752944, EPI_ISL_752945, EPI_ISL_752946, EPI_ISL_752947, EPI_ISL_752948, EPI_ISL_752949, EPI_ISL_752950, EPI_ISL_752951, EPI_ISL_752952, EPI_ISL_752953, EPI_ISL_752954, EPI_ISL_752955, EPI_ISL_752956, EPI_ISL_752957, EPI_ISL_752958, EPI_ISL_752959, EPI_ISL_752960, EPI_ISL_752961, EPI_ISL_752962, EPI_ISL_752963, EPI_ISL_752964, EPI_ISL_752965, EPI_ISL_752966, EPI_ISL_752967, EPI_ISL_752968, EPI_ISL_752969, EPI_ISL_752970, EPI_ISL_752971, EPI_ISL_752972, EPI_ISL_752973, EPI_ISL_752974, EPI_ISL_752975, EPI_ISL_752976, EPI_ISL_752977, EPI_ISL_752978, EPI_ISL_752979, EPI_ISL_752980, EPI_ISL_752981, EPI_ISL_752982, EPI_ISL_752983, EPI_ISL_752984, EPI_ISL_752985, EPI_ISL_752986, EPI_ISL_752987, EPI_ISL_752988, EPI_ISL_752989, EPI_ISL_752990, EPI_ISL_752991, EPI_ISL_752992, EPI_ISL_752993, EPI_ISL_752994, EPI_ISL_752995, EPI_ISL_752996, EPI_ISL_752997, EPI_ISL_752998, EPI_ISL_752999, EPI_ISL_753000, EPI_ISL_753001, EPI_ISL_753002, EPI_ISL_753003, EPI_ISL_753004, EPI_ISL_753005, EPI_ISL_753006, EPI_ISL_753007, EPI_ISL_753008, EPI_ISL_753009, EPI_ISL_753010, EPI_ISL_753011, EPI_ISL_753012, EPI_ISL_753013, EPI_ISL_753014, EPI_ISL_753015, EPI_ISL_753016, EPI_ISL_753017, EPI_ISL_753018, EPI_ISL_753019, EPI_ISL_753020, EPI_ISL_753021, EPI_ISL_753022, EPI_ISL_753023, EPI_ISL_753024, EPI_ISL_753025, EPI_ISL_753026, EPI_ISL_753027, EPI_ISL_753028, EPI_ISL_753029, EPI_ISL_753030, EPI_ISL_753031, EPI_ISL_753032, EPI_ISL_753033, EPI_ISL_753034, EPI_ISL_753035, EPI_ISL_753036, EPI_ISL_753037, EPI_ISL_753038, EPI_ISL_753039, EPI_ISL_753040, EPI_ISL_753041, EPI_ISL_753042, EPI_ISL_753043, EPI_ISL_753044, EPI_ISL_753045, EPI_ISL_753046, EPI_ISL_753047, EPI_ISL_753048, EPI_ISL_753049, EPI_ISL_753050, EPI_ISL_753051, EPI_ISL_753052, EPI_ISL_753053, EPI_ISL_753054, EPI_ISL_753055, EPI_ISL_753056, EPI_ISL_753057, EPI_ISL_753058, EPI_ISL_753059, EPI_ISL_753060, EPI_ISL_753061, EPI_ISL_753062, EPI_ISL_753063, EPI_ISL_753064, EPI_ISL_753065, EPI_ISL_753066, EPI_ISL_753067, EPI_ISL_753068, EPI_ISL_753069, EPI_ISL_753070, EPI_ISL_753071, EPI_ISL_753072, EPI_ISL_753073, EPI_ISL_753074, EPI_ISL_753075, EPI_ISL_753076, EPI_ISL_753077, EPI_ISL_753078, EPI_ISL_753079, EPI_ISL_753080, EPI_ISL_753081, EPI_ISL_753082, EPI_ISL_753083, EPI_ISL_753084, EPI_ISL_753085, EPI_ISL_753086, EPI_ISL_753087, EPI_ISL_753088, EPI_ISL_753089, EPI_ISL_753090, EPI_ISL_753091, EPI_ISL_753092, EPI_ISL_753093, EPI_ISL_753094, EPI_ISL_753095, EPI_ISL_753096, EPI_ISL_753097, EPI_ISL_753098, EPI_ISL_753099, EPI_ISL_753100, EPI_ISL_753101, EPI_ISL_753102, EPI_ISL_753103, EPI_ISL_753104, EPI_ISL_753105, EPI_ISL_753106, EPI_ISL_753107, EPI_ISL_753108, EPI_ISL_753109, EPI_ISL_753110, EPI_ISL_753111, EPI_ISL_753112, EPI_ISL_753113, EPI_ISL_753114, EPI_ISL_753115, EPI_ISL_753116, EPI_ISL_753117, EPI_ISL_753118, EPI_ISL_753119, EPI_ISL_753120, EPI_ISL_753121, EPI_ISL_753122, EPI_ISL_753123, EPI_ISL_753124, EPI_ISL_753125, EPI_ISL_753126, EPI_ISL_753127, EPI_ISL_753128, EPI_ISL_753129, EPI_ISL_753130, EPI_ISL_753131, EPI_ISL_753132, EPI_ISL_753133, EPI_ISL_753134, EPI_ISL_753135, EPI_ISL_753136, EPI_ISL_753137, EPI_ISL_753138, EPI_ISL_753139, EPI_ISL_753140, EPI_ISL_753141, EPI_ISL_753142, EPI_ISL_753143, EPI_ISL_753144, EPI_ISL_753145, EPI_ISL_753146, EPI_ISL_753147, EPI_ISL_753148, EPI_ISL_753149, EPI_ISL_753150, EPI_ISL_753151, EPI_ISL_753152, EPI_ISL_753153, EPI_ISL_753154, EPI_ISL_753155, EPI_ISL_753156, EPI_ISL_753157, EPI_ISL_753158, EPI_ISL_753159, EPI_ISL_753160, EPI_ISL_753161, EPI_ISL_753162, EPI_ISL_753163, EPI_ISL_753164, EPI_ISL_753165, EPI_ISL_753166, EPI_ISL_753167, EPI_ISL_753168, EPI_ISL_753169, EPI_ISL_753170, EPI_ISL_753171, EPI_ISL_753172, EPI_ISL_753173, EPI_ISL_753174, EPI_ISL_753175, EPI_ISL_753176, EPI_ISL_753177, EPI_ISL_753178, EPI_ISL_753179, EPI_ISL_753180, EPI_ISL_753181, EPI_ISL_753182, EPI_ISL_753183, EPI_ISL_753184, EPI_ISL_753185, EPI_ISL_753186, EPI_ISL_753187, EPI_ISL_753188, EPI_ISL_753189, EPI_ISL_753190, EPI_ISL_753191, EPI_ISL_753192, EPI_ISL_753193, EPI_ISL_753194, EPI_ISL_753195, EPI_ISL_753196, EPI_ISL_753197, EPI_ISL_753198, EPI_ISL_753199, EPI_ISL_753200, EPI_ISL_753201, EPI_ISL_753202, EPI_ISL_753203, EPI_ISL_753204, EPI_ISL_753205, EPI_ISL_753206, EPI_ISL_753207, EPI_ISL_753208, EPI_ISL_753209, EPI_ISL_753210, EPI_ISL_753211, EPI_ISL_753212, EPI_ISL_753213, EPI_ISL_753214, EPI_ISL_753215, EPI_ISL_753216, EPI_ISL_753217, EPI_ISL_753218, EPI_ISL_753219, EPI_ISL_753220, EPI_ISL_753221, EPI_ISL_753222, EPI_ISL_753223, EPI_ISL_753224, EPI_ISL_753225, EPI_ISL_753226, EPI_ISL_753227, EPI_ISL_753228, EPI_ISL_753229, EPI_ISL_753230, EPI_ISL_753231, EPI_ISL_753232, EPI_ISL_753233, EPI_ISL_753234, EPI_ISL_753235, EPI_ISL_753236, EPI_ISL_753237, EPI_ISL_753238, EPI_ISL_753239, EPI_ISL_753240, EPI_ISL_753241, EPI_ISL_753242, EPI_ISL_753243, EPI_ISL_753244, EPI_ISL_753245, EPI_ISL_753246, EPI_ISL_753247, EPI_ISL_753248, EPI_ISL_753249, EPI_ISL_753250, EPI_ISL_753251, EPI_ISL_753252, EPI_ISL_753253, EPI_ISL_753254, EPI_ISL_753255, EPI_ISL_753256, EPI_ISL_753257, EPI_ISL_753258, EPI_ISL_753259, EPI_ISL_753260, EPI_ISL_753261			
see above	State Laboratories Division, Hawaii State Department of Health	State Laboratories Division, Hawaii State Department of Health	Pamela O'Brien, Sabrina Diemert, Drew Kuwazaki, Razvan Sultana, Edward Desmond
EPI_ISL_753262, EPI_ISL_753263, EPI_ISL_753264, EPI_ISL_753265, EPI_ISL_753266, EPI_ISL_753267, EPI_ISL_753268, EPI_ISL_753269, EPI_ISL_753270, EPI_ISL_753271, EPI_ISL_753272, EPI_ISL_753273, EPI_ISL_753274, EPI_ISL_753275, EPI_ISL_753276, EPI_ISL_753277, EPI_ISL_753278, EPI_ISL_753279, EPI_ISL_753280, EPI_ISL_753281, EPI_ISL_753282, EPI_ISL_753283, EPI_ISL_753284, EPI_ISL_753285, EPI_ISL_753286, EPI_ISL_753287, EPI_ISL_753288, EPI_ISL_753289, EPI_ISL_753290, EPI_ISL_753291, EPI_ISL_753292, EPI_ISL_753293, EPI_ISL_753294, EPI_ISL_753295, EPI_ISL_753296, EPI_ISL_753297, EPI_ISL_753298, EPI_ISL_753299, EPI_ISL_753300, EPI_ISL_753301, EPI_ISL_753302, EPI_ISL_753303, EPI_ISL_753304, EPI_ISL_753305, EPI_ISL_753306, EPI_ISL_753307, EPI_ISL_753308, EPI_ISL_753309, EPI_ISL_753310, EPI_ISL_753311, EPI_ISL_753312, EPI_ISL_753313, EPI_ISL_753314, EPI_ISL_753315, EPI_ISL_753316, EPI_ISL_753317, EPI_ISL_753318, EPI_ISL_753319, EPI_ISL_753320, EPI_ISL_753321, EPI_ISL_753322, EPI_ISL_753323, EPI_ISL_753324, EPI_ISL_753325, EPI_ISL_753326, EPI_ISL_753327, EPI_ISL_753328, EPI_ISL_753329, EPI_ISL_753330, EPI_ISL_753331, EPI_ISL_753332, EPI_ISL_753333, EPI_ISL_753334, EPI_ISL_753335, EPI_ISL_753336, EPI_ISL_753337, EPI_ISL_753338, EPI_ISL_753339, EPI_ISL_753340, EPI_ISL_753341, EPI_ISL_753342, EPI_ISL_753343, EPI_ISL_753344, EPI_ISL_753345, EPI_ISL_753346, EPI_ISL_753347, EPI_ISL_753348, EPI_ISL_753349, EPI_ISL_753350, EPI_ISL_753351, EPI_ISL_753352, EPI_ISL_753353, EPI_ISL_753354, EPI_ISL_753355, EPI_ISL_753356, EPI_ISL_753357, EPI_ISL_753358, EPI_ISL_753359, EPI_ISL_753360, EPI_ISL_753361, EPI_ISL_753362, EPI_ISL_753363, EPI_ISL_753364, EPI_ISL_753365, EPI_ISL_753366, EPI_ISL_753367, EPI_ISL_753368, EPI_ISL_753369, EPI_ISL_753370, EPI_ISL_753371, EPI_ISL_753372, EPI_ISL_753373, EPI_ISL_753374, EPI_ISL_753375, EPI_ISL_753376, EPI_ISL_753377, EPI_ISL_753378, EPI_ISL_753379, EPI_ISL_753380, EPI_ISL_753381, EPI_ISL_753382, EPI_ISL_753383, EPI_ISL_753384, EPI_ISL_753385, EPI_ISL_753386, EPI_ISL_753387, EPI_ISL_753388, EPI_ISL_753389, EPI_ISL_753390, EPI_ISL_753391, EPI_ISL_753392, EPI_ISL_753393, EPI_ISL_753394, EPI_ISL_753395, EPI_ISL_753396, EPI_ISL_753397, EPI_ISL_753398, EPI_ISL_753399, EPI_ISL_753400, EPI_ISL_753401, EPI_ISL_753402, EPI_ISL_753403, EPI_ISL_753404, EPI_ISL_753405, EPI_ISL_753406, EPI_ISL_753407, EPI_ISL_753408, EPI_ISL_753409, EPI_ISL_753410, EPI_ISL_753411, EPI_ISL_753412, EPI_ISL_753413, EPI_ISL_753414, EPI_ISL_753415, EPI_ISL_753416, EPI_ISL_753417, EPI_ISL_753418, EPI_ISL_753419, EPI_ISL_753420, EPI_ISL_753421, EPI_ISL_753422, EPI_ISL_753423, EPI_ISL_753424, EPI_ISL_753425, EPI_ISL_753426, EPI_ISL_753427, EPI_ISL_753428, EPI_ISL_753429, EPI_ISL_753430, EPI_ISL_753431, EPI_ISL_753432, EPI_ISL_753433, EPI_ISL_753434, EPI_ISL_753435, EPI_ISL_753436, EPI_ISL_753437, EPI_ISL_753438, EPI_ISL_753439, EPI_ISL_753440, EPI_ISL_753441, EPI_ISL_753442, EPI_ISL_753443, EPI_ISL_753444, EPI_ISL_753445, EPI_ISL_753446, EPI_ISL_753447, EPI_ISL_753448, EPI_ISL_753449, EPI_ISL_753450, EPI_ISL_753451, EPI_ISL_753452, EPI_ISL_753453, EPI_ISL_753454, EPI_ISL_753455, EPI_ISL_753456, EPI_ISL_753457, EPI_ISL_753458, EPI_ISL_753459, EPI_ISL_753460, EPI_ISL_753461, EPI_ISL_753462, EPI_ISL_753463, EPI_ISL_753464, EPI_ISL_753465, EPI_ISL_753466, EPI_ISL_753467, EPI_ISL_753468, EPI_ISL_753469, EPI_ISL_753470, EPI_ISL_753471, EPI_ISL_753472, EPI_ISL_753473, EPI_ISL_753474, EPI_ISL_753475, EPI_ISL_753476, EPI_ISL_753477, EPI_ISL_753478, EPI_ISL_753479, EPI_ISL_753480, EPI_ISL_753481, EPI_ISL_753482, EPI_ISL_753483, EPI_ISL_753484, EPI_ISL_753485, EPI_ISL_753486, EPI_ISL_753487, EPI_ISL_753488, EPI_ISL_753489, EPI_ISL_753490, EPI_ISL_753491, EPI_ISL_753492, EPI_ISL_753493, EPI_ISL_753494, EPI_ISL_753495, EPI_ISL_753496, EPI_ISL_753497, EPI_ISL_753498, EPI_ISL_753499, EPI_ISL_753500, EPI_ISL_753501, EPI_ISL_753502, EPI_ISL_753503, EPI_ISL_753504, EPI_ISL_753505, EPI_ISL_753506, EPI_ISL_753507, EPI_ISL_753508, EPI_ISL_753509, EPI_ISL_753510, EPI_ISL_753511, EPI_ISL_753512, EPI_ISL_753513, EPI_ISL_753514, EPI_ISL_753515, EPI_ISL_753516, EPI_ISL_753517, EPI_ISL_753518, EPI_ISL_753519, EPI_ISL_753520, EPI_ISL_753521, EPI_ISL_753522, EPI_ISL_753523, EPI_ISL_753524, EPI_ISL_753525, EPI_ISL_753526, EPI_ISL_753527, EPI_ISL_753528, EPI_ISL_753529, EPI_ISL_753530, EPI_ISL_753531, EPI_ISL_753532, EPI_ISL_753533, EPI_ISL_753534, EPI_ISL_753535, EPI_ISL_753536, EPI_ISL_753537, EPI_ISL_753538, EPI_ISL_753539, EPI_ISL_753540, EPI_ISL_753541, EPI_ISL_753542, EPI_ISL_753543, EPI_ISL_753544, EPI_ISL_753545, EPI_ISL_753546, EPI_ISL_753547, EPI_ISL_753548, EPI_ISL_753549, EPI_ISL_753550, EPI_ISL_753551, EPI_ISL_753552, EPI_ISL_753553, EPI_ISL_753554, EPI_ISL_753555, EPI_ISL_753556, EPI_ISL_753557, EPI_ISL_753558, EPI_ISL_753559, EPI_ISL_753560, EPI_ISL_753561, EPI_ISL_753562, EPI_ISL_753563, EPI_ISL_753564, EPI_ISL_753565, EPI_ISL_753566, EPI_ISL_753567, EPI_ISL_753568, EPI_ISL_753569, EPI_ISL_753570, EPI_ISL_753571, EPI_ISL_753572, EPI_ISL_753573, EPI_ISL_753574, EPI_ISL_753575, EPI_ISL_753576, EPI_ISL_753577, EPI_ISL_753578, EPI_ISL_753579, EPI_ISL_753580, EPI_ISL_753581, EPI_ISL_753582, EPI_ISL_753583, EPI_ISL_753584, EPI_ISL_753585, EPI_ISL_753586, EPI_ISL_753587, EPI_ISL_753588, EPI_ISL_753589, EPI_ISL_753590, EPI_ISL_753591, EPI_ISL_753592, EPI_ISL_753593, EPI_ISL_753594, EPI_ISL_753595, EPI_ISL_753596, EPI_ISL_753597, EPI_ISL_753598, EPI_ISL_753599, EPI_ISL_753600, EPI_ISL_753601, EPI_ISL_753602, EPI_ISL_753603, EPI_ISL_753604, EPI_ISL_753605, EPI_ISL_753606, EPI_ISL_753607, EPI_ISL_753608, EPI_ISL_753609, EPI_ISL_753610, EPI_ISL_753611, EPI_ISL_753612, EPI_ISL_753613, EPI_ISL_753614, EPI_ISL_753615, EPI_ISL_753616, EPI_ISL_753617, EPI_ISL_753618, EPI_ISL_753619, EPI_ISL_753620, EPI_ISL_753621, EPI_ISL_753622, EPI_ISL_753623, EPI_ISL_753624, EPI_ISL_753625, EPI_ISL_753626, EPI_ISL_753627, EPI_ISL_753628, EPI_ISL_753629, EPI_ISL_753630, EPI_ISL_753631, EPI_ISL_753632, EPI_ISL_753633, EPI_ISL_753634, EPI_ISL_753635, EPI_ISL_753636, EPI_ISL_753637, EPI_ISL_753638, EPI_ISL_753639, EPI_ISL_753640, EPI_ISL_753641, EPI_ISL_753642, EPI_ISL_753643, EPI_ISL_753644, EPI_ISL_753645, EPI_ISL_753646, EPI_ISL_753647, EPI_ISL_753648, EPI_ISL_753649, EPI_ISL_753650, EPI_ISL_753651, EPI_ISL_753652, EPI_ISL_753653, EPI_ISL_753654, EPI_ISL_753655, EPI_ISL_753656, EPI_ISL_753657, EPI_ISL_753658, EPI_ISL_753659, EPI_ISL_753660, EPI_ISL_753661, EPI_ISL_753662, EPI_ISL_753663, EPI_ISL_753664, EPI_ISL_753665, EPI_ISL_753666, EPI_ISL_753667, EPI_ISL_753668, EPI_ISL_753669, EPI_ISL_753670, EPI_ISL_753671, EPI_ISL_753672, EPI_ISL_753673, EPI_ISL_753674, EPI_ISL_753675, EPI_ISL_753676, EPI_ISL_753677, EPI_ISL_753678, EPI_ISL_753679, EPI_ISL_753680, EPI_ISL_753681, EPI_ISL_753682, EPI_ISL_753683, EPI_ISL_753684, EPI_ISL_753685, EPI_ISL_753686, EPI_ISL_753687, EPI_ISL_753688, EPI_ISL_753689, EPI_ISL_753690, EPI_ISL_753691, EPI_ISL_753692			
see above	Clinical virology Laboratory, Children's Hospital Los Angeles	Center for Personalized Medicine, Children's Hospital Los Angeles	Gai et al
EPI_ISL_753697	Specialized Lab for COVID-19 Detection, Department of Genetic Engineering and Biotechnology	Specialized Lab for COVID-19 Detection, Department of Genetic Engineering and Biotechnology	Shamsul H. Prodhan, Md. Asraful Jahan, Hammadul Hoque, Nurnabi Azad Jewel, Md. Nazmul Hasan, Hafiz Al Ashad, Rahatul Islam, Salman Ahmed
EPI_ISL_753698	Balai Besar Veteriner Maros, Sulawesi Selatan	National Institute of Health Research and Development	Puspa,KD;Subangkit;Pawestri,HA;Nugraha,AA;Ikawati,HD;Pangesti,KNA;Soekarso,T;Puspandari,N;Muflihanah;Mangidi,R;Setiawaty,V
EPI_ISL_753699	RSUD Ulin Banjarmasin Kalimantan Selatan	National Institute of Health Research and Development	Subangkit;Pawestri,HA;Puspa,KD;Nugraha,AA;Ikawati,HD;Pangesti,KNA;Soekarso,T;Puspandari,N;Pasaribu,M;Setiawaty,V
EPI_ISL_753700	Specialized Lab for COVID-19 Detection, Department of Genetic Engineering and Biotechnology	Specialized Lab for COVID-19 Detection, Department of Genetic Engineering and Biotechnology	Hammadul Hoque, Ajit Ghosh, Nurnabi Azad Jewel, Md. Nazmul Hasan, Shamsul H. Prodhan, Amit Kumar Mondal, Fahmid H. Bhuiyan, Md. Rakib Wazed Nayon
EPI_ISL_753701	Specialized Lab for COVID-19 Detection, Department of Genetic Engineering and	Specialized Lab for COVID-19 Detection, Department of Genetic	Nurnabi Azad Jewel, Ziaul Faruque Joy , Md. Nazmul Hasan, Shamsul H. Prodhan, Hammadul Hoque , Md. Mobarok Karim, Shahrear Arefin, Md. Tahsin Khan

	Biotechnology	Engineering and Biotechnology	
EPI_ISL_753703, EPI_ISL_753705, EPI_ISL_753706, EPI_ISL_753707, EPI_ISL_753708, EPI_ISL_753710, EPI_ISL_753711, EPI_ISL_753713, EPI_ISL_753714, EPI_ISL_753715, EPI_ISL_753716, EPI_ISL_753717, EPI_ISL_753719, EPI_ISL_753720, EPI_ISL_753723, EPI_ISL_753724, EPI_ISL_753726, EPI_ISL_753728, EPI_ISL_753731, EPI_ISL_753733, EPI_ISL_753734, EPI_ISL_753735, EPI_ISL_753736, EPI_ISL_753737, EPI_ISL_753739, EPI_ISL_753740, EPI_ISL_753741, EPI_ISL_753742, EPI_ISL_753743, EPI_ISL_753744, EPI_ISL_753745, EPI_ISL_753747, EPI_ISL_753748, EPI_ISL_753749, EPI_ISL_753750, EPI_ISL_753751, EPI_ISL_753755, EPI_ISL_753756, EPI_ISL_753757, EPI_ISL_753758, EPI_ISL_753762, EPI_ISL_753765, EPI_ISL_753768, EPI_ISL_753769, EPI_ISL_753771, EPI_ISL_753773, EPI_ISL_753776, EPI_ISL_753777, EPI_ISL_753778, EPI_ISL_753779, EPI_ISL_753780, EPI_ISL_753781, EPI_ISL_753782, EPI_ISL_753783, EPI_ISL_753784, EPI_ISL_753785, EPI_ISL_753786, EPI_ISL_753787, EPI_ISL_753788, EPI_ISL_753789, EPI_ISL_753790, EPI_ISL_753791, EPI_ISL_753792, EPI_ISL_753793, EPI_ISL_753794, EPI_ISL_753795, EPI_ISL_753796, EPI_ISL_753801, EPI_ISL_753802, EPI_ISL_753803, EPI_ISL_753804, EPI_ISL_753807, EPI_ISL_753808, EPI_ISL_753809, EPI_ISL_753810, EPI_ISL_753815, EPI_ISL_753816, EPI_ISL_753817, EPI_ISL_753818, EPI_ISL_753819, EPI_ISL_753820, EPI_ISL_753821, EPI_ISL_753826, EPI_ISL_753827, EPI_ISL_753828, EPI_ISL_753829, EPI_ISL_753830, EPI_ISL_753831, EPI_ISL_753832, EPI_ISL_753833, EPI_ISL_753835, EPI_ISL_753837, EPI_ISL_753842, EPI_ISL_753853, EPI_ISL_753854, EPI_ISL_753855, EPI_ISL_753856, EPI_ISL_753857, EPI_ISL_753858, EPI_ISL_753859, EPI_ISL_753860, EPI_ISL_753861, EPI_ISL_753862, EPI_ISL_753863, EPI_ISL_753864, EPI_ISL_753865, EPI_ISL_753866, EPI_ISL_753867, EPI_ISL_753868, EPI_ISL_753869, EPI_ISL_753870, EPI_ISL_753871, EPI_ISL_753872, EPI_ISL_753873, EPI_ISL_753874, EPI_ISL_753875, EPI_ISL_753876, EPI_ISL_753877, EPI_ISL_753878, EPI_ISL_753879, EPI_ISL_753880, EPI_ISL_753881, EPI_ISL_753882, EPI_ISL_753883, EPI_ISL_753884, EPI_ISL_753885, EPI_ISL_753886, EPI_ISL_753887, EPI_ISL_753888, EPI_ISL_753889, EPI_ISL_753890, EPI_ISL_753891, EPI_ISL_753892, EPI_ISL_753893, EPI_ISL_753894, EPI_ISL_753895, EPI_ISL_753896, EPI_ISL_753897, EPI_ISL_753898, EPI_ISL_753899, EPI_ISL_753900, EPI_ISL_753901, EPI_ISL_753902, EPI_ISL_753903, EPI_ISL_753904, EPI_ISL_753905, EPI_ISL_753906, EPI_ISL_753907, EPI_ISL_753908, EPI_ISL_753909, EPI_ISL_753910, EPI_ISL_753911, EPI_ISL_753912, EPI_ISL_753913, EPI_ISL_753914, EPI_ISL_753915, EPI_ISL_753916, EPI_ISL_753956, EPI_ISL_753957, EPI_ISL_753958, EPI_ISL_753959, EPI_ISL_753960, EPI_ISL_753961, EPI_ISL_753962, EPI_ISL_753963, EPI_ISL_753964, EPI_ISL_753965, EPI_ISL_753966, EPI_ISL_753967, EPI_ISL_753968, EPI_ISL_753969, EPI_ISL_753970, EPI_ISL_753971, EPI_ISL_753972, EPI_ISL_753973, EPI_ISL_753974, EPI_ISL_753975, EPI_ISL_753976, EPI_ISL_753977, EPI_ISL_753978, EPI_ISL_753979, EPI_ISL_753980, EPI_ISL_753981, EPI_ISL_753982, EPI_ISL_753983, EPI_ISL_753984, EPI_ISL_753985, EPI_ISL_753986, EPI_ISL_753987, EPI_ISL_753988, EPI_ISL_753989, EPI_ISL_753990, EPI_ISL_753991, EPI_ISL_753992, EPI_ISL_753993, EPI_ISL_753994, EPI_ISL_753995, EPI_ISL_753996, EPI_ISL_753997, EPI_ISL_753998, EPI_ISL_753999, EPI_ISL_754000, EPI_ISL_754001, EPI_ISL_754002, EPI_ISL_754003, EPI_ISL_754004, EPI_ISL_754005, EPI_ISL_754006, EPI_ISL_754007, EPI_ISL_754008, EPI_ISL_754009, EPI_ISL_754010, EPI_ISL_754011, EPI_ISL_754012, EPI_ISL_754013, EPI_ISL_754014, EPI_ISL_754015, EPI_ISL_754016, EPI_ISL_754017, EPI_ISL_754018, EPI_ISL_754019, EPI_ISL_754020, EPI_ISL_754021, EPI_ISL_754022, EPI_ISL_754023, EPI_ISL_754039, EPI_ISL_754043, EPI_ISL_754044, EPI_ISL_754045, EPI_ISL_754054			
see above	Charité Universitätsmedizin Berlin, Institut für Virologie/Labor Berlin	Charité Universitätsmedizin Berlin, Institut für Virologie	Victor M Corman, Jörn Beheim-Schwarzbach, Barbara Mühlemann, Julia Schneider, Talitha Veith, Terry Jones, Christian Drosten
EPI_ISL_754059	Specialized Lab for COVID-19 Detection, Department of Genetic Engineering and Biotechnology	Specialized Lab for COVID-19 Detection, Department of Genetic Engineering and Biotechnology	Md. Nazmul Hasan, Md. Akkas Ali, Shamsul H. Prodhan, Hammadul Hoque , Nurnabi Azad Jewel, Md Mohsin Bapary, Diptha Chakraborthy
EPI_ISL_754064	Kathmandu University	Nepal Health Research Council	Pradip Gyanwali, Meghnath Dhimal
EPI_ISL_754068, EPI_ISL_754069	Nepal Korea Friendship Municipality Hospital	Nepal Health Research Council	Pradip Gyanwali, Meghnath Dhimal
EPI_ISL_754072, EPI_ISL_754073	National Public Health Laboratory	Nepal Health Research Council	Pradip Gyanwali, Meghnath Dhimal
EPI_ISL_754074, EPI_ISL_754075, EPI_ISL_754076, EPI_ISL_754077, EPI_ISL_754078, EPI_ISL_754079, EPI_ISL_754080, EPI_ISL_754081, EPI_ISL_754082, EPI_ISL_754083, EPI_ISL_754084, EPI_ISL_754085, EPI_ISL_754086, EPI_ISL_754087, EPI_ISL_754088, EPI_ISL_754089, EPI_ISL_754090, EPI_ISL_754091, EPI_ISL_754092, EPI_ISL_754093, EPI_ISL_754094, EPI_ISL_754095, EPI_ISL_754096, EPI_ISL_754097, EPI_ISL_754098, EPI_ISL_754099, EPI_ISL_754100, EPI_ISL_754101, EPI_ISL_754102, EPI_ISL_754103, EPI_ISL_754104, EPI_ISL_754105, EPI_ISL_754106, EPI_ISL_754107, EPI_ISL_754108			
see above	National Public Health Laboratory, National Centre for Infectious Diseases	National Public Health Laboratory, National Centre for Infectious Diseases	Tze Minn Mak, Sophie Octavia, Zhenyang Zhou, Lin Cui, Raymond Tzer Pin Lin
EPI_ISL_754109	Specialized Lab for COVID-19 Detection, Department of Genetic Engineering and Biotechnology	Specialized Lab for COVID-19 Detection, Department of Genetic Engineering and Biotechnology	Hammadul Hoque , Nurnabi Azad Jewel, Md. Nazmul Hasan, Shamsul H. Prodhan, Md. Mosarof Hossen, Md. Mashiur Rahaman
EPI_ISL_754110, EPI_ISL_754111, EPI_ISL_754112, EPI_ISL_754113, EPI_ISL_754114, EPI_ISL_754115, EPI_ISL_754116, EPI_ISL_754117, EPI_ISL_754118, EPI_ISL_754119, EPI_ISL_754120, EPI_ISL_754121, EPI_ISL_754122, EPI_ISL_754123			
see above	UCLA Clinical Micro Lab	Los Angeles County PHL	P. Hemarajata et al.
EPI_ISL_754124, EPI_ISL_754125, EPI_ISL_754126, EPI_ISL_754127, EPI_ISL_754128, EPI_ISL_754129	USC Clinical Lab	Los Angeles County PHL	P. Hemarajata et al.
EPI_ISL_754130, EPI_ISL_754131, EPI_ISL_754132	Los Angeles County PHL	Los Angeles County PHL	P. Hemarajata et al.
EPI_ISL_754133, EPI_ISL_754134, EPI_ISL_754135	USC Clinical Lab	Los Angeles County PHL	P. Hemarajata et al.
EPI_ISL_754136, EPI_ISL_754137, EPI_ISL_754138, EPI_ISL_754139	CHU Purpan - Laboratoire de Virologie - Institut Fédératif de Biologie	CHU Purpan - Laboratoire de Virologie - Institut Fédératif de Biologie	Latour J., Ranger N., Dubois M., Carcenac R., Harter A., Boyer P., Tremeaux P., Izopet J.
EPI_ISL_754141, EPI_ISL_754142, EPI_ISL_754143, EPI_ISL_754144, EPI_ISL_754145, EPI_ISL_754146, EPI_ISL_754147, EPI_ISL_754148, EPI_ISL_754149, EPI_ISL_754150, EPI_ISL_754151, EPI_ISL_754152, EPI_ISL_754153, EPI_ISL_754154, EPI_ISL_754155, EPI_ISL_754156, EPI_ISL_754157, EPI_ISL_754158, EPI_ISL_754159, EPI_ISL_754160, EPI_ISL_754161, EPI_ISL_754162, EPI_ISL_754163, EPI_ISL_754164, EPI_ISL_754165, EPI_ISL_754166, EPI_ISL_754167, EPI_ISL_754168, EPI_ISL_754169, EPI_ISL_754170, EPI_ISL_754171, EPI_ISL_754172			
see above	Laboratoire de Microbiologie	National Reference Center for Viruses of Respiratory Infections, Institut Pasteur, Paris	Marion Barbet, Sylvie Behillil, Méline Bizard, Angela Brisebarre, Camille Capel, Etienne Simon-Lorière, Vincent Enouf, Maud Vanpeene, Sylvie van der Werf, Marie-Sarah Fangous
EPI_ISL_754173	Specialized Lab for COVID-19 Detection, Department of Genetic Engineering and Biotechnology	Specialized Lab for COVID-19 Detection, Department of Genetic Engineering and Biotechnology	Md. Nazmul Hasan, Shamsul H. Prodhan, Hammadul Hoque , Nurnabi Azad Jewel, Mumtahin Ahammed, Mahedi Hasan Ridoy, Avishek Sarkar
EPI_ISL_754174, EPI_ISL_754175	Charité Universitätsmedizin Berlin, Institut für Virologie	Charité Universitätsmedizin Berlin, Institut für Virologie	Victor M Corman, Julia Schneider, Jörn Beheim-Schwarzbach, Barbara Mühlemann, Talitha Veith, Terry Jones, Christian Drosten
EPI_ISL_754176	Specialized Lab for COVID-19 Detection, Department of Genetic Engineering and	Specialized Lab for COVID-19 Detection, Department of Genetic	Shamsul H. Prodhan, Hammadul Hoque, Nurnabi Azad Jewel, Md. Nazmul Hasan, Md. Mainul Hossain Bakul, Md. Rabiul Awal

	Biotechnology	Engineering and Biotechnology	
EPI_ISL_754177	Specialized Lab for COVID-19 Detection, Department of Genetic Engineering and Biotechnology	Specialized Lab for COVID-19 Detection, Department of Genetic Engineering and Biotechnology	Nurnabi Azad Jewel, Md. Nazmul Hasan, Shamsul H. Prodhan, Hammadul Hoque, Asim Debnath, Sudipto Sakib Duranto
EPI_ISL_754179	Specialized Lab for COVID-19 Detection, Department of Genetic Engineering and Biotechnology	Specialized Lab for COVID-19 Detection, Department of Genetic Engineering and Biotechnology	Hammadul Hoque, Nurnabi Azad Jewel, Md. Nazmul Hasan, Shamsul H. Prodhan
EPI_ISL_754180	Department for Virology, Molecular Biology and Genome Research, R. G. Lugar Center for Public Health Research, National Center for Disease Control and Public Health (NCDC) of Georgia.	Department for Virology, Molecular Biology and Genome Research, R. G. Lugar Center for Public Health Research, National Center for Disease Control and Public Health (NCDC) of Georgia.	Salome Javashvili, Nino Berishvili, Tata Imnadze, Giorgi Tomashvili, Ana Papkiauri, Meri Pantsulaia, Gvantsa Brachveli, Gvantsa Chanturia, Ann Machablishvili, Nato Kotaria, Marine Murtskhvaladze, Lela Sabadze, Mari Gavashelidze, Tamar Jashiashvili, Tea Tevdoradze, Ketevan Sidamonidze, Ekaterine Khmaladze, Ekaterine Zhghenti, Roena Sukhiashvili, Mariam Zakalashvili, Lela Urushadze, Magda Dgebuadze, Davit Tsaguria, Ekaterine Zangaladze, Adam Kotorashvili, Maia Alkhazashvili, Irma Burjanadze, Anna Kasradze, Khatuna Zakhashvili, Paata Imnadze, Amiran Gamkrelidze.
EPI_ISL_754181	Department for Virology, Molecular Biology and Genome Research, R. G. Lugar Center for Public Health Research, National Center for Disease Control and Public Health (NCDC) of Georgia.	Department for Virology, Molecular Biology and Genome Research, R. G. Lugar Center for Public Health Research, National Center for Disease Control and Public Health (NCDC) of Georgia.	Meri Pantsulaia, Nino Berishvili, Tata Imnadze, Giorgi Tomashvili, Ana Papkiauri, Gvantsa Brachveli, Gvantsa Chanturia, Ann Machablishvili, Nato Kotaria, Marine Murtskhvaladze, Lela Sabadze, Mari Gavashelidze, Tamar Jashiashvili, Tea Tevdoradze, Ketevan Sidamonidze, Ekaterine Khmaladze, Ekaterine Zhghenti, Roena Sukhiashvili, Mariam Zakalashvili, Lela Urushadze, Magda Dgebuadze, Davit Tsaguria, Ekaterine Zangaladze, Adam Kotorashvili, Maia Alkhazashvili, Irma Burjanadze, Anna Kasradze, Khatuna Zakhashvili, Paata Imnadze, Amiran Gamkrelidze.
EPI_ISL_754182	Wyoming Public Health Laboratory	Wyoming Public Health Laboratory	Noah Hull, Taylor Fearing, Channing Weber, Ashley Norberg, Bailey Bowcutt, and Wanda Manley
EPI_ISL_754183	Wyoming Public Health Laboratory	Wyoming Public Health Laborator	Noah Hull, Taylor Fearing, Channing Weber, Ashley Norberg, Bailey Bowcutt, and Wanda Manley
EPI_ISL_754184, EPI_ISL_754185, EPI_ISL_754186, EPI_ISL_754187, EPI_ISL_754188, EPI_ISL_754189, EPI_ISL_754190, EPI_ISL_754191, EPI_ISL_754192, EPI_ISL_754193, EPI_ISL_754194			
see above	Charité Universitätsmedizin Berlin, Institut für Virologie/Labor Berlin	Charité Universitätsmedizin Berlin, Institut für Virologie	Victor M Corman, Jörn Beheim-Schwarzbach, Barbara Mühlemann, Julia Schneider, Talitha Veith, Terry Jones, Christian Drosten
EPI_ISL_754196, EPI_ISL_754197, EPI_ISL_754198, EPI_ISL_754199, EPI_ISL_754200, EPI_ISL_754201, EPI_ISL_754202, EPI_ISL_754203, EPI_ISL_754204, EPI_ISL_754205, EPI_ISL_754206, EPI_ISL_754207, EPI_ISL_754208, EPI_ISL_754209, EPI_ISL_754210, EPI_ISL_754211, EPI_ISL_754212, EPI_ISL_754213, EPI_ISL_754214			
see above	Molecular diagnostic laboratory of Federal Budget Institution of Science "Central Research Institute of Epidemiology" of The Federal Service on Customers' Rights Protection and Human Well-being Surveillance	Group of Genomics and Postgenomic Technologies of Central Research Institute of Epidemilology	Samoilov AE, Kaptelova VV, Korneenko EV, Saenko SS, Dudorova AV, Speranskaya AS, Tivanova EV, Shipulina OY, Akimkin VG
EPI_ISL_754215, EPI_ISL_754216, EPI_ISL_754217, EPI_ISL_754218, EPI_ISL_754219, EPI_ISL_754220, EPI_ISL_754221, EPI_ISL_754222, EPI_ISL_754223, EPI_ISL_754224, EPI_ISL_754225, EPI_ISL_754226, EPI_ISL_754227, EPI_ISL_754228			
see above	Wyoming Public Health Laboratory	Wyoming Public Health Laboratory	Noah Hull, Taylor Fearing, Channing Weber, Ashley Norberg, Bailey Bowcutt, and Wanda Manley
EPI_ISL_754230, EPI_ISL_754231, EPI_ISL_754232, EPI_ISL_754233, EPI_ISL_754235	The Republican Research and Practical Center for Epidemiology and Microbiology (RRPCEM)	WHO National Influenza Centre Russian Federation	Elena Gasich, Kirill Bulda, Anatoly Krasko, Andrey Komissarov, Artem Fadeev, Anna Ivanova, Kseniya Komissarova, Dmitry Bazhenov, Daria Danilenko, Ksenia Safina, Elena Nabieva, Georgii Bazykin, Dmitry Lioznov
EPI_ISL_754238	Laboratory for Respiratory Viruses, Cantacuzino National Military-Medical Institute for Research and Development	Cantacuzino Institute Virology	Luiza Ustea, Mühlemann Barbara, Mihaela Lazar
EPI_ISL_754239	Santosa Hospital Bandung Central	"School of Life Sciences and Technology & School of Pharmacy-Institut Teknologi Bandung; Molecular Genetics Laboratory-Faculty of Medicine-Universitas Padjadjaran; Laboratorium Kesehatan Provinsi Jawa Barat"	Marselina Irasonia Tan, Yunia Sribudiani, Catur Riani, Azzania Fibriani, Husna Nugrahapraja, Tarwadi, Ema Rahmawati, Savira Ekawardhani, Hesti Lina Wiraswati, Ryan Bayusantika Ristandi, Rifky Waluyajati Rachman, Cut Nur Cinthia Alamanda, Lia Faridah, Miftahul Faridl, Karimatu Khoirunnisa, Hammam Riza, Soni Solistia Wirawan, Agung Eru Wibowo, Irvan Faizal
EPI_ISL_754241	Dinkes Tasikmalaya	"School of Life Sciences and Technology & School of Pharmacy-Institut Teknologi Bandung; Molecular Genetics Laboratory-Faculty of Medicine-Universitas	Husna Nugrahapraja, Marselina Irasonia Tan, Yunia Sribudiani, Catur Riani, Azzania Fibriani, Tarwadi, Ema Rahmawati, Savira Ekawardhani, Hesti Lina Wiraswati, Ryan Bayusantika Ristandi, Rifky Waluyajati Rachman, Cut Nur Cinthia Alamanda, Lia Faridah, Miftahul Faridl, Karimatu Khoirunnisa, Hammam Riza, Soni Solistia Wirawan, Agung Eru Wibowo, Irvan Faizal

		Padjadjaran; Laboratorium Kesehatan Provinsi Jawa Barat"	
EPI_ISL_754242, EPI_ISL_754243, EPI_ISL_754244, EPI_ISL_754245, EPI_ISL_754246, EPI_ISL_754247, EPI_ISL_754248, EPI_ISL_754249, EPI_ISL_754250, EPI_ISL_754251, EPI_ISL_754252, EPI_ISL_754253, EPI_ISL_754254, EPI_ISL_754255, EPI_ISL_754256, EPI_ISL_754257, EPI_ISL_754258, EPI_ISL_754259, EPI_ISL_754260, EPI_ISL_754261, EPI_ISL_754262, EPI_ISL_754263, EPI_ISL_754264, EPI_ISL_754265, EPI_ISL_754267, EPI_ISL_754268, EPI_ISL_754269, EPI_ISL_754270, EPI_ISL_754271, EPI_ISL_754272, EPI_ISL_754273, EPI_ISL_754274, EPI_ISL_754275, EPI_ISL_754276, EPI_ISL_754277, EPI_ISL_754278, EPI_ISL_754279, EPI_ISL_754280, EPI_ISL_754281, EPI_ISL_754282, EPI_ISL_754283, EPI_ISL_754284, EPI_ISL_754285, EPI_ISL_754286, EPI_ISL_754287, EPI_ISL_754288, EPI_ISL_754290, EPI_ISL_754291, EPI_ISL_754292, EPI_ISL_754293, EPI_ISL_754294, EPI_ISL_754295, EPI_ISL_754296, EPI_ISL_754297, EPI_ISL_754298, EPI_ISL_754299, EPI_ISL_754300, EPI_ISL_754301, EPI_ISL_754302, EPI_ISL_754303, EPI_ISL_754304, EPI_ISL_754305, EPI_ISL_754306, EPI_ISL_754307, EPI_ISL_754308, EPI_ISL_754309, EPI_ISL_754310, EPI_ISL_754311, EPI_ISL_754312, EPI_ISL_754313, EPI_ISL_754314, EPI_ISL_754315, EPI_ISL_754316, EPI_ISL_754317, EPI_ISL_754318, EPI_ISL_754319, EPI_ISL_754320, EPI_ISL_754321, EPI_ISL_754322, EPI_ISL_754323, EPI_ISL_754324, EPI_ISL_754325, EPI_ISL_754326, EPI_ISL_754327, EPI_ISL_754328, EPI_ISL_754329, EPI_ISL_754330, EPI_ISL_754331, EPI_ISL_754332, EPI_ISL_754333, EPI_ISL_754334, EPI_ISL_754335, EPI_ISL_754336, EPI_ISL_754337, EPI_ISL_754338, EPI_ISL_754339, EPI_ISL_754340, EPI_ISL_754341, EPI_ISL_754342, EPI_ISL_754343, EPI_ISL_754344, EPI_ISL_754345, EPI_ISL_754346, EPI_ISL_754347, EPI_ISL_754348, EPI_ISL_754349, EPI_ISL_754350, EPI_ISL_754351, EPI_ISL_754352, EPI_ISL_754353, EPI_ISL_754354, EPI_ISL_754355, EPI_ISL_754356, EPI_ISL_754357, EPI_ISL_754358, EPI_ISL_754359, EPI_ISL_754360, EPI_ISL_754361, EPI_ISL_754362, EPI_ISL_754363, EPI_ISL_754364, EPI_ISL_754365, EPI_ISL_754366, EPI_ISL_754367, EPI_ISL_754368, EPI_ISL_754369, EPI_ISL_754370, EPI_ISL_754371, EPI_ISL_754372, EPI_ISL_754373, EPI_ISL_754374, EPI_ISL_754375, EPI_ISL_754376, EPI_ISL_754377, EPI_ISL_754378, EPI_ISL_754379, EPI_ISL_754380, EPI_ISL_754381, EPI_ISL_754382, EPI_ISL_754383, EPI_ISL_754384, EPI_ISL_754385, EPI_ISL_754386, EPI_ISL_754387			
see above	Respiratory Virus Unit, National Infection Service, Public Health England	COVID-19 Genomics UK (COG-UK) Consortium	PHE Covid Sequencing Team
EPI_ISL_754388, EPI_ISL_754389	Wyoming Public Health Laboratory	Wyoming Public Health Laboratory	Noah Hull, Taylor Fearing, Channing Weber, Ashley Norberg, Bailey Bowcutt, and Wanda Manley
EPI_ISL_754390	USC Clinical Lab	Los Angeles County PHL	P. Hemarajata et al.
EPI_ISL_754391, EPI_ISL_754392, EPI_ISL_754393	Genetica Molecular and Subdepartamento de Virologia ISP Chile	Insituto de Salud Publica de Chile	Javier Tognarelli, Barbara Parra, Loredana Arata, Jaime Lagos, Gisselle Barra, Patricia Bustos, Rodrigo Fasce, Andres Castillo, Jorge Fernandez
EPI_ISL_754394, EPI_ISL_754395, EPI_ISL_754396, EPI_ISL_754397, EPI_ISL_754398, EPI_ISL_754399, EPI_ISL_754400, EPI_ISL_754401, EPI_ISL_754402, EPI_ISL_754403, EPI_ISL_754404, EPI_ISL_754405, EPI_ISL_754406, EPI_ISL_754407, EPI_ISL_754408, EPI_ISL_754409, EPI_ISL_754410, EPI_ISL_754411, EPI_ISL_754412, EPI_ISL_754413, EPI_ISL_754414, EPI_ISL_754415, EPI_ISL_754416, EPI_ISL_754417, EPI_ISL_754418, EPI_ISL_754419, EPI_ISL_754420, EPI_ISL_754421, EPI_ISL_754422, EPI_ISL_754423, EPI_ISL_754424, EPI_ISL_754425, EPI_ISL_754426, EPI_ISL_754427, EPI_ISL_754428, EPI_ISL_754429, EPI_ISL_754430, EPI_ISL_754431, EPI_ISL_754432, EPI_ISL_754433, EPI_ISL_754434, EPI_ISL_754435, EPI_ISL_754436, EPI_ISL_754437, EPI_ISL_754438, EPI_ISL_754439, EPI_ISL_754440, EPI_ISL_754441, EPI_ISL_754442, EPI_ISL_754443, EPI_ISL_754444, EPI_ISL_754445, EPI_ISL_754446, EPI_ISL_754447, EPI_ISL_754448, EPI_ISL_754449, EPI_ISL_754450, EPI_ISL_754451, EPI_ISL_754452, EPI_ISL_754453, EPI_ISL_754454, EPI_ISL_754455, EPI_ISL_754456, EPI_ISL_754457, EPI_ISL_754458, EPI_ISL_754459, EPI_ISL_754460, EPI_ISL_754461, EPI_ISL_754462, EPI_ISL_754463, EPI_ISL_754464, EPI_ISL_754465, EPI_ISL_754466, EPI_ISL_754467, EPI_ISL_754468, EPI_ISL_754469, EPI_ISL_754470, EPI_ISL_754471, EPI_ISL_754472, EPI_ISL_754473, EPI_ISL_754474, EPI_ISL_754475, EPI_ISL_754476, EPI_ISL_754477, EPI_ISL_754478, EPI_ISL_754479, EPI_ISL_754480, EPI_ISL_754481, EPI_ISL_754482, EPI_ISL_754483, EPI_ISL_754484, EPI_ISL_754485, EPI_ISL_754487, EPI_ISL_754488, EPI_ISL_754489, EPI_ISL_754490, EPI_ISL_754491, EPI_ISL_754492, EPI_ISL_754493, EPI_ISL_754494, EPI_ISL_754495, EPI_ISL_754496, EPI_ISL_754497, EPI_ISL_754498, EPI_ISL_754499, EPI_ISL_754500, EPI_ISL_754501, EPI_ISL_754502, EPI_ISL_754503, EPI_ISL_754504, EPI_ISL_754505, EPI_ISL_754506, EPI_ISL_754507, EPI_ISL_754508, EPI_ISL_754509, EPI_ISL_754510, EPI_ISL_754511, EPI_ISL_754512, EPI_ISL_754513, EPI_ISL_754514, EPI_ISL_754515, EPI_ISL_754516, EPI_ISL_754517, EPI_ISL_754518, EPI_ISL_754519, EPI_ISL_754520, EPI_ISL_754521, EPI_ISL_754522, EPI_ISL_754523, EPI_ISL_754524, EPI_ISL_754525, EPI_ISL_754526, EPI_ISL_754527, EPI_ISL_754528, EPI_ISL_754529, EPI_ISL_754530, EPI_ISL_754531, EPI_ISL_754532, EPI_ISL_754533, EPI_ISL_754534, EPI_ISL_754535, EPI_ISL_754536, EPI_ISL_754537, EPI_ISL_754538, EPI_ISL_754539, EPI_ISL_754540, EPI_ISL_754541, EPI_ISL_754542, EPI_ISL_754543, EPI_ISL_754544, EPI_ISL_754545, EPI_ISL_754546, EPI_ISL_754547, EPI_ISL_754548, EPI_ISL_754549, EPI_ISL_754550, EPI_ISL_754551, EPI_ISL_754552, EPI_ISL_754553, EPI_ISL_754554, EPI_ISL_754555, EPI_ISL_754556, EPI_ISL_754557, EPI_ISL_754558, EPI_ISL_754559, EPI_ISL_754560, EPI_ISL_754561, EPI_ISL_754562, EPI_ISL_754563, EPI_ISL_754564, EPI_ISL_754565, EPI_ISL_754566, EPI_ISL_754568, EPI_ISL_754569, EPI_ISL_754570, EPI_ISL_754571, EPI_ISL_754572, EPI_ISL_754573, EPI_ISL_754574, EPI_ISL_754575, EPI_ISL_754576, EPI_ISL_754577, EPI_ISL_754578, EPI_ISL_754579, EPI_ISL_754580, EPI_ISL_754581, EPI_ISL_754582, EPI_ISL_754583, EPI_ISL_754584, EPI_ISL_754585, EPI_ISL_754586, EPI_ISL_754587, EPI_ISL_754588, EPI_ISL_754589, EPI_ISL_754590, EPI_ISL_754591, EPI_ISL_754592, EPI_ISL_754593, EPI_ISL_754594, EPI_ISL_754595, EPI_ISL_754596, EPI_ISL_754597, EPI_ISL_754598, EPI_ISL_754599, EPI_ISL_754600, EPI_ISL_754601, EPI_ISL_754602, EPI_ISL_754603, EPI_ISL_754604, EPI_ISL_754605, EPI_ISL_754606, EPI_ISL_754607, EPI_ISL_754608, EPI_ISL_754609, EPI_ISL_754610, EPI_ISL_754611, EPI_ISL_754612, EPI_ISL_754613, EPI_ISL_754614, EPI_ISL_754615, EPI_ISL_754616, EPI_ISL_754617, EPI_ISL_754618			
see above	Wadsworth Center, New York State Department.of Health	Wadsworth Center, New York State Department.of Health	Kirsten St. George, Daryl M. Lamson, Alexis Russel, Matthew Shudt, Melissa A Leisner, Jonathan Plitnick, Navjot Singh, John Kelly, Sara Griesemer, Erasmus Schneider, Erica Lasek-Nesselquist
EPI_ISL_754619, EPI_ISL_754620, EPI_ISL_754621, EPI_ISL_754622, EPI_ISL_754623, EPI_ISL_754625, EPI_ISL_754626, EPI_ISL_754627, EPI_ISL_754628, EPI_ISL_754629, EPI_ISL_754630, EPI_ISL_754631, EPI_ISL_754632, EPI_ISL_754633, EPI_ISL_754634, EPI_ISL_754635, EPI_ISL_754636, EPI_ISL_754637, EPI_ISL_754638, EPI_ISL_754639, EPI_ISL_754640, EPI_ISL_754641, EPI_ISL_754642, EPI_ISL_754643, EPI_ISL_754644, EPI_ISL_754645, EPI_ISL_754646, EPI_ISL_754647, EPI_ISL_754648, EPI_ISL_754649, EPI_ISL_754650, EPI_ISL_754651, EPI_ISL_754652, EPI_ISL_754653, EPI_ISL_754654, EPI_ISL_754655, EPI_ISL_754656, EPI_ISL_754657, EPI_ISL_754658, EPI_ISL_754659, EPI_ISL_754660, EPI_ISL_754661, EPI_ISL_754662, EPI_ISL_754663, EPI_ISL_754664, EPI_ISL_754665, EPI_ISL_754666, EPI_ISL_754667, EPI_ISL_754668, EPI_ISL_754669, EPI_ISL_754670, EPI_ISL_754671, EPI_ISL_754672, EPI_ISL_754673, EPI_ISL_754674, EPI_ISL_754675			
see above	University of Wisconsin-Madison AIDS Vaccine Research Laboratories	University of Wisconsin-Madison AIDS Vaccine Research Laboratories	Gage Moreno, Katarina Braun, et al. AIDS Vaccine Research Laboratories
EPI_ISL_754677, EPI_ISL_754678, EPI_ISL_754679, EPI_ISL_754680, EPI_ISL_754681, EPI_ISL_754682, EPI_ISL_754683, EPI_ISL_754684, EPI_ISL_754685, EPI_ISL_754686, EPI_ISL_754687, EPI_ISL_754688, EPI_ISL_754689, EPI_ISL_754690, EPI_ISL_754691, EPI_ISL_754692, EPI_ISL_754693, EPI_ISL_754694, EPI_ISL_754695, EPI_ISL_754696, EPI_ISL_754697, EPI_ISL_754698, EPI_ISL_754699, EPI_ISL_754700, EPI_ISL_754701, EPI_ISL_754702, EPI_ISL_754703, EPI_ISL_754704, EPI_ISL_754705, EPI_ISL_754706, EPI_ISL_754707, EPI_ISL_754708, EPI_ISL_754709, EPI_ISL_754710, EPI_ISL_754711, EPI_ISL_754712, EPI_ISL_754713, EPI_ISL_754714, EPI_ISL_754715, EPI_ISL_754716, EPI_ISL_754717, EPI_ISL_754718, EPI_ISL_754719, EPI_ISL_754720, EPI_ISL_754721, EPI_ISL_754722, EPI_ISL_754723, EPI_ISL_754724, EPI_ISL_754725, EPI_ISL_754726, EPI_ISL_754727, EPI_ISL_754728, EPI_ISL_754729, EPI_ISL_754730, EPI_ISL_754731, EPI_ISL_754732, EPI_ISL_754734, EPI_ISL_754735, EPI_ISL_754736, EPI_ISL_754737, EPI_ISL_754738, EPI_ISL_754739, EPI_ISL_754740, EPI_ISL_754741, EPI_ISL_754742, EPI_ISL_754743, EPI_ISL_754744, EPI_ISL_754745			
see above	Wadsworth Center, New York State Department.of Health	Wadsworth Center, New York State Department.of Health	Kirsten St. George, Daryl M. Lamson, Alexis Russel, Matthew Shudt, Melissa A Leisner, Jonathan Plitnick, Navjot Singh, John Kelly, Sara Griesemer, Erasmus Schneider, Erica Lasek-Nesselquist
EPI_ISL_754770, EPI_ISL_754771, EPI_ISL_754772, EPI_ISL_754773, EPI_ISL_754774, EPI_ISL_754775	Emory Molecular Diagnostics Laboratory, Emory Healthcare	Piantadosi Lab, Emory Department of Pathology	Ahmed Babiker, Anne Piantadosi
EPI_ISL_754779, EPI_ISL_754780, EPI_ISL_754781, EPI_ISL_754782, EPI_ISL_754783, EPI_ISL_754784, EPI_ISL_754785, EPI_ISL_754786, EPI_ISL_754787, EPI_ISL_754788, EPI_ISL_754789, EPI_ISL_754790, EPI_ISL_754791, EPI_ISL_754792, EPI_ISL_754793, EPI_ISL_754794, EPI_ISL_754795, EPI_ISL_754796, EPI_ISL_754797, EPI_ISL_754798, EPI_ISL_754799, EPI_ISL_754800, EPI_ISL_754801, EPI_ISL_754802, EPI_ISL_754803, EPI_ISL_754804, EPI_ISL_754805, EPI_ISL_754806, EPI_ISL_754807, EPI_ISL_754808, EPI_ISL_754809, EPI_ISL_754810, EPI_ISL_754811, EPI_ISL_754812, EPI_ISL_754813, EPI_ISL_754814, EPI_ISL_754815, EPI_ISL_754816, EPI_ISL_754817, EPI_ISL_754818, EPI_ISL_754819, EPI_ISL_754820, EPI_ISL_754821, EPI_ISL_754822, EPI_ISL_754823, EPI_ISL_754824, EPI_ISL_754825, EPI_ISL_754826, EPI_ISL_754827, EPI_ISL_754828, EPI_ISL_754829, EPI_ISL_754830, EPI_ISL_754831, EPI_ISL_754832, EPI_ISL_754833, EPI_ISL_754834, EPI_ISL_754835, EPI_ISL_754836, EPI_ISL_754837, EPI_ISL_754838, EPI_ISL_754839, EPI_ISL_754840, EPI_ISL_754841			
see above	Wadsworth Center, New York State Department.of Health	Wadsworth Center, New York State Department.of Health	Kirsten St. George, Daryl M. Lamson, Alexis Russel, Matthew Shudt, Melissa A Leisner, Jonathan Plitnick, Navjot Singh, John Kelly, Sara Griesemer, Erasmus Schneider, Erica Lasek-Nesselquist
EPI_ISL_754842	CHU Amiens - Labo virologie	National Reference Center for Viruses of Respiratory Infections, Institut Pasteur, Paris	Marion Barbet, Sylvie Behillil, Méline Bizard, Angela Brisebarre, Camille Capel, Etienne Simon-Lorière, Vincent Enouf, Maud Vanpeene, Sylvie van der Werf, Castelain Sandrine, François Catherine
EPI_ISL_754843, EPI_ISL_754844, EPI_ISL_754845, EPI_ISL_754846	Laboratoire de Virologie Hôpital Robert Debré	National Reference Center for Viruses of Respiratory Infections, Institut Pasteur, Paris	Marion Barbet, Sylvie Behillil, Méline Bizard, Angela Brisebarre, Camille Capel, Etienne Simon-Lorière, Vincent Enouf, Maud Vanpeene, Sylvie van der Werf, Andreoletti Laurent
EPI_ISL_754847, EPI_ISL_754848	Korian Villa d'Azon	National Reference Center for Viruses of Respiratory Infections, Institut Pasteur, Paris	Marion Barbet, Sylvie Behillil, Méline Bizard, Angela Brisebarre, Camille Capel, Etienne Simon-Lorière, Vincent Enouf, Maud Vanpeene, Sylvie van der Werf
EPI_ISL_754849, EPI_ISL_754850,	Laboratoire de Virologie Hôpital Robert Debré	National Reference Center for Viruses of	Marion Barbet, Sylvie Behillil, Méline Bizard, Angela Brisebarre, Camille Capel, Etienne Simon-Lorière, Vincent Enouf, Maud Vanpeene, Sylvie van der Werf, Andreoletti Laurent

EPI_ISL_754851, EPI_ISL_754852		Respiratory Infections, Institut Pasteur, Paris	
EPI_ISL_754853, EPI_ISL_754854, EPI_ISL_754855, EPI_ISL_754856	CHU - Hôpital Cavale Blanche - Labo. de Virologie	National Reference Center for Viruses of Respiratory Infections, Institut Pasteur, Paris	Marion Barbet, Sylvie Behillil, Méline Bizard, Angela Brisebarre, Camille Capel, Etienne Simon-Lorière, Vincent Enouf, Maud Vanpeene, Sylvie van der Werf, Pilorge léa
EPI_ISL_754857, EPI_ISL_754858, EPI_ISL_754859, EPI_ISL_754860, EPI_ISL_754864, EPI_ISL_754865, EPI_ISL_754866, EPI_ISL_754867, EPI_ISL_754868, EPI_ISL_754869, EPI_ISL_754870, EPI_ISL_754871, EPI_ISL_754872, EPI_ISL_754873, EPI_ISL_754874, EPI_ISL_754875, EPI_ISL_754876, EPI_ISL_754877, EPI_ISL_754878, EPI_ISL_754879, EPI_ISL_754880, EPI_ISL_754881, EPI_ISL_754882, EPI_ISL_754883, EPI_ISL_754884, EPI_ISL_754885, EPI_ISL_754886, EPI_ISL_754887, EPI_ISL_754888, EPI_ISL_754889, EPI_ISL_754890, EPI_ISL_754891, EPI_ISL_754892, EPI_ISL_754893, EPI_ISL_754894, EPI_ISL_754895, EPI_ISL_754896, EPI_ISL_754897, EPI_ISL_754898, EPI_ISL_754899, EPI_ISL_754900, EPI_ISL_754901			
see above	Innovative Genomics Institute, UC Berkeley	Innovative Genomics Institute, UC Berkeley	Stacia Wyman, Haridha Shivram, Phil Frankino, Liana Lareau, Shana McDevitt, Justin Choi
EPI_ISL_754902, EPI_ISL_754904, EPI_ISL_754905, EPI_ISL_754906, EPI_ISL_754907, EPI_ISL_754909, EPI_ISL_754910, EPI_ISL_754912, EPI_ISL_754913	Laboratory Diagnostics and Clinical Immunology of Developmental Age, Medical University of Warsaw	genXone SA, Research & Development Laboratory; The Faculty of Mathematics, Informatics and Mechanics of the University of Warsaw	Maciej Sykulski, Grzegorz Nowicki, Monika Makowska-Woniak, Jakub Grabowski, Natalia Drwska-Matelska, ukasz Krych, Micha Kaszuba, Anna Gambin, Urszula Demkow
EPI_ISL_754914, EPI_ISL_754915, EPI_ISL_754916, EPI_ISL_754917, EPI_ISL_754918, EPI_ISL_754919, EPI_ISL_754920, EPI_ISL_754921, EPI_ISL_754922, EPI_ISL_754923, EPI_ISL_754924, EPI_ISL_754925, EPI_ISL_754926, EPI_ISL_754928, EPI_ISL_754929, EPI_ISL_754931, EPI_ISL_754932, EPI_ISL_754933, EPI_ISL_754934, EPI_ISL_754936, EPI_ISL_754937, EPI_ISL_754938, EPI_ISL_754939, EPI_ISL_754940, EPI_ISL_754941, EPI_ISL_754942, EPI_ISL_754943, EPI_ISL_754944, EPI_ISL_754945, EPI_ISL_754947, EPI_ISL_754948, EPI_ISL_754949, EPI_ISL_754950, EPI_ISL_754951, EPI_ISL_754952, EPI_ISL_754954, EPI_ISL_754955, EPI_ISL_754956, EPI_ISL_754957, EPI_ISL_754958, EPI_ISL_754959, EPI_ISL_754960, EPI_ISL_754961, EPI_ISL_754963, EPI_ISL_754964, EPI_ISL_754965, EPI_ISL_754966, EPI_ISL_754967, EPI_ISL_754968, EPI_ISL_754969, EPI_ISL_754970, EPI_ISL_754971, EPI_ISL_754972, EPI_ISL_754973, EPI_ISL_754974, EPI_ISL_754975, EPI_ISL_754976, EPI_ISL_754977, EPI_ISL_754978, EPI_ISL_754979, EPI_ISL_754981, EPI_ISL_754982, EPI_ISL_754983, EPI_ISL_754985, EPI_ISL_754986, EPI_ISL_754987, EPI_ISL_754989, EPI_ISL_754990, EPI_ISL_754992, EPI_ISL_754993, EPI_ISL_754994, EPI_ISL_754995, EPI_ISL_754997, EPI_ISL_754998, EPI_ISL_754999, EPI_ISL_755000, EPI_ISL_755001, EPI_ISL_755002, EPI_ISL_755003, EPI_ISL_755004, EPI_ISL_755005, EPI_ISL_755006, EPI_ISL_755007, EPI_ISL_755008, EPI_ISL_755009, EPI_ISL_755010, EPI_ISL_755011, EPI_ISL_755012, EPI_ISL_755013, EPI_ISL_755014, EPI_ISL_755015, EPI_ISL_755031, EPI_ISL_755032, EPI_ISL_755034, EPI_ISL_755035, EPI_ISL_755042, EPI_ISL_755043, EPI_ISL_755044, EPI_ISL_755045, EPI_ISL_755046, EPI_ISL_755047, EPI_ISL_755048, EPI_ISL_755049, EPI_ISL_755050, EPI_ISL_755051, EPI_ISL_755052, EPI_ISL_755053, EPI_ISL_755054, EPI_ISL_755055, EPI_ISL_755056, EPI_ISL_755057, EPI_ISL_755058, EPI_ISL_755059, EPI_ISL_755060, EPI_ISL_755061, EPI_ISL_755062, EPI_ISL_755063, EPI_ISL_755064, EPI_ISL_755065, EPI_ISL_755066, EPI_ISL_755067, EPI_ISL_755068, EPI_ISL_755069, EPI_ISL_755070, EPI_ISL_755071, EPI_ISL_755072, EPI_ISL_755073, EPI_ISL_755074, EPI_ISL_755075, EPI_ISL_755076, EPI_ISL_755077, EPI_ISL_755078, EPI_ISL_755079, EPI_ISL_755080, EPI_ISL_755081, EPI_ISL_755082, EPI_ISL_755083, EPI_ISL_755084, EPI_ISL_755085, EPI_ISL_755086, EPI_ISL_755087, EPI_ISL_755088, EPI_ISL_755089, EPI_ISL_755090, EPI_ISL_755091, EPI_ISL_755092, EPI_ISL_755093, EPI_ISL_755094, EPI_ISL_755095, EPI_ISL_755096, EPI_ISL_755097, EPI_ISL_755098, EPI_ISL_755099, EPI_ISL_755100, EPI_ISL_755101, EPI_ISL_755102, EPI_ISL_755103, EPI_ISL_755104, EPI_ISL_755105, EPI_ISL_755106, EPI_ISL_755107, EPI_ISL_755108, EPI_ISL_755109, EPI_ISL_755110, EPI_ISL_755111, EPI_ISL_755112, EPI_ISL_755113, EPI_ISL_755114			
see above	California Department of Public Health	California Department of Public Health	CDPH IDLB COVIDNet
EPI_ISL_755115, EPI_ISL_755116, EPI_ISL_755117	San Diego County Public Health Laboratory	Andersen lab at Scripps Research	SEARCH Alliance San Diego with Tracy Basler, Jovan Shephard, Brett Austin
EPI_ISL_755118	Biolab Diagnostic Laboratories	Andersen lab at Scripps Research	Issa Abu-Dayyeh, Ahmad Tibi, Lama Hussein, Lina Mohammad, Zein Naber, Amid Abdelnour with SEARCH Alliance San Diego
EPI_ISL_755119	San Diego County Public Health Laboratory	Andersen lab at Scripps Research	SEARCH Alliance San Diego with Tracy Basler, Jovan Shephard, Brett Austin
EPI_ISL_755120, EPI_ISL_755121, EPI_ISL_755122, EPI_ISL_755123, EPI_ISL_755124, EPI_ISL_755125, EPI_ISL_755126, EPI_ISL_755127, EPI_ISL_755128, EPI_ISL_755129	Biolab Diagnostic Laboratories	Andersen lab at Scripps Research	Issa Abu-Dayyeh, Ahmad Tibi, Lama Hussein, Lina Mohammad, Zein Naber, Amid Abdelnour with SEARCH Alliance San Diego
EPI_ISL_755130	San Diego County Public Health Laboratory	Andersen lab at Scripps Research	SEARCH Alliance San Diego with Tracy Basler, Jovan Shephard, Brett Austin
EPI_ISL_755131	Biolab Diagnostic Laboratories	Andersen lab at Scripps Research	Issa Abu-Dayyeh, Ahmad Tibi, Lama Hussein, Lina Mohammad, Zein Naber, Amid Abdelnour with SEARCH Alliance San Diego
EPI_ISL_755132	San Diego County Public Health Laboratory	Andersen lab at Scripps Research	SEARCH Alliance San Diego with Tracy Basler, Jovan Shephard, Brett Austin
EPI_ISL_755133	UCSD EXCITE lab	Andersen lab at Scripps Research	SEARCH Alliance San Diego
EPI_ISL_755134, EPI_ISL_755135, EPI_ISL_755136	San Diego County Public Health Laboratory	Andersen lab at Scripps Research	SEARCH Alliance San Diego with Tracy Basler, Jovan Shephard, Brett Austin
EPI_ISL_755137	UCSD EXCITE lab	Andersen lab at Scripps Research	SEARCH Alliance San Diego
EPI_ISL_755138, EPI_ISL_755139	San Diego County Public Health Laboratory	Andersen lab at Scripps Research	SEARCH Alliance San Diego with Tracy Basler, Jovan Shephard, Brett Austin
EPI_ISL_755140	UCSD EXCITE lab	Andersen lab at Scripps Research	SEARCH Alliance San Diego
EPI_ISL_755141, EPI_ISL_755142, EPI_ISL_755143, EPI_ISL_755144, EPI_ISL_755145	Biolab Diagnostic Laboratories	Andersen lab at Scripps Research	Issa Abu-Dayyeh, Ahmad Tibi, Lama Hussein, Lina Mohammad, Zein Naber, Amid Abdelnour with SEARCH Alliance San Diego
EPI_ISL_755146, EPI_ISL_755147, EPI_ISL_755148, EPI_ISL_755149, EPI_ISL_755150, EPI_ISL_755151, EPI_ISL_755152, EPI_ISL_755153, EPI_ISL_755154, EPI_ISL_755155, EPI_ISL_755156, EPI_ISL_755157, EPI_ISL_755158, EPI_ISL_755159, EPI_ISL_755160, EPI_ISL_755161, EPI_ISL_755162, EPI_ISL_755163, EPI_ISL_755164, EPI_ISL_755165, EPI_ISL_755166, EPI_ISL_755167, EPI_ISL_755168, EPI_ISL_755169, EPI_ISL_755170, EPI_ISL_755171, EPI_ISL_755172, EPI_ISL_755173, EPI_ISL_755174, EPI_ISL_755175, EPI_ISL_755176, EPI_ISL_755177, EPI_ISL_755178, EPI_ISL_755179, EPI_ISL_755180, EPI_ISL_755181, EPI_ISL_755182, EPI_ISL_755183, EPI_ISL_755184, EPI_ISL_755185, EPI_ISL_755186, EPI_ISL_755187, EPI_ISL_755188, EPI_ISL_755189, EPI_ISL_755190, EPI_ISL_755191, EPI_ISL_755192, EPI_ISL_755193, EPI_ISL_755194, EPI_ISL_755195, EPI_ISL_755196, EPI_ISL_755197, EPI_ISL_755198, EPI_ISL_755199, EPI_ISL_755200, EPI_ISL_755201, EPI_ISL_755202, EPI_ISL_755203, EPI_ISL_755204, EPI_ISL_755205, EPI_ISL_755206, EPI_ISL_755207, EPI_ISL_755208, EPI_ISL_755209			
see above	UCSD EXCITE lab	Andersen lab at Scripps Research	SEARCH Alliance San Diego

EPI_ISL_755210, EPI_ISL_755211, EPI_ISL_755212, EPI_ISL_755213, EPI_ISL_755214	Biolab Diagnostic Laboratories	Andersen lab at Scripps Research	Issa Abu-Dayyeh, Ahmad Tibi, Lama Hussein, Lina Mohammad, Zein Naber, Amid Abdelnour with SEARCH Alliance San Diego
EPI_ISL_755215, EPI_ISL_755216, EPI_ISL_755217, EPI_ISL_755218, EPI_ISL_755219, EPI_ISL_755220, EPI_ISL_755221, EPI_ISL_755222, EPI_ISL_755223, EPI_ISL_755224, EPI_ISL_755225, EPI_ISL_755226, EPI_ISL_755227, EPI_ISL_755228, EPI_ISL_755229, EPI_ISL_755230, EPI_ISL_755231			
see above	UCSD EXCITE lab	Andersen lab at Scripps Research	SEARCH Alliance San Diego
EPI_ISL_755232, EPI_ISL_755233, EPI_ISL_755234, EPI_ISL_755235, EPI_ISL_755236, EPI_ISL_755237, EPI_ISL_755238, EPI_ISL_755239, EPI_ISL_755240, EPI_ISL_755241, EPI_ISL_755242, EPI_ISL_755243, EPI_ISL_755244, EPI_ISL_755245, EPI_ISL_755246, EPI_ISL_755247, EPI_ISL_755248, EPI_ISL_755249, EPI_ISL_755250, EPI_ISL_755251, EPI_ISL_755252, EPI_ISL_755253, EPI_ISL_755254, EPI_ISL_755255, EPI_ISL_755256, EPI_ISL_755257, EPI_ISL_755258, EPI_ISL_755259, EPI_ISL_755260, EPI_ISL_755261, EPI_ISL_755262, EPI_ISL_755263, EPI_ISL_755264, EPI_ISL_755265, EPI_ISL_755266, EPI_ISL_755267, EPI_ISL_755268			
see above	Biolab Diagnostic Laboratories	Andersen lab at Scripps Research	Issa Abu-Dayyeh, Ahmad Tibi, Lama Hussein, Lina Mohammad, Zein Naber, Amid Abdelnour with SEARCH Alliance San Diego
EPI_ISL_755269, EPI_ISL_755270, EPI_ISL_755271, EPI_ISL_755272, EPI_ISL_755273, EPI_ISL_755274, EPI_ISL_755275, EPI_ISL_755276, EPI_ISL_755277, EPI_ISL_755278, EPI_ISL_755279, EPI_ISL_755280, EPI_ISL_755281, EPI_ISL_755282, EPI_ISL_755283, EPI_ISL_755284, EPI_ISL_755285, EPI_ISL_755286, EPI_ISL_755287, EPI_ISL_755288, EPI_ISL_755289, EPI_ISL_755290, EPI_ISL_755291, EPI_ISL_755292, EPI_ISL_755293, EPI_ISL_755294, EPI_ISL_755295, EPI_ISL_755296, EPI_ISL_755297, EPI_ISL_755298, EPI_ISL_755299, EPI_ISL_755300			
see above	San Diego County Public Health Laboratory	Andersen lab at Scripps Research	SEARCH Alliance San Diego with Tracy Basler, Jovan Shephard, Brett Austin
EPI_ISL_755302, EPI_ISL_755303	Laboratorio de Bioingeniería, Instituto de Ciencias de la Ingeniería, Universidad de O´Higgins	Center for Mathematical Modeling and Center for Genome Regulation. Santiago, Chile	M. Latorre, G. Galvez, J. Ortega, Bastias M, Sanhueza D, Travisany D, Allende ML, Maass A, González M, Bustos F, Arriagada G, Montecino, M, Orellana A, Castro E, Meneses C.
EPI_ISL_755306, EPI_ISL_755307, EPI_ISL_755308, EPI_ISL_755309, EPI_ISL_755311, EPI_ISL_755312, EPI_ISL_755313, EPI_ISL_755314, EPI_ISL_755315, EPI_ISL_755316, EPI_ISL_755317, EPI_ISL_755318, EPI_ISL_755319, EPI_ISL_755320, EPI_ISL_755321, EPI_ISL_755322, EPI_ISL_755323, EPI_ISL_755324, EPI_ISL_755325, EPI_ISL_755326, EPI_ISL_755327, EPI_ISL_755328, EPI_ISL_755329, EPI_ISL_755330, EPI_ISL_755331, EPI_ISL_755332, EPI_ISL_755333, EPI_ISL_755334, EPI_ISL_755335, EPI_ISL_755336, EPI_ISL_755337, EPI_ISL_755338, EPI_ISL_755339, EPI_ISL_755340, EPI_ISL_755341, EPI_ISL_755342, EPI_ISL_755343, EPI_ISL_755344, EPI_ISL_755345, EPI_ISL_755346, EPI_ISL_755347, EPI_ISL_755348, EPI_ISL_755349, EPI_ISL_755350, EPI_ISL_755351, EPI_ISL_755352, EPI_ISL_755353, EPI_ISL_755354, EPI_ISL_755355, EPI_ISL_755356, EPI_ISL_755357, EPI_ISL_755358, EPI_ISL_755359, EPI_ISL_755360, EPI_ISL_755361, EPI_ISL_755362, EPI_ISL_755363, EPI_ISL_755364, EPI_ISL_755365, EPI_ISL_755366, EPI_ISL_755367, EPI_ISL_755368, EPI_ISL_755369, EPI_ISL_755370, EPI_ISL_755371, EPI_ISL_755372, EPI_ISL_755373, EPI_ISL_755374, EPI_ISL_755375, EPI_ISL_755376, EPI_ISL_755377, EPI_ISL_755378, EPI_ISL_755379, EPI_ISL_755380, EPI_ISL_755381, EPI_ISL_755382, EPI_ISL_755383, EPI_ISL_755384, EPI_ISL_755385, EPI_ISL_755386, EPI_ISL_755387, EPI_ISL_755388, EPI_ISL_755389, EPI_ISL_755390, EPI_ISL_755391, EPI_ISL_755392, EPI_ISL_755393, EPI_ISL_755394, EPI_ISL_755395, EPI_ISL_755396, EPI_ISL_755397, EPI_ISL_755398, EPI_ISL_755399, EPI_ISL_755400, EPI_ISL_755401, EPI_ISL_755402, EPI_ISL_755403, EPI_ISL_755404, EPI_ISL_755405, EPI_ISL_755406, EPI_ISL_755407, EPI_ISL_755408, EPI_ISL_755409, EPI_ISL_755410, EPI_ISL_755411, EPI_ISL_755412, EPI_ISL_755413, EPI_ISL_755414, EPI_ISL_755415, EPI_ISL_755416, EPI_ISL_755417, EPI_ISL_755418, EPI_ISL_755419, EPI_ISL_755420, EPI_ISL_755421, EPI_ISL_755422, EPI_ISL_755423, EPI_ISL_755424, EPI_ISL_755425, EPI_ISL_755427, EPI_ISL_755428, EPI_ISL_755429, EPI_ISL_755430, EPI_ISL_755431, EPI_ISL_755432, EPI_ISL_755433, EPI_ISL_755434, EPI_ISL_755435, EPI_ISL_755436, EPI_ISL_755437, EPI_ISL_755438, EPI_ISL_755439, EPI_ISL_755440, EPI_ISL_755441, EPI_ISL_755442, EPI_ISL_755443, EPI_ISL_755444, EPI_ISL_755445, EPI_ISL_755446, EPI_ISL_755447, EPI_ISL_755448, EPI_ISL_755449, EPI_ISL_755450, EPI_ISL_755451, EPI_ISL_755452, EPI_ISL_755453, EPI_ISL_755454, EPI_ISL_755455, EPI_ISL_755457, EPI_ISL_755458, EPI_ISL_755460, EPI_ISL_755461, EPI_ISL_755462, EPI_ISL_755463, EPI_ISL_755464, EPI_ISL_755465, EPI_ISL_755466, EPI_ISL_755467, EPI_ISL_755468, EPI_ISL_755469, EPI_ISL_755470, EPI_ISL_755471, EPI_ISL_755472, EPI_ISL_755473, EPI_ISL_755474, EPI_ISL_755475, EPI_ISL_755476, EPI_ISL_755477, EPI_ISL_755478, EPI_ISL_755479, EPI_ISL_755480, EPI_ISL_755481, EPI_ISL_755482, EPI_ISL_755483, EPI_ISL_755484, EPI_ISL_755485, EPI_ISL_755486, EPI_ISL_755487, EPI_ISL_755488, EPI_ISL_755489, EPI_ISL_755490, EPI_ISL_755491, EPI_ISL_755492, EPI_ISL_755493, EPI_ISL_755494, EPI_ISL_755495, EPI_ISL_755496, EPI_ISL_755497, EPI_ISL_755498, EPI_ISL_755499, EPI_ISL_755500, EPI_ISL_755501, EPI_ISL_755502, EPI_ISL_755503, EPI_ISL_755504, EPI_ISL_755505, EPI_ISL_755506, EPI_ISL_755507, EPI_ISL_755508, EPI_ISL_755509, EPI_ISL_755510, EPI_ISL_755511, EPI_ISL_755512, EPI_ISL_755513, EPI_ISL_755514, EPI_ISL_755515, EPI_ISL_755517, EPI_ISL_755518, EPI_ISL_755519, EPI_ISL_755520, EPI_ISL_755522, EPI_ISL_755523, EPI_ISL_755524, EPI_ISL_755525, EPI_ISL_755526, EPI_ISL_755527, EPI_ISL_755528, EPI_ISL_755529, EPI_ISL_755530, EPI_ISL_755531, EPI_ISL_755532, EPI_ISL_755533, EPI_ISL_755534, EPI_ISL_755535, EPI_ISL_755536, EPI_ISL_755537, EPI_ISL_755538, EPI_ISL_755539, EPI_ISL_755540, EPI_ISL_755541, EPI_ISL_755542, EPI_ISL_755543, EPI_ISL_755544, EPI_ISL_755545, EPI_ISL_755546, EPI_ISL_755547, EPI_ISL_755548, EPI_ISL_755549, EPI_ISL_755550, EPI_ISL_755551, EPI_ISL_755552, EPI_ISL_755553, EPI_ISL_755554, EPI_ISL_755555, EPI_ISL_755556, EPI_ISL_755557, EPI_ISL_755558, EPI_ISL_755559, EPI_ISL_755560, EPI_ISL_755561, EPI_ISL_755562, EPI_ISL_755563, EPI_ISL_755564, EPI_ISL_755565, EPI_ISL_755566, EPI_ISL_755567			
see above	Maine Health and Environmental Testing Laboratory	Tewhey Lab, The Jackson Laboratory	Matluk,N., Dewey,H., Iosue,F., Barter,M., Lynch,R., Munger,H. and Tewhey,R.
EPI_ISL_755568, EPI_ISL_755569, EPI_ISL_755570, EPI_ISL_755571	SA Pathology	SA Pathology	Lex Leong, Julien Soubrier, Chuan Kok Lim, Song Gao, Mark Turra, Karin Kassahn, Ivan Bastian, Geoff Higgins
EPI_ISL_755572, EPI_ISL_755573, EPI_ISL_755574	Center of Advanced Studies and Technology, CAST	Center of Advanced Studies and Technology, CAST	Ferrante,R., Mandatori,D., De Fabritiis,S.
EPI_ISL_755588	Regional medical sciences center 6 Chonburi	National Institute of Health, Department of Medical Sciences, Ministry of Public Health, Thailand	Pilailuk Okada; Siripaporn Phuygun; Thanutsapa Thanadachakul; Sittiporn Parnmen; Pakorn Piromtong; Warawan Wongboot; Sunthareeya Waicharoen; Malinee Chittaganpitch
EPI_ISL_755589	Laboratoire national de santé, Microbiology, Virology	Laboratoire national de santé, Microbiology, Microbial Genomics Platform	Anke Wienecke-Baldacchino, Catherine Ragimbeau,Jessica Tapp, Fatu Djabi, Lise Pignon, Raoul Salmon, Tamir Abdelrahman
EPI_ISL_755590, EPI_ISL_755591, EPI_ISL_755592, EPI_ISL_755593, EPI_ISL_755594, EPI_ISL_755595, EPI_ISL_755596, EPI_ISL_755597, EPI_ISL_755598, EPI_ISL_755599, EPI_ISL_755600, EPI_ISL_755601, EPI_ISL_755602, EPI_ISL_755603, EPI_ISL_755604, EPI_ISL_755605, EPI_ISL_755606, EPI_ISL_755607, EPI_ISL_755608, EPI_ISL_755609, EPI_ISL_755610, EPI_ISL_755611, EPI_ISL_755612, EPI_ISL_755613, EPI_ISL_755616, EPI_ISL_755617, EPI_ISL_755618			
see above	Helix/Illumina	Genomics and Discovery, Respiratory Viruses Branch, Division of Viral Diseases, Centers for Disease Control and Prevention	Peter W. Cook, Dhwani Batra, Eileen de Feo, Jan Antico, Christine Tran, Matthew Tolentino, Shannon Wickline, Kim Gietzen, Brad Sickler, Jingtao Liu, Eric Allen, Phil Febbo, Summer Galloway, Nicole L. Washington, Simon White, Geraint Levan, Kelly Schiabor Barrett, Elizabeth Cirulli, Alexandre Bolze, Ary Ascencio, Charlotte Rivera-Garcia, Ryan Cho, Jason Nguyen, Sherry Wang, Jimmy Ramirez, Tyler Cassens, Efren Sandoval, Magnus Isaksson, William Lee, David Becker, Marc Laurent, James Lu, Clinton R. Paden, Suxiang Tong, Duncan MacCannell
EPI_ISL_755620, EPI_ISL_755621, EPI_ISL_755622	Canterbury Health Laboratories	Institute of Environmental Science and Research (ESR)	Xiaoyun Ren, Matt Storey, Nikki Freed, Muhammad Faisal, Jing Wang, Hermes Perez, Anja Werno, Antje van der Linden, Arlo Upton, Chris Mansell, David Hammer, Dragana Drinkovic, Gary McAuliffe, Hana Sofia Andersson, James Ussher, Jill Sherwood, Josh Freeman, Julia Howard, Juliet Elvy, Mary DeAlmeida, Matt Blakiston, Matthew Rogers, Max Bloomfield, Michael Addidle, Michelle Balm, Sally Roberts, Sarah Jefferies, Sharmini Muttaiyah, Susan Morpeth, Susan Taylor, Timothy Blackmore, Vani Sathyendran, Veronica Playle, Virginia Hope, Erasmus Smit, Lauren Jelly, Olin Silander, Joep de Ligt
EPI_ISL_755624	Middlemore Hospital	Institute of Environmental Science and Research (ESR)	Xiaoyun Ren, Matt Storey, Nikki Freed, Muhammad Faisal, Jing Wang, Hermes Perez, Anja Werno, Antje van der Linden, Arlo Upton, Chris Mansell, David Hammer, Dragana Drinkovic, Gary McAuliffe, Hana Sofia Andersson, James Ussher, Jill Sherwood, Josh Freeman, Julia Howard, Juliet Elvy, Mary DeAlmeida, Matt Blakiston, Matthew Rogers, Max Bloomfield, Michael Addidle, Michelle Balm, Sally Roberts, Sarah Jefferies, Sharmini Muttaiyah, Susan Morpeth, Susan Taylor, Timothy Blackmore, Vani Sathyendran, Veronica Playle, Virginia Hope, Erasmus Smit, Lauren Jelly, Olin Silander, Joep de Ligt
EPI_ISL_755626	PathLab Bay of Plenty	Institute of Environmental Science and Research (ESR)	Xiaoyun Ren, Matt Storey, Nikki Freed, Muhammad Faisal, Jing Wang, Hermes Perez, Anja Werno, Antje van der Linden, Arlo Upton, Chris Mansell, David Hammer, Dragana Drinkovic, Gary McAuliffe, Hana Sofia Andersson, James Ussher, Jill Sherwood, Josh Freeman, Julia Howard, Juliet Elvy, Mary DeAlmeida, Matt Blakiston, Matthew Rogers, Max Bloomfield, Michael Addidle, Michelle Balm, Sally Roberts, Sarah Jefferies, Sharmini Muttaiyah, Susan Morpeth, Susan Taylor, Timothy Blackmore, Vani Sathyendran, Veronica Playle, Virginia Hope, Erasmus Smit, Lauren Jelly, Olin Silander, Joep de Ligt
EPI_ISL_755627, EPI_ISL_755628, EPI_ISL_755629, EPI_ISL_755630, EPI_ISL_755631, EPI_ISL_755632, EPI_ISL_755633	LabPLUS	Institute of Environmental Science and Research (ESR)	Xiaoyun Ren, Matt Storey, Nikki Freed, Muhammad Faisal, Jing Wang, Hermes Perez, Anja Werno, Antje van der Linden, Arlo Upton, Chris Mansell, David Hammer, Dragana Drinkovic, Gary McAuliffe, Hana Sofia Andersson, James Ussher, Jill Sherwood, Josh Freeman, Julia Howard, Juliet Elvy, Mary DeAlmeida, Matt Blakiston, Matthew Rogers, Max Bloomfield, Michael Addidle, Michelle Balm, Sally Roberts, Sarah Jefferies, Sharmini Muttaiyah, Susan Morpeth, Susan Taylor, Timothy Blackmore, Vani Sathyendran, Veronica Playle, Virginia Hope, Erasmus Smit, Lauren Jelly, Olin Silander, Joep de Ligt

EPI_ISL_755634, EPI_ISL_755635, EPI_ISL_755636	Middlemore Hospital	Institute of Environmental Science and Research (ESR)	Xiaoyun Ren, Matt Storey, Nikki Freed, Muhammad Faisal, Jing Wang, Hermes Perez, Anja Werno, Antje van der Linden, Arlo Upton, Chris Mansell, David Hammer, Dragana Drinkovic, Gary McAuliffe, Hana Sofia Andersson, James Ussher, Jill Sherwood, Josh Freeman, Julia Howard, Juliet Elvy, Mary DeAlmeida, Matt Blakiston, Matthew Rogers, Max Bloomfield, Michael Addidle, Michelle Balm, Sally Roberts, Sarah Jefferies, Sharmini Muttaiyah, Susan Morpeth, Susan Taylor, Timothy Blackmore, Vani Sathyendran, Veronica Playle, Virginia Hope, Erasmus Smit, Lauren Jelly, Olin Silander, Joep de Ligt
EPI_ISL_755637	LabPLUS	Institute of Environmental Science and Research (ESR)	Xiaoyun Ren, Matt Storey, Nikki Freed, Muhammad Faisal, Jing Wang, Hermes Perez, Anja Werno, Antje van der Linden, Arlo Upton, Chris Mansell, David Hammer, Dragana Drinkovic, Gary McAuliffe, Hana Sofia Andersson, James Ussher, Jill Sherwood, Josh Freeman, Julia Howard, Juliet Elvy, Mary DeAlmeida, Matt Blakiston, Matthew Rogers, Max Bloomfield, Michael Addidle, Michelle Balm, Sally Roberts, Sarah Jefferies, Sharmini Muttaiyah, Susan Morpeth, Susan Taylor, Timothy Blackmore, Vani Sathyendran, Veronica Playle, Virginia Hope, Erasmus Smit, Lauren Jelly, Olin Silander, Joep de Ligt
EPI_ISL_755638	Canterbury Health Laboratories	Institute of Environmental Science and Research (ESR)	Xiaoyun Ren, Matt Storey, Nikki Freed, Muhammad Faisal, Jing Wang, Hermes Perez, Anja Werno, Antje van der Linden, Arlo Upton, Chris Mansell, David Hammer, Dragana Drinkovic, Gary McAuliffe, Hana Sofia Andersson, James Ussher, Jill Sherwood, Josh Freeman, Julia Howard, Juliet Elvy, Mary DeAlmeida, Matt Blakiston, Matthew Rogers, Max Bloomfield, Michael Addidle, Michelle Balm, Sally Roberts, Sarah Jefferies, Sharmini Muttaiyah, Susan Morpeth, Susan Taylor, Timothy Blackmore, Vani Sathyendran, Veronica Playle, Virginia Hope, Erasmus Smit, Lauren Jelly, Olin Silander, Joep de Ligt
EPI_ISL_755639	Bundeswehr Institute of Microbiology	Bundeswehr Institute of Microbiology	Malena Bestehorn-Willmann, Mathias Walter, Christina Bugert, Joachim Bugert, Roman Wölfel, Markus Antwerpen
EPI_ISL_755640	Instituto Adolfo Lutz - Central	Instituto Adolfo Lutz, Interdiciplinary Procedures Center, Strategic Laboratory	Claudio Tavares Sacchi, Claudia Regina Gonçalves, Erica Valessa Ramos Gomes, Karoline Rodrigues Campos
EPI_ISL_755641	Instituto Adolfo Lutz - Regional de Santo Andre	Instituto Adolfo Lutz, Interdiciplinary Procedures Center, Strategic Laboratory	Claudio Tavares Sacchi, Claudia Regina Gonçalves, Erica Valessa Ramos Gomes, Karoline Rodrigues Campos
EPI_ISL_755642, EPI_ISL_755643	Instituto Adolfo Lutz - Central	Instituto Adolfo Lutz, Interdiciplinary Procedures Center, Strategic Laboratory	Claudio Tavares Sacchi, Claudia Regina Gonçalves, Erica Valessa Ramos Gomes, Karoline Rodrigues Campos
EPI_ISL_755644, EPI_ISL_755645	Lab LOC - Itapecerica da Serra	Instituto Adolfo Lutz, Interdiciplinary Procedures Center, Strategic Laboratory	Claudio Tavares Sacchi, Claudia Regina Gonçalves, Erica Valessa Ramos Gomes, Karoline Rodrigues Campos
EPI_ISL_755646, EPI_ISL_755647	Instituto Adolfo Lutz - Regional de Santo Andre	Instituto Adolfo Lutz, Interdiciplinary Procedures Center, Strategic Laboratory	Claudio Tavares Sacchi, Claudia Regina Gonçalves, Erica Valessa Ramos Gomes, Karoline Rodrigues Campos
EPI_ISL_755648	Instituto Adolfo Lutz - Regional de Taubate	Instituto Adolfo Lutz, Interdiciplinary Procedures Center, Strategic Laboratory	Claudio Tavares Sacchi, Claudia Regina Gonçalves, Erica Valessa Ramos Gomes, Karoline Rodrigues Campos
EPI_ISL_755649	Instituto Adolfo Lutz - Regional de Santo Andre	Instituto Adolfo Lutz, Interdiciplinary Procedures Center, Strategic Laboratory	Claudio Tavares Sacchi, Claudia Regina Gonçalves, Erica Valessa Ramos Gomes, Karoline Rodrigues Campos
EPI_ISL_755650	Instituto Adolfo Lutz - Regional de Taubate	Instituto Adolfo Lutz, Interdiciplinary Procedures Center, Strategic Laboratory	Claudio Tavares Sacchi, Claudia Regina Gonçalves, Erica Valessa Ramos Gomes, Karoline Rodrigues Campos
EPI_ISL_755651	Instituto Adolfo Lutz - Central	Instituto Adolfo Lutz, Interdiciplinary Procedures Center, Strategic Laboratory	Claudio Tavares Sacchi, Claudia Regina Gonçalves, Erica Valessa Ramos Gomes, Karoline Rodrigues Campos
EPI_ISL_755652	Lab LOC - Itapecerica da Serra	Instituto Adolfo Lutz, Interdiciplinary Procedures Center, Strategic Laboratory	Claudio Tavares Sacchi, Claudia Regina Gonçalves, Erica Valessa Ramos Gomes, Karoline Rodrigues Campos
EPI_ISL_755653, EPI_ISL_755654	Instituto Adolfo Lutz - Central	Instituto Adolfo Lutz, Interdiciplinary Procedures Center, Strategic Laboratory	Claudio Tavares Sacchi, Claudia Regina Gonçalves, Erica Valessa Ramos Gomes, Karoline Rodrigues Campos
EPI_ISL_755655	Instituto Adolfo Lutz - Regional de Campinas	Instituto Adolfo Lutz, Interdiciplinary Procedures Center, Strategic Laboratory	Claudio Tavares Sacchi, Claudia Regina Gonçalves, Erica Valessa Ramos Gomes, Karoline Rodrigues Campos
EPI_ISL_755656, EPI_ISL_755657, EPI_ISL_755658, EPI_ISL_755659, EPI_ISL_755660, EPI_ISL_755661, EPI_ISL_755662, EPI_ISL_755663, EPI_ISL_755664, EPI_ISL_755665, EPI_ISL_755666, EPI_ISL_755667, EPI_ISL_755668, EPI_ISL_755669, EPI_ISL_755670, EPI_ISL_755671, EPI_ISL_755672, EPI_ISL_755673, EPI_ISL_755674, EPI_ISL_755675, EPI_ISL_755676, EPI_ISL_755677, EPI_ISL_755678, EPI_ISL_755679, EPI_ISL_755680, EPI_ISL_755681, EPI_ISL_755682, EPI_ISL_755683, EPI_ISL_755684, EPI_ISL_755685, EPI_ISL_755686, EPI_ISL_755687, EPI_ISL_755688, EPI_ISL_755689, EPI_ISL_755690, EPI_ISL_755691, EPI_ISL_755692, EPI_ISL_755693, EPI_ISL_755694, EPI_ISL_755695, EPI_ISL_755696, EPI_ISL_755697, EPI_ISL_755698, EPI_ISL_755699, EPI_ISL_755700, EPI_ISL_755701, EPI_ISL_755702, EPI_ISL_755703, EPI_ISL_755704, EPI_ISL_755705, EPI_ISL_755706, EPI_ISL_755707, EPI_ISL_755708, EPI_ISL_755709, EPI_ISL_755710, EPI_ISL_755711			
see above	Toronto Invasive Bacterial Diseases Network	McMaster University	Allison McGeer, Patryk Aftanas, Hooman Derakhshani, Angel Li, Kuganya Nirmalarajah, Emily Panousis, Ahmed Draia, Jalees Nasir, Michael Surette, Samira Mubareka, Andrew G. McArthur
EPI_ISL_755712, EPI_ISL_755713, EPI_ISL_755714, EPI_ISL_755715, EPI_ISL_755716, EPI_ISL_755717, EPI_ISL_755718, EPI_ISL_755719, EPI_ISL_755720, EPI_ISL_755721, EPI_ISL_755722, EPI_ISL_755723, EPI_ISL_755724, EPI_ISL_755725, EPI_ISL_755726, EPI_ISL_755727, EPI_ISL_755728, EPI_ISL_755729, EPI_ISL_755730, EPI_ISL_755731, EPI_ISL_755733, EPI_ISL_755734, EPI_ISL_755735, EPI_ISL_755736			
see above	Toronto Invasive Bacterial Diseases Network	McMaster University	David Richardson, Allison McGeer, Patryk Aftanas, Hooman Derakhshani, Angel Li, Kuganya Nirmalarajah, Emily Panousis, Ahmed Draia, Jalees Nasir, Michael Surette, Samira Mubareka, Andrew G. McArthur
EPI_ISL_755737, EPI_ISL_755738, EPI_ISL_755739, EPI_ISL_755742, EPI_ISL_755743, EPI_ISL_755744, EPI_ISL_755745, EPI_ISL_755746, EPI_ISL_755747, EPI_ISL_755748, EPI_ISL_755749, EPI_ISL_755750, EPI_ISL_755751, EPI_ISL_755755, EPI_ISL_755756, EPI_ISL_755757, EPI_ISL_755758, EPI_ISL_755759, EPI_ISL_755761, EPI_ISL_755762, EPI_ISL_755763, EPI_ISL_755764, EPI_ISL_755765, EPI_ISL_755766, EPI_ISL_755767, EPI_ISL_755768, EPI_ISL_755769, EPI_ISL_755770, EPI_ISL_755771, EPI_ISL_755772, EPI_ISL_755773, EPI_ISL_755774, EPI_ISL_755775, EPI_ISL_755776, EPI_ISL_755777, EPI_ISL_755778, EPI_ISL_755779, EPI_ISL_755780, EPI_ISL_755781, EPI_ISL_755782, EPI_ISL_755783, EPI_ISL_755784, EPI_ISL_755785, EPI_ISL_755786, EPI_ISL_755787, EPI_ISL_755788, EPI_ISL_755789, EPI_ISL_755790, EPI_ISL_755791, EPI_ISL_755792, EPI_ISL_755802, EPI_ISL_755803, EPI_ISL_755804, EPI_ISL_755805, EPI_ISL_755806, EPI_ISL_755807, EPI_ISL_755808, EPI_ISL_755809, EPI_ISL_755810, EPI_ISL_755811, EPI_ISL_755812, EPI_ISL_755813, EPI_ISL_755883, EPI_ISL_755884, EPI_ISL_755885, EPI_ISL_755886, EPI_ISL_755887, EPI_ISL_755888, EPI_ISL_755889, EPI_ISL_755890, EPI_ISL_755891, EPI_ISL_755892, EPI_ISL_755893, EPI_ISL_755894, EPI_ISL_755895, EPI_ISL_755896, EPI_ISL_755897, EPI_ISL_755898, EPI_ISL_755899, EPI_ISL_755900, EPI_ISL_755901, EPI_ISL_755903, EPI_ISL_755905, EPI_ISL_755906, EPI_ISL_755907, EPI_ISL_755908, EPI_ISL_755909, EPI_ISL_755910, EPI_ISL_755913, EPI_ISL_755915, EPI_ISL_755916, EPI_ISL_755917, EPI_ISL_755920, EPI_ISL_755921, EPI_ISL_755922, EPI_ISL_755923, EPI_ISL_755924, EPI_ISL_755926, EPI_ISL_755927, EPI_ISL_755928, EPI_ISL_755929, EPI_ISL_755930, EPI_ISL_755931, EPI_ISL_755932, EPI_ISL_755933, EPI_ISL_755934, EPI_ISL_755935, EPI_ISL_755936, EPI_ISL_755937, EPI_ISL_755938, EPI_ISL_755939			
see above	Toronto Invasive Bacterial	McMaster University	Allison McGeer, Patryk Aftanas, Hooman Derakhshani, Angel Li, Kuganya Nirmalarajah, Emily Panousis, Ahmed Draia, Jalees Nasir, Michael Surette, Samira Mubareka, Andrew G. McArthur

	Diseases Network		
EPI_ISL_755940, EPI_ISL_755942	California Department of Public Health	Chiu Laboratory, University of California, San Francisco	Charles Chiu, Xianding (Wayne) Deng, Candace Wang, Jill Hacker, Debra Wadford
EPI_ISL_755943, EPI_ISL_755944, EPI_ISL_755945, EPI_ISL_755946, EPI_ISL_755947, EPI_ISL_755948, EPI_ISL_755949, EPI_ISL_755950, EPI_ISL_755951, EPI_ISL_755952, EPI_ISL_755953, EPI_ISL_755954, EPI_ISL_755955, EPI_ISL_755956, EPI_ISL_755957, EPI_ISL_755958, EPI_ISL_755959, EPI_ISL_755960, EPI_ISL_755961, EPI_ISL_755962, EPI_ISL_755963, EPI_ISL_755964, EPI_ISL_755965, EPI_ISL_755966, EPI_ISL_755967, EPI_ISL_755968, EPI_ISL_755969, EPI_ISL_755970, EPI_ISL_755971, EPI_ISL_755972, EPI_ISL_755973, EPI_ISL_755974, EPI_ISL_755975, EPI_ISL_755976, EPI_ISL_755977, EPI_ISL_755978, EPI_ISL_755979, EPI_ISL_755980, EPI_ISL_755981, EPI_ISL_755982, EPI_ISL_755983, EPI_ISL_755984, EPI_ISL_755985, EPI_ISL_755986, EPI_ISL_755987, EPI_ISL_755988, EPI_ISL_755989, EPI_ISL_755990, EPI_ISL_755991, EPI_ISL_755992, EPI_ISL_755993, EPI_ISL_755994, EPI_ISL_755995, EPI_ISL_755996, EPI_ISL_755997, EPI_ISL_755998, EPI_ISL_755999, EPI_ISL_756000, EPI_ISL_756001, EPI_ISL_756002, EPI_ISL_756003, EPI_ISL_756004, EPI_ISL_756005, EPI_ISL_756006, EPI_ISL_756007, EPI_ISL_756008, EPI_ISL_756009, EPI_ISL_756010, EPI_ISL_756011, EPI_ISL_756012, EPI_ISL_756013, EPI_ISL_756014, EPI_ISL_756015, EPI_ISL_756016, EPI_ISL_756017, EPI_ISL_756018, EPI_ISL_756019, EPI_ISL_756020, EPI_ISL_756021, EPI_ISL_756022, EPI_ISL_756023, EPI_ISL_756024, EPI_ISL_756025, EPI_ISL_756026, EPI_ISL_756027, EPI_ISL_756028, EPI_ISL_756029, EPI_ISL_756030, EPI_ISL_756031, EPI_ISL_756032, EPI_ISL_756033, EPI_ISL_756034, EPI_ISL_756035, EPI_ISL_756036, EPI_ISL_756037, EPI_ISL_756038, EPI_ISL_756039, EPI_ISL_756040, EPI_ISL_756041, EPI_ISL_756042, EPI_ISL_756043, EPI_ISL_756044, EPI_ISL_756045, EPI_ISL_756046, EPI_ISL_756047, EPI_ISL_756048, EPI_ISL_756049, EPI_ISL_756050, EPI_ISL_756051, EPI_ISL_756052, EPI_ISL_756053, EPI_ISL_756054, EPI_ISL_756055, EPI_ISL_756056, EPI_ISL_756057, EPI_ISL_756058, EPI_ISL_756059, EPI_ISL_756060, EPI_ISL_756061, EPI_ISL_756062, EPI_ISL_756063, EPI_ISL_756064, EPI_ISL_756065, EPI_ISL_756066, EPI_ISL_756067, EPI_ISL_756068, EPI_ISL_756069, EPI_ISL_756070, EPI_ISL_756071, EPI_ISL_756072, EPI_ISL_756073, EPI_ISL_756074, EPI_ISL_756075, EPI_ISL_756076, EPI_ISL_756077, EPI_ISL_756078, EPI_ISL_756079, EPI_ISL_756080, EPI_ISL_756081, EPI_ISL_756082, EPI_ISL_756083, EPI_ISL_756084, EPI_ISL_756085, EPI_ISL_756086, EPI_ISL_756087, EPI_ISL_756088, EPI_ISL_756089, EPI_ISL_756090, EPI_ISL_756091, EPI_ISL_756092, EPI_ISL_756093, EPI_ISL_756094, EPI_ISL_756095, EPI_ISL_756096, EPI_ISL_756097, EPI_ISL_756098, EPI_ISL_756099, EPI_ISL_756100, EPI_ISL_756101, EPI_ISL_756102, EPI_ISL_756103, EPI_ISL_756104, EPI_ISL_756105, EPI_ISL_756106, EPI_ISL_756107, EPI_ISL_756108, EPI_ISL_756109, EPI_ISL_756110, EPI_ISL_756111, EPI_ISL_756112, EPI_ISL_756113, EPI_ISL_756114, EPI_ISL_756115, EPI_ISL_756116, EPI_ISL_756117, EPI_ISL_756118, EPI_ISL_756119, EPI_ISL_756120, EPI_ISL_756121, EPI_ISL_756122, EPI_ISL_756123, EPI_ISL_756124, EPI_ISL_756125			
see above	Department of Virology and Immunology, University of Helsinki and Helsinki University Hospital, Huslab Finland	Department of Virology, Faculty of Medicine, University of Helsinki, Helsinki, Finland	Teemu Smura, Ravi Kant, Phuoc Truong, Hussein Alburkat, Hannimari Kallio-Kokko, Jenni Virtanen, Maija Suvanto, Sari Hannula, Harri Kangas, Pekka Ellonen, Olli Vapalahti
EPI_ISL_756126, EPI_ISL_756128, EPI_ISL_756129, EPI_ISL_756130, EPI_ISL_756131, EPI_ISL_756132, EPI_ISL_756133, EPI_ISL_756134	Wyoming Public Health Laboratory	Wyoming Public Health Laboratory	Noah Hull, Taylor Fearing, Channing Weber, Ashley Norberg, Bailey Bowcutt, and Wanda Manley
EPI_ISL_756136, EPI_ISL_756137, EPI_ISL_756138, EPI_ISL_756139, EPI_ISL_756140, EPI_ISL_756141, EPI_ISL_756142, EPI_ISL_756143, EPI_ISL_756144, EPI_ISL_756145, EPI_ISL_756146, EPI_ISL_756147, EPI_ISL_756148, EPI_ISL_756149, EPI_ISL_756150, EPI_ISL_756151, EPI_ISL_756152, EPI_ISL_756153, EPI_ISL_756154, EPI_ISL_756155, EPI_ISL_756156, EPI_ISL_756157, EPI_ISL_756158, EPI_ISL_756159, EPI_ISL_756160, EPI_ISL_756161, EPI_ISL_756163			
see above	Respiratory Virus Unit, National Infection Service, Public Health England	COVID-19 Genomics UK (COG-UK) Consortium	PHE Covid Sequencing Team
EPI_ISL_756164	Wyoming Public Health Laboratory	Wyoming Public Health Laboratory	Noah Hull, Taylor Fearing, Channing Weber, Ashley Norberg, Bailey Bowcutt, and Wanda Manley
EPI_ISL_756165, EPI_ISL_756166, EPI_ISL_756167, EPI_ISL_756168, EPI_ISL_756169, EPI_ISL_756170, EPI_ISL_756171, EPI_ISL_756172, EPI_ISL_756173, EPI_ISL_756174, EPI_ISL_756175, EPI_ISL_756176, EPI_ISL_756177, EPI_ISL_756178, EPI_ISL_756179, EPI_ISL_756180, EPI_ISL_756181, EPI_ISL_756182, EPI_ISL_756183			
see above	Department of Virology and Immunology, University of Helsinki and Helsinki University Hospital, Huslab Finland	Department of Virology, Faculty of Medicine, University of Helsinki, Helsinki, Finland	Teemu Smura, Ravi Kant, Phuoc Truong, Hussein Alburkat, Hannimari Kallio-Kokko, Jenni Virtanen, Maija Suvanto, Sari Hannula, Harri Kangas, Pekka Ellonen, Olli Vapalahti
EPI_ISL_756184, EPI_ISL_756185, EPI_ISL_756186, EPI_ISL_756187, EPI_ISL_756188, EPI_ISL_756189, EPI_ISL_756190	Wyoming Public Health Laboratory	Wyoming Public Health Laboratory	Noah Hull, Taylor Fearing, Channing Weber, Ashley Norberg, Bailey Bowcutt, and Wanda Manley
EPI_ISL_756191, EPI_ISL_756192, EPI_ISL_756193, EPI_ISL_756194, EPI_ISL_756195, EPI_ISL_756196, EPI_ISL_756197, EPI_ISL_756198, EPI_ISL_756199, EPI_ISL_756200, EPI_ISL_756201, EPI_ISL_756202, EPI_ISL_756203, EPI_ISL_756204, EPI_ISL_756205, EPI_ISL_756206, EPI_ISL_756207, EPI_ISL_756208, EPI_ISL_756209, EPI_ISL_756210, EPI_ISL_756211, EPI_ISL_756212, EPI_ISL_756213, EPI_ISL_756214, EPI_ISL_756215, EPI_ISL_756216, EPI_ISL_756217, EPI_ISL_756218, EPI_ISL_756219, EPI_ISL_756220, EPI_ISL_756221, EPI_ISL_756223, EPI_ISL_756224, EPI_ISL_756225, EPI_ISL_756226, EPI_ISL_756227, EPI_ISL_756228, EPI_ISL_756229, EPI_ISL_756230, EPI_ISL_756231, EPI_ISL_756232, EPI_ISL_756233, EPI_ISL_756234, EPI_ISL_756235, EPI_ISL_756236, EPI_ISL_756237, EPI_ISL_756238, EPI_ISL_756239, EPI_ISL_756240			
see above	UW Virology Lab	UW Virology Lab	Pavitra Roychoudhury, Hong Xie, Lasata Shrestha, Meei-Li Huang, Keith R Jerome, Alexander Greninger
EPI_ISL_756241, EPI_ISL_756242, EPI_ISL_756243, EPI_ISL_756244, EPI_ISL_756245, EPI_ISL_756246	Wyoming Public Health Laboratory	Wyoming Public Health Laboratory	Noah Hull, Taylor Fearing, Channing Weber, Ashley Norberg, Bailey Bowcutt, and Wanda Manley
EPI_ISL_756247, EPI_ISL_756248, EPI_ISL_756249, EPI_ISL_756250, EPI_ISL_756251, EPI_ISL_756252	Department of Virology and Immunology, University of Helsinki and Helsinki University Hospital, Huslab Finland	Department of Virology, Faculty of Medicine, University of Helsinki, Helsinki, Finland	Teemu Smura, Ravi Kant, Phuoc Truong, Hussein Alburkat, Hannimari Kallio-Kokko, Jenni Virtanen, Maija Suvanto, Sari Hannula, Harri Kangas, Pekka Ellonen, Olli Vapalahti
EPI_ISL_756253, EPI_ISL_756254, EPI_ISL_756255, EPI_ISL_756256, EPI_ISL_756257, EPI_ISL_756258, EPI_ISL_756259, EPI_ISL_756260, EPI_ISL_756261, EPI_ISL_756262, EPI_ISL_756267, EPI_ISL_756268, EPI_ISL_756269, EPI_ISL_756270			
see above	Wyoming Public Health Laboratory	Wyoming Public Health Laboratory	Noah Hull, Taylor Fearing, Channing Weber, Ashley Norberg, Bailey Bowcutt, and Wanda Manley
EPI_ISL_756271	Hospital General Universitario Gregorio Marañón	Hospital General Universitario Gregorio Marañón	Sergio Buenestado Serrano, Pedro Sola Campoy, Pilar Catalán, Patricia Muñoz, Darío García de Viedma.
EPI_ISL_756272	Hospital General Universitario La Princesa	Hospital General Universitario Gregorio Marañón	Sergio Buenestado Serrano, Pedro Sola Campoy, Pilar Catalán, Arturo fraile Torres, Andrés Von Wernitz, Carmen del Arco, Patricia Muñoz, Laura Cardeñoso, Darío García de Viedma.
EPI_ISL_756273	Hospital General Universitario Gregorio Marañón	Hospital General Universitario Gregorio Marañón	Sergio Buenestado Serrano, Pedro Sola Campoy, Pilar Catalán, Patricia Muñoz, Darío García de Viedma.
EPI_ISL_756275, EPI_ISL_756276,	State Laboratories Division, Hawaii State Department of	State Laboratories Division, Hawaii State	Pamela O'Brien, Sabrina Diemert, Drew Kuwazaki, Razvan Sultana, Edward Desmond

EPI_ISL_756277, EPI_ISL_756278, EPI_ISL_756279, EPI_ISL_756280, EPI_ISL_756281	Health	Department of Health	
EPI_ISL_756282, EPI_ISL_756283, EPI_ISL_756284, EPI_ISL_756285, EPI_ISL_756286, EPI_ISL_756287, EPI_ISL_756288, EPI_ISL_756289, EPI_ISL_756290, EPI_ISL_756291, EPI_ISL_756292			
see above	Department of Virology and Immunology, University of Helsinki and Helsinki University Hospital, Huslab Finland	Department of Virology, Faculty of Medicine, University of Helsinki, Helsinki, Finland	Teemu Smura, Ravi Kant, Phuoc Truong, Hussein Alburkat, Hannimari Kallio-Kokko, Jenni Virtanen, Maija Suvanto, Sari Hannula, Harri Kangas, Pekka Ellonen, Olli Vapalahti
EPI_ISL_756293, EPI_ISL_756294	Center for Biotechnology and Cell Therapy, São Rafael Hospital, Salvador, Brazil	Center for Biotechnology and Cell Therapy, São Rafael Hospital, Salvador, Brazil	Carolina Kymie Vasques Nonaka, Marília Miranda Franco, Tiago Gräf, Ana Verena Almeida Mendes, Renato Santana de Aguiar, Marta Giovanetti, Bruno Solano de Freitas Souza
EPI_ISL_756295, EPI_ISL_756296, EPI_ISL_756297, EPI_ISL_756298, EPI_ISL_756299, EPI_ISL_756300, EPI_ISL_756301, EPI_ISL_756302, EPI_ISL_756303, EPI_ISL_756304, EPI_ISL_756305			
see above	UW Virology Lab	UW Virology Lab	Pavitra Roychoudhury, Hong Xie, Lasata Shrestha, Meei-Li Huang, Keith R Jerome, Alexander Greninger
EPI_ISL_756312, EPI_ISL_756313, EPI_ISL_756314, EPI_ISL_756315, EPI_ISL_756316, EPI_ISL_756317, EPI_ISL_756318, EPI_ISL_756319, EPI_ISL_756320, EPI_ISL_756321, EPI_ISL_756322, EPI_ISL_756323, EPI_ISL_756324, EPI_ISL_756325, EPI_ISL_756326, EPI_ISL_756327, EPI_ISL_756330, EPI_ISL_756332, EPI_ISL_756333, EPI_ISL_756334, EPI_ISL_756336, EPI_ISL_756337, EPI_ISL_756338, EPI_ISL_756339, EPI_ISL_756340, EPI_ISL_756341, EPI_ISL_756342, EPI_ISL_756343, EPI_ISL_756344, EPI_ISL_756346, EPI_ISL_756347, EPI_ISL_756348, EPI_ISL_756349, EPI_ISL_756350, EPI_ISL_756351, EPI_ISL_756352, EPI_ISL_756353, EPI_ISL_756354, EPI_ISL_756355, EPI_ISL_756356			
see above	Innovative Genomics Institute, UC Berkeley	Innovative Genomics Institute, UC Berkeley	Stacia Wyman, Haridha Shivram, Phil Frankino, Liana Lareau, Shana McDevitt, Justin Choi
EPI_ISL_756362, EPI_ISL_756363	Trinidad Public Health Laboratory	Carrington Lab, Department of PreClinical Sciences, Faculty of Medical Sciences, The University of the West Indies	Nikita S. D. Sahadeo, Arianne Brown-Jordan, Sarah Hill, Vernie Ramkissoon, Roshan Parasram, Naresh Nandram, Avery Hinds, Jerome Foster, Stanley Giddings, Karla Georges, Marsha Ivey, Rahul Naidu, Risha Singh, SueMin Nathaniel, Rajini Haraksingh, Jaya Jayaraman, Chinna Chinnadurai, Adesh Ramsubhag, Nuno Faria, Oliver Pybus, Christopher Oura, Gabriel Escobar, Christine V. F. Carrington
EPI_ISL_756375	Siriraj Hospital, Mahidol University	National Institute of Health, Department of Medical Sciences, Ministry of Public Health, Thailand	Pilailuk Okada; Siripaporn Phuygun; Sittiporn Parnmen; Navin Horthongkham; Ratana Tacharoenmuang; Pakorn Piromtong; Natchaya Khiadsang; Thanutsapa Thanadachakul; Warawan Wongboot; Sirikanda wimol; Sunthareeya Waicharoen; Niracha Athipanyasilp; Archiraya Pattama; Chutikarn Chaimayo; Ruengpung Sutthent; Wannee Kantakamalakul.
EPI_ISL_756376	National Institute of Health Research and Development	National Institute of Health Research and Development	Ramadhany,R; Rukminiati,Y; Agustiningsih; Adam,K; Wibowo,HA; Pawestri,HA; Subangkit;Puspa,KD; Nugraha,AA; Ikawati,HD; Pangesti,KNA; Soekarso,T; Susilarini,NK; Hariastuti,NI; Nikmah,UA;Mursinah; Febriyani,A; Herman,R; Susanti,N;Herna; Febriyanti,T; Nurhadi,M; Kurniawati,J; Kipuw,NL; Muna,F; Indalao,IL; Rizki,A; Puspandari,N; Setiawaty,Vivi
EPI_ISL_756377	Lighthouse Lab in Milton Keynes	Wellcome Sanger Institute for the COVID-19 Genomics UK (COG-UK) Consortium	The Lighthouse Lab in Milton Keynes and Alex Alderton, Roberto Amato, Sonia Goncalves, Ewan Harrison, David K. Jackson, Ian Johnston, Dominic Kwiatkowski, Cordelia Langford, John Sillitoe on behalf of the Wellcome Sanger Institute COVID-19 Surveillance Team
EPI_ISL_756378	Lighthouse Lab in Alderley Park	Wellcome Sanger Institute for the COVID-19 Genomics UK (COG-UK) Consortium	Jacquelyn Wynn, Mairead Hyland, The Lighthouse Lab in Alderley Park and Alex Alderton, Roberto Amato, Sonia Goncalves, Ewan Harrison, David K. Jackson, Ian Johnston, Dominic Kwiatkowski, Cordelia Langford, John Sillitoe on behalf of the Wellcome Sanger Institute COVID-19 Surveillance Team
EPI_ISL_756379, EPI_ISL_756380	Lighthouse Lab in Cambridge	Wellcome Sanger Institute for the COVID-19 Genomics UK (COG-UK) Consortium	Rob Howes, The Lighthouse Lab in Cambridge and Alex Alderton, Roberto Amato, Sonia Goncalves, Ewan Harrison, David K. Jackson, Ian Johnston, Dominic Kwiatkowski, Cordelia Langford, John Sillitoe on behalf of the Wellcome Sanger Institute COVID-19 Surveillance Team
EPI_ISL_756381, EPI_ISL_756382, EPI_ISL_756383	Lighthouse Lab in Alderley Park	Wellcome Sanger Institute for the COVID-19 Genomics UK (COG-UK) Consortium	Jacquelyn Wynn, Mairead Hyland, The Lighthouse Lab in Alderley Park and Alex Alderton, Roberto Amato, Sonia Goncalves, Ewan Harrison, David K. Jackson, Ian Johnston, Dominic Kwiatkowski, Cordelia Langford, John Sillitoe on behalf of the Wellcome Sanger Institute COVID-19 Surveillance Team
EPI_ISL_756384	Lighthouse Lab in Cambridge	Wellcome Sanger Institute for the COVID-19 Genomics UK (COG-UK) Consortium	Rob Howes, The Lighthouse Lab in Cambridge and Alex Alderton, Roberto Amato, Sonia Goncalves, Ewan Harrison, David K. Jackson, Ian Johnston, Dominic Kwiatkowski, Cordelia Langford, John Sillitoe on behalf of the Wellcome Sanger Institute COVID-19 Surveillance Team
EPI_ISL_756385	Lighthouse Lab in Alderley Park	Wellcome Sanger Institute for the COVID-19 Genomics UK (COG-UK) Consortium	Jacquelyn Wynn, Mairead Hyland, The Lighthouse Lab in Alderley Park and Alex Alderton, Roberto Amato, Sonia Goncalves, Ewan Harrison, David K. Jackson, Ian Johnston, Dominic Kwiatkowski, Cordelia Langford, John Sillitoe on behalf of the Wellcome Sanger Institute COVID-19 Surveillance Team
EPI_ISL_756386	Lighthouse Lab in Cambridge	Wellcome Sanger Institute for the COVID-19 Genomics UK (COG-UK) Consortium	Rob Howes, The Lighthouse Lab in Cambridge and Alex Alderton, Roberto Amato, Sonia Goncalves, Ewan Harrison, David K. Jackson, Ian Johnston, Dominic Kwiatkowski, Cordelia Langford, John Sillitoe on behalf of the Wellcome Sanger Institute COVID-19 Surveillance Team
EPI_ISL_756387, EPI_ISL_756388	Lighthouse Lab in Alderley Park	Wellcome Sanger Institute for the COVID-19 Genomics UK (COG-UK) Consortium	Jacquelyn Wynn, Mairead Hyland, The Lighthouse Lab in Alderley Park and Alex Alderton, Roberto Amato, Sonia Goncalves, Ewan Harrison, David K. Jackson, Ian Johnston, Dominic Kwiatkowski, Cordelia Langford, John Sillitoe on behalf of the Wellcome Sanger Institute COVID-19 Surveillance Team
EPI_ISL_756389	Lighthouse Lab in Cambridge	Wellcome Sanger Institute for the COVID-19 Genomics UK (COG-UK) Consortium	Rob Howes, The Lighthouse Lab in Cambridge and Alex Alderton, Roberto Amato, Sonia Goncalves, Ewan Harrison, David K. Jackson, Ian Johnston, Dominic Kwiatkowski, Cordelia Langford, John Sillitoe on behalf of the Wellcome Sanger Institute COVID-19 Surveillance Team
EPI_ISL_756390, EPI_ISL_756391	Lighthouse Lab in Alderley Park	Wellcome Sanger Institute for the COVID-19 Genomics UK (COG-UK) Consortium	Jacquelyn Wynn, Mairead Hyland, The Lighthouse Lab in Alderley Park and Alex Alderton, Roberto Amato, Sonia Goncalves, Ewan Harrison, David K. Jackson, Ian Johnston, Dominic Kwiatkowski, Cordelia Langford, John Sillitoe on behalf of the Wellcome Sanger Institute COVID-19 Surveillance Team
EPI_ISL_756392, EPI_ISL_756393	Lighthouse Lab in Cambridge	Wellcome Sanger Institute for the	Rob Howes, The Lighthouse Lab in Cambridge and Alex Alderton, Roberto Amato, Sonia Goncalves, Ewan Harrison, David K. Jackson, Ian Johnston, Dominic Kwiatkowski, Cordelia Langford, John Sillitoe on behalf of the Wellcome Sanger Institute COVID-19 Surveillance Team

		COVID-19 Genomics UK (COG-UK) Consortium	
EPI_ISL_756394	Lighthouse Lab in Alderley Park	Wellcome Sanger Institute for the COVID-19 Genomics UK (COG-UK) Consortium	Jacquelyn Wynn, Mairead Hyland, The Lighthouse Lab in Alderley Park and Alex Alderton, Roberto Amato, Sonia Goncalves, Ewan Harrison, David K. Jackson, Ian Johnston, Dominic Kwiatkowski, Cordelia Langford, John Sillitoe on behalf of the Wellcome Sanger Institute COVID-19 Surveillance Team
EPI_ISL_756395	Lighthouse Lab in Cambridge	Wellcome Sanger Institute for the COVID-19 Genomics UK (COG-UK) Consortium	Rob Howes, The Lighthouse Lab in Cambridge and Alex Alderton, Roberto Amato, Sonia Goncalves, Ewan Harrison, David K. Jackson, Ian Johnston, Dominic Kwiatkowski, Cordelia Langford, John Sillitoe on behalf of the Wellcome Sanger Institute COVID-19 Surveillance Team
EPI_ISL_756396, EPI_ISL_756397, EPI_ISL_756398, EPI_ISL_756399, EPI_ISL_756400	Lighthouse Lab in Alderley Park	Wellcome Sanger Institute for the COVID-19 Genomics UK (COG-UK) Consortium	Jacquelyn Wynn, Mairead Hyland, The Lighthouse Lab in Alderley Park and Alex Alderton, Roberto Amato, Sonia Goncalves, Ewan Harrison, David K. Jackson, Ian Johnston, Dominic Kwiatkowski, Cordelia Langford, John Sillitoe on behalf of the Wellcome Sanger Institute COVID-19 Surveillance Team
EPI_ISL_756401	National Institute of Health Research and Development	National Institute of Health Research and Development	Adam,K; Wibowo,HA; Ramadhany,R; Rukminiati,Y; Agustiningsih; Pawestri,HA; Subangkit; Puspa,KD; Nugraha,AA; Ikawati,HD; Pangesti,KNA; Soekarso,T; Susilarini,NK; Hariastuti,NI; Nikmah,UA; Mursinah; Febriyani,A; Herman,R; Susanti,N; Herna; Febriyanti,T; Nurhadi,M; Kurniawati,J; Kipuw,NL; Muna,F; Indalao,IL; Rizki,A; Puspandari,N; Setiawaty,Vivi
EPI_ISL_756402, EPI_ISL_756404, EPI_ISL_756405, EPI_ISL_756406, EPI_ISL_756407, EPI_ISL_756408	Lighthouse Lab in Alderley Park	Wellcome Sanger Institute for the COVID-19 Genomics UK (COG-UK) Consortium	Jacquelyn Wynn, Mairead Hyland, The Lighthouse Lab in Alderley Park and Alex Alderton, Roberto Amato, Sonia Goncalves, Ewan Harrison, David K. Jackson, Ian Johnston, Dominic Kwiatkowski, Cordelia Langford, John Sillitoe on behalf of the Wellcome Sanger Institute COVID-19 Surveillance Team
EPI_ISL_756409	Lighthouse Lab in Cambridge	Wellcome Sanger Institute for the COVID-19 Genomics UK (COG-UK) Consortium	Rob Howes, The Lighthouse Lab in Cambridge and Alex Alderton, Roberto Amato, Sonia Goncalves, Ewan Harrison, David K. Jackson, Ian Johnston, Dominic Kwiatkowski, Cordelia Langford, John Sillitoe on behalf of the Wellcome Sanger Institute COVID-19 Surveillance Team
EPI_ISL_756410, EPI_ISL_756411, EPI_ISL_756412	Lighthouse Lab in Alderley Park	Wellcome Sanger Institute for the COVID-19 Genomics UK (COG-UK) Consortium	Jacquelyn Wynn, Mairead Hyland, The Lighthouse Lab in Alderley Park and Alex Alderton, Roberto Amato, Sonia Goncalves, Ewan Harrison, David K. Jackson, Ian Johnston, Dominic Kwiatkowski, Cordelia Langford, John Sillitoe on behalf of the Wellcome Sanger Institute COVID-19 Surveillance Team
EPI_ISL_756413	Lighthouse Lab in Cambridge	Wellcome Sanger Institute for the COVID-19 Genomics UK (COG-UK) Consortium	Rob Howes, The Lighthouse Lab in Cambridge and Alex Alderton, Roberto Amato, Sonia Goncalves, Ewan Harrison, David K. Jackson, Ian Johnston, Dominic Kwiatkowski, Cordelia Langford, John Sillitoe on behalf of the Wellcome Sanger Institute COVID-19 Surveillance Team
EPI_ISL_756414	Lighthouse Lab in Alderley Park	Wellcome Sanger Institute for the COVID-19 Genomics UK (COG-UK) Consortium	Jacquelyn Wynn, Mairead Hyland, The Lighthouse Lab in Alderley Park and Alex Alderton, Roberto Amato, Sonia Goncalves, Ewan Harrison, David K. Jackson, Ian Johnston, Dominic Kwiatkowski, Cordelia Langford, John Sillitoe on behalf of the Wellcome Sanger Institute COVID-19 Surveillance Team
EPI_ISL_756415, EPI_ISL_756416, EPI_ISL_756417, EPI_ISL_756418, EPI_ISL_756420	Lighthouse Lab in Cambridge	Wellcome Sanger Institute for the COVID-19 Genomics UK (COG-UK) Consortium	Rob Howes, The Lighthouse Lab in Cambridge and Alex Alderton, Roberto Amato, Sonia Goncalves, Ewan Harrison, David K. Jackson, Ian Johnston, Dominic Kwiatkowski, Cordelia Langford, John Sillitoe on behalf of the Wellcome Sanger Institute COVID-19 Surveillance Team
EPI_ISL_756421	Lighthouse Lab in Alderley Park	Wellcome Sanger Institute for the COVID-19 Genomics UK (COG-UK) Consortium	Jacquelyn Wynn, Mairead Hyland, The Lighthouse Lab in Alderley Park and Alex Alderton, Roberto Amato, Sonia Goncalves, Ewan Harrison, David K. Jackson, Ian Johnston, Dominic Kwiatkowski, Cordelia Langford, John Sillitoe on behalf of the Wellcome Sanger Institute COVID-19 Surveillance Team
EPI_ISL_756422	Lighthouse Lab in Cambridge	Wellcome Sanger Institute for the COVID-19 Genomics UK (COG-UK) Consortium	Rob Howes, The Lighthouse Lab in Cambridge and Alex Alderton, Roberto Amato, Sonia Goncalves, Ewan Harrison, David K. Jackson, Ian Johnston, Dominic Kwiatkowski, Cordelia Langford, John Sillitoe on behalf of the Wellcome Sanger Institute COVID-19 Surveillance Team
EPI_ISL_756423	Lighthouse Lab in Alderley Park	Wellcome Sanger Institute for the COVID-19 Genomics UK (COG-UK) Consortium	Jacquelyn Wynn, Mairead Hyland, The Lighthouse Lab in Alderley Park and Alex Alderton, Roberto Amato, Sonia Goncalves, Ewan Harrison, David K. Jackson, Ian Johnston, Dominic Kwiatkowski, Cordelia Langford, John Sillitoe on behalf of the Wellcome Sanger Institute COVID-19 Surveillance Team
EPI_ISL_756424	Lighthouse Lab in Cambridge	Wellcome Sanger Institute for the COVID-19 Genomics UK (COG-UK) Consortium	Rob Howes, The Lighthouse Lab in Cambridge and Alex Alderton, Roberto Amato, Sonia Goncalves, Ewan Harrison, David K. Jackson, Ian Johnston, Dominic Kwiatkowski, Cordelia Langford, John Sillitoe on behalf of the Wellcome Sanger Institute COVID-19 Surveillance Team
EPI_ISL_756425	Lighthouse Lab in Alderley Park	Wellcome Sanger Institute for the COVID-19 Genomics UK (COG-UK) Consortium	Jacquelyn Wynn, Mairead Hyland, The Lighthouse Lab in Alderley Park and Alex Alderton, Roberto Amato, Sonia Goncalves, Ewan Harrison, David K. Jackson, Ian Johnston, Dominic Kwiatkowski, Cordelia Langford, John Sillitoe on behalf of the Wellcome Sanger Institute COVID-19 Surveillance Team
EPI_ISL_756426	Lighthouse Lab in Cambridge	Wellcome Sanger Institute for the COVID-19 Genomics UK (COG-UK) Consortium	Rob Howes, The Lighthouse Lab in Cambridge and Alex Alderton, Roberto Amato, Sonia Goncalves, Ewan Harrison, David K. Jackson, Ian Johnston, Dominic Kwiatkowski, Cordelia Langford, John Sillitoe on behalf of the Wellcome Sanger Institute COVID-19 Surveillance Team
EPI_ISL_756427, EPI_ISL_756428	Lighthouse Lab in Alderley Park	Wellcome Sanger Institute for the COVID-19 Genomics UK (COG-UK) Consortium	Jacquelyn Wynn, Mairead Hyland, The Lighthouse Lab in Alderley Park and Alex Alderton, Roberto Amato, Sonia Goncalves, Ewan Harrison, David K. Jackson, Ian Johnston, Dominic Kwiatkowski, Cordelia Langford, John Sillitoe on behalf of the Wellcome Sanger Institute COVID-19 Surveillance Team
EPI_ISL_756429	Lighthouse Lab in Cambridge	Wellcome Sanger Institute for the COVID-19 Genomics UK (COG-UK) Consortium	Rob Howes, The Lighthouse Lab in Cambridge and Alex Alderton, Roberto Amato, Sonia Goncalves, Ewan Harrison, David K. Jackson, Ian Johnston, Dominic Kwiatkowski, Cordelia Langford, John Sillitoe on behalf of the Wellcome Sanger Institute COVID-19 Surveillance Team
EPI_ISL_756430	Lighthouse Lab in Alderley Park	Wellcome Sanger	Jacquelyn Wynn, Mairead Hyland, The Lighthouse Lab in Alderley Park and Alex Alderton, Roberto Amato, Sonia Goncalves, Ewan Harrison, David K. Jackson, Ian Johnston, Dominic Kwiatkowski, Cordelia Langford, John Sillitoe on behalf of the

		Institute for the COVID-19 Genomics UK (COG-UK) Consortium	Wellcome Sanger Institute COVID-19 Surveillance Team
EPI_ISL_756431, EPI_ISL_756432	Lighthouse Lab in Cambridge	Wellcome Sanger Institute for the COVID-19 Genomics UK (COG-UK) Consortium	Rob Howes, The Lighthouse Lab in Cambridge and Alex Alderton, Roberto Amato, Sonia Goncalves, Ewan Harrison, David K. Jackson, Ian Johnston, Dominic Kwiatkowski, Cordelia Langford, John Sillitoe on behalf of the Wellcome Sanger Institute COVID-19 Surveillance Team
EPI_ISL_756433	Lighthouse Lab in Alderley Park	Wellcome Sanger Institute for the COVID-19 Genomics UK (COG-UK) Consortium	Jacquelyn Wynn, Mairead Hyland, The Lighthouse Lab in Alderley Park and Alex Alderton, Roberto Amato, Sonia Goncalves, Ewan Harrison, David K. Jackson, Ian Johnston, Dominic Kwiatkowski, Cordelia Langford, John Sillitoe on behalf of the Wellcome Sanger Institute COVID-19 Surveillance Team
EPI_ISL_756434	Lighthouse Lab in Cambridge	Wellcome Sanger Institute for the COVID-19 Genomics UK (COG-UK) Consortium	Rob Howes, The Lighthouse Lab in Cambridge and Alex Alderton, Roberto Amato, Sonia Goncalves, Ewan Harrison, David K. Jackson, Ian Johnston, Dominic Kwiatkowski, Cordelia Langford, John Sillitoe on behalf of the Wellcome Sanger Institute COVID-19 Surveillance Team
EPI_ISL_756435, EPI_ISL_756436, EPI_ISL_756437	Lighthouse Lab in Alderley Park	Wellcome Sanger Institute for the COVID-19 Genomics UK (COG-UK) Consortium	Jacquelyn Wynn, Mairead Hyland, The Lighthouse Lab in Alderley Park and Alex Alderton, Roberto Amato, Sonia Goncalves, Ewan Harrison, David K. Jackson, Ian Johnston, Dominic Kwiatkowski, Cordelia Langford, John Sillitoe on behalf of the Wellcome Sanger Institute COVID-19 Surveillance Team
EPI_ISL_756438	Lighthouse Lab in Cambridge	Wellcome Sanger Institute for the COVID-19 Genomics UK (COG-UK) Consortium	Rob Howes, The Lighthouse Lab in Cambridge and Alex Alderton, Roberto Amato, Sonia Goncalves, Ewan Harrison, David K. Jackson, Ian Johnston, Dominic Kwiatkowski, Cordelia Langford, John Sillitoe on behalf of the Wellcome Sanger Institute COVID-19 Surveillance Team
EPI_ISL_756439, EPI_ISL_756440, EPI_ISL_756441	Lighthouse Lab in Alderley Park	Wellcome Sanger Institute for the COVID-19 Genomics UK (COG-UK) Consortium	Jacquelyn Wynn, Mairead Hyland, The Lighthouse Lab in Alderley Park and Alex Alderton, Roberto Amato, Sonia Goncalves, Ewan Harrison, David K. Jackson, Ian Johnston, Dominic Kwiatkowski, Cordelia Langford, John Sillitoe on behalf of the Wellcome Sanger Institute COVID-19 Surveillance Team
EPI_ISL_756442	Lighthouse Lab in Cambridge	Wellcome Sanger Institute for the COVID-19 Genomics UK (COG-UK) Consortium	Rob Howes, The Lighthouse Lab in Cambridge and Alex Alderton, Roberto Amato, Sonia Goncalves, Ewan Harrison, David K. Jackson, Ian Johnston, Dominic Kwiatkowski, Cordelia Langford, John Sillitoe on behalf of the Wellcome Sanger Institute COVID-19 Surveillance Team
EPI_ISL_756443	Lighthouse Lab in Alderley Park	Wellcome Sanger Institute for the COVID-19 Genomics UK (COG-UK) Consortium	Jacquelyn Wynn, Mairead Hyland, The Lighthouse Lab in Alderley Park and Alex Alderton, Roberto Amato, Sonia Goncalves, Ewan Harrison, David K. Jackson, Ian Johnston, Dominic Kwiatkowski, Cordelia Langford, John Sillitoe on behalf of the Wellcome Sanger Institute COVID-19 Surveillance Team
EPI_ISL_756444	Lighthouse Lab in Cambridge	Wellcome Sanger Institute for the COVID-19 Genomics UK (COG-UK) Consortium	Rob Howes, The Lighthouse Lab in Cambridge and Alex Alderton, Roberto Amato, Sonia Goncalves, Ewan Harrison, David K. Jackson, Ian Johnston, Dominic Kwiatkowski, Cordelia Langford, John Sillitoe on behalf of the Wellcome Sanger Institute COVID-19 Surveillance Team
EPI_ISL_756445	Lighthouse Lab in Alderley Park	Wellcome Sanger Institute for the COVID-19 Genomics UK (COG-UK) Consortium	Jacquelyn Wynn, Mairead Hyland, The Lighthouse Lab in Alderley Park and Alex Alderton, Roberto Amato, Sonia Goncalves, Ewan Harrison, David K. Jackson, Ian Johnston, Dominic Kwiatkowski, Cordelia Langford, John Sillitoe on behalf of the Wellcome Sanger Institute COVID-19 Surveillance Team
EPI_ISL_756446	Lighthouse Lab in Cambridge	Wellcome Sanger Institute for the COVID-19 Genomics UK (COG-UK) Consortium	Rob Howes, The Lighthouse Lab in Cambridge and Alex Alderton, Roberto Amato, Sonia Goncalves, Ewan Harrison, David K. Jackson, Ian Johnston, Dominic Kwiatkowski, Cordelia Langford, John Sillitoe on behalf of the Wellcome Sanger Institute COVID-19 Surveillance Team
EPI_ISL_756447	Lighthouse Lab in Alderley Park	Wellcome Sanger Institute for the COVID-19 Genomics UK (COG-UK) Consortium	Jacquelyn Wynn, Mairead Hyland, The Lighthouse Lab in Alderley Park and Alex Alderton, Roberto Amato, Sonia Goncalves, Ewan Harrison, David K. Jackson, Ian Johnston, Dominic Kwiatkowski, Cordelia Langford, John Sillitoe on behalf of the Wellcome Sanger Institute COVID-19 Surveillance Team
EPI_ISL_756448	Lighthouse Lab in Cambridge	Wellcome Sanger Institute for the COVID-19 Genomics UK (COG-UK) Consortium	Rob Howes, The Lighthouse Lab in Cambridge and Alex Alderton, Roberto Amato, Sonia Goncalves, Ewan Harrison, David K. Jackson, Ian Johnston, Dominic Kwiatkowski, Cordelia Langford, John Sillitoe on behalf of the Wellcome Sanger Institute COVID-19 Surveillance Team
EPI_ISL_756449, EPI_ISL_756450, EPI_ISL_756451, EPI_ISL_756452	Lighthouse Lab in Alderley Park	Wellcome Sanger Institute for the COVID-19 Genomics UK (COG-UK) Consortium	Jacquelyn Wynn, Mairead Hyland, The Lighthouse Lab in Alderley Park and Alex Alderton, Roberto Amato, Sonia Goncalves, Ewan Harrison, David K. Jackson, Ian Johnston, Dominic Kwiatkowski, Cordelia Langford, John Sillitoe on behalf of the Wellcome Sanger Institute COVID-19 Surveillance Team
EPI_ISL_756453, EPI_ISL_756454	Lighthouse Lab in Cambridge	Wellcome Sanger Institute for the COVID-19 Genomics UK (COG-UK) Consortium	Rob Howes, The Lighthouse Lab in Cambridge and Alex Alderton, Roberto Amato, Sonia Goncalves, Ewan Harrison, David K. Jackson, Ian Johnston, Dominic Kwiatkowski, Cordelia Langford, John Sillitoe on behalf of the Wellcome Sanger Institute COVID-19 Surveillance Team
EPI_ISL_756455	Lighthouse Lab in Alderley Park	Wellcome Sanger Institute for the COVID-19 Genomics UK (COG-UK) Consortium	Jacquelyn Wynn, Mairead Hyland, The Lighthouse Lab in Alderley Park and Alex Alderton, Roberto Amato, Sonia Goncalves, Ewan Harrison, David K. Jackson, Ian Johnston, Dominic Kwiatkowski, Cordelia Langford, John Sillitoe on behalf of the Wellcome Sanger Institute COVID-19 Surveillance Team
EPI_ISL_756456	Lighthouse Lab in Cambridge	Wellcome Sanger Institute for the COVID-19 Genomics UK (COG-UK) Consortium	Rob Howes, The Lighthouse Lab in Cambridge and Alex Alderton, Roberto Amato, Sonia Goncalves, Ewan Harrison, David K. Jackson, Ian Johnston, Dominic Kwiatkowski, Cordelia Langford, John Sillitoe on behalf of the Wellcome Sanger Institute COVID-19 Surveillance Team
EPI_ISL_756457, EPI_ISL_756458, EPI_ISL_756459	Lighthouse Lab in Alderley Park	Wellcome Sanger Institute for the COVID-19 Genomics UK (COG-UK) Consortium	Jacquelyn Wynn, Mairead Hyland, The Lighthouse Lab in Alderley Park and Alex Alderton, Roberto Amato, Sonia Goncalves, Ewan Harrison, David K. Jackson, Ian Johnston, Dominic Kwiatkowski, Cordelia Langford, John Sillitoe on behalf of the Wellcome Sanger Institute COVID-19 Surveillance Team
EPI_ISL_756460	Lighthouse Lab in Cambridge	Wellcome Sanger Institute for the COVID-19 Genomics UK	Rob Howes, The Lighthouse Lab in Cambridge and Alex Alderton, Roberto Amato, Sonia Goncalves, Ewan Harrison, David K. Jackson, Ian Johnston, Dominic Kwiatkowski, Cordelia Langford, John Sillitoe on behalf of the Wellcome Sanger Institute COVID-19 Surveillance Team

		(COG-UK) Consortium	
EPI_ISL_756461, EPI_ISL_756462, EPI_ISL_756463, EPI_ISL_756464, EPI_ISL_756465, EPI_ISL_756466	Lighthouse Lab in Alderley Park	Wellcome Sanger Institute for the COVID-19 Genomics UK (COG-UK) Consortium	Jacquelyn Wynn, Mairead Hyland, The Lighthouse Lab in Alderley Park and Alex Alderton, Roberto Amato, Sonia Goncalves, Ewan Harrison, David K. Jackson, Ian Johnston, Dominic Kwiatkowski, Cordelia Langford, John Sillitoe on behalf of the Wellcome Sanger Institute COVID-19 Surveillance Team
EPI_ISL_756467	Lighthouse Lab in Cambridge	Wellcome Sanger Institute for the COVID-19 Genomics UK (COG-UK) Consortium	Rob Howes, The Lighthouse Lab in Cambridge and Alex Alderton, Roberto Amato, Sonia Goncalves, Ewan Harrison, David K. Jackson, Ian Johnston, Dominic Kwiatkowski, Cordelia Langford, John Sillitoe on behalf of the Wellcome Sanger Institute COVID-19 Surveillance Team
EPI_ISL_756468	Lighthouse Lab in Alderley Park	Wellcome Sanger Institute for the COVID-19 Genomics UK (COG-UK) Consortium	Jacquelyn Wynn, Mairead Hyland, The Lighthouse Lab in Alderley Park and Alex Alderton, Roberto Amato, Sonia Goncalves, Ewan Harrison, David K. Jackson, Ian Johnston, Dominic Kwiatkowski, Cordelia Langford, John Sillitoe on behalf of the Wellcome Sanger Institute COVID-19 Surveillance Team
EPI_ISL_756469	Lighthouse Lab in Cambridge	Wellcome Sanger Institute for the COVID-19 Genomics UK (COG-UK) Consortium	Rob Howes, The Lighthouse Lab in Cambridge and Alex Alderton, Roberto Amato, Sonia Goncalves, Ewan Harrison, David K. Jackson, Ian Johnston, Dominic Kwiatkowski, Cordelia Langford, John Sillitoe on behalf of the Wellcome Sanger Institute COVID-19 Surveillance Team
EPI_ISL_756470, EPI_ISL_756471, EPI_ISL_756472, EPI_ISL_756473	Lighthouse Lab in Alderley Park	Wellcome Sanger Institute for the COVID-19 Genomics UK (COG-UK) Consortium	Jacquelyn Wynn, Mairead Hyland, The Lighthouse Lab in Alderley Park and Alex Alderton, Roberto Amato, Sonia Goncalves, Ewan Harrison, David K. Jackson, Ian Johnston, Dominic Kwiatkowski, Cordelia Langford, John Sillitoe on behalf of the Wellcome Sanger Institute COVID-19 Surveillance Team
EPI_ISL_756474, EPI_ISL_756475	Lighthouse Lab in Cambridge	Wellcome Sanger Institute for the COVID-19 Genomics UK (COG-UK) Consortium	Rob Howes, The Lighthouse Lab in Cambridge and Alex Alderton, Roberto Amato, Sonia Goncalves, Ewan Harrison, David K. Jackson, Ian Johnston, Dominic Kwiatkowski, Cordelia Langford, John Sillitoe on behalf of the Wellcome Sanger Institute COVID-19 Surveillance Team
EPI_ISL_756476, EPI_ISL_756477	Lighthouse Lab in Alderley Park	Wellcome Sanger Institute for the COVID-19 Genomics UK (COG-UK) Consortium	Jacquelyn Wynn, Mairead Hyland, The Lighthouse Lab in Alderley Park and Alex Alderton, Roberto Amato, Sonia Goncalves, Ewan Harrison, David K. Jackson, Ian Johnston, Dominic Kwiatkowski, Cordelia Langford, John Sillitoe on behalf of the Wellcome Sanger Institute COVID-19 Surveillance Team
EPI_ISL_756478	Lighthouse Lab in Cambridge	Wellcome Sanger Institute for the COVID-19 Genomics UK (COG-UK) Consortium	Rob Howes, The Lighthouse Lab in Cambridge and Alex Alderton, Roberto Amato, Sonia Goncalves, Ewan Harrison, David K. Jackson, Ian Johnston, Dominic Kwiatkowski, Cordelia Langford, John Sillitoe on behalf of the Wellcome Sanger Institute COVID-19 Surveillance Team
EPI_ISL_756479	Lighthouse Lab in Alderley Park	Wellcome Sanger Institute for the COVID-19 Genomics UK (COG-UK) Consortium	Jacquelyn Wynn, Mairead Hyland, The Lighthouse Lab in Alderley Park and Alex Alderton, Roberto Amato, Sonia Goncalves, Ewan Harrison, David K. Jackson, Ian Johnston, Dominic Kwiatkowski, Cordelia Langford, John Sillitoe on behalf of the Wellcome Sanger Institute COVID-19 Surveillance Team
EPI_ISL_756480, EPI_ISL_756481	Lighthouse Lab in Cambridge	Wellcome Sanger Institute for the COVID-19 Genomics UK (COG-UK) Consortium	Rob Howes, The Lighthouse Lab in Cambridge and Alex Alderton, Roberto Amato, Sonia Goncalves, Ewan Harrison, David K. Jackson, Ian Johnston, Dominic Kwiatkowski, Cordelia Langford, John Sillitoe on behalf of the Wellcome Sanger Institute COVID-19 Surveillance Team
EPI_ISL_756482, EPI_ISL_756483	Lighthouse Lab in Alderley Park	Wellcome Sanger Institute for the COVID-19 Genomics UK (COG-UK) Consortium	Jacquelyn Wynn, Mairead Hyland, The Lighthouse Lab in Alderley Park and Alex Alderton, Roberto Amato, Sonia Goncalves, Ewan Harrison, David K. Jackson, Ian Johnston, Dominic Kwiatkowski, Cordelia Langford, John Sillitoe on behalf of the Wellcome Sanger Institute COVID-19 Surveillance Team
EPI_ISL_756484, EPI_ISL_756485, EPI_ISL_756486, EPI_ISL_756487	Lighthouse Lab in Cambridge	Wellcome Sanger Institute for the COVID-19 Genomics UK (COG-UK) Consortium	Rob Howes, The Lighthouse Lab in Cambridge and Alex Alderton, Roberto Amato, Sonia Goncalves, Ewan Harrison, David K. Jackson, Ian Johnston, Dominic Kwiatkowski, Cordelia Langford, John Sillitoe on behalf of the Wellcome Sanger Institute COVID-19 Surveillance Team
EPI_ISL_756488, EPI_ISL_756489	Lighthouse Lab in Alderley Park	Wellcome Sanger Institute for the COVID-19 Genomics UK (COG-UK) Consortium	Jacquelyn Wynn, Mairead Hyland, The Lighthouse Lab in Alderley Park and Alex Alderton, Roberto Amato, Sonia Goncalves, Ewan Harrison, David K. Jackson, Ian Johnston, Dominic Kwiatkowski, Cordelia Langford, John Sillitoe on behalf of the Wellcome Sanger Institute COVID-19 Surveillance Team
EPI_ISL_756490, EPI_ISL_756491, EPI_ISL_756492	Lighthouse Lab in Cambridge	Wellcome Sanger Institute for the COVID-19 Genomics UK (COG-UK) Consortium	Rob Howes, The Lighthouse Lab in Cambridge and Alex Alderton, Roberto Amato, Sonia Goncalves, Ewan Harrison, David K. Jackson, Ian Johnston, Dominic Kwiatkowski, Cordelia Langford, John Sillitoe on behalf of the Wellcome Sanger Institute COVID-19 Surveillance Team
EPI_ISL_756493, EPI_ISL_756494, EPI_ISL_756495	Lighthouse Lab in Alderley Park	Wellcome Sanger Institute for the COVID-19 Genomics UK (COG-UK) Consortium	Jacquelyn Wynn, Mairead Hyland, The Lighthouse Lab in Alderley Park and Alex Alderton, Roberto Amato, Sonia Goncalves, Ewan Harrison, David K. Jackson, Ian Johnston, Dominic Kwiatkowski, Cordelia Langford, John Sillitoe on behalf of the Wellcome Sanger Institute COVID-19 Surveillance Team
EPI_ISL_756496, EPI_ISL_756497, EPI_ISL_756498, EPI_ISL_756499, EPI_ISL_756500, EPI_ISL_756501, EPI_ISL_756502	Lighthouse Lab in Cambridge	Wellcome Sanger Institute for the COVID-19 Genomics UK (COG-UK) Consortium	Rob Howes, The Lighthouse Lab in Cambridge and Alex Alderton, Roberto Amato, Sonia Goncalves, Ewan Harrison, David K. Jackson, Ian Johnston, Dominic Kwiatkowski, Cordelia Langford, John Sillitoe on behalf of the Wellcome Sanger Institute COVID-19 Surveillance Team
EPI_ISL_756503, EPI_ISL_756504	Lighthouse Lab in Alderley Park	Wellcome Sanger Institute for the COVID-19 Genomics UK (COG-UK) Consortium	Jacquelyn Wynn, Mairead Hyland, The Lighthouse Lab in Alderley Park and Alex Alderton, Roberto Amato, Sonia Goncalves, Ewan Harrison, David K. Jackson, Ian Johnston, Dominic Kwiatkowski, Cordelia Langford, John Sillitoe on behalf of the Wellcome Sanger Institute COVID-19 Surveillance Team
EPI_ISL_756505, EPI_ISL_756506, EPI_ISL_756507, EPI_ISL_756508	Lighthouse Lab in Cambridge	Wellcome Sanger Institute for the COVID-19 Genomics UK (COG-UK) Consortium	Rob Howes, The Lighthouse Lab in Cambridge and Alex Alderton, Roberto Amato, Sonia Goncalves, Ewan Harrison, David K. Jackson, Ian Johnston, Dominic Kwiatkowski, Cordelia Langford, John Sillitoe on behalf of the Wellcome Sanger Institute COVID-19 Surveillance Team

EPI_ISL_756509, EPI_ISL_756510	Lighthouse Lab in Alderley Park	Wellcome Sanger Institute for the COVID-19 Genomics UK (COG-UK) Consortium	Jacquelyn Wynn, Mairead Hyland, The Lighthouse Lab in Alderley Park and Alex Alderton, Roberto Amato, Sonia Goncalves, Ewan Harrison, David K. Jackson, Ian Johnston, Dominic Kwiatkowski, Cordelia Langford, John Sillitoe on behalf of the Wellcome Sanger Institute COVID-19 Surveillance Team
EPI_ISL_756511, EPI_ISL_756512, EPI_ISL_756513	Lighthouse Lab in Cambridge	Wellcome Sanger Institute for the COVID-19 Genomics UK (COG-UK) Consortium	Rob Howes, The Lighthouse Lab in Cambridge and Alex Alderton, Roberto Amato, Sonia Goncalves, Ewan Harrison, David K. Jackson, Ian Johnston, Dominic Kwiatkowski, Cordelia Langford, John Sillitoe on behalf of the Wellcome Sanger Institute COVID-19 Surveillance Team
EPI_ISL_756514, EPI_ISL_756515, EPI_ISL_756516	Lighthouse Lab in Alderley Park	Wellcome Sanger Institute for the COVID-19 Genomics UK (COG-UK) Consortium	Jacquelyn Wynn, Mairead Hyland, The Lighthouse Lab in Alderley Park and Alex Alderton, Roberto Amato, Sonia Goncalves, Ewan Harrison, David K. Jackson, Ian Johnston, Dominic Kwiatkowski, Cordelia Langford, John Sillitoe on behalf of the Wellcome Sanger Institute COVID-19 Surveillance Team
EPI_ISL_756517, EPI_ISL_756518, EPI_ISL_756519	Lighthouse Lab in Cambridge	Wellcome Sanger Institute for the COVID-19 Genomics UK (COG-UK) Consortium	Rob Howes, The Lighthouse Lab in Cambridge and Alex Alderton, Roberto Amato, Sonia Goncalves, Ewan Harrison, David K. Jackson, Ian Johnston, Dominic Kwiatkowski, Cordelia Langford, John Sillitoe on behalf of the Wellcome Sanger Institute COVID-19 Surveillance Team
EPI_ISL_756520	Lighthouse Lab in Alderley Park	Wellcome Sanger Institute for the COVID-19 Genomics UK (COG-UK) Consortium	Jacquelyn Wynn, Mairead Hyland, The Lighthouse Lab in Alderley Park and Alex Alderton, Roberto Amato, Sonia Goncalves, Ewan Harrison, David K. Jackson, Ian Johnston, Dominic Kwiatkowski, Cordelia Langford, John Sillitoe on behalf of the Wellcome Sanger Institute COVID-19 Surveillance Team
EPI_ISL_756521, EPI_ISL_756522	Lighthouse Lab in Cambridge	Wellcome Sanger Institute for the COVID-19 Genomics UK (COG-UK) Consortium	Rob Howes, The Lighthouse Lab in Cambridge and Alex Alderton, Roberto Amato, Sonia Goncalves, Ewan Harrison, David K. Jackson, Ian Johnston, Dominic Kwiatkowski, Cordelia Langford, John Sillitoe on behalf of the Wellcome Sanger Institute COVID-19 Surveillance Team
EPI_ISL_756523, EPI_ISL_756524	Lighthouse Lab in Alderley Park	Wellcome Sanger Institute for the COVID-19 Genomics UK (COG-UK) Consortium	Jacquelyn Wynn, Mairead Hyland, The Lighthouse Lab in Alderley Park and Alex Alderton, Roberto Amato, Sonia Goncalves, Ewan Harrison, David K. Jackson, Ian Johnston, Dominic Kwiatkowski, Cordelia Langford, John Sillitoe on behalf of the Wellcome Sanger Institute COVID-19 Surveillance Team
EPI_ISL_756525	Lighthouse Lab in Cambridge	Wellcome Sanger Institute for the COVID-19 Genomics UK (COG-UK) Consortium	Rob Howes, The Lighthouse Lab in Cambridge and Alex Alderton, Roberto Amato, Sonia Goncalves, Ewan Harrison, David K. Jackson, Ian Johnston, Dominic Kwiatkowski, Cordelia Langford, John Sillitoe on behalf of the Wellcome Sanger Institute COVID-19 Surveillance Team
EPI_ISL_756527	Lighthouse Lab in Alderley Park	Wellcome Sanger Institute for the COVID-19 Genomics UK (COG-UK) Consortium	Jacquelyn Wynn, Mairead Hyland, The Lighthouse Lab in Alderley Park and Alex Alderton, Roberto Amato, Sonia Goncalves, Ewan Harrison, David K. Jackson, Ian Johnston, Dominic Kwiatkowski, Cordelia Langford, John Sillitoe on behalf of the Wellcome Sanger Institute COVID-19 Surveillance Team
EPI_ISL_756528, EPI_ISL_756529	Lighthouse Lab in Cambridge	Wellcome Sanger Institute for the COVID-19 Genomics UK (COG-UK) Consortium	Rob Howes, The Lighthouse Lab in Cambridge and Alex Alderton, Roberto Amato, Sonia Goncalves, Ewan Harrison, David K. Jackson, Ian Johnston, Dominic Kwiatkowski, Cordelia Langford, John Sillitoe on behalf of the Wellcome Sanger Institute COVID-19 Surveillance Team
EPI_ISL_756530, EPI_ISL_756531, EPI_ISL_756532, EPI_ISL_756533	Lighthouse Lab in Alderley Park	Wellcome Sanger Institute for the COVID-19 Genomics UK (COG-UK) Consortium	Jacquelyn Wynn, Mairead Hyland, The Lighthouse Lab in Alderley Park and Alex Alderton, Roberto Amato, Sonia Goncalves, Ewan Harrison, David K. Jackson, Ian Johnston, Dominic Kwiatkowski, Cordelia Langford, John Sillitoe on behalf of the Wellcome Sanger Institute COVID-19 Surveillance Team
EPI_ISL_756534, EPI_ISL_756535	Lighthouse Lab in Cambridge	Wellcome Sanger Institute for the COVID-19 Genomics UK (COG-UK) Consortium	Rob Howes, The Lighthouse Lab in Cambridge and Alex Alderton, Roberto Amato, Sonia Goncalves, Ewan Harrison, David K. Jackson, Ian Johnston, Dominic Kwiatkowski, Cordelia Langford, John Sillitoe on behalf of the Wellcome Sanger Institute COVID-19 Surveillance Team
EPI_ISL_756536, EPI_ISL_756537, EPI_ISL_756538, EPI_ISL_756539	Lighthouse Lab in Alderley Park	Wellcome Sanger Institute for the COVID-19 Genomics UK (COG-UK) Consortium	Jacquelyn Wynn, Mairead Hyland, The Lighthouse Lab in Alderley Park and Alex Alderton, Roberto Amato, Sonia Goncalves, Ewan Harrison, David K. Jackson, Ian Johnston, Dominic Kwiatkowski, Cordelia Langford, John Sillitoe on behalf of the Wellcome Sanger Institute COVID-19 Surveillance Team
EPI_ISL_756540	Lighthouse Lab in Cambridge	Wellcome Sanger Institute for the COVID-19 Genomics UK (COG-UK) Consortium	Rob Howes, The Lighthouse Lab in Cambridge and Alex Alderton, Roberto Amato, Sonia Goncalves, Ewan Harrison, David K. Jackson, Ian Johnston, Dominic Kwiatkowski, Cordelia Langford, John Sillitoe on behalf of the Wellcome Sanger Institute COVID-19 Surveillance Team
EPI_ISL_756541	Lighthouse Lab in Alderley Park	Wellcome Sanger Institute for the COVID-19 Genomics UK (COG-UK) Consortium	Jacquelyn Wynn, Mairead Hyland, The Lighthouse Lab in Alderley Park and Alex Alderton, Roberto Amato, Sonia Goncalves, Ewan Harrison, David K. Jackson, Ian Johnston, Dominic Kwiatkowski, Cordelia Langford, John Sillitoe on behalf of the Wellcome Sanger Institute COVID-19 Surveillance Team
EPI_ISL_756542, EPI_ISL_756543	Lighthouse Lab in Cambridge	Wellcome Sanger Institute for the COVID-19 Genomics UK (COG-UK) Consortium	Rob Howes, The Lighthouse Lab in Cambridge and Alex Alderton, Roberto Amato, Sonia Goncalves, Ewan Harrison, David K. Jackson, Ian Johnston, Dominic Kwiatkowski, Cordelia Langford, John Sillitoe on behalf of the Wellcome Sanger Institute COVID-19 Surveillance Team
EPI_ISL_756544	Lighthouse Lab in Alderley Park	Wellcome Sanger Institute for the COVID-19 Genomics UK (COG-UK) Consortium	Jacquelyn Wynn, Mairead Hyland, The Lighthouse Lab in Alderley Park and Alex Alderton, Roberto Amato, Sonia Goncalves, Ewan Harrison, David K. Jackson, Ian Johnston, Dominic Kwiatkowski, Cordelia Langford, John Sillitoe on behalf of the Wellcome Sanger Institute COVID-19 Surveillance Team
EPI_ISL_756545	Lighthouse Lab in Cambridge	Wellcome Sanger Institute for the COVID-19 Genomics UK (COG-UK) Consortium	Rob Howes, The Lighthouse Lab in Cambridge and Alex Alderton, Roberto Amato, Sonia Goncalves, Ewan Harrison, David K. Jackson, Ian Johnston, Dominic Kwiatkowski, Cordelia Langford, John Sillitoe on behalf of the Wellcome Sanger Institute COVID-19 Surveillance Team
EPI_ISL_756546, EPI_ISL_756547, EPI_ISL_756548	Lighthouse Lab in Alderley Park	Wellcome Sanger Institute for the COVID-19 Genomics UK (COG-UK) Consortium	Jacquelyn Wynn, Mairead Hyland, The Lighthouse Lab in Alderley Park and Alex Alderton, Roberto Amato, Sonia Goncalves, Ewan Harrison, David K. Jackson, Ian Johnston, Dominic Kwiatkowski, Cordelia Langford, John Sillitoe on behalf of the Wellcome Sanger Institute COVID-19 Surveillance Team
EPI_ISL_756549	Lighthouse Lab in Cambridge	Wellcome Sanger Institute for the	Rob Howes, The Lighthouse Lab in Cambridge and Alex Alderton, Roberto Amato, Sonia Goncalves, Ewan Harrison, David K. Jackson, Ian Johnston, Dominic Kwiatkowski, Cordelia Langford, John Sillitoe on behalf of the Wellcome Sanger Institute COVID-19 Surveillance Team

		COVID-19 Genomics UK (COG-UK) Consortium	
EPI_ISL_756550, EPI_ISL_756551, EPI_ISL_756552	Lighthouse Lab in Alderley Park	Wellcome Sanger Institute for the COVID-19 Genomics UK (COG-UK) Consortium	Jacquelyn Wynn, Mairead Hyland, The Lighthouse Lab in Alderley Park and Alex Alderton, Roberto Amato, Sonia Goncalves, Ewan Harrison, David K. Jackson, Ian Johnston, Dominic Kwiatkowski, Cordelia Langford, John Sillitoe on behalf of the Wellcome Sanger Institute COVID-19 Surveillance Team
EPI_ISL_756553, EPI_ISL_756554	Lighthouse Lab in Cambridge	Wellcome Sanger Institute for the COVID-19 Genomics UK (COG-UK) Consortium	Rob Howes, The Lighthouse Lab in Cambridge and Alex Alderton, Roberto Amato, Sonia Goncalves, Ewan Harrison, David K. Jackson, Ian Johnston, Dominic Kwiatkowski, Cordelia Langford, John Sillitoe on behalf of the Wellcome Sanger Institute COVID-19 Surveillance Team
EPI_ISL_756555, EPI_ISL_756556, EPI_ISL_756557	Lighthouse Lab in Alderley Park	Wellcome Sanger Institute for the COVID-19 Genomics UK (COG-UK) Consortium	Jacquelyn Wynn, Mairead Hyland, The Lighthouse Lab in Alderley Park and Alex Alderton, Roberto Amato, Sonia Goncalves, Ewan Harrison, David K. Jackson, Ian Johnston, Dominic Kwiatkowski, Cordelia Langford, John Sillitoe on behalf of the Wellcome Sanger Institute COVID-19 Surveillance Team
EPI_ISL_756558, EPI_ISL_756559	Lighthouse Lab in Cambridge	Wellcome Sanger Institute for the COVID-19 Genomics UK (COG-UK) Consortium	Rob Howes, The Lighthouse Lab in Cambridge and Alex Alderton, Roberto Amato, Sonia Goncalves, Ewan Harrison, David K. Jackson, Ian Johnston, Dominic Kwiatkowski, Cordelia Langford, John Sillitoe on behalf of the Wellcome Sanger Institute COVID-19 Surveillance Team
EPI_ISL_756560, EPI_ISL_756561, EPI_ISL_756562, EPI_ISL_756563	Lighthouse Lab in Alderley Park	Wellcome Sanger Institute for the COVID-19 Genomics UK (COG-UK) Consortium	Jacquelyn Wynn, Mairead Hyland, The Lighthouse Lab in Alderley Park and Alex Alderton, Roberto Amato, Sonia Goncalves, Ewan Harrison, David K. Jackson, Ian Johnston, Dominic Kwiatkowski, Cordelia Langford, John Sillitoe on behalf of the Wellcome Sanger Institute COVID-19 Surveillance Team
EPI_ISL_756564	Lighthouse Lab in Cambridge	Wellcome Sanger Institute for the COVID-19 Genomics UK (COG-UK) Consortium	Rob Howes, The Lighthouse Lab in Cambridge and Alex Alderton, Roberto Amato, Sonia Goncalves, Ewan Harrison, David K. Jackson, Ian Johnston, Dominic Kwiatkowski, Cordelia Langford, John Sillitoe on behalf of the Wellcome Sanger Institute COVID-19 Surveillance Team
EPI_ISL_756565, EPI_ISL_756566	Lighthouse Lab in Alderley Park	Wellcome Sanger Institute for the COVID-19 Genomics UK (COG-UK) Consortium	Jacquelyn Wynn, Mairead Hyland, The Lighthouse Lab in Alderley Park and Alex Alderton, Roberto Amato, Sonia Goncalves, Ewan Harrison, David K. Jackson, Ian Johnston, Dominic Kwiatkowski, Cordelia Langford, John Sillitoe on behalf of the Wellcome Sanger Institute COVID-19 Surveillance Team
EPI_ISL_756567, EPI_ISL_756568	Lighthouse Lab in Cambridge	Wellcome Sanger Institute for the COVID-19 Genomics UK (COG-UK) Consortium	Rob Howes, The Lighthouse Lab in Cambridge and Alex Alderton, Roberto Amato, Sonia Goncalves, Ewan Harrison, David K. Jackson, Ian Johnston, Dominic Kwiatkowski, Cordelia Langford, John Sillitoe on behalf of the Wellcome Sanger Institute COVID-19 Surveillance Team
EPI_ISL_756569	Lighthouse Lab in Alderley Park	Wellcome Sanger Institute for the COVID-19 Genomics UK (COG-UK) Consortium	Jacquelyn Wynn, Mairead Hyland, The Lighthouse Lab in Alderley Park and Alex Alderton, Roberto Amato, Sonia Goncalves, Ewan Harrison, David K. Jackson, Ian Johnston, Dominic Kwiatkowski, Cordelia Langford, John Sillitoe on behalf of the Wellcome Sanger Institute COVID-19 Surveillance Team
EPI_ISL_756570	Lighthouse Lab in Cambridge	Wellcome Sanger Institute for the COVID-19 Genomics UK (COG-UK) Consortium	Rob Howes, The Lighthouse Lab in Cambridge and Alex Alderton, Roberto Amato, Sonia Goncalves, Ewan Harrison, David K. Jackson, Ian Johnston, Dominic Kwiatkowski, Cordelia Langford, John Sillitoe on behalf of the Wellcome Sanger Institute COVID-19 Surveillance Team
EPI_ISL_756571	Lighthouse Lab in Alderley Park	Wellcome Sanger Institute for the COVID-19 Genomics UK (COG-UK) Consortium	Jacquelyn Wynn, Mairead Hyland, The Lighthouse Lab in Alderley Park and Alex Alderton, Roberto Amato, Sonia Goncalves, Ewan Harrison, David K. Jackson, Ian Johnston, Dominic Kwiatkowski, Cordelia Langford, John Sillitoe on behalf of the Wellcome Sanger Institute COVID-19 Surveillance Team
EPI_ISL_756572	Lighthouse Lab in Cambridge	Wellcome Sanger Institute for the COVID-19 Genomics UK (COG-UK) Consortium	Rob Howes, The Lighthouse Lab in Cambridge and Alex Alderton, Roberto Amato, Sonia Goncalves, Ewan Harrison, David K. Jackson, Ian Johnston, Dominic Kwiatkowski, Cordelia Langford, John Sillitoe on behalf of the Wellcome Sanger Institute COVID-19 Surveillance Team
EPI_ISL_756574, EPI_ISL_756575	Lighthouse Lab in Alderley Park	Wellcome Sanger Institute for the COVID-19 Genomics UK (COG-UK) Consortium	Jacquelyn Wynn, Mairead Hyland, The Lighthouse Lab in Alderley Park and Alex Alderton, Roberto Amato, Sonia Goncalves, Ewan Harrison, David K. Jackson, Ian Johnston, Dominic Kwiatkowski, Cordelia Langford, John Sillitoe on behalf of the Wellcome Sanger Institute COVID-19 Surveillance Team
EPI_ISL_756576, EPI_ISL_756577	Lighthouse Lab in Cambridge	Wellcome Sanger Institute for the COVID-19 Genomics UK (COG-UK) Consortium	Rob Howes, The Lighthouse Lab in Cambridge and Alex Alderton, Roberto Amato, Sonia Goncalves, Ewan Harrison, David K. Jackson, Ian Johnston, Dominic Kwiatkowski, Cordelia Langford, John Sillitoe on behalf of the Wellcome Sanger Institute COVID-19 Surveillance Team
EPI_ISL_756578	Lighthouse Lab in Alderley Park	Wellcome Sanger Institute for the COVID-19 Genomics UK (COG-UK) Consortium	Jacquelyn Wynn, Mairead Hyland, The Lighthouse Lab in Alderley Park and Alex Alderton, Roberto Amato, Sonia Goncalves, Ewan Harrison, David K. Jackson, Ian Johnston, Dominic Kwiatkowski, Cordelia Langford, John Sillitoe on behalf of the Wellcome Sanger Institute COVID-19 Surveillance Team
EPI_ISL_756579	Lighthouse Lab in Cambridge	Wellcome Sanger Institute for the COVID-19 Genomics UK (COG-UK) Consortium	Rob Howes, The Lighthouse Lab in Cambridge and Alex Alderton, Roberto Amato, Sonia Goncalves, Ewan Harrison, David K. Jackson, Ian Johnston, Dominic Kwiatkowski, Cordelia Langford, John Sillitoe on behalf of the Wellcome Sanger Institute COVID-19 Surveillance Team
EPI_ISL_756580, EPI_ISL_756581	Lighthouse Lab in Alderley Park	Wellcome Sanger Institute for the COVID-19 Genomics UK (COG-UK) Consortium	Jacquelyn Wynn, Mairead Hyland, The Lighthouse Lab in Alderley Park and Alex Alderton, Roberto Amato, Sonia Goncalves, Ewan Harrison, David K. Jackson, Ian Johnston, Dominic Kwiatkowski, Cordelia Langford, John Sillitoe on behalf of the Wellcome Sanger Institute COVID-19 Surveillance Team
EPI_ISL_756582	Lighthouse Lab in Cambridge	Wellcome Sanger Institute for the COVID-19 Genomics UK (COG-UK) Consortium	Rob Howes, The Lighthouse Lab in Cambridge and Alex Alderton, Roberto Amato, Sonia Goncalves, Ewan Harrison, David K. Jackson, Ian Johnston, Dominic Kwiatkowski, Cordelia Langford, John Sillitoe on behalf of the Wellcome Sanger Institute COVID-19 Surveillance Team
EPI_ISL_756583	Lighthouse Lab in Alderley Park	Wellcome Sanger Institute for the COVID-19 Genomics UK (COG-UK) Consortium	Jacquelyn Wynn, Mairead Hyland, The Lighthouse Lab in Alderley Park and Alex Alderton, Roberto Amato, Sonia Goncalves, Ewan Harrison, David K. Jackson, Ian Johnston, Dominic Kwiatkowski, Cordelia Langford, John Sillitoe on behalf of the Wellcome Sanger Institute COVID-19 Surveillance Team

EPI_ISL_756585, EPI_ISL_756586	Lighthouse Lab in Cambridge	Wellcome Sanger Institute for the COVID-19 Genomics UK (COG-UK) Consortium	Rob Howes, The Lighthouse Lab in Cambridge and Alex Alderton, Roberto Amato, Sonia Goncalves, Ewan Harrison, David K. Jackson, Ian Johnston, Dominic Kwiatkowski, Cordelia Langford, John Sillitoe on behalf of the Wellcome Sanger Institute COVID-19 Surveillance Team
EPI_ISL_756587	Lighthouse Lab in Alderley Park	Wellcome Sanger Institute for the COVID-19 Genomics UK (COG-UK) Consortium	Jacquelyn Wynn, Mairead Hyland, The Lighthouse Lab in Alderley Park and Alex Alderton, Roberto Amato, Sonia Goncalves, Ewan Harrison, David K. Jackson, Ian Johnston, Dominic Kwiatkowski, Cordelia Langford, John Sillitoe on behalf of the Wellcome Sanger Institute COVID-19 Surveillance Team
EPI_ISL_756588, EPI_ISL_756589, EPI_ISL_756590, EPI_ISL_756591	Lighthouse Lab in Cambridge	Wellcome Sanger Institute for the COVID-19 Genomics UK (COG-UK) Consortium	Rob Howes, The Lighthouse Lab in Cambridge and Alex Alderton, Roberto Amato, Sonia Goncalves, Ewan Harrison, David K. Jackson, Ian Johnston, Dominic Kwiatkowski, Cordelia Langford, John Sillitoe on behalf of the Wellcome Sanger Institute COVID-19 Surveillance Team
EPI_ISL_756592, EPI_ISL_756593	Lighthouse Lab in Alderley Park	Wellcome Sanger Institute for the COVID-19 Genomics UK (COG-UK) Consortium	Jacquelyn Wynn, Mairead Hyland, The Lighthouse Lab in Alderley Park and Alex Alderton, Roberto Amato, Sonia Goncalves, Ewan Harrison, David K. Jackson, Ian Johnston, Dominic Kwiatkowski, Cordelia Langford, John Sillitoe on behalf of the Wellcome Sanger Institute COVID-19 Surveillance Team
EPI_ISL_756594	Lighthouse Lab in Cambridge	Wellcome Sanger Institute for the COVID-19 Genomics UK (COG-UK) Consortium	Rob Howes, The Lighthouse Lab in Cambridge and Alex Alderton, Roberto Amato, Sonia Goncalves, Ewan Harrison, David K. Jackson, Ian Johnston, Dominic Kwiatkowski, Cordelia Langford, John Sillitoe on behalf of the Wellcome Sanger Institute COVID-19 Surveillance Team
EPI_ISL_756596	Lighthouse Lab in Alderley Park	Wellcome Sanger Institute for the COVID-19 Genomics UK (COG-UK) Consortium	Jacquelyn Wynn, Mairead Hyland, The Lighthouse Lab in Alderley Park and Alex Alderton, Roberto Amato, Sonia Goncalves, Ewan Harrison, David K. Jackson, Ian Johnston, Dominic Kwiatkowski, Cordelia Langford, John Sillitoe on behalf of the Wellcome Sanger Institute COVID-19 Surveillance Team
EPI_ISL_756597	Lighthouse Lab in Cambridge	Wellcome Sanger Institute for the COVID-19 Genomics UK (COG-UK) Consortium	Rob Howes, The Lighthouse Lab in Cambridge and Alex Alderton, Roberto Amato, Sonia Goncalves, Ewan Harrison, David K. Jackson, Ian Johnston, Dominic Kwiatkowski, Cordelia Langford, John Sillitoe on behalf of the Wellcome Sanger Institute COVID-19 Surveillance Team
EPI_ISL_756598, EPI_ISL_756599, EPI_ISL_756600	Lighthouse Lab in Alderley Park	Wellcome Sanger Institute for the COVID-19 Genomics UK (COG-UK) Consortium	Jacquelyn Wynn, Mairead Hyland, The Lighthouse Lab in Alderley Park and Alex Alderton, Roberto Amato, Sonia Goncalves, Ewan Harrison, David K. Jackson, Ian Johnston, Dominic Kwiatkowski, Cordelia Langford, John Sillitoe on behalf of the Wellcome Sanger Institute COVID-19 Surveillance Team
EPI_ISL_756601	Lighthouse Lab in Cambridge	Wellcome Sanger Institute for the COVID-19 Genomics UK (COG-UK) Consortium	Rob Howes, The Lighthouse Lab in Cambridge and Alex Alderton, Roberto Amato, Sonia Goncalves, Ewan Harrison, David K. Jackson, Ian Johnston, Dominic Kwiatkowski, Cordelia Langford, John Sillitoe on behalf of the Wellcome Sanger Institute COVID-19 Surveillance Team
EPI_ISL_756602, EPI_ISL_756603	Lighthouse Lab in Alderley Park	Wellcome Sanger Institute for the COVID-19 Genomics UK (COG-UK) Consortium	Jacquelyn Wynn, Mairead Hyland, The Lighthouse Lab in Alderley Park and Alex Alderton, Roberto Amato, Sonia Goncalves, Ewan Harrison, David K. Jackson, Ian Johnston, Dominic Kwiatkowski, Cordelia Langford, John Sillitoe on behalf of the Wellcome Sanger Institute COVID-19 Surveillance Team
EPI_ISL_756604, EPI_ISL_756605, EPI_ISL_756606, EPI_ISL_756607, EPI_ISL_756608	Lighthouse Lab in Cambridge	Wellcome Sanger Institute for the COVID-19 Genomics UK (COG-UK) Consortium	Rob Howes, The Lighthouse Lab in Cambridge and Alex Alderton, Roberto Amato, Sonia Goncalves, Ewan Harrison, David K. Jackson, Ian Johnston, Dominic Kwiatkowski, Cordelia Langford, John Sillitoe on behalf of the Wellcome Sanger Institute COVID-19 Surveillance Team
EPI_ISL_756609	Lighthouse Lab in Alderley Park	Wellcome Sanger Institute for the COVID-19 Genomics UK (COG-UK) Consortium	Jacquelyn Wynn, Mairead Hyland, The Lighthouse Lab in Alderley Park and Alex Alderton, Roberto Amato, Sonia Goncalves, Ewan Harrison, David K. Jackson, Ian Johnston, Dominic Kwiatkowski, Cordelia Langford, John Sillitoe on behalf of the Wellcome Sanger Institute COVID-19 Surveillance Team
EPI_ISL_756610, EPI_ISL_756611	Lighthouse Lab in Cambridge	Wellcome Sanger Institute for the COVID-19 Genomics UK (COG-UK) Consortium	Rob Howes, The Lighthouse Lab in Cambridge and Alex Alderton, Roberto Amato, Sonia Goncalves, Ewan Harrison, David K. Jackson, Ian Johnston, Dominic Kwiatkowski, Cordelia Langford, John Sillitoe on behalf of the Wellcome Sanger Institute COVID-19 Surveillance Team
EPI_ISL_756612	Lighthouse Lab in Alderley Park	Wellcome Sanger Institute for the COVID-19 Genomics UK (COG-UK) Consortium	Jacquelyn Wynn, Mairead Hyland, The Lighthouse Lab in Alderley Park and Alex Alderton, Roberto Amato, Sonia Goncalves, Ewan Harrison, David K. Jackson, Ian Johnston, Dominic Kwiatkowski, Cordelia Langford, John Sillitoe on behalf of the Wellcome Sanger Institute COVID-19 Surveillance Team
EPI_ISL_756613, EPI_ISL_756614, EPI_ISL_756615	Lighthouse Lab in Cambridge	Wellcome Sanger Institute for the COVID-19 Genomics UK (COG-UK) Consortium	Rob Howes, The Lighthouse Lab in Cambridge and Alex Alderton, Roberto Amato, Sonia Goncalves, Ewan Harrison, David K. Jackson, Ian Johnston, Dominic Kwiatkowski, Cordelia Langford, John Sillitoe on behalf of the Wellcome Sanger Institute COVID-19 Surveillance Team
EPI_ISL_756616	Lighthouse Lab in Alderley Park	Wellcome Sanger Institute for the COVID-19 Genomics UK (COG-UK) Consortium	Jacquelyn Wynn, Mairead Hyland, The Lighthouse Lab in Alderley Park and Alex Alderton, Roberto Amato, Sonia Goncalves, Ewan Harrison, David K. Jackson, Ian Johnston, Dominic Kwiatkowski, Cordelia Langford, John Sillitoe on behalf of the Wellcome Sanger Institute COVID-19 Surveillance Team
EPI_ISL_756617	Lighthouse Lab in Cambridge	Wellcome Sanger Institute for the COVID-19 Genomics UK (COG-UK) Consortium	Rob Howes, The Lighthouse Lab in Cambridge and Alex Alderton, Roberto Amato, Sonia Goncalves, Ewan Harrison, David K. Jackson, Ian Johnston, Dominic Kwiatkowski, Cordelia Langford, John Sillitoe on behalf of the Wellcome Sanger Institute COVID-19 Surveillance Team
EPI_ISL_756618, EPI_ISL_756619, EPI_ISL_756620	Lighthouse Lab in Alderley Park	Wellcome Sanger Institute for the COVID-19 Genomics UK (COG-UK) Consortium	Jacquelyn Wynn, Mairead Hyland, The Lighthouse Lab in Alderley Park and Alex Alderton, Roberto Amato, Sonia Goncalves, Ewan Harrison, David K. Jackson, Ian Johnston, Dominic Kwiatkowski, Cordelia Langford, John Sillitoe on behalf of the Wellcome Sanger Institute COVID-19 Surveillance Team
EPI_ISL_756621, EPI_ISL_756622, EPI_ISL_756623	Lighthouse Lab in Cambridge	Wellcome Sanger Institute for the COVID-19 Genomics UK (COG-UK) Consortium	Rob Howes, The Lighthouse Lab in Cambridge and Alex Alderton, Roberto Amato, Sonia Goncalves, Ewan Harrison, David K. Jackson, Ian Johnston, Dominic Kwiatkowski, Cordelia Langford, John Sillitoe on behalf of the Wellcome Sanger Institute COVID-19 Surveillance Team
EPI_ISL_756624	Lighthouse Lab in Alderley Park	Wellcome Sanger	Jacquelyn Wynn, Mairead Hyland, The Lighthouse Lab in Alderley Park and Alex Alderton, Roberto Amato, Sonia Goncalves, Ewan Harrison, David K. Jackson, Ian Johnston, Dominic Kwiatkowski, Cordelia Langford, John Sillitoe on behalf of the

		Institute for the COVID-19 Genomics UK (COG-UK) Consortium	Wellcome Sanger Institute COVID-19 Surveillance Team
EPI_ISL_756625	Lighthouse Lab in Cambridge	Wellcome Sanger Institute for the COVID-19 Genomics UK (COG-UK) Consortium	Rob Howes, The Lighthouse Lab in Cambridge and Alex Alderton, Roberto Amato, Sonia Goncalves, Ewan Harrison, David K. Jackson, Ian Johnston, Dominic Kwiatkowski, Cordelia Langford, John Sillitoe on behalf of the Wellcome Sanger Institute COVID-19 Surveillance Team
EPI_ISL_756626	Lighthouse Lab in Alderley Park	Wellcome Sanger Institute for the COVID-19 Genomics UK (COG-UK) Consortium	Jacquelyn Wynn, Mairead Hyland, The Lighthouse Lab in Alderley Park and Alex Alderton, Roberto Amato, Sonia Goncalves, Ewan Harrison, David K. Jackson, Ian Johnston, Dominic Kwiatkowski, Cordelia Langford, John Sillitoe on behalf of the Wellcome Sanger Institute COVID-19 Surveillance Team
EPI_ISL_756627, EPI_ISL_756628, EPI_ISL_756629, EPI_ISL_756630, EPI_ISL_756631	Lighthouse Lab in Cambridge	Wellcome Sanger Institute for the COVID-19 Genomics UK (COG-UK) Consortium	Rob Howes, The Lighthouse Lab in Cambridge and Alex Alderton, Roberto Amato, Sonia Goncalves, Ewan Harrison, David K. Jackson, Ian Johnston, Dominic Kwiatkowski, Cordelia Langford, John Sillitoe on behalf of the Wellcome Sanger Institute COVID-19 Surveillance Team
EPI_ISL_756632	Lighthouse Lab in Alderley Park	Wellcome Sanger Institute for the COVID-19 Genomics UK (COG-UK) Consortium	Jacquelyn Wynn, Mairead Hyland, The Lighthouse Lab in Alderley Park and Alex Alderton, Roberto Amato, Sonia Goncalves, Ewan Harrison, David K. Jackson, Ian Johnston, Dominic Kwiatkowski, Cordelia Langford, John Sillitoe on behalf of the Wellcome Sanger Institute COVID-19 Surveillance Team
EPI_ISL_756633	Lighthouse Lab in Cambridge	Wellcome Sanger Institute for the COVID-19 Genomics UK (COG-UK) Consortium	Rob Howes, The Lighthouse Lab in Cambridge and Alex Alderton, Roberto Amato, Sonia Goncalves, Ewan Harrison, David K. Jackson, Ian Johnston, Dominic Kwiatkowski, Cordelia Langford, John Sillitoe on behalf of the Wellcome Sanger Institute COVID-19 Surveillance Team
EPI_ISL_756634, EPI_ISL_756635, EPI_ISL_756636	Lighthouse Lab in Alderley Park	Wellcome Sanger Institute for the COVID-19 Genomics UK (COG-UK) Consortium	Jacquelyn Wynn, Mairead Hyland, The Lighthouse Lab in Alderley Park and Alex Alderton, Roberto Amato, Sonia Goncalves, Ewan Harrison, David K. Jackson, Ian Johnston, Dominic Kwiatkowski, Cordelia Langford, John Sillitoe on behalf of the Wellcome Sanger Institute COVID-19 Surveillance Team
EPI_ISL_756637	Lighthouse Lab in Cambridge	Wellcome Sanger Institute for the COVID-19 Genomics UK (COG-UK) Consortium	Rob Howes, The Lighthouse Lab in Cambridge and Alex Alderton, Roberto Amato, Sonia Goncalves, Ewan Harrison, David K. Jackson, Ian Johnston, Dominic Kwiatkowski, Cordelia Langford, John Sillitoe on behalf of the Wellcome Sanger Institute COVID-19 Surveillance Team
EPI_ISL_756638, EPI_ISL_756639, EPI_ISL_756640, EPI_ISL_756641	Lighthouse Lab in Alderley Park	Wellcome Sanger Institute for the COVID-19 Genomics UK (COG-UK) Consortium	Jacquelyn Wynn, Mairead Hyland, The Lighthouse Lab in Alderley Park and Alex Alderton, Roberto Amato, Sonia Goncalves, Ewan Harrison, David K. Jackson, Ian Johnston, Dominic Kwiatkowski, Cordelia Langford, John Sillitoe on behalf of the Wellcome Sanger Institute COVID-19 Surveillance Team
EPI_ISL_756642, EPI_ISL_756643	Lighthouse Lab in Cambridge	Wellcome Sanger Institute for the COVID-19 Genomics UK (COG-UK) Consortium	Rob Howes, The Lighthouse Lab in Cambridge and Alex Alderton, Roberto Amato, Sonia Goncalves, Ewan Harrison, David K. Jackson, Ian Johnston, Dominic Kwiatkowski, Cordelia Langford, John Sillitoe on behalf of the Wellcome Sanger Institute COVID-19 Surveillance Team
EPI_ISL_756644, EPI_ISL_756645, EPI_ISL_756646, EPI_ISL_756647, EPI_ISL_756648	Lighthouse Lab in Alderley Park	Wellcome Sanger Institute for the COVID-19 Genomics UK (COG-UK) Consortium	Jacquelyn Wynn, Mairead Hyland, The Lighthouse Lab in Alderley Park and Alex Alderton, Roberto Amato, Sonia Goncalves, Ewan Harrison, David K. Jackson, Ian Johnston, Dominic Kwiatkowski, Cordelia Langford, John Sillitoe on behalf of the Wellcome Sanger Institute COVID-19 Surveillance Team
EPI_ISL_756649	Lighthouse Lab in Cambridge	Wellcome Sanger Institute for the COVID-19 Genomics UK (COG-UK) Consortium	Rob Howes, The Lighthouse Lab in Cambridge and Alex Alderton, Roberto Amato, Sonia Goncalves, Ewan Harrison, David K. Jackson, Ian Johnston, Dominic Kwiatkowski, Cordelia Langford, John Sillitoe on behalf of the Wellcome Sanger Institute COVID-19 Surveillance Team
EPI_ISL_756650, EPI_ISL_756651, EPI_ISL_756652	Lighthouse Lab in Alderley Park	Wellcome Sanger Institute for the COVID-19 Genomics UK (COG-UK) Consortium	Jacquelyn Wynn, Mairead Hyland, The Lighthouse Lab in Alderley Park and Alex Alderton, Roberto Amato, Sonia Goncalves, Ewan Harrison, David K. Jackson, Ian Johnston, Dominic Kwiatkowski, Cordelia Langford, John Sillitoe on behalf of the Wellcome Sanger Institute COVID-19 Surveillance Team
EPI_ISL_756653	Lighthouse Lab in Cambridge	Wellcome Sanger Institute for the COVID-19 Genomics UK (COG-UK) Consortium	Rob Howes, The Lighthouse Lab in Cambridge and Alex Alderton, Roberto Amato, Sonia Goncalves, Ewan Harrison, David K. Jackson, Ian Johnston, Dominic Kwiatkowski, Cordelia Langford, John Sillitoe on behalf of the Wellcome Sanger Institute COVID-19 Surveillance Team
EPI_ISL_756654	Lighthouse Lab in Alderley Park	Wellcome Sanger Institute for the COVID-19 Genomics UK (COG-UK) Consortium	Jacquelyn Wynn, Mairead Hyland, The Lighthouse Lab in Alderley Park and Alex Alderton, Roberto Amato, Sonia Goncalves, Ewan Harrison, David K. Jackson, Ian Johnston, Dominic Kwiatkowski, Cordelia Langford, John Sillitoe on behalf of the Wellcome Sanger Institute COVID-19 Surveillance Team
EPI_ISL_756655	Lighthouse Lab in Cambridge	Wellcome Sanger Institute for the COVID-19 Genomics UK (COG-UK) Consortium	Rob Howes, The Lighthouse Lab in Cambridge and Alex Alderton, Roberto Amato, Sonia Goncalves, Ewan Harrison, David K. Jackson, Ian Johnston, Dominic Kwiatkowski, Cordelia Langford, John Sillitoe on behalf of the Wellcome Sanger Institute COVID-19 Surveillance Team
EPI_ISL_756656, EPI_ISL_756657, EPI_ISL_756658	Lighthouse Lab in Alderley Park	Wellcome Sanger Institute for the COVID-19 Genomics UK (COG-UK) Consortium	Jacquelyn Wynn, Mairead Hyland, The Lighthouse Lab in Alderley Park and Alex Alderton, Roberto Amato, Sonia Goncalves, Ewan Harrison, David K. Jackson, Ian Johnston, Dominic Kwiatkowski, Cordelia Langford, John Sillitoe on behalf of the Wellcome Sanger Institute COVID-19 Surveillance Team
EPI_ISL_756659, EPI_ISL_756660	Lighthouse Lab in Cambridge	Wellcome Sanger Institute for the COVID-19 Genomics UK (COG-UK) Consortium	Rob Howes, The Lighthouse Lab in Cambridge and Alex Alderton, Roberto Amato, Sonia Goncalves, Ewan Harrison, David K. Jackson, Ian Johnston, Dominic Kwiatkowski, Cordelia Langford, John Sillitoe on behalf of the Wellcome Sanger Institute COVID-19 Surveillance Team
EPI_ISL_756661, EPI_ISL_756662, EPI_ISL_756663, EPI_ISL_756664	Lighthouse Lab in Alderley Park	Wellcome Sanger Institute for the COVID-19 Genomics UK (COG-UK) Consortium	Jacquelyn Wynn, Mairead Hyland, The Lighthouse Lab in Alderley Park and Alex Alderton, Roberto Amato, Sonia Goncalves, Ewan Harrison, David K. Jackson, Ian Johnston, Dominic Kwiatkowski, Cordelia Langford, John Sillitoe on behalf of the Wellcome Sanger Institute COVID-19 Surveillance Team
EPI_ISL_756665,	Lighthouse Lab in Cambridge	Wellcome Sanger	Rob Howes, The Lighthouse Lab in Cambridge and Alex Alderton, Roberto Amato, Sonia Goncalves, Ewan Harrison, David K. Jackson, Ian Johnston, Dominic Kwiatkowski, Cordelia Langford, John Sillitoe on behalf of the Wellcome Sanger Institute

EPI_ISL_756666, EPI_ISL_756667, EPI_ISL_756668		Institute for the COVID-19 Genomics UK (COG-UK) Consortium	COVID-19 Surveillance Team
EPI_ISL_756669, EPI_ISL_756670	Lighthouse Lab in Alderley Park	Wellcome Sanger Institute for the COVID-19 Genomics UK (COG-UK) Consortium	Jacquelyn Wynn, Mairead Hyland, The Lighthouse Lab in Alderley Park and Alex Alderton, Roberto Amato, Sonia Goncalves, Ewan Harrison, David K. Jackson, Ian Johnston, Dominic Kwiatkowski, Cordelia Langford, John Sillitoe on behalf of the Wellcome Sanger Institute COVID-19 Surveillance Team
EPI_ISL_756671, EPI_ISL_756672	Lighthouse Lab in Cambridge	Wellcome Sanger Institute for the COVID-19 Genomics UK (COG-UK) Consortium	Rob Howes, The Lighthouse Lab in Cambridge and Alex Alderton, Roberto Amato, Sonia Goncalves, Ewan Harrison, David K. Jackson, Ian Johnston, Dominic Kwiatkowski, Cordelia Langford, John Sillitoe on behalf of the Wellcome Sanger Institute COVID-19 Surveillance Team
EPI_ISL_756673	Lighthouse Lab in Alderley Park	Wellcome Sanger Institute for the COVID-19 Genomics UK (COG-UK) Consortium	Jacquelyn Wynn, Mairead Hyland, The Lighthouse Lab in Alderley Park and Alex Alderton, Roberto Amato, Sonia Goncalves, Ewan Harrison, David K. Jackson, Ian Johnston, Dominic Kwiatkowski, Cordelia Langford, John Sillitoe on behalf of the Wellcome Sanger Institute COVID-19 Surveillance Team
EPI_ISL_756674, EPI_ISL_756675, EPI_ISL_756676, EPI_ISL_756677, EPI_ISL_756678, EPI_ISL_756679, EPI_ISL_756680	Lighthouse Lab in Cambridge	Wellcome Sanger Institute for the COVID-19 Genomics UK (COG-UK) Consortium	Rob Howes, The Lighthouse Lab in Cambridge and Alex Alderton, Roberto Amato, Sonia Goncalves, Ewan Harrison, David K. Jackson, Ian Johnston, Dominic Kwiatkowski, Cordelia Langford, John Sillitoe on behalf of the Wellcome Sanger Institute COVID-19 Surveillance Team
EPI_ISL_756681, EPI_ISL_756682, EPI_ISL_756683, EPI_ISL_756684, EPI_ISL_756685, EPI_ISL_756686, EPI_ISL_756688	Lighthouse Lab in Alderley Park	Wellcome Sanger Institute for the COVID-19 Genomics UK (COG-UK) Consortium	Jacquelyn Wynn, Mairead Hyland, The Lighthouse Lab in Alderley Park and Alex Alderton, Roberto Amato, Sonia Goncalves, Ewan Harrison, David K. Jackson, Ian Johnston, Dominic Kwiatkowski, Cordelia Langford, John Sillitoe on behalf of the Wellcome Sanger Institute COVID-19 Surveillance Team
EPI_ISL_756689	Lighthouse Lab in Milton Keynes	Wellcome Sanger Institute for the COVID-19 Genomics UK (COG-UK) Consortium	The Lighthouse Lab in Milton Keynes and Alex Alderton, Roberto Amato, Sonia Goncalves, Ewan Harrison, David K. Jackson, Ian Johnston, Dominic Kwiatkowski, Cordelia Langford, John Sillitoe on behalf of the Wellcome Sanger Institute COVID-19 Surveillance Team
EPI_ISL_756690	Lighthouse Lab in Glasgow	Wellcome Sanger Institute for the COVID-19 Genomics UK (COG-UK) Consortium	Harper VanSteenhouse, Yumi Kasai, David Gray, Carol Clugston, Anna Dominiczak and Alex Alderton, Roberto Amato, Sonia Goncalves, Ewan Harrison, David K. Jackson, Ian Johnston, Dominic Kwiatkowski, Cordelia Langford, John Sillitoe on behalf of the Wellcome Sanger Institute COVID-19 Surveillance Team
EPI_ISL_756691, EPI_ISL_756692, EPI_ISL_756693, EPI_ISL_756694	Lighthouse Lab in Milton Keynes	Wellcome Sanger Institute for the COVID-19 Genomics UK (COG-UK) Consortium	The Lighthouse Lab in Milton Keynes and Alex Alderton, Roberto Amato, Sonia Goncalves, Ewan Harrison, David K. Jackson, Ian Johnston, Dominic Kwiatkowski, Cordelia Langford, John Sillitoe on behalf of the Wellcome Sanger Institute COVID-19 Surveillance Team
EPI_ISL_756695, EPI_ISL_756696	Lighthouse Lab in Glasgow	Wellcome Sanger Institute for the COVID-19 Genomics UK (COG-UK) Consortium	Harper VanSteenhouse, Yumi Kasai, David Gray, Carol Clugston, Anna Dominiczak and Alex Alderton, Roberto Amato, Sonia Goncalves, Ewan Harrison, David K. Jackson, Ian Johnston, Dominic Kwiatkowski, Cordelia Langford, John Sillitoe on behalf of the Wellcome Sanger Institute COVID-19 Surveillance Team
EPI_ISL_756698, EPI_ISL_756699, EPI_ISL_756700, EPI_ISL_756701	Lighthouse Lab in Milton Keynes	Wellcome Sanger Institute for the COVID-19 Genomics UK (COG-UK) Consortium	The Lighthouse Lab in Milton Keynes and Alex Alderton, Roberto Amato, Sonia Goncalves, Ewan Harrison, David K. Jackson, Ian Johnston, Dominic Kwiatkowski, Cordelia Langford, John Sillitoe on behalf of the Wellcome Sanger Institute COVID-19 Surveillance Team
EPI_ISL_756702	Lighthouse Lab in Glasgow	Wellcome Sanger Institute for the COVID-19 Genomics UK (COG-UK) Consortium	Harper VanSteenhouse, Yumi Kasai, David Gray, Carol Clugston, Anna Dominiczak and Alex Alderton, Roberto Amato, Sonia Goncalves, Ewan Harrison, David K. Jackson, Ian Johnston, Dominic Kwiatkowski, Cordelia Langford, John Sillitoe on behalf of the Wellcome Sanger Institute COVID-19 Surveillance Team
EPI_ISL_756703, EPI_ISL_756704	Lighthouse Lab in Milton Keynes	Wellcome Sanger Institute for the COVID-19 Genomics UK (COG-UK) Consortium	The Lighthouse Lab in Milton Keynes and Alex Alderton, Roberto Amato, Sonia Goncalves, Ewan Harrison, David K. Jackson, Ian Johnston, Dominic Kwiatkowski, Cordelia Langford, John Sillitoe on behalf of the Wellcome Sanger Institute COVID-19 Surveillance Team
EPI_ISL_756705, EPI_ISL_756706	Lighthouse Lab in Glasgow	Wellcome Sanger Institute for the COVID-19 Genomics UK (COG-UK) Consortium	Harper VanSteenhouse, Yumi Kasai, David Gray, Carol Clugston, Anna Dominiczak and Alex Alderton, Roberto Amato, Sonia Goncalves, Ewan Harrison, David K. Jackson, Ian Johnston, Dominic Kwiatkowski, Cordelia Langford, John Sillitoe on behalf of the Wellcome Sanger Institute COVID-19 Surveillance Team
EPI_ISL_756707, EPI_ISL_756708	Lighthouse Lab in Milton Keynes	Wellcome Sanger Institute for the COVID-19 Genomics UK (COG-UK) Consortium	The Lighthouse Lab in Milton Keynes and Alex Alderton, Roberto Amato, Sonia Goncalves, Ewan Harrison, David K. Jackson, Ian Johnston, Dominic Kwiatkowski, Cordelia Langford, John Sillitoe on behalf of the Wellcome Sanger Institute COVID-19 Surveillance Team
EPI_ISL_756709	Lighthouse Lab in Glasgow	Wellcome Sanger Institute for the COVID-19 Genomics UK (COG-UK) Consortium	Harper VanSteenhouse, Yumi Kasai, David Gray, Carol Clugston, Anna Dominiczak and Alex Alderton, Roberto Amato, Sonia Goncalves, Ewan Harrison, David K. Jackson, Ian Johnston, Dominic Kwiatkowski, Cordelia Langford, John Sillitoe on behalf of the Wellcome Sanger Institute COVID-19 Surveillance Team
EPI_ISL_756710	Lighthouse Lab in Milton Keynes	Wellcome Sanger Institute for the COVID-19 Genomics UK (COG-UK) Consortium	The Lighthouse Lab in Milton Keynes and Alex Alderton, Roberto Amato, Sonia Goncalves, Ewan Harrison, David K. Jackson, Ian Johnston, Dominic Kwiatkowski, Cordelia Langford, John Sillitoe on behalf of the Wellcome Sanger Institute COVID-19 Surveillance Team
EPI_ISL_756711	Lighthouse Lab in Glasgow	Wellcome Sanger Institute for the COVID-19 Genomics UK (COG-UK) Consortium	Harper VanSteenhouse, Yumi Kasai, David Gray, Carol Clugston, Anna Dominiczak and Alex Alderton, Roberto Amato, Sonia Goncalves, Ewan Harrison, David K. Jackson, Ian Johnston, Dominic Kwiatkowski, Cordelia Langford, John Sillitoe on behalf of the Wellcome Sanger Institute COVID-19 Surveillance Team
EPI_ISL_756712,	Lighthouse Lab in Milton Keynes	Wellcome Sanger	The Lighthouse Lab in Milton Keynes and Alex Alderton, Roberto Amato, Sonia Goncalves, Ewan Harrison, David K. Jackson, Ian Johnston, Dominic Kwiatkowski, Cordelia Langford, John Sillitoe on behalf of the Wellcome Sanger Institute COVID-19

EPI_ISL_756713, EPI_ISL_756714, EPI_ISL_756715		Institute for the COVID-19 Genomics UK (COG-UK) Consortium	Surveillance Team
EPI_ISL_756716	Lighthouse Lab in Glasgow	Wellcome Sanger Institute for the COVID-19 Genomics UK (COG-UK) Consortium	Harper VanSteenhouse, Yumi Kasai, David Gray, Carol Clugston, Anna Dominiczak and Alex Alderton, Roberto Amato, Sonia Goncalves, Ewan Harrison, David K. Jackson, Ian Johnston, Dominic Kwiatkowski, Cordelia Langford, John Sillitoe on behalf of the Wellcome Sanger Institute COVID-19 Surveillance Team
EPI_ISL_756717, EPI_ISL_756718, EPI_ISL_756719, EPI_ISL_756720, EPI_ISL_756721, EPI_ISL_756722	Lighthouse Lab in Milton Keynes	Wellcome Sanger Institute for the COVID-19 Genomics UK (COG-UK) Consortium	The Lighthouse Lab in Milton Keynes and Alex Alderton, Roberto Amato, Sonia Goncalves, Ewan Harrison, David K. Jackson, Ian Johnston, Dominic Kwiatkowski, Cordelia Langford, John Sillitoe on behalf of the Wellcome Sanger Institute COVID-19 Surveillance Team
EPI_ISL_756723	Lighthouse Lab in Glasgow	Wellcome Sanger Institute for the COVID-19 Genomics UK (COG-UK) Consortium	Harper VanSteenhouse, Yumi Kasai, David Gray, Carol Clugston, Anna Dominiczak and Alex Alderton, Roberto Amato, Sonia Goncalves, Ewan Harrison, David K. Jackson, Ian Johnston, Dominic Kwiatkowski, Cordelia Langford, John Sillitoe on behalf of the Wellcome Sanger Institute COVID-19 Surveillance Team
EPI_ISL_756724, EPI_ISL_756725, EPI_ISL_756726, EPI_ISL_756727, EPI_ISL_756728	Lighthouse Lab in Milton Keynes	Wellcome Sanger Institute for the COVID-19 Genomics UK (COG-UK) Consortium	The Lighthouse Lab in Milton Keynes and Alex Alderton, Roberto Amato, Sonia Goncalves, Ewan Harrison, David K. Jackson, Ian Johnston, Dominic Kwiatkowski, Cordelia Langford, John Sillitoe on behalf of the Wellcome Sanger Institute COVID-19 Surveillance Team
EPI_ISL_756729	Lighthouse Lab in Glasgow	Wellcome Sanger Institute for the COVID-19 Genomics UK (COG-UK) Consortium	Harper VanSteenhouse, Yumi Kasai, David Gray, Carol Clugston, Anna Dominiczak and Alex Alderton, Roberto Amato, Sonia Goncalves, Ewan Harrison, David K. Jackson, Ian Johnston, Dominic Kwiatkowski, Cordelia Langford, John Sillitoe on behalf of the Wellcome Sanger Institute COVID-19 Surveillance Team
EPI_ISL_756730, EPI_ISL_756731, EPI_ISL_756732, EPI_ISL_756733, EPI_ISL_756734, EPI_ISL_756735, EPI_ISL_756736	Lighthouse Lab in Milton Keynes	Wellcome Sanger Institute for the COVID-19 Genomics UK (COG-UK) Consortium	The Lighthouse Lab in Milton Keynes and Alex Alderton, Roberto Amato, Sonia Goncalves, Ewan Harrison, David K. Jackson, Ian Johnston, Dominic Kwiatkowski, Cordelia Langford, John Sillitoe on behalf of the Wellcome Sanger Institute COVID-19 Surveillance Team
EPI_ISL_756737	Lighthouse Lab in Glasgow	Wellcome Sanger Institute for the COVID-19 Genomics UK (COG-UK) Consortium	Harper VanSteenhouse, Yumi Kasai, David Gray, Carol Clugston, Anna Dominiczak and Alex Alderton, Roberto Amato, Sonia Goncalves, Ewan Harrison, David K. Jackson, Ian Johnston, Dominic Kwiatkowski, Cordelia Langford, John Sillitoe on behalf of the Wellcome Sanger Institute COVID-19 Surveillance Team
EPI_ISL_756738, EPI_ISL_756739, EPI_ISL_756740, EPI_ISL_756741, EPI_ISL_756742, EPI_ISL_756743	Lighthouse Lab in Milton Keynes	Wellcome Sanger Institute for the COVID-19 Genomics UK (COG-UK) Consortium	The Lighthouse Lab in Milton Keynes and Alex Alderton, Roberto Amato, Sonia Goncalves, Ewan Harrison, David K. Jackson, Ian Johnston, Dominic Kwiatkowski, Cordelia Langford, John Sillitoe on behalf of the Wellcome Sanger Institute COVID-19 Surveillance Team
EPI_ISL_756744, EPI_ISL_756745	Lighthouse Lab in Glasgow	Wellcome Sanger Institute for the COVID-19 Genomics UK (COG-UK) Consortium	Harper VanSteenhouse, Yumi Kasai, David Gray, Carol Clugston, Anna Dominiczak and Alex Alderton, Roberto Amato, Sonia Goncalves, Ewan Harrison, David K. Jackson, Ian Johnston, Dominic Kwiatkowski, Cordelia Langford, John Sillitoe on behalf of the Wellcome Sanger Institute COVID-19 Surveillance Team
EPI_ISL_756746, EPI_ISL_756747	Lighthouse Lab in Milton Keynes	Wellcome Sanger Institute for the COVID-19 Genomics UK (COG-UK) Consortium	The Lighthouse Lab in Milton Keynes and Alex Alderton, Roberto Amato, Sonia Goncalves, Ewan Harrison, David K. Jackson, Ian Johnston, Dominic Kwiatkowski, Cordelia Langford, John Sillitoe on behalf of the Wellcome Sanger Institute COVID-19 Surveillance Team
EPI_ISL_756748	Lighthouse Lab in Glasgow	Wellcome Sanger Institute for the COVID-19 Genomics UK (COG-UK) Consortium	Harper VanSteenhouse, Yumi Kasai, David Gray, Carol Clugston, Anna Dominiczak and Alex Alderton, Roberto Amato, Sonia Goncalves, Ewan Harrison, David K. Jackson, Ian Johnston, Dominic Kwiatkowski, Cordelia Langford, John Sillitoe on behalf of the Wellcome Sanger Institute COVID-19 Surveillance Team
EPI_ISL_756749	Lighthouse Lab in Milton Keynes	Wellcome Sanger Institute for the COVID-19 Genomics UK (COG-UK) Consortium	The Lighthouse Lab in Milton Keynes and Alex Alderton, Roberto Amato, Sonia Goncalves, Ewan Harrison, David K. Jackson, Ian Johnston, Dominic Kwiatkowski, Cordelia Langford, John Sillitoe on behalf of the Wellcome Sanger Institute COVID-19 Surveillance Team
EPI_ISL_756750	Lighthouse Lab in Glasgow	Wellcome Sanger Institute for the COVID-19 Genomics UK (COG-UK) Consortium	Harper VanSteenhouse, Yumi Kasai, David Gray, Carol Clugston, Anna Dominiczak and Alex Alderton, Roberto Amato, Sonia Goncalves, Ewan Harrison, David K. Jackson, Ian Johnston, Dominic Kwiatkowski, Cordelia Langford, John Sillitoe on behalf of the Wellcome Sanger Institute COVID-19 Surveillance Team
EPI_ISL_756751, EPI_ISL_756752, EPI_ISL_756753, EPI_ISL_756754, EPI_ISL_756755, EPI_ISL_756756, EPI_ISL_756757, EPI_ISL_756758, EPI_ISL_756759, EPI_ISL_756760	Lighthouse Lab in Milton Keynes	Wellcome Sanger Institute for the COVID-19 Genomics UK (COG-UK) Consortium	The Lighthouse Lab in Milton Keynes and Alex Alderton, Roberto Amato, Sonia Goncalves, Ewan Harrison, David K. Jackson, Ian Johnston, Dominic Kwiatkowski, Cordelia Langford, John Sillitoe on behalf of the Wellcome Sanger Institute COVID-19 Surveillance Team
EPI_ISL_756761	Lighthouse Lab in Glasgow	Wellcome Sanger Institute for the COVID-19 Genomics UK (COG-UK) Consortium	Harper VanSteenhouse, Yumi Kasai, David Gray, Carol Clugston, Anna Dominiczak and Alex Alderton, Roberto Amato, Sonia Goncalves, Ewan Harrison, David K. Jackson, Ian Johnston, Dominic Kwiatkowski, Cordelia Langford, John Sillitoe on behalf of the Wellcome Sanger Institute COVID-19 Surveillance Team
EPI_ISL_756762, EPI_ISL_756763, EPI_ISL_756764, EPI_ISL_756765, EPI_ISL_756766, EPI_ISL_756767, EPI_ISL_756768, EPI_ISL_756769, EPI_ISL_756770, EPI_ISL_756771, EPI_ISL_756772			
see above	Lighthouse Lab in Milton Keynes	Wellcome Sanger	The Lighthouse Lab in Milton Keynes and Alex Alderton, Roberto Amato, Sonia Goncalves, Ewan Harrison, David K. Jackson, Ian Johnston, Dominic Kwiatkowski, Cordelia Langford, John Sillitoe on behalf of the Wellcome Sanger Institute COVID-19

		Institute for the COVID-19 Genomics UK (COG-UK) Consortium	Surveillance Team
EPI_ISL_756773	Lighthouse Lab in Glasgow	Wellcome Sanger Institute for the COVID-19 Genomics UK (COG-UK) Consortium	Harper VanSteenhouse, Yumi Kasai, David Gray, Carol Clugston, Anna Dominiczak and Alex Alderton, Roberto Amato, Sonia Goncalves, Ewan Harrison, David K. Jackson, Ian Johnston, Dominic Kwiatkowski, Cordelia Langford, John Sillitoe on behalf of the Wellcome Sanger Institute COVID-19 Surveillance Team
EPI_ISL_756774	Lighthouse Lab in Milton Keynes	Wellcome Sanger Institute for the COVID-19 Genomics UK (COG-UK) Consortium	The Lighthouse Lab in Milton Keynes and Alex Alderton, Roberto Amato, Sonia Goncalves, Ewan Harrison, David K. Jackson, Ian Johnston, Dominic Kwiatkowski, Cordelia Langford, John Sillitoe on behalf of the Wellcome Sanger Institute COVID-19 Surveillance Team
EPI_ISL_756775	Lighthouse Lab in Glasgow	Wellcome Sanger Institute for the COVID-19 Genomics UK (COG-UK) Consortium	Harper VanSteenhouse, Yumi Kasai, David Gray, Carol Clugston, Anna Dominiczak and Alex Alderton, Roberto Amato, Sonia Goncalves, Ewan Harrison, David K. Jackson, Ian Johnston, Dominic Kwiatkowski, Cordelia Langford, John Sillitoe on behalf of the Wellcome Sanger Institute COVID-19 Surveillance Team
EPI_ISL_756776, EPI_ISL_756777, EPI_ISL_756778, EPI_ISL_756779	Lighthouse Lab in Milton Keynes	Wellcome Sanger Institute for the COVID-19 Genomics UK (COG-UK) Consortium	The Lighthouse Lab in Milton Keynes and Alex Alderton, Roberto Amato, Sonia Goncalves, Ewan Harrison, David K. Jackson, Ian Johnston, Dominic Kwiatkowski, Cordelia Langford, John Sillitoe on behalf of the Wellcome Sanger Institute COVID-19 Surveillance Team
EPI_ISL_756780	Lighthouse Lab in Glasgow	Wellcome Sanger Institute for the COVID-19 Genomics UK (COG-UK) Consortium	Harper VanSteenhouse, Yumi Kasai, David Gray, Carol Clugston, Anna Dominiczak and Alex Alderton, Roberto Amato, Sonia Goncalves, Ewan Harrison, David K. Jackson, Ian Johnston, Dominic Kwiatkowski, Cordelia Langford, John Sillitoe on behalf of the Wellcome Sanger Institute COVID-19 Surveillance Team
EPI_ISL_756781, EPI_ISL_756782, EPI_ISL_756783	Lighthouse Lab in Milton Keynes	Wellcome Sanger Institute for the COVID-19 Genomics UK (COG-UK) Consortium	The Lighthouse Lab in Milton Keynes and Alex Alderton, Roberto Amato, Sonia Goncalves, Ewan Harrison, David K. Jackson, Ian Johnston, Dominic Kwiatkowski, Cordelia Langford, John Sillitoe on behalf of the Wellcome Sanger Institute COVID-19 Surveillance Team
EPI_ISL_756784	Lighthouse Lab in Glasgow	Wellcome Sanger Institute for the COVID-19 Genomics UK (COG-UK) Consortium	Harper VanSteenhouse, Yumi Kasai, David Gray, Carol Clugston, Anna Dominiczak and Alex Alderton, Roberto Amato, Sonia Goncalves, Ewan Harrison, David K. Jackson, Ian Johnston, Dominic Kwiatkowski, Cordelia Langford, John Sillitoe on behalf of the Wellcome Sanger Institute COVID-19 Surveillance Team
EPI_ISL_756785, EPI_ISL_756786, EPI_ISL_756787, EPI_ISL_756788	Lighthouse Lab in Milton Keynes	Wellcome Sanger Institute for the COVID-19 Genomics UK (COG-UK) Consortium	The Lighthouse Lab in Milton Keynes and Alex Alderton, Roberto Amato, Sonia Goncalves, Ewan Harrison, David K. Jackson, Ian Johnston, Dominic Kwiatkowski, Cordelia Langford, John Sillitoe on behalf of the Wellcome Sanger Institute COVID-19 Surveillance Team
EPI_ISL_756789	Lighthouse Lab in Glasgow	Wellcome Sanger Institute for the COVID-19 Genomics UK (COG-UK) Consortium	Harper VanSteenhouse, Yumi Kasai, David Gray, Carol Clugston, Anna Dominiczak and Alex Alderton, Roberto Amato, Sonia Goncalves, Ewan Harrison, David K. Jackson, Ian Johnston, Dominic Kwiatkowski, Cordelia Langford, John Sillitoe on behalf of the Wellcome Sanger Institute COVID-19 Surveillance Team
EPI_ISL_756790, EPI_ISL_756791, EPI_ISL_756792, EPI_ISL_756794	Lighthouse Lab in Milton Keynes	Wellcome Sanger Institute for the COVID-19 Genomics UK (COG-UK) Consortium	The Lighthouse Lab in Milton Keynes and Alex Alderton, Roberto Amato, Sonia Goncalves, Ewan Harrison, David K. Jackson, Ian Johnston, Dominic Kwiatkowski, Cordelia Langford, John Sillitoe on behalf of the Wellcome Sanger Institute COVID-19 Surveillance Team
EPI_ISL_756795	Lighthouse Lab in Glasgow	Wellcome Sanger Institute for the COVID-19 Genomics UK (COG-UK) Consortium	Harper VanSteenhouse, Yumi Kasai, David Gray, Carol Clugston, Anna Dominiczak and Alex Alderton, Roberto Amato, Sonia Goncalves, Ewan Harrison, David K. Jackson, Ian Johnston, Dominic Kwiatkowski, Cordelia Langford, John Sillitoe on behalf of the Wellcome Sanger Institute COVID-19 Surveillance Team
EPI_ISL_756796, EPI_ISL_756797, EPI_ISL_756798, EPI_ISL_756799	Lighthouse Lab in Milton Keynes	Wellcome Sanger Institute for the COVID-19 Genomics UK (COG-UK) Consortium	The Lighthouse Lab in Milton Keynes and Alex Alderton, Roberto Amato, Sonia Goncalves, Ewan Harrison, David K. Jackson, Ian Johnston, Dominic Kwiatkowski, Cordelia Langford, John Sillitoe on behalf of the Wellcome Sanger Institute COVID-19 Surveillance Team
EPI_ISL_756800	Lighthouse Lab in Glasgow	Wellcome Sanger Institute for the COVID-19 Genomics UK (COG-UK) Consortium	Harper VanSteenhouse, Yumi Kasai, David Gray, Carol Clugston, Anna Dominiczak and Alex Alderton, Roberto Amato, Sonia Goncalves, Ewan Harrison, David K. Jackson, Ian Johnston, Dominic Kwiatkowski, Cordelia Langford, John Sillitoe on behalf of the Wellcome Sanger Institute COVID-19 Surveillance Team
EPI_ISL_756801, EPI_ISL_756802	Lighthouse Lab in Milton Keynes	Wellcome Sanger Institute for the COVID-19 Genomics UK (COG-UK) Consortium	The Lighthouse Lab in Milton Keynes and Alex Alderton, Roberto Amato, Sonia Goncalves, Ewan Harrison, David K. Jackson, Ian Johnston, Dominic Kwiatkowski, Cordelia Langford, John Sillitoe on behalf of the Wellcome Sanger Institute COVID-19 Surveillance Team
EPI_ISL_756803	Lighthouse Lab in Glasgow	Wellcome Sanger Institute for the COVID-19 Genomics UK (COG-UK) Consortium	Harper VanSteenhouse, Yumi Kasai, David Gray, Carol Clugston, Anna Dominiczak and Alex Alderton, Roberto Amato, Sonia Goncalves, Ewan Harrison, David K. Jackson, Ian Johnston, Dominic Kwiatkowski, Cordelia Langford, John Sillitoe on behalf of the Wellcome Sanger Institute COVID-19 Surveillance Team
EPI_ISL_756804	Lighthouse Lab in Milton Keynes	Wellcome Sanger Institute for the COVID-19 Genomics UK (COG-UK) Consortium	The Lighthouse Lab in Milton Keynes and Alex Alderton, Roberto Amato, Sonia Goncalves, Ewan Harrison, David K. Jackson, Ian Johnston, Dominic Kwiatkowski, Cordelia Langford, John Sillitoe on behalf of the Wellcome Sanger Institute COVID-19 Surveillance Team
EPI_ISL_756805	Lighthouse Lab in Glasgow	Wellcome Sanger Institute for the COVID-19 Genomics UK (COG-UK) Consortium	Harper VanSteenhouse, Yumi Kasai, David Gray, Carol Clugston, Anna Dominiczak and Alex Alderton, Roberto Amato, Sonia Goncalves, Ewan Harrison, David K. Jackson, Ian Johnston, Dominic Kwiatkowski, Cordelia Langford, John Sillitoe on behalf of the Wellcome Sanger Institute COVID-19 Surveillance Team
EPI_ISL_756806, EPI_ISL_756807, EPI_ISL_756808, EPI_ISL_756809, EPI_ISL_756810, EPI_ISL_756811, EPI_ISL_756812, EPI_ISL_756813, EPI_ISL_756814, EPI_ISL_756815, EPI_ISL_756816, EPI_ISL_756817, EPI_ISL_756818, EPI_ISL_756819, EPI_ISL_756820, EPI_ISL_756821, EPI_ISL_756822, EPI_ISL_756823			
see above	Lighthouse Lab in Milton Keynes	Wellcome Sanger Institute for the COVID-19 Genomics UK (COG-UK) Consortium	The Lighthouse Lab in Milton Keynes and Alex Alderton, Roberto Amato, Sonia Goncalves, Ewan Harrison, David K. Jackson, Ian Johnston, Dominic Kwiatkowski, Cordelia Langford, John Sillitoe on behalf of the Wellcome Sanger Institute COVID-19 Surveillance Team
EPI_ISL_756824	Lighthouse Lab in Glasgow	Wellcome Sanger	Harper VanSteenhouse, Yumi Kasai, David Gray, Carol Clugston, Anna Dominiczak and Alex Alderton, Roberto Amato, Sonia Goncalves, Ewan Harrison, David K. Jackson, Ian Johnston, Dominic Kwiatkowski, Cordelia Langford, John Sillitoe on

		Institute for the COVID-19 Genomics UK (COG-UK) Consortium	behalf of the Wellcome Sanger Institute COVID-19 Surveillance Team
EPI_ISL_756825, EPI_ISL_756826, EPI_ISL_756827, EPI_ISL_756828, EPI_ISL_756829, EPI_ISL_756830, EPI_ISL_756831, EPI_ISL_756832, EPI_ISL_756833, EPI_ISL_756834	Lighthouse Lab in Milton Keynes	Wellcome Sanger Institute for the COVID-19 Genomics UK (COG-UK) Consortium	The Lighthouse Lab in Milton Keynes and Alex Alderton, Roberto Amato, Sonia Goncalves, Ewan Harrison, David K. Jackson, Ian Johnston, Dominic Kwiatkowski, Cordelia Langford, John Sillitoe on behalf of the Wellcome Sanger Institute COVID-19 Surveillance Team
EPI_ISL_756835	Lighthouse Lab in Glasgow	Wellcome Sanger Institute for the COVID-19 Genomics UK (COG-UK) Consortium	Harper VanSteenhouse, Yumi Kasai, David Gray, Carol Clugston, Anna Dominiczak and Alex Alderton, Roberto Amato, Sonia Goncalves, Ewan Harrison, David K. Jackson, Ian Johnston, Dominic Kwiatkowski, Cordelia Langford, John Sillitoe on behalf of the Wellcome Sanger Institute COVID-19 Surveillance Team
EPI_ISL_756836, EPI_ISL_756837, EPI_ISL_756838, EPI_ISL_756839	Lighthouse Lab in Milton Keynes	Wellcome Sanger Institute for the COVID-19 Genomics UK (COG-UK) Consortium	The Lighthouse Lab in Milton Keynes and Alex Alderton, Roberto Amato, Sonia Goncalves, Ewan Harrison, David K. Jackson, Ian Johnston, Dominic Kwiatkowski, Cordelia Langford, John Sillitoe on behalf of the Wellcome Sanger Institute COVID-19 Surveillance Team
EPI_ISL_756840	Lighthouse Lab in Glasgow	Wellcome Sanger Institute for the COVID-19 Genomics UK (COG-UK) Consortium	Harper VanSteenhouse, Yumi Kasai, David Gray, Carol Clugston, Anna Dominiczak and Alex Alderton, Roberto Amato, Sonia Goncalves, Ewan Harrison, David K. Jackson, Ian Johnston, Dominic Kwiatkowski, Cordelia Langford, John Sillitoe on behalf of the Wellcome Sanger Institute COVID-19 Surveillance Team
EPI_ISL_756841, EPI_ISL_756842, EPI_ISL_756843, EPI_ISL_756844, EPI_ISL_756845, EPI_ISL_756846, EPI_ISL_756847, EPI_ISL_756848	Lighthouse Lab in Milton Keynes	Wellcome Sanger Institute for the COVID-19 Genomics UK (COG-UK) Consortium	The Lighthouse Lab in Milton Keynes and Alex Alderton, Roberto Amato, Sonia Goncalves, Ewan Harrison, David K. Jackson, Ian Johnston, Dominic Kwiatkowski, Cordelia Langford, John Sillitoe on behalf of the Wellcome Sanger Institute COVID-19 Surveillance Team
EPI_ISL_756849, EPI_ISL_756850	Lighthouse Lab in Glasgow	Wellcome Sanger Institute for the COVID-19 Genomics UK (COG-UK) Consortium	Harper VanSteenhouse, Yumi Kasai, David Gray, Carol Clugston, Anna Dominiczak and Alex Alderton, Roberto Amato, Sonia Goncalves, Ewan Harrison, David K. Jackson, Ian Johnston, Dominic Kwiatkowski, Cordelia Langford, John Sillitoe on behalf of the Wellcome Sanger Institute COVID-19 Surveillance Team
EPI_ISL_756851	Lighthouse Lab in Milton Keynes	Wellcome Sanger Institute for the COVID-19 Genomics UK (COG-UK) Consortium	The Lighthouse Lab in Milton Keynes and Alex Alderton, Roberto Amato, Sonia Goncalves, Ewan Harrison, David K. Jackson, Ian Johnston, Dominic Kwiatkowski, Cordelia Langford, John Sillitoe on behalf of the Wellcome Sanger Institute COVID-19 Surveillance Team
EPI_ISL_756852	Lighthouse Lab in Glasgow	Wellcome Sanger Institute for the COVID-19 Genomics UK (COG-UK) Consortium	Harper VanSteenhouse, Yumi Kasai, David Gray, Carol Clugston, Anna Dominiczak and Alex Alderton, Roberto Amato, Sonia Goncalves, Ewan Harrison, David K. Jackson, Ian Johnston, Dominic Kwiatkowski, Cordelia Langford, John Sillitoe on behalf of the Wellcome Sanger Institute COVID-19 Surveillance Team
EPI_ISL_756853, EPI_ISL_756854, EPI_ISL_756855, EPI_ISL_756856, EPI_ISL_756857, EPI_ISL_756858, EPI_ISL_756859, EPI_ISL_756860	Lighthouse Lab in Milton Keynes	Wellcome Sanger Institute for the COVID-19 Genomics UK (COG-UK) Consortium	The Lighthouse Lab in Milton Keynes and Alex Alderton, Roberto Amato, Sonia Goncalves, Ewan Harrison, David K. Jackson, Ian Johnston, Dominic Kwiatkowski, Cordelia Langford, John Sillitoe on behalf of the Wellcome Sanger Institute COVID-19 Surveillance Team
EPI_ISL_756861	Lighthouse Lab in Glasgow	Wellcome Sanger Institute for the COVID-19 Genomics UK (COG-UK) Consortium	Harper VanSteenhouse, Yumi Kasai, David Gray, Carol Clugston, Anna Dominiczak and Alex Alderton, Roberto Amato, Sonia Goncalves, Ewan Harrison, David K. Jackson, Ian Johnston, Dominic Kwiatkowski, Cordelia Langford, John Sillitoe on behalf of the Wellcome Sanger Institute COVID-19 Surveillance Team
EPI_ISL_756862, EPI_ISL_756863, EPI_ISL_756864, EPI_ISL_756865, EPI_ISL_756866	Lighthouse Lab in Milton Keynes	Wellcome Sanger Institute for the COVID-19 Genomics UK (COG-UK) Consortium	The Lighthouse Lab in Milton Keynes and Alex Alderton, Roberto Amato, Sonia Goncalves, Ewan Harrison, David K. Jackson, Ian Johnston, Dominic Kwiatkowski, Cordelia Langford, John Sillitoe on behalf of the Wellcome Sanger Institute COVID-19 Surveillance Team
EPI_ISL_756867	Lighthouse Lab in Glasgow	Wellcome Sanger Institute for the COVID-19 Genomics UK (COG-UK) Consortium	Harper VanSteenhouse, Yumi Kasai, David Gray, Carol Clugston, Anna Dominiczak and Alex Alderton, Roberto Amato, Sonia Goncalves, Ewan Harrison, David K. Jackson, Ian Johnston, Dominic Kwiatkowski, Cordelia Langford, John Sillitoe on behalf of the Wellcome Sanger Institute COVID-19 Surveillance Team
EPI_ISL_756868, EPI_ISL_756869, EPI_ISL_756870, EPI_ISL_756871, EPI_ISL_756872, EPI_ISL_756873, EPI_ISL_756874, EPI_ISL_756875, EPI_ISL_756876	Lighthouse Lab in Milton Keynes	Wellcome Sanger Institute for the COVID-19 Genomics UK (COG-UK) Consortium	The Lighthouse Lab in Milton Keynes and Alex Alderton, Roberto Amato, Sonia Goncalves, Ewan Harrison, David K. Jackson, Ian Johnston, Dominic Kwiatkowski, Cordelia Langford, John Sillitoe on behalf of the Wellcome Sanger Institute COVID-19 Surveillance Team
EPI_ISL_756877, EPI_ISL_756878, EPI_ISL_756879	Lighthouse Lab in Glasgow	Wellcome Sanger Institute for the COVID-19 Genomics UK (COG-UK) Consortium	Harper VanSteenhouse, Yumi Kasai, David Gray, Carol Clugston, Anna Dominiczak and Alex Alderton, Roberto Amato, Sonia Goncalves, Ewan Harrison, David K. Jackson, Ian Johnston, Dominic Kwiatkowski, Cordelia Langford, John Sillitoe on behalf of the Wellcome Sanger Institute COVID-19 Surveillance Team
EPI_ISL_756880	Lighthouse Lab in Milton Keynes	Wellcome Sanger	The Lighthouse Lab in Milton Keynes and Alex Alderton, Roberto Amato, Sonia Goncalves, Ewan Harrison, David K. Jackson, Ian Johnston, Dominic Kwiatkowski, Cordelia Langford, John Sillitoe on behalf of the Wellcome Sanger Institute COVID-19

		Institute for the COVID-19 Genomics UK (COG-UK) Consortium	Surveillance Team
EPI_ISL_756881	Lighthouse Lab in Glasgow	Wellcome Sanger Institute for the COVID-19 Genomics UK (COG-UK) Consortium	Harper VanSteenhouse, Yumi Kasai, David Gray, Carol Clugston, Anna Dominiczak and Alex Alderton, Roberto Amato, Sonia Goncalves, Ewan Harrison, David K. Jackson, Ian Johnston, Dominic Kwiatkowski, Cordelia Langford, John Sillitoe on behalf of the Wellcome Sanger Institute COVID-19 Surveillance Team
EPI_ISL_756882, EPI_ISL_756883, EPI_ISL_756884	Lighthouse Lab in Milton Keynes	Wellcome Sanger Institute for the COVID-19 Genomics UK (COG-UK) Consortium	The Lighthouse Lab in Milton Keynes and Alex Alderton, Roberto Amato, Sonia Goncalves, Ewan Harrison, David K. Jackson, Ian Johnston, Dominic Kwiatkowski, Cordelia Langford, John Sillitoe on behalf of the Wellcome Sanger Institute COVID-19 Surveillance Team
EPI_ISL_756885, EPI_ISL_756886	Lighthouse Lab in Glasgow	Wellcome Sanger Institute for the COVID-19 Genomics UK (COG-UK) Consortium	Harper VanSteenhouse, Yumi Kasai, David Gray, Carol Clugston, Anna Dominiczak and Alex Alderton, Roberto Amato, Sonia Goncalves, Ewan Harrison, David K. Jackson, Ian Johnston, Dominic Kwiatkowski, Cordelia Langford, John Sillitoe on behalf of the Wellcome Sanger Institute COVID-19 Surveillance Team
EPI_ISL_756887	Lighthouse Lab in Milton Keynes	Wellcome Sanger Institute for the COVID-19 Genomics UK (COG-UK) Consortium	The Lighthouse Lab in Milton Keynes and Alex Alderton, Roberto Amato, Sonia Goncalves, Ewan Harrison, David K. Jackson, Ian Johnston, Dominic Kwiatkowski, Cordelia Langford, John Sillitoe on behalf of the Wellcome Sanger Institute COVID-19 Surveillance Team
EPI_ISL_756888, EPI_ISL_756889	Lighthouse Lab in Glasgow	Wellcome Sanger Institute for the COVID-19 Genomics UK (COG-UK) Consortium	Harper VanSteenhouse, Yumi Kasai, David Gray, Carol Clugston, Anna Dominiczak and Alex Alderton, Roberto Amato, Sonia Goncalves, Ewan Harrison, David K. Jackson, Ian Johnston, Dominic Kwiatkowski, Cordelia Langford, John Sillitoe on behalf of the Wellcome Sanger Institute COVID-19 Surveillance Team
EPI_ISL_756890, EPI_ISL_756891, EPI_ISL_756892	Lighthouse Lab in Milton Keynes	Wellcome Sanger Institute for the COVID-19 Genomics UK (COG-UK) Consortium	The Lighthouse Lab in Milton Keynes and Alex Alderton, Roberto Amato, Sonia Goncalves, Ewan Harrison, David K. Jackson, Ian Johnston, Dominic Kwiatkowski, Cordelia Langford, John Sillitoe on behalf of the Wellcome Sanger Institute COVID-19 Surveillance Team
EPI_ISL_756893, EPI_ISL_756894, EPI_ISL_756895	Lighthouse Lab in Glasgow	Wellcome Sanger Institute for the COVID-19 Genomics UK (COG-UK) Consortium	Harper VanSteenhouse, Yumi Kasai, David Gray, Carol Clugston, Anna Dominiczak and Alex Alderton, Roberto Amato, Sonia Goncalves, Ewan Harrison, David K. Jackson, Ian Johnston, Dominic Kwiatkowski, Cordelia Langford, John Sillitoe on behalf of the Wellcome Sanger Institute COVID-19 Surveillance Team
EPI_ISL_756896	Lighthouse Lab in Milton Keynes	Wellcome Sanger Institute for the COVID-19 Genomics UK (COG-UK) Consortium	The Lighthouse Lab in Milton Keynes and Alex Alderton, Roberto Amato, Sonia Goncalves, Ewan Harrison, David K. Jackson, Ian Johnston, Dominic Kwiatkowski, Cordelia Langford, John Sillitoe on behalf of the Wellcome Sanger Institute COVID-19 Surveillance Team
EPI_ISL_756897	Lighthouse Lab in Glasgow	Wellcome Sanger Institute for the COVID-19 Genomics UK (COG-UK) Consortium	Harper VanSteenhouse, Yumi Kasai, David Gray, Carol Clugston, Anna Dominiczak and Alex Alderton, Roberto Amato, Sonia Goncalves, Ewan Harrison, David K. Jackson, Ian Johnston, Dominic Kwiatkowski, Cordelia Langford, John Sillitoe on behalf of the Wellcome Sanger Institute COVID-19 Surveillance Team
EPI_ISL_756898, EPI_ISL_756899	Lighthouse Lab in Milton Keynes	Wellcome Sanger Institute for the COVID-19 Genomics UK (COG-UK) Consortium	The Lighthouse Lab in Milton Keynes and Alex Alderton, Roberto Amato, Sonia Goncalves, Ewan Harrison, David K. Jackson, Ian Johnston, Dominic Kwiatkowski, Cordelia Langford, John Sillitoe on behalf of the Wellcome Sanger Institute COVID-19 Surveillance Team
EPI_ISL_756900	Lighthouse Lab in Glasgow	Wellcome Sanger Institute for the COVID-19 Genomics UK (COG-UK) Consortium	Harper VanSteenhouse, Yumi Kasai, David Gray, Carol Clugston, Anna Dominiczak and Alex Alderton, Roberto Amato, Sonia Goncalves, Ewan Harrison, David K. Jackson, Ian Johnston, Dominic Kwiatkowski, Cordelia Langford, John Sillitoe on behalf of the Wellcome Sanger Institute COVID-19 Surveillance Team
EPI_ISL_756901, EPI_ISL_756902, EPI_ISL_756903, EPI_ISL_756904, EPI_ISL_756905, EPI_ISL_756906, EPI_ISL_756907, EPI_ISL_756908	Lighthouse Lab in Milton Keynes	Wellcome Sanger Institute for the COVID-19 Genomics UK (COG-UK) Consortium	The Lighthouse Lab in Milton Keynes and Alex Alderton, Roberto Amato, Sonia Goncalves, Ewan Harrison, David K. Jackson, Ian Johnston, Dominic Kwiatkowski, Cordelia Langford, John Sillitoe on behalf of the Wellcome Sanger Institute COVID-19 Surveillance Team
EPI_ISL_756909, EPI_ISL_756910	Lighthouse Lab in Glasgow	Wellcome Sanger Institute for the COVID-19 Genomics UK (COG-UK) Consortium	Harper VanSteenhouse, Yumi Kasai, David Gray, Carol Clugston, Anna Dominiczak and Alex Alderton, Roberto Amato, Sonia Goncalves, Ewan Harrison, David K. Jackson, Ian Johnston, Dominic Kwiatkowski, Cordelia Langford, John Sillitoe on behalf of the Wellcome Sanger Institute COVID-19 Surveillance Team
EPI_ISL_756911	Lighthouse Lab in Milton Keynes	Wellcome Sanger Institute for the COVID-19 Genomics UK (COG-UK) Consortium	The Lighthouse Lab in Milton Keynes and Alex Alderton, Roberto Amato, Sonia Goncalves, Ewan Harrison, David K. Jackson, Ian Johnston, Dominic Kwiatkowski, Cordelia Langford, John Sillitoe on behalf of the Wellcome Sanger Institute COVID-19 Surveillance Team
EPI_ISL_756912	Lighthouse Lab in Glasgow	Wellcome Sanger Institute for the COVID-19 Genomics UK (COG-UK) Consortium	Harper VanSteenhouse, Yumi Kasai, David Gray, Carol Clugston, Anna Dominiczak and Alex Alderton, Roberto Amato, Sonia Goncalves, Ewan Harrison, David K. Jackson, Ian Johnston, Dominic Kwiatkowski, Cordelia Langford, John Sillitoe on behalf of the Wellcome Sanger Institute COVID-19 Surveillance Team
EPI_ISL_756913, EPI_ISL_756914, EPI_ISL_756915, EPI_ISL_756916, EPI_ISL_756917	Lighthouse Lab in Milton Keynes	Wellcome Sanger Institute for the COVID-19 Genomics UK (COG-UK) Consortium	The Lighthouse Lab in Milton Keynes and Alex Alderton, Roberto Amato, Sonia Goncalves, Ewan Harrison, David K. Jackson, Ian Johnston, Dominic Kwiatkowski, Cordelia Langford, John Sillitoe on behalf of the Wellcome Sanger Institute COVID-19 Surveillance Team
EPI_ISL_756918	Lighthouse Lab in Glasgow	Wellcome Sanger Institute for the COVID-19 Genomics UK (COG-UK) Consortium	Harper VanSteenhouse, Yumi Kasai, David Gray, Carol Clugston, Anna Dominiczak and Alex Alderton, Roberto Amato, Sonia Goncalves, Ewan Harrison, David K. Jackson, Ian Johnston, Dominic Kwiatkowski, Cordelia Langford, John Sillitoe on behalf of the Wellcome Sanger Institute COVID-19 Surveillance Team
EPI_ISL_756919	Lighthouse Lab in Milton Keynes	Wellcome Sanger Institute for the	The Lighthouse Lab in Milton Keynes and Alex Alderton, Roberto Amato, Sonia Goncalves, Ewan Harrison, David K. Jackson, Ian Johnston, Dominic Kwiatkowski, Cordelia Langford, John Sillitoe on behalf of the Wellcome Sanger Institute COVID-19 Surveillance Team

		COVID-19 Genomics UK (COG-UK) Consortium	
EPI_ISL_756920	Lighthouse Lab in Glasgow	Wellcome Sanger Institute for the COVID-19 Genomics UK (COG-UK) Consortium	Harper VanSteenhouse, Yumi Kasai, David Gray, Carol Clugston, Anna Dominiczak and Alex Alderton, Roberto Amato, Sonia Goncalves, Ewan Harrison, David K. Jackson, Ian Johnston, Dominic Kwiatkowski, Cordelia Langford, John Sillitoe on behalf of the Wellcome Sanger Institute COVID-19 Surveillance Team
EPI_ISL_756921, EPI_ISL_756922	Lighthouse Lab in Milton Keynes	Wellcome Sanger Institute for the COVID-19 Genomics UK (COG-UK) Consortium	The Lighthouse Lab in Milton Keynes and Alex Alderton, Roberto Amato, Sonia Goncalves, Ewan Harrison, David K. Jackson, Ian Johnston, Dominic Kwiatkowski, Cordelia Langford, John Sillitoe on behalf of the Wellcome Sanger Institute COVID-19 Surveillance Team
EPI_ISL_756923	Lighthouse Lab in Glasgow	Wellcome Sanger Institute for the COVID-19 Genomics UK (COG-UK) Consortium	Harper VanSteenhouse, Yumi Kasai, David Gray, Carol Clugston, Anna Dominiczak and Alex Alderton, Roberto Amato, Sonia Goncalves, Ewan Harrison, David K. Jackson, Ian Johnston, Dominic Kwiatkowski, Cordelia Langford, John Sillitoe on behalf of the Wellcome Sanger Institute COVID-19 Surveillance Team
EPI_ISL_756924, EPI_ISL_756925, EPI_ISL_756926, EPI_ISL_756927	Lighthouse Lab in Milton Keynes	Wellcome Sanger Institute for the COVID-19 Genomics UK (COG-UK) Consortium	The Lighthouse Lab in Milton Keynes and Alex Alderton, Roberto Amato, Sonia Goncalves, Ewan Harrison, David K. Jackson, Ian Johnston, Dominic Kwiatkowski, Cordelia Langford, John Sillitoe on behalf of the Wellcome Sanger Institute COVID-19 Surveillance Team
EPI_ISL_756928	Lighthouse Lab in Glasgow	Wellcome Sanger Institute for the COVID-19 Genomics UK (COG-UK) Consortium	Harper VanSteenhouse, Yumi Kasai, David Gray, Carol Clugston, Anna Dominiczak and Alex Alderton, Roberto Amato, Sonia Goncalves, Ewan Harrison, David K. Jackson, Ian Johnston, Dominic Kwiatkowski, Cordelia Langford, John Sillitoe on behalf of the Wellcome Sanger Institute COVID-19 Surveillance Team
EPI_ISL_756929, EPI_ISL_756930	Lighthouse Lab in Milton Keynes	Wellcome Sanger Institute for the COVID-19 Genomics UK (COG-UK) Consortium	The Lighthouse Lab in Milton Keynes and Alex Alderton, Roberto Amato, Sonia Goncalves, Ewan Harrison, David K. Jackson, Ian Johnston, Dominic Kwiatkowski, Cordelia Langford, John Sillitoe on behalf of the Wellcome Sanger Institute COVID-19 Surveillance Team
EPI_ISL_756931	Lighthouse Lab in Glasgow	Wellcome Sanger Institute for the COVID-19 Genomics UK (COG-UK) Consortium	Harper VanSteenhouse, Yumi Kasai, David Gray, Carol Clugston, Anna Dominiczak and Alex Alderton, Roberto Amato, Sonia Goncalves, Ewan Harrison, David K. Jackson, Ian Johnston, Dominic Kwiatkowski, Cordelia Langford, John Sillitoe on behalf of the Wellcome Sanger Institute COVID-19 Surveillance Team
EPI_ISL_756932, EPI_ISL_756934	Lighthouse Lab in Milton Keynes	Wellcome Sanger Institute for the COVID-19 Genomics UK (COG-UK) Consortium	The Lighthouse Lab in Milton Keynes and Alex Alderton, Roberto Amato, Sonia Goncalves, Ewan Harrison, David K. Jackson, Ian Johnston, Dominic Kwiatkowski, Cordelia Langford, John Sillitoe on behalf of the Wellcome Sanger Institute COVID-19 Surveillance Team
EPI_ISL_756935	Lighthouse Lab in Glasgow	Wellcome Sanger Institute for the COVID-19 Genomics UK (COG-UK) Consortium	Harper VanSteenhouse, Yumi Kasai, David Gray, Carol Clugston, Anna Dominiczak and Alex Alderton, Roberto Amato, Sonia Goncalves, Ewan Harrison, David K. Jackson, Ian Johnston, Dominic Kwiatkowski, Cordelia Langford, John Sillitoe on behalf of the Wellcome Sanger Institute COVID-19 Surveillance Team
EPI_ISL_756936, EPI_ISL_756937, EPI_ISL_756938, EPI_ISL_756939	Lighthouse Lab in Milton Keynes	Wellcome Sanger Institute for the COVID-19 Genomics UK (COG-UK) Consortium	The Lighthouse Lab in Milton Keynes and Alex Alderton, Roberto Amato, Sonia Goncalves, Ewan Harrison, David K. Jackson, Ian Johnston, Dominic Kwiatkowski, Cordelia Langford, John Sillitoe on behalf of the Wellcome Sanger Institute COVID-19 Surveillance Team
EPI_ISL_756940	Lighthouse Lab in Glasgow	Wellcome Sanger Institute for the COVID-19 Genomics UK (COG-UK) Consortium	Harper VanSteenhouse, Yumi Kasai, David Gray, Carol Clugston, Anna Dominiczak and Alex Alderton, Roberto Amato, Sonia Goncalves, Ewan Harrison, David K. Jackson, Ian Johnston, Dominic Kwiatkowski, Cordelia Langford, John Sillitoe on behalf of the Wellcome Sanger Institute COVID-19 Surveillance Team
EPI_ISL_756941, EPI_ISL_756942, EPI_ISL_756943, EPI_ISL_756944, EPI_ISL_756945, EPI_ISL_756946	Lighthouse Lab in Milton Keynes	Wellcome Sanger Institute for the COVID-19 Genomics UK (COG-UK) Consortium	The Lighthouse Lab in Milton Keynes and Alex Alderton, Roberto Amato, Sonia Goncalves, Ewan Harrison, David K. Jackson, Ian Johnston, Dominic Kwiatkowski, Cordelia Langford, John Sillitoe on behalf of the Wellcome Sanger Institute COVID-19 Surveillance Team
EPI_ISL_756947	Lighthouse Lab in Glasgow	Wellcome Sanger Institute for the COVID-19 Genomics UK (COG-UK) Consortium	Harper VanSteenhouse, Yumi Kasai, David Gray, Carol Clugston, Anna Dominiczak and Alex Alderton, Roberto Amato, Sonia Goncalves, Ewan Harrison, David K. Jackson, Ian Johnston, Dominic Kwiatkowski, Cordelia Langford, John Sillitoe on behalf of the Wellcome Sanger Institute COVID-19 Surveillance Team
EPI_ISL_756948, EPI_ISL_756949, EPI_ISL_756950	Lighthouse Lab in Milton Keynes	Wellcome Sanger Institute for the COVID-19 Genomics UK (COG-UK) Consortium	The Lighthouse Lab in Milton Keynes and Alex Alderton, Roberto Amato, Sonia Goncalves, Ewan Harrison, David K. Jackson, Ian Johnston, Dominic Kwiatkowski, Cordelia Langford, John Sillitoe on behalf of the Wellcome Sanger Institute COVID-19 Surveillance Team
EPI_ISL_756951, EPI_ISL_756952, EPI_ISL_756953	Lighthouse Lab in Glasgow	Wellcome Sanger Institute for the COVID-19 Genomics UK (COG-UK) Consortium	Harper VanSteenhouse, Yumi Kasai, David Gray, Carol Clugston, Anna Dominiczak and Alex Alderton, Roberto Amato, Sonia Goncalves, Ewan Harrison, David K. Jackson, Ian Johnston, Dominic Kwiatkowski, Cordelia Langford, John Sillitoe on behalf of the Wellcome Sanger Institute COVID-19 Surveillance Team
EPI_ISL_756954	Lighthouse Lab in Milton Keynes	Wellcome Sanger Institute for the COVID-19 Genomics UK (COG-UK) Consortium	The Lighthouse Lab in Milton Keynes and Alex Alderton, Roberto Amato, Sonia Goncalves, Ewan Harrison, David K. Jackson, Ian Johnston, Dominic Kwiatkowski, Cordelia Langford, John Sillitoe on behalf of the Wellcome Sanger Institute COVID-19 Surveillance Team
EPI_ISL_756955	Lighthouse Lab in Glasgow	Wellcome Sanger Institute for the COVID-19 Genomics UK (COG-UK) Consortium	Harper VanSteenhouse, Yumi Kasai, David Gray, Carol Clugston, Anna Dominiczak and Alex Alderton, Roberto Amato, Sonia Goncalves, Ewan Harrison, David K. Jackson, Ian Johnston, Dominic Kwiatkowski, Cordelia Langford, John Sillitoe on behalf of the Wellcome Sanger Institute COVID-19 Surveillance Team
EPI_ISL_756956, EPI_ISL_756957, EPI_ISL_756958	Lighthouse Lab in Milton Keynes	Wellcome Sanger Institute for the COVID-19 Genomics UK (COG-UK) Consortium	The Lighthouse Lab in Milton Keynes and Alex Alderton, Roberto Amato, Sonia Goncalves, Ewan Harrison, David K. Jackson, Ian Johnston, Dominic Kwiatkowski, Cordelia Langford, John Sillitoe on behalf of the Wellcome Sanger Institute COVID-19 Surveillance Team
EPI_ISL_756959	Lighthouse Lab in Glasgow	Wellcome Sanger Institute for the	Harper VanSteenhouse, Yumi Kasai, David Gray, Carol Clugston, Anna Dominiczak and Alex Alderton, Roberto Amato, Sonia Goncalves, Ewan Harrison, David K. Jackson, Ian Johnston, Dominic Kwiatkowski, Cordelia Langford, John Sillitoe on behalf of the Wellcome Sanger Institute COVID-19 Surveillance Team

		COVID-19 Genomics UK (COG-UK) Consortium	
EPI_ISL_756961, EPI_ISL_756962, EPI_ISL_756963, EPI_ISL_756964, EPI_ISL_756965, EPI_ISL_756966, EPI_ISL_756967, EPI_ISL_756968	Lighthouse Lab in Milton Keynes	Wellcome Sanger Institute for the COVID-19 Genomics UK (COG-UK) Consortium	The Lighthouse Lab in Milton Keynes and Alex Alderton, Roberto Amato, Sonia Goncalves, Ewan Harrison, David K. Jackson, Ian Johnston, Dominic Kwiatkowski, Cordelia Langford, John Sillitoe on behalf of the Wellcome Sanger Institute COVID-19 Surveillance Team
EPI_ISL_756969, EPI_ISL_756970, EPI_ISL_756971	Lighthouse Lab in Glasgow	Wellcome Sanger Institute for the COVID-19 Genomics UK (COG-UK) Consortium	Harper VanSteenhouse, Yumi Kasai, David Gray, Carol Clugston, Anna Dominiczak and Alex Alderton, Roberto Amato, Sonia Goncalves, Ewan Harrison, David K. Jackson, Ian Johnston, Dominic Kwiatkowski, Cordelia Langford, John Sillitoe on behalf of the Wellcome Sanger Institute COVID-19 Surveillance Team
EPI_ISL_756972	Lighthouse Lab in Milton Keynes	Wellcome Sanger Institute for the COVID-19 Genomics UK (COG-UK) Consortium	The Lighthouse Lab in Milton Keynes and Alex Alderton, Roberto Amato, Sonia Goncalves, Ewan Harrison, David K. Jackson, Ian Johnston, Dominic Kwiatkowski, Cordelia Langford, John Sillitoe on behalf of the Wellcome Sanger Institute COVID-19 Surveillance Team
EPI_ISL_756973	Lighthouse Lab in Glasgow	Wellcome Sanger Institute for the COVID-19 Genomics UK (COG-UK) Consortium	Harper VanSteenhouse, Yumi Kasai, David Gray, Carol Clugston, Anna Dominiczak and Alex Alderton, Roberto Amato, Sonia Goncalves, Ewan Harrison, David K. Jackson, Ian Johnston, Dominic Kwiatkowski, Cordelia Langford, John Sillitoe on behalf of the Wellcome Sanger Institute COVID-19 Surveillance Team
EPI_ISL_756974, EPI_ISL_756975, EPI_ISL_756976	Lighthouse Lab in Milton Keynes	Wellcome Sanger Institute for the COVID-19 Genomics UK (COG-UK) Consortium	The Lighthouse Lab in Milton Keynes and Alex Alderton, Roberto Amato, Sonia Goncalves, Ewan Harrison, David K. Jackson, Ian Johnston, Dominic Kwiatkowski, Cordelia Langford, John Sillitoe on behalf of the Wellcome Sanger Institute COVID-19 Surveillance Team
EPI_ISL_756977	Lighthouse Lab in Glasgow	Wellcome Sanger Institute for the COVID-19 Genomics UK (COG-UK) Consortium	Harper VanSteenhouse, Yumi Kasai, David Gray, Carol Clugston, Anna Dominiczak and Alex Alderton, Roberto Amato, Sonia Goncalves, Ewan Harrison, David K. Jackson, Ian Johnston, Dominic Kwiatkowski, Cordelia Langford, John Sillitoe on behalf of the Wellcome Sanger Institute COVID-19 Surveillance Team
EPI_ISL_756978, EPI_ISL_756979, EPI_ISL_756980, EPI_ISL_756981	Lighthouse Lab in Milton Keynes	Wellcome Sanger Institute for the COVID-19 Genomics UK (COG-UK) Consortium	The Lighthouse Lab in Milton Keynes and Alex Alderton, Roberto Amato, Sonia Goncalves, Ewan Harrison, David K. Jackson, Ian Johnston, Dominic Kwiatkowski, Cordelia Langford, John Sillitoe on behalf of the Wellcome Sanger Institute COVID-19 Surveillance Team
EPI_ISL_756982	Lighthouse Lab in Glasgow	Wellcome Sanger Institute for the COVID-19 Genomics UK (COG-UK) Consortium	Harper VanSteenhouse, Yumi Kasai, David Gray, Carol Clugston, Anna Dominiczak and Alex Alderton, Roberto Amato, Sonia Goncalves, Ewan Harrison, David K. Jackson, Ian Johnston, Dominic Kwiatkowski, Cordelia Langford, John Sillitoe on behalf of the Wellcome Sanger Institute COVID-19 Surveillance Team
EPI_ISL_756983, EPI_ISL_756984, EPI_ISL_756985, EPI_ISL_756986, EPI_ISL_756987, EPI_ISL_756988, EPI_ISL_756990, EPI_ISL_756991, EPI_ISL_756992, EPI_ISL_756993, EPI_ISL_756994, EPI_ISL_756995, EPI_ISL_756996, EPI_ISL_756997			
see above	Lighthouse Lab in Milton Keynes	Wellcome Sanger Institute for the COVID-19 Genomics UK (COG-UK) Consortium	The Lighthouse Lab in Milton Keynes and Alex Alderton, Roberto Amato, Sonia Goncalves, Ewan Harrison, David K. Jackson, Ian Johnston, Dominic Kwiatkowski, Cordelia Langford, John Sillitoe on behalf of the Wellcome Sanger Institute COVID-19 Surveillance Team
EPI_ISL_756998	Lighthouse Lab in Glasgow	Wellcome Sanger Institute for the COVID-19 Genomics UK (COG-UK) Consortium	Harper VanSteenhouse, Yumi Kasai, David Gray, Carol Clugston, Anna Dominiczak and Alex Alderton, Roberto Amato, Sonia Goncalves, Ewan Harrison, David K. Jackson, Ian Johnston, Dominic Kwiatkowski, Cordelia Langford, John Sillitoe on behalf of the Wellcome Sanger Institute COVID-19 Surveillance Team
EPI_ISL_756999, EPI_ISL_757000, EPI_ISL_757001, EPI_ISL_757002, EPI_ISL_757003, EPI_ISL_757004	Lighthouse Lab in Milton Keynes	Wellcome Sanger Institute for the COVID-19 Genomics UK (COG-UK) Consortium	The Lighthouse Lab in Milton Keynes and Alex Alderton, Roberto Amato, Sonia Goncalves, Ewan Harrison, David K. Jackson, Ian Johnston, Dominic Kwiatkowski, Cordelia Langford, John Sillitoe on behalf of the Wellcome Sanger Institute COVID-19 Surveillance Team
EPI_ISL_757005	Lighthouse Lab in Glasgow	Wellcome Sanger Institute for the COVID-19 Genomics UK (COG-UK) Consortium	Harper VanSteenhouse, Yumi Kasai, David Gray, Carol Clugston, Anna Dominiczak and Alex Alderton, Roberto Amato, Sonia Goncalves, Ewan Harrison, David K. Jackson, Ian Johnston, Dominic Kwiatkowski, Cordelia Langford, John Sillitoe on behalf of the Wellcome Sanger Institute COVID-19 Surveillance Team
EPI_ISL_757006	Lighthouse Lab in Milton Keynes	Wellcome Sanger Institute for the COVID-19 Genomics UK (COG-UK) Consortium	The Lighthouse Lab in Milton Keynes and Alex Alderton, Roberto Amato, Sonia Goncalves, Ewan Harrison, David K. Jackson, Ian Johnston, Dominic Kwiatkowski, Cordelia Langford, John Sillitoe on behalf of the Wellcome Sanger Institute COVID-19 Surveillance Team
EPI_ISL_757007, EPI_ISL_757008, EPI_ISL_757009	Lighthouse Lab in Glasgow	Wellcome Sanger Institute for the COVID-19 Genomics UK (COG-UK) Consortium	Harper VanSteenhouse, Yumi Kasai, David Gray, Carol Clugston, Anna Dominiczak and Alex Alderton, Roberto Amato, Sonia Goncalves, Ewan Harrison, David K. Jackson, Ian Johnston, Dominic Kwiatkowski, Cordelia Langford, John Sillitoe on behalf of the Wellcome Sanger Institute COVID-19 Surveillance Team
EPI_ISL_757010	Lighthouse Lab in Milton Keynes	Wellcome Sanger Institute for the COVID-19 Genomics UK (COG-UK) Consortium	The Lighthouse Lab in Milton Keynes and Alex Alderton, Roberto Amato, Sonia Goncalves, Ewan Harrison, David K. Jackson, Ian Johnston, Dominic Kwiatkowski, Cordelia Langford, John Sillitoe on behalf of the Wellcome Sanger Institute COVID-19 Surveillance Team
EPI_ISL_757011, EPI_ISL_757012	Lighthouse Lab in Glasgow	Wellcome Sanger Institute for the COVID-19 Genomics UK (COG-UK) Consortium	Harper VanSteenhouse, Yumi Kasai, David Gray, Carol Clugston, Anna Dominiczak and Alex Alderton, Roberto Amato, Sonia Goncalves, Ewan Harrison, David K. Jackson, Ian Johnston, Dominic Kwiatkowski, Cordelia Langford, John Sillitoe on behalf of the Wellcome Sanger Institute COVID-19 Surveillance Team
EPI_ISL_757013, EPI_ISL_757014, EPI_ISL_757015, EPI_ISL_757016	Lighthouse Lab in Milton Keynes	Wellcome Sanger Institute for the COVID-19 Genomics UK (COG-UK) Consortium	The Lighthouse Lab in Milton Keynes and Alex Alderton, Roberto Amato, Sonia Goncalves, Ewan Harrison, David K. Jackson, Ian Johnston, Dominic Kwiatkowski, Cordelia Langford, John Sillitoe on behalf of the Wellcome Sanger Institute COVID-19 Surveillance Team
EPI_ISL_757017,	Lighthouse Lab in Glasgow	Wellcome Sanger	Harper VanSteenhouse, Yumi Kasai, David Gray, Carol Clugston, Anna Dominiczak and Alex Alderton, Roberto Amato, Sonia Goncalves, Ewan Harrison, David K. Jackson, Ian Johnston, Dominic Kwiatkowski, Cordelia Langford, John Sillitoe on

EPI_ISL_757018		Institute for the COVID-19 Genomics UK (COG-UK) Consortium	behalf of the Wellcome Sanger Institute COVID-19 Surveillance Team
EPI_ISL_757019, EPI_ISL_757020	Lighthouse Lab in Milton Keynes	Wellcome Sanger Institute for the COVID-19 Genomics UK (COG-UK) Consortium	The Lighthouse Lab in Milton Keynes and Alex Alderton, Roberto Amato, Sonia Goncalves, Ewan Harrison, David K. Jackson, Ian Johnston, Dominic Kwiatkowski, Cordelia Langford, John Sillitoe on behalf of the Wellcome Sanger Institute COVID-19 Surveillance Team
EPI_ISL_757021	Lighthouse Lab in Glasgow	Wellcome Sanger Institute for the COVID-19 Genomics UK (COG-UK) Consortium	Harper VanSteenhouse, Yumi Kasai, David Gray, Carol Clugston, Anna Dominiczak and Alex Alderton, Roberto Amato, Sonia Goncalves, Ewan Harrison, David K. Jackson, Ian Johnston, Dominic Kwiatkowski, Cordelia Langford, John Sillitoe on behalf of the Wellcome Sanger Institute COVID-19 Surveillance Team
EPI_ISL_757022, EPI_ISL_757023, EPI_ISL_757024, EPI_ISL_757025, EPI_ISL_757026	Lighthouse Lab in Milton Keynes	Wellcome Sanger Institute for the COVID-19 Genomics UK (COG-UK) Consortium	The Lighthouse Lab in Milton Keynes and Alex Alderton, Roberto Amato, Sonia Goncalves, Ewan Harrison, David K. Jackson, Ian Johnston, Dominic Kwiatkowski, Cordelia Langford, John Sillitoe on behalf of the Wellcome Sanger Institute COVID-19 Surveillance Team
EPI_ISL_757027, EPI_ISL_757028	Lighthouse Lab in Glasgow	Wellcome Sanger Institute for the COVID-19 Genomics UK (COG-UK) Consortium	Harper VanSteenhouse, Yumi Kasai, David Gray, Carol Clugston, Anna Dominiczak and Alex Alderton, Roberto Amato, Sonia Goncalves, Ewan Harrison, David K. Jackson, Ian Johnston, Dominic Kwiatkowski, Cordelia Langford, John Sillitoe on behalf of the Wellcome Sanger Institute COVID-19 Surveillance Team
EPI_ISL_757029, EPI_ISL_757030, EPI_ISL_757031	Lighthouse Lab in Milton Keynes	Wellcome Sanger Institute for the COVID-19 Genomics UK (COG-UK) Consortium	The Lighthouse Lab in Milton Keynes and Alex Alderton, Roberto Amato, Sonia Goncalves, Ewan Harrison, David K. Jackson, Ian Johnston, Dominic Kwiatkowski, Cordelia Langford, John Sillitoe on behalf of the Wellcome Sanger Institute COVID-19 Surveillance Team
EPI_ISL_757032, EPI_ISL_757034, EPI_ISL_757035, EPI_ISL_757036, EPI_ISL_757037, EPI_ISL_757038, EPI_ISL_757040, EPI_ISL_757041, EPI_ISL_757042, EPI_ISL_757043, EPI_ISL_757044, EPI_ISL_757045, EPI_ISL_757046, EPI_ISL_757047, EPI_ISL_757048, EPI_ISL_757049, EPI_ISL_757050, EPI_ISL_757051, EPI_ISL_757052, EPI_ISL_757053, EPI_ISL_757054, EPI_ISL_757055, EPI_ISL_757056, EPI_ISL_757057, EPI_ISL_757058, EPI_ISL_757059, EPI_ISL_757060, EPI_ISL_757061, EPI_ISL_757062, EPI_ISL_757063, EPI_ISL_757064, EPI_ISL_757065, EPI_ISL_757066, EPI_ISL_757067, EPI_ISL_757068, EPI_ISL_757069, EPI_ISL_757070, EPI_ISL_757071, EPI_ISL_757072, EPI_ISL_757073, EPI_ISL_757074, EPI_ISL_757075, EPI_ISL_757076, EPI_ISL_757077, EPI_ISL_757079, EPI_ISL_757080, EPI_ISL_757081, EPI_ISL_757082, EPI_ISL_757083, EPI_ISL_757085, EPI_ISL_757086, EPI_ISL_757087, EPI_ISL_757088, EPI_ISL_757089, EPI_ISL_757091, EPI_ISL_757092, EPI_ISL_757093, EPI_ISL_757094, EPI_ISL_757095, EPI_ISL_757096, EPI_ISL_757097, EPI_ISL_757099, EPI_ISL_757100, EPI_ISL_757101, EPI_ISL_757102, EPI_ISL_757103, EPI_ISL_757104, EPI_ISL_757105, EPI_ISL_757106, EPI_ISL_757107, EPI_ISL_757108, EPI_ISL_757109, EPI_ISL_757110, EPI_ISL_757111, EPI_ISL_757112, EPI_ISL_757113, EPI_ISL_757115, EPI_ISL_757116, EPI_ISL_757117, EPI_ISL_757118			
see above	Lighthouse Lab in Glasgow	Wellcome Sanger Institute for the COVID-19 Genomics UK (COG-UK) Consortium	Harper VanSteenhouse, Yumi Kasai, David Gray, Carol Clugston, Anna Dominiczak and Alex Alderton, Roberto Amato, Sonia Goncalves, Ewan Harrison, David K. Jackson, Ian Johnston, Dominic Kwiatkowski, Cordelia Langford, John Sillitoe on behalf of the Wellcome Sanger Institute COVID-19 Surveillance Team
EPI_ISL_757119	Lighthouse Lab in Cambridge	Wellcome Sanger Institute for the COVID-19 Genomics UK (COG-UK) Consortium	Rob Howes, The Lighthouse Lab in Cambridge and Alex Alderton, Roberto Amato, Sonia Goncalves, Ewan Harrison, David K. Jackson, Ian Johnston, Dominic Kwiatkowski, Cordelia Langford, John Sillitoe on behalf of the Wellcome Sanger Institute COVID-19 Surveillance Team
EPI_ISL_757120, EPI_ISL_757121, EPI_ISL_757122, EPI_ISL_757123, EPI_ISL_757124, EPI_ISL_757125, EPI_ISL_757126, EPI_ISL_757127, EPI_ISL_757128, EPI_ISL_757129, EPI_ISL_757130, EPI_ISL_757131, EPI_ISL_757132, EPI_ISL_757133, EPI_ISL_757134, EPI_ISL_757135, EPI_ISL_757136, EPI_ISL_757137, EPI_ISL_757138, EPI_ISL_757139, EPI_ISL_757140, EPI_ISL_757141, EPI_ISL_757142, EPI_ISL_757143, EPI_ISL_757144, EPI_ISL_757145, EPI_ISL_757146, EPI_ISL_757147, EPI_ISL_757148, EPI_ISL_757149, EPI_ISL_757150, EPI_ISL_757151, EPI_ISL_757152, EPI_ISL_757153, EPI_ISL_757154, EPI_ISL_757155, EPI_ISL_757156, EPI_ISL_757157, EPI_ISL_757158, EPI_ISL_757159, EPI_ISL_757160, EPI_ISL_757161, EPI_ISL_757162, EPI_ISL_757163, EPI_ISL_757164, EPI_ISL_757165, EPI_ISL_757166, EPI_ISL_757167, EPI_ISL_757168, EPI_ISL_757169, EPI_ISL_757170, EPI_ISL_757171, EPI_ISL_757172, EPI_ISL_757173, EPI_ISL_757174, EPI_ISL_757175, EPI_ISL_757176, EPI_ISL_757177, EPI_ISL_757178, EPI_ISL_757179, EPI_ISL_757180, EPI_ISL_757181, EPI_ISL_757182, EPI_ISL_757184, EPI_ISL_757185, EPI_ISL_757186, EPI_ISL_757187, EPI_ISL_757188, EPI_ISL_757189, EPI_ISL_757190, EPI_ISL_757191, EPI_ISL_757192, EPI_ISL_757193, EPI_ISL_757194, EPI_ISL_757195, EPI_ISL_757196, EPI_ISL_757197, EPI_ISL_757198, EPI_ISL_757199, EPI_ISL_757200, EPI_ISL_757201, EPI_ISL_757202, EPI_ISL_757203, EPI_ISL_757204, EPI_ISL_757205, EPI_ISL_757206, EPI_ISL_757207, EPI_ISL_757208, EPI_ISL_757209, EPI_ISL_757210, EPI_ISL_757211, EPI_ISL_757212, EPI_ISL_757213, EPI_ISL_757214, EPI_ISL_757216, EPI_ISL_757217, EPI_ISL_757218, EPI_ISL_757219, EPI_ISL_757220, EPI_ISL_757221, EPI_ISL_757222, EPI_ISL_757223, EPI_ISL_757224, EPI_ISL_757225, EPI_ISL_757226, EPI_ISL_757227, EPI_ISL_757228, EPI_ISL_757229, EPI_ISL_757230, EPI_ISL_757231, EPI_ISL_757232, EPI_ISL_757233, EPI_ISL_757234, EPI_ISL_757235, EPI_ISL_757236, EPI_ISL_757237, EPI_ISL_757238, EPI_ISL_757239, EPI_ISL_757240, EPI_ISL_757241, EPI_ISL_757242, EPI_ISL_757243, EPI_ISL_757244, EPI_ISL_757245, EPI_ISL_757246, EPI_ISL_757247, EPI_ISL_757248, EPI_ISL_757249, EPI_ISL_757250, EPI_ISL_757251, EPI_ISL_757252, EPI_ISL_757253, EPI_ISL_757254, EPI_ISL_757255, EPI_ISL_757256, EPI_ISL_757257, EPI_ISL_757258, EPI_ISL_757259, EPI_ISL_757260, EPI_ISL_757261, EPI_ISL_757262, EPI_ISL_757263, EPI_ISL_757264, EPI_ISL_757265, EPI_ISL_757266, EPI_ISL_757267, EPI_ISL_757268, EPI_ISL_757269, EPI_ISL_757270, EPI_ISL_757271, EPI_ISL_757272, EPI_ISL_757273, EPI_ISL_757274, EPI_ISL_757275, EPI_ISL_757276, EPI_ISL_757277, EPI_ISL_757278, EPI_ISL_757279, EPI_ISL_757280, EPI_ISL_757281, EPI_ISL_757282, EPI_ISL_757283, EPI_ISL_757284			
see above	Lighthouse Lab in Glasgow	Wellcome Sanger Institute for the COVID-19 Genomics UK (COG-UK) Consortium	Harper VanSteenhouse, Yumi Kasai, David Gray, Carol Clugston, Anna Dominiczak and Alex Alderton, Roberto Amato, Sonia Goncalves, Ewan Harrison, David K. Jackson, Ian Johnston, Dominic Kwiatkowski, Cordelia Langford, John Sillitoe on behalf of the Wellcome Sanger Institute COVID-19 Surveillance Team
EPI_ISL_757285, EPI_ISL_757286	Department of Virology, Public Health Laboratories Division	Department of Virology, Public Health Laboratories Division	Massab Umair, Aamer Ikram, Muhammad Salman
EPI_ISL_757287	National Institute of Health Research and Development	National Institute of Health Research and Development	Rukminiati,Y;Agustiningsih;Adam,K;Wibowo,HA;Ramadhany,R;Pawestri,HA;Subangkit;Puspa,KD;Nugraha,AA;Ikawati,HD;Pangesti,KNA;Soekarso,T;Susilarini,NK;Hariastuti,NI;Nikmah,UA;Mursinah;Febriyani,A;Herman,R;Susanti,N;Herna;Febriyanti,T;Nurhadi,M;Kurniawati,J;Kipuw,NL;Muna,F;Indalao,IL;Rizki,A;Puspandari,N;Setiawaty,V
EPI_ISL_757288, EPI_ISL_757289, EPI_ISL_757290, EPI_ISL_757291, EPI_ISL_757292, EPI_ISL_757293, EPI_ISL_757294, EPI_ISL_757295, EPI_ISL_757296, EPI_ISL_757297, EPI_ISL_757298, EPI_ISL_757299, EPI_ISL_757300, EPI_ISL_757301, EPI_ISL_757302, EPI_ISL_757303, EPI_ISL_757304, EPI_ISL_757305, EPI_ISL_757306, EPI_ISL_757307, EPI_ISL_757308, EPI_ISL_757309, EPI_ISL_757310, EPI_ISL_757311, EPI_ISL_757312, EPI_ISL_757313, EPI_ISL_757314, EPI_ISL_757315, EPI_ISL_757316, EPI_ISL_757317, EPI_ISL_757318, EPI_ISL_757319, EPI_ISL_757320, EPI_ISL_757321, EPI_ISL_757322, EPI_ISL_757323, EPI_ISL_757324, EPI_ISL_757325, EPI_ISL_757326, EPI_ISL_757327, EPI_ISL_757328, EPI_ISL_757329, EPI_ISL_757330, EPI_ISL_757331, EPI_ISL_757332, EPI_ISL_757333, EPI_ISL_757334, EPI_ISL_757335, EPI_ISL_757336, EPI_ISL_757337, EPI_ISL_757338, EPI_ISL_757339, EPI_ISL_757340, EPI_ISL_757341, EPI_ISL_757342, EPI_ISL_757343, EPI_ISL_757344, EPI_ISL_757345, EPI_ISL_757346, EPI_ISL_757347, EPI_ISL_757348, EPI_ISL_757349, EPI_ISL_757350, EPI_ISL_757351, EPI_ISL_757352, EPI_ISL_757353, EPI_ISL_757354, EPI_ISL_757355, EPI_ISL_757356, EPI_ISL_757357, EPI_ISL_757358, EPI_ISL_757359, EPI_ISL_757360, EPI_ISL_757361, EPI_ISL_757362, EPI_ISL_757363, EPI_ISL_757364, EPI_ISL_757365, EPI_ISL_757366, EPI_ISL_757367, EPI_ISL_757371, EPI_ISL_757372, EPI_ISL_757373, EPI_ISL_757374, EPI_ISL_757375, EPI_ISL_757376, EPI_ISL_757377, EPI_ISL_757378, EPI_ISL_757379, EPI_ISL_757380, EPI_ISL_757381, EPI_ISL_757382, EPI_ISL_757383, EPI_ISL_757384, EPI_ISL_757385, EPI_ISL_757386, EPI_ISL_757387, EPI_ISL_757388, EPI_ISL_757389, EPI_ISL_757390, EPI_ISL_757391, EPI_ISL_757392, EPI_ISL_757393, EPI_ISL_757394			
see above	Department of Virology and Immunology, University of Helsinki and Helsinki University Hospital, Huslab Finland	Department of Virology, Faculty of Medicine, University of Helsinki, Helsinki, Finland	Teemu Smura, Ravi Kant, Phuoc Truong, Hussein Alburkat, Hannimari Kallio-Kokko, Jenni Virtanen, Maija Suvanto, Sari Hannula, Harri Kangas, Pekka Ellonen, Olli Vapalahti
EPI_ISL_757395, EPI_ISL_757396, EPI_ISL_757397, EPI_ISL_757398, EPI_ISL_757399, EPI_ISL_757400, EPI_ISL_757401, EPI_ISL_757402, EPI_ISL_757403, EPI_ISL_757404, EPI_ISL_757405, EPI_ISL_757406, EPI_ISL_757407, EPI_ISL_757408, EPI_ISL_757409, EPI_ISL_757410, EPI_ISL_757411, EPI_ISL_757412, EPI_ISL_757413, EPI_ISL_757414, EPI_ISL_757415, EPI_ISL_757416, EPI_ISL_757417, EPI_ISL_757418, EPI_ISL_757419, EPI_ISL_757420, EPI_ISL_757421, EPI_ISL_757422, EPI_ISL_757423, EPI_ISL_757424, EPI_ISL_757425, EPI_ISL_757426, EPI_ISL_757427, EPI_ISL_757428, EPI_ISL_757429, EPI_ISL_757430, EPI_ISL_757431, EPI_ISL_757432, EPI_ISL_757433, EPI_ISL_757434, EPI_ISL_757435, EPI_ISL_757436, EPI_ISL_757437, EPI_ISL_757438, EPI_ISL_757439, EPI_ISL_757440, EPI_ISL_757441, EPI_ISL_757442, EPI_ISL_757443, EPI_ISL_757444, EPI_ISL_757445, EPI_ISL_757446, EPI_ISL_757447, EPI_ISL_757448, EPI_ISL_757449, EPI_ISL_757450, EPI_ISL_757451, EPI_ISL_757452, EPI_ISL_757453, EPI_ISL_757454, EPI_ISL_757455, EPI_ISL_757456, EPI_ISL_757457, EPI_ISL_757458, EPI_ISL_757459, EPI_ISL_757460, EPI_ISL_757461, EPI_ISL_757462, EPI_ISL_757465, EPI_ISL_757466, EPI_ISL_757467, EPI_ISL_757468, EPI_ISL_757469, EPI_ISL_757470, EPI_ISL_757471, EPI_ISL_757472, EPI_ISL_757473, EPI_ISL_757474, EPI_ISL_757475, EPI_ISL_757476, EPI_ISL_757477, EPI_ISL_757478, EPI_ISL_757479, EPI_ISL_757480, EPI_ISL_757481, EPI_ISL_757482, EPI_ISL_757483, EPI_ISL_757484, EPI_ISL_757485, EPI_ISL_757486, EPI_ISL_757487, EPI_ISL_757488, EPI_ISL_757489, EPI_ISL_757490, EPI_ISL_757491, EPI_ISL_757492, EPI_ISL_757493, EPI_ISL_757494, EPI_ISL_757495, EPI_ISL_757496, EPI_ISL_757497, EPI_ISL_757498, EPI_ISL_757499, EPI_ISL_757500, EPI_ISL_757501, EPI_ISL_757502, EPI_ISL_757503, EPI_ISL_757504, EPI_ISL_757505, EPI_ISL_757506, EPI_ISL_757507, EPI_ISL_757508, EPI_ISL_757509, EPI_ISL_757510, EPI_ISL_757511, EPI_ISL_757512, EPI_ISL_757513, EPI_ISL_757514, EPI_ISL_757515, EPI_ISL_757516, EPI_ISL_757517, EPI_ISL_757518, EPI_ISL_757519, EPI_ISL_757520, EPI_ISL_757521, EPI_ISL_757522, EPI_ISL_757523, EPI_ISL_757524, EPI_ISL_757525, EPI_ISL_757526, EPI_ISL_757527, EPI_ISL_757528, EPI_ISL_757529, EPI_ISL_757530, EPI_ISL_757531, EPI_ISL_757532, EPI_ISL_757533, EPI_ISL_757534, EPI_ISL_757535, EPI_ISL_757536, EPI_ISL_757537, EPI_ISL_757538, EPI_ISL_757539, EPI_ISL_757540, EPI_ISL_757541, EPI_ISL_757542, EPI_ISL_757543, EPI_ISL_757544, EPI_ISL_757545, EPI_ISL_757546, EPI_ISL_757547, EPI_ISL_757548, EPI_ISL_757549, EPI_ISL_757550, EPI_ISL_757551, EPI_ISL_757552, EPI_ISL_757553, EPI_ISL_757554, EPI_ISL_757555, EPI_ISL_757556, EPI_ISL_757557, EPI_ISL_757558, EPI_ISL_757559, EPI_ISL_757560, EPI_ISL_757561, EPI_ISL_757562, EPI_ISL_757563, EPI_ISL_757564, EPI_ISL_757565, EPI_ISL_757566, EPI_ISL_757567, EPI_ISL_757568, EPI_ISL_757569, EPI_ISL_757570, EPI_ISL_757571, EPI_ISL_757572, EPI_ISL_757573, EPI_ISL_757574, EPI_ISL_757575, EPI_ISL_757576, EPI_ISL_757577, EPI_ISL_757578, EPI_ISL_757579, EPI_ISL_757580, EPI_ISL_757581, EPI_ISL_757582, EPI_ISL_757583, EPI_ISL_757584, EPI_ISL_757585, EPI_ISL_757586, EPI_ISL_757587, EPI_ISL_757588, EPI_ISL_757589, EPI_ISL_757590, EPI_ISL_757591, EPI_ISL_757592, EPI_ISL_757593, EPI_ISL_757594, EPI_ISL_757595, EPI_ISL_757596, EPI_ISL_757597, EPI_ISL_757598, EPI_ISL_757599, EPI_ISL_757600, EPI_ISL_757601, EPI_ISL_757602, EPI_ISL_757603, EPI_ISL_757604, EPI_ISL_757605, EPI_ISL_757606, EPI_ISL_757607, EPI_ISL_757608, EPI_ISL_757609, EPI_ISL_757610, EPI_ISL_757611, EPI_ISL_757612, EPI_ISL_757613, EPI_ISL_757614, EPI_ISL_757615, EPI_ISL_757616, EPI_ISL_757617, EPI_ISL_757618, EPI_ISL_757619, EPI_ISL_757620, EPI_ISL_757621, EPI_ISL_757622, EPI_ISL_757623, EPI_ISL_757624, EPI_ISL_757625, EPI_ISL_757626, EPI_ISL_757627, EPI_ISL_757628, EPI_ISL_757629, EPI_ISL_757630,			

EPI_ISL_757631, EPI_ISL_757632, EPI_ISL_757633, EPI_ISL_757634, EPI_ISL_757635, EPI_ISL_757636, EPI_ISL_757637, EPI_ISL_757638, EPI_ISL_757639, EPI_ISL_757640, EPI_ISL_757641, EPI_ISL_757642, EPI_ISL_757643, EPI_ISL_757644, EPI_ISL_757645, EPI_ISL_757646, EPI_ISL_757647, EPI_ISL_757648,
EPI_ISL_757649, EPI_ISL_757650, EPI_ISL_757651, EPI_ISL_757652, EPI_ISL_757653, EPI_ISL_757654, EPI_ISL_757655, EPI_ISL_757656, EPI_ISL_757657, EPI_ISL_757658, EPI_ISL_757659, EPI_ISL_757660, EPI_ISL_757661, EPI_ISL_757662, EPI_ISL_757663, EPI_ISL_757664, EPI_ISL_757665, EPI_ISL_757666,
EPI_ISL_757667, EPI_ISL_757668, EPI_ISL_757669, EPI_ISL_757670, EPI_ISL_757671, EPI_ISL_757672, EPI_ISL_757673, EPI_ISL_757674, EPI_ISL_757675, EPI_ISL_757676, EPI_ISL_757677, EPI_ISL_757678, EPI_ISL_757679, EPI_ISL_757680, EPI_ISL_757681, EPI_ISL_757682, EPI_ISL_757683, EPI_ISL_757684,
EPI_ISL_757685, EPI_ISL_757686, EPI_ISL_757687, EPI_ISL_757688, EPI_ISL_757689, EPI_ISL_757690, EPI_ISL_757691, EPI_ISL_757692, EPI_ISL_757693, EPI_ISL_757694, EPI_ISL_757695, EPI_ISL_757696, EPI_ISL_757697, EPI_ISL_757698, EPI_ISL_757699, EPI_ISL_757700, EPI_ISL_757701, EPI_ISL_757702,
EPI_ISL_757703, EPI_ISL_757704, EPI_ISL_757705, EPI_ISL_757706, EPI_ISL_757707, EPI_ISL_757708, EPI_ISL_757709, EPI_ISL_757710, EPI_ISL_757711, EPI_ISL_757712, EPI_ISL_757713, EPI_ISL_757714, EPI_ISL_757715, EPI_ISL_757716, EPI_ISL_757717, EPI_ISL_757718, EPI_ISL_757719, EPI_ISL_757720,
EPI_ISL_757721, EPI_ISL_757722, EPI_ISL_757723, EPI_ISL_757724, EPI_ISL_757725, EPI_ISL_757726, EPI_ISL_757727, EPI_ISL_757728, EPI_ISL_757729, EPI_ISL_757730, EPI_ISL_757731, EPI_ISL_757732, EPI_ISL_757733, EPI_ISL_757734, EPI_ISL_757735, EPI_ISL_757736, EPI_ISL_757737, EPI_ISL_757738,
EPI_ISL_757739, EPI_ISL_757740, EPI_ISL_757741, EPI_ISL_757742, EPI_ISL_757743, EPI_ISL_757744, EPI_ISL_757745, EPI_ISL_757746, EPI_ISL_757747, EPI_ISL_757748, EPI_ISL_757749, EPI_ISL_757750, EPI_ISL_757751, EPI_ISL_757752, EPI_ISL_757753, EPI_ISL_757754, EPI_ISL_757755, EPI_ISL_757756,
EPI_ISL_757757, EPI_ISL_757758, EPI_ISL_757759, EPI_ISL_757760, EPI_ISL_757761, EPI_ISL_757762, EPI_ISL_757763, EPI_ISL_757764, EPI_ISL_757765, EPI_ISL_757766, EPI_ISL_757767, EPI_ISL_757768, EPI_ISL_757769, EPI_ISL_757770, EPI_ISL_757771, EPI_ISL_757772, EPI_ISL_757773, EPI_ISL_757774,
EPI_ISL_757775, EPI_ISL_757776, EPI_ISL_757777, EPI_ISL_757778, EPI_ISL_757779, EPI_ISL_757780, EPI_ISL_757781, EPI_ISL_757782, EPI_ISL_757783, EPI_ISL_757784, EPI_ISL_757785, EPI_ISL_757786, EPI_ISL_757787, EPI_ISL_757788, EPI_ISL_757789, EPI_ISL_757790, EPI_ISL_757791, EPI_ISL_757792,
EPI_ISL_757793, EPI_ISL_757794, EPI_ISL_757795, EPI_ISL_757796, EPI_ISL_757797, EPI_ISL_757798, EPI_ISL_757799, EPI_ISL_757800, EPI_ISL_757801, EPI_ISL_757802, EPI_ISL_757803, EPI_ISL_757804, EPI_ISL_757805, EPI_ISL_757806, EPI_ISL_757807, EPI_ISL_757808, EPI_ISL_757809, EPI_ISL_757810,
EPI_ISL_757811, EPI_ISL_757812, EPI_ISL_757813, EPI_ISL_757814, EPI_ISL_757815, EPI_ISL_757816, EPI_ISL_757817, EPI_ISL_757818, EPI_ISL_757819, EPI_ISL_757820, EPI_ISL_757821, EPI_ISL_757822, EPI_ISL_757823, EPI_ISL_757824, EPI_ISL_757825, EPI_ISL_757826, EPI_ISL_757827, EPI_ISL_757828,
EPI_ISL_757829, EPI_ISL_757830, EPI_ISL_757831, EPI_ISL_757832, EPI_ISL_757833, EPI_ISL_757834, EPI_ISL_757835, EPI_ISL_757836, EPI_ISL_757837, EPI_ISL_757838, EPI_ISL_757839, EPI_ISL_757840, EPI_ISL_757841, EPI_ISL_757842, EPI_ISL_757843, EPI_ISL_757844, EPI_ISL_757845, EPI_ISL_757846,
EPI_ISL_757847, EPI_ISL_757848, EPI_ISL_757849, EPI_ISL_757850, EPI_ISL_757851, EPI_ISL_757852, EPI_ISL_757853, EPI_ISL_757854, EPI_ISL_757855, EPI_ISL_757856, EPI_ISL_757857, EPI_ISL_757858, EPI_ISL_757859, EPI_ISL_757860, EPI_ISL_757861, EPI_ISL_757862, EPI_ISL_757863, EPI_ISL_757864,
EPI_ISL_757865, EPI_ISL_757866, EPI_ISL_757867, EPI_ISL_757868, EPI_ISL_757869, EPI_ISL_757870, EPI_ISL_757871, EPI_ISL_757872, EPI_ISL_757877, EPI_ISL_757878, EPI_ISL_757879, EPI_ISL_757880, EPI_ISL_757881, EPI_ISL_757882, EPI_ISL_757883, EPI_ISL_757884, EPI_ISL_757885, EPI_ISL_757886,
EPI_ISL_757887, EPI_ISL_757888, EPI_ISL_757889, EPI_ISL_757890, EPI_ISL_757891, EPI_ISL_757892, EPI_ISL_757893, EPI_ISL_757894, EPI_ISL_757895, EPI_ISL_757896, EPI_ISL_757897, EPI_ISL_757898, EPI_ISL_757899, EPI_ISL_757900, EPI_ISL_757901, EPI_ISL_757902, EPI_ISL_757903, EPI_ISL_757904,
EPI_ISL_757905, EPI_ISL_757906, EPI_ISL_757907, EPI_ISL_757908, EPI_ISL_757909, EPI_ISL_757910, EPI_ISL_757911, EPI_ISL_757912, EPI_ISL_757913, EPI_ISL_757914, EPI_ISL_757915, EPI_ISL_757916, EPI_ISL_757917, EPI_ISL_757918, EPI_ISL_757919, EPI_ISL_757920, EPI_ISL_757921, EPI_ISL_757922,
EPI_ISL_757923, EPI_ISL_757924, EPI_ISL_757925, EPI_ISL_757926, EPI_ISL_757927, EPI_ISL_757928, EPI_ISL_757929, EPI_ISL_757930, EPI_ISL_757931, EPI_ISL_757932, EPI_ISL_757933, EPI_ISL_757934, EPI_ISL_757935, EPI_ISL_757936, EPI_ISL_757937, EPI_ISL_757938, EPI_ISL_757939, EPI_ISL_757940,
EPI_ISL_757941, EPI_ISL_757942, EPI_ISL_757943, EPI_ISL_757944, EPI_ISL_757945, EPI_ISL_757959, EPI_ISL_757960, EPI_ISL_757961, EPI_ISL_757962, EPI_ISL_757963, EPI_ISL_757964, EPI_ISL_757965, EPI_ISL_757966, EPI_ISL_757967, EPI_ISL_757968, EPI_ISL_757969, EPI_ISL_757970, EPI_ISL_757971,
EPI_ISL_757972, EPI_ISL_757973, EPI_ISL_757974, EPI_ISL_757975, EPI_ISL_757976, EPI_ISL_757977, EPI_ISL_757978, EPI_ISL_757979, EPI_ISL_757980, EPI_ISL_757981, EPI_ISL_757982, EPI_ISL_757983, EPI_ISL_757984, EPI_ISL_757985, EPI_ISL_757986, EPI_ISL_757987, EPI_ISL_757988, EPI_ISL_757989,
EPI_ISL_757990, EPI_ISL_757991, EPI_ISL_757992, EPI_ISL_757993, EPI_ISL_757994, EPI_ISL_757995, EPI_ISL_757996, EPI_ISL_757997, EPI_ISL_757998, EPI_ISL_757999, EPI_ISL_758000, EPI_ISL_758001, EPI_ISL_758002, EPI_ISL_758003, EPI_ISL_758004, EPI_ISL_758005, EPI_ISL_758006, EPI_ISL_758007,
EPI_ISL_758008, EPI_ISL_758009, EPI_ISL_758010, EPI_ISL_758011, EPI_ISL_758012, EPI_ISL_758013, EPI_ISL_758014, EPI_ISL_758015, EPI_ISL_758016, EPI_ISL_758017, EPI_ISL_758018, EPI_ISL_758019, EPI_ISL_758020, EPI_ISL_758021, EPI_ISL_758022, EPI_ISL_758023, EPI_ISL_758024, EPI_ISL_758025,
EPI_ISL_758026, EPI_ISL_758027, EPI_ISL_758028, EPI_ISL_758029, EPI_ISL_758030, EPI_ISL_758031, EPI_ISL_758032, EPI_ISL_758033, EPI_ISL_758034, EPI_ISL_758035, EPI_ISL_758036, EPI_ISL_758037, EPI_ISL_758038, EPI_ISL_758039, EPI_ISL_758040, EPI_ISL_758041, EPI_ISL_758042, EPI_ISL_758043,
EPI_ISL_758044, EPI_ISL_758045, EPI_ISL_758046, EPI_ISL_758047, EPI_ISL_758048, EPI_ISL_758049, EPI_ISL_758050, EPI_ISL_758051, EPI_ISL_758052, EPI_ISL_758053, EPI_ISL_758054, EPI_ISL_758055, EPI_ISL_758056, EPI_ISL_758057, EPI_ISL_758058, EPI_ISL_758059, EPI_ISL_758060, EPI_ISL_758061,
EPI_ISL_758062, EPI_ISL_758063, EPI_ISL_758064, EPI_ISL_758065, EPI_ISL_758066, EPI_ISL_758067, EPI_ISL_758068, EPI_ISL_758069, EPI_ISL_758070, EPI_ISL_758071, EPI_ISL_758072, EPI_ISL_758073, EPI_ISL_758074, EPI_ISL_758075, EPI_ISL_758076, EPI_ISL_758077, EPI_ISL_758078, EPI_ISL_758079,
EPI_ISL_758080, EPI_ISL_758081, EPI_ISL_758082, EPI_ISL_758083, EPI_ISL_758084, EPI_ISL_758085, EPI_ISL_758086, EPI_ISL_758087, EPI_ISL_758088, EPI_ISL_758089, EPI_ISL_758090, EPI_ISL_758091, EPI_ISL_758092, EPI_ISL_758093, EPI_ISL_758094, EPI_ISL_758095, EPI_ISL_758096, EPI_ISL_758097,
EPI_ISL_758098, EPI_ISL_758099, EPI_ISL_758100, EPI_ISL_758101, EPI_ISL_758102, EPI_ISL_758103, EPI_ISL_758104, EPI_ISL_758105, EPI_ISL_758106, EPI_ISL_758107, EPI_ISL_758108, EPI_ISL_758109, EPI_ISL_758110, EPI_ISL_758111, EPI_ISL_758112, EPI_ISL_758113, EPI_ISL_758114, EPI_ISL_758115,
EPI_ISL_758116, EPI_ISL_758117, EPI_ISL_758118, EPI_ISL_758119, EPI_ISL_758120, EPI_ISL_758121, EPI_ISL_758122, EPI_ISL_758123, EPI_ISL_758124, EPI_ISL_758125, EPI_ISL_758126, EPI_ISL_758127, EPI_ISL_758128, EPI_ISL_758129, EPI_ISL_758130, EPI_ISL_758131, EPI_ISL_758132, EPI_ISL_758133,
EPI_ISL_758134, EPI_ISL_758135, EPI_ISL_758136, EPI_ISL_758137, EPI_ISL_758138, EPI_ISL_758139, EPI_ISL_758140, EPI_ISL_758141, EPI_ISL_758142, EPI_ISL_758143, EPI_ISL_758144, EPI_ISL_758145, EPI_ISL_758146, EPI_ISL_758147, EPI_ISL_758148, EPI_ISL_758149, EPI_ISL_758150, EPI_ISL_758151,
EPI_ISL_758152, EPI_ISL_758153, EPI_ISL_758154, EPI_ISL_758155, EPI_ISL_758156, EPI_ISL_758157, EPI_ISL_758158, EPI_ISL_758159, EPI_ISL_758160, EPI_ISL_758161, EPI_ISL_758162, EPI_ISL_758163, EPI_ISL_758164, EPI_ISL_758165, EPI_ISL_758166, EPI_ISL_758167, EPI_ISL_758168, EPI_ISL_758169,
EPI_ISL_758170, EPI_ISL_758171, EPI_ISL_758172, EPI_ISL_758173, EPI_ISL_758174, EPI_ISL_758175, EPI_ISL_758176, EPI_ISL_758177, EPI_ISL_758178, EPI_ISL_758179, EPI_ISL_758180, EPI_ISL_758181, EPI_ISL_758182, EPI_ISL_758183, EPI_ISL_758184, EPI_ISL_758185, EPI_ISL_758186, EPI_ISL_758187,
EPI_ISL_758188, EPI_ISL_758189, EPI_ISL_758190, EPI_ISL_758191, EPI_ISL_758192, EPI_ISL_758193, EPI_ISL_758194, EPI_ISL_758195, EPI_ISL_758196, EPI_ISL_758197, EPI_ISL_758198, EPI_ISL_758199, EPI_ISL_758200, EPI_ISL_758201, EPI_ISL_758202, EPI_ISL_758203, EPI_ISL_758204, EPI_ISL_758205,
EPI_ISL_758206, EPI_ISL_758207, EPI_ISL_758208, EPI_ISL_758209, EPI_ISL_758210, EPI_ISL_758211, EPI_ISL_758212, EPI_ISL_758213, EPI_ISL_758214, EPI_ISL_758215, EPI_ISL_758216, EPI_ISL_758217, EPI_ISL_758218, EPI_ISL_758219, EPI_ISL_758220, EPI_ISL_758221, EPI_ISL_758222, EPI_ISL_758223,
EPI_ISL_758224, EPI_ISL_758225, EPI_ISL_758226, EPI_ISL_758227, EPI_ISL_758228, EPI_ISL_758229, EPI_ISL_758230, EPI_ISL_758231, EPI_ISL_758232, EPI_ISL_758233, EPI_ISL_758234, EPI_ISL_758235, EPI_ISL_758236, EPI_ISL_758237, EPI_ISL_758238, EPI_ISL_758239, EPI_ISL_758240, EPI_ISL_758241,
EPI_ISL_758242, EPI_ISL_758243, EPI_ISL_758244, EPI_ISL_758245, EPI_ISL_758246, EPI_ISL_758247, EPI_ISL_758248, EPI_ISL_758249, EPI_ISL_758250, EPI_ISL_758251, EPI_ISL_758252, EPI_ISL_758253, EPI_ISL_758254, EPI_ISL_758255, EPI_ISL_758256, EPI_ISL_758257, EPI_ISL_758258, EPI_ISL_758259,
EPI_ISL_758260, EPI_ISL_758261, EPI_ISL_758262, EPI_ISL_758263, EPI_ISL_758264, EPI_ISL_758265, EPI_ISL_758266, EPI_ISL_758267, EPI_ISL_758268, EPI_ISL_758269, EPI_ISL_758270, EPI_ISL_758271, EPI_ISL_758272, EPI_ISL_758273, EPI_ISL_758274, EPI_ISL_758275, EPI_ISL_758276, EPI_ISL_758277,
EPI_ISL_758278, EPI_ISL_758279, EPI_ISL_758280, EPI_ISL_758281, EPI_ISL_758282, EPI_ISL_758283, EPI_ISL_758284, EPI_ISL_758285, EPI_ISL_758286, EPI_ISL_758287, EPI_ISL_758288, EPI_ISL_758289, EPI_ISL_758290, EPI_ISL_758291, EPI_ISL_758292, EPI_ISL_758293, EPI_ISL_758294, EPI_ISL_758295,
EPI_ISL_758296, EPI_ISL_758297, EPI_ISL_758298, EPI_ISL_758299, EPI_ISL_758300, EPI_ISL_758301, EPI_ISL_758302, EPI_ISL_758303, EPI_ISL_758304, EPI_ISL_758305, EPI_ISL_758306, EPI_ISL_758307, EPI_ISL_758308, EPI_ISL_758309, EPI_ISL_758310, EPI_ISL_758311, EPI_ISL_758312, EPI_ISL_758313,
EPI_ISL_758314, EPI_ISL_758315, EPI_ISL_758316, EPI_ISL_758317, EPI_ISL_758318, EPI_ISL_758319, EPI_ISL_758320, EPI_ISL_758321, EPI_ISL_758322, EPI_ISL_758323, EPI_ISL_758324, EPI_ISL_758325, EPI_ISL_758326, EPI_ISL_758327, EPI_ISL_758328, EPI_ISL_758329, EPI_ISL_758330, EPI_ISL_758331,
EPI_ISL_758332, EPI_ISL_758333, EPI_ISL_758334, EPI_ISL_758335, EPI_ISL_758336, EPI_ISL_758337, EPI_ISL_758338, EPI_ISL_758339, EPI_ISL_758340, EPI_ISL_758341, EPI_ISL_758342, EPI_ISL_758343, EPI_ISL_758344, EPI_ISL_758345, EPI_ISL_758346, EPI_ISL_758347, EPI_ISL_758348, EPI_ISL_758349,
EPI_ISL_758350, EPI_ISL_758351, EPI_ISL_758352, EPI_ISL_758353, EPI_ISL_758354, EPI_ISL_758355, EPI_ISL_758356, EPI_ISL_758357, EPI_ISL_758358, EPI_ISL_758359, EPI_ISL_758360, EPI_ISL_758361, EPI_ISL_758362, EPI_ISL_758363, EPI_ISL_758364, EPI_ISL_758365, EPI_ISL_758366, EPI_ISL_758367,
EPI_ISL_758368, EPI_ISL_758369, EPI_ISL_758370, EPI_ISL_758371, EPI_ISL_758372, EPI_ISL_758373, EPI_ISL_758374, EPI_ISL_758375, EPI_ISL_758376, EPI_ISL_758377, EPI_ISL_758378, EPI_ISL_758379, EPI_ISL_758380, EPI_ISL_758381, EPI_ISL_758382, EPI_ISL_758383, EPI_ISL_758384, EPI_ISL_758385,
EPI_ISL_758386, EPI_ISL_758387, EPI_ISL_758388, EPI_ISL_758389, EPI_ISL_758390, EPI_ISL_758391, EPI_ISL_758392, EPI_ISL_758393, EPI_ISL_758394, EPI_ISL_758395, EPI_ISL_758396, EPI_ISL_758397, EPI_ISL_758398, EPI_ISL_758399, EPI_ISL_758400, EPI_ISL_758401, EPI_ISL_758402, EPI_ISL_758403,
EPI_ISL_758404, EPI_ISL_758405, EPI_ISL_758406, EPI_ISL_758407, EPI_ISL_758408, EPI_ISL_758409, EPI_ISL_758410, EPI_ISL_758411, EPI_ISL_758412, EPI_ISL_758413, EPI_ISL_758414, EPI_ISL_758415, EPI_ISL_758416, EPI_ISL_758417, EPI_ISL_758418, EPI_ISL_758419, EPI_ISL_758420, EPI_ISL_758421,
EPI_ISL_758422, EPI_ISL_758423, EPI_ISL_758424, EPI_ISL_758425, EPI_ISL_758426, EPI_ISL_758427, EPI_ISL_758428, EPI_ISL_758429, EPI_ISL_758430, EPI_ISL_758431, EPI_ISL_758432, EPI_ISL_758433, EPI_ISL_758434, EPI_ISL_758435, EPI_ISL_758436, EPI_ISL_758437, EPI_ISL_758438, EPI_ISL_758439,
EPI_ISL_758440, EPI_ISL_758441, EPI_ISL_758442, EPI_ISL_758443, EPI_ISL_758444, EPI_ISL_758445, EPI_ISL_758446, EPI_ISL_758447, EPI_ISL_758448, EPI_ISL_758449, EPI_ISL_758450, EPI_ISL_758451, EPI_ISL_758452, EPI_ISL_758453, EPI_ISL_758454, EPI_ISL_758455, EPI_ISL_758456, EPI_ISL_758457,
EPI_ISL_758458, EPI_ISL_758459, EPI_ISL_758460, EPI_ISL_758461, EPI_ISL_758462, EPI_ISL_758463, EPI_ISL_758464, EPI_ISL_758465, EPI_ISL_758466, EPI_ISL_758467, EPI_ISL_758468, EPI_ISL_758469, EPI_ISL_758470, EPI_ISL_758471, EPI_ISL_758472, EPI_ISL_758473, EPI_ISL_758474, EPI_ISL_758475,
EPI_ISL_758476, EPI_ISL_758477, EPI_ISL_758478, EPI_ISL_758479, EPI_ISL_758480, EPI_ISL_758481, EPI_ISL_758482, EPI_ISL_758483, EPI_ISL_758484, EPI_ISL_758485, EPI_ISL_758486, EPI_ISL_758487, EPI_ISL_758488, EPI_ISL_758489, EPI_ISL_758490, EPI_ISL_758491, EPI_ISL_758492, EPI_ISL_758493,
EPI_ISL_758494, EPI_ISL_758495, EPI_ISL_758496, EPI_ISL_758497, EPI_ISL_758498, EPI_ISL_758499, EPI_ISL_758500, EPI_ISL_758501, EPI_ISL_758502, EPI_ISL_758503, EPI_ISL_758504, EPI_ISL_758505, EPI_ISL_758506, EPI_ISL_758507, EPI_ISL_758508, EPI_ISL_758509, EPI_ISL_758510, EPI_ISL_758511,
EPI_ISL_758512, EPI_ISL_758513, EPI_ISL_758514, EPI_ISL_758515, EPI_ISL_758516, EPI_ISL_758517, EPI_ISL_758518, EPI_ISL_758519, EPI_ISL_758520, EPI_ISL_758521, EPI_ISL_758522, EPI_ISL_758523, EPI_ISL_758524, EPI_ISL_758525, EPI_ISL_758526, EPI_ISL_758527, EPI_ISL_758528, EPI_ISL_758529,
EPI_ISL_758530, EPI_ISL_758531, EPI_ISL_758532, EPI_ISL_758533, EPI_ISL_758534, EPI_ISL_758535, EPI_ISL_758536, EPI_ISL_758537, EPI_ISL_758538, EPI_ISL_758539, EPI_ISL_758540, EPI_ISL_758541, EPI_ISL_758542, EPI_ISL_758543, EPI_ISL_758544, EPI_ISL_758545, EPI_ISL_758546, EPI_ISL_758547,
EPI_ISL_758548, EPI_ISL_758549, EPI_ISL_758550, EPI_ISL_758551, EPI_ISL_758552, EPI_ISL_758553, EPI_ISL_758554, EPI_ISL_758555, EPI_ISL_758556, EPI_ISL_758557, EPI_ISL_758558, EPI_ISL_758559, EPI_ISL_758560, EPI_ISL_758561, EPI_ISL_758562, EPI_ISL_758563, EPI_ISL_758564, EPI_ISL_758565,
EPI_ISL_758566, EPI_ISL_758567, EPI_ISL_758568, EPI_ISL_758569, EPI_ISL_758570, EPI_ISL_758571, EPI_ISL_758572, EPI_ISL_758573, EPI_ISL_758574, EPI_ISL_758575, EPI_ISL_758576, EPI_ISL_758577, EPI_ISL_758578, EPI_ISL_758579, EPI_ISL_758580, EPI_ISL_758581, EPI_ISL_758582, EPI_ISL_758583,
EPI_ISL_758584, EPI_ISL_758585, EPI_ISL_758586, EPI_ISL_758587, EPI_ISL_758588, EPI_ISL_758589, EPI_ISL_758590, EPI_ISL_758591, EPI_ISL_758592, EPI_ISL_758593, EPI_ISL_758594, EPI_ISL_758595, EPI_ISL_758596, EPI_ISL_758597, EPI_ISL_758598, EPI_ISL_758599, EPI_ISL_758600, EPI_ISL_758601,
EPI_ISL_758602, EPI_ISL_758603, EPI_ISL_758604, EPI_ISL_758605, EPI_ISL_758606, EPI_ISL_758607, EPI_ISL_758608, EPI_ISL_758609, EPI_ISL_758610, EPI_ISL_758611, EPI_ISL_758612, EPI_ISL_758613, EPI_ISL_758614, EPI_ISL_758615, EPI_ISL_758616, EPI_ISL_758617, EPI_ISL_758618, EPI_ISL_758619,
EPI_ISL_758620, EPI_ISL_758621, EPI_ISL_758622, EPI_ISL_758623, EPI_ISL_758624, EPI_ISL_758625, EPI_ISL_758626, EPI_ISL_758627, EPI_ISL_758628, EPI_ISL_758629, EPI_ISL_758630, EPI_ISL_758631, EPI_ISL_758632, EPI_ISL_758633, EPI_ISL_758634, EPI_ISL_758635, EPI_ISL_758636, EPI_ISL_758637,
EPI_ISL_758638, EPI_ISL_758639, EPI_ISL_758640, EPI_ISL_758641, EPI_ISL_758642, EPI_ISL_758643, EPI_ISL_758644, EPI_ISL_758645, EPI_ISL_758646, EPI_ISL_758647, EPI_ISL_758648, EPI_ISL_758649, EPI_ISL_758650, EPI_ISL_758651, EPI_ISL_758652, EPI_ISL_758653, EPI_ISL_758654, EPI_ISL_758655,
EPI_ISL_758656, EPI_ISL_758657, EPI_ISL_758658, EPI_ISL_758659, EPI_ISL_758660, EPI_ISL_758661, EPI_ISL_758662, EPI_ISL_758663, EPI_ISL_758664, EPI_ISL_758665, EPI_ISL_758666, EPI_ISL_758667, EPI_ISL_758668, EPI_ISL_758669, EPI_ISL_758670, EPI_ISL_758671, EPI_ISL_758672, EPI_ISL_758673,
EPI_ISL_758674, EPI_ISL_758675, EPI_ISL_758676, EPI_ISL_758677, EPI_ISL_758678, EPI_ISL_758679, EPI_ISL_758680, EPI_ISL_758681, EPI_ISL_758682, EPI_ISL_758683, EPI_ISL_758684, EPI_ISL_758685, EPI_ISL_758686, EPI_ISL_758687, EPI_ISL_758688, EPI_ISL_758689, EPI_ISL_758690, EPI_ISL_758691,
EPI_ISL_758692, EPI_ISL_758693, EPI_ISL_758694, EPI_ISL_758695, EPI_ISL_758696, EPI_ISL_758697, EPI_ISL_758698, EPI_ISL_758699, EPI_ISL_758700, EPI_ISL_758701, EPI_ISL_758702, EPI_ISL_758703, EPI_ISL_758704, EPI_ISL_758705, EPI_ISL_758706, EPI_ISL_758707, EPI_ISL_758708, EPI_ISL_758709,
EPI_ISL_758710, EPI_ISL_758711, EPI_ISL_758712, EPI_ISL_758713, EPI_ISL_758714, EPI_ISL_758715, EPI_ISL_758716, EPI_ISL_758717, EPI_ISL_758718, EPI_ISL_758719, EPI_ISL_758720, EPI_ISL_758721, EPI_ISL_758722, EPI_ISL_758723, EPI_ISL_758724, EPI_ISL_758725, EPI_ISL_758726, EPI_ISL_758727,
EPI_ISL_758728, EPI_ISL_758729, EPI_ISL_758730, EPI_ISL_758731, EPI_ISL_758732, EPI_ISL_758733, EPI_ISL_758734, EPI_ISL_758735, EPI_ISL_758736, EPI_ISL_758737, EPI_ISL_758738, EPI_ISL_758739, EPI_ISL_758740, EPI_ISL_758741, EPI_ISL_758742, EPI_ISL_758743, EPI_ISL_758744, EPI_ISL_758745,
EPI_ISL_758746, EPI_ISL_758747, EPI_ISL_758748, EPI_ISL_758749, EPI_ISL_758750, EPI_ISL_758751, EPI_ISL_758752, EPI_ISL_758753, EPI_ISL_758754, EPI_ISL_758755, EPI_ISL_758756, EPI_ISL_758757, EPI_ISL_758758, EPI_ISL_758759, EPI_ISL_758760, EPI_ISL_758761, EPI_ISL_758762, EPI_ISL_758763,
EPI_ISL_758764, EPI_ISL_758765, EPI_ISL_758766, EPI_ISL_758767, EPI_ISL_758768, EPI_ISL_758769, EPI_ISL_758770, EPI_ISL_758771, EPI_ISL_758772, EPI_ISL_758773, EPI_ISL_758774, EPI_ISL_758775, EPI_ISL_758776, EPI_ISL_758777, EPI_ISL_758778, EPI_ISL_758779, EPI_ISL_758780, EPI_ISL_758781,
EPI_ISL_758782, EPI_ISL_758783, EPI_ISL_758784, EPI_ISL_758785, EPI_ISL_758786, EPI_ISL_758787, EPI_ISL_758788, EPI_ISL_758789, EPI_ISL_758790, EPI_ISL_758791, EPI_ISL_758792, EPI_ISL_758793, EPI_ISL_758794, EPI_ISL_758795, EPI_ISL_758796, EPI_ISL_758797, EPI_ISL_758798, EPI_ISL_758799,
EPI_ISL_758800, EPI_ISL_758801, EPI_ISL_758802, EPI_ISL_758803, EPI_ISL_758804, EPI_ISL_758805, EPI_ISL_758806, EPI_ISL_758807, EPI_ISL_758808, EPI_ISL_758809, EPI_ISL_758810, EPI_ISL_758811, EPI_ISL_758812, EPI_ISL_758813, EPI_ISL_758814, EPI_ISL_758815, EPI_ISL_758816, EPI_ISL_758817,
EPI_ISL_758818, EPI_ISL_758819, EPI_ISL_758820, EPI_ISL_758821, EPI_ISL_758822, EPI_ISL_758823, EPI_ISL_758824, EPI_ISL_758825, EPI_ISL_758826, EPI_ISL_758827, EPI_ISL_758828, EPI_ISL_758829, EPI_ISL_758830, EPI_ISL_758831, EPI_ISL_758832, EPI_ISL_758833, EPI_ISL_758834, EPI_ISL_758835,
EPI_ISL_758836, EPI_ISL_758837, EPI_ISL_758838, EPI_ISL_758839, EPI_ISL_758840, EPI_ISL_758841, EPI_ISL_758842, EPI_ISL_758843, EPI_ISL_758844, EPI_ISL_758845, EPI_ISL_758846, EPI_ISL_758847, EPI_ISL_758848, EPI_ISL_758849, EPI_ISL_758850, EPI_ISL_758851, EPI_ISL_758852, EPI_ISL_758853,
EPI_ISL_758854, EPI_ISL_758855, EPI_ISL_758856, EPI_ISL_758857, EPI_ISL_758858, EPI_ISL_758859, EPI_ISL_758860, EPI_ISL_758861, EPI_ISL_758862, EPI_ISL_758863, EPI_ISL_758864, EPI_ISL_758865, EPI_ISL_758866, EPI_ISL_758867, EPI_ISL_758869, EPI_ISL_758870, EPI_ISL_758871, EPI_ISL_758872,
EPI_ISL_758873, EPI_ISL_758874, EPI_ISL_758875, EPI_ISL_758876, EPI_ISL_758877, EPI_ISL_758878, EPI_ISL_758879, EPI_ISL_758880, EPI_ISL_758881, EPI_ISL_758882, EPI_ISL_758883, EPI_ISL_758884, EPI_ISL_758885, EPI_ISL_758886, EPI_ISL_758887, EPI_ISL_758888, EPI_ISL_758889, EPI_ISL_758890,
EPI_ISL_758891, EPI_ISL_758892, EPI_ISL_758893, EPI_ISL_758894, EPI_ISL_758895, EPI_ISL_758896, EPI_ISL_758897, EPI_ISL_758898, EPI_ISL_758899, EPI_ISL_758900, EPI_ISL_758901, EPI_ISL_758902, EPI_ISL_758903, EPI_ISL_758904, EPI_ISL_758905, EPI_ISL_758906, EPI_ISL_758907, EPI_ISL_758908,
EPI_ISL_758909, EPI_ISL_758910, EPI_ISL_758911, EPI_ISL_758912, EPI_ISL_758913, EPI_ISL_758914, EPI_ISL_758915, EPI_ISL_758916, EPI_ISL_758917, EPI_ISL_758918, EPI_ISL_758919, EPI_ISL_758920, EPI_ISL_758921, EPI_ISL_758922, EPI_ISL_758923, EPI_ISL_758924, EPI_ISL_758925, EPI_ISL_758926,
EPI_ISL_758927, EPI_ISL_758928, EPI_ISL_758929, EPI_ISL_758930, EPI_ISL_758931, EPI_ISL_758932, EPI_ISL_758933, EPI_ISL_758934, EPI_ISL_758935, EPI_ISL_758936, EPI_ISL_758937, EPI_ISL_758938, EPI_ISL_758939, EPI_ISL_758940, EPI_ISL_758941, EPI_ISL_758942, EPI_ISL_758943, EPI_ISL_758944,
EPI_ISL_758968, EPI_ISL_758969, EPI_ISL_758970, EPI_ISL_758971, EPI_ISL_758972, EPI_ISL_758973, EPI_ISL_758974, EPI_ISL_758975, EPI_ISL_758976, EPI_ISL_758977, EPI_ISL_758978, EPI_ISL_758979, EPI_ISL_758980, EPI_ISL_758981, EPI_ISL_758982, EPI_ISL_758983, EPI_ISL_758984, EPI_ISL_758985,
EPI_ISL_758986, EPI_ISL_758987, EPI_ISL_758988, EPI_ISL_758989, EPI_ISL_758990, EPI_ISL_758991, EPI_ISL_758992, EPI_ISL_758993, EPI_ISL_758994, EPI_ISL_758995, EPI_ISL_758996, EPI_ISL_758997, EPI_ISL_758998, EPI_ISL_758999, EPI_ISL_759000, EPI_ISL_759001, EPI_ISL_759002, EPI_ISL_759003,
EPI_ISL_759004, EPI_ISL_759005, EPI_ISL_759006, EPI_ISL_759007, EPI_ISL_759008, EPI_ISL_759009, EPI_ISL_759010, EPI_ISL_759011, EPI_ISL_759012, EPI_ISL_759013, EPI_ISL_759014, EPI_ISL_759015, EPI_ISL_759016, EPI_ISL_759017, EPI_ISL_759018, EPI_ISL_759019, EPI_ISL_759020, EPI_ISL_759021,
EPI_ISL_759022, EPI_ISL_759023, EPI_ISL_759024, EPI_ISL_759025, EPI_ISL_759026, EPI_ISL_759027, EPI_ISL_759028, EPI_ISL_759029, EPI_ISL_759030, EPI_ISL_759031, EPI_ISL_759032, EPI_ISL_759033, EPI_ISL_759034, EPI_ISL_759035, EPI_ISL_759036, EPI_ISL_759037, EPI_ISL_759038, EPI_ISL_759039,
EPI_ISL_759040, EPI_ISL_759041, EPI_ISL_759042, EPI_ISL_759043, EPI_ISL_759044, EPI_ISL_759045, EPI_ISL_759046, EPI_ISL_759047, EPI_ISL_759048, EPI_ISL_759049, EPI_ISL_759050, EPI_ISL_759051, EPI_ISL_759052, EPI_ISL_759053, EPI_ISL_759054, EPI_ISL_759055, EPI_ISL_759056, EPI_ISL_759057,
EPI_ISL_759058, EPI_ISL_759059, EPI_ISL_759060, EPI_ISL_759061, EPI_ISL_759062, EPI_ISL_759063, EPI_ISL_759064, EPI_ISL_759065, EPI_ISL_759066, EPI_ISL_759067, EPI_ISL_759068, EPI_ISL_759069, EPI_ISL_759070, EPI_ISL_759071, EPI_ISL_759072, EPI_ISL_759073, EPI_ISL_759074, EPI_ISL_759075,
EPI_ISL_759076, EPI_ISL_759077, EPI_ISL_759078, EPI_ISL_759079, EPI_ISL_759080, EPI_ISL_759081, EPI_ISL_759082, EPI_ISL_759083, EPI_ISL_759084, EPI_ISL_759085, EPI_ISL_759086, EPI_ISL_759087, EPI_ISL_759088, EPI_ISL_759089, EPI_ISL_759090, EPI_ISL_759091, EPI_ISL_759092, EPI_ISL_759093,
EPI_ISL_759094, EPI_ISL_759095, EPI_ISL_759096, EPI_ISL_759097, EPI_ISL_759098, EPI_ISL_759099, EPI_ISL_759100, EPI_ISL_759101, EPI_ISL_759102, EPI_ISL_759103, EPI_ISL_759104, EPI_ISL_759105, EPI_ISL_759106, EPI_ISL_759107, EPI_ISL_759108, EPI_ISL_759109, EPI_ISL_759110, EPI_ISL_759111,
EPI_ISL_759112, EPI_ISL_759113, EPI_ISL_759114, EPI_ISL_759115, EPI_ISL_759116, EPI_ISL_759117, EPI_ISL_759118, EPI_ISL_759119, EPI_ISL_759120, EPI_ISL_759121, EPI_ISL_759122, EPI_ISL_759123, EPI_ISL_759124, EPI_ISL_759125, EPI_ISL_759126, EPI_ISL_759127, EPI_ISL_759128, EPI_ISL_759129,
EPI_ISL_759130, EPI_ISL_759131, EPI_ISL_759132, EPI_ISL_759133, EPI_ISL_759134, EPI_ISL_759135, EPI_ISL_759136, EPI_ISL_759137, EPI_ISL_759138, EPI_ISL_759139, EPI_ISL_759140, EPI_ISL_759141, EPI_ISL_759142, EPI_ISL_759143, EPI_ISL_759144, EPI_ISL_759145, EPI_ISL_759146, EPI_ISL_759147,
EPI_ISL_759148, EPI_ISL_759149, EPI_ISL_759150, EPI_ISL_759151, EPI_ISL_759152, EPI_ISL_759153, EPI_ISL_759154, EPI_ISL_759155, EPI_ISL_759156, EPI_ISL_759157, EPI_ISL_759158, EPI_ISL_759159, EPI_ISL_759160, EPI_ISL_759161, EPI_ISL_759162, EPI_ISL_759163, EPI_ISL_759164, EPI_ISL_759165,
EPI_ISL_759166, EPI_ISL_759167, EPI_ISL_759168, EPI_ISL_759169, EPI_ISL_759170, EPI_ISL_759171, EPI_ISL_759172, EPI_ISL_759173, EPI_ISL_759174, EPI_ISL_759175, EPI_ISL_759176, EPI_ISL_759177, EPI_ISL_759178, EPI_ISL_759179, EPI_ISL_759180, EPI_ISL_759181, EPI_ISL_759182, EPI_ISL_759183,
EPI_ISL_759184, EPI_ISL_759185, EPI_ISL_759186, EPI_ISL_759187, EPI_ISL_759188, EPI_ISL_759189, EPI_ISL_759190, EPI_ISL_759191, EPI_ISL_759192, EPI_ISL_759193, EPI_ISL_759194, EPI_ISL_759195, EPI_ISL_759196, EPI_ISL_759197, EPI_ISL_759198, EPI_ISL_759199, EPI_ISL_759200, EPI_ISL_759201,
EPI_ISL_759202, EPI_ISL_759203, EPI_ISL_759204, EPI_ISL_759205, EPI_ISL_759206, EPI_ISL_759207, EPI_ISL_759208, EPI_ISL_759209, EPI_ISL_759210, EPI_ISL_759211, EPI_ISL_759212, EPI_ISL_759213, EPI_ISL_759214, EPI_ISL_759215, EPI_ISL_759216, EPI_ISL_759217, EPI_ISL_759218, EPI_ISL_759219,
EPI_ISL_759220, EPI_ISL_759221, EPI_ISL_759222, EPI_ISL_759223, EPI_ISL_759224, EPI_ISL_759225, EPI_ISL_759226, EPI_ISL_759227, EPI_ISL_759228, EPI_ISL_759229, EPI_ISL_759230, EPI_ISL_759231, EPI_ISL_759232, EPI_ISL_759233, EPI_ISL_759234, EPI_ISL_759235, EPI_ISL_759236, EPI_ISL_759237,

EPI_ISL_759238, EPI_ISL_759239, EPI_ISL_759240, EPI_ISL_759241, EPI_ISL_759242, EPI_ISL_759243, EPI_ISL_759244, EPI_ISL_759245, EPI_ISL_759246, EPI_ISL_759247, EPI_ISL_759248, EPI_ISL_759249, EPI_ISL_759250, EPI_ISL_759251, EPI_ISL_759252, EPI_ISL_759253, EPI_ISL_759254, EPI_ISL_759255, EPI_ISL_759256, EPI_ISL_759257, EPI_ISL_759258, EPI_ISL_759259, EPI_ISL_759260, EPI_ISL_759261, EPI_ISL_759262, EPI_ISL_759263, EPI_ISL_759264, EPI_ISL_759265, EPI_ISL_759266, EPI_ISL_759267, EPI_ISL_759268, EPI_ISL_759269, EPI_ISL_759270, EPI_ISL_759271, EPI_ISL_759272, EPI_ISL_759273, EPI_ISL_759274, EPI_ISL_759275, EPI_ISL_759276, EPI_ISL_759277, EPI_ISL_759278, EPI_ISL_759279, EPI_ISL_759280, EPI_ISL_759281, EPI_ISL_759282, EPI_ISL_759283, EPI_ISL_759284, EPI_ISL_759285, EPI_ISL_759286, EPI_ISL_759287, EPI_ISL_759288, EPI_ISL_759289, EPI_ISL_759290, EPI_ISL_759291, EPI_ISL_759292, EPI_ISL_759293, EPI_ISL_759294, EPI_ISL_759295, EPI_ISL_759296, EPI_ISL_759297, EPI_ISL_759298, EPI_ISL_759299, EPI_ISL_759300, EPI_ISL_759301, EPI_ISL_759302, EPI_ISL_759303, EPI_ISL_759304, EPI_ISL_759305, EPI_ISL_759306, EPI_ISL_759307, EPI_ISL_759308, EPI_ISL_759309, EPI_ISL_759310, EPI_ISL_759311, EPI_ISL_759312, EPI_ISL_759313, EPI_ISL_759314, EPI_ISL_759315, EPI_ISL_759316, EPI_ISL_759317, EPI_ISL_759318, EPI_ISL_759319, EPI_ISL_759320, EPI_ISL_759321, EPI_ISL_759322, EPI_ISL_759323, EPI_ISL_759324, EPI_ISL_759325, EPI_ISL_759326, EPI_ISL_759327, EPI_ISL_759328, EPI_ISL_759329, EPI_ISL_759330, EPI_ISL_759331, EPI_ISL_759332, EPI_ISL_759333, EPI_ISL_759334, EPI_ISL_759335, EPI_ISL_759336, EPI_ISL_759337, EPI_ISL_759338, EPI_ISL_759339, EPI_ISL_759340, EPI_ISL_759341, EPI_ISL_759342, EPI_ISL_759343, EPI_ISL_759344, EPI_ISL_759345, EPI_ISL_759346, EPI_ISL_759347, EPI_ISL_759348, EPI_ISL_759349, EPI_ISL_759350, EPI_ISL_759351, EPI_ISL_759352, EPI_ISL_759353, EPI_ISL_759354, EPI_ISL_759355, EPI_ISL_759356, EPI_ISL_759357, EPI_ISL_759358, EPI_ISL_759359, EPI_ISL_759360, EPI_ISL_759361, EPI_ISL_759362, EPI_ISL_759363, EPI_ISL_759364, EPI_ISL_759365, EPI_ISL_759366, EPI_ISL_759367, EPI_ISL_759368, EPI_ISL_759369, EPI_ISL_759370, EPI_ISL_759371, EPI_ISL_759372, EPI_ISL_759373, EPI_ISL_759374, EPI_ISL_759375, EPI_ISL_759376, EPI_ISL_759377, EPI_ISL_759378, EPI_ISL_759379, EPI_ISL_759380, EPI_ISL_759381, EPI_ISL_759382, EPI_ISL_759383, EPI_ISL_759384, EPI_ISL_759385, EPI_ISL_759386, EPI_ISL_759387, EPI_ISL_759388, EPI_ISL_759389, EPI_ISL_759390, EPI_ISL_759391, EPI_ISL_759392, EPI_ISL_759393, EPI_ISL_759394, EPI_ISL_759395, EPI_ISL_759396, EPI_ISL_759397, EPI_ISL_759398, EPI_ISL_759399, EPI_ISL_759400, EPI_ISL_759401, EPI_ISL_759402, EPI_ISL_759403, EPI_ISL_759404, EPI_ISL_759405, EPI_ISL_759406, EPI_ISL_759407, EPI_ISL_759408, EPI_ISL_759409, EPI_ISL_759410, EPI_ISL_759411, EPI_ISL_759412, EPI_ISL_759413, EPI_ISL_759414, EPI_ISL_759415, EPI_ISL_759416, EPI_ISL_759417, EPI_ISL_759418, EPI_ISL_759419, EPI_ISL_759420, EPI_ISL_759421, EPI_ISL_759422, EPI_ISL_759423, EPI_ISL_759424, EPI_ISL_759425, EPI_ISL_759426, EPI_ISL_759427, EPI_ISL_759428, EPI_ISL_759429, EPI_ISL_759430, EPI_ISL_759431, EPI_ISL_759432, EPI_ISL_759433, EPI_ISL_759434, EPI_ISL_759435, EPI_ISL_759436, EPI_ISL_759437, EPI_ISL_759438, EPI_ISL_759439, EPI_ISL_759440, EPI_ISL_759441, EPI_ISL_759442, EPI_ISL_759443, EPI_ISL_759444, EPI_ISL_759445, EPI_ISL_759446, EPI_ISL_759447, EPI_ISL_759448, EPI_ISL_759449, EPI_ISL_759450, EPI_ISL_759451, EPI_ISL_759452, EPI_ISL_759453, EPI_ISL_759454, EPI_ISL_759455, EPI_ISL_759456, EPI_ISL_759457, EPI_ISL_759458, EPI_ISL_759459, EPI_ISL_759460, EPI_ISL_759461, EPI_ISL_759462, EPI_ISL_759463, EPI_ISL_759464, EPI_ISL_759465, EPI_ISL_759466, EPI_ISL_759467, EPI_ISL_759468, EPI_ISL_759469, EPI_ISL_759470, EPI_ISL_759471, EPI_ISL_759472, EPI_ISL_759473, EPI_ISL_759474, EPI_ISL_759475, EPI_ISL_759476, EPI_ISL_759477, EPI_ISL_759478, EPI_ISL_759479, EPI_ISL_759480, EPI_ISL_759481, EPI_ISL_759482, EPI_ISL_759483, EPI_ISL_759484, EPI_ISL_759485, EPI_ISL_759486, EPI_ISL_759487, EPI_ISL_759488, EPI_ISL_759489, EPI_ISL_759490, EPI_ISL_759491, EPI_ISL_759492, EPI_ISL_759493, EPI_ISL_759494, EPI_ISL_759495, EPI_ISL_759496, EPI_ISL_759497, EPI_ISL_759498, EPI_ISL_759499, EPI_ISL_759500, EPI_ISL_759501, EPI_ISL_759502, EPI_ISL_759503, EPI_ISL_759504, EPI_ISL_759505, EPI_ISL_759506, EPI_ISL_759507, EPI_ISL_759508, EPI_ISL_759509, EPI_ISL_759510, EPI_ISL_759511, EPI_ISL_759512, EPI_ISL_759513, EPI_ISL_759514, EPI_ISL_759515, EPI_ISL_759516, EPI_ISL_759517, EPI_ISL_759518, EPI_ISL_759519, EPI_ISL_759520, EPI_ISL_759521, EPI_ISL_759522, EPI_ISL_759523, EPI_ISL_759524, EPI_ISL_759525, EPI_ISL_759526, EPI_ISL_759527, EPI_ISL_759528, EPI_ISL_759529, EPI_ISL_759530, EPI_ISL_759531, EPI_ISL_759532, EPI_ISL_759533, EPI_ISL_759534, EPI_ISL_759535, EPI_ISL_759536, EPI_ISL_759537, EPI_ISL_759538, EPI_ISL_759539, EPI_ISL_759540, EPI_ISL_759541, EPI_ISL_759542, EPI_ISL_759543, EPI_ISL_759544, EPI_ISL_759545, EPI_ISL_759546, EPI_ISL_759547, EPI_ISL_759548, EPI_ISL_759549, EPI_ISL_759550, EPI_ISL_759551, EPI_ISL_759552, EPI_ISL_759553, EPI_ISL_759554, EPI_ISL_759555, EPI_ISL_759556, EPI_ISL_759557, EPI_ISL_759558, EPI_ISL_759559, EPI_ISL_759560, EPI_ISL_759561, EPI_ISL_759562, EPI_ISL_759563, EPI_ISL_759564, EPI_ISL_759565, EPI_ISL_759566, EPI_ISL_759567, EPI_ISL_759568, EPI_ISL_759569, EPI_ISL_759570, EPI_ISL_759571, EPI_ISL_759572, EPI_ISL_759573, EPI_ISL_759574, EPI_ISL_759575, EPI_ISL_759576, EPI_ISL_759577, EPI_ISL_759578, EPI_ISL_759579, EPI_ISL_759580, EPI_ISL_759581, EPI_ISL_759582, EPI_ISL_759583, EPI_ISL_759584, EPI_ISL_759585, EPI_ISL_759586, EPI_ISL_759587, EPI_ISL_759588, EPI_ISL_759589, EPI_ISL_759590, EPI_ISL_759591, EPI_ISL_759592, EPI_ISL_759593, EPI_ISL_759594, EPI_ISL_759595, EPI_ISL_759596, EPI_ISL_759597, EPI_ISL_759598, EPI_ISL_759599, EPI_ISL_759600, EPI_ISL_759601, EPI_ISL_759602, EPI_ISL_759603, EPI_ISL_759604, EPI_ISL_759605, EPI_ISL_759606, EPI_ISL_759607, EPI_ISL_759608, EPI_ISL_759609, EPI_ISL_759610, EPI_ISL_759611, EPI_ISL_759612, EPI_ISL_759613, EPI_ISL_759614, EPI_ISL_759615, EPI_ISL_759616, EPI_ISL_759617, EPI_ISL_759618, EPI_ISL_759619, EPI_ISL_759620, EPI_ISL_759621, EPI_ISL_759622, EPI_ISL_759623, EPI_ISL_759624, EPI_ISL_759625, EPI_ISL_759626, EPI_ISL_759627, EPI_ISL_759628, EPI_ISL_759629, EPI_ISL_759630, EPI_ISL_759631, EPI_ISL_759632, EPI_ISL_759633, EPI_ISL_759634, EPI_ISL_759635, EPI_ISL_759636, EPI_ISL_759637, EPI_ISL_759638, EPI_ISL_759639, EPI_ISL_759640, EPI_ISL_759641, EPI_ISL_759642, EPI_ISL_759643, EPI_ISL_759644, EPI_ISL_759645, EPI_ISL_759646, EPI_ISL_759647, EPI_ISL_759648, EPI_ISL_759649, EPI_ISL_759650, EPI_ISL_759651, EPI_ISL_759652, EPI_ISL_759653, EPI_ISL_759654, EPI_ISL_759655, EPI_ISL_759656, EPI_ISL_759657, EPI_ISL_759658, EPI_ISL_759659, EPI_ISL_759660, EPI_ISL_759661, EPI_ISL_759662, EPI_ISL_759663, EPI_ISL_759664, EPI_ISL_759665, EPI_ISL_759666, EPI_ISL_759667, EPI_ISL_759668, EPI_ISL_759669, EPI_ISL_759670, EPI_ISL_759671, EPI_ISL_759672, EPI_ISL_759673, EPI_ISL_759674, EPI_ISL_759675, EPI_ISL_759676, EPI_ISL_759677, EPI_ISL_759678, EPI_ISL_759679, EPI_ISL_759680, EPI_ISL_759681, EPI_ISL_759682, EPI_ISL_759683, EPI_ISL_759684, EPI_ISL_759685, EPI_ISL_759686, EPI_ISL_759687, EPI_ISL_759688, EPI_ISL_759689, EPI_ISL_759690, EPI_ISL_759691, EPI_ISL_759692, EPI_ISL_759693, EPI_ISL_759694, EPI_ISL_759695, EPI_ISL_759696, EPI_ISL_759697, EPI_ISL_759698, EPI_ISL_759699, EPI_ISL_759700			
see above	Department of Virus and Microbiological Special Diagnostics, Statens Serum Institut, Copenhagen, Denmark	Albertsen Lab, Department of Chemistry and Bioscience, Aalborg University, Denmark	Danish Covid-19 Genome Consortium
EPI_ISL_759701	RS UNS, Surakarta	UNS/RS-UNS, Faculty of Medicine	Betty Suryawati, Yulia Sari, Hartono, Maryani, Afif Avicenna Gufron, HanaHana Apsari Pawestri, Kartika Dewi Puspa, Hartanti Dian Ika, Arie Ardiansyah Nugraha, Vivi Setiawaty.
EPI_ISL_759702	RS UNS Surakarta	Universitas Sebelas Maret (UNS), Rumah sakit Universitas Sebelas Maret (RS-UNS) Surakarta, National Institute of Health Research and Development, Indonesian Ministry of Health	Betty Suryawati, Yulia sari, hartono, maryani, Afif Avicena Gufron, Hana Apsari Pawestri, Kartika, Dewi Puspa, Hartanti Dian Ika, Arie Ardiansyah Nugraha, Vivi Setiawaty
EPI_ISL_759703	RSUD Bung Karno	Universitas Sebelas Maret (UNS), Rumah sakit Universitas Sebelas Maret (RS-UNS) Surakarta, National Institute of Health Research and Development, Indonesian Ministry of Health	Betty Suryawati, Yulia sari, hartono, maryani, Afif Avicena Gufron, Hana Apsari Pawestri, Kartika, Dewi Puspa, Hartanti Dian Ika, Arie Ardiansyah Nugraha, Vivi Setiawaty
EPI_ISL_759705	RSUD Bung Karno, Surakarta	Universitas Sebelas Maret (UNS); Rumah Sakit Universitas Sebelas Maret (RS-UNS), Surakarta; National Institute of Health Research and Development, Indonesian Ministry of Health , Jakarta).	Betty Suryawati, Yulia Sari, Hartono, Maryani, Afif Avicena Gufron, Hana Apsari Pawestri, Kartika, Dewi Puspa, Hartanti Dian Ika, Arie Ardiansyah Nugraha, Vivi Setiawaty.
EPI_ISL_759706	University of Wisconsin-Madison AIDS Vaccine Research Laboratories	University of Wisconsin-Madison AIDS Vaccine Research Laboratories	Gage Moreno, Katarina Braun, et al. AIDS Vaccine Research Laboratories
EPI_ISL_759707, EPI_ISL_759708, EPI_ISL_759709, EPI_ISL_759711, EPI_ISL_759713, EPI_ISL_759714, EPI_ISL_759715, EPI_ISL_759716, EPI_ISL_759718, EPI_ISL_759719, EPI_ISL_759721, EPI_ISL_759723			
see above	Instituto Nacional de Saude (INSA)	Instituto Nacional de Saude (INSA)	Borges et al
EPI_ISL_759724, EPI_ISL_759725, EPI_ISL_759726, EPI_ISL_759727, EPI_ISL_759728, EPI_ISL_759729, EPI_ISL_759730, EPI_ISL_759731, EPI_ISL_759733, EPI_ISL_759734, EPI_ISL_759735, EPI_ISL_759736, EPI_ISL_759737, EPI_ISL_759739, EPI_ISL_759740, EPI_ISL_759741, EPI_ISL_759742, EPI_ISL_759743, EPI_ISL_759744, EPI_ISL_759745, EPI_ISL_759746, EPI_ISL_759747, EPI_ISL_759748, EPI_ISL_759749, EPI_ISL_759750, EPI_ISL_759751, EPI_ISL_759752, EPI_ISL_759753, EPI_ISL_759754, EPI_ISL_759755, EPI_ISL_759756, EPI_ISL_759758, EPI_ISL_759759			
see above	University of Wisconsin-Madison AIDS Vaccine Research Laboratories	University of Wisconsin-Madison AIDS Vaccine Research Laboratories	Gage Moreno, Katarina Braun, et al. AIDS Vaccine Research Laboratories
EPI_ISL_759761	Instituto Nacional de Saude (INSA)	Instituto Nacional de Saude (INSA)	Borges et al
EPI_ISL_759763, EPI_ISL_759764, EPI_ISL_759765, EPI_ISL_759766, EPI_ISL_759767, EPI_ISL_759768, EPI_ISL_759769, EPI_ISL_759770, EPI_ISL_759771, EPI_ISL_759772, EPI_ISL_759773, EPI_ISL_759774, EPI_ISL_759775, EPI_ISL_759776, EPI_ISL_759777, EPI_ISL_759778, EPI_ISL_759779, EPI_ISL_759780, EPI_ISL_759781, EPI_ISL_759782, EPI_ISL_759783, EPI_ISL_759784, EPI_ISL_759785, EPI_ISL_759786, EPI_ISL_759787, EPI_ISL_759788, EPI_ISL_759789, EPI_ISL_759790, EPI_ISL_759791, EPI_ISL_759792, EPI_ISL_759793, EPI_ISL_759794, EPI_ISL_759795, EPI_ISL_759796, EPI_ISL_759797, EPI_ISL_759798, EPI_ISL_759799, EPI_ISL_759800, EPI_ISL_759801, EPI_ISL_759802, EPI_ISL_759803, EPI_ISL_759804, EPI_ISL_759805, EPI_ISL_759806, EPI_ISL_759807, EPI_ISL_759808, EPI_ISL_759809, EPI_ISL_759810, EPI_ISL_759811, EPI_ISL_759818, EPI_ISL_759819, EPI_ISL_759820, EPI_ISL_759821, EPI_ISL_759822, EPI_ISL_759823, EPI_ISL_759824, EPI_ISL_759825, EPI_ISL_759826, EPI_ISL_759827, EPI_ISL_759828, EPI_ISL_759829, EPI_ISL_759830, EPI_ISL_759831, EPI_ISL_759832, EPI_ISL_759833, EPI_ISL_759834, EPI_ISL_759835, EPI_ISL_759836, EPI_ISL_759837, EPI_ISL_759838, EPI_ISL_759839, EPI_ISL_759840, EPI_ISL_759841			
see above	Department of Virology and	Department of Virology,	Teemu Smura, Ravi Kant, Phuoc Truong, Hussein Alburkat, Hannimari Kallio-Kokko, Jenni Virtanen, Maija Suvanto, Sari Hannula, Harri Kangas, Pekka Ellonen, Olli Vapalahti

	Immunology, University of Helsinki and Helsinki University Hospital, Huslab Finland	Faculty of Medicine, University of Helsinki, Helsinki, Finland	
EPI_ISL_759842	Rumah Sakit Universitas Sebelas Maret (RS-UNS)	Universitas Sebelas Maret (UNS); Rumah Sakit Universitas Sebelas Maret (RS-UNS), Surakarta; National Institute of Health Research and Development, Indonesian Ministry of Health, Jakarta.	Betty Suryawati, Yulia Sari, Hartono, Maryani, Afif Avicena Ghufron, Hana Apsari Pawestri, Kartika, Dewi Puspa, Hartanti Dian Ika, Arie Ardiansyah Nugraha, Vivi Setiawaty.
EPI_ISL_759892, EPI_ISL_759893, EPI_ISL_759894	Temporary Specimen Collection Centre	Hong Kong Department of Health	Alan K.L. Tsang, Peter C.W. Yip, Edman T.K. Lam, Rickjason C.W. Chan, Dominic N.C. Tsang
EPI_ISL_759895	United Christian Hospital	Hong Kong Department of Health	Alan K.L. Tsang, Peter C.W. Yip, Edman T.K. Lam, Rickjason C.W. Chan, Dominic N.C. Tsang
EPI_ISL_759896	Queen Elizabeth Hospital	Hong Kong Department of Health	Alan K.L. Tsang, Peter C.W. Yip, Edman T.K. Lam, Rickjason C.W. Chan, Dominic N.C. Tsang
EPI_ISL_759897, EPI_ISL_759898	United Christian Hospital	Hong Kong Department of Health	Alan K.L. Tsang, Peter C.W. Yip, Edman T.K. Lam, Rickjason C.W. Chan, Dominic N.C. Tsang
EPI_ISL_759899, EPI_ISL_759900, EPI_ISL_759901, EPI_ISL_759902, EPI_ISL_759903, EPI_ISL_759904, EPI_ISL_759905, EPI_ISL_759906, EPI_ISL_759907, EPI_ISL_759908, EPI_ISL_759909, EPI_ISL_759910, EPI_ISL_759911, EPI_ISL_759912, EPI_ISL_759913, EPI_ISL_759914, EPI_ISL_759915, EPI_ISL_759916, EPI_ISL_759917, EPI_ISL_759918, EPI_ISL_759919, EPI_ISL_759920, EPI_ISL_759921, EPI_ISL_759922, EPI_ISL_759923, EPI_ISL_759924, EPI_ISL_759925, EPI_ISL_759926, EPI_ISL_759927, EPI_ISL_759928, EPI_ISL_759929, EPI_ISL_759930, EPI_ISL_759931, EPI_ISL_759932, EPI_ISL_759933, EPI_ISL_759934, EPI_ISL_759935, EPI_ISL_759936, EPI_ISL_759937, EPI_ISL_759938, EPI_ISL_759939, EPI_ISL_759940, EPI_ISL_759941, EPI_ISL_759942, EPI_ISL_759943, EPI_ISL_759944, EPI_ISL_759945, EPI_ISL_759946, EPI_ISL_759947, EPI_ISL_759948, EPI_ISL_759949, EPI_ISL_759950, EPI_ISL_759951, EPI_ISL_759952, EPI_ISL_759953, EPI_ISL_759954			
see above	Department of Virology and Immunology, University of Helsinki and Helsinki University Hospital, Huslab Finland	Department of Virology, Faculty of Medicine, University of Helsinki, Helsinki, Finland	Teemu Smura, Ravi Kant, Phuoc Truong, Hussein Alburkat, Hannimari Kallio-Kokko, Jenni Virtanen, Maija Suvanto, Sari Hannula, Harri Kangas, Pekka Ellonen, Olli Vapalahti
EPI_ISL_759960	Surabaya Public Health Office	Institute of Tropical Disease, Universitas Airlangga	Aldise M Nastri, Jezzy R Dewantari, Rima R Prasetya, Krisnoadi Rahardjo, Febria Rachmanita, Gatot Soegiarto, Laksmi Wulandari, Resti Yudhawati, Soetjipto, Yasuko Mori, Maria I Lusida, Kazufumi Shimizu
EPI_ISL_759963	RSUD Sidoarjo	Institute of Tropical Disease, Universitas Airlangga	Aldise M Nastri, Jezzy R Dewantari, Rima R Prasetya, Krisnoadi Rahardjo, Atok Irawan, Gatot Soegiarto, Laksmi Wulandari, Resti Yudhawati, Yasuko Mori, Soetjipto, Kazufumi Shimizu ,Maria I Lusida
EPI_ISL_759968	RSI Darus Syifa'	Institute of Tropical Disease, Universitas Airlangga	Maria I Lusida, Krisnoadi Rahardjo, Aldise M Nastri, Jezzy R Dewantari, Rima R Prasetya, HM Faiz, Gatot Soegiarto, Laksmi Wulandari, Resti Yudhawati, Soetjipto, Yasuko Mori, Kazufumi Shimizu
EPI_ISL_759969, EPI_ISL_759970, EPI_ISL_759971, EPI_ISL_759972	Department of Medical Microbiology, St. Olavs hospital	Norwegian Institute of Public Health, Department of Virology	Kathrine Stene-Johansen, Kamilla Heddeland Instefjord, Hilde Elshaug, Marie Paulsen Madsen, Rasmus Riis Kopperud, Hilde Vollan, Karoline Bragstad, Olav Hungnes
EPI_ISL_759973, EPI_ISL_759974	Furst Medical Laboratory	Norwegian Institute of Public Health, Department of Virology	Kathrine Stene-Johansen, Kamilla Heddeland Instefjord, Hilde Elshaug, Marie Paulsen Madsen, Rasmus Riis Kopperud, Hilde Vollan, Karoline Bragstad, Olav Hungnes
EPI_ISL_759975	Haukeland University Hospital, Dept. of Microbiology	Norwegian Institute of Public Health, Department of Virology	Kathrine Stene-Johansen, Kamilla Heddeland Instefjord, Hilde Elshaug, Marie Paulsen Madsen, Rasmus Riis Kopperud, Hilde Vollan, Karoline Bragstad, Olav Hungnes
EPI_ISL_759976	Hospital of Southern Norway - Kristiansand, Department of Medical Microbiology	Norwegian Institute of Public Health, Department of Virology	Kathrine Stene-Johansen, Kamilla Heddeland Instefjord, Hilde Elshaug, Marie Paulsen Madsen, Rasmus Riis Kopperud, Hilde Vollan, Karoline Bragstad, Olav Hungnes
EPI_ISL_759977, EPI_ISL_759978, EPI_ISL_759979, EPI_ISL_759980	Medical Microbiology Unit, Department for Laboratory Medicine, Drammen Hospital, Vestre Viken Health Trust,	Norwegian Institute of Public Health, Department of Virology	Kathrine Stene-Johansen, Kamilla Heddeland Instefjord, Hilde Elshaug, Marie Paulsen Madsen, Rasmus Riis Kopperud, Hilde Vollan, Karoline Bragstad, Olav Hungnes
EPI_ISL_759981	Norwegian Institute of Public Health, Department of Virology	Norwegian Institute of Public Health, Department of Virology	Kathrine Stene-Johansen, Kamilla Heddeland Instefjord, Hilde Elshaug, Marie Paulsen Madsen, Rasmus Riis Kopperud, Hilde Vollan, Karoline Bragstad, Olav Hungnes
EPI_ISL_759982	Oslo University Hospital, Department of Medical Microbiology	Norwegian Institute of Public Health, Department of Virology	Kathrine Stene-Johansen, Kamilla Heddeland Instefjord, Hilde Elshaug, Marie Paulsen Madsen, Rasmus Riis Kopperud, Hilde Vollan, Karoline Bragstad, Olav Hungnes
EPI_ISL_759983	Medical Microbiology Unit, Department for Laboratory Medicine, Drammen Hospital, Vestre Viken Health Trust,	Norwegian Institute of Public Health, Department of Virology	Kathrine Stene-Johansen, Kamilla Heddeland Instefjord, Hilde Elshaug, Marie Paulsen Madsen, Rasmus Riis Kopperud, Hilde Vollan, Karoline Bragstad, Olav Hungnes
EPI_ISL_759984, EPI_ISL_759985	Furst Medical Laboratory	Norwegian Institute of Public Health, Department of Virology	Kathrine Stene-Johansen, Kamilla Heddeland Instefjord, Hilde Elshaug, Marie Paulsen Madsen, Rasmus Riis Kopperud, Hilde Vollan, Karoline Bragstad, Olav Hungnes
EPI_ISL_759986, EPI_ISL_759987	University Hospital of Northern Norway, Department for Microbiology and Infectious Disease Control	Norwegian Institute of Public Health, Department of Virology	Kathrine Stene-Johansen, Kamilla Heddeland Instefjord, Hilde Elshaug, Marie Paulsen Madsen, Rasmus Riis Kopperud, Hilde Vollan, Karoline Bragstad, Olav Hungnes
EPI_ISL_759988	Dept. of Medical Microbiology, Stavanger University Hospital, Helse Stavanger HF	Norwegian Institute of Public Health, Department of Virology	Kathrine Stene-Johansen, Kamilla Heddeland Instefjord, Hilde Elshaug, Marie Paulsen Madsen, Rasmus Riis Kopperud, Hilde Vollan, Karoline Bragstad, Olav Hungnes

EPI_ISL_759989	Norwegian Institute of Public Health, Department of Virology	Norwegian Institute of Public Health, Department of Virology	Kathrine Stene-Johansen, Kamilla Heddeland Instefjord, Hilde Elshaug, Marie Paulsen Madsen, Rasmus Riis Kopperud, Hilde Vollan, Karoline Bragstad, Olav Hungnes
EPI_ISL_759990	Oslo University Hospital, Department of Medical Microbiology	Norwegian Institute of Public Health, Department of Virology	Kathrine Stene-Johansen, Kamilla Heddeland Instefjord, Hilde Elshaug, Marie Paulsen Madsen, Rasmus Riis Kopperud, Hilde Vollan, Karoline Bragstad, Olav Hungnes
EPI_ISL_759991, EPI_ISL_759992, EPI_ISL_759993, EPI_ISL_759994, EPI_ISL_759995, EPI_ISL_759996, EPI_ISL_759997, EPI_ISL_759998, EPI_ISL_759999, EPI_ISL_760000, EPI_ISL_760001, EPI_ISL_760002, EPI_ISL_760003, EPI_ISL_760004, EPI_ISL_760005, EPI_ISL_760006, EPI_ISL_760007, EPI_ISL_760008, EPI_ISL_760009, EPI_ISL_760010, EPI_ISL_760011, EPI_ISL_760012, EPI_ISL_760013, EPI_ISL_760014, EPI_ISL_760015, EPI_ISL_760016, EPI_ISL_760017, EPI_ISL_760018, EPI_ISL_760019, EPI_ISL_760020, EPI_ISL_760021, EPI_ISL_760022, EPI_ISL_760023, EPI_ISL_760024, EPI_ISL_760025, EPI_ISL_760026, EPI_ISL_760027, EPI_ISL_760028, EPI_ISL_760029, EPI_ISL_760030			
see above	Division of Emerging Infectious Diseases, Bureau of Infectious Diseases Diagnosis Control, Korea Disease Control and Prevention Agency	Division of Emerging Infectious Diseases, Bureau of Infectious Diseases Diagnosis Control, Korea Disease Control and Prevention Agency	Ae Kyung Park, Il-Hwan Kim, Heui Man Kim, Jeong-Min Kim, Namjoo Lee, Chaeyoung Lee, Sang Hee Woo, Eun-Jin Kim
EPI_ISL_760031, EPI_ISL_760032, EPI_ISL_760033, EPI_ISL_760034, EPI_ISL_760035, EPI_ISL_760036, EPI_ISL_760037, EPI_ISL_760038, EPI_ISL_760039, EPI_ISL_760040, EPI_ISL_760041, EPI_ISL_760042, EPI_ISL_760045, EPI_ISL_760046, EPI_ISL_760047, EPI_ISL_760049, EPI_ISL_760050, EPI_ISL_760051, EPI_ISL_760052, EPI_ISL_760053, EPI_ISL_760054, EPI_ISL_760055, EPI_ISL_760056, EPI_ISL_760057, EPI_ISL_760058			
see above	Hong Kong Department of Health	School of Public Health, The University of Hong Kong	Daniel Chu, Haogao Gu, Pavithra Krishnan, Daisy Ng, Gigi Liu, Carrie Wan, Malik Peiris, Leo Poon
EPI_ISL_760059	Laboratory of Genetics and Personalized Medicine, Zan Mitrev Clinic	Laboratory of Genetics and Personalized Medicine, Zan Mitrev Clinic	Kungulovski et al., 2020
EPI_ISL_760060, EPI_ISL_760061, EPI_ISL_760062, EPI_ISL_760063, EPI_ISL_760064, EPI_ISL_760065, EPI_ISL_760066, EPI_ISL_760067, EPI_ISL_760068, EPI_ISL_760069, EPI_ISL_760070, EPI_ISL_760071, EPI_ISL_760072, EPI_ISL_760073, EPI_ISL_760074, EPI_ISL_760075, EPI_ISL_760076, EPI_ISL_760077, EPI_ISL_760078, EPI_ISL_760079, EPI_ISL_760080, EPI_ISL_760081, EPI_ISL_760082, EPI_ISL_760083, EPI_ISL_760084, EPI_ISL_760085, EPI_ISL_760086, EPI_ISL_760087, EPI_ISL_760088, EPI_ISL_760089, EPI_ISL_760090, EPI_ISL_760091, EPI_ISL_760092, EPI_ISL_760093, EPI_ISL_760094, EPI_ISL_760095, EPI_ISL_760096, EPI_ISL_760097, EPI_ISL_760098			
see above	Division of Emerging Infectious Diseases, Bureau of Infectious Diseases Diagnosis Control, Korea Disease Control and Prevention Agency	Division of Emerging Infectious Diseases, Bureau of Infectious Diseases Diagnosis Control, Korea Disease Control and Prevention Agency	Ae Kyung Park, Il-Hwan Kim, Heui Man Kim, Jeong-Min Kim, Namjoo Lee, Chaeyoung Lee, Sang Hee Woo, Eun-Jin Kim
